# Genome-wide Identification and Characterization of Natural Antisense Transcripts by Strand-specific RNA Sequencing in *Ganoderma lucidum*

**DOI:** 10.1038/s41598-017-04303-6

**Published:** 2017-07-18

**Authors:** Junjie Shao, Haimei Chen, Dan Yang, Mei Jiang, Hui Zhang, Bin Wu, Jianqin Li, Lichai Yuan, Chang Liu

**Affiliations:** Key Laboratory of Bioactive Substances and Resource Utilization of Chinese Herbal Medicine from Ministry of Education, Institute of Medicinal Plant Development, Chinese Academy of Medical Sciences, Peking Union Medical College, Beijing, 100193 P.R. China

## Abstract

*Ganoderma lucidum* is a white-rot fungus best-known for its medicinal and ligninolytic activities. To discover the underlying genes responsible for these activities, we identified and characterized the natural antisense transcripts (NATs) using strand-specific (ss) RNA-seq data obtained from the mycelia, primordia and fruiting bodies. NATs were identified using a custom pipeline and then subjected to functional enrichment and differential expression analyses. A total of 1613 cis- and 244 trans- sense and antisense transcripts were identified. Mapping to GO terms and KEGG pathways revealed that NATs were frequently associated with genes of particular functional categories in particular stages. ssRT-qPCR experiments showed that the expression profiles of 30 of 50 (60%) transcripts were highly correlated with those of the RNA-seq results (*r ≥ *0.9). Expression profiles of 22 of 25 (88%) pairs of NATs and STs were highly correlated (*p* ≤ 0.01), with 15 having *r* ≥ 0.8 and 4 having *r* ≤ -0.8. Six lignin-modifying genes and their NATs were analyzed in detail. Diverse patterns of differential expression among different stages and positive and negative correlations were observed. These results suggested that NATs were implicated in gene expression regulation in a function-group and developmental-stage specific manner through complex mechanisms.

## Introduction

*Ganoderma lucidum* belongs to family Ganodermataceae, order Polyporales, class Agaricomycetes and phylum Basidiomycota. It is a white-rot fungus and best known for medical activities in East Asia countries. However, it can also cause severe diseases in economically important trees and perennial crops^1, 2^. Due to its economic and ecological importance, studies aiming to understand the molecular basis for its ability to breakdown woody plants and to produce bioactive compounds have been actively pursued. We previously sequenced the genome of *G*. *lucidum* ^3^. Then, we systematically identified protein coding genes involved in the biosynthesis of polysaccharides and secondary metabolites, and wood degradation from the sequenced genome. Later, using non-strand-specific RNA-seq data, we systematically identified the long intergenic non-coding RNAs (lincRNAs) in *G*. *lucidum* ^4^.

Through the analyses of total transcripts in eukaryotes using high throughput technologies such as RNA-seq^5^ or tilling arrays^6^, a large portion of the transcripts were found not to encode proteins, and were subsequently named non-coding RNAs (ncRNAs)^7^. The ncRNAs can be classified into two groups: housekeeping ncRNAs and regulatory ncRNAs. Housekeeping ncRNAs include ribosomal RNAs (rRNAs), transfer RNAs (tRNAs), small nuclear RNAs (snRNAs), and small nucleolar RNAs (snoRNAs). Regulatory ncRNAs include long ncRNAs (lncRNAs) and small RNAs. The lncRNAs differ from small RNAs primarily by a length cutoff of 200 nucleotides^8^. lncRNAs have been found in a large varieties of organisms, ranging from yeasts^9^ to mammals including mice^10^ and humans^11^. Based on their positions relative to the protein coding genes, lncRNAs can be subdivided into three groups: natural antisense transcripts (NATs), long intronic ncRNAs, and long intergenic ncRNAs (lincRNAs)^7^.

NATs, a subset of lncRNAs, are defined as the transcripts transcribing from the opposite DNA strand (i.e., antisense strand), as compared with the strand (i.e., sense strand) from which the protein coding genes are transcribed. In the following text, we will call transcripts transcribed from the sense strand as sense transcripts (STs). By this definition, STs correspond to protein coding genes. Some of them have NATs and the rest of them don’t. NATs can be divided into two groups: cis-NATs and trans-NATs. A cis-NAT is transcribed from the opposing DNA strand at the same genomic locus as its ST, and the overlapping region is completely reverse complementing. By contrast, a trans-NAT is transcribed from a different locus and the overlapping region is usually partially reverse complementing^12^. NATs have been shown to control nearly every level of gene regulation through interactions with DNA, RNA, or protein (reviewed in ref. 13). NATs play roles in the expression regulation of their STs via several mechanisms. For example, NATs can inhibit the transcription of their STs through steric hindrance of the transcriptional machinery; competition for transcription factors and silencing by RNAi. Furthermore, NATs can disrupt post-transcriptional modification and translation of their STs. This is achieved by NATs’ ability to form RNA/RNA duplexes with STs. Alternatively, NATs can mask the specific signals on their STs required for mRNA splicing and stability (reviewed in refs 14-16).

NATs have been found in human and mouse^17^, other mammals^18^, thale cress^19^, maize^20^, and fungi^21^. In fungi, genome-wide analyses have been carried out to identify NATs in *Saccharomyces cerevisiae* ^22^, *Candida albicans* ^23^, *Aspergillus flavus* ^24^, *Magnaporthe oryzae* ^25^, *Tuber melanosporum* ^26^, and *Schizosaccharomyces pombe* ^27^, basidiomycetes *Cryptococcus neoformans* ^28^, *Ustilago maydis* ^29^, and *Schizophyllum commune* ^30^, and *Neurospora crassa* ^31^. Among the large numbers of NATs discovered, only a few have their functions studied in detail. It is found that NATs play important roles in a wide range of biological processes, such as cellular metabolism, mating and pathogenesis (reviewed in ref. 32). For example, in *S*. *cerevisiae*, GAL1ucut is a NAT transcribed from the GAL1-10 cluster loci. Under repressive conditions, GAL1ucut was transcribed and could successfully silence the otherwise highly expressed genes located in the GAL1-10 cluster^33^. Furthermore, antisense of depressing factor 1 (ADF1) is a NAT transcribed from the opposite strand in the locus coding the mating depressing factor 1 (MDF1) protein in yeast. When the cells were grown in rich medium, MDF1 interacted with MATa2 and suppressed the mating pathway. However, when the cells were grown in poor medium, ADF1 repressed MDF1 and activated the mating pathway^34^. Finally, in *U*. *maydis*, UM02150 coded for a probable xylitol dehydrogenase. And ncRNA1 is a NAT complementary to the 3’ UTR of UM02150. Deletion of ncRNA1 reduced pathogenesis of the seedlings infected by *U*. *maydis* ^13^. Considering these examples, it is reasonable to expect that NATs might also play important roles in cellular metabolism, mating, pathogenesis and etc. in *G*. *lucidum*.

In this study, we performed strand-specific RNA-seq experiments, developed a computational pipeline to identify NATs using the new dataset. A total of 1954 sense and antisense transcripts (SATs) pairs were identified, including 1630 cis-SATs and 324 trans-SATs. This study unraveled another critical type of potential gene expression regulator in *G*. *lucidum*. The discovered NATs will be invaluable to the understanding of the processes for the production of polysaccharide and triterpenoid, the mating between mycelia, and the degradation of woody materials, not only in *G*. *lucidum*, but also in other organisms.

## Results

### Strand-specific RNA-seq analysis

To identify NATs of *G*. *lucidum*, we carried out strand-specific RNA-seq (ssRNA-seq) analysis of RNA samples extracted from three developmental stages: mycelia, primordia, and fruiting bodies. A statistical summary of the RNA-seq analysis results are shown in Table S1. In total, 50.6, 48.3, and 56.5 million of 100 bp pair-end reads were obtained for samples of mycelia, primordia, and fruiting bodies, respectively. The raw data were subjected to data cleaning to remove reads with low quality. After which, the reads were mapped to a *G*. *lucidum* reference genome sequence (Accession No: GCA_000271565.1) using TopHat (v1.3)^35^. The average coverage depths for the three RNA-seq data sets are 50-fold, 55-fold, and 60-fold, respectively. The corresponding mapping rates are 56.20%, 55.5%, and 54.3%, respectively.

### Computational identification of cis- and trans-NATs

**Figure 1:**
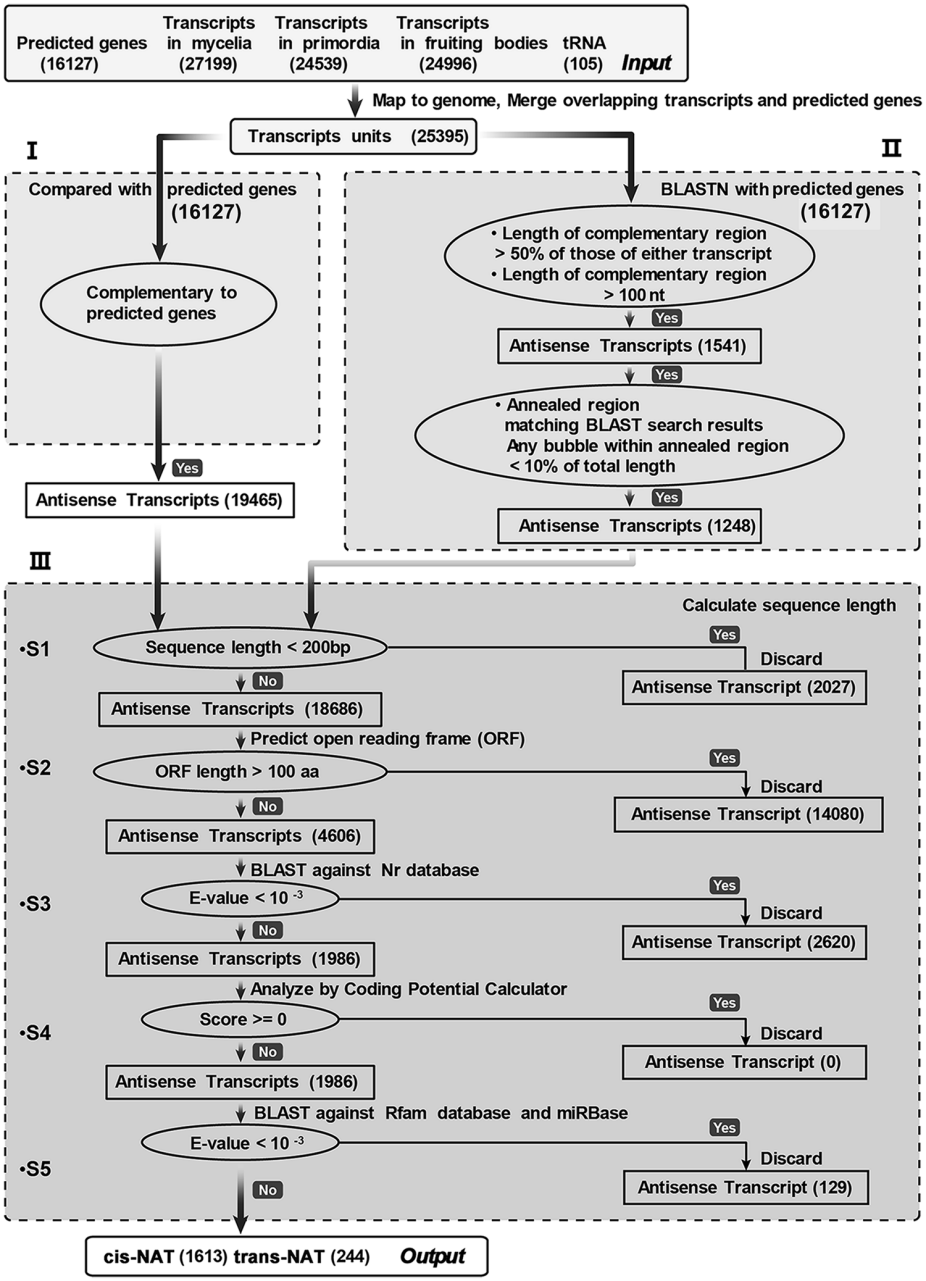
Bioinformatic pipeline used to identify NATs in *G. lucidum*. The pipeline contains three modules. The predicted genes and transcripts were combined to form transcripts units, which were then subjected to the identification of candidate cis-NATs (module I) or candidate trans-NATs (module II). The transcripts were then filtered in five steps to identify non-coding antisense transcripts (module III). The name of the filtering step and the corresponding criterion are shown above and in the corresponding diamond respectively. The number of candidate NATs retained and discarded after each filtering step is shown in parenthesis. ORF, open reading frame; aa, amino acid.

We modified the bioinformatic pipeline for lincRNA identification^4^ to identify cis-NATs and trans-NATs based on their previously described characteristics^15^. A flow chart describing the pipeline is shown in Fig. 1. The entire pipeline can be divided into three parts, which identifies: (1) candidate cis-NAT (Fig. 1, module I); (2) candidate trans-NAT (Fig. 1, module II); and (3) cis- and trans- NATs (Fig. 1, module III). The *G*. *lucidum* genome has 16127 predicted genes obtained previously^3^. Using the program TopHat and Cufflinks, we identified 27199, 24539, and 24996 transcripts from the samples derived from the three stages, respectively. These include the transcripts for the 16127 predicted protein coding genes and other non protein-coding transcripts. All these transcripts predicted from the above RNA-seq data were then clustered. Genes or transcripts on the same strands and overlapping with each other were merged, producing 25395 clusters or transcript units.

Those transcript units complementary to and overlapping with any predicted genes in the same loci were selected as candidate cis-NATs, resulting in 19465 antisense transcripts (Fig. 1, module I). By contrast, those transcript units that partially overlap with the predicted genes in a complementary orientation and are located remotely were selected as candidate for trans-NATs, resulting in 1248 transcripts (Fig. 1, module II). These antisense transcripts were then subjected to a series of filtering steps for non-coding RNA identification, based on several criteria, such as sequence length > 200 bp, maximum length of coding proteins < 100 aa, Coding Potential Calculator (CPC) score < 0^36^, and not sharing sequence similarity to those in the nr protein database (cutoff e-value = 1e-3). Lastly, to remove the precursors of housekeeping lncRNA, such as tRNAs, snRNA, and snoRNAs, the antisense transcripts were searched against housekeeping lncRNA databases (http://noncode.org/, http://gtrnadb.ucsc.edu/), with an e-value cutoff of 1e-10. To remove the precursors for small RNAs, such as miRNAs, shRNAs and siRNA, the antisense transcripts were searched against the small RNA datasets^37^, with an e-value cutoff of 1e-10. By this pipeline, 1613 cis-NATs and 244 trans-NATs were identified (Fig. 1, module III; Table S2), corresponding to 10% and 1.5% of predicted protein coding genes. These numbers are significantly lower than those predicted in other fungi. The sequences of all cis-NATs and trans-NATs can be found in Supplementary Files 1 and 2 respectively.

One of the interesting observations is that the mapping between the STs and NATs were multiplex. A total of 1175, 417, 26 and 12 cis-SAT pairs formed 1:1, 1:n, n:1, and n:n relationships. In particular, for each pair of ST and NAT, if the ST was mapped to multiple NATs and the NAT was mapped to multiple STs, then the relationship is designated as “n:n”. STs and NATs having “n:n” relationships are likely to derived from duplicated genes in the genome. Similarly, 156, 78, 29 and 61 trans-SAT pairs formed 1:1, 1:n, n:1, and n:n relationships. One possible reason is that some of these NATs are actually part of a much longer NAT due to the inherent bias in the library construction, as well as the DNA sequencing steps.

To determine if this is indeed the case, we selected two SAT pairs belonging to the 1:n type (GL23730 vs. AT14969 and AT14972; GL16401 vs. AT12077 and AT12078). Strand-specific PCR was used to amplify the regions between the two adjacent cis-NATs for each ST. The relative positions of the STs and NATs on the genomic sequences were shown in Fig. S1a and S1c respectively. When the cDNA was used as template in the PCR, products with the sizes consistent with those estimated based on the distance between the primers on the genomic sequences for GL23730 and GL16401 (Fig. S1b lane 2 and Fig. S1d lane 2) were amplified. No products were obtained when the RNA samples were directly used in the PCR reactions (Fig. S1b, lane 3 and Fig. S1d lane 3), suggesting that there were no contaminating genomic DNAs in the RNA samples. Similarly, PCR reactions with no templates added did not produce any products (Fig. S1b lane 4 and Fig. S1d lane 4). These results suggest that the two NATs (AT14969 and AT14972) for GL23730 and the two NATs (AT12077 and AT12078) for GL16401 were likely to be connected. Consequently, all 1:n NATs should be validated in the future to determine if they resulted from missed coverage or incorrect assembly.

### Characterization of NATs

**Figure 2:**
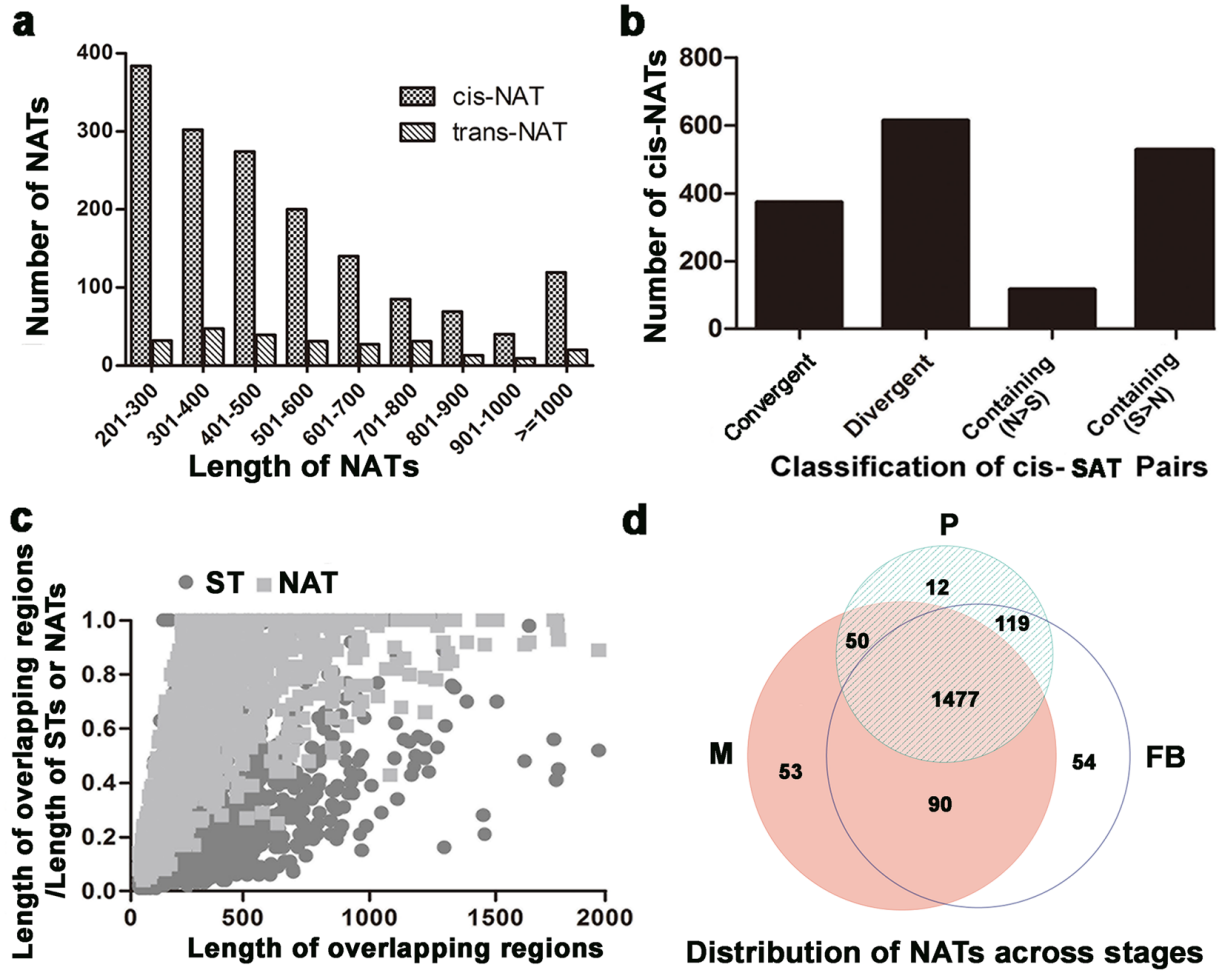
Characterization of NATs identified in *G. lucidum*. (**a**) Length distribution of cis-NATs and trans-NATs; (**b**) Distribution of cis-NATs across different types of SAT pairs. The X axis indicates the types of SAT pairs, which were determined based on the relative position of the NAT and ST in a SAT pair. “Divergent”: the NAT is located at the 5’ end of the ST; “Convergent”: the NAT is located at the 3’ end of the corresponding ST; “Containing N > S”: the NAT spans the entire region of the ST; and “Containing S > N”: the NAT is embedded in the ST; (**c**) Relationship between the ratio of the length of overlapping region to those of the corresponding NAT and ST and the length of the overlapping region; (**d**) Venn diagram showing the distribution of NATs across the three developmental stages. M: mycelia; P: primordia; FB: fruiting bodies.

GC-content has a strong impact on expression fold-change estimation and can mislead differential expression analysis if failure to adjust for this effect^38^. To determine if additional GC-content normalization is needed, we examined the GC content of STs and NATs. The GC contents of cis-NATs were similar to those of STs with NATs and lower than those of STs without NAT (Table S2). As a result, no normalization is needed to compare the expression levels of cis-NATs and STs. We also compared the length of cis-NATs and trans-NATs (Fig. 2a) after binning all NATs. As shown in the figure, the length of cis-NATs mostly ranged from 201 nt to 700 nt, with an average length of 523 nt. In contrast, the majority length of trans-NATs ranged from 201 nt to 800 nt, with an average length of 580 nt. In terms of length distribution, the most abundant bin was the 201–300 nt for cis-NATs and the 301–400 nt for trans-NATs. The lengths of cis-NATs and trans-NATs were then subjected to two sample Student’s t-test, the difference is statistically significant with *p*-value < 0.05.

The cis-NATs can be categorized into four subtypes based on their locations relative to the STs: convergent (tail-to-tail or 3’-to-3’), divergent (head-to-head or 5’-to-5’), STs containing NATs (S > N), and NATs containing STs (N > S)^39^. Since these relative locations might reveal information about the NATs’ potential roles, classification of NATs was conducted and the results are shown in Fig. 2b. A total of 373 convergent (22.9%), 614 divergent (37.7%), 526 STs containing NATs (7.2%), and 117 NATs containing STs (32.3%) were found. The most abundant type of NAT is the divergent type, different from what has been described in previous genome-wide studies on viruses, prokaryotes, and eukaryotes^40^, in which the convergent type is more abundant than the divergent type. Next we examined the relationship between the length of the overlapping regions and the total length of the corresponding STs and NATs (Fig. 2c). As shown in the figure, the overlapping regions generally represented a much larger portion of the NATs than that of the STs, suggesting that larger portions of NATs tend to overlap with the corresponding STs.

We then use a Venn diagram to show how the NATs were expressed across the three developmental stages (Fig. 2d). Using an RPKM cutoff value of 2, we found that there were a total of 1855 NATs expressed in at least one of the three development stages. Among them, 1477 (79.6%) NATs were found to be expressed in all three development stages. In contrast, 53 (2.9%), 12 (0.6%) and 54 (2.9%) NATs were expressed only in the mycelium, primordia or fruiting bodies respectively. The results suggested that most NATs were expressed across all stages and there were only a few stage-specific NATs. Because of the possible errors in the identified trans-SAT pairs, we focused our analyses only cis-SAT pairs in the following text.

### Functional enrichment analyses of the STs having NATs across all three stages

One approach to understand the potential functions of NATs is to examine if there are any functional enrichment in the SATs. For simplicity, in the following text, functional categories for a SAT pair actually mean the functional categories mapped for the ST belonging to the SAT pair. The 1630 cis-SAT pairs corresponded to 1403 STs, which were subjected to enrichment analyses for Gene Ontology (GO) terms and KEGG pathways. We retrieved the mappings of STs to GO terms and KEGG pathways from the previous study^3^. For each GO term, hypergeometric probability was calculated to obtain the probability (*p*-value) of observing more STs associated with this GO term or KEGG pathway giving the number of all genes (16127) and STs (1403). Multiple test correction was performed to calculate the False Discovery Rate (FDR, *q*-value) for each *p*-value. Detailed results can be found in Table S3 for GO terms and in Table S4 for KEGG pathways. The mappings of genes to GO terms and KEGG pathways can be found in Table S5 and Table S6 respectively.

A total of 600 of these 1403 STs were mapped to GO terms (Table S5). In total, 9 GO terms have *q*-values < 0.01 and the numbers of STs mapped to >  = 4 (Table 1). Among them, the GO terms with the largest numbers of STs mapped to are Oxidation reduction (GO:0055114, 84), Oxidoreductase activity (GO:0016491, 65), Transmembrane transport (GO:0055085, 63), and Monooxygenase activity (GO: 0004497, 48). In contrast, the ones with the smallest *q*-values are transmembrane transport (GO:0055085), monooxygenase activity (GO:0004497) and iron ion binding (GO:0005506).

**Table 1:** List of significantly enriched GO terms and KEGG pathways for SAT pairs expressed in at least one of the three developmental stages.

Category ID	Annotation	No. of CDS in the Genome	No. of STs in this Category	Q Value (FDR)
GO:0055085	Transmembrane transport	340	63	<0.0001
GO:0004497	Monooxygenase activity	258	48	<0.0001
GO:0005506	Iron ion binding	260	45	0.0005
GO:0009055	Electron carrier activity	250	43	0.0005
GO:0020037	Heme binding	257	44	0.0006
GO:0055114	Oxidation reduction	618	84	0.0006
GO:0006810	Transport	174	33	0.0006
GO:0043169	Cation binding	91	21	0.0010
GO:0016491	Oxido-reductase activity	482	65	0.0046
ko03030	DNA replication	26	10	<0.0001
ko00500	Starch and sucrose metabolism	18	7	<0.0001
ko03410	Base excision repair	18	7	<0.0001
ko00627	Aminobenzoate degradation	10	5	<0.0001
ko01120	Microbial metabolism in diverse environments	113	20	<0.0001
ko00630	Glyoxylate and dicarboxylate metabolism	16	6	0.0001
ko03420	Nucleotide excision repair	29	8	0.0001
ko01200	Carbon metabolism	65	13	0.0001
ko04141	Protein processing in endoplasmic reticulum	54	11	0.0003
ko04146	Peroxisome	54	11	0.0003
ko03018	RNA degradation	48	10	0.0004
ko00680	Methane metabolism	15	5	0.0005
ko00561	Glycerolipid metabolism	10	4	0.0006
ko01100	Metabolic	486	55	0.0007
ko00620	Pyruvate metabolism	47	8	0.0038
ko03008	Ribosome biogenesis in eukaryotes	58	9	0.0051
ko03050	Proteasome	32	6	0.0055
ko03013	RNA transport	81	11	0.0078
ko01110	Biosynthesis	189	21	0.0087

Similarly, a total of 168 of these 1403 STs were mapped to 196 KEGG pathways (Table S6). The results show that 34 KEGG pathways have *q-values* < 0.01 (Table S4). Among them, the KEGG pathways with the largest numbers of STs mapped to are Metabolic (ko01100, 55), Biosynthesis (ko01110, 21), Microbial metabolism in diverse environments (ko01120, 20) and Carbon metabolism (ko01200, 13). The pathways with smallest *q-values* are DNA replication (ko03030). This information can be used to elucidate the cellular functions of NATs in the future.

### Functional enrichment analyses of the STs having NATs in individual developmental stages

Next, we asked the question whether or not SAT pairs expressed in particular developmental stages showed any functional enrichment. We identified the STs that were expressed in each of the three stages, which were then subjected to enrichment analyses for GO terms and KEGG pathways using the same procedures described above for all STs. The results can be found in Table S7 for GO terms and in Table S8 for KEGG pathways. Overall, for GO terms, there are 11, 14 and 12 enriched terms found in the mycelia, primordia and fruiting bodies with *q-*value < 0.01, respectively, And there are 8, 6 and 11 terms found in the three stages with 0.01 < *q-*value < 0.05. For KEGG pathways, there are 2, 5, 2 pathways in the mycelia, primordia and fruiting bodies with *q-*value < 0.01, respectively. And there are 12, 9 and 9 pathways in the three stages with 0.01 < *q-*value < 0.05.

We then compared the significantly enriched functional categories of SAT pairs among the three developmental stages. As shown in Table 2, there are four GO terms and three KEGG pathways showed differential enrichment among the three developmental stages. Among them, GO terms GO:0003676, GO:0006066 and GO:0016614 were found to be significantly enriched in the fruiting bodies. One GO term, GO:0043169 was found to be enriched in both primordia and fruiting bodies. One KEGG pathway, ko00680 was found to be enriched in the primordia only. In contrast, two KEGG pathways, ko01200 and ko03018 were found to have been significantly enriched in both mycelia and primordia. Taken together, these results suggest that NAT might play important roles in a developmental-stage specific manner.

**Table 2:** List of differentially enriched GO terms and KEGG pathways for SAT pairs expressed in particular developmental stages. N: not significant.

ID	Annotation	Mycelia	Primordia	Fruiting Bodies
GO:0003676	Nucleic acid binding	^a^N	N	0.01 < q < 0.05
GO:0006066	Alcohol metabolic process	N	N	0.01 < q < 0.05
GO:0016614	Oxidoreductase activity, acting on CH-OH group of donors	N	N	0.01 < q < 0.05
GO:0043169	Cation binding	N	q < 0.01	q < 0.01
ko00680	Methane metabolism	N	0.01 < q < 0.05	N
ko01200	Carbon metabolism	0.01 < q < 0.05	0.01 < q < 0.05	N
ko03018	RNA degradation	0.01 < q < 0.05	0.01 < q < 0.05	N

^a^N: not significant.

### Validation of ssRNA-seq results using ssRT-qPCR experiments

**Figure 3:**
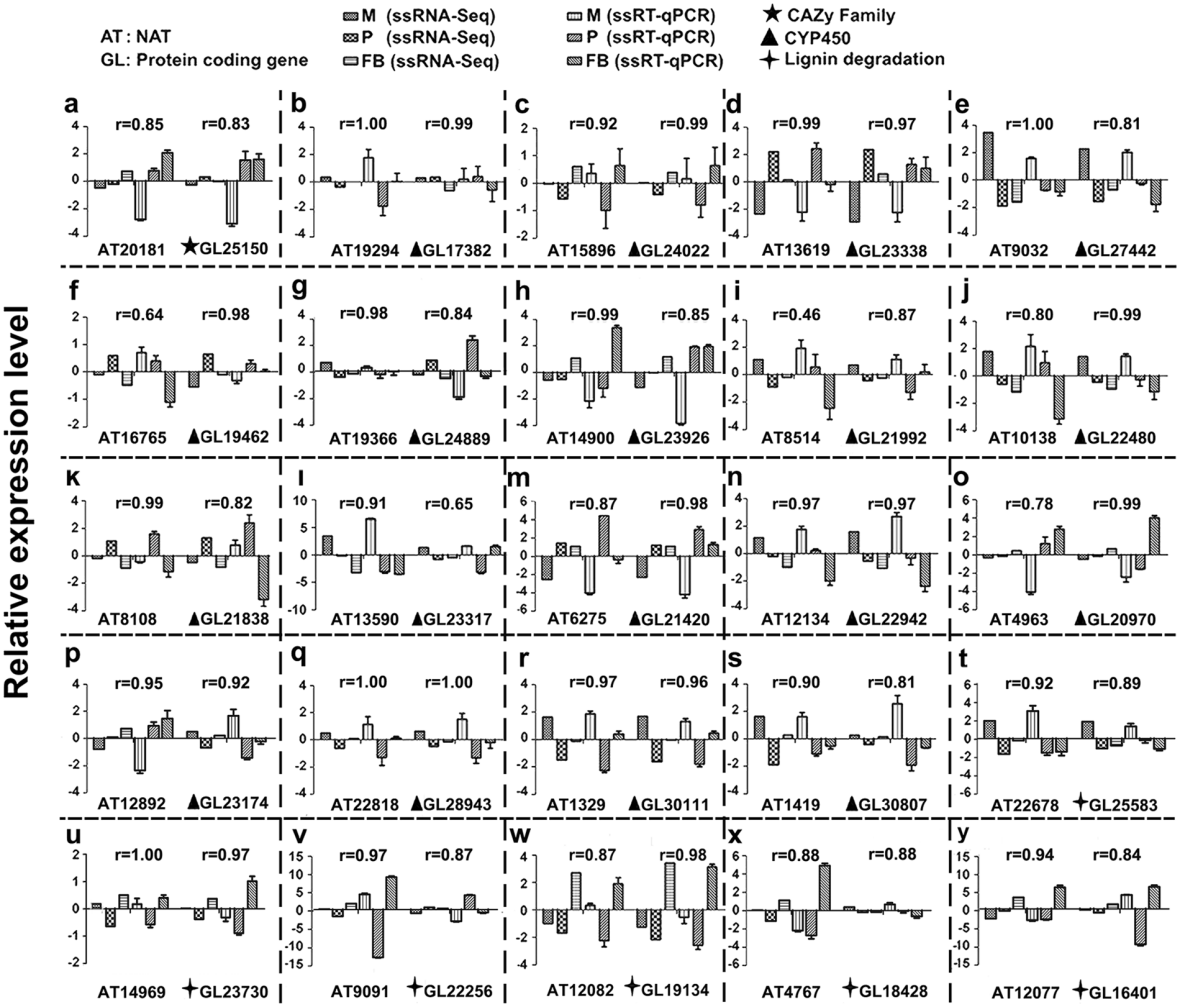
Validation of ssRNA-seq results with ssRT-qPCR experiments. Twenty-five SAT pairs were selected and subjected to ssRT-qPCR analysis. The expression levels obtained from the ssRNA-seq and ssRT-qPCR experiments for these SAT pairs are shown in panels a-y respectively. For results from both experiments, the expression levels at each developmental stage were normalized to the mean expression levels across the three stages. In each panel, the names of the NAT and ST are shown at the bottom. The X axis indicates the conditions under which the expression levels were measured, including the developmental stages and technologies. The Y axis indicates the relative expression levels obtained from the ssRNA-seq and ssRT-qPCR experiments. The error bar for the ssRT-qPCR results corresponds to the variations among the three biological replicates, each of which has three technical replicates. The gene families to which the STs belong were indicated in front of the gene names. The details of the genes can be found in Table S9. ▴: CYP450; : Lignin degradation; ★: CAZy family. M: mycelia; P: primordia; FB: fruiting bodies.

To verify the ssRNA-seq results, we applied ssRT-qPCR technology to quantify the expression levels of a selected set of NATs and their corresponding STs. The genes chosen for this comparison are those members of the CYP450 superfamily and those participate in the lignin degradation pathway (Table S9). For each transcript, the expression levels obtained from the ssRNA-seq and ssRT-qPCR experiments at particular stages were log transformed and then normalized against the average expression levels across the three stages (Table S10). The normalized expression levels of STs and their NATs were shown in Fig. 3, panels a-y. In each panel, the expression levels at three developmental stages obtained using two different technologies for the corresponding pair of NAT and ST were shown side-by-side. Furthermore, we calculated the Pearson’s correlation coefficients (*r*) between the expression profiles obtained from the two technologies. The significance of the correlation was tested with the null hypothesis *r* = 0. As shown in Table S10, all pairs of data have *p*-values < 0.01, suggesting that the null hypothesis should be rejected and thus the expression profiles are significantly correlated. In particular, 30 out of the 50 ST or NAT transcripts (60%) had *r* values ≥ 0.9. Taken together, results from our RNA-seq experiments correlated well with those of the ssRT-qPCR experiments.

### Expression correlations of STs and NATs

**Figure 4:**
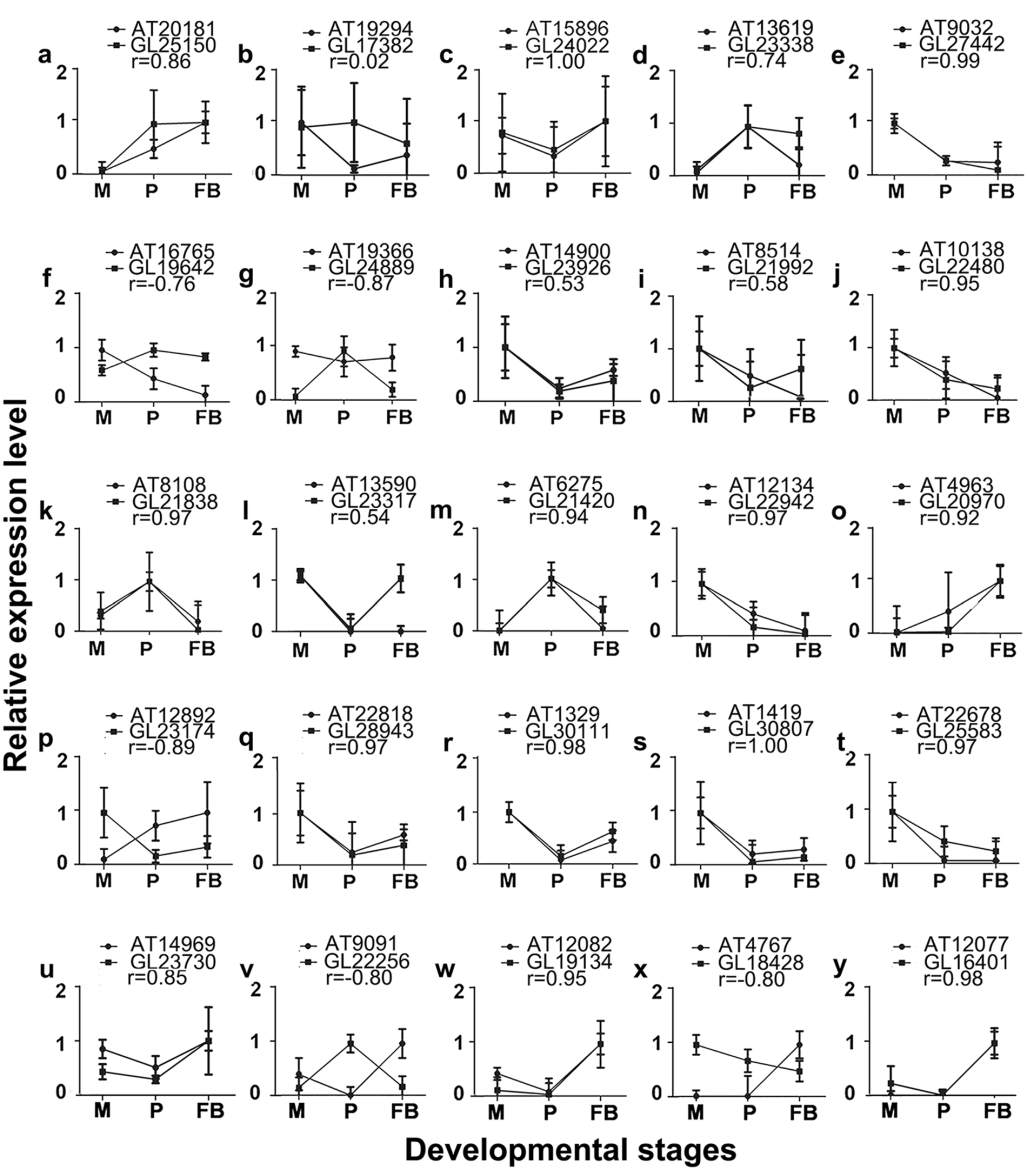
Correlation of expression profiles for NATs and STs. Panels a-t show the expression files of STs and NATs from 25 SAT pairs also described in Fig. 3. For each panel, the X axis indicates the corresponding developmental stage. The Y axis indicates the relative expression levels. The names of the ST, the NAT and the Pearson’s correlation coefficient between their expression profiles are shown at the top. The error bar denotes the standard deviations among the three biological replicates, each of which had three technical replicates in the ssRT-qPCR experiments. The details of the genes can be found in Table S9. M: mycelia; P: primordia; FB: fruiting bodies.

Assuming that high degrees of expression correlation of STs and NATs might provide some clues for any potential functional relationships between them, we compared the developmental expression profiles of STs and NATs in the 25 SAT pairs used for the validation of the RNA-seq results (Fig. 3). First, the expression levels of each ST and NAT at any particular developmental stage were log transformed and then normalized to their corresponding maximum expression level. Then, the Pearson’s correlation coefficients for the expression profiles of STs and NATs belonging to the same SAT pairs were calculated and the statistical significance of the correlation was tested (Table S11). The panels in Fig. 4 were sorted alphabetically based on the functional annotation of the STs (Table S9).

**Figure 5:**
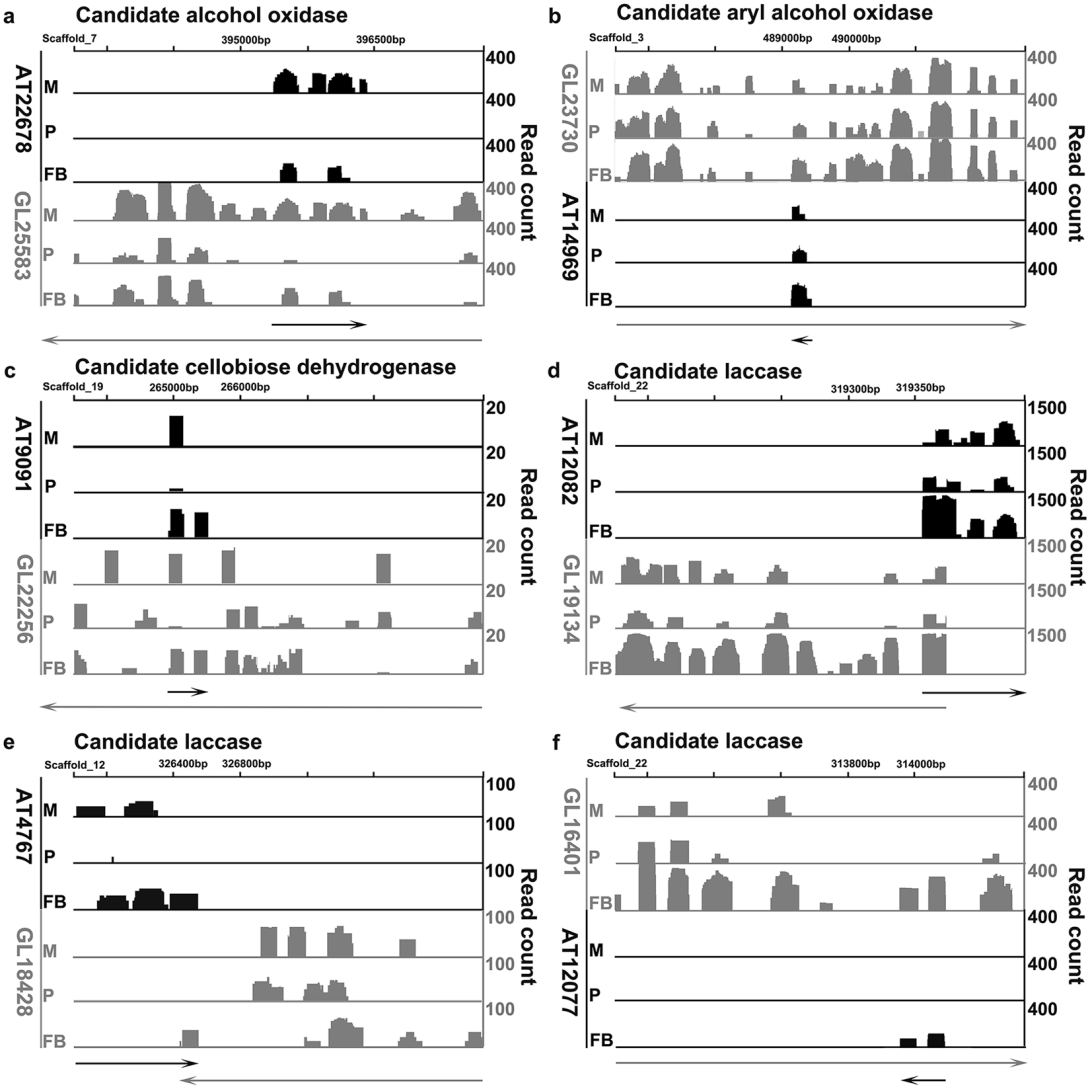
Gene structures and relative expression levels of the six SATs involved in lignin degradation. For each panel, the “X” axis shows the chromosomal region with the scaffold number and coordinates, while the “Y” axis shows the read counts. The transcript expressed on the positive strand is shown on the top and the transcript expressed on the negative strand is shown on the bottom. The read counts for the STs are shown in black and those for the ATs are shown in gray. The name of enzyme encoded by the ST was shown above each panel. The names for the ST and NAT were shown to the left of each panel. The details of the genes can be found in Table S9. M: mycelia; P: primordia; FB: fruiting bodies.

As shown, five of the SAT pairs had positive correlation coefficients between 0 and 0.8 (Fig. 4b,d,h,i,l). One had negative correlation coefficient between 0 and -0.8 (Fig. 4f). Fifteen SAT pairs had positive correlation coefficient > 0.8 (Fig. 5a,c,e,j,k,m,n,o,q,r,s,t,u,w,y). In contrast, four SAT pairs had negative correlation coefficient < -0.80 (Fig. 4g.p.v.x.). The significance of these *r* values was tested with the null hypothesis *r* = 0. For the SAT pairs having coefficient > 0.8 or < -0.8, only one pair of SAT: AT19294 and GL17382 had a *p*-value > 0.05, for which the null hypothesis could not be rejected. In addition, two pairs of STs and NATs showed 0.01 < *p*-values < 0.05. All other pairs of STs and NATs have *p*-values < 0.01 (Table S11). The high degree of correlation between the expression profiles of STs and NATs suggests that NATs might play important roles in the expression regulation of the corresponding STs.

#### NATs involved in the expression regulation of lignin-modifying enzyme genes

In total, NATs were found for six putative lignin-modifying enzyme (LME) genes. We then carried out detailed analyses of these 6 LME genes in order to (1) validate the expression analyses shown in Figs 3 and 4; (2) examine possible roles NATs might play in the expression regulation of STs; and (3) determine if NATs are involved in lignin-degradation in *G*. *lucidum*. We examined the structures and expression levels of these six SAT pairs in detail (Fig. 5). The transcripts located on the positive strands were shown on the top of the panels, and those on the negative strands on the bottom. The first SAT pair contained AT22678 and GL25583 (Fig. 5a) with the NAT embedded in the ST. GL25583 encoded a candidate alcohol oxidase (Supplementary File 3a). Alcohol oxidase catalyzed the reaction that converts a primary alcohol and O_2_ to an aldehyde and H_2_O_2_^41^. Both GL25583 and AT22678 appeared to have introns. Both GL25583 and AT22678 were expressed the highest in the mycelia and their expression levels went down to the lowest in the fruiting bodies. The expression profiles of GL25583 and AT22678 were positively correlated with an *r* of 0.97 (Fig. 4t).

The second SAT pair contained AT14969 and GL23730 with the NAT embedded in the ST (Fig. 5b). GL23730 encoded a candidate aryl alcohol oxidase (Supplementary File 3b). This enzyme is an extracellular flavor-protein providing the H_2_O_2_ required by ligninolytic peroxidases for fungal degradation of lignin^42^. While GL23730 appeared to have intron, AT14969 did not. Both ST and NAT were expressed comparably low in the mycelia and primordia, and then the expression levels increased to the highest levels in the fruiting bodies. Their expression profiles were positively correlated with an *r* of 0.85 (Fig. 4u).

The third SAT pair contained AT9091 and GL22256 with the NAT embedded in the ST (Fig. 5c). GL22256 encoded a candidate cellobiose dehydrogenase (CDH, Supplementary File 3c). CDH is an extracellular enzyme produced by various wood-degrading fungi. It oxidizes soluble cellodextrins, mannodextrins and lactose efficiently to their corresponding lactones by a ping-pong mechanism using a wide spectrum of electron acceptors including quinones, phenoxyradicals and etc^43^. Both AT9091 and GL22256 appeared to have introns. Interestingly, GL22256 appeared to have different isoforms among the three developmental stages. The expression profiles of AT9091 and GL22256 were negatively correlated with GL22256 expressed the highest in the primordia and AT9091 expressed the highest in the fruiting bodies, suggest that GL22256 might function specifically in the primordia (Fig. 4v). Interestingly, the expression levels of this SAT pair were very low with the read counts near 20, and their expression profiles were negatively correlated with an *r* of −0.80. The implication of low expression level remained to be investigated.

All the other three SAT pairs related to laccase (Supplementary Files 3d-f). Laccase belongs to the small group of enzymes called the blue copper oxidases and is widely distributed in higher plants and fungi, it is a versatile enzyme involved in lignin metabolism^44^. The fourth SAT pair contained AT12082 and GL19134 in a divergent configuration (Fig. 5d). Both AT12082 and GL19134 appeared to have introns. They were expressed lowly in the mycelia and primordia and highly in the fruiting bodies. The expression profiles were positively correlated with an *r* of 0.95 (Fig. 4w). The expression levels of this SAT pair were the highest among these six SAT pairs with the read counts near 1500.

The fifth SAT pair contained AT4767 and GL18428 in a convergent configuration (Fig. 5e). The NAT appeared to be alternative spliced in the mycelia and fruiting bodies. The expression level of GL18428 was the highest in the mycelia and then decreased gradually from the primordia to the fruiting bodies. In contrast, the expression level of AT4767 was highest in the fruiting bodies and the expression profiles of the ST and NAT were negatively correlated with an *r* of -0.80 (Fig. 4x). As a result, this candidate laccase gene was most likely to function in the mycelia, whose expression was then down-regulated in the fruiting bodies.

The last SAT pair contained AT12077 and GL16401 with the NAT embedded in the ST (Fig. 5f). The expression patterns of the ST and NAT as well as the correlation of their expression profiles were very similar to those of the fourth SAT pair. Both the ST and NAT were expressed at very low levels in the mycelia and primordia and then the expression levels were increased significantly in the fruiting bodies. The expression profiles of the ST and NAT were highly positively correlated with an *r* of 0.98 (Fig. 4y). Examination of the expression profiles of these six SATs revealed complex interactions among STs and NATs that will be discussed below.

## Discussions

### Genome-wide identification of NATs

Species of *Ganoderma* have attracted world-wide attention from three aspects: as therapeutic fungal bio-factories^45^, as plant pathogens^46^ and as “bio-bags” of ligninolytic enzymes^47^. To fully realize the potential of *Ganoderma* species in drug discovery and biofuel production, we have carried out a series studies to obtain the complete genome, transcriptome, lincRNA from *G*. *lucidum* ^4^. As a subset of lncRNA, it has been well-accepted now that NATs are important gene expression regulators in a wide variety of organisms including fungi^48^. As a result, discovery and characterization of NATs in *G*. *lucidum* would facilitate not only the discovery of genes that are related to its medicinal, plant-pathogenic and saprotrophic characteristic, but also the elucidation of their mechanisms of actions.

In the present study, we carried out a systematic analysis of NATs in three developmental stages of *G*. *lucidum* using ssRNA-seq technology and a self-developed computational pipeline. In total, 1613 cis-NATs and 244 trans-NATs were identified. We then characterized these NATs in detail in terms of their length distribution and classification. Next, we validated the ssRNA-seq results using ssRT-qPCR experiments. Next, we examined whether or not SAT pairs were associated with particular functional categories in terms of GO and KEGG pathways across all three developmental stages or in particular stages. Lastly, by analyzing the expression correlation of 25 SAT pairs that encode CYP450 proteins or LMEs, we explored if NATs were likely to regulate the expression of the corresponding STs. A total of 15 SAT pairs had NATs and STs significantly positively correlated, whereas 4 SAT pairs were significantly negatively correlated. The above results suggest that (1) NATs are involved in particular cellular functions; (2) NATs can be either stabilizers or repressors of STs; and (3) the interactions between STs and NATs can be rather complex (see below for more discussions).

### Non-random Association of NATs to particular functional groups of STs across all stages and in particular stages

With 1613 cis-NATs and 1403 STs, it is interesting know whether or not these cis-NAT were randomly associated with the STs. If not, what kinds of STs were associated with cis-NATs more frequently. We answered this question first by enrichment analyses of STs having NATs. STs belonged to a wide variety of functional categories were found to have NATs associated (Table 1), particularly those involved in transmembrane transport, monooxygenase activity, DNA replication and etc. Many of these associations were statistically significant (Table 1, Tables S4 and S6).

We then asked a more specific question, is the non-random association of NATs to STs specific to particular development stage? To answer this question, enrichment analyses were carried out for STs and NATs expressed in particular stages. In the mycelia, primordia and fruiting bodies, STs of different functional categories were found to have NATs (Table 2, Tables S8 and S9). For example, SAT pairs associated with three GO Terms were significantly enriched in the fruiting bodies. The first GO term, GO:0003676, represents Nucleic acid binging, which are represented by mostly DNA helicases and zinc ion binding proteins (Table S2). These results are consistent with the production of spores in the fruiting bodies. The other two GO terms are GO:0006066 (Alcohol metabolic process) and GO:0016614 (Oxdoreductase activity). Genes mapped to these two GO terms are mostly flavor-oxidase likely to be involved in the lignin degradation pathway. Actually, in the laboratory, while the mycelia can be cultured on the PDA medium, the primordia and fruiting bodies can only be developed on logs. So the expression up-regulation of LMEs is actually anticipated in the fruiting bodies. Taken together, the associations of NATs to STs were not random for particular stages. In the fruiting bodies, the enrichment of particular groups of STs with NATs is consistent with the physiological behavior of the fruiting bodies. These results raised the hypothesis that the expression regulations of STs by NATs are function and developmental stage specific.

### Complex interactions between STs and NATs

Having found that NATs might regulate the expression of STs in function-category and developmental-stage specific manners, the next question is how NATs regulate the expression of STs. Previous studies have showed that NATs can control nearly every level of gene regulation: pre-transcription^49^, transcription^50^, post-transcription in nucleus^51^ and post-transcription in cytoplasm^52^. The regulation can be mediated by molecular interactions among DNA–RNA, RNA–RNA, or protein–RNA^13^. On one hand, the single-strand characteristics of RNA allow it to bind DNA or another RNA molecule through complementing base-pairing. On the other hand, NATs can form particular structures, which can serve as molecular decoys to retain proteins and preventing their functions, or act as platforms for the assembly of protein complexes^53–57^.

The mechanisms by which NATs regulate the expression of STs can be learned from the types and degrees of correlations between their expression profiles. A positive expression correlation may result from the increased stability of the ST mRNA by binding with the NAT. In this case, the NAT acts as a “stabilizer”. In contrast, a negative expression correlation suggests that the NAT repress the expression of the ST. In this case, the NAT acts as a “repressor”. There are three main mechanisms that NATs might act as repressors, these include transcriptional interference^58^, chromatin remodeling^59^, and RNA marking^60, 61^. However, a positive expression correlation might simply result from the opening-up of the chromatin region where the SAT pair locates, without reflecting any direct interaction between them. In this case, the ST and NAT are “co-regulatees”, which means that they are co-regulated by a common upper level regulator. While it is commonly assumed that the NATs regulate the expression of their corresponding STs, the possibility can’t be excluded that the STs might also regulate the expression of their corresponding NATs inversely.

Using the types and degrees of expression correlations to deduce the exact interaction between a ST and a NAT also has limitations. For example, a positive correlation between a ST and NAT can result from (1) the ST and NAT being co-regulatees; (2) the NAT acting as a “stabilizer”; or (3) both (1) and (2). As result, future experiments are required to determine exactly how a ST and a NAT actually interacts. For example, one can “knock-in” and “knock-out” a NAT and then observe the change in the expression level of its ST. Alternatively, a time-course study can be used to study how the expression levels of a ST and a NAT change over time^62^.

Although the correlation coefficients for the expression profiles of all SAT pairs were calculated (Table S2), the expression levels of only 25 SAT pairs were validated with ssqRT-PCR experiments (Fig. 3). As a result, only the expression pattern of these 25 SAT pairs can be used to learn the potential interactions among STs and NATs (Fig. 4). Complex potential interactions between STs and NATs were revealed in Fig. 4. Firstly, the expression levels of STs and NATs alone varied among different developmental stages (e.g., Fig. 4a). Secondly, the expression levels of the ST and NAT in a particular stage varied significantly (e.g., Fig. 4v, stages “P and FB”). Thirdly, The expression patterns of genes involved in the same functional groups (e.g., laccase) varied significantly (Fig. 4w,x,y). Fourthly, the correlations of the expression profiles for a ST and a NAT varied significantly, from highly negative (*r* = -0.89 in Fig. 4p) to highly positive (*r* = 1.00 in Fig. 4c). So the expression regulation might be mediated through a complex multi-layered regulatory network.

Another interesting question is whether or not the correlation of expression profiles for a SAT pair is related to the pair’s configuration. For the 25 SAT pairs shown in Figs 3 and 4, 2 of 3 (66.7%) SAT pairs belonging to the convergent type were negatively correlated. In contrast, 11 of 13 (85%) SAT pairs belonging to the divergent type were positive correlated. And 7 of 9 (78%) SAT pairs belonging to the S > N (ST containing NAT) type were positive correlated (Table S9). It should be pointed out that none of the 25 SAT pairs belonged to the N > S (NAT containing ST) type. These results suggest that the convergent type was more frequently associated with negative correlation. It is possible that two “convergent” transcriptional machineries would collide into each other, leading to the expression repression of one of the transcript. As a result, the convergent configuration is more frequently associated with negative expression correlation. Additional experiments are needed to test if the above speculation is correct.

Several overall observations provided additional clues for the potential roles of NATs in *G*. *lucidum*. Firstly, while most NATs were found to locate at the 3’ end of STs previously^63^, more NATs were found to locate at the 5’ end of the STs in our study (Fig. 2b). Secondly, we found more SAT pairs that are positively correlated than those that are negatively correlated. thirdly, the expression levels of STs with NATs were generally higher than those of STs without NATs (Table S2), consistent with the previous observations in Mouse^64^. All these three lines of observations favor the hypothesis that more NATs might act as the co-regulatees of their STs in *G*. *lucidum*. In another word, the positive correlation observed for STs and NATs are more likely to result from the opening-up of the chromatins in the regions. This genome-wide analysis of NATs has provided a wealth of information regarding the regulatory network in *G*. *lucidum*. Additional experiments are needed to illustrate the exact mechanisms of actions of NATs.

### Technical considerations for this study

There are several technical limitations that might contribute to potential errors in the analysis that must be taken into account when performing downstream analyses or experiments. First, based on our definition, STs encoding protein and NATs are complementary to STs that do not code for proteins. With this definition, we eliminated from our analyses the pairs whose complementary transcripts were either protein-coding or non-coding. Secondly, inherent bias in the RNA-seq technology may have affected the results. Since lncRNAs in general are expressed at about one tenth of the ST, some NATs that were expressed at very low concentrations may not have been identified, leading to the underestimation of NATs. Thirdly, the samples used for this study are from three developmental stages: mycelia, primordia and fruiting body. As a result, we can only identify NATs that are expressed in at least one of the three developmental stages. And we might miss those NATs that were expressed transitively, but play important functions. In the future, performing additional transcriptome analysis at various conditions would likely to reveal additional NATs. Fourthly, because some regions of the NATs were not covered by the reads, a single NAT may have been broken into multiple NATs, leading to the overestimation of the numbers of NATs discovered. In fact, our analysis of the ratios between NATs and STs revealed multiplex relationships such as 1:n, n:1, and n:n mapping ratios. The n:1 relationship may have resulted from the incomplete coverage of particular antisense transcripts. Thus, obtaining the full-length NATs has to be a prerequisite for the determination of their functions. Last, the identification of trans-SAT pairs was also dependent on the similarity cutoff used for the complementary regions.

## Materials and Methods

### Strain and Culture Conditions

The dikaryotic strain *G*. *lucidum* CGMCC5.0026 was obtained from China General Microbiological Culture Collection Center (Beijing, China). The mycelia were cultivated in potato dextrose agar (PDA) medium in the dark at 28 °C for 7 days. The primordia and fruiting bodies were cultivated on *Quercus variabilis* logs as described previously^3^.

### ssRNA-seq analysis

Samples were collected from mycelia, primordia, and fruiting bodies, and then subjected to RNA extraction using the plant total RNA extraction kit (Tiangen, China). Genomic DNA contaminations were removed using RNase-free DNase I (Tiangen, China). RNA integrity and quality were examined on Bioanalyzer (Agilent). The strand-specific RNA-library was constructed following the SMART-seq protocol as describe previously^65^. The resulting cDNA was cleaved into 300 bp to 500 bp fragments to construct the sequencing libraries, then sequenced using an Illumina Hiseq 2500 platform. In order to get a relative high through output sequence data, the resulting raw reads were subjected to the following filtering process using FASTX-Toolkit (v0.0.14, http://hannonlab.cshl.edu/fastx_toolkit/). First, adapters were removed from the read sequences. Then reads containing at least ten percent of Ns were removed. Last, low-quality reads, such as those with more than half of the bases having a Phred base quality score of less than 5, were removed. The raw sequence reads were deposited in GenBank under the accession number (GSE94910). Data were analyzed using the Tuxedo packages (v1.3.2)^35^. First, the raw reads were mapped to the reference genome using TopHat^35^. Then the transcripts were reconstructed through Cufflinks using default parameters. The mapping of RNA-seq reads to the reference genome was visualized using Tablet (v1.12.12.05)^66^.

### Computational identification of NATs

We developed a reference-based computational pipeline for NAT identification from RNA-seq data (Fig. 1). The pipeline starts with the clustering of all transcripts identified using the Tuxedo package and the predicted genes into transcript units, which are defined as a combination of all overlapping transcripts and genes on the same strand. The following analyses were carried out by three modules. The first two modules identified candidate cis-NAT and candidate trans-NAT respectively. And the third module passed the output of the first two modules to five filtering steps. In particular, the module I identified all transcripts on the opposite strand of the predicted genes whose overlapping region had a length of ≥ 10% of either transcript as described previously^39^. The resulting transcripts were called candidate cis-NATs. The module II contains three steps also as described previously^39^. In step 1, the “high-coverage 100-nt pair” was identified by comparing all identified transcripts and all predicted genes using the BLASTN program. If the two transcripts had a continuous complementary region longer than 100 nt, they were defined as a “100-nt” pair. If the complementary region of the two transcripts identified by the BLAST program was longer than 50% of either transcript, they were classified as ‘high-coverage’. In step 2, the trans-SAT pairs obtained from step 1 were examined to determine if they could melt into RNA–RNA duplexes in the complementary regions using DINAMelt^67^. In step 3, if the annealed region identified by DINAMelt coincided with the BLASTN results, and if any bubble within the annealed region had a length not longer than 10% of the region’s length, the corresponding antisense transcripts were selected as candidate trans-NATs.

The candidate NATs were processed further with the module III, which can be divided into five steps. In step 1, those transcripts whose lengths were shorter than 200 bp were removed. In step 2, those transcripts encoding ORFs of more than 100 amino acids were removed as these transcripts are more likely to encode proteins^68^. In step 3, the remaining transcripts were searched against the Nr database using a cutoff e-value of 1e-3. In step 4, all transcripts were subjected to analysis with Coding Potential Calculator (CPC), in which transcripts with scores > 0 were removed^36^. In step 5, to exclude small RNAs and housekeeping lncRNAs such as tRNAs, snRNAs, and snoRNAs, the remaining antisense transcripts were compared to housekeeping lncRNA databases and miRBase by BLASTN using a cutoff e-value of 1e-10. The remaining transcripts were output as cis-NATs and trans-NATs, respectively. The scripts implementing this pipeline will be provided upon request.

### Functional enrichment analysis of STs

All STs were subjected to Gene Ontology analysis as described previous^69^. To perform functional enrichment analysis, the mapping of all *G*. *lucidum* genes to GO terms was retrieved from the previous study^3^. For each GO term, we calculate the hypergeometric probability, which is used to determine whether the GO term is enriched in STs beyond what might be expected by chance^70^. The probability is calculated using formula (1):

1 $$\begin{array}{rcl}P(x=i) & = & \frac{(\frac{n}{i})(\frac{m}{N-i})}{(\frac{m+n}{N})}\\  & = & \frac{{\rm{m}}!n!N!(m+n-N)!}{i!(n-i)!(m+i-N)!(N-i)!(m+n)!}\end{array}$$

Here, “P” is the hypergeometric probability. “n” is the number of all genes in *G*. *lucidum* that are associated with the GO term. “m” is the number of all genes in *G*. *lucidum* that are not associated with the GO term. “N” is the number of all STs. And “x” is number of STs that are associated with the GO term. The *p*-value was then subject to multiple test correction and the False Discovery Rate (FDR, *q*-value) was calculated and used to determine the statistical significance of the enrichment for the GO term.

### Strand-specific reverse transcription quantitative real-time PCR (ssRT-qPCR) analysis

ssRT-qPCR experiments were used to determine the expression levels of STs and NATs as described previously^71^. Twenty-five SAT pairs were selected based on their functional annotation (Table S9). The primers were designed by the IDT web server (http://sg.idtdna.com/scitools/Applications/RealTimePCR/). To ensure the specificity of the primer sequences, the primer sequences were searched against the reference genome sequences; those that mapped to multiple regions were discarded. To distinguish STs and NATs, 18 to 20 nucleotide tags unrelated to *G*. *lucidum* were added to the corresponding primers. The tag sequence for NATs was “GCTAGCTTCAGCTAGGCATC”, and that for STs was “CCAGATCGTTCGAGTCGT”. The primer sequences are listed in Table S12,. The genes ubiquitin and glyceraldehyde-3-phosphate dehydrogenase were used as the internal control as described previously^4, 72^. Total RNA was extracted from the mycelia, primordia, and fruiting bodies using the plant total RNA extraction kit (Tiangen, China), and then digested with RNase-free DNase I (Tiangen, China) to remove contaminating genomic DNA. A NanoDrop 2000C spectrophotometer (Thermo Scientific, Wilmington, DE, USA) was used to measure the RNA concentration, whereas the integrity of RNA was analyzed on a 1% agarose gel. Reverse transcription was performed on 1.0 µg total RNA in 10 µl volume for each sample using RT-PCR Kit (TransGen China). The conditions of the RT reaction were as follows: 45 °C for 35 min, 85 °C for 5 min. The resulting cDNA samples were diluted to final volumes of 30ul with sterile water.

For each transcript, the qPCR was carried out with three biological replicates, and each biological replicate had three technical replicates. Each qPCR reaction consisted of 1 µl diluted cDNA, 10.0 µM forward primer, 10.0 µM reverse primer, 10 µl 2x TransStart Top Green qPCR SuperMix, and 0.4 µl passive reference dye (50x) for a final volume of 20 µl. Analysis was conducted on a ABI 7500 Fast instrument (Applied Biosystems) using the following cycle conditions: 94 °C for 30 sec, and 40 cycles of 94 °C for 5 sec and 60 °C for 34 sec. Amplification specificity was assessed by melting curve analysis, which was performed using the following conditions: 95 °C for 15 sec, 60 °C for 1 min, and 95 °C for 15 sec. The relative abundance of each transcript under test was obtained using the comparative Cq method^73^. After the data were obtained, the expression level was normalized against the maximum value among the three stages. The normalized expression profiles were then subjected to Pearson’s correlation coefficient calculation.

### Statistical analyses

Statistical significance of Pearson’s correlation coefficient was performed by calculating the t value using formula (2):

2 $${\rm{t}}=\frac{r}{sqrt[(1-{r}^{2})/(N-2)]},$$

as described by VassarStats (http://www.vassarstats.net/). Here, “*r*” is the Pearson’s correlation coefficient and “N” is sample size. Directional *p*-value was then looked up for the given t value and N. The statistical significances of the differences between the lengths of cis-NATs and trans-NATs, were tested using Students’ t test implemented in the JMP software (version 10, SAS, Cary, North Carolina).

## Electronic supplementary material

Supplementary Information

Table S1

Table S2

Table S3

Table S4

Table S5

Table S6

Table S7

Table S8

Table S9

Table S10

Table S11

Table S12

Supplementary File 1

Supplementary File 2

Supplementary File 3a

Supplementary File 3b

Supplementary File 3c

Supplementary File 3d

Supplementary File 3e

Supplementary File 3f
