# Supplementary material for: Genome-wide Identification and Characterization of Natural Antisense Transcripts by Strand-specific RNA Sequencing in Ganoderma lucidum
Source: Sci Rep. 2017 Jul 18;7:5711. doi: 10.1038/s41598-017-04303-6 (PMC5515960; doi:10.1038/s41598-017-04303-6)
Supplement: Supplementary file 1 — Supplementary Information [file 41598_2017_4303_MOESM1_ESM.pdf]

Supplementary Information

Genome-wide Identification and Characterization of Natural Antisense Transcripts by Strand-specific RNA Sequencing in *Ganoderma lucidum*

Junjie Shao#, Haimei Chen#, Dan Yang, Mei Jiang, HuiZhang, Bin Wu, JianqinLi, LiChai Yuan, Chang Liu&

Key Laboratory of Bioactive Substances and Resource Utilization of Chinese Herbal Medicine from Ministry of Education, Institute of Medicinal Plant Development, Chinese Academy of Medical Sciences, Peking Union Medical College, Beijing 100193, P.R.China

#contributed equally
&Corresponding Author: CL: Tel: +86-010-57833111; Fax: +86-10-62899715; Email: cliu6688@yahoo.com.

JJS:shaojie415@126.com
HMC:hmchen@implad.ac.cn
DY:dyang@implad.ac.cn
MJ:mjiang0502@163.com
HZ:18238801021@163.com
BW:bwu@implad.ac.cn
JQL: liqlcglff@126.com
LCY:lcyuan@implad.ac.cn
CL: cliu6688@yahoo.com; cliu@implad.ac.cn

Figure S1 Analysis the connectivity of adjacent NATs using strand-specific RT-PCR. Two genes were chosen for this analysis. (A) The structure of gene GL23730, the relative position of its two NATs: AT14969 and AT14972, the relative position of the forward and reverse primers were shown; (B) Strand-specific RT-PCR with the primers indicated in (A), lane 1: Standard 5000bp DNA marker, lane 2-4 indicate different combinations of materials and experimental procedures; (C) The structure of gene GL16401, the relative position of its two NATs: AT12077 and AT12078, the relative position of the forward and reverse primers; (D) Strand-specific RT-PCR with the primers indicated in (A), lane 1: Standard 1000bp DNA marker, lane 2-4 indicate different combinations of materials and experimental procedures; RT: Reverse Transcription. ★represents the bands predicted assuming the two NATs were connected. ✦ indicates a band having a size that is different from those predicted assuming the two NATs were connected.

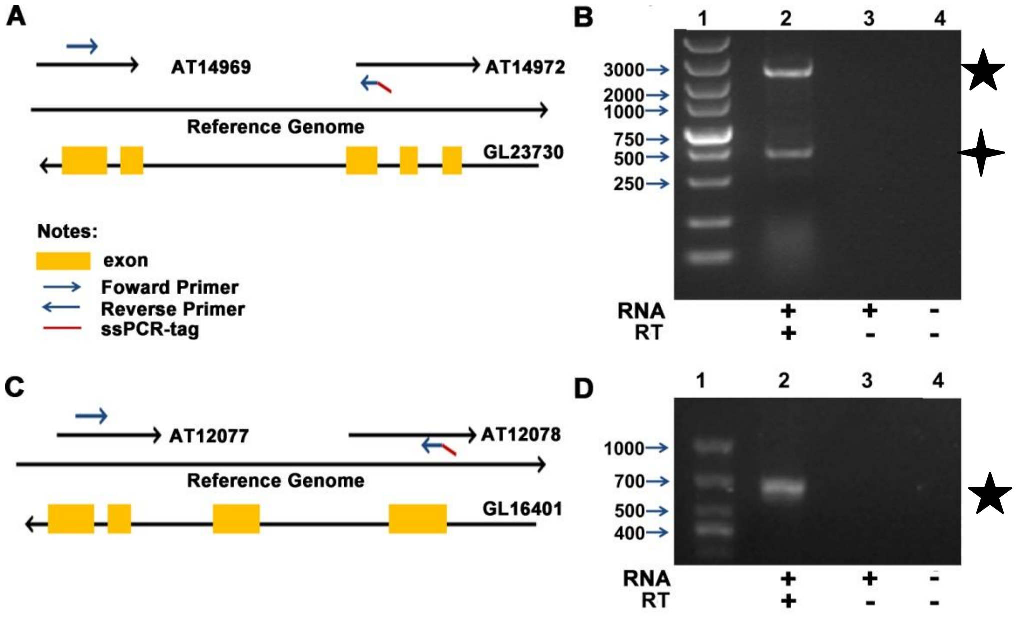

Table S1 Summary of RNA-seq data

Table S2 Detailed information of SAT pairs identified in this study

Table S3 Enrichment Analysis of STs based on GO terms

Table S4 Enrichment analysis of STs based on KEGG pathways.

Table S5 List of genes and their associated GO terms

Table S6 List of genes and their associated KEGG pathways

Table S7 Differential enrichment analysis of STs for GO terms across the three developmental stages

Table S8 Differential enrichment analysis of STs for KEGG pathways across the three developmental stages

Table S9 List of SAT pairs selected for ssRT-qPCR analysis

Table S10 Correlation of expression profiles obtained by ssRNA-seq and ssRT-qPCR experiments

Table S11 Correlation of expression profiles of STs and NATs across the three developmental stages

Table S12 Primers used in this study

Supplementary File 1 Sequences of cis-NATs identified in this study

Supplementary File 2 Sequences of trans-NATs identified in this study

Supplementary File 3a BLAST search results for GL25583

Supplementary File 3b BLAST search results for GL23730

Supplementary File 3c BLAST search results for GL22256

Supplementary File 3d BLAST search results for GL19134

Supplementary File 3e BLAST search results for GL18428

Supplementary File 3f BLAST search results for GL16401
