# Supplementary material for: Genome-wide Identification and Characterization of Natural Antisense Transcripts by Strand-specific RNA Sequencing in Ganoderma lucidum
Source: Sci Rep. 2017 Jul 18;7:5711. doi: 10.1038/s41598-017-04303-6 (PMC5515960; doi:10.1038/s41598-017-04303-6)
Supplement: Supplementary file 14 — Supplementary File 1 [file 41598_2017_4303_MOESM14_ESM.doc]

>AT3098

CACGGTGTGCAAGTTCACAACATCTATGATTACAACAGACCACTAGTATCTTCAACACGGAGTGTACAGAATGTCCGTAGGGTATGCAACGTGTTAACGACCGGAAGTTATTGACAATACAATCCCAGGCCTTTAGTTGGTCGTGGCGGTCGTGTACTGGGTGGTCGCGAGGCCGACCTGGTTGTTCGTCACGTCGTACGCGAGGTAGAACCGCTCGAGCCACGAGTAGCCGTTGATGAAGTCGAGGCCCTGGCCGCTGTTCGAGCCGAGGTCCGCGACG

>AT3102

CCGACTAGAGAATTCCGTCCTGCAGCTAGGGCGGGGTTACCTTATTCGCATCATGGCGGGTCTATCATATGTACTTGAAACGGGCCGTGGAGAGGTTACACGATACCGCACTGGGCTTGTTCTTGTTTTCGGCTCTACGATCCACACGCTATACTAATACATGCATACGCCATTCCCTCCCTTCCAACTCCTGTGATCATCCGGGGCATCGGTACACAAGAATGTGTGATCCTGGATGAGAGATGTCAGCGTTTTTCCTGTGGGGAGAGTGGTAGAAGCCCACCGTCGTGTTCTAGTCCGCTTTCTTGTACCACTTGACGATCCAGCCCTGCCTTCTCAGCTCTGCCACTTGATCGTCGCTCGCTCGTGTCGACCCAATATCAGGGCTTTCGTCGAGGTCGCTCTCTGAGACGCCCCTCCTCGTGCGGAGGACGAACAAAAACACTTTGATCCCTGTCGATGGATCTAGGCCCCATCGATCTCCAAAAAGGCATATCTGGGGTTCACCCATCTTGATGACCCCCCCGATCACGTCAAGGGCCCAGTCGAAGACGAGAGAGGGGTAGGGATTTTTGTCTGCCTTCGCGCGTTCGCGGAAGTTGAGCTCATCCTGGTCCTTGGTGGGCTCGGCTTTCTCTAGCGCATCCCGCACATCCTGTTCTGTCCACGGAATGCCAAGGTACCACAGAGATGGTTTATCGTGGAAGGGTGGTTCGGGGGCGGACATTGTAGGGTAATAATGGTTGAGAGCTGATTGTGATGTGGAGTGCAGAGGGAATGTACGTTGTTGAGGTCATGAACCGTAGTACTACTATCGAACCTAAGCGTTGTCACGTGAAACTCGCCCCAGCGATGGTTCTCGCTCACCCGTCCGATGCGTATTCAGTAAAAACAGCATGGCTCGCGGGAGAGTAGATCAGCAGATTGCGGCAGGCCCGGTAACCTCGCCACAGTGCAGTCTCGAGAGTTACGATCAGCATCGGCCCCTAGGCCGAGCTGAGAGATGCCCGCAGTGTGGCGAGCGAGGCAACGTCTGACCTTTTCAGTTCAGCACCGGAGGCACGAAGTCTCGGAGAGGCGCTGCCAGAGGGGCACGGAGGCTACTATCGGCGAGTCCTGCGCTCGAGGCGAGTCAAATTTAGTAAGCTGGAGAGTATACAGATGCAGTCCAGAACACAGGGACA

>AT3114

ATCGTGATGATCGGCACAATGTGCCAGTCGATCTTTCGCCACAACCGCTTCTCCTCTTCGCGGGTGAGCACGGGCGGAGGTGGTAGCGCCTTGTCCCCGCTGTAGGATACGACATGCGATGGTTCCGCCGGTGGACTCACTTCTTCTACCCTCAACTCGATATCGAAGTCCTTTTCTAGCGTCGGGCGCGTCTTGCCGTCCCCTAGTGAGCGACTCATGGCAAAAATATGAGGGTGTAGGTAGCTGGCGAGGGTATCGACAGACTGGGGAGAACTGGACTGGCCGTCGGGGGAGGTTAGAGTTCATATACGCGGCAGCAGTGTGGAGGTAGACGTACTCAGCTGACTTGTTCATAGCCGTGCGGAGGTGTATAAAGTTGGTTCGAGGGACGCCATGGTTCTTCTCGCGGAAGGCTTCCCGTGCGAGTCTGGCGGACGAATCGGCGCGCGCGAGTGGCAATGGCGTGACGCGTGCAAGGATTTGAACGACCGTGCGATGGGGGCACGTCGGATGGCTGGCCAAATGCATCGATACAAATACCCGTTACGCCAGACTTGAGACGCGTTCCATGGCAGCGGTGTGACGTTCAGCGCGCATTTGGGACTTCCATGGTTGCCTCACACAGGGTTTGGCCAAGCCGGAGGGGGATAAGGAACGAACCGCTCGCGAATAGCGCAATACTCCCGCCAAGCCGTTGCTGCAATGTCCGCACTTTACGAAAGGGTGTGGGTGTGCAGCGGTAAAGACTTAAGTTACTCTGGAAGATGGCACAAACCTCCCATCGGCGTCGCAGGAGCACAACACGGGCGC

>AT3144

ACGATGTCGTATCCATCTCCTTTGAAGTCGACGCATGTGTATGCGTAGTACCGACCGGGGCCGAACGACCCAGCGAACTGGGCCCCGCCGATCACGCGGCCGGTCTTCTCGTCGAGTTCCATGTGTGAGTCGGTGCTGCTCTTCTGACCGTCGTTGGTGATATCGACGGCAATGCGAGGGGAGGTCGTCCCCGCCGGGAAGGTGTATC

>AT3155

CGGCCATCTATGCGCACACCGCCGTCACGACGTATTCCATCTGGCGCACAGCCATGATACTTGTGCGTGATATCTCACGCAAGTCTTATCCATGATTGAACGAGGTCTCGGAAACACATATTATACATCGATATGTACAATCATGACAGGCATTCAACACGATGGACAGGTTTCGCTCACCTGTCTCTTCCATTTCGATAGCGACCCCCTCCATCTCCCGGCCAGCTTCGCCCTCAACTTCCTCCGTTGTCCGGTCTGGAGCCGCATGCCGCCTCTTGTAGAGACGAGAGAAAGTCATCTTTATGTCGCCGGTGTAAGATGCTATGCGAGGCTGTAGGAAAAAGAGTTGAGATGTGCGCC

>AT3157

TCCGGGTCATATATGCAACTCAGGAGGAGGGCAAAATCTTCTGCGTTGTCTCCGTGCAGTTCCACGAGCGGCACCCCATCGTAAGAAGGCTCGTCGGACGCGGCAGCGTCAGCATCCGCAAAGAGGTGGGAAAATATCGTGGATCGGAGCCCGAGGAGGAACTTGTGCACGCGGAACAGTCGATATGTTGGTCGACTTTCGTCGGCTTGGGGTGACATCTTCGCGGCGAGTACTACGTCGCCGCTCGGGATGTAGAACTTGGGATGGCGTTTGAGGACGGCATTGGGA

>AT3176

ATTTGCTCCTGTCTCGAGCTAAGTAGAGATAAGGTGAGACCCAACGACAGTAGAAACGGGACGGATGCGTACAGCCAGTGCTCTGAAAGTTTGACATTTTTCAAGAAGTGCATAGATGCGAAGGTTGTCAGGACGTACGGAGGGCCTTTGACGACTGGCATCGACGCCTGCGCGCCGAAGTCTTGGACGAAAAAGATCCATGCGCCGATTGTTATAGTTACGGTGGCAGCTGTATCAAAAAAGCTACG

>AT3177

ATCGAGGACGCGACTACACCGATAATGATGCAGGCGAGGGATTCCCTGACCCTCGCCAGTTGATCGTCAGACATCGGCGTCGACATCGTCGGAATGAAGCATGGAGGGGAGGAGGGAGGTCGGGAGACGAGGGAGATCCGAGACCCGAGACCGAGCACGTAGACATAAATAATGCTAGGCTTGGATCCATGTCGACGGGTTGTCAGTGGGTCGGTTAGAAACAGGGGGCGTTAGACTGTCGAAATAGTATGTGCTTGCACAGACGTGGTATGACGGCGATTGTCGAGACAAGACCAACATGAGGCACGCCGCAGCCCAAGAATAAGAACCGGGGTCCTCGATCGTGCTCTCGGCCAATGAGCTTCCACTCCGACAAGCTCGGAGCCACTCCGTTGGGACCACGGAGAGTGAAAAAGCCGATCGGAGTTTCAACCTGGGATCAAGCTTCGATCAGTCATTGTCATTGACTCCACTGCCAGCGACGAGACGAGACGAGACTGGGTAGCACGCCCTCTCGACGCGTGCGAAAAGGTGCCTACTGACCTCCATTAATCTAGGCCATCGATAT

>AT3182

TCACCCTTATTCTGGAATGCCTTATCCTGCGAGAATACATAAACGACAAGCGGGTGGTCCCGCTCGCGGATGAATCGAAGAGCCTCATCGGTGTCCTTCACGGGAACAACCAGAAGCACGGGTCCAAAGATCTCCCTGGATATCAGTTAGAAATTGCATGCATCCAATAAAATCTGAAGGCTTACTCGCTCATCAGCGAGTCATCCCCGGAGACATCTCTGACAAGTGTCGGCGCGACGTATTTCTTGGCGACATCGACCTCGCCTCCGAACACAATCGTGCCCTTGGTGGCATCGAGAAGCTTCTTGATACGCTGCGCGTGTGATTCGCCGACGATGCGAGAGAAGGAGTCTGACTTCTCAGGGCCGTCCGGGTAAAACTCGCGGTATCTAGGCAGACATCAGTTGCACCGTGACAGTACTAGTACCCCAGAGGAAGAACAACACTGTGTGACTTACACATCTTTGACCGCGTCGACGAACGCGTCTTGGTGGTCCTCCGGCACCAAGACGTATTCCGGACATACACAGACCTGACCGGAGTTGAGGCAGCGACCCCATAAGATTCGCTTCGCGGCGAGCTTGAGGTCGCTCTTAGGGTCGAGAACGACCGGGTTCTTCCCTGC

>AT3187

AAGGAGGAGCGCGAAGTCTTCCGCTTTGTCTCCGTGCATCTCGGCAAGCGGCACTCCATCGTAGACTTCGGCAGAAGCAGCGGTGGAGTCGGCGAATAAATTGGTAAATGTGGCGGAATGGTGCTTCAGCAGAAACTTGTGGACGCGAAAGAGGGTATACTTGGGCGGGTCCTCGGGCGCAGCCGACTCGGGAGTCTGCTTCACAGCCAGGACGACGTCGCCATCGGCGAAGTACAGTTGGGC

>AT3191

TGTAGAGATAAACTCATCGACAGCTCCCCGCTCGCGTACTTCTTTAGCCCGTGATACGGCGGACTCGAACATATCGACTAGCGCTACGACCGGCACGACCGGCAGACGCATCTTGCGGTAACATTCGATAATGCTTGCCTCAGTCTCCGGTATATAACCTGGCGTTGCGACTCCGCCATCCCTTCTTGACCTCAGTAAGCCGTTGACGGACAGGCGAAGCCAAGGCCAACTCACTGTTTGAGATCCTCAGGCGCAGTTGACAGCCCCTGGCCCTTCGAATTCCGGCCGGAGAGAATGGCCTCTTCGAACGCATCACAGAGCTCTGTAAAGGTGGTAGAGGCTTGACCACCCTTAGGACGCATGAGAGGAACACGATTGAAGGTGACGTCATGAAGCGGGATTCCGGTCAGGCGACTGAGGACATCCATAGCGTGAAAGAGGAGTGAAGCTGGTGTGGAAGCTGGCACGGTGTACGACTCGAAGAGGACTATCGTCCTTGGCTCGGGGGTGCAGAAAGCAAAGACGAGGTGATCTGTCGCAAAACCAACGAGGCCTACGTATTTATCAAGACCATATCGATGGTAACGCGGGCCGAGCTAATCCGCTCGCCGCCCATTGTCAACAACTTGAGCACGCGGCGTGCTCGACAGC

>AT3195

GGTCCGGATCGTACCAGATTGACGTGACACCAGGCTCGGCGAGGGGTCGTTTAAGGCGGACGCGGGGGCCACCCATGAGCGGAAGTGATGTCGTTGAGGATCCGGGTGACTCAGCCGCGACGGTGGTCGTGAATCGCAGAAGGGGGAAGTAGCGAATCGTCCGATAGATACTCGCGAGCAGAGAGCGCAGTCGACCATTGCCGTCATACGACTCCTGAATCTTGAGAATTCCCTGAAGTGTCTGCTCTGTGGCGTCCCAAGAGTGGGATAGAATTGCGTAGGTGGTCGTACGAGGATCCTTCTCCACGAACCGGCCAGTCAGGGTGTCCAGCAACCGCATCGCTGCCT

>AT3201

GTTCGCGAGGACCCCATGGAGCCAAATGAATGGGTTCACGAGGGAGCGCGCGGCCTCCGATCGGTCGACGTTGCGGTCCGCGTCGGCCAAGGTCATGCGCACATAGATGAATACTTGTGCTATAGCCTTGACCAGGATTGAAGCGGTCGTTTTGTTCAGTGAGAACTTTGACGTCGCGATGCCGCTAGCGTGGTCCACAGTCACACCACATTTCTTGTAGACACTGGAACTGGTGATGGGCATGTCTATCACCTCCTCCGGCACACGATCGTAGTCGTCTGGCGACCAGGACGCCATGAGGACGAACAAGTCACCCATGAAATCGGTCTCGCTCCTCTTCATCCGGGCGTCAAGCATCAACCAACTGCGGCGGATTATCC

>AT3206

AACACCCCGGGCCACCGTTAAGCCTTCACCGGCCACCTCTTCCTCAGCACGCGCGACCACGGCGAACGCACGATAAGCGAGAAGAAAGAAGAGGCGTACCAAAGCGTGAACGGCGCAATCTCGGGCTCGTGCCGTGCCTCGAAGAACCAAAACCACTGTTCCCCCAGTTTCCCATTCCCATCAAGCCCCGCCGCTGCACACGCGCGCGTGAGACGGTGGGAGGAGGACGCACCATGGACATGTTCGTGCCGACATCGATGTAGCCCGAGAGCTGGTTCACGCCGGGGGTGGTCTCGCACACGCCCGAGTTCTTGACGAAGCGGAGCTTGGTGTCGTTGAGGTTCCGCGACGTGAACTCGACGTAGTTCGTCCCAAGGTCGGTAGAGGCGTCGACGTGGGACTGCCCCGTCCTGTCCGCAAACGTCGGCCACTGCGCGGCGAGCGCGGATGAGACCAGGGTGGCGAGGCCTGCGAGAATAAAGAGTCGCATAGCGAGAGCCCAGAGCGAAGTGAGTCTCAGATCGAGGACGGAAGAGGTCGATCGGAGGCTGGCGCGGGGGAGCGTATGTTCAAGGCGGGGCTGCCAGGGCCCTTATACTTGTGTGCGCGCGCGGTGATTGCATTGCTGAGTGTCATGTGTTCGCGGCTCGGTAAAGGTGAGCGGTTTGTCGTGTGAGGAAGAAGCGTGCGCGTTGCATGGTGCGACGGCTGAAATAGTTCCCGCTGGGGACGACGATAGGGCCGATGATAAGAGTGTCGGTCAAGGTCGCCTGACGGGGCGGGAGTGAAGGTATGCGTGAGTGTGGGCCCATATGGAAGAGGGATATATGTCGACTAGTTCAGTGATAGTCGAGTGAGTGAGAGATGGTGGACGGATGGTGACCGGTGAATAGCAGA

>AT3215

GGTCCGTATACCGTCGAATAAGTTACAACTGACGGCGGGTATCGAAGTGTACAACAAGGCCAGACGGACGCCCCGCATGTAAAGAGCGTTGTTCATCAGTTGGTGGTAGCGTTCGTGAAGGACGTGGTCGCGAATCCGACGCGGGAGTTGGCAGAGTCGTAGACGTAGTAGAACCGCTCGAGGAACGTGTACCCGTCGATGAAGTCGAGGCCTTCGCCCGAGTTCGAGCCGAGGTCGGCAGTGATGAGGTAGATGCTGC

>AT3237

GAAGTTCAAGCTCGACCCTGGACGCAGCAGCCAATATGACGCACACGTGCCAAGCCCTGTCTTTACGGGCCACGATGGAATGGGATGTGTTCAGATAGAGTCTGAAATAATCGATGGAGTAGAAGGTGTGCTCAGAATCGAAAGAAAAATCTACCCAGGATAAGTAGTCTAGCCTTCCGATGGACATGGAAGACGTACGTTGAATCGCTCGGCTGTCTAAGACAACCATTGCTTATCATTTTTATGATGATGCGGAAGCTTCAGATCGCATGAGCTCCGCCTATAGAACTTGTGGGTCTCTGGAAGATGGATTAGAGCTGCACGGACGAACGAGGGTGCCTACCTGAGCGAGGTGTCCGGGTCATGCACTCAGAACCGCCGTCTACACTTTGACTGTTCTCCGGGTCGGATGTGGTGGGAGGACGAGAAATTGGTTGCGTAAAGTTCGAGGACTGCCGTCTGAAGTGGTACCTGTACATGAGGTCAGAAGGCGGAAGCC

>AT3241

TACCTGACGAGAACAGCAGCGTCCAGGACGCAAGCCTCTTGTTACCCTCCGGCCGACAGTGCTCCATTCTGCCAAAGTCCGTGCTGGGTACCTAGTGTAGGCAGCGTGTTGATGTGTTGGTGGCCATCAGACTGCGCCGAACGGACTCTGGAATAATCGATAGAGTAGGTGTGCTCCGAATCGAAAGAAAAATCTACCCAAGATAAGTATAATGATGCAGAGGCTTCTGATCGCATGACTTCCACCTACAGAACATGTGGGTCTCTGGAAGATGAATAAGAGTAAGCGCTGCACAGACGGGCGAGGGTGCATACCTGAGCGAGGGTTTCCGGGTCATGCGCCGTGACTCAGCCTGAGAACCGCCGTCTACACTTCGACTAGCCTATTCTCCCGATCGGACGTGCTGGGAGGAAGAGGGATTGCTTGCGTAGAGGGTGAGTACTGCCGCCTGAAGTGGTATCTGTACACGAGGTCAGAAGGCGTGGAGTCGGTGCAGTCAGAGAGTGAAGCAACAGGCCAAGGCCGCGTGAGGCCTGAGCAAATTAAAGTTCG

>AT3243

GCCTCCTCGCGCCTACCATCAGCGTCCATCCGCATTGGTGTCCTGGCATCGGACCGGCGATGATACCACTGGCCGCAATAGCATCTTTACCGACGGTCTCTACCGCCCGGCAGTGCGAAGGGGGTATATTAGGACTCTCCGGGTGACTGTCCGCCGTGACGTCCTGAATCTCGACGACCGGAGCGTGCGCTAGGGTCGGCGGCCGTCTTCAGCTCGATGCGGACAAGGTAATAGTTCGTCCTTGCGAAGGCCAGGCCAAACCTTATCGTCAACGTCGTGGGTCCGCCGGGGATGTCGAGCTTAACATCGCTGACATACAGCATATCCTTCCATGGAGTCAAATTCTGCCATAATATCGATATCCTAGAGCCTAGACCGTCTGCCTGAAGGACTGGGAGAGGAGAGCCCTGAAGAACCGGTCCCGACGTCCTCACATGGGCCTCGATGTCCAACTCCTGATGATTGCTGGCATGTACTCTGAGAGCATGCTGGTAGTCTACGGTGATGGTGTGAGTGTCATGGGAAAGTGTAACCAAATGAGTGCGCGGGTGGGCGTCGTCTGGGCCACGTAGAGTGGCCGTGTACCCCTGGGCGGATAGAGCTTCACGAGTCTTCTCTGGCAGCACCAACTTGATCGTCTGATGCCATTTCTGCTGAGCAAGGCTGCGCGTTATTTCATCTCTTTCAGGCTGGGGTATGTAGAGTGTTTTAATGGATATTTGGCGGCCAGAGGAACGGAAATGGGTGATAGCAGCTGGAGACAAAGGAAAGAGATCAACGAGCCAGTGGTCGTAGATATGTCCAGAGCTGTGCAAGAACTCGATATCGATATGTGACCCCGGGGACCGTATATAGCAGAGGCGCGCCAGGAGATGTCCGGGGAAGGCCTTGTGTCCACATCCAAGAATGATGAGGTACCACTGGGAGAGTGGAATGTCAGTGACGTTGTGAATATTTTCTGGAGGGAAGTAATCGGATAATGATATCACGGGGAACTGCGTGCGGATACCATGCGGGGTGAAGGTGTAATCTGTGATGCCAGAAAGTTCATTTGGGCGGAACTGAAGTCGACAAGCAACTTCGTCATGAGAGAGTGTTCTGATTTCTGTGGCACGGAAACGATTGAGAGATGAGGTGAGAAGTGGGAGTTGGTCGTCAAACAAAACAATTGATTTTATTCGTCCGCCCGTGTCAGTATGGTCGGCCTCGTGGAGTCGAGATGAAAGACCTCGTGGCACTGGCATCCCGAATTGAGTCCATGCGAAGAGGCTCCGATCCGGGGTCCGTCGCATGATCTCCTCCTGGAGCCGTCGGAAAGCGCCCTCGCGCTCGCCATAAAGCGTGGGCATGTTGATATTGAAGATTCCGAGGAGCGAATATGCCTGATCCTCCACCCTCGTCGTCTCGCGTTGTGCGGCCCACGAGAAGCGCTCGGCGACACCGAAATCGTCGAGTGATTCAATGTTCAAGAGGGCATTGCGTTGGATATTTGTGATCTCCACGACCACGTC

>AT3261

GTGTGCCATCCGGCCGGGTGAAGGTGTACTCGACCTTGCCCTGGAATTCGTCGTCGCCGCCCGCCTCTACCACCCGGCGAAACCAGCCGTTAGCGAAGAATATTGGGAGGGGTTGCGCCGGTTCGCGCCAAGAGCTCTCACTGCGGGCCGAGTGATAGGTACGCACTGCAGAGGTTCGTCGTGTACGTGTTCATGCCGTAGAACTCGGACGAGCCGGTCACGACCGCAAGTTCTTCGGGTGTGAAGCGCGGCAGGCGGTCGCCCAGCATCTCCTTCATATATTCCGGATAATCGCCGAGGTAGATCGGGTCCTGCGAGCGCGATTGGTGAGCACATCGCTATGTCAATGGGGAAATCACAGGTAAAGGGAAGGCGCCATAGGGTTGAACATAAGATTGCATTTGTCGGGAGGG

>AT3271

TCGATATCCCTTCGCTCAGAATCAGTGCGATCGTCCACACAACCGGATGAAAGAGGCGACGATCCGAACGCACTTGTGCGTCTTGTCCACACCTTTCGGGGCGACCCTAAGCTCGTCCTCCGCACCGAAGATGTTCTGCCTTCCGCCCTTCGGGTGTGGTTGCGCGGGCCCGAACAGTGCCTCAAAGTCCGGACTTGAGATAATTTCGCGGAACCGCCGCGACGAGCGTTGGAGGTTACTTC

>AT3272

CCGCTCCGCGAGAAACTTGCAGAAAACCCTGTCTTGTACGGTGTTTTGTCATTACTGAAGCGCATCTGCAGCGCAGTCAGCATCCGTCGTTGCTGAAACAAGGGAAACGTCACTCACATCGCGATATATCCTGTGGATGACGTCTTTCGGAGGCAAATGCGGGATCTGCGGATCTACTTCCACGAGTATGTCCGTGAAGTTCTCCACGAACGCCTTCCACTCAGTTTCCGCTACCCGGTATACGGGTTCTTTCGTAGCAAAATGGTGAGCCTGTGTCACGATGAATGCTCGGAAAGCACATACCGTGGAGCTTGAACCTGCGCATCAAGCCAGTCGC

>AT3280

GCGTCGCCCTTCTTCGCGTAGTCGTCCATCTCCAGCTTTACAGTCTCCCACTCTTCCCATATACGCTCGTGTTCATCACCAGCAGCCGCGCTCGCAGGCCTCTCCGCGGCGGCCTCTTGTAGGAGCATGATGGCCTCCTTGTGTTTCGTGAGGGTGTCCTGGGTAATCGTTCCGGCTCGGACTCCGTGGCAGAGCGACTTGGCGAGACGCGTAGACAGTCTCATAATCAATTCCGGCTTCGCATCCGCCTTGTCCTTCAGCAAGTTCCAAGAGCGGAGCACGGTGTCGACACATTTTGCGTAGTCGCCGATCTCGTATAAGGCAGCACTGAGGTTTGAAGGGTACACCGGGTCGTTAGGATCAGCATTCTCTGCC

>AT3302

ATACATATCGTCCTGGTCAGCAAGGCGGGGTACTGTAGTGGGCAAAGGAACATCAACGTCCATTCCCTCACGAGGAATGAATGAATGAGGACACATACACAGGCTGATCGGAGCCTTCAGCCGGAATATCTCGGCAAAAACTGCCTCCACGTATGGAAGACTGGTTCTGTCGGAGACGGATGGCAGCCGCTCGGTTCCAACTACGCGATCGACCTCCTCCTGAGCCTTCTCGAGCACATCCGGATGCTTCGTCATGAAGGTCAGGAATGCGGCAAGGATGATCGTTGTCTGCATCGCCGTGTCCTTCGTCAGCCACGGACATTAGCCAATACCAGTGAGTCGGGCTACTTGCCGTATCACCGC

>AT3303

CATTCTCGACCCACCGGTGGCCTCTATCTGCCAGGGCGTAGTATTGCTTCTTATATCCTTCCGCCTTCCGCTTGAAGCCCATACCGGGCAGAAACGAAGGAAGGGCTGCAACTGCGACGATGCTTGAGTGACGCCACCACCACCACCACAGTAGGGGGCGATGTTTACTCACGGAATGGAAGCATATCAACGAGGTAGTTGTTCGGCATCGCCGCGTCCGAGAAGTTCTCGCCGAAGACATCTGCCTCCTTCACGAACTCGTCGTCACGGCTTCGGACACGGTACCCATGGGACACCTCGAT

>AT3304

CCTGCGCATCCGCGTACTGATGGACCTGTCCTTGGCTCATGGCAGCGTGCAGAAGTTTACGTTGGAAGCGAAGTCGAGCCGCGTCAGGCTCGAGGACGGGGCCGTTGTCGTAACCTGACCTGAGAAGCGTCTCGATTCCGTCTCCGATATACACAACCGGATCCCAAGTACTCACAATTCGACCATCTTGGTCAGTGGTCTGGTCACGTAGTTCGCCGACTTGGCTTCCATGAGCTCGCGAGCGACCTCGACGGAGTTGATGACGATGACCGGCGTGCGGAAGATTTGCAGGGAGTATATCGGGCCTAAC

>AT3319

ATGCCAGCGGTGTTGTGGAAGACCGAGCTCGTACTGGTGGTACTGCGAATACAGCGGTGGAAGACGCATGCCGGGCCATCTTGATGACTGACGTACTTGGCCTTGGCCGGGAATGAGGGTTTCGACGATGACCTCGCGGGGGTAGAACACAGTGCGAAGAAGAAGTGTAAAGAGGACGAAACTGGTAGTCAATATACATGACACGAGGAAGCGTCGCGTGACGAAGCGTAGATAGAGCGCAGCTCTGTCAGTCTTGGCCGGATACCAGTTGGCATGCTGTAGGTTGCTCGGGAGGATACTAGTCATGCCAGGGGGGGGAGCCCGAGCGGAGGGTGTACTGGCACCAGTACTTCTCCGGGGGATATCGATAAATCCCAACCGGCTAGCAGGCTGATGCAACAGTGTAAGTTCGGGTGCCGGCACCCGCACTTAGATTGCATGACAAAGGGTTATAACCGAGACACGGGCAGGGCGCATGGGACGATAATGCGCAGGAAGACGGCTGCGGAGACTACCAGACTACCACATAGGATGTGTGGTTTCGTGCGTGCCTCGGCGATATCTCGATTGGGC

>AT3320

GAGGGGAATAAATGAGAGTTTTGCACGGACACCACGGGGGGTGACGGTGTATTCTTCCACCTGGAGCCCGAGGGAATCGAAATACCCCTGAGAAAGGCTATCACCTTTGCCTCGAAACCGATGGAAGGACTTAGGAGATGATGCAAAGGGTGATTGGCTACATCCAAAGCTATGATGGAGCCGGGGCGGCTGGGAAAGGGCGCGCATTCGCCCCAGGCAAAGAGGGTACGGTCCGGGATTCGCTGTAGAATCTCCTCCTGGAGCCGCTGGAATGCCTGTCTACCCTCTCCATAAAGCGTAGGCATAGTGATCGCGAAGATCCCGGGCAACGAGTAGGCCTCATCCTCGACCCGAGTCGCTTCGCGTTCAGCCGCCCACCGCATCCGCTCCGCAACGCTCT

>AT3336

ACAACGTCGTTGTTCCATACATACCACCACTGGATGTCATTCTCCTGACCGCCTTCGTGCATCGCCTCCTTTAGCTTCGCCACGATGTTCTGGAACCGCTCGCGAGATCGAACTGACTTTGAACAACGTTCGTTCATGTCGTAATTGGACCACAACGAGACCATCCCATCCCATCCCAACGAAAACGGTCGGGCGACATGCAGCGTGATGCCGCACCTCGTCTCGATGTGGCTCACCGCCGCAGAGCGCCCTGCACTCAGAAGCGAGTTCATGAACTCCTCGTCCTCCTCGTCCTCGCGTGAGATGTCCGCAAAGTCGAACTCCGACTTCCCGTCGAAGAACACCCTCGTTTCCGGCCGCAGTTCCATAGTGAGGTGGTGGCGCTTCGCGTATTCGAGCATGTAGCGGTGGGTGAACGGAATGCCGTAGTGGTAGAGAGGAGGCTTCGAACCGTCCTCGGGGATCATCTCCAGGGGGAGCGTCTTGAAGGGACCGCGATTCTCCATGAGTTGTAGCCATTTCTG

>AT3337

TGGGCTTCCTGGGCCGCCTTGGAATAATCGGCAGGGTTCTCAGGATCGTCTTCCAGGAGATACTGCCCGTTCCAGAACAGTCTGGGGTCAGCATCGGCAGGGGTAGGTTGGGACATGAGACATAGAACGTCAACAACAGGTCGGTGGTAACGTTGGTGGTTGATTCAGAAGCCTCGATTGGCCCCACCCTGCGTGAAAAACTAAGACCTTGTATTAATGGAGAGACCTTTCTTCGCCGATCTGTAGATTCAGCGACTTGAAAGCGAGAGCTAGAGAAGCGTGTCCTTGAGGTCGAGCG

>AT3341

GTAGGGTACTATGTATGCGGCAAAGTCAAGGAAACGAGTAGACGCCTTCAGCGCCTTACGCACGCAAAGCGACGAGGCTTCGGCCTGAGCACGTTAGTTAACCTCGCTCTTGGTGAACTTGGTGTTCGCGAAGCCGACGCTGTTGCGCTGGGGCACGTAGACGTGGTAGAACCGCTCGAGCCACGTGTAGCCGTTGATGAAGTCGAGGCCCGAGCCGCTGTTCGAGAGGAGGTCCTGGATGACGAGGTAGATGCTGTCGTTGTCGCCGCCGATCGCG

>AT3399

TGCAGCAGCGATTGCTAGCGCATATAGCCTCGCATTGACAGCTTCGATCTCTCTCCTCTTCTCCGCCACCTCCCGAATGATCCACAGCTCAGGAAGCCAAGACGGCGGCCGGCTTAAGAACGGGAACACTTGCAGCACTTGCGCACTTGCATAGAGCAAGGGTTCAGCGATCGTCGCGACCCTCTGGGTCATATCGAAGTACTCGCGATCGCCTTCGCCCCCGGAGAGCTCAATACCGTACGTGACGCGCATTCCTGCCTCC

>AT3400

TCCTTGGGAGATTGCAGCAGATGGCGAAGTAGCCTTCGTGCGCTTTTAAGCTGAGTGGGCTGCCATTGTGCGACGGCCTCCGGGCCAAATGTCTGGTGGAATGCGCGGCGGTGCTCGCACCATTTGTTCCCGTACGGCGTGAAGACGAAGTACTGGTCGAGACCGAGGCTGCAGAGCGTCACGGTGCACGCATTTAGCAAGGGACAAAAACGGATCGTGCAATGTGAACCGCATACAGCTTGACCATAACTAGGTCCGGACGGTTAGAGAAGTTGGCCGACTTCTTGTCGAGCAAGTCGACAGAGTGGTCAAGAGTGTTGAGGGTGATGACGGGCTGCCCTAGGACGTTGAAGTAAATGATGTCACCTGTT

>AT3429

GTTGTCATTGTACAGTGGAAGATATGTTACAAACGTTCATGGGGAAGCTCGAAGAACTTCACAGTCTGCGGATAGGCGTAGCAAATGAGAATTATTCTCAACATTGTGATAGCGATGTATTCTGAACAGGAGACCCTCATAAAAGCGGCTAACGGTCAACCATTGTCGACAGAATGCTCCGCAACACTGCCGGAATGTCCGCCGTGTTGGACACTCGGTAGCCTCGTCCAGGCAGGTTCTTTGGT

>AT3432

GTACTTGAGCTGCGGGCGGCCCATCTCCGGTTTCGCCATGCCGCGCCGCTTGTAGACCGTCGCCTCGCCCGTCAGTCCCTCCGTCAGACGGCTGTCGTCAAGCTCGCCGTCGGTTTGCCGCTTAACCCACACCCGTTCCTCTTCACGAGCCGCCAAATCTAATCGAACGCCGAGTTGAACATCAGTAAGACATTACGCCCCCGCTACACAGGACTCGGACTTACTCTCGAGCAGGTCATGCAGCTGTGCAATGTGGCCCTCGACGGCAGCCAGTAGGGATCCATAGAGCTTCGCCTCGCCCGCACTCATGTTGAGCTCCTCGAGGCGCCTTGCCAGCTCCTGGCGTGCCATCTCGCGCGCCCTCTCCTTTACATGGTCAGGGACGTCGTTCTTGAGAGCGTCTGAGA

>AT3434

CCAGGGCGGTTCGCAAGGAGTATGAGGCGGAAATTGGGGTGAACAACGATGTCGCTGTCGCGTTCCTTCATCTGGCGCACACGGCGGCCGTCGGCGAGCGTGAGCTGGCCGTGACCCGCAAGGCTGCGGAAGATGGCGACGACGTGCTCCGCAGCCTTGTCGGCTTCGTCTATAACGATGACCCGTCCCATTTGTATGGCTCTAAGAAGCGGCGAGTCGGTGTACTTGATGACGCCGTTCTCCAGGATGGTCTGGAACATTAGCTGGTTCACAGTGGTATCTCTATGCAGTTGAATGTACTCTCTAGGGCGTCCCAAGAGCTGT

>AT3465

CATTGCAGGCCCGGGTGGCAGCTCCGCTAGCTTTTGAATCCGACGAGCGTGACCGCACTGACATCCGAGACTCGAACGACTCGCGCTCAAGCGATTCGGCCAAACACTGTCGCTTCGTCGACGGCTGCCCCTGTGCTCCTTGGGTATGACGTCACTTCCGACATTACATTCACAACCTCTGCCATCACGCTGGGCCGATAAAAGCCGCTCGTGGTTGTGCAAAACATGGGCATCAGCGATGGCGCTATAGCCAGCAAGTCTTCAAAAGGTTCAAGATTATCAGCTCAAGTATGTCTCAGGCTAGGCCACAACGCTAATACATTGCATAAGCAGGACCGTAACCTCCTGCCGGCCGTCAAGGTAAGTTGCGCAGGGCCTTCGAGTTGATGCCGCGGTAGATATAGTCGGAGAGCCTCTTGCCGCCGACTGAAGCCACGAGGGTCTTCGCGTCCGACGAGGACGGGAAGGGGCTCTCGTCAACCTGGCAGATGGTGCCTGCGGCGGGGAGCGTCCCATTCGCGAAGTATGTGCGCGAGTGCTGGATGGTGCACTTCGAGGTGAGTGCCGTCGAGGTGTGCTATCGATGA

>AT3469

TGCGGGGTGAAAGCAATGGTATAACTACAGGGTATAAAGGGTGCGATGCGCGACGAACACGGGGACCGGATACAAAGGTCAACTAAGAACCGAGCCGTTGCTGCGACCCCACCACACTGCACCCCCAATCACCGACCGCGTCACCCTTCCAGAGGGCGCGCGGTGACAGCAAAGCGAGGGCGCGGCGGAGGACGCGGCGGGTAGTACGCGACATCGCGGGTATCTGCAAGCCTCAGCTTGTGCCTCACGGGCAGAAGGTGATGGTGTAATCGGCCTTCTTGCCTGAAGGGCAC

>AT3479

GGATATCTTTTGGCTTGGTTGAGTCCGAAGCTCAGAGTCCGGTAAAGGCGGGGAATGACTGCTGTAAGAAAAGACCTGCTAACAAGCGAGACGGAGACGAGGTCGTTCCCAGACAAGTACGCGAAGATGAGTTGGAGACAGTCCGAGTTGATGTTGTCTGCAGATACCATGGCTAGCGGGATAAAGAGGTGGTTATATGCGCGTCGCACGATGGTGGATGCCACTGGAGATGAAGGTCGACGTCCTGGATGAAGGCTGCG

>AT3906

ACATTCTGCGTCCCGGATATCTGTGAATTCGATCTCGGAGAGGTTGGGGAGCATGCGAAGCAGGGGGTGTGGGGAGAATTCGTGCGCGTTCATCGAGACCGACTGGACACACGGTTGAAGGTGAGGCTTGGTTTGGAGGACGGCACAAAGGTCGAAAAGCTGGTTCCGGTTCTTGAACTCGACACGGCCGAGGAGGAGAATGGTGCTGCGGGGGCGAAGATCGCGGCAAGTGAGGGTGAAGCTGTATAGCGTTCGGGTGTCCTTGGAGTGGTCGATGACTCTCTCGATGACC

>AT3925

CCCCCAATACCCAAACAGATGCACAGCCGCCGGTATCGCATGAACATCGCCCGCGACGGTGGCCGCCGCTGTGCATGGGTTCTCACCATTGCGCTAGGTCCCGAACCTCGCGCTCCTTGAGCGAGGAGCTCGTGTGCTCCTCGATTGTTCCCAAGGGTAATAGTAACCTTTCTGACTCGATACCATGGCACCGCGGGTGGTGGCCGACTCACGGTGGGTTGGTATTCTGCTTCGGGGTCTAGGCGCCCACGGTGTCTGACGTTCAGAGGTTCAGACGCCGGAGGAATTACTGATGGACCGCGTCCGCAAGCGTCCCATCCCGCATACGGATCGGCGCGGTGAGCGGCATGATGACATCCTGC

>AT3926

CATGATGTCGCGGCCTTCGCCGATCTGGTGTTTGAGCGCGTCGTCCCCCTGCTCGAGCTGAGCCTTCTTCTCGTGGAAGATCTGCACGGAGCGGTCGTGCATGATGTCCGTGATCCGCTTCAGCGACCGGATGTACTCCACCGGGGTCCGCTCGGCGACCCATCGCCTGAAGGACGCGGGCCCGCACTTGCTCGCGTACGGAAGCAGTATGCGGAAAACGACAGACCGGGCTATTTGTGGGC

>AT3939

CTTTTCCTGCAATGACTACAAGCTTGTTTTGCGTATGTGCCGCGGCCTGATGGAATGAGGGACTCTGGCTTCGCACGTTGCTTGCCATCCTTCATGTTGCATACAGACGGTGTGGAGGACGGAACAGAGGAAATGAAATGCGGGGTAAACGGCAAAAAGACGGAAAGAAAGACGAGAGGTCCGAATCCAAGTCCGAGTCCACTTTAGATCCTTGCTTACATCGCATAAGCACAGCTCGTACCTG

>AT3957

CATATGCGATCTCGTTTGATTGAGTAAGATAGAGTCGCCGTTGTGCTACTTGACAACGCATCGGGCCCTTACGACACCTCTGTACATGTTTTCAGTTTTCGGACAGTTCATACGTATGTCTCTTTGGTACGGAATATGTGCGAGGGAAGGAACATACGATACGATGTATGGAAGTGGCATACGGTAGCAACGTTCACGTGCTAGATCCATCCTTTTGACTCTACCAAGCATCGTGGTGGGATAATGTCAGATGGCCGATGAGCCCTTCATGGGGTGAGGAACTCGTCCGAGTCGTCAAAGCACTGGAAAATCCGGCCCTCATTTGAAATTTTCACGTGTGTCCAATGGCGATAGGTGATCGTTGGCCGCGAACGGGAGGTCGATGAAGAGGCGGCTGCGGCACGACCCCAGACGTCGAAGACCCGCGACTCGGCAGACAGCACCGCGGCAAGCCCCACTGCTTCGAACGCGGAGCCGCTCGCGGACCTGTGAACTCGGACGAGGACAAACAGACGGTCCGCTGAATGGGGACTCGCCAGCGAGGGCTGGAGACGTGTCGCCGAGCTTGAACCAGGACGGCCTGAGCGGGGTCGCACCGCGGGCATTCGGATCTGCGGTGGTCGTCGAAAAGATCTAGATGGCAGAAGCGAAAAAAGCTCACCGCAGAGCGGCGAATGCTGAGCAACGGTCCGGCGCGGTGCCCTTGAACCCTTTCCCCATTGTTACTTGCGGCGTGGCAACCGGTCGCCGAGCAGGACCCGATCCAGCGCTCCTAAGGCCTGGGGCGAATGTCGCACCGGGGACGAAGGCGGCACGCATGTGGCCGCGACGGTCGACACTGACCTAGTACACCATAGATGCAGCGTTTTCGGCCCCATGTCGATGGAACGCTGGAGGAACGCCTTCGAAAGGTGCTGAGGGGATCGGAGAAGGAGTTGGGGACGCTAGTGGTGGATAGAAGAGTTTAAGGTCGCACAAGGTGAAAGGCACGGATCTCGACGTCGTTGAGGGTCCTCGAACGTCGAGATCATGATG

>AT3966

TCCCCCGAAGCTCCACAGTCACCTCGTCGATCTGCTCGTCCTGTACCTGTGGGAATTTCAACAGCACCTCGCCTTCGACGTCGCTGCCGGCGCAGTAGGTAGTCGGCGGGATTACCAGCGAAAATGGGCCGGTTTGGGGAGGAGCGAAGGTGTAACAAGCCAGATCAGACGACATGTTGCGTATGCCCTAGCCATGACAAGTCCGCACATACTCTTATATGTACACGCTCGAGGCAGGAGGCGGTGCAATGGAGGCACATCAAAAGGACTGTCGTAACTACTAACTGGAGGTGGGAGCGTTACGGCGTGGCGGTGCAAGTGGATGGCAAGTGAATTGAGCCCAGAAGTCCATGCACATGTTCGAGATGGCGCGCCCGCATAACAGCACGTTCGGGTGCAATTAATATGCAGCGGCTCGCCGTATCTCCCACAGACATAGGAAACGGTATGACCACGGATGGGGTGAAGATGGGACGCACTCAGCCCCACGCAGCTGCTGGTATCACGGCATTCAACTGATCGCGCT

>AT3968

CGTACGTATGGACGTTTTCAGTGAGGTTGACGCAGACCTCGTCGAATTTCTCCTCTTGCAGCTGGCGGAAATTGAGTAGAACCTCGCCCTCGACGTAGTTGTTGCCACAACGTATGGTGGACGGGACGACGACAGTCAAGGCAGACGACATAGAGGCGGTAGCATGCTGTGAATAAGCGTCATTTACGATCCGAGTCGGGGCTGCAGGCTGTGATCAGGAAATACATTGTCTCATCCCTAGAACCGCCATCTCCTGGCCAACTCTGCCCAGGACCTTCCGCGAATTCAGCCTATCGAGCTCACGAGTCCCCAGCAGGACGTTATTGAGTGGCCGTAGCTAGTAAACAAACGAATGTGTTCGTTCCTCTCACTGTTGCGTGCGTGTTGTGGACTTGCACTGTTGTTCGCACGCGTTCCTCATTCTCAGCGGAACGCAGAAGGCGAAGTCAGCATCGCCAGAAAAACTTGCCTGTATCGTGGTGCCGGGGGTCCCGGTTTGGTAGGGAAGTAGCGGAGGCATCGAGGCTGAGCTTCACTCCCAGTGAGAACCCG

>AT3974

CGAACATCTTCTCCCCAGATTTTCGGGTCGCACTGCGCCTTCCTGGTGGAAATAGCGATCCGAGAGCCCTTAGGGATAAAGTACTTGCCACCACCGATGGTAGCATCTTCGAGTGCCTCGACGGCACGCATCGCTGCCGGAGGGCTCAGGCGAAGCGTCTCCCGAAAGCATGCTGGCCAGAAGGCGTCAGCGTCGCGCGTAAAGTTCAACGGCTCAGGACCGTAGTAGCGTACCGGAGATGTACTTGAGCTTGCTGACGTCCTCGACACGAATGGGCTGATCACCGAGGATCTCGTCGACTTCCTCGCGGACTTTCCGCATTGCTTCTGGGTTCTTGCAGAGGTAATAGAGGGTAAAGGTGAGAAGCCCTGAT

>AT3976

TCGCCATACTGGTCCGCGAGCAGCTCGAAGGTGCGCAGCGGGACGTCCTTGTCTATCTGGGTGACGTGGCCAAGGAGCGGGAGCGCGGGCGGCGACGGGATTGGAGTCGTCATTATGGCAGCGAAAACGGCGAGGAGGGAGGGTAGTGCGGGCGAGAAACAGTTATCGATTGATCCTAGGCGTTAGATGCGCACACAGCTTCCGATGCTAAGTTCTATCAATGTCGCTGGCGTGGCGCACTGGCACCGTTAGCAAAGAAGTCGGTGGAAGCAAACAACTGGTGGCGGGGATCGTCGGTGCCCTTTCTTAGGGCCCGGCACAGGTTTATGTATGCTGCGATCTCGCTGGACGCTGGGGTGGCCCGTTGTCTTGATTTGGTGCCTAGACGCGACCATCGTCCGTCTGGATGTAGATGACACAGCAGCTTCAGGCCACATTCTGTGTCATGTCCGGGATGAATGCACGCCGAACCGGAGTCAATGAGTTTGCCATTTCGCCTCCGCACCATTCCACCGATCCGGTAGTGATGCCACCGGTCGCAACAAAGCGCGTTACCAGCTTGCGCCAGTTTACGCTTTACATACATACATAGCCGCTGCGTAAATTCTGTGCGCA

>AT3982

AATTATCCCTCCCCAAATGCGTCACGACGTAATGGGCTGAAAACGTGTCACGAAGTATGTACACCCATTGCACGCACCGATCGTTCTACTTCAAGTTGGCGCTTTCAGGGCGTATACCGGAAGTCAGGAGCCTATGGGTCCGTGATAATAGTATGGCAATGTCAGCATCGGTGCTGTATGACCCTCCCGCACGGAATTTAAAAGAAGCAAGTACCTTATCAGCGAGTTCCGTGGTGTCTACGGGGGCGTCGGGAGGCGGTTTGCCATATTGCATATCTCTACGGCTATTTTCCCGCCGGTAAGATACCGTGAGGGTCACGGCCACGAGTGCCGCTAGAAGGTCCAGAGCACAGTTGACGGTAAAGCCTTTCCTGCCCGTGCAACAAAACGGAGTCTGGTCAGAACATCACCGGTACATGCTCATCGAAGAGCCACCCACATATACCGTGGACTCTCTGTCAGTGGGTACATATGCGAACCCAACACGGCGCCACTCTGACCGATAGCCTGGAAAAGGGGGAGTCCCGTCGCTTTCTTCGTTTCAGACCC

>AT3989

TGTATTCATGCTACGCGTGTACAAGGGAGCCTGGAGCCCCATTTAACTCTATACTCAAGTTCCTCTGCTGTACGGCGATATGGAGACTGGAGTGCATGTATATGCGCACGACGAGTTTCTTGATTTTCTTCCTCATAATATAGTATGTCGAGATCTTTCTATGTATGTCCATCTACCATAGCTGTTACGGAGTGTACCGGAAGTCAGGAGCCTGCCCAAAGAATACGGCGTGTAAGTCTCGACAGAAGCACGAAATGAGACTGTCAACACCTTGTCAGCAAGCTCTGACACGTCTACTGGCGCTCCGGGAGTCGGTCTCCCGTACCGTTCATCCCGACGTCGGTTTTCCCGTGTAAAATACACCTGCAATGTACCGTTCCTCAGCTGCGACTTCCACAGATGTAAGGATAACTGGACTCACAACAAGGGCGCCCGCGATCAAGACCGACAGAAACTGCAAGCCGCAAGTGATGGCAAAGCCTTTCCTACGGCAGACAGTCTCTGATTGAGCTTCACTTTGATACAAGATTTCGCGAAACAAACGCCTATACTCACGCATAACGCGGCGCATCGGTCGTCGGGAACAGATGCGACCCCAGTATGCTCCCGCAGTGCCCGATGGACATATACAGTGGCATACCCGCCGCCTTCTTCGTCTCTGAGCCGAGGTTATGGGTAACTGCATGAGGGTAGGTTCACACCGGAGCATCGCGGAGGGAGGGAAGCGTCCACGCACACCACGTCAAAACCAACGCAACGTGCGCGGCCGTCCCCGCGCATGTGCAAAACGTAGCGAAGTAGCGGACATGTTCGTTCGACGGGAAAGCGACGAGCAATCTGCGCAGCATAAGGTCCAAGTCGCCCTCAGTTCATGCATGTGGAAGCGAGGACGAGGAATACGAACATGTATGCTATGCCCGCCAGGATGCCCGCACCCACAATGAATACCCCGCGGCGCTGCAGCCGATCCGAAGTGTACATCGTCAAGCAGAGAAGCATGACGGTGACCGTGTATGGCGGGACTGTGAGGATTTGAGCCACCGCATCGGCTACAAAAGCGCATGAGTAAACACAAATAACCGTCGACGGGCAGAAAATTCATACTGACTGAAGCCAAAACTTGCAATGATGGTGGGTAAGAATGCTTGGAGGCCGGACAATGCACAATTGGCCCCAAAAAACATCGCACTAGCGGCATACACCTGGAAGAGAATGCACGATAGTGTGCCGCGT

>AT3993

GAGGATGTACATGGCGGTGAGAATGCTAGCGTTGGGTTGAACAGTCTCGCTAGGCAGACACAATTGACGGAGTCACTCACATGAAGTACAGGCTCCCTATACTCGAATGATCGTGAGCCGAGGATAGATCAACGATATTCACGCATGAATACGCACCGTTATACAACATAACACGTGCTAGTGATGGTCCCTCCAGTATTCCCCTTTGCGCTTTGTAAGATGTATACGTCTTGCGCCACGTTACGAACAGAACAATGAGTTCTCCCGCAATGTTGGAAACCCTAGTAGCGATGTTTACTGGG

>AT4003

AGCGGCGCTTCCGAACCTCCCAGCCCTTCCACTTCTTGAAGTTTTCCCCTCACTGGGTGTGTAGACACGAGCGAAAGTCGGTATTGATCATTCCCGTATTCGATCTTTCGAACTGCTGATCCTTCAGGACTGCGCTCACGCGCAGCCATGTATACCTTCTCTCAGGGCCCCCACTTTGGCCAATAGCAACGTCCCTGGCAGCTGTGCCGGCGTCTTTCACGTCCC

>AT4004

TCCCCATAGTCCATGTCCTCGAGCAAATCCAGGATCAGCTCCAGCTGGTCCTTCGTGACACGCGAAAGCTCGTGGTTGGAGAAGAACGTCAGGATCGGAGTCGGTTGTCCCCATACGAGGTTGAGGTCCAACTGTAGTCCTGACACCCTCTTGAACCACATATCGAAGCTCGCCCACGACTGGCACGCGTCCAAGTCTCCAGGTTTGGCGCAAAGGGCCGGCTTGGTGTGATACTTGGCGAGGCCCTTACGCTTCGTGTACGCATAGACTAAGTTGCGAACGAAGGGCACGCCGTACCAGCATAGAGGCAGTTTGGTGGGGTCGGCCACGTCCACATAGGCAGGGTCAAGCCAACGCGGAATGGTGCCTATGGGTAGTGTCTTGCGGACGAGGTTTCCCT

>AT4006

ACTTTGATGAACAAATTGCAAGGGTCAATGAGCCCGCTGTGGCTGGTGGATCCCGGTCCGCTGGGAGGAGCCAGCTGCACCTGCTGTCCAGGGCCGTGGATGAATACAGGTTGTTGACGAGGCCCACCGTAGGGGGATTGCCGAGGCGGGGAATACTAAAAATGATCATCTAGCCCTATTCGTGCGACAACTCTTCACGCGACATACCTGGGCAGGGTATGAGAACACGGAGCCGCTGGGCACGAACGACGGCGCGTTGGGCGAGAACTCGCTGACGATGCCCCCAGCACGGCGGGGCTGGTATATCTGGACGACGATGTTCTGACCCTCGACGTCAGCACAGTGCATGGACTCTTCTGCCTCGCGCGCGTCTTCTTCTCTCCAGAACTCGATCACGCCAGTGTCCTTGCCGAACGGGGCCTGCGTCCGCACCGAAGCGAGGGCGCCAAACACACGGAAGAGGTCGTAAAGTTGTGAATCTGTGTATCCAGGTGGGAGTTGCTTCACAAGTCGTGGGGAGGCGTTAGGTGGAGGCAAGGGCGTGGGAGGCGTCGTGGATGGGTATGGGGAAAGCGTAAGTGGAACATTCTGGGTCAGGGTAGGTATCGGGCGGCCCTGTAGTGTTGCAAGCGCTTTCTCCGCTATGGCAGAATATGGTCAACGTCAGGAAAGACTGACAACGGGAATCAATCAATGAAAGCAATCTAAAGGCCGCACGGGCCCCGCGCCTATCCGAGATTTGCACCTCCCGAAAGACGGTTACGAGAAGGCGGCGCGAATTTGTAAGTGCAG

>AT4013

GCCCTGCTGTGTTCGTTATGCGGGATCTTTCTCCCCACTATCATCTTGCTTTGTAAATGCTGTCGAGAATGCAACCAATGACAACAAACGTGCAGCGTACGGCGAACCAACCGGTATTGACCGAGGCATGAACGAACCATAACAGTACCATAACCACCGACTCCCGCCGAGGCGAGCAAACCTATGCGCCCACAAACACCCACTGCTCAAGCTACAAGCATCAAACTTCTCTGACAACCTCCAAAACACCCCGGAGAAATCCAAAAACAACTCTAAATGATGAGAACACAAGCAGGTACCAACCCCGCAACAGATAACGAACCCACTCCTAGCCGGGCCCGAGCGCACAATCGCGTTACATCGGCGGAAGTCCACGCGCAATCCGGTCCAGAGCAGCGATCGCGTCTTCGCGCGACATGCTGTTGAGTACTTGTGCGGCTGTTAGGGTTGGTGTTGTCTCGGAACCCATGGTCGCGTGCACGACAAGCCGTGCGATGACTTCCCCCT

>AT4015

CAATGCTTTATATTGTGTTGGAATACATCATCCCCACAGATGAGAGCAGCCTAAACAGTGTGCGTCGACGAGGTAGCGCCGGGGGAAGTCACAGAGAGTAACAGAAGATACTCATGACCCTCACGCAACGCGCCAAGGCACCACCTCTTGTTCTCAATGTAGCTGCTGAGGACTTCCTCGGGTTCACTATGATCGACATAAAATGACGTATCTGCTAGTGAGATGGAGCCATCGTCGGCTTCCTCGAACACGGACCCATGTAGCTGCGCCTGCCGCAAGTATTTGATAGTTGAGCTCTGGAGGGCCTTCGGAGTGCTCTCTTGGATATCTGACAAGTTGATTCTACCAATATCTTGGTCTGAAGGTCGAACTTTAAGCTTGGGCGGGATGGAGGGTGAGTAAGATGATTTACCTAACATGTTGCAGAGCGCATTACAGGCAGCGGGATGGGTGGAGGCGATGCCCATCATGGTGATGCCAGCGAACCAGGGAGAGGCGGTGAGGGCCCCGGAGGAGGTTTGTCGCTATGCGTTTCCTGCGCTCGTTGGCATCGACGACAAGCTGGGTGACCCAGCCAACTGTTTCTGCGAACAAATATTAGGTGCCACGACGACGTGAAGTGGACATGAACACACCTGTTTCAGAAGACCATTTCGTTGCGAACGCTTGGCCCACGAGCCTGCCGTCCTTGTACATCATGTGTAGGATGGAGTTCTTGGGGTCGCCCAAGCACTCGGCCCGCAGCTTAGCAGGCGACATCTTGACACGAGCACCTAGAAAGATAGTTTAGAAGTTAGGAAACGGGAGAAGTGGTTTGCTAAGTTTCTTACCTGGTTTCAGCGGTTTGGGAGCTTTGGCACCCCAAACCCCATAATTTTCTGAAAATAATGTAGCACATGCGACGAGCTGCTCATCGGTGACCTCGTCACCGAGTACGATGCGGGGCTTTAGGCCAGGTGGGATGGAGTCACTGGTACTGGAATGATAGATGGGTGCAACGGAGTCCTGAGGAGTAGTATGGGATGGGGAGGTCATGATGGACGACCATCACTTACAGGTTTCGCGATGGTATTACCGCCGATGACTAGTGACTAGTTGACTCCCCCAGGCTGAGCTCGGTTGACGTAATACTTGGAAGGGCTATAATCTCCGTCGACCTCGGGACTCGGGGAGTTTTCTAGGTAGCATCTGCTCTGTACGTTAGTCCGGCAATTTTGATACTTAGTCTCAGAATTCTAAGACGGCTAGCTTGGAAGAGGAGCTAAACTAATTGTTGTCGCGAACATTTTG

>AT4021

AAGGCGCATATAGTGAGGTCCTACCATCCTGGAGGCAGCGCGCAGTTGAAAGGCTTACAATGACGGACCGTCCGTTGGTATACACAATCTTGTCCTTGCTCGAGCTGAGCTTCGTTGCCGCTCCTCGCGCGGTGGCCGGGTTTACTGGATGCAAAACACCTTTCTTGAAAGACATTATACCAGAAGATGGGGGCAGATGATGGTATGTGGAAGAGAATTGGAGGGATTAACTATGGCTGTGGCTGCGCTCA

>AT4041

GTGGCAAACATCTAGAGTATTCCGACTACTAGATCAGGTCGTGGCGGCGGGAATGCGCGCTAGCAATTGGCTCGCGAGTGGGCTGACGAGCGGGAACGACCCGAGATTCGTCTTGAAGTGCTCTGGCGGCTGTGGGTAGAGCTTGCCGCTGGCGTAGGAAGCCTTCGCAGCGGGCGCGATGTAGGGGTGGTCCTCAGTGTTGAATACTGTGACGTCTAGAAAAGTTTCAAAGTCAGACAACATTGTTCACCAGAAAGCGAGACGTGAGAGAACGCGTGTACTAACGCTCGTCCAGACCGAATATGGTTGGGGTCGTCGAATATGAGGTAGAGGTACTTGAGCACCTCTGCGAACCAGAAGCTCTCCATGTTGCCGACAAACCCTACGGCGGCCATAAATGCGGTCCTAACGCGCCGTAGAGTGGGCCCAATGTCTGACGCTTGGTGTTGGGATGCATTGGTTCATCTAGGGGGTATGCGCCGCGGACAAATAGCAGGTGGGATGGTAGTACTCGCGACGACAAGCGGATGGGCGGTTTCAGACGTAGGTACAATAGAATGAGGGCGTTCAGAAGCATGGCCTGTTGGCCTACTCGCGGATCGTCTAGGGAATAATTACAGTTCAGTGCACATATCCTACAAGCGGCATTATTTGAGGGGTAATTGGTCTCCTGGAGTCGCAAGGCGGCCAACGCCTACAAGAAGAACGGCTCGGTGAGGACAC

>AT4049

GCGGCGAGCCTTTCCGGAAGGTATTTAATCACCTGGGGGCTAGGGATGTTGAGGAATGGGGTATGCCAGGTAACGTGCCACTCACAAACGTCTCTCGAGAAACCTCCCAAATCCTTTGACACCCATCCACCACAGGCCACTGAGTCCAGCTAACAAAGGTGATCTTCGGTGTCCCCAGGTTCCGACTGGTCCATGCAGCAGGCAGTGGAAGAAGAAGCGAGGTGGTGGTTTGGAAGAGAGTCAAGAGACGAAGCTCCTGAGCGCCCCGCTTGATTCTCAACTTTCGCTACCGAGCCCTCGCCGAGCCTGTCCCCCGCGATGAAGTTTGACCATCGAATGCGTTCACCTCTGACCCCAGTGGAGGAGAGCAGACACTCGGTACTCAATTCTCGCT

>AT4050

ATGGTGGCCATCGAGCAGCGATGGGTGCTCAGGCTCTGGAGGCACCCCGGTGCGATACTCTCCGCGAAAGCGTTGATGAGTAGGGGCGAACATTTGCGATCCGAGCATTTACACCTGTCGAACTGCAGTTTCTTGATATTGAGAAGCCCATGAAGGACTGCTGGGTTAAATGGGGTAGATGGTTTGAACCCGCCCACGTTCCCGCCCACACTGAGCTCGTGGATGGAGAACAGAGAAATAATGCGTAACAAGCCTGAAATCGGCGATTTGTGGTGCCAGGCGTACACGATCGACGTGCAGAACGTCGAATCGAACGAGAGCCTGGAAATCGAGGTGGAACGGGACAGGAATATTATGCTCTTCAGTGCAGGGGGGAGGCGTCGAGGTAGGTTGAGGGGGAAGGCTATAAACGAATCCCCGAAAACGTAAGGACAGGAGATTTCGCAACAGCTGCAAGAGTCGAGAGACAACGGCAGCATCAAGCTTGGTGAGAGGAACAGATAGGTCGGCCTTGGTCTCGCGCGTCGCGCCTTCTAAGGCGAGTGTTCGAGTCTTCTTGCATATTGCTGGGTTGTCTTGTAAGAAGGATAGAAGGCCATCGAATGAGGCGACCTCAGTAACAAGCAAGCTTGTGGAGAAGAGACGCTTGATAGCCACAGCACGCACATAAGGACACGTCACAGTGCATGCGCGCAGCGTCCGTCTGTCGTCTTTAATTTGCGAGACAATGATCGACAGTAGTTCCACAGGCAGATCACACAGAGTGCGATGCTTGTGGGCACGGGCGAGCACAGGCTGGTGGTGGTGGTTGGTGCGAGACATGTCGGCGTCCTGGCAACTTTCCC

>AT4064

CGAGTCCGGCGATCGTGATGGACGCGAGGTGCCCATCGGCATCCCAGGTGAGCGGGAGGACCGCCTGAGGGAAGAATACCCCCGAGCTACTAAGGATTCCGTCGTCCGCAAACGGAAGGATGGTCGCGACTTTGTTGCCGGTCTTGGGGTCCGTCAGGTTCCCGCTGAAATGGCGAACGTCAACGTCTGGGTGTGTAGTACGACTCGATTCACAGTGCACGTAGCCCTCACCCGCTGACGACGCCATGGAGGCGAATACCGAACGGGCCCGTGGTGTTGATACCGGAGGCGATAGTAAGCTGCCCTGCGAAGAGCGGTTCAAACTGGGGCGTAGGAAGAGTACTCTGTGCAGACACTATGGTAGAGCGCGTGCCCGCGGCAAGGATGGCGATGAGCGAGAGGAAGAACGAGGAGCCGCGCATCTTGTCCTGAATGTTGGTGTGAGGTGCTCGATGTACTACTGGCAGTGGACGTTGGC

>AT4066

CACCGTTATATAGCATGACTTGGACGAGCGACGGGGCCTTCAATACTCCCCGTTGCGCTTTGTACGACGTATATGTGTTGTGCCATGTTATGAAGAGCACGATAAGGTCCCCTGCAATGCCGAATATGCGAGCTCCTAATGTTACTATATAATAATGAGTTTCCTCTCTCGCAACTATAATCGCGTACAACGACATACGCCTGGGTGTTGATGCCGTGACCAATTTGAATTCAGCTTGACTTCGGTTAGGATAAAGCGTGCTCTTACCGATTCGCCATCTGTGGGGTAAGAGGGGTCGCGACAACACTACAGTTGAAGGGATTCGGTTGCAATGCAAACTT

>AT4070

CTCATGATCGTCTCTGCGACCTCGTCGTGCAGCCAGACAAGCTACCAGCTCGCCGTCTTGCGCCGTGTCGATTCCAGGCCCAGCTCGCTCCGCGACGCGCCAGGCTCGGGCGACGTTCCATAGTTCGTATGCCTTCCAATCCTCTTCACGACGCCCGAGGTACCCGCTCGTCGTAAGGGAGAGAAACTGGAGGGAGAAGAGCGGCCGAGCGAGTGCGGCCGCCGTCCCGTCAAAGTCGAACGCGGACCCGCGGATGGCGCGTACGAAGTGTTCCGAGTGCTGGATAGCCCAGTCCGCGCCGTAGTAGACGGTGGCGTGGACGACGACGCGCAGATGGGTGAGGCTTTGCTGGAGTATAGGGAGGAGACCGTCCTGTGCAGACCATTAATGAGAGATAGGCGCGTACACGGATGATACGAGAACGGAACACCGTACCAGAAGGTCGTCCCATTGGAGTCGCGCAAAGGCCTCTGGATCTAATAACGCGGCCGAAACGTCGCTATTAGAGTACACCAAGCAAATCATGAGGTGTGTTAGCGCATGGGTCAGCTCTTGAGATAGCAAGCGTTGA

>AT4111

GTTCCTGCAGGTATCAAGTAGCCGTTATATTCGTCATCCTCCATCGCGCGGTGTGGAACACCTAGTGGAACGACGGAGTGCCACCGAGTGCTCTCTTTCAATATCGCATTGATGTAGGGAAGTGCTTCTCGATGACAGAAATCTGGGAGCTGATCGAGACCGACAACTTCGTCGAGTTCCACATGGGCCTTCCGCTGAACTTCAGGGTATGTTGCCATCGCTAGGAAGAATGCGCTGAGAATAGTCAACGTCTGATACCGCAACATGAGCCGAGGAGCCTGCGAGATACGACAACGACACTTGCCGTGTCCGCACCCGCTTTGTGAAGGAAGTCTACTCAGCCACGGCGAACAACGACGACGAAAAGGATGGGGATGCTCACCAGCGTAAGCGAGGCCCGTAACGTCCTTGAAAATATCCTCGTCTTCAGCGGAGACC

>AT4112

CACCAGGGCCTCTACGGTGGCATCATACGCGTCACCCCTGAGCGCAGAGATGACGGGCTTCCACTTCTCCGCGTTCCTCTTGAAAGTGGCGCCAGGAAACCAGGATGGCAGTCGCGCAAGAGCTGGGAATAGCTCGACCAGATATCGGCCGGGAGCCATCACATCGGCGAAGATCTTGCCTCCCTCTTCTCCGATGGCCACTAACCGGTCGTTCTCCTCCGCGATCTCAACCCCATATACAGCGTCCATGATGGAACCCCCGACGTAGCTTGAAGACGCGGTGAGCACATGCCAACATCGTCGACATCAGAGGGCACGACATACTATCGACTGTGATCGAGGAACTTGGCCGGCTCGTCGAGCAGCTTATTGAGCAGACGTTGCACGCCGCGGATGTAGATCGGCCTGTATTGCGCCACCGCACCCTGATGAAAGAAACTATGGAACACACGGCGGCGCTGCCTCCAGGCGGTTGTGTAACCCATGAGGCCAAACTCGTATATAAGGCCG

>AT4140

AATAGCCCCACGTCCTCTTCTCCTCCGGGACGGGGAGAAGATCTTCATTCTGAGGCGAATTCGGTGAAGCCGTGTGAGCCGATCGTCCAATGCACGCACCTGCAAGAACAGCTCCGAGTACGTCAAACTCGGAGGACGATCAACTTGTAGAAGGTGGACGATCCCCATGGTCGGCTTCTAAACGCGACGAAGTGGGGAGCGAACTTGCAAGCGGGACGTGGGCAGACGAGTCGTCGCCACTGCCAGCTGCGCCGTACTCCGTATTAAACGCTCTCGAGATAACGTAGACCTGGGGCTTCTAGTAGTAGATTCTATCGTCGTCGGGGTGCTTCTGATAAGGCATGAAAACTCAGTCTCAACCTCAACCTGTCAGTTCAGCCGAAGATAAGCATGGGGTCTACTGCGCGGCGTTTTAAGCAGTTACTGTACGAGTAGAGTCCAGCGTATTACGGTTGGAAACAAGCCCC

>AT4143

GTGGGGCTGACAGCAGTTCTTCACAATACTATGGTTAGCGTTAGGGAATAAACATTAGATAGCAAGTCATAACGTGGTTCCAAAGGAGTGTCCCGGCGAAATATGCGATGAGTACAATAACAGAGCGAAACCAATACCTCCCTCTAACCAAACCCGTGCGTCATACAAGGATCACGGCGAGCAATAGTGCCATAAGGATAATAATCAAGAAGACGATACACCACCCAGACTTGGTTTCT

>AT4144

GACCGATCGACGCCGCGCTCGAGGTCGTCGAGCATCCTGCGACGCGAGGTAAGTATGCTGTGTCTCGGCGACAACTCTACACGCACTCGTTGTGCTCCACAATCTCACGCCCCATTAGGCCGGCTTGCTCGTGGATCGTATTTAGCGTCCCCGCGATTGTGTCAATGGTCTCGTCTTGTTGCCGAATCATGAGCTATACCGGTCCCGTCCTTTGTCGTTAATGGCATTTCGTATGACACAAAAAAACGACGCACCTGCTGCTCCTGCCGAGCCCACTCAGCCTGGCTGTCCTCATACTCCGGACCCGGCTCCGCAAGACGCGACGTAGGCGCAAGTCCGAGCACGGATGTAGGTCGTGACCGCCGGCCACCCTCAACTTCCGCGCGCATGG

>AT4167

CGGGTGCGGGCGTGGAAGGTCTGAATGAAGTAAACGATGGGATGTTTATTTCCCGGGAGGTGCGATAGTGAGAATTCGTCGCGCGGATACAATTATTCTACCGATAGAATCCCAACTATAAACCAACCAGCGCAGGATGGTCAGTACTTGACGGGCTCGACGGGCCGTCGGACGACAATGGCAAACCGATGTGCATCCACGCTCATTACCAACCAACCACTGCGCATATAGGCGAGGGATCCAAGGGAGATCGGGTTTCTTCAGGGGTGAGAGCTGCACGGCTCCGTTAGGATTCAGTTGGATAACAGCCGTGCATCTCGCTCACTTCAAGCATGGACTGCATGGTCTTTCCTGCGCAGTAAACATCAAAGGAGTATGGATCTAGCGCAGTGACGCCTGAAGGTTTCTCCTCCTGGCTCGAAGGTAGGACGATTGAAGGGTGGTAACCGGGTCCTAGAGCGAGTTGTCGGGAATTGCCAAAGTCGATGATGTACACTTTCCCGTCCACTAGTCGCGTGTCAATCGCCGCCTGCCACGGTAACGCGTACGCAATGTTGTCGAAACATATGTCCTGTACCAATGTTCACCATCAGCGAATTAAGCTACGGCCAAGTACGAAACTTACTAGGTGTGTGATCCGTAGACGATGGAGATACTCGACGCCCTGTACACGTGTTGGAGTTAGTGTTCCAGGCATCCGAGTGGTGGACACACTTCGATGATTTGATGAAAAACATCCAGGAAGAAGCTAGTTGGTCGATGAGTGTAGCTAATCGAACCGAGACTTCCCACAAACGGCATTACGAGCAAGCGAGGCTCAGAAGGGATGATCTCCGAGGGTAAACTGTGGTTCGGGGAGGAGGGATTCTCCTGCAAGCGCTCAGATATCGGGCCTTCCTCGGTAGCGGAGTCTAAGACTTTAACGACAAACTGGGGGATGGTGCTCCCATGAGGTCGCGCTGTATAGTATGGACCTCCATACTAAAAGTGAAAGGAAATTAGTTGGCGAACACGTCAAGGACGACGAGCTCCAGACTCGAAGACTGACCGGCTTGAGTGCTCCAAAGAGCTTGATATTCCTGGCTCGTAGGTCAGGGTGTTCTTTCAACCAGTCTGGGACTTCGAGTGCTGAAGGCATGGTAGTTGTCGGAGCCTGGAGAAACTGGAGCGAGCGAGAGTGGCGGACTTCTGCAACGTTATGGTTCAACGTTCACGTCGTTTGCC

>AT4170

GCGGGCGAAGTCCCATTGTTAGCTCGATATGTCGTAGCAGAGACGGTTATGTCGCGCAGCTTACACATGCCCCAAGGGCGAGCGCAGATGCGAGGCCCCGACCATCAGCAAACCCGCCAGAGGCAATGTACGGGACTTGGAGCTCTTGTGCTGCGCGGGCGAGCTGACGACATTCATGTCGTCAGAGGGACATCTAGACGGGCTAAAGTTGTGGGTGGTGGCGCACCAGGACGAGGCCGCCAATGTCTTCCTCTCCAGGATGGCCCGCACCTGGAAAAGAAGGTCAGGCCATGCCGCTAACGTCGGAGCGGTTTAGTCTTGCATTCAAACCCATCTATGCTTAGGCAGTCGACGCCGAGACGCTGCGCAGACTGGT

>AT4171

ACGATTGTTCCCTGCGGTCTCGAAGATCCGCACGCCCTCCTCGACTGCGGCTCGCGCGTAGCCCTCGTAATCTGGGGGATTGATGCTCGGCAGGAGTGTAATGTTGACGCCAAACGGATACTTCGTCAGCGTTCTCGTCTCCTGGATGGCTTTTCGGAGCGCATCGGGAGAGGGCTGGGTGAGCGCGGTGAGGACACCTAAACCTCCCGCATTCGAGACGGCCGCGGCAAGCCTCGGCACGCCAACCCTTTCGTCCTGTCGTTGAGCCACTGCTTTGGAGGCGAGTAGGCAGCGTGCTCACCATTGCATGCCGCCCTGCACTACTGGTCTGGTGAACACATGTCAGTTTGCTTTACTCGTCAGGACATGGAGAAGTTATCTTACACTCGGATACTAAGCAACTTGGTCAGTTTCGTCTCCAGAGGCATTGTGGTATCACTTTGGGATCACAGGGTAGTATTGGGTAACTGAGAGAGCTCAGAGAGGTATAAGGACAAGCAGAGGACCACCAGGGCGGCCTCAGTCAGCTCAGCAGCGCGCTCGGTTCATCGTGCCCCGCAACCGGCACCCCATGTGTTATCC

>AT4188

CATACTGCCAGAAGGCCGTGTACGCCCATCCGGCAGGGAGGGTGCCGATGCTCGACGCGTACCGCGCAATCCAGAGAGGGTTCGTCGACGCGAATGCCGCGCTGTTGCCGGTGCAGGTGGTCCACCAGTCAGTTGTCGTGTATATGACTGTGATGGAGGTGTGACTGAGCAAACGGTTGCATCATGTATCTGAATCACCCTGGATATTCTCACCTGGGTAGACGCCAGTGTGGGAGTGGTAGGTGTCAGAGAAGTCTTTGATCCATGAGACCATCGCTGACGCGCTCAAACCGTAGCATTCGGCGCCGCTGGGGTTGTCT

>AT4189

CAAGCGCACCAGGCAGGGTGATGCCATCGCTGGACCACCCTCCCCCGTGGGCAAGAAAGTACTGCGCCTGCGTCGCCCCGGACGAGGCATCCGGGTGTGCGAAGTGGTATCCTCCACGGATGAGACCGGCGTTGGTGGCCGCGACGTAGTTGTTGGAGAACTCGGGGTCGAGGTAGGCTGCGGAAGGTGCATCATGAGTAGGCATTGAGGACTCGCAATGTGAACAAGACTGAAGAGAGCCCACTTGTGCCCTCCGTCGCCTTGATGTAGGCGAAGGAAATGCCGTTCGAGGCCGCCGCGGGGAAGTTGACGGCGCCTTGGTAGTGCGAGACGTCGATCCCCTGCGGGGCGGCGCGCTTCACGGGTGTCGCGGACGTAGTGGCCACGGTCACCGCGAGCGCGACCAGAGATGCAGTGGTGAGTTTCATCCTCGATATCTGGGCAGGTGTGAGCGCTGACAC

>AT4207

GCCAGGGAAACAGTGTTTGTCGAAGACTGCATGCGACGAAATGATCACCTTCTTCGCAGCAGGGTCCCAAAATCGCCAGGCCTTTGTGCCCTCTGGGTAGCCAATGAAGATGCACTTGCGCGTGTGGGGCTGCAGCGCTTTGCGCTCCTTCTTGCGAATAAGCACATAGGCCAGGCATCCAAAAACACGAAAATAGGACACATTAGGCTTGTGTCCATCGCCTTTCCTGGCGGTGTAGGGAATGC

>AT4224

GCTGCCTAGTTTCTTCGTTTATAGTCTACACCTTGTATTGCAATCCATTTTTATGTCTTCCTTATCCCTTTATGCATCATGAGCAAAAAATAAGCTTGTTGTGTGATACAATGTGTGTACATGAGCAGAAAGTCAAGCAAAACCAGTGCTGAGGAATGTTCAGAGCTTCGATCCACGCTTGTCCAACCATTGCAGCAGCTCTCCCGTCGCATTCTGTGGTTGCGCGAGGCGTCAGTTCATGGCGTCGCAATGCGCTGTGTGCATGGCATACCTGTACCCATGCCGCTCTGGCACGGATTGAATCAAGGGTTTGCTTGAGGGCCATCTCGAATTTCGAGGTATCCTTGTCCTGCAAAATTGAGAGTCAGCAGATGGTATCGATCACTTGGGCGAAGTGCCTTGCCTTGAAGAACGCCCTAGTTTCTTCGTAGTCCTTCTCCGAAGACAAGCCCATGAAGCAAATCTAGCGGGTCCGAAGTCAGGGAAGCCCCGTTAACCAGAGAGGCGGGGACCATACCTCGGTCCATCGGATGAGCCCGAAGTTACCCGCGTAACGCTTCTGAAACTAGATAACCGACATGCGTCAATCAGGATAGACGCGACCAGCCTCTACGGCTGACACACTCACAGTCCCAAAGTGCTCCTTGAACTTCTGTGCGACAAACTTCCTCGTCACGTAGTTGCGCTGCAAGCCGCCGGCGTAGTAGAATGTGTCCTGGTCGCGAGCCTCGTTCAAGATGAACTCGAACGTTTCCTCCGCGAGCCCGAGGTCCTTGCTGGCACCCATCGCCCTCATAGCAGACAGGCCCTGGAAGGGGTTCTTGGGCTTGGACGCGATGCGCCTGACGGCCTCCCACTCCGCCCGTCCTCCTTCCCGCACAGCCTGATGCGCACGCTCAGCAAAATGTCCGCGTAGAGGTGCACAGATAGACTCACGATACGGAAAATCACACCAGTCAGATCGCTCGGGATCTTGGAATCGTCGCCGGTCTTGAGGAAGTGGTCAAAGCGGCTTTGCAGTTCCTTCACGACCCTGC

>AT4233

GGCCGGCGGATCCCACTTTAGACACATAATACGCCCTTACTTGATCGAAGGCAGGGTCGCATGGGTGGGGGGATGGGCAAGGGGGCGCTCCTCTGTACTTGTGAACTGAGCGACACGAAAGCCTTCGTTAGCATCGAACTAAAGCCAGAACCGCAAAGTTGGACTTACACATTGTTAATTTGTTATGAAGTTATGGTAATAGAAACGACCAAGTGCATATATGGACGCACTTTGTCTCCTCTGGATGGGATTGAAGCATAACTGCACTTCTGCTCGGAGGATGGCGCGAGCTTCCAGGCGAGGGGGGGAGGGGGGGGTTGGAGAAGGAGTCTACCAAGGGCGCCAGCGTACATGATGTCGTCCTTATTCGCCGTGTTGGCTTTCGCCGCTCTCAGGATCTTGTCGTAACAGTCTAGTTCATCAACTCCTAAGCCTAACCTCCGGACGTCATATGAGGCTATCGAGGGTAGTGATACGGACGGCATATTGGTGTAATGGTCTAGGTCAAGTTAGGAGCAGGCTGAGAAGGTGATTAGAGTATTGGTTTGAGGAGGAGGGGATGGGACGCCTTAAAGATGTGGGAAGGTGTGCAAAGTTCAACCCGGGATCAATGAAGGCGACCTGGAGTCTTCAACTTCCATCACAGTGAAGAGCGACGTCAACGTGCCACTGATTGGCTACCCTGGGCTTGGCTCCGAGTCGGAGCGTTACGAACGACCTAACTGCTGCTCAGGAAAACACCAGGAAGGTTGACATATCGACAGCCTCCATTGCACAAGACGGGAACGGTGTCTGCGGAGATGGATGAGCCGCGCGATTCGGCAGGCTGGAATGAGCTTTGTAATGGACTAACATTCGTCGAAACAGCGCACTGGCATATTGACTCATTATCAGGTTTGTGTTCAGAGCGACTCGGAGTAGAATGTGCATGCCTTGGTCAGACATGACTCGGCATCGGT

>AT4237

GCGCTGTAACTTGAACAATGACTCTGATGTCCCGTAAGCCTGTGCGCTGTTGCCCATCTGGAGCCCGTTCCAAGCGAAGTCAATCTGCTAGCAATCTATGCATTCGTATCAGAGGTATCCTTGTACTTTAGATTGCATCTCGGTTGCAGTCTGATCCGGAGGCCTCGCCCTGCATTATAATAGGCACATCCCTGCAATCCGGTGAGCTTTGTACGATCATCGACCTTTATGACTCGAACGCGTTGCGCTTCTTGGTGATTTCTTTGTAGTGCGGTAGGTCTGCGCAAATCTGGTCGAAGCTGCATACCGAGTCAGCACTTCCCGACACAGCTGCGGCACTGATAGCCGACGCTTACGGTGCGA

>AT4241

ATCACCATGTTTCGAGCTAATAACTTGCCCGAGGTCGTCCCATGTCCCATTCTCCATCGTTGGACTAGTGGCCACGCCGATGGCAGAATCCTGACTGCCACTACCATTCGATGCAGCGTAGTACATGACGTATTTCCCGTCGACAAAGTTTACGTCTGGCGCCCACAGACCGCAGGGGCCGGGGTTGTCAGTCTTGGAACAGTTCCACAGTGCGGAGCCCTGGGTCATCCACGGACTACACATCAGTGAGCAATGGGATCCCGACACGCACATGAGAAGCGACTCGCCCATTCAGAGAATCGCTAATAAAGATGTTAATTAAGCCACCGGTAGAAAACACGAAGTACTTCTTGCTGTCGGGATTGTACTGAATTGCGGGGTCGCGAACGACGATGGAGCCTGTGGGCGACTAAGTATGAAGCGGCAAGAGGCTAGAAGGGCTCTGACGACTCACTGCTTCCGGGGAGGGGGTTCGGGACAGACAGCGCTAGCGCGGCAAGAGAAAAAAGTGATCCAAGGAGGAAGTTCATGATGACAGCAGGGCGAGGTACTCAGGATGCTACGCCCAGCCCAAAAGCGATGAAGATGGGGTGCATATATGGCGTTCGTGTAGCGCCATTGGATCCCTTCCGTTCGCGTTCGCCGTAGGCTCCTCCGCCCAGCGTCGCTCAATATGATCAGCTTTGGAGCCGCTTGCATCGGTATGCTCCAACGGACCGCATTGAGGGTCAAACGTGTGTGGACGCCGCTGTCATTCTTCTTTAGATTGTTTGGAGAGCTCCGCAACACGAGACGCCCTCCCTGCGGAGCATCTGGCATCTGTAGGGTATATCGGTACCTTGAGAAATCAAAGAACGCGCATAAAACGAGCCTGGCACTCACCTGAACCCGTCGTGGGACCGTTGCGCAGTCTACCTCAAGAACCTCACGTCAGTGAAATCTTAGTGAACATATCGTGCTTGAGAGCGTCTGCTTACGACGTCACAGCAA

>AT4257

TGGCCCACTCCTTCGACCTTTGACATCTACCTCTACCAATTAACCGTGGCTAGAGGATTACACTCCTAGTAGGATTATAGCATGCTTTTCGAACGATATCCATCCACGTAGTGTTGCGGTCACCTGTCAATCCGTTGAAGTAGACCAACTGTCACAGTTGTTTGAAGCACTTACCTATCCTCCAACAGCCCTTCCTCCCCCCCGTTCGCGCTTAGGATCGGGTCAAAGCCCTTCGGGAAGACTTGCCTGACATTCACGTTGATCCTGGTTCCTCTAAGGTACTCCTCGTAGACATCCCATTCTTCTTCATCAGATCCCACATCCGGACCCTCAAGGTGGGGTGGAAGCGTCCCCTCGAGTACTCTTGGTACGCCTATGAATACCATGGCTCATGAGAAGAGACACTACAAGCAAACGCTCGACAAAAGACCGACCGTGGTACGCCCGACGGCACGCAGGACCCGACATGATGACGATGTCCCCGGAACGCAGAAGGATGGGGGTAGGTGGATCGTCGCGCGTGAGGCCTCCAATGAGGAAGACTGCC

>AT4261

CGGCTGTGCCCGGGGATGAACAAGAATTTGGGCTCGTCCTCGTGCGAGGCTGCATGCCAATGGTGTTGGGGAAGGGCGAGCTCATGCTCGTGGTACTGCGAGTAGAGGAGTGGCAGACGTAACCCTGACCATCTCGATGACTCACGTTCTTGGCTAGCGAGGGGAAGGCTTCGCGAAAATACAGCCTGCGAAGAAGAAACGTGACGAGGACGAAACTGGTTATGCCGAATACCAAGTGGCGTCGGGCGACATAGCGTAAGCAGAGGGCAGGATTAATCTTGTCTGTCTTGACCGAATACCCGTTGAGGTCGCAGAGGTTTATAGGAAGGAGAGTCATGGCGAGACAAGATAGAGAAGGAACTACACTAGCGCATAGGTAGGTTGCGAGGCTAGCTCTTGGAGTTCGACCAAATTTCACATTGAGGCCTAGGATGTCCAAGGTACGAGTCTGTCAAAGCGATTGCGAGCCAGGCCGTGTCGAAAACATTCTCCGACGCGTCGGGGTTGTGGTAATCGGATTTTGTTCTGTTCAATACCGGATTAAACCCCCGTCATTCACACGTTGGTCTCCCCGCCAAGATAGGAATCATGCCCAGTGGTCCTAATGATTCTTGAGAGCAAACCCCATGCGACGTAGAAGGGAGTCATCACCTGGAAATCCGCGCCCAGATGGGTTGATTCCAAATTTCCCTGGAAATGGATTAACTGTGAATGCAACTCGCAGGGGATCGCATAAGATTTACCCGTAACCACTATGTGTTGACAAGCTAACGGTAGCGCCACTGCAAAGACAAACCGGCGCCCTCAAACACGATGTCCGCTTCCAGCAACCAATTCCAAGAGTATAAGTAAATCGTCTGGCGTAGACGAAGCTGAGCGGGCGTGCCAGATTTGGTTTCGCGCGACACTTGATATCCGATATCGTAGCCACCACTCGGATCGAAAAGGGAGGGGCTCGTGACTGATTCAGATCGCCTTAACCTGCATGACTGTGGAGCATGCCGTAGAGAGGCTTCAAGAGCCTACGCCGCGTCTTTCGGGTAGGCTACGGAACACGGCGCTCCCGCTCGCGCCGGGTAGGATCTACGGTTTCGTAGTAAGTAGCCTGAAGTCGTACAGGTGGAGGTGAGCACCCCTCAACGGCTTTATCCTGCCCATAGTTTCGGACCATTCATGGGCTCGACGTGGGTGAAGATGCAGATGTTCCCTTTGCGACAAGCACAGTCACGGCGGCGGT

>AT4278

GTCTCGGGCGCGTGGTGATGCACACGCACGCGATCCGACGGTGGAGACTGCCAACTGGCGACGACACGGATCAGCGTCGGGATGCTATTGCCATGTCGGACAGGGGAAGATCGGGCGCGGACAGGTCGTCTAGAAATATATCTACGGTTACAAGCGGTGTGGTGCGTGTCAAGAATACAAGGGAGGTGGAGATACCAAGGCTACATACGGAGGGACCGGGACCAACAAGCGCATCGCGCCAGCCATGATGTCACACCGGGGTCGCTGAGATGGTGAGACGAGCGGCGTCCTAGGCTTCAATCTTCACGCCTCGTCACTTCCCGTCCGCCCCACGGTGGAGAGGCACTGCTCGAGGACCTCCACCAGCGCCGGATTCTCGAGCGCGGCGGC

>AT4288

GACGAACCTAATCATACCCTGGCATGACTTCATGATCTCCAGGATGGGTTCCGTAAGAGCGGAATCGCGAGGGTTCGCGCGAAAGGCCTCGGTGGCTGGTGCAATGACGAACTCGACAGTGGGGAGGGCCATTTCGCTCGATGTAAGTGGAGGTCCACAGTCAAGGTTGTTGGATGGATTGAGGATTGGCGGCTAGGCGAGGTCTAAAGAGTCAAAGTGCGAAGAGCGGGGGCCGCAAGATCGATGAGGGTGGCGATTGTAGATGTGCGTGCGTCGATGGATGGGAGGATGTTTATATAACCGTTGCTGTCCTTGCGCTGGGTATGCACTTACCCCGCTTTTCATCTGCCACTGTCCCGGTTGCAGTTGTCCCTGGCTGGTGACATCCCTCGACATCGCTAACAGCAAATGCGGTGCAAGTGCTTGAGTCTTGGCCTTGACATCGCCACGGCAAGCCCTCTAAGTACCTCCGGAGACGGGCCTCTGCACAATTCTCCCTATCACTCAGAGCACCGGTCAAAATTGCCCCGGTCACACATGACCAATAGTGCTGTAACGGTCGTGGACGTGGAATCGCCGTACCACATGGTCTTATCAGGTATGGAGAGCACGGGAAGGGGTTTGTTGCTTACTGAC

>AT4293

TCCTCGGGGTTGATCGGTTTGAACTGGACGGCGTCATCCGTGTACTCCCTCCGGCATGCAGGGCAACGTTTGTTGAGGTTCTCCTTGATGTGGTGCCAGCAGAACTGGCAGACCTGTGAAAATAGTCGCTTGAGCAGCAGGGCAACTGGATGGACCCGAGATCCCGCACCTGGTAACCGCAAGGGCAAGGCTTGAAGTTGAGATCGGATATGTCCATCTCCTCGAGACAAAGCGGGCACTCTGCATCCTCCTGTGGAAAACATCGAAAAATGAGCTACGTCTTCCCAGGAATAATGCTCTCGAGGGGAGTACGCACCGCCTCGTCGTCGCTCCAGTACGCGTCTTGCACTCCTGCAAGGACGTGCGACTTTGACTGGGGAAGGGCATGGGTGGCGTGCATGGCAGGTGCGGGCAGGGGCGAGTTAGTATGAATTCTGGTAGCCATTTGAGAGGCACGTTCAATAACGTCTGAGGGGCGATTGAGAGGGATGAGGGCGACGGAGGAGACGCAGAACAGTTGCAGAGCTTCAGGGAGAGTGGTAAACGCGACAAATGGGGCGCCAATTGCCGAGTTTTGCTGTGGCACAAACAACACGACCAAATACCCGACCCTCTTTAGACGCCTATAATTGGATCTCTGCACAGCTCAAGCACAGCTCGAGCTTGTCGTGGCGTCGCGGCCA

>AT4294

GCATCCCCACATTCGACTACCCGAATTCAGTCTTGGGCAGATGAACGGCGATCAGTGCGATCGTCAAACAGTAACACACCCTGTAACACAAGGGCAGTCATCGGGCATATCAATTGACCAGAGCTTCACGCCACGGTAAATGTCATGGATACCAACTTGATGGAGCCGGAGACGGCGTTCAGTGTGGTCTTCCGTGGAGCCACAACTAAAAGCTGTGGGACGGACCGGGTGCCATGAAACTATACATTTGCGGCTTCGAGGTGTTGTCCTTTATACCTAGACAACCGATGGAGATGGCGGCGTGTCGAGAGACGTGATTCTTGACCGAGATGATTGAGAGGCGGCCACCCTGCGGTCTCTGACGGACAGTACTGCCTGTCGCGGTGGATATGGTGTTTCCAAGAGAGCGAGAGCAAGAATAGATGCTGTAGATTGCGATGGACAGAGAGCAGAATGGTGTCGGTACTGATACAGATGATTTCCTTCTCGATCCTCCGAATCACCAATCGTCGTCGTCGAACGCATCCTTCCTACGCTG

>AT4299

ATACGGCGTTGTACAAGGAATTGCTTCATGGACCCAGTTAACTTGCCCGAGCACAACTTGCGATATTCATCTGCAATATTACCAAACTCTGGCTCATGGTAAAACAGGTGCTCGCGTTCGACGGGCGGGTTGACTGCCATCCCGGCAGTGCGGTAGGACGAGAACGGCTTGTATACTTCATAAGGAGCGCGTGGAAGGAGGCTGAATACCAGATTCATGAAGTCGGGGTCG

>AT3991

CTGCAAAAAGATTCATGATGGCCAGCATGGGTATGATCCGCCGATCGAGCTTGCGCACCAGCTGCCTCTCTTCCACCCTTGTGAAGGCTGCGCCCGTTTCCCGCCCGCTAGCTAGAACATCGACGACAACTTGTACCTTCTCTTCCGTGTTGTCTACGTTGCGCAGAATATAGTGTCCGGGCATAGTCGATTTCGCAGTAGAGAAAGGAAAGCAGAGAATGGGCTGAAGGACACGGAGGCTCAGGGAGGCCGATGCATATACCCCTCCACACGCCTAAATATACATTGCTTCCTTCCCCGGGAAAAATAACGGGCATCCACGTCAAGCTCGTAAGCCGTAAATAAACTGTAGGGAGCGTACCTCGTAAGGCGAGAAAGGTTTGGCATATATGCGGTCATTTGATTCCCGGTTCAAGCGCTTCGTTCTTCGTACAGTTCGATATCGCGTTGGGGTAGACGATTCGCAATCCTAAGTGGATAAAACCCGTGCGCGATAAGTCAGCGTACAGATGACCTACATGAAGGATAAATTCACCCAAGTCATCGCGGAAGAGGAACGCGGTGTCGATTGACGGACGGAAATAAGAGCGCGGGAAGTAAAACGCACGCATGCCCAAAACTCGGTGCGTGAACACATTGTTACTGTGTCAGTCACCGTACGCGTCGCGCACACCCCGCCCCGCGGCCCCACCCCCAGCAGAGAGACGACCCGAACGGATTGTCCAACGACGTCGTGCATAGATCTAGACGTGTCAGTCGGCGGCCACACGCCTCCGAGTCTGGCTATCGCCGTGCCCCGTCATTGAATGACACCCCCAAGCCTCCCCATCCACGGCACCAGAATTATCGTTCGGGAACGGAAAATCGAGGATGGGGGCAAGGAGTGAGCTCAGGTTCAGATGGTAGTGTTGAGGTTGGGTTGCAATATGACTAGACAAAGCATCGCAGTCTCTGGGATCGCTCACCGAACTTGAATCACAAGAGAAGTGATTCCTACCCTTAACTTGACAGCACAAGTTTCGGTTCAAGCTCCGGAGACGAACAGAAAAGGCGGCCGGGATATGACCGCACTGCGAGAGAGATGAAGGACTTTGGCAAGGAGACGGACA

>AT4682

GCGCCAGCAAGCAGTGTCCATTGGAAACTGTTACAATCTGACCAGGGTTGTATCTATAGTATCGATACAGAATTGTGTTGGACAGAACAAGCCAGAATCGTGGATAGCAGCTTGATAGACAATATGAGAGTGTACCGCGAATATGCTGAACGGGATGGCTAGATGTGGTCGAAATGCCAAGCTACAAAGCTTCCTCGACAGATTTGGGGTACTGAGAAGTGAGTGTGAAGCATGTGAAGAACCATAGACATCGACATCAAGAGGATTGAGCGCCAAGCTTGACCCCATGGGATGCACTAATAGATATTGGGAGCATCGGCAAGGCACGTTGGGGGATAGACCTGTGGTACCGGAAGGCACGGGGAGCAACCAGGAGGTGAAGCTAAGGGGAACCGGGCGATACCCGATAACTACAAGCCCCATTGAACCTCATCCTTCGCTGGAGGCTCGCCGGTTGTAGTATTCCAGCCATGGCCAGCCGGGACATCCCAGGCTCCGTGGCACGTCCACGCCCAATCGGTCGTCCGGATCGTCTCGCGCTGGGCCGCCACAAATGCGGAAACCGGGCACAGTCCGTGCGGATCGTCCGGAGGGCAACCGCGCACGTTCCTCAGGGGTGTGACGCCGTCGTTGACAACGATACGGATTTGAGGCTCTGGTCTCGCCTCACAAGAGAGCACTGAGGGGAAAAGCACGTTGTGAAAAGAACAGTATGGAGAATGGGAGATGGACATACGTTGGAACTGCACGTTGGATGCGAAAGGTGCAAGATGCGAAGTCTTGAAGGAACGGTTGACTGGGATGTGGTCCGAAGGAAGGGGCCCATCCGTGGCGAAATTCGAAAGATTGAGAGCCGTCAAGACTGGAGTGGGTTGGTCAATTTCGGGCATGCGGGAGAAGTAAAAACGGTGTACCATTCAGGATCACCGTGTCATGCGTTGCGTCAACGTAGATACTTTGGCCGAGAGGAAATGTAGCAGGGTTGTCATTGACCGTTGCGTTGGTCGAGGAGTTGTGGACAGAGATCGGAGTGTTGGTAAGTCGGGCAACGAGTTCTTGGATATACCCAACTCCCAGAGCACGAGCAGTAGGGGAGCCGAAAGCCGAATTATACCAGAAGTTGAGGTCGACGCTGAGAAGCTCGG

>AT4687

TGTCGGTTATGGATTGCCAGTTTGGACCTATACCAACTAATTGATAGACGGCTAGGGCCTGGCAAGCGGATGATACGTACTGATGTCGCCAGTTGTCTGAGAGGGTCAAACGACATGGAACCTATGCAGAGCGGAATATGCAGAATGAACACCCACCCGCCAGCTCTGCCCAAACCGCCGAGCCCATAACCAAGGTTGCTCC

>AT4693

GCCTTGTTTTCTAGCCTGTGAAGGTCACCCGACGGACATGGTTTGAACAGGTTTGTATCTGGCCAGCTAGCGCCACAGTAGAGAGGCCTGTTCGCCGACTACGGGTCCGACGACCGTGCGCGCAGGCTGAACCTAGTGCTCAACCACGCGCGCGAACTCGAGAACTTGGAGAAGGTCAACGGCCTCCGGGCAACGTGTAGTACGGGTGTGAGCATCTTGAGCTTGAACGTTTCCTTCAATTGCTTCGTGAAATCTGGAAGGAGAATGAAAAGGCACATACCAGTGGTTCAATGCGGCGAATTTACTGTCTTCGCAGTACGATGGTCGGAGACGCACCGAGGGTAAGAGGGTCAGGCGACGGCCTTCCTAGAAGCATCGGGCGAAAGACGACGGGCACAATTTGCTTGACACAGCGAAGAGTTGGCCAAGAGATGGAGGGATGGGATTAGACACGAAGCATTAGTGCAGGGTAGTAAGTAGTTCTCGGGCATCGACAAGTACAGTGCCGTGTACGCTAATGATGCGTCGGCTTTTTCCCTGACCTCAGACATATAAACGCATGCCCTCACTGGGATGCAGCACCCTACGCACCGTCTACGAACATATTCCGCGTGGCCGCGGGGAGCGTGGCGGTGATGCCGTCCAGGTCCCTCGTCAGGCGCACCTGGCATCCCAGGCGGGAGGTGTCCGTGAGCCCGAATGCCATATCGAGCATATCGTTCTCGTCATCCTGGTACACGCAGCGGGTTGGGTAAGAGAAGCGGGGAAGGGGAGATGAAATGACAGGCGCACCTCAGGCTCCGGGAGCTTGTCGTATGAATCCTGGTCGAGGATGACATGACATGTCGAGCACGCTACCGAGCCTTCGCACGCGCCTGGCCGCGAATGAGCGGAGAGAACGCGTGAAAAGGGGGGGGAGAGGGAGCGCACCTTCGAGATCAATGTCATACTCATGGGCGATGGAGAGGATGTCGTCGCCGTCGTTGGCCTCGACGGTCTTGATGAGGTTTCCCTTCGAGTCCTTGAAGTGGACCTTTATTCTGG

>AT4706

TTGGGCGACGTAAGTATGATATCACATTGGGCGGCACCTTCATGGCGACGTCAAACTACATGGTCAAGTACATAATCTTGAAGGGCACGAATGCATCTAACGACAGCAAGTGCTCAAAGGCGTGCATTGGTTGCGAGCCGCGGTCCACCGCTGGAGTCTTTCTCACAGGACGAAAGCAAGATGTCATAAATGGCTTGAGCCACACCTTCGGGGTTCTCCTGGATCGCC

>AT4707

ATTTCTGGCCGCTCGCTGAATACCACATGAACCGGGTAACGGGCGCACGTTGTGCTCGCCACTCTTCCCGCGACTATGTGGTCATTAGCGTAGTAAAACCCCACTTCCTGTGTCTTACGACACTTGGTTGTGACTTTGGACTTTCTTGTAGAGTGGTCAGCGACTTGCACAAAACCGTGCTCCTATATGTATGTGGTGAATATCGTCGATTTCGCAAAAAAGAGAGCATACTCACGACATAGAGATCAAAGACTCGAGAATCCCAGATTTTCCAGGGATACCGCTGAAGCATCCACTCTCGAAGTGTTGCGTAGTCGTCCCAGACTTCCTGGCGTGCGAGCACTGAAGAGCTGTTTGTGTCCCCTTTCTTGAGGTCTCGCTCGGTGACCGGGGGAGGTAACATGACCGGCTCCACCAGTATGAGCGCACGAAGGGGGGGCGGGCTGTTGTCGGCAC

>AT4715

GCCCCGCCAGCCCTCGGGTTCAAAGCTATCGATGAGGACGACACCTCTGACCTGGCGCGAATCACTTCCAACGCCGATTCGAACGGATATTGAGAAAGGTTTGAGCGGAGGTCATGGTGCTATCTCAAGCGCTGCGAAACGAGGAGAGTGCATTCTAAAGGTCTGGAAATTATAGGTTCACGGTGTACAGTAGTACAGGATGATTGGGGATATCGCATGGGAATAGTCGACAAACGAAGGTGATCCACACGGAAATCATGAAACACGAGACGAGGACGCAGGAAAAAGCTCATGTCCGTCGACGTGAACCTGCTGCGGCAAACATGGAGTTCCTTTCCTCCTCAGGAGCAGACTCAACGAGCGCGCGGAAGTGACTCTTCTCGTCGCGCAGCAGATCGCTCAAGTTTCCGAACTCGACCTGTAAGCAAGACGTGGG

>AT4719

TGGCCATGTTCAGAGAGAATCCGATGTTGGACGCGTTGAGCTTGGTGAAGTAGGTCAAGTATATCGCAAGGGACACAGTGAAGATCGTTCCGACGAGGTCCGCACGAACGATGACCCAGCTACGGGCTCGGTTAAACACAGAAGAGGGCGAAATGTCGAGACATTCTGCGTACCGGTTTAGATTGTAGAACGTGTTAGATGCCCGAGTCCAGCGATCAATTCGATCATGCATATCATTCTTGAATGACTCCTCCGCCCCATACGCACGGATTGAGA

>AT4720

ACCGAGGCTAGCTACTGTAGAGCTGAGATGACCAATGAGAGGCGCACGTGTCTTGCTCTGTTCCCGCTTGACTGGCAGCTGAGCCTTCATGTACGCCCTCGCATACAAACCGCTGATGATGCCGAACACAGCAACGACGGCGGTGAAGATCGGAGCCACAATCACGACAAGGAAAAGTCGCACAAGCATCGACGTCGACAACTCCAGCAGCTTCAGGAGGAAGCTAGAAATCTGTCCATCGACTGGAAGGCGG

>AT4725

GTAGCTGGTTTCAACCAAGTGTCGAACGCTGTTGTACTCCGCCAGCGGCGGGAGGTCATCGAATGGCAAATGACGCAAGTGCTGAGCCCTCCAGATGAGCGATTCCAGCCAGCCATAGGTCACAAAGGAGAAGATGGAGGCCTTCTGCTCTGGACGTGCATCGGGGTCGTCGGTTCGACGTGGCGAGATGAGGGGAAGAACGATAGCGGCTAGTGTAAGAAGCCCGATCTTGACCCACAGGAGGTTCCCTTCTGCACCATCGATGGGTGTCAGATCGTACGTAGCGAGCGGCCAGATATCGCGGTACGCATACACTGCCCAGGCGGCCAACAAGACAATATTCCGAACGGCCTTGAGTTGGTTTCTCCAACGCGGAGGCAGGCACGTCAGTGCGAGAACGAAGGCCTAACGGAACGTATTCAGTGCCTCGGTGATTGGTTGGGAAATATACCACTCACGAGAATTGAGACGCGTGCTCCGTAGAAGAGCCACGAGTAAACTCGATCCTTGCGGCTTGGAGACTGTGGCGAATCGTTCGGTGTTGTAATGGCAGCCAGGGCAAGAAGAACAGAGACAGTGGCGGACAGTGCTAGATCATAGATATTGAACATTCCTTCCCGTTTGCTGGGTGGTCGATGGAGCGCCTGTTCGAAGTGTTGAATTGAACGCTGTTGTGGAAGTCCACCGCCGGAGCCTCCTGGATAACGCATGAGGGTAAGACGTATGCAGGTATGCTGAGACAACAAAGTGGATAATCTAGCGAAGTGGCGTGGATGGGAGAATGAAAATGGGGAAGTGGGTTGAGGACGGTGACACCTACCTGACCGTGGTATATATGGAAAAATGGGTATGCTAAGGAATGATACTAGGGTGCCGCCAAAGCGATGAAGGTCCGGGCGCTGTTCCAGAACAAAAAATGCTTATGTCGGTGTGAGAATCAAAAGGAAGGAAGGAGACAACGCTTGGACCTTTTTGGTTTTCCTGTGCGACAGGAAGGAGAGAAAGCGAGTCGGAGTGTGTGATGGTGTCGCAGCGCGCGGAGTCACTATTTAGGCGATCGTTGGTAGTGGCAGTACTCCACGCTGACCAGATCCAGATGTTTAGTTGAGCAATAGTAGTTACAGAGCGGGATATCGAAGAGACGAGGATATCGCGATGTCGTTAAGCCGGATATCCACTTGGCGTCCTGTCTGTCTTTGAGGGCGAGGCAGAGGAGGGGCGTTGTGGGCGAAACGAACATGGGCTCCTGCTGGTGGCACAATACAACGTGCCATAAGCATCC

>AT4743

ACTCTGGTTTTACTCATTGACCATCATTGGGTAGGCGAACACAATTGAGTATGCTGTACCGCTTCCCACTACAGCGCTACTTCGATCTCCGGGAACCACTCGTAGACTTCCAGTGCAATATGCCGCCTCCGAATCGCCACGAGGACAACATTCTTGAGTGGGATCGTTGCAGGGCCGTGCACTAGAGGAAACCGTTCCCGGCGGTCTTCTGACATGCTC

>AT4748

ACATTCAGTGGCCTTATAGTAACCGGCTGCAGCGAGCGCACCTTCTTCGGGGCTTGGCATGTTATCCGTGAGTCGTACGCGAAAAACTTGAAGCAATGGTAGATGTGTTTGACAGCACGTTCGCTCCTGGGCCCGTCTCTTATATGCAGCGGGGGAATTTGGGGGGTCGCTAGATGGCGGTACGCCGATCGCCTCTTATTCGCAATCTGGCGTTCGTTGTCACGGATGGCCAACAATATTCAGCTCCTTGAGTGCAGACTTTAAGGTTATTCAGGTACTATTGGAGTAGGTACTCACAAAGCTACGTCCCAAACCACTCATATATTCATTGAGAACTAGGTGATTTGTTGTAAACCAAAGGCCATTGAAGGAGTGGATCGTGTCCTTCGCCGGTGTAAGTTGGTGAAGGCTGTTTGCGCATGAACTCAACCAAATCGTCGTGCCTTGACTCGAGCACGCGGCCACAGCCA

>AT4765

GTTTGAAGCCAGCACGGCTACGATGCAAGACGGCCATGAGCGCGCTCGTAAACATCAGATCTGCCAGCAGTGGTAATGCCAATGCCGATACAACTAGATCCTGTGCAAATACCGCTCTAGAACCACGTTCGGCTGTCGAACAAAGGTAAAGCACAAACGCGCAGGATCTGTGATAAGTTCCACACTTTGCCGACCTCTCTGAGACTGTGAGGATCCGTGAGATCTCGACTGAGTGCTAGAGCTTCAGGGTAACCCACGATAAGTATGCTGCGGCTGGAAGGGAAGGATTCATAACAGCATCAGAGGATATATGAGATGGAAGACGACGCACCGATGCATAATACTAGATGCCGATGTAAAATGTGTGATCAAAATCGGGGTACGGCATGTAGAGAACGCACCAATCTCTCCTATGAGCAACAGAACCTGATGCAAGGCATTCAGATGCCTCTTCGGTGAGCATTCAGAGGAAGACTTACAGCGACCCCGACGAGGCCTCGATAGTAACCTCCCACTGCCACTCCCTATTAATGGTTATTGAGCATCATCCAAGTCCAAGACTTACGCAGAAATACGCGGCGAGCAAAGAAGCTGGTTGCTAAGGCGTTAGAGTTGACACCTCGGCATTCAGCGACGAAGCAACTCGCATTTGGGAAACTAGCACTATAGC

>AT4766

TCAGTCCGAAGAATGTCTGAAGAAGGTCATTCCCCAATGCTGTTCTGGCCGTAGGTGTGATAGAGGTGGTATTCGAAGGGGCGGGGTCCATGGCCTCCGCAGATGAGAGCGATGGGACGTTCGCGATTTCGTGACGGGGATTGGGAGACGACGGACTCCGAGACCGTGCATGTCTATATACTCGGGGACCGTGGCAAAATGGCCGCGCAGAAACCTTGTCCGGCATTTCTATCAGTCTCGGAAGCTGTACCACAAACCTTGAGATTGAGTGGCAGCGTGGCAACGGTAGCAAGCCTGGGAGACGCGGCCATAAGAACGGCGTAAAACAAGACAGTACGCGATGAACCACTGCCCATTGCCTCACCATGATGTGCGGATCGACCTCACGTCCGAGGAGCATCACTTGGGACACCTCGTGGTTCAGAAGCGCTTGTGGGAAGGAGGCCTACTGGAAGCGTCGAAGTGAGTTGCCGGCATGAGAACAGACATGACGTTGCTCCCAGGCTACACGCCACTTCAGGCTGAGGACCCGGTCATGTAGGGTCGTGTTTGGGGAAATGCC

>AT4767

AATGTATGTATTTGAGAGCCAGTATTGGCACTAGTATGAAGTGTATGTGTATGTTGAGCTTGAATGAGACAGACTTCCCGACGTGTCGCACTTATAATCTATGTAAGGTAGACAGACAAAATCACGCTACGCGCCCTGTTGGAAGCAGAAGTTAAACATGGGGTAAGGACAGATCTATAGAAGATGCTATGGTAAATGTAGATCCCCGGGCAAGTATGCATCCTCCTATAGTCCATAATAGACGAAAACGAAATAGTCACCCTGGGTCGGCATCGGTTTTGAATCAAGGTCTACATCTCCAGTCAAATGTCATCACTGCTGAGTGCGTTGTACGTCGGACAGAGGTTCTTCCAGTCATCGCTCGTGGTGAAGGAGGACGTCGTGTTAGTGTCTACTGCGAACACGACCGCGAACCCGGCCTCC

>AT4776

ATTCAGGTCAAATGGATACTGCGCCCTGGCACAACTACAATATGAGCCGAACTATAGGCCTGCATGCTCGAGACCAATGGCATGAACCGGGAACCCTAAGGTTCCTAGATCCAGGGCTGAAACGCTGCAGCTGACAGCGTTGAGAGGATCGCCGTCGGTGCAAATCTGTCTAGGTCATCACAGGGAGTCAATACTGGCGTCAGGTCCCACAATTTCCTTCAACTTCTCTGCTGCGCGGCGGCGGAACTCCGCAACGTGACGGACACGTTTGTCTCCATCCTCATTCTTGAACTTCTCCAAACAAAAGATCCGCAGAGCATCCGCCTAAGCATAACACATCAGTTCATACTGACGTGCAGGAGATGACAAGTTCATCACCGTGTCTGGGAAGCCGCCGTACGCTTCGACCAGTGCGTCGTTGAGTCCCTTCTCTGACGCGAAATTCGGTTTCACGAGTGCACTGGTGAGCGCATCGATTGCGTCTTCGTTGCGTGACAAGAGCAACAGAGCTTTCGCCTTGC

>AT4778

GAAAGACGGGCTGGCTTCCTGGAAACTGGTTGTCGTCCATCCGCCCCGTGAGAGGGTCTCCGAAACCCATTTGTATGAGCTGAACGACCCAAAACGATCCGCGCTTGACGCGAAGCGAGGCGTAGTCAAAGGTGGAGAGATCGACTTGGTTTTTCTCCTTTGACTCGGATACCGCGTTCATCACCGCCTCCAACATGGCAGGATCCCGGCCGGAAACATGTCGCATGATGTCGCTCAAGTCGCTGCCTCCTTGGTATCCTTGGAGGATTTGCTGCGTAGTGTGGGGA

>AT4790

GGGGCGTGATGCGGCGGCGCCTGACAACGACATGGATGACCATTGATGATCCAGGATGTCATAAGATTTCCTAAGCCTATCATACAATACTTCCCATGATAATCCTATACATTGCAATCTGCTTCCCATGGCTTCTTTCGAACGCTGGCCTTGGATTCGCTTGGAGTTTCGGACTGCCCGGTGTCGGTGCAAACAGGTCATATGTGAGCGCATGCCAGAATGACGGAAGGGTAAACTTACGCTCAGGACGTTAGGTATGAGTTTGTTATTCGGGATTTCGTCTGTTTTGCCCTGCTCAATGAGCTCCTTTAGCTGCGCAAACGTGAGGGTGGTCTGCTCCGGGTCCCTCGCTTGCGGTTGAGGTGCAGGCGCTGCGGGAGCCGGAGCGAAACCAATCCCCAATTTACAACCATTGCGGAAAGCACGTGCTGCGTCCAGGTCGAGTGATGTCCCTGATAGTAATACGCTGGAGGCTTTTTGAGTACATGCGAGGATAAGGAGGAAGCAATACGTAAAGGACTGATTTGCCACGAGCATCGGGAATAAAG

>AT4791

GGACCTGACTAAACATCCTCCTTGGTATCCAGCGAGCGGTTAAGGGTAACTTTGATTCTTCACAAATGGTCTGTGCGTACTTACGCAAAGCGCACTGCGCTGGCGGGATCGCGGTGAACTCGGCGGCGTGGAATGTCTGACGGCATGTCTCTCCACCCAATGAACTTCCATTCGCCACCTCCACCATAATTCGTAGGGAACTCCGTTGCTTGATCTCCAAGAAGACGGAGTGACTCACCGGTTGAAGTAGAACACTTCGGATCGAAGCATGAGCTCCTCTCGCTCTTCTTCTGTCTTGCCCTCCAGTGCACCGCTGTTCACGATGCCAGCGAGACCTTGCTTGCAAGTGTACGGTCGTATGAGCTGCTGCGCTCCGTGCGGACCTAGAAAGGATCAAGCTGCACCTGGTATTCGGCATCGTCCTCGAAGCGGTAGTTCGCGAATCTTGCCAGCGGATCACTAGAAGGACTCGGTGAAGAGTTCTGTGCATCCATAATTCAAAGCGGGGCGGGGCGCTGTAACGAGGCAAGGTGATAATTAAAGGCAGGGTCGTTGCCGGCGGCAAGGACGGGGACGATGGTGACGAGAGTCGGGACCAAGGTGTAAACGCCAAACGCCTCTTTGCGGGCAGGTAGGGGCGGGTGTAGGGCGGTGTTAGCGCTCCGCTGTCGGTTAGCCGAGCGAGTCCCTTCGACTCCCACCGTCGGTCAGACTGACACACGACACAGAATCCCCACTTCCTCCGGCCACCATCAGCCCATGGGAACGCAAAGGCGAAAAGGGTCTTGGTCATTTTAATTCGCCGGGGCCCATGGCGCAATTTACCGTCAGAACCAGCCCTGTTTTCTCCCATTGGGCTGACCCCAGTGGCATCAGCAGGCCTTGCTTCTCCTCGGACTGAGCACTTTGTGTGGGACTGGTTCCGCGAGCCGGACGCGCTGATCAGCAAAGGACCCCGTTCTATCGCGAAAGGGAT

>AT4799

TCAGCGGCAGCGAGGATGCGCATGGCCAATGCCAGCGCGTCGCCTGGTATCCCGAAACGCTCCAGCACCCGTTCTGCGAAGAGCGGGCCAAAACGCTGTCGGACGGTAGGCGAATTCGAGAGGCGGAGGAGTCGGGACAGGAAGAAGAGGAAGGGGTCTCCGCCGAGATAGTACATGGTCCCGTTATCGTACGCGCGGTTGTCGAGGGTGTTGTAGACGAACTCGAGCGTCTTGGCGAGCTCTTCTCCGCGGCCGTTGGCGT

>AT4801

AGTTGTGCGAAAGTGTCTTGTATGGTGACTTCCGTATCCGTAACGGACCACATCGGCTTCGGCTGGGCCCGTAGCCACTTGGCAGCATCAGCCACCGGATCACGAAAGAGGCATCGAAGGGATCGAGAGAGCTCCTCGAGGCCGGTGAAGACGAAGCCTTTCATCCCGAAGGACATCGCGGTCGAGACGTTTGCGACCTTGTCGTCTACGAAAGCCGTCCTCCTAGGGTCGATACGAGCCGAGTCGATAACGTGCTGGTAGAAGCTAAGGTCCGGTTTGCGTTCGCCGAATGAGGCTGAGATGAATATATCGTCGAAGAGGGACCAATCCTGCGCCATTGCTTTCGTCTTGAGCACATCCCAGTGTGGAAACGGAATGTTCGACATGGCGAAGAGGTGCACACCGGTGTTCGTCTTGAGATCCTGAAAAAATGAGAACATGCCACGGTCAGCGCGTAGGGACGCGCGGCAAGTGTGGATAGTAGTGGAGATGTCGGACGGGGCCAGGGAGTACTCAGAGGCGAGGCGCGCATAGCACTCCTCTCCCTCTATTTCGCCTCGCTCGTACTCGTACCAAGCGGCAGAGTTCAGCATTCGGCGAAGTGTATCTTTGGGGATGGGGAGATGGGGGTGTGAGCTGTGCAAATCCGACGTGAAAAGCACATCACCAAGGTCCAAGATGATGTTGTCACATTGCC

>AT4842

GCTGTGCTCCTTCTGAATGACCTCCAAAATGGCCTACGTACCTCTTCCCCACCCCGGCCAGAGCTTCTCGCGCGATGTCTAGACATTGGTGTGAGATACTGTAGCTTGGGTTCAAGCCACGCAACGCAGGTAGATCAAATTGCCACTTACCATGGTATAAAGCACGTCCGGGTCAGCTGAATGTCTTGACTGCTGCCAGTGAATCAGGCTCGAGTCAACGTGGCGGCTCGTAAAAGGCGACCGAAGCGTGAATGTACTGTCACGGAGATCGATGAAACACGGCTAGCCTGAAGAAACTGAGAGGCGGGTTGTGAAAGCCAGTGAGCAGGAGAATAGCGGGGAGGGCGTCGTGACTGTATGGACCTGGT

>AT4872

GAGCAAATAAAGGCGTCTTCTGCCTCCTCGGAGTGCTCAGACCCATTGCCCTCCTAGCAACAGCGTTATGGCGCGGTGTACCCGCGATGGAGGGCGTTCCTTCGAGGAAGAGAGGGCCCGGCGCGGGGGCACCAGGCATCGATTGCTCAACGCTGTCCTCGTCGAGCTGCATTTCGACATCGTCGAGGTCGGAGGTAGGTATATCGCGCGGCGGAGACGACATAGTGCGAGAGAGCGGGATGGGGATGAGGAGAACGGGGACGAGGACGAGGAGGATGGGGAAGGGGAACAAGACGATGGAC

>AT4874

ACCTGCGGCTCGATTGACGACGGTCGTGTCCCCGACGAGCTGTACAAAGAGCTCTATGCCTTGGAGGAGGGACAGTCCTGGAGGTAACAGCGAGGTAAGCGACATGCTGACGAGTGCCGTGGTCCAAGAGCATGCTGCTGTATTGCAGGCAACTTTATACTACGAGCCGCAAGCTTTAGCAATGGGCCCGCGAGACGCGGCCACGACCCTATCTTCATCGAATGTTGAGCGTTGAACTATATGCCACGGTGGCTTGCATCATATCCGTGATTCGCGGTCAGGTACGTAGACTCCTTGAATAGCTCTTGAGTTCCTTTTCTATGAGGTCTCGGGCTTGCCGAGGAGCGGCTGCGGACGAAATAACCACCGGGTGTGGGAAGGGCCGAGCCCTTTTTGCCCTCCAAGAGAATTAAGAAGCGCGGCGTCGACGATCGTGTGATGGATGCGGCCTCGAGACTGTGCTAGGGTCTTCCGCCGCACTGTTCATCTACGGCGTGAAGGAAACGAACCTGCAAATCGAGAGGGAGCGGTGCACACCTCGGCATCCTATCCGACATTGCGGCCCCTTCTCCATCTCCGCCGATTGACAACATGGGTTCTTAACGGATCCCGAAATCCCGCAACCTAGAGATACGTTTCAGATTGGGCAGCCAAGAACACAACGCCTCCCCTAGCAATGCCCAAAATGCCATATATACGAGGATACAATATCCAAACACAGAACAAAGGCGAACTGACAGATGGACTCCGAAGCCCGCTTGATGCGAAATCTAAATGTCCAAGGCCACTGCAGAAGGGCGGGAAGCGCTCGACGTGTCGGACCGTGCTCGGGATGAGTCTGCTTTCGCAATGTCCCCGAGTTCAAAGTGCATCGTGGTCTGATCGGTCGGGAAGTGCTCTCGCTGTCCGCCGATGACGCCGCGTAAATTCAGAGCAAGGCGTGCCGCCTAA

>AT4887

TTTTGTCCGCCACTTGCTTCTGGAGGTCATCAGAAAGCCGTTGAGGGTCGTTTAGGACGATAGCCGCTTCTGGGGTCGCGCGGCCTTTGCCCTTATCAACACTTGCAACGGCGTTCGCAGTCTGTGGTGTGGACTGAGCGCATACAGTGACAAAGAGATCGCTCCGCGCACCGTGGATGGGGCCGCGGGTGGGGGAGTGAGTTGGTCCCTATCCTCCAGTGGCGGCTCCGCCCTAGGTTCGAGCTCAGGCATCGATGAGGGTCCTGCTGACGGCTGACGTGTGTTTTCCCGCATCGGCTTGCCTGGAGAGGCCACGGTGGGACTATCGAGACAAAAAGTGGTCGTAGAACGAGAACGAACGCGAGATAGCGTGCGCTTTGTTGCGATGGTTGGGGACTCGGGATCGGAGTCAGGAGCAGACGCAACCACAGACACTTTGCGCCTCTTCTTGCGCCTTTTCTTCTTCTCTCTGGTGTCTCGACCTCCCTGATCTTCTGCAACGTCCCGTGGCCTCACTCGCTTCGGGTCGCGTTCTTCTGCGTCTTCTGAATCTGACAAGATTCTCCTCTTCGCATTCCGGCCTCTAGAAAGGGACGTGACAGGCTCTAGTACGCGGACAGGTGAAGACATGCAGGCCAGTCCAGAATAGCGGATTAAACCGTGAGAGGAAGACGCGTCCCTGAGAGGCAGGGAGTAGGTTGTGGCGGTCGTGGTAGACAAGCACGAGCAGAGCACTGAGCAGACCGTGCC

>AT4897

CGCCAGCAGGGACCGTGAACGAGTTGATGACCACGGTCGTACACTTGACGGCATCATCGACGTCGTCCAAGGACGCGATGGTTCCAGTGCAGTCGCTGGCAGCGGGCGCAGCGACGACTGCGCCGGCCAGCGCGAGGAGTGCAGCGAGGCGAAGGAAGGGGGACATCTTGCTGTCGTAGAGGGATTGAACTTGAGAGTACGAGGACCTGGGCACGCTGAAGGTAGAGCACGAGGTTAACACAGGTTGAGACCGTCCTTATATACAGTCGACTCGGAGAAATTCGCGGCGCAAAATCGTGGACAACGCAGGCGAGAAGAGGCCCGCTAGCCGTTGGTCCGTGGGCTCTTCACATCCAGCGGGTCTCATGAGCCTTCTCGTACGAGCAGCACATGTAGTCACGGTGGGAAGAGGGCGGAAAAAAAGAGAAGCACAGCATCATGTACGTCTTCCGCAGAAACGAATAACGTTCTACTCGGGTGCATCTAGCAGCGTTGTGCAGCTTCAATGAGAGGTGCGGTAAATTGGCTCGGAAATTACAAGCTCAAGTGCCCCATGATCGAAGAATAAAATAACAAGATGACCACTTAC

>AT4900

AAACGACAGTGATGCGGGATCGTATGTGTCTGCAGGAATGTTTTAAGGAGACTGCAAGAGGGATGGACCGAGTACGCACGGTCCGTTGGAGAACTTGGGCCCCGAGGATGGCGCCTGAGACGATCCTGCGCCACCATTTGCCTGCTCTGTGGCCTGTGCGTTGGAGGCGTGAACGGTGGAAGAAGAGGGAGGGGCGGGAGAGGGAAAGTGCATCGCGCCCGCTGGCGTGTGTGAGGACTCCATGTCGAACCCGGGTGTGCCCTCCATCGGACGGCTTGTTTCGGTGAGCGAGCGGCCTCTGGGGCGTCGATGTCGCGGCGGGAGAGACGGGATGAGGAAAGACGGGGCGGCAGCCTAGAAAGCAGCGGCGCGGTGGAGGGAGGTGAAGGAAAGAGAGCAACCAAGGACGAGAGGCTTGCGCGGGAAATTCGCCCGCGACCGGAGTAGCAGCCGATCCCTGCCCAAGATCCCTCCCGCGGGCGGCCCCC

>AT4937

GCTTGGTAAGTTGGTTCGTTTCCGCAGCCGTAGAGGCTCTGAGCGACCCAGCGAAGACAACCACTACCAATACACTTCTGATGTGGGAGGCAGAAAAGCATTGAAATGCGGTGGTATGTATTGCCAACGAACGTCGAAAAATGCTACAAGGAGGTCCAAAGCCCAAGTCGAGTTGATACATATTTTAACTATAAGCAAACAACCACGAAAGCGAAAACACCCAGCAACAACCAAGTAGTAACAGCAAGTACGAAATATGCATGGGGCAGATGTGAGCTGCCACAGGGGATATATGCTTAGCGCGAGCGGGAGAGTTCATCGATGTAAACCTCGAGGAGCAGCAGCTAAGGAGCGTGAGGGTGAGCGCAGGCCCCAGGCCCGCGCAGGATAGAATGTCTATCGCGGGGCAGTCTAGAGGGCGAGCGCGACAGCGCCGACGGCTACGAGGGCCGCGAGGGCGCTGACACCGTGCGACGCTGCGCCGTTGCTGCCGCTCGCAGTCGTTGCGGTTGATGAGGACGCAGCAGAGGTCGATGAGCTGGAGCCTGAGGGGGAGGTCGCGGTCGCAGAGCTTGCGGCGGAGCCAGTCTGAGTGGCTGAGCCGGAGGAGCCAGAAGCAGTCGAGGTCGCAGTGAGGGTGACGCCCTGCATGGAAGTTGTCAGTCAAATTCGCTCACGTCTTCGGGACGAACGAAAAAATATTGGCAGAGGATGCGCAGGAAGGGCGTAGAAAACGGCAGTGCGATGGAGGAATCGGGGATCGGGAGCAGGAAGCATGACGAGAAGGAAGGGGTGACAGGGAAGCGAGGGGAACAATGGGGAAAATAGACGTACAACAGCGAGACAGAGCTGCTGCGCGGCAGCCTCGGCATTGGTCAGGTCAGAGCCTGTACATGCGCCGGTGATGCAGCTGGTGACGGAGGAGATGAAGCCGGAGTCGTGGCAGAGGCAAGAGTCGTCGAGGGGGTCGCAGCTGCCGAAATTAGCGTTGGTGAGGCAGGTTTCGGAGCAGCCTATGAGAACAAAGACAGTCAGTTCGATGACGATGGTTGAAAAATAGTCAAAAACATACTGGGGAAGGACTGCCTGCGCTGGAGGGACGCAGCAGAGACGGCGGCGGCGAGAGTGAGAACGGCGAAGAAACGCATTGTGTGTGCGAGTGTGGAGATGAGAGTGGGTATGAAGTGGGGTGAGGTGTGGGCACAAAGGGTGCAGTAAGGGGAGAGGTGACGCGGAGGGACGCTAACTCAAAGGAGGAGTTGCTAAGGACGACGGGACGTCTGGAAGAGGGCTTTATACGCCCGGTGAATAAAGGGGAGAGCGCGGGTAGGCGCGGGTTCGGTTTCCCAACACATCAGTGGGGAGGGTCATTGTGGGACACATGGTCAACAAACAAACGGTCGCTTTCGGTGGCTCTCGCTTGTCAATGGGCGGGATTTTCCACTGTTGCGCGCGCACGCGAGGCGAGCTTCTGGCGTTCCTGATCTCTTGGTTCCTGACTGATGTCAGATTGTTTGTCAGACTATTCAGAGGATGTTCCCTGCTGGTTCGGAGGACAATATGAGAGCCTTGGGGAGTACAAAGAGCGACAAGGACGACGAACTGTCGGTGAGAGGAAGAAAGGGAGTCCCATTGTGGGACCAGCCAAACACCGC

>AT4941

CTATCATCGGAGTCTGCATGCGGTCGGATGTGTTTGCGAACCGCTTGTCGAGATACTCGAATATCGCATCTGGGCTTCCGAGCACCACGATAGACTGCCCGAGAATCTGGAGATACATGACGTCTCCTGCAGCGATTTCGATTGACTTTATCCGGGTCAAAAGTCCTGGGGGGTTGGCTCACCATGTTGACGGCTAAGGTCACAATAACCGATCCATTGTCTGGACCGAGGCATGTTGAACATATTGCCGACTATCGGGAGGCGGCTCGGACCTGGGGGAAGGGGACGCCCGCGAGTGCGAGCTCGTCATCGCGCGATAGATACGAGATAGAGGACGACTAGTAGACCGACAATCACCTGTACCAGGGCAAGCAAGCCTAGGCGACCTGCGTTCATGGTCACGCCTGAGAGGTACATTGCAGGTGACGGGATAGAGGTTTTTGATCCAAACAAGGCGATGGACGGCAGGGACAGAGGATGGGCAATTGTCCGGATCCTGAGGGAGGGGGCATCTTATGCTAAGTATCGGCATAATACGCATGCGGAAGACAAAACCCCACCCGATTCGCTGTGATCAGGCCAAGGAGCTCCCACTCCATCCGGTGTTCGTGGTCGGAGTTGGGAGCGCACCCGGTAATTCCGCATCCGTGACGACATCCGCTTCCCGTCTTGGCTCGTCTATGAAATCGGGAAGTCAGTGGGA

>AT4947

AGGATGTACGCACACGATCTCATAGACGCGATCTGCATCATCGTCTCGGCTATACCAACCAGACTGGAATCGCGATAAACCTCCGCTCGACGGTATGAAGGCATATGAACCGGGTGGCGAGCATCCGCAACCACAACATACAGGACGAGGGACTCACCATTCCGAGGTTATGCCTCTGATCGGCGTTCAATAGCGTCTGGTACTCCCTGTGGGTCGGAAGCTTCTCCAAGACGATCGTGGCGGCCTTGGCGAATTCTACGAATGCACCTTCGTAGTCTTTAGATTGAAGAAGGGTTTCTGCGGTGCGTCTAGACTTCTCGGCGGTGCGTAGCCAGTGCTTCAGGCCCTTGGAGGGATCCCACAGGTTCTCCTGCGCTTGCGCGGCGAGTTCGGCGATGGTTGCGGGACGGTAGTAGTTGGAGCGAGAAGAGCTGGGACCTGAGCCGGCAGTGGGGTGAGAAGGGTATATGGCTGAGCTCATGGTGGTCGTCGGAGGGTAGGAGAAGGGGCTGGAAGGAGCAGTAGCCAAGCAGCCTCTGTCTATCCCGGGCGACGGACGTACCGCCGCAGTCTCACCGAACGCCCGTCACGCTCACACACAC

>AT4963

TTCAAATGTAACAGCGCAGACATTCTTGATGATATCCTCATCTTCGGCCGAAATCGTGCCCGATTTGCCCATGCTTCCGAGCAATTTCGCGGCAATTGACTCGGTAGCTTCTCCGTGCTCCTTCAAAGGGATGGGATGAGGACGGTCAACACAACGCCGCGAAGATCGTAAATCAACGGACGACGGACCAGCTTGGCCTTGGTGTGCTCGAACGGGACGTTCCTGAGTCTGTCTCCCGCCGCCATCCACTTGGCCCACAGCCTTTGCGAAGTCGCACCGGGGAACCACGGGGGAATGTGCCGAAGAAACGGTAGATACTCGACGAGAAATTTCCCAGGCACCAAGCCTTCCGCCGCTCCGGCGATTGCTTCTTCCGATATCTGCACGTAGCCGTTGTGTTCGTCTTTGACGTCAATGCCGTACACTATCTTCAGTATCGCTGATGCAAAGTTTCTGTTTTCGCGTACCCTCAGCCTGAGCTTGAAACACCAATATTGAACGGAAAGGCGGTAGACTTACAACCGGATAAGCTGGTTCAAGCGCGAAGGACTCTCGAGCAGCATCTCGAGGAAGACATGAGTGACGGTTTGCTGTATTGGCCTGTACTGCTCAACGGCGTCCCGGTGGAAATATTGCCAGAAGGCGCGTCTATGACGGCGCCACCGCTGTCCATACGGCATGAGACCGAGGATCGAGCCATTCCCAGACCTGTACGGATAGCGCGTCACTCGTGAGTAGTTGACGTACTCCCAGAGAACTCACAGTTCTATCATTGGAGACTGAACGCGGTCCGATGTATTTGCGGATCGTTTGTCTAGATACTCAAATACCGCCTCAGCGCTTCCGAGCACCACGATGGACTTTCCAAACACTTGGAAGTAGAGGATATCCCCTTCGACAATGGGGGTCAGTGTTCAGGCCTTGGACGGAGCACGGCATTGCTTACCTAACTGGCGACTCAAGTCTCTAAACCCGACCCATTGCGTCAACCGAGGCATGTTGAACATGTTGCCGACGATTGGGAGGCGTTTCGGACCTGGGGGCAGAGGACGGCCACACGTGCGAGTCCGCCATCGCGTGGTGTACCAGAAATAAGGGGCGAAAAGGACGACGAGTTGTACTAGGACCAGCACGCTCGCCGGACTCGTCGATTGGATCATGGTGAAGAGGGATGTTTGACACGAGCAAGATGATGTGTGTGTCAGAGGGCTGGTGACCTGAAACGTAAGAGCTCGCTGGGACGCAGAGCTTTATACCCAGCCCAGGTGGTCCGGAAGTGCGTCTAGGATACAAGGTTAAACTTAACGCACCTAGCTCAGAACACACTGCTGACTCGCTGCGTGCCTGACATATAGTCCTCCGAGTCCCAATCGAGGGCCAAATGTTTCCCACTCCATCCGGCTTTTCCATCGGCATCCGAGAAGAAAAATACGTCCGGGTGGTTTCATAAGGGTTGCACCCGTCAGCATGCCATATCTGTCATCAAAGAATGAAGGAGAGAAGTATTGGGGCATAGTATGTTTATGGACTCGAAGGTCTGTGGGCGTTGTCTGAGCGCTTCGTGACGATCGCGAGCCGTAGTTTACGGCAGGCATTCCACATGTGGCGTCCTGTGTGAAAGCCAGTCGTAAGATAGCGGTAGGGGAGGGGGCCAGTTGACGCCGTCTGAACGTTGCTAGCTGGGGGTTACATGCTTGTGGAGGAGATGCGTCGGACGTCAAGCCAGGGCCCGGCAGTACTGCGCTTTGGGAATCTGCATATCATCGGGTCAAAGGAAAGCGAAGAAAGGCACCAGAAGGAAGAGGCTCACTGTTTCGACTTCACGTTGCAGTAGT

>AT4966

CGTGAAGATGATCGACTGGTCCTGCTGCGTCCTCCGCATGTGGTAGTAGGCATGGTTCGCGAGCTGGAAGATGTGGGGCGGAAGCCGGACCTTGTCGGGGGAGGTGTCGCGGTACTCGGCCGCGTACTTGTGGAGGACGGCGTCGGAATTAGAGGGAACATACTTGTGGGGGTTTACTGCGACGAGGGCAGAGGTTCCGAGGTTAGTGTAGATGTTGTCTATCATAAACCGCTCGCGGATGCATGAGACGATGATGTCCTCCGAGACTGGGGAGAGGGTCGCCAGGTCGGTGACGGACTCGAGCTGCTGGTGCAGCGTCGATTGTCGTTGGGTCATGATGGCGGACTACGGCACCGGGGTGCTCCCCGCTTCCTGATTGTCGAAAGAAAAGAAAGGAGGGAGGAGGACGAGAGGGAGAGGACAAGCGTCGTCGTTGGTATGGCAGTGGGAAAGCGTGGTGCTGGGC

>AT4971

ACATCCAACAGAGGAAGTTGAGCTCCATGCCGCTTCCATAGTCGATACGCGTGCGGTTACCCCATGACTCGGTGAAGTACACACTGAGCTCAGGGATTGCGGCGTCCGGAAGGCCAGGGATTCCTTTGTGTATCTCTGGAGCAAGCTAGATGCCAAAATATACATGCATATCAACGCTGGAGAATGATGGAGGAGGCAGGAGCATATAGGGACTAGACATACCTCCTGGACCTTGTCATAGAACGTTCGAAACGCAGGGTTTCCAAACCTCGAGGCCTTGTTATCCACAGGCGGTATGCCCTTGGCCGTCTCAGACACCCTCTCAAGGACGTTCAATACGGCTTGTACACTCTAATTCCCATCCAAACATGGATATGGGACCAAGTTAGCGAGGAAACGAGATGGTAGCGTTCAATGGGTAGACTTACGCACAGGCGAAAGTTGAATATCGTCTGTGAGCTTCACGCCAACGACGCGTTCGTTGAGTGTGATGATGTACGAGACGATGGCTTCATGCGTCTTGGACGCTTGGAAAGTCTCGAGGTTCTCCTTAGTCAAGATCAGCTTGCGCGGGTCGATGTATGCGTGGTCTTTCAACTCGGCAGGTTCTAGCGCGAAGTTAGTGATCGTAGGCGTGAATACTGGGTCAGCCATGATGGAGGATGTGTGTAGAGCGCGCCGCGGGATGGTGAATGAGGGAGAGCAAGTTGTGGCTCAGGTGGTCCGACAATCTC

>AT4987

CGCGGGGCATACGCCTCTAAGGTCGTGGAGTGCCTCGAAATGCATTGAGGTAGGGCGGGATACATTTGTGCCAAGGATGCTAGCACACTACAGCATAAGGATGGACTGCGGATGGGGCTGAGAGTGGTGGGCTGAGAGCGGCCGCGCCGGGAACCGGGCACTGTTCAGACCTTCAGGAACAGGTCTGGTATTTCCGTATAACAGGTAGACGCGGGCGGTACAATGAAAACGCTTCATTGCTCTCCATTGATAATAGTTTGCGCGCCTTGGGATTTGAGACGGAACCGAACATCGGAACGTAGTTCTACTTCGCGTTGGTAAGCTATTTCTGACCAATGTTGAATGTTCAAAACGGTCTCACAAGGATATCCCGTGCATGAGAGGTCACGGCCGTGAAGGAATCAGATCTTGCAGAATCCCTGTCACGTCCCCTCCTACCGACAGCGGCCACTGCTCGTCCGAAATTGAGGCGTCATGGGGCAGTGCGGACCAATTGGGAGACGGAGTAGCGCGAATATGCGAGAAGTCTTGCACGGCAGGATAACTTTCGCCGTCTGAAGCTTCCTACGAGAATGGAGGACAGTCAGCGGCGGCAATATGTTGCTAGTATGATGCTACATGTATGACACGAGCGACTATCATAACGATGGAGAAGACGTACAGGTTCGGCGGAATCTGCCTGTGTAGGCACTGACATTGATGTTGAACGTCGGAGCACTGCGGGATAAGCTGCGAAACGCATCGCTGCGCAGTGCTCGATGATGGGGTTCGAGGAGATCGTGATATAGACGTCCACGTTAGACGACGTATGTATGCGAAACTGT

>AT4988

CGGAAGACATCGCCATCGCAAAACGTATACAAAGGAACATACCTGCCGGCTTCCAAGTGCAATGACACAGTTCTTAATGTCATGCAAGAGCGCACTGCCCGTGATGATGGGGAGCACCAGGATCGTATTGCTCAGATTCCTGACATGGAGTGCCGTGAACGTCAGCCCCCGGGGGTAGTCCGGATTGGCATCGGAGGGTATGAGATTGACGACACAGTGGTCCAGGTCGGAGATTGCAAGGTCGGAGGCTGAACACCAGGGCGACGATAACGAGGCTAGCGAGATATACTTGTGCGAGTGACCAGAAATGGATAGCCCCGATGTGTGCGGCATGCTCGCAAGAGTAGCGCTCATTGGTGGCGCAGTAGACAGGAGTGGTGATGAGACGGGTGGGGAGGCTGTAGGTTTGGCTGCTTTCCGCTTGAACGCGAACTTCGCCTTGGGTGCGCTTGCAGTGCGAACACCTTCTAAGTCCGACTCTATCGACTTGATCTTCTGTACAGAGTCATCTCAGCTGAGTTTTGAGTATTTACGGAAGAAAAGCTCACAAGGTCGAGGTTGCGTTGATCATAACTGGGCAGGTATGGAACCGCATCGGTGAAGTCCTTCCTCAGCTTTGAGAGCTCGACCGTCGCTTGATCGATGGTCTCCGGAGCGGCGCCACCCTCCCTTAGCCCATGAAGCGTGGCAGAAAGGTCTGCCGAGGGCGTTGGTTTAGATCGAGAGCTTGGGTCGAGCAATGAGAGGATGCCGACCCTGCGCGGCAGCATCTAGCCGTCCATAGTATTGTTGCCCAAATTCCTGATTGGTAGGTTCGGCCTTCGTCTCAGACATTGTACAACAGGAGGTACAATGTGGTTAAGCGTGTGCGGTCGTCGATTGTAGACTGAAGGCCAAGAGGTCGAGGACAACGTAGGACAACGTCCGAGTCGCTGACAAGTGAGTAAGTACTA

>AT5014

ATGTGGTGATAAATGCATTGGATTTCTAACCATGATATCTAGAGCCTCGCTTTAGGGACGGTCGACAGCTTACCACTAGCTAGCATGGTTTTAGCCTGTTCCCGCAGAACACGTCGCAAGAGCTTGCCGCTGGGGTTCTTCGGAATGGCGTCAACAAACTCCACACCCGCAAGCCACTTGAAGTGGGTCTTGTGATCGGAGAC

>AT5015

TCTTTGACTTTCTTCTTTTCCTGGGGATTTCCTTGGACACGCGCCTGCGCATCGCGGTTAAGCACGATGAACGCGAAGGGAAGCTCTCCGCTATAATCGTCGGGAATACCGACAACGCAGACGTCCGCGACATCAGGATGGTCGAGAAGGTGGCCCTCTAGCTCCGAAGGTGCCACTTGGAACCCGCGCACTTTGATGAGCTCCTTGATGCGGTCGACGACGAAGACTTCCCCGTTTTCGTTGAGCTTGACTTCATCCCCAGTGTGGAGCCATCT

>AT5016

TACGCGGTCGGGTTGTCGTAGTATCCCAGGGAAAGGGCCGGGCTCCTCAGATGTAACTCCCCAGGTTCTCCAAACTGAGCGAGCGTCCCATCTACCTTGACGACTCGCGCTTCCACGCCCGGGATGAGGAGACCCGCGCATCCCGGAGTGCCAATGCGCTTGTCGAGTTGGCAGAACGTGATCATTGTCGACGTTTCGGTCATCCCTGAGTATTACAGTATCCAAAACCAGTAAATAGGAAGAGCGCAGGTGCCGGACAGGGAAGACGCACCATAGCCTTGCCCAACGAGGGCGTTCGGGAGGACCTTCGTCAGTTGGTCAGTGAGCTCGCCGCTGACAGGCGCAGCGCCGCACGCGACGAATTGGACAGAGCTGAGATCGTAGTCCTGCACGGCAGGGCTCTGC

>AT5046

GCAGGCTACTCGGTCGGCAACCTCCGGCGCCATGAAGTCCACGTTCCAGGACACGATCTTGATGCTGGAGACGGGAATAGACGGTTTGTCGTATTTGGTAGTCCAACGGGAGTATTCGGGGGAGAATGCATTGACAGGGAGGTCACGAACGGTACCGTAAACGCTCATGTCTTGGTCGGCGTCGTTGGACGTGTGGAGGTTACGTACTCCCAGGCGGCTGAACGCTGGGTAGTCAACGAGCTCGGGGTGTGGGCTATTTAGGGAGGTCGTATCAGTATCCTGCGCACAGTCAGAGATGCAGTGATAGCTGACTATGGTGTCTGCAGCTGACTTGCAACCCTGAGCGGGGAACACGCATAGTGCCTCGCCGAGCATCTAGTCCCTGCAGCCATTCCATGAAGACCAACGACTAGCCGCCTAGAAGGGTCCTGCGTAAGATGCGTTGCTTGAAAGTTTTGACAAGGAAACCTACCGCGTCGGCTTGATGCCGTAGGTTTTGGAGAAAAAAGCGATGCCTCCAAGGAGGATGGGTCGTAGGCTGCTCAAGGATGTCATCCTGTGGAAAAGTTGGCGCAGATGGGTTACAGGGGAGAACAAGTCGTGAGGGAATAGCAAGATGGCGAATCCGGCCGATATAGCCGCCCAAATGCTGGTCCGGAACTTGAAAGCCGCCTGCAGATGAAAGGTGAACTCCACGTCGAGCC

>AT5047

GCGTGGAAGAAGACAGCCCACGTGTATCTCATGCGCGAACCCAAACGATGCCGACATCGCGGCCTGACGGAGACTAGCCCCACCTATAGCTGGGTCTCCGCATCTTCTAGTCAACTCCCTGACGGCGACGTAGTTAGCGCACTCCAAAGCCCTCCAGAAGACACGGATATATGCGCGGTATGAATTCTTCGTGTTGCTTGATATGTATCGTATGCGATTGTACATCGTGAAATGACGGGTGCTGGCGGCGACGGTACGCTTCAGAAATCGAAGCTCTTGTCCAGGTCGGCCTTCAGGTGGTCGCGCATTCCAGGAGGTAGGATCGGCTGCTTTCCGTCTGCGATGTAGCCTAATGTCGTTATCAGTGCGTCACTGGGAACGACAAAATATTAGCGTCGACTCACGGAATGGCATCTCGACGCTCTCCTTCGCGTTAAAC

>AT5048

ACCCTGATCCTGCACCACAATCTTCGTAACCCCAAGTGCAGTGTCTGTTGTCATCTGTACAGGACCAAGATGTCTTCCGGGAAATCCCAGAGCGCGCAACTCCGACGTCCTGTCTCGCGGGGGGTCGTCACCTGTCGTCGAATCGTCGTCCTTTGAACCCCGCCGGTAGATTTGAGTCGATGGCATCCTCACACACCTAGAATACCCTATGCTCTTGCAATTAGTAAGCGTTGTCGTTATGAGCTCACAATGGACTTACTCCAAATAAGAACCACTCAAACCGGCCATCGCCGTCCTCGGGCGCGAGCTCTTTTTCTGCTCTCGGATCCAACAGACAGACCCTCTTCAACGGCACGCCCTTCTCCTCCATGAGCTCCAGTATGGACTTCTGGTGCGCATATGCGCCCGCATGGTCTTTGGAAGCGCTCCCCTCTTCTGCGAACAGCGAGGTGAGCGTGCTTGCAGAGCTTTCCGAGAGGTGGGTGAAGTGCACCGCGGCATCCGGGCCTGCGAGGGCGCGCA

>AT5399

GCGGTGCAGTTGCTTGTCTACGACTTCCTGCGAGACGGCGTTTTTCGGGCGGAGGGCGGACACTGTCATAGGAATGGCGAGCGAAGTGCCTTCGAAATGAACGTCTCCGCCGCCGCCAATCATGTCCAAGTTGACCATGCCGGTTATGGTCCGCTCCCCGATGAGATCCTTCATCCGCCTGTATATCTCCAGCTCGCTGGTGCGGTTGACTGTCCTCCAAGCGACTTTGAGGACCGCGGGAAGACCTTGGGCGGTAACCACCCGCCAGACGGACGTTCCCCGCCCGAATAGCGTGTGTGAGAGCCACAAGGGGTTCTCGCACGTAGTCCAGCTGTGATCGTCGTCCGGGCCGCCCATCTTGACAACGAGGGATGGATACGTCTTCTTGCCGAGTGATTTGTAGCCTTCAAGGTACGCGGTGTTGGGGTCCTGACCAAGATCGGACGGGCTTAGCTCCCAGGTCAAGCGTAAGACGATACGGATGAAGGTACGAAGTCCTTCACCCTCCAGGAGGGAGTAGTGCGGGGACAGCACGACCCCTACCCGGTCAGTCCAGCTGAGACAGAATTGGTGACCGCAAAGGATTATTCCGTACACGTAGAGCTGGAAAGGGAGACAGACGAGAAGGCTCCGTGCGTAGTCCGTCGCCTGACAAAGGGCAGTAGTCCCGCTGGGAACTTGGGTGGGGGAAGGCGAATCAAGGCTACAGCTCTTGACTTCCACGTACGAAACGAGGTGGCGCCACTCCATAGGTAGCTCCTTGGCCTTGTCGAGAGGCGGCAACAGAGGGCCGCCTGTGAGCGGTCTGGGATGCTCTACCACCGCAAAATCGGGCTTGTATTTGAACGCACTGGGGTCCGTCGGATCCTGCGAAATGAGAGGGGTGTGGCCAGTACAGATGAACTGTCGATAGTATTCCCTCTTTCTGCCGCCTAACCTGGCGGCGAGCTCTCTGTCTATGGTGTCAATAATCTTCGTCTATCGATCGTGACAGTTAGTGCTTGGGCTTGGTAGAGCTAGTACTAGGAACACGTACGAGTGGTGGATAGAGAGCTTCCTCTAGTTTCCTAGTAGCATCACTTTGGACGATCGACTCGGAAACACCCTGAACAGCATTGCCCTTCCTCGTATTGCGGGCCCTCTGTTCAATGGTTCGGATCTCGTCGCGCTCCGTAGAACACCTCGTCTCGACTACATGCCCAATAGCATCAACAATGGAGGTGTCTCCGGAGAAGTGCGTGGAGTTCAAGTTGTCAGAAAGAAGTTTGTGGAGAACCCCCTTCCGATCGTACAGGACGCGGTTCTGTGTGAGCTCCCTGCCGAAATGCTTGCGGA

>AT5400

GGGGGACAAAGCGCACGTAACGATCGACCAGAAACAAGGGGATGTGAAGTGAGGGCAATACCGGATGCCCAATAACCCCCCGAAAACAGTGTTACCGTACTATGGTAACCCAGAGAAGTCCGAAAGCGTAGAACATAGAAATCCAACTCGGGTATTAGCGCGTATGCGAGGCGTGAAAGTGTTCGTGAAGTCCGTGACGACGACAGTAGAAGAGAGCATCGAGAGCAAGGTTTGCCTGTAGACTATAGTGCCTAGGTCCATATCCTGAGGAGGAGAGCCGTGGCTACCGCGGAGTACGCTGCTGAGACATACCCGGATATATGTCTGTGAGGCCGCGCGCTGTTGGGGCTAGAGGCATCCTGTGTACTTCCCGATGACGACGACGTCGCGAGGGCATTCCCGCAGATGCCCGAGCCAACGAGCAGGGCGTTGGGGACGATGACGGCGGGCTTGCCGCCGCCGACGTAGCTGGCCTCTAGAGTGCTGCCGCTGCCGATCGCGTACTGAGTACACGACAGGATGCTACAGTGGACGACGCC

>AT5401

GTGGCGGCAAAGGGGATAGCCTCGACACGAAGACATACAGGAGAACCGACGTTCGTCAGCACGGTGATGACCGGTCCCTTGGTGAACATGATACTCTGGGACGTCGAACTAAGCACCTCTATCTGGGTGGTCACCCAGCTGGAGGAACTACTGATCATGAAGTTCCGGAACTGGTTGAGCTTCGCGATCATCTGGTAACTGGAGCCGTTGGCGTATCCTGACGGCCACAACGGCTCACGATTGAACTGTCGATGCCACTAAGTAACCATCGAGGCGGGAAAGGGTATCTGTGCTACTTACAGGATCCGTGTTGCCGTTGAAACCCTGTTCCTGCCCGTAGTATACAATGGGAATTCTGTGAATGGAGCTAAGATACAGCGTTCCCATCAAAATTGTCAAACTGAGCGCGCACCCATCGGCCATGAAACTGAAAGTCATGGCATTCCACATGGTTTGCGGATCGACGGAAAGGCTCGCCCAGCGCGGAACATCCTGGTCCTCGAGGAAGTTACCGAGGAGCG

>AT5413

CGTAGCCTGAATTTTACTGCACGTGGTTGGCGTTGGGGTTCTGAACGCTCAGGTGGCCATGGGTCTCCGTCCAGCTGGGCTGTTACCCGTCCGCATGTTGGTCTTCGATATGTGACTGAAAACGCTACAGCGATCCCACAGCTCATATGCACAAGGTATGCACAAGAAATTCATATAGTCGCGTCGAGCTAGGTCAAGTAATACAAACCATGGGATATGGACTGACACACCGATGTTCCAAAATGGCCACGCCACAGCCTGTTTTCGAGCACCGCAAGATCGTTCGCATACTTCAAGCACCGCCCAAGAGCCGTGTGAAGTTCGGTCGGAACGTCTCCCAGCACTCCTGGACCAGATC

>AT5427

CGACATATTGGCTTAAGAGGGATTCTTCAACTACTCCTACGCGTTTGATGAGGCCTCTATCCAAGAGAACGTCGCCCTTGACGACCTCCAGACCGTCTGCGCCGCCCGTCCAGATTGTCGCATTCTGAAAGTCAATTGAAAGGCATCAGCTACCAATGGAGATACAGACGAAATGATCACATACTGTGATCAGAGCTGCTGTTGTCCCGGGCACGTAGCGGTCGGAATGCGATCGAGTGTTGAAACCGGACGGAGGGCCCGGCTTCGCGCTAAGAAGCTGGCACTTATCCAGCCATTCAGCAGCGCGTAACGGGACTTGGACGCTCTCCGACGTCTTCAGAACCGCGGAATAGAGCCGCAAGTACTGGAGGGCAACCAGAGATAAGGCGACAGCCAAGAGAACTCGCAGACCCCGAGACAGCCTGCGGCGAGGCTGTGTTGCCTCGCGGGCGTACGACGGCAGCTTCGGCTCTTCGTCGTACATGAGCGTGCACTTCGTCGAAGTTGAAGGAATAGCGAAAGGACGGCGGACGATTCGAATCCTGGCAGGCCAGCTGGAATCAATATTTTCCCTCGCGATGGCGCAATGCCGAGCTAAACCGAAGCTACTGCTCCGGCGATTGCAGCAGATGGCGATGACCGAGCTTCCTATTTAATATTAGTGGTGAGAGTGAGGCTGA

>AT5428

TGGGATAAGGACGGAGACGAACACAGTTCGTCCAGGTAATCCAGCATATCCTCTGTGAGCGGCACTGAGATGCAGAGAGACAGGCTGACAAGAGATTTGGAGGATAGGACAGGGAGCACGGCGCGGAAGTCCGTGCCATCATTTATGTCTTCGGTGGAAAGCTCTTGAAGAGCGGGGAATGCGCCCGGGATGTTCATAATCGGCTTTGATGTATCGAAGCGTCCACATAGCGTCCGTAGGTTCGGCAGCGCGGCAAGATGGCGGAACAATGAAGTGTCGATGGTCGAAATATGTGCGGTATGGAGGTTGGTGAACTTTGCCAGGGCACGATGAATTTCTTGGTTGAAATTCCGGAGGAACGGGGCCTTTAGATGTATTAGCTCCTGGTGCGCGTCCGCTACAGTAGCCAGAATTTCGGGAGTGGCAAGTTCATACTCTGTAGTAATGGCAAACAGGACCAGTGTCGGAGAAGCGCATACGGCTTTCAGGAAGGCGAGATAAGGCGCCCCGTGAAAGCCCCACCCCAGGGAGAGCACACGGAGGTTTGGAAATATGGGGC

>AT5438

GTAATTGAACCACTGGTCGAGCGACGTCTTCCCCTGCTCCTCTAGCGTGTCCGCAAGCTGTTGCACTCGGCGTGCAGTGAGGTGCTCGTATTCCCTGAGCGCTGCTGCGCCAAGACCTCGAGTCCACGGGCGCCTGCGACGCGCGTGCTCTTCGAGATCCTGGATACCAACCATGGGGTAATTCTGGCCGAGGAACGCTCCGACGA

>AT5439

CCCACCTGTCCTCACAACATCTCCGTGAAGCGCATGTAGATGCTTGAAATACGTGTTCCGCCTTCCCGTGATGGACATGAAGGCATGGCAGAAGCTGGACGTGCGATACCAGAATGGTCCCGGGTACCGCGCGAGAGGGTGCCAGGGCGAGAGGCGGTAGACCACCACAGATAGGACCAACGTCAGGTAATAGACGGCATAGCAGGACAAGAGCGTCCGGACCTCCGTTGTCGAGGACGGCAGGTGCTGAGTATATGTCAGGATCGCGACGGGAGGACCAAGGAGCAAAGCTGCATGGAGTGGGATGCAGTAC

>AT5441

GGGGCGGGGGCGAGTGCCTCGGCGAGCCCGCGGGTGAAGCGGCAGACGGTCGGGGCGTCGGATGCCATGCCGGTCAGCGTGACGCGGACGGGGCGGGCGCCGGCGCCGTGGGTCTCCCAGCGCGCCACGTCTTCGTCGTCCCGAAACCCGCGGAAATCGAGATGGAGGCTGAGTTCCGCGCTGGGGGGACTTGAAGGAAGGGGATTGAGGCCGGGATGGAGCGGGGCGGCCGGTGGCCACGCAGCGTAGTTGACCCGATAGTCAAGCACGAGAGCCGTGGTCGTGGGGAAGACGAGGTGCGTGAGGAAGCGGGGGATGTAGGCAGGTGCATCCTCGATTTCGAGGAGACGGAGACGGGGGAGGGGGATGGATTTCGTCGGCTCGCAGGGCGGTCGGCGGCGGGCGCGCAGGTCCGAGTCGTCTATCAGGCTGCCGGACAGAGACAGCGACTCTAGGTTTGGGAAGAACTCGAGGGCGTTGTGCATGGCACGCAGTGTAGGAACGTTGCCGGCGGCGCCGGGGGAAGGGCGGATGGTGCAGTTCTTTAGCTTGAGGTGACGTAGGGATAGACACGGCACGAGCGGGGTGTAGAAGTGCGTGCGCTGCAATTCGAGGCTTCGAATGTTGGGATAACGACGAAACTTCAAGTCTAAAGTACACGCCTTCGTGTACCGCGAGCAGCAATGGAGGATGGCGAGGTGTGTAAGATAAGGCATATGTTGTTCGAGGACTTGCGCCATCTCGTCAACACTGTACGAAGTGGAGCCTACTTTGAAGGAGGAAAGGCGACTAGCATGAGGTAACAAGACGTCAACGATAACCTGGTTAGAGTAGGGGACAGAGAGGGCAAGGCTTTCAGGTGCGGACCGCGCGAGGGCTGCCCGAAAACGACCCATGTGGTGTTTCGGGTTCCATGCTTTCCAGGTTGACAAACTGAGGAGGTTCACCCAGAACTGAGGTGTTCTGAAAATCAGTCGTCGCCAGTATCGACAGACACGCAACACAGGTATGCGTGGTCGAGGCATGACGGCGGGATGTACGTGTGAAAAGATCTCCATAAGCATCTCTGGAGGGAGGTACTGGTGGATAGGGGCTGCATCGTTATATGCGGAGCGAAGAGCACGGACGTGAGCTTTGTTTTTGGAGATAGCGTTGCAGGCTGCATGACCGTAGCACTGGCGAAGGAGGCGGTTGTGACTTTGATACTCGGCGGTCCTTGATTGGCACCACTCCCGGATTTGGCTGGGGGCGAGTTGGCTGAGCGACATCCTAT

>AT5484

TGGCCAGAATGAGAATCTCCCAGGCCCTGGTATGCTCGAGGGGCTGCAGACGCGGGCGAACTCGGGGACGGTGTGCAATCCATAGCGATACAACTGTGCGTGCAAGTCGCTGTCCTTGAGCTTGTCGAAGAACTGCTGATTCTCTGAGCGCATGCGGAGGAAGGAGCGCATGATGTCGGACCATTGCTCTTCGGATATTTGCGGGAAGGCTCGGGTGCG

>AT5489

ACTGCTAGGTGCCTCACAGAATACCGTGTAGAGGACATTCCTTAGATCGCCGCAGGCAAGTAAGAGAATGTTTGGCGAAGTCTCAGGAGCAACATCCCGCGTCAAGCACGTAGCGGATGTGTTTCCGACGGGGTGGAAATGGTACCTTCCGGGCCAGTAAAGAGACAAAGCCATGAAGACACGACGGGGCGGGAACGAAGAGATTCCGGCCGTTGGGTTGAAGTCGAAGAGAGAGGTTGGGTCGCGACGCCGCCTCCCACAACCGTTGCGCGTCTATCGATATCCCGTGGAAGGTACTGCTGTAATGGCATGAGCATCGTGCAATGACGCTCGACAGCATTAGTCGGCGGCCTACCTAAGTCATGGCATACTTTATCCGAATAAATACCATCTAGACATTTTTGGCGTAGATCCCAAGGCGAATCAGTACCAGAGTAGTAACGAGCTCCCGGTAGAACACAGTTCGTTGGACACGGGTCATTCCCGTTGACAGACAATGCGCATAAAGATGAGGCACACCTCCTTGGCGATCCTGACCAAGGTAATCGTCCCATCTCCATCTTCTAGCTGAGTCGGACCGACGATGATGTCGAATACGCCACTGCCAGGCCCCGACCCACCCATCTTGCTCTTGCCGTTCGACAACTTCTAAGTATTGCTTCTGAGACTGTTCCGCGCGCGTTCTCCGTCCCGCCAGCTACCCGTCAGCGCCATGGCCGTGGTCGTACCTCAGCAGTCCGAAGCCAACAGCCCGAGCTGCTGTTTTCAAGCAGATATATACGCACCTTGACTTTGTTGCGTCCGTTCTGAGTCGGTCTAGTACACCGTCGTGGATTCAGGCAACGCGGGGGCGACCCGCACTGTGGTTGTTGCA

>AT5532

GTTGCAGATGAAGTCGGTCGCGCCGACGTAGACGAGGGCCCGGATGCCGCGCTCGAGCAGCGCGGCGAGGTAGTAGTGCGCCGGGAAGGACGAGGAGTCAAGGGTCTCTGTGAAAGCCTGGTTGACGGGGCCGGAGCTCCACTGGAAGTTGGGCCCGCGCACGGATGGGTCCACGCCGAGGAGGGACTGGATGTGCTTCTTGTTAAGGAAGTCGCCGATGTGCCTT

>AT5534

CACCGTAGGATTCGCCAGCGAGATGAAGCGCTCTCCCCTTGAACTTGTGGAAGTGCTCAAAGAAAATCGACATGAATGCCGTGATGTCGTCCCCCGCATCCTTGGTCGTTGCCTGCCAATTTTTAAAAGTCAACCAACGCCATCACATGATACGAGATGAGCCAACATACGACGGACTCTCCGTATTCCGCGTAAGAGAACCCGACGCCCACGGGTTGATCGACAAAGAAGATGTTCGCTTGGTCGTTCCAACCGTACGGATTCGCCTTGGTCTCGTTCGCGCTCGTCACTCTGCAAGGACCTATAGAGCTATATATTAGCCGAGGCTCGTCATAACTGCACCTCGAGATGAATGGATTTACGCACCGAGTTCCATAAACAAGCCCATGCTGGAGGATGCACCCGGGCCGCCGTTAGTCCAGAAGATGATGTCATCCGTGTCGGGGTCGCGACGGGACTCGAAAAAGTAAAAGAACAAGTGCCGCGCCTCGATGTCTATGTACCCCGTGTAGGCTCTAGGTGCGGATGAACGGCTCCGGTCAGATCAAGGCGTTTTGAGACTAAAAGGTTCAAACAACGCATACCGCACGGACCCATCGCAGAACTGCGAGTGTTTAACGCGCACCTTGTGACGCGAAAACGCCGGATGGCTCAACGTCGTGAATTCTGTGGACGACAGAACGCTGAGATCTCCCAGGGGCGTGAAGAGCCCATCATCATAGGTGCTGAATGTATTCTGTCCTCCGAAGGGCAGCTGTGTCGCGCTGGAGGCAACGACGGCGAACGCCAGTAGGCCGGAGACCAGTAGCTTCATGTGGTGGTTGGGTGCAAGTCCTCTCCTAACGTCTTGGCCCGTGGTAGGGACGAAGATAAGGATGCACGGTCATGTGGATGGGAAAAAGAGGCCGGAGCCGGGAGATGACCGGTGACCAGTGGGACGGTCCGCAGTCGCGCGTACCGGTGATCATGGCCAATCGAATCTTAAGGGCTCTCGAGGGTGGTTCTTCTTCAAGTTTAGAGTGATACCCTCAGGGTGGTCAACCTGGAATTTAAGCATTGAGGACTGAAAGACTCAAAGAGGCGGGCGACGGCCCCGGCGGATATCGATGATGGTCACTGGCACC

>AT5542

CGTGAGGGCGAGCCCCATGTCGGTGGGCGCGTCGAGGCGTTCGTAGATTGTAGGCGCGTACCCCTTGAGCTTCAAGAGCATCCCCAAGACAGGTCCGGCGACACCGGCACCAGCGATGATGACCTTGGTTCCGCCCATGTCGTTACAAAGAGTTCTTGCAATTGCGGCTTGAGGTTTGCAGTTGGAGTTTGGAGGTAGTGTAGACTGTGTAGGGCAGGGGTTTTCT

>AT5554

CCCCGGGGAAGCTTTGGATGGTGGTGGCGTCGACGGCGACGATGAGGTCGTTGTCCGTGTTGTGTTGGCCGCAGGAGCCGAGGCCGGGATGGAACCAGGTACCTATTTGCACGAACATGGTCTTAGCGCTACTGACGGGGTCCGTGAAAGAGGGGTTTGAGGGGACGCTTACCATCGCCACCCGTCTTGGAGAGTGCGAAGCTGGAGGTCACGGCGGAGAGGGCGACGAAGAGTACAGCAGCGAAGCGCATATCGGTTAATTTAGAGGAATAAAGTTAGGATGGGGTTGAAAGTATGGATGGGAGTGCGGGTAGAGACGAATTGTCAATGAAGGTTTCGCCCATCGTCCGAGGGTTTTATACCTGGACAACTCTCTATGTCAACCTTCATGATGTTTGGGCAAGACGCTCACCCAGATGTGTCCGGCTGGACTGGACAACACCATACGGCACGGTGAGATGGGCCATATGCTGTTCATATGCTGGACAGAATTCGCAAACCCAAGGAGTGCGACCATCACGATGTTAGATAACCTCGTCGTCGGGCTTCAGGGAGGGATTCGGAATACCGGCCATGCGCGTGCGCGTCGAACATTATGGCGTCCAGAAGGGAGGCCAGCAATTGATTC

>AT5557

GACGTTCGCTGCACCTCCTCCCATGCCTTTCCAGAATCCAAAATAGCCGATATGCGGTTGTTCGCGAACACGTTCGGGCGCACCTCGCGAGTAATATGATGGGTAGCGAGCAAACGCAGCATATGGCCTAAGGATAACGCGTTAGCTCTTGAGCATACTACACCCCAAACAACTACCGACTTACTGAGCTTCCCCTCGTCGACATTCACATGCCGACTGAGCTCGCGGACGTGCATTCCATGCGGACCGGCAGAACGAAGAATCTCGACCGTGTGGCTCTCTTCCAAAAAGCGCAAG

>AT5567

GTGCAGTCGGAAGGTAATGGTACATTCCGGTATAGGTACAGTCTACGGGGAAGTCTACTGGCAAGAAGTATACTACACAACAGTAAGGACTAAATGTGATGTCTACGATAGCCTACGCGAAGTATCCATGCAGGATGGCTAGAAAACGTCCGAACAGTCTACATAGAACACCTAGTCGAACCCCTTCATCACCGTAAGGGGAGGAAACTCGACTGCTGCCCCTCGGCCACGGTGCGGTGGGAGGAAGAAGGGCTCAGCGGCCAACACTGCGCGAGCGCGGGGCTGTCGGGAAGCGATATCGGCCCTTTGGTGTACGAGGGCGGGCGAGAGAACGGCTCGTTGGCAGGCTCCTCCGAGATGCTGGAGAGAGTCGACGTGGAGGCCT

>AT5572

GCGCCAGCGACCTCGGTTTCTCTTCTCATGCGGGGAGCTTTATGTACTCTCTATCTACTGACAATGTGTCTACATACAAGTAGCCCCGATGGACATATCAAACAAGAGAACGATCGATCTGTTGGATACTCTCGTCTACCGTAAGCTCGCTCCCCAGAACTGCCGCATGCCCTGCCTTGCTGAAATCCGAATCATCCTCATGAGCAGTATGCATCGTCTCGACGCGGATAGCAAGAGATGGGTGGTGACCCTGGCGAGTTGAAGGTCAAGGTGAATGCTCGAGGAGCAGGTTCACTGTGCGAGGAGACTGCTCACGTGCTTGTTTTCGGAGCTCGGCCTGCTGTCTAACCCCACCAAGGCGAGATCTGCGATTATGCGACTCTCTAGGCTCATGCCTCTCGCGTCCACGTTGCGCACGAGGCGTCGGGCGTTCAGGTTCGCCAAGAGCATGTTCAAATAGATCGGGTTGAGTGGCAGCTCCACTGCGAAGAGCTGCAAGCCATCAGGAAAGGCGGCGATCTGGGGAAACACAAGAGTCAGCCTCGACGGCCTGCAAAGCTGAACGCGTTATGGGGGAGCACCAAGGCGACGTCGACGAGCGCGATGATTGTTGTCCACAGCCCGGTATTCACTGTCATGATAATGAGGCGGTAGATCATCTTGTTCGACCTGGGTGAAATCGTGAACACAGAGGAGGTACGCATAGCTCTTCCAACGGCCGCACCTGCGGATGTCGGGCTGACGATTCTTTGCCAGGAGATAGACCATGGCAATCGTAATCATGATGTCGACGAATGCACCAACAGAGCGACCGGCGATCTCCAGC

>AT5576

TGACCAAAGAAATCCTCGGGCGTCTCGAGGATCTGTTTACTGAGAATGGCGGCAAGGTCCTCCTGAATTACATAGTAGCGTTTCACGGCGGTCGCGTTGAGTGCCGTATGCGCGAGTTTGCGGTGCGCGCGCCATTCTTCTTTACAAGGGAGCAGAGGCATGCTCTGCCATATGCAAGTCAGCGTGGTCCTGAAAGTCCCCTTACGCCCCGGCGGAATGACTTGCGACGCACCTGTCCCAGGCCCATCAGGTCACCGACCACGGTGAAGGATGGCCTGTCGGAGTAAATGCTTCCCCTTTTCTCCAAGAGGTCGTTTATGGCCTTCATGGAGTTGAGGACGAGGATGTTGTTTCCCAGCCCATGGAAGAAGACCAAGTCGCCTAACGGTGACGTCAGCATCGTTCCGCGCGCGAGGGAGGTACTGAGCGAGACAAACCGTATACCGTTTTATATTCGGTGAACTTTGTCCACGCCCCTTGGGGAGAGACATCCAGGATGTTGCCTACGAGAGGCTTCGCTGGAGGGCCCGGAGGATAGCGTCGCTTCGGGGACCGAAAGAAGGAAACCGCGGCGAGCGCTACGAGACTCGCCCCCAGAACGACATACAAGTCAGAGAGGGCCATGGCTGGTAAAGGTGCTCCAACCAACTCCAACGTCCGAATTTCGGTCGTTCTGGCACTAGTTGTTGGCAGCACTAGGTGCACTTGCTCTGTCCAGCGTCCCCACTAGGTCTTCGAGTGGCTTCAGGGCTGCGAGTCTGCGACTCGGACTGCCGGGCTTCGGCGCGACAACGGAGGACAGTGTTACTTAACTAGCGTAAGGAAACTGGTTGACGCGT

>AT5583

CCACCACCTGCAGAAAATATACTGAATAAAGGGCACCGCACCAACGTGTACGTTAACGTGCCCGCTTGGCTGACCGAGCCTCAATCGCTCTCATACGAATGAAGGATCAGCCCCGCCGCCTAGAGAAAATCAGCGTCAGCAAAGCGAATCCGTTGAAAGGGGAAGTTTGTTACCTTCTCTGCAACAGCATACACCGTTGCCTGTATATGCGCTGCAAGTTGCATCGGGAATATGCTGGCGTCAACCACACGGAGGTTCTTCGTGCCGTAGACCTTCAGCTCACGATCCACGACGC

>AT5584

TCTGCGTCGAGGGATCGGGGAGGTTCTGAGCCGCGATTAGGTTCGAGAAGGGTGCGGTCTTGCCGAGCTTCTGCATGTACTTGAGCACGTCCACGAGAACCTGAGCATCAAACTCATGCGCCAGGAAGTTCGGGTTGATGGCGGGTGCTGCGAGTGGGTCGTCGGAGGCGATGTGCTGCATGGAGTAGTTAACTTAGCACATGTATCTTCGCATGACGTCGTCGCACTCACCACACTGCCGCGGCTAGTCCCATGCTACATAAGCG

>AT5596

CAGTAGGGCCGTCAAAGCCGTCCACGTGAGAAAGGTCGGCTTCAACATGAACGGCATCCTGAGCAGAGATGCCAGGGTACGTGTCGGTCGGTGTCTTGGCGAAGCTTTCTGACTTCTTCATGAACGGAAGGAGCTGAGGCCAACCCCAATTTGTGTCGCCGGCGAGCTCTGCCCACGCATCGTATTCAGCAGCCGTGCCCCGATTCCACGCCATTCCATTGATGCCACTCGAGCCTCCAAGGAGCTTGCCCCTAACC

>AT5597

GACCGGCAGCCTCAGGTGCCGCACATGTTGCAGCCAGCGTTTTGCGAGCTAAAAAGACGAGAAGTGTCTGGAGAAAGGAAACGGAAAACATAATGGAAAAGGTTGTTGGGGAGGCGAGGTTTCCGGGTCGGGTCGCAAGCCATTTTCATCACAAGGCGTATGCTATGAGAAAAGGTATGCGTCGGCCACATGATACAAGCTGTAGTGCCATGCAGCTTAGTGCAAAAGATGGGGGTGGTAGACATGAGACTATGGTCACCATCTCCAAGTCAAGGTCAAAACGCAAGTTGCATGCGAGCGCTA

>AT5610

CCTCGTCTATATGAACGACTACGCGACCGTATGAACTGACGTCTTTGTATCCAAGAAGCGGCCAGACAACCTCGGAGACACTCGCCCAAGTCGTGGCGGCGAGGAATAACCAAAAGAGAAGGTTGTGAAACGCCCCAGCGGAAATAATCCTCACGCGAGCGCGAAAAGGGTGTGTGTCGGTCTGGTCAGCGGGAAAGGAGACGAAGGCCGACGGCAGGAGGACGGTAAAACCGAGGCCGGCGGAGGTGAGTGGTAT

>AT5616

GGAGGGTCGTAGAGGAGAGAGGGGGCTGGGCCGGGTCTGCGGTATCTGACATTGTCGGAGGGGCGTGCGGTTGAGAAGGCACGTTCCGAGGAACTATAAAGAGACGTCGATCTGTGCGGCCACGATCCTCATTGCTTGGACTATCACTGCTCGAGGATGAGAAGGATCACTGGACAGCCCAACGGATTTGCTTCCGGTCATGCGGATTGGTGGAAAAACCTCTGGCTGTGGCGCGTGGGTTCCGGTGCCTCCGGGGCCAGGACCCATCGGATCTGCGCTGTTCCAAGAATACATGGCGCCTCGGGCACTGGCATGCCAAGTCTGTCAGACATGAAACG

>AT5636

ATCCCTTGAAATGTTTATGCAATTATGCAAAGCACTATAAGTACGTTAGACTAGTTGCTGAGCAAAAGTGCTGCATTATAGTAGACTATGCTACATAAATAGCAATAAAAATTTCACATTGACACACAACCCCTCACATCCAAACCTAACTCCTTCATCTCCTCTTCTGCTGCTTCACGCTCATTTTGTTGCAATTTCTTTCGAGTTTCAATAGGAAACCAATCCTTCATAAAGTTGAGAAGGGCCCAGTCTACATTTGCTTTTGAAAAAAAGGTGAATAATCACAATCAATGTCCTCGGTAAAAACTTACAACGATCAGCAGACAACACTGTTGGTGCCCGACACATCGGACAATGCTCCTCTCCCCGTTTTTGCAGCTTTA

>AT5651

AATACGTGGCGTCAACGTCATCGAACGCGTCTGAGTTCAGATAAGAGTCGATCAAGAGCATGGTCATCGGATGGTGGGGTTGGTGTTCGAGATGAGAGTTCGAGTTATCGAGAGAGAAAGAGGGCCGCAGGCGTTGGTAGCTGATATACCGGCGCATACAACCATCTGCCAAAGCCTGCCCCATGGATTTGAAATGAATGAAGGATGCGTGCGTTTTCGGTGTGATTTAGAGTCTTTTGTCAGTCAAGTTGGCGACTCGTTCCTTGTACGGTGAATAAACGTTTAAGTGCAGGATGGCCGCGTGGCGTGGCACTGGACAATGGTGCCATTGGGACGGTGGTGGTAACGGAAGAGTGGCCATGCTGACGACCAGCGGCTCAAGTCTTCAGCCTACATCACACGTTTACACGTTGTGAACAAGGCTGAGGAGAGCATAATGATGGAGTGCTTCTAAGCATACCGATTTGAGTATCGGGAGTAGTGGCCACTGGTGCCGGGAGGCCAAGGGATCCTTTCAGGCGGAATATTCATGAAGGAATCTACTGT

>AT5663

TCGTCGCGTGCGAACCCGAGACCATTGGGCGAGAACGCCAGAGCAGCTTGATGAGACGTTCTTCGAAGTCACGGCATGTCGGGATGATGGTGTCAATGGAGTCTGACCAAACCACGAGCACGCGCTCGTCGCGCTGAATTCGACAGAATGTTGGTCAGTAAAATATATGCGGCATGTCCAGGGTACAAGACCTGAGTGTATGCGCGACCGTTCAAACGCGTATGTCAACTTGTTCTGCTCCAACGCCGCGCTTTACGACGAGAC

>AT5670

TCTACGTTATGGGAGTGACATACGGCTGCTGTCAGTCCCCAGTCCACGGTCCTCACGCAAGGCCGCGAGGTCCCTCTCCATGTGTCTATGTAAATTGTGCATTTCAATTATATCTCAACCATTCCCGGGACTGCATAAGTTAGGGGGGAGGTGATGCTACACGAGAGACTCCATTCCAAGGGACAACGCCGGATTCGGAGCGAAAACGAACAGAACGCCAGTCAAGTATGAGTACACGCCTCGACGGCTGTCCTGGAAGGACGTGCGGCCGAAAAGCAAAAGCATAGAAAGACAAGATTCAAACCAAAAGAAAGGAATGCATGTTCGTACGTGGTCGCCAACCAGTCCCCCCACAAGATGCACCTCGCTTCACTATACGTTGCCTAGAACTTTTCGAAGGCGGTGTGGCCGGAGTAGCTGGGGGCGAAGTAGTCGGTGATGGTCGTCCGGACGGACCTGTTG

>AT5498

GTTCTGAGTAGTTCCGATGGACCACACCGTACCCGTGGTGGGGGTCAACACCGGGGGCACGAACACATCCTGAGCAGAGCGAGACACGACGGCTGCGCCGAGTGCAAGCTGCGAGTAGAGCGCGGCGGACGCGATGACGTAGGCGAGCTTGGTGAACATGGCTGGAGTCGTTGGGGTCGCTGCGTTCTTTCGGGAGAATAGAGGCTTGGGATGCGAGTGGGGTTGACTACCTCGAGGAGGGCCTGAGGATCCTGGCGAAGACGGGAGGGTACTTATACAACCTGAACGTCGGGCGCCAAGGCTGGATGCGAACGCGTCCTTGCCATCGCCTCTGAGACCGAGTGTGGCGGGACGGTGGGCGAACCTGTGAGAGGGCTATCAAGCCTCGGCCCCGCCTGGCTCAAATGTTACAAGGCACCAAGAATCGCAATCCAACACCAAGTGAGGCTTTGGCTTAGAGTCCATTTGCCCGAAGCTCGTTGGCCCGCCCGGGGTCGGGGCTGTTGTCGTCGTTGGTGAATTGTCCTCGGCCGTTCGAGTGCGTGTGCCCATCAACCATAATCAATCCCCTTCTGCTCCCGTACCTCCGGGCTCGCGCGGGAAAGAAACGGTGTTAGATCGCCACCCAAACCCACCTGGAACCCGAGAACTGCAGGGGAGGGGGCGATGAGTAGCCTCAAATAGGTTCACATGGTACTGTGTCCATCCATGCGTGCGGATAAGTTACAGTGTACGTACTACGGCACCAGTGCGTTTGATTGTGACGAGGCAGAGGCGGCGGGTTTCACGGGATGTAGATCTTCGAGTGTCCTTCCGCGGTATGATGCGGTCGCCGTCAGGACACTCGGAATAGAAACCCGTGTGCGGAAACAAGGGGCGTACC

>AT6097

CCCACTAGAATCTGAACCGCTCGATGAACTGGTGCTGCCAGCACGAGGTCTATTCCACACCACAGTTGCATTGGTGAGATCCTCGGGTCGCGGCTCTGAGATATTTACGACCAGCGTGTTGTCCGGATTGATGGGACACGGGGTAAAAGAGAGCGTGATGGCCCGGTCGAAGTCCCGTTCTCTCAGCATCGACCCCATATACGTCCCGTCGACGAACGTGTTCCTGCGGCCTTCCCATTCACAGATGTGATCGGCCGGGCAGTCGTGCCCTGGGGTCAGAAAATAAGGCGATGAAGGGGGGAAGGAGTCGGGGGGCCAAACAGGGTTCTCCCAAGCTGCGAGCGTTATAGCGATCATGGGGTCCATCTCGAACCGAACATTCGCCCAGTGCGGTCGCAAATCTCTACCGTCACCCCGACCTTCCGAGGACTCTGGGGAGGCATTTTCGCAGCGCCCTAGTTGTACGATCATTGTGTTGCGGAAGTGGCGAGCATGAACAGTGTTTCTGTGGTCGAATCTGAAGGTTATTCTAGCCGGTGGCGACCCGGTCCAGGGCAATGACTGAGGTTCAGCCGACTCGACTATGAGCCGATGCCGGGTTCGCAGCTCCTTCAGGTGGCGTTCGAGGATGCGGAACGGCGTCTGAAGACCGCTCGTGAGAAGGACTGGTGGAGGGACGGCGGTAGCGGTCTCGTGGGGTGGCTGAGTGGCAAGATACACCTGCCTCCACTCCGCAACGACCGGTTTGTTGAGCAGTTTGACAGGTCCAAACGTATCTCCTAGAGCGATCAAGCGACAGAATGCGGAACGTCGGCCACCGCGAAGCGATAAATGGCCGCCGATGTCGTAGACGGGATAGTCTTCGTCAATAGAGAGAGGACAGCGCTGCATGAGAAGCCCAAGACGGATATTACCCCGCGACAGGCTCAACAGTGCAATGGTGTGCGGGCCGTCTTCTATCACGGGAATGCGAGCAAGGACGCCATTCGCGGTGACGGTGAAGGTGAAGCTGCCAAC

>AT6104

TAGCCTCAGCGAAGGCGTTTTCGTGAATGTATGGACCGTGCGGCATGACTAAAAGGCATAGAGGGAAGGGAAGGGGAATGCGTCGCGCTCTGTTGCAGGCAGTGAAAGACGTCTAACAGCCCGAGTAGTGGTTGAAAAGGAAAGAGCACAATCGAAACAGACGGGGGAGTTCGTTCGCAGTCGGCGTATGCCGCTCGGCTAGTTGAACGGACAACGCGTTAGGGCTCCTACTCTTTTCGGGGTGCAGAGTAAAGCACCTTGGGTCATCCTTGA

>AT6105

TGCGCTATCCAAACAATATTTGATGCACATAATCTCGGAAGCGGTTCTGCCTTCGCGCTATGGTTGACGTCAGTAATACTTCAGTCATGCAAAGTACCGGTAATACTAGGACTGGATGGCAGTGGGTCCAGTACACTGTGGCAGACTTCAAGCCTAAGCGCTGTCCCTTTACAAACCGTTATCCGAATCGTTCGCCGAGCGCACCATGTCCACTAGAGTCTGTGCCGGTAAAGGACGCCACCCTCTTTACTCTCATTACATGTGCCGAAGTCATGCAAAGACCCACCAGATCGGTCTAAAGACCTGCAATTAAGGCTCCAAACTGCACAAAACATTGTGTTCCATATAGACACTCATGTGGCCTCAAAAGCCTCGAAGTTCAGGACATTTTTTGAATGCTCAAGATTACGGGGCTAAAAACACGGTACGGGTCTATCGATTCCGATGAATCGAAGAAGTCAGGTCGGCACCGAGGACCCAAGAGACGGCCAATAAGTATTTTCTCTCTAATCATCCAGAGACGGTCAAAATTTGCAGAAGAGAGCAGATTTTTTTACACCTTGCTAAGAACACCTTTCGGAGTCTGGGACGGCATTGACCCCAAAATGTACACGAAGGAAGTGAAGGAAGAGGGCAGGGTAGTGACACACCACCACTCGAAACTGTGCTCTCGAGCATAGCAGACGAGTGCTCTTACAACGAGATAGAAACTGACGTTGTGAATAAAGCTGAGGGGTGACAGTAAACCTAAACCGAAGACATATGGCGTACCATTCGTTCTAAGCTTCCTCCCTCCATGATGCCTCCAGTCCACGAAAAGCGGGGATATTTGAAGGCGGCTCATGGATATCACGGACTCGAATACCAGTGCTCTATCCAAGGTCCCTTCGGATCGTGAAGGCTGAATGGGCTTAATCAAGGCCACAGAACCGAGGGGTTTTCGAGTTTTCGCAGTGCGAGTGGGAGTTTGTTCGAAGGGGAGATTGTCTAGGATCCTGAGCGGGATTTGGATGGCAGTGAGCGGGAAGCAAATGGTGGAGTCTAGTAGTCC

>AT6134

GGCGTGACGGAAAACATACGGAACACACGCAGGATGGGGACAAACCCCTCGCAAACTCCGGCCAACTCGAGAAGCATAGAAATGACAGTGGGGACTCACCAATGCAGACACGAGCTGGCCAATCGACCACCATATGGACAAGATGGTGAGCAGATACTGGTGTGTGCCGGGAACGAAGTCGAGGAACACCGCCGAATCGATGGGCAAATTGCCCCCAACGCCCAATCCTAGTACCGCGAGGAGAGAGGCCAGGGTGACGAAGTTTGGGGAGCCGCCGGCCGCCAATCCGAATACGCCAGCCAGGAACAGCGTGATGTTAAACGAC

>AT6136

CACCTGGTACTTGCCCATCCCGATCTCCTGGAGCGCGGCGTTGAGAACCCGTGCTTTCGCTTGGTATACTGGATCTACCGAGCCCTCCTCGAACATGTCCTCGTTCGCATAGATTGGCAACTCCGGGACGATCGCGAAGGCTTCGCCCTTGGCGTCTAGAACTACCGTTTTCTTTTCGAGGGAGGGCATGGTGGGTTGGCCTACTTCGATCCGACAAGTGTGGGGTCGCGAGATGAACGAAGGGGGGGAGAAGAGCTTTTATGAAGACCGGGGAGCGGGGAGGTAGAGCGGGATGGTGCAGAGTCCGAGAATGCAGGAGCTGTCGAACGGTCTGCCGAGCGTGTGCGCGAGTCTGGCGCGAGTCAGAGATTTTCTGCGACAAGGCGTCGGGGGTCCAGGGACCGGGTGTATGCTCGTCGCTCCTCCGGCGACGACGCCATCGTAGTTTATACTAATATAGCCTGCGGATCC

>AT6139

TACATATTTGAGTACACGCCAGGCACCTGAGCCTTCTCCACCGCTTCTGGAAGAGGCCCAAGCCAGCGCTCAGGCTTCCACTCGTACGCATCCTCACCCCAGAGCGCCTTAGAGCGGTTGCACGCCATGATCGCGATGACGATGTTCGTGCCGCGGGGTATGGCGAGCTATCTTATCGTCGACCCATCCGTGCCGCGCATCGGTTGGGAGAGGGGGAGGACTGCGTCTTGC

>AT6146

CCAGCTGGTGCGTCGCTGACTCTCGGTGACGCTGATGTGGATGGCAAGTCCTGGTGATTTGTCCGTGAAACCGGCCCGTCATCTTCATCCCACATGTTGGCTCTAAACAATATGAGATCAGAAGCTCGGAGTCATATAGGAGGGACGACTCACAGTGCACTTTGAATGGTGTGTTGTCGTATGGGAGAATGGGAGCCTGTAGTGGATTGGACCATGTCAACGCCGCGGGGTGGGGCCATGTTCAGAACTTCAGACTGGACGGGATCCTGCGCCACCGGCGATGAGTTGGTTGATGACTCGGATGCGGTGGGTGCGGCAGTGGTTGTTGGGGGATCAGAGGGCGACTGTGGTCTGGGAATCTCAAGTTGAGTGTTCAACTCGGAGGGGCGAGAAAGTGAGGGACGAGAGGGCGACGTAGGCATACTAGAAGTGGTGATTGGATTGAGCGCCGGCGGGTGGGCGATAGGCCGTGGACGTTTTGGTGTTGGCGTGCGAGTATGTGTGTCGGACGCGTCCGAGTCCAGCAAGAGCTCTGTCATCACTGGTCGCTTTCGGGAGTGAGCAGCGACGTCGCGACTAACAACATTTGCCGGGAAGGCATCGGGGGCTGGTTGAGCTGAGTCTGGCTGATCCAGAGAAACAATGCCCGACATCTTCCGATGATAATTTTTGTACCATGAGGAGTATGTCGTGGTTGCGATGCGCTTGACCTTCCAGTTATTAGCACAAAACCCAAGCTCCGGGAAGGCCTCAACCATTTCC

>AT6147

GTATTGTTGAGCTCCCTTCGGAGGCGAGCGTTATCCCATTGAAGTCCGTGCATGGTTGTGGGCGCCGAGGGCAAAGACGGGCTCAGTGGAGTCTCTAGCCCATACAGAAGTAGTTCCGAGTCACCAGTACGTGTCGTGGAGCGGTGAGGGGAGAGTATTGGGGGATGAAAACTCGGACCGGGCGGAAAACGATCGTCGGAAGTGAAAGGAGTGGCGTGCATACTGGAATATGAATATGGAATGTGGCCGGAAGTCATACTTTGGGTAGACCCTTCTGGTGCAGCCGAAGATGCTCGAGAGTACGTCCGAATAAATGGTGGGCTTGTGAAAGGGAGTAGATGATTTGATTGGGGTGAAGTGAAGTGGGTAGTTGGGGGTGCGCGAGTCGGTGAGGAAGACGGCAGCAAGAAATGGTTCGAAAGCTCTTGTGGCGACTCAGAATCCTGTCGTGGTTCGGGGTCTGGAGGGCGTCCCGCACTCGTAGTACCAGAGTAAGCTCAGGGGAGGTCAAGCAAGTTGTGGATGGAGAGGAAATTGGCCGTTTCGTGCATCCGTTTTGAACGTAGGCCGATTTGGTCGACTGTGCCACTCTTGCTTTGATCTTAGCCGAGACAGGCACATTGTGACATATGTCGCATCTGAAAAGAGCTGTCTCATATATGTTAATGCTATACACGACGAGTCTGGGTGTGGATAGTGGGAAGAGAGACGGTGGAAAGGACACCGACATCCGAGTCATTCAGC

>AT6151

TACTCTGTGACGAATCGTCATGCTACTACGCAAAAGAAGGTGTTCTGGCCCGAGCCATACTGTTCAAACTCGGAAGGGTAGTCGATGGCGGGCACGTCCGACGACCCGGTGTCAGAAGGCTGGTCGACAGGGATGGGTTCGGCGAGAACGAAAAATTCGTCCATGGTAGGAAGAGTGGAGCGAAGGTACTTGGAAGATTGGTGATGATAGGCAGTGTTGCGTTGGACGTACGGTGCAAGTTTTATTAGAGAACGGAAGTGAGCTATTGTGCTATGGTCGTGGAACAATGCGGCTATGGAGTTATTGTGCGGAAGGGACTAGC

>AT6165

ACGTGTGGGTTACTCTCGTAATGCCATTTGATGCTGCTGTGGTGAGACGGGTCGTGCCTCGAAGACGAAAGCGATCGCGACGCTGCGCGATGCGAGGAGCGAGACGCTCTGCGTTGCACGCGAGGCTGGGCTGCATTGCTGCCGTTGTGGGTGGTCTGATAGTTCCGTGCGCCGTTGGAGGCAGGAGTCGAGGGGCTGCTTCCGCCAGGAGTCTTGGCCGCGTTCAATTTATGTGAAAGGGCGTCGAGTAAGGATTCTGGGCCACCCGTTCTTGAGAATGGATTGGCAGCCTCAGTGCGGGGTGTAATAGACGGTGATACGTAACGACTCTGCGAACAGGGGGTCAGAACTACACTTTCTGCTATTTGAGGTGCGTATATGCGCATACAGCGCCATGCGGCCACCGTTCCGCAGTGGATGTCAACCGCTATGCCTCCTCGAGCATCTGTTTTGGTCCACCTGACCTAAAGTCGGCCGCAGCGGTATATGCACCGGTAGACCCTCCCTCCGTCCTTACCAGCACAGGACGGTGATACACACCTTGTCTCTGAGATCCTCCACAATACTTCTCAGGGGGACAGCGGGCCTTGAGAGCAAAGAGCCCCGTGCAGGCGTGGTAGAGTTGAGCAGCGCAGGAGTCGATGGGCCAAGCGAGTGCAGAGAAGACTCATCCGGAGAAGAGGGTGCCGAGTCCCCGAGCACGGTCGTGCTGGGGAGGAAGAGGGATGCGATGATGATTCTGTGATGCCTCAGCGAGCTCATCGTGGTCGTTGACGAAGATTTTGGGCCGCCTGGGGTCGCCGATGGTCGAAGGAAGAGAGCTGAAGAGAGACGGCAAGAAACAAGGGCTGAGCGACGTTTTGAGT

>AT6169

TCCTGCTCGCGGTCGCCCATGACGTCGTCCTTACGCCCGCCGACGTGGTCCTAGGTGCCCTCGGCGTAGCCCTGCCCCTGCGCAGCGTTGTACTCGCGCTGGCCCTGCGCGTGCTCCTCCGCGCCGGCATGCTGCCAGCTCTGCGACCCGATCGCGTTGCCGATCGTCTCCTCGACGCCGCCCTTGGCCGAGTGGTACTGGCCGGAGGTCTTGCTGTAGAGCGTTGATGGGTCGTGTTATCGAGAGATGAAAATGAAGGCTAGGGTTGGGGTGGGGGCGCACCTAGGCTGTTGGAAGACATAGTTGACGATGTAGGGTGTTGTGATGGGTAAGGGTGGTCACGTCGAGTATGTTTGGGTTCTGAGGTCTGCGAGCAGGATGCTGGCTTTCTTATATGCGCGTAGAAACTTGTCGCTCCTTCATGATGTCGTCCGAAGATTGGCTTGGAACGTGAGGGGTGGATGGCGCATCGAGGAGTCCGTTGCTCGATGCGCGTCCCACTGAGGAGCCCTGGAGGCGTAGACGCA

>AT6177

CGCGGCCGGGCCCGCTCGCGTCGAGCACCGTTGTGTCGTCGGCACGGATGATCAAGGTGCTATCGGAGGTGTATGTCAAGTTCTTCTGCAGCGCTGTCGCCTGGTCGACGTAATTTCTACAGGATCGTGGGTTGAATCCATTCAGCCTAGGTGGGCGTGGTAAAGAAGCAAGAGGAGCTGAACGTACACTCGGCCATTTGTCGGGTCGGCAATAGCCATGTGCTGGAAACCGCTGAGGAATTGGCTGCCTACATAGCTCTCGGCCTGAGAGTAGGTCGCGCCGAGGGCGGAGGTCGTAAGGGAGGCGAAAGCGAGAAGAGCGCGTGCGGAGAACATGTTGCTCGAGTCCAGTAGTGAGAAGAGGACCACGACGATCGAACGAGGAAGCCTCAACGGGGAATAGCTCACGAAGAAACCATGTCATGGTCCCCGATATATATGCGATCTGCTCAAGTCAAGCTAGGGGAAAACATAACGAGTGTCGCTTCGGCCGCAGCGAGCCTCGGATGCGGTCGCACATGTAGCACGCGGGACAGAAGAAATCCATAGGTCGTTGCGATGGGGAGAATGCAACACAACAGAACTCGTGATGGAAAGAAGACAGCCAGTCGGATGCACATGCACATGCGCACACATGCACTCCCACCTGAGC

>AT6183

GGAGAAGACGGCAATAGCATAGTAAAGCTGAGGGTGGAGAGCAAGCCTAGCAATGGAGCTATTCACGAGCTGGTCGAGCCAGAGGGCAGTCGCTGTTAGCGTGAAAAAGAGCGAAAGTTCGGTGAACATGCAGTTAATCAAGAAGAACTGCCAGGCATTCTGATTAGCCTTTGCCCAGCTTCCGAGGGCACCGAGAGACCGACGCACCTTGTATAGACGCATGACGCCGGGCGAGGGCATGACGCGACGGATGGTGGAGGTGCTATAGCCCTTGACTATGTGCCAGGCCAGGAAGGACGTGGAGACCAGAGCGGTGACGTTCAGCACGAGCTCGGGAATCTGAAGTGCGACACGAGTCGTGAAGTACTGCGGAAGGACATCGAAGTGGCACGGGGTGGGCGCGTTCACGATCAGCATGTTGTACCGGAACTGGACGTCATACGTCCGCCATAGCGTGTAACTCGCCCATCCCGTTTGGAGCACGCGAGTAAACGCGATCGCCC

>AT6185

TCTGCCAGCGGCCGCTGGGACCCTAAGGGTTGCAAGCAAGTGGTCCTATATAACTATCTAACTGAAGGTGTTGGACGTGAGCAGGAATTCTCTTGTCATCCTTCGCATATGTGAGCACAGCAAGTCTAGATATACAATATCGACGTCCTTCTAGCGGAGACTTGTCCTCAGCTATGTTCCGGAAGATCGAATCCGGCGGTATGCAACAGCGTTGCAAACTGCGATCATGCCCAGCTATTCAGCAAAACGCCGTGACCGCGAGTGCAAATACTTGGAGCGCTTACCTGAACCTTCGCAATGGACTTGATCTGATCAAAGTCATACCCGTTCCGATCACTCAAGTCGCCTGTAT

>AT6207

TATCCGTAGATGGGCCACGTCAGCGTCGACGGATTCGACCACACGGGCGGAGTACATACTGTCGCTCGCATTGAAGCCCTCAGCAGGAGAGCCTGTAGGGCATGTATCGAACTCGCACGGCTGAGACAATGGCTGAGGTGCCGCCGAAGAAGAGCCCGTCCCGGATTCGTTCTCTGCCGGGGCAGACAGAACAACCTATTTTCGCAATAAGCAAATCTGATAAATGGAGACCAAGATAAATACACGTACATCGAAGCCCGCATCCACGAGTGCATCGCGTTGAGCACGGATCTGCGCGACCGCCCATCCATCGTCATTGGTCAGGACGAGGTTCTGGGCGAAGGCGCCTCGGTAAAGTAGCGCGACCGAGATGGCAGTAGCAAGGGATGCCCAGACCTGCATCTTGAAGTCAAAATTGAACCTTTTTAAAGGGTTGATAGTGGATGCGGCAGGCCGCGTCGAGGCGTTGAGGGGAGAAGGCGGGCTTTCAGCTCTATTTAACTGAATGATGACGGCACTGGCGAACCTCGCACGTTCGTCCGGTCTCCTTTTACCACGCCTGGAAGCCATGTGGGTAGAAGTAGGGTGAGAGAAGAGTGTAACTAATGAGGCATGCGGAACAGCTTTCCATAGGACCGCGAAGGAGAGGAAGGCT

>AT6212

ATCCCGACCAGGACGCCGAAGAGCACACAGAGGATGGCGAAATCGATCCTCTGGCGTACAGTCATGTATGAATAGTCGTCGCGAGACACCCTCCCCAACATCGCGCGGACGAGAGATGATAGTGACGGACGCTTGTGGGTAGCGGCAAACGCTATTTCTCGCGGTGAGCACCCAAAAGCCATGTAGCTGACGAAGTGAAACGCACTAGGAGGAGTGGGCGGCGCTTCGCCAACCAAGAACACTACAGGGGCCGCAGCTGTGAAAATTATCGCCATGACGAGTATCTAGTCTCGCGTAGTGCTGATAAGAGGCGCAATTAGCTGTCCTACGGCATTCCCGACAGGGTTTGCTATGTGGGACAGAAAGAGGAACCTCAGCTGAGGCAAAACGATTAAAAGGATCGATGACCTACAGAGACTCATGATCATCGTTGCGGTGGTCCGGCTCCTGAGGTCAAACCATCGTTCCGAATAGCTCGGGACAGCGACCTGCAATATCGGCAGCGACAGGGCTGCCAGTAGCTGAACAACGCTCAGAAACCACCGACAGAGAGTCTCGGCACGGAATAAGTACTCACCTGGCCAACCATGATTAACGCGTAAGAACCTTGGATGCTGAGGCCATGTGTGCTGCCGGCAAACCTGACCCAGGCGGATACCACGAAGAGCGACCCAGCAATATAGCACTAGGACGACTGAGAACGAGTCGAACCGAGCGCACACATGGGGGTGCTCACCGCTTTCTGGACTCCTAATCTCCCATACAAGAAGGGCATGGCGAAAGCGGAGGGGATGTAGACGACGTTGACAATATTTCCGAACCAGTTGACTTGGTCCAAAGTGAAGCCGAATTCCAACACGACTTGCGAGACGAGGAAGCCTCAGCGAGTTATCCCATACGCTAAGAGTACAAGAACG

>AT6213

TCCATACCCCTTCCGACGGGACCTAGCGTTGCATGCGAGAGCCATGACCCCGGCTTCGGCATGTACATAGCAAGAAGACTTGTCTAACTTCCTGGCTGCAAGGTCTCGAACCCACTTCAAGGCTTCTTCACAGGCCTCCGACGGCAAGCGACGCTGGATCTCGTCGACAACATATTGTGTGGCCTGATCGCCTGCAGAGTCTGGTGTGTGCGGAGGTTTCAGGAGGGAGACGACGGTCACCTTCGCAGACACCAGTAGACGCAGGGTGCCCGGCGAAGAGACACTCAGGATAGCGAGCGCCGGTCGCCTGAGCATGCGTAGGTAGCGCAAGGCGTAATCGCTGCCTGGCTTGCAGTCGGCCCGAGTT

>AT6239

CCCGCGACGTTGCCTCTCAAAGCCTATTTAATGATTCAAATTCAGAAGGAATTAATAGTACGCAGGTCAGTTAGAAGGACACGTACATGATTGGTACAGTAAGAAGCGTAAAGACGCCTGTCGTGATGCCCATGTTGGAGTAGACGACGAAGGTGGTCGATTTCACGATGTTGTTCTCGGCGGCCTTGCTTTGCGCGCTGAGCGCGAATGTGACAAGCCCGAAGATGGTCTCCAGGACAGCCCAAGGCCCCACGACGCGTATGTGCACCATAGTAACAAGCGC

>AT6245

GGTGGCCACCTCGGTATATTTCATTTTCTACTCTAATCCGGTATATGTATACATGTCATCGGCCCGCTGTATAAGCAGTACCCTGTTACTAAACTGTTCTACCTCGGAGGACTTACGAGACACGTTCCACCGGAAGGTCAACGTCGAGGACAAATTCCTGAAGACAGTACAAGCATTAGTATGCGCGTCGAAGATCGACACTCACCGGTTTC

>AT6253

GCGCCGAGGTTAACGAAAACGAAACCACTGGTGAGACGCGCAAGTTGGCGCGTCGCGAGACAGTTCTATAACCTGAGGTTCATCCCCAAAACGTGTACATATATGGGGCTGAAGACTGACCAAAATCCCGTGGAGAACCAGTAGCCCTGCAAACAGCCCGACGCTCTTGCCCGCCGTGACCTCGTAGGTCTCACCTCTGCCGACGACGACTGCGGCCCAGATCATGTTCGCGAGCCCGAACTCGGTCGAGGACAAGCCGAGAATCTGTCCAAGGATGTTCAGCCAGCCGACGACCCATCCGACGATAGCGCGCTGTTTACGGGGCACGAGCTGCGCGGATGC

>AT6256

TGAGTGGCATTGAATCTGGGCAGGCTATGTGAGGAGGGGCAAGCGTCTCAGGGCGGAGAATAGCTTTAATCTAGCACTTGATGTATAATAGATAGAACGGGGGTTTAGAGCAATATATTGACCAAGAAGTCTGTACATATCCTGTGAGAATCGATACCAAAGTCCGCGCATAACCTGCAGGAAATTTTCATCAGCGACAGACGATAGCAGCTGAGTATGGTGTTGGAACCTAGACTATCATTGCGCCTCCGCTCGGGTCTGGATAGCGTCCGCCATCGCTTCAAAACTCTGCATGAGCGGCGTAGAGATGAACCACTTAATGAGGTATGCGCCAAACCCAGCGAAGACCTCCCGCGATTCATAGAACGTCTTCCCGTCCGCTGTCGTGCTCAACGCTTGCCATCTCTCTGCCTTGACGATCCATTGAGGCAGGAGGCTCTGCCACGCCATACGATGCTCGTCATCATCATAGGCGATGATTAATTCGAAGGCGTCCTGTAGCCTTGCAGAATCGTCCAATGTCGGCGGGATGTGCGTCTTCATGAGCAGGTATTTGCCCGCGGCCGGCGTTTGATCTGTGAGTACCGTCTTGCCAGTCTGGTCGGTGATCTGCTGGCTCCGGCTGTGTACATAGCAAAGTCGGTTTCTATGCTTAGTCA

>AT6257

CCGGGTACGTCGTGAAATCCGAGAGGATCTCCCAAACGGTCTCTATGGGCGCATCGATCTCCCGCGAGACATAACAGGTGAGCGGGCCTGTGTAGGAAGGGTCCGGGAGGTTGGTCGGAGGCGAGGTGGACATGATCGACACGGCGCTTGGGAAACGAAACCGAATTAAAGGCTTGGCAGGGATCCTGCGGGAGAGCGTACGAGGTCGTCTTCTACCGCAACGTTACGCAATTCGATGAAAGTCCTTATGCAGTGGGAGAGCCCGAGCAGAGAACGGGAGCACGGTTGTTGGGATGAGGCAGCCGCTG

>AT6259

CCGGGGGGTATGACTGAATACATGTAGATGATACTGACGCAAGTTGCCCCAATTGCTCATCCCCGTCAGGACGATGATCCCTTGCCAGAAATTTCGAGGGATGTAGCAAAGCTCACGTATGATGATCCCCATAGCGAAGCCCATTGCCTGGTAGAGGAATCCCATCATGATCAATGGTCCCAGGGCCGAAGCGTTCGCGGGCGTGAATGAAGGGACGATGTTCGCGAAGATAAGAGCGGGGAGAGCGATGTTCATGCTAATGATGGATACTCCGCGGGACGCCGCTGGAGGAAACATGTCCAGCTTGACTAGGACGAAGCCGCATACAAGGGCGGCGAACCTGTAAAGGGTGAAAACAAGCTACGAGGAACCACTGAAAGGTTCAAGACCACGTACGTTTTGATGAGAGGCATCACGC

>AT6261

CGCTGTCGAGTGGGGCATCGATATCGACACTGACAACATACTCGATCATGCCTGGAAAGACCGGACGGCGTTCTAGTTCTTGGAGGGTGAGATGAATGGCATGAACCGGGTGCCATGCTTATAATCACCATCACTTACACACGCCCGACGGGCCGTAGGCACGGAGTGGTGCGTGAGATTAGAACAGAAGGGTGAGCGAATATTCATATCCAGGTCTTGACACGCGGGGAAGAGCCCGATGGCTCGGAGAAGAATGCTTGGGAAGATCGCGCGTCGCTTAACGTGTCTCTACCGCCTTCCTCATCTTCGCCAAAGTCAAGAAAGGCGTAAAGGGACACACAGGTATCTCAAGCATTCATCTCCCCGAACCATCGCGCCCACGTACGATGCCCGTGTAAGCACATTTGAGTCACCGCGCCATGCATTCCGCAGGAGGTAGTAGAGCTCGCTGGACCTTGCCA

>AT6275

TGGGCAAAGATGAGGTACATGCACGCACCTACTCGAACGATGTCCCCATATTTATCGTGTAGGGCCGCGAACCATCGAGTCTGCTTCCCCCGAAGTGCGATAGTCGCCAGCCAGAGTTTGGAGATGCGGCACCAGAACGGTCCGGGATAAGACGCGAGGGGATGGACCGGAGAGATGCGATAGAGGACGACGGACGTGACTAGGGTGGAAAGGTACGTTCCATAGCACCGGAAAAGAGTCCCAGATGAGGATGGAGGGTGTCCTGACCGAAAGAAAGTGGAACAGAAAACGAGCGCAGGGGGAACAGAAAGCAGGATAAAGTGGACGAGCGTGGAATACGACTCATGGGTGCGGAAAATCTGGTGCGCGACCTAGAGGAACTCGTGTAAGCACAGGCTGGTTTGGGAGGAGGCCAAGATGCTAATGGGGCAAGGGACACACCAAGGCAAGCAGCGCTGAAGCTAGGACGGGTTGGAGACCTGTAGAGAAGGTTGTCGGAAGTTGCGAGAACGACATGTCAGACCAAAGGGACAACTGCTCGCTAAGAGTGGTAGGCTATTCAAGGGCCGCATGATGCAGACGTAGTACTAGTATATATGCTGTGAACGAGATGGTCACTGGTGGAGCGCCGAACACAAACTCCAGTCGATGCGCTC

>AT6281

AAGGCGCATGATATGTAATGTATTAAACTGCTGTACGTACAAGTCCTATGTCGAGCATTAAGATACTGACTACGCTTGGTTCATCCCTGGTAGGGCCGTATGAGCATGGGCAGCTGAGAGCCCTTATCCAACTCGTTCCGCACAGTCGGCCGTTGTACTAACAGGCCCATGGGGAGTATATCCTCGTGAGGCACAGCCAGTTCGAACTCGAACGTGCGCACCAGCGCGAACAGCAGCGCCTTCAGCCTGAAGGGGGTATTGTCCGAGACATCAGCATAGATGCGCCGCCTGAGAGGAGAAAACGCATATACGCACTCTGTGACGGAGAACCTGTATCCGATGCACGCGTGCGACCCTCCGAGGAACGTAAGAAGGTGTCCCCACACTCCAGGGACACTGGAGATCGCCTCAGGTGGGTCGTCCCATCGCTCGGGCCTT

>AT6283

GACTTTTCCCCATAATGCGAGCTGCCTCCTTGCGCCCATCTATGGGCTTGCTGTATTTCGTGGAGTGCAATATGACATGATTGATCGCACGAGGGTCCGCGGTCCACAAGCACGGCAGCTGAGCACGACACAATTGTAAGCAGTGGTAGAGCCCTGAGACTCGGACACGGAATAGGCTACGAACTAGCAAAAATATGCGGGCCAGGAAGGTAGAGCCATAGTCCTCCATCCACTGTTGAGTGATGAGTGGATCGGCGACCTTGCCGAGCTGTTGGAAATTGCCGTATAATATGCTCGCGCTGGGCGGTCCTCTGAGAGACAAGAGAGGCGTCACGTAAAGAGGAACGAGCTTCTTGCGGACGAACCAGGCGACGGCGATGAGCCCGCAAACCCAGTATGGAGAAACGCTATTCGTGTAACCCATCCTGAACGGGGGACAGAAGGCGTTTGGAGGGAGGAGGGCAAGGGGGGTGCAGTCGACGGTCGTCCCTGTGCTGCTGCAGCTGTAACAGAGAAGGCGTGGAAAGCCGATTAAGTTCACACGGCCGCCGAGCTCTGGAGCGGGAGAAGCCTGATGGAGAGTTGGGTAAGTTCCCGGTATCGAGAGCCAGGGCGGAGGTAGCAGGCAGAGACAATCT

>AT6291

TAGAGTTGGAAGTACCGGACTGTCTGTTGCAAGAGCACTCCCTCGAGACTACGATTGAATGGTTAATTTCAGCAACTGGTGAGGATACAAGTCGACATACAGAGTATTCAGCCAAGTGCTAACAAGCCATGACCCTATACCAGTGAAGAAAGCCGATGGAATAAGGCTAGTAGCCGCTTCTATGCTTGTGAGAATCATTGTGGTCGCCA

>AT6295

CACGAGAACGCGAGAAGCGCCTCAAGGGCGGGCACACGAATCTCGTGTCGCGACACACATCCCAGCACGGATGGGACGATTGCGGAAATGGTACCTTCTTCTCCCGAGGCTAGCGTATCACTAATGATAGCAAGGGATTGCAACGCCGATCGCGCAAGGGGTGGGTTCCGAACAGTAGAGATAATGCGTTGTATGATCTCCACAATCTTGACTACAGATGAAGAAAGAGCGGCACGTGCGCTTTCAGTCACCTGTTTAAGCCGCTCTCCAAAGAGCTTGAAGGCACCAGCTTGAGCCTGTATAACAGATCTCAGATGCTTGCATGCTGCACAACAGACCTCATTGCCTTACCTGAGGAGACCCGGACTCGAGTATCGAAAGGATGCCAGCGACGAAATCCGGCGCCGACATTACACCAAGCGCGCTCGCCATCGCGGAGCGGGCGGCGTCTCCGATCTCAACGAAGGATGTGTCTTCCGGTTTAGCGGTTGAGGTCGCCAGCAACAACGCTAGAAGCTTTTGACCGGTCTCCCGCTCCTTGTCCGATTTGGCGACAGAAGTGGTGCGAAGGCGCTTGAGAGCATGATCGCAGAAAACTAGCAGAGCCGCCGCCCGCCTCTTGGCTGCCTGATCTGCGTGGGCTTGCACA

>AT6296

AGATAATTGCAAGTCCGCGGAATATCGTTCGTGAACGGTCAGAAGGAGGAACAATGAACCGCTCGATTCCGCGACACTCTGCCGCACGACTCGGTTCGACACTCGGTCGACGAGAAGCATTGCCGTTGGCGCGAGGAAATCGGCCGGCCCTAGAGTGTCAACAAGATGTGAAAAGAAGTTGACTCGGCGATGGCGAGGAACATGGTTGGCAGCGTTAGTGAAGACGCGCAGGAACTCCCTCGACGCATGGTACAAGTTCATGCCCGACCCGTGCTGCTCCTTCAGCGATGCTACCATGACAGGGACGATGCTGTCGATAGTCTGAACAAAATGAGATATTGTCAACAAAACTAACCATACAGAAGTACACAGATATACTAACCTTCTGCACGACACGGAAACTGTAGGTGTCATCGCGGTGGAAGACGTTGGAACCCATGAAGGTGAAGATTGGCATGACGTTGTGGAGAACGGCATCAGGAGCTATACGGGCCAGGGTCGCCATGAGAAGGCAAGCTTGGTGGAAGCTTTGAGGATTATCC

>AT6314

GGGGCGGACGTGTGAGGGAGTGCATTCTGGGCGTCCAGTGTGGTGTTGGGGTGTGTAGGCTTCCGGTTGGCATTGGTGCGGGGATTTGTGGACGGGCTGATGGTGTTATAAAGATCTGAAGGGTGTCTGTGTGCAGGATGGGAGGCGGGGTGTTTGCGTCTGGGAGAGGTAGATGGGCTTGGGGAGGGATGAGGGGAGGTGGTGCGGGATGGGGATTCACCGTTGACGCCACGGGCGAGAAGGAGATCAAGAAGGGGGTTGTGTTCTGCTGGCTTGAAGATGCTATGTGGTGGATAGGTGAGGAAAGAGAGAGGGAGAGGGAGAGGGAGAGGAGGAACTGACCCTCCACGATCGCGGTAGCGAGCCTGTTGACGTTGGATGCGGTGTGCCCTGCTTGCCTGGACGGTACGTGGGGGAAGAGACATTGTGTGGCCAGTGGCTGGCGAGGACTCGGAGATGGTCGCGGTGCTGCCAGCAGTGGTCCTGGTCT

>AT6318

GCACAGCGCTTCTTCCCTTTGATGCTTGCCCACATTGCCCCAGACCCCTCTATGAGGGCCTCGCATCGGGCATTGAATGTCTTCAACGACCTATCCTCATGCTTGCTCCAGTGCGGCACCAGGCCCCACCGCATGGTTTGCATAAGCACGGACATCGGCACACCTCTAAGCTGCTCTGACACGTCTTCAGCTTGTGCAGGATCCGTCTTCTTCTGAGAGGCATCCTCCGTTGCTAAGCGGCTAGTTGAAGGACCCGGTTGTCCGGATGAGCTGCTGGAGGAGCTACTTCCTTCGTCATTCTGTCGCCGTATGACCGGTGCCTGCGAACGAGGGGCT

>AT6333

CCGGCTAAAAAGCTCCAACTCCGCAACGCTGGTCATAGCGTCATGAATGCTGGTCAACCCAACAGCGACCGCATCCTTGAACGCACGCTCTGCGTATTTGCGCATCTGCCCCGTCGACCACTTGGGCACCGGCACGAGACTTTGTGCGGCGTCGACGAAAATGCCTGTCGGTTCTCCCGAGGAGTCGCGCAGTATTTCGCCCCCCGGGACGGTCTCCGGGTAGCTTCCGCCAAGGTGTACTTTGGTGAGATCGAGTGCGCGGGGTGAGACCCAGACTGCGTGTCCATCTACTCGGTGCAGCACGATTGGTAAAGCGGCTAAAGCCGGACGAGACGCAATATCGGCCTATGTTTTTGTTAGACCAGCAAGTAACCTAGCATGGCAGAAAGGGACATACAGCGGTGGGGAAGGCGCCGTTTTTATCAGACCAACGGGTATGATCCCAGCCCATAGCTTCGATCCAGACGTCGTGGTTGGACGAGTGCTTGCCTACGTAGTCCTCTAGGGTATCTAGGAGCTCGGAGAGGGACCGGGCTTTGTCTAGAGGCAACTGCATTTTAAATCCGTACTGGAGAATGTGGGCATGGGCATCTAGACACGAGTTAGTGTACATGTCTAATACTTTACGGGCAATAGGTAGTGAAGATACCTGTGAGGCCGGGAAGGACGATTGAGCCCGGACGGACATTGATGACGGGCAGTGGTTTCTTCGCGGAGGGTTCACCACCGTACCATTTGTGAACGAGTTCGTTCTGATAATTGTCCCACCAAGACTGAACCTCAGCTGTTCTCTGTTAGCAATCGAGAGCAAAGATTACGTCGTAAATGACTTACGTAGCGTGCCCGTAGTAAGAATTGTCTGTTTGTCAACGACAAAGCAGTCGACGGAGGGTTTCAAGGGATCAGCAGTATAGATCTTTCCGGCTTCTCCGCAAAGGGCATAAGAAGCTGGGAGGACGGTAAAGGAGGTTGGGGTGTGGATGAATGAGTAGTACCACGTGGCGACGCCAGCAGCGGCCGAGGCAACAAGAGTGTATACAAATTGCTTGTTTACTGAGCTGGCATCCGAGCGAGGCCCTCTGGTTGGAGGAGGTCGACCACCTTTGTTGGTTGTGCTCATCGCGTCGGTCGTAGGCCTACTAGCTATTAGTATAGGTTGAAAGTTGTCTGTGAGTGCATTCA

>AT6340

TTACAGAAAAGGACCATGGTCCGCACTCGGGCAGGGTCGACGGACGCGTAGCACATCAATAATCGTGTGTTCTCCAACGCGAGCCTGTTCTCAAATCCAATGTCACAGGCGATGCCGAAAGGCAGAGCAGATGAGGGGTCGAGGTTGAGCTTGACGATGGGTATTCGAGCATGCGGGAGCGGCTTGACACGAAACTTGGTCTC

>AT6341

GTCCGAGAGGGCGGAGAGTTGGACACACCAGAGTTCTTGAGGCTGAAGCCGTTGGCTGAGCTGCCGAAGGAGAGGAGCCTGCTATCGGGCTCGATTGTGCGGATGAGGCGTTCAAGGAGCTTGCGAACGTCCTCCTTGACGGCCATCTCCTCTGTGGTTGGGAGTAGCTGGACGACGAACTCGAAGAGGCATTGTGAGAAGTCCTGGATGAAGCGTTGACGCGAAACGAGTCTCCGTTGTTGTTGTGGGGGCGAATTGGGAGTAAGGTATTGCTGACGAGAGGTGGACTGGGCGGCGGCAAGTGGGGATGGGGCGAAGGTTGTTGCCATAGGTGAGGACACTGCGTAGGTGCAGAGGACGATCCGACCAGCAGACTGTGGAGAGATGAGGAGTCGTGAACGATAAGCCGGTGCGCGTACGTTGCTTGGGTGGTCGTTGGAGGCCTGGGGGATGAGGATGGGTGGGGAGGATGGCAGAGGTGAGAGACGGGCCTTGTCCTGCGTTCAGGTGTTGTTGAACCCG

>AT6350

GGGCCGCCGGGGGTGATGGTCAGGGTCAGGGTCGTCGTGACCGTCTCCACGCCTCCGGACGTAGGCGGCAGGACCTGGCGTGGCGGCAGCGGACCACCAGCAGCGCCTGTAAACGTGGGGTGAACTTCGGTGACCATAGGATAGGCCAATGTCGGTACGAACCGAAAGTGGCGAGAGCGGCGACGATGAGGGCGACGGCGAGCTTGTGCATCATCGTGGCTTTTCTTGAATGGGAGTACAGGTCGGAGAAGGGTTGCAGGACGAGTCTGAACCTGCAGTCTCCTCTCCCC

>AT6356

ACTCCTTGTATGCGCGCGCCATGTCCCAAGGACGGTGCACCATCCTCTTCTCAGGAGGCAGGAACTGGTTCAGGTACAGTACACATTGCGTGTACTCCTCTAAGAGCTTCGACTCGCGTGGCTGCGGTTCCCGGCTCTTGATCAAGTTCAGGATTGTGATAGGCGTCCCATACCGTTTGAAGAGGTCGTCGAAGTGGCGAGCGGCTGACGTGTAGAACGGGTCTATAACCGGG

>AT6364

GCCCTTCTGTCCGAAGCTGTTAAAGAATATCGACGCCATTGGCTGTCCGATGGGGCTGTTCAGAATGCCGTTGACGTCGGTGCCCATACAAAATGCCAGAACCATGTTGATAGCTGTGAGCATGCACAATTTCAGATTTGGCGGCCAGCCAATTCGAGCGGCGTTCCGTACCCCATCCGAGTACACCTGCAATGCTAATGGCATAGATAATCGCCCACGGGACTGCCGTCGCCGCGTTGGATGCCTCTTCACTGATGTGCACACTCGAGTCGAAGGAGC

>AT6374

ACGGGCCTCGAGGGGGGGGGGACAGTCTTCTTGGTCCGGCGCACTTCGGACTGTGGAGTTGTCATGGTACGGTGATCTGGTGGCACGAAGGTGTTGTTATTGATACAATACAGTGCATTCCCTAACCAAGCCCTGTGTGGAGGACAAAACAGATACAACAGTGGAAAATTGGAGTGATATCGCAACAGACGCGTGGAGATATTGGGGCGTTTCGCGCATGCTGGGCTGGTGTGGTCGACTGGGTCGGCCGCACAAAAGTCGTCGCTTGGCCGGCTTGGCCATGGACCACGGGCTTGTCCTCTTAGACAAACCCCACGTTCGACATCGGATCACAACCCTAGTTGTTCACGGATAGATTGTTTGACCATCCGCTCATTTACAGCCCGGTTGTCGCGGTAGAGTTTACAACAGTCGATGTTAGCGCCGCTTTCCAAGTTCGGCTCTGCGAAGGATGTGGCCCGGTGTGCGGTGCACGCAAGAATATAATTTGGTTGCGATACCACATCTTCTCAGAACAGATCCCAGGCACAGTTCCGTGAGTAGCGGGCGTACCTGCTAGCATTGAGATCACGCTCAATATGACTTTCTCCACACTCTGCACAGGGCTCCAGCGCTCGGATGCTTGCTCGTACATTGTGGGGTCGTCTCCCGGCGTGTGCAAGATGGATATGCAAACTGTACCGTCTGCGTAGACTGTAAA

>AT6375

CGAACGTCAGGATAGCAGGGAAGACACCACCCTCAAAAGGAGTGTCCTTCGGGCCTACGATCAAGGCCTCCCAGGTGAAGAAGTCAGACTCGGAGATTGGACCTTCCGGGCGGGAGTGGTTGGTGGAAGTGTGATGAAGGTCAGCGCTGGACCGGTGGGTATGTTTGGGACGAGGTTGCCGTACCTGCCGTGAACATGCCATCCGGTGCGCCTGTGGACACAAAATTTGTATAGAGAAGGTAAGCCATTGTGGGCGAACGTTCGGGTAAGGTCGAGAAGATGTCTCGCCACACCGACCTTGGGAGGTGAGTTGCTTGTACTCGGTCATGAGACGGCGCAAAGCGGTTGCGCTTCCACCCCGTGAGTTCATAATGGATCGGCCTTAGACACAGAGACTGGAGATGATGGCGAGAGACCGTAAAGGCAGCGAGGACGAATGTAAGCGGGAACGAGATGGGCGTCGAGGATGAGCAGCGCACTCCGCCTGTG

>AT6382

GTCGTCGCTACATTATTAATTTATTATACAATCGTTGCTGTCGTATACGTGGAACAAGACCTGATACTACAAAGCATGGACACAGAGCATGAAGTGTTTAAGGGTAAGTAGTGGGCGGGCGTGTCTAACACCAATGCCCATTTTACGGTGAGTCGCCGCTGCCGCGCGCATCCGCCTCGACCTCCTCCAACCCCGCTGTATCTGCGAGTCGACGCGCCATGATGGTTCGATAGCTCTCGTATGATCGCCATTCAGCGCCTAATGTCTCCTCGCTCTGTTCGTTGCCATGTGTGGTCAGAACACGGCACTACAAGCATAGAGTGGGACCGATACTTACCAGGTCAAGTGCGGCCAACGCAGAAAGCACCGGCACGAGCCTGCGCTGCGCCTCTGCGTGGAACACGTCTGGGCGCGGCGACGACGCTAGGCGCATCGTGTGCGCAATCGGGCCTCGCAGGTCCGGATTGCTGATGTGCTTGCCCTCCATGTAGAACGCGAGGTGCCGCCACAGCAACAGGAGCAGCATCTCCAGCGAGTCTGCAGCCGCCGCCACCGTCAGTGTGGGCAACTGAAAACGAAGCGAGCACGACTAGGACGACTTACTCAGGATGGTCAGCGAACGCCGCTTGGCGTTGGCCCGCCACGACGCGAGCTCGCGGCCGACGAGGGTCTGCTTTTGTCGCATGTCGAGGTTCTCGAGGAACGTGGGGTCTGAAATGTGCATGATCTGACAATATGGGCAATCGGTGATCGGTTGTTAGACAAGCGGATAAGCAGGTCAGCGTTGTGCTGCTTCGGTTGCTCGACGAAAATACAGACCTCCTGAACGTTGTCCACCCGGATGTGGTCCTTGGACGCGAGCTCGGCAGAGAGGCCCACGATGCGCATCAGTACCTGTGAGAGGTCGCGAGAGAGGTCGTCGAGTGCTTCAATCGCATCGCCGATACTAGGCACAGTCGCTGTTGGCGGAAACCGTTCAGCTTTCTTAGACATTGTTGCCCTGAAAATAAGTGGTGCTTACCAATGAATTTGGAGTGTCTCTCCTCATGCTTGGGCGTCGTGAGCGCCGGCGAGAACACAAGCGTGAAGTCGGCCTCCGCGAAGTCGCTCGCAGTGCCGAGATATGCCACGATCGCGTTCTTGAGGAGCTCATCCTTAAGGAGCAACGCCGCACGGAATTTGGATTGAGGGGCGTATTCTGTAACCATACGGCCGTCAGCCACATGGCACGTTGAATTCGGATGGAGAACGCACCCAGGGATTGAACACTAGCATCAGCCAATTCTGCATCGGTCTGGGGCTTGACGATCTCCGTCCAGTGGCGGTCGGGTAGGCACTTCGCACCCAGACTTGTGATGGCCCCATGCAAGGCACCAAAGCCCGAACTCGAAACCTGGGAATGGCATTCGTCAATGTGATATTAAAAGTAAAATGGGAACGAGATTACCAGCTCAGTCTTCGGAACGAGGTGAACTACGCTAGCACATATCGAAACTAAGAGACGGATTTCTTCCAACATTGAGATGGAGAGCTCGTCCACTTCGTTTTTCAAGAGCAGAATGGCTGTGTCCCGATGACTTGACAAGAATTGTAGAGC

>AT6391

TCCTCATCCGCCACCTCCGTCGACTCCGCAATGTCATATAGGCCAAGATTATGCCGACGAGCGCGACCAAGCCTCCCGCAACGCCCCCTGCGATTGCTGGGACGGAAACGCGTGTCTTCGAAACACCGCTCGCGGCTGAGGTCGACACAACTGAGGAGGGGGACTGAGACTGGGAGGACGGCCCAGGGTTAGTTGAGCTTGAAGTCTCCGTTTGCGATGCTCCGCGCGCAGTGTTGTTGTACTCGATCCAGTCCAGGGCGAATAGATGGTCCTCTCCGGCGTCTACCACGTCGATGACGAGGGTGTGCGTGCCGGCGGGCAGGACGCCAGACTGGAAGAGGCGAATGCCTACGCCCACCCCTTGCGCCGTGCGACTATAGACGATCTTCCCGTCGATGCTGTACGTCGACTTCGCGACCGTGTTGAACTCCGGTCCGTAGACTGCCACTTGCTGGCCTGGAAACGGCGGATAGTGAAGACATGGGTGGACGGATAGTGAGGCATGGATATACTGACCTAGGAAATCAAGTTCTGCAGTTCCTCCGGGAGCGTTGCAGAGGGTGATACCTTTCAATCCCTCCCTGGGGTAGAGGGAGAGGGTCGCGACAGATGCGTTGTATTGAGGTACCCAAGCGTCAACGTTGCCTCCGTATATGATATTCGTGTATACCATATCGTCCATATGGTTGTCGCTACAAGTCCAACAGGGATCGGGCATGAAATTGAACTGTCTAGGCACTGGTCAGAGGGGGGGGGTAAAGCAAGGAGAGCGTAGCATACCTGGTTGCGAGTAGATACGCTAGAGGGAATCGTGGGCGAGTGAGTGCGGGGAGCAGGGCTTGAGGTACCAGACACGTCCGGGCGTTATGGCAGCAGCGGCAAAGATTCCTAGTTCCTTAGAACTTAGAAGAAGCGGCTGTGGGACATACATATGCCGCGCCCACAAGCTGTACAGGAGAGCGCTCGGATGTGCTTCGTTTTGATGCAGCGCCGGAGAAGAACGCGTCGCTGTTGCGAGCAGATGGTCCACATCTACGCCTTTGTGACCTTGCCCCTAGAAGCCCCTGGGTTTCATGCGATGGCGTGTATAGCAACAGTACTGTACCGCCGTAACAGCCGAACCAGAAGTGCGCATTCGAGGTTCGGATCGTGGGTATGAGGATGGCCCAGAGCTGGACCGAGGTCATCCGAAGAGGGGGAGAGGGATGAGAGTTGGATGAGGGAGGGTGCTGCCCGAGCCGCACTAGGACGGTCTGGACCTGCAGGCGCGTTGAAGTAGTGAAGGCCACACGATGCTGGATTGCTAGAAGTGCCAAGCTAGGCAAGAGGTGATTCTGTCGGCTATGCTCACTGGTCGCCGGTGAAGTTCTAGAAGAGTCCCCTGCTCGACGGTCAAGTGTGATTAGAAGCGATGCGAAAGGAAGGACGGCCAGTACATACTTACGTAGGTCGAGTCTCGGCGTTCCTGAATTTTACATAGCTCGGTCGCGGACCCGGGGTGCAATCTGACCCGGGATGCGTATTGGGCTCCAGAAGCACAAAGCCCTACTCTTGGTC

>AT6392

GTGGGGGTGAACACTTGGTATTTACTGGTAGGTGCACGATCCGTACAATTTCGTCAAGTTTGTTTCTGAGTTCATAGTTGCGTTGTAGTACTGAGTAGAGACGGTAGAAACGGCGACTCTTTCAGCAAAGACAACCTTGGTCCGACGTTGGCTGCGGCGACGGCTGCAAGAAGTTTCGAGGCATCGCTGTTCACCAAAGGCGTTTCAGCAACCAAAATGAGGCACTCCCTTACGGAAATGAGCGCATATATGAGGTTCATAAGCTCACCTTTTCCCTATCCGGATGACTAATGTCGCGCCGTCGTCCGAAAGCCAGAGAATGTCTACGTAGGTGTGAGATGATACTAGGCCGAAAGATTGCCCGAGAGCATCTTGAATACTCTTTCGGATAGTTAGAGGGTCGGTACAAGGCGGGGAAATGCCAATACGGATGTAGTGGTGGGCTGAGGACAGACGACGAGGCATTTAATCTCGGAGAGGGCAGATCCGCAGCGGTCTATAAGAAGGTAAATCTCAGCACTGCGAGCATCCACATGGAGAACTCAATTGTTGTCTTACGAACAACGGGTGAAATAAATGAGCCCGAAAGACTGCGACGGCGGCGTACTTGAGGAAAAGAATACAAACGGGGGCCGTTGAGAGGAGTCGGCGGGGTGAAGAGATGAAGGTCGAGAGGGAAGGAGTATAAGTCTGCAGCGGCG

>AT6402

CCCCGCATGCCCATCGTGAAGACGCCCTCATACGGCCGCGCGCGTTCCGCGCCGGCCTTCCAGAAGTCATACACATTCTGCTGGTTCGTGGAGTAGTCCCATGGTCCTTGTCCGAAGATGTCCCATTCCGGGGGTAGCGAGCGCATCATCGGTTCTTCGTGGCTCGTGCCCATCACAATCCCGTACCAGTCCGCGAGCGGTTGGTTCTGGCCATCGTCGACACAGAAAG

>AT6405

CTCCGTCTTATAGTTGTTAGCAATGAGACAGGCTTGCGACGCGCGCAGGTTGTTTAGGCCAGCTGCGTAGTACATGTTCTGGAGAGTCAGTGTCGCCTGGATAGGGTGATGGACGAGCTGGAAGAAGGCCCCCTTCGTCTCCGAAGGAAGGGCATTGTACAACTTGGTTGACGCATCACTTGTAGCCTGGAGCATAGCAAGCACGTTATCTGCCCTGGAAAATGCGCCACGATTGTCAGATACAACTATTCGTTGAGATACAAGACATCGGCCAGGTTACGCACTCTCTGTAGTTGACCAAACTATACGTGGTCGAGTTCACGAGCTCGGGCTTGCGCCTCGCATTGTGCCGTGTGACATTGTGGACAATGTTCGCGACCACTGCGGCATTGGTGGGCGACAAGTCGAACTCGCGCTGCGC

>AT6407

ACCCGGGTCATCATTCAAGCCATTTCGCTAGTGCTTCGGCGGCGGTGGCGGAGGCGCGTCTGTGTCCGCCTCGAAGAGAACCGTTGGCTCAGCGGCGGGGTCCGTTCCCGTCCGAGGCGGAGAGGCGGCTTTCCGCAGACGTTCTGCGGTGATCGCTAGCGGTTCTGAATCCCCGACTCGTTCGTTGTCATCATCGTAGTCGTCATCCAGGAACTCGCGCGCCGACGGTCCGTCGTCGGTGGAGAGGCGTATGCGGCCCTCGCTTTGATTCCAAGTTTGTAGTCTG

>AT6426

GCGGGCGAGTCTGCGAGGTGCACAGTCGGAAACTGCTAGCAGATATGGCTCAGGTCCGACTCTAAGGTTATTCGTGACGAGGAAGCATGAGAAGGCCCGGGAGTTGGCTGAGGTGGCGGGATGGACAACATATCCCGGAAGACTTGAGAGTGCTTCGCGAGCGGGCCGCGGTAGACGCGAAACTCGACGTCCTTGGCAATGAGGATGATCGTCCCGTCGTCGTACCAGAATTCCGCGTCCTTTACGGTCTCTAGAGATTGGGATCGTGCGCGGTTCCTGGTAGAGATGCGAGCCGGCTTCATAGCAGTACGCTTTGTGCGAGCCATATTGTGCTGCATGGAGTTGTCAGGTCAAGCAGGGTTGCACGACGGGAGACGGTTTGAGACCGTG

>AT6428

GGGCGGCGAGGACGAAGTTGAGAAGGTGCATCATCTTTGCTTTGGTGCTAAAGGATGAGTGCGATTGGCTGTTGTCTGAGGACTGTGCACAAGAAAGTAGAAGTGGAGGGAACTGGGGAACAAGAGCGACGGTGGAGGGGGTTTTATACGTATCAGTGCAGACTGGATTTTCGAGGGTCGCCGTGAGTTGGGGCGAAACAGTGTACCATCCACCTGCGCAGCAGATTTAGCCGCCG

>AT6771

GGACTGAGCAACCTGGATGGTAGAAATAAGACTATGCTTGTTGAACGCAGAAGTGGCGAACACAATGTAGTTGGAGGTGGTGGTGCCGTCTAGTGAATAGACGTAGGACGCCAAGCCGATTCTATAGCCACAAGGCGGGCTGGGGTGAGCGCAAGCATTCTGGACAGGTAGGCAGGAGAGGAACAAGGACGCACCCTGTGAAGAGAAACCACTTTGAGTAGCGGCCGTATACCTTTTCCGCAGCCTCGACGGCCTTAACACCGAGATGGGCGTCCTCCTCAGAGTGGAGCACTTCGACTATCTCCTTGTCGAGTCTGGGAATCGTGTCTTCTGGAACATCACCGTGGATAAGTCCCGGGTTGGAAGCCTCCTCGTTGTTGTATGACATAGTTCTGAGCGAAAGAGGGCCCGAGCCGTTCCGCCAGGATTCGGGGGAGGGCTAAGAACCGGGTGCTCCAGGCGAAGAGGAACGTCGAGATGGGGGGAGACGCAGGAAGGAGGACGGGGGTGATAATTACGATTAGGTCGCATTATATACAAAGCGTCAAGCAAGATGGAGT

>AT6773

GCGCGGAGCGGATGGCGTCCTAACGATTATCGAATGCAAAGGCTACCGTAATGTACATGTCCGTATAATGATAAAATGGCGTGCAGATATCTACGTAGTGTGTCCAAGATCCTTCAGTATCCAGACGGTCGCGCACCCGTCTTCGATGTATCGTAAGCTCGTATCAATCGCGCCTGGTCTTGCCGCGACGCATTTTCCGTCTGACTGAAGGCAAAGCCGCGGTTTCGCCTCATACGCTGAACAGCGCGAACTTTCTTGATCGCCTTCTGGAATCTGGT

>AT6774

TACTATTTCCATACGAAATCCCGCGTCAAGCAAATGATTGGGACCAATATAAGAACGAAGTAGAAAACGGCGTCGGTCCACAATCGGGGAACGATGCCACGGTACTCTGTTGAGAATCCGATGGCAGGGGCAACAACCGCGTACAGTGGAAGGAACGCCATGGTGAAGACGAAGGAACCGGGGATAGCTATGTGGATAATAA

>AT6784

GTGTCAATGGCATCGATGACAGGTTTTGTGCGGAGCTTGTTGACGATGAGGACGGAGCGGGGGGAGATGCCATTGGTGACGCGTCTACCTTGTGCAGCGGCATTCAGATAGGTGGGGTTTAGAGAAGCGAGTGTTGAAGGTAAGAGAAAGAGAGAAGGGAAAGTATGGATCGGATGGACGGGTTACGGACAGAGGGTCGAAGAGGTCGAGTAGGGAATGAAAAGATGGGAACAAGCTAAAGGGACGTACGTTGCGTATTGTCGGTATCCCTGAAAAGTATCTTTCCTCTGCGAGGAGACGTTTGCTTCTTGAGGTAAGGGGGTTAGCCTTTGAGGCGAAGGGGAGTGGAGGTGGCGGCAAGCTCGGAGGCGAAGGGACGACGTTCTGGCTGCGACAAGGGCAGGAAAGAGTGTTGGCGGCGGCCGCCACATCGGGCCACAGAGATTGAATGTTGGAGAGGACGAAATGGAGAGTACTCGGGCTCAGAGGCAGGGTTGATTGTAAACAAGAACGGTGGAGAAACGAGACTTGTGTCGAGGGGATGAAGGCAGGATGTGATCGGGGGGGAGGATCCGGACGAAGTTACATCAAGTTGTGAAAGGATAGAATGGTGAAAGGTATCACCGGAGGGCTGGCATAGAGTGTGGAGGTAGGATGGGAGTCGGCGACGGAGGATGGTGGACGAGCGAATGAGCAAGCTCAAGGAAGGCACGGCGTTCGCTTGGGCTGGTCGGCCTTTCGGACCAACTTGAAGCTGCCAGTCCGAGTCCGACTGGTCCTCCCACCGTCGAGGTCACTAGCTCAGAAATGCTCGGGAATACCTCGCGTAGCATACAAACTGGAGTCGAAGAGATCCACTGGCGAGTAGTGACACACGCGAGTGTAGGGATTAT

>AT6787

TACGCTAAGTTAGTTGCTATCTTACACGAGACACGAAGTAGTTTGCATGCATAATTCAACACCCTTCCGACCCAACGATGATACCACCCTGCTTCATGTTCCATCCCAGCTGCTCATACGTCCACCCGTGAATCTTCGCATCAAGGCTGACGAGGTGGAACTTGAGGAGAGACCGGTGCATCCCGGGTTTCTGGTGAATACCGTCGATCGCGGCCACATCGTCGTTCGTGAGCTTAATCAACTATGACATTTCTGCTGTCAGCGAGAGGAACGGCACGGCTGGGCGGCAAAGTGCGTAGCGTACGGTTATGTTTTGCTTCATCCGCTCCGGATTTTCGCTCTTCGGAATGACGACTGTACCGCGCTGTATTCCCCAACTAAGCGCAACTTGAGCCGGAGTCGCACCATGGGCCTGCGCGATCCTGAGCATGTCTGGATCCTCCAGGAAGACGGGCT

>AT6792

GTGTGGTCAGCAGGCTTGTCGGGGTAGAAACGGCTCATGGGTTGTGACAGACCTGCAGTTTTGTTGTTGATGACCATGATAGTGATATACTCGGCCATGACAGGATCTTGTGTGAGGTGGGAGAGGTCAGCCATGACAGAGCGACTCAGGCGACTCGGATACGTACCTGGTTCCATGCTGTACTGCCGCTTCATGAGCTCATCCTGGATCGCGTTCTGGAGTGCTGTGGCACGCTCGGTGCCCATCGTGAGGCCGAAAGCCATGACTGTGGATAGAGAAAAGGCAAGTGGTCGTGGTCGAGAGTCGAGACAGAGCAGGGAGACTCTCG

>AT6820

CGGCATGGTGACGATGGAGTACTTGTGTGGCGTGTCGATGATGGCCACGGGAGGTAGAACACCGGGGAAGGTACGAAGATCGGTGAATAACGACTTGTGCTGTAGGAGCGTCCGGAAGATGCGATATTGCGAGCTGTCCTTGTCGACAAGCTTCAACATGATATCTCGCCCCGTAACGTCTTGGCCGCACGCCAGTCTAGTCTA

>AT6823

TGCACGAAATAGTTTGTCCCTGAGTAATTTGTCGGCGCACATCTTGGACCAAATAAAACAGATGCTGAGGGGCGGGCGAGGGTCTTCGATTGCGAGGGCGGTGGTGAAGTGGGCGTTCAAGCGTTGGTTGATCTCGTCGAAGCGGGCATGCACCTTCTTTAACCCGCTTCGCGTGTTGAGATCTAACGTGGTGCAGTACGTATGAGTGGCGTGCCCGATATGAAGTTGAACACATACCCGGAATATCGAGATAGTTGCACATGCGTTGAACCAGAACTTCGAACGGGTCTGGTGTGTTCCGATCGATGTTGTAACCGGTAAACACAGAATCTGGAACGATTCCGAATTCCTCTATGGGAATGTCTATAAAGCGAATATTTGGACGTAGGTCCATTCTTGAGAGAGGGAGAGGTTCACGGGGATGGAATGCTGGGCGGTGGCTCGGCGCAGTTAGGTTTTGACTGCCACTGAGCGCACGGAAGAATACAGGTGAGAAACAAGTGTACG

>AT6833

CCTCATATTCGACATGCAACTCACTTTGGCATGGTTACAATGGAGTACTTGTGTGGCGTGTCGATGATGGCGATGGGAGGTAGAATGCCTGGGAAGGTACTAAGGTTGGTGAACAGCGATTGGTGCTGTAGGAGAGTCTGGAAGATGCGATATTGCGAGCTGTCATTGTCGACAAGCTTCAACATTATATCTCGCCCCGCAAGATCTTGGCCACACGCTAGTCTAGTCTACCAAGAGAGATTAGGATGAATCAGTAGCCGTGGCACCGAGTCCAGCACACACCAGTGTCAGAAATGTTGTCGATGAGATCTCTTCGTACGATATGCAACGAGCGTACGGGAGCCCAGCCGGGGTGTTCGTTAATGGCGTATGCCAGTATCTAGAAATAGCGTTCTCGTTCTTGGGACGCAGGCCGTATAGCTCAACACCACGTAGAAGAAGCCACTCCCTAGATGTCTCCCAGAACTCAAGCATCTCGTCTATTGTGGGAAAGTTGTCCTTGCCAAAGTCGTCGATAGTGCACGGTATCTGAAACGCCGAGTATTAGATATAAGTCATACTTAGTAGTACCGCTGTTTTCGCCCACTTACTTCCGGGATGGCTGGGGACGTCTGCGTGTCTTCCTTGAGGATAGCCTCGCTCATCGTTGCAAGATGTGTAACTTCTGGTGGGAGTGCGCCGTCATGGCAGTCCAAGCTCAACCGAGATCACC

>AT6843

GTCGCCGGTGTGTCGTCTCTTGCATTGGTACTATCCCGAAGTCGTTCTACAAGGTGAGATCGGAGTTTATCTCTCTCGAACAGACACTGTCGCCGATCAATCGTTGAACGTCAACGATTATGCCGTCAGCCTGGGGGCCAAGCCACGATGCAGGCGGCAGGATCAGGAATCAGGATGCTGGGGCGCGTCCTCGCTTTGGAAATCGTCGTCGACCCACATGAGCCGGAGTTTGTCATGAATATCATATCTGTTGAGGGGGATCCTGCCGACTTCCATCGACCAGCGTTGAAACGCTGGGGCAGTCAGTTCAACATCCTGAGTGAAGCCTGAGGATTCGTCAAAGTACTTGTGGATGAACAAACCCACAGACTCGCCTGGGAGGTTGTGCTTGAAGAAGCGGATGGCATAGTAGATAAGGATGTGGAAGAAGCTCTCGAGCTCGTCTGCGACGCCGATCGGATTGGAAAAAAAGTCGAGAGCGCGGGCAGCTGAGAACTGCCGGGTACCCTT

>AT6848

ACCTCCGTGCGCATACAAGACGCAGCGCATCTCGTCCATCTCCGGCTGATAGGTTGGAGGCATCTCCTGACTTCCATTCTCCGTATCCTGCGGCCCTGGCGAAGATGGATTCATCGACCTCTCCCGCGCTTTCTGCTTCCTCTTCGCTTCTTGATAGTCCTTCTTCGCCACGATCCACTCCGCATCCACGCTATGCCCGGCCATGCATCTGAGTGACACTCGCGAAGAGGATCGGTATCAACACTAACCCTTTGACGCCGCGTACTTGCCACCATTTCGTACCGCCCACAACGCGTTTCGTAACCTCCTCGCCACCGAGCGCTTTGATGAGATGCGCCGCGGCATCGTCGCAGCATGACATTGGCACAATGAGTCGGACGACGTGCACCCATGGAGGACTTGGCGTGCGGGTGTTGGAGAAGGCTTGTAGTTCCTCTACGGTGTGC

>AT6849

CAAAAGCTCCTCCCGCCTCAGCCTTGTCGACTCCTCGGACTCGTGGTCCTTCGCGATGCGATCGAAGTAGTGCTTGAGGAGAGTCTCGAAGATGATGGGCCCCAGCGAGAGGCCGGTCTTGCGGGTGAGCGCGTTCACCATGAGGCCGTTCCGGTTCGGGGTCGAGTCTCGCGACCTGGAGGACGTTGAGTATAAGTTGCCGACGGTCCATGCCCAGTCAAAACGGCCTCGAGAACCACCGAGATCTGTGGAGAAGAGGATCAGCGCCTGATTTTCTCGACAGATTTCGTGGATTTGGGTGAAACTGGGACAAACTCGCACGTTGCTTCGACAGCACTCCAGATATCTTCACGGCGTGTTCT

>AT6850

GGCCCGCATGCCACCGCGCGCGCGAACAAACTTAAGATACCAGCTATCCAGCACGCCCCTTTCCCGTCCTCTGATTAAGATCTAGTTAGAATTGCAACCTTGCAGCGACAAACTGCAAACACAAACAACTCACCTCACGGTTCGCCTGGGTGACCTCGTCCTGGTACACTTGACTCCATGTCAGCATGAACGCCTCCGGTGGTGCCAGACAAACGAAACAAACCTTCCGCTGTGCGGGCTGGAGCGCAGCGAACCAGCACTCCGCAAAAGAGACATTACGGTTTCCTTGATTTGGTCACACACGTGAAAACTCCTGGGGGAAGATGACACTTTGAGAAAGCGCTGCCGCATCGCAGAAGGTGGACACTCACTTGACGCTGACAGAGTTCGGCTTATACTTGTCCGCCGCCTTGACAGAACCACGGCGTCCTTGCTGTGACCTACCCGTAGTAGTAGCTGTCTCGCCGGGAGCGATGCCGCCATCGATGGGGTGTCCGTGGTGAATTCGTAGTGGTAGCGATGGCCTGGGACACGATAGCCGTGCATCCGAAGGTAGGGATGTAGTGGCAGTCTGGGATGGTTCGGACACCATTGGTGCTCCGCTCGCGGGTTGCGTGGGTTTTGTCATTGGGGCGATACTTTGAAGAGTAACAGCGCTGCGTGCCAACGAAAATTCAGTGCATGCACTGAAAATGGGGGAGTGGTTGGCACTGTGTGCCGAAGCCTTGGGTTTAACTACGTAGGAGGGTGATGGAGGGGTGAAAAAGGCTGAATGGTATGACTTTACGTACCAGAGCCTTGTTCCATCGTTCTTGGAGTGAAACGGGTGTCTCCTCCACACACGAGTCTCCGGAGGCCATATTGTGGGAAATAAGCTCTACTGCCATGCGTCCGAAGTGAATGCTTGTATCCTGCGGGACCGAAATGTGCGCTGAGGGAGGAGGCTGGGGCGACAAAGGCGGGATAGGAGGAGACGCACCAACCGCACTCATAGTGGCGACAATAGCCACATCGATATCAGCCTCCTCATCACCTCTGATGGTGAAAAGGTTGACGTCAGGATGGAGCAGGGGGTGCGAAGATGAACTGGGGGAGAGAGCAGACGAGGGCTGTGGAGAAGGGGATGAAGACGACGTTTCCTGGGACGACTGCTTGTCAGAAGTTTGTTTAAGCGCCGTCGCATGAGTGCTCTCTGGGCGAGCGCGCTTGGTTGTCTTCGTTCCCTGCAGTGCGAAAATGCAAACTACAGTACATGTCATTATCTGGTCAGATTGCGGGCAATCTGGACGACGGAGCAACAGACAGAGGGCTTCATTGATGGTGGTTGTGCGGAGAGCATATATGAAGCGCCCAACCCCAGTTAGATTTCGAGGGATTCAGACCGTTCTCGACTGATTTTAGGCGCTCAATGCCCGATATCCACCATTATCCATCTATCTCGACAGCGTTTTGACTGGGGTCCATGCTTGGATCTGTGCGACCGACCACACACATTTTGGCAGGAAGCTTGGACTAGACTTGAATAGGCAGCCAAACAGGTCCTGAGTCGCAGGCGATGTTGAATTTAATCACATTCAGATTGGGACCGAGGTGGAATGACAGATCAACTGACACTCACCACAGCCTTCCATGAGTGCTTGGCTCGAAGTTGGGCGATCGCTCGACTGGAAGCCTCGTCGATGCGGTGAGCAGGTGGTCCTGGTGGTCCTTAGTTACTAGATGAGGGCGGTGGGATATGTCGGTGATGGAGGCCGAAGGAGCGATAGCGCGGAGACAGTGCAGATACTCGTAAGCATCTCGGAGACGACGTCAACGCCAACCTTGTTATCAAGTACGTACACCCTGCGCTCTCCCACTATGTCCTTCTTGTCGCCCCCGTCGCTCGAGTCTATTCATCCAGCCTCGGGCACCTCGAAGAACGTGTGCGAGCATGAAGTTGATGGCAGAAGCTAAGTTTGTCGA

>AT6852

CGCGCTTCTGGCCGACGCGCACGCCGTTCTCCTGCAAGGGACGTGGGTCGCGGGGGGTCAGCCGCCACCGTGTTTGGTGCTTAACCACAGCCACTGGCACCCGGCCGATGCTCACCCTCGCGACGGCCTTCAGCTGGTCCCAGGTCAGTTCGCGCAGCGTCGTCTCGTCGAACCCGGCCATTGCGTGCCCAACAATAGCACGATGGGTGGAGGATGGGAGGATGTAGAGGGGAGGGGAAGGTGGTGATAGTGGTGGTGGCGGAAGAACAAGTAGCGCTTAGCGCTTGAC

>AT6875

GCAGCGAGAAAAAAAAGATAAGACAACAAGGGGACAAGGAGCGTGTGATTAGCGATACAAAGGGAGATTCCATAGCGAGAGCAGTTGCAGTCCGCATAGAGAAGCTTGCGGTACCAGAGCGGTACATTCAGAATTACAAGATGCAAGGTCAGAGGAAAGAGAAGGAAAGATCGCGATTACAAGCCCGCGAGAGCCTGAGATCTATTTGAGAGCGGCAGCAATACTGTACCAGGCGAGGGCCGGATGTGCATACATATGCATCGACAACGCGAGTAGACGGCGAGCCGAGTTGGCCTACCACAAATAGCCCTGCAGTTTGGCATAGGCGACACCTGGCTGCGGCTCCGTCGGAGGCTTCTTCTGAACCTTCACGCGAGCGGCCCCAGCGCTATGTGTGGGATGCCCACCGGGCGTGACCGGCATGCCCGTTGCGGGCGCGGTCGAGGCGGACGTCGACAATCCTGGCGTGTGAGGCGCAAGGGTCCCGCCCGGATGAGCCACGATCGGCGTAAGAGGGCTGACGGCCCGACGCTGGGGCTGGGGGACCCACCGTGACGCGGACGCGTGGGTCTCGGCGTAGTTGGGGTAGGGGCCGCGTCCCGCGAAGTCGCGGCGCACGCGCCTCTCGAACCCCCGGACCGCGGCAGCGTCGACCTCGACGTAGTTGTCGAGGAAGCGCCGCAGGTCGCGCTCGCCGCCCTCCACCTGGTCGCGGTGGAAGAGCCCGTTGCC

>AT6876

CGGGGAGTGAAAGGCGGCCGCGTCGGAAAAGGGGTTGACGTTCGGGTAGTTCATGTTGAATGTGCCGGACTCAGCAGAGTAGTGGTATGGCTCCCGATTCCTTCGATGGTTTGAGAATGACGACGATCTGCCGCAGCTTCAAGCTTAAATACGTGATACTACGAGGCAGTGAAGCGTGCATGGTGACGCATGGCAGAACAAGCTCGGAACCTATAAGACAAAACTTCAGCAAGCTACTGATACGGCGGAAGTTTCCGCTACTCCGCAAGTGCCTTGACTTTTGAGACAACTTCTAATGGTTGTCGCGTTCGAAAAGGCGGGGAGTACAACGAAGGCCGTGTAGCTAGGGATATCTACTGGC

>AT6886

CCCCCGTCGCACATCAGGATCCCAGCGGTTAAGCTCGTAGTGGCAACTGGTCATGCACCTGTACAGACACACGGGCGGCGCCTCTGCATTACTCGTATGGAGGAAATAAGACACTTCGGTATGGTAAGGCGTAGCAATCGTTCTATGTCGTAGCAGGCGCGTATCGGTCGCCTACGCGCATCCCACCATCGGGTCAGAGTCGCCGCACACACATTACGGGGGAAGGACGCACCTGGACCACACAAGACACGAGACAGTTCACAATCTATCCAACACGGTCCCAGGGTTCTCGCCACGCGGGATCTTTTCATCGCTGTCCGCCGTAGCGCGTCTTGCGGCCGATGTCGAATGGCACGTACTTGTACTTGATCATGTGCCTGC

>AT6901

AGCCCAAAGGAAAATATCCAAGTTCCCTAGATCCACAATACACACCTACAACTTCGACTTCGAACCAGTCACACCAGCCTCCTCTAACGTCTTGTACGCCAGGTGGCCCATATACGGGAACCCGTTCACGTACATGAACGCCCTCTCCGAGCTTGCCTGCCGCCGCGCATACCGCGCCGCAATGCCCTGCATGATTATAGCGCTCT

>AT6907

ACGGCTGCGAGCGGGTCACATGCAACTATAGTATGCAACAAGGAAACATTGGAAGTCCGAGTCCATGGGATCGTCGGCATAAAAAGAGAGTTGTACAATGGATGCAAGACTACTGTATGACAGAACGCTGCAGCAGGAGCCTGGGTATGGCGGATTAGCATGTTCTGGTGAAGGTGAATCTTACTGGGAATGGTCGATGGCAAGCCTGCTCTAAAACAATCCTAACATTGCGTTATGAAGGGATGGCAGATCGCCTTACCAATTACAATTACACTACAGCATGGATAATAGGGTTAGGAACCCCCAAAATGCTACCTCCGGATCTTTAGCCGTACATGGAGCTAACCAGTTGTCTCGACTGGAAATACTGGCACTACGCGGCATCAGTGCCTCCATTCGGCTATTGGATGGATAATAG

>AT6912

TGGGTGTCCAAGCTTGCCGAGAGCGGTGGCAGTCTGTGACAACATAAGCAACCCTACATCTTCTGCCGGACACGTCCTCACTGACAATATACAACGGCATCTTGGAGAAGTGGCCAAGGATCTTGGTTGACCAGTCCACATGCGTCACGTCCCTGAGGATACGTGGTCTCCACCAAATCCTGTTGGCATCAAGTGTTGGATGTAAGCAAAAAACAGCCACCATGAAGCGTGCTCTCTCGTATCTCACCGGTGGTACGCGACGTGGTTCTCGCGGTTCGTGATCTCGTCTTCGGCAGCAGAGCTGTAGTACGCCCACGCCTTCTCGGGCATGACCATCCTCGCGATAGCCTATATATGCGGAGAGCATCTGTAAGCACGGGACGAAAGGAGGCAGCATATACGCGCGGAAAAATGCACCTCGAAGTCATGCAGGTTCAGGATCTCCGAGAGCGGGGGGCGGTTCGCAACCCGCTCTAAACGTTCCTTTTCCACATCCGTGACCACGACCTCGACCTTTTGGACCGTCCCGGGGTCCACGGATCCTAGGC

>AT6931

AACGGCCTGTATCCGCGGGAGCAGAGGACCAGTAGTGCCATCATTCCAGCCAGAGAGGAGCATCGTGTACAACATCGAGGCGTACATGGCGTTCGCCTGCCGCCTCATCGCTCGCGACCTTGCGGTTGTGCCGGTTTCCTGCGTGGTGGGTGCTAGACTCTCCACGGATCGGAGTTCAACTTGAATTTGGTCTGGGTCATGCATGCTAGTAGGAGCTTCGGGCTGGCGTTCTCGCAAGGAGACTCCGAGGTCGATAGGCACGGGTTTGGGAACCACTTCCTCTAGTGGAAGTGCCTCGCTGGGCATCGTGAAACTAT

>AT6937

GGCGGGAATAGGTGGACCTAGGACGAGCACGATGTTCACCGCAGGCCTGAGAAACGGTTGTCGGGAGGTGGATGTAGCACAAAGCAATGCCAAAGTCACCCCTGCTCCGCGTCCTGAGCACGGGCCTTCTTGAGTGCTTGCTTAACGCTCTTGATAGAGTTCTCCCGCGCGGTGATAAGCCGCTTCGCCTTCGCGACATCAAGGGAGTCCTCTAGGGTAGCGTAAGGATTTGATGTAGGCTTCTTCGTTCCAGCGGATGTGCTCGACGCCTGAGTTGACTGTGATGCGGATTTTGCCACGGTGATCGACGCGAGCTCGGCGCGCAGCGACGTCAGGATATCGTCCAGGGACGTGAGGGTGGCGACGTACGTCGCCACGTCGTCCTTAAGCCGGAACGTCCGGCACACCTCGCGCGCG

>AT6968

TCTTGTTGATCTGCACTCGGCACGTCCAACATGTGCACCAGCTGCTGCAAGCACTCCGGCCAGTTCTTGGGCTCCAAGATGCCGAGGAACGCGACGATATCCTGCGCGGCCGCACCTCGGACCATGGCCGATGCATCGCTGAATGCGGTGAGGACGGCGGCCTTGACAAACGCCATAACGTCCGGCGCGGCTCGGAGAATGAGGCGTGCGTTGTTCTTGAGGAGATAGCCCGCAATGGTCCGGATCCGGTCATCTTCTTGTGGCATAGTAGAGAGGATGTAGGCTAGGTATGCGATGTAGTCGGGTGCACGCGTGAAGTTGTTCAATTTCTGGGAAATGAGAGTCGTAAATGAAGCTGCGCTGGGGTATGAATGGGGAGACGATGGGGACGTACGTGGGTGATGTTCCGCTGTACGGCGGAGTTGCGGGTGTCTGTGGACTCATGGATGGTCTGCAGGATTTCCTGCAATCCTGCTTGCTGGGGCGTCCAGGAGGTCATGGTCGGAGGAGGAGAAGATGAAGGGGCGGTCCCTGCGTTCTCAATCGTCTAACCCA

>AT6972

CCCGGGGAAAGCGAGGTATGTTTAATACTACGCATGTCATTGACGATCGCGATACCTCTATGTGAATTACATCATTACTTTTATGTGACCAGCACTGTGTCGCGACTCGAGCTCAACCGCATCCATCAAGGACCACAAGAGCTGCTAACTCGCGGGGAGGGGATTTTGTTTTGTGTTTTTACTTCAGCGTAGCGAAGCCGACAGCGTTGTGCTCAAAAGAGAACGCGGTGTAGACGTTCTTCATGAAGCTG

>AT6979

TGGTGGATAACAGGTCATGGCCTGACGATACGCCTGTTCCGAATCCGCTATCTGCGGTCAACGCGATGTACTCTTGCTGCGTACGGTGGCTTCGTAAGTGACGTGTGAGGAGGATCCAGATCTCTTCGTCCAGCTCTGTGGCGTTGGAAGGGTCCAGCCGCAGCTGTGCGCGGAAATGGGTCGCTGAAGAAACTAAATCAAGGAGCTGAAATGACTATCTGGTACTAGCCACTCACATGTGTCGCTGCCTTTCCCGCTCCGTTTCCACATGCTGATGGTACGGAGACTGAGGGCAAAACCAGCCTCGTGACAGAAAGAGGGACTCACCCCTGGAACGTCAACTTGTGCTTGAACATTTCGGGATTCCAACTGAAGTATATACCGTCGAACAAATGATAAACTGAATCAATGGGCACGTCGACCGAG

>AT7008

GCGTTGCGCGCACACGTAACATTTCGCATATCGTCTGACAATAACCGCCGTGGTACAAACCTGTAGTACTTAGAGCAGCACGTTGTGATATCAACAGCACCGCGATCGAGAAGACGTACACATAGCAGTGTACCTTAGGGTCAAGCGCCGCCCCATCGAGAGTCCCTTCCGGTCGGCAGCTTTGCACCCCGTCGCCCGCGAACGGCCAGAAACTCAGACAATAAACGCCACGCAATACATTATTTTCGGACACGACAGTGCGGTCATCATGCAGGTAGCTCGAACTTCTGTCCTTGCAACACCCTGTGAGCGTCGGCAAATTCGTCAGCATTTAGACCGGATATGCGAGCGTGAGACGAGGACACGCACTTCGTAAAGTAGACGTAGAAGAAGTCAAGGTACAGCGCCGTTTGGACGAGGCCCGCAGTGACAGAAATCACGTCGATCCTGTGCTCGGTCTCCGTAAAGT

>AT7016

CCAAGTTCTCAATGAATCCAGGGAACGTCCACAAAATCGGCGTGAACCAGAGCGTCAGCGATAAGCTGCCCAAACTCCCTGCCAAAAAGGTGTATGCTTCGCACTACGCCGCCACAGAAGATCAGCTTGCAAACACAAAACGCGACGGATCGGAAGACGCACCTTGAGGGGGTCCTCCCGTGTGAAGATGGTAACAAGTGGCATGTACGTGACCGCGATGATGGAGCCGACGATCCAAGTTCTGAAGTACAAGCTTCGGAACCAGTCCTGCTGTACCGAGCCAGAGTTGACGAGGAAGAGCCAAAAGCAGAGCTCTTCCAGATGTGTTACC

>AT7031

CCGTGCCAAATGCCGCATGAAGTCCAGCTACGGAGTCGCCCAAGCTGATGTTGGGACGCACTGGGGCGCCGGATAATGCACCAGTTTCGGGGTCTGGGGCGCCGTTGATATAGCGGAAGCCGGACTCTGCCTCACAGACCGAAGCGTAGCCCGGACGGGAAGCCCATGGCCCAGTCTGGCCGTATCCACTGACCCGCGTGAAAATCAGTGACGGATTGAGTGGATGAAGATCTGCGGGGCCGAGATTCCACTTCTCGAG

>AT7032

TACCTCCTGCCCTCCTCCTTGCGCATGTCGATGGTTACACTCTTCTTGTTCCGCGCGATGCTTCTCCACCAAGGGCTGACACCGTCGATGTCGAGCTCGCGCCACACACGCAACGGGTCTCCGACGTTGGGAGGCTCGACTTTGATCACTTCTGCGCCGTAATGGCTACGCAATTGACCGCAGAAAGGCCCCGCAATGACCTGTCCGACCTCCAAAACCCTCACCCCTGCGAGGGGTAGTGTATGGAGAGCGGCAGCCGAGTGGGTGCACCGTCGCAGGGTCCGTTTTGAGTGAATAAGTGCCCTTCCAGATGGACTAGGTCGCTGTGTGTTCAAATGTGGCGCTAAGAATATTGGCAGCGCATGCAGTGCGGCGCATTCGCGTCGTAAAATGTGATTGAAGGCAAAATGCCCGAGCACTATCATCTAATACGTAGTGAAGTCTCGTAGTGAGCTATTGGAACTTGAAGTTCGGACGCAACGAAGTGATGCGAGTGTGTCTGAATACCTACGATGACTTGTTGTGTCCGTTAGTTAGCCCTCAATTGTAGCATCCGAGTCTGTCCAGACCCCAAGGGTGTTGAGGAGTGGCGTCCAAGTGAAACCCGACATTAGTACCA

>AT7035

GCCCCGCGCACCTTATCAGTGAAGTCCCAGACGGTGACACCCACACACCCGCGCACGGCGTTGCACGCCCCGACGACGGTCGCGTAGTCAGCCTCCTGCTGCGCGAGGCCCGCCTCGGTCGGCGGGAGCGCCCCGAAGCGGATGTCGAGCTCGGTGATCGCGACCTCGATGCCGAGCGCGGTGAACTCCTCCATGTTCTGCTGTAGGGTGGCGGGCACCTGGCCGACGATGAGGTGGCTCTGGATGCCGACGCCGTCGATCGGCACGCCGGATGCCTTGAGCGACTTGACGAGGTTCTTGAGCGCCGTCGACTTCGCGCCTGACGTATTACGTTTGTGAGAAGGTCACGGGCGAAGGGGATTGAGGGGAATGAGGCGTACCGGTGCCTTCGATGCCAAAGTCGTTGATGTAGAGCTTGGTGTGGGGGTCCGCGCGGCGGGCAGCCTTGAGCGCGAGGGGGATGTAGGTCGTGTTCAACGTGTTGAAGAAGATATCCACGCGGTACGTCCCGTCGTCATTGAATGGTTCTGCCGGACAAGTCTTAGTCAAACTCGACTCCGACCTACGTGACCGGCATACCATTGATGACGTCCCAGGAGTCTGTAAGTCGCGAATGGTGCACTTAGCGAGGCTCCACTATTTTCACTCTCAGGAAGACGCCTACAAACTTGGCCACGATAGTGGCTTACGAGCGTGAAGCAGTGGCGTTCGACGATGTGCGCGAGCTCCGGAGCCGTGAAGGTACCATTGCTCAACCAGTCCGGGAGCTGGTTGTACCAAACGCAGTTGTGTCCGCGCAGCCTCTTGCCAGTCGTCTTCGTAAAGTCCGCGATTACGTCTCCGTCCGTGAAGTTGAAAACTCCCGGCTCAGGCTCAGTATCAAACTA

>AT7052

CATTCTCGACTTCGTTGGGTTTCGAAAGCCAGGTCCCAAGGCCAATCTGGGGAATCTCCTGGCCGGAAGAGAGCTTCACAGTCTTTCCGAAGGACATGGCGTTAGAAGACGGGCAGAGACGAGCGGTGCAAGCGGTGGGAGAGAAGTCTATCGGTCGACAGCTGGGATTTATACGCCGGGGGATATACAGTATCAGTGCCCTTCTTTGAACGATTCGGAAGATTGATATACGTGGGGAATCCTCGGGCCGCCGGTCCGTAGTGGCTATGAGGAACTACCGGGAGGCCGTGGAAGGAGCTGCTGACCGGGCGAGACTGTTTGAACACGGTTGTTCAGGCCG

>AT7006

CTGCCGTGCCGTGGACGATAAGCAACTGAGGAGCTTCGTGAGTTCCGTACCAAACGGACTCCGTGCTCGTACGCGATGTACCGGGAGTTTCTGTGGCCAACGACGGTACTCCCTCCACAATAGCTGTTCTCCCTATCATCGGAGGTCGTCAAATCACTCCACCGAGTTGCGCATAAAACGTTGACCCACCCCGCTGAGCATATCGTTCAAGCATCGTAATGAGCCCTTGTGAATAATACGAGGGTCTCTTGCGTTAGCTTCGTTCTTTGCAGGGTCATGAGAGAGATTCTGTGGCGCGGCCGCCTCAGCCTTCGTACTACGCCCGCACGGTGCATCGGCGCGTTCCAGACACTCACACTAGGGTTGATCGAGGTACGGACGACGTAGTTGGGCAGGACCAAGCTCAACAGCCACCCGAAGAAGCGCACATGTCTGGCAGTCGCCGTCGCGCGCAGGATGAACGGGCTCGATGCGACCACGCCAGCGAGTGACGCAACCGTCCCTTTCGAGGGCGGTGCCGACGCTCGGGTCGCAAATGCCAGCGCGAGCGCGCCACCCTGCCAATGCGGTAGTATCAGGATTC

>AT7354

CCCTCAAATCTCTTTCGTGTCCTACCAACATGTTCACGTGTCTAAATCTCAGTCATTCAACGCTATCCCTCCGCCCTCAGGCCCTCAGGCTCCGGTGCACAAGACATGCATTGTGGTGACTCCAGGTCCGCGCCCGAAAGAACGGCTTCATCCAGAGGCATACGGACAGCCTACGTGAACACCCGGACCCGGGAAGGCAGTACGTCCTAGAGACAGACGTAACAGCCAAGTCAGATAACCGCATCGCTTGTATATGAGCCACTGACTCATCCACTTGGACTTTGTACATACGGCGCCGGCGAGGGGACCGCTATACACCACTGTCGAAGGTCTGGATCGCGATGTCGGTCGTTCGAATCGCGACATCGGTAGTCACGGTGCGCATCCCAGTGTGGTCTCCGCGCGGCTGCGTCGCGAACGCGAGCTCGAAGCTCAGCCCTTCGCCAGGGCCCCTACCGTCCCC

>AT7361

TTGACGGTCAGTGCAGCCGTGCCCGCACACTCTTGAGCTTGGGTGTAGTTTTTGATAAGGTTCGGACTCGGAAACTCCAGGGCCGTATACGGAGGAAGACCCCACAGCGCATTCGCCGAGAAGAGAAAATAGTTGAGGATCGTGCTCTGCATGGACAGGATCGTGTACACCAAGTACACCGTTGTCGAGGTGAACAGGGCAAGGAGGGTAAGAGCGAGGACGATCGCTGGAACCCGGATTTTTCCACGGCCGTTACGTCTAG

>AT7377

ACCTTGCTGAGCGAACATCAGGAAGCCTTCGCTGCCCTCCCCCTCGGGAACGTCTAGATGTTTGGCCTCTATCCAGCCGAACAGCGCTATACGTTGCTCCAAGACTCGATCTCGCTCGAGATCATCCGTTGTGACGGGACGTGGTGGGACGACACGAGCAACCTGAGGGGTGAACGTACTACGGAAAGGTTAATACATGCTCCGTCACTGCTATCATGCCTACTCACAAGTCATACAGCCTGTTCATGACCAACTTCTCCATTCCCTCTATTGCATTGTCGAACTCCGTGTCTGATGCGTTCTTCCACACTTCGCATTCTCGCATTCGTAATGAGAT

>AT7422

CACCTCTTCCGAAGCGTCAAATCGGCCGTCCCCGGATGTGCAGCCAAGCTCTCCGCCGCCCCACCAACGACGATCGTGATCGCCTGCCCCGGCCCGCTTTTGAGGATGTTCGAGCAAGACTGCTTCGACACAGAACAGATGCCCATCGCGAGGATGATCTCGCGGTAAAAGGGTACCCGGAAGTTGGATGCGAGAGTGAGGAGGTGGGGGACGATGCCGGGGAACGCGGACGAGAAGCCTGTGC

>AT7432

CAATGCTCTCAGCTCCGGGAGATAGGAGAGTGGGTGGTCATACTAGAGGTGTAGCTGAATGTTCAAGCTAGCACGGCTATGCAATGTGGGTAGTTTCCGCTGAATGGCATGAAGCGTAGTCCTCTGATAGAGTCCAGCGTGTCTCCGCTACGCACAACTCGGAACTGCAAGCAATGTAAGTCCTTGAAGCTGTCGAGGGACAGCACTGTATCAAGGTGCTCCAAGCCGTTGGTCCTCATCGCTTTGACTTCGCCATCCTCTTTCATCAGCAGGTCTTTGCCCAC

>AT7464

CTACGATATCCACAGGGCCGTCGACGAACTTCTCCAGCGATGTGTGACGACGTTTGGTTCCCTTCCAAGACGGTACTATTACCGACGAGGGCGAGGAGCCCGCATTTCCAATAGGATCGCCCTCCTGAGCTACGCTAGTCTTATCCGCCTCGGAGGGATGCGCATTGTGGGCGGTCTTCTGCTTCTTTCGCGGAGGCATGCTGTAATGCGAGGGAGACAGAAGACAATGTGGCTAGAAGCAAAGGTTGGGACGTACCGAGACGCCGAAACACGGTGAGCTTTTCAAGGATGAGCTGAGCTACTTAGTCCTACTAGATCGGGGCCAGATCGAGCGAACAATAGAGCCCGGAGGCTGATCGTGAGAAGTCAGTACCCAGTGCAAAACATCGTCGCTATGACTCTGAATCTCAATTCTAAGCATTAGTTTAGTTGGCCTTCGTGACTTCGTGCACGACCAAATCTGCCTGCTCCCACCAAATAAGGAGCTCCGTACTAGCTCATGCGGGTGATGGAGGTTTGAGGCCGCCACAGTCCGTGTGC

>AT7466

GAACTGAACACTAAATGAGCGAACGCCGGTTCGCTGAAGAACTCGGGCTTCTCAGGCAAATCCTCAACCTGGCGTAATGACGATTTCCAGATCGAGATGCTGGTGGCGCGGTTGAGGAAGAACGAGCGAAAGGCTTTCGACGTCCTGGTGAGATTCAGAAGATCCTTCGGGTGCAGGTGCCCAAATATCTATCATAAGGAAGGGTTAGAACATCTTGCTCTACTAGACGCAACGACGGTGAGTACATGAGACGCTACGAACCTGCAGCGAGACATCGAGAGGCAGCGTTTGAACGTTCTCCAAGCTTCTCTTTCGTGACCGACGAGCAGAGCGTGAATTGGTCGTATGGTGATCCGTTGTCTTCAGATCGTCGTGAGGCGCATTCGTGTCATTACCGTCTGAGGGTTTCACCTTCTTTGCGGCCATGTCAACGAGAGTGGAGGACGAAGGTGATGAAGAGGTTGACGAAGGCTGACGAAAGGTTCGAGGCAGACTGAGACTGAGACCAGCACCAGCGGGGACCGGCAAAAACGATGCTCGCGCTGTCGTGGGTCTTGGCAAAGCCGGTGACGTCA

>AT7469

GTTCGGGCGGGCGCAATGCTAGGCAAGAAACGGGTCATAACTTCTTTCGAAAGCGGATAACAATCAAACAACTTACATGGCAATGAGTCGAGAAGAGTAAATGGATGAACGCCGGGATGCTCATCCAGGGTGGTCTTGCGGGCAAGTCTTCAGCGTTCTCCAACGCCGCCTTCCACAGTGGCTCGTTGCTTCGATGTAAGAAAAAGTCGTGGAACATCTTCGAGGTCCGGGATAGGTTGAAAAGATCTAGAGGGTGTAAGTTCCCGAAGATCTGAATGTAACGTGGTGAGAGATTGAATATGACAGGTGGTAGGCATGCATACCATAAGCTGAATTTCCACAGCAAAGTTGGGAATCTCCCTAAGGCAGCCTCTGCGCAACTTGATGACACTTGAGACCGCAGGCGCGACCTGTAGTTGATCATTGGACGTATCCGCCGTGACGGTGTTGACGGCTTTGTTGCTCGCTCGGGTCGTACGATGAGTACTAGTCGTTGAAAGTTCTTGGATACGGTCGGTCTTGGCCTTCTTGCGTGGAGGCATCGAGGAGAGGAGATAGTGGTGGTGAACGTGGTTGAAGAGAAGTGTCGAGGAGAAGCGCCGGGACATGTACGAATATAACGCGTCGCCGGATTAGGCGAACAATAAGTAACTCCCGCAAGACGATCATCGTACTGAAATTGATGCGATCTCGATT

>AT7497

GCGCGTCGAAGGCCAACACCAAGGCAGCGCAGGGGCTTCCGCTCCAGACGCTGCATGTGATGCTCGGCCGTGTTTCCGGTAGGCACCACCCGACCACAACTGCCGCGCAGAATGGACGGAGGCATCACTGGCGCGGGGCGGCACGCTCGGGAAAACAAAGTTTATAATCTGTCATGCCGTCGGTGGGGGAGGCGGAACAAGTCATGAGGAGACCCCATGAGAAGCTCAGGATACGGCTCAAAAGATGCGGCGGTCTGGACAAAGGGCGAACGCATAGCATATGAATGACAATAGGTGTACGTGGGAGGAGAACATTGATTGACCAAGATATCATGGGATGGCCCCGCTTTCGCATGCAAATGAAACGACGTGGTCCCACCGTGAATACCAAGAAGGGTACATTCAAAGGTAGAAACAGCGCTCCTTAAGGGCCCGCATGCCGAACAGTTCCTGCGAGAAGGGTGTCAACCGACGTCAGCACACAGATAAGGCTCCGAACATCATGGAGGAGGCGAGGAAGGAAATGAAAACCCAGATTGACACCGGAAAACGGGGTGACAGTTTACTCGCAACATAGCAGCCGGGCTGACCTCGTGGGCGAAAACAGGAGGCCTGGGCCCCAGAAACAAGATGATGGAGCAGAGATTGTGTCCATCTCACGAACGAAGCTCAAGGAAGTGCACGTACCCAGTCCGCCTTGGAGACCCAGAGGTCGGCAACTCCGTCCATCTTACCCAGCACCGCCGCACCCTTCCACGCGAGCACCCGCGGGTCAACGTCCTTGGGTGGAGGGATGATCTGGACCTTCTCCATGTTCGGCACGAGCGGTGTCGCAATAGCTTGAAG

>AT7509

TTCCCAGTGTGGGGTCTAAGACAGATATGGCCATCCCACATATCATTGACTTGCTAACCATAGACCCATTTTCCATGCATGCAATGCGGTGAAGGCTGTCCCTGCTGTGAAGCTTGGAGTGTGGCCTAGCGACACCATTCTGTTCGGCCGCCACCCTACGATGTTCCTCAGATGGTCCCACTTAGCTGTGGAAGGCGTCGCGTTCAGATACTATGTCATGAACCAGTACGTCGAAGACAAGGGCTTGAGCGGCTTGCAAGATGCGTCCGCCGCGAGCCGAGCAACGACAACCCGGCAGCGCATCAGATAGGCTCGTCAAACTGCGCGGGGACGTAGAATGTGAGCACGCCGCCCGCGTCAGAGACGTCCCCAATGTAGACATAAAGAATCATGCAGTGGTTCGCACATATGC

>AT7519

GCGACCTAAGACACGGGCGGCGTATGAACAAGTGGGACGTGCGTACCCATTTCACCAATCTTCAGGACGTCTATAGTGGGAAGGGATAAAATCACTAAGATATGCGGCCTGAGGCAAGGACTCACTGAAAAAGGTATCTCCTACAATCCGTCCGAAGAGGGTATGCTTGCCGTTCAGCTCGTCCGCACGGTCTGGACCGGGTGAACATCGCACACGGAGGGCGCGAATTGGCAACACACTCACTGAGCGTGATGAAGAACTGGGAATCATTCGAGTTCTTGGCACCGTTGTTCGCCATTCCGACTAGACCACGGTGCACGAAACGTAGGCGCGGATGGATCTCATCCTCGAAGGGTTCTGTACGAGTGCCATATGGTCAATGTTGAAAGAATGGAGCAGCGATATGTACCTCCAAAGAACGACTCCCCGCCCCCTCCTGTGCCCGTCCTA

>AT7548

GTCCGGCTAGTGCAGAGGATGCGAAAATACCGTAACTAAAAGGCGGCGTTGGTCAATCCCTTTATTATCATGTCTCCAAGCCGAGAAAAGGTACCAGTCAGCCAAGTACGTAAACACGGCGACGTAGAGGATGAACAGAGCCGTCATGATCACCTACCAGAAGATCAGACACGCGATGTAATTGAAAACGCGAGGAGGGCGACTCACAAAGATTCCCATGACCATCCCGATCCAAGGTGCCGACGGGGAGGCCGTCCAAGCGTAGATGAACATCCCCGCTGGGAAGAGTAACGCCGCAAGACATGCGCTGAAGAGACGAGCCTCTGGTCCTTTCTTAGAGTGATATTTTCT

>AT7549

GGGTGAACGAGATGTAAATCAGCGTCCGCAAGCTCGCGCGGTCGACCTCCACCCGCGCACGGTACTGCTTGTTCCCCGTCTCCTTGCGTATCTTCTTGGCCAGCCGCGTGAGTAGGACCACCGTTCGCGTCTCCCGCATGCAAATCAGCATGGCGACGGTGAGCACGCCCAGCAGGCTGACGCCCCAAAATTCAATTTAGCGCGATCGTCGCGCGCACAGTGAAAAAAAAAAAAAGAACGGGGCGCACATTACGTGAATCCACTGGATCCAACGCCACTCGAGATGGGGATTCTGTTCGATCCAGCCTGCAGAGAGACAGCCTATGCCGGGTCCGGCGAGCACCATCATTGCGAACACGGACATTGGGAGTCCACGCCTAC

>AT7553

GTCGATGAAGTCTATGCAATAGATGCAGATATGTACGGCTACAATGTACACTAGCCCTTGGAAAAGTCTGCGTGCGAGTGTCATATTATGATGCGTGGATCGGACAACTGCTGGAGCGATATGACATTGTTTAATATTGTACCCGGATCATTGACGGTGTGTATATGCGGTATGATCAAGGAGGAGCAAGGGGAATAACCACTAGGACACATATGGCATATCGGCGCGTCTATCATTGCTTGTGGATGACTTGTGAGGCAAACTTGCTGTGCGCACGGATGGATGGTCCTTTCCAGAGCAACACCTGAAAAACAAGTGGATTAGTGACGCGCCGAAAGCTGTGAAAGTATGATTCACGAAGGGGATGGGTATCATGACGACCGCAACGAAGCCAAAGAGCGAAGTACCCCAGTGGTATGTTAGGTGGTCATACATCTGCTGGGTGAACAGCGGGAACGCCGTTCC

>AT7570

GCCAGCGTTCGTGTAATATTATCTATCATTCCGTCGCAGTGTTTTCGTTTGGAGCAATGCATTACACAATCTAGTGTCGTCATGGCTAAGTTAGTTGGCCATACATGTTACGAATACAGAGAAGAGTGAACCAGAGAAAGCTATCGAAAACACGCAGAGCACAGACACGTAATCCAGAATGAGGCCGAAATACGGGAGGGCACTACGAGCCTCCACGATCAGCCCATATACGGATGGTCAAGTCCGTGTCCATAGACCCAGAAGCGATCATATTTTGTTGTGGATGGGTCTGTGGAAGGGCATGCTTAGTACGGGTAGTTGCATTAATAGACCAGGAGTAAACGCACTGCGACTGCAACCACAACATCGGTGTGGCCCTCGAGCGTTTGCACGATCTCCCTGCTCTGGAGGTCCCACAAGTACACCTTATTATCCTCGCTACCGGAAATTATCCACTTGCCTCCCGTGACGCTAAAGCATGCAGCGATGCAATACTTCTGATTCGTGTGTCCAATGTAGGTCTTCAGACAGCGGGACGTCTGGTAGTCCCATAACCGGATCGCGCTGTCGTGGGCGGTGGAGAGAATGTACTTCGAGTTGGGAGAAAATTGGACATGTTGGCTTTGCGGCCATCCGCTCAGTACAAGGCGTAGTAGGAGAACATAAGAGGC

>AT7574

GGGCCGTTCGGTGAGGTGGTTTGGTGCGAACACGTGCTATAAGCTGACAGAGAAAATGACAAGAAATGACGACTCATTCGAGAGAAACTACGACACGTAGAGTTTAACTTCGCTTGGTTGGACTTACATGGACCCCGTTTCCTCCTGTCGAGCACCTTTCATCCACTCCCTTTCAGAGGAAAGAGCCTGACCCCACTCTTTGACCTCCGGGTGTTTGAGGAGGGGTAGGCTGCTGTCAAAGAAGGGTTGCAACGTCGCCATCCCGTGGTTCTTCTCATACTCAAAGGCTGGATAGTGATCAGATTGCATCACCGGTTTGC

>AT7575

GCGCCTCCTTGCTGTCGCCCTCTTCATACACATTCCACAGATCCATGATCCGTTGAGCGGCTGGGCTGTTGTGCAACATCTCGTGGACGAAGTTGTGCATGGCTTCCTCTTCTAGTCGATGTTTCTCCGCCTTTGGGATGCCCTCTCGGGGCGCAATGTCTCCAACTAGCTCGAGTCAGCACTGCAAGCAGACACCGGGCGTGAGTCTCACCTTGCGCCTCTGCGAGATCGTGCACCAAGGCTATCATAACACATCTACGCTGAATGGGTTGACAGATATCTAGCTTTGGGGGAGAGACCCTTACTTGGAGAGGTCGAGACTAGGATCCGAAGTACACATGGCAAGAATCGCCATGCGGTAC

>AT7591

CAGTAGAGAGAGGTTTTAAAGTAGCATTCTGGGCGAGGTGGTTCACAAGGTGGGAGACTGGCTCGTCCCCCGGCAAGGCCGATATCAGAACATCCAACCCCACAGGCATGTAGCTTACTAGCTCGTGATGCACATCCTCTGGGTAAGACGAGGTGGGGATGAAATGAATAGCGGCTCGACATTCCTGAGAATACGATGCCAATTCGGAAGTAGGAACGCGGTGCGCAGGGCCCTCTAGGACGAGCAATACTACGTAGTGACCCTGCCGTACCAGCTCGCAGACCAAGGAAACAAGGTAGCCCGCCTCGGGAGCGTGAGCAACTAGAAGAAAAGCACCGGTCCCATCAGGTGTGGTCGTGTTCACGGTTTGTGTAACCTGGCTGGATGGTTCCATACTTGATTGGTCGACTGGGAGCGGGGCAACAATGTGACCGTTGGGCACGAATCTTTCGCACCACTGCAGGGACCCATTAGAAGAGCCAATCACCCAACCGCCAGACAGTGTCGATCCAGCGCGAGAGGCATAGTCCCCGAGCGCAGCCCAAGGTTCGTCGAGTTCCACATTCCTGATGGGAGGCAGCAACGATCGCGGGAGGACGAGTGGAGGGACCAAGTATGCAGCCCTTTGAGGAGTGGACGAGGGTGTCAAGCAAGCCAGTTGAAAATCGACCCCGCGGGGACCCGTAGGTGAGGAGACTTGAGGCGCGGTGTTGAGGAGGAGGATGTCGCGCGTCTCCGCGTTGAAGTGCTCGAGCCCCTCCTCGTCCGCTAGGAGCACCCAGTCCGTACCAGTTCGTCCAGCCGCCTGGATGAGAGCCGCACCCAAAGCCAATCCATCGGGCCAGTGGGAGATAGAGATCACGACTTCGTGCTCCTCGTCCTCAGAAAGGATAGTACGTAAAGCATGGCGAATATGGGTGTGAAGGGATTGGGGACTGAGCAGAACGACATCAGAGAGAGAGGCGGAGGTTCCAAGGAGGCTCCCCAAATGGTAGCGCAGGTCCGCTACCGACTGCGAGGTCACAGGTAGGATAGCGGTCAGAGACGGGATGCCTGAAGAGGGTGTGTTGTCCGCGGGAAGTGACGGAGACGCGTGTGGTGTGTTATTCAACACAGCGGGTTGCCCAGCGTGAAACACCCAGGGATGGGCTATGCCGTGTCCGTTCGCGGGTGGAGGCTGTAGTTGGTGCACGAGAGTATCAACCTCGAGTTGAGGCATGTCGTTGGACAGAAAAAGAAAGCGAATGAACCAGATGGAGAAACAAAAGACCAGAATGCTAGATAACATCTTAGAATATAGCCGATGGGATTGTCGACGCGCTTTGAGCACCGGAGAACTGGGTTGGGACATTGTCGACAGGGTTTCTCAGCGTGTAAATAGCCAGCGGAATAGATCCAGTCCGAGAAAATAAGACAAGTGCAAGTGGTGAGGCGACGGCAGATTGAGGAGTGGCCAGTGGCTCGAACAACGAGAGTGGACCGTGAATTCAAGTCAAGGACTCGCGGCGAGGTTCAACTTGTTGACAATGAGTTCA

>AT7611

GCTGTGTGCGTGCCTCCGTGTTCAAGTTCGACTTGCATATCGCGTAGTGCAGCAGGTTGAGAACCCACGTCTTCATAAGCGAATCCCCCGCGCCCGACAAGGTCTTGATGAGCGTCGAACACAGAATCGACAGGCTCGGGTGGCTCTCGAGGGACTCGCGCAATGTCGTGAGCAGTTCCGCCTGCTCCTTCGAGTCGGCCGGCGCCGCTAGCGCGAGCGAGAGTTGCGACAGGGGGTCGACGGGGGCAGCCATGTTGCAGTCCCGAACTTAGCGGGCGCGGCCGCTCTTTTCTATAGGTCGTGAAGGGAGTGAGGCAAGCGATAGACAGACCAGAAAGCAGACAGAGCCAGACGTTCGGC

>AT7614

CCTTACTGAGACGCTTCCGCTTCGCGCCCTTGACGAAGGCTACAGATACCTTTTCCTGCTCTGGGGATCTTCCGTTTCCGTTGGGAGGGTCTGAAAAGGCCGTGTTGGAGGGTAGGCCGTTGGTAGCAGAAGCAAGATGGGTTCTGTGGGTCTTTGAATCGGTGGGAGCAGACATATCGGGGGCGCGACGTTGGAGAGTGAGGTCGAGGTCAAGGACGGGGTTTGGTTAGTGAGGGCAAGCGAGGTGAGAACGCGATCGTCGGTTGGGGTCCGAGATGGCGGGGAGGGGCGATGGGTCAGAAGGGGGCGGGGGGAGGGCGGGAGAGCGTGGAAAGAGAGTATGTCGCGAGAAGAAGGCGAGCGAGTACTAGTCTAACGGGCGAATATCATGGGGGTATTATAATGCGACTGGCCCTGCCAGGGGAGATGAACTCGTTCGGCGGCTCA

>AT7641

ACAGCGCCCCCAAGCACTCTGTACGCTTTGAGCTTGTAGTCGTCACCAGCGTAGACCGCGTCCAGCGCCTTCGCCAGATCGGCGTGACGCACAGGGAGCAGCGCCGGGGCTTGTGGACACAGATCCGAAGACTCATTTGGTGGTAAAGCTCCCAAAAGAGTTGACCTCGGTGCTTTCGAGACCTGCTGGCGATGCCAGGGCCAGCCGATGCAGGCGAGTAATGAGAAAGAGAGGACCACAGCCCAGGCTACATGACGCTTTCGTTGGGGCTGAACCGTGATATTCTGGTAACCATGTTTAGACGAAGTGGCACGTCTAGAAGTATAGTGTTTCGAGCGGTTCTCTGAGCCTGGCTTCTGAAA

>AT7643

TGGGGCCTCGAGACCCAGCGGAACAGCCCGTTGCGCAAAGCCGGGACCAACACCCATGAATCCATTGTGTGCTTGGACCATGCGATGCCGGGAAGATGAAGAACCAAGGTCCGGACGGACGGAGGAAGCGGTCCAAGCATGAACAGCCCGACGGGGTGCGTGATCTCCAGCGTCTTGGCGTTCGGGCAGACCTTGAGCAGGTCCAGCATGAACCCCGTCGGCACGCCCGACACTGAGAGGTGTTGCACGTTCGGGAGGGTGCCGTAGGTGAACTCCTCCCGCCTAGAAATCGACCGCAGGTACAACGCAAAAGTCGTCCTCGCGGAGATAGTGAATGAGTAATTAATAGAGAGGTGGGTAACCTGCGGGGGGAACGTCTGCGCGTCGAGCTGTTCGAAGGCGTCGTCGAAGCTCCGGTCCGTGTTCTGGAGGGAGATGTGTCGGAGGTTGGGGAGGCGGTCGTGCTCAGTGACTCGGTGGAGGACTGTCGAAACCGCTGTAGACGCGTCGTCTTTTTGGGGACGCAGCCAAAGACCGACGGCGGCAGGGTCTTCAGATAGCCCAGGTTCGCTGGGCGGAAGGCTTCTGGAGACATGAAACGTGATGGAGCGACATATTTCGTCGGCGACTCGGTTCGCCTCCATGGTGAACAGATTGCACGACTCCTGTGTAGATGTACGTTCGGGGAGGAGTCCGAGAAAGCCATCTGCGCTCAGAGGGTTGGGGATGTGGACGTCACGCGAGGTGATGTAGGCGGTAAGCGTGAGCCATGTGCGGTTGACCAGAGCGATGTTCTTCAACAAGGCCGAACGCTCCCGCGGTGCTTGCGGCACTTCCC

>AT7978

GGAAGTGCTTGGGTATTCGAAGAACCGATTCCCTTGAAGAGGAGTGGGTAAGGCCGTCGAACCAGTGTGGGGGGGGAACGATACCTTCAAGGTCCCTGCCTATGAAGCCGGTCTGGGTCTGGGGTGAGCTTTCTCTTGATTCGCGATGGCGGATGGACGCACGGGATGTCTGAACCTCTTGAACCATCTGGCAAGGAAGGACATGACAACGGAGAGCTTAAGTAGCCCAAGTAAGCCTTA

>AT7980

TCGAAAACGATGAATACGGATATGAGGATTAGAAATATGCCGTTTGCAAAGCCCGAGAGAGTTTCTATACGACCGTACCCATAAGTGAATCGTTCGTTTGGAGGCCAGCGCGCCATGACGGATGCAATCAGGCCCACAGCAATGGCCATACAATCGAAGGCCATGTGATTACCATCGGATATGAGGCCTAGACTGTTAGTCCAGATGCCATACAACATCTGAACAAGCATGTAGCACATGTTAAGCATCAAAAAATAAAATATTTTGCGTGACTCCGGATTTGATAGGATATCCTTCAAGTAGGCCTGCACCAGCCGACTTGTCGACTTGTAATAGGAACCAGCTCCCAGTGAAGACCGATTTGGTCGAGGATACATGCCTATACATAAAATCAGCCAATAGACCCACGCAAACCCTGTTGAGACACTCACCATAGAGCAATAAGACTGGGACGACGAAGTCAGCAAGCTCGGGTGAGTGTCCGAAGCCTAGATAGCCAAAAACAATAGCGGCGGCGTATGTCGTTATGAAGGAGAAGGTTACATAACGAGGCGATGCTACCGCGATGTTCGTACTGACAGAGGCTCTAGGTTCGAGATGAAGGACGATGTATGCTAAGCAGGGAAGTACTGCCAGCGCAGTGAGACCCGGCATGTGGTGGCTGGCGTCACCCAACTGCAAAAGACGTGAGATTTATGTAGAGTGAGTGCAAAGCCGGATTCCACTGACCAGTATGTGTCTCAACACGTAGAGTGGGAGACTGAACACGCATGCGCCTAGCATGCTGAACGCAGCAGACATCGTCTGTCCCAAGGAAGGCGATAGAACGCGTTCTGTATGTTCTAATGCCGCAGATGCCACCGCTTCAATAAAGAGGGCCCCATAACCAGGAATGTAGTCGCTGAAGTTTTCTAGAGAGAACCGGGTATCCCAGAGGAAAGATGCGAACAGGGCGAACATGATGGCGGAGACTTGC

>AT7984

TACCAATGCCTGAATAGCCGGGATTGATTGGGACTCTAGGATAGAATCCAGACTAAGGACCGCCCGGCCGAGCTGGTAGTAGCGAGTCGCGTCCGTAGACAAGGACGGCCTATCGAGGTCGAGGAGGGCCCCAAGCGCGAGAACGAGGCAGAAGACCGCTAGTCGGTGCGAATCCTTGGGAGCCGGCTCGATCTCCATTCGCTGGTCGTAGATTCGGGAGAAGATGGTATCTTCAAATTCTGCTTCTGGAATGGGGGTGTACCTATGGCGTGCAGTGTGTATTAGCCAGAAGCGATCGGCGAATGGGGAATAGGTCGGATGACGTGACGTACATCCACGCAGCGTGTCTGTAGTAAATTGTGACCAGTTCACGGGCCTTTGCTTGATCGGGAAGGGAGGCCAACACGGTCTCGCGGATGCCGGTTGCTGTCTCGCTGACTGCGGTATAAAAGGGGAAGGTGTGACTGAGCCAAGGTATCTCTGCCGGCAACTGCATCTGAGGGCTTTCCTCCTCATCGTCACTCCCTGCTTCTTGCTACACGACCAACCAGAAGCCCTAAGCCTATAGACGGTATCTACGTGTCTTGATAGGCAGCGCACCTGCAACAAGTACTGCAGGGATAGATGCATTGTCAAAACACAACCGGAGGGAAGATCATCGTGGGAGCAGCGTACCCAAGCATTGGCCATCCCGCCGAAGAATTGGGTGTGGCCAGATTCGGAGATGGATCTACACAGGGCAATCGAAGTTAGGCGAAGGGGCTGAAAGATCAGCATGACGAATTTTACAATGAGCCAACATTGATAGATTCAACGACCTCCTGCTCTTGCTCCTGGGCCTGTTCCTCTGGCGACTCTCTCTCTAGCGGTCGCTTCAAGGCACGGAGCTCATCCGACAGAAGTGGATGGACTTCGCTTGAGAGGTTCGAATGGGCATCTTCGAGTGCGTCCTCCAGCGCGCGGACACGGTTGGCGAGCATTGTGATTTTGTCATGGAGTACTTCGGTGTTGGCGAGTACAAACCTAATAAGAATTGGCGTCAGGGCGCACCGGTGACTGCAGAGCTGACGATATGCTTAGGACAACGCTACCTGTTACCCTTGCCTGTGG

>AT8004

TCACCGGTCGAAGTGTCAACATGGGTGAAAGTGACGAGCTTGTACTTCTTCGCCTTGAGCGCGGCCTCGATGTCCGCCAGAGGCACGGTGCCGCCAAACGGTGCGTGGAGCTCGTCCACGGTCGCGCCGTACGTCCGAAGACTACCCACATCATGCGGTCAAAACCTCGGTATGGTGTGAAGGGGAGGAGCCCAAGAATGACTCGAGTAAGACCCACCAATCGGCGAAACTGTCTCCGAAGTATCCGGTGTTCAGTACGAGGGCGTTCTCGCCCGGCTCCACAAGGTTAGCGGAAACCTAGTTGCAAACGCGCGAAGGGAGGGAACCATCGGAAAATAAACCTCCATTCCAGCGCGTAGGATATGATGTGCCGGCATGGATGAACGTACCTGGTCCCAACCGAGGGTACCGGAGCCGGCGATGAGGAAGGGCTGTGCGTCCTTGGTGAAGAGGACGTCCCGCGTCATGCGGATCGCCTCCCCGAAGATAGGAATGAAGTCCGGGGAGATGTGGGACATGGAGGGGTGCGCATTGGCGTAGAGGACCTGGTC

>AT8006

CCCACGTTCCACTTTCCGTACCAATGGTATAGTCGCGAATGTAGTCGTAGTTGTCGTTCAATGCTCTTTGGATTATCTTGGTAGACTGCTCAACGAAATCCAGGAACTCCGGTGCAGAAAATATGCTTAGTTTCTCGTCTTCCGTCATCTCTGCAGACAGTCAGTTCACACATCCCGAGAAACACCACGTGAACCTATAGGCACCTCGGATTTCCTGCGCAATCTCCTCATCCAGCTGCTTGCTCTCGGCCTCCAACTCTTGCTCATGCGCTAACCGTTCCGCCTCTGCGGCTTCTCGTTCATGGAGTATCCTCTCCCTGATCTCCTCTTCATTCGCTGCGGCAGGCTCAGTCTCAATCTCCATTGTCTGGACCTCCTTGTTGTAGATGACGCGTTCCTG

>AT8009

CGCACATCAAAGCCATGGAATATGAACTAGAATTCATTCGAAATAGCGGGAAAGCCTCCATAGGTGTCGTAGATATCGTGTACTTCGGTAGCCAAGTCCGATAGATCGTCCTATAGTAATGGTACACGGGGGATCATACAGGATGGTAGGGCCGAGGTTCTCAGAAGGAGACGGAATACGAGTCGAAGGAACACGCTCGAACCGGCAGAGCCTGTCGTACGACTCTAGAACAAGCAATCGCAGCACAGTTCTGACGCGCACAAAACGCCCAGTCAATTTCCGACACACGGTTGAGAGACTTTTGTAGAACGCACCTTCAGCACAGCAACACAAGCACATTCCCGCTAGGCAGGCGCAGCAGGTATCCGATCCACCACCCTTGTCTTGCTGTTGTCTGCAGGTGCCCCTTTAGTCACACCGCCGCATTCTCGCTTTCGCAGTAACGTTACGTACACGTATACGGTCTGGGGGGGAGGCTGGGGCTGGTAAGGTTGACCGTAGGGAGGTTGGCCGTATGCTTGCTGTGGCTGCTGGGGGTAATAACCGCCCTGACCG

>AT8026

ACCCCTTGCCCTTAGCCTCGCTGATACAGTTCGCGAGCGTCCACGGGGAGACGTATCCCGTGGTCGCGTCGCCGATCGTCCCGGGCTTGCCGATGACGAGCTTGCTGCTCGAGACCCCAGACGCGATGATCTGGAACAGCGCGGTCTTCGGCCACCTCGCGGACGAGCTGGTGAGCAAGCCCGAGCAGGTCGTGTACTCTGCGCCCTCTGTGGTGGGCATAGCACGTTCG

>AT8027

GCCGGGGTAGTGCGAAGGTGTAAACCTGTCGATCAAGCTCAGCTTGTACTGTGGACCGCAATCTGGGGGCAGGAAGCTTACCATGGCGCGACCGCTATTGATCACAAGGTTGACGTCAGCACTTGACGAGAAGTGTACGAAACCGTATTGACAAACGGGCATGCGTAATAATGTAGCTGCTGCTGGAAGGCAGCTTGGAGCGAAGCTGCTTAGTAAACTTGATGAGCCAAGCCTCGGCATGGCCAGAATCCATAGCGCTC

>AT8028

GTCTTCACCCAGCCGGCGAGGTTGTTAGCGGTCGCGACTGGATCCACACCGGCAGTCGTCGGGACGTCAGTCGAACCGAACGCGGCGACCAAAAGCTTGATCCCGGCTGCGTGGTATTTCGATTTGACCGAAGAGCGCTCGCTTGCAGAGAGGGTAGTCCACTCGTACGCCTTGTCCCACAAGGTCGCTGGAGACCAGAAAGA

>AT8042

CTGCCAGTGAGTAGTACTTCACAGCCTGACCACTAACTCTGTATCTCCCGATCTTGGGACCTCCGTTTATGAATTGCCAACACATCTTCAACGGGCACGTCCCCGGTGGACTCGCTGCCCTCCGAACGCATAGCGAGATATTCTGAGACACCGAGGGAGTCCTCGTCTACGCCTTCGTTGCCACCCTCCCGGTCATAGTCTGCGGGATCTATGGTAGCGGCAAGCGAACCGAGCGCGGGCATAAACTTGACCGAGCGAATGCTGATGC

>AT8054

ACCCATTGTATGCGATATGGACCTCAGGATACACACCATCTCGTCGTGTATTTATTCCTTTCTATGCGTCGTCGATCTATTAGAAGTTGGAAAGCAAATACATGATGCGGGGAGGACAGCGCGAGAACGCGCCGGGTCCGGTCCAGACTGGTGGTCGCGACTCGAGACAGGCGCGTGCGGCTACTCCGAGCGGCCCCGTTTCACAATCGCTCGAGGGTGGGGGATGTGGCAAGGAATGAGATTGTGAATGCTTTACAGAACGATCCCGGCTTTACGCCTCCTGCTGCTTAGCCCGCTCGAGCTTGAGGTAGTACTCCTCTTGTTGCTGGACTGTGAACGAAGAAAACGTGCGGAGTTAGTGACAGAACGGGAGATCAACCTGAGACCTGAGGATGCGCGGATGCGCGAAATGCGCGAGCAAGGATCTGCGAAATGGATGGAATGCTTACCACGCTTGAGGTGAATGCCATACCTGTTCCGGGGCTGTGAGTTCACCTGACATGTAAGAGGAGGAATGTGGACGAAACTGACCAGAACGTGAAGCCCGCGCTGATACCAAGGCCAAGGGACGTGGTAAGGTCAAGCCAGAAGCGCTTCCTGAGCTTTCCAACGATGGGTGCGATGGGCATTGTGGAGTTTTGGCGGGCGTGGGGGACGTCGAGGAGGACGGGGGCAAGGACACTGGACAGGGCAAAGTCGGAATGAGAATGGGAGAGGAGGCGC

>AT8061

AGGGGGGGCCAAGAAGGAGATTGTTGAAAAAACAAAACAGCGAAAAAATGGCTATGTATGCAGAGTGGTAATGGGGACGGTATCGCAGAGTCAGGGCAAGTGATAATGGCACACGGGATTAAGCAGTGATTTCTGAGGGCAATCTGGTCGAGCATAGCATCGTCGATACTCAATGGAATAATGGTATAGAAAGGCACCATACTCAACGGTGACTAGCCGTTCCTGGCAGAAAGGACAGAGATCCGCCGTTTGCGCGTGCCAGGCTCCATCTCCGCCGCCGCCAAGAAACAGTTACGAGACGGAGAAGATCCACCCATGGGCACGAACACATCTGGCTCGAAGGAGGTCATAGTTGAGTCGACGGAGGCAACCGTCGAGTCGTACACCCACGAGAGGCGGGCGTTTTCCGTGTCGGCCGCACCTTCAACCACCGAGAGCTTCCTCCCAACGATCCTCTTCTTCACTGC

>AT8064

GTCCGTGTTAATTGAAGAGTGGGGGAATCCAAACCGTGACCACTGCGTCTCGAACGCATGCAATTACTCCGTCTGAACTTCTTAGCAAATACATCCCACAAAACTCGAATGGTACAACACACCAGCACTAAACCCACTTGGTACTCATTGTCAGTTTTCGGCTTTAAAGATGAATAGAGCTACAAGAGATGCCGCATTGCCGGTCAACCGAAGCGGGGAAAGGAGCGCGGGTGAGGCCAACAAAAGCATTTGAGCGGAGAGAAAGCTACAAGGACAGCGCGAGTCTGGCCGCATGAGAAAGCCCGTGTCGTGTTATGACAGTGGCGAGAGTGGCGTGCGGTCTGTGGGGGTGACCCGGAAGCTGGACTTGTCCTGCATCGAGTCTA

>AT8092

GGGGGAAGTACACTAGCTTACCAGTAAGCGCGCTTCAACCTCCAGAGCATCAGCGTCTGTCCATGGGACACTGGATTTCAAGCTCATTGTGCAAATCTCTGCCCAGCGTACTTCATCGGTCGGTGTCAAGACAACACGGGTCTTCTCAGGCTGCTCAAGCTCGGGCTCATTACTCTTGGCCGGCCGGTAGTCAAGCAGCTCAACAATGAGGCACCCTATACAATTGTTACATCATGCTGCTTTCAACGTTGCGTTCTACTTACCTTCGTAAAACGGCAATTCGGCATCCCCGAAAAGGCCTAGGAAATCCACGGGAATATGGTGGCTGCGGACGTCGTCCAATAATGACTAGACAGATATATAGTGGTGATAAGTCGACATATTGATTGGATACTCACCGCGACGTGATTGTTGTAGAGAAACTTAGAGCTGTTGTTCAATATCCAGTGCTCTCGGTAGAGCCGAATCGTGAATGACGGTGGGGCGTGTTCGTAGTCCTTGAGGACGCCTTGTAAGAAGCGGCTTGTATTATAAAGAGCCATACTACGGCGGATAGCTTGTCCTTTGGCCCAGATTGAAGGTCCGAGTGGATGTCGTTTGAGAGTGCGGCTGGGGAGACGGTGGCTCGAACTGAAGCGAGCTCGAGGATGACCAAACACGGTCGTGGCTTCCAACCC

>AT8100

ACCCAGATTAATGAGGCCTACAGTATGCAAAGGTACAGAAGACGACCATCCTGATACATGACACGAGCCTCAGCACGCCAAACCTTCCCAAACTGAGAGACAATCACGCAGCCGTGACTCCCGAGACTCCCGACGACCCTCCGTTCGCGGCGGTGGCCACCCTGACGAGGGGTTGACTGCTTCCTCCTATCACCCATGCTCCAAACGAGGGGGCGGAACGCGCCTCACGCCGAGCCTCCGGCCGTCGCGCCCATACCAGGGACCATGGTGGGCACGTTGAGCAGCTCCGTGGCCTTGCCCGTAAAAGCCCCGAACAATCCGCTCAGCTCCTCCATCCGACGGCGCAGCGCGTTGTACTGGCAGACGAGGTCCTGGTACTCTTGTGTCTGTACGCGGTGGGTCTCTTCAAGCGTCTGCAGCCGACCACGGACCTA

>AT8108

GCGGGATGGGCCTCGAACTAGAGATGACATGAGCTAATCTCCAAGTGCCTGAGGCTGTGAACAAGGTGTGGTCTGACCTTCTCCCGTCTGAGCAATGTGTCAAACACAAGCCCGGCATCAGGCAGTTCGTTGCGGGGAAGCGGGTGAGTTTGCGCCACGCTGCGCGAATAAGGAGTCCCAGCCTTTCCCAAGTCAGGGATGGCTGGGTTGTTGTTTCCACCGTCCGCACTCCGGAAGGCGAACTCGGGACCAATGGAAGTATCAGGCGGGTGCGGAAGGTCTTCGTACACTGCGGATAGAAGTTATGA

>AT8149

GGGCTGTGTGCACTGTCAATGAGTACCGTGGTAAGTAGGATACACAGACGAAAGCAATATTAATAGATGTCAGTTGCCTCTAATCCGTAGGGCTACATAATAGATCCTTACATACCATAGTCTGCTTCGCAATCACCAACAGACCATGACCTTCTCAAAATCCTCTGCTACTTACTGACGACAAGGTACAACATAATCAAGCGGATCCAGTACTTGAACACCATCCATGGCCAATCATACAAGCTCAATCAATAACGACCGGCCATCCCGACGAGAAGTCAACATAATTGATGCCGAGGTACGATGGCCCCCCAAACGCGTCGTTCGGCACGTAGTGGTACACCATCACGTCCCGCCCGCTCACGGGGTCCTGGAAGAGGCTCTGCCCGCCCGGCGCGTAGACGTTGTCGTGCGAGCCGAGGACGAGCGAGCCCGTCGGCGGGTCGAGGTCGAGCGTGATGTCCTCGC

>AT8154

GGCCCGTGAAGAACATTACCTTAAGACCCAAGCTCGGTACACGAATTCCTTCTCCCGGCATGTCCCATTTTTGCCATGGTTGCGAGGCCTTGCATGGCGAGGCCTACCACAGCGGGCAAATTAGGATGGCCCAGCGTGTGCAATGCTGCCTCTGGCCCTGTCATGGACCCGTTGTGATGACGAGGGCGAACCTATGTGCAGGACCGCTGAAATTTTCGAAACAGCGCGGGGTGTTTATATATGAACTTAGTACATATTGTAGCGGGGTGGGGCGGCGCAGCATGGGACTGTTCCATTGATCACACAGATTCCGACCAACCTTTAGAAGCGGAAGTAAAATAGAACCACCTAAAGGCAATCCAGAAGAATTCAGCAAACACCCCGTTTCTGAGGATATGGACTTCGAGACCTCCAGTATGTACCCCCGAGACAGGGTAACGGTACAGATAAAGCTGCAAGGTGGAGGCTCCGAAGCTACTGATCCGCATACACTCTCCATCAGATAATCCAGAATTTGTTGATGATGTTCAAGCCGTCATGCAGAGGTTCGATGTCAGGGAGTAGAGGAGGCCGAGCGCGAGGATAAATGTTGACCGAGGGAGTTTACAGGGCCCTGCGTTCTAGAAC

>AT8163

TGACCAGTTACAGGAGCGAGATCACGTTCTAGAATCATCTGCCAGATGACCTCCGGCGCCCTCGTCGATGCGTTGGTAGGCCCGAAACTTGGAACCGAGTGCACAGATGGGACCGCACCGTCGACCCCATGGTAGGTGACGCAGGATGGCTGAGTGTCGTAGTTGCCCAGAGCGTTTGCGAGCGAGCTTGACGGCGTGATCGACCTGCAGGCCGGCGTATTGTAGCTCAGTGGAAAGGGGGTGAATACAGCGGCGTCCGGTGATGAAGTGGGGAACGGGTGATATGCGATCGGAAAGGGACCGACAGGGCGTTCGCGCAAGTGCCCGGCCAAGCTCCCGGCAGTGTACCCATTTTCGAGAGGCGCACAGTCCTGGATGGATGGGGCATCGTGGAGGGTCTGAGCAGACAGTGGCATGGCGGCATACGGAGTCATGGGAGTGCGGATCTCACTGCCAGTGACGTAGAGAGCCGTACTAGTCGC

>AT8189

GCTGCCGCGGAATGCGGCGCTCATCTAGTCGTGTATTCTGCTCAAATCTCCCCCGGTCATGGTAACCCTCATCCGATGCCTAGAGATTTCGGGCCGCTCGCCTCGTCCAGCGCGGCCCTGTCTCCTGCACCTGGCCTGGAGATACTAGTTCACTCCGGACCAGGGAACGTTCCCAACTAGCTCCACGAGTCTAGAACTGCGGTCCTCTTGCGAACCCTCGGTCGCCCGCAATCGCCCGCTGTACGCGCACGGCAATCCCTCCCCCCCGACGTATATGTGATTAACTTTCGCCCTGCACCCCCTCCATCCTTCAACGCAGGTTGATACAACATCTCGTTCGAATTACACATGGGGACTTCTGAGGCGCTGGCTTTCAGGTCATTCAGCGTATGGGTAGTCAATGTAACCCTTCGACGCTTCAGGCGTGTAGAACTCGGCTGGGTCCCATTTTGCCAGCGGAATGTTTTTGAGGAGCCGCAGCGGAAGGTCGGGCTGTAAACAGACAGGGTCAACCAACGTCAATGAGGGCGAGGAGATGCTCTGACATTGCTGATGAACGGCTTTCCAAAGGCGATGAGCTGCCCTGTCTCCTCCGCAGCTCGTAACGCTGTCTCCCGGGTGTAGCCACCTGCGGTGACGAGTGGCCTTGGATCCCAGATTTCACGAATAAAATCGTTGGAC

>AT8193

CGTCCGGGTCGTTCGTGATGACTTATACCGCACCGTCCGGGGATCGCGCAGAGGCCTTGGTGAGCATCTTCACATAGGGCTGGCCAGTAGGGGACGCGGTAACGAGTGTCGCGGCGGAACAGAGGTAGGGGAAGAGTGTAGTAGAAAACTTCATCTTGATTAGGATCGAGTTTGAGAATTAGAGAGCTGTTGTGCACATATAAGGTATTGAGTATGTGGGACGATGAGTGGGATCCACATAAGCAACCTTGCCGTACTTACCGATGCTGATGTAGAACAGAGGAAGGGAAAGAGGCAGAGGCACAGAGTCGTTCAAGATCCTCTGGTATTGTTAAATACGGTCCGCTAGTGGGCACATTCAACAACCGTCTCGGAGGTACATGGGCGGCATGTTACGTCCGATGGCGATGTTCTACGTGGGCGAGCTTCTCTCAGCGGCCTATGCCCTCTTCAGAGAGCTTAACTTGCGACTATGCCAGC

>AT8414

GGGGTGCCTTTGTGTACTGAACGATCTCTTCCGAAAGCGCGCTGGTGGGGACCAGTCCACGATACGCGATCTGGCTAGAGTAGACGGGCTCCACCATGTCGCGCAATCGCTCGGCTTCCTCGGCTTCACCACGTTTCTCCGCCTCGTCTGCGAACTCATTGAACATCGTGCGCCGGACCGCGGAACGGATGCCGTCGCTGCCGATGACGAGATCGCAGGTCGCTGTCGTCCCATCCTTGAAGTTGAGCGTGATAGGCTCAGTCGCAGAGTGCTCAGAGTATGATTTCAAGCGTTTGGAGAAGTGGATCTTGTCGCCGGCGTCGAGGTGCTTGGCGAGGAGCTGCTGCAGAAGAGACCTGTGGAAGG

>AT8415

CACCGTCACGGTCCTTGGTACCGGTGATTGTAAGTAGTTCGTCCTCGAGTCCGAGCTCCTTCATGATCCACCAGATTCGTGGCGTCATTCCGATACCGGCGCCGATCTCGGTGAGCTCAACCGCAGACTCGTAGAGGTCGAAGTGGACGTCTGATGCGTATTTCTTGAAACATAATGCGAGGACGAGGCCCCCCATCCCGCCCCCGCTGTAAGACAAAGTAAAGTACTATGAGAACCCAAGCGTAGCAATTCGAGTGGATACGTACATGATGGCGACGCGCAGCTTTGGCTGGTCGCTTGCAGGCGTCATGTTGCTAGTTCTCGGTCTCCGTTCCGGCTTGAGGAGCTTGCTCGATGTGTTGCGATCGTCCGTATATATGTATCGCGGACAAGCGGGGGAATGATTAATGATTTAAGTTACCACCGTATCCTCGCTCGAGACTCCAAGCTTCCATACGCGTCGACGGGAGGTCACGCAATGCTCTTTAGGGCACGGCCGCCGGCGAGGAATTGTCAGCAGTAGTCAGCAGGGCATATGCGCAGGGTCGTTCCCTTTGATCAACGTTGATCCTCGCCCGACGGCCTTGGGC

>AT8416

GCCGCATCAGGAGCTCGGGCAAGTACCTCCCTGCTCTGCTATCCTCCGCGCTCACCATCTCCCATCCCGCGCGGCACAGATGATAGTTGTCCCCATTGCGCGCCGAACCTTGCTGGACCCCCTGCGAGTACGGTCGGCGGACGTCATCGTAGATCGCGAGCGCCGCGGGGAGATTCGCGAGCGTGACGGCGGGATGGGAAAGGATGGTCGCGAGGACCAAGCCGTCCTCGAACGCTTGGCCCATCCCAGCACCTTGATGGGGTAACAT

>AT8421

ACACTATCTGAGCTGTACGCGCGCGTTCCAGGACATCCTCCGGGAGCGCGCTCGTAGGTGCCAACCCGCGATACGCGATCTGGCCTGAGAAAACGGGCTCGATCATCGCGCGCAATCGTGCGCCTTCCTCAGCTTCACCGCGAGTGAGCTCAGCCTCGTTTGCTAGCTCGTTGTAGATTGTACGACGGACTGCGGAACGAATCCCGTCGCTACCAATGACGAGATCGCAGGTCGCGGTGGAGCCATCTCTGAAGTTTAGCGTGATAGGGTCTGTGGGCGATGTTTCCGAGTAGGAAGCGAGACGTTTCGAAAAGTGGATCTTGTCGCCAGCGTCGAGATGCTTGGCGAGGAGTTGCTGGAGGACCGACCGCTGGAACGTGTGC

>AT8425

CTCCCGCTCCGTCTTCGCCTGCACCCGCTGGAACGTCTCGCGCATCGTCTCGAGGCCGTCGGTGAGCTTGGCCACCTTCACCCTGACGTGCGGCTTGAACGGTGGGCCAGCCGCGCTCTTGCCGGTGGTCTTGCGCTTCTCGGAAGACGACGACTTGGACGGTCGAAGTTCGGGGTGGGCGAGTTCATCGACCATCGCCTTCTCGTGGCCGTAGGCTTCCGTACTCTCGACGGCGGCGGCGTCTGCTCTCGTCTCTTGGATGCGGTTGAAGTGGTCAGTGTTCCTCGCTGTCTGGACTAGGGTCATATCCGGGTCTGAGACGAGATGGAGCCGTCCGTCTGAGGGTTTGATGATAGGGAGTTTCTTCAGGTCGGAGTGCTGGCTGAGTTTGAGCTCCACCTTCGC

>AT8487

GCGTCACGTATGCTACGCCGAAGACCGTGTACGGTAGCATGGCCTCCACGATCATTGAGACGACATTGTTGAAGACGCTGGGTCCGTCATCCACCCGATCAATGGCCTTCGCACTTCTGCGTGCTTGGGAGATGATGAGGCCGCAAATGAACAGTGAGCATGTAACGTTCATGGTAATCCCGACTGCAGAGTAAGCCAGGGCAATGCGAGGGGCGATCCCGACGAAGAAGTCACCCCCCGGTCGGCCGGAATAGTAGC

>AT8488

GCCGGCCGTACCAGGTATGCATCGCTCATCCAGTTCATCACGACCGACGACGCGCTGCCCAACGTCTGATACCACACCGCCGCGTGGTCCGTGTAGTACTGCCCCGAGCCACCGGGATACCCCGCATGGATGATCCACATCTCCTCCCCGAAGAAGTTTTGCGCAAAAAGGTAGACGGTGATCATGCACAGGAGCCCCGTACTGAGGAAGAGGGTCCGCCAGCACTTCTCCTGGCCTGCGGTTGCGTCGGCCCGGCGCCGACGGCGGGCTCGGCCATCTAGCACGAACTTCACGGTAGCGAAGTATAGGACCAGCTCGACTCCTGTGGGAAGGCAATGTGATGATCGCCCGTCAGCAGACACAGGGGTATGTTGC

>AT8503

ACCGATTGGGGCACGAAGTTTAGCCCAAGCAACTTGTCTGCGAAGATGTTGTACATCAGGGACCATGACTGCTGGTCACCGTAGTTTCCAAGCAAATGTGAGCCATCAGAGGACGTTGCGCGTGACAAGAACGAGCTGAGGAGGTCAAACGCCTTGTCCTACACACAACTCTCATTCAGTCCGCCACCTCAAGAAATGCAAACATTACTCACGCCATACTGCTTCGCATCAGCATCTTCACCGACCGCATGTGCGATCTCAGCCATGGCCTTCACGCCGATTATGCCCTTGAGAGCGATGTTAGTTAATGCACTTGCACCCCCGCTGTCCTGATTTTGTCTGGTA

>AT8514

CGATGGGAACAAGTAATTGCTGAAGAATTTCAGTCCAGCCAATAGCGCCAGGACCGCTGCTGCCAACGGGAGGTACGTCGACATGATGTTTTTGCTGATTGAGGATCTGTGGGGTGGAGAGGAAGATGCAGATCTTGTGGGCTCGTCGGGGTCCTTATAACCCCTCTGGGAGGTGAAGAGAAGGAGAAGCCGCGTGGGAGCGAGCTCCATGACCGGTGATAGCGAGTCACTGTCAATGTCAGAGTGCCGTTGTCGCTCGGTTCCGCAACTTCCCCGCCTCTCACCCCCACACCTGATGCGGCACCGATAACCCCCAGCTCGGTACCGGAGGACACAGTCGTCTGTGCATTGTATTCAGGCATAGCCAGGCGGCAGATCGCCTCCATTGC

>AT8530

ATCGGAGACGCCGTACTGAGCATTGTTCGATGCGAGGTACACGACGAAGAATACGACGACGCTGGCTGAGTACATAAGTCCCGACTCAATGACGATATGCATGGTACGTCTCAAGGCCGAGGTATCGCTAAGTGCCGTACCCGCAAACGCCGTTCGAGAACGGCGCTCGACTGTTCCGATCTTGTACACTATTAACGCTAGAAAGCAACCGCAGGTCAGCACGACTAACTGGTAAACGAACATGGAGAGACCCGAAATACAGGTGGCGACAAGGTTGAGGGCAAGGGTGATGGAGAGGGTGGAGGTGATGAATGGCTTGAGTTCTCCTGCGTTGAGGAATGTGTTGGTGTGTAGCGTAAATTCGATGTAGCACCAGAACGCTGATACAACTTTCGGGCTGTCATGAACCATCCGTGAAAGACGCGAGAGGGAAGTTGGTCTCACTTATGCCGGCAACCCAAAGAACG

>AT8567

CATTTTGCAGCTGTCTCGTTTGGGCGCCACACTCCTTCACAATTTTGAGGAAACTCGTGGTCGCAGTCTTCCATAGAGGCTGATCCTTGCCAAACTTCGATGGGGCAGGACATTCATACTTCAACTTGATAGGAATGGAATATGCCTGGAGGCGATCGTGAGCGACGTTACTAAGGTACCGCGAAGTAATCAATCTTACAGCGAAGAGCGCCTCGATGGTGCCATCTGCGTAGATGGACGCATCGGACTTGAATCGGAGAAAGAGGTCGACAAGGTGAGGTAGCGTCTTCTTCGACAATGCAATGTAGGTCACGCGATTCACTGTGGACCGGGAGACCTTGGACGCGGAGGGAAGTCCGCTAGAAAGTGCGGGTTGGATATCGAACGCTGCGAGGAAGGGAAGGGTGGCGTATTCGGAAAGATCGCGAAGGACGAGGGAGATAACACCACGCGCTGTAAGCTCAATACTGTTAACAGCTTCCATCACGACCG

>AT8580

TTGTGGTTCACGAAAGTGCTGCTCCCGCTGGTGCCGTCGGACGCCCTCTTCATCAAATCGCTCAAGGCCGTCCACGAGTTGTCACTACCGTCGACAGTAAGGCCGAGTGCCCCCTTCAGCCATGACGACTCGTTGAACGTAGCATCATATAGGCCCACGGCCCCGACCAAGAACATGATGGCGCAAAAGGCGAGAGCGATAGAAAGCAGGATAGGACGTCCCCGCTTACGGTTGCAGCGGCAGCGGCGGGATTCGGCGTCATGGGTCTGTGCGTATTGGTGACCGTAGTGTCCGTGTGGGAAGCTCTTCTCGTCGCTACGGTCGTAGGGAGTTGCAACGGGGGCATTGGTTGCGAATGGTTGGGACGTAACAGTGGGCATAGTCAAGGGGATCTATAGCAAGCACGTCGGGTGGAGAGTAAAGCACGGTACGGTTTCAGCACATGCCTGCTGCACCTTTGCCCTCTATAATGCAAAGTACTCACCCGCGTCGAACAATGAATGGAAGGTCCTTCGGGAGACAGTTCTCAGCTGGGTCGGAGGGAGGGAAAGGCGGGTGGTGGGCGGATAGACGACTTGGACCTAAGTGACTTGAAGGGTTGTGGGGGATGACCTCACAACGCCCCGACCGCAGCGCCATGCCTTTATGAGATTTTGGAGGTCTACCTGCGTTTATGCGGACTGCCAAGGATTCTCGTGCCCGGCGCGAGGAAGGTTGGAAGCCGATACAAGGCGGTGCGGCTGAGTGGGTTAATCCTGTGGAGTCCTGAGTCCAGTCCGCAC

>AT8589

TCAGCCAAGAATCCGGGCTTGAACTTGTGGTAAGGAAGCGTCTGGGATCCGAGAGCGCCGAGGACTCGATCTGCATAGCTCATGGCGAGGGCCTTGATCATCCCCGTGTCTGCGGTGCCGGCGCCGCCCTTTCGCTTTGGGAAGAGCGTACCGAACTCCTGTGCAGGGGTGAAGGTGCGCTCGAGCACGCTCGCGATGAGGCTGCTGTCGCAGTAGACATCCTTTCCGATTGCTAGGACAGGTATCAGCCGGTACGTCACGCCCAGTCGATCGGCGAGGTCGGGACGGGGGAGTGTCATAGGTACC

>AT8590

CTGGTACAGTATGACAGGAAGTTGAGTAGCGGCGGACGTCATGTTCACGAAGAGGCCTGTCTCGCGTTCTCCACAGGGGTTCCCGCACCAGATTTAAGAGGAAAGCGAAAAACTAGTGACTAAGATGGCGCTGGGTTGAAGGGCCTCGTATGAAGTTGGGGTGCCTCGAATTTGAACACCATGGACGGTTGCCGGTAATCCCGGGGTGAAAACAGGTGGGGTATTCAGAAAGTCACATGCGTTCCTACACACGTAAGTGGCCGAGATCTCGGGACTCGAGGAGAGTAACTGCCAGCAATTACCAGCAAGTACATCGACGAGGTGAATCGTGACGAAACTTGATAGGACTG

>AT8613

ACCGGGGGGCAGACGATTTGCGTGAAACGTTTGCGTGGGCATCATGACGTGCTTGAAGAACCCGGTCCTCATCCGCTCTGCCGTGGGCGGGACCTTCGGCCATGGCACATTGAGGAGAGGTGCGATGGCAACAGCGTATTCCGGATCCATGCCAGAGTTTTCAAATGTCAGCGCACCACCTTCCGTGGCTTCGCTGTGCTTTATATCTCGCTGACCATGAGATCAGCCACCACCCCGATGTCTGAGTCACGCTCAAGATACAAGCACACCGTCCAGAAGATTAGTGTGTGAGAGTGGTTGCGGACAACACGTAAGGCTGAAATCGCGTGGACCCTGGATCGAGTCGACGCAGCTTTCGGAGGGCCTAGGGAGATTGGTGCATTAACTTGTAAGTATGGTGCTCGGAGAGTGGGAGGTGACATGTCCAGCTTCAGTCGAGAGAGAGAATGCGAACCTATGACAGGTCCCATGGGAGCCTCCATGAAATCATCATCGATTCCCTTTTGTCATTGACACAAGTGTATCGATGATGATTGGTAACCAGTTCGAGAGCCAGAAACTAGGACCTGCTTCTCTAAGTCCGCGCAAGCACCGGCGGCATGAGCGGGTAGTGAC

>AT8614

CTCTTGGACACATTGCCATATATTGTAATCATACCACTATGTAATCTGTAATCTGTAATGCCTAACCTACATCAATGACGACCCCGACTATTTCAACGCCCCAGGGACACGAGATTTGAAGGAATGCCTATCAAATATCATGGGACAATGGCAAAAAGTCATCAAGACGACTTTGCCAGGCCCAGGAGCCATCGCAGACCATCGCGAGCATCATTGTCGACTTTTCGGGCCACGGATATCCACGGAAACGTGTAATAGAATCCATGCACAACCCCATCGT

>AT8616

TGCGATATCAGCGACCAGTGCCAAACTGGAACGGGCGAAGCACGCACTTGTCTGGTTGAGCGAGCGGATGGACGACTGTCGGTTCACGAAGGTATTGTCCCGTAATCGGGCTCTCGGAGAAAAACGGGTCATCGCGAGCCTGGAGCACCACGGCAGCGGCTAGGCTGGCCCCTGCAGAATCACCTCCTATAATGAATCCATTCTTTAGGGACACGCGAAGCTTTGATGCGTTCCCGACGACCT

>AT8632

CACCTGAGATGGGTGCGCTGCCCAACGCGCCGAAGAACGCGCCGGCCTGAAGCACGGAGACGACGTTCGAAGAGACGTCATTGGCCTTCTTCGTGTTCGCCGACCCGTCCGCGTTGACCAGCCCGAAGTGCGTCTTGAAGTAGGTCATCGAGACGACGCCGCCGCCGATGCCGCTGTCCCCGCCCTGCTGTCAGCGCGCCAGGCT

>AT8633

ACAGTAGACCCGTATGTTCTGGAAGGTGCCCTGCTGGCCCATGTTGAAGCCCGAGACGCTGACGAGGGGGATGGGAGACGGCGTCGAGTGGAGCTTGAATGAGGGTGGTCCCGCGGGATGCGTCCAGTGCCACCGCCTGGGGCAGGGTTTTATGCTTGGGAATTTCCGGCCAAGCGACCCCACGGGCCGTGTGTGATTGCGGGGGCGAGCAGGACCCCTGACAATTCACCAAAATGCCTGATCCCTTTTCTGAAAATTGGCTTCGATTTGTCCGAAGGATCAGGCCCCAATTTGGGGGCGATTTGGCCGGATTAAATCAGGCCTTCCGGCCTCGAGCCACCGTTTGTCCGGACGAGCGGATGAGGCGTTCTGAAGCGCGATTTTGTCCGGATTCGAGAGGATCAGGCTAATTTTCGCCTCGTTTTCACTTTTTGGTGGCGAAAATGGCCATTGTGCCGCATTTGACTGGAGAACTGAAACTCTGCGATACCAGCATATGGGGAGCGAGGAGTCAGAGGACTTG

>AT8641

AGCGCACGGTTGCCCATGTAAAGGGCGTACGACGCCGCATCGAACAGGAACAGCAGTTCCGTCGTGGAGATGTTCGCCATGTCGAAGTCTGGCTCGCTATAGAGAATTCCTGTGTCCTTAGATTCAGCAGGTGGTTTCGGAGAGTGTAGTGCCTAAAAGGCGAGAAACAGTCAGACTGGCAGAGAGAGTGTCACAAGAAGAGAGGTGGAGACCCGAGCTGCCTCGGATGAGGGTGTGCGGGTTGGAGTCGCCTCACTTCGGCGGTTCTCTTGAAGCCGGTTAGTCAGGCTCTCCGCGGATGACACTTTCGGTGGATGGGAGCATTAACAGTACCAGCCTGACTGAACTCGAGGACTGCGCAGCGTCCCACGACCTAGGACACAAGAATGGAAC

>AT8643

ACGCCGTCGAAGATAGCGTGCACGCCCAGGCCGCCCGTGATCTCGCGCACGCGCTCCGCGACGTTCTCGCTCTTGTACAGGATGACGTGGTCCACGCCGTGCGACTTGGCCACTGCCGCCTTCTCTGCGGTGCTCGTCGTGCCGATGACGGTCCCGCCGCGCGCCTTGACGAGCTGCGCGAAGAAGAGCCCGACACTGCCCGCGAC

>AT8654

TACCGGTAACCTTAGAACAAAGGCCGTCCAGTGCATCAACTTCACGCATGAGAGTGCCTTTCCCCACTCCTCCCATGGAAGGGTTGCATGAGAGCTCGCCGATGGTGTCGAGGTTTTGAGTCAAGAGGACTGTCTTTGCGCCTGTGCGCGCCGCCCCTGCTGCAGCCTCACACCCCGCGTGGCCTCCGCCAATGACACAAACTAGAACGTTCGAGAGGTGAGTCTACGTCATGAATCTGGGGAAGTATGCTGGCGTACCATCATAGTGCGGATACGCCGCCCGACTCAGGGACGCATATCTTCTGTGAACCGCAAATCTTCCGCGAGACACAAATCTGCCGAGGCACCTGTGCATGCAGGTTGGTGAGCAACCCCGTTTGTTTTTGTTTTGCGGTGGTGGTTTGAGGGAAAGCCCAGCCTGCT

>AT8657

GCCATGACTGATCCAGAACGAGATCAAGGAGAGGGGGATGTCCGCGTGCTGCCACAAGTCGAGGAGAATCCATGAAATATTATACACGTGAACCGAAGACAAACACACCGGTTAATCCTCCTCTATAGCATGGTTCGTGTCTAGCCCCTTCGCATCCTGCCGCACTTCCTCGTCTTCCAGCGCCATCGTATCCTCCGGCTCTTCCGGCTCCTCCTCGTCTCCCTCCTCATCCTCCATGTCCTCGTCCACATCCTCATCGCCATCTTCCTGGTCCTGATCCAACATCTCCTGGTCCTCTTCGGCGTCCTTCGTGGGTGGTTGCTCGTGGCCCACCCCTGAGGCAGGCTGAGCAGCGGAGGCTGGGGGACGGATGTGAATGGTAGGAATGGGTTGTTGGTTTGCGTTATCGGACGAGGCAGCGGCCTTGCCTTTGCCCTTAGGCTTGGACGCGGCCGACGCAGACTCGGACGTGTCTTTCGCTTTTGCTTTACCGCCTTTCCGCTTGTCTGCTCTCTGCATCTCGCGGTATGCTGCAGGGTGGTGAGGAGATGAGGCGTGAATGCTCGGGGACGGGGCCGGACGTACTTGGTAGCTCCCCTTGGATAGACTTTGCGATATCGTTGAATTCTACGAGTTCAAGCGCCTTGAGGACGTCGGTGGCGGAGATGCTCTTGTGTTGTTTCGAACTCGCTACTTCGTGTGC

>AT8665

ACTCGTGCCGTCAGTGGCCTATGTTTTTTTTCCATCGGAGCCGCAGACAATGCAGCGATAGAGGGTACGATACACCGGCGCGACAAAGAACCAGGGTTGATTTGAGTCGCTCCAGCAAAGATCAATCACGACGAACGCACTCACCGTCGCGATGTCGAAAGGCAGGGTGCCGACGGTGGTAAAGGTCACAGGCGAGCCGCTGTCTCTGCCAAGAGTGCTGTTTCGGCGTGTCATCCGGCGAGCATGACGCAACCGCAACGAGATCGGGAACACAGAAAAATCAGGTACCGCACGGGCGCTTGATCAGAAACGACGAGTGATTTGAGGGCGATGAAGACATATGGTGCTCACACGCCGGAGGACGAAAGCCGCAAGAAGCCAAGGGAGCTGTTGCC

>AT8694

TCGAGTGCTTTACCCACGCCGTCGATGATGGGCGGGGGGAGCGGTTGCCAGGAATGATACTTGTCATGGGCGGGGATGGCGTTACAGGATGAATCCATGATAACCATCATCGCCGAACTTCCCTTCAAGGGGAACACACCACCCTCAATGAGCGCAGGGGCATAGTGTTTGGCGGCGAGCATCTTGTGGGCTTTGGAGCCGTAGGAGTCACAGAACTTCACGACCACAGGCGTCTGGTGGTCCTCCACCGTCACCTTCGCTCGGAAGAGCGAGACGGTTACATCGTTACGCAACCTATCGGTGAACTGTACGCGTGGGAATGGATTGGAGGGAAGAGGCGTGGGGTCGGGAAGACAGAACCTCGGGTTCGGAGTGGTCGGGGACAGTGACTGGTAGAGGGTGCGGAGATCTTCCAGCGTCTCTCGTACTGCTCGAGCGGTCCGAGCAAGTTCCTCGATATGGGCGCGCGTTCGTGAGTCCCGCCCGCCACTGAGCTTGAGGTCGAGGATCGTCTTAAAGCTGCACATATCAGCGAAGATGCCGTATGTGATCTCGAGGTGAGCTCCATCGACGGCAATGAGAATTATCGGACAATGCGACGCAGCTCG

>AT8696

CCATGAACTTCATATGTATCTCCGCGACTTGAAAATCCCCGGCCCCAGCCATCCCCAGTTCGTTTTTGAACTCTAGGATGACGCGACAGGCACGAACACCTTCCTGTATGATAGTGAATACCACGCCGTCGGCGCGCGCACCATGGGAAGCGCTCTGGCGTGGGCGCTTTCTGCCGGGCGCAGCTGAGTGATGCTCCTCGTCATGCTCCCCAAACAAGACTTGGAACTCTGGGACGAGGTCGACCTTGAAAAGCTTCTCAATGATGGGACGTATGCGATGGAGCGAGCCAGCTCGGTATCGTAGGTCGCGCTAGACAAGGAGAACAGTTGGGTAACCGTGTCCAGTATGTCGCCGTCAGGGTTGGAGGGAGGCTCGTTCAGCGCTTGGGAGAGGTACGCCATGGGAGGAGAGAATATGGATGGATCTGGAGCCGTGACGTCCAAAGGGCGTCCTATAACGACGTAGTGACGGCTGATCGCCGTACGAAGGTCCTTCGACAGGTCCGAGGGGGCTAATGCCTCTTTGG

>AT8735

TTGCCTCGTAGGTGAGAGAAAGGATGTCTGCGTCGTCGCCGGCTTTGAGGGTGCAAATGTCATCGTCCTTTGCACACTTGAGGACCTTTGTGAGGGACGTAAGGTTGACACCAAGGGGTATGGGACGGTCGCAACGGAAACGTTTGAAGCCGGTAGCCTCGAGATTGACAGAGACGAGGGCGACGTGGGAGTTGTCCATGGCTTGGAGTTTCTGGCGCGTGCGAACGCGTTAGTCGCGTCGTGATTGGGAGACGAGCAGAAAGAGAAGGTACGCAAAGAGGACTCACGATTCCCTCCTCGTTACAGTCAAAGTTGGCATCTGTGACGAGCTCCTTGACGGCTGGACGCGTTCAGTAAGCGTGGGGCAAGACAAACGAGGACGGGGGGGGACGCACCGTCGAGGAGACGTTTGAGGACAGCGGCTTCAGACAACTTTGCTTCCAACATTGTGTAAGGGGACCGCGGTGGTGAATTGGGTCGCGATGGGAGTCGGCGGTGGCGCGGTGGCTGGCGGGAGAGGCGGCGACGGGGGTGGGCGAGAC

>AT9011

GGATCTGGTCATATCCATACCCGTTCGGCAGTGGATGACCGCGTTCGTCACCTAGCGCCGAAAAACAGACCGCCGTGCTTATGAGAGTCACTCGAGTACAAGTAAAATATTATTCGGACGTACATAAAATCCGATCTGGAAGCCGAACATGACACCTTCATACCAAAGAATTGCGAAGAGGTGCGGGCGCCAGACCACTTTGAGCGGTGCGAGTACGACGGCCCGCCAGGACGGGCGAAAGCACGCCATCTGGAACCCTGTATGACCGAGCAGAGTTTCGATGCGGTAGCGCACGGTGTGGGGCTTGGCGAAGGGGTGTGGATTTGGGAGAT

>AT9014

TGGCCTCCTGAGATGTAAGCGTTTCTTTAGCTACTTGTGGGGGAGCCTTGAATGAAGCTTTACGATGGTCGAACATCGTCTAGATGTTCAAGTTTCGATCGGAAGTTGTTGTAGAAAGAAAATTAGTCTCTACTCAATATTGTGTCCTGCGGTATAAGTCTTACGGTAAGGTTGCCTTCTAATACAATGGGAGATGTGAGGATGAATTCGCCGCTATATGCGTCCAGAGGCAGCGTGCTCAGCGGGAGCAACCTGAGTTATGTGGCCGAGTCCAAGCAGCTGCCT

>AT9019

ACCACGCCGATTGCAATTGGATCAAGGGCCTGAAATTTATGCAAGAAAAGCAATATCTTCTAAACAGGGCAGGCAACTACCGTAGAGTTGTCTAATACAATTCCTGCCTCGTGATCATCCAGCCACCAAGGTGCCTTTCAACCACACGGCGTTCCGCTTCAACATACCAGACCTGATCCGAGCGGTCATCCTCGTAGTCAAGAATCGTGGTCAACGCCTCGCCGGCTATAGTACAAAGTCAGCCTTTGAATCATTGTGCCGGCTCCACCGTGCAAATGAAACCTACCAGGAAGTTTGCCACGGATCATCTGGATCACGTTTTCCAATCCTCGGCGTGCCTCTGAAAGTTCCGTTCCGTTGATACTAGTACTTGGCTCAACGTTCCTTGCGGCGGTCTCAGCCAAGTTCAGCAAGTTGTCTGTGAGAAGACCACTCATACGCGAGGACGGCGACTCATGAAGGTCCTCAAGGATCGATGCAACGCAGGACAGCATGTCGGGGGTCCAGAACCGCTGCCGCGCAAGACTGCTTCTTAGTTGCGTGACAGTGGACCGCGCACACATGAGGACCTCCGAGTCGTTCCCGTTGGCTCCGGTGGTGTCCGGGGACACGAGCGACACCGTGCAGCAGCGCAGTAGGAGAGCGATTGCATGGAAGAGTCTCTCGCCGAGGGCCAAAGTCAACTTTTCTGGGCCTTTGTGCGTCCTCTGGCGGTGCA

>AT9029

TTACGGGAACCCAGTACGTTCCGGGTCCATACCCTTGGCCATTCGCCCAGGACGGGGCAAAGCGTGTGTAGCTGAGAGGAGCTACCTGCAGCTCGCGCCTTATCTCTTCAGCGTGGGTGATATTCGGCTCGTTGTCATGATTCTATGCACAAGATTATCTTTATCAGCCTCGTAAATCAGCAAGGAATATCAATGTCTGTGATAGGTACTCCGTGAGTAGACGAGAAAGGCAGCCTCAGTTTGTTGAGAGGGCCGACAATCTCGTCGATGAGTTTAGTCGAATTTTCCCGGAACGTGTCTATCAAGTGGCC

>AT9030

AACACGTAATCCGGCTTCTCGCTCGTGAGGACCGTGTTCATGAGCTGCGTGCTGTTGACGTCCTGCTGTGGACCCCAGGTATCCCAGGGGTTCTCGCCGTAGTGCAGATCGGAGAAAACGGTGATCTTGAACGTGCCGTCCTTCCTGAACGTAAGCCGCGGCCTGTTGGCGTACGGGTTCAAGTTGAAGTCGTCAGTCTGAGGAGCCGCTCTCGGGGACAAGCTGACGTATTCTGTAGGCCACGGAGCGGGGTTAGTTGGAAGGTTTCAGGCACCAGAAACTGAGACTCATACCTTGGGCGACAGGGAGTGCCGAGCAAAGTTGAAGGCTAGAGGCAGCCGCAAGGACATGGGCGAAGAACTGCAACCCAAGCAGCATCGTCGTTGCGTGTCGTGAGGCCTAGGAGAGCTACCTCATGCCGACAGGGTGAGGCTGTAATATAACGAGATAAAAGCCGCAAGAAGTCCGATACAAGTCAGCGAACGTGCGTCCTGTCAGCAGGAGTCGGCGGCAGCCGCGTTGTGCTTTTTATGCAGAGCGTTCATGTGGTCATTTCTTCTCGAACCGGCCTTCCGCGGTCATTTGACAGGCGAAAATGGGCTGATCTGGACCGGTACAGTCAAGGTACAGTCGCAAGGGCTGAATATTGAATATTGAATATTGAATGCTGGTCATTCAGGACGAGGTCATGGCACTTGAGGTCATGGTCA

>AT9032

CGCGCGAGAGCCTGAGGGAGTCGGACACGAGCTGGTCGTCTATGGACTCTGTCCGGAAACCGAAGGTGAGGGTGAGAATATTGTTCAGGGAGGCGCGGCTTGCGAACTTCTGGGGGTTGATTGGGACCGTGCCCGCTTTGCTGCGGACATATAAGTCGTGGATCATCTCTTTAGCTTCCCGCTCAAGCCCCGGCATATAGCCGGCGACCGCGCGAGTGGTCAAGAACATGGCCGCGAGGCGGCGGTGTTTCC

>AT9041

CCTTGTTGGCTTTTAGCAGATACACATCCCTAAATGACTGGACGGCGAACGTGTGTGTGAGGTCGATGCTGCTTGGGGCACTGGGGGTCATGGTGGACCACAACCGACTTCCGGGCAGCGACGACGACGACAAGGTTTACCTGAGCATGGCCCTTGGCGCCGTGTACGTATACTTGAAAGAGCGTTGAAGTGAAGCTCGTCCAAAGCGCCGTAAACGCATGGAAACCGGCGATGACGGCATTCAGTGGACGGACGCCTTCCGTTCGAGAAGTTGGTCGGAGGGGAGAGTTGTATAAGGCGCAGTAATAGAGGGAGATCGAGAATAGGTTCCATGATCAGACATGGTAGATCGAGGCTGTCGCCATGTGCCGTAGGTGCAAATCATAAACGTGACCTTGATCCTCAAGAGCTGAAGAATTTTCAGTACATGGCGACTCCCCGCAAGGGTTCATAGGCTCGATCGTGAGACGACTCACCCACCTCAGGACATGGAATCGATCCAGGTATTATTTCCGTGTGGGATGCGACCGTTACTGTCGACTGAAGGAAGAATATGGCTTGTTATGTGGCAGCCTAGCCTGTGAGCGGATGCTATAACTATCTGTCGCATGCGTGAGCTTCCATAGCGCGGCTGATTCCCGATGCCACTCTGCCAGGCTTCGAAGGATCCATCGTCAAGAGCTTATAGGCGGTGTCGAGAGAACATGTCCAGCTTCAACCTGCGGGGAAGGCATCCTGAAAATAGGGCTGTCAGTGGCCGGTGTGAAGTGGATACCGTGACGTCGGCCACACCTTATCGCCGCCGCCGTGCTTCAAAGATTCGACCAGACGGGTGCTGCAGGCGATTTGTTGGAGCCTAGCGCACAACTGTGCATGCTCCCCGTCGAACCCACCGACAACGACACCTACTACAGCTGGGAGCCCCATCTACGACATTCGAAAGAACCAGCTCGGTCCGGGTTGTAGAAGCCGGCCCAAACTTGAATCTTCGCCTGGGCCCCCATGGCAATGTGCTTCTCGCAGGTGAGCGC

>AT9054

GCCCTGGATGTCGGCGATGTTGAGCTCCAGCGCGGTAGAGTTGCGCGAGCCGTCAGGCCCTGCAAAGGTGGCAGCGCCCCAAGGCGAACCTGTGGGTATTGCGTAGGTTTTGCATCGCAACATGTCAAATGGAAGTAAAGAAGGGTCGTGCATCGCGGGAACACCGAGGGGTGTAGAAGAAAGCGGGGGACTCACCGCCATGGACCTCTGAGAGGTCCGTGAGTTGTTC

>AT9086

AATGACGAGAGCGGCGAGAACGAAGTTGATCAAGTGCATCATTTTTGCACGGCTGGGTTGGATATTCACTGATAGGGGATCCTCGCAGAGGATGTGTTGGCAGCGAAGAGTGGAGGAGTAGGTGCTTAGAGGAAAGACGGACGGCAGAGGGGGTTTTATATGGATTCGTTTAGAGGATTTCCATGGGGTGAAACGGTGTACCATCGCGTCACGGCCTGCGCAGTAGGTTTAACCGATGCGGTGATCGCTAGGAGTTCGACATGCCAAACAGTACACCTCGGAAAGCATCAGTCGCAGCACCAGCCCGTCGTCTTCGTTCTCGCCACCGACACCCAGCCATCTCGTCTAGGACGTTCACCTTGATTAGCACTCAAACGAAATTTAATGCTTATCACGAACCTTTAGCAGTGCACAGCGCCGAGAGTTTCACAGAGATTTGAACCATGTTCAGCAGACAGGGAAGGCTTGGACGGTCCCTCGCCTTATCCACTAGCCTCTGAACGACTTGCGGCACTGTACGACTCGGGTGTCCTGTGGAAGTCCGATATCATCTGCGTTCAGCACATTTCTCGTGCCCGGCTGCATTGATCACCTGTATTTGGATTAAAGTATTGCTGATAATTGCCATGATAGATATGAAAGTCGATGTTAAGTCGGTTGAGTTGC

>AT9091

TCGTGAAGATATTGCTAGCATTATACGGAAGCGAGGAGTTGACAGAAGCCGCCCCAGGACGAACGGTGCCCTGCGCATAACGGGTGACACCGTCGCTGGCACCGTAAGCCCGCCAGAAGTTCAACCTAGCGAGAAATCATTGTCAAATTTCGATAGTGACACGTACGGGGACAAGCAACGTACTTTGGCGAAGCACCCGCGAAGACACCAGAGCGGTCCTTGAGATACTGCTGAGCGTCCGCCGGACGTGGATCGCTCCAGACGTCGGCCCAGTTGTCGTACGCGTCAATGC

>AT9095

CGTGTAGATCACCCGCTGCTTTGCTTGTAGACACTGAGCAATAGCGTACTCCGCCACGACGGTCTTTCCGGCACTTGTGTGCGCCGAAACCAACACGCTCTCATTGCGCTGTATGGCATGCACAGACACTTGCTGGAACGGGTCCAAGGTGAACTTGTATTCGCGCACGGGTTTCTCGGGAGGGACATGTTGGGATATGGGTACGTAGGGGTAGCCCGGAGGCACCGCGACTTGATGTCGCACCTACAGATCGCATCAGGAAGAGAGTACGGCTAAGTCGCTGAGCATGTACTCACCTGGTGCCGAAGCTCCAACCGCGAACCCGCCTCCACCGCGCCGGTCAAGCCCGCACTCGCAGCCACTTCGCGTTTTGCCTCAATCTCCACCTCATCGACGACTATGGGGTTCGGAGAGGCCAGGCGCGGCTTCTTGGGAGAGGGAGGCTCTTCGTCGTAGAACGTCGTAGATGGCCCGCGTTCGCCATTTAACGGGGCCGTTGGCGGCGATGCTTTGCGCTTCTTCGATGGGGCGCCATTGGAGGAATTAAATTCTTGCGTAGGAACGTCACCATCTGCTTGAAGCGAGTCGGCCTTCGGCGGGGCCTCATCCAGAAAAGAGAACAGGTCGTTGGAGTCCATGAGGAAGTATGTTCGCAATGGGAAAGAGCGCTAGTGAATGCAAGCTAAGTATACCAAGAGTCGAGTATAGAGGTCAGGATTCTAGGAAAGATAGTTGAAAGGATGAGAGCCCCCTCGAGTGTGGTACGGATGACCGTAGCGGTCT

>AT9097

TCCTTAACATTGTCAAGATGTACTACAAGTACTCGACACGTCAACCGACGGCGAGGCACTACATAGCACCCTCATAATCCGACCCCGCGCCGTCACCGTACGGCAACACCTTCTCCGCCTTGACGCATGCTCGCCACCACCGTTCGCCCTTCTTTCCCGCGTCATCAATAGCAGCACTGTCTTCCTGGGATAGCGCTGAGATCATGAAGGCAGCGGTCATTACGCCGCACGGCGGAGACATATGATGAGTGCACTTAAGACCCACCTAGGTCCCCAGCAGCGAAGAATGCGTCGACACGCTCCTTGTCTCTGCTCGACGTGACCACAACAATTCCCTTGGACTTGGCCCACGCCATCAACACCTGCTCGGGCGTGCAGCCCAGGCGTGTTGCGATCTCGCTAATCGGCTTGTCCACCGGGCCACCGGGGTAGGCTCTGATTGAGGCAAGTGGGCTATATCCCTCGGTGACTATTCCATGCTTGTTTCCGAACTCGATGACTGGGAGCTGGGCGGCATAACTGTATGGCTGGACTTGAATCTGGGGACGCAATTCAAGAACATGAACTCCATCCGCCTTCGCGGGGGCATTCGGACAAGGTCGACTTTGGGAACGCATGCTCACCTGGTTTACAACTGGCTTGATCTTCGCCATGCTTAGGATAATCTCCAACTGATCCACGTTGAAGTTGCTGACACCAATACTCCTAGTAGTAATGTGTCGATTCTGTAGTCAGCAAAGCATTGAGTCAGGACTTACTTAGTCCGACCCCCTTCTTTGAGCTTCTCCATCTGGGTCCACGCGTTCGGAATGTCAGGGGTAGCATAACGAGGATGATGAATGAGATACAAGTCAACGTATTGGACCCCCAGCTGAACGATGTTTGTAGATAAGCGCACGCAAGCTATGAGCGTTGGTAGAAGCTGTGATACTCGCCTTCTTCAGGCTCTGCTCGAACGCCTCCGGAATGCTGAGCTCGTCCCGCCCAGAGAACTTGGTCGTAATATAGAAATCTTCCCGAGCCAGGCCGCTCTTGCGAATGCCTTTTCCGGTCTCCTCTTCGTTCTTGTAACCTTGGGCAGTGT

>AT9105

CACAGTCAAGAGCGTCGGCTGGATTGCGAGACTGTGGGTGGTATGGTTGATAACTGAGACGGCGGTGGCCCAGCCGGTGAAAAAGAGCAGGAGCGGCCAGCAACGGAAGACGGCGGTGGCGAGAACGGCTGCAGCGAACCTCTCGATTGAATTTCGCTTGCCGTGGCCTTGAGCGAAGGGATCGACGGCGTACATTGCAGAGGATTCCATGGTGCCGGGAACGATAAAAGAGTGACACGGAAGGGATCAGGGAGCACGCAGCATGCAAGGGGTCGTAAAAAGACGAGGGGGAAGTAATGGCCGTCGTGAGCGATCGTTAAACTTATAAAGCAGGAAGATCTTGGCGGATACGCCCTTGTCCAGCTTGGCGGCGCAGGGGAAGATGTCCGGACTCCTCGTTTTTGCGGTGACCAGGGAAACTACCCTTTCAAATCGATCAACGCCGAACCGAGACCCATCAAGC

>AT9107

GGGTCTCTGGGGGTAAATTTTCGTGATACATAGATACATCCGTACATCCGTGAGAGATATGTTAGTAAGTAGTAGCGGTACCGGTATAATCGTAGGATGGGCCTGGCGTTACAATGCAGTTCGGGTCCAGAGGGATCATGCATAGAGCGACACTGATATGCGCCGGTTACGGCCACACTAGCTGCCGCCTACTGCTTCTTCGTCTTCGCCGTCGCCCTGTTCGCGAGCTGGAGCGTCGAGGTGATATCGCTCCCGAGGCGCCAGACGATATACGCCGGGAAGATGAGCCACAGGCTGAGCCGTGGCAAGGGTTCGGAATTCGGGAATTGAGACGGACGCCCGAGTGGTCGTTGACATTGCATTGACATCAGCCCGTACGGCATCCCAGCGGTGCGTGCACAAGTTCTCGTCAGCGAAACGCTCTCCTCAAAACTTCCGCGATTACGGCAGGAGCACTAGCACCGGACGAGACGGGCCACTCACCCATTCGGGACCATCCAGTACACGAAGAGCGTCTTGAAGTCGTTGTGGCCAACCGCACAACCGTTGCAGTAGTACTCCTGGAGCCAGTAGAGGATAGTCTTGGAAAGCGTCATGGTCGCACTCGCGAACCCAAGGAGGGGCG

>AT9126

GCGGCGGATGGTGCGGACTTCACGATCTCGAAGGTTGCTGACTTGATGTGCGTAGAAGGACTCCCAGGCGAAGTATTTGATGAGCCGGATGCCAGAAAGAACCTATCAGGTAGTGACCGCATTGGTGAGAAATTTATTATCGGTGAATAAGTAGGAGAAGCGGGATCACACGTACTTCGCTTGTCATGTGCACGCGCTGATCTGTGAGAGTGACGCCTTTCAGGCGCTGCTTGAACATGACTTGGGCCAGGATGAACTCGATGGGTCCGCCC

>AT9129

ATGTATTCGCGGGTACTTGCCCGATCGCGATATTTACGAGTGTACAGCACATGTAGATGTCATGAGGAGAGTAAGCGTGCTGTGCGTCTAGTCCGTGGAGTGATGCGTTACGGATGCGGAGGTCGACGGGGCAACGGAAATCTGGCCTTGAACAGTGGACCGAATCTTGACAATGTCCTGTCTCGACAGATTCGCTTCGTTGCACAGTGAGCGGAAGATCGAGTCCTCCTTGTCGAAAAGGTTTAGGGGAGTATCGAACTCGGCGACCCTGCCCCCGTCCATGACAAGAATGCGGTCGTAATATACTATTTGAGGGATGAATTTCTGCCACCATCATGAAGCGAGAGAAGACAGTAGCTCACCGATGGTGTTCAGGCGGTGCGCAATGCACAGAAGGGTCGAGGACGCGAACTCCGTCTGGATAGTCCGCTGGACCTTCGCGTCTGTCTCAACATCCACACTGCTCGTCGCCTC

>AT9131

CTACTCAGGGTGTAGGTCAGCACAACACCAATCTTGCTGGGATCGACGGTGTGCCTAAATCCCGAGGCGAACAGACAGATACCTAGAATAAGCATGTTTCCGAGGATGTCGAGCCGCACTCCGAGCCAGCGCTGGATCGCAATGGTCATGTAGTATGCTCGGTTCTCAACATCTTGGCCTTGCTCGGCGTTCCGGACAAAACGGTCCTGGGAGCGATACGCACGGACAGTACTCAACCCAGTGAGCGTTTCTTCAAATCCATGAGCTTACTGTCATCAACCAGAAGAACGGTCGAGGACTTACCTGTATACGATGCGTAGAGGACGGACCGCAGGTTGGAGTCCAAACGCTTGGCTTCCACCGAAGTGCGGCGGTAATAGACGGCGGCGGTGTAGTACAAGACGATCATGGGTGCGAAGATGATTCCCAAGTACGGGAAGGTGTAGAACACCAGACCAGCAGTGCCAAAAACTGAGCTAT

>AT9148

TACTTCCAGGCCGTTGGACGCGCAGGCTGCGACGATCCAGCGTGATACCGTCTGTGCTTATTTTTTCTATGGCCATTGTCGCATCCTCTCGAATGGCGAAGGAGACGTAGCCGACGCCCTTCGACACGCCGGTGGAATGCTCAAGAACAACGAACGCGGTTCGCACAGGCGCAATGTCAGAGAACAGTGTCTTTAGGTCCGTTGATGTCGCAGTATAAGGGAGGTTTGATACGAAGAGAGTAGACCTGTGCATCTTTGAGAACAATTTGAAAGCTATGAACGAGTTACGACGTACCCATGGTGCTTCTCCTGCCCTTCGTGCTCATCCTCTCGCTGCCCCCGCTCCTTTCTCTTCCCCAGCGTTGACATAATTCGCGTATTTTGCCTCAAAGCGGCGCAGAGACTGAGCAGAGTAGAGGACCTTGGAGGTGAAAGCAACGATCGTCGAAATGTCAGTCACCCATCCAACTCCTCGGTAACGTCGTTACCTTAGACCTCAGATTGAGACACTTCCAC

>AT9151

TCCGGGTCGGCACTACGTGGTAAGGGACATGTCAGATCATGCGAAGAGGGTACAGTTACAGTTACAAGAACACCGAGACCAGCCTGGGTCGGAAGTATCGACATCCGGGTACCTCTACACAGCAAACGCCATGGGCGTCCCTGCCCTGCTGTAGACAGATGGCCATTGCTTTGACTCCTGATATGCCCACATGAGTCTCGGGAAGTGTACAATGCGTACGTGTAAGGGAGAAGCCCTTACCTCCTCAACAATAATCTCTTC

>AT9178

AAGGCCATCGCACGGTTCCAGGGAAGGGATGTGGGAAGGAACGGGCATACGGGAGCAGCGAAGACGGGGGAGAAAGCGGGTCGCGTTTGAACACTGATGCAAACGTGTAGTGAACGACGCGTCGCAGCTGTAGCTAAATACACAGAGTAACAATATGCTAAGACATAATCCGCAGTATAAGACGGGTAAGACTAGGACAAGACGGATAAGACGAGTAAGTGAGACGCGAGCACGGAGCAAGCTCGCTTACATAACATAAATTCCAACAAGGGATTCCCAAGTGCCAGTCGCCGTGAA

>AT9191

GTCCCGTTCACCGCGCCCGGGAGAGTCCTCCGCATTTCGCATTTGAGTCGCAAAAGGGCTCAACGGAGGCGATTTCCATTGTGCCCAACAACCGAGCCAGATATAGAACGCGGGCACTCCACCCCGAAACACTAAATCCCTCATCCCTCATCCCGAACGCTTCCGAACTCGTAACGTAAGGGACTTCGTTTTAGAGGATGATGGCGATGCAGCCAATAGAGATCAAGCCACCCTGT

>AT9192

GGGCCGGTGCTGCAGCTGCTGCCACCAGAGGGGGTGGAGGCAGGAGCAGTCACGGTGATCGTCGTGGTCTTGGGAGGGCTCGGGCTAGCGGTGACGGTCACCGTCTTGGTCGTCGGCGGTGGGGTATACCCGGGCATGGCGGCCACAAGGATGGTGAGGGAAAGGATGAAGTAGATGGAGGTGACGACGCGAGCGAACATGTCGGCGATGCTGGCTATGCTGCTGGTGAGGCTGCTTTGGTAGTCCGGAGGGTGTGTTCGAACTGAGGAGTTGCGATGTTGTCCACGACGTTTTATACTTCAGCATCGGCCCCCTTGAAGCCCAAAGTGCCGCGGCACGTATTCACCTTCCAATCATTGGGCTCGAACGTAGTCCGGCGCCAGCCAAGAAATTTGGACAGAAGAGCCAGATGGTCCGTGTGCCAGTGCTGCTGCCGCTGAATTGACGACCTTGAGAGAGAAAGGGAAAGGTTTGAACCCACCCGGCTGTTTCGCGCCCATCCGACTGTGCTGGACGCTTTGTCAACTTCGCTGTCAGGTTTTTCATGATGGGGATTCGAGATCATACTTTCCCCGGACTGGACCGTTAATGAGCTGGTGTTGAATACTTGAGTACTCACCTCGGTAGCGGGCGATCGAGGTTCCATTTCCGCACCGGGAAAAGGATGAGGGGCCCGGGTCGAGTCAGGTTCTCCTGGATCGATCCGCCTTCAAAAACTGCGTTGGCGATCCTTCCTACTTACAGTGTCACTGTCAGTGTCACGATGCCAGAAGGGCACGATGGCTGCAAGCAAAAGTTAGGTACTATGGACGCGACAGCCTGACAGGCCAAGGCGCCCTTGTCGCGGAACGAACAGGCCAAGGAAGGC

>AT9197

ACTGCGGGAAAAGCCCTGCTAAAAGAGTCCACTGGTATATTCGATCATTCGAACACTAGGAGCGAAACATGAAACATAATGGTCCTGATCCTGCTACGAAGCTGGGCGCAACGTCCTCCACTACTCGCCCGACCGTGCACGTCGAGCGTGTGTGTGCGTATGTGCGATTAGAGGCTAACGGGGAGGCAGCCGATGGAGATGAGACCACCCTGTGTTCAAGCGAATAGGTGAGCATGTCGGAACTCACGTGGGTCTAGGCTTGACGAAAGAACACTCACGTTGCTGTTGTCCTGGCAGCACACAGCGTTGGACGAGCAGGCGTCGCTGCCTCCAACACCGATGACGTTGATGGGGGAGCAGTCCAAGCCGACGGGGACGTTCAAGTCCGAGAGGACGATACCAAGCAGACCGAGGATCGCACTCGCGCTTGCAGAGTTGGCCTAAACAATGAATGTACGTGAGTTTAATGGTACGCGAAGGGGTATGCTCTGCTGACTGACAGTCGTGACGGTGTTGCAGCATTGGACGGGGCCGGTGCTGCAAGACGAAGCGGGCTCACCGCCTCGGACCTCGAGGGGGGTGGCGGCCGCAAGAACGGGCAGAGCAGTGAGAGCGAGGATGGCGACGCGGGAGAACATGATTGTTCTTGTGAGGAGAGCGTGGACTGAGGATGCGAGAGAGCTGACTGGTGTAGACCTGAAGCTTG

>AT9206

GTCTCCAGCGAACGAATGGAAGAAGGGCCGGTCTTGAAAGTACTCGAAGCCCTCATAGAGCTCATGCTCCCAGATAATGCCGCGAGTGCCGGACAAGTCAACGAGCTTGAAGTTGAACACACCCTGCTGGTCCTTCACAAAGGCAAGGGCGCCCTGTATGCCCGAATACGACCACTCGTTAGGGTTCGGATGGGCGTAATATATGCGTGCGAGCGCAGCAGTGAAGATCTTGTTGGCGGGGGCGTTCACGGCCGCCTTGACCTTCGACTTCTCGTCGGCGGATAGCGTGGATTGAGCAGGCATCTCGTATGCGACAATTGGAGGGGAATGTGGAGAGATGAAAGTGGGTTATGGGACAGGGAGCGCAGACCAACTGCGCCCCTCGACTGCACAGGACAAGTTGTGGACAAAACGGCA

>AT9209

GCCTCCGCAACCGCAGTCAGACCTTGAACGTCAAGCTTGTTCTCGCTTAGACTCAGCACCTTGAGTGTCCTATTCCGCTTCAAGACTTGGGCGATATAGGTGATACCATTCCGGATGTCGTTCCCCTTCAGATCCAGCGTTTGCAGAGCCCCCAACCTTGGCAGGTTGTCAAGAGCTCGAACCTGCTCCAGAAGTGCGGCACTCGGGCCGTGCTTGAGCGTCGTGTCGAGGCTCCGTCCATTGGCATGCGCAGGATGCGACGGTGGTGGTGGCACCGGATGACGCGCGGTCACACCGCCCTGAGCGGAGGAAGTGATGATGGGTATAGGCTTCCCAGAAGCTGAGAGCGGGTTCGTGTTGCCAGGGGTCGGCACTCCGTTTGACGCTCCGCGTCGAGCTCGGGGTATATAAGGAGTGTACGTGGTCTGCGGCGCTGCGGATGGATGTCGTGGTGGTGGCAAGACAGGTCCTGCTTTCGGAGGCCCATGCGGTGCGCCGCTGGGGATGGTCAGAGTAGGAAGTGTTCGTGTCTGGGGGATAGGCGTCGGCGGAGGGGAGTTTAAGGATAAACCGGACGTAGATGTGGCCGGGGACGGAGGACCAGATCCATTCTCGCTTGTTGAAGGGAAGCGATCTGGATAATCCTTGATCATGAGCGCAATGGCGACGGCGCCAGTGGCACTTATGCGGTTATGTCGCAGGGAAATGTTTCGCAACGACGACTTTCGTACAGAATGCGCTGCAAGCAAATGAGCAGGCATCAACAAACCACACTCAAAAGGCTCACCCAGCGCGTCAAGTGCTTGTGGTTTAAGGGCACAATCGTCCAGACGTAATGAGACTAGGCCAGGGATGGGGGCTTCAGGAAGCGCAGCTGCAATGTACTCGATGCACTTCTTGTCTAGTACGTTCTGCGAGAGGTCTAGGAATTGCAAGCCTCTCGCCTACACGAGAATTAGCCCAAACAACTGCACGTTGATGGAGAATGACGCACCTTAGAAACATATGCACCGATAAGACGCAATCCGGCAGCCTTCAATCGTCTATTTGACGAGACCGACAAGAAAGTGAGTGTCCCCGGTATTAGTAGGGAGTGGAGAATTGGTTTCAAAATCTTGGAAGCTTCGTTAGAACTAATTTCTTACGGGTGGAAAGCGTCCACTCACAATCTCATCCAGATCACATTCCTTGAACACCAACTTGCGAAGCCCCCACTCGATTGTGAACACGTCGGACAGCACAGATGCGACGCCGAGGTTCAGTTGAATCCCAGAGAAGTCCACTGAGCGCGGGTGAGCACCACCTCCGCGCTATTGAATAAGGTAGTGTGATGAGGTGCTGCCGGCGAGAAATGTATGGACACGCACTTTGAACGCTACGGATATAGCGGGATCAGGAACCTCCTCGCGACCAATGCAGCACTCCCGGTAGAACGCCTCCACCTTGTCCATGCCCCACCAGTCATCATCCAAATGTGAATCGGAGGAAGTATTGTTCCCCCGAATGACACGCTTTCTCCGTTCAAGTTCACGCTCCTCGATGGACCTCTTCTCCTCCTTTAGTGACGGGGTAGATGGGGGGTTGGCCGCCAAGATTGGGTAGACCGTTGTCATCTCCGGAAGGATGAAATGAGCGCGTTTGAGCGCCGTGGTCTCATCATTGGTTGTAGAAGGCTGCTGGTTCTGGGTCGGGAGGAGCTTAGAGAGTCGAGCGAGCGAGAAGAACGACTGTTGAGGAGGCGGGGGACGCTTTAAGATAGACTTGCCAGGCGTCGGAATAGTTACTGCGGACGATGAAGCTGACGGTGACAACATAGCTGCATCGCAGAGGAAGGAAGACGACGTGCTGGAGCCTAGAGGTCGAGATGATGAGGAGAGCCAAGCCAAGACCGATTCGCGGAACCTGGAATGATCTGACCGGCAACACGTATTGATTCAGCCAAGCTCAATTGTCCG

>AT9213

CAGGTCGCCAAGCTCCTGTGTTGTCGTGTCCACGAGTCTCTTCACATCATCCTCGACGCTAACGTCGCCGTAGACGGTCACAATACGAGTACCATGTTCCGCACGAATGCCCGCCGCCAGCTCCTCTAGAAGGTCTTTGCACTCGGGGAGGTCGAACAGCCCCAGGTCCAATCCATCCTTAGCCAACCGCAGGGCGATGCTGCGACCGATGCCTTGAGCCGCCCCCGTGATGATGGCGACCCGTTTGGTGGAGGCCGACGCCATGGCTT

>AT9229

TCTTTACAGCGAAATAGCCTACATGCCAATAGAATCTACAGGAATGATGAAAACTGAGGAGAATGAAACGCACCAAGAGAGTTTCCATCGAGAACGTGCTATCGAAGGCGAGGGTTAGAACGGGAACAACGCAATATACGATACATAACTTACCTGACATAGGTATACGCTGCCGTTGGCACCCATCCCTAACCTGCAGACCTCCTGGAAGAAGGCCTTGAAATATCCCTCCGCCATGTTTTCCGCGCGGAAGGCGCCTCTGTTGGATGATGACCCGCCATTGCTGATAGGACTTGCTGGACGCGACGTCTCGTTTGCAATCTCGAGCAACTGGAAATAGTTGGCAGCCCTATTCGAAGCACGGCCAAGGCTCTCTTCGAGGTCGTCGTCGTCGGGCTCACGAGGTCGCCTGGGATCTGGTGAAATGGAGCGGTGACAGTAAGGACAACGTGCGTGAGCGTGTCGGGCCAAAGTCCCCGTCTCTGGAGGCTGCGGCGAGTGCGTCCGGATTGAGAGAGCATGGGATGTTGGGTTGTAGAGCACGACTTGGTTCGAAACATGGAGGATAGGTTGCCATTCAGGATCCGCGGGAGTGATTGCAATGAGCGCACCGGATGGCGAATACGTATCCAAGTCGTCAGACATGTGAAGGTTAATGCTAAATATTAAGATGGGAGAGATCCTAAACGGTGGTGGTCATGGCGGTCGTGAGCAAGCTGGGGGGCCAGGTGGCGGGCCAGGTGGCGGGCACACCACAAAGCTTCTAGCGCCCATGGCCTCGCTCGGCGTCCAAATGCGGCAACCACTACTTGTGGCGAGCAGCAATGCTTTCAACGTACTACTTACCCTGTCTGTCTACACGTTCAAGTTAGACGCACATAAACTGAGCAGAAGCCAGATGTTATAGGGGAGGAGTCTTTGAGTTGGCA

>AT9239

TTCAGGATGTTCAGGTTGCTGACGGTCGTGCCGAGGGCGGAGGACGTCGCGGTGAACGCCTGGATGGTGGCGATAACCGTGTCGGAGCTCTGGGCCATGGTGCCGACCGCGCTGAGGAGGACGACGGGGACGAAGGTGACGAGCTGGCTGAAGAACTTCATGACTGTCGCTGGGTGGGACTGAGTGTCGGGAGATGGCTTTGCAAAGCTTGAAAGCTTGGTAGAACAGCTGAGAGATGGGGGATGGAGAGCAACAGCTGGACTTGAGGCTGGTTTTATACTTGCAGCGGAGTAGCGTAGATGACCAGTTGCTTGACTAGGACACCACCGAGTGGGCAACCCATTGCGAGCTGCAGCTTGTGCCGCCGCGTCGGTCTTCCGAGTCGCCCATTGTGCGCCTACTTTTGCCGAGAGGCCAGACAGACAGACAGCGGCGAAGGGCGCATCAACGAACCTCCATGAATCGAGAGCCAAAACGCTGAAATGGTTGATCAGTGCCGACGCCACCATGGCCTAGGACAACGAAAGCCTGCTTTCAGGTGATCCAACAGGACAAGGACGAGCACCCAGCTTTCCTGGGGTGTGACTAGGTGT

>AT9244

CCCGTCATCAAGGGCTCTCTGGCACCGGACGACAGCCCAGGCGCACTTGTCACGACACTTCTCGTAGTGTATCGTGAAGGGGTCGTCGTCGGGCTCCCATCCTGCGAGATTCTTCCCGCACACAAAACATGCCACGTTGTCGTTATTGTCAGAGTCGGGGTTGAAGTAAAAGCCAGCTTCGGCGAGGGTCTTGGGAGTGGCTTTAAACGACGAGGGATGGGGCCATACGGGTTTGCTAGACGACGCCTTCGTTCGTTTTGACTTTGCAGGGCCAAATGAGTCAATACGGTTCTGATAGTAGTCCATCGTGGGGCCCAAGAGAAGAAAGTGTGGAGGGAGGGGGGAAGACGGACAACAGCTGAGTTACAGAGACGACAGCAATGG

>AT9246

CTCCCCCGGTTTCTGCGGCATCTAGGGCTTGACATAGTGAATCCAGATGTTAGCAAACGGCGGACAGATATGTGGCCGGGAAGCATGAAGACCGAGTACGTGAGTAGGGGCCCCAGGCGCCAGGAAAAATTTTGTCGCGAGTGCTCAGGCTGTCGTCGATAGGAATCCTTGTCGTTATTCGGTTGAACTGCCGTGCATAATGATATTGTGGTTCTGCGTTCAATTAGGACGTTACCGACGACCAACGCGAGCCGCCGAAACCAGGAATTACACCACCAGGTGGCACATGGGCTTTTGACGAACACCATCCGGAGAACGTTGACGGTAGATATTGAAGGTTGGGCTGCTATCATTGAGCGTTGTGAATAGACAACTGTCGCGTGTCTACTCACCACTGGTACAAGGCGCCCCCTCCGATGAACCCTCCGACGTCGCTTCCGATGGTCACCGATAACCCTACTGAGGGTTTCTTCGCTGCAAGAACAGCACTTCTGGGGTCGCGTCGTGAGAGGGCTTATGCTCCCGGTCGCACACGGAGGACGGAGATCTCAAGGCAGAAGGCGCGAAGAGCAAGGATCAATGGTCTGACCTG

>AT9262

GGGCTAATCTTATATTCGTAGCAAGGATACGTGAAGACCCTTCATATACTGCAATAACCAGTACAAAAGATAGTACGATACAGGCAGTATCCAGCATCGTCTTAGGCGCTATGGAAGACGCGGGAAAGTACGCCTGGAGGTCTTCCACATCGTGTACGAAGCCGCCCACATATCCATCGGGGCGGACAGCTACAAGGGTCAATGTATATCCGGCCACATGGTATCCTTGTGTGCATGGCCCTCCTTGTCATGGTACACGTCGGCGCTCGCGACAGCCGCCAGGACGCCGTTGTGCCCGAGGATGACTGTCCGCACTAACCCCTCCGGGTAAGCCTGTGCAGCTTCGATAACGACCGCAACGTCGGCGCCCTCGGGGGGAAAGACGAGCAGTGTATGGTAGTACGGCTTGAAGATACTGAAAAGGGCTGTCTCGGCTCCGTTGGCGCCGACAAGCCCGGGCGCATCAGGCGCACGGTCGCGGTGCCCCGCGTGCACATCCCCGCCGGAATAGCCCTCATATGCATGTGCCTTCATCTCATCCTCGGTGAGGCCTAGGCTCCCATGGATATCGTGCCCCAGGGGGCTCCAGTGGTAATTGATTTCGTACTGGCTAAGTGCCTGCCAATTGCGCCATTGCAGGAAGGTCCGGGGCTTGTCCTGTGGTGCCGTATCAGGGTCCTCCCCCTTTCTGGCAGCAGCGTGTCGATAAAGGCTGGATGTGGTGGCGAGCATGAGTGCCATGACGGGATGGCGTTCGGTCTCGTAGGCGGATAGGAGGGTGGGGAACGCGATGCCCTTGTAGACGAGAGCGAGCTTCCAGCTGAGGTTAAACTGTCGAGCCTGTCAGGAGTCCGAACAGGGTGGCGATGGAGAACAAATGAGTCCTGGACGCTAGTGTCCTTGA

>AT9265

CAGTTCTTCGCCCTCTTTCTCCTCATCCTTCTTCTTCGACGCTGCGCCCTTTACATCCTCCTCAGGCTTTTCCTCGGGCTTCTTCTTCTTCGGGTCTTGCGAAGGGACGGGTACGGGGAATTTGGTGGGCTCAGGCATGGTGGAGGAAGTGGAGGATGATGAGGACGAGCGACGAGCGTGCAGGATGGGTTTGCTGAGTGGACGCGAC

>AT9267

ACCGAGACAGAACATCCCTTGCAAGAGGAAATTGGAGACGGTATAGAGCACGGGAATAAGGTAACCATTGAGTGCTTCAGCAAAGGCGGGGAGCGCAACAAAGCTGCTCAGAAAGCCTAGGAAGATAGACGCGTATGCTCAACGGGGACCGGAGAACGGTGAGGTCGAGGTGAGGAGACGGACCTAGTATAGAGAGAGTGAACCATGCCACCTTGAACCCAAACGCCAGCATGCCCTGTGTTGAAACATACAGGAGCGCCTCCAGTGACGGCTAGATATTGTCGGGGCCGCCAGGGGGCTGGGGATGGGGGGAAGAGGAAGGAGAGAAGGCCTCAGGCGGGGGAGCTGGGGATGGGCTTTTACATGATGTTGTGAGAAAGCTGATATCCCAAATTCTGGACATTGCGGCGCTGCTTCGAGAGAACGGGATCGCAGCCCTTCTGCATATAGCAACTGGGGTCCCGAGCACACATGGAACCCCCGCCATCAGTCCGACACCGACAGATAGCGCGCCGTGATCTAATATATATACGCTACCCTTCAGATTCGCGCGCCAGGCAAGAGGGCGCGATCTCGGAGTTGGCCAGGCTATTAAGTCAGAGAATGGACCCGATGGAGAAACACCAACGTCGAGAGGAACAAGGTGCGCTATGACGCCGC

>AT1541

CGCCGGTGGAGGTGACGACGACAATGTCGTCGGCCGTGCATAGCACCTAGAGAGATGACAGGAAAACACTGTGAGCCAACATCATTCCTGCGCAGGCATCCGCAACCTGTGCACACACACCTCTCCCGGATAGATGTTCGAGCACTGGCCGTTCACGTTGGGGTTGTTCGCAAGGACGAGGTCGAACGTCGTCCCGGTGCTGGCCGCGACGGACTCGCAGCTGTCCCCCGATACGACGACGTGGGTGACCTCGCAGTCTTGTCCCGCGATCCCGAGGCATAGCGGCTGCATGGCACATGATAAACGTCGGTATCGAGCACAACCCTCTGCCAGACCTAGAGCTCGACGCGGCGTACCTCTCCTACGGCCAAGTTGCTGCAATCCGCGTCGATGGTCCCGTTGTTCACCGTC

>AT493

CCGGTGCCGATGACCGTCGTCACAATCGCGCTCGCAATCTTGTCGTGCAGCTGGTCGGGCTTCACGTTGCGCGATAGCAACACATCCTTGATGAACTCTTGGACTGGGTCCCAAGACACATAGTGGAAGGGATCTGCGAGATCCATATTTGCGAGGCTTTTGGCCAGCAAGCCCGGGTCTACGCTCGAGGTATGGAAAGATGTCAGCCGGTTTGCTGGAGATAGGAGACAGTTCAGAATCTTCTTCGATGGACAGGAACCGGGCGGGCTTACTCCACGACGCTCTCCATAGGCGCGTCTCTCCGACAAATGCGGCAATATGAAGTATCTCTAGTAGTTCTTCCGTGGATAGGGCTTTGACGATGGCAACGAACTCGTCGCGAAACTGTGCCACGGATTCTTGATCGTAGTCAAAGTCGTTGTCGTCGCTGGGGTCCTCCTCCTGCCAGACGGCATTTTCCTGTACCATGTCGAGCCACAGCCAATAGCGATACATCGCCCTGTCGAAGCGCAGAGCCTCTTCAGGAGCCAACACGCACTTGAACGATGTGCGATCCTTCCTCCTAGAACCGCATTGTGGCGGATGAGAATGTGGTGTACGGGAAGAGGAAAGGAACAACCATACCTCTGGCTGTAGAAGTTGCGCAAAACCCCAACTGACCACGCATTCTGTTCCACTGTCGGTATCAGCTTCGGGGACGTCGTGAAGTCCAATGTGGCGAAGTGGTTTTCATCAGCAAGCCCGGTGATATCACCTCGTATTACTAGAAAATCTTGATCCCAATGTGAGCACTGATGCTGGTACTCTGCCAGACGCGCGGCCGGGTGCAGACCCAGGGCCGACCCGACGACATTCTCGGCCACGGCGCGCACGATAGACTTGGGGTGGGCTTGAAAGACTTCATGGTGCGCCTTCGAGATCCGGATTGCTACAGACAGGGTGGCGAAGTCAGGCGCGTCTTCCAATATTTCGTGGATAACGTC

>AT1009

GCCGGGGAAGAGCTATTATGATTACACATCCCATGAGCACACTTCATTACGACGATTACACGCTACCCGTTATAGTCTGAGTACTGACACATGGATCCGACCTAAACGCGAAACGAAACGAAACGGTATGGGTATTCAGCGACCTTTTGCCAATATACCTGCCCAGTACGATGCCTCCGGCGTTTCCTGGACGGCTTCGCCGACTGCGGCTTCCAGACCATCGCCTGCGCTGGGGGTTCGATATCCTTCGGCGATGATGCTGGCGGTAAGATCGAGAACCAAGCGCCGCAGCGGCGATTGAGCGGGCAACTTTGCATATGGTCCAGCAAGCTTTCCGCGTGTCGGATGCGTAATAGAGAAGTACGGAAGGAGGCGGGAGCGTAGTGCTGCGG

>AT1013

TCGCCAATCAAACGCACACAGCCAGTTATCAGGGGTGTGAGGTGTGCTAGGATGAGATGAGGATGAATCTCCAGTAGCTCACGTAACCCAAGGATAGCATCTGAGAGAGGTGAGCCTCGTTGATAGTTTCGAGAATATGCCATGTCACCTCTTCGAGTGCTGGCATTGTAGTGCTTCAAATGGACGAGGAGTGTGTCGAACGTGAGTTTCCGTTTCGTGCTGGGAACTTCCACATCTTTCTCGATAGCAATTTTCTGCGTAGGTAGGGC

>AT1028

GGACCTGGTTCCTGCGCAATGCTGGGCGCGCGGAGGTGGTCGGCGCCTTGGGCATGGCGTCGTCTCAGGGTCGCGAGTGCGAATTGGAAGGTAGCGCGAGAGAAGGAGAGTAGGGGGTCGAGCAGACCAGAATCGCAAGCTGGACGGATGGGGGCTTGGGCGATTTTCCGCGCTAGCGTATATATGAGCGGGTGGTGGGAATAGAGAAGCGCGGTCGGGAACTGGCGAGAAGGGGGATCCCGCTGGCGTACGCACCCAGAGCCTAAGACGGCGCTCGTCGAGGAACGAGGGAGAATTCAAACCGCTCCTTCTCTCCACGCACGGAAATGTGTATACAGGATGAAGCGTTTTTGATGCCCTTTCCTTCGTCACAGTCTTCCGCCCGCTGGGAAGGCACACGGTAATTCCTGGGGCCTGCAGTCTAGTTCCGTCCGGTCCAGTACCAGGTCTGCACGATATCGGCGCGAGCGGTCGCCAAGTTAGCTTCCGGGCTTCCGAGCCCGGGCGCTGGATTCGGAGGAGGACGACTGGTGCTGTGGACGAGATCGGGGTCACCGAGCGATGAGTGTCTCTAGCCGAACCGGCGGCGGCCTGTCAAAATGACGGGTGTGGCGCGGCAGCCCCTAGGGTCCCTGGACGGGCGTGGTCTCAGACTGCAGAGCAGAGCGTGTGGAGGCGGCGCAAGAGAGGTTGAGTTCAGGAGAGCCCGGCACACCGCCATATACCCCCCCCCACTCGAGTCCGTGACCC

>AT1030

ATGGTGAGTGCTGCAGAGGTGTGTTGGCTGCGACGTTTTCCTCACATCAGCGTCGTGTGCGAAAACGGTACTCCTCCTGGGAGCCTCCGGAGGTTTGATCTGTATAAATTTGGGGCACATACATGAACAGGTATAGAACCCCAACCTAAAGGCGAAAAGGACAATGTCAGCAGGATTCGATCGCGGATAATGTGAGTACCCTGCGTCAATAGGCTTCCGAAGGATACCAAGCGAGCCTGCTGCCAGCCGGCGACACTTGGATTCAAGGTCGGGAGGTGGAAGGGACGCGGGCCGCGGCGGTATTGAGAAGCTCCTGGATAACACATCGTCGCCCGGGCGCACGCAGTTACCACAGAGACAGATAGAAAACTGATGATGACCTGGACGCCTCGCAACCCTGGTTCGATCTGGCCCACCACCTCGTCGGTCACAAGATGACACCCTTTGCTCCTGCGCGCCAGCGTGAACGTCTTCTGCCAGCCCATGAATAAGTGCGAACAGAGAGATGGAAGGAGGATGGGCAATACAAGGAAAGCAAGGTCTATCGTTA

>AT1045

TCGGGGCCTTTCTGTGCAATCCTTCAACTCCCGTGAGCCTCGAAATTGTGAAAGCAGTACGTACCGTGTCCAAAGCGATGGCGACTGGGCGGCCCTGAGGATCGACTACAAGCAAATGAACATCCGCGTTTCACTGACACCGAGGAAAGAAGACATACGCGACACCATCTTCCTGGTGTTGTCGATCACGCTCTGATGGTACTCGTACTCTCCATGGGAGAAGTTCATCCGAACTGGAAGCGAGTGAGGTTGTATGCGCGCATAGATGCCATTGAGAACGCGGGGGTATGCGGGCCAAGAGAA

>AT1046

ACCTTGCGGTGGTACTTGGTCTCCTCCGTCGGTACCTGGGCCTTGGTGGGGTCGAGCGAGGCAGCCCATTCGATGCGAGAGGTGATTAATTCGGCCATGACGAGGACGAGTGAGGCAGAAGGGTTGGGGAGAAAGGACGAAAAGATGTGCGATTGGGTTTATCTACGGTGGGAGGGGCGGAGCTAAGGTGGATGTGATGGTTCGATGACACTGATCTAGGTTATCTAGCCTCTCTTTGCGCCCGCATATGGCCATCGACTGCTGCTATTCGTCCAACTTCGAATTCGCACCACATTGGCCAAGGGATCGCCCAGGCCATTACGTCCCCACGAACGTGTCTGGTCCCTTCCTGCGT

>AT1062

CGGCCAGGATGGTGGTGAAGAGGAAGAGGTAGTGGGTGAGTATTGGCGTGAAGTCATAGCCCTCGCTCCCGCGTGGTGGCATGCTGGAGGCAGAGAGGGAGTGGTATAGAGCACAATGAAGGAGAGACGGTTGAGGCCAGGGGAGGGAAGAAGGAGAGGAGAGGAGGGTTGGAGAAGGGTATGCGGGAACGGAGAGCAGGGAGTTGAACGTGAACGAC

>AT1071

ACCAGTGAAGTGTCGCAGCTGCGCTTCCTCTCTACTTCCAGTCTCCGCTAAGATCTCCGATGCATGCATGCCATGTAGATCCTCCACAGTATGCGTGTCAAAAACACTTTTGCCACCCCGAGTCGTCGTCGCCTCAGCATGGACGGCGGCCGAGCGCGAGAGGTGTCGACGTGCAATGTCGGTAAGGAGTGACGAGGCCCTAGAAGGAGTTAAAGTTGAGGATGGAAGGCGCTGCGCCCGCGCGAGCTGACGGAGTAGCGTGGAGGCCATCCTCGTCGCTGGCTGGAACGTGGAGAAGAGGCTCTATCGTCGTCGTCGTCGTCTGCGTCGGTGTTCGCTTTTACTCGGTGCTAAACGGACCGTTCT

>AT1077

CTAGATGCCCGAGAGGGATTGTCGTGCCAAAGAGGCCGGGGACTGTGAACTCGGCACTGACATCACCACATACGTCTGTAGCAAGGACGTCTGTAGTGGTCGCCCGTTGGCGCAAGGTCGTCGGAGTGGTCCGAGCTGCAACGAACGAGCTGCCCAAGGCAAGTAGAGGGAGGAATGTGAACAGACAGCCCATTTTCATGAGGAGTATCCCTGAGTGAACGTTGGGCGAGGAGCATGGGCTAACCTGCCGTGCTCTTTATACGCAGCTTTGACACCACGCCTTCCGAAAACCCGGAGGTGGAGGAAAAACGCAGGTCCTTGAAGCGCACAAACGACACCGAATCGC

>AT1101

GCAATGTAGGCAGCTGCACGGTTAGAGTACAAGACCGCATTGCTGTCATCTAGCTTGATAGCTTGGGTGTATTTGGATCGGGCCGCGGAGAAGTCGTTTCTAACATATAAGGCATTTCCGTCGGCTTTCAGCTGTTCAGCCCGTGTTTTGGTATCGTCCGATGCCATGACGGTCCTCCGCTAGTCGATCAGTGGACAACATGAACAACCGGGATGGAAGACGGAGCAGGATGCCCCGGAACCTGTTCTGTGCTTCCCACTCCCCGCTCT

>AT1102

TCGGTGGATTACTGACGAGCACGACAGGAAGCGCGGTAACTAATGAAAAACAAAAAACGATACAAAAACATGCGCATTACGGCGACTCATGGCGATGACCCAAACGCATGCTCGAGCGATCCAGTCACTACTACTACGCGAGCGTGGACTTCATACCCGAAAGTGCGGCGTAAAAGGGAGGGAACGATCAACACCGAGACGACGCTGCTGCGCGCAATTACAAACTAAGAGAACATCGCGGACAGAATACTGGGAACATAAACCTCCACCCCTGACTGAGTGCTGAGTGCGCGCCAATTCCTTTCGTTCCAGCCACCCGCCGTACGACACATCACTCCGGACGGAGAGCGCTCACTCGCTGGCGACGAGCCCTGCCATCATTCGTTGCACGTCTCCAACGCCGTTGGGTGCGCCGCCCACGACACGAGGGGGAAGGCCTGGGTGCCTAACTCCCAGCGCCGCGCCGAGCATCCTCTTGCCCGGATCGCCAATACGCGGAGCGCCGTGCTCAGGCTGAGCGGCGAGGACAGCAGGGTCAGCGGCTGTCGGATCAACGTGCGTGACATCTCCACCGAGGGATGAGGAAATGAGTGCGTTGAGCGTGGGGTGGGACGGGAGGTTAGGGAGGGTGGGGGATGGCTCGCGGTCGCGCACGCCCTGGTCCACTCCGTTGCCAATCGTCTGAGCGCTGCCGTTGGAGGTGTTGTTGTTACGGTAGGCGTGGCTGACGGAGAGGCCGCGGCCATGCGAGACGGAGTTGCGGACGTTGTGGCCGCGCCCGGAGCTGTTGTGCGTTCTGGAGTTCACGGACTGAATGACGTCCTGAGCATCAGGGCCATCGTACGGTTTGATGATAGCGGTGCCTGAAGAGTTAGGGGAGGTGAAGATGGGAGGAGGGAATGCGAGAGCATGCAAGTACGCCCGCTTAGCTGTGAGGCAGACATTACACCAACGTTAGCACCCGGAAGACAATGTCGATAACGATGAGGACGGTATTCAGCCGCATATATGAACACTCACCTATGGTCGCGTCATTAAATACAATCAGGAGGCTAGTGTCATTAATCCACTTGATCTTGAAGCCACCGTTAACGCCATCCCACTCCGAGAAGGCGGCTTGGATGTCCCTTGTTTTGAGCTCCTTTGGGAAGCCGGTAAGGGACAAGATGCGAGTGACGCAGGCGGAGAGCTGGGAGAGAGCTGCTGCCATCGTAGCGCACGAGATTGTAATGTGATCTGTGTAGAGGAGGGGGGAGCTTGAAACGGGCGTGTGGTTGCTGTGCGAGCGGCGGGTGGAGGCGAGTTGAAAAGAGTTAAAGGATGATAGAGCTATACGTTAAGCGGGACAAGACGGCGGAGGAGGAGGAAGGAGCGAGGAGATGGACGTGCAAATCTGAGGAGCCTCAGCAGAGCCAAGAAGCGCACTGGGTTTCAGGCGCCGGTGCGCATCAGGCGCGGTCCGCCTAATGCCATTGCCATTGCCACCACCCCCACGCCTTCCTTCCATCGTCCGTTCTGACGGACGACCACGCCCTCTCGCCTTGTAAGGTACCATATACATACGCTACAGCCGAGCACAACGCTCCTGCGCACATCCATTCAGCCCGCCGCTGCCCAGGAAACTGGCACAAACCCTTCCTTTGCGACGCCCGGACTCTGGCAACAAATCCC

>AT1127

ACCGCGACCACCCACATATGATAATATCGTACCATTCAAGTTGCTTTCTTCATTTTCTATTACGGGGGACGTCAACATTAACCGTACGGACCCCGCCTGTCGACTAGGCGGAAGGCCTCGTCGTTATCTCACCACTTGCGGCCTTCGGGAGCCTTGCCGGCCTCGTGCAGGCCGCATATATCGCCCTTGGAACGCTCCTTTATGATACCGCTACGCGTTGGCTATTACTTGTGTAAGTTATGTTTGTCGTATCCTGATCATGACACTTCGCATTCATTTCACACGTCGCGTCGTCCTTCGAAGTTTTATTTTATATCCATGCTGCATGCCCGGTGCCCGTCTCGCGTCTCGTAGATGTGTCTTATTAGTAATGTGATTGTCCCGATAAGCCTAGAGCCCAAACTTCAAGGAGACCGCTATATGCTCCTGTAGAAAGAGTGCCCTGTCGAGTCCTTGCTTGCTGCAGAGGTACTTGAGTAACACGCTTTTAGAAAGAGTTCTCCGTTGAGTCTTTGCCTATTGCAGAGGCGCTCGAGTTGCCGGCGGAGATCGTGGTCAGAATGCGGTCAATGTAGTTGGCACCACTCCTATGAAACATAGAGCATATCAGTTTCACGAGGCTTCACGACAAGTAGTTTGGCCTTGCAATACGTACCACTTTTGGTGCGTGAGCACGTCGCAGCTGATCTCCTTCTCTTTCTTCTGGATGAGGTTGACGTACGCGAGCATACCGTCAGTCTTGAATGCCGAAGCGAGCTCCGCTGTCCACGACGT

>AT1133

AAAGGGCGCATCTCGGAATGGCACTGGTTCGCAACACCCTCAGTGAGCAGACGGCGCAAGACGAGGTGGAACATGGGGAGACTCACCTCATGACAATACCACATAGGTTCCCTGGAGGACATGGCGAATAAGGACGAGGAAGGAGGAAGGATGGTGGTTGGAAGCAAGCAGGGCAGAGCAACCCAGGCTGTCAAGAACAGGCGAGGAAATGACGTCGAGGC

>AT1148

TACTAGGATGATTTTCTTGAATTGCTCAGGACGGATGTAGCCATCCTGATCCTTGTCTAGGAACTTGAATGCCTGGCGGAGTCGTTCGCCCTGGAGCCCCTTCATTAACTGCGTGAATTCGTTGTCTGCATTGGCGTCAGTCACAGGTGGAATAACACTAGTATTCGGACGACATACACCCGAGAACGTGCGCGCCGTTCTTCTTCCC

>AT1150

TTTGAGGGCTGGAGCCTTGAGTAAAAGGCCGGCCTTTTCAGGAGGAACTTTGGCCGCAAGGACGGGGATGGCCTTGCGAAACGCGTCCTTGTCAAGAGCACCATCCCTGAGCCCGAAGCCACGGCCCTTCGGAAGAACAAGCTGTTTGCTGATGCCTTCGGACATGTTGTGAACGGCAGATGTGAAGAATCGTCGGTTCAAACAAAACCGACCGATCATCTGCAAGAGCACGACGCTCTAACGACAAGAATTTTGGACGGCGTGCGCGGCGGACGACGAAGAGGTGATCAGGGACCAGGTCGAGTTAAGTAACTGTAATGGTTGGGTGGGTTGTCACGCATTCGGCATATGAGCGCCCACAGGTT

>AT116

ATACCTCGTACACGACGTTGGTCCTCAAAAGAACTGAGCGCTTCAGCTTGAGCCGAATGGCGACCAGACTTTGTGCAATCTCTGCTCTCGACACTTCCTGTTGTTCTCTGGTGTTCGCGAAGGAGACGATGGAACAGTATACCGTCCCTAACTCTGCGACAGTGGTGGCCATCAAAGAGCGTTGATACTTCCTCAACGTTGTCGACGGTGGCAGG

>AT1171

ACCGGGGCGCGAGCAACGACGAAAGGGGGGAGAGCCATTTGGATACTGGAGGATGAGCCGTTTGGAGCGGAGGGGTGTGGGTGAGAGAGTAAGGAGTTGTTTGAGTGGGGGCTGCTGTGCTTGCAGGTGTGGCTAAAAGTCCACTAGATGAGTAGTGTGTCGATGGGGAATACTCGAACGCCAGAGTCCCAGCCTTAAGTAGCACGCCCGCCATCGTACCACCTACGCAGCCGCATAATGCATTCAAAGCATGTCTTGAGGCATTCCGAGTGGTAAAGCAGGACAACGGTAGGAGTCGGAACACATCGCCCAGCCCAGGACACACCAGCCCCAAGTAGAACGACATAGAACAGTAGTGCATGCCTATAGCACCCAGAATGGCAGCTCGCAACAGCTCC

>AT1176

TTGGGGGTAGTCAACCTTGGCTCCCCTACGAGCCCTGCTGACCCGGGACGTCTGCCATAAGAGAAGCCAGAAGGTGGAAACGGCTGCAGCCGCGGGGTACGCAAACCTGTCCAAGAGTAGAGGAATAATATTGGATGTGCATGAGTGCAAGGATGTGTTTGCGTAACGCACTCTTTGGGAAGAACAATGCCGGACATGATCGTTTGGCAAGCGAATGGATGTAGAGGTGCAGGAGAATTTGGGATAGGAAGAAAGGTATCGTGGGATGTGCGGTTGGCGTTCTTCACAGGAACGGCTTCTTATACCAGTGGAAGCAACGATTGCCCGGTATCGGAGATGCCTAGCCACACCGAAATCTGTATTAACAATGTGTAAGTGTACGCGTCACCACCGGCTGCAATCGTGTCGGGTTGATCGGTCTTTTTAGGTTGGCTTAGGCAGGGAGCAGACATACGTGGCGTAACAGGAGAACATTCGCGAATGAGGGCTGCGGCGGTCTCATGCTAAGCGTCGTCAGGGAAGGACACTGGTCGAGATCCGTTGAACGTGTAAGAATGTGGAAGCGTACTTATCTACGTCCGGTCAAGGGCGCCTCGCGAGGCTGCAACAGGAAACTGCTTGGGGGGGGGGAATTGACAATATTACAACGACTCAGAGGGCCTGAACAGTGAACGACAGTAAGTAGTACGACAGTGGCCTGGATTGGTGCTCTCGTCAATGGCGACGTTAACCAGT

>AT1180

GGGGGGCGCATGTGACGGCTCGCCTCGAGTCTTCGCGGGCGTGGTGAGAAATAGCAAGTCCATATTGACCGATAAGTTCTTCATGGTAGGTTTCGGACCACACGAGAGTAACCGGGATGTAGGACGGGAAGGCCGGGCTCCTACGCACTAGCAGCTAGGGAATAGGCAACGGCCAAGTATGGCCTCATCAAGACGTCACGAAACCGTGGAAGTGGAGCTTTCGAGGAGGCATCACACAAGGTGGGAGTAGATACTTATATAAATCCTGCGCCCTTGTTCAGCACGATCTGTCGCATGCGACGAAGGATAGGTACAGGTGCTCACAGGCAATCACATTACTTTCTGCGCCGCCGCCCTTCTCCGCCCTTCGCCAAGCCTACTCCAGATCACGTTCAAGACCCATCCGCGAGGAAGCCATTGGAAGACCTTAGCCCAGCCAGTCATTTTCCCTAAACTCATATACCTCGGCGGTCTCCTCCGAAGCGCGGCGTTCACAGTCGTTTGGGCATACACCTCTGTCGGCATGGGACTGTTCTCTTGTACGCGAGTGAGCCGTTCCAAGATGAGATCGAAGTAGTCCTTGTACAGGCTGTCCGGGGGTAGGTGGACGTGCGGCGTCTGGTTGGCAGCGATGTTGGACTTCACGGCGCCAGAGGCGAGGAGGACGACGTTGATGGGTTGATGTTCCATGGCTTGCACTCCATGTAGAGCGAGTCGGTGAGCGAATGTAACGATGCCTTGCTTGCACCGTAAACACCGCC

>AT1192

GCCCCTGGTGTTCATTATCCGGACGACGGAGTCGTGTTTCTTCTTCGCTGAAGTTTTCGCGGGTGCAGGCTTGAACATGGTGGCGATTGTGAGTTGTTTCTTGCCGTCGGCACCCTTCTTCGCGGCTTTCTTTGGAGGGGGTGGGGGTAGCTTGTCCATTTCTTCACGTTCAATGAGAATTTCGTCGCCAGGCTTGATATAGCCAGTGCCCTTCACGGTACTCCAAGCGTTGTCGACGAGGAAATAGCCGAGATACATAGGGCTGGACTCCAACGCTTGCGCAGGGGGCGATAGCTTTCGCTTCTTCGCAGGTGGCCCGTCAGGTTTGACGGGCTTGACGGGCTTCTCGTCAAGACCAAGAGGAGAGGACTTGCGCTTCGCTTCATCCCGTCCCGATGATCCGTCGCTGGACACGCTTGAGGCCCTCGGCACATCTATAAACTCTGGCATCTCCACGTCGAACTCGATGCCATCGTCGTCATCAGTGTCTAGTATTGGAGGGATACTTCTCTGCCTGTAGTTCATCGGCTCTGGCTCCTCGTCATCGCTATCAGCAAAGAATAGAGGCTTTTGCGTTGGAGACCTGCGTTGCGTCGCGACCGCGTCCTGGGCGGGCAAGTTTACTACTGCGATAGGCTGGGAATGAGGTTGCACATCATTCATGTCTTCGTCGTCAGAACCAGCAAAGAACATAGATCTCTCGCTCGCCGTGGCCATAGCTGAGGAAGTGTGGGGAGTGACTGGGAGCAGTTGATGATCGAGGAGGAGGGACGCGTCGAGTCGCGTCCGAGATATTCCCA

>AT1214

TCACGTCACCCAAGCTTACATCCTTCCGGTCCTGAGCCTGAGGCTGTACATACATCGCACTGCGCATGCATCCTGTCAAATTATACTTACATGTACGTTACGAGATCCGCCACCCCCAATTAGTTCCCTGCTTCGGATACATATCCCCCCCGTCCGGCGCCGCTCCTAAACTATAACTAAGCAAAGCACTGCAGGCTGCAGGCAGGCAGACTTCCGTCGCAGCAGCATCTGATGACCTAGCTCTCCGACTCCGTCTCTGGCCCCTCCCCGTCCCCGATGTTCTTTATCCACGCTCCGATCGCGCCACTGAAGCCCTCGTGCCGCAGGAACAGCGCGCGCTTCGTCCCCTTGCTCCAGA

>AT1225

CGCCCAGGGAGACGAGAAAGCCTGCGAATCGAGGTCCTGGTGTGTGTCCCTCGCAACAATCTTGACCTTGTATCGCACATTCTCGGATAGGACATGGGCCACTGTCAAGCCGATGACTCCGGCGCCGAGAACAACGACATGTGTCTGAGACGATTTGGACGACATGTCCGGGTGATTTGGCAGCGAGATGAGTTGACCGCAGCGTGGCTCAAAGACGATAGTCCTTGTAAGAGTGGGCGTTCAGAATCACTATGGACCATCATACAGTCAGTCGATAACGAGTCATTCCGGGCCGCAATTCGTGCTTGGTATATAGATGGCAGTCTAGATTGGAAAGATATCGATCAAGTTATCACACGCGATGCTAGGCCAGCTTTACACAATTGAGGTCCCAGGCACTTACAGAACTGAATGGCCAAGACTGGTCGAATGCACTTTCGAGCAGCATATGAGGAGTGTTGGGGTCGAGTTGGCTGTCGAGGTCGACGTCAAGGGAAGGCGGCAAAAGGCATTGGC

>AT1227

TGGCTCTGGTTCTTGACTTCTGTTCGAGCCGATGCAGACCCTCATATTCACTACATATTGCACGTAAATGGGGGGAAAGTAGTATACATATCATATACTTCATGTGGTTTTCCTCCTGATGAGAGGGAACATGTCTCCCGCAGGGTGGTGCCAGACATGCCCACGTTCTTTCTTAACGTATGCGAGGTAGGTCACCCCTCCGGCAATGATGCTAATATAGGACGCGAGCGCTGCCCACTTGAACTGAGAAGGCTCGCTAAGCACCATGGTTAGGATGTACTTG

>AT1248

GCTGGGTTAAGTTATTGACGCGATGAGTTCGCTGGGTGTGGATCCACTCGTTGATGGGCTTTGCGATCTCGCTCGGCCGGTTATCCGTAATCCAAGATTCGAAGCGGTCCAGGAAATTCAAGGCTGCAAAGTCCGTTATGTCCGCCTTGAGGGCGGTATCGATCCTGAAAGCAGTAGCTCTATGAGTAGGCAGATGCAACGACTCCTGTCCGAACGAACGCTTACCATGGCTCGATTGCTGGAAAACCGAACGTCTCCACAGCTTTGTTGAGGCTCCCTTCTACAAACAACGCCGATATACCAGCTTGAGGTAGACATTTGTCCATTGTTTTCGTCCACATCACAAGGTCCTTCCACCTGCACGCTGCGAAGGGCACGCTGGACAAAACTTCATCGTAATCGACGTCGTCGTCATCAGTATCCAGGCGGCTGAGAATGAATTGCACGAGCAGCTCCTCTTCCGAAGTAGGAGTGGTTGTTTCGGACGATTCCAGATCCTTGCAAGCCCGCGAGAGCGCTTCGTCGCCATAGACGATGTTGTAATGTGCCCATAGGGGCCAGATCACCACCAC

>AT1254

GCTATAGTACTACTGTTACAGACAAGTTACGAAACATCGGCGCCGGAAAATGATATACATCGACTCCGAGTCCAGCATGGAAGATTACATTGTGTGCTCACGCTTCCGCTTCTTGACCACGACCGGCAACGGCGGTCGGGTTAGAATAAAGTAATTCAACATGTCGTTCTCGTATGCGATCGCGTCGAAGTCTTCGCTCAACGTCAGGTCGAGCCGCTGAATCATCGCGCTCACCACCATCCGCAGTTCCTGCATGGCGAGCCCCTTGCCCGCGCAATTGCCTGGCCCGAATGAGAATGGGAAGTAGGCAGCCATGTTGTGCACCAGCTTCATCCCGATTCCATTCTTGTCTGCGGCCGCCGCATGGATCCAGCGCTCCGGCCAGAAGTTGTCAGGAAGGGGGGAAAAGTTGCGACCATCTCGGTGTATGCTGTACATGTGCACCGTCGTGATCGTCCCTTCCGGAAAGTAGCTGCC

>AT1255

AGTACGGCATATCCGCGTGGTGCTTCGTATTCACGGCGGTTTCGCCCGGTGGGTAATAGGCATCGACTTCTGCGCGCAGCCGGACGTATGCTTCCGGGTGAGTGAGTAGGCAGTAGAAGATGCTCGACAGCACGCTTGAGGTAGTGTCCGATCCAGCTATAATGGCCAAGGTTCCGTCGGCTATAACCTCGGGAAGGGGAGGCGTAGGCTCGGCGCCGTCCTCGTTGTTCTGGACAGCGTTCCGTCAGAAATATGATACGAAATGAACTAGCGGTGAAATCCCTACCAGGTAGTAGAATAAGTCTTTTCGTGAGATGTTCCCTAGTCTGACACGCTCCTCGGCGCGCTGGATGCAGAAGCTACGCATTTGTTTCATTTTCGCTCCAAGAGTAGGGAACGTGCGGATGTAATCTGCCAGCCATGGGAGATGGCCAAAAGTGTCT

>AT13

GGAGGCAGGTAAGTGGCCTGCAGTTCCTCGGCCACGCAGGCGTTGTACGTATGTGCACGGGTCGAGCAGGAACCTGAGGATGTCGCGCGGGACGGGGCCCGCGGCGGCGCGGATGGAGCGGACGGCGGCAGGGTCGATCGTCTCGTAGCGCTCCCTGTGCTTTGCGTCAATTCGGGGATGATCTACACGTGATAAGCTTGGGGGAGGAGGGGTGGGGCGGGAAGTCAGCCTGGGACTCGGAGGGAACGGGTCACGGGCCAAGGGCCACGGGATGTACAGCGTGAGGAGCTCTTTGTCGCTCCAACGGGACATCCACCAGGTGCGGACGGCGAGCTCGCTTTCAAAAGGTTTGTAGCACGTCTTGTCGGGAGAGAGAGCAGCTATGAAGAAGGTTCGGGGCCTAGCATCGAGGGTAACCGAGGAGGGCGGGGGCAGCATTTCAGTGGGGCAGTCAGCCAGTGACCAAAGTCGGGGAGCGTTGCCGACATGGTTCGGAAGATGCTGGGAGGCTATGAAGTGGGACGGCGCCATCGCCATCGCACCAGATTCGTCGAAGAGATACATCCTGCCATAGTGCGTGGTGAAAAGAACTGGCTCCTCCTACGATAGACACTGCAAGAGGAAGAAGACGAGGAGAGCGATTTTGCCTGAAACCCGAAGGTAAACATGAGCGGAAGGCCGAACAAGGGCGCAAAAGACTGCCAGGGGAAGCGGTGCATATGTACAACATACCGATTCCCGACTGCCCGAAAAGCACGACACCTTCGATCGCGGCCGGCTGTTCCAAGTCGCGGGCGTTGCGAAAGCGTTGGGCGAAGAGGAGCATTTTCTCGCGCCCC

>AT1311

AGCGTAGATGGAGTTAAATCCGATGGTATGAAAAATCATCATGAACCCAGCGAAAACGAGAAGGCATTGGTAGGTACGGGTCGGTTCTTCTGACCGGGATTCCGCCCCCGCATCCAAGCTGTTGTGCCTCGAGTTAATGTTGGCAGAATTTTCGGTGTTGTCTGGGAGGTCAGAGAAAGTACGGAGAGCGAGCACTTGGCCGGGCCGAGTCGAGTCCATGTTAGTCAATGTTCAACGAATGGGACCATACTGTATGTCGGGCTTTGTCGGGTTCAGGATCCGAGGCTTGCCTCATGTTCTCACCGGTGGTGGTTTTCTGAAAGACGGCC

>AT1313

CAGTTGCTATTGCACGTCGGCGACTCGATGGGGGTTCCCAGCACTGCCAATGCCCATCTCCACGACGAACTGTTCCCTTCCCAGCCTTCGCATCTCCTCCACAACCTCGCCCACCTTCTCGAACTCAGACCACGTGGACCTGGATCGCCCGCGCTGACCGGGACCCCCGGCCTGGTCGGACTGCAGTACAGCTACTGCTCCTGGCGA

>AT1314

GGCCGACCTTGGCGAAGCCGGCGAGCGCGAGCTCGTGCGCCCACTGCGAGAGCGAGCGGCGTTTCTGGGGGGACTTGAGGTGGTGCGAGACGAAGAGCGCGTGGTAGCGGAACGAGGGAGAGCTCGAGCTCGAGGTGGTCTGCCTGGTGGAGGCCCGCGGGCCCGTCGTGCCAACCGCTTCTGCTTCTGCTTCTGTCGCGGTCGCGGTCGCGGTCGCGCAGGCCATCGCCTGCTCCTGCTC

>AT1322

CGTCCAATACGTCCTCTTCCTCACCTGCCGATGGTTGCGACTGAGAGCCCCCATTGCTGCTCGGAGTATCTACGGCAGGCGGCGAGCCGAACCATGCACCAGGGGGTCCCGCATTGTCTTCCAACAATGGGTGGGGGGTGTTAGAGACGGTCGCTTGGAGCGTTCGTAATGCGGTCTCGAGGGCGTTGGAACGGGTCTGCAGTTGCTCGATCTTATTCTTGAGATCTTCGACATCCGCCACTGCCTGCCTGGA

>AT1326

CCGAAGGCGTCGATCGCATCCTCCTCCGCCTCGTCAGAGGGTGGCTGTTCGCGAGGAGATGATTCTGAGCCATCACTACCGCCGGCCTTCGCCGAGCTCCCGGAGGGAGATGCCGAGTCGTCAGGACTTTGGTCGTTGGTATCCGCCCGGAGTAGCGGGTGGGTGCCCTGTGAGAGGGCCCTTAAAGCAGCCTCAAGGCTGGCGACCTTTTCTTTCAATTCCTCGACTTCTGCCGCTGCTTTAA

>AT1329

ACGGTGGGGTCCAATGCCTCCTTGAGAAGAGGAGTAGCAGCCTTCAGGCTGTCCTTGAAGCCCTTGGCGACTTTGTACAAGTCGGAGTGGGGGTCGCTCCGGGTTGCACTCGTAATAGGCTGTGCCTCAGAATCATAGCGGCCGTCCGGGGCGGTAGGAAGAGGTCGTGAGGAGAGGTACTGGGCATCGGCAAGAGACTGTGTGGACGAAGAGTTAAAATCAGACGGCGTCCAATGGGATAGAGTTGATAACACGTCGCGCTTACCACCACATCAAGTAGACCAAGCGCCATCTTTATGCTGTGTGGTGTGAGTGAACGAGGGATTCAGAGGAGATATGGGTAGGCGAGAGTAGGGAGGCTGGCTCGAGTTGGCCACACTAGATATACCAAATGAAGGGCAGGCCAGTCCAGTCGAGAATGGCTGCCAGTGACAGCCTCGATGTAGAGCGGAGTAGGCTATTGTGTCCGCAGGGGATTCCGGAGCATGAAAATTCCGTATTCGGCGTCACACAGGGCGTTCGGTGGGGAGTAAGAGCGGATCGGTTAGATGATGAATTTGTCCCCGCGAGTCGACAAAGACCCACCTCGGCCGCGATCTGTGATCACCTTTCGCCGGGCTCTGAGGTTGAGAGCGTCTGACGAGAGGACCGAGGTGCCGGTCCACTGGAGAAGAAAGCGGGGTGAGATGAAGGGCCGATATGCAAATGCGGAGGGATGTATATTCCAATGCTTGCGTTGACTTCCAGGCATTTGTAGACAGTGGAGTCGAGCTTCG

>AT1339

TATGCTGTCTGGATAACTGAGGTTTCCCTCGATTGCCACCTCCACATTGGTGAGTGTGGGGAACTTGATGGGCGTGAAAATGTTGTACTTGGTGCCCTTCGCAAACAGGATGGTCGCGTTGGTCGAGTAGTTTCCCGATACAAGAGCCGCCGTCAAAGCCGGAGTGTCATCTTGTCCGTCCGTGTGAGATACCACGAACGTCTTCCAAGCGGAGACGTACGAGGCCAAGCACAAAAGGAGCGCCAAGTGCGTGCTGGCCATCCTCAGGGTATTTCAGGAAGTCGAAGTGTCGGCTTAGCACT

>AT1355

GAAGACGCATACAGCTCAGTATTATTGCTTCTGGGACGATATGAGGGCTAAGAAGGTCGAAAAACGATACTTACCACCGGTGGACTTGCGGGCAGTTTGCTGTCGAGGGCGTCGTCAGCGGCGACGTCGAGACGACGAGAGGCGATATCAAAGATAAAGTGTCTACTGACCTTGGTACGGGCCATGGGTGTTCGATGAGTTGTAAGATGTGGGGTTGTGGATAGAAATTCGACGAGCAACGGCGAGCTTGAGGGGCGCGGGGACGGCGGGAGTGGTGGTTGAGAGCGATGAGGGGCGGAGGGGCGACGAGCTGGCTCTGCGAGTCTTGTTGAAGGTCCGATGGCGTGTTTCCCACGGTTGAAACTTCCGAGCGAACAAAGCACCAGAACCTCTCGCGCTTGGGCCACCGTTCCGCGCGC

>AT1371

ATGCTGATGGTTATGTCATACACGGGTAAGATAGCATACAGAGGGACGAGGGACGAAGGACGGTTACGGATACAGATAGAGATACAGTAGGCGATACGATACACAGTAGACAGCAGGTGATACAGCCGCAGAGTCCAGGACCTCGCAACATACACATACACATACACATCCGAAGCGGAGCGCGAACGAAAACAGAGGCGGCAACAGGTAGCGACGAAACGGAATAATGATCGTGCAAGGAGGCCAAACGAAAAAAGCATGGAGACTGGAGGGTCGTGGCCGTTCATCCCATGTCGTAAACCATCGTAAGGAGGACATTCGTCCTCGTCTATGAAGCGACCCAAAGCCCGCGAAGAGAGAAATCCAGAGACAGAGCATGAGAGAAGAAGAGAAGAGAAGAGAGACGTGAAGGTCGTAACGAAAATGTCTCCGACACATACCGCAAGCTGCTCATCGACTTTAGATGCGGCTGATCTTGGGCATCTGGAAGCCCTTGTGCACGCGCATGTGCTGCCCGAGGTTGTCGTGGCGGCTGAAGTCCTTGCCGCACCCAGGGTACGGGCACTTGTGCGCTG

>AT140

TATGGGGCAGTATCGTAGCAGACCGGCGGGGAGTGTCGTTCGTCCGCGTCAAGCATTGAAGCCGAGCTCGTGCAGCCGGTACCGCGTGGTCTTGACATGGAACTCAAGGTAACCCTGGGGGGTGCAGTGCATTAGTTCGATCCCTTGTCTTCTGGCAACGGGAGAAGTCCTGCCGGCTGGCCCCTGTGGCGGCACTCACCTGCACGAACCGCTGCCACAATGCGTCTAGCTCAGGGTCGACAGACATGAGGGCGTTGAGCCTCTGTGCGCTGTCTATGACTTCGGCCGCGAGCGTTCGCAGGACGTCCGTGGGCGACTTGCGTTCAGCCGCCGCTCGCAAACGCACGTAGTTGTCAGCCTCGCCGGCGAGGGACTCCTTGTAGAAGGACAGCCTATCAGACGGTCGCATGTCAGTTCACTCCTAGTACAGACCATCTTGAGCCCCAAATATGCCC

>AT1407

GTTGCGACTGGGGCGACACCCAGTTCGTCAGCGCCACATCGCCGACGTTGTTCCTGAAGCCGACCATACCCACAACGGCCGTCCATCGGTGTTGGCAGAGCCACCCATTCTCTCCGCCGCTCCCCGAGCAGGTGCCGACCCCTCCGTTGGGTGCGCCGGCGTCGGTGTCGGTGAAGCCGCTGTAGCTCGAAAGGATGGTGGGGGTACCATAGGGGTGCGCGAGCGAGAAGATCATCGCAGAC

>AT1409

GTTATCGAGCTGGCACGTTTGCACCTCAAGGCGGTTGTCGTAATTTACGATTTCGTCGTCAGGCTCTAGACCGCAGTGGTGGAAGTCCTGGTACTGATAAATGCCCGGATAAACGTAGTGGGTATACGAAGAGCCTGCAACGCCTGTGCCGCTGTCTGAACCCGTCATATGGTTCCAGATGGTATCTGGCGCCGTGGGTATGAGAGATAAGGTTAGTGGTGGCTAACGGCGGCCATACTGAAACGGTGTAGCAGCCACAGTGCTCACCAACGATAACGCCTACGCCGGCGGTGTGGCAAGTGGCGATCATGTTCGCGAACTGGGTCCTGTTGCCGCGTTTGGAGGTGAGGATGTAGGAAACGGGTTGATAATCCGTCCACCATTGGTCTCCGGTGACGTGCTCCTGCGGCGGGCTCACTGAGGCGGAAACGCATGAGCAGAATGGACGGCGACAGCGACCCCGTGGACCAACCTTGAACGAAGCCGTAGCCAGCGGGACCGATGAAGTCGGTGCACTCTGCGGCGACGCTGTCCCAGGTCCATTCGAACATCTGGATAATGACAGTCTTCTGCGTAGACGGCGCGCGCGTGGCCAAGGACGTCCTGTCATTCGCGCTGGCGAGGTAAGGACGCCCCTGGAAAGGGACGCAAAGCGCACCTAACGTGATGGTAGCGAGCCAAGACCAGATCCGCATTGGAGCTTTGAGAGCAACAGGAGGGGGGGAGGCGAGGAAACGTGTTGGACGGTCTGAGGAAAACGAGAGTCGGAGACTAATATAGTGCTGAGATGATGTCCAAAATCGGACGGGGGCGGTAAGATTGTTGCGCACCAGGTGGAAAATGGGAAATCCGAAAAGAAGGATTTTCAAGTAGGGTTCCGTCACGACAGGAAGCTCGAATGCGATGTCATCTGGTCAGCCCCCTTTGCGAAACATGAGTCCTCCCAATCTTCGCAGCTGACGCAGACGCCAGGGGACAGCCGTCCTGTGACGAAACAAGGGGGTCTGGCGGTGGTAAACTCGCGGTCCAAGGCTGTGGTTGGGAAGAAATAGCTACGAGCGAGTCGCCTTAAATTCAGAAGAGGGATCAGCATTGGTCACAGGGCGAGGTTTTAAAGCGTCCGCACCGGAGAACATGCTACGCCGTAAATAACTTAAAACCTGCAGCATGTGCTTGCGACGAGTTCTGGCCACAACGCTCCGAGCTCGCGCCAACAGGAATCACGCGCGTCTTTGCTCTGGCACGCGCACGCGTGAAGACGTGGAGTGTGAACTTGAGAGCCTCGCACGATCCCCGGACGCCATATGCAGCAAGGTGCATCTCTGCC

>AT1419

ACCCTTGGGAATAATCACGTTCCCGAGCGGGACGTCGCAAGTGGTGTACCGCATGGCCGAGATCGTGTCGCCCTTGAGGCGCATGACTTCGCGGATGAACGAGTCCATCAGACGCGCCTCCCGTAGGGACTGCGCGGTCAGGCGGAGGGCACCCGTCTCGTCCTCCTCGAGGACGTTCGCGAGCTCCTCACGGAGCGGAGCGAGGTATTCCGGGCGCGTGGCGAGCTGGCACAATACCCACTGCGAC

>AT1426

TACAAACTGGATGGGGAGAACAAAATCAAGGCATGTCTCTGTCTGATCTCCTTATACCTTGGCCGCTCACGACTTTGCACGGCAGATCACGTTCGCAAGGAAGTAATGTCCCGGCGGAGGCGCTACTCCTGTTTGTCTGCTATCGCACATAGACGTGGACCCTGTCTCCCTATTGTACCGTCGTTGTATTCTGGTGCCCCCGCGCAATGTATTATACATGCTATCGGGATCGATAGCTCTTGGAGTCGAAGTCGTTGAGTGTGCAGATCTGAGCCGTACAGCCTGGCGCGGGCCTGGCGTGCAACGCGGCATCACGCACATTGATGAGAGGAATTCGGTGCACAACGCATTACGGACATTAGCATAGGGTACAATACACTGGTGTAGGAGACGATGTCTATGCAGCCTCTGCGGCGTCCTTCTCGCGGTCTGCCCGAATACGAGTCTTGACGTCCGCAACCATCTGTTCCCTCTCTGCCCGACGTTCAGCAATAAGACGGAGGATGTTCCCCCTCGACGCCGTATTCCCTGTCCGCCACTTCCATCCCTTCCCGTCCGAGCCCTTCGTGTGCCGCAAGAAGTACGC

>AT1428

GATGGCCGACTTCGCCGTAGAGATACCAATACCCTGTAGGCATCGTGAGGTCGTATGCGTAGAGCACTGAGCGTAAATTACGAAATGCGTACGCCAACACCCTGATCACCGATACCAAGGATTGCTTCGGAATCAGACACAACGAACAACTGGATATCGCGCCCCTTGGTCTGTTCAAGGAATTCCTCTTCCATGGTATCCTGTTCTGGGAAGGTGAGATACAAGCCCTCGCTCCGGCGGAAAAGATGGGAATAGTTTGCTATAGCATCACCCTATGCAGGATGGTCAGAAACGTGCATATATGCGCTCTTGACTACGCGAGGTACTCACCTGGGTCGGTGTATAGATGATCGGGATAAGCTCTCTCAAATGGCGGCTGAGCAACGCATAGTAGAGAACCCAATTCTGGTCCTTCACGCTCTGCAGGAAAGTGTTCTTACGAATCGGCTGTCGGCGAACGCAAAGTTGGTCATAGGCTCGTTGACATTGCTCCTCGAGCGAGTTGATGCGATAGGGAAGTCGACCAGAGAGACCGAACGCTCTGCGCTCTGAGGACGTGAAGCCGGTGCCCTTGTTAAAGCGAGGGTGTGTGAGGATGGTGTCGCCGCGCAAGGCGACTCGGAGGACTCTGTTCGTCGCCGTTGCCATCGTTGAGGGGGTATAAGTTGGAGTAAGAGGATCTTGGGTTCGAAAAGTGAGAAGATGGACGCATATTTATAGTTGGACCAGGAGAGATGACGTTCATGGCCAGCCTGAGCCATGAGTCCAAGAAGGTTCA

>AT1434

CGTCAGGGGGTCGTTCGGCGCTGTTGAGGAGGAGATAGGTGTCGTACTCAGGACGTGCGTTGCCCGAGTTCGGTCGCCAGCTGTCCTTGATGACCACGATCTTCTTCGTCGCGATGTCATACGCGACGTGTACTTTCGTCCCGCGGCCCCCAAAGGATCGACTAGCGGTCATGGGCTTCCCTATAAGGAACTCCCGTCGAGACACGGGGTCGTTACGAAGAACAGCCCTCCTGTCG

>AT1450

CCCCAGATCCTCCCCCTAGATAGATCTTCCCTCACCCTCTACCTCCCACAACTTCTGCCAAAACGGCTGCGGCGGTCATGCAACCTCGACGCCGCCCACCTTGACAGCGACTTTGTAAGGGAACGGAGAGATGCTCTGCGCGTTGGTAATAATAGGCACGGCAGAGCGACCCTGCTCAGACGACGCGTGCGTCAGCATCGTCTGCAGGCTCTTTGTCAAGGTTGAAGCGAGATCCGAATTGAACTTCTGTCGTTCTGGT

>AT1462

CTTCCACACACTCTCGTCCGAAAAGGTCCCACTGTAAATCTTGCGCTGAGAGAGAAAAGCGGGTAGGCTCGAGCTCGCAGGCTGAACATCGTCACGGTGACCACCAACATGGACTTTGACACCAGACGTGGCCGATGCCCGGGACACCGTGCTCTCGATGCTCTGCTTGGCGTTGGTGCTAGTGCTGGTGTTCTCGTGCCTTGTCGTCCCGGGGCGGCAATGCGCCAGTTCTTCGCGACGATAAGTTGTCT

>AT1474

AGGGCATGTGGTTGTCGTTTCCGACTGCCCAGCCTACGTATCCGAATACCCCGAAGAGCATGCCGAGCATAAAGACGAGCCGATATTCGGGCTCGTAGACTCCGCGGTTACGTTGAGACATCCAGACGATGCCCCAGTCGTTGAGAGGCCCTGTGATGAGCATTGCAAGGACGATGCCGACAATGCCGCCCAGGTTTGTTAAGCC

>AT1475

AGGAGAAAGTATGAAAGGGAGTGGTCGGAAGAAGATCTTCCACAAGCTATCATCAGAAAAGGTGCCGTTGTAAACCTTGAGCTGGGAGAGGAATGTCGGGGGTTTCGGAGATAGAGCGTCAGGGCCGACGTTGTGGATCTCTACATCCGGTGTTGCTGGCGAGCGGGACTTCTTCTCGACGCTGCTCTTGTCGCTGACGCTGACGCTGTCGTGGTGGGAGGACTTTTGCTGATGGGTTGTCGATACTCGGCGATGGTACGTGGTCTGGACAAGGTACGTCCTATTTGTCTCGTAGTAGATAAGACACCGATGAGCATGACTCACCTCGGGCACAAAAAAGAAGACTGCCACGAAGGACAGCCCACAAGCAATGCTAACGAACCAGAACCCGAGCTGCCAGGATAAGTTCTGAATGACGTAGCTGTAGA

>AT1477

GGGTCGTCTGAGGGCTGGGGTATAAGGACGATATGCGAGGACGTGCCAGTCCCATGTTTGACTCGCTCGTTCGCTTTGTCGATTGCAACTTGATGTCCGTGTACGCTTCCGCGATCTGATAGTAGTTCAGTTCCCGGGACATTATCCAGCTTGTGATCATCTAGGAAGACTGGTCTTAGGACATGATGGAAGCAAATGACA

>AT1489

GGGTGGGAAGACGAGGATATCCTGACGCTCTCGATCTCGGCGTCCGTAGGGCTCTTCTCCAGGCTACCGGAGTCAACGTCCTGTTTGGTTTTCTGGAGCTTCATAGAGGCCTCGCGGTCAAACGTGGTCTGCAGCGTGGTATCATGAACAAATTGTTCTACCGCAAATATGGAAAAACTCACCTCCGGGACGAAGAAAAATACGGCGATGAAGTTCAGCCCCAACGGAATGCTAACGAACCAGAAGCCGAACTCCCAGGACAGGTTCTGGATCACATAGCTGC

>AT1495

GATCCCGTTGCTGGACAGCTGGCGGCGCGGCCTGCACAGCGGCGAAGCCCGAGAGGAGGAGAGCAGCGAAGAATCCGGCGCGCATCTTGTGTGGCTTGTGGGGTCGTTTAATAGGGCGAGGTTGCGAAGGACGAAAACAGCACCGGGCTGGAGCAAGGACGAGGACGAGGACGGAAGGTCAAAGCGAAGTGGAGAAGAACGAAAAGGCGGGACGGCACAGACAGGCGACAAGCCGCTGATCGCCCACGCTCGCTGCTTTATAGCAGCAGCGAGGTGAAGGCGCGATACAGGATACAGCTTCACCGAACAGTGATCGTCCGGGCACCCTGCGGAGTGGGAGCCCGCACGGTATTTCCGCGGC

>AT1498

TTCACTAGATAAAGAAAGTGTGCGTAAGCGTACGGTACGTGTGCGTGTGATATGGTATAGTAGCAGTTTTACCGCGGTATGGCTAACCGGGGTCTCCTGCGCCGAGTTGTGACACGTGGTCACTGACTACTCGGTAGAATGGTTATGTGTAGGATAGCGGTCTGATCGGTGACGTAAGGTGTAAGTATTATATGGAATTCCCAGTAAGTGGGTTGTACTGATGCAATGATGCCGTAAAGGGAATAGGCGGGGGTCGTCGAGGTGGCGGGGAAGCGGAAGATGACGTGTCCGGGAAGGGCGGGGTAAAGGGATCGGGAGACGTACGCCGTGACTTCTGTTCCTTGGAACAGCGGAGTGTTGATCCTTGTTTGTATGAAGGTGGCGTGCGCCGGGTGGATGTGTCGGGAAACGGTGATGGTGATGGGGCTGTCTATGCCGGCGTCAGAGTCTGAGTCAGAGTCGACAGCGGTGACAGATTCGTCGGAGTCGGAGTCGATGACGGGGGCTGAGGACTGATGGCGGAGATGCGGGGGAGGGATTGGGATTGGTGGTGTC

>AT1510

GACGAATGGAAATGGCCTGAGGAATATTGTCCACAGGCTGTCATCGGAAAACGTCCCGTTATAGATCTTGAGTTGCCGGAGGAAGGAGGGAGGCTTCGGACATGGAGCGTCAGGATCGCCGCGCGTCCGAGCCATCTTCTCAGCGCTGTGCTGGTCACTGTCGCTATCGGAGTCTTTGTTATTGAGCTTGGTGGGACCGATGGTGGCGCCTTGGCGACGATAGGTCGTCTGGGCAAAACCGTCTCATCTTTGTCAGGGAAACGCCAGGAACAAGACTAACCTCGGGGACGAAGAAGAACACTCCGACAAACAACAGTCCACAAGCGATGCCGATGAACCAGAACCCTAGCCGCCAGGACAGGTACTGGACGACGTAGCTGTAGATGAAAGGGCCTCTGCGCCGTCTCCGTCAGCATGATTCCTCGTATCTGTGCAAATATCGGGCCTGATTGCAACTCACAACGTCGTGCCTGCCCCCAAGGAAAAAGTCCATATGA

>AT1512

GGATCTCCAGGATCGTCCGAGGGCTGAGGGATGAGCACTATGTGCGAGTGCTTGCCGGCCCCGCGTTTAGCCTGTCTTGCATCATCTATCCCGGCGTGGCTGGGTTTGGCCGAGAGGAGTCCGGTTCCGGGGACTCCGCTGTCCAGTTTATGGTCCTCTAAACGTTCCAGCGACATGTCGGCACTTCACAGCTTCTAAACGATAAGACCACCTCGACACGTACCGAGGATCCCGAACGCCATGCTGGTCAAGGAGACTAATAGTAAAGGAAGGGGTGAGGGTGGGGCGGAAGCGTGTCACACCAGCCAACCTCTGCCGTCGTACCATATAAGAGTCCGGGGAAAGTGCCACGCGGGGGAATCGAGTGCAGGGAATCGCGATTCCCTGCGAATCGCCTCTCGAATCCTTTTCTCGAATCCCCGACCGAATCGTCAATTCTGACTACCCCTATTGCAGTGCCCCCGAAGTCGGGCGTGGTCACAGGCAGTGTCTGGTCCGATCGGGCAGGGACAAACGGAGCCGCCATTCCCTGAACCGGCTGGCA

>AT1515

GGGCCCTGGTATAGACGTCCAAACGTACTGTACCACACCGTTGACATGGACGCGGACAGGAATTCAACATGAACCAAGTCTGCGAAATATAGAAAACGAATGAGTGCGCTACCAAAATCCGACTTGGACATGCATAGCACATAGTGACGATAGGTGATAGGACGAATGGAAATGGCTTAAGGAAGATATTCCACAGCCTGTCATCCGAAAACGTCCCATTATATATTCCGAGCTGCCGGAGAAATGAGGGGGACTTCGGATGTGGAGCATTATTATCGTCGAGGTGCTGCAGGTCCCCGACAGCGGTGTCGGATATAGGAGGCATCTTCTCGCTGCCGTCCCTTTCTTTGTCGCTCGCGTCTTTATTGGCC

>AT1542

GCGGGCGCAGTTCGAGACCGCGTTCGCGGCTGCGACGACGCGGGTCAGCGAGTCGAGCGTGGGGGGATTCGGCTCGGTTGAGATCGACTCACAGCCGCAGATAGCAGCCAACGCGAAAACGGCAACAGCAGAGCGGGAGAGCATGTTTGAGATGGCGTTGGAGAGAGAGGAGGGAGGAGGTCGGCTGGCACAAACAAAGGAGAGCGATGGGGGATGAGGTGAACAAGAGGGGCCCTTTTATACCCTCGAGCTGGGGCTGTTAAGGTGATTGGAATATCGCACCCCGCCGTAAGAAGACAAAGTGACTCGCCCGGGACTGGGTTACGTACGATCGGCAGGCAAGAGTGGCCACATCAGTAGGGGAAGAGCAAGCGGTCAGACAAGGTCGGGAAACTGCATCGCGGCTGCCGGATGTGCAGCTTCCCGATCCATGTCGGAAGCACGCCGCAACAGGACCTAGACCAGGGTCCGTCTAGGTTCGACCGACGACCACGTCGCGTGGCAGACGTAGTCACAGGTAGTCACCCTTCTTAAGCGTCGGCTATGTGAGGCTACGTAGGTCTACGTGACATGGCCGAACATAGAAGAACTTCGTCTGTGGGCCTCTGACATACAGTGAATCATTTACTGAGAGCGGAAACGGAAGCGGTAGTAACTCAACACACCTCCCATTATTCAGTAGTACGGCCACCGACGCCGTCGGGACTCGGGAGACTCGCAACAGCTGCCACGCGTGCGCCCACGGCGCCGTTCGGGTTGCATGTGAGTGTTGGACCTTGTGCCGTCGCATAGGAAGCC

>AT173

GTGGCACACAGTGTCCGCAAGATGGGGCCAAAGAGGTCGACAAAGTCGTCGATGTGCCCTTGGCTATGGGAGGGTGAGTCTCTCTGAGAGTTCCACAGATCGACAAGATTGACGAGCCATCCACGGCTGAGTATGACGTTATGCAGCGTCCTGTCGTTGGGACCAGGCTTCGCGACGACGAAGGCTGTCGAAAGGCCCTCTGCCAGATGGGCAAGTATCACCTCGCGACCCTCCCCACGCTGGCGAAGGAGGAAC

>AT177

GCGATCCTTCTGACCAAACAACCTCGAGAGGTCGGCGGACCCCTTGATAAACACTGGTGGCGGCCCTTCCCGCCAGCCGCTGTCGCCACTAAGCTTGTCGATGGTATCGGGCCACAGATCAGTCAGCAGCTCCACGATGGTACGAGCACAGTCCGTGATGCCGCAATGAGATCTGTAGTTCACCGCAAGGTGAAACGTGCGTGGGGGAATATGGGATTGCTTTACGCCCC

>AT179

ATGGTCCTCCTCCAGCTGACTCCATTTCTTCGGGAGGGGCCGACGGTGTCCCTCGTCACCCTGTCCGTTCCTGAGGCCCACTAGTTCTTCCTGACTGAGTTCTGCCAGTCGGAACGACTCCATGAGTTCGTCCGTCGTCCGTTGCACTTCGCCTGCCAGCATTCGCGAACGGGTCACAAAGAGCTGACGGGGCTTCGACAGGCCCCACGGCTCCGACAACCACCCGCGTTCAATGGAAATGATCTTGTACATCA

>AT190

TACCGGACGACGACACCATCATTGCCAGTGAACTGGAATAGCTCCTGAAAACCCTGTCTTCTGACGAACTTCGGTTTCGGGCCCATTCTCCACCCTCTGTCTGGGCTTAGTCCGTCAATCGACTCGGGCCACAGGGTGGTGAGTAGGTCCACAACCGTCTGCGCACAGTTGATGATGCCGGCATGGGACCGATAATTCACTG

>AT192

CTTCCAGCTCGCTCCATCTGGTCGGCAAGAGTCGAGAGTACTCCGCTGGTTCGACCTGACTTCTCCAGATGTCCACTAGCTCCTGCTGTGTCAAATCCGCCATCCTGAAGGACTCCAGGATCTGGATGACCGTGCGTTGCACTTGCCGAGACAGCATGTCCGATCGCGTCACGAACAGTTGACGTGGTCGCGGGAGGTCCCAGGATGCCGTCCACCAATCGCGTTCGACCGAGACGATCTTATACATC

>AT193

ACGTCTTTGTCCTGGATATTCCCCAAATCTGCCGGCTCTTCTGACTCTTCGGTCTCCTCGAAGTTTTCCGTGGACTGGTAGAACTTGACGGGCTTGAAAACGTCATTCGCAGTCCTCCGAGGCTGGCTTCGTGCTCGACAACTACGCCACACCCATCCTTTTCAAGATACGCTGCGTTGAAATGAGCAATACGCACTGATCGCGGTACTTCTTCCCTTGTCGTCCGAGGTCCCGGCTGAGGGCCTCCCAAAACTCCCTCTTCCTGTGAATTTCCGCATGGTTGTACACGCCAAATATCCGCAATGC

>AT21

CGTACGTCTTCGAGATACACCGCCTTCCCCGTTTCGGAATCTATCAAGCAAGGAACGTGAGCAGGCCGGACCGGCCGCGTACGCTCAGCTCCCACGTCGTCGAGAATGAATTGTGGAAGCGTGATGTCGTCACGCGGGGGCACGAATGGCGGGCCTTGCGAGTGGAAGGTCGTCATGGATGATGGCAGGACTAGAGCACTGCAAGGCAAAGGCAGCGACGGGGTTAGCAATGGTGGAGGGGGAGAGAGAGAACAGCCGCGC

>AT211

GAGGGGCGGGTGGAGACAAAACCTCGTCGATCGTAGTTATCACTCCGAGTGTGCATAATTTTGCAAAGGCAGGTTCGCAATAAAATGGATGACAGTAAAAGGTACATAAGAAAGTCAGAAGCGGCAAACACTCACCATGGCGCTGCATGACAGAGAGGAACTTGACGATGACCTTCGAGGAAGGGCGGACGAGCACCTGGCGCTTGCCACGGCGCTCGGCGTTGACGATGTTGTTCTACGGGCACAATCAGCACATCCTGCTACCACATAGTCGAAAAAGACTCACCAAGCAGTCGTTAAGGACGGATACGCGAACCATGGTGACTGGTTAGGAGATACCGGATGAGACTAGGGATACAAGGGCCGCGGTAAGTAACTTGCAACGCTCGTCTGAGTGTCGTGCTACAGAAAGCGCGAGTCAAGACGAGCTAGATCACAGTGCAAGACGGTGAAAGAACAGTCGTCAAGCGCGGTCTGCGGCAACACATTGGAGAGTCGGAGTGCATCATTCACCTTGATAGGGTCTTGAGGGGCGGCGAGGGCGACGAGGGGATGATCCTG

>AT232

TGTGCTAAGAGGGATGTCCGCTCGTATTCGCCGCTTGTTCTCGGGCCGAGGGCATTAATTGAAGTAAGTAGGCTAGGTAACCCGGATTCATCAACATGCTGTATTCCACATGCTATTCGTACCTAGTACATCGCTAAGAACAAATCTGGCTTTTCGAGGACGACGTTACGATACACTGCATTCGTACAACAGATTTCTCGCAACCAAACACGGGACAAGGAATACCACCAGTATCGAGCCACACGGAGGTCATGAACGTTTGAACCGCCGTGATTGGCAACCCGTGTTGAACGTATCGGTGTTCCCCGACTAAGCGTCCACCTTCTTGAAGATGAAGACCTCGTTCGGAGGATAGACGGGAGGGTTGCTCGGAGCGGCGATGAGGGGCCTCAGGATGATCTGC

>AT238

GTCCAGTCCTTCTACAACTTCGTCCACCGCCCACGCGTCCTTGGGAAGGAGGTAAACTAGGATACCGTAGTCAGGTAATGTGTCCCAGAGGGCATCAAGGGTGGCTTCAAGAAAGAAGCGAGATGCAGTGGCGAGAGAAGGCATCGTGCGAAGCCCTATGAAGTCTATCTTCCGGGCTGGCGGGCGCACAAAGTATTCGGGCACATTCAAGCTTGGCGACAGTGCGTCTGGCAGACTTTCCTGGGATATTGTCACCCACTCCTTGATGCCTACGAGCTGGTCGCCTATTTTCCGAATCAACTCCGGTGGTAGACGTTGCACGGGGTGAACGGAATGGGCCATCTAAGAGCAGTGGTCAAGGAGGTTATGTGAAAGACGTCGACGAGGATAGGGGCACCAGCTGCGCGGCCTTTTGTAGTGACCGTGAGCGATTGGTAAAGAGTACTGGTACGTATA

>AT240

TCACTCTGTCGCTGCTGCATGTTCTGAATGATGGTCCATATTTTCGCAAACTCCTCATTGTTCTGCACGGTTGCCCCACTAGCCCGAAGCTGGTTGGCGCGCTGAAAATGAGGAGGCGGGTGGAGCGTGAGTGGGCGAAGAACACATGGAGGATATGGAGGGTTTGTGGTGGGAAATAGGAACACACCTGGAGGAGATGCTGCATGCGGTCGCGCAAGCTGGCATTGGGCTGCTGTTGTGCGGGGAGGTTGGCGAATTGTTGTTGGGCGACCTGGTGAGGATTGGGGGCAATGTTCGGGGCGGCCATTCCGGAGACGATGGCAGCGGCGCGACGAGGATACCGCGTCGCGCAGATCTCGTGAGTTGACTGAAGTCCTCGGCCACTCG

>AT257

GCTGTGAAGGTTAGGCTAGCGCACCGAACGGCCGGATCCGACTCGGACTTACGGGAGGGATGAGCGCACCACGGACGGCATCGACAAAGGCGACAAGCAGGATGGAAGCGACCAGCGCAGCGTTGGCGGATGTCATGCAACTCTGAGCTTTGGTGAGTTTGAACTGTGTCGTCATCTACACCGTAAAAATCTGATGTTGTTCTCC

>AT258

GTGGCTGTATCCGAGGACGCTGCAGCAAGTGCACGGAGGGACCGAATAGCTTCGGAGTTGTGGGCACAGTATCAAGCCGAGCTGGAGAGCCGTCGCCAACAATAGGAGATTACAAAGCTGGATACGTTACTTGTACACGTAATGACAGGAGCGGGTACATGATCTATATGCTACGTGATCCACTCGCTGTGATGTCCATCTCGTCCTGCAACCAGAATGCCCGGGCAGCTGCTTGATCATCAGAAAAGCTCAAGTACTCTTTAACACAGTCCTGGGAACGGAAGACGCGACGAGCACGAGAGAGGGAGTATGGTGAGAGATCCTCCTTTTCGTTGACCTTCTCCCATGCCTTCTTCCGAATCGGGGAGAACTCGGATCCGAGTGATGGAGTTTGGGGCTCACCCGTCAGGCTGTCGACAAGCTTGCCAACCGAACTGCACAACAGGCTAAGTTGGCTCTGGGTGTTTGCAGCCTTTGGACGCTTGCTCGAGACAGGGGTCGATGGTGGG

>AT26

GCGTCGGGGGCGTCGGGGTCTGCGGTTGCAGCAGCGGCGGTTGCTGCGGCTTTCTTCCTAGGAGGCATCGTGTCAAAGTCAAAGTCTGCGCGGTGCGTTGTGTCGAGGTGTTGGTGGCGTGCAGGTGCAAGATGATGAAAAGGAGGGTTGCAGGTCGTGGTTCGAGTACAAGTTCGAGTTGAAAGGCGTGTGGGGACTGGGTGTTCGCGAATCGCGAATCGCGAGGTGCCTGTCAGTTCAGTCTCAGATCAACGTTAACGGCCAGCCGCCACACACGCGCTTTAAATGAGGGCGC

>AT279

CATACTCGGACGACGGGAGTGATAAACTCGACGAGGAACTTGTTCAACTTCAAGAGCGAAGGGAAGAAGTGGTCCAGGTAGTTAATGAGAAGACCCTTGATGTGTGAACCATCGTGATCCTATCCGAAAGCCTCAGCACTTCATCACACAGCTCGACATTTGTAAAGGATCCCTACCTGATCCGTCATCAACATGAGACGACCATACCGCAGCCCGTCCACGCTGGTATACGTCTTGTTGTGCTGTAGGCCCAGGATTTTCTTGATGTTCTGGATCTCCTCGTTCTTCATGATCTGGTCATGCTTTGCATCGCGGACGTTGAGCAGTTTTCCACGAAGCGGGAAGACACCGTAAGTGTCGCGACCGACGACACCCAGCCCCGCGACCGCGAGCGCCTTGGCCGAGTCACCTTCTGTAAGGATGAGGGTGCATTCGTCGGCCCTCCTAGAACCGGCCTTGTTGGCGTCACACAGCTTGGTCAAGCCCGTGATCCTGAA

>AT297

TGACATGTTGCCAACCAGCTCGAGATTGCGAGCGGTGTCAGAGTCAATCATCATTGTACCTTCCACCTGGGTGTATCGGATAAGTAGAGACGAAGCAGCAAAGCGCACGTTGAGCTTGAGTTCAGCATGCTTGAAGAGCGCGCTGGCGGCAGAGAGTGCATAGTATCTGTGGCAATGTTGAGCACGCAAGAGATGCTAGAGTTTAGGTGCATATATGCACTTATTGGACACGGCGACGAGGGTGGCAGCACGTTCGTCGTTGTCGACCATGAGCTGGTTGATGAAGTCTAGCCCTAATGTATTTATCAGTGCTGTGCACGCAAGCGAGAGACCCGAGTACTCACCTGCGTTATCGCTCCAATATTTGCGCATGACTGGCTCGATGGGTACACCGTCGAACTCGTCCATGATGCATTGAACGAGAAGTGTTGTCGTCTGCGGGGTCTTCGCACCAGATGGGAGCGAAACATCGGACAGGGACATGAACGTGTCAGGAACAAGGACGATTGAAGGATAGTGGAGATGCATTTGGTGCAACGTCTTGACGTAGGTCGGGCAATCGGCGAGCTGATTCAACGTCAATAGACTGCCAACCAGGTGCATATATGCAGCATCTATGTCTTACCTGGACCAAGGAAACTCGGCCTGTATCCTTTTCAAGGGCGGCGATACCCACTTCGCGCCCTACTCCGCGTCCTTCGATGACTGCGACTATGTAGCTGAAGTCATGTCTAGACGACGCAGCGGTCTGGGGCCGACCAGTATTGGGGCGCGAATGGGCAGTCCGGGTACGGGAAAACGCTGTCGCCGGACGCGATGATGAAGGACGGCCAGTGTCCGTTCCAGTGAGAGACATGGTTGGTCAAGGAAGAGTTGCCAGTGAGGTGATGAGACGAAGAGGAGCGTGGC

>AT303

GGGCTTGGCTGAAGGCGAAGGGGAATGTCTTTCCTTGAGTCTGTCTAGTGATGGGCTGAATATTGGGGGAAGGAGGAGTGCGGAGCGAGTACGAACTCCTTGTCGAGCCATTGGAGGGCAGGACATTCTTTGGTGGTGCTGTAGAATATTTGGACGCGAACCTCGTCTCCTACTTTGCGAGATACAGGAAGAGGAATGGTCAAAGGCGTCCCCATAACATCGTGATGTTTGCCGAGGGAGAACTAGATCGCTGGGATGTGAGT

>AT316

TACCCGAAGCTTTGTGGAGGGTATGCTCCTTGATTTTGGTCCACCCGCCCACACCGGGAGCTTGTCCTGGCTGCTCCTTCCAGATAATGACCTTGCCGTCGTAGGAACACGACGCGAGGATCGGGCCAAATTTGGGATGCGCCCATGCTACTTGCCAAACTGGGCCGGTATGGCTGCAATCGAGGGGTTGGAGGAGAAGTTGTGTGATTGTATGATGGACGGACTCACCCCTTGAGCGTATGCCCACTCACGGGCTGGGTCTCTCCGTCGACAACATCAAAGACCTTTACCGTCCTGTCAGAAGAACAGGTCGCGAGGCGTTTTCCGTAGTAGTCGAGTTGGGCATCATGCT

>AT319

ACCTTGCGTTGTACCACTATGCTAGGAACCAGAGAGAAAGCCTTGAGACGATTGCTCTCTACAAGCCATGAGAGAGCTTGATCCAGAGCACGAGAGTATGAAAACGGTGGATAAAGGAGGTGTTGGAGTAGTCGATGAGCACCTGTCTGGCTTAGGGCATAAGCATGAGTGCATTTCGGTGCAAATGAAGGATGTAATGTGATCTCCGATGATGATCCTTGCGATATCTTGT

>AT322

AACGCATGCCGGAGGATGGTCATGTACTCCTTCTGTTTCAAAGCGGTCTTCCCGTTCTCTTCATGAAGGACATCCGCAAGCGTTGCAGGGTCCCAGCTGCTCAACACGCTACGCAGTCGGGATAACAAACCGCGAATGACGGTCCCTGTGCACAATGTTATCTAGGGTAAAACTGAAGCAACGAGATTCGCTTACGGTATTGATCTGTAGGAAGCGATCTACACATCTTTTGCGCCTCTGCGGAAGGATAGTCAGGCTCGACGAAGAAGAACGGGGCTAGAGCTGGTATATCGGTCAATTTAGCCGTCCGGCCCTGT

>AT325

GCCCAATCCAAAGCACCACGAATGCCATCGACTGCACCAGGGACTGCGCGGGACTAGAGCAGGTTGAGGACTGGTCGAGGGGGAAGATGAAGATGGACCAAACCCTGTCTGTATCCTCTATTCTTAATATCCACTTGCCACCCGTCTTCTTGGCGTATAGGTAGTTGTAAAGGGCGGTGCGGAGACCACCCAAATGGAGCGCGCCTGTGGGCGACGGTGCAAAACGCAAGAGAGGGGCCATGGGTAAATGCAGAAGACTCTGAGTCTGGCCGCGACAACAGTGGGCAGGTTCAAGTTGAGTGCAGTAGCAGAGTAATCCGAGTCGTAGTCGCACA

>AT327

ACAGGGCTGGAAAATGAGAGTCCCAGATATCTCCACATTCCTACTGTAGCAAGAGGGTTCGATGCCTTGGCTAGAGCCGATGAGTGGGCAAATGACGAGAGCGGCAGTTTGGCAGATCTCGTCGAAGGAGCCAAATTGGATCCCCATGGCGGAGGGATAAGGAGGGACTCAGTCGGTCAAGGGAGTGTATAGAGGCCGAATGCGGTGCGCAGGGCGGAGAGCGGATCGGCCGAGAGGGGCGGAGAGGGAGGTAGAGGGATGAAGTGGGCGCTGTCGGTGTCGAGGTCGTCGTGTGTGATGGCGAGCAAGGCAGCCAGCAGAGGCCCGCTGTGGTGACTCCGTGACTGTGGGTGACACAATTGTTGTCACGGAGGCTGTCGGTGGACCCTCCCTTGAAGTTGAAACGTTGAAGCTTGAAGCTTGCGTTTCTCCTTCAGTCCCCGTCGTCCCGCAATCCATATCCA

>AT33

GGCCTCGTTTGAGAACGCCTCGGCCCCGGAAACGAGCGCGAAGGTCGGCTGGAGGGAGCAATATTCCAGTTCCTCGGCCACATGGCGGTTGTATCCCCCAGGGTCGTCTAGAAACTTGAATATATCGCGGGGGACGGGGCCAGCGGCCTCGCGGACGGAACGCACTGCCGCGGCGTCGAACGTTCGGTAGCGCTCCCGGTGTCTGGGATTGACGTTCAGGAAAGGG

>AT34

GGGGAGAGGACAGCTACAAAGAACATCCGCGGTCTGGCGTCGAGGGTGACAGACGAGGGCGAGGGCGCCGCGGAAAGAGGGCAGTCGACCAGCGACCACAATCGGGGGGCGTTGTCGACATGGCCCGGGAGATGCTCGATGGCAACGAAGCCAGACGTCGACTTTGACTTCAGTGCCGCCACACCGCACTCGTCGAAGAGATACGTCCCTCCATAGCGCGTGGTGAAGACGACCCGCTCCTCCATGG

>AT364

CGAATCCGCAAGGTCCAAGAACGCAGGGGGGCTCTGAATCCTGTCGCCCGGGTTTTGACGAGAGTCGGTGGGGGAAGTAGAGGCGTCCAAAGTAATGTTCATAGCGAAAGAAGAGGGAATACTTTTGCTCCAAATTGGTCGAGAGAGAACAGATTGAGCAGAACGGTTAGAGTAGTGAGTGAGATAAGCCTAATTGCACAAGGGTAATCCTGCGTTGCGCGGCCAGCAAAGGCGTGAGGTGAGTGGGAGGAGTGGGGGAGGAGTGGGGGAAGGGGGTGATGGGGACTGACCTGCCAGCTGATTTTTAGCCCGTCTATAACGACTATGTTGGTACAATGTATGGGGAGTGCTGCTGGGGGCGGGGATGAGGATGAGGAGGAGGGCAGAGAGAGAGTGGGGGACGCACGCTGGGCGAATTTTGACAGCAGTCGGATGGGTTGGGAGGTGACAAGGACTATTAGCGGTGTACAGGAGCGTCCTTACTGGGGCAGAGCAAGCAACTCTAAATGCA

>AT366

TCGGCCCCACCATGGCAGAGATGCCGTCCTTGATCATGCTCCGGCTACCCACGCATGTCACAGGATAGACGAGTCTATGTCCTGTGTAGGCGGCTAAAAGCTGAACAAGCTGGGCGGCGTACCCTAACGCCGTCGCGACGCTGTCTTCTGTCACTTCTTTGAATGCCGGGAGCGACAGGGGTGGCGCAGGGTCGGTGGCCGCCACAGGGATGGGAAGGGGAACGTTTAGAATGGAGAAAAGCAAGTCCGGGGGAGAAACGAGCTCTATGGGGTATATGAAAGAAACAATGGATATCAGGTTGCTGCGCATAATCGGGAGGACGTTGCGCATAGACGACAATCGAGCCCTGGACGAGGCCTTCATTAGCGGGCGAGAGAAAAAATAGTGGGTAGATTGGCATACCGCTCCTCGGCGATAGTCTCTTCTGTTCGTGCCTCCTGTCCCAGATCTTGTTCATGCAGCTGTTGTGCTTGCTCGAGGAGCTCGCGTCGCCTTCGTAAGGCTTCGCGTTGGGCGGCTATGTGATTTCGCA

>AT369

AGCGGGCAACACGAACCCCATCTATCCCAATACATTGAACGATAGAATACGACTACTTTTACAATGTCCATCGATGCGACCTAGAGGACCGGGGTCGACTGGTCACGAGTATACTCTCCACTTGCTCGTGGGGACAGCTGCAGCACGCGTCAGAAGACTGACGCCGCAGCAGCGATGATGGGAACATACCTTGCAGCTTCTTGGCGGTCGGAATGAGGAAGAAGATGACCGAAAGGAGACCAAAGCCAATGCATATTGCAGAGACAATGTAGTAGCCGTCTTGCACCGTAACGCAGTCCCCTCCCAGCTTCTGACATGCAGCTTTACCATGCTCCGAAACGCATTCTTGAGC

>AT376

GGGCTGATGTCAACACTACGTAACGATGTATCCCTTGATAGAAATTTACTTACCTTTTTGCGTGTTCGGCGATACGATAAACATCCGGCGTCGCTTCGTGAAGCATCATGAGAATAATCTACCACAGCAGAAGCTAGTCAGCCAAACGAACGGAACTAATAATGAACACCGGAGAAGCTGACCCTGAAAGACATTTTGGCAATGGCCTCTTCACTCTGAGATTCTGCCTCGGCCTGCGCAGCCTGGCGGTACCGATCGTGTGGGAAGCTACACATGTCACGAGCACGCTGGATGAGCCAGCCCTCCGCGATGGCAGTCTGGAGGCCACGGATGGACCGTCGCGTTGAGCTGGACTCCTTGTCCTTCTCCCTGAAAGTGCTGACAGCCTTAGAAACGTTCCACTGGTCCTTGGAGAGTTCGTCCTCAGTACCGCTGCCACTGCTATCGCTAGAGTCGTCCCAGTCCATGAGCAGCGCGACCTCTGTGACTTTGAATCTGGTACGTCCACGTGAGCGATGAGTAGAAACATGATGCGATCCACTAACGTTGGTCCAAAAAACGCGGCCCAGATGACAAATTTTCTTGCGTCCTCTGGCAGTTCGCGGAGGTGGTTGACGAGGAAGTTGAGGTCGCCAGGGGCTGCAGTCATGAATTTCTTGTCGAAGAGCGTCTTCTCTATTTGAGCGATGTCAAACCTGG

>AT377

CACATGACTGCCGGTACAACGTCGTCAGGATACTCCGGGCAGAAAAAGGATTTCCTTCCGAGGAGCTATGGACTATCCGAGACAGATGCGCAATGTCTTCCTTCGATCGATGCAACGTGCGCGCGATGAGGGTAGACACTGCGGGATAATTCAGTGGCTCGAGCGTGATCCATGTCGTCGACTTCGAAGCGAACGTGCGGCGGATCCGATCTAGTTTCTCCCT

>AT378

ACGAATCCGGTGCTTCGTGCAGATCATCTAAAAACAGCGCGAACAGTCGTATCCCCGCAAGGACCGAGAACACGTTCTCGACCAGCGACTGGAAGCGGGCAGGCAGCTCGTTCGTCTCCAGAGTCTCTCGTGGCGGTTCGATCGTGATATCAAACAGCGAAAAGATGGTCTTCAACTCCGGCGCGCCCTCGTAAAGGAGCGGGACGTTCTGCAGCTGGGGTCCTAATCGATGCTTCAAGTCCGTCACAAAGCGATGGAGGTCCGAGTGGAACACGATGAGTTGTCGGAGGACGTGCGACAAACACGCAAGCTGAGC

>AT393

TTCGCATTGTAAGGTGTCTCTGCCCGTTAGATTTGTCGTCGTAGCGTATGTATTGTACAGAGGCGTAATATATGATATCCGATCGCAGGGGTGTTGTACAGTGGCTCTCTGGAGCTGTAGGTATCAAGGTGGCGATTGTTTCCGAGACTTTCTCCGTCTCGTCGATCTTGTAGGAACCGCATTTGTTGCCTTTGCCTGTGGGCGAAGTTCTCATTAACATTGACAAGCATCCTACAGAGGTAGGACCGGGGCTCACCTTTCGTTTGAGTTTGCGAGTCACCTTCTCTACCTTTTCCTCTTCCTCCTCGCCGGATAGGGGTGTCAGGTCACTCTCGCCCGCTTCCTCGACTTCGCCCTGTGCGCAACGCGTGTTGAGATCGCGACCGACACCGAAAGAAACACCGCGTACATTGACGACTGTAGCTCCGCGCTTGAGCTTCTGAAGCTCTGCAACATACGCGGACGTCCGTCCCCCGACCGTTATCCACTTGATCGTTGCCGGTTTTCCGTCTGGCTACGATTCATCGCCGGTCAGAGGGCCTATCAGCATTTATTGCGTCCACAGACCATTCTTGGCTAGCGACTAACCAATTTCAGGGTATGCTTCTCC

>AT395

CTTACGAATTCTTCATGCGTGACCCCTGCGAAGACGATTGTATCTTCCACCGTGTCTACGGCTGCAACCGTCTTCCCTTGTGCAAGTTCTCGTAGGAGCCTAGCAGCACGTTCGACTTCTGCAACCACCAAGCAGCCCGGTGTTAGTATGAGTTCTGGACACATGAGGATCCCGGCTACAAGTGGCCCTGGCAACACACCGGGCAATTCAGGCATCGATGGACACGTGCCACCCTGACCTTGTGAGGCAGAATTAATATTGGAATCGCGAACACCAGCAGCTGGGCCAAGTCTTCGAATTCGGAACTCCGTCAAGTGGTGGTCACCCAAGTTCAAGTAGAAGTAGCAGC

>AT396

ATGCCGTCTCTTTTATTTTCTCAGGCCAAGGTACAGTCAGTCAGCCTATGCTGTTCCATCTGTGAGTACCGCCACTTGCAATGTAGGAGCGCATCAAAAGGGTGAAAAGGCCATGACGAAGGGTAAAGAAGGTTCCCAAGGGTGGCCGTTGATACCATATGGTATGGATATAATACAATAGTACACTTCCGAATCGGGTCCTCTCTGTCGACGGCCAGCTTCTTACTTGCGTAACTCCTTCTCGACATTCTGCTTGAAGTCCCGAGTGGCTTCTTTTGCGCCAGCAGCGGCCTCATGAGATCATTATCAGTATCGTATCCAGGCAAGTTAGGGACGTTCAGACCGAACTCACTTGATTTGCTTTCTGGCCCGCGGCGGTCGACTTCTCGGCAGCCTTCTTCTTGCCTACACCTATTGGGGGGAAAGTAAACCAGAGAGCCATAGTGTTCTTTTTTGGGGGAACACGCACCCAGAGTCTCTTTCGTGGCGTCTGTAGCCCTTTCACCCTTCTCGATTGCCTCTGCCAAACCCTTCCCGACAGACCTATTTACCTATACGAGTAATTCCGGTGTTCAGTTCAGTACTTGACCCACGCGGCTGAAGTAGAGGCGTACCTTATCTGCGACCTCTGCGACCTTTTCTGTTGCAGTCTTGCGTGAAATGGCCGAGGCATGGAACGTACGCGAAGACGATACAACGGCAGAGCGGGTAGAGGTGGTGAGCGCAGTGCGAAGCATAGTAGACATGGTTGCGGTCTGAGTCGTGGATGTGGATGAGATGGTCGTCGGTCGTAGTAGAGACTTGAGAAAGGTGAGCAGAAAAGAGGTTGTTACCCCGGATCTAAGTAGGGCCTCGCTTTCGACATGACGTCGATGTCGGGGCCCTGTCTGTGCAGTGTGGATCTCTGAGGAGAGCACGGTGGCAGAAGGCGCCGCCA

>AT41

CGTTGCTGGAATGAGGGCCTGGATGAACTCAGTGGCGGCGTATACGACCACGAGAAACGCGCATCCGGGGATGTTCAGGACGTCGTTGACCATGCGGATGACGTACGGGACGCATTCTACGATGTCCCTGTACCTTCCCAGTAGCGGTGCCAAAGGAATGCCTGGGAGGTCAAGCTCCAGCTCTTCGAAGAAGTCCCAAACACCTATGCGCAGGTGGTGGATGCGCGTTTCGGGGTTGGTCGGGTCGCTGTCGCCATTCGGATGCTTGCCATTCAGTTGAGCCTTCTCGTCGCCTGCTGCAAGAGAGTGGGTAGTCATTGTTAGAGGTGCGCGGTGGGACCACAGATGCTCTCAGTTTAAATACTACGACGGCGAGTCAGCCTGAGCCTCCGAGTACAGCCAGGCCGCAACCGACGCGGTCCTTACGCCATCCCCGGAAGGACGACGCGCCT

>AT410

CGACCTCGTAGGCTTCGGCCTGGACGGCAAGAATATGCTTCTTGAGCTCTTCTTCATCGTCGATGCTGGTTTGAGTTTTCATGAAAGCGAACCCTTCCTCGTCGAGCTTGTACAGACTCTCGTCCAAGGGGTTCAGCTTTCCGAGCTCCTTCTCCAGGTGTGCGTTTGTAGACATGATTGGATTCGGGATGTGCACAGACGATGAACGTTGAGCGGCGGCGTGAACTTGATCAGGCAGAATCTGCTGCAGCTGGGGCTCTGCGAAGCTCGTGGAAGTGTTATATGGGCTTGAGACAAAACCGCTCACACACGGTCACCATGATATATACGAAGTTGAAAGCGCTGAATC

>AT412

GGCAAGGACATGCTTCTTGAGTTCTTCTTCGTCCTCGATGCCGGCTTGGGTCTTCATAAAAGCGAGTCCTTCTTCGTCAAGGTCGTACAGACGTTCATCCAGGGGGTCCACCTTGCCGAACTCCTTCTCGACGCATATTTCTGCAGTTATAGACATGACGGGACGATCGGCGGAGAGGTGAAAGTCGGGCGGAATGACGTTGAGTCTGCTGAGCCGTGACCACTGACGGCAAGTGTTATGTGGGCCTG

>AT428

TCCGACTTCCGTCACTTGAAGCTGCCCTGCTGCTCGGAGCTTCGGCAGAGACGAGGAGATCATCGCCTTTAGATCACCGAATAGCTCCCCGCCTGCGTGCGGAATCACCTCCACATTCACCCAACACAAGTCCGCGTAGACTCGAGGTCGCGCGAAGGTTGCGTCGAGCGCAGGCAAGTCCCAAAACTGGACCCCAGGGACATCTAGGTACCCGACCGCT

>AT43

TTGTGCCATTGCCAAGTGTTGCGATTGCAGCGGCTCTACAACACACAGTACGACAGTGGGAAACGTCGGGCGGATAATGTGCAGGAGGCGACAAGGAAAGAGAGAGTATGGTAGGGTAGTACGAAGGTACAAGTGTGCACGAAACACGTTGCTCCAGACAGAGGGAGAGAGGGGTAGGATAAAAGTGCAGTGCAGAAGACGGGGGATAGAGCGCGGGTAACATAGAACAGAGCGGGTAACGCGATGCGGGGGAATACGTCGAGTCGGTACAGAACCACAATCGGACCGAAGGCGATGGAGGGGAGGGAGATGGTAACAACGATCTAGTCGGTGCGGCCCTTGGGGATGGCGCCCAGGGGGAGGACGAACCAAGAGTTGGCGCGGTCGCTGAGGAGCTCGGCGGTGCCGACGTAGAACGCGATGAACGCGGTGATGATGCCGAACGCGCCGCCGGCCTTGTCGAGCACGACGGCGTTGTTTGCGGAGAACTTCCCTGCG

>AT455

GTCGAGCAGGGCGAGAACACCTATACGTATTGAGGTTTTTCCGACAGGCTAATGAAACATGATGCATATACACGCTACAAGTACACGACACTATCGACTTAGAATGCACTTTGACTCTTTTTCGCGCATATGCAACCTATAACTTCAAGTCGGGGTTCGCGGGGATGGGGCGTTCTGCCTGAGGGGTGATGCGGGCGCCGTGGCGGCGATGGCCGGTGTTTT

>AT470

GAACTCGAGGGTGAAGCACCGCGACTCGACCGCGTCGCGAACGAGCCAGACGTCCTCCAGGTAGCTCAGCGCCGCCTGGTCCGCGTTGTGCTGCGCATGCACGCCGATCATCTCGTGGTTCATGAGCGCAACCGGCCAGAACTTGCTGATCGCCTTCACCACCGCGCGCCGCTTCTCGTACACCGGCAGCATCTTCTCCTGCGCGCGTCGCTCTGCA

>AT482

ACATAGTGTACGACGCACCAGTTGTCTGTGTCTGATACATAGACTCCATGGTCGGTTGTGATGCCCTTGCGCGGTGGATTTCCGAGATCGACGGTATCCTTGGCGAGGTCCGCAGCCTTGGCGGTCTCCCCGGTGAGGGTGGTGTAGGCCTCTTTAATGGCACCGATGGCGGAAGACATCATGGTAAAGTTGTGAGGAAGTTGTGCCTCGTACGTGGCGAGCGAGGTATATAAGTGCCCAAGTACGGTGGCTGAAATCGAGGCAACGACGTCACCACGAATCACCGTTGACGAAGTGTTTCCGCTTTAGGAAGGAGAGAGTAGCCATCCTGTCGGTCGTGTACAAGCTATGATGACACACGCCGTCAGGACTGCTGCCGTTAGCTCCGATTGGCTCCCGGAATGGGCA

>AT488

GTATATTCAAACACTTTAACTGGTCGTCCATGGCCACGAGAAGGATCCCCAAGTATCAAGAATATCAAGACGTCGGATGCAGTTATCAACACAGCGACCCAGAGTGGGAGGGCTGGAAACAATAGACACAGGCCAATCGCCGAGCCAAGCAGTTCTGCAAGATCGGTGCTGATTATGGCGATTTCGGAGAGTGCGTAGAGCGGATAAAGAACACCATATCGAATGAGGCGTCTGTGTCTCGGGCGGTCATGGAGGAGCACTCTAC

>AT495

TATGCGTATAATCCGTGTCATCGCCACGTCCAGAAGCGTATAGTACATATGCGTGCGGGCACCTGAGAGGAAGCCCGCCCTGAGGGTTGCCAAGAAAATGCCATAACAGCATCAAGAACCGGCCCCATCCTTCATTGGAGCCCTCCTGTTGGTCATCCGGGTCGTTCTCATCAAATACTTCAATAAAAAAGGAACTGCAGATGGCCTGTTCAGCATCCAGCCCTCGACACCCAACTCTTCTTGAGCTTAAGTGACGGCACAAACCTTGGGGTTGGCGATTTGCCGCCCTAAGTGCCACCATCGAAAGCGTGGCCGGTCAGAAGCGGAAGAAGCGGGAGTTCTGGAGGGGCCGCGCAGGCTCTGCGAGGGCCGCCCTCTTGCTGGCCCTCTTGCTGGAGGTGCACGCCCCATCGTCGGTCCAAACTCCGCTACCACCG

>AT496

TCATTGCCACCGGTGTAAACCGTCGAGGAACTCCATGCCGCTACGCCGGCACAGGACCCGCCGGACGTCGGGGAAGCACTCGAGGATGTAGTCGTTGTTGTCAGGGTAGAGGTCGTAGACGACCCGCTGGACGTGGTCGAAGACTTAGTGGTTGTGGTTGGTGACGTAGACCCATTTGCCGAGCAGGCGCTGACTGTACGCTTCCTCAGCAAGGAACGGGATGGAAAGTGCGGTTTACGTACTGGGGCTCCAATCACTGTTGGCATCCCCACTAGGAGTGCTCTGCGACCACCACTTGGCC

>AT512

ATCGCCGCGTGAGTTGGGGTATAACATTGTTGTACATGTACTGGAATGCAAATTACAATACATATGCAATGTGTCCGTGACTAGCTTGCCACACATATGCGTTGCATATGTGAAAGGGGTTGCATGCTCATTCGCCGAAGAGTTTCTGGACGCGCTTCAAAACCGAGTCCTTGTTGGGATCTAAAGGGAGCCGTTAGTTTAGAGCCGAGCTGGAAGGCATTCCGGACAAACCGTATGGATGGTCCTTGGACGGGATCTCCAGGAGAGCGGGGAACGGTGCTTGGTACTTGTCTACCATGGGTCTAATCTTCTCGGCGACCTGGAAGGGCGATTCATAAACGTTGAAAGTTTGCAGCAGAGGACAATTAGAGAGGCGTACGTGCTGGTTGATGAGCAGGATAGCAACATCCTTCCGGGAAGTAAATTCCTCGAACGCAGCTTCGATAGAGGGTATTTGTGTTTCTAGATTCAAAGTCGTGTGTGAGCTTCAATAATAGTGTGCGACCTGGCCACGCACTCGAATCTACAACAAGGAAGTTCTTGGTCTGGTTCTGACCAACGTTGCCTATGCCGGCTAGTAGGAGCCCAGTGACTGAGTCCTGGGCGACTCAGCATACATACAAATAATGGAATTGTAGACATACCTCATCCCCAATGACCGCAAGCAAATTCCGATCCTGGTGGCCAGCCATCGGGTCCTAGGTCAGGGTCGTTGTGGCCGAAGTCGCGCTCGCCGCGCCCCCCAGCCGCGGCCGCCATGTCCCA

>AT519

CCCGTTGCAAAGCTCCACATCGCCCACTGCGTGACGTCGTCGACCAAGCTATCTTCCAAAGGCCCTCGTCGCCGCTCAATGGAATTCCATCGAAGACGGGCTGCGTGGTATAACCTGTAGCCAGTACATGACTCAGTCTCCACTTGCAATGGTAAAACAGGGGTCTTACCTTGCTCGACGAACGTAAAGCGCCCTCGGCCACTCCTTCTGTCCTGCGTAGTGCCGCGCGATATTGGACTCATTCTGGTACCGGTCCAACTGGAGATAGTAGCTAT

>AT52

GCGTCGCGCGCGATGTTCGCGTCCGAGAACAGGCGGAACTCGCCGCCGAGGGGCGACTCGACCTCGCCGACGTTCGAGCCGTTGCCGGGGAAGAGCGTGCCCTTGAGGAGCGTCTCGACGAAGAACTGCGCGTCGAACGAGCTCGGGGTCGAGTCGAAGGGCGTGCCCGGGATCGTCGGGTCGACCTTGTCCTGCGCGGCGACGGAGTGCGACGCGAGGAGGTCGACGACCTCGATCGGGGAGAAGCCCGCGTCGCTCATGCGCGCGAAGATCTTGTCGTTGTCGTCCGACGGCTCGGGGAC

>AT520

AGCCCGTGTACAGCGTCCACAGTATTCTCTTCACCCTGCTGCCGGAGAGAAACGGAGGCCTTGTGAAGGAACTCTCCGTACTTGCGGTGCAGCCCAGTGAAGTACTGGTGACGGGGATCAGTCGGGTCGGAAAGAGCAAAGCCCGAGTTCAGCGGTTCGATGCAGGCGATCATCTCAGGTATTTCGAGCCTATAGGAAGGGCGTAGTCAAGGCAAGCGCGAAGCATGTGGATGATACGACTCACAAAATATCGCTTGTCTCCATCGCTGACCTCTGCTCTTCGGGGGGTATGAACTCCTTGGCCAGTGTCGGGACTCCGGAGAATGCATTGCGAACAAAGCTCAGGTGC

>AT539

CCGACCCAATGGCCAAGGTGATCTGCAGACCACCATTGTGCCTTAGAGAATTTCAGACAGAATCGAGTGGGGTACGCACGTCTTTCAATATCTTGACCTTGGCAGTCAGCCCCTCCAAATGTTCGTCATTCTGACCCTCCAGATCGTCGACATAGCGCTGGCTCATTGGGGCGTAAGGGTTCTCGCGTCCCGCGGCGGAACCCCCGAACGGCGACGAACGTCCAGAGGCGGGTGGGGTGGTGTATTCGGCGGTACCAAGGAGCTGGGATCGGTTGTGCACGTTGGTTCTATACCTGAGGACGAGAGGACGACGAGAGGGGCAGTTCATCAATCAGCATGAGGGAGGATGTATGTAAACTGGCAGTGATTGTGTGCGCACCGTTGCGACATGTTGGGTGAGCAGACCTGCTATACTCAAAACTTCCTAGAAGTCGCGAAGGGAGAAAGAGAACCAGCCAGAGATGAGAGAGCAGTTTAGACGAACACAATAGCAGGTTCGACGTGTTCCGCCACCTCAATGGCGCCGACAGCGCTTTTCCAACTCGGTTCTGTACTTGTACTCGTCC

>AT543

ACCTTGTTTTGGGATACCCTCTGTGATATCTACGGTGTTCCTCAAGCATGCGACGCAGAGGTTTGCAGAATTCGGGATGATGGGAGTTCCACAATCGGCACAGAGTACACGGTGGACTGCAGGCGCGGGGACGAACTCCATCGTAAACTTGAGCGATCGGTTGGCGTTGACGTTGAGGGGCACGATTTGGGTGAAAAGGCTGGAGAGCCTTCTTTCCTTCTTCTCTGGGCGAAGATGAACGAAGATAGAAGTAGGATTTCGTCAAATGAGATCTAGAAGGCCACTGGCAAGTTGCTAGCAGCGATATAGTAAGTCAAACACACAATTGTAACCGGAGAGCG

>AT55

GGCGGATGAAAACGTCTTCGGGGAGAGAGAGATGGGAAAGAACTGCAGGCAGAAGTTTGTACGGGCAGTCCGTAAACACAAGAGAGCGAAGATAGGGAAGGGGGATGGGATCGAAAGGGGGGTGCCAGGAGCCCATATACGGCTCCTCGTAAATGAAAACGTCGACGTGGAGGAATTCGAGAGCAGGAGTGTTGCAAAGAAGTCGGAGTAACTCAAACGGATGAAGCAGCTCCTGCGTCTCGGGATTGAAGTAGAGGACCAGGTGGGTCAAATTCGGGAATGCAGAAGATGGCAGCCAGTCGACTACGGGAAAGAGGGCAAGGGCCCGTAGTGACGAGGCATCGTCAAGCAGCCGCGCCCAAGATACTACGCTGAGCGACGAGTAGCCCGCGAGACCCGATGTCATAGTAAGGCATTCGAGTCGAGGTGCTGGCAACGATGCGAGCAAAGGGACGACATAGTCCCATGTGGGGAGCATGCCAAGGTCAAGCCTACGCAGTCGCGGTGCAAGACCTCTGACAACAGATCTGAAAGGTTCAGGCAGGCGTCCACCGCAATCCACAAGTTTGTGTTCCTCATCGAGGAAGAGGGCGACCGGTAGCGGTCGCGAACGTTCCAGGAAGGCGTCCATCTGGTCCTGACATCGCCCGTGCACGCGTGTCCACAGGGCGGGGGTTCCGATGGCAACATCTCGCCAGTATCGACAAACATGGGTGAGGAGGAGCAACGGTTTTGTGT

>AT567

TAATTCTGTTACGGGTTTGGGGTCTGGGAGGGGGCGGCCTACCCTCTGAGAGAGCGACGGCCATGAGGTGGTCGTAATGAGCTGTCTCTTCGATCTCCACTAACTCGTCTAGAAGTCCCTTGTTCGCGACGTTGTCGACGTGATGATCTTTGGCAAGATTGAGGAGACCCTCAGCCTCTTCTGCAAACAACGAGAGTGCAAGGTCGACATCGCTCGTGCCGGCAGTGTCGGAGGAGCTCCCGCGTCTTGCGAGAATGTCATTGATTTCCCTCACACTCATGTTGGATATCATTTTCATGAGTGCGGTCAATCCAGCATGAGAGCTAGTTGGCGATCGGTTCCTAGACGACTGTGACGACATGGGAGAGTTAGGAAGAATATGGGTGAATGGTGAAAATATGGCGGTTTTCAGCTTGAACTTCTTGGGGAGAGGGGGATAGCGGGCAGGCAAACTAGTCAGGGGCCCTAGACCATTAAGTATTGTGCATTCCTTGGCTTCACTGCATTGGTCACGAAGAACGTGGAGATCAACGTAGATCAACGAAGATCAGGATTAATAGGAAGGTCGGACCTTCATCGTGTTGTTTGCTGCAGTTAG

>AT578

GTCGGGTTCCGACGCAGTACGTTGAGGGCGTGCCCCTCGGGACCCTCACCAAGATAATCAGACGAATATTTGCCCTGCGTAGAGGTAGCAGTCAACGAGGAGGGGGTGAGGAAAGTGGGGATCCGAGCTCGGCTAGTCCGCGAAGCGCTAGTTGAGCTACTACTCCCCCTCAAAGACGCGAACTCCTTCGTGACGGCGATGCGGACGACGATAAGACATTGCGAAATTCCGAAGAAAACCGGGAGGACGGAAGCCATTACGAATCCGACGTCCAGGTTAGTCTGCAGGGAGTATGAAAATTAGCGACCAAAACTCGGCGATTGTCATTGGTGCAAAAGAGCGTACCACGACACGACCGTTGGGCGCAAGGGAGCAGATCTCGTAAAGGAGTAGGCCGACCCAGGTGACGAACGCAGACTCGATGCATGCTCGTAGGACAGTCTCATACACGTCCAGATGGCCGCTCAGCTTGGCAATGTAGC

>AT603

ATCGCGGTGGATCTTGACGTTCTCACCAAGCTGACCAGTCTTGCCCTCGACCTTGATACGGTCGTGCAAGAACTTCTCAAAAGCCGCGCCATCGAAGACACCATCACCGGCGGGACGGGAGTAGTCAATGACGAACTTGTGCTTAGCGGCAGAAGTCTTGGTCGCAGCCTTGGGCTACAGTCACGGAGAGTGCGGGGAAGAGGAAATACCAGAGTTAGCCTACTGAGAAGGTAACTCAGCAAAGCAGTACTGGGAGCCAACTAACCATCTTGACGGTGAAGAGAGGGAGGGTGGACGTCGAATCTTGAGGCGTTGAGGACACTTGCGAT

>AT610

GTCCTGGGGATCATTAACATTCGCAACCTTCCAGTCAAGTGCATATATCTACTTGTACTTGTATCTTGGATCCATACGGTCGAATAGAACCTGATTTGTTTCAAGTCCGTGGTAAAGCCAACCCTCATATAGCACTGCGTGGTAAGTACACCCAACGAGCCATGAAGTATACTACTTGACTCAGCTGGAGCTTAAATCGAATACAGTACATGGCGTATCATATATCATGGCCGACATAAATAGTAAATAGCCATTGTGAAGAGTACTTGTCCAAGGACTGTGTGTCCACAAACATCTACCAAGCCAACCTTGCAACAATGCTCTTCGGGTACTGCCTGACGATCAGCTTCCGC

>AT617

CGCAAGGAGTAAATCGGTCGGATCCGCGCCGCTCGTATGTACCCATATCAATTGGTCTTCATAGTCCATCCGTTGAAAGATTGGGTCACCGCTGCGCTTCTCGACCACCGACAACAGACCCACGGCAGCGTCCCCAAACGTCCTCCACCCGTGAGTACCCGAACCTATTGCATCGCAGAGATACGAGAGAAACCGTTGGATAGCATCAAGATTGAAGTATGGGGGGGTTCCAGCCATCCACATCAGGGCTAGAAATTGAGTAGTCTGTCGTATAACTCGCGCGGATGAATACGTCAAATTGTCAAGCAAGAGCCTGACCAGTTCAGTGTGCGTGTACAGCCGCTAGTGTAGTAGACACACACCTGCTTGAGGCTTGAGGTAACGAGTTCTAACGTAGAGAAAGTTACATCTGTCGAAGAATTGGCAATTCAAGCTATTGTGTAAGTACTCACTTTCATTTAACCTCCCACAGCATTCGGCCTTCGGGTCGGAGAATACATTGGCGGCTACTTTCAAACTAGATGGAAAGAGGGAGAGGGCTTCTAAGATCGAGTCCTTCGCT

>AT628

TCCAGGTGCCTGGTGGATAGTTGCGCGAGGGACCAGGCGGCCAGGTGACGGACGCCCTGGTGTCAAGTGAGTATGGGCTGCCGCATTCCTTGAACAGGCCCCCGATGAGACAAATACTTTGTCTCGAGGGGGCATCTGGAGAAGAAGGTCGCATCAGAGGTTATAACCACAATGGCAGCTCGGGGAACAACTTCACTCCTTCAAACAAATAAAGGGGTTTATCTTCCCTATTGCGTGGCGGTCGCCAGTCAAAATAATCCTTCGGGAGTTCGGATATCCGACGATACACGCGTTCTGATATCATCTGGTCAGTGGTCTTCGCTGCAAGACTAGCAGCGAGCTCGTTTTGAGCATGCTCTCTGTCGTCTACATCGAGCTGGACCCTCTGCGAGTAAGAATGATTTGTCCACTAGACGCAGACCCATCAGTAACGATAGAAAGCCCTGTCAGGTACAGACGTTACGTTGGGTTGATAGTTGAGGTTGAGTGCCGAGAGAGAAGTTATATAGTTCTCTTCTTCCTCCCGTTCGATCTCCCGTTGAATCTCTGCTTGAGTCTGTGTGA

>AT656

GGAGAATGCCAGCAATGCCTATGCCAAGTTCACAGGTAGTGCTCGTACACACCCTCACGCTGAGTTGTGGCAAAACACACCTGATTCCGCGTCATTGAGATTGGTCGCCATTGGTGGTGGTGGACGTGCATTGGCCGCGGCCTGATTGGCCGTCGTATTGATGTTCCCGGCAGGAGCTGAAGTGCGTTGTTCATATTCAGGATCATCGGCTTCAATAGTCTCGAATAGAGGTGTGGAGGAAGTCATGGCGAAGGGAGGGGAAGGGAAGTAAGTAGCATAAGTAAGTAGCCGTAGTGACAGAGTCGCCGCGCTTAGCCTTGTCTGCGCATTATGCATACCA

>AT660

CCGTCGCGATGGCATCGACTCCCCAGTAGTTCCAGCGGGCGTCGTCGACGAAGTCCAGAAGAGGGCTCCAGGAGTCGTCGGAGTTCAGCTTGTACGCGCCGCCGATGTCCGTGCGGGCGAACGCAAGACCCTTTTGGGACGGATTGAAGACGATATTAGGGACGAAGCCACCGCCACCACCGATCTTGACGTTCTTCCAGGTATAAGTCTGGGAAGACGCCGCAGCGACGATGGAGATCGCCGCCGCGGCGGACAAAGTCGCCAGAAGTGTCGGGCGAAGCATGACTGAGTAGGATGCCTAGGATTTTGAGGTGGG

>AT670

ATTTTTTGTTTGCGACCGTAATGGGTTCCATGCACGCCTTGCCCACCAAGAACCTGATGTACTTCTGGTCCGAGGCCTCAACTGTGCGGATTCTTGCTGCAGGTTCAGCCATGTTGACCGGCAGAGTTGGACGAAGAAAGCAGGAGATGTGTGCGAATTGAGGCGACGTACGGAAGCAAGAAACGGCATTGTGGACGCTGGGGGGCAGGCTTG

>AT679

CCCGCAGCACATATCGTCCGGTGCGCCCAGCATCTTGAGAGCTTTGGCCTTCAAGAGCTCCCTTCGAACCCGCCGGACGCGAGCAAGGTTCTCACGACTAGCAGTGGTCCCCTTGAAGTTGTGAATGAGGACGCCGACGTCGATAGACTTCTGGAACCACTCCTCGGCGCCGTCGAGGTTGCCCATGTGGAAGTAGAGCTCTCCGAGGCCGCTCAAGAGGTTGGCGAGGCTCGGAGACGGAACGGTGGGGTCTACGAGATCCAGAGCCTTCCGGAGTAGGCGTTCCGCATCATAGTAACAGCCGCGGGCATCGAGTTGGCGTGCCTGTAGGGTGAGGTCGGAGACTTCGATGTCGAGCGGTGAGGTGCTAGGGGAGCTGTCGACAGGGAAGGTGAGTGTATCGGTGTCCATTTCGAGAGGGTGATGAGGGGGGATGGACGAACGGGACGCGTAGCGTTTGAAGCTAGGGGAGACGGTTTATAATAAGTAGTGCTGTAGGATCAGGACTCAGGAGAGGTAGCGGAGACGCCCCCTAGAACACCATGCATCATGCCACACTCGGCGGGATGGTTCATGCAGCCGACTGGGACTCGGC

>AT68

GGCGGATAAAGAGGGCGGCGAGGATGAAGGTCAGGGTGTGCATCATGATGGTAGGAAGGCGGGTCGAGCAAATGGGGATAGGATGAGGGCTGGGAAGAGTACGGTCGATGGAGGCGAGGTTGACCGCGTTCAATGCCCGGTTCGTCCTAATATACCGCTTTTATTTGGAAAGTGCGTAGTAAGCCTGGAGCAAGGCGATTTGCGCGCTGCGCGGTGCACAACGGCGTCATGATTGACCGAAGGCTCTCGCTGGGCGGCCCCTCCGCTCCAGAGAAGCGGTGCTCTACGAGATGGATTGACACACCGCCGGGACGGAGTGAGGAGTTGGTGGCCAGCGAGCGGTCAGCGAGGAGGAGTAATTCCCACATCTGCGAAACAGAGGCTGC

>AT681

CGGACGCAGTCTGAATTTCAGATAGCCTACCATGGCTTATTTCTCGTTTACTCACCTCGTCTGCCCGTGAAGAACAGCTGCGTATGAGGTGACCGGGCAAATGAGGGCGTCGATAGGGCGCCCCGTGCCCGTCTGGGATGCGGTCGCCTGCCAGCGGTCCAGCAAGGCCTTACGCAGCGCCTTCCTCTCCTTGTTCAACTGCCACACCTGATACGCGCTGAGGGGCATGCGGGGAATGCGAAATGCGGGGACGTGAGTGGGGTCGGCCTCGGTGTCCATGGAGTTGATCATTGGTTCCCCAGTCGTCAGACAAGAGTTATAATCCTCGCTCCCGTCCGCAAGGAAGGCAGACCGCT

>AT688

CGCCAGACGCAACAAATTCCATAACAAGGGGCATGATAGCATTACAAAAGCAGAGCAGACAACAAGATAGCAAGGTATATGGGGTAAACAAGATGTTCAGATCGGAAGCATCCTATACTCAGTCTGTAGATCATGGCAACAAGTTCGTCCCAAAAAAATCACTGCTCTTCGGCAGCAGGACGACCTTTACACATCATCGGCAGTCTTACCGTCGACGCCCTCCGGGCCTTTGACTGCGCCCGTCTCATCTCGTCTTAGTCGCTGTCTTCTTCATTGTCAGCGCCTTCGGCGGACTGCAGAGGCGGTTGTCCAGGAACACGCTGTCCCAAAATCCTTCAGCTCTCGCCGCACATAAAACAA

>AT694

GTCCCATACATCGTATATGTTCTATACCTGAATCTCGGCTCTGTCACGCATGCGCCACACATTCTCTTGTCTTCTATCTAGCTCATTCGGGTTGAATTCAAAATGTTGTACAGCATCTATTTGTAAAGAGTCCGTAGTTATGCATCTCGACATGAAGTCGTCAGAAACCGGGGTCTGCAATGTACCGTACTACTTCCTGTGAATGCGTATGCGGCGATAGATGTCTTTGGCTCATCGGAAGTTGAAGCAGTGGAGCTCAGGTGTCATGAGGCGTCTGCGCAGCGTCTGCTTTGGCATCCTACAAGGCATCCTACAAGAGCACGAATGCAAGCATGGGAACCACGAATGCCAGCCACTTCGTCTGTCGGAGGGCGTGTTCGCTCCCTGCCCTGTTGACGAGGCCAGTGAGGGTTTGCTTCCGATACACCTTCAGGGTGTTGATGTTCCAGGACGCGTTCT

>AT696

GGCTCGAGATGCGCACACTAACAACGTACAGAACGGTGAACATGTGTAAATATCCGACATTCATCCCGGACAGACCTGTTGCGGTACACTCCGGAAGTAAGTGTGGTGGTGTTGTCTCCCTTCATGATGACTTTGCCATCCTCCGTCACGTAGACGAGGTTGTTGGCAAGGGCCGTTGCCCTATCAGTATAGCTGAGAGCGACGGATCGGAGGTAGGGCAGGTGC

>AT709

CTTGTCGCGTACAAGCGTTTCCTCCACCGCAGAAAGGTTCTCAAGCGGACCCAAAAGCACATGCGCGGACTCCGGCCCACCGATGTATTCCTCAAAGTTCCTCCCCAACTCCTCCGCGAGAGCCAGAAGAACCTCATCCTCGTCGTCGACCGAATCTTGTAGGAAGGGTATCAGTTCATCGCGTGCACGGTCCGGGCCCAGCGCGAGAGCAATGGTCGATATACGATGGATGGCGTTCAATCGCAGCTGGACGTCCTCCGACCGCAGCTCATCCATAAGGATGGCAATCGGAGCAATCTGAGGTTGCAGCGCGACACGGTCAGCGGATGACTTCAGCATACGACAATGCAGAGGCCACAGACATCGTCTAGGGATGTATTTGGGTCCATGTTGTGGTTGCTTGCAATGAACCGTCTCGGCAAGATGTTGCGAACTAGCAATAGGTGAGGGCGAGTGACGTGCAGGTGCTGGCGTTAAGCAGGGGCACTAGAAGGGCAGCGGGGAAAGAGAGCGTAGAGTACGAACAGAATGAATGGATGGATGAAATCGAGCTCGTTGATGACCTTGTTTGGTCACTTATCGCGCCCTCCATCCTTTGAACACGGGCG

>AT716

GAGGCGTATTGAGATATCGATCCATGTTTCCATCATGCGTGTCACTAGTAATACATGAATATACGATGGCAAATGAAGCGAGGCAAGGCCACGTTTGTTATGATGAGACAGATGAGTCCACACCCCCAAAATCCTTGCTATAGCTCCAGAAATCCAAAACCTGGACGCTATCGGAAACATCCAGACCCGCTCGGAGTTCCGGCAACAAACCAACCCGTCCCAACAGAGACAGGAACTTCGCGCGTGAATCTGGGTATACGAACAGACCTAGAATGTCTGAACCCTACGGGAGGAACATGAATCAGCAGGTGGATAACAACATGTACGGCTCCATGCTTCCTCACATGGCGTCGTATGACGGCAGCGACATGTTCCCTGGGTGCCTGCATTAGGAGGACACCGACATCGGCGGAGAGCTTATCACCTCGCGTCTCTGGTGAACTCGTCGACGTTACATCCTCGGTCGAAGGTATCCCTGATGAGACCTGCTTCCCCGCTTCTCCGTTCTCTGAGGTATGAAGTAGCGGAACCAAGAAAGGAGAAACGACTGGGATTCGTGGCGACGAACGGAAAACTGTGGCTGCTTCCTCAGAAACCCCCAGCCAGTGGGCCCAGCTCTCGAGTATCCTCCCGTCCCTGGTGAGATGGCGGAGGGCGTGGTGGCATCGATCCCAATCGCCCTCGTCGCCTCGCCATACCCCAGCCTCCTTGCTGTCGTCCGATGGGGTAAACATGGCCGATGGCGGCCGCGTCGCCCCCTCTTCGTGATGTTCCAGCTCCGGGACGACGGAGACATTGCCGGTGTTTCCGAGTAAGCTATCGGCTTCGTGCATGAACTGGGCCGGGCGCATCATAAGGCGTATCCACCTTCGACGCCGCACGAGTCCGCGGTTGCTCATGAATCCCGTATCGGGTCCCCATTTCTTACTTCGAAAGGAGCGGGAATATTCGAAACCGTCGTATTGCACTTCGCCGTCGCCGCGCATATCGATCATCCAGGCCCTAGAGACCCACTTCCATGAGCCGTCCGGGAGTGGATAGTCCTCCAAAGTGACGGTGGGCTGGTTCTTGCGAGGAGACCTTGCCGCGGTGGGAATGGTGAAAGGGGGCGGGTCCAATGGCAGGAGAGTGAGTGGAGAATAGTACGCAGTAGAGAAGACCATGGCTCTGAGACAAAGTCAGTTGGAGGAACAACTGAGGCGAAGGTGTGAAGTCATTCACGCACCCTCGTTGGTTCTCGTACAAAACCGCCCATCTATAGACGTCTTTGTCGTAGTTCTCGTCGACTTCCGCAAATACATCTCCAGCAAGATCCACCAAGTTAAGGGAAGCAGGAGGTTCAGAAGTCGACTCCTCTGGTCCTTGAATATAGAAGCGGGGTATTGATGACTTCCGGGACTTGGTCCTTGAGCGGTGGAGGCGGGGTAGTTGGAGTGAGACGCTGGACAATAGCCCACGCTTATTTAGCCGTTCCCGGGCTGGATCCGCGGCCGCATAGTCGTCCCTTTCGAGGGTGGCAGGTAAAGGTGGTGGCAGAGATTGTGTAGAGTGTAATGAAGACGGACCTGCCGGAGAGGAAGCGAAGGAAGACACGTCGGACATGGCTCCGAGTCAAAGGTGAGTTGA

>AT722

GCGCCGGGATTTACTCACATGATAACCTCAAACATTTGACAGCGAAATGCGATACATTAGACGTCGTAGATTCGACGTAGAACCTCAAGCTTCAACGTTCGTAGCGTACTTCACGCTCTATCGTCCAACCCCTGAAGCCAGGCCTCCGAAAGGGCTGGTTGACGCACATAGGGTTATAGAAGGGATTGGCACTCGCGCGGTGCATGGCATAAACAGCTTGAAGGAAACACGTTATGTTTCAATTGTCTAGATTCTTATGAGCTCGAAGATGAGTCCAACATTGAGCTTCGGCCCAGAATTCCAGAGAAAACCTTAGTAATAGATACATGTCATTGATGCAGATTATAGAGAATGCCATGATAGATGAGACGACATAAGAAAGAAGTGGAATCATGTAATGACGAGAGCGTAGCCGTCATGGAGATCCGCCAGCAGTGCCTTCTCCCTACATAACGCCATGAGCGGCCAGACCAACAAACAAACTTAGACTGGGTGACAGCACTTACTTGAGATCGAGCTTTGCATTTTGCACGGCAGCTAAGGCTGCCGCCCTAATCTTGGAAGCATCCAGGCCAGAAGGGAGTGCACAGATGAGGCACATTGGACAATTCGCCGGCTGAAGCCGGTAAGAAGCAGGAATCTGGGGAACATGCTCGTCGGACTTGGGGGCCATAGCATGGGAATAGAGAGGGACTCACGCCGGATGCACGGAGCGACTCCACGAGGTTATGCACGAACATCAAGGAGCGTGTGCGCAGATCATGAAGGTCGTGAAGGTCGGCACCTGAACCCCCACGATGAGCATGGGCTGGTCGTGTGACCGACCGGGGAGGAGAACGCACACGTAGAGGACGAATGGCAACCAGTGGGGATGGTGCAGTGCAACCGTACGGAGTGTGCGGACTTCTTAATCAGTCTACGGTAAGGATGTCAGACACGGACGACTACCAGTGGTAGAAAGATGATACATACTGAGCGAAGGGTTCCACTGCGTCATCCAGATTACCCAGCTACAAGAGAAGCGTCAGTCAGCGTTACGCGAGATGACGAGGATCGTAGACAGACTTCGCTGAACTCGACGTCCTTGGTCGAGTGGACGGTATCAAGATGGACGGGGAGGTGGACAGCGGGGGCCATTACAGACATCATGGTGGAGTGGGGAAAGTGAGGGAGGGTGTGGAGAAGAAGCAGGGCGGGGGGGGTCGTTAAGAGTGATGAGGGGGTGGGGTGGGGTGGGGTCTGGAGGATGTAGAGCGTATTAAATGTATGGGTTGGGTGTAGCCTTCGCGGGGAATACCGGAGGAGGGAATGCCTGGAGGTGTGATCGCTATACCGAGGGTCATGTTCTCGGTTGCAAGAATATACGACAGCATGTTCAGCGCGTTACCGTGCGAGCCACAGCCTCGGCGTATCAGGCCGTCACGAGGGTGCCAGCTGGACGGAGCTCGCCCACGACTGGACTACCATGACAGGACTGACTGGAGGACAAACGCAGGACGGAGCGCCGTTCGACGCCGCGCAGGAAGAGATACATACTGTAGAGATAACTTAAAGTCAATAGTCGAGTCTCGAGATGCGGCTG

>AT736

TGTTCTATCTATGTAGCATGACCGCCGAGATCATCGTCGAAATCACACAAACTATACGAAGATAACCACAGTGGCGGGCATTCGTGATGCTCTAGTACAAGTACAAGAAGGAACAAGCACAAGTACAACAAATGAAACGAGTAAGTACAAAGCCCGACGACGAGCACCAGATATCTGGATTGCCTGAAGACGATAGACCAGAGAGTACCATCGAGAGCGGTCGTGACTGATGGATCGTCAGGCACCGTAAAAGTCATAAACATGCAGAAGGATCCAAGAACAGCGACAAGAAGGGTCATAGTCCGTTTGCACTGTTGCGACTCTGACGGCTCGAAGGTCGCCTGCCACCGAGCTCCTTCGCGCGCGACCAGGCGTTCTGGAAGACCCATATCTCTTTCCGTTCACGGAGGCTGAAGAGGCTCCGAGTGCCCATCGGCACACTCCCGGCAAGTTTGAAGCCAGCACTTTTGAAGAACCGAAGAACGAAGGCGATGAGAATGCTCCTGTCTACCTCGACTACAAGCGACAGTCAGTTGGGTAAGGAGAAGGGAGAAGAGGCGTGACGCACCGCCATAGCTGCTCCTCTTGATCTCGAAGATGTGCCGACCATCCTCGGTGGCACGATCCGCGGTGATCTTCTTCGGGAAATATGCGCGCAATTCCACGCCTAACATTTCGCTCAGCTCGTACGGGGTTTCCATGAGTGTTAGTCTGTCGCCTGCTTTCGAGAGCATCATGAGGAAGAATTCAGGTGCGGGGTCGGATGGCGATTCAACGAAGATGAGTTGAGGAGGCTTCCA

>AT740

CCATGGCTACCAATCCTAGATCACCCTCCTTCTTCAGGTCTGCTTTGACGATCGCCAAACTGCGGCCAGTGGACTCGCTGATGGCCTTGACCAGCAAACTCTCGCCAATACCTAGTTCTATGCCCATATAATCAGGGCAAAGCTGGGACACGATAAATCTGAGCAGTGTATTCAGGAATGGAAAAGGTGCAACGTACTCGGTTAATGCACAGGTAAACCGCTTGCAGCAGCGACTTCGTGTCTCCTTTCGCTTTGCGTTGAATGACCAGGAGTAGAAAAGATGTCAACAGAGATGTCTTTTCGATGCGCTTGGTGGTAGCCTCGATCAGCGAGAATACCTTAGCCAATGCAGCATAAGGCACAC

>AT78

CCGCCGCCCAAGCTGCAATAGGGAAGAGAGAAGCTGCAAATATGTAAGAGAACATCATCCTGTAGAACGCTCACAGGGGGGAAGCAGGCAGAGGAGAAGAGGCGGAGTACAGCACGGGTTAGACCTGCCTGCTATATATCAATGCAAACGCAAAGGAAACGAGGTGCAGAACAAATGCAAAACGGGTGCTGAAACGCAAAGTCGCCCCGAGGTACCCAAGCGAGTGTGCCACGCCGATCTATCGCACGCCGCCACACTGGGAGACGAGCTTAGAAGGGATGCCGTGGCGTTGTGGTCGTCGTG

>AT783

GACGGGTGGAAGGCCTGATATCCGGGTTATGAGTACGTCCATTGATCTATGACACGACGCACGATGACACCCACAGAGCACAGGACAGCACCTGCATGCCCACCTGAGCGTCCGCCAACCATCACTGCAACTGCCCGCCCTATGCCTTCACCTTCTGGCTCGCACATATGTAACCCATGAGCAGGGCCAGCGAGACTGGGACCTTGGTTCGGAAACCAACGCGGATTGTTTGGGGTCTGCACAGGCGGAAGCGTAAGGTCATAGATCCGAGCAGTGAAATCCCTAGATGTGGTGCAGAACAGTTCTGGGCTAGATGGATGCACCGACAAAGATGTTATAGGCTATGAGACGGATGAGGTCAGTCAGCCCGATGAACTGCGAGCCAAGAAGGAATCGTACCCCTCCATGACCCCGCAGTTTCCCGACAATTTCGCGGGTTGTTACATCGTATATGAGAATGACACTGGAGACGGTGAAAACTAGCAGTGGTTTGAGTGGTTCTTTTCGACTGATAGCCCATGCGACGTTTGTGATCTCCGCATTGGCCTAGCCGAGCGTAAGCCAAGGAAATATACCGAGCAGTTGACCACTCACGTCGGATACACTCAAAACGGTTGACTTGCCGGTAGCTAGCTTAGGGAAGATATACAGGCAGCCTGTTCCACCTACAGCAATGGCCCCCATCCACTCTTTGGCCACATCCCGCCATCCGGACACCAGATCCTGCTGCATGTTGCCATCCCAGAGAACATTTGAAGACTGTGTTGTCCAGGGGAAACATGCAACGCAATGGAAGGCGCGGATACGGGACTGGTAGATGATTCAGTATGCCGTACTAACCTTGACTGAGTGAGGGACGCTACAGACATTCAGTAGGGTGAACAAACTGAACGGCGAGGAGTCAGTGGGACCTCTGAACCAGGGTTGAGAATCAAGATCCATGTTTGACGGGTATCGGGCATGTAGAGAGGTAGTTAGAGCAACGATATGTATTGCGAGGGATGAAAAACGCGTGGTACGAGGTCAAGGCGGGGACGGAGGCGTTGTGGAGGGGGA

>AT789

CCTTCATGTTCCCGCGACACGCATGCACCCCAATCACCAGGTCAGCCGGGCGGTCGGCGGTGATCTGGTTGTACACGGCGATATGGGTCTTCAATAGCTCCTCACGATCCACTCCCGCAGCTTCCATACCCCCGATTGTCACTTCCGAGCAATAGAACGCAAATCCAGGATCGTCAAATTGAACACGTCGGCAGCCATGGTCGTAGAGGTCCTTGATTTCCTCGCGAAATACCTTCACGATGTCGGCGAAGTACTCGGCTGTGATCAACCACGAATCAGAGTTGAGTAAAGTAAATAGGCTGTAAATAGCCACCAAGACAGACAGGGCTATACCGTCATTCTTGTAGACCGCGTGTTCGTAGGTATGCTCCGAACCGTGTCGCAAATGCATCCACGTTGGTCCGCACATCGTGATCTTGATGTTCGGGACATCCTA

>AT790

ACCTTCGAACTTCGCCGTCCGTAAGGACGATCAGGCCGAGATCCTTCTGTAACTTGACGATTTGAGGAACAACCTGGTCCTCGAGGGCCTTGATATCCTCGGCGGAACATTGTCCCGAATGGAAAGCGATGCGCTTGTCCACAAGCTCCTTGGGGCGAAGGAGAGAGCCAACATGGTCGGCATGAGGGAAGAACTTTGCAGCAACGCTAGCCGGAGCAGTCATGGTGGGAATGCGGTGATGGGGTGTCTCGCTCGCGACGAGACGAGGTATCG

>AT795

CACCTACTCGAGAGGACTGGCCCGAGACTTCCTGGGAGACAATGCCACGGCCCATGTAGGCCCAGGTGTCCCTCTCTGGCCTTCTGATGTGGACCTTGACTGGGATGTCGCGGAAGGGATCGCGGCCCTGGGGGACACCGACTACAGCTGCTCGTATTTGTTCGTAGAGGCGGTCCATTTCGGTCAGAACTGGCACAGCCAAGACAGGTATGTAGGCAAATGCGCGCGGGTGACGCAGGCCGGTACTCACGTGTATTGACATTGGTGCTGGGATGTTGTGTATTGAACGTTTGGTGGAGAGGTTGGAGATGCTGGTGGGATGAGGTTCCGATGTCCATGGAGGCGTCCATGACGTCCTCCATGTTCTGATCTTCAGCAT

>AT800

CAATTCCCATGCCGAAAGCAATGGTTGCGACAACGATGCCCTTGTCGGATTCCATGAAGCTCAACTGAACCCGCTCACGCTCTTCTGAAGGGAGGCCGGCGTGATAGATCTGAGCATCGATTTGATAAGAGCGCAAGGTATTCGCCACCTCCTCGGCGTGCTTCTGCAGCGTTACGTAGACGATGGCAGGCCCCGTACGCTCTCTCAAGGCGCTTATGAGACGGGTGGTCTTCTGAGTCGCGTTATCTAAGACAGCGACGTTTAAAGAGAGACTGGTCGGCCGTCGTCAACTGAATTATTCGACAACATGTGGCAAGGACGCACTTGTGTCGGTACACGGGGATCCGGAAGACGCCTGCGTTGCGGTCGATGAAGAAGCTGTCACAGATGTCTTTGGACACAGAAGCAGTAGCCGTAGCAGTGAGACAAAGGACTCGTTCGACGTCCATTTCTTCGGCAAAGCGAGCAATCTTGAGGTACTCTGGCCGGAAACTCGCACCCCACTACGTCGAAATGAATAGTAAACATATGCCCGTCTGTTCGATGTAACACCCAAGACTCGACGCGACAGACCTGAGAGATGCAGTGCGATTCGTCTACAGCTAGAAGGGAAATCCGCACACGACGCATCATGGTCATGAAACCCTCGTTGTTTAGTCTATGTCAACACTCAAATCACGACCGAAGGTTCCTATGACATGTCATACGATACTCACCTCTCAGGCGCGACATACAAGATCTTCATCGACCCAGTGATGACCTCGTTCTTGATCCATGCGGACCTACTGGCGTCTTGAGTGCTGTCCAAGTTCGCAGCCTTCACGCCCTTCGCGACAAGCGCGTCCACTTGGTCCTTCATGAGCGCAATTAGAGGCGATATCACCAAGGTCAAGCCCTAAA

>AT801

GCTCGAGCCGGAAAGACGCGAATCCAAAGGTGTTTTTGAGTATTTCGTGCGCCTTGTGCATCATGGGGTCACTGGGAGGCTGCACGTCCCCCATGTTGATGCGGGAGCTTAAGACAGGAAGACAGAGGTCGAGAAGAGGAGTCGACGACGCGTCCGCCGAGCTACTGCAGCTCTAAGCAAGTGCGTCAGACTGACGGTGACGGCCAGAGTCTCGAGGCTGAGC

>AT807

TTGCCGTGCACGCGGACAGCTCTTCCTTGAAATTGATACCTCCGGACCGTTGCATATGTCGGATCCCATGCCGTCCAAAGTATCGAGACAACCACAAGACCAAGCAGTAGTGAGGGAAAGAAACATGGGAGCGAAACAAGACGATAGGAAGCGGCGACTGACAAGACGACATGAGACAGGGATTGCCGCCATTGCATAGTTTGACTTACTGGACACATGACCAACTACTGCACACCCCGCGCTGGCCGCCCACAGACCACCGCGAATACGCCATATCCACAACTCGCGGTTCAGCTTTTCTCTGTCCTTTCTTGTGGCTGAGGTGCGTCTCCGATCGTCCGTGCCCCGAGTGTTACGCAATGCGCTACCAAGCGCACGCACGCGGGCCATTTCATCCTTTTTCTTGATTTCTTGTTCGATGGTTGGCAGACATTTTGCGCACACAGGAGGATAACGAATTTCGATAGAGCGCTGGTACTCTGGGAGCAACTCTAAACGCTTGTCGTACTCGGGATCCTGGTGAATGATGAGGAATGGAATGGGTGCGTAAAGAGGAGGGCACCCGCAATAAAATACAGGAGACGTACTTCAGGCGACGGGAGGTAATTCGCTAACAAATTCTGGAGCAACATTTGGTTCGTCTGGCAGGTATGGCAGAATGAGGTATTTCCATGCATTGAAGGGAGGCGGTCTTTTCTTGGAGACGCTACGGGAAGGCAGACTTGTTGGAGATTGGTCAAAGTCAGAGACAGGGCAGTGTACGTACCTCGTCGCGCGAAGGAGTCCTTGTTGGACTTTTCGTCATGCATAGCTGGATAGTCCGAGACGATCTCTCCCCTAGCACTGTAATGGTTCATACAGTCACAATGGGGGCACCGAAACGAGCGCGGGTTTCGTGGGGCGGGAGTTACGCCGGACTGGCAGTAAAAGCAGAAGACAGCGCTGGGCTGCTTGAATATGCGAGACATTAGGCCAGACTAGAGGAGGAAGCAGATAGACCGCAAGACCGACAGCATCGGTGGCTGAGAGAGAGGTACAGTAAGTAAGGGCTGTCACTGTCAGTGTCTCCGTCCGATGCCGCCG

>AT829

CCAAGGCCATGTCTCCGTCAACTGGGGAAGATGTACCAGCGTTTCGGGAACGACATTAGTGCGGAGGTGGATTTCGCACCTCCGAAGCGATCAGCGATAGACCGAATACGAGCGGGGCGAAAAGTGCCTTCTGTATCTATGTACGCGACCTGAAATGCGATGGTAAGATGGCGAAAAATGAAAATGATCGGAGTAACATAGTCTCCCCTTTCCCGATGCACCGCCCATATCGGAAGGCAGCTGCGCAACGACGCTCATGGTGTGTGCCAGCTGGGTCTTCCCAGTCCTGAATTCTCCGTAAACTGCCG

>AT837

TTTGCGCAGGTTACATACAGATCAGGATGTCCAAACTACACAATGCAAACATAGAAGTATCCGAGAGACGCGTACGCGGGCGTACGGATGCTTGAAGATGATTACGAGGCATAGCAGGGTGAAAACAAACATTGTCGGTACTGTGGCCTCAGAATAAACTAGAGGGCGATGTGCGGCTATCCGTGCGATCATTGCTGAGGAGCAGGATCGTGCCGGGAGCCACTTGGCTGGGGGGCATAGTGGCGGTCGAAGTGGAAGGATGGGTAATTAACGCCATGGGCGTACTCTTGCGGCACATGGCCCTGGGAGAACGTGAAAGGCTCCTGCAAACCGGCGGATGTCTGCGAGATGTGCGACGGCGGTGGGTGCGGTGGGTGCCGCTGGGGCTGGGAGTAGCCTGGCAGAGCACTGTATGAGGTTGACGCATCTACGCCTGTCGGAGCCACCATCGGCATGTGCGGGAGCCCCTCCGTGGTCATCAGCTGGCTTGGCCACCATTGATACCCGGGGTGAAAGGATGTAGACGCCTCTGCCTCCGGTTCATGCACGCATAGAGAATGCGCGACCATACGTGCGTGGTGAGCGGAGTCCCGGTTGCTGTTGCTCGAGCTACTAGTACTGCTGTAGGATTCCACAGGGGGCATGGCGTACACATCGCGCACGGAAACGTCGGGACTGTTGTAGCCTCCCAGGGCGTCATCGGCATGACGCTTTCGCCCACTGCCGGTCTGTGGATAATACTTGTACTCGTTTAAGTGTGTCCTTGCGCCTTCCAGCAACGCCCGAAGACGGGAAGTGAAGGGCCAAAGCCTCTGT

>AT839

GGTCCTACGCGCATTGTAAGTAAAGTACAACCCACGAACAGCATCTGATCACTTGCGATTGGCATCAGGCAGCGTATACAGGTCATCCCACCTTTGTTGCGGTGCAGGCCCGCTGTAGGCCAGTCTTCTGCGTGTCCGTGAGTAATCGGCTCCAACCCCGCCCGCACAAGATGAAGACCGGGATGTGGCGCTGACTGGAGAAGAAAGATACGGCCATTGAATACTGCGTCCAAGCATGCGAGACAATTGATCCTGCCACTCTCTTGTCGGAGTCGCGGACACGGTGGGGTCTAATGCAGATGGTAAGGGGTGCATACACAAAACGCTTGCAAGCGTACCTTGAGACACTATCTCCCGCGAAACACGAGACTGCCTCCGGGAGCGTCTATCGTCTCGCAAAGGAGTATGTTCGGGTACGTCGATGAGCGATGTATACAGACTATCGGCAGTCGGCTCTCGCTCCTGTATCCGACTCGCCGCCCCCTACACGAAAGGTCAGCGTCACATCGGCAATGCAGACAAGACGACGGACATATGGACCGGTTTGCACACGATCCAAGATGGCATTCGCGAAAGCATCGTGTCGAAGATCGGTTATGAGGTCTTGTGCCCGCGGTGACTCCATAACGGTTGCAAGGATGCATTCGACCTGGTTCCATCGTTGCTCTATCGAGTGGATGTAGCCTTTGGGAGGACCACGTTTGAAGCTGGGGCCTGGAAATGCAGTTTGAAATATTCGGGGATGACGACGCAAGTGGAATGCGCATTCAT

>AT844

TGGGTTGAGAAAGCGGGATACCCATTATCCATGGATACTGTAAGAGGTCTATTTCTACAAGCTATTATACCTGTTCTGTCCGTGCGTAGCGAATATCTAGGGCTGCTGTCGTCTAAATTCCGGGAGTGAGCCTGAAAGAGCAACGGTTAAATCGTCGCAAATGGCGCTGTAAGCGACATCATACCAATCGTATTTAACCAGGTCGTCTGCATTAACGAGCGCCGCAGAGGCAAGCTCCCGTGCCTCCATATACACCCAGAA

>AT846

ACGGACTCACCTTGATGAACGTAGGCCCTGCGCGTTGCATTTGCCTGGTAAGAAACCCATACCACCAGACTGCCCCCCATCTGTCTCCTCCAAACTTGCTCTCCGGTTGGCCGATGAGGAGTATCGGGGCCGTCAAGAACACGGGGATGAATACGAAACAGAGGTAGACAAATCGCTTTGCCGTCAGGAGAGGCTCCAGTATCCGCTCGCGCAGGTATGACATGACGCGACCTAACAGCGTCCGATCGGATTCGGAGGGGGACATAATGACCGGGTCCAAGGCTTCCGTCTCCGCCGTTTCAGCGCAAGGTATGAGGTCCGGAGAGGCGAATACATTAAGTGGCTCTTGACGCGCAGGGGTAGCAGCAAAAAGGAAGACTCCACCGGCAACAGGTATTACCCATAGGAACCTTTTGGAGAACAGTGGGCCCGGGGCTGCCGTACGCGTGAAGAACTGGGACGAAAACCGGGGGGAGGTCGTCCAGTGCGAGGCATGGCGGAAAGACGAAGTGACCCGTGGGGAGAAAGGCTGCCTCAGACCAGGCCGAAGGAAGCTGCGGAGGGCTATAGGCGGCATGAGGACATCATGGACCGCAAAGACGAGGACGACGAGTCGGACAAGGAGCAGGGCACTCGAAAAGACGC

>AT853

CTGGGCACTCTGACAGACTCTGGCGTGCAAGAACGCGATAGAGATAGAAAGCAATTCCATTGAAGAGCAGAAGATACAAGAACGCGACTCGTACGAGGTACAGGATGTGTTAGCCCGGTCCAACCTGTACCGTGAGTTGTCAGGGCACGCATAATATCGGTGATCGTAGAGGCATACCTTCGGAATATCGTGGTGCTCACTGAGGGCTTCGAGCTTGGCATTGAACTCGCTGACACGATCCTTGTGAGTCTTGCTTGCCAACCTCGCCACCTTCTCAGCCAACTAGGATCCCAACACCTTCAGATGTCGGCGTGGAGTAACGTAGTATGAAGCACACACCCTCCTCTTCTGGGTTTCCTCAAATCTCTTCTCCGCCGCGGTCTTCCTGTCACTACTGCTGCTGCTAGCAACGGCGGGAGTGCTACGCCCGCTGCCAGATGGCGACGCGGTCTTCGCTTCATCTTCCTTGATCGCCCGCTCCAATGCCTGTACGCGGTCCTCGTCAGGAGAAACAGCCTTCTCCTTGTCCTTCTTCGATTTCTTCTTCCTACAGTAGTGTCAGCATATGCCACAAGAGAACGCGCCACAACGCTCACTTTTTTACGATTCCGCCATCCTTGACGCCTCCTTTGAGCTTGAGAGAGCCACCGGGACGGAAGTCGTAGTCTGAGGACATTATCCGCGAAGGCGTGGCGGTCGTTCAACGTTCAACTTCAAGTCATGGAGGGTGCCGTCACG

>AT858

CGAGCTTCTTGTGCAGGTTGTGCCGCAGCCACAATATACAACAGTCCAATAGTGCGCAAAGCGGTGGTCGAGGTGGCGGGACCACAGGTAACGGCGGTTGTCTCTAGACATTTCGAAGACCAACTCGAATGCTACACGACGTTAACGCCGAGAGTAAATGGCACACACCTGACGACATAATCTGATGTCCTCTTCGTTATCCTGGTGCAGAGCTAAAATCATGTCGTCGTCTTCCACCAACACTAAAAGCATGTTAAGTACCAGATTGGCATATGCTATAGCACGGGGTGAAGAGGTCGACGAGGCATGAGATAATAAATATGACGACAAGGAAATCAAGGCAAGAGGAAGGGGGGTGGTCCTGGTGGCCATTTTCCTTGCCGAATCTTCCTGGGTATGAAGAGTGCTCGCGAATACCTTGCGAAAAGTCGGACTCCCGTGCAAGAACTCGAACAAGGGTAGAAGGATGACACATGCTTCTATTGGTCTATGACGCAAAGTCAGTCCGTATTCCAGAAACATCTCATTGACGAATCCGCACTGGTTTTTGAATAATTTTCGGGGAGGGTCTACTGGTGTTGATGCAAATGCGCGGTCTGGACGCAATGAAGACAGAAATGAATTGAAAGTCGTAGCGAGGGTCGGCGAGGAGTCGTCAGAGATGTCTTGGTATGCCCTGGCACTAATGATCAGACAAGCCGCCGATAACGAAGTCCGACCGATGCACAGACGACATACTTGACGACGGCTAAAGCTGCAAAGTTTGCTGCCCAGCATACCGTACGCATGAAGGTCATATCCTCTGTGTCCCTGATGCGCCTGAGATACGGGTTGAGCTTGCCCGCGTCAGACTTGTGAAAGTTGGCTAGGAGCGCGAGAAGCAGGACGGCTTCAAAGGTAAAGTGTTCTGTGTCCGGAGATGT

>AT870

CGTTGTCGGACCACCCGGAGACTAGGACTAAAGGAGGACCGATCAGCCCACGATTATATTGTGCGATAGCCCTTGCTTACCCATTGCCTGCACGGCTTCCTGACGAGTGACGGGTGAAAAGAGAGTAGCACAAGAGATCGCCTGGACTTCTTCCAAGCACTTCATGAACATGTCACTAGGATTGCCTGTAAACGCAGCGCGATCAGGGGTTCCTGTTGAATGCAACGTGCACGGCGTGAAACATACCACCTCCATCGCGAACCTTGGCCGCGACCATGCAAATTACGTCTACTGCAAACGGCGACCGCTCATGAAGACCATCGTAGGTGTCCATGCTCGCGTCGAAAACGGGCAAAAACGTAGAGCAGCCATGATAGAAT

>AT887

CTCTCTCGTTTCTTTGTTGGATACCACACCACACATATACGACTACTGCAACCTCCTTTACCTATCCACTCCTATTGCGGTTCTCCGCGCGCTCTAGCCATGCGAGATCTGTCTCGTCGGCAACGGACTGGATGGGCCCGTTGCGCGTGCTGGCGTTCGGCGACATTGTGATTTTGTACTGTCTCGGCGGTGACGTGTAGGTAAGGATAGGTATCTGGGTCAACGTTTCCTGGAAGCGACGTCGCAAGAACCTGCGGGGAAGATGTGACCAAATTGTCGGTAGGACTTGTCTCGTATCATCCCCGTCGGCTTGTGCGGAGCTGGCCTTGAGGCGGGCTACTCCTGGCGGTGAAGATAAAGCTTCGAGCTCTTCGAAAACTCCGGCGCCCTGAAGCCCTGTACCGCGAGCACCCGCACGTATCAGGGCTTCCTGGTCGGTCTGAACCGTAACCTTGTCGCCTTCCGCACGATGCTGAAACGTTATCTGCAAGGGAAACAGCGTCTTATCCAACTGATGCTTGAAGAACTTCCATCGAAGGTTGACTTCTCTGCGTTTACTGAAAGGGCCAAGCGTTCGCGATTCGGACGATTGAGAGTTGAACCGCTCCTTGGGAATGGTTGGTGGTAGGAGGATTGCATCCGGCTTGAGAGGCTTCTTGCGAGAGACCTCCGAGGAGACGAGGGCCTTCAACTCTGAGGACCAGATCGGAGGGCGGGAAGACTCCACGGTGGG

>AT890

GTTAAAAGGTTAACCTATAGTCCACATAAGCACGCCAGCAGCAACATTCGCAAGTGCACGTACCAATGAGATGTACTGAACGTCCAGGATATTTCTCTGCAATCTTTTCGCACAGGACCTTCGCACGATCAATGGGCGAACTAGTAGCCGGCACGCGCGTCGTAAGAACCTGATTGTGCGCCAACGGGTGTCATTAGTCTGCTGCGCTTGGGCGGGATTACGAGAATGGAAGCGCGAAACAGACATACCTCGGTCCCATTCATTTCCAAAGCGTCCCTAATCCCTCTCCAATGTTGGATCTGCAAGGGAGCAATCGCCGGCCCGAGGGTGACCGTGTCGAAGCCCATGAGTCCATGACAGAACACGAT

>AT896

GGCGTGTTGTGTTTGATGGACTACGGCTGAGGCCTGAAGGCCTGAAGGCTCGAGTAAGTCCCGGCCGCAGTTACACATCTTCGCTGGGCTACTATACGACCTAGCTGCGCGCACATGGGTCGAGCTAACGTAGTGTTACAACACAACAAGGACAGCAATACTTGATGTAACATTACTCCACTGGCACATCTCCGGCGATTTCAAAGCCTAACGCTCAGAAGTCGCACTCAACGAAGTTGTTGCCGCTCGCGCCGTGGTGGGTCAAGCACGCTTTAAGCGGCTCGTCAGCAAGAGAGGATACACTGTTTAAAACGAGGAGTGCACTCACCGCAGAAATCTCCGCTATCGTCGTAGATGACGCGGTCGGCACCGGGGCTGCCGCCGCTATAGGCATCGCCCTGCTCCAGCGGGAACTCGAAGAACTCGCCTGAGCATGAGGGGAAGGAGAAACCCTGAAGACGCCATCAGTTGTTTTATGTTATTTGTCGGAGGGAGAGGTGCCAGTGGTCAACGCTTGCCTCGTAGTCGTGGTACT

>AT917

GACCGCGCCCTCTATCCAGCTGTTCGCATAGTATTGTCTTAGAAACCAAAGGAAATGTAAATATTTCGTTGGTCATTGGTTGCCTGCGGTTCTGTCAGAAGCGAAGCAGCGACATACGTGCCGACATTCGCCAGTACAGTGTCCCTAAACGTGTTGCGACGAATATTTCGTTTCTGATTGAAATTCGAGTGTGCAAGAGACCGCTTACTGGTCGCCTGTGATCTTGACACCATAAGCGCCAAATTGG

>AT924

GGGCGGTCTGGACAGAGGGCAGATGGGTCAAGCCTATCAAGGGCAAACACCCAGGTGTGACGTACCAACGATACCATGTCGCAGAAGTCCGAGCACGATAGCTATACCGACGCATGCCCATGACGAGTAGTGCGGCCATAGAGGGTTGAGGGAGAACAGGGAGCGCTTGGGGGGCATGATGACGTCGTATCTCTGGTGTCCGATGCAATTGAAAATAGCGCTTCAACAGCGTGATGGCATCTGGAAAGGCTTGTGGGGCTTGTGAAGGCGGGATGGTGAAATGAGGCAAAAGCTTGTTCTCCAGCAAGTGAGAGAGAGGTGTAGAAAGCGCGCGGGCGCG

>AT925

TGCGCGCACCCTGGCAGAAGCATCCAATGACGAGAAAAATGTATTGTGACGAGAGTCCTTCTTTGATTTGGGCTTAACATTACTGTCTAAGTCGACCATGTCCGAAAAGGAGCGTGAGGGAGAAGAGTCTGAGAAGAGTCTGACTATCCGAAAAAAAATACAAGGTATATGGTATATGGCAACTTGCGAGTCGATGACAAGATGGTGGGAGCGCAACAAGATGGAGCGTTATGAGGTCAGATAGACCCCGACGAAAATCCGGTCCTACGAATATATCAGTCAGACGAGCAGATTGGAGACGCGGTGCTGTTTTGGACTTACCCCTTGCAGTGATTATTGGCGGAAGAAGGTATGCACGACAAGTATCGACACCATGAGCATGCAGCGTAAGGGTCGGATGTGTAGAAGTTGCGTATGAGGACGACGAAGAGAACAATCGCCAACGGGACTGAGATGAGTCGCAGTGCGATCACAATGGTCCGATGACGGGTGGACGGGCTGATGATGGGGTAGAGAGCCATGCCGACGAGGAGACCCATGAGCAGGCCGCCGAGGTGTGCGAAGTTGTCCACATCTAACGCGAAGCACTCAGCAGCGTTCCTCAAGACGTAGATGCAGAAATCAGCTTACAAGGGATGAACCCAAGAGCAACACCGATAATCAACTCGATGACCATGAATACCAGCCTCTTGCCTGGCTGATATGTGTACCTCCAGTGCGCGAAGAGGTCGACCCAGGCAACCTGAAACG

>AT945

CGAGAGCAAGCCGACCCCAAGCCCCGAAATGACACGGCCAATAACCATGACCCAGAAACCTGAGGTGAAGGTCTGGATGGCGCCGCCAACGGCGAAAACGACAGCCCCGCTGAAGAGCGTGCCTCTTCTGCCAATGATATCACCGACCTGCCCGGCGGCTATGGACGTGACTGCCACATAGAAGTCATTCGCAATATAGCAATCAGAACTCCACTCCGTTGAGCTTCGAATCCGAATGGGAACTCACTGAAGGCTCCGATCTCGAGAACGGCGACCATGGTCCCGACCTCGACAGGCCCAGGTTCGTTGAAAAACTTCCGGAAGTGCGGGCCAGTGATCACACCGGACATAACACT

>AT948

TCACTTGCTGATACTCTCTTGCCTCGTGGGTCTCCTCCAATACTCGCTCGACGTTCTCGATGTTCAGCTCCTTGTGGATCTCCTTCAGAGCCGCATTGCCTTGTTGTAGGCCGTGCAGGACTGATACCTCAACAAGCGAGAACTCGATTGTCGAAACCTACTCCGAGACCGTGATGAGTGGCGACGTAGGCATGGAAACTTGGAGCGTACCAATTGCTCCAGGTTTTCTAACTGAGCATCCGTCTTGACCAGCAGCCCTTCCTGATATTTCCGTCTCCGCAGGGCGACGAGGGCACGGTCCTTGTTCCCCGCCGCTAGCTGCTGCTTCGCGATCTCATGCTCGCGGTCCAGCACCACTTGTATCTTCCGCAGCCAGATCAGGCTCCGAGTCGTACAGCAGCGGCACGAAGGCTGACCTTCTTCTGGTACTTTTTCACTTCGTCGCGCTGTAGTTTCAGGCTGTGAACGATTACATCTTGGTGCGCACACGAGGGCACGCGAGGGGGTACTGACTCTAGGATGGCACGGTCTTGTTTCGTGATCTTCGGTACGGACTGGTTACCGCCCATGGAGATTGTGTTCGAGATCGGGGCAGAATATCAGCGAAGAAGGGTAGGTGTGAGAGACGGAAGAAACGGCGAGGAGTTTGTGGGTGTGGTGAGCGTGGAGGCTAGAAAC

>AT96

GGCGTACAAAACACTCACCAGCTCCTCGCCGTGGAACGCGATGGCGCACGCAATCTGGCGCTCGCCATCCTCGTTCGCAATACAGTCCGCAGTTCCTTCCTGTGACAGTCCTAAGCATTGACTCCGAGGGTGATGGCCATGCGACGAACCTTGGTGAGGACGGCGATGGAGGCGTACGAGTTGCCGAAGTTGATTCCTACCACAGTGGCAATTGACGAGACTTCGATAGGTGCTTCTGCTGTTCCATTTGGCTGGGCCATTTTTGTGGGTGTAAGACGAGCTTTAGAAGGAAAGATGGGTATAAGCGGAGACGGAGGAGGAGGAAGAGTAGGTCTTTGATC

>AT960

CGTGTCCACTGTCGAGCGCGCGGTGACACCGAAACCCTGGTGGCGGATAAGCATTAGCGCAAGGATGAGTGGGGCATCGCTCACTAATGGAGTGTCATTCATGCTGTACACAACCACTCCCTGGTGTTCAGGTGTGTCCTCCGTTATCCGGCCGAGGTGTGCTTTGAGTACATGGTTGCCGTACAGGAAGGGCATCTCTCCATTTGGCTTGATCCAGACCTGAGTCGCCTGTTGAGTTGGGAGTAGATGTAGAGAAGTAGATGGTCTCAGCGCACCTTGTATTTGGCATACTGTGCAAGATAGTCCAACGCGGTGATATGGAGCTTGAATTTTCCGCTCTTGGTGAATTTGCCGAAGCACGTCCCAAGGCTCACGAGATTCGGTCGTGCAACAGAGATGGCCAAACGCATGGAGGACTCGGAGACATAGAAGACGCGGTCTTTCTGGAGACGGAAACAATGGGGTTCGTCTTGTCGGTCAATAAGGTGGACCAGGTTTTTACCCTGTCATAAATGATTGGTGGGTTGTGAGGAGAACAAGAGGAAGCAACTACCCACAATGTAGTTGGCCAGCTTGGTAAAAACCGTCTTGCTTTCTTCTTCTGTGAGTGGACGCATAGCCTGAGTATGTTCGTGGTGTACAGGGAAAGGCAAGGAAGTAAAGGTAAATTAAAAGAGGAACACAGGCCGGCGTTCGAGCGCAATTAAACCATTGGGCGCTTA

>AT977

CTCTTTGTCATGTACCATACAGCATTGGCCGTGTCACGGTAGACACTCGAACACGTAGTCGCTTTATCCTTAAACCGGTTCTAGGATGCAATAGCGGGCGTATATCAGCGGGAACTCAAGAAGCTGGTTTAACCGATCCTACGCTAGTACATGAGCTAGTACAATTACCGGTGGCGGGGTATAGCGCTACTCCGACAAGGACACTTCTTCCTCTAACTGGTCACCGTCCGTTCCTGATTGTTGATTAGTTCGATTGCTGGAAATGAGGTCATGGTCCTCTGCATGGCGAGAGTCCTCGAGACCCAAAGCTAGGAAACTAGAAACGACGATCACCGCCCCAAACACTACCTGAGCCTTCGCAGGCCGCCCCAAAAAGAAGTCTCCCAAGACGGCCAGGGGCATCGTAAGACTAAGCCCGACGGTGACGACTAAAGGCGTGGTCTTGAGCATCGCAATGACGTAGATGTAGTCCGACGAGAGTGTAATTGTCATCTGCGATGTAAAATGAAGACAAATATGAGCACCGACATAGCTGCGCCGCCAGGCGGCTGATACGCACATTGATCAACAAGGCCGTCACCACTTTGCTTGAATAAGGCAATTGAAACGGCTCCACGCCAGTGAGATGCAAGACGAGGCCGATCGGCCAACAGGTGAGGATGTTGAATAAGCCGACGAATCCGAAGAACAACTGCATGTCGATACGCGACTCGGACCGAATGCGAACTTTGAGCAACGTCACATAAAGGGCGTAAAAGAGGGCCGAGAAGAGCGCAAGGGCATCGCCAACTATTGGCATGGATGGCAGGGATGACATACCCGCGGACGGGGTCGCAGGTCCAAGCGACTGCGGCTGCGAAGAGTCCGAGAGAGAGACCAGGACGACACCAAAAAAGCTAAGGAGACGTGCGTCAGCCTCGCTATAAGCACATGTACGACGTAACAGTCAGAGCGCTGGACAAGGGGGATGCCCAAGTCATCGCCACATACCTGGTGATAACAGCGGCAATCTTGATTAATGTCAGGCTCTCTACTCTGAAGATCCGGCCAATGCCCAAAGTGAAGAAGCCTGAACGGAGACATATACGACCGGAACCGTTGTTGCTGTGTAAGTACCAATTTGCATCCTCGGAACGAGTCAGGTGAATCGCTCACCGCTCATACTGGACAGGATTGTGGCACTAGCGACACTAGTGTAGTCGAGTGCCGCATTCACCGTCCAGTTCGCAATGAACCATAACAGACAGAAGACGGCTGCGAGATGGGCGGTCTCCCGGATGGTCAACGGTTGTGAATATTTGTCTTCACCAGCGCCGCCCTC

>AT98

AAGGAACCGCGGCCTTGATCTCTCTGACGGCGCCCTTGATCTTCTTCCGAAAATCGTCGTTTGCGGGGTGCGTCAGCACTCCCCTCATCCGATACGCCCCCAGCTCGACGACGAGAAGGTCCTCGTCTTTGAATATCATGTCACGCTGCATAACCATGCTGTCTATGGTCATAGACAGCACCGTGCCGAGGAGGATGTACAGCTCCGACAGCCGCAGGGAGGTTAGCCGTGGGCGAGACCCCGGCGGTATGTGGCGTCGATAGTGGCTATGTGTGGCGTACGCCGCGGCGCAGACGGGCAGGGGAAGGAAGGTGAACCACTATTGTCCACGATGGTAAGTAGGTAATCGACTTGCGATTGAGGACGTATGTAGGACTGGGCTTACTGAAATGCGCCGCGCCTTGGGGTATTGAGATGGGCTCAGCTCGATCGGCGGGTCCGGCTCGGACTCGGATGCTGCCAACGGTGGATGCCTGGTCGAAGTCACGTCCTCATACGAGTTGGTGGACCTGTTCGAACATGTTGGGGGGACTTGCTTTGCACGAGACGTGAAATACTCTGCGATGCCCATGCTCAAGAGTCAAGAGGTTATGGGTCAAGCAAACCGTAAACCAGCGCC

>AT982

CGCCGCCAAAGCACGAGCCCAGTGGCGCGCAGACCGTTCAACGTCGAGGAGAAATTGCAGATAGGAGATGGAGACCCATTCCTCGACGACGGTCCTATCCGTAGCGGATAAGCGGGGAAGCCTGAAAGCCGGGGAAGATGGGTAGAGAGCGGCAGAGTGCTCGAGGACGGTGAGGTGAGTCCTGTAACTCATTGTAGGTGAGCTGTACGTCTGTGGGGAGGATGTTGTGTAGAATCCACTCATGGGTGGATGTACCTTTATACAGATAGGTCGATGGGACGCACCAGGGGAGAGAAGGAGCTATGCATGGCGGTAAAGCTGTCATACGAAACAGTCAAAACGTCAAGCCGTTCAATATGGGAGTCCAGCTGGTAAATTGGACAGGCCGCCAGTCGGAACGTC

>AT11063

GGCGTACTGGAAAACGGATGCTTGGTTGAAGAAGGCAATGGAACCACGGCCCTTGACACCGTTGATGGGTTTCGAGTTCGCGGGAGTGGGGGTTTCACCGTTGGGGGTCGTGACAATGTGGTCTGTGAAGTCTGTGGGCGTGTTGAATGAGCGAATGGCGAATGGCAAGTGCCAAGCAATACTGGCAGAGACTCACTGGAAATGTTGAAGTTGAAGTGGATGAACAATGCAGATGGATAGAGGAAGAGGACGCCAACCGAGAGCTGGATAGTTGAAGAGCCGTCTACACACTCCTTTGCAAGAGGGGAGGTGTATTTGGCCGCGCCCACTTGCAACAGGGCGCCGGCAAGATCCGCAAAGAGTTGGCGAGGAGGCAGGAGAGCGCAAAAGCGAGGGCAAGCGAGGAGGCAACATGAAGGCACTTGCAC

>AT11064

TGCCGGGGCCCCCTTGCAAGTGGGCGCGACCAAATCCCCTCTTGCAAGGTGCGTGGATACTACAATTGAAGACACCTGTTGGATGTCAGAGTCGATGAGTAAGCACGAGCACGGAGTGGGGGCTCACCGTAGATGAAGAGTGCACACATCATGATGGCAACTGGCACCTTGCACGGAATCGTGCGGACGGGCTCCGTTGATGCAATAGTTGTACCATAGGCCGAATTTGGGTTCCGCAATGCCATGACTGCAGGCCATCGCGAGTAGGCGTTCGATGCACCGGCCGACGCGAGCCGCCAACATGGGAAAGCGTGAGGGCAGAGTGAGCGCTGCGAAATCGCTGCAGGGATGGGGTTCGAAGTGAGAAACGTGAAGAAGCACGGGCGCCATTTACCACGAGAAACTCTCATGAGTCGACACGTGATAACCGCCTTCTCCAGCAACCAAGTGTTCGCTATTT

>AT11083

GGTGGGGATCGTGGTGGACGCGAGGGCCGTGGGGGACATCGATAGGGTCTGAGCCTAGAGCATTGCGGAAGAGCAGGCGTCAGCAAATGGGCGTATTCGGGTTCGAGTCGGCCGTCACGAATCGCGCGGGCTCAATCGAGGGAAGCCGCACCGGAATAAAGAAGGAAGAGGAGAGGCGCGGCGATCGCCTAGAGACGCGAAGCGGATATGCGGTGCCCGCAATCGGACGAAGGCGTACAGGAACACAGGACGCGAACATGCACCTTGAGCTCTGGACACAGACGC

>AT11118

CTTGGTGAACCAGATGTCCTGGATGTACGTGTTGGAGGGGTACTCTGCGCAGTCGCTGGCGCTCGTCTCGTAGCACTGGTCGATGACGACGGGGCTGTCGACCGCGCTCTCGACGAAGCCGTCGAAGGTGATGTTCTTGACGAGCCCCGAGCCGACGTTCTTGCCCGCCCACGCCTTGATGCGCGCGCCGTTCTCCGCGTTCGACATGCGG

>AT11126

GGCCCGTAATGCTGTGCGTCCATGAGCGATCGCCGATCGGAGGGTCGCCCGAGAAGCGGCTGGGACTCGCCGTGAAGAACCAAGGCGAGACGGCGGTCATGAACGTCCGCCCGCCGAGGTGGCGGATGTGGTGCCGGTCGGTGTCGAGCTTCGCGCACTCGACGTCCTCGCGCGGCGAATCGGCGGTCAGGTGTATGGGCCAGCC

>AT11127

GGCGGGTATGTGCGGGTACCGCCTGGGGTCGATGAAGAACGACGGGACGAAATGTATCTAGGTAAGGAGCAGCAGGATCGTCTTCGCCGCGGCCACGAACGGGAGATGAGAACGCACCGGTGCCACTTCTTGCAACGAGTCCTTGATGAACGCGCACGCGTCCCGTAGGTCCGAGAATCCGAATAGACGGTCCTGTCCGGCGAACGTCGAGACGAGGACTTTGCCCTCGTACAAGAGTTGATGTGGATGTCTGGCGGTCGCGACGAGGTAGTCGCGCAGGCGCCCTATGTCTTCTGGCGAGTCTCCTGGAAGTACACTGGAAACAGCGTCAAGGCCCGACATCGACTCGTACATGACCCAACATCGCTCGCGCTATATTTGTGTTGGAAACCATAGCACAATCGACCTACGTCATATCGAAGCTGAGGAACAGCTTGAACGGCAACCTCAAATCCAGAGCGGCGGCGTAGCACAGCGACACGGACCGTCTCTGCCAGTCTTCCAAGCCG

>AT11170

GAGGGCTCACCGTAAGAATGACCACCCGTGCATGCAGGTCGCGTTCGCGGGTGGTGGAGCGACCTTGAGCGTATGCGTAGATGACGACGAGGAGGGCAACGGCAACAGCGGCATGGGGCCAGTACTTGAAGGGGAGGACTGTGGTTGCGGCCGTATACAATACTTGAAGGACCATTGGAGCTAGCTCCCGCCCACGACAGACAGACCGAAACAGAGGAGTAAAGGTAGAGCACAAGTTGACCAACCCTCAA

>AT11177

AGGCCAATAATCACGTCGCCGTAACCAAAATGTAGTACAGATGGTCGTATGTACTGTAACACGTATGCGACGAAAACGAACAGACAACTGAGCCCGGACTCAATCTAAAGTATCTCATCCTAGGGTGCCCTCCGAATGGGCCCGCGGCTTCTTGGCCATCATCTCAGATGAGAAGCATCGCATGGCAGTCATCCCGGGGGTGTGTTCATGGTGAACGTCGATAGTGTGGCGAGGCCTGCCACGCAACACAGCGGATCATGCATCAACACCCGCAAAACAACAGCGACTGTATGATAACTCACCAAGAGAAAGACTAATCCCATCAACCAAGTGTGCGCCATCCTAAGCGGCCCGCCAAACTGAAAACAGGGCGCGTGAGGTAGACCCGCAGGAACTTCGAAGTCAGGGCCGTCAGCAAGAACGTAGAGGGGACCTTGGGCTTCTCGACGGTCAACTGCATGAGCGGTTCCGGAAGGCATGAAAGCGTTTCGTCGGCCCAGACGCGTGCTGCACCGACGTTCGCGAGAGGCGCATATATCCAGGAACCGAACTCGGGGGCGGGGGGGGTGTGTGGAAACCTCGATCAGCGGTCGCGGCGACGAGCAGACATTCGAACCTGCGTAGATGCTGTGTAAGCTATACACGGGTGACAGGTGCTGGACATTAGTGGCCGTACGGAGACTTTGCTGCGCGCGAAATCGAAAGTTTATGGGCTCCGCATCGTCCAGCGGTATATGCGCGAGGCGCATATATCGAACCATCGGGACTCCGATGTGCATCCGGCTGGCCCTGAGTACTCGTTTCGGTCTGATCACAGCTTCCTTCACTGGCCCTTGAGATCGTGCTCGCGCATCCTGCTAGTGAACTGGAGACCGATGAAAACCTGGCC

>AT11181

AGGAATCGAATATAAAGACGCTTCAACCGCGCCCAGGCAGGGAAGCAGCTCTTGACGCAGTCCGACCACCATTGTCTATGCACCTCCCAGTCATGGATGCAAATGACGAGGAGATGTTTAAGACACTTCTCCTTGGTGCCGCCTGCAGAAGAAGCGCCTCGAATCCATGCGTCGGTAATGGGACCGATAGTGCGTAGAACGTTCGCAAATTCCCGACGGGTAGACCGGGGATAGTCATATGCGGAGAATTCGAGACGTGAGCGGTGTGGGGGCCTCCATGAGTTAAGTATATGCTTGAAAGCCCTCACAGCCGACTGTGTGTTCGCCGAGAATCCTGGTGTGAGCTGCAAGACCAGATGGCGCAGGTGTGTGCATGAACTCAAGTCGAGCGCCCC

>AT11200

GGTACACAAGCTTGTCGATACGACGCGCGTCCCATCCAGGACCGTACGGGATATGCAGCGACTGCACGTCGTAATCGTTCATCTCGATGCCCCCGATGTCGATGTTCGCCTTCAAGTCGCCCCTGAGCTTCTTCTTGCGGCGGCTGTAGTCGATATCCGCGTCCGGTCGCGTGCGCAGCTTGCGCCGGTCGATCAGGTAGCGCTGCCTGGTGTCCGCGTCCACGAGCCGCTCCAGCACGAGAAGGCGTGCGAGGCCCT

>AT11204

ACCGAGGATATATGGGCATACTCTCGAATATCTTCTCGCCCGTACCCCGTACGATCACGAAATTGCCGATGTGATACTGGTTGGCGAGCTGCTCGAGGCCCGGGATGAGCTTGTGGATGAACGGGATGTTCCAAAGCTGGTGCATTGGCGCGTGTATGGACTTGCGGACATCATCATGTTCCTCGGACTTGTCTAACAGCTTCTGGAGGGCGACGACGACATCGTTGGGATCCTTGGCGTCTGGAAGGCTGTCGGGGTGAGTTTTCTCGACCGGCCGTGTCAGGGCGACATCCTCGGGGATGTGAGACATGGTTGGGGAGCAGTGAGAGGGGGTGGTGGTTGTAGGGGAAGAAGAGGTGCGCTGTAATGGATGAGAGCGAGCATGGCGGACCGAATTAGCAGCAGGGACAGTCGGCGGGAGCTGAACTGGAGTCTGAGAC

>AT11207

GGTGGGGTTCAAGCTTCAAGCTTCAAGGTCGAACGACTTGGCAGTAATTCTGAGCGGACCTAGTGATAACTTCGTACCCGCCGCATGAAGCCAAAAGAAAAATTAGCGCATATGCGCAGTGATAGAAGGCGTATAAAATAAATACGTCGAAGAAACGTATGTAAATGCCAAGGAATGGATACGAATATGAACGTGGATGCGGATTGCCAAACGCTTTTGCTCGTGCACAGATGTGCGGTGTGGAAGAGAAGCGATATGAAGGATGGAGGAAGTGGTATAGATACAAAAGCAGCGCATTCGCTCGAGTGCGAATGTGAATGTGCGATAAATACGCATGGGCGAAACCTGAAAGCATGGGCGATCCGATAGCGTGGTGTGTGTGTGTGTGCTTAGCATGGTGCAGAACGTAACGTGAGGGACATAAGCGAGACCGTACGGGTGCATGACGGTGACGGGACGAGAACGAGAGAGCAGCTCTGCCTATGCGGCGTGGCTGTTGCTCGTGCCGCCGCCGTACACGCTCTTGTCATAGTGGTACGCTGGTGGGGGGTTGCCCTCAAGGTCGTGCTCGACGTACCCGCGGGTGGTGAGCTCCCCAGGGCGGAGCTTCTTCTCCTTGGGCCGTGAGTACATCGTTGACAGCGTGCCGTTGCGCTCGAGATCGGCCGTCGAGCTGCTCGCTTGGTTCGCTTGGTTCATGGCTTCGTGGAAAGACCATCCGCCGTCGGAGCGGCGGTATGCGACCCGCATGTTCTCGCCAGGGGTGTGAACTGTTCGTTAGATGATT

>AT11208

ACCGAACTTTGGCACCTCTACATCAGGAGCGTTCACCCCGACCCCGAATGGAGTAACCCTCGCCGCGAGATGCGACGGGTCGACCGAGACCATGTATGTCCGCCTGTGTGACGAGATCTCCTCCGCCCGCTCCTGCGCCTTCCTGCTCCGCAGATGTCTCCGGATGAAGTAGAATGCGAGGCCGCCCAGGACGAGTGACCCTACGACCGCGAGCGAAATGCCGAGTGCGAGTGTCGTCGATGTATGTGGCTGGTCTGGGGCCGCGCTTGGGAGGGAGCTCGCCGAGGAAGAGGGAGAAGGGGAATGTGGATGGTCGCTTGAGGAGGACATGGTGGGTGGGGAACGAAAGTCGCCAAAGCTGGCGGAGCCGGCGAAGGGCACTGTCACTGTAAAGTGCGATCAAACTTGTTAGGACCCGTGTCGTGTGATGTCACACGCCCCCGTGGGCCGTGATGCTGGAAGCAACCCTCACCAAAAGCAAGAAAAACTGCGAATGAAAAACTTGAAAGAAGGTGAAAGGTGTGCAGAAGGCCTGGCTAGTAAAACGAGAGTGTAGGATAGTGCTCGGACAGGGGAGAGGGCGCGAAGCGAAGAGGGCAATCTGGGGTCGATATCTATATATTCCCGCCCGAAAACTGACGTTGATGCACTGCGCTTCACGACCACCCAGCGCCGCCAGCGGTCTCGGTTTTTGCTCTGCGACATCTGCACTAGTACTGTGCAGTGAGCACCATCTGACCGACATTCGTGATCTTCGGCTTGTTCGCTTGTCTTGATGAAAAAAGGCACCGCCACCCAGACAGTCCATTTATATGCCGCGTGCCTCCAAGTAAGTACTAAGTACTTAAGTTGTGGCTAATTCGCGCACGATTGCACAATGGACACCGCAGATGAATCGATGACCATCGGCCCCATTATCTCCGACGCTTGCAAGTCCTGGGCATCAAGCTCAAGAGTCGATGAAGCACAGTCAGAGAGTCGTGCATGTTGCGTGCCTGTTG

>AT11221

GGAGGATCTGGTCCTTGATCTCGCGCTGGCGCATGCCCTTCATCGCCTGCGCGAAACGACGGCCGCATCGTCCTTCAGTGCCTCGGCGAGCTCCTCTTCCGGCGTGTGCTGCGCAGTGTGGTAGTAGGGTTCGTCGAGGGATGGATATTCCAATGGGTTGCCCTGCGGCGAGCGCTCGCGGTAGACACGCTCTATGTCATCTATGGCTGCGCCGATGACGGCGTGCAGGGCGCGGAGGGTGGAGAAGGTCATGGCGGTCGTCGAGATTCCTTCGTGAGGAGAGGAATGCTCATCGGGGTGGGTTTATAAGCTCGGACGCATTGGACA

>AT11223

TCCTGTCACGAATTTCGAGACATAATTCGTGGAATTCGAGCATATCCATATCGTCGCCGCCTTTCCGCCGGGATTCGACGGTCTCGCCGGAAATGATTGCCATGAAGGTCCGAATGAGCTTCGATAGCTGCCGAAGTCGGTCCCCGTACTCGTGCCGGAGACGTTTCTCCTGCTCGGTCGGCTCCAGGGCGTGCACACCGCCGGTAAAAGGCTCATGGGCTGTGACGAAGACGTCCCAATACTCAAAGTCTTTGCCAATGATGCGCTGACGCATCGCAGGAAAGCAGAGGGCATAGGTGTCTAGGATGAAACGCTTCTCCCGCTGAGAGAATGCTTCACCCCGCCGTACACCAGAGGTAGTCTTGGAATCCTTGCGCAGGGCCATGAACTGGGTCAGCCACTTATCGACGTTTGACCCGAATTCGGGAGGGGTAGATGGCGATAGACAAATGTAGAGCGATGTGTCCCCTGAGGGACGAGTTTGAGCCGTGGCAGCCGCAGCGTTACATCCGGGATGGAAACTAAATAACGTCGTGAATGCCTGGACAAGGTTCCGAAGATCGGCGGTGCTGTCGCAAGGTTCGATAGAGCGAGGCGGATAGAGTATGTAGTCGAACAGATGTTTGAAGATGTGGGGGTGCAATGCGGCAATGAGAGTGTCGATGTCGGAGGAAAGCACTGGCATGAGGCTCAGGGAGAGTTTTGTATGCAAGTCAGAAATTGGAATTGAGGCCTGACGACGGACGTTCTGAGT

>AT11230

CCATCGCGCTCTGGCTTCGTTCTAGCTTGTATTTTTTTTTCGTGTTCGACGCCTCATTTGTCCTGCGACGTAGTATGCTATGTATGCTGCGCCTCTAGCTAGTACGCACCGCGCTCTCGTGGGCGGGCACCTATCCCCCTCTATCCTGCCAGTGGTATGTGATCGTAGGCACCGTGTAATTTGCTCTACCGTACCGATTCAACACGGATACCAAACACGTGTCGGACACTTTCTCGTTCTATCAACAACCCAAGTCTTGCAATCGTATGTTGCCTATCCGTAGGGTGGAGGATCACGGGAAGGTGGCCGGAGACCGCGCTGGTGCTCCGTTTGCGCCAAAGTGTCTGAGACGCCGAGTCCTCGTAATAGGCTGCCGTCCGCCGGGTACGTCGTGACATTCGAAGGCGAAATGCATCGAGGTGAGCGACTCTCGATTGGTGCTGCGTACAAGACATAGATGGGACGTACACGTCGAATAGGACTTCGCCCACTCCTTCCGCGTTCGCGAGGCACGTCACGGGGACGTGCACCGAGTCGTCCACCGCACCCGCGTGCTTTCTTTGTCGTATACTTGAACGAGGATGTGGACGACGTCCGGGGGTTTGCGCTGCTTCGCTTCGACTCGGAGATGCTTCTGAAGACTGACCCGTTGAAGGCGGGCTCGATGGCAGCACTAGTCTCCGCGAGAAGGTCAAAGCCATAATCTGCGGCCAGGAAGGGCGGCAAGTGGCACAGTATGTACAGCACGAAGACCAGAAAAAAAAACGCCGTCCTCGCTGCGGTCAGCGTGCACCGGAGGCGATCTTTGAGCGAGATGCTGTCAAGACGAAGATGTCCTCGTCAGGGAATGAAGAGGATCAGAGTAGGGCATGAAGGACGACGTACTTGGTGCCACTTTTGCTGCAGGCGGGGACTGGCACATTCAACAGAGAGCCGTTAGTTGTTGGACAATATCCAAGTCCAACGCAACACTCACCCATCTGGCGACCGCACCTCAGACCATCCACAGCTTGATCCTGTCGGCAGGGGGTTCTTCTCAACATCATCGGTCACCATGAGTAAGAACATTATATACGAAGCTACGCGGAGGAGGTGAACACGACCGGCAGGAGTTCGTCGTCCGGACAGACAGCCGTCCATGCAGCTCCTCAACAGACGGGCGCTTGGTAAACCCTATTCTCGGTCTACAGGGTTGAACCAGCTCGGTGCCTTTCGTACGACGACGACAACTCAACTCGCCACCGTCACTAGGATAAAGTGCAGCCAACAGGCCTGTCGTCGATATTTTGAGTAACTGCCAATTCAAAATTCAATCTGCTGTCCGAGGAATAGGATTTACTTAGTGCGTACACTCACCGCATTCCCTCGCGCCCTCACATCATCGGCCGAGTTTCACACGTCTTCAGGACATCGACGTCGGAGTGAACTGGCAGCCCGGGAACTACACGAAAAGCCTGTCAAACAATGATTTTGTCAGGAAGGCGCGCATATATCGGCATGCCTGAACTCACACACCTTGGAGGTTCTCCCACCTGACCTGGTGCGTGCCCATCATCGCAGAGTGTCCGAACCCTACGAACCTGCCCTCGTTCTCGGGTACCTCGAGCCACGGGAAGTACCCGAGCTGGCGTGTTGAACGCGAGGCTGAACGGCGCGACGTAGCACGTCGGGGCGACGGCGCTCCCATTGAAATTAGCAGACTCTGACTCGGCGGCGGCGGACACCTCGGGCGCTGGCGCGCGGTAGTCGAATTCGGCGTCGGTGCGGCCGAGGGAGCGTTTGTCTGGCAGGGCCCATTCAGTCAGGCAGGAGACGAATCGTAGGCATTCGTCTCCGCTGTACGTCAAGAAACGTGGTCAGAATGAGGCATCAGTACGGTGAGCGCTGAC

>AT11255

GTGTGGACTGAGAGAGTGGCGGAGGACGGAGGTGATCAGCTCGCGTTGAGCTGTACGAGTAACATTGAGCGAATCTGGGCCCTCATTTGCACGAGCAAATCTTCGAAGAAGAGTGATCATTGTTTCGACGTAGTTTAGGAAGGCCGGGTCGTCGGAGGCGACGAGGAGAGTGGGGGCGGGTCCGTTCACGTCGAGACACACTGCGTCCCGGGAGACACCCTTGGTATGGATCCCGAGGATACGGACGACGGCGTCCTTCAGGGTGTCACGGATTTGGGGGAACCGCTCCCCTTGCGTATAGCTGGCCACCTCGGTGCTCTCGAATAGCACGAAGAGCAGCTCCGCGACACTGGGTGTGAAGTTGGGTTGCTGAATGGTGTCGGCC

>AT11264

AAGTAACCAGGGCCGGGCGTCAGCCGACGACAGGACACAGGGAATGCCGGGATCACTAGTACAAACAACATCGCACCGCGATGCTCGCGTTAATGGCTGCTCGACAGGCTCGAGGGAAGAAAATGGTCTGCGTACTTACACTTCTCCGCGATGTGCGGCCGCGCGGCGAGCTTGTCCTCCATGAGCTTCCGGAACACCTTCTGGAAGTCGCCCTGCATCGTGCTGTCGCGCTGCGTGTACGCGTGCATAGACTACATGCACGCGCGCTCCGTGC

>AT11112

TAGTGATGTCCTTTTCATCGCACAGACCGAGGCCGATGATGTAGAACATCCCAAGTTTGTTGTGCTCACGGTGTGAACAGTGAAGCGAAGGAGAGGGTTGGGGGCAAAGTGAGATCTTGATCAAGAACACGGACGGGAGATAGGATCTAAGATCTATGTTCTACTCAACGCCCGCAGTCAGTAAATAGCAATGTGATTAGCTAACTTGAAGTGACAGTCCAACGATCTTGATACAGTTCTGCGGACTGATGCAAGGTAACGTATCTCTGGACATTCTCAGCGAGTGCCTTGCGCTTTTCGATCCAGACAAGTCCTGGAACTTTACCTCTACTAGCCTAGGCAGATCAAGAACTAGTGACTACCCCTCCGAGCAAGAGTCGACTTGACAACGGTCGGAGGGGACGTGCCCTTGGGAAGAGTGGGTTGGGAAGTTCTACACGACGAGATGCCAATTATGAAAGGATGGCCCAAGACTAGTCGATTAGCGTCGAGAGCACTCGTACAGTAAAAATGACAACCATCCACCCAAAGAACAACAGCAGGTATTGAATAATCTAGACACAGATGCGCTCACCATCTACGGTCCAACTGCGCCATCCAGCCCAAGGGACACCATCTACCTGCCACCCTTCACCAGACCCTTGTCAGAGTCATCTTTGCTCGGGTCGCGCTCGAGATCCATAGGATCGCTGGCGCCATGAGTAGTCTTCGAGTTGAGTGGCGGATCGCCGCAACCGCAATCGTATGTCGAATCGAGGTTGGCGCAGTCGAACTCGGGCTTCTTGAGCCGCTCGTTGACTTGGTTATGCAGGAAGCACAGCCTGGGGCGGAGGCATAAGGAAAAGAAAATGGGACATGGAGCATATAGATAGCCTACCATGTGGCCGCTGCTCTCCTCGACGATGTCTACATCCAACCCACTGATTCAGAATATGGAGCACGCGTTTGAAAAGATTTGGAATGCACTCACTTGGGGTGGGTACGTCTTGAGTAGCTTCTGGAATTCGGCAGCGCACTCTCCGCAGGGGTATAGACGGGACATGAGGTGGAAGTAGTTGTTCAGAGCATCGCGTTCATCTTGTTTCGGTTCCTGGGAATGATAGAAGGGAAGTAAGGCTGGAGCG

>AT11520

ATACCTCCTCAAACGTTCCAAACCCCCCAGGAAGACCAATGAACGCGCAAGAGCGCTTGGCCATCTCCGCCTTCCGCTCGTGCATAGAATTCACAACAACCTGCGATAAGCCTCAGTACACCTTCCCTTCACCGACGAAGCAGGCACACACTCACAACTTCCGCCTGCCATGGTACCGCGCGTCACAAATTAGCGCGTCAGCCCTTTCCCTCCGGTTCCGAGCCTTCCATGGCACAGCGCGGAGCTAGCCCAACCTGAAACTCGAACGCACCTTTTCCCTCCCCTTCTCCTTCAGCCCCACGTATGTCGCACTGTTGCCCGCGACCTGGTCCCTCTCCCCGCCCGCGGCGACCATCGCATACGGCACGACGCCCGTCACCTCCCCACCCGCGTCTAGGGCCGCGCCAGAGACGGTGCCCATGAGCCCGTTCGACCCTCCCCCGTACACCAGGGGCCGCCCCGCCTCGGCAAGCGCCTTTCCTAA

>AT11523

TACCGAACCCTAGAGACAGGTCCCAGACGATGATACCAAGTCCAAGCAGATTCCAACGAGCAGCACAGTGAAAAGAGCCCGAGACACAGAAGAGAACGCCAAATAGCAGCATCGAGGACGAGTGGATGACACAATACTACGCTACTATTGCAAGGAACCTGTCATCTCGGCCTCGCAGCCGCCCTCAGCGGTGTCGTCCTCGTCCTGGTGCCGGATCTTCATGCTCTCGGCGGTGACGCGGTCCCGCCGCCGCTTTTCCATGATGAGGAACGTGAGCACGACGACGTCGATGTCGAGCGTCGTCGGCTCGATGGCCAGGCAGTACTTGCGCTGGCCCCGGAAGAACCCTTCCGTCACCATCTCCTGCACGGGCCGCGCGATCTCTCGACGGGTCTTGCAGTGGTAC

>AT11534

GGTGTGAGATCTCCTACAGGTGATCCGACGTGTCCATCTTCGCGAGTTTCTATCCGCTCCTGGAGCAGAGAAGAGGTTCTCCGCGTCCCCTCGTATCCAGAATCAGGGCTTTGTTGTCTTGAGGACATCCAGAACGAGCGAGTGAGGAGGTACACCAGCAGTAGGTAAGTGGCAATAGGTCCGCTGAGACGGGGGCGGGGCAATTTGGGGGTCAACAGCCAGCTTCCTCAGCGAGATAATATACGAGAACCCGTACGCGTATGTGAGAGGCGTTAGGTGGCGGACAATGGCTTCGGCTGAAGGATCGATGGGACTGCTCGAGAGTCCTTCAGCTCGTTATGGGAGGCATCGGGGTTGCAACCTCAACCTTTGTCTGGAGTCACTTTGTGTGTCATACCAAGCAGACCATGTAGAACAGTGTTTTGTGTGTCATGAAGGACCTTTGTTCAAGCTGAACACCCTGGTACCGTGCCGAAAGGCGTAATGTCGGAGTTCGAGGATCCATGGCATTAGGCTAGTATTGGCCGCCTCTCATTTGCTTCC

>AT11545

CCTGACCTCCGTAGGAAAGATGAAGAAGACTCACGTAGAGCTTGGCATAGGGTGTGCCCTGAGGATCAACGAGCGTACTTTCATTCTCGAGGATAATACCGGACTCTGTGAACATAGACACATCAAGGGGAACTCGCCACATCAGACGACGCGACCTACTATTCTCGCTCACGGCAACAATCCGCTTTTTGATCTTCCAGCCAGGGCCGCTTACTGAGGGAAGAGGCTTAAGGATCTCAACCGACTGACTGCCGTGCAACTACGTACAAGCAGATCCAATTAGAGTCGTTTCTGGGATGGGGGAAGCAAGAAGGCTTACACCGCGGTTGGGGTCAAACTTGGGGAGACCGGGGGCACGCTTACCGCCCGTCATAAGTTGACGGAAGTTCACGA

>AT11551

GGGCCGGCACGACTGGCCTCCTTGGCCTGGGTTCGGATAAGTACACTAACTTGAGGTACGATACATAAGCTCGAGACGTGGTAGCAACTACACGGTCCAGTCCAGATTGTACGAAGAAAACAGACTCTAAGACTCTAAACACTACACTTCAGTCGGACTGTTATGCGTCGTCAGGTATACGCCGGAGAACAACGATTCCATCTGTCGTTCCAGACAACAGTGTATTTCCGTTCGGAAGAAAAGCAACCCGCTCCACAGGCTCATCGTGTTCGCAAAACTGTGCCACACACGACCCATCG

>AT11553

CTTGCGCCCAGAGCGAAGCGGTCCCTGTGCGCATCGGTGCCTTGACGACGCACACAAGCCTAGGCCGGGAGACGCGAAGGAGGGCGTGTTCTAATTCTTGATAAACCTGCGGTTGATGTCTACTGTAGGACAGCAGCTCTCCCACGACTTGCAGGTCAGCCACCGAATCCAGTTCCAGCCCGACTTCGAGCTCCCTGAGTGTCGGGCGATAGAGATCCCT

>AT11587

CAAGGGCATATTAGATTAGGCGGCGCCCGCATGGTCGTGCCGTAAGGGCGTGGAAGGAAGGGATATATGGTTGGGTAAGCGGTGGTTATGCAGTGATCTATGCAGAGTGTAGTGCGTGGAGAGATAGGGCTAATCGATACAGTTAACGGGATTGGACATACGGGGCGGAGACAACACATGTACACGTAAATTACTATTGCAATGAAAAGGGTCCGTCCGTAACTAGGTACCGAGCGAGGGAATGTCCGTGAGAATCCGAGGGAGAGCCGACCATCCAGGAATCCGAATATAAACAACTGAGTCAACAGGCCTGAGAACCGAGGAGAGGGCGTGGGCAGCGTGGTGCGAGCCGCCACGGCGATAAAGTACAGAACACCTAGGTATAGTAAACGAATATCCTACCCTAACACAATTATACACGCGTTCCGATGAAGCGAAGATGGGGGAACAGGCGACGAGAAGCCATCATGAATGGTCATGGAATGGAGGGAACAAATGGCTGACGTGATGCGGCTCGATCTTCTTGCACCTTAGCCGCATAGCTGCCTGAAGGAAAGG

>AT11589

ACGTCCGGTCCGTCTGAGGGGATTGAGAGCCGGAGCTTGAGGACGACTCTAGTGGTGATTGCTCGGCTTCAAGCTCGCGGAGACGTGCCTCGAGCTTGGTGATCTTAGCCTGGAGTAGCTGGGTGCGGCTAGTCCTTTTCTTCTCGTAGTACTGACATTCGTCGCCCCTGTTGGCCTTTGTGCACTGCCCGCACACTGGGCGCGCGCCGTCACATTTCTGGGAGGACGAATAGGTGTCAGAGTGAGCGGGGGCGGCGAGAGGTGGAGGAGGAGAGACTGACCATCTTGCGTTTGCGGCAGCAGAGGCAGGCCATGCCGCGCTGGAGAGCAGTGGGTGCCTTCATGGCGTGGGCGGTCTGAGGGAGGTGCGTAGGAGATCGGGGGGGAGGAAACGCGATGTGTGTGTTCAAGTAAGTCCGAGCGGTAATCTACGAGAAGCGCAGGCGCTCCCAGTGCCGTAAACGCGCTTGCATTTGTCCAATCCATCTGGACGAGCGCTGCACGGCGTCTCGGTCTTCTTCTCGAAATCAGGGCCGATCTCAACACTCGACCACCCCCGCATCCCACATCCCACATGGCGACACGCAGGCCCTCTGCGCCCGCTGCGCCCACC

>AT11591

ACCGGAACGTATTTGTGCGTTGTATCATTTCATACAACATGATGGAATGTGTTCAGCGGAGCCTTTAGAGCACAGGAATCACAATTGATCGCGCAATTCATCTTCCGCAGTCTTCCACTTCCACTGCCTCTCTCCATAGTTCACGAGCTCATTCTGGTTGTCCGTCGAAAAATCCATCTTGTTCTCCCCCATTAGCACCCTTTCACCACGAGAGAAGAGGAACCCATCTTCGAATTAGCGTTTTTCAGACACAGAGCGGCCACCCGAAACACGCACCGGATGTAGTGCAAAAGGCTCCCTGGCTTCTCCCGTTCTCCCGGTAAACTTTTCTTGTGCTCTTCCAAATCCTCGCTTCTGATCTCGCCCTTCTCGACGCCCTTGATCGCTGAAACGAGGTCCCGCACGTCCTCGTAGCTGACCACGCTCCCAGCGATGCGCACATGGTCCGGGATGTTGGGCACAGTGGCGGGGTCCAGCGTGAGGATGGCGAGCCGCCCGGCCGCCCGCCCAATGTCCGCCGTCGACGTGAAGGCGACCCGCTGATTGGGCGGGCCAATGCATCCGTACCGGTTCTTCTCCACGTCGAACCCTGCTGCGTCACAGATACCCCACTGCGGTCAGCATGATATCCGATATGTGCACCG

>AT11614

TCACAAAACCCTTGGAGGCGATGTTCGTGATGTTCAAGGTGGAAACTGTAACCTGCAGGGAGGCAGAGGCGGTGGTGACGCTCTGGATGCCGGTGACTACGGCTTTCTCGGTCTGGGCGAAGGCTCCGGCGAAGCCGAAGGCGACGACAGCGACAGCGACAAGCGCGTTCTTGAAGAACTTCATGGCGAGCAGAGAAGAGGTGAGAGAGGGAGGGAGTGCTTTGAGACGGATGGGTGTTGAGCAGTGGAGTTCGAGCGATGTCGAATGAGGAGCCTTGGGAGAGAGACGACCCCCTTTAAATATCTATCCAGAAGGCGCCAGATGGTGAGGG

>AT11616

TCATCCTAGCGAGTACCGGATGTAAGCACAACCGCAACCCAGCGTACAGTTGCCTATACTACCAATACCAAACGAGTACTCACAGGTTGGCATATATCCCGGGGATGTGAGCCTCCTCTACAGACTGAGGGAGCGGCTGGAGCCAGCGCTCAGGCTTCCACTCCAGAGCGTCTTCGCCCCAGATGGCCTTGTTCGTGTTGCAGGCGGGCATGTTAGATATGACGACAGTGCCTTTCGTGATGGGGACTTCTGTCATCATCGTGCCGTCGATGCCGCGGACTGGCTGGGAAAATGGGAGCACGGTGTCTCTTTTCGC

>AT11618

TTGCGTTCCCCGACAGCGATGAGGGTCTCTTGATCTCCGCGTTCGATCGCAGCTTTCTTCTCGTTGTATATATCCAGCGACCGCTGGTGCATAATGTTGCAGATCCTCTTCATGCGCTGGATAGGTTTGCTCGGGAGGCGGTCGAGGAGGAACCTCCGGAAGCGAGGGGGACCCAACTTCACTACCAGAGGCACAAACACCCGCGCCCATTGGACGCCGGCGAACGATGGC

>AT11619

CAGCCTCCGCAAAGTCGTTGTGAACATCCTCAGTGAGAGGATCGAAGGAGTAGCCCATCATGCCTTGTCCGACCAGCTCCAGCGCAGTCCGTCCCATCCAAGCCAGGACATCGATATCCTTCGGACCATCCTCGACCTGGCGCTCGAGGGCCGTCCTCAGCTGCAACGCAAGCGCACATAGCTTCTGTCAGCTTGATTGAACGATGGAAGGAGACAAATCCGACAAAGCGAGACTGCAGCTCACTTTGCCAGCGACGCCGTAGAATATCGGGGACAAGCCCCGCATATGGGCAGCGGAGAACGCAGGGTTAAGGAGCTTGCGCTGCCTCCTATGTTCTGTGTCAAAGCTTGCCAGCAGGCCCGGCCC

>AT11621

TCAATGTATTGGGTAGCTATTAAGCGATAGGAGTGTGTCCAACGAAATGACACCCACGATATTCCAGAAAAACAGGGGCAGTGCTAAACGGATAAGCGGTGGGTGGGCGTTGATATCGGTATTACACAGTGTATACACTTGGATATGGCGTACCTACGTAAGCCATATAATTGCTAGGTGTGTTCCGGCATCAGAAAAGCTTTGTCGAGGCTTTGAAAGAAAAACGACGCAGAAAAGCAAGACCCAAATCAAGGAGCGAGGCTGACTTTAAGGACCATCTCGGGCTTCATGCTCGTGTAGCTAGCCGCCGGGAAGGAAATTCCCGCAAAGTTCCACACGAACGGCTTCTCCGACAGCTCGAACTTGAAGCGAGAGAGGAGCATGG

>AT11625

ACCTGAAAAGAGCGAGCCGGACGGGGGTCCTGGAAGATTATCGACCGGCGTTCGCAGCACATAGGGGCTAACAAGGCGCCAGAGGAGGAAGATGACAGCAAGTAAGCCGGCTAAGAGGATTAGTCCGGCCATGTTGAAGACCAAATATGCTGAAGGAAATTGGTCAATGGTCATAATGAGCGAGAAGGAAAGAATGGGGTGTGGGTGACAACTTGAAT

>AT11622

TCCCGGGGATGTGAGCCTCCTCGACAGACCGCGGCAGCGGCTGGAGCCAACGCTCGGGTTTCCACTCCAGCGCATCCTCGCCCCAGACAGCCTTGTTCGTGTTGGAGGCAGGGATGTTGGAAAGAACCATGGTGCCCTTCACGATGGGGATTTCCGTCATCATCGTACCGTCGATGCCACGCACCGGCTGGGAAAGTGGGAGGACCGTATCTCTCTTCGCC

>AT11624

GTCCTTGCGCTCGCCGACAGCGAGAAGCGTCTCCTGGTCTCCCCGTTCGATCGCGGCCTTCTTCTCGTTGTAGATTTCCAGGGACCGCTGGTGCATGACGTCGCTGACCGTCTTCATACGTTGTATAGGTTTGCTCGGCAGGCAGTCGAGCAGGAACCGGCGGAAGCGCGCCGGTCCTAACTTCAACGCCGTCGGCACAAACACCCTCACCCCCTGGATGGCAGAGGATGACGGTCTGAACGGAAGGTCAAGATTAGCATCACAGGGGGTCTCGGGGAGGGCGGAGCCGAACCACACACACGAATGACTTGACGGCTTCCGCGAAGTCGTTGTGAACCTCTTCCGTAAGGGGATCAAATGAGTACCCCATCATGCCTTGTCCGACCAGCTCGAGCGCGGTGCGTCCCATCCAGGCCAAGACGTCGATATCCTGCGGGCCATCCTTGACCTGGTGCTCGAGGGCCGTCCTCAGC

>AT11635

GCCGTACAGATAGATCGGATGAGCAAATAGCGTGACGATTAAGCTTTCCTTCCGATAACTACACTATGGTCTACGCGGCATATGGTAGCAAAACGGGTGTCTGAAAAGTGTCATGTAGACTGCCAACGAACTTTCAACTTCCCCAAACCGCGACAACGAATCTCCCGAGGAGGGTCCGCGAGCGCGGCCAAGAGGTCCGTGGGCTCAGGTTTGATGCACCTAATCGGTGAGACACGGACCGTGAGGACATCGTCCAGGGCGGGAGTAAACGACAACGTGACGCTGTAGAAACCGCAGCTGTCACAGTACTTGAACGTCCTCGACATCCCCGACCATGTGCAGACGTGGTCGGTTTCACAATCGTGCATCACCGACTGCAAGGGGTCGGTGCCGTATGGGCGGAGCACCAGTCTGGCCCAGTGGGCGTAGGTTGGCACCTCCCCCGATTTCGCGCGCTGACACCGCCCAAGGATGATCCAGATTCCTATCTGCGTTGTCTCCTTTAGCTCCAGGACCACCCTGCCTCGCCGTGGCTGAAACAGGGTTAAGGAGTACCCTCGGCGGAGGAGGTATCCGATTGAATCCCAGGCGAAGTGGAAGTGGGAGTTGGCCCGAAGCAGCTGGCTTAACCAGAGATAGCGCTTGAGGTTGAACTTGTCTCCAGCGCGGAGGCGTGCTGCCACTGCGGAAGGAGAAAGCGGCGATTCGGAAGCGGGATCATCCCGAATGTATATGTCTGTCCATTTTGCTGTCCGTCCGTGGAAGCGCAAGGTCTTCAAATCGCCTTCAAGGAGAGTCGTACGGGGCTGAAACGTAACCAGAGTCTGGGGATCCTGTTGATACTTGATGCGAATGCCGATGCAGTACAGGAGGGATGGCTCGAGTAAGGGATGTCTCTTATCCGAGCATGGGGAGAGGAGAAGCGCGAGATGTTGGCGCTTGTCTTCCGTGCCGTCCTTGTAGGAGAATAGAATCGCCATCTGAAGGTGCTCGATCTCGAGAAGCGGGATATGTGCACGGATGCCATATGGGGTGATTTCGAACGTCGGCATATCGGGAATGGGATTCGAATAGTGGGAGGCTCGTACCCGGCAGAT

>AT11637

GCTCCAAAATGTCGCTGAGTTCTGCCTTCGTCCCCAGCACCTCCCAACCCCCGGATACGAAAAACAAGACAGCTGGTGCAAGCAGCTCCTGCAGCGTCCATCCCCGCTTGTGCCATTTGCAGCTCCGGAACGCGGAGGTTTCGTGGGCGAGGGTGACCGCATCGACAGTGGGGACATCGGCGAGGTATGCATAACAAACATTGGCGAGGGAATAGTAACGGAACATGGAGTTGAGAGCTTCGGAGAGCTCGGTGCTGCTCGTCTTGTCAATGCAGCAGGTGTCGACCCATAGCCAGTCGTCCCCGAGTGTCTCTGCGAGCTTGCAACATTCCCGGATTTTGTTGGAAACGTAGTCGCGGGGGTTTAAACCCGCTGCGGCACAGGAGGCGCGTAAAGCCTGGAGATCCTGGAAGGATTGCTCGTTGGGCGAGCACCAGACGTGAGAAAGAATGGCATATCCTCCAGGCACGATTT

>AT11643

CACGAAGCCAGAGACCGCGAAGTTCAGGATGTTCAGGTTGTTGACGGTCGTGCCCAGAGCCGAGGACGTTGCGGTGAATGACTCGATGGCGGTGATGACCACGTCGGAGCTCTGGGCCATGGCACCGACGGCGCTGAGGACGACGACGGGAACGAAAGTGACGAGCTGGCTGAAGAACTTCATGGTTGTAGTTGAGTGGGACTGGGTAGTACCGGGAAGTGGGTTCGAAAGGCTTGGTAGAGCAGCTGAGTGAGAGATGAGGGGATGGAGAGCAATGCCCGGAGTTGACGGTGGTTTTATACTCGCGGATGAGTGGTATAGGCGACCAGCTGCTCGACGCGGCTGCCTTATGCTGCCGCATCGGTCTTCCGAGTCGTCCATTGCGCGCTAGCTTT

>AT11666

TAGGCGGTGATAGTGGAACAGTAAATTGCGGCAATGTATAAAGCATGTAGATGTACAACATGGTGTGGATGACGCTGTTCCTTAAGGGAATTAAGCGAGGTAAGGTAGAGGATGCTGCTGGGGCTCGATCGCCCTTGGCGAGAGCCTAGAAATGAGTGTGAGGTTCGCAGCTCCTGCGAAGCCGGTCAGCTGGACATGCCGTCATAAGTGAGTGGTAATAAGTTATAAAACGGTTGGGAGCAGCCTTCATAGCTTCTTGGTGACGGCCACAGAGAAGTCCCCGAAGAATATGTTCTGGTCCTTGGGCGTCGCCCAAGACGCGTCGTTACC

>AT11678

GCGGCCGCCTAAGCAGCACTGCTATCCTTGCTTTCCCCACCGGATTCTTTGACTTCATGCGCATCTGGTGCAGCAGCTCCAAGCACCAGTCCATCGCGAGCTCCGTGATATGCCTCAGCTCGAAGCCCTTCTCCTGGTCGGGGAACGCGACAGGGTGACGGTAGCTGAACCACAAGTACAGCACGATGACCTTATGCACGGTCTCGAGCTTCGCGAGCACCTGCACGACGTCCTTCTGCACGGGCACGGCCCTGTTGCTCTCCATGAACACGAGCGCGGCGTTGAGCTTCGC

>AT11681

GGGCGGGAGCCTCAGAGCACGGCACGAGCTATAACAGTAATAGGAGTTCTGTGCAGTCTACTCTATAACATCGTGGGCAACCATCTGAACGACGAGCTCATGCGCGACAGATTCCAACTAGCTACTCCTTCAGAGGGCTCAGGCTCCGCTGGGTGCCTCTCAGACTCGTCCGATATTCAGGACCCCCGCGATCGATAGGTTATTTCGCCTCCATTCGGGTGGATACCCTCAGATGCCGCTAATTGGTGACTCGCGCTTACGCCTTCTCCCTCCTGGACATGGCCGGCGGCAGGACCTATGTTACCGACTGGCCCCACAGCATCTGCAAAGACTACTGAACTACCAATGCTGAGTGAGGTGTCCGAGGGATCGCCAGCCGCCAGTCCGGCTACCGTCCTCTGATGCGCTTCCTGGAGGTCGAGGAGAAAGTGGGATACGAGGACGGAAGAAAC

>AT11703

TCGACGTGGTTGCAGCACTGCATGGAGCCCGTGTTGCAGTTGTCAGCGGCGGCCGCAAGGACAGCGAAAGCAACGGTGGAGACGACGGCAAGGCGGGAGAACATGATGTTCAGGTGTGTTGGAGTGGGCTAAGGCGAGGGTGGTGATTAAAGGCGAGTGGAGAGTGCGTTTGACTGCTGCTGTCCGGCCTCGTAACCCCACAGCTTATATGCATCCAGAGGAGCGTCTGTGCGCTGCACTTCACTTCGCGGCACGCCGAGCACCTTCACGCCGCCTGTGCTCGATCCAGCTTCGAAGCTGGCCGGGTACAGCTGGCAGTCGACGCGAACGACTTCGGAGCGGGGCGCGCGTCGGCTACAGGCCTCATTCTTGAGTGGGCGCAGCGAGTGCGGCGTGCCGTACTCGCGTCAACCCTGAACCTCGCTTGACCAGCTCTTGAAACAACGCTGAATGCCGAGCAACATGCGCACCTTGACGTGTTTGCGGAACGGCGCTGCCGTTAAATGCTTGTTGGAGCCATACATCCTACGGCCATTGCGGC

>AT11705

ACAACGCAATGCTCGTGACGAATGAGACCACAGCGCGGACTACTGCGAGCTCCGTCTCCGCGCGTTCCCAGAATGTCAACGACTGGTTGATCAAGATTCCAGTCTGAATGGCCTCAAGGGTGAGACTGACGAGGGCCGGACCGATGTACAACCCCGGATGCGCCTCCAGACCCTCGAGCGAGAGCGGCATTGAGGCCTCCCTGATCGCCAAGGATCGTCAACAACAGCAAACACAGAGGAGGCGCAAACACTACTTGCCAACGCAGAGCGAACACAGTTGGAAGATGCGATGGGGAAGGGCGAAGGTCCAAACGCACCCTTGGAAGCGAGGGCATATTTAGGTCGGAAGCGGTGTCTTATAGACCCCGCAGCTATAATTGAACGTCATTCGGCACGCCCT

>AT11733

CCGGGGTCCCTTCGATCTCGCTCTGGATACCGTAGCCTGCGTTGTTCACGACCACGTCGAGACGCCGAAAGTGTTCCTCCGTGGCCTTGAATGCCGCCTTGATCTGCTCTTCGTTTGTGACGTCGAGAGAAACGATGAGCAGTTGGGTCGAGGGGTAACGTTGGGATAGTTCAGAGAGGGTGTCAGGTTTACGGGCTGTCGCCACAGCTCGTTCACCCGAGGCAAGGACGGCCTCCAGAAGGGCTCTTCCGAGTCCCTGATTGCTACCTGTGAC

>AT11738

CTTGAGGTTGCGAGTGGTGGGCTTGCCCAAAGCAAGGATAGCAAGGGACGCAAGGATCAAGCTCGAGGCAACCATCTTGGATGTCTCGGCTGCGAGGGGCCCGGGGAAAGGTCAATGAGACCTTGAGTGATCATGATGGATCACAAACGAAGAAGCTTTATACTTCGTCAAACCAGCACGGCGCATCGCCATGAAATAGTGAAGCAGGGGAAGGTAGTCGATGACGGCAACGGTGCATAAATCTTGATGGGCCAACCACCTATGCATTTTGGCATCCTGCCATGATCCTGGGGCCCTGGGCCGAGGTGAGCGATCGATAGGTGGCACGGGTATCGAAAAAGGGAAAAGGGCGGGGACTTGGCTGGACTGGGTCACAACTTGAACACTCA

>AT11745

CATCTGCAATAGCCTCGTATAGATTCTTCCCTTGACGCCCTTTATGAGACAATGAGTTCGCGGCGAGGTACTCGGCGAGGTTATCGTCGTCCTCCATCGTGTACGGCACGCGCTCCCGCTTCCCCGGCGACCGCGATTGAGGAGGTGGAGGCGGAGGTGGGGGAGGGGACGCTGGCGCTGAGGCTGCGCTGCCATCGGCCTTGACCTCGACCTTGGCCTTGGCCTCCTTCGCGGCAGCGCGAAGTCGGATCGCGATGCGCCTGTCAAAGTACTCCTTGTCGGTGCGGTACCGCTCGCGCCAGGACTGCCATGTGTGGCGGCGGTGCCATGGCCATTTCCCCTCGGCCTAGGATGGGCCGGAAGGATAGCATTGAGACACGGAAATCGGTGGAGGGATGGGGGAGACAGGGAGACGGGGAGACGTACGTTCTCGACCAGCCGCTCGTAGAGCTTGTTACCTAAGCGTCCTTCGGGCTGGGGGTTGTACTTTGCTATGTACTGAACAAGGTAGTTATCTTCTGTGTCGGTGAATTCGTTGC

>AT11748

GCGGTGCTCGAAGTAGACCGGGTGGTTCCCGTACAGGTTCTGGCGCTGCGGGAGGCCGTACGCGTCCCGGGACCACATCGTGCGCGTGTAGTCGTGCGTCGGGAGGCGGAACGAGTCCGAGTGCTCGCCGAGCCCGTAGATGTTCGCGTTCGCGGGCAGGTCCGTCTTCACGCGCAGGTACTGGGGCTCAAAGATGATCGGGTGCGACGCGGTGGAGAACAGGACCTCGCGGGTCTTGGAGCGGTAGATGGAGAATGAGAAAGGCGAAACGGTGTAGTTGAACTGGATGTTGGAGGTCGAGGGGCCGGCGTCGCTGACGGCCTTCGGACGGGCCAG

>AT11983

CGCACCTGCGCCGACGCTGCCGTAGGAGAGCGGCATGGAGTAGCCGGCAAAGGGCACCATTTTCGCGCCGTTCGAAACGTGGAAATCGTACAGACCCGTCTTCCTGAGCTGCACAGCGGCGAGATTAGCGTAAGAAAGCGGGTGCGACAGTGAGAAGCACTTACTGGCGCATCCGCCGATGTGGCCATGCGTCGGACATTTGAGACCAGACGGCCAGCTCGCGCGACCGGCGCGCGCGAGCGCAAGAGGATATTTCTCGCAACGAGAGCGGTAGCCATGAGAGAAGGGAGTAAGAGAGAGGGCAGATGGGGGGCGGAGGCAAGAAAACGGGGGCGCGGCCGGTGCGCGGACGTCGTTTGGAACGAGCGGAGAGCCGACGCGAGGGCGAGGGACGTAAAACTCGCGTCTATCTTGTTATCTCTTGGGGGCGATTAGATTAGATGGCTATAAGGAAACACGCGCGGGCCGACGTGGCAGATCAGCGGGCGACGCTCGTTTCACAACGCGCATGATGTGGCTTGAAGCTCTGTTGCTCACTGCCA

>AT12002

AGGGTGTCGTTCCACATCAGGTCGTCCGCGCCGTGCTGCTCGCGGACGGAGTTGTGCGCGGTGAGGTACGCGGAGATGTCGCCTGAGGAGGTGGCCTGGACGCCAGCGAAGAGGGCGAGGAGGAGAGGGACGGAGAGGAAGAGGCGGTTCATCATCGCTGCAGAGTTGGCCTGTTTGGACTCGGTGTGCGGCGCCCGGGGCTGTAAAGGGATGTGTGGGGTGGGGTGTGTAAGGAGAACGAGAGGAGGTGAAGGTGTTTTATGTAGGATGCAGGTCTAACTGCGGAAGCCTACGACGCGCATAGACGCGGTGGAGCAGTATGGGATACGTGCCTTCGGATCAAAACCTCAGCGGTGGGCCTCGGACTGACATCTGGGCATGACGGGATTTCCGAGCGCAAAAAGATTGGAATGCCCATTAACAAACCCA

>AT12005

CTCCCGATCCAAGTGAAGCGGATAGTTCGTCATCCCGGCGCTTCCTGAAATCCTCCACCATGTCTCTTCCCCAGACGACGACGAGCCACTGGTCCCACATAGAAACCTTGAACGCTGACTCTGGATACTATACCAGAGAGGGATCCCATGAGTCCAAAAGTTGGCAGGCAGATCGAGGCGAGAGACGTACCTTCCTGTACCCTTCC

>AT12019

CAGCCTCCCGCACTCGAGGAACACCTCCTTCAGCTGACATATGTTCGGATGCTGGCAGCTCTCCAAGACCTCCACCTCGCGCAGGACGATATCAGAGTGCGTTGTCCTGAGCCGTGGCACCTCCTCGTCTGACGACTCATCGGAAGCCTCCTGGAGACTATGGACGGGGATCATTTTGACGGCATACCACTTCCCTTGCTTCTTGTGAAGTGCCTTGACCACCGTCGCGAACGAGCCCTGCCCGAGGACGTTGAATAGGTCGTAGTGCTTATTGAGGCCGCGGCAAGGGTCATCCGGATTGTCGACCTGCCGATAGATGAAGCCT

>AT12020

ACTCACCTACATCCGCACTGCACGGAAGACAGATATCGCTCCTAGGAGAGCTGCCGACGGTGTACTTCCGCTTGGCTTTTGAGAGCTCGATGCTATGGGCCTCGGCGTCGGCCGCGTTCCAAGGAATGAGGCAGCCCCAAAGCTCAGGGACGTCGCCGACTCTCCTTTTCTTGGTTCTACGGCGTGAAGCAGCGTCACCTAAGTTGCCCGAGTTCCCGCTACCGGTTTCGCTGAGGTATCGCATGTGTAAACGAGCCGGGTGAGGGGGCAGGTCGTCGCGGGAGGAGAACGAACCGTGGGAAGATAATAATGGCGAGAAGTCACTAGTCAATGTTGATCCCGGCCTACTAATGGAGGTACCGGTGGCTTAGGACCCCATTCTTGTTTTCACCCTCATCACATTTTATACGGCGA

>AT12022

ATTACGAATACCAACACCCCAAGGCTCCAGCTGTCGACGGCCTTGTCATACCCATCAGGGCCTTGGTCAAAGACCTCCGGCGCAACAAATCCGGGCGTGCCGCATTTAGTCTTGAAGCGCTAGCCATCCTCACCAGTCAGTGCCAATCGAACACTTGACTACCGACGTACCTGCATTATGGACAGAGAGTCCATGACCTTAGCCAGACCGAAATCCGCTACCTTGACGATGGGTGGTCTGTCTTGGGTAAGTAGCACGTTCTCTGGTTTCAGATCCCGGTGGACGACGCCCATGCTGTGGA

>AT12044

CAGTACTCACCTGAACCAGGAATCCGAACAAGGCCCTCGCCGCTTCATCGACATCTCCGTACAGACAACCTAAGTATACCCAAATATTATTAGCAATGCGACGAAGGCCGATCGAAGAACAAAGACACACCAATCAACTAACAGCGCCCACCCCAATATCTGTGTCGGAACGGGAATGAGTATGATATTACCCCCAGTGAAGCGAGTCGCGAATGAGCTTACCGGGCCCGTCAATGCAGTGGCTGCGCAATCCGAGACATGTTCTTTCAGAAACCTTGGACCTTGGGCTCCACACCTTTCCTACCATAGTGAGCACATCCAGGTCTATGGTGCATGGATCATGTGAATGTATACGTACGTGGCGTCGACCATGGCGAGGGCTGCCGTAAGGTGCATACTGAGCGATGCAGTGGTTGAACAAAGAAAAAAAATCGATATTTTCATCAGCCGTATGCAATGAGGGTGTTCTGCGTCA

>AT12051

CCCGTTGTTCAAGAATCAAAATACTAGCGAACACGAACACAACTACGTACTTCCCAATGGCCAACACAGCGTAGACATGATGTGCGGTCAACCACACTTGGTTTACTCCAAATCTTCGGCCTCATCGTTCCCCGAGCCCGGATAGATATCACCGTCAATAATGCTTGGGCCCAACGATTCTCCGATAGACCCGATGACTCGATCAAACCGCACCAATGACGCGGTCCCGGCCCCACTCCCTCCGGTCGAACCTGATAGTCTCCGTCTAGCGTCTTGTAGATTCATCATGAAGCGTGCGGTGAGGA

>AT12057

GTGGCGAGATAATATACAACATGAATACCATAGGGCAAAACAAGTAGTCCAACCCACTCAAAAGCGACGAAGCCATCCCCGCTGGTTCCGAAACATGTCGTCTGAATGCCCGGCAGACGCGAGCCTGAGAAACATCAACATCCTCATGGCATTCATTCTTCAAAGTGGCACGCGTTGAAGCGGTATGAAACTTGTGCGCGCGCCCAATCTGCGATACCCGAAGAAACGCCTCCGGCTGCTTTGGCAGAAACCAGCCTGACAACCCTGTATATCATGTGGGAAACGTCAGCGCAGTCAGGGTAGAGGATATTGAATGCCTGATGCGACGTACTTTGGCATGGCCGCGATATGCCAGTGGACCGCGGCTCAGTTGCTGCCAGCGCGAAATCTATGTGAATGTGTTAGCGG

>AT12077

GAGGTGCGCATGTGGATCGTGCTTGTCGTATAAGACCAGAGGTCCACGCAATCCATCGCAATATTGGTTACCAAGGTGGCTGTGGTACCAGTACGTGCCTAACCGCCGCGGTGAAGTTAAGCCACAAACCTGATGAGCGGGAGTACAGTTCGTGGACGTACCTGAGATGCCCGTGGTCTCGAACTCATACTCGAAGCAGTTACCGGAGGTGATGGGGCATTGGTTGATGAAAGACGCGCCATCTGCCCAGTTCGTCGTGTGCTGCAGG

>AT12078

ACCGAGTAGCCAGCTATCACCGGCCCTGGAAAGGTGCCATCGGCAAGGACGGCATTGCGTGAGGTGCCGTCTGTGGAAACGGGCTTGTTTACAACCGCCAGATCGGTAACGGGCCCGATCGCAGCCCGGGTGGGTTGGGCGATGGTGGCTAATATGAGAGCGACGACAGCGGTGCACGAGTTCCCCATGACCATGCTAGCTTGTGAACTGGTGAGGAGGTGGGGATGTGTAGACGGATAGACGGGTAGACGGGTAGATGGGTAGGGAGGTAGAGGATGGGTGGACGGGCTGTTGCTCTTTCCCGCTTATAAGAGGTTCAGGTGCGGGATTGCAGTCGAGTCCGAGAACCTATCATGGGAGTTATTGGAGACCGACATTCTGCTTCCGCCGACTGTGGATAATAGGAAGGAACCGATCGAAAGACAGCCTGGCGGGCATACGAGTCCACAAAACAAGGTC

>AT12082

ATACCGAGTGGCCAGATATCAACGGACCAGGGAAGGTACCGCCGGCAAGGACGGCATTACGGGAGAAGCCGTCTGGAGCGATGACCTTGTTGACGATGCGCAGGTCGGCGACGGGACCGATCTGGGCCGAAGTACGTCGAGCGAGTGAGGCCAGTAGGACGAAGGCGACACGGATGAAAGAGTTGCCCTTGGCCATGATAGCTCGCGAGCCCGTGGACGGCAGTGGGAAGCGGAGGAGGAGTTGGGGACAACGAACCTGGCTGTATTCGTCGTCTCCTTATAAGCAGTTTCGGTACTGAAGGTGGCTGACGAGGCGAGCCTCCGTGGAGTGGAGGCACCATGATCGAAGGACAGCGGGGCGGGATCAGAGCTGGCGGGCTGACGGGCTGACGAACTGACGAGCTGAGGCTGGTAATGACACAAGATGAAAATAGATGGGTCATGGATAGCAGGCTGACAGTCCACAGGAGGTCAGCATATGGCACGAGACCAGGAGAAAGTGGTATGAAACGCACAATCATGACTGCCCGACAGTGCTACCACAACTTTCACGTTTATCCAGCCACGTACAGCTTCACGTTCGCATGCAGCAGAAAACGCGTAAGACACTATCACAGATGGACTGAACGAAGCAAATGAAACAGGGGTTAGACAAATGACCCATGCCTGGCACCGACTTATGGCGAGGCAAGCCGGAGTAAGACGCAACGCTTGAT

>AT12103

ACGCACCTTGTTCACGTTGTTCACCACCCCGTCCACGACCTGGCCCTTGAACGGCTTGAACACGATCGCGCGGTACCGCGTCACGAACTCCGCCTGCCCGCTCCCGGGCATGACCATGCCCTTCCCGATGTCGAGTATGGAGACGACGGCGATGATGTAGCCGAATTCGCCAGAGCAGGTGCCCTCCACGTCGGAGTACAGTTTTGATTCGAGGAACTGGAGCATCCGCGGGCCGAAGTAGGATGGGTGGAGGTGGATTGTATGCGA

>AT12105

GAGTGGGAGAGCAATCAAAGACAGCACGACAATGACGAGAAACACTGGAGCAGGACTATGATATAAGCTTTCGGAGTTGGTATATCCCTTCTCGCGCCACAGCGACGGTATCGTAGACAACGCGTTCTGGGGCGGTTTCAGTGCGCGAGCGGAGCCGCCATCGGAGGAGGGCTTCGTCGGTGCGGCCATAGATAGATTCGAAGATGTGGAACGCGGCGTCCGCGGAGGGCCTCTGGGCGGGGTTCTTCCACTTCATCATGTCGACGAGGGGCTGGATCAGATCGAGACCATGGTATTTCTTGGGGGGAGTTATTGTTATTGAGAAGGCGAAGAAAGTTGAGAGGAGTAAGAGCCTACCGAGAGGAATTCCTTGTAGTAAAGGTTTCCAAGGGCGAAGACGTCGGCACGGAACGCGTCGTATGGGACCTCGTTAGAGAGCTCTGGGATCTCCTTGTCGCGGCCCGTGCGTCCAACTACTAGGAGGGATTCTCCTTCTTCAAACAGCCTGGATAGTCCAAAATCGACGAAGTAGTAGCGGACGTTACCGCTTGAGCGTGGAAGAGGTCGGGCGTCGTTGGCGCCATCCGGTAGGAAGTCGGTTCTGACTGGATGATGACCCTCTGGGTAAAGCGTCCGCCCGTCCATCATCACATTGGCCGCAGCAATATCCC

>AT12106

TGGTGGAGAAAGCGTAAACCCTGGAGTCGGGGTAAGCATTACAAACCGCTCAAAGTCGGGCGGACAATACCTCCAGGGACTGACGTATGAAGTCCATGACCTCATCGATTGTCCCAAACTCGGGATCATTGAACTGACGCAAGTGAGGCATGAAAATTAGTGAGATATGTGGTTCTGATGGGTCTTGAAGGATATCGAGGACAGGTATGCAGTGGTTTTGAGGATCGCGCTTGTCCGTGAGGTATCGTGCAATGTGCAGTTCTTGGGTATCATTTCTTGTACACTTGACCGCGAGACGGAAATTGTCCGAACGACGAGTGACGTCTATAACCCCGGGAGTCTAAATACAAGGGGTTGGGGTTAGCACAGTTGTAAGCGTACAGGCTAGCACTGGCAGCAGGATACACACGATAAGATCGATAGAATCTTCGCAGAACATGGGATCCATGTTTTTACCAAGCCAAGAGGGCTGCCAATCTGGATGATACCTAGGCCGTAGCTCATAACCACGGTCGTAGAGGAAGAAATAGCGGTCTCTCCAGTAGATCTCTCCAGGGAGCAAGTTGTAAAGGCCATTCTCGGTATCCTTTGCATGACGGGCCGCGCATTCTGGAGAAACATATGCGTAACGTGGAAGGCGGGCGGGAGTAACGCTCATTTGACTGTCGCCCAGATGAACTTGCTCGAAGAACCAGTCTAAGAATGTGAGCGCCCA

>AT12108

GTAGGCGTTGGTCTGTTGTTCGGTGTTTCTTTCCCTGCCCACGACTCCTTCTCCCCACGTTCACGTTGTCGAGGCTGAGCTATGTACTATGTCCTTCCGATGAGTCACATGTATTTATACGCCGTTCAGGAGACGAATGGCCCTCTATGATATCGATTTTCAGGCGATTTTGCGTTCGTGGTCGGGCGATCGAAAAGGTCCTGGGGTAAGACTCATTTTTGTAAGCGCACGTCTGAACCAGAAACAAAATGTACATCTACGATAAAACCCTCTACAAATGCGGTCAGATCGTGTATCTGTTCGACACAAGTATACCACATTAATTGACGCTGGTCGCAGGTGAGGGAAGCGGGTAAGGATGCGCACCTGAACAGCGGCGACAAGTCGCCCATCTCCCCGAAGTCGCCCCTGACCTCGCCCCGGGCCTGCATCGCATCCTTTTTCCTGTTCAACCGCGCGAACTCGAACATGGCCGCTATGCACAGAACAATCCT

>AT12116

CCTTCGCAATGTACCTGGTCGGATCCTGCCGGCCCTGGGGATCCTGCAAGAGCTCGATCCCGGTCGGGACAGTCGAGCGCTCGACCTGGAGCCCCTTCTCGGCCATAGCCTCCAGGAAGATGTCCTCGACCCCGCCTTGATGGAGGGTGACCTTTGATCGACAAACATCATTGGAGATCGTTAGACAGCGCGCCTCGCAGAACTC

>AT12125

TGCCAGACGAGGTACCGATGAGTGCTGTGCGACAGGCATCGCACTCAAAAGTCTAGTGAGAGCTGCTTGGGGAGGTATGTGCGAATCACACTGAGCACGCCGTCTCCTCCCCAGACGAATACGCGTCAGAATCGACGCTGGGCTGGCGGGTGCGCCGGGAGGCTGCCAAGTTCTTCGGAAGGGAAATCGTGCCGCGTTGCTTGCGTGAGCATCGAGGGCGATGCACTTTGGTAGACGCAGGCGACCACTGCTTGCGCGTTGGCTGCGCAAGGCGGGTGAGGCGAACGTTCCTCACACCTTGAGTAGCCAGTTCCCGGTCCACGCAGCGTATCGCACCAGTACTGCCCAGCATGCGGAAGTCGTGAGCGCGCGTGGCGGTGCCTGATGTGCAGAAGGGGACCCGAGCAGGCTGGAGGCGATGAGCGGAAGCCTTCCGAGATTGGGCACGAGGTCCCAAATGAGAAGCGTTGGGATCGCGTGAGATGGTGGTGGTCTCGTTGGCTCGACCATTCGATGCGCTCCTCGTATCTTCTGCCAATGCAACACTGGCATTGACAACAAGGGCTGTCGAGTTTGTGGACGTTATGTCTACGGTCTCAATCGTAGACCCGCTAG

>AT12134

TGGGCTTGAGAGGTTTCGAGGTTTCCTCGGAAATATAGAACCTTGTAGATACATTAAAACGCCCACCGTCATTGAAGTATAGACGAAGAATTGAACATCTTCTGCGAAAGGATCCAACCATGCCACGCGGAGAACCTGAGGGGGATACGGGTCTCTGAAATGCAACAGTACTGATACACGTCCATCACTGCGCGAAGTCGAAGCTAGTCACAAGGTCCACAGCGCCGGGGAATCGAGGCGATAGTTTGACAGGGAAGTCGGATGGGAGACTGCGGATAATGTTAACAACCCACGCGACGGAGAAAAAAATCAATGGTAGACGGACCTGACAAGCCCAGAACTCCATTCGACCTTGGAAGGTACGCCTGCGGGGTTCGAGATATCGAACACGTGCAACATCAAGGCGGCTGTGAGCCAGACACTCTCGTCTGCAAAGAACCTGCCAGGGCAGATCCTTCA

>AT12155

TTGCGTTCACATCCTTCAGGTCGTGAACGCGTGCACCTAGCCCCGTAATCGAGGAGAGAAGTCAGTATCAGTTCTGCGCTAGGGAAGGAAAAGTGGAGACAAACCTTCTTTCCCTTGCTCCCCGAAGGTCATGGCACCCTATAAATATTAAATATAAGCGATATGAGCTGTTTAGCCTAATGCGTTATGAGAGGTTTCAATGATATGTTGTTATACCATTACGATGTTCAGCGTGCTCTTTTGCTCAGTAGCCATTCGCAATCGCCTTCACTGTTTCGAGAGGTAGATGTCAGAGATACGAGACGAAGGGTGTGAACTCAAACCT

>AT12159

GGCGGGCCATATATCGCGCTCCGTGGCTCCCCCCGGATCCCGTCGACGGTCGAGAGGACAGTCTGAGTGACGGCGACGGAGAGTGGGAGTGAATGGGCATCTCGGTCGGCGGGGAAGACCTGTACATTGGTTCATCGTACCGGACGCTGCTCGAGGGCGACTACTATCCACAACGTTAGATACGATACATACGTAGATGAGGAATCGGGATGTCTGTGGCATGTGTCATCGTGGTCCGATACCGATGTCGAGAGCATGGCATATGTATGCGAACCTTCCGCTAGTTTGTTTGCTGTACGATTACAAGTGTTAAGGTATTAAGGAGAGTAGTGTCTCGCGGCGACAATGCAATGGGGGAGATGGTGGTGGGTGAACAGAACAAACAAGTACAACTGACAGCAAGATCCGATAACTGAACGAAGAGATGAATGCAAGGTTATATGGTGTAGGTAAGGTTATGTCGATGTTACACTTTGGTCCTCTTGTAAAACAGAATGTACGCCGGCTTGCCCTG

>AT12161

GCTGGAAATCCAGGAAGTCCTTAAACAACACGCCCTTCTTCCCTGCCTGGCTCTCCGCCCGAAACTCCGGCGAGTCCATGATCATATGCACGAGAGACGCAGACTTGGTGAGCGCGCTGAGCGCAGCCTTGAGGTCCCCTTCGTTCTCCTTGACCGCGGCGAGGCGCATCTGGGTCCGGGCGGAGGCAAGAAGGCTCGTGGGGGAGGCACCTCGGACCTGCTGAGCCCTCTCCTTTGCGCTCGCCTTGATCTCCGCTATGGTGTCGCGTTGGTATGAGGCCCCATTTTGCATTGCTGGTTGTGGGGGTTGGTCGTCGGGTAGCGGGGGTTACAAGAGGCGAATGCTTACCGGACAGCTTCGGAGTGAAAGCAGTGGGAGAGGCGGGGGAAAGAACAGCGATGCCGGGCATGAACCGGCGTATACGCGTTGTTTAAGAGGGAAAAAATAAGGATAAGTGGGCGAGAGGGAGGAGAATGAAGGTGATCAAGAGGACTGC

>AT12007

GTCCTGCCGTAGCAGCTCCCCTGCTTGCCGCGCTTGTACGCGGAGAAGATCGACCGGTCGCCGGTGGTCACTCCGCCGCGCATCACCACTTCGCCCCTTCCGATGTGGATTTGGGACAAACAACGCAGGGGAGCCGATGAGCACATCGAGCAGGATGACGAAAGAGGCGCGCCAGCTGGCATCCGTCGCAGACAAAATCTGGGGTTAACGCTCTCTTAAGTTAAGGTGCGGGAGGCAAATTTTACATGGACTTCGCGAGGATGTAATTCGCGACTCCCCCGTGGCTGCGGTTCCCGCCCAGGTCGACGGAGTCGAGCGGGAAGTCTGGGATAACATAGACGCGGTCGTGCCCGTGCCCGTGCGCGAGCACCACGGCCACGCGGAGGAAGATTACAGTGTCCGCCACTACTCGGCATACACCCTGCATCTGCCAGTGAAGATCAGCCGAGGACACGAGAGGGGAGGGGTATAACGTAACGAAAGACACGGAGAGCACTCACTGTGCGAGATACGTGGTCCAGTGCGAGCTTCATGAGCTGTTTTGTGGTTTCGTCGAGGTTGTCGTCGCTCTCCATGGCGCCGCGACTCCGAGCACGGGCTCTCTCGATACCGTCTGACTTGAAAACGAGCCAGCGGACGGCGGCGTCCACATCTATGCGAGACTGTTCCGTAGAACCG

>AT12445

TTCCCATCAAAACAGACTGCGTCACTACTCAGAGAAGAGGAGCAAGGTACATGGTGCACAAGATTCTCGATAGGGTATATTGCGGCGTGTATTCCGAACCCTATGTCTACATTCAAAGTTCAACCAGAACTTTCCTGATTAACGCCATGTTTTCAGACATCTCCGAGCTTCCCTTGGCAGTGGTATGCTCGTCGATCGAAGATAACAACTTTCTCCGGGCCGGACGTGGTGCGAGCAGAACCTGATCGCTGGCGGATTGTCTGCGACGACCCCGGATAGCCTTGAGCCAGCCAGAGGCGATGCCGCCCGGGGGCACGACCCACTTCTCGGCGAGGTCGTAGTCCGGGGGCACGATGCGGCCCCCAGAGAGCTTGCGGAGGCCCAGGCGGTGCCGTTGAGGAGGATGCAGGAGACGATGCCGGCGATGACGCCGTACGCGATGTTGTAGGTGAGCGGGATGATGATGATCGTGAGGAACGCAGGGATCGCGTCGCCCACGTAGTCCCAGTTGATTTCGCGCGTCCTACGGGGGAGAAGGAAGGGTGCACGGTCAGCAATCGCCAATCGATATAAACTTCCGGAACAGCCAGTAGTACGGGGCATTCGGCTGGACGGACGGAATAGAGGAATGGAGAGAGGGGGGACTCACGTGCGGATCATGAGCGAGCCGACGGTGACGAGCGCGCCCCCGGTCTTTCAGGAGGGGATGGGCGCGAAGATGGGGCGCGAAGAAGACGGAGATGAAGAACGACGGCCGCGGTGATGCCCGTGCGGCCGCCCTCCGCGATGCCCGTCGCTGACTCGATGAACGCGGTGACCGGCGACGTGCCCATGAGCGCGCCCATGGAGATTGAGAACGCGTCGACGCAGTACGTGATTGTCGAGTTCTCGAAGTCGAGCGTCACCAGGTCGCGGAGTCCCGCAAACTTTGCCATAGAGTA

>AT12463

ACTAACTTGAGGAAATCAATGTGAACTCAACCTCATTACCATGACATGTAGTAAGAAGGACGCTTACAAATTCAGCTGTGGCGCCCGTGAATCCAACCACAATCACCAGTAAAATCAGACATATCCACGGATTGACATCTACCCTACACTCCTCGTGTTCATTGGACGTCAGCGGTGGGGCGGGGGATGTCATGGACCTTAGGGTATGAGTTCTTGAGTCCACATGCGGAAGGGTATGATCGTGCAAAACCGGAGACGGTTGTGGACTTAGGGTCGCGAAACGTCGGGTTTCCCGTGGATTCCGCTCCGAGGAGGGCCTGCGCGAGCGAGGAGGCGACTGTATGATGACCGAACTGATGATATCGCCTTCATGGAGAGGCAATGTGTGGGCGCCGTGATGTGCAGGGAGGACCTGATTGCGGTACAGACATGACGTAACGTAGCTGCGATCGCGGCGGCGAGTTAGCACAGAAGCGAGCGGCGAGATTGGGATAGACATACATGAGGATTAACATGATGGATATTCCTCGACTCATGCGCAAGATGTCCCATCGCACGGAGTCGCTCACGAGGGGAACGAAGTTAGCAGGTAGCCCAGATTGGTCACCGCTTCCCACAGCGACTGAGCCACGATCGAGGGCGGCAAAGAAGATCGTCGGAACGAGGATCCCTAGAAGGCTGTCAAGATAAGTCAGTCGATGATATCGGAAGAGGTCAAAAAAGACAAACCCTGTCATGAGAAGCGAATGGTTCAAGTTGAATTGGGGCGAGAGTTTTTGTTCGTAAACTTGAGTACCCTGCACGAAGAACGAAACTCCTGGAATGAGAAGGAGGTGGAGCAAAAC

>AT12473

TCGCGAATTCGTCAATTATGAAGGCCCGAGGTAGCCGAAATATAAATACACGGGAACTCTGTGTGGTATGGGGGAGCGCATGAAGATGTGCAGGTAAGTTAAGTGATTATCACAATTCAGGAATGCGATGGGTAGGATGTTGCAGATAAGCGATGCAGGACGTAGGATCATGGCGATGCGATGATAGCAGTGTACAACAGAAGCGAGCGTGTAAGTACACGAAGCGCCGCGAGGCCCCGTCCATCAGTCGAAAGCAAGATAGTGGGTGAAGCTGTGAAGAGCGAACGGGCACTTATGTCATGACAGCACGCCACGCACGCGCGACAAATTCCTGCCCGGCTTCGGCGGCCAGTTCCGAAGCCGCCTCGGAGCTTGCAGAAGAGACGGCCGCCGCGACGGTCTCTGTGCACGCGAGCATGAGCTCGGCGGAGGGTTGTCCGGGATAGAACCACGCAGTGAGAG

>AT12502

TCTGCGGTGTCGGATTCAGATGAGCCCAACAAGCCCCGCCTGCGCACAGTATCTCGTGCGAACGACGCAGGGCGGCGGGAGAGGCGGAGACGATAACTCACTGGTTGTAGCTCATCTGTGCCAGCTCGTTCATCTGCGTGCCCGACGGACCCCACGGGTCGTTCGATGTTGCCTCGCGCACTTTTGCTTGGACATCTGAGTACCCCTTGGTGTAATTCTTGGCGGCGCGGACGATGCCCTTGCCGAGGTGCTGCATGCTAGACATGTTGGATCCCGGACTGTCCTCTGGAAGCGATGAGAAGAAAGCGAGGGGGATCAGGGAGTGAGGCCTAGTGGGGCGCGAGTGTGGTGGTGGTCACCGGCAGCGGCTGAGACAG

>AT12510

ACCCGCCTAAGACTGCCTTGGACTCCGACGATCCCGATTGTCGTCCATCGAGTGACTCCTGAGATTCTATGATAAACTTCGGAGGAAGCGGCGGAGGTATGTCAGAAATTGCCTCATCATCTGCATCCTCTAGTACATCGACTAATAATCCTTCCTCGAGAGAGGAGTCTTTAGTCACGGATACGCTCTTCGAGTTTCGGCCAGGCACTTTCGCGGCCGTTTGCTTACTCA

>AT12514

TGTGTCTGTATGAGTGGTGGTTTTTAGCATTCTCGGGATGTGTACAACACGACAACAACGTCAACTTCTCCCATCCTCCTCCCGCACTGCAACAACCCTACACATCCTGCATCCACCCACAACTTCGCTCCAACACATCTCGTGAACACTGTAAGTTCTATCTTCTACCGTCTAAATCTATGCGTCTCTATCTTGTCTAGAACAACACTACGCTCTCTATGATCTATGCATGCAGTCGTACTCGTGAAAGTCATGAAGCCAGTCGACAATCTCCATGGCCAGGTTCGAAGGAAGCATCGACACTAGATCAAAAAGAGGGGGGGTGCGGTTCTGTTTTGAACAAACGACAGCCAGACGAGAAGATAGAACAGAACGGCGGCGTACACGTTGGCGACGATCAGGCCTCTCCCGGCCGCACCCGATCGTGCTTGCGAACGGTTCGGGAGGAAATTAACGTGACGCCTTCGCCTACTCATCGCGGGTACAGTAGGGTGTGACGATGGGAGAAAAAGCGAGACAGCACGATCAGAACAAAGCACGGTAAAGGCAGAAAAGGAGAGGGGCAATCAGTCAACGCGAGGACGCTTCCTGCTCACATTCCCGTTCGCAGCGGCCGCCTGCTGGCCGTCGACGGACTCGAAGTACTCGAGCGAGCCCTTGGGGTCGGCCTTCATCGTCTTGTCGCCCTCGTTCCAGTTCGCGGGGCAGACCTCGCCGTGCTCGTCCT

>AT12537

GCGGGCTGATGCGATATCCATCCTGTTTCGGTGGTGTAGTGCCACCTACGATATCGTGATTTGTGGCCACAACAACATGGACAGGAGCGACGAAGTGATCGACGGTGATATGTGGCTTGCACATCAGTGTCGGTGCTGGTACAACGCAACTGATCCCGACCTTCCCGACCGCGCATATACCGTGGACAAGGATCCACCAAGGGACCACAAAACCGACGGAGTTGGCTACATACGTGCAGTGCGGGTGTAGCGATAATATTCTGTCCTGTGGGGTTAAGAGCAACCTGGGTATTAGTTTATAATTGTACGACGATCACCAGATATTCGACTTGACATGTTCAAGTTCTCCTCTCAGCTCGACCGTATCAAGTAGCGTACCGTTGCACTCAAGTACCAAGTACTGGTGTTACTTGGCAAGGATTTGAGAGGCTTCTTCACAGCTATTTGCCGATCGCATCGGTGCCTAGTTATGCATCAACGCACATAATACATCATCCCGTCCACCGCGGTCAAGATAAGGCGGGACAGAGGCTGCGCCTGGACCGGGATCTATCAGCAAACAGACCAGTTCATAATAAGGA

>AT12545

ATGCGGCCAGATAGAGAATGACCGCAAGGGGGAGGATCGCCCAATCCACTTTTCGCCATGTCCGCTCGACTAGTTCGGGTTTGAACCGGTGAAGCTCTGCGCGCGCGTCCTCTAGTGCAGACGCAGACTTGGTCGAGGACGGATAACTTGAGGAGGAGGCGTCTGCCACTTTGGCGACGGACATGGTTTCAGCAAGGTTGCAGCCTGAGAAGCTGGGAGAACTCTGCGCAGACAAGATCGTTTATGAGGCCTGTGGAACGCATTCCGAACAACTGTCGTCGGCCCAAATTTCGTGGATCTTGAAACGATAAGAAGGCGGTTATCGGGGAGAGTAGTTATGGTGCGATATTGCGTGGACGAGCATGTCTGGTGTTGTCATCGGCAGGCATGAGCGCGAACACGTGGACCTTAGACTTGTGTGCCATATGCGCCGCGCTTGACTTGCACGGCGAATTCAAATTGTCTACGAGCCTCTATTGTGGCGATGGTGTAGCCTGTCTTCTGTGTGTATAAATGTTACTCACATTGTTGGAGGCGGACGGATACTCTAGGTCATGACC

>AT12553

CTCCCACATCTCCTCAACTTCCGCATCCGTGACCCCAAACTCGTTGAGTTCCCCGCGCTCTTCCTCGTCCTCCGGGTCACGGTCGCATTCATATTCGTAGTCGAGGCTGTCGTCATCGTCCTCGCTGTCCGCGCCCGTGAGCTCGGTCGCATGGTCTGCTTCGCCATCGTCCTCCTCGCTGTCGTCGTAGCCAGGGCCATGGTCGTAGTCGTCCTCGCTATCCCCGTCTGACCTCCCATACGGGTCCTCGTAAATCCCGATCACCCACAGGTCCTTCCGCCACGGCAGCGGCACAAACGCCTCCGCGAGGATGCGCTCCTCCACTTGTCCAACCCCGAGGTCGAGCGGCTCCTCCTTGAC

>AT12554

GGGGCGTGTTTAAAGCGGCACGCCTTCCCGTCGGGGCAGCCGGTCTCGTTCCTGTTGTAATCCCTGCACGCGATGCCGTTGCCCGAGTGCGCGAAGTCCTCTGAGTCCGGCTCGTCCTCGACCGCCATCGTGACATGGTCGCGCCGTGAGCTGATGGAAATGTCCTCCGGCCATTCGAGGCTGCCCATCAAGTCGAGCCCTTTCTGCAGGTCCGCCTGCTCCTTGAGCGCCGCCGCGTTGGACTTGTCGATGGTAAGCAGCCACTT

>AT12566

CGGGCGGAGAGAGCGTTTCCAGGCGGCCGCGGGTTTCAATGGCAGTCGACTTCAGCCCGCATGCGCCCAACTGCTGGGGACCTGCGTAGCGATTAAAATAGTCCACAATCCACGAGGAGGGATGGTCGCAGGTCGAGCGAAGGTGGCGCACAGCGTAGTAGACGAGCACGTGGAAGAACGATTCGAGTTCGTCCGAAACTTGGACCGGGCGGGAAGGTTCCTGGAGCAAGTTGACGGACATGAACTGGTATGTTCCCTGACAGACGATTGAGTTGTGGTACAAAGCAGCTGAAAGTGCGGCGAACGTACCAAGCGGTAAGTCTGTGTGGTTATTGGCGGAGCGTTCTGGTCATCCACTGGCTTCGACAGCTCCCAGTCGCTGAGGATGCCCGTCCACACCAAAGAAGC

>AT12567

TATCCAATGGCAAGCAAACCTCGTCTACTACGATGCGGTAGTGAGCGTGTTGGTGCAGGGGACCGCGCATGGCTAGATCGGCGCAGTATTTGTCTTGGGGCAACGCCGAATGACGGGGATATGAATACGGGGAGTGGCGTGGAGAAGTGGACGTGGAGAGGGAAGATGCCCAGTCGCTCGTGACTGTCACCTGTTCTACGACGTCCCCATGGGAGACCAACGTCGGGACGCCCTCAATGCCGGCCAAGTTGAGACGTCGAAGGACGTCGCCCTCCCGGTCTGCAGACAGATATGACATTCGCCAAACGTCTTTGAGCCACACGAAGCGATCCGTTTGAGCGTCCAGGGCGACGTAACCGCGCGTTCCACGACCAGCCACACCGTCTGCGATGAACGCGGGCTTCCCAACGAGAAATAAACGTGTCCCAGCCTCCGAAGAAAGCTCCAGCTGGTAACGCGGCCAGTCGACGGCGAGAGAAGCGCGAAACAATGATCGCGCGTACTCCAACACAATGATCCGCCAATCTCCCCGGCCTCCAGGTCGCGCTCAGTGTGGTCGACGTCGTCAGCATTCTGTCGAGCCGCGCAGTCCATCTGCAGGAATTCCGGGATGCCTGGGAGGATACGAGTGGCGCTGATGTCGAGTCCAAGCCCAGCACTGGCGTCAAGGCGAGAGATGCGCCAGAGTAGGTCGCAGAAAACTGTGTGGTTGTCAAAGTAATCGACCGCAGGCGTGACAATGACCCCAGCACGATCCCAGTGGGTGAAGCGAACCTTGCGGCCGATAACGAAAAGCATGAAGATGGCGACGCGAAGCTGGGCAGCGAAGAGGCGTTCCACGGTGGTTGAGATTCATCTAAATGACAGGGCTTGGATGT

>AT12570

AGGAGAAAAGTTGTCTGCATCTTTGAAAGCCTTGAAAATATTTGTCGCTCTGCGTTTGCTACGGGATGTGCGCTTCAGAGAACCAAGGAAACGGCGGCGGAATTCGTCGTATGCTAGGCGGACAATGTGGTTCATGATGTCGAAGAGTTACAAGACGAAGGGAGGTGGAAGCGATAGAGGGTGTTGGACAGCGTAAGTGCAGTGGTTGAGAGGTACTCCGGCCTTGGCACACAGGCCGATGAAAGACACAATGGTGGTTGAGGTCAAGGTGAACATCCCCA

>AT12597

GCGGGGAGCTCAGCAGCGTCCTGCAAAGATAGAGAGACGAAGTCCCGTTGGGATTGGGGCAGCGGCGCGCGACGAGGACCACCAGAGAAGTGATCGCGAGCACGAAGAAGATGCCGTAGAGGACGCTTTCGACGCAGGTCAGCGCGAGGTTGACCTGGAGGACGGTTATGGAAGGCATTAGGGCGTAGCTGAAGAGGCTAGGTCAGAAAACTGC

>AT12602

TCCCATGGAGCGAGGAATTCCCGTAAAGGGCCCGTCTTTCGGCACAAGGGGCTTCGACAGGTTGAGGCTTGGGTTGGTGGGGCTGGAGAAGTACGCATTGCAGAACTCCTTGTGGTCGACGAGGACGGCAGCGTGGGCCATGTCGTCTGCGATCTATGCGCGATACTGCTTGATGTCTCCACGCGCTTCTTCGAGGGTACCG

>AT12635

CACCAGGACACGCGAGCCACGCGCCCGCCTCGTCGATGTGCACCTCCCCCGACGGGTGGCGGAACCCGAGGAAGCGCCCGGGCAAGATCGGCACGACGTCCCTCTTGTTGTTGACGCGCGTCACGGAGAGCGCCCCGCGCCGCCGCTGGGCGTCGACGTAGTCCGCAAAGTCCAGGTTGCCGACCCGCGGCAGGCCGTACCCCACGAACCGGGTCGTGATGTGGGGGATGTGCAGCGGGAGGTAGAGCGCGTCGAGGAGGCCGATCGCCG

>AT12647

CGCCGCTTACCCTTGCTGGGTCGACCGCGGCGCCTCCCATGCTCGCCCGTAGCCAGAACGGCGTCCTTCCCGAAGTCCCGTACCTTAAGGCGGCAGTTGGCGAAGTACTGGTACACCTGGCGGGCAGTATAAAAGTCGCAGCCGGGGACACGCCGGACCCTCTCCGCGAGCTCCTCCTGCGCGTCCGGGCCCGGCCAATCGCTGACGTTGGTGAAGTAGTCTGGACGTGGTGCGTCAGGGTATCAAGCACTTTGCGTGTCAGCGTGCGACGTCGTCGTGCCTCCTGAGAATGAAACGCCTACCGTTGAGAATGGCGTAGGCCTCTGGCGTCAGGCGGCGTTTGCTCATTGAAGTTATCCTCTTCTGGCCCTCCATCAGATACAACACGGATCGACGTGCGGCAACCGAGGAGCGTACTTTGTGGCGGGAACGGTGCAGACGAAGAGGCCGAGGTGCCAGTGTCGGGGTCGGATCAAGGCATAAGAGGAATGTATCTATGGAGGTGCACCTCCCGAGCGCATGGAGGCTGTTGAGTTCAGAGGGCCGCCAGCGACCTCGAGAGCGTGCAAAGGAACCGTGGGATCCCTTCGC

>AT12860

CCGCGTCTAGACGCACAGGTCCGTCCGTCCCGACGGGGCGCCTATGGCCATCTCCACGAGCGTTCAGTGCAGCGAGACGTTCGAGGCGGTCAATGGAGGAGCGACGTCGCCGGTTGCTGACAATAACAAATGGGAAATGCCTTGTATACATTACTACATGTACCCGGCACATCTAAACCATCCTTGTGCCATCTCCGGAGTAACCTTGTAGCATGCCTCGCTGATGTCGGAGACACCGAGCTCACGGTTGGCTCGACGGAGCTGGGACTTGATATATCCGAAAGCAAGCTCGATTGGGTTCCGATCAGGGGAGT

>AT12866

TCCGAGATACCCGGGGACGTCTTGTAGTAAGGATCATAATACTACTAGCTAGGCCTGCTTGATCTAGTGCTAGACAATGCTACCTCGACTGAAGGCAACCACATATCCTGGCTTCTGTCCTTCGGACGGCATTCATCAATCATCCAGATCGGGGACATGCACAACACAGCGTCTGCCATGGCGGAGGGGAAAGAAGATGCACAAAGATGCGAGAAAATAGACGGTGAGCGTGAGCATACACAGAGCCTCCGACACATATCACGGCAGGCAGAAGTACCGGAAAACAATCTCAGACCAACCGAGAGCGATCCCCAGACTCCTCGCCCGTCACAAAAATCGGGAGCTTGACAAATGGGACGGCGGCCCTCGGAAACACCCGCTGGATGAATGCAGTCATCTCGCTTGAGAGGTCCGCTTCGCGCGGGTTGAACATGACCTGCTCGACGTCGAGATCCCGCAGCGGGTGCGGCTCCGCGGGGTAATACTCCAGCTTGTCGAACGCGCCGACGGCGAGGTCCATGGCGGGGAGGCGGAGTGCGTGAAGATGGGGGCAGCAGCGTGCAAAGTGCGCGACGGACTCGAAACCCGCGCGACCGCCGTCGACGGTGGCGACGTTGATGAGCAGCTCCTCGATGTCCGGCCACGCCTCTGCAAGCCTGCACATGTCGTCTGATGTGAGATGGAGGATGAAATCCTTGAAGTCAAGCGTGACGTGGCGCAGGTAACGGAGGGGGAGCAGTGGTTGGAGGATCGCCCCGAATGCACCTTTGGTGTCCAGCGCTTCGCCGGGGAGGGTAAAAGGACTGCGAATGCTTTTATCGCAGTGGACTGACAATGTTTCTAGAGCGGAGAACCTCGTCGCGATGGTTTGGAGGCACGGAGTTGCCTCGCGAATGAGCTCCTGTGGATCCCGTTGGCGCTCCCACAAAGACAGGGAGCGAAGGTGAGGAGCTTGGACCTGTTCCAGCAGGTCACGAGCAGAGTCCCAGCCGTAGGGAGCGCACACTGACAGGCGGTGCAGGCGGGCAAAGA

>AT12867

GGGGGTTGCGGTGGAGGATGGAAAGATCGCATCTCCAAGGGTTGATGTTAAGAACGATGGTTTTGAGGTTGGGGGCTCGCGCGCAAACCTCGGAAAGGAGTCGATCTGGCTTAAAAAGCCTGTCCGCGGGTCCCTCAAATCGAATCTTCATCTCTCGTGTCGACGGAGAAATGAGGCGTACCGGGGCAGTGTGCAGACCACCCACGCCTTTTGTTGCGAGGTCTACGGACTGAAGAAAGGGGAGGATCGGATCATTAGGCATAAACTCCGAAAACTCAGCCCAGATAGGCCACGTCGTCTCTAACGGATCGCCAGAGAGCACAAGATGGATATTACGGACGCGTGTTGCATAGTCGCGAAACCGCGTCCAGTTGGGG

>AT12892

GCCGGAGTACAGCGTAGAACGTTTATCGAGAAGATCATTCGCAGCCTCGAGCGTGTCCAGCACAACGATGTTGGATCCGAGCACATTCAGGCCGATGATGTCTGAGTCTGTATGGCAGCATATGTAAGTCCACATTCCCCTATGAGGTTCGGGGTTACTTGGCCGATCACTTACTATACTCGCGGCACCAAGCGTGATACTTTAGCCATGGCTCTTCGTGCGGGGCCGGGACGTCGCGCACGTTCCCTACAAAAGGGAGTCCTGTTGGGCCGGGAAGACGGCGTCCGCGCCGCCTAGACTGAAGGAAGGACACGAGTCGACGGAAGAGTAAGAGACAGAGAACTCCAGTGACGACCTGGGCGAGAGAGGCCATCGTAATTCAAGGGCGGGATTGAGAAAGAAGGGTCGCAAAGAGGTGTGCGGTATATATACGCGATCTGGCAACTGGCGGTAACGCCGTACTATGAAATTCTAAAGGCGTCCCGCTTCTGTAAGGAAACTTCGCCGTGGGTGCTGTTGGAAGTTGTGGTCGCGTCAGCCTGAGCGTCCTCTTGGCCAGGCATTGGAACGGAAGATGACATGTTGGTACTCGGCAGAAAGAGGGGATGGTTTGTAGGAGGTGGGTGGCAGGAGTCGAATAAAGCACCCGATGGGAGCAGGGGAAGGGTACATACCTGCGTAGGACCCGCATCCGGCGCCCCGCCCCAGCGTTATTGTTACCGGCTAGAGAACCTTGCGAAGCCATAACAAATTCTATCCGGCGAAACATTTGGATGCAGGCAAACAGATAGCACTGTCCTCCTGCGCGCTCTGAATGATGTTTGAAGACCGACATAAGACGTGAGACATTCATCTAATGAAATCATCCGAGCC

>AT12897

GCCCCGTCCATACGGAAATAGCAATATCCACGTCTCAATACTGAGGCTCCGCAAGTCTTGGGCCACACCTGACCGGAAGAACTGCGCGAGCTTAGGGAAAGCTTGTGCGGTGCGGGCTCCAATGCTCAGCGCGCGAAGACTCCCGGGAGCCAAGAGGGGTTCAAAAAACCGGTAGTACTCCTCGAGGCCTTGCGTCATGTCGAGCACCAGATCCCGAATCGCGAGGGATGCTAACGGGATCGAGTGATGGATGGTCTGTTGGGAAGGTGGGCCGGTCAGACTCATTGGATCAATCCTGATGACCAAAGTGTGGACAGAAAAAAGAGACAGGAGGTCTGGGAGGATGTGTGTGACACCGGCGCAGTTGCCGAAGGACAGCCGCTGGAGCGAGTGTCGCCATTGCGCTGGCAGTTGATGTAGCAGCAGAGGGTTGACGAGGTCGTCGTAGCCCACAATGGAGACATATCGCAAATTGACGAGCCCCAGGACAGGAAGGGTTGAGGCTAATGACTGAACGTCGAACGGCCGTTGCCATGCTTGACGCCATGCGTTCGACTCAGAGCTCCAGACAAAACGCACATGTTTAACATATGCCGCGAGTCTAGGATCTGCAGAGAAAAGCTGGGACAAGCGTCCATGACCATGGGAACGGGAAGGGATGATGGCGAAGAAGTAAAGGCGAGCGACATCGTGCCAGAGCCAACAGACCCACATGCAGCTAGAGAGCGTGTTGTGGTCCGAAAATGCCAGCAGTTCGAATATAGCGTGAAACAATTCAGGTGGGAGGAGGCTGATGGACCCCGATAGTTTGTGGGCCGGTCGTGTGAGTGCTGCGAAGGCCCGAAGAATACAGCTTGGC

>AT12933

GTCGCTTGGCCAGGCGTTGAGACTTCATGTCACTATTGCTCAAACGAAGGATCAGAGTGACGAGCGAAGTTGTCGTGGTGACATCTGTCACTGCCAGAGATGCGGATAGCACCGTGGCCAAATTCTGTGAAAATATAATGAACGAGGAATCGAGGCATCGAGGTACGAACCAGGCTGAGGAAGATCGGTAGGGAGACAGGCGAATACACCCACAGCGATACTAGCGAGGATACTAGC

>AT12936

GGGAAAGACCGGTGAGCCGATGGCTGATGGTTTCCAGGATGCGGAGGAAGGTGAACATGTCCTTCCACCTCATCGCGCAGTGGACGTGCTTCAGGTTCGTGAGCTTCGGGAGCGCATTGGAGAGCATCCCCGTCTGGAACGTTATCGGGAACGTCTCCCGCCCAGGGACGAAGACTCGGAGGGTCTTGACATGGCTGGCGAAGTTGGTGTCGGTGATGATTCGGGTGAGGATGTCCGCGCTGCGCTGCGACTCTCGCTTTTCT

>AT12937

GCAAGTCGATTCGCTGGAACAACAGTTCGCTGGCAATGCGCGACAAAACGTGCGGCACATGGAGCAACGACTTGAGATCGCGAGGCGGGAGCCACAACGCTATCTCGCGCCAGATTTCTGTTGAGAATCCGGGCGTCGTATGGGGCTGAACGAGTGGCTGCGAGGTGGGCATTTGCATGGAGTCAGTAAGAGGAAGGTATGGGAAGATGGAACAACTATAACGGGATCAGGAACGCGC

>AT12946

GGATATAGTAGCTGTCGGGCGGCGACAGCCGGAGTGGGCTCAAGGCTATCGGACCCTGCGGCTGCAAGCAGATCGAGCAATTCATTCGAGTCCTTCAAACTCTCAACATACCTGCGCACTAGACGTCGGTTGTAGTGCGTTGCTCGAGACGGCGGAAATATGCGTGAGACGTACCAGATACGCAGGAGAGACCTTCCTTCTCGGCCTTTTGGCGGTTCGCGACATCATCCGTCAAAAGGACGACAAGCGGTAAGGATGGCCTCGATTGGCCCCGAACTGGCGGGCGGGCAAGGGATAGGTGGCTATTGTACCAGGAAGTTGCCTTTCGAATGCCTG

>AT12947

GAGATAGGAATACGTTGGTGTCCGGCAACACGAAATGCCCGGTGGGAAACGAAGGGTGGTCCAGAGATCCCGAGGATGGCAAGACGGCGTCCTGACTCGCGCAAAGACTGCAGCTATGTATCCCACATGCAACATCATCACGGAGGTACCGTTCGCGAAGAACTAGGCAGATCACATTGGTTACGGAAATGAACCCGAAGCGAAAGTCGGGGCGTACCCTTGATGACCTTCCCTCTCGCCGTTTTCTTGAAGAATTTACGCTGAGTGACGACGGCCTCCGCCCGCGGCCGCTTGCGAATTGAAATTTCGGGCATTCAGGCCAGTGTGAAACGGTAGTCAATCGTAAGAGCAGGCAGATGCTTTGGAAAACAAGCTTGAAGAGAAGAGGGGGAGCGACTGTGCAGGTCCAGGACAAGATCATCCGGCCCGACGGGATCGGACAGTT

>AT12965

TCGTACCTTGTAGCTCCATATCATCACCAAGAGAGCGGACGGGTTGTCGTAATTGACAACGATGTAATAGTAGACGAAATGAGCGCTCAGAAACAGATGGGCAGCGTCGAGAAACCTGCACAAGCACGCTGAGAAGGTAAAGGCATTGATCACGGACGGACTCGACACTCACAAAAGCCAGCACACGGCAAGCTTCGTCCACCACCTATCTGCCGGATATTGCAG

>AT12981

GTCCGGCGATTCATATGATATAATGCAAAAGCGTTGTCTACTTGTACAAAGCGCCTAGGGGCGTGAACCGCTTCCGATGTGGTACGCGAAACCAGAGGTTGGTTGCTGAGTGGGATCGTCTGTCAATGTTGATCGCCGGCGGGATGAAGCTCAGACCGCGGTAAAGGCGGGCCGAATACCTGCGAAACGCTAGCCATCCGCGCGAAATCAGAACCCCCTGAACTCGGGAGAGCCGACATCCATCCTTTCAAGGACTCACCATGTTCAGTGCTTCTCCCGATTTTTACGCTTCAAGTACACTGGTTCCGGGCCGTAGGGCGAGCACGAGCGATGCATCCAGGGGCTCCGAGTTGCGGAGACGTAGAGTCTGCAAAGCGCCTCTCATGGCCATCGCAAGCCCGATAACGATACCCTTGTACAGGGAAGGTAGAGGGATGTGTTGAGCGAGCGCATAGACAAGAGCATTCGTGTCAGAGGGTTTTGCGAGACAGGCGGGCTCAGCAAAAGGCGCGATGATTGTTGGGTAGGATCTTTCCCGCCTGTGGGGCCCTGTAAACGATCCCGGCCCATCGGTGAGGGCAATCTGCCTTGGCTTGTATGCTGCCG

>AT12988

ATGACGAGGACACGGTTCGCAAGAGTCTTGTTGAAAGGACCCATGTACCGCTCTACAGCACGTTCCGGCCAAAACGCGCAAGAGTATATGGGGTTCGGCCAGATCGTGCCGACTGTCAAGAGGTAGAGGCAGAGGCAGAGGCATCAACGTCCAAGTGATAAGAGACACCGCCGACAAAGCAACATACGCATGTGGGAGACGTTGTGGGCCGTGCTGACGACCGTATCAAAAACGTCCGTCATCGTCGCAGTGTTGTTCGGCGTCCTGTCTGGCGCGTCGCCGCACAAAATCGCCTGGGTTGTGTACGAGACACTGGAGTTATCCGCACGCTTCGTCAAACTAACGAGTGGCCTGTAAGGTGCCAGTGTAGGCAGTGATCTGTAATGACGACATACCTGCGCTTCGCGAGGTTGAACCGCTCCGTGATACTCCTGCTTGCCGTCGACGTCGAGTTGAGCTGCTGCGCGACTTGCGGGAGGGTCTCGTTCGTGAGGGTCATCCACGTCGACGGAACGTACATCGCCGGCC

>AT13006

CTGTGCCCAGGCGCTCCCGGCAGCTCAAGTCCGACCAAGGCTGCTCCTTGTCAAAGAAGTGTTTCTCCGCTGCACGCGCGTCTGTGGACGAGATAAGCTTGGCGCGCTCTGCGTCGTCTGGTTGGCGGGTACAGAAGTAGCCGTGCCTCGTCGTATGCTCTCGACCCATCAGGACGTCGACCCATTGCTTGCGCTTCTCCGTCGAGCCAGAAGGAATGGTATCGGG

>AT13012

GGGTCGGGAAACGATGTCGGCAAAGTGGCCAGAGTCTCGGAGGAGGTCGGGGATCTTGTGCGCGACGGGCAGGAGGCCACGGCGCTTGCGACGGGTGTCACCCACCGCCCTGTAGAACTCGGCAGTGGATGGCGCGCGGACGTTGAGATCGCTTCCGTCTAGCATGATGGGAGCGCGGGCCCACTCGTTGGAGACGAAGAGGCCGCCAGGGCGCAGGACGCGCGCTATCTCTGGAAGAAGCGCCCCGTAGTCGCAGATCT

>AT13018

ACGGGGTAGGAATGGCCGCGTTCGTCGCGAAGCCCGAGGCCAGGTCGCCCGCCAGGGTCGTGGGCGTGGTGTACCCGCTCGGCTGCGTCGCGGACGTGGTGACGATCGCGTAGCTCGTGTACGACGAGCCGCTCTTGATGCACGACAGGCCCGAGCCGTACGCGCTCTCGCACTTGTACACCGCCTCGTTCGTCTGCGACCACATGTTCACGTTGCTGAGCGTGATGCCCGAGCACGGCGCGCCGTCGGCGCAGATCAGCTGGATCGGCGGGCGCGCGACCCCGTC

>AT13187

ACAATCTGAAGACCTTGGGAGCGTAGCGGGTCTGTATGCTGTAGCCCTCGCTTTGGCGGGTGTCACCAGCAATAACGGTGAAGTCCGCACCAGCGACGGCGAGGATAGTACCGCCATTGTCGGTGTAGGGGTTGAACTGGCGCTGTTGAGGGTGGGCGAAGGAGCCGTCGGGCATGGTGAGTACGGGGTGTGCGGGGTGGATGTATGCGGATGTAGGTCGGGATGGTTCGGATGATGAGCAAAGGGGAAGACGAGAGATGGGGGGAGGCGGTGGGTGTGTATATAGCTTGA

>AT13197

CTCACATCTTCTCCCCAAACCTTCGGATCCCTATGCATGGTGTAGGTGCTAATGATGACCGCCGTGCCCTTCTCGACGGCGTACTTGCCGCCGCCAATGACGGTGTCCTCTGCAGCCCCGACCGTCCTCCCTGGGGCGGTGGGACCCAAGCGAAGACCCTCGCGCATTACGGCTAGACAATGATCGACTCGTTCAAGACATACGTTCCATAGAAAAACGTAACTATTGCTTACCGAGGAGGTACGGAAGCTTGCCAACGTCGTGGACGGTCATGGGGCGATCGCCGATCTTAGTGTCGATCTCCTCCCGAAGCTTACGGTAAGCCTCGGGGTTCTTGAGCAAGTAGTAGGTGATAAAAGTCAACATTCCTGGAA

>AT13199

ACGGTGAAAAGACCGTCGCCAGTGCCGTGGCGGACTTCATTGAGGCCGGCGGTGACCTTCTTGGGGAAGCGTTTGTCGTCGGAGACCTCGTTCACTAGCTCGTACGAGCTGATGTAGACCTTCTTTTCGCCTGAACATCCGAATGAGATCCTGTATGCGGCTACTGCAGTAAGATACTTAAGGGACTGACCGAAGACATTGAGCTGGAAGATCTCGCCATACTGCTGGGCGAGGAGATTGATCGACTTGATGGGTAGATCCTTGTCTAAAGTGTTGATGTGGCCAAGGAAGGGCACGGCGGGGGGACTGGGGATGGGAGTGGTCATGCTGGTCGAATCGTAGCTGGTTGTGTGGAGCTGTATGCAACCGACTTGGCTTTATATGAAGGGAGGCGACCAACAGAGGGGGCGCCCGATCCAGCCCCTGTCATCGAGATTGGCTTCGATAGGACGTCAAGTTCGAGAATCTGCGTGGACGGCAATGTGC

>AT13233

GGCTGGATCTGCCGGTCGAGTCGGAGAGAGCTGCGCGGCCCTGATCTGTGCGGAGAGTGTCGCGCGGGGAATCACGAAGTAGGAGGCGGACCCGCCATCAGTGCCGCCCTCGGTGCAGAAGGTGAGGACGACCATTGACGAGGAGGAGGGATCGATGCGGAAGTGCCCTCCGGGCGTCGTCGCATGTTCGCTGGTATGAACCTCATACGACAAGAGGTAATCCGCTGGGTTCTGGGCGGGCCATTCGAGTACACAGATGGGACGGTCGGGCGTGGACTCGGAGAGCTTGTGTATGTAGAGATACTGCGGGGAACGTCTGAGGGAGACGAGTAGGTACGCGTCGTCCAGCAGCGTAAACAGCACCGGCTGCTTCCCAAAGTTCATCCGCT

>AT13235

TTCAGCTGTAACGAGCAAATCTTGAGCACGATCTATGGCCCAGCTGTTTATCCATCGGTTACGATCGCCCGGAGTGCCGACAGGCATTAGCCAGCGGCGGGACTGAATTCCAGCTTGCGCAGAGCCTTGGACAAAGACTGAAAGGAAACTCTTAGAATGGACGGTATCGCCATAGAGGACCAATGTGGAGCCGTCCGCGCGCATCGACATGTTCCATTCTAGATTGCTAAATTGGCGGACATACTCCGGATGTGCTGTGAGGTCTTCGTAGTCGAAGATGCCGTTGCGGAAGTTTGAGGAGTACTGGCGGAGGCGTTGCAGTCGCTGGGACACAAGCAGAGTGCTAGACTCCCCGTCGACCATTCCGTTGAGCGCGAGCTCGATTCTATATTGCAGAGAGACATC

>AT13236

GTTCCGTGGAGGCCTCGATGTTCGCGCCGCGGCCTGTCTGCCATTATGGAGTCCTCCATTCAAGAAACAATACTGTAGAACAAGATATGGATCCAGACCGAATCTTCAAGCGGGTCCCGCCTCCACCCATCCATCTCATCGAGACTCGAGACACGGGACCAATCCTCACGTCCAGATCAGCTTGAGGGTGCGTTCCTGAGTCAGTGAGCCGGCAGTGATCATGACAGCGAGAGCGGTGGCTGAGAGCCAGAACGCCCACCTCTTTGAAGAACTTGTGGCGCGCTTGCCAGTTGGGCGGGGCGGAAGGGACGGGAAGAGAGCTTGCAGAGGCTTGAGG

>AT13270

ACGACTGGTATGGAGAATCTGTGATGAGGGTGAGGTCAATCACGACGCCGTCCTCGGCGCCATCGCCCTCGTCGCGTCGTGCGAGATCTCTTTCCTTTCCCTTCCATCGAGGGGCAGGTTGAAGTTGAGTCCGGCGTGGGACGGGCATGTGGACGGCCGGGCGCCAAGGGAAGGCAGAAAGGATGAAGGAGGCAGGAGGAGCGAGGAGAGCACGGAGAGAAAGAGCGGAGAGTGGGGACGAACGAGGGACGATGGCGGGGGATAAGTGAGAGGTTGGAGAGAAAGCAGGGACG

>AT13274

TCTGCGGCAATGGGTGTCACGCACTTGAATGAATCGAATGTCATGAGCCACACGACTGAAACTGGAAGACGTACCTCTGGTCAAGGCTGCATGTCGCGAGATGGAGTCCATAGAAGTCGTAGGAGGCGTCAGTGACCAGGTCGTTGTGCGCGTCTGGGATGAGGCCCGTTTGAATCATGTTGCACGACCCCTCCAGTCAAAAGGTGTACACCGATCGAGTTCGGAGAGAAATAAGCGGGGAGAACGTGTGGTAGACGCAAGTCGCCAACC

>AT13285

GGGGGGGGGGAGCGAGGGACGCACCGTTGGCCTCGTACGTGAACCCGAGCCGGATGAGCGCGTAGAGCGTGAACGACGAGTCGCGGATCCACGACGCGCGGTAGTCCCAGTTGCGCGTCCCGCCGATGTACTCGGGCAGGCTGAACGTCGGGCTCGCGACGACCGCGCCCGTCGGCTCGTAGATGAGCAGCTTGAGCGCGAGCGCGCTCCGGTTCACCGCCTCCTTCCACGCGCCCGTGTACGTCGAGCGCCCGATCCACGCCGTCCAGTACTTGTTCGTTTGCTGCAGG

>AT13303

ACTAGCATCGACGTCGTACGTCGCAATGTTGCTTGCGATGTGGGGCGGATCCGGCGTTATTGAATATGGCGATGATCCTTGAGGCAGCTCTATCCGGACCTTACTAGACCGGGCGACGGGGCACGCCCTCTCGATGCTGCGATCAAACGTCGAGCGAAGCGACCAGTCTGGGGAGATGTTCAGTTCAGTCGTTAGCATCCCCGAGCACATCGGAAAAGTAATATGGCCATCGTGGGCTAGAAACAATCACAGGCCGTGGAGAAACGATAAGGCTATGGGACTAACCTCGACGGTTGTCATTTGAATAACGAACCGGGTCGAATACAGCCTGGAAGGCCAGGCGTACTTCCACGCCAGCACCCTGCCGATAACGAACATGTACTCCCAGTCCGTGCCAATCGGCATCAAACAAGCGGTGGGGGTTGAGGAGACTGGCGATACCGGAAAGAGACTTGCACGGCAATAGCTTCAAGAATGGCGTCAAGTTCTCTGTACAGACGTGTTCGGAAGGGAGAGTTGCATGTCGGAGTTGATGAGGAAAGGTGAAATTGGGGAGGGAGCCCTCGGGCTGGAAGGTGAGCACTGGCGACGTTGTGCGTTGTTCATCCAAGTGGCCCAACGACGCGCAGAACAAACCGGCAAGTGCGTTGCGCAACCCTTGCCATCGCTGATCAACTCTTCACAGACGGTGAGTTGCTGTATGCTATAAAATGGAATGTCTACGCACGAGGTTGAAACTGTATCCCCCATCCAAGCCCAAAGTTCCGCGCCTGTGCTAACCCCTGGCTCGTTCGGATACCCCCACGCGTCGTAGTTCCATTTTCCAGCATTGAGGGTGAGATGGAGCTCTGTGACTGCGTATTCACGAAGGATTTGACCGAAAGCAAGCGGGAAGAGGGTGTAATGCTGCGCTATTCACCAGCTAAGCATCGGCGCGGGTGGAAAACGTGGGGAACTTGACCGGTACGCACCTTGGTCGTCGTCGTACAACATTTGTGGATCTCTCGGAGTTGCTCCCTTCAGCAGTGTCGTAAACGAGAATCGGGCTGCAAGTTTGCCATCCCGCAACGGGCGAAGCTGGAGGTCCTCGTCAAACTGTTCATGCTGTGCGAATACGGTTGCGACGTGGAAGGCCAGCAAGAAGCACACTTTTGGGCAGAGGGTCCAGCGCATAGTTCGGTAAGATGCTCTGGATATATGCGTACGATACGCCTAGGATGAGGATGGAGACATGAGACCGTAGGCGGATGGATGAGGTGGGATGAGGGCAACGTGTGCACCACTGACACCGGTTG

>AT13309

GGGTCTGGAGATACAAACATGGATCACGTACTGAATGTGCTCGCGATACCGAAGATAATGTTCATCAATAACCCAAAGTGCGCTGGGCGTTCTGGGTCATACCGGACGCGTTTTGGTATGGGGAGAAACCCAAAGTCCTTCCACTCCTCCGCAGCGGCATTCTTGAGGGCCGCCTGTGCCTCCTTCTCCTCCGGCTGGGCTGGTGGTGCTGGGAGAGTCTCGGTGGTAGACGGCGCGGCGGACTCGCGTCTACCCTCCGGGTGAAGCTTGGATGATGGGCCAGAGGACATGGTGGTAGGGAGGAGGGGAGGGCGGACACGAGGTGGGAGGGGAACCAACTTTAATCCAGAAGGCCATGATGTTTGTAAGAAGAGCTGCAGAAATAGAAAAAAGTCGCGAACGACACGAAAACCTCGAACGCGCGCTCCGATTCGGCATCCC

>AT13315

GGCAGGGTAGTCAGTGAATGCTGCTGTGAGGGCCATCGAATGGATATGTACATAGGATCGGCCCGCCTTTGGAAACTTGGTTGGGTACCACCGCGTCGATGATTCCAGAGACGTAGGGTTCGTACGCGGCAGTCCAGTCGGCTGCATCAGTCCTGAGTGTTCCGGCGATTTCGCTGGTGATCCAATGAGCGATGCCACCTGCGGTCGTTTCTGCGTTGATCTGAAGCGAAAAGGTTGGGGTTATTGAACTAGAGTTTGCCAGTGAATGGACGTCGCACATATGGTCCTGCCAGGCATACGAATCAGATATGAGGTAGTGAAATAGGGCAACACACTTACCTGGGCGAAGAACGATGAAGATGCCAGCCAGCTTCGCAGCATCGAAGATAGGCTGGAGTGCCCGCCAGTCGTTGAAGTCTACCACGCCACGCGAGGGGTTCGTCGCACCCACTGCGG

>AT13327

CGTGGGGTTCATGAGCGTATGGGCACGAGTCTGAGCGGAGAGCTGGGCAATGAAAGCGGAGAGATCGGGCGGGAGGAAGGCGATAATGGTAGAGGCGTACTCGGCGTCGGAGATCCTGACCTCGAGCGTAATGAGGGTCTCGTAGGCAAGGCGGAGACGGTCGACCTCGGCATGCAAGTTGGCACCGGACATGTAGCGCATGTTGTTGAAGTCACGGCGAAGGTTGGCACGCTGGAGGAGGGACTTGGCGCCCATCTCCGAGCAGATGCTTGACCAGATGGCGGCGACATTGCCCTTGCGCTTGTGCTTCGTGAAGACGGCCGGGGCGACCTTGAGGTTGAGGAGGTAGAGCGCGAGGTTCTCCTTCTTGTCCCATGCGGCGATGGAGGCGATCTCGAGAGCGGTGGGGTTGTTCGGAGCGGCAGGAACCGGGCGAGCGGACGTACCGTCGAAGTGAGCCCAAGCCTCCTTCTGCGTGACGGCAATCTGGAACTGGTCACGGAACAGAACAAAGTTCTTTCCGGAAGCG

>AT13329

CGGGCATGAATCGCGGCTCGCTGGGGTTGTTCGCCTCGTGGTACTGGTGGTAGTCATTTGTGATGCCCGACCAGGTACGCGGGGTCGAGCAGTTAAAACGTTGCGGGTACGCGTCCTTTGCGAATCAGGTACATACGCTGCCGATGCACAGACTCAGAGACGCACTACTCGTACTCACTAGACCGCAGATATCGACATGACCTGTGCCGTTGATGAACCCCGGGCGCATCCCGGGGTCATTGTAGGTCAGCGGGATCGCAATGCCTCCTTGTCTGGACTAGTCTTCGACCTGCTGAAAGTACTCC

>AT13343

GCACTTCGTTCTGAAACCCTTGTAGTAGTCTTCCTGCTCCGTCTGCGGGTCGGGTTTCGACTCCTGTTTCGGCGGCTTGGTTGACAGAAGATGGATGGCGTCCTCATACGCCGCGTTAATGTCCGTCTCCGCGGTAGGCACCACGACCTCGACTTCGTTCTTGTTGGCCCCAGTCTTGACTTCACCTAGATCAGTAGAGACCTTGTTGTCTCCCTTAGTACCCCATGAAACGTCGCTGCGAATGCGGGGTTACGCGACGATCAGGTCAGGATGTCTATGAC

>AT13528

GTCCGGCGTTGCTACAAGCCCGCAAGGCTATCCGCGACGTACGAAGTGGGGTTTTAAAATGACATTCGACATACCATACCTCACCAGCTTATACACCGACTGATCATCAAAACGAGACACGTATTCCATTGCATCTCCAGCTTCTCTGAACGCCATTAATCGCTCTCAAGAGTGGGCAGCGCTGAATGTTGACAGGAAGTCAGTAGGAGCAAGGCTTGGCAACAGGAAAGGAAGGATACATACCCGACCTGCCAGAGAGCGTCAGGAGAAAGGCACAGGCCCTCGGAGTGCACGGGTTGGATGGGCGGGAAGCACGCCGCGCCAGCTGCAGAGGCGACTTGGGCCTATGTAGTGGACGGACGGGTTATCAGCTAGATAGGTTGCAAGAGATACGGGGAAGAAGACGTACGAAAGAGAGCCAACAGTACTCCACGGAGTTAAGGGACAGGTTCTCGAAGAAGAAGCGGCACGTCCCGAACGTCAGCGTCTGAGCATGGTTCGCCTCGACCAGGAAAGTAGCCGCT

>AT13529

ACTGACAAAGCTCCCCGCACACGTCCGCGGTCGGGGCTGCGGCAGCGACGACGGTACTAGTAGCAGCCTGGCGCTTGTTCACAGAAGGTCCACATGAGAGCTTCGCAAACTCCACGGTCTTTCCCCCGACGTCAATAGAGGAGGTCGACACCGTCGTTGACCCGGCGCAGAAGGTGGAATCACGGGCTATGAGATTATGCTGGTCAGCAACGGGCAGCAACACGATGGACTCTCAGGCGGGAGACTGACGGAGAAGAGTGGCATAGCTCGTCGCAGCGACCGCAATAGCAGAGAAAAGCGCGGCGAAAGTAGTCTTCATCGTGACCTGTAGGTGGTGCGGCGAAGGTTAGTTGTGGTGATGAGTGCGGCTCAAGAGAGAACTGAGGTAGTGCTCTGGAGAAAGCATTCACCTTCTGTAAGTCTTATAAGCCCGGAGGCTCTCTGAACCGGCGACTTTGTTCCAGGATACGAATCGCTCCTTCCAATGTTCGGATGCTGGAGGCGTTAGATCCAGGCGTGCAGACAAGAATATCCAATGTCTAGAAAGAACCGGTCGACGTCCTCGCCCCGCCCGCCAGGTCAAGGCACGCCTG

>AT13537

GACCATGAACTTGCGACCTATGGCGATGCGCTGTTTGAGGGGGACGCGTCCCCATTTCTCCTGCGCGGCGGCGGCATTCTGGATGGACTTGTCCAACTGGGTCAGGGAAGGGTAGGTACGAATGACAAGGGGGTGCTGATTGTGTGGGATGAGGGTCGTCTGGGATGCGGGGTCTGGGTCAGCTTGAATGATGGTTGCGGCGGGATTCAAAGACATGACTCGACAGGCGAGTGCGATGGTTGTGGGAGGAAGAGATCAAGGACAGATGAGCTCTCTCGGACCAAGGATTTACTCTCGGTCA

>AT13574

GGATTTGATCATCGTCGGATACCATTCCGTCTCGTTCCAATACCCCGAGCGGTCGCCACACACTTGACCGAGTCTCACAATGGAGATCGGGATGCTATGCATGTCGGCAACGTCGCAGAGAATTCGCTCTGCGATCCACTTTGCTTCGGAGTAGCCGTTGCCTAGGGCGGATGATGGATCTAGGCATCCCTCGGGTACAGGTTGCTCGAGCGTACAATCTTTATGGTCAGCGAAATAGCATCGGAAAGAGGTCAGACGGCTTACTACGGAGCACTCCGATGGAACTGATAAATTGAATACGGGGGGGTACAACGAATGGGGAACTCAAGGATAGCTCTATGAAATTCCGGACTGCCTTGAGGTCGACCTCGTAGGAAGGAAGTGCGAGGTTGAAGTCGACCCTCCAGGC

>AT13578

TACCTGAGCGCTCTCCGCCTTCGCGACCTCCTCCCCGACGGCGACCTTCGCAGCGAGCGCGTCCCGCACGCACCGCTCCCAGGTCGGCACGAGCGCGCGCGCGGGCATGTCCAGGATCGCGAGCTCCCGCCCCATCTTCCGCGCGCCGAAGTCCGCGGCGAAGACCCTCCCGGGCTGGGTGGAGGCTGGCCGGAGCGGGGTGGCGAACGCGTGGGCGGCGCTCGCGCGGAGCGTGCT

>AT13580

CGGGCGACATCTTCTCGGTGTCGGCTGCGAATTCCCTCAACTCCTGTCCGAGCCCTACTCCTCGGCAGTTAAGCAGAACGTTCAAGATCGCTTGCGAGGCGCACGCATCATCGCTGAGCTGGTTTGCAAACCATATTGAGCGGGCGTCGGGATCGTCGAGGTCGTCCTCGGCGCTGAATTCGGGCTTCGTGCTCCCGTCTGTCTCACAGAGATAGCAGAAGATGAGGCCGTAGGGGTTGAGGTGGTCTGTAGCGAAGGCCTCCACACTGTAGAGTTCGGTCACTTCGAGCCCCAGCACGCCGAGTTTCCGGATGAGCGTAGTGAATACGCCTGTGGTAACGAACAGTTTTAATGCAGCACGGGGCTCGCCTTAGCAGGTTGGGACACCCACCAGGATCTGATTCAATGACTGCAAACGGCGCACCGACAAGGTCGTGTTGGGAACTTACATCCATATTTGTGATTTTTTGTGAACTTATATCCTGGACTAAGTCTGGTTGTGCGCCATTCAGGCTGGGTGCGATGATCAGGACCTGCTGAGGATGTCCTGCTTCGGTGCACAAGGAAAAAGCGAAGACTGCAGCTTGTAGTTGCCAGGAGAGACTG

>AT13590

TCGCACGATGAGCCATAAGAAGTAGAGGACCAGGCCCGACGCGACTAGCACTGCGAGTTGCGCCATGTTAGAAGAGACGGTAGGTGGTTGTAAATGTGATAGCAGAGTGTGAGGACATGCATGAACCACGAACCCAACTTATACGGGATGCGATAGGCGTTCAAGCACCGAAGCCACGGTCCGCTGAGGCATGACTTTTACGTGCGCGAGAAGTCCCAGAATCCGTCGGCAAGAGGTCC

>AT13594

GTGGCAGGGAAGGAGGAATTGTATAGATGGTGTAAAAGCGTAGTAGAAATAGGTATGAGGATATCGAGTACATAGGTACAAAAATGACAAAGCAAAGTGGACAGCGCGACGAGCAAGCGGCAAAGAAGATGTGATGTACAAGGATCAACATATCAAATATCAACTCGTTGAAGCTTTGCAGGCTGGGCGCCGTTGAAATCGTGATGTGCCAATATTGTGACAGCAAAGTCGTTATGGATTGAGGGGTTGTGGCTGGTCGAAGATGGACTCGTTCCAAAACATGATATCCTTCTCCTCAAAGTGTTTCCCATGTCTGTCCCAGTTACAGTTGTCTGGTCAGCGTGGGATCAGCTTGCATCATAGGTAGGGAGCGGACTCACTTAGCTGCATGTGGTAGACCATCGCGTCTGCGCTCTCCGGGAAGAAGAGTGTATCATCTTTCTTGTCATCTGGCGTGAGTGTGTCGCAGAGGCGACAATGAGGTCTCTAGGCATTGCTTGAGAAGCGCATCCACACGCTCACGAGTGGACTCATCGGCAGTGAGGCGCTTCGATGCTTCAGATGAATCTGCGCCTTCGGGAAGGTATTTGATACAGGACGCGAGGTCGTGGACGTTTAGCCAGATGGGCATGTCTCCCGCCACCTCTGACATCCTG

>AT13596

TGCATGCGTTCGGACTGTGTGATATATTCGATCTATCATGTCTGACATTTGTGTGCCTAGCGATCTCATTGCGAGCGACATTCTTGCCGTGCCATGTACTTGATCTACTTCAACATATGGCAATAGAAATCAGAGAGCTCAGTAGGACAAGAACCCCGACTTCTTCCAAGCTGCCAGCCACATCCTCGCTCGCTCTCCGTCCAGTTGCGGCAACCGCGCCAGCGACTCGGACACTTGCACCGCCTTGTCCGTTGCTATGAACACTAGGCCCATTGCCTCCCTGTCCGGCGTCATCGCCGTGCTCTGCGCCTTGTAGAATGGCAGAAGGCGGAGTGCGGGGTTGAGTCGCATCGCCTCAACTTCCTGCGCGCTGCCCTGCTCGATGCTGCCCTCGAGCGCGGAGAGCCACGTGGCGTACGGAACGAGAGGCACGTTGAGCTCCTTTGCAATCGGCTCTAGGAGAGTGGACCATGGAACCGGACGGGGATGGACGAGGTGGACGATCGGCTCCGGAGAGTGGCGCATCTCAGTGAACGCCCTCGCGGCCTCATACCCAGGGAACCACGTCACTTTCTGCAGAGAGTACAAACAGTCAGTAGGCTGAGACTGGGCGCAATGGGGGCTTGTGTTTGGACGCTACGCACCCCACTGTGCTCGGGCAGACACTTCTGGAACAAAGCGGACTTGATGAGGGACGGAAACCACTCGCGCTCGTTCCAGTAGCCGTTCCTGTCACCTGCGACTTGCCCGAGGCGCATCACGATGGTGTGCACGCCCCGTCGCTCTGCGACATTTTG

>AT13606

ACGCCGCGAAGGAGAAGAACCGGATGCACTGATAAGGCATGATCTGGAAAGTTGACAAGTTATACGCTACTCTCCTACTGGATCTGTTTCGACAAGTTGTACCGGGTAGGCTTCCTTCTGGATGTTGATAATATGGGCCTTGACTTGTGCGATATCCTGTATACCCGTATATAGTTTGACGAGGTCCAAGTCTTGTTCGCGAAGGAGCTTGAACATCTTCTCGTCCAGAGGGAATGCATTGGGGTCGCGCTTCGACGGCTCACCGCTAGCGCGGTCACTTGCTGTATCTGCCGACATAGGGAAGGAAGAGACTTAGTAGGGGAAGGGAGTGGCTAGCATGGTCGAGACTCGAGGGAGAAAAGGTATATGTGCGCTCGTCAGCGGACCCCGTGCCCCAGTGATCCCTACGTAACACATCTCCGCCGTGGTGATTTTCTGGAGATGAGAAGGACTAGCGCGGCTGCGAAAGGGCTCTCATAGGCTGGAAAAACCTGCAAAAGAGTCACCAGGTTCTACGGGGGCTGGGCTCGACCAATGAGTCCACCTATATCTGTCAGAAAATCAAACCTAGCTGGATGGAGAAGAAGAGAATTCGGACCGAGATCATGTGGGAGAATAGTGCAAGATAGTGCATGGCACGACAGGCAGGGCGTTATTAGTCGCCCAAGATAATGTTCGAAAAATCGGACCCTGTTGTAAGACTTAGCTCTATGACTTGT

>AT13608

ACGTGGTCATGAGATTGCCATTAGGGTCGAAATCAATGCCATTGATATAGGCATTGTCTGCGGACGCAGTTAGAAACCACACATGCAGTCAAACGGTGGCGCTGACTGTTTACACCCTGTCGCCTTTGGTGAGCCAGTGGAATAAGCGATTGAAGAAGAATGGCATCCTCGTACCTCAAGATATTTTCCGATCAAACTCCATCCCTGGTTTGGGGTATACTTGTACAACCAGTCATCCCCAAGCCCAGAACGTCCAACGCGCAGCTCGAATAAGAGATCGGCGCTAGCGTCTTCAACATTGGAGGTGGGGACCGAAAGAAAGCGAGGGTACGTGACATTAATGAAGTAAACGGATTTGTCCAGGGATTCAGTTCCAGGCAGTTGGTCC

>AT13610

TGATCATTAACAGGGCCCATACTTCGCAGCGCAGTTGTGTGCAGCCAGACCCGAAGTCTGCGTCTCCGCGGTGAAGAGCGAGCCGCCGTTACTCGCCGCGTACCACACGTTGCCGACCGCCCAACCGGAGCCCGTGGCGTAGTGGACGACCGCCGACTCCTGCGCCGCGATGAAGCTCTTATACTTGGATACGCGGTCGGGCGCGTTGTCGAGGTAGAACTGGAGATGCTTCATCCAGATGCCCTTGAACATTGCCTGGTCGTTGTTGCACGTCGACGTCGGATTCGCATTGTCACATGACTCCTTGAGGATGCCGTTCTGCGTGTTGTGCTTGATCGCGCCGTCGAGCGTGATCTCGGCCTGCGTGAGGTAGCTCGAGTCGCCCGTCGCGCGGTAGAGCTCGGCGAGCCCGGAGGCGACGACGCCCTGGTTGTATGTCCAGGTTGTCTGGCCGTTGTTCTTGC

>AT13611

GAGTGCTCCTGTAGTCAGCCACTTTCAACAAAGCAAGCACGATCCACTGCAACGGTTCGACATGTCAGCAGTCTTCTCCCCGCCCCTGCTTGTGCGCTTACGCCTGCGTCGTCGTTGCTACCGTTCAGCTGGGAGACCCACGCGTTGCGGTCCTGCCGCAGTGCGAGTTTGCCGATGGTCGTGGAGTCCGCGACGTTGAAGTCCGTCGAGCCCTTGGCCAACATGAG

>AT13613

GCCCCGTTCTTACAAGAATAGTCAACGCGGCCTTACAGAGCGAATTGGGTCGGCATTACAGACCAAAAGGCCCCCCAACTCATCAATCTAAAGTCTAGCCGCGTTCACTAAGCATGGGTCAAGACGGCAAGGAACATCTGCCCTACCAAAGAGCGTGTAGAAGTGCCACCACGGTTCGCGCGACGTGCCCATGTCAAACAGCCATCCAGCCGCCCTCAGACGAGGAAGGTCGATACCGGTGGCAGCAACAACAATATAGAACACTGCAGGGATGATACAGAAATCTGAACGCAAGGGATCCGGATCAGACATTCCCACCAAGGTTTGGAGGAAACATGCTCACAGATAGGGAAGATGAGCTGGTGATCCCATCTATGAGTGATTACGCGCAGGAGGGCAGCAAGACAGAACGCTGGAAGCCATAAGACAAGGTTGCCGGCGTTGAGGAAAAGGTACTTGAACGTCTCAAAGGATAGCGAAAAGTCGTCGTCGGACATTCTCGTGCCCAC

>AT13619

GCCGTTACCCCAGTATAAGCAATAAGATGACCCACCATTCCCCCTTTCTGGAATAGACCTTTGTACCGCATGGCCCACCTCATCTATCAAGTCGGGCATGATAGCTGGCAGCATCTTATTCTGCAATCTCTCCTTCACGATGGTGATGTGGTACGGGTCATCTCGCATCTTCCAGCCGACGTTGTATCTGTATTGCAGAAGCTAGAAATGTGGTAAGCCCCGTAATTACGACGCCAGGATCGTCACGTAGATCATAAGCAAGGACTGACTGTTTGGATGGCCTCGGTGAAGGACAGCTCGTCTTCAGGTCGTCGTCTGATGTCCTCAATCATCTTCGGGCCTGACACGATGACAAGCCACCGGTCCAGCAACGGGACCTTGAACGCCGAGCCATAGTACTGCCACCGATAGCCGTTTAGGTTCAGACCTTTTCACGCAAGCGAATAGGGGAACGAGTATGCGTACCTTCTTGTAACCTTCGAAAAACAGCTCCCTCAACCGCCGGGTCCAGAGAAATGCTGGGAGGTACGACAGGCCTGTCACCCAGGAACCCCCCACGGTCGGTATGTCGTCCAACTATGAGTGGACCCACTCAGCAGTAGGTCGCCTTCACCATATGCGGGGGAACCACTTACGGGGCTGATGTTCCACCGGTATACGACGATGCCAACCAGAATACCGATGCAGATGAACCCGACGGCTTGGTCGTCCGCCATCGTTGGATCGGAAGGTGCTGGCAAAAGGGACGATAACGTCTGCGAGC

>AT13630

GGCAGAGCACACAGCAATGCACCTTCCTATCATCCACACACGCAGCACCTACGAGCCACCATCCAGTTACCTCCCTGAGGTCACCAGCCTGTCTATCTGCTCCCTCCCCAGCCCCCTCTTCATCCCCGCCTCATCCAGGATCACGCGCACCACCTCCCGCCCGCCCTTCCACAGCCGCACCTTCTTCAGCTCGCCCCGGTGCGTGAGTGAGCGCTGCGCCCCAAATGACTGGCACGGGTGCCGCTCGCGCTAGTCCTCGTGGATATCTGGCCATTGCAGCCCGCCCGGTCGGACTCCTCCCTGCACACGAACAGCGCGTCCGGCAGGGCGAGGGTCGTATTCCAAATCTGCTGGACAAGGTGGCAGCCAACAGAAGCCCGTCATCTTGTCCAGTATAAGTCCTGGAGGACATGTGACACGAGATGGAGCGAGGAAAGGGGGACAAGTAGGCCTAGGGCTGCCTAGGGTGACAAGCAGGAGACCGAGCAGGACGATGAGGTCGAGATTCTGATTCA

>AT13631

TTCTGCTCCGTTCCAAACTGAGTTGAGTTGTATTGCTAGCATGCAGGTCTATTAAACGTCACAACAAATTGTCAAGAAAACGAAAGCTACGAAGGCAACGAAAAGCAAGAAAGACGACAAGTAAAACACGTCGAGAGCGGATTCATAGCAGAAAGCAAACAGCAAGGTCACGGTAAGTTAGTCGAAGGTTATGCCTTCTTGAGCACCGACTCCTCAAGAAACGCGATATCTTGTTCCCAGAGAGACTTG

>AT13637

TCACCAGCCCCATCCCCGCGCTGTTCTGTATCAGCTGCCCGCCCTGCGGCACGTTCTCCTCGCGGAAGAACCGCACCGCCTCCTGCGACACCGCGACCGCGCCCCAGAAGTTCACGTCGAACATCGCGCGCGCGGGCTCGAGGGGCACGACCTCCGCCTCCCCCGCGACTCCGTACCCCGCGTTGTTGAACACGACGTCGACGCGCCCGAACGCCGCCTTGGCCATGGCGAAGGCATCCGTGACTTGCGCGGGCTG

>AT13665

CAAATGTAAGATATATTAATATCAATATGCAACGCCCCCTGAAGCACGCACTACCATCTAGGGGTCAGAGTAACCGGCAGTGCCGGCCGCGAGGAGACGAAGTACTCCAGATAGTTGGCCTCGAACTCCCTCGGATCCCATCCGTCCTTCAACTGGAACCGGAAGCGCTGCATCAACGCGCATATAACGGTCTTCATCTCCTGCATCGCGAACCCTTTGCCTACACAGTTCATGGGCCCGTGCGAGAAGGCGAGGAACGCCGCGTCGTTGTGGTTGAACTTGAACTCAAACTTGGAGTCTGACCCGGAACACGGCTGGCCTTGAAGGCGACCTGGTAATCCGGGGGTGGTGCTGTCGAGAGATAGGTGTCCTGACCCGATGAGCCAGCGTTCGGGCCAGAATTCCGTGGGAAACGAGAAGTTCCGGGGGTCGCGGTGGAGCAC

>AT13666

GACGGCTTGTAGATAAGGCATGTCGCGATGATGCGTGGCAACATGCGCGTCTTCTTCTCGGGGATAAAACTGGTCGACTTCCTTCTGAAGCGCCGTGTACGTCCTCGGATTGACGAGGAGACAGAAGAAGAGGCTCGTGAGGGCACTCGCGACGGTGTCAGACCCTGCGACAATGACGAGCAGACCGTCTTGAACAAGCTCGTTCACAGGGGGTGGCGCGTCGGTGGGCTCGTCCTCGTTATTCTGGATGCGCAAAGTGGTCATTGGGTACCTTGAATTAGAAGAACGGATTTCGCGTACCAGAAAGTGGAATAGATCCTGTGTCTTCGAGTCTTGAGCTAAACGCCGTGTCGTGCGGGATGCTCCGTATCTAGTCAGTGCATCGAGGGACGCTACGGCGGACGCGAGAAGCCCCATCCACACCCCAATGTGAG

>AT13668

TACTCGAACGATGTCCCCGTATTTATCGTGCAGTTCCGCGAACCATCGGCTCTGCTTCCCGGTCACCGCAACGCTAGCAAGCCAGAGCTTGGAGACGCGGCACCAGAACGGCCCCGGGTAGGACGCGAGCGGGTGGACCAGCGAGAGGCGGTAGAAGGTGACAGACGCGACCAGCATTGATAGATATGTTCCGTAGCACAGGAACAGAGCCCAGGGCACGGAGAGAGGGCCGACTGATATTGAGATAGCGGTGCTGAAGACGAGACATGGCGGGACGAAAAGGAGGACAGAATGGATGATGGGGGAATACGACTCGTGGGTACGGAATACCTGATGC

>AT13687

CCATCCACTGTGAGGCCGTTGGGTTCCCTACGGATCTTGGCCCGCTGTGCGCCCTCCCATCTGTTCCGATGGCTGAAGTACAAACTGGATGTCGTGCTCGATCATGCGCCACAGCGCATGGCCGATCGTGTGGGTGCCGATGATGAACGAGCGCCTGCAGTGGAAGAGGTATGAGAGGAAGTCGTCAAAGACGTCAACGGCGGTCTTGCCTGATGGGGGCGGCACGAGGCACATGCCGTTCATCTTGAGCTTCGTGGCGTTTGGGGACAGGTCGGCGGGAACCACGCGCTGTTGATAGCACTGGCGCGCTCCGAAACCGGGCAAGTGAGCAGGCCCAGCTGCAGGCTGGCAAGCTGCGAGGATGTCACGAATCACGAAGAGACCGGAGATGATAACATTGAGGCGCGGGATGGGTGTGACCATGAATCGGTTGTTGCACAGGCAAATAGGAAAGGAAGCCAACTGACTG

>AT13616

TCGGGCGCGGCAGAGGGAGCATATCCGGTCCAGATCCAAGCGCGGGAACAGGTCGAGGAGGTACCTCGCCATGCCCTCAACCCCGTCGTCGGAGAGGTCGGGTCCGAACAAAGGTAGATCGGTCGTTCAGGCGATATGTATGGGCAAGGACAAGTGGTTGAGCGGGTGAGAGAGCGAAGGCGAGCGGGTCACTGGCGCGGGGAGGTGCGCGCAGTGCGTGTAAAGAGTGTGTATGCCATTGATCGACACGCTGGACTGAGCGGCGAACCCGCGGTCGAGGGAGAGGTGTTCGAGCTTGGGCCATGCGCATGCGAGGGCCTCGAGATCCACGTCGTCCATGCTTGCGCCTCAACGCGCCGGCGTATTGACGAGGGTAAAGGAGCGCAGGTCACGGAGGGGGAGG

>AT13846

CGCGAGACATAGAAAGGCATGGTAAAATCAGTATCGTATGCGGCTTCGGTGGTAATAGGCAAGCCGGACGGTGTGCCCGCCCTGCGTGGGGGCATACATCGACGGTGAGAGCGGGGATGTCGAACGTCTTAATTGTCGCATGGTACATTGGTACGAGACGCCAGACTGGACGTTGCCAGCGTAAATATTCACAGTTTCCAAAAATCCATTACGTGCTGTACTGGAAAGAACGTCAGAGGCGCCGCGATTTGGAAAGGTCGGTCGAAGTGCTTACAAGTACATGAGAGTGGGGAGTCCGCGTGTGCGTGAGGGTCATCATAACGTAGATAGCTACCCCGGCGTCGTCTATAGCATCCCGCCGCCGCCAGAGCTTTCGCCACAAGTCCGAGATCCGGGAGGGGGCCTACGCTCAAGGTTGTGCGTGTGTGAAAGGGGTAGTCTACCTGTTCCTGGCAGCATTGGCCTGGTTTTGGGCCTGAGCTAGGACGCCCTGCTGCTGCTCGGCGAGCACTCGACGCTTGTTAAAGTAGTCGATCTGGGGAGAGCCGTATGTCGTTGAGGTGTCAGCTCATGACGTTGAGCGATGGACATACCCAGCTTTTGGCACAGCTCCCCTCATAGGGCCCCTTAGTGTCCGCACAGGTGTTTCCCTCGTCGCCGGGTTTGACGACTTTGGCAACATCCAGACATGCGAAATACGCATCCCGACTTTCCCAGCACTTCTGACGGTCCTTTCGTGATGTTGCGTCGGGGGCCTCAGAGCTCTTGGAACCAAACCAGCCCATGATGCTGAAGATGAGGCAGACGGAGGGGACGGCAACGTGGCGGTCGATGACGAGGAGTTTGTGC

>AT13851

GCGGCTGTGTATTTCACGATACTGATTCTAGAACAAGAACCAACTACTAGAAGCGCGCCTTATTCAACGAGATACAGTCTTGACTTGGGATGTTACAAACAATATTAATTCTTCCCTCGATGCAACGGAGAAAGTATTAAATTACGACGGGTTCTATGGCAGGAGGTTGTTGCAACGCGACCACTGTGGGTTAGGCTTCCAGTCGACTACGCGCGCGGAAGCGAAGCAGTAGTGTATCAACTGGCCAGATTGCCCGGCATTCCAAAGAGTACAGTCTCGTGCCCCTGAGCTTTGGGTGGCGCTGCCCTCCGCTGTGCCTGTGCAATGGTGAAATAGGACTTGTCTCCAGGAACATCGATCGGGAAGAAGGCATGCGTTGCATCGTGCTGCAGCCGACGCAAAGGGCTGGACATGCACTGGCCGCACAAGTCATAGTCTGTTCGTGGAGAGGGTAGTATTTTACAGATGTTTTAGTAGGGAAGGTTAGCGACGTACCCTCGCACTCGAGACACTTGTGTCTGACTCCGACGACAGGATTGCAGTTGCAGCCGTCACAGCATACCCCATCGTGAGGAAGGTATTCTGGGTGGGGTGCCGCGCGTGCCCGGTTGTAGTCGTGTAAATCGTGTGGAGTCGTAATGGGGAAGAATCCGTGATTACAGCTGTGCAGCATCCTCTTTTCCGGACGGGAAATACATCCCTCGCATAAGTCG

>AT13859

ACGGGGAATGGATGTGCGGCCTCGCCAATGAGTACGAGTCGGCCCTCATCTTCTACCCAGTCCTCGAGGTCGTCGTTCTCGCGGACCGTTATTCGTGTGGCTTTGGAAGCGTTCTTCGTGATGGCATGCAGTCTATGAAGAGTGGCACGCAGACGTTTTAGCCCGAAGGCAGGCGCACAGGTCTGCACAGGCACTGACCGTGGGTCTACTGTTTCTGATATTGAGGGTACCGGCACTGATGGTGCATCGCCGTACTTGCCCTCCGCTGCCTCGTCCGGGCCGTAGAACTGAACCGCGAAATCGTTGCCCCCATG

>AT13914

GAGGTGATGGCTTCAGAGAGCTCCGAAGAGCTGGTTCTGTCGATGCAGCATGAGTCGACCCAGCCGAAGTCGAACCCGTGCTTCCGCGCGACTCTGCAGAAGGACCGGATCTTGTCGGAGAGGGATGAGTCGTCCAGGATAGACGCCCCAGGCGTGGCAGCATCAATGCGACGTTGAACATCCTGGTACGACTGCTCGGGGTTTTGCGCGTCCTTCGACCAAACGTGTGACAAGATGGCGTAGCGGACCTGACGAGGATCCTCGACCCAGTGAAACCGCCCTGTTTTCGTGTTCAAAAGGCGCATGGTGAGCTAAACGGATGTCAGGTCCAAGCTTGGAATGCAAGATTAGATAGCACCCACTGAACCATCCGCGTACTTAATGGTCCGACTCGATAGCGTCTCCCACGCAGATGTGGATATAATAGTGAGTGGCCACCAACTGTTACGCGTTGTCAATTCTACCGCGGAGGCGGGGACTGCGAATAGACAAACAAAGAACCGACTGGATGACGGACAGGGGTATCGAGAC

>AT13917

TGGCCGGAAAGCGGATCGCCGGATACCTGTGCTTCGAAGATATCAATCTCCGGCGCCGAGCGGCCGACATAGGTACCGTCGGAATGGAAGGGCCCTGGGTGCGACTCGCCGGGACAAGTGCAGCGAGAGAGCCTCTGGCCAGGAAGGTAAGAAAGCTCGTACTTGGACTCCTCGTAACCAGTAGTTGTCGCAGCGGCGGGCAAGCCATTGAGCGTTTGGTTTGGGGCTGTACCGACGTCGCAAGTGTCATAGCTGT

>AT13918

TTCGAGGCTAGCGCCATATCCTGCACGCCCAAGGTTTCCCATCGTCCAGATCGCAGGCCACAGACCGACGACGTTGTTGACGCCGGGAAGCACGACCGACGCTAGGACGATACCTCCGGTGAAGCAGAACTTGTTCCAGCTCGTCATCATGCCCCCTTGGAAGTGAAGGTCATGGGTCTCCTTGCGTTCGAGGACGATCTCGAGGGAGCCGTTGCGGGTAGCGATGGCAGCGGGGTCGTACCACTCCATGTTGTTCGTGACCCAGTAATGCAGGTCGACGGCCTCCCAGTAGGGATCGTCGCCGGGGTAGAACGAGCGGCCTTCGACGTTGAACTCGTCGCTGAAGATGAGCTGCCACGGCTTGCCTGAGATAAAGTCCGGATAGGTGTACGCGTCCTGTGGCGTATCGAGGTCGATGAGGCCCCAGTTGCCGGGTATGGAGGGAAGCTGGCCGGACGCATTGATTCCGCCCAAGCCGAAACCGTTGATGTTTGATTGACGTACACCATTGAAGTAGGACACCACAGGG

>AT13936

GGCTGCGATCTCCCTCGGTGTGCTTTAGGACTTGCGAGAGTGGGTTGAAGGGCACCGCGCATTCTGCACCAGAGATGAGACCTTGCAAAGACATCGTTGCAGTGGTGTATGATATTGAAGGCAAGACCTGGCTGGACAAGTGACGGTGACTGAAGAGAGCAAGTGCGTAAATACGCCGGAATCTGGGGTCGTGCAGTACCGGTCC

>AT13942

CTGTTTACACTTCGTGAGAACGGATCACAGATGACGTTACTGGTATAATCGTTCGGCTTCAGCTTCAACGTTTCAACTTTGGTTCAACTTCACTTCGTTTATCGCTAAACAGCAGAGACTCAATCGCCGCGCTGCGTCGACTTCAAGAACTCTTCTTCGTTCCAGCCGTAGTATTCCTTCAGCTTCTTCGCGAAATCCTTCAGTTTCATGGGATGGATCGTCGGATCCGGGTGCGCTGTCCAGACATCGTGAGACACTACGCAAGGCGCTATAAGGAACGCGGGCCAACGTACGCTTATGGA

>AT13962

CCGGGGAGATGCGATGGTGACCTTAATACGTTGCTGATAGTGACTGAAAGTCAAGATCGTCTTGTGAAGGTGACGAACGCCAAGAGCGTGCACGCCTTGACTGCATTTGGGGGGCTCAGAGAGCCTCAATGGTTGAGACATATGTTCACTGGGGAGCTTGGAGGGTCCGGATAAGTTAAACCAAGAACGCACCGCACCGATGACACACGTCTACGTATCGTGGAATGCCCTTCCGCCAATTGGACATCCGTTTAATCGAATTGGAATTACTTCTTGAGGCTAGGATGCAAAGCTAAAACATATTGGATGCGTATTGCAGAAGGTCCCGAGGGGGGGATTGGACGCTTGAACACTGAAGGAGGGGCGATGGTGCAGCTGAGAGTAAGGGACGATTCCGGCCGCGGCGACGACTGCAGCGACGACAACTACGATGAATCCGGCGACAAAGACCAGTCGGACGATAACGAAGATTAGATTGGTAATGAGCACAAAGGCAACGAAGACGACGGAGGGGATGAGGACGACGAGAAGAAGCAGGAATCTGCTGCAGGATGGCGACGAAGTTGGT

>AT13964

GCCTCGGGAGAGATGTACGTCGCCTCGGTAATGAGGAGCGTGCCGGGGACGGAGGCCCGCTGCTTATAATACTCGACGAGGAGATCGGTTGGGACATGCGCGTCGCTGGCTCGGAAGCGGGTAAGGGGGGCAAGGACGACGCGATGGCCGAGCTTTACGTCGCCGATCTGGATGGGGGAGAAGAGGGCAGGGGTGGTGGTAGTCGACATTCTTGCACACTGTTGTTGCCGGGATGAAGTGAAAGGCCCGTTAAGATCACGGCGAGCACTGTTTTGGGCTCTGGGTGGAGGATGACGGTGAGCTTTTATACAATACGAGCCCCATGTGTAGACGCCATAAAAGCATTTTGTAAAATGTGGATGACACATGTAGACTAGTGTGTCGGTGCGGATTCTGCGTAGAAGAAGAAGCAAGGAAAATCCACACGCACAGACATATTGTCATCCAAACAGGACGGGTTCTCCGAAGGCGGAGGCTGATAAATACAGGGATTTG

>AT13985

GCCGCGTAGGCTGACAGTGACACTTGTACACCCATGATGGGTCTTAGAAACTTGAGCGTAACGCTCGAGGCTTCTTGGGTACACTAGAAAGGCCCTCGACAGGTTACACTGGTCCTCCTGGTATACAAGCTGCCTGGAGTGCAATTTTCGTGTCACGTTAATTTCCCCTCAAACACAGCAACCAGAGGGCAGCAGAATGGTATGATGCAAAAGAGTAAACACAGCTTGCCGCCAGTTGAGTCCCGGAATGATGTGCTCGCCTGAATATAAGACGTTGAATAAATATTGCGAGCAGAGTGCAAATCTAACCCTTAGGCAAGCCGATGCAGCATGCCTAAAGCTCCTGGTGAGAGCCCTGGCTCTCGAGCTTGACGAGTTCGACATCGAAGACGAGCGCAGAGTTGGCAGGGATGACGCTGCCGAAGCCCCGCGAGCCTGGACAGCATAGCAATCAAGTTATGTCTCGAATAACGGAGTTCAATGGGACCCGGCATACCGTATGCCATATTGGAAGGGATGGTCAAGGTACGCTTCTCGCCCTGGCACATTCCCTTCAAGCCTTCCTCCCAACCACTGATGACCTGGTAAGCCCCG

>AT13986

TACCAACGTAGTGCATATGCAGCTTGTCCCCCTTCTGAGCGGTAACCTTGCAGTCGTCCGGCACGTAGGTGCGGTCAATCTTGAGCTCCGTGGGCTGGGGCTGGGCCGCGGCCTCCTCGGCGCTAGCACCGAGGGCGAGCACGAGCATGGAGACGCAAGCAAGAACGCGCGTGAAGATCATGGTCGTAAAACTGGGGCGTAGAGGCAGGAAGGGAGTAAAGCGGAGGGTAGGTACAGGTGGCGGGCACGGACACGGATG

>AT13990

GAGGGGTCAGGTGAGGGTGTGATGCGAGACTGTAGCGTAAATTTGGACGGACGAGTGGGTTTGGCTCGTAACACCACACGACGCAAGTAATATACATAGGTCGGCTAGCCCTAGTACGCGCAAGAATGATCATTGCTTCACCAACTACCTCAAAACGCGTGAAGGCTAGGCCGACGAATACGTTCGCTATCCTGACGATCGCTTACGACGTGAACATTTCGCCTGCTTAGGTACAAACTACCGCAATTATCTCTCTCAAGACCAACAGAGTGCATGTCCCAACGTCAGAGAAACCCACCTCAAGCACACGTAGGTATCTCACGGCATCTCGGATCGCGCATGGATCGCACGAGATGGCATCATGGAGACAACACCGAGGTTCCGGCGAAGATATGCAGATCGCCGACGACGGTCTTCGCAGATGGGTATCGGATCCGGAGATGGCCTCGAAGCAT

>AT13991

TGCGCGGGGAGGCTCTCAGGTGAGCAGGACGTTGGAGATCGATGCAGCGTGGCCGTGCGATGAACGCACGCCTGGATATCTCACTTGCGGGTCGGAGGTGTTGGTGTGATGGGGTTTGGATGGGGTGGCCTTGGAGGTATCGGGGACATTGAGATATGCATCTATCATGGCAGTCTAGCAACAGGGTATCGGTATCCAGAGAGGAGGAGTGGGGAGATATGCCAATGGTGGGGGAGAGAGTACT

>AT14015

CCGCGAACCATGCTGCAATCAGGTTGATCACGTTGTAAATCGTCTCTAGTACGAGCAAAACCTTCCGCGCGACGCTGTGTCCCGACGACAAGATCTGACCGACATGAGCAATGGCGTACGTCGCGGCGAAAAATGAGCCGTTGAGCCAGCGGCGACGCTGGCTGATGAACTCCGGGAGTGCTTCGGGAACGTCCGTCTCCGCGATCGCACTCTTGACGTACTTGAGGACCCAGTTGGAGTTGCGCTTCGCGACAAGCTCGAAACACAAAATCCGCT

>AT14024

GGGGTTAGCCATGGCGGTGCTACGTATGAAGATGTCCCCAATGACCACGCTCACCCAGCGAGACCGTTCCTCCAGGCGTTGTTTGAGGTGTCGTATCTCTCCGGGAATATCCCAGCGCTGATGTTGGAGTTCGCATGGTTGTGGACGCTTTGGATCAAAGTGTCTCGCACGGCGTTATCTGACACGAATGCGGACGTGTGCAAGGTCCAGGCTG

>AT14026

ACGTTCGACGCCTTCTGCGGGCGCAGCGTCAGGGCCGGGGGCCGCGGGATACGCGCTTCCAATATCCATCGCCGCAAATGGTTGGCCGGTCAAATTGGCCTGGGCTTCCAAGAGGGGCTGGAGCAGCGCGCCGCCGATCGACGCGTTTAGGTACAAAAACATCGGGAACGCCGCGTAAATATGTTCCACAGGGTTGACACGGCTATGCACGCCAATAAGAGGGCGAAAAGTATATACTGGGCATAAATGGGGATACGAACCGGTCCGTGCCCTGGTTCTTCATGAAGATCTTGATGTCGCCCGGAACGAGGTTGTCGTTCGAGTCGGTCCCAACCGTGAAATCGAGTGTGCTCATGGTCTGTCATGTAGCGAGCGAGACGATGTCCGAATACTGTGAGGAGACTCCAGCAGCTGCGTTGATTATCTTTTGGTCAAGAGCCACCGCGCGGTCGTGCGCCCC

>AT14172

CGCGAACTTGCCCTTGTACTCGACGCCGAAGACGTGCAGGTCGAACTCGACCCACAGCCACGCGTGCGGGTTGCACGCGTCGCCGCCCTGCACCCAGAAGCGGAGCGTCCCGTAGATCTCGGGCACGCCGATGGCCATGTTGATCCCCTGGCCACAGTGCAGGTCGCCCTTGTACGTGCCCGCCTTGACGGAGGCGAGAAGCGGGTACTTGACGTTCACGGTCACCTGGT

>AT14175

GGCGGGAGTGCCGACTGCTATTGAGCCATAGAAGCTCGAGTCTGCGCCCTGGTTGGTAAGGCTAGCAGAGGGTAAGGATAGAGTCGAAGAATGCGAAGGGTGTATGCGCACAGGTTGAAGCCGCTCGCACGCTTCTGCTGGGCGCCCCTGGTGCCGTACTTGAATTCGAGCAAGTCCTTTTGACTCTTGAGCCACTCAGTTCCATTGTGCACTGGTCTCCGTCGAAGAAGCGGTATTGAGCGGGGTATTGACGGAGCTGGCTGCGGAACGGCGAGACCCTCGGGGGCGAATAAGAGCAACAGACATGAGACAGCGAGAGAATGCGGTAGGAGGTGCATGGGCGGGGAGGCCTGAGGGAGAAGAGCGTGGGGAAGAGGAATGAGAGTTGATGAGAGAAAAGTGGGTGGCCAAGGAGGGAGCGGAGGGAGTTAAGTATAAGGCAGGAGGTGCTGCGCACAGCGCGGCAGGCCAAGGATAGAGCGGTACCGGTGCTCCACTGAGGAATGGCTCCGCGCGCACCACTATCCACGAGCTGTTGATCCCTGCTGGTTGAGTGGTATATGCGAGGCCAAAGAAGGGCGGTATCCAGCAGGGCGAGCAGGACGGGGAGGGTGGGGAGGAAGGTGAGGGGAAATAAGAACGCTCGGGGCTGCGCTCTTGGCC

>AT14176

GGGGTGTTCGCGGCGAGTCGACTGGGTTTGACGGGAGCTGCCGTCGTTCCCGCCATGTGGCATCGTGGAGGGCGCCGGAGGAGGGAACGGAGCGTATCTCATCGTGGAGGTATTCGGGTTCTGCGCGGAATATGCGGGAGAAAGAGGCCGGGCATGCTGGGGATAGTACGCTGAGGAGATGGCTGTTTGGCTAGCCGCTCCCGGGTCGGACGCCGATCGCGATATCCCAGTGGCGTAGGGATCGGCGGGTGTCGCGTAGGGGAATTGGGGATGCGCGGCGTGATACATGGTTTGGGACCCTGGCTGAATCCCAGATGCATAGGCCGCCTGAAGGCCCTGCTGGAAGCCAGACGGGTCTGGACCTGCCGAATGATAGCTAGGGACGTTGGGGTCGATTGGGTACCGTGGATAGCCATTCCGGTCGGAATATACATCGGGGTCTAGGGACGTAGTGGCGTTTTCCACGGATTCCCGCCACGAATGATTGTCACTTCCAGCGTTCCATGTTGACTGACCGGGATAGTTGCCGTGAGGG

>AT14198

CCCTCTTGATCAGCTCTTGCTCGACAGTCGGAACGGCTCCGTACATCCTCTTCCTGTGCGCGCCGATATCAATGCCAGTGCGCACGCCGAGTCCGATGTGCGTCCAGCATGCGTGAGGGGCCGCCGATCCTTGCAGGTACAAAACCAATAACTGAAAAAGGTGCGAGGTGCGTTTAGATTGTAAGATTTCCGCCGAAAATAACACGTATACTGGGGCGTACCGCGCGAATTTGAAGGTCGTACACGTGCGCGTAGGCAATGAACGATTTGTGGGTCCGTTCGACCATCTCGAACCATCTCCACCCCGCCGACCGCCTATTGTCTATCCCTTGCCACAGCACGCGCGGATCGTCACTGAAGCGGGCTCCATTGGCACAGACGAGCAGCACGGTCGCGCCGAACGCTCCGTGGTAGCGGTGTAACCTACTCTGGATGCCCTGCTTGAACGTGGGTTCGTGGAGGAGGGGAAAATAGTCGTTCATCTCCCGGAAGTAATACTCGACTAAAGTATCCATTAGATCTTGAGGAGGGAATTCCTTGAAAAGGGGTACCGCATCCTCTAGGGAAGCCCATAGCCACTAGAATGCCCGTAAATACAGTTAGCGAAGGGATAGGGGCGGCCGACTGACCGGCGAAGTAGCAAACTGCTTTCTGACAAGCCGTTTGATGTCCATAGCTGGCTGCTCGGGGTTCGTCGTGGCAGTAGAATCGCTGGCATCAGCTTCCACCTGGCCCTTCAGATGCTGTGCAGCACGGAGGAAGACTAGTCCACTCGATTTCCCGTGATAGCGATACGTCATTGGGTTGATGGACAACCTATTGAAACCTTCAACGACGTTCTTCTGCACCTCGCTCTCATCGTCGCTGGGGTCAAGGTTGTCGGACACAGCGGAGATACTTGGGCCGGCTGCCATCGGGGGACGAAGAGTTTTAGACATTGATGGCTCCGCCGGCGGTGCTGGTGGCGGGAGGGATGTTGAGGAATCGATTTCTTGGTCGGAAAGCCCGGCCTCGAGTTCCTTTTCTATGTCCACATCGGGGTTGAGC

>AT14199

TCTTGCGTCTGCAATAGTCACACGCCCGTTGGACCTTGCGCTTTTTGGCCGATGGTTGGACGTCGTCATCACGGTCTGGAGACTCGGAGGACATGTCGGGGTTGTGTGGTGCGAAGATCTGACGAAATGTTAGAAGGCACCATGGAATAACGTGAATGTGGAGTTAAGTAGTACCTATCGCAGAGATGCACAGGGACACGCACAGCTTAAACGTGTCAGAAAGGGTCACTTAAGGCAGTGCTGGCCGCAGGACTACCAGGAGATAGTCGGCGGAGTCGGAGAGCCGATTCCGGTGGATGTATGGCTAAACAGACCTGTACGGTGTCACTTTGTTAGTAACCGCCTCAAGTGTTTGAGAGACGATACGTCGATTCGGATGGGATAGGATCACAGATGGGAGCTTGAAGGGTAAAGGAACTTGGTAGCTCAAGAATCGTGGCCGCTACGAATGTGATCTACATAGCGTGGCC

>AT14212

GCCGGACTGAGAGGCCCACTCCGTCAGAGACTGATTGAACACGTCCACTGTGACGTTCCTCAAGCACGCGACTTTGTTGTCATTACCTGAACACCCCGCGCCGGCCACGATCTGATCAAAGGTGGCTTGGCAGACCGGGTTGTCGACGTCTCCGGCTGGGATGGGGGCGCCAGATTGCATGAAGGCCGCGCGAAAGAGGCCGTCGGTGTCGCCGTCGTTGTGCAGCATGTGCATCGCGACGGATATTGC

>AT14251

AGGGAGTATTGCCTACATTGAGAATCAGTATAGCACGGAATGATCAGCCGCCAGGGATGTGGCTTGTCACATCCCAGCTGCATGCCATGCTCCTAATCCCACGTAACAATGGGCTCTTCATAGGGCTGGTGAGCAAGCACTTGCATCACCTGCAACGTAGCATCTGGTGCTATCAGAGAGTCGAAGCTGGGACATATCCAGTTGACATACCTTGTCAATGCTGAACCCCATCCTCAATCCAACCCGCAAATTTGAACCCTCTCCCTCTGCTGTGCTCCTTCTGAATGACCTCCAAAATGGCCTACGTACCTCTTCCCCACCCCGGCCAGAGCTTCTCGCGCGATGTCTAGACATTGGTGTGAGATACTGTAGCTTGGGTTGAAGCCACGCAACGCAGGTAGATCAAATTGCCACTTACCATGGTATAAAGCACGTCCGGGTCAGCTGAATGTCTTGACTGCTGCCAGTGAATCAGGCGCGAGTCAACGTGGCGGCTCGTAAAAGGCGACCGAAGCGTGAATGTACTGTCACGGAGATCGATGAAACACAGCTAGCCTGAAGAAACTGAGAGGCGGGTTGTGAAAGCCAGTGAGCAGGAGAATAGCGGGGAGGGCGTCGTGACTATATGGACCTGGTGGTCGGGGTAGAGGCTT

>AT14260

GCCCGCTGAGGTCCCACACTTCCGCTATGATCGCCACGCTCTGCAGGTTAGGCAGACCGGCGTCGAGCACCACCCGCTCGAGCATACCACTACTACGGAGCAGTCGCAGGTGCATCGTGGGGAGGTCCGATCTGGTCTTGAGCATCATGCGACTGCAGAGGGTGACGGCGCGCAGTTGGTCCGCGCGGGATTTCGAGAGGAAGTCGTCGAGTCCGCGAATGAGGGGTCGGGGGTGTGCGCGGAGCGTCAGGGAGGC

>AT14265

CTGAACGCCCACGCGGGCTGCTTGTCCGAGCGCAGCGTGAGGAGCGACCGCCGGCTCGCCTGGAAGAGGAAGTCGCCCTGGAACGCGGCCAGGCGCTTGTACATCGGCGCGCGCTGGTTGTCCGTCCCCGTGCCGAACGGCGAGCCCTGCGCGGGGTCGTTGGGGTAGAGCGCGAGGAGCGGCCTGACGTCGTCGCTGGGCGCCCCGGGGAAGAAGTGTTCGCGCAGGTACTCGAGGAACTCGTCTTCGGTGCTGTTGATGGCGCGAGAAGCGGAGTGGGAGTTAGCTGTGGCAGTGGCAG

>AT14285

GTCCATCGCTGCCGTGACCGCCTCGGCAGGAACTTCACGGAGGCACTCCAGGGCGTCAGGGGCGGACGAGCATCCCGTCTCCGAGACGATGAAGTCAAACGTTGGCTGCAGCTCGGAGATATTGCCTGAGGGAAGTGCAGAGCCCGATTCCATCCACCCCGCGCGGAAGAGACCGTCGGTGTCGCCACCGTTTGAGATCATCTGGAAGGTCACCGCCTGGCTGCCTGCACTCTCCC

>AT14301

AAGGCTCCTGTAGTCCCTGAGTCCTCTCCGTCCGATCGGCACTAGGGCGCCTTTCAACGACCTTGCGGGGCATGACTGGGGTCTGGTCGTCTCCGTAGCGCTTCTTCCACAATCCCATCAGCAGCTCCACGTCGTACAACTCGGACGCGATGGGGGGCGCGGGGGTGCTGGTCTTCGCGATCGCGTCCGAGTTGTACACTGGTAGTGGCTTGAAGCGCTTGGGCTGGCGCTCCCGCGCCTTGATCGCGGCGTCATACCTCATGACGGCGTAGTGGGCCTTGAAATGGCGCAGGAGCTGTGCGATGAGGTCGTTGAGGGGACTGGGACTAGCGGAAGGGTTGGTCATGGGAGACGAGGGTTCGGGGTCTCCAAAAAAGGTGATAGGCTTCCACCCGACGAGTGGCAGATGGCCCTTGCGCACGACTTCCTCCTTCGTATAACTGCATAGAGCGCTGTCGTCTTCGCCCGTTCTCGATGAATTGAAGTAGTCCTCAATAAAGTATCGAGGGGAGCCCTCGGACAGGCTGCTGTGGAGGTACCGGACGGCGAGGTACAGGAGGACGTGGAAGAAGGACTCGAGCTCGTCCGGGATCGAGACGGGGCTGCCAGGATAATTGACTGAATTGACAGACATGTACGCCC

>AT14316

CTGGCGAGAATAGCCGAGGGCCCGAGCTTTAGCTCGTGCTCGGAGGTATCTGGGGAAACGTCAAACTCGACAGAGCGTTCGGACGATGACATGGTGGGCGTCCTCGGCGAGAAGAGAGCAAGAAAGTTGACGCTCCAGGACAAGTTCGCCAGGTACTTATATTATTTAGAGATGTACCGTAGCGAGCCCGTCGCAGACATGGTGCCTTTGTTCATATTCCGCAGCCTGCACCTGCAGTAGCATAGGTAGTCAACCGCAGACCGGCTCGGAGTGAGGAAGAGAGGACCGCCTTTGATCATGAACTCTGCCGAAGCACTACCGCAGCGTATGTGCCACGACGTATAGACATTGTGCTTCGATATGGACTTGCAGTGTTCGGACGAAGGGACTGCTCGTGGCGGATGCGATGCTCTATAGCGTGACCCCTC

>AT14315

ATGCCGGGCTACTATCGTACGTATTGAGCTGGGTCTCTCTCTATAGTATAGAATCTATACGAGTAACGTGAAATGTATCTCCATAACTATTCAGTGGATCAGTACGTGTGGGAAGTGGATTTAAGTTTGCCTCCGTACCGATCAAAGCTGCATCCCCTTCTTGCGGAAGAGGCGATTGAGTCCTTCAACCTGTGCCTGCCATACGCCCCGAGCAAGCTCGATGAGACGGTCTAGATATTCACCCAGGATCGTGTCTGCGGTCGCAATGAAATCGAGATCGCTCGGGACGATCATCCTGCCACGTCCATCCGGACTTCCGTGCCTCAGCTCGGCCAGCAGCTTTCGCAGCTCGGCCAGATAAAATTGCCGCGCCTTCGCGTATCGGACTCCACCGACTCCTTGAGCCACGACGTAGCACGTCCCGCGGCTGCTGTGCATAGAGCGTGGTTTGTGGAGGACGAGCTTGTCGGAAAGGTGGTCGAGGAGGTAGACGAGCTGTGCTGAGGGGTAGCACTCGACGTGGGATAGTCTGGTAACGATCGTTCCTCCGGGGGCGACGGACTCGAGCCCAATGATGAGTTGGGCAATAAGGAGCCTATCGCGGTAGGACCCGTGAACGGTCTTCCACTCTTCCTGCGTTGGTTCAGCCTTGAGCTGAGGATGGTGGTAGGTGCGTAGGGCGTGTGCGTCCATGAGGACAAGGGGAAAGCGTCTCAGAAATCCTCCAGGGAGATTCCGAGTGGTGTCGAACGCCATGTCTGCCAGTGCTTCTAGTGGCTCGAGGTCGTACTCAAGTATGTCTTTCTCGACGAGTTCGTAGCGGGACATGTAAGCGTCTTCAAGCAGGAAGGCGTGACCGCCTTGCGCCACTGGCAGCGAAATACCAGTTCCCCGAGCGCGTTTGTTCTTCTGAAGGACGTAGGAGCTAAATCCCCCGGGGGAGCATCTGTTGGTTTTCAGTGGAGACAATGAGTGTTCAGAAAAGGTGAGTTTGCACGGCGAGCCTGCTTACGCTACGTCGAGGAACTCAATGGGCTTCAGGACAGGAATGCAACGAGGCGAGGCGAGATCGACTTCCTTCAGCACGTTCTTCATCATCTTGAACCAAACAAATTGCACCACTTCGCCCGGGTTATCTGCGACGCTACGTTGCCATTGGAAGTGATCGTCCAATCGATCTTCATCCCATCC

>AT14520

AGGACAAGTATTATATGATACCTTTCGCTGCTGCGGTGTCAGAGTGTGCTGTACACAATATACTGAATAGGAGTCGACGAAAGAGTAGAGTAAGGGAGAGGGATTTTAAACGGGTCAGGATGGCGGAGAGCCGGTGCGAATTAAAGACATCCGTACATACAACGTTCAGCTGACGGTGGGATGGATAATATAGCCCAACTCATGTACAGGTTTGAGACTAAGGCCTGGCCCACAAGTCCGCTGGGACATCGGTCATGCACATCGCATCTTCATCTCCAGGCTGTGGTAAATCATCATCGCGAGCATCTTCTTCGTTCGCCTGGGCCGCATCCTCGTCCGCACCCATCAAATACGTGAGGTTGAGCCCAGTGCCGAACTGCGATCCAATGGACGCGATGAACGCAGGGAGCTCGGAATCACCGCCCGGGCTACTCTGGTGGCCTTGGGAAAAATTCAACGAAGGCATTGCCGACGGCGATGATGGAGTGGTTGTCGACCGGTTCGCTTGGT

>AT14550

CTTGGGAGAGTGGGAACGACTGACAGCGGAAATACGCCAGGCCAAGTCCAACGTAGTAGAATAAGGCGAGGTAGCGGTTGACAAAGAACAGAATTGAAGAGATAGCGATCTCAGGCTTGCGCTTCCAAAAGAAACGCGATTCTCGTTTGAGTGTCAGAAGATAGTCGTAACCTATCAAGGCTGCTTGAGGTAAACGCGACGGTTAGGAGGGCCTGAGTATTGCGCGTGCGGTCCACGCACCTGCGCCGCATGCCGTCATAAGGTTTCCTGAAAAGCTAATGTGTCTGAGTCGCGAACACGCGCGGACAGTACGTACGCCTACTGCACTTACTTTGCTTCTCTGATTGTTATACTGTGTGTCATTTCTGGGCGGGAAGCGAAAAGGTTAGAGCTAGAGATTGAAAGAGGAGGGCACAAGCAGGCTCAAGGGCAACCGACTTATCATGCTTCAAATGGAACTCAAACACTGCGCGATGGCCGGAACTTGCTGTCTTGCCGGTAGCTTTAGGAATCGCCGTGTCCTTGATGGTGTACCGTGGGTGCCTTGCGCACAGGGTAGGTTTGAACGA

>AT14557

GGTGCGGGGGCAAGGTGTTCGAGCTCGACGCTGCGTTGACTAGCACTTGCGTGGACGCGGACGCGGATGCAGATGCATATGCCTCGGTGCCGATGATCGTGAAGGGGGCACTGATGTTGCCGGAGGAGCCGAACACTGCGATACTCTGTGCCAATTTATCCCAGTCCTGGTTGGCTCCGACGGAGTAATCAATATTCGTGCACCAGTAATGGGACCCTTCGAATCTTGC

>AT14558

GGACAGCTCCGAAGGTTTCATGGATGCCTGGGATGTGGGGAGGAAGACCGGAGTTGGACTCCAGGGAGGTGGAATTAGACATCTTCACTGTCATATTGTGGCCTGTCAAGACATGCCGCCTGGGGGGGGGGTGGAGCGAGCAATAGTGTGCGGGGATACTTTGATGCTTATACTAAGTGCGCAATGGCAACCGAGCGGTCGGTCGGCTATAACGTCCTACGTAAGGAAGATACAGTTCAATGTCAAGACCCCAATTATCGGAATCAAATCGGAATGATGAAGATTGGAGTGCTCATGAGGCTTCATGAGGCCTGAGGCCTGGAGGGAGTGGTTGTAAGGCATCCAGCCGCGGTGATCGGTATCTGAGGTCTACCGTCTACGTTGATCGGCCAGTAAGCGTGACATGAGATTGACTTCGCCTTTATGTACCTGCTGTACTTAACCTTTATGAAGGAAGATGGGCGTCATGTCCATGGCGTTGTATATGTACAAGCTGCTGACGCCACCGTGACAATATCCTGTCCGGCGCGTTGGAAGGAGCCCGAACCTCTAAATGAAAGCCTACACAAAACAGTGGGGATCAAGAGGTCGGTGAACACAGAGATGCATATCTTACCAC

>AT14573

GACCTTGTATGGGGAACTAAAATCCCTAACGGGCGCTCGGAAGGGCTTGACCTCACTGAAAGCAGACGCGGTACCCGTGGTACCGCAATTCATAGGGTCAGGCGAATCGGGTGTCGCGAGATGGGCTACGGCATCGAGCAAACGCAAGTGGGGCGGGATCGCGCGTGCGGGTGGCCATTGTTTGTGATCAGGATGATTCAATGCTTGCGATGTCAGAGACTCGTCAACGTGAGATATGGTTAAAGAGATGATGCGCTACTTCATACCCATGATACTGCACGAGGGCACGACTGCATACACCTAGACGGTCGTCCGCGGGATGTAGAGGATGCCGGTGTTCTCGAGCAGCGCAGGGGTTGTGCCGGCGGCTCGGCTGTCCAACGAGGTTGACATACAGCTCGGGACCGAATTGGGATCACAAACGTTGGATCGGTCCGAAGGGGCTGACCATGGGGAAAGCATCCATTATCGAGTTGT

>AT14578

GCCATGCCCCTAGGTGAGCGCCTCTCCAGTCGCGAATGACTCCAGAACAAAAACGAATGTCGGGGAAGGACGCCCGCGTCGACACCCTGAAAGTACCCAACACAATTACGCGGGCAGTGATCCGCTGAACGGATAGGGAAATGTACGCGAAGCGAATCGAAGTGAGAAAGGAAAATGGGGAAAGCGTCTAGGGAACTGGCGCGTCGCACGGAACCTGCTGCCAGAGACCGCTGACCGGATCAACCAACGTGCGGAAGTTAGTCGAGGTCCCAGGAGACGGCGAGCGAGTTGGTGTTGGCGGAGCCGAGCTGCTGGA

>AT14597

GCACATGATCCTGGGACATACCGCGCCAAAGCAGACGTGATTGCCACTGGAGTCCGTCAAGTTGAGGTCAATGTTCGCGTCCAGGGGGTGAATGGGGAAGCTCTTGCCGCCAAATTTGAAGGTGACATTGACCTCCTTGTCGCACGGGAGCTGCCACACGTTACCACTGAGTTCCGAACGGCTTACGAGCTGTGCACCGGGAATGTCTTTGTAGAATGCCTCCGCTACTGCACT

>AT14626

AGCTGGAGGCTTTCGAGGACGTCAAACCAAAATGATAACGACACCAATCATGACAGCCAACGGCACAACAGCAAGGCGTCGACGGCTGAGCGCACCGAAAGGTACCCCCGAAGAAGCGACCGAGGCAAGGTTTCCCGCGTCCGAGGCCGTCGGAGAGGACAGTGGGGGAGGGGATGAGTCGGCCGTTACCAAAGCGTTGATCGGAGTCGTACTGCTAGCTA

>AT14628

ACATCACCGACGGTCCAGACGTCCGCTCCCCCTGGTCGTTCGATTGGAGGTGGAAAGTCCGCGATGAGAACCTTGTCAACCTTCGTACCGTCCCTTGGCCACGATCCAGGGCTTGCTCGTACAATGAGGATCCACGAGAGCAACGAGCATAGAGACAGAATGAGCCTGGCCATCGTATACGACGTTGCCTACGACCAGAGGAGAATCAAACAAGAACTAGAATGGCCACGCTGTCGCATGTTGCTGGCCTAGTCGTTGGTATTGGCAAATCGTGTCGGTCGCCGTGATGAAGGGC

>AT10000

GTCCAGGGGTACTTGTCCGTCCACGTCACGAGCTTTTCATATATCCACCCGAGCAGGCCGACTGGAGAGTCGGCGAGGCCGTAGCCGAGCGTCTGTGGACGGGTGGACTGCTGCATCATATATCCCCGCCCGCTGGCTACCCATTGGGACGTCTTCGCCAACCTGCGCTGAGCCTTATTGTCAAACGGCACCGTGAGGAGGGTCCTTGTGTACAGGAGTGGAGCCGTGGTGAGGGTGGGGATATT

>AT10003

CTGGGGTCCCTTGAATCCCGGCTTTTTTGGTGCTTCTGAGAAACCAAAGCCAGGAAGGTTCGGTGCGACGACATGGAAGCTGGGATGGCCCTCGCCGCTGGCCGTCAGCAGAGGAAGTATCTTCTTCACTTCGAGGAAGTGTCCGGGCCCTAGATATGCCAGTGGAGCTTTGTTAGGACACAATCTACTAAACGGACCGGCCACTCACATCCGTGAACGAATAGTAGCGGGATCGCGTTCCTGACGTCGCTCACTTGATGCACGTAGTGCACGTTCAGCGTCCCGAACCCGTCCACCTCGATGTCACGCGTGAACATCGGGAGCTTGTTGATCTCAGCCTCGGTCTTCCGCCAGTCGAAGCCTCTTTTCCAGTGAGCGGCCAGCCTCTTCATGTCAGCTAGGGGTGCGCCATAATCCCATCCCGCGCCGTCCAGCTCGTCGGGAAAACGGACGAGGTCGAGCTTCTTCTGGAGGAGCTCGATATCCGCATCTAGGACGGCGAGCGTGAAGGGGGTTTCAGTGCTGATGGTGTTCGGCATGGTGTTCGAGGGTAAAGGTGGAAGGGGATGAAGAAGACGCAGTGGCGTTACGGCGTGAACTTGAAGCGCAGAGTTGCGGGTAGGTTGTAGGTAGACTCTGGAGAGGAGGCTGAGGAGCTGAATGTATATTCATATTCAAATTGAAATTGAAATTGCGGGCTGAGGACGGGACCGCTCGGGCGTTGAGTCAGCTCTGCAGCGGTATGATGCTCTCACCGCCTGTGACTGTCAAAACGTGAACACTCGAGATGATATGAAAAAAGTGTATGCCAACTGGGGCACGTCGATGCTGAAGCAGCTGAAGCTCAGTCATTTATGTATGTCAAGGTTGTCCGAGTCGTCA

>AT10004

TTGGCCTCCTATTACTGATCATTTTGTGTCGCTTCATGATTCAGCTATGCCGATAATCTACACCCAACTTCAGTGCTGGTCCCCATATCGATCACAGCGTTGAAAGACAGCTAGCAAGACGATAGCAAGCTGCAGTCCGAGGCGTTACGCGCCGTCATACCCAGTCTTACCTGGAACGGCGCCGTACGCAGGTCCGCCCTTCGCATACATCTTGCGAAGGTCTCCCGCAAGCTTCTCCGGCACCTCAAACGCCGCGAAATGTCCCCCAGCATCATGCTCCGACTCGAACACGAGGTTGCCAATGGTGTACGTCCA

>AT10015

GCAGCTGCGGAGAGAACGAGGATGTCCAGTACCATGTCGCCACGCTCTGCTATGGAGCATCTGTACTTGCATGGGTAGAGTGTATACAAAAAAGGACAAGGTTGCTCACGACTGGCAGGGGCCATAGAAGGAACTGTCAGCCAAGCAGCTCTGCGATGTATCCGAGCATTTTAAAGGATATGTACCTGATCAGATTTACCGGGAAAGTAGGCAGTCATGAGAAGGGTGAAAGGGACAGTGGCGAGCGAGGAATAACGAATCACAAGGAACAAGATCGAGGAATTGGACGTTGAGCGGCGCCAGATGTACTTTCGCTCCCTTCCCAGTGTCAACATCCAGTCGTATATGCCCA

>AT10023

GCGCTGACCGGGACGTACCAGCTGGCGCCACTCCGCCCGATAGGACGCACACCGGGCCGGGTCGTGGACGAGTACGGCCTGTCCGACGCGAAGGGCAGAGAGACGGGCGGGGACGACGCGCCGGAAGAAAGCGACGTCGTCGGGCTCCCAGTCGCCCCGCATGAAGTCGAACCCGAGCACCTCGAGCGCAGGCAGCTCGCGCAGAAGCTCGAAGTACGGATCCTCGTCCCGGTCGTCCCCGTGAAGGACGATATCGAGCCGGCGCCGGTGCCTCTTGCCC

>AT10024

TGGGCTTGAGGTACTTGAAAGCGGTCAGGTTCGGGAGGGCGCGGGGTTGTGCGCCGAGTGCGGGGGGAAGATCTCGTCGGGATCGGTCAAATCGGTTGGAACTATGGTGAGCGACGTTAGAGCGGCGCAGTGGCGCAGGATTTCGTCGAAGCCCACGAGGCGGAGTGATATAATGAAAATTAGCTCGAGCTCGTGGAGCCCGCTGAACGACCCGCGCCTCCAGATGACGTCGCCGGAGACCGACAGACGGGTGAGCATGTCGGTTCTGATATCGAGTATGCTGCCGTAGACTTTGCCGCCTGTGTTGTGGCCATGTCCGGCAATGGTTCCCTGTCGGATCACGGAGAGTGATCGGAGCGACGGAAAGCGGTATATTTGTGGAAGAACAACGTCCA

>AT10043

GGGGTGCGTTTACGCGGCGCGGGACCGTCACACCGGCAATGGTGCTCGCAGACAAGTTCCATCCTGACGAATGCATCCTTGCAATCCTCCGACCTTGGACCTTGCCCCACGGGCGGGGTCGTGATCGTTTCTACCTCAACTTGAGCGCCATCCCTCCGAGTCTGTGTGTTCTGACAAAACCGCCGCATGATCCTCAAACACGCCAGGCTCAATCCAACGCTGCTACTAAACAGGTGCATTAGCAGTAAAAAGGGTAGCAATATTATCATTTACCGCAGCCGGGGTACTGGGGCGCATGGTGAGGCCCGCACCACCGGTGGGATCCGTCGGTGCCTCAGCAGCCTCCCACAGCTCCACCTCGAGACACACCTCCGGCCCCTCATATGCGACGACAGACTTCCAGAACGCGCTGCCTATC

>AT10046

TCCCGGAAGACCGGGTCGGGGAACGCGTGGTGGATGGAGAGGCGGGTGACCTGCGGGGGGAACGGACCGTGCTCGAGGTGCCAGAAGGTGTTGTCGTACGCCCCGGCCCAGTCCGTGTACCGGAGCGCGACGTGGCGGAGGTGCGGCAGGCACGGTTCCCAGCGCCATTCTCTGGACCTGGCGAGGGAGCGCAGGAAGGCCGTGATGGCGTCGCTCATGCTGACCGCTGCACGGGATGACGCGGGGTACGGAACGGGCTCGGGGAAGATGGTGCCCCAGGGTGCGTAGTAGGAGACGGTGTCGTGGATGTGGAACGTGACGGAGCGGCAGATGGTGTTGGTGGCCTGCATTTTCATCATCGTGAACAGGTCGGCTACCCTGCCGGGGAGTTTGGATAGACGTCGGATGAAGGTCATTCCGTCCTCGCCTGTACAGATGTGGACATCGCGGGAGGTGACGTAGGCAACAAGCGTGAACCATGTGCGGTTCACCAGCACAGTGTTCTTGACGAACGGCAATCGTTCCTGTGGTGTCTGTGGTGCGTCCCAAATGTCACTGAGAATGAGGTGGATGAGTTCCGGAGGAAGCCAGGGAATGTATGACTCGGGGGCTATTTGTGTAGCCGCAGATGTTGACGCGGTGTTTGGGGCCTCTGAGACCCCGGCGTGTTGAGTGCTGGGCCTCTGCCACTGGGCCGGTATACACACCAGCTTCGCAAGCCAGCGAAGCATTTTCGAGCACGACGGCATGAAATGCGAAAGCTAGAGACAGCAAGGAAGAAAGTATCAGTAACAGCGGTTGTGCGTTGCGAATGACGGCGGAAGCCCCTTACCACAGACAGAGGAGAGACTAGCGAGCTGGGTGCACTTGCGAAACTTCGCATGCGATAGAGCATCAGTTGTGCAATAGTTTTCCTGGAAGACCGCGGCCATCGTGTGCCCCTCGGACGCGTGGTCCATGCAGATAGCGAGGGACTGCAGGTGGGATAGGTCCTCGAACACAGAGTGGAGGCCGACTGTGTCCATTGGCGAGAGGAGGGCGAGGTCGACTAGTGCAGAGAAGGTGGACCGGGTGCTGCACGCTGTGAAGACCTCGCCGCAGATGCAGAGGTGGCGGAGTGAGTGCGCGTTTACGTCTGCGGTGACCGCCATCCGGTTGAAGCGAGAGGAGCCGGGTTGAATGT

>AT10063

TACCGTTGAAGTACATAGCAAGAAGCATGACGAAGTAACCGACCGCGAACTGTAACATGAACAAAGCCGACCGCAGGAGATGCTGCCATAACTTGAGTTGATCAATGCGGCCTCGGGAGTCGAGTCCCTTGCGACTTGCGGTATCGCTTTCCTCCTCCTCGGTACGAAGCTGAGGGGCGGACGCGGCGTTGGCGCGATGCAAGTAGCGATCGAACTCGCGTTGGAAGCGGCGGAGACCCTCGAGGGCGATGACGAGGAAGATGACCCCGATGCACGAGCCCGCGAAGGCGCCCTTACTCGTGACGTGCCAACTACTCGAGAGGAAGCAGCTGTCGACAGTGTACCAGTTC

>AT10067

TCGGGGCGAACTGAGGGTTGTTCTCGATGGGAACCTCGTCCGCTTTTGCCGCGCCGACTCGAGTACTAGTGGACTGCCAGAACGGCGAACCGAACGAGCTCCCCTCCGAGCACCAGATCGTCGCACAAAGCACAGCAATAACGATGACGAACAAGTTGACGAACACGATTGCCACGCCGTGCGTGTAGTCCCTGTCGCGGAAGACGAGGACGAGCTGTGCGATGTTGAAGAAGACGGGGAACACGAAGTTGAAAACAGCGATCCAG

>AT10069

TCCAAGCCGCAATAATGGCGAAGACGTTCAGGACGAAGACCGGACGCCGCCTGAGCTGAGAACTAGAAAAGAAGACGAGAAGAATAGCGACCGGCACAAGCAACCCAGCGAAAAAAGCGACAACAATGAGCATGTCGGTCACAGCGGCGGGGCTCGCCAATGAACCGAGCCCCTCGGGCTGCATGAGTGCCGGGGGAATAGTGGTGCTGCTCGACATGGTTGCAGGTGCAGGCGGTTCCGCGGCGGTTTGTGGCAGC

>AT10071

GCATGTCCCGCCTCCGAAGATGAGCGCAGCTTGCCCGGCGTACTTCGTCTTGCCGCCTTCCTCCCAGGGTGGAGTAAGCCGGACGGAACTGGCCCCCTCCTCGTGCGCCCAGATTCCGGTCGCCACCGTAGCGAGGCAGAGGGCGATGGTTGCGGCCTGGTCGAAAGTGATGTTGTCCGGGAACTAAACGCGACGTTGAACGGCTGAGGTAATACACGGATGATGATACCACAACACTCACCTTCGCGACG

>AT10099

TGCAAGCGCGCAACCAGCCACAAGTCTGCTCCGTTTACCTTCCTGAGCCAGATACCTTACAAGCACGTTCGCGCCGAGGGAGAAACCGAGGCCGATGAGCTTCGCCTGGGGATATCGGTGTGATATGTACATCAGCGCCTGCCGCGCATCGTCGGTGTTGCCGCAGTGGTACAGCTTCTCGCTAGTCACAGGGGTGCCTGCACATCCTCGGAAGTTGACGACGACAGCTCGGTAGCCCAGACCGCCTTCCTCAACCGGTGCACAAGCTGGTGCGAGGATGGCTCGGACGTACGCC

>AT10121

ATTTACGGGCGAAGTGTGAAAAGCTAATGGCGCATGTGCACGGAGTGGTCAACTCACCGAGGATATCGAGCCTTTTCTCTTCCCGTAAGAAGGTCGCTGCGCTCTTTTGGACCTTGGTGGGTAACGCGAGGTCAAGCTCGAGCCAGAGCACCTCGCCGTTGCCCGGCTCCAAGCCTTCGGACCGCAAACGAGCGATCGCTTGCAGCGCTCGGTCTTTACTGCGAGCTGCGAGGTACACCTTTGCACCGCGCCGTGCGAACTGCTGTACCATAGAAAAACCGATGC

>AT10122

GTGACGATCATGACCTTGCCGACAAGGTCCATGGCGTGTTCGGGTGATGGGTTGGCAGATCCAAAGCATCCGCCGACGCACTCGCCAAAAAGCGTGCAGAGGTCCATCGAGGAATTGACTGGGGGCAGTAGTAATCCAGCAAACAAGACAAGGGGATGCATCGAGTCGTCTGAAAGTTGCCGTTTATGTCGTCCGCCTAGCGTCCCCGCGCGCCGGCATGAAGAGGCGTTGCCCCTCAGCGCACTGATGCGACATATGTCTCGATG

>AT10126

GCACTTGTTAAAGCCGGACTTGTGCTTCCGGAGGTAATACACGAGCGAGCCTGTGATGATGCAGTCCGCAAGCGTCCCCGCGCCGAGCCCGATGCCGACCTGGGGCGTGATCTTTGTCGGGAGCACCTCGAGATCCTTCAGTTGGAAGCTGGCCAGAAAGGCGTCAGCATGGGCAATCGGTCAGGCGCCCGGGATGGCTTGGTCTTACATCTTCACGGTGACG

>AT10138

ACCATCGCCGGAGATCTGGAAGTACTTTGCAAGAGCCAGTTCGAGCTCCTCAACCATCACCGGAACCTGAGGGATCACGCTTCGTGTCGTAAATTTGGCTTCGACGGCATGGACGTGAGGGGCATCTTCGAGCGACGCACGTCCAAGGAACCTTTGCAGCTGGATGAGCTGAAGGGGCCCGTCAGTAGGCCGTACGTACGCAGTCGAATGAGAAGGTAAGACTTGCCTCAAGAAGCCCGTCCAAGACTGACAGCTCTTCCGCCGGACGTCTCTTGAGCTCGTCTATCATGTTTGGCCCGGACAAGATCACCACCCATTGGTCCAACAGCGCGAACTTAAATGCGGAGCCGTAGAACTGGTGTTCAGTGTCAATACAATGGCCGTGCCTGCTGACGGTGTACGGAAGATAAGTGGACTCATACCTTTCTGAATCCAATCCCTAGAGGTTCCATTGCATGGAACATGTTGTAGAATGCCGTCAGGTAGGACAGGACGGGCACGGAAGTCCCGCCAACAGTCGGGAGATATCTCAGCTGTTCGAGAAGTTAGTTCGCCGTGGCATTGCATCCTCAGCTGTACCGACTCACTGGGTCGGTGTACCAACGCGCAAAGAGAACGGCAATAAAAGCGAGGGCAAGGGCAGATGAGATAGCCTGCGGGTCCTCCACCATTGCCAGTACGAATTGATGTGTTTATCCAGATGCGCCTGGATGCCGTCGAACGATTATAAGCAATGTTGCCGTGAGAGAAGGAAGCCGGCCTAGGAGAATGGTCTACTCACCAACCGCATGAGTCGACGGGCAGGGCTTCAGGGGCTCTAACAGTGAGATGTCCGTTGCCGGCGTAGCGTCCGATATCACACTATCCTTGCAAAGGAAAACATGATGATTGTCAGCGAGACTTAGTGGGCATCGGAAAGGTTCTCCGCTACATACAAGCGTATAGGATCGATACGAACAGTAGCGTTCCCCGGAGCACTGCACCGCCAAGCCCGCGCCGACGCCGGAGGAACTGTGGACTGAAGATCGAGACCGCTAGAGGTGGGCGACGGGTATGCGTGGTCTCCTGGGAGCGGCAGGTCAGCACGGCGCTGTCCAATGCAATGGCAGCCCGGC

>AT10145

TGCTCGGACTACTATCATAGATGGGCACAATAAATTGGGAGTTCCTGGTAGTTTATCGCTAACACGGGATGAACGTTTTGGGGTAAAGCACGTAACGTCAAGGCGAGGGATTTGGCCATCACAAAGACTGCGACAACCACGCTGACCTGCGTCTCCTGAAAAGCACTACGCCTTCGCGGGATGGATGAACAGAGAACCCCATGCAAGTTTCTTTCGGCCGTCTGCCATCACCGCCGAGTTTCAAGTCGTAGTGGACGATAATGTTGGCCAGAATCACCTTCATCTCCATCACTGCGAAGAACCGTCCAGGGCTATGGGGCATGCTAGTTAGTCTCACCTTAGTGCGGCTGAAGAGAGCAGTTCGAATGACGTACCATGCATGCGGACCATATCCCCAAGGGAGGTACGTCGCTGAAGCGGTCGTGTACTGGTGCTTCACACTGTCCCCATCTTGCTCGCGCATGCGAGAGAACCGGAACGGATCGACCACCTCCGGGTCTGGGTAGTACTTCTCGTCGTGGTGCATTGGCCCATAGGCGGCGACGAGGACGGTCCCTTTCGGTATGAAAGTCCCGTCGCTCAGCCGCATGTCCTTCATCGCCTTCTTCATGAGAGACGCTGTACAATAGCGACATGTCGACATTAGCGTGTGGCTCAGATGCGACAACTCGATAGAATGCATACTAAGTCCAATACCCGTGTATCGTTGGGATTCCTTCATGAAGCTGTCGAGCTTGGCCATTTTGCTGATCGCCCCGGGTGTCCAACCCTCCGAAGAGATCAGTGCCTCGATCTCATCACGGAGGGCTTCGTGGGTCTCCGGCATGGCTGCTAGGTGGTACAGGACGTGTGTAAGTGTCTAATCAAAAAGAGGCCAGTCAGAAGTAGCACGATGTGTAGAAGGCGGTCTCATAACATACGTTTGTAGTTGAGTTGATGGCCGCGAAGTTTGATCCCAAGAGGCGTTCGAGTACGTATTCATTCGACTTGCCTTTGGGAACTCCTCTATCCAGAATCCACTGCAGCATGTCGTTCTGAAAAAAAGTTAAGACGAATGCAGGATGTCATTTCAAGTGCGAGTGCTTACCTGTTTGCCGGGCCACTCTTCTCCGTACTCCTCTATTAAGCCCTTCCGCTCTTCAATGACAGGGCCGAGAACAGACAGGCCGGTGACCACGTTCTCTGGCACTTTGCTCACCCTCGGTCCAATTATACT

>AT10146

CAAAGAGGGAGCCCAACGAATATGCGACTGGTACTTCGTGCAGTCATGTCTCGTAAGAGATCCCAAGCATTCATAGCAACCCAATCTGCTCGGCTCGAGCTGTCAGCTATGGCATCCAGATCGAAAGTGAGGCTACGTACCGCCCTTTCCAGCAGAGATAAACTCCTTTAGCGCACACTGTACTTCTTCCGCTACCATCGGCGCAAAGCCGGGGATACCGCGAGTAAGGTTCTCTTTGATGATGGTCGATGATTCGTGACCCGTGTCGGCAAGCATCTCTGGCCCCGCCGTATATGCAGTCTGAGTGAACTGCGACTGAAAAGTCACGCGGGTGTTCCGCTGAGAGAGAGAACCGAAGACAAACCTCTCGAATCCCATGGTATGTCGAAAGCTCATCATCGGGACGCTTCTTGACATCCTCGATCATGTTCGGCCCGCAAACGACGACGACCCACTTGTCCAAAAGAGGTATTTTGAACGC

>AT10151

GTTGGACCACATCCTCAGGTACGTAGATATCATCGAAGAAACATAACCATATTCCGCAGAATAGACCAGGTGAATGCACATAGCTCTACTCCAGGTCAGGACGCAAAATGTGCTGTTGGCGCAGACGCACAGGATGCGAATTTGCTGCCATATATACATTGCCTGTTCTCTAGCTTAACCCTTATCAATCGATGACGGTCGACTCGTTTATTCCCCGAGATCGGCCGCAATAGAGTTGACGAACGATATTTGCTGCGCGCGGTAGTTGTCCGGAATGACGGCAAACGTCGCCTCGGACAAGTTGGACGTCAGCGCCGTAGTATCTAACTGAATCAGGAGCT

>AT10154

GCGCGCATTGTTCAAGATCCTTCTTCGTACCTAATGTCATCGTCGCTCAGGCTAAGCCCTTATGATAGGGGGGAAAACTGCGAGGGCCCAATGGTTCTCAGAGGAAGTACTAGCGGCGTGGCCGTATTTGTTGACACTGAAGGAAGGATCAGCGGCTTGTATTGAGTAAGAAGGGCAATAAAAGTAGACCATCTACCCCAAGCCGAACATATTTCTGCGTATGAGGTCCCTTTCCTCACCCACGTCGCTGACTGGGAGGACTAAGGGGTACATGCTCATGGAAGGCTAGCATGATATGTGTGGTACAGACGCTTATCTTCCCACTTCCAGCTTGTCACTTACTGAACAACCCGAGCCAGAGAATGACATAGATTCGACATGATCAGAAACGTGTCTAGGGGTCGTCTACCTGACCCGTCCGTAGCCGTCCGTCCACAAACGAAGTGGCACGCATTCTCACTCTCGCACACCTGTGGACATGAAACGTCGCGCGACTGGTTGCTGAGGTGCTCGTGATCCGTATAGGCTCCTTCGCGGCTATGTTAACCCCGACACGACTAGCACACGCCGTCCATAAGCCCCGATCAAGGCACCGCTGAGGACCAAGACGAGCGCCAACATGCATTTGTCCACGTCGACGCTAATAAACTGATCAACAGGACGACGCACCGTTGCAACAATCCCGCGCATACACCCACTAAAGATCCTCCCATACCATAGTCATCCTTCCCGGGAAGGTCTTCTGTGGGTCCGTTCCTCCTCGAGGTGCCCAATGCGCGGTTGTTGAGGTTGGCCCATGGAGTGGACTCGGAATCGATAACGGAGAGCCCCATCAATATCGCGAGTCCTAGCCAAGTCACGATCTGATACGCATGTCAGGAAGACTGAGAAACCTAGGTGCGACGTGACAGCAGCAGGGAATGCATGACCTAGACATAACGCAGAGTTGGGAAGGGAGGTGGTGGAGAAGAGACAAGTGTGTGCGTTG

>AT10159

TCCGCTACGTCACCGGCGATTACATGGATATTCTTAAGGGTTGCGGCGGTGCCCTTGAGGTGCGTGGATCCTTCAGGATTACGGACGATCGCAAAGACGACGGTGTCGGGTCGACTAGCCTGTGTAACAAGATTTGATCAGTCGCGTGTCAAATGTAGCAACAACGTCGGGACATACTAGCTGACGGACATACTCGAGGCCAATGCCACGAGAGGCACCAACTATGGCGTAAGAGAGCATACTGTACAGGGTCAAAGCGACAATACGGCTAGCCCTCTGTACAGACGAGATTGTCAGCTATAAGTATGGGGTGAGTCTGTGCGTCTTGACGTACTTATATACGCTGTTCGGGCCGAACGAATCTATGTCACCAGTGGCGTGTATGCCACTGCAGTCAGATTCAGTGTGAGTCAGCCTCCTATCGCCGTGTAAGGCTCCCAGAAGAAGCGCGGTCTATAGAGCGATCGGGAATTGTGCAGGGCGGCTATAGCCTCCAGTGGCTCCCACGCGGTAGTTGAGCGATTCTCACACACTGCAAGGAAATGAAGGTAAACCAGTCGCCTAATGG

>AT10165

TTCCAGCGTGCTGTAATCTTCTACATCGCCTGCGATTACATGGACGTTCTTGAGGCTCGCAGTAGCGGTGTGTAGGTGGGGCGATCCTGCTGGGTTTCGCACGATGGCGAAAACAACAGTGTCCGATCGACTGGCCTAGGGAAGGGGGGAAAATTTGTAGAGAATCGAATGCAGCAATGAAGTCGTGGCCTACCAGCTGTCGAACATACTCAAGGCCGATGCCACGAGACGCACCAACTACTGCGTAGGAGGGCATGTCGATTTCAGAGGGGAAAGTGACGGACAAGGTGGAGGAAGGTGTGAGGCTATCA

>AT10175

TGCTCTCACATCTCGCGCCTGAAGCTCCCCCCTGCACGCGGAGCAAACATTGCGAAAGATGGCCTTGGTGTGGATAGAATCCACCGAGCTACGCAACGCAGCGCGGCCTACCACCGATGACAGCTTGCCCTCGACCATCGATAACACTAAATCCTTCAACCCAGCGCAACACTCGCCAGGATCTGGGCACCTCCTACTTGCCTGAACCGTGAGGATCTCGCACAATATGTCGGCCCGCGCCTTGCTGAGTTTCTCCTGGGCGGTATAACACCGTTTGAGGTCATCGGAGGAAAGGTGCTCAACGCACCCATCTTCTCGCTTCCAGCCGTCCATGAGATCGGCGCCCAGCATGCAGCAGGAATAGAGGGCGACGGGTAGTAGAGATGGGGTATCGGTCAGTCGTGCAAT

>AT10176

CGCCCGTCGTCCCGCTACATCCCTATCCCTCACATCGTTCCAGCACGCGTCGCACAGTTCGTATCCACCAAGCTGTTCGAAGACGTGTTTCCAAGTGTCAATGACCATGGATCCATCTGAAGCCTCCTTCGCGTGGACCTTCAACACGTGCGCAAGCATCCGTCGCAGAGCGTCCTCGCAACACGACCTCTGTTTGCACCTCTCGCTCGATTCCGGGCGGAAGATGGCGTAAACCGAGGCCACGCTAGAGTGGGAGAGCTCGGAGCTCGCGGCGATGCAGCGCTTCACGTCGTCCTGAGAGAGGTGCTCCATTGACCCGT

>AT10187

GGGGCAGGTGAGGGAGAACCGCGCGATGTTGTGGAAGGCGAGGAGGGGCGCGAGGAGGTCGACGAGCCACTCGCACATGCGCGGGCCGCCGTCCTCGTCGAACTCGATGGTGATGTCCGTCAGGGAGGCAGCGGAGGGGACGGTCGCGAGCGCCTCGATGAGGGGGACGTAGTCGACGTAGCCGTGCGCTAGTGTGCGTGGGGGGCCGTCCCACATGGCTTGTACCGTCGCGGACGCGAGTGAGGGGGCGTCGAGCGCGAC

>AT10205

TGCGCTTGCACCGATCAAAATCGTATCACGCCTCGACCTCGCGCTCCCTTGAGGCAGCCTCTTGGAAAGAAGTTGCCACAGGATGATCACGTAGAGGGCGACGAAGCAACCACATAGCAGAGTCTCTGCGAAGCAGCATCCCAAAGCGAGGTGGACGGCCGAGCTCATCCCTGCGATCTGAAATGACGGTGACTTAGTGTCTATCTCGAGAACGAGTATTTGTGACCAATGCATACCTAAGCGAGTCACTGCTAGCTCACCGGAGCTTCTCAGCACCCAGACGAGGTCGAGGGCTGTCTTATCGGGGTGTCACCAGAGGTGAGATTGAGCGGGAAAGTCGGGAGCGGGGCAAGACCTCGCTCATATCTCTAGGCAGCCTCTGTGCTGATATTCCCTCTCATCGCGACCTGTATGCTGTGCGCAGCATTTTGGATCATCTTGGCGGCACGAAGAAGCAGCCTTCATCGAGATAACAAGTCTAACTCCTCCGCCTGTGAGATCCGACTTCGAGGCGTGAGCACATTCGTGGCTAAATAAAACGGGGCGGTGCGAAAGTTCAGCGATGAAATTGTTCTCGCATCGCCTGTCGAGAGAAATGCTCACCATAAATGGCAAGGAGAATACGCGACTTCTGAAGAATGAGCGCGTTATGTGGCATTGGATGCGAGGAGGTCGAGGGAATGCGTGATGGAGTGCCCCACATGGCAATACATTTGGACGCCTTCAAGCGCTTCTGTGGTAAGTCATCACTGGGAGGCCTGAGCCTCGCCCGTGAGAGACCGCGTTCCGGGACGTTAAGATGCCGGAAGAATATCATGCTCGGCCCTGGGGGTGGGACGACCGGGCGTCCTTCGTCCAAGCCGTCTCGAGGCGTCAACGAAGAGACGCCGCGCTGTCTTCTAAACCTGAGACAATTTTGGATAAGTGCGTTGGCATAGGCGACCGACAGTCAAGGCAGGAGTGCGTACCCGGCGTCTTGACTCTGGTACACGCCGGTCGTCAGTTTCAGCGGCAACTCGGTAATGCAAGCACCGACCTATCCAAACGCAATCTTTAGGGATCAGAAGCTTGGTCAGAGTCTTTGCTTTCTTCACAGAACCAGCATCGCCTTCCAACCGTTCTATAAGTAGTGTTGGCCGTGGTGCCATATCTGTGCGTCCAAAAACGAGGGAACATTGCCTTTCGGCTTCTTCTTGATGAACCTGCC

>AT10207

GGTGGAGGGGGATACTGCTGTATCGCCTTCGAACCAAGATGGGGAATGTAGCCGAAGGTGCTGCGAAATTCTCTGGGTGAGTCTGCGAGAAGGTACTGCCAGGAATTGACATGGTCGACGTGCCCGGCGCGGTTGCCAAACACGAGACCCCTGTCAACAATAATGTCCTGACTGACGCAGCACCCAAACGCAAAGAGACTCGTGTCACACTCCTGATGCTGCATGATCTCATATTGGAGTCGAATGAACGCCCTCTTGCCCTCGCCATAGTTCGTCGGCATGCTGACCCCGAACAGACCCATCAGGCAGTATGCCTCGTCCTCTACCCTTGTCGTCTTGCGGCTCGACGCCCAGGACATGCGGTTGGAGGCTGACCACTCTGCTGGTTTGTCCATCCCCTTTAGGACCCCAGTGGGGACTCGAGTGATCTCCTTCAGCAGCTCAGCGAGCTCGGCTCTGTTTCCCAACTCGTGCCAGTTGGTGGATAGGAAGATGACACAATCGGGGGCGATAAGTTCCTGAAGCGTCCACCCGCGCTCGTGCCATCGGCTCTTCCGAAACGCGGACTCGGCCGCTTCCAAGACGTCGTCACTGGGAACGTCCTTGAGGTAAGCGTAGCATACCTCGGAGGCCTTGTACCACTGGTACATCGAGTTGATGGCCTCCGACAGCTCGCTGCTGCTCGTCTTGTCGATACAGCATGAGTCGATCCAGACCCAACGGTACCCCTTCTTTTCGGCGAGGATGCAGCACTCGCGGATCTTCGAGGAGAGCGCCGGATCGTCACGGGGGTTTGCACCCTTCGCTCGGCATCGCTCCGCGATGGCGCGGACCTCTTGAAAGGTCTGCTCGCCGTCGTCGTCCCAGGTGTGAGAGAGGATGGCGTATCCTCCAACGGCATCGAAGCTCCTAGCGAAGTGGTGTAACTCGGCGCGGTCGGTGCGGAGCAACCACATGGCTGTGCAGTGTTCTATGCGAAGAGAATCAGCCGTTGTCCGCAAAGTAAATGGTCAAGAGCTTGACGTACTGGGCGATAATTCCCACTAACGAGGAGGAGCGGAGTAGTAGAAAGGGCAGCGGGGTAATTGGTGGCGATGGGAGAGAGGAAAGTGGGGTATTGGCGTGGTCGGTGAGAGGAGACAGCGGGCTGTGGGCGTCGAAGGTGAGCTAGGCAAAGTAAATAAAGAGAATGTGTGTCTTCGGCCACCATGCAGTGTCATATTAAGGTTCAACCACCGGAGGCGGTGCGATGCACATAACACACCAGGTCTCTCCGGGGGTAAGCAGGACGCGCTGTCAGAATAACGCTCTGGCCGAAGTGAAGGATGCAGTGCCATCGGAGGCCCTGAGTAGCCTGCGTCTGTTCATGCTCCTGATATGCTTGATGAATATCTTGTCCCGAGCTGGACTGTGTGGAGCATGC

>AT10209

GTGTCATAAGAATGTACTATGCTAGCTCCGGACTCCGATCAAGCGAAGCCGTGAGCGGCATATAAACTGTGTCGGGCATGGGTGGCCTGCCTTGCGACTGCCTTGCGTACGGAGTGTATAGTCGGTTCGTATTGTTGGATGAATTGTGACTTGGGTCGATAACAGCGAGAGAAAAGGCGATTCCCTAAGGGACGGTGACATCGGCGAAGTCCCCATGTCAATGATGCACAACGCATTGCCACGTACCATGAGCGGGGAGAGAGCACACATGTTCGCGTAGAACAGGATATTCGTATCCATGAGAAATGTCACAGGGAGCGAGAACGACGCTACGCTGTACACCGCGGCGGACTGGATTGCGGCGTCGCAAAATTCCTGTGATCGGAGCAAGTTGACTACTGAGCGCGACCGCCACTCCCAGTTCTTGACGATGTCGGAGTCGCGAATATGGGCTTGCATCATGGGTCCTGCGTGGTCGAGGTAAAGATGATTAGCAT

>AT10219

GACCTGGGGAAGAGGATACGAAGCAAGTGCGTGAGGTCTTGAGGAGAGTCGGTGAGGCGCACGATCGGGCATCCCTCGAAACGTTCATCGGCATCCACGCACGCCACCGCGAATATGTCAGCGAAGACCGTCGACTGGTAAGAAAGGAGGCAACGGTAGACGCGGAATGCCACGTCTCGAGCAACGAGGATGAGATTCCCGTCAGGGAACCAGAATTCATTGTGGGGGTAAATACCCTCAAGCGGGTAGAGCAATGATGTCCT

>AT10236

CACCGGCCGGCTGGATGGACAGAGACAAGTGCGGAGCACATGTCGCAGGTCCTCGGGCGAGTCGGACACATGCACAACAGGGCACGAATATGTTTGCTTTTGATCAATGCGAACGTTGCGTACCACCTGGCAGTCGGCAAACAACTGCTTGACAGAATACCGGAGAAATGGACGCCAGGAGGCCCTTGTAGAAGCGAAATTCGACATCGCATGCGGCGATAAGTATGACCGTACCGTCCTCGAACCAAAACTCCTCATCTCTTTTGAAAACCTGTCCATCAACCACC

>AT10255

CACCGACGTCGCAAGCACCTCGTACTCGCTGACCGCGTCGCGCAGCCAGTTCTCGCCTCGCCGACGGTTGGTGGGCAACAAACTCCGGTCGCGCTCGTACACATCCTTGACGCCAGGTATCCGCACCAGGCCATTACCGCACTCGAGTTTGGTCTCGATGACGCCTTGCTTCTGTGGGAACGCAGACGCATCACCGACGACGAGGGCGAGATATGTCTTCTCAATTTGTCTCCCCGTCATCTGGTTTGAGAAATCGCGTGCGCTTTGTCGGTGGC

>AT10256

TTGTGCGTCAACGTTCGGTTCCTTCTGCTTGTTCTGGGGATCCGAAAGTTGTGAGATGAGCCCATTCGGCTTGTTGACCACGACAAGACCACGGTCAGCATATAGCAGGAGCTTCTCTGCTGGTAGCCGGTATGTCGGTTTGGGTAAGGCTGGTGTCAACATTGTCGGCGCATTTGAAGATGCTTGAGAGACTGTTGACGCATAACTAGATCGTTTTAGCCTCCGAGTCGATGACGCTATCTTCCTGCCTAGCTCGGCTGGCATCGGTTTATTGAGGAGTTTTTGGTGGGAGCGAACTTGAACGCCGATAGAAGGAGTTAGGATCAGTTAACGCACTGCTACGGCGCGCGAGGAATGACCTGAAAACGACATTGGATGAATATGTGTGATGATTCTCAACAGCGTACGCACCAGCTGAAGGAAAACCTGACGTCGCGGTCGAGGAGCAAGGTGCGC

>AT10258

CTGAGATCTCTCAGTGGCCACATCCACCCGACCCCGGGGGTGTGGTAGAGCCGGAAGTTGTCCGAATCCCCAACTCTTCTAACGTATCCCTTGATCCACCGCTTGCGCTTCATCATGTCTGGGGTGACCCACCCGGCGTCGGGAATGCGCTTTAGGTAGCGTACGTACGCGCGGTCCGCCGCGAGGCCCGCCGCCGCGCCTAGCACGAGGAGGATGTATGGGGGCAGCTGTGCGAGCTCAGCGTTGAGGGCCGCGAGGAGGGCTTGGGGGTCTTGCCAGTGCTGTTGGAGAACTGAGGGAAGGCGGGACTGCAGCTGGGCGCGGTAGTGCTCGAGGTGTTCGCGGGGGGACTGAGGTGGAGGAGGCGGGGATGGGGACGAGCTCCAGGGAATCCAAGGCATCGTATTGATGCCCTGCCAGAGAGGATGGGTCGGTGGTCGAGGACAAAGCTGGGCCTTG

>AT10267

GTAGAGACCTGTCAACCCCTTGGTCTGCACGGTTTCGCGGATGATAGCTATGGGGCTTTGTTTCTAGCGGCGGCGCAAGGATGAGCGTTGGAAACTGGACGTAGGAGCGGTATGACTCGAACACAGGTGCGAAGGTGCGCACCTTCCCACTAAACTGGGACCGCGTCTTCACGAACTCCGTCGGGTAGGTGATAAACCTGCAAGCCCATGAATGCACACGGTCAGCGGGGCTGCGCGCCGTCAAAGCAAACAGGAAAGAGGGACAGACTGACGCTTCGACTGCACCGGCGGTTGTGCCGGCGATGAGGGAATGCATGGGTTTCTCCTTCTTCGACATGGCCGCCTAATCTTCCGAGGCTCGCACTCTCCAAAATCAGGTCAGTTTGTAGAGGATGACGGGATAGAAGACACGCAGCAGCGGGTCAGTCGCACGAGCTGAGCAACGCGGTACGAGCAACAAGTACGAATATGGTGCGCGCTCATTCGCGTGTGGCCATCGGACTCGGAAACCGGGGC

>AT10276

GGCGCCTGGCGATCGTAAAGAACTCGGTCATCAGCTATATAACTGATTAGTTAATGGTGGAGAAGATTGAGACGCAGCTGGCGCATCTAGAATTAATCTACAACAGTAAGGCCAGAAACACGCACTGCGACAGGACGTTCAGCTACTTAAAAGTTGTAAAGACCGAACGTCCTCATAATCGGTGCGCCACGCCTCCGGGAAGGACATGAGCACCAGCACAGCGTCCTGCGTATCCGATAAATACGCGTGACGACGGCGACCCGCTGCCATCGAAACGCAGTCCCGTCGACGACGTCTAGATGTATGGTGGGCCGAGAGCTAAGAAAAACGCATGGCGCAAGCTTGTAAGCATGTGCGGTGCGGCTGCGCGAGACGAGGCGACGCACCTTGGCGGAACACTCCAAGCGTGAACTCGACGACGAGCAGAAGGACACCGCCTCCAGGCGCGAGACGGAGGACCTTTGGCACGAAGCCCTTGTACAGCGCCGCGGGGCCCTCTTCACGGAAAATCGTCACCAGC

>AT10281

GGACGGGACCGACCGGATCGTATCCACGCTAAAAACAATTCAATGTCTGAAGCTGTTGTTACTAACCAGCAGGAGCAGCGACGGAGCGTACCTCATCGGGGGAGCGGTAGGCAGGCTTGACCTCACACTTTGTGTCACAAGGAGAAGATTCGCCCTTCGCAGGTCCTCTGCCGGACGCGCGACGCTTCATCTTGTGATAATTAATGAACAGACACTGCTCGCGCACGTCCGACGAAGTACCCGCAGGTTTACCTCCTGAGTGGCACACCGGGAAGCGATGGCCAACGAACCTCGGGCCTTCATCCTCGGACAACTCACAGCCGAGCTTTTCGCGAGCGTACTTTTCCTCCGCCTTGTGGAATTTCTTGCCTGCGGACGCGCCGAAAGCCCACCGCCACGCCTTCATGGTACCGGAAACGAAGACAAGGTCTTGCTCGTCGAGGCAGAGCTGCAACACGCCGTTAACGAACTCGAGCCAGTAGTCCAAGTTCTCTTGCATGTAACTCCTGATCGACAGGTCCGACTCGATTGAGTCGGTGGTAGTGTGGGAGTCGGTGACCAGGAACGCCCCAGGGCCGGCCGTGCATCCGAACTTCCATCCAGCGACGAGGCATCCCGGGTCTGAGTCCGCTTGTCCAGCCATACCGAGAAGATCCGCGTTCGCTGTTA

>AT10286

GTCCGCGCACGCCATATCCTCCTCTTCCTCGTCCATGGCAGTCGAACCTCCGAAGTCCAGCCCGGTTCCGACCAGCTCTCCGATTGACGCAATGAACGCGGGGAGCGTCTTGTCTAGTGGCGGCAGTGAGTGAGTGGGACAAGCATCGATCGGCCTGCCACCGAGAAGCTAGAGACGAAAGACTGGGCTGGATTCGAGGTATGAAGTACCTCGGGATACAACGGGAGGCGACAGCCAGACGGTGACGGTGACGGCCCAGGCCAACCCGATACCTGAGTTCTGAGTTGGTTCGTCAAAGAGAGGCGGCGAACTTGAACGTATCCTAGATCTAAATGTCAGATAAAGGGTACCGTACAAGTATGACACAAAATGCAGCCCAAGTAGTTAGTGTACAATACTACGCCGGGAGTTGTTATGGGCAATAAGAGAGCAGCAAGCCACAAATATTACAAATCCGGTGTAAGCCTGAGTAGTAGAGTTATTAGGCGCGTACGCCACTCTCTGTCCGCACGCGGTGAATGCACAATGAGAATACGGTAAGGATCTCCCTATCGTAAGTACTCTCCCGCAAGAACCGTCGCTTGTTCTCCACAGATGGAATCCGTCATAGAAGCCCGCAGCAATGTAGCTTCCATCAGGTGAGAAGCAAGGGGACCAATAAAATTCATCATCGAAAACCGCCTTCAACCTCCCACTCTCGGTATCCCATAGTTCGAGTTTTCTGTAAGGGGCCCCAGTTGTCCGCCGGAGGGTCAGGATAGAACACTGGTCGGATGAAAACACGAACTTGATGGCCCCATCTAGTACGTTCGAGTCGGGATTGGAAAATGGTATTATGGCGAGCTTTTTTCCACTGGTCTTAAGGTCCCAGAGGCATATGGACTCGACATGGACCGAAATCGTGGCTAGTCGACAGCTGCGGGTGTCAAATGCAGCGACCGTAACAGCCGATGCCCAACCGAAATCCTTCTCGACAACCTGAGACCCGGTGAAATGCCACAGATTGAAACAAGGGTAGGCATCGGGGCCTAGAGGTCTGTGAATGGCGAAAAACCAGGATGCGTCCGGGGAAAACTGGACACCAGAGACTCTTGTCTTGGACCATCGGCGCGCAGTGAAAATACCGGGGATCTCGTGGCATACGCCGCAGCGTGCGAGTGTGCCGTCCCAGACTCGGACAGACCCGTCGCGGGACCCAGTCGCAATCAAGGCTCCGGAAGAGGACCAGGAACAGCACTCGACTGTATCTGTGTGGCCGACGAGGGTGGTGACGAGGGTGGCATGAGCGCGTAGGTCCCAGACTTTGGGAGTGTTGTCGTCCCCAGTGGAGCACAGGTATCGGCCATCCGGAGAGAAGGCGAGGCTGTGGAGATAGTGCTCATGTGCGACCCAGTCGTAGACGATAGTGCCGCTTCTGGAATCCCAAATGATTATGGTCGAATCATTTGAGCCGCTGGCGAACCAGTGGCCATTGGGAGAGGCAACAAGTGTGTGTACCTTGCC

>AT10287

TGTGTCTGGGCACTCTATCCTGAGTCGAGATTCTCGATGGAGCTCTGGAAAGAGTTGTCTGCTCCGGGCCTTCCAGAACGAGGTTCTGTACGAGGTAGCGGCATAGCTGGTGGTACGACCCAGGCTTTTAACCAATAACGTAGGTCGGAGGGAAGAGTTGCGCAATGTCGTCTCGAGTTTGCGGCGAGCATCCAGCGTGACGTCTCGAGAAAGAAAGCAGTAGAGGTTGTGTAGGGGTGAGGCAGATGCGAGTATGACAGTGACTTCTAGTCTATGTGGATCCTTCGCCTTGATACCTCGAATGTTATCGAGCAGTTGTGTAAGTTTCGGTATCATGGTATCCCTGCTATAACTGCCCGAGCCCGGGCCCGATAGCGGCGGATCGGTGAATTCAGCGACTCCGACCGATAAAGA

>AT10288

GCGAGTGAGGTGAAGTCGTTTGATGAGGCGCTGGCGGATTACTTCGTCTCCGCGATAAGGCACATCCGTCGCAATTCTGCACGCCGAATAATCAAGATGTTGAATTCCGGGAAAGGCGAGAAGGAGGCGTCCGAAACTGGCGCAATTGGGGAATGTGAAGTTTCTGAAGGTCAAATGGCGGATGTGAATCCCATAGTGCCCGAACCCAGATGACGCTGAGCGGTTCAACGAGGGAGCCTTGGTAGCCTTGTAGCCGCCGATGATTGCCCAATGATCGAGATTGGGCAACTTGAGGAGAGGCAGCGGGACA

>AT9582

CTGGCAGTAGAAGTGTGTGCTTCTCTCCCTCCAGCTGTGGCCAAGGGTACTTATTTACCGAAGCCCATGCGACATGACGCCGTTCGTGCCCCTCAAACTTGCTATTTGGTAAGACGCTGAACTAAAAGCTCGTCTTCCTCGACCTGGATACGTAACAACCCAACCCATTGCGCCGTGCCGCATTCCATGAGGGAAAACCAAAATAGCATCTAGGAGCCCATAAATTATCTCAAAATGTACCTCCGTTCCTTCCAAAGCTTGCCCCGCGACGTTTCTAGAGTTCAAGGTGCTTGACGGGGTTGGCGGGACTCTCTGCCTCCTCCCCGGCACTCTGGCCGGACTGTAGTCTACGAGCGAACGCCGCGATTTCCCCGTCGTACACCTCCCACTCCGGTATCTGTCGTGCCCTGTCGAGCTTCGGCTCTTTCTGCAGATAAACGGGTCAGCGCCGACTCCGCCGCAAAGGACAAAGGCACGTGGATCGCGTACTGTGAGCG

>AT9604

CCCGGCTTAGCTCCTCGAAGACCTCTTGCATCGTTGATGTGCTTTCCTTCTGCCTCTTGAGAGCGTTGATGAGTATCCTCTCGTCCTCAACCGATAAGTCTGCATGTACGGCCTTCCTCCCCTCTCCGTCTCTTGTAGTCGGCTTCGTCGCGGTATGCTTCATCTCCTTTGAGACGGCTGCAACTAGTGACTGAAACCAAGGCGGTTGCTCCTCTGGTGCAGGCACTAGCAACTGCGGTGGCGGTGGAGGCTGTATTGACACCGGACTCGGGGCAGGGTAGTACGGTGCGGCCGTGCACGGGTTGAAGACGAGCTGTAGCGGGAATATTGGCC

>AT9605

TGGGTCTCCATCTGGAAATGCAGTAGACATACCACTCGAGATGACACCCCCTGCAATTCGCCATAGCTCTTCTGGCCCGCGGCAGATTCATTACGCACCCCATCCAGGATGTGAAGATCAAACCGCGACTGATGATGCCGTGCGCTTGCACGCCGCATGCCGTGAAAAGAGAAGCGAACGACTGAGTCGTAAATGGGGGAAGGGTTTCA

>AT9615

GCCATGGCCAGTGCGTTCGGCCTGCCATCGCTAGGGGACGCCGATTCCAACGGTTGAGCGAGGTATCGATGTACGATGTCAATCGCTGCGGTGTCTCTGTCCGTCTTGAAATCGCGAATGAGGGATAATGCCCTGATGCTCGGAAAGATGGCGGGGAAGGACTCCTTGAAAGCTAGCGAGGAAACTTGGCCGTGTAAATTGGAGGAGTCGTCATAGGAGTAGGCGATGC

>AT9628

GCGCCTTGCATACCTCGAATGAGGGGTTGCCGAGGGATAACCAGCGGCCAAAGGCCGCAACGGGTTCCAGCTCCAGTTGGACGCGCACATCTCCCAGATGATCCCTTTTGAGGCATTTGAGGATCCTTGTAGCTAAGCGCATATCATACGGTGTACTCGAGTTGTCGTTGGCCCATTCCCAATGGGGAGAGAAGCCCTCATCATGCGC

>AT9633

GTTTGCCCGTCCTAAACAAACAGCGCGACGCGCCATTGGCACAAGTCATTGCGGTGATGCATTCATCCATGGTCATCATCAAATATGCCGAGCACCTCACGCCACCGCCGTTGGCAGCATCGATGGGAACCTTAAGGTCGGGACCCAGCAAGCGTGCCATGGCTTCCATGAACTTGGCTGCGGCGTCGCCGTGTTTCTTCTG

>AT9647

GTAACTTGAATTTTCGGCGACGATCTAGCGCATCATGTTCCGTTGACGCTTCATCCGATCAAAGGGCTCGGAATGTATGTCAACCTTTGCGCGATAGGATTCGAATTCGCCATACGATCGACACTATGATGGCACACGCTGCGCCGTCGTCGTCAACAACAACTGAGAATGAGAAAGCGACCGAACATGCGCGGCACTTTGTACGACCTTTGAACGCAGAGCGCGATATCACCACCACCGTCTAATAATCTTCAGTCTCGACCGTCAAATCGATGATCTCCGTGTCCACGGGTCGGCTCGCTTCGTTCTCCTTGAAATCCCCACCCCCGTTGCGCTTATCGGCGGTCTCCATCTCCTTCTGCGAGGTGACGACGTCGATATCAATGGCGAGACGCTGCTCCCCAGCCCCACCTAGGGAAGACAGCAGACCGGCCTTGGAGTCCGCACCGTCGGCCAGCAGCTGGAACTCCCTCAGGTACTGGTTGATTGC

>AT9668

TGTGCGGACATATGGGCATCACCTGCCAGCCCGTAGATGTTCACGATAAGGCTCAGATACCGATTGAAGAAAAACAAGACGGCTGCGCCTGTCCATTCGCCCTTCCAAAACAGCTCTACCTCGCGCCCTAGCGTTATGAAGTACTCGTATATCACCAAATCTGGATCGAGGGTTAGAACCGTCACACGATGTTTGGAGGAGTGCGGTACCGGATCCGGCGTTTGCGCAGTAGTCCGAGACTCGCCTAGACTCGGAGCAAGGCTAGTCAGCCCATGAAAGAACGCGTCAAAGTACTGGGAAAATACACACCGGCTGAAAAACTCCCCAATGAAGGCGGGGATATCAAGCTGGGACGGCATGGAAGAACACGCAGGCCGCACGGAAGACGCGACGGGGTTCGTGGCAGGGGGAGCTCTGAAGGTGCTGGCGAGGAAGCTTAGTCCAACGGCAGACCTAGTTCGCCACGGAGACTTTATGGCCAGTCAATTTGACAGGCCGGTGACATGAGGGTTCCCCCCCTGTCCGATCTAGGACCGGACGACCAAATGCCGATGGGGGCGCGCCAAACAACTTCTTCGCAGGAAGTCATACGCCTCAACAGCCTCATGTTGGAGTAGCCGCCCACAGAAGTAATTGGGCGGGACCGAAGGAGTGACGTACGAAAGACAGCGCCGGCACTGGAGATTGAAGAGACTTTGACGGGGACATTTGAGGACATGACTGTTGAAGGAATATGAGCCTCGAGGCCTCCCTGAATGTCCATTACGAGGTAAAATGTCCGAGTGAGCACGGTTCTTGGGACAGATATAATGAACGAGTAGATAGAGTGCGTGCCACTGATGATGCATTCCGAAAGTCCCGGAGGTTCAAGAATGATGCCGGTCTC

>AT9670

TGCCTGGTTGTTAGTTTAGTTCGGTGTATGTACATGCGTTGGGGAATTGGGAGAATGCGTATGGACGCAGGACTAGTACTAGTACTGGTGATCAACTTGGGGAAGGACATGAGAAATAGCATGCGATAGCTCGACATATCAGGGCGGAGGGGCGGAGGGGGGGACAGGGATGTGCGGCATAGGAGATCAGTGCGAGAGCGAAACCGATTATACAGCGAGTAGCGAAGCATATCGGAGATGGCGAGGTAGAACATGTCCGTTATATCCTTTGGAGAGCCTTGGGTATGTGGGTATCATTGCTTGGGGGTATCTTTCTTCATGAAGCGACTATTCAGGACCTGGGGCACGCTGGTCAGCGGTGGGTAACATAGTAGGGGAAATCAAGTAACGCACCTCGGGCATCATGCTTCCGATCTGTGTAGAGAAATGCGCTCAGAAAGATGGTCACGAACGTAAAATGACCATGCTTATGCTTACTTGCGGCACGGTCCAGTTGATACGGTCTAGAACCCGCTTCTTCTTCTTCCGCTTCTTGTTCTCCTCCTTGCGCTGCGCGTTCCTCTTCTCGATGTCCTTGACTTTGGCACAGCATATCTTTCTGCGATGCATCGGGGTCAGCTGTGGTCTCGCAGG

>AT9676

TTCGATGGGTGCCGGGCCATGTCCCACACGATCAGCCGCCTCGCGGTGGGGTGGGGGGATACCGCGAGGAAGGGGTCTACTGAAGGGGGCACGGTGGTGGACGGCGGTGGTGCCTGATTGGCCGCCGATCTCCCGTGCGCTGACATGAAGGGGTAGTGGCCGCGCCCGTCGACGGTCGCCCGCGCGTCGCTCATCCACGGAAGCGGCACTGTCCGCAACTGGGGAGTTGGGGGAACCCCGGTTTGCCAGGGGGCATGGGAGGAGGATGACGCAGGTGGGGACTGTTGTAGCCGTTTCCACGGGGAGTGCGCGTCGTCCTTTGTTGAGTTTGATGAGT

>AT9679

ACGACAACAGCTCCCACACGTGGATAGAATTGAATTTTTTCCGGAATGCCCACACCGCTTGGTCCGACTGGCGCCGACATGCTGGGTCCAGTGCCTTTCCGTCTCCCCTCAGAAGATAAGGTTCGCCCGGTGACATGCGCTGGGAAGACAGAAGGCGGCGACTATATCGTCCTTCGGCTCATCATAATAGGCGACGCGAAGCCTTGGGAACCGCCGAGGGTTGACAATGATAGGAAACTGGGAGGGCCTTTTGTCGGAGAGTGTAGGGAAGGAGGCATATGAACATCGGGAATCAGTTGAAGAACCGCCATAGCTCCATGGTATCCCATCCATCGGGTGCCGCGGAGGTCTGCTTCTAATCGATGTCGCTATGCGCGCATCGACGCCCCGAGATCAGGTGCCTGCTACTGCATACCTCTGTGCGGTATACGGATGAGGATAGAACAGAGACCGGGCCTGGACGACCACTGGTGTTGAGCCCCCG

>AT9700

GGGTTGGGACATGGATAATGCATTGTACTGAACAGATACAACGTACTCGGAGACACGTGGAGTGTTCTACACAAGAAAGCAAAACACATCACGCGTTGAGGGACAGTCGATATTGACCTGGGTCGACATGTCGGCCGAGCATGCGGGTCCGGGGGTTGGTATGAAAGGTGGTGGCATGGGCGAATTCGTCAAGGACAACGATGAAGAGACTCGACTAGAATGCGTGACAAGGAATAACTCACAAAGCACACATTGTGGAGCTCGGCATCGGAGATGAGTTGTTGGCTCACTGGCGGGTTTGGGATACGTCCAGCCGGTAAGGGCAAAGATTCCTTGCCGCAGTATGTGGAGAGGTAGAACAGCATGTCTTCGCCATTGTATATAGAGAACGGACCAGGCGGGAATGAACACCAAGAGCCGTGGGGGGTTCGAAGGGTGTCGCCGTTGTTCCGCGGCCACCGGCCGTCCAATGTCCATAGCTGCATGGGCTTTGATCAGGCTTTTGATACTGGTCAGACGGGAGGGGTATCCAGATATACGGGTGCGAGGAGTGCGCTTGGTAGCAACTAAGGAGATGGAGAGTCGAGGGTGTTGTGGCTC

>AT9723

GAGACGAGGCCAAGGACAAGCCTCTTATTGCGGGGAAAGTGTCTGAGAGGTTCAAATCCTCCTGCACTCTTTGAGTCGTATTCGAGCTGTAGTGGCTACCGGTAAACAGAGGCAGGGGAGCTCGCAAATGGAACGGGGACGTACGTAAAAGCAGTCGAAGTCCGAGTCCACGCCGGTGAATAATCTCTCGGCGATCCGATCGTACCCTCCATGCGTGAAGGGCGTACCGTCGGGCTGATCCGCGACCGGATCAAAGGAACAGTCGAACG

>AT9724

TCAAGTTCGAGTCTTTGTACATAGGTCACTCTCCGAAATCCTCCCTCCAGTCATCGTCTGCCGCTTCCCGTGGACAAAATGTTCAATTCGATACCTAAATCTCCCTTGAACCACGCACAGAGGGTCATGCGTTCAAACTACTAGCACCTTGCTGCTTCTGACATCCTTCGCTCCCTGGACATCCTTCCTCGACGACGACGGACTAGTATTAGTCATATTGGTGCCAATACACATCAACCCGCTTCGACGAACTGGGGCGGATAGTATTAGTTCACGGAAGACATCCCAGCACGCTGCTCGTCTGATGTAGGGGAGGAAGGTGAGCATCGGGCATGTAGTTTCACGGATGGCACTCGCCGTTCATAAGAGGGATGATGGCCTTCCTGACGCGCCGCCCGCCCTTGGTGAGAGGGTATTCGCATGACGCGTCGATTCCGCGCTTCGTGCATCGCCCGCACGGGTGGCCTTGATCGCACGTCTTGTGCGCATCGCTGC

>AT9730

TACCTCCGAAAGCGCTGATGTTCTCGCGGACCCACAGGAGGGCAAGCCACTGGTCCTTGAAACCGAAATTGCCGTCGATCTTCGGTTTGTCGGACGCTAGGAAGCCAAACGCGGACAGGCGGTACCCGATGTTCACCCACACCTCCGAGCGCTCGGTGGCGATATACTGCGCTTGCGATCCCAACCCGTGGGGGGAGCCGAATTGCAAGAACCTGTC

>AT9739

CAAATGATGTCATTGCTAACATCCCAGACTGCGACGGCTTCGTTTTTGTCGTCGGAGGTGGATACGAGTCGTTGGCTGTCCGGAGACAATGCAAGGGCAACCACGGGGCCCTGATGCGCCAGCCATTCCTGCAGCACTGTTCCGCGCGACGTATCCCAGATGATGATAGTTCCGTCCTCGGAGGCGGTCATTATCCGTTTGTTGTCATGAGATGCGACGAGGCACGTCACTGCAGCCTCATGCCCAAGCACGTCTGGAGTATTTTGTTTGTCTGGATCACAGCAATCAGTTGTTTGTAACACACACACAGAGGTGAGCGGAACAGCATTTACCACGTACGAAGGTAATGAGCCCCCGTTGGTGCATAATCGGAAAGGCGTGTTTGATGGTTGGAGACCAAAATTCAGCTCTTCCTATCCTTCCCTTACTGAACGGCAGCATACGGAAGATGATGCTATGGAGAGGGAAGGTGGAAAGTGTCCCTTCTAACTCTTTGCATGCCTCTAACATCGCAGCGCCT

>AT9752

GGGCCGGGAACGCCAGCCCCAGCACACCCGCCGTTCCGTTCCACGTCGCGCTGCTAGTCGCGTTGTCGACGGCGCCGATCGTCTGGTTCTTCATCGTCAGTCCCCCGATCACGATGGTATCGCGGCCAACCTGGCCGGTCGGAAGGTCGGCTAAGTCGGCGAACTCAGGTAGAAAGGGGATCTCCGAGTCGTTGACGGGTACGAACGAAG

>AT9754

AAACCCTGGGTGGGGAAGTTGACCGCGTTCAAACCGCTGACCTGGGTGTCGAGGCTGCGAGAGGCGGTGATGAACGATTGGATCGCACTGATCACTTGGTCCGAGGTCTGCGCCATGGTCCCGACAACGCCGAGGAGGGCAACGAAGAGCGCGGTGAGGAAGGTGCTGAAGAACTTCATCGTGGTGAGGATAGAAGGTGTGTAGAAGCTGTTTAAGCTGCTAGGCTCTGTGAGATGTGGAGTGTAGGATGTGAGATAGAGGATGAGGGCCGGTGGGGTACAGGCTTTTATACTAGAAGCCGCCTAGCTTGTACATGCCCTATGGCATGAACAGACCA

>AT9783

GCGCCGCCGTACGTGTCCGTGCTCTCCTCCGTAAGCGAGTCGAACGAGTAGCCGAGCCCGGCCTGCCCGACAAGCTCGAGTGCCGTCCTTCCCATCCAGCTCACCAAGTTAATTTCCCTAGGAGCACCCTTCACAGTAGCCTCGAGTGCAGAACGAAGCTATTGCCCTTCCGACATCAGTCTACTGGCCATGCCGTGCGTACAACATGCGGCGTTACCTTATGTGTCACTTCATAGAAGATGGGAATCATCTCCCTCATGTGAGTGATATTGAAGGCCGGGTTGAGCATCTTCCTCTGGTGACGATGCTGTTCACCTGCATGCCCGCTTGAGCCCTCCACGAGGTAGCAATAAGTAATGAACGTGGGTT

>AT9784

CCGAGGAAAATGGAAGGACTCGGAGGCCCGGGAAGATTATCCAGAGCGCTCTTCACAACGAACTGCCGGCGGAGCTTCCAGAGAAACCATGTCGCACCGCAGATAAGGACGGCCTGGAGAGGGGTGCTGACCATCGTCGCGAGTGGCGAAGAAGAAAGTCAGCAGTGAAGGGAATGAGCACGGGCGGGGGTGGAAGGTAGACGCGAGGTCGTCGAAGTCTAACCTTGTTATATGGTATGATCATGATGATGCAGGCAACCAGCCATGTGCTAATAACCAATGACAGCAAGGCGTGTATTACGGACAGGGCATTGCGGAACGGAAAGGGAGGTTGCGGAGACCTGCGTCGCGAAGCGAGACTAT

>AT9801

CATCCGGGTCCAGGGGGAATGGCTTCGTGCTGTAGACGGGCGCAGTCGAACATCAGTCCCTCTCCGCGCTTCTTCTGCGCCCGCTGAGTCAAGTGTCGAAGGACTCACATGTCCGGAGCGGTGTGGTCGAACTCGTCATCCTCACCATGCTCCGCCTTCCCCAGGTTCTTCTCGTCCGATGAACCGCGTGAGCTCGAGGTGCGGTGGAGGTCGTCGTGTGCCCGAAGTTCGGGCTCGAAGGCGGTAGACGACATAGCGGCAGCACAGGCTCAGACACTGGCAGGACAGAGAGAGTCAGCCGCAGTGAAGGCGGGGGCTGGTAGAGAGGGACCGCGAAGGTCGGGAAGGGGGTCGTAAGGCCTGAAGGAGGTCGGAGGACAGAAGGGACCCTAGGGTTACCGAGCAGGCGACGTTTTATATTGTGGCTGCCTTGGCCGACTGGCCACCTCTCGTCCGCCATTCCCGGCCTATCTAATTAGTTGTCATCAGTCCAAGCCCTCGCATGGAATAAGTAAGATGGAGTCAACTTCGAGACAATCCGCTGATGGGCGACGGCGGAGGCGCACCGCACCATCTGGACGCGTGCG

>AT9803

ACGGGCAGGACGGGGACCCTCTTGAGCTCGTTCTCTGGGGCTTGCTGGTAGTCGTTCGGGTATACGACAGGCTGCGTGCCGAGCGCGTTGACAAACGACTTTATGAACGCAAGCTCAGCCTGTTCCGCCATGGTCGCGATGGGTTTGGATACGTGGCGGGGAATGTGAGCGGGATGGAATGAGGGAGGGTCGTGGCGAGCAGACGATGCGGTTCGCGAATAGCCTCCGCTTGTGGCGGTGTGCTGTGTGCAGAGTCCA

>AT9805

CGGGCTCAAACGGGCCCCCCTTTTCGATGGAGTTCCTAAATGACGTAGGCCTGCGGTCGAATGACCCATCCGGCCGGCCTTGCTCTGCGAGAGGATCGAAAAACTTCTTCTCCTGAGATGCCATCTTGTGTGGAAGTGGATAGTCGACTGCGAAGAGGGAGTGTCAGATAACGACGCTGAGGACAAGTTGGTTTAGGAAGAACGGCAGTCGGGAATAGGTAGCCGAACCCGAACGCTCGATGGCCTGTGCTACGACGCGGAATGAGATATGACACGGGAGGGGTGAGATGAGGCCCA

>AT9851

ATGCGTTGACTAAAGGGAAAATTCTGATAACCACGGCGGCCACCGCCACACCTAACCCGCACCCACGAGCTTGTGGCCCAGGGCCATACTCAGACCAAGTAAGGATATACAGGAAATGGAAAGTGAGACAAATGAAGACCGATTGCTCAAGTGTCCATCCAGATTCTCCCACTGTCCGATTGGCTTGTATGCTATAATACTGTGCATGGCAACCGCATGCTATAGGAGTGTGCCACACCTACCAATCCTTATACTAACAGAGACGTAGCCCACAGCTCAGAGCTGCACAGCTCATGAGTCGACGTCCATGCACGTCCCGTCATCCGTATCCGTCGGAGCTTGTAGGCCATTCCGCGCGCCAGGAGATAATGTTGCAGTCGGAGATGCGACGTGGCTCGGAGGGGCTAGCGCCGACGGCGAAACGGGCGAGCGGCCGTCCGGCGAGCGCGATTTCGATTTCTTGCCCTCCTTGATCTCGCGCAGCGTGCGCGCGGAGGGGTTGAAATAGCCGCACTTGGGGCATACATATTCTGTGAGGGCGATCCGCCTCGTTAGCCACAGCCTCTTTGTCCCGCACATGCGTGAAAAAATGTGAGGGGACCAGAAACTCACGTGTATCTTCCCATTGGCTCTCCTTGACGAGTCCATTGTGCGCGAAGCACTTCTGACAGATGAGGGCGTAGCGAGAGGCGGCACCCGGAGAGGCGGCATCTTCGTCGCCCAAGATCGCGTCAGCTAGCTTGTCGAACCACTGTTTGCGTGGGGGAGGAAGGGGGCGCTGGGGCGAAGCTACACGGAAAGCAATGTACGTCAGTCAACGGTGCTTGCGGATCGCAATGATGATGC

>AT9856

AATGAGGACGCCGAGGCCGACGAAGAAGATTATGCCGCCGATAACGCTGCCTGCTATTATTCCCGCCAAATTCTTCTTCGGCGGCATCACCGGGTTGGAAGAAGTTGGTGCGGGGATGGGCGTCCCTGCACTTGAATGGGTGGAGGTAGGCAAGGCACTTGTAGGTAGCATCGGGCTCGAGAGTTGTGCAGGAATGACGGTTGGTGCTT

>AT9858

AGTTCAGGCTGGAGACTCGTTGTATGTATAGCGAGTTCAATATTTTCCCGCTCCACTGCGTATTAAAAGAATGGCGAGAACTTAGAAGACAGAGTACAGTCTACTTACACGAGAACAGGGGAACCGTGGGGCAAAGACCGTTGTCTAGATCAGGCGTGCTTGTTCATTTACGAAGTAGGGCTGGAGTCTTGCAAGGTTAAACTGTCGCAGGGGCCTTCCTCTGTGTAAGTTGGTGCTATATCCCTAGAGGATTCCGGGCGCGTTTGAGTGCCAGTGCCGCCCTATATAAATGGATGTCAGCGACGTAAACCGGGAATAAAATGATTCCAGTAGCGTACTTCCGGTAAAATACTCTCTTGGGGACTTTCAGGTACCACTGGGCCCTTCAGAGAGACGAAGTCGTTATCTAAGGTATCCCTCGCCACGGCCAGGGGCCTTTTGAGGCGATGACGAGTGATTTCCAGGTCCGCGTTGGGGTGTGTCCTCTTCACGGCCTGCCATACACTTTCCAGGGCTGAGATTGCCTCTGGCGAAATTATATCGAACTTCCGGTCTGGGCGGCTTCTCAGGGAGATGAGCGGCGCGATAAGGATTTGAATGCTGTAGCTGAAGTGCACGAAGCGGCCTGCTTCCCTGTCATTGGTAGACAAGTCGTTACCGACTGATCCCCAATCCAGGCTTTGGAGGGTATCTGCGAAGTACGATAGGATGTCCCGCCCGCGATTGCACAGGGCTTTCTCCTCCTCGAAGGATAGTGAAGTACCGCCGGAAAGCCAGTAGAAGAGATACTTGGAGACGCCTCGGGCGGTCTTCATCCACGCCGTAGAGACAAAGTCCCGGGATGCCGAGGGGTTCCCGTCCTTGAGGTACC

>AT9891

CGCCTACTGTTACAATCGGCATAGCACAATAGCAGCGCAGTTCTAACCAATAAGTAATACGGTGCGCGTGACCCGCCCACATGCATCGCGGCAGCGCGATCCTCGGCCACGCACGACATGACAGAGAACGCCGGAGTACCCCATACGGCATCCAACGCCCCCAAAGGCCACACCGTCCTAGCCGCGCGGGTTCCGAACCATCCAGAAATCATCGATAGGCATACTGAGGAGGGTGGCATGACAGGCACCTCGGTGCTTAGTCGCGCGAGCATGCCGTAAACGACGCTCAAGCGGAACCCTTGTACAGCGACACCTTGAGAGGCAGCTCCGGTTTGCTGCCGTTGACACCGACGGTCGGGAAGCGGATGCCAGAGATGTTCCATGCGATCGGCTTGTCGGACAGCTCGAAGACGAAGTTCGAGACCAGGAGTGACAGGACGACCTCT

>AT9898

TAGGCTTACGTCCCAATATTGGAAGGAATAGATTAGCGCAAACGCGAGAGATAGTTGCGCCGTTGTCGAGAAACCACATAGAAAGCGTGCAAACCATGCGTAAATCGTCCATGCCAATCCGATCAAACCATCGACGACCCAGGTATAACGAGGATGAAGATACGGTAGACCAGTCGATCACATTCGAGAAAGGATTGCCATCAATGTAGGGATTGTACACACATGCGATTGGAGGACCGTGGGGGGCATCATCATGACAGCTCAAGAGGACAATACACATCGTACACCAAACGCCCATTCGTCGCGGGCCGTCGTCTGACCCTCGCTACATGGCATACCTATCCATGGGAGAATGTTGGTCAGAACAAATGACAATGATACAAAATGGGCCAGGACGCACTTCCGTGTCCACTCCCGGGCTGTCGCCTCGTACCGCTGCCGATCGGTCTTGTACAAGTGCGCGATGTCGGGCACAAGCGGATCGTCGGGATTCGGGTCCGTGAGCATTGAGCAGATCGAAAGGAGGACTGCAAACAGCACAATGAGTCAGAACATCCACGCAGATCACAATCGATTCGGCATCGCCGCTCAGCGCTCCGAATGCGGATAGACAAGAGATGACACCCGTAATCCCGCCCTGCGCACCTTTCGAGATGGTCAATGCTGGACTCCATTGGTCCCTCAAGATATCGAGACAGATGGAGCCGTTGGCATTGATGTTCGGGTGGTAGATCTTGGTCGTGAAGCTGACCTTGGGCGGCTTGAAAGGGTAGTCGGTGGGGAAGGTGATCGAGAGGAAAAAGACGCCGCCGGCGTAAGGTGAATCGCC

>AT9910

ATGGTGTCTCAATCGCTCAAGGTCAAGTCAATGACATCCCCCGCCTCCATCCTGAGCTGAGATGAAGACCCGGAAGCGGCGTGTTCCTCTTTCACCGCCCTTCGTGGCGCTTCTTTGCGTCGCTTCTTCTTCTGCTCCATCCGAGTGATCCTGTCCATGATCTGCGAGAGCTGACCCTGAAGCCACGAATACCGGCGCAGTCTTCAACATGGTCAACTCATCCTCGTCATCATCCGAAAGCCCATCCATGGGTGGCTTGACATCCGATTCCTCGTCAGTGATCCCATCGCCGGACCCGAAACGGCGGCGCTTTCTAGGCCAGGCGTTGTCCGCCTTACTATCCCCCTTCGTTTGTGATTGCTGGGACGATTCGAGTGGCTGGATAGGGAGGGGCGCGATGCCTTGAGCCTGCAGATGAGCTGTAATGATATATATCAGCGACCGTAAAACACGTATCATGGGGCTCACGATACTTGCCCAAGGATTTATAGCGAAAAAGAAACTGGGCCACCAGCCTTCCGTCCGGTTCAACATCCTTGATGGTCCATTTCCCCGGGGTATATGGTTTTTCGACGGCTTCTCCGAGGCTTTGAGCAATTTGTCATCCAACAATGTTTCCAACCACAGCAGCCCAACGCACGTTACGCAATGTGTTCCCGCCTTTTTGGATCGTTCATGTACGAGACCTACTGGGGGTAACGTACACATGTAAGGCCTTGAACTTGTAGTATTGCTCTTGGGTATCAATTGCACTTGCTGTACTTCAACGCAGATAGTCCCAAGATCTTCGTTTGCTGGTGCATGCAAGGCATCATCCACGTCTAAGTGCATCCATCAACCATCGTCTTGGACGATAACTTGGATCATCCGAGAATTGCTCACCTGTTGTTAGTAGCTTAGAGAATTGGAAAGTGTGCTTCGTCGTAGAGCTCGTACGGATGTCCCTCATAGCACCCTCTTTATGTGACGAGCACCATGTCCTCGATCCGACCCTGTCGCCGTCCAGCTTCACCATCATGCTCAAGTGATCTAACGACGTGTTCCGCCACTCCACGGCGAACCCCTAAGCGGACAAACACGAATATAATAACGCGCGTGAAGGCAGATGGGTGACACTCACCTTACCCGCCTCGCTGGGGATGAAACACGCAAGGGCCCTCCCATCGTCTCCCGCCGCTAGTGTCGTTTGGTACTCGGGGAGCCGCTGTCCTTCACTTGTGATCCAAACTTCACACTGGTCGACATGCATACTTAATTCCCAGAGAATTAGGTAGAGGCTGTGGGGGTACGGACTGCGAATCCGATTGAACATCGGC

>AT9930

GTCCACCGTATCGACACCCTGCAATGATGAAGTCCTGCAAGTCGCCTGGCAGCTTTCCGGGAATGGAGTAGAGTTGATGCCGCCTGTAGAAGACGGAGCCATCTTGCATGGCCTCCCATGACAGTGTCGGATGTTCAGTCATTCTTGCCAGCGTAGAGAGAGAAATAAGCCAGAGGGGTGAAGATGGTGACCGAGTTCAAGGACACTCCAGAGCAGTGGGAGGCAAGCCGCCGGGCGCCCCCTTTGCCTCCGGCACTTGTCGC

>AT9935

ACCGGCAGCAGGTGAACTTTCACACCAGTCGTTCTTGAAATAGTCTGTGTTGTGGATGCAATCAGGAAAACGGTCGTGAGACAAGCATAGGGAGACGCACCTGCCCTCTGCTCCCTGTCAGGGACTGACTTGGGCTTGTCGCCGCGGATGGAGCATACGCCCATGGGTTCCTGGTCGGTACACGGCGGAGACTGGGTGCCGAAGGAAAACTTGCCTTGGCGAGTAGAAGACATGGTGGTGGTTAGAGGATAACGAATGGAAGATTCGGTCGAGCTGCGTTGATGCTTCAAGGAGAAGACGCCTCCT

>AT9984

GGGGCCTGCGAACCGTGCGAGACTGACGATGCTCTGCGCGATGCCGTTTGCGAAGCCGACCAGATGGGGAGGGGACACTAGAAATGGGGTTGGACGGGAAAACGAGCCGGCACGTCAGCACAGGTACGGCGTACAGGGATGGGAAATGGAGAGTGGGAAGGAAAGAGCGGGAAGACGCACTATAATTGAGCAATACCGAAATCGCGGTGTAGCTGAATGTGCCACCGCAAAATCGGACTGCGCTAGAAGTTCCAAAATTTTCATCAGCGTCATTGCTCACCACGGTTGAAATGTAGGATAAGGGGTTTTGGTTGAAATGTAACTTACGTGCTGAGCGACAGCGCTGGTGTGCATGAGAACGATCAGTAACCTCATTTTGTTGACATCACGAAGCTAATAGATGCTGCGAGAGAGGGAACGTACCGGCCATAAGGATGGGGTTGCCGTCATCGCTTGGGCTTGCAAATACGCGGTAAAGAATGACCGTGAGATATGACGGAATGAAGAGCATCGTTCCCAGACGGAACATGGAGAGGTGCGAGAACCTTCCTCGCGGAGGGCTGTAAGCGGGTGTGGTCAGATCTCGGTCCTCCTCGGCATGTGGGGTAAATGAAGAAGACACACCCG

>AT9986

CGTATGCGCGACCTTCATTACTTTGATCGGTGATTGAAGCGACTGTACCCCGTGCGACGCCGATTGCGCCCGCGAAGATGCCTTGCATGAGGCGAATAGCAATGGCTTGCTCAATTGATGTTGAGGTGCCAAACAGACAACATGTGATGGCGCTCCCAAGCAACGAGATGGTCAAAACAATGCGTGGGTTGTGCTTCGAAGCCACAGTGGCCTGA

>AT9987

TTGACAAGACAATCATTGACAGGACGGGAAGAGGTGCGGCATACACTTCTCCCGAAGCTTGAAGTGCACTGGGGATTGCTGGACGCTCGAGCGGCTGCGCAACACCACTTTCCTCGTCTCCGTCTCCCACTCGACTCCATCGGTTGAAGATGTTTGGTTTGCCGACGAGGCTAGATATAGAAACACGAGTGCTTGGTCTTCGCGTACCCTGCGCTGCGTCTCCTTGCGCTCCTGGTATCTCGACTTCCGTAGCTTGAGCCGAGTCGCTTGGCCCAAATACCCGTCTAGGAGCCATGATAGCGATTAACCAGAGCGCCGACCAGACCTAGCTGTATGCGCGTAGGGAAACGAGCACCCAGGATCAGAAAGCCGGTACAGATATAGTGTCCGCTGCTACTGCGGCGAGGAATAGGAACGAGCAGCAGCTGAGGAACGAACAGCAGCTGTAGAACGAGGTGGACGATGGTGGAGGTGGTTCTGCGGACGCTGCTCAGCTCTGTGGCCATACCTATGAGTCACGTGAGTGGCTGTCATCCACTCATCATCGCCTCGGGATCGCGGTCGCGCATGTCACAGTACCCATCCTATCAAGT

>AT10092

ACCCCGACCAGCGTTGATATCACTATACGGCAACGAGTCAGTTATAGAGGTGTGTCGGTTCGTTGAAAGTCCTCACTCGATATTCCGCATGCCCAATTCCTGGCACGCTTTGATGACGTAATCGTTGATTGGCTGAAGAGAGGTCATGAAGCCTCAGGCCCATGCAGGACATAACCAATCGTGTATGGCACTCACTGCAGTCTCGGAAGGGAAGCCGGTATCGATAGGCCCAGACGTCCCATGGTCTGCCGGGTCCACCCCCGGATGTTCGGAACTAGGATGGAACCTCTCTGCAGTCAAGAAGTACCTGCGGACAATGGGAGACAGTAAGAGACACCTTC

>AT9620

TGGCCCGCTTTGTCGAACGGACAGCCTGGGCGACCTCTTCTGCGACCTCGTCCTCTTCCATCCTCTCCTCGACGCGATGCCTCAGGCGCTCCAGAGCCTCATTATCCCTATTCAACCGGACTTTTGAACAATCCTTTCCCGGCCGACCGCGTTGACCGACCCGTTGCTGTCAAACAACATCTGCAATGAGCATAGGCGCGAATACGGGCACAGAGGCTGCTTCGAAAGCAAAGAAACAGCAACGGACCTTGCGCGGACGGCCGCGCTTGAGCGCGACCGTGGCGTCGAGCTGGTTGTTCTTGTGGATGGGCGAGGACAGGGGCCCGTAGACCGTGGGGAAGCCGGGCAGGCGTGCGTCCCTTGGACATTTCTGAGGGCTGGCGTTCCAGCCCATGCCCCATGAATCCGGACGGCGGGCACGTATGATGGTGTAGAACCTGTATGGTGTGTCGGCGGTCGACGTACGTATGTACGGGCACCATCCGTCTGATCTGCACTCGCGCGAATTCGGACTCTTGTGGCGCGTACGGTTGTCCTGTACAAGGGCACAGGTTGAGATATGAACTACGATCCCCCACGGCCCATGCGGATGCGCACCTACAACGTGATCGGGGTTGAACAAGCCGCGCTCGACGTACTTGTTGTACAACTCGGCCGTCATATCCGTCGCGAACGCCTTCCGAAACACTTGAACGTGCCGTCCTAGATATCGCTGGTCGCTGTTCCGGTTGTGCGCGAATGGTACATCGATATTGAAGCATCCCAGGCAGTCGTGCCAGTAGCCTTGCGACAACCACCTATGCATATATGCGACGATCCCGTCCTTGAACGCGAGGTACAGCGCGTCGTCGATGCAGAGCGCATATGGAGGCATCCCGGGCCACAAGCACTGTTAGTGGAGGAAGTCGACGGCGAGGTGCTTGCCAAACCCGTTGAACACGGTTTGGTTGTGAACTTGTGACAGAACTCTTTCACTAGGGACACGTTCATATGAAGCGGGGTCTTGATCAAGATGTCCGATCGCCATTTGTCCAATCCTGCGAGCGCGGCGTCCGTCTCCTTGATCCAGCACGGACCACTGTTCTCGTCCCAC

>AT15890

ACAATTTGTTCCGCGTAAGACCCATCCGACATCTTCTGACACGGCGCCACTGGGGGTGCGGCTTCGTACGTTGGCGGAAGTGCGATAAGGGACGAGCTCGACTTCCAACCTCCCACAAAACGAGGCCCAACGCCCATATATACCAGATCCTCCTGCAGAACCCCGCACGGTGGGATCTAGGGTGCCCCTAGGTACCACCACACCGTTCGCGACGGTACTCGGTTCTAATTTCTCCGATGAGCACCGTGGAGTCGCACCCCCGGCGGTGCCGGAATGAAAACTCAAAGCACCCCGCATGTACTTATAGCGTTGACGTCGCGACGCCCATCCTTGGTCTCCGCACCTATCCGCACAGGACACGGAGCGCGGGCGATCATGCGCGAAGATCTGCCAGTGAGGACAGGGAAACC

>AT15896

GGGGCGGACCAGGGGTGGACATACCGAGCCAGAATGACGTCGGCTCAGGGCCGGGGATGTCAGAGATGGACGGCCGGGTGAGCAGGCGGTAGAGGAATCGCAGAAGCGCGAGTCCCGCGACGACAGCAGCGAGTTGGACGAGCTCAGCCATGGCGGGGGACGGCAAAGAAAGAAAGAGTCGATAACGGGATGCGTTCGAGTGAGGGGACAAATCCTTGCGTTCTTATAGGCGACGTGGGAGTGAGAACGCCGGGAAGGGTAGGTGGGCATGGGGTCTTCAATGTTCTCGCACATTGATAGGAGGGCATTCGCTACTCCGCGAAGGATTTGGACTATTTGGACACGGGTAAGTGTACTTACTAACGCTGCGGGAACGGGCCCGGCTCAGTCTTCAGATATCATCGCGGACTGTCGCCGCGGGCTCTCTTTGCCAAATAGGACTTGGCGCTCTCCGGCTCGACAGCATACTTTAGC

>AT15910

TGGGCCTCTTCTAGCGCACGCGCGTCGAGGTGGGAAACCATTGCGACAGGATCCATTTGGATCACGGCGTACGCACTACCGGGGGGGCAGAACGGCAGAGACATTACGAAGGATGGAGATGGTTGAACTAGGGAGAAGGACCCGGTCAAGTGCGAAACAGAAAACGGCAAGCTGGGCTTCAGGGCAGCTGAGGAAGGACGAAGGAGGTCGCGAGACGTGTCCTGGGGGCCGTTACACTCGTTAAATACTTCTTGTTAGACGTTTGAACGCTTGGCCTTGGCGTCCTTTCTGCTCCTTTGTCACCCTTTTGTTGTTTCTTCTTGCGCCAGAGGCGCTCGTGGATTCCGGCGGTTGCACTGCGCTTTGCAATTCTCCACCGGACCACGGCCATGGCCCGAAGACGCACAAACCAAACACCTCTCATATTGCTCGGTGCCACTCCTGCGGACAACTGCATGATAACCGAACAATTACCCTGCTGAAGCAGGCTTGCAAGCCTGGCAAAGCGGTCTGAAGTTCGAAGTCTTATCCAAACCTAAGCGT

>AT15912

GCGGGGTGGCATGCACACGGATAGTTTCTGGGCGAACGCACCAGGACACTGCCTGCCCGCAAAGAGGCGTGCTGTGCGGGCCGGCGCGGGCTGGCGCAGACCCCGGGTGATACGGTGCATACACGGGTAGAGTTTGAGCACACGCACCAGGACACTGTGTGCGCACGCAGAGGCGTTGATATCTCAGAGACCATGGCGGAACTTGCTTGAGGCACCGGGTAGTATGACGGAATGGTTTCAAGGTTCGTGGGGAAGGAAGGGTTTTCGAACGTGAACGCTGACGCGCAGCCTGGATGTAATGATTTACATTAAATAATATGTTCGTCAAAAGCTGGCGGAGGCGGCGAGGAGGGCTACATAACGACAAAGGAGTGTGAGGTTAACCATCAAGGCCATTAGATGTGGATGCAGAACATTTCGCGAACACCCGAGCCCAGCTTTCGAAGGAGTTTCACCGGCCGGAAACCTAAAGTGAACACGTCAGATGTACCCGAGAGCGACGATGACCCAAGTGTACTCACGCTTCGGTTTGCCAGCGGCACCGTCAACAGTTTTTAACTCATCAACTTCCGACGCGTACTCGCTGGCCGTCCCACTCAGGCAACTGTCCGAGTCGGAATCAGAGTCACCGATGTATGGGTCTTGC

>AT15923

CTGGGGGGCAGAGCATCCAGAGCGGGGAAGGAGAAGGGCGGGCGTCGGGAGGGGCCTGTGCTACGTCCTGTTCGTGGCCGGCCTGGTGCTGCATAGCGGCCAACTCAACGCGCACTAAATAGTGACCTCAAATGACGTCTCGATCAGTCCGAAGAGGGCCGCGGCCTTCTGTCCGTGGATCTGTCAGCGGGGTCCGAGCCGGCATGGTGTACACAGCGGGAGGGGTGGGGGAAGCAGCGCCGTGAGAGCCTTGTGTGCAGGGACTCTCCATGATGCCGGGAAATTCTACGTCCTCGATATCCCAATCGGATGGGTACGCACTACACGACTCGATATCGAGGAGTGCTCCATGGCCCGTCGC

>AT15945

GCACCGCATCGCTCTGGTTCCGTTGCTTGTCCTCGTTCTTCGCCTTCGTCAGCGTGCCCAAGACGAGCCCGAACATCGTCTTGCCCCGCTTGCGCTCGCGCGGCTCGACCGCCATGTCCAGCCGCGGCCGCTTCTTCGTCTTGTCTGCGCCCTCGGGCGGGGCGAGCTCGGCGGAGACGGACACGGGCGTGCCTTCCGCCGGTTCGGGTGTGCGTTGCATTGGTCTATGGTAGCGAAGAAGAGGGCGGGGAGAACGACGTTAGTGAACGGGAAGTAAGAGGTTGGAGATAGGAATGCGAACTCTTCGGTGGCCATTTGGTGGGATATGGGTCCGGGGAGAGGAAGAGTGTGGGAGGGAGAGGAGAAGGTTGTGCTTCGTTGACGGCGGGAAGTCGTGACGCAGTCAATTCTTACTTGA

>AT15951

GCCTCGACGATACCCGACCCTTCTGTACCGGAGATGAAAGGGAAGGAAACAGGTATTATGTTGAATTCCTTGTTCTTCCAGTCGATGGGATTCAACCCGGCTGCGATGATCTTGACGAGAACGTCTTTGGGACCGGGTGAAGGGATGGAGACGTCGCCCAGTGTCCACTCGCTGTTCTTCTCTGGGAGGATGAGAGCCTTCTGAGTAGACATAGTGCAATCGAATACACAGCTGGGTGAAGAAAGTTGGGCTAGAGGTATGAAACGGATGTTGCAGTTCGGTCGCCACGTCGCTCACGTATATGTAGGTTGTCGCCATAGAGGTGCCTCCACCCGACAGGTCAGTACCGGTCAGTACTCAGTACTCAGTACTCCCGACCGGCATAGGTGAGAGCAACCGATTTTGCTGCGGATCAGCCGATTTTGATTACTTATTCATTCGTAACTCGTCGTCGTGTCGGAGGAATGTGGGTACGGGTGTCTGATATAACTCAGACGTTAGC

>AT15962

ATAGTGTCATTGATAAACGTCCGTATGCAAGGACGTCTGCGCGTACAGCATGAAGTCCAAGTATTGTACCCACAACCTCGGACAAAGTGCGGTCTTCTGGCTCCATTGTTGTATGAAGAGCAGAGTTCCGAGGTGTTTGCAGGGTAGATTGCTATGTTGACGCATACATGAGCGGCCGATATATGATGAGAGAGAACCGAAACGACATACACCTAACGCCACACGTAGCTTTCCCTGAAAGACGCCAACTTCGTTGAAGACGTAGCCAGGTCAGAGCTCGTCGTGTTCGTGCGCTGGCTTGAGTATCGCCCCAGAGATGTTCGCGAACCAGGCTCGTTGGTCTTTAGGCGATGCCCAATCTGCAGTATTACCTG

>AT15985

GACGCCTCTCTGCCGGTGACTCGGGATACCAATCCGCGTCTTCTGGGATGTGTACGAAATGGAGATCCCTGAGGTTGGGTACCTTTCCCCGCAGGACGCTAGGAAAGAGGGCGAAGATGCTTGTTGTCGTGTGGAGGTAGCATCCAGTGAGGATCAGTTCGCGCACGTAGCCGGAGAGATGACAGCCGAGGCCGAGAGCAATGGAGAACCTGTACAATGTCGCCGCATCTGAGAGGGCGACAGAGTGGAACAGCACCATCTGTGCGCGGACGCGCCATGTTCGGCATACGAGGGCACAGTTGCGAAGGGCGTCGCAGGACTGTAGTTGGTCGTCAAGCGGGGTCGAGTACAGCGTGTCTATCACATTTTCGAAGACTTCGGTAGGGATGTGACGTACGTGCCAGTGCTTGGGCCATGGTGTTATTGCATCCCATGCATGCAGGCGAGACAGAAGGGGGATGAGACGAGCCTAGCCTA

>AT15999

TCAGCGGGGCCATGACCATCTGCGTGGCGTTCATACAAGTGATGATGCTGAGGAACAGGGATTGTTGAGGGACTTGACACGAAGATAAACGTTGGCAGGGGCTCACATGAAGCAGAAGCACCCTATGTCGCGCGTTAGCAGATATCGATGAGTCGGGAACGGATGGTCCGTACCGTTACGCAGCATGACCTGAAGCACTGACGGTACGTCGTACAGCGCATGCTTCAGCCGATACACCGTGTATGTGTTCATCCAGGTGACAATAAGCACGATAAGGTCTCCCATAATGAGACAGCTTCTCGACGCTATGGTGACTGCAAAGGGTGTGTTTTC

>AT16006

AGGGCCGTAGGGATGACGCTGGGCCGTGTGGCTTCGTATTCATATTTGCGAAGCAGAACAACTTCGGGATATCGCACAAATCAGGATAGACGAGAGCAGCTGACATTACATTAAGCATCACAAGTCCGTTGGTCCCCTGAGGGAGTGTCCATATGGCATTCAAAGCCAGTAAAACGAACGTATGCGCCCGCCGCACAAACATCTAGTCTCTAGGCAGTGACTGATGTGTGTGCGCGAGGCGAGCCGGATGGGGCTTCAGTCGGAGCACATTAGGCTAGTTGACCTCGCTCTTGGTGAACTTGGTGTTCGCGAAGCCGACGGTGTTGCGCTGAGGCACGTAGACGTGGTAGAAGCGCTCGAGCCACGAGTAGCCATTGATGAAGTCGAGGCCCTCGCCGCTCTTCGAGCCGAGGTCCGCGATGACGAGGTAGATGCTGTCGTTGTCGCCGCGGATCACGGTGTTGA

>AT16025

CACGCGCGCACCATAAAGGCGGTGGTTAGATCAGTTGATCTCCGATGGGCTTGAGGAATGGCTGCGGTCCTGCAACGATCTCGACGCGGTTTCCGCCGCATTCAACACTGCTGAGCTCGATGCGTACCAGTGCCTTGTGCTTTATATATTATGAAATGCGTCTAAATCCATGATATTCAAGCGGGAGTAGGCAGATCGGCGATGGCGAGAGCTAAGGGTAGTATCACCAACTTCTCCTTGTCCCACTACAGATGGCACCATCAGGAAAGCACATTGAGGTGAAGCGAACAATACACTCACCACCCCGCTCGTGTAGTACACATCGTTCTCCATGAGCTTGCAGACGGCCTCGAGGCTATCGGCCTCGCAGATGAAGATCGACCCGACCATCTTCTTCTCTGCGCCAGGAACGATCGACTCTGGCGTGAGCATCGCTCCTCCGACCTCTGACGAAATGGATGAGTGGTCAGCTCATGAGTCGTAGGGGTGCAGACGGGTGAAGGACTCACTGACGATGCCGTTCCTGGCCTGCATCGTAGCGTTGTCTAGGTGAGACTGACGGACGTTCAGCCTACGCTGGAAGGCACCCTCGTCGGACATGTCGGGTGCGTAAACGATGAACTTGGGGCGCGCTTGGGAGGACATTGCGCGATTAAGAGCGGAGAAGTGAGGCCTCAGAGATCTGCATGTCCTCAAGCTGTATGTCCTTTGGAACAGATTATACATGTCGCGAGATGGCGTAGGCTAGGAACTGGTACTCGTGAGACACCTAGACAACGCCCGAACCA

>AT16175

ACCCTCTCAGCGCTAACGCCCCAGAGCCTTCCAAAGTATAACGCCGCGACTATCCGCCCACTGCCAAGAATCTCCGCTTTGTCGACGACTGCATAGTAGGCCACGTTGACGAGCATGTACACCAGTGTGACGGACAGCATGGCCAAGGGTGCGGCTCGCTTCATCGTCTTCACCGGGTCGCGCACCTCTGCCAGAGCATAGTTTGCGTTCGAATAGCCAATAAAGCACC

>AT16176

GTTCTCCAACTTGAAACCCGGTGCCCGGAGCAGCGAGGCAAGACCGCTGAAAGCAATGCCAACAAGCACCAGGATTTTGAAGATTCCCAGGGTGTTCTGTAGCCGGAGGCCTGCTTTGAGCGCCGTGCCGTGCAACAGGAGAGCGAACGTTATGCACAGGACACCAGCGAGACGCGTTGTGAAGGGGGATGGTATTCGCGTGGGGTCAAGAGCGTGCAGAGCAT

>AT16187

TACCACTCAAAGTTATCCAACAAGCCTGTGGTGATCAAGGTGAGGACTGCTGGACGGGTCCGAGAGAAGACGGTCCACGTACTCCAAGCAAAGTATGACTTGATGGGTACGCCATCCTCGGTGATTGCTTTCAATACAGCATTCGCATAACTCTCGTAATACTCGATTCGGCCTTCGTCGTGAACGACTTCCTCTACTGGACGAATGTTCTCGTCCTTCTCTGAGAAACCATTCTCCGTTACACTGGTAGAGGCCGGA

>AT16193

ACGCGGATAGTCGCTGCCAGGCTGGTGTGGGGCCGTGGGCGGGGGGACGAGTGAGAATAGAGTGCAAGCGAGGGACGTTTCTGCGCGCGCCGTCATTGCTGCGGGTTGGCCTCGAGATAGCCTGACTTGTCGACCGTGGATGTGATACCCTTGTCACCGCTCATCGCGAATGTCATGGTAGCCAGCGACCCTTCCTCTGATATGTGCGACGCGAATGAATTGAGGGCACTGAGGTGGCCGCCCTTGCGGTCGAGGGGCTGCAGGACGATGGGGCCGGTGTCGTGATTGAAGCGAAGAGTGCTGAGGTCCTTTGCTGGAGTCTGGTCCGTTCCCTCTGCAGTACGATTCGGA

>AT16208

ATGGCCCAAAGTGCGAAGACCCAATTTGACAGCATGAGAGGGAAACCGCATCCCTTGATGAGTGTGCGCTTCGTCTCCTGTGTACGGGAGAAGACAAGCAAGGTGCAGTAGCCGACTTGTAGGATGTATAGGACCACAATGTAGATCCCGATCATATGTGGTTGCGGTGTGATTGAAGTAGGATACAATTCAGCGATTCTTCCTGTAGCACATCAGATATAAAGAACGGGAGCTTCTCTGTTGATGCGTACCAAGGTTGGGATCCAACAGAAATGTGCATATCGTTATAGTGGCAATGTTGATGAGCAACGACAGCGGAGTCAGTACCTGTAGTCTGAAGCCCGCGTCATAAGATTTTTCCAGATGCTCACTAAACAACGATTTATGATACGCACCTGATGAGGGCAAGGTACTCTCGTACGGCCGGATGCGACGTGTCGATCGGTATGTTCTCCATCATAAGTTGGATGATCTCTGTCCGCCTTGGGGAAAGATAAGCTAGTGTATGCGCGAGACCGTGAAAGTCGTAAGGAAGGTAGGAGCTCCATGCCGAGGGCGAGGTCAGACCGGTGGAGGCAGAGGAAATGACATCATCGCATACA

>AT16211

GTGCTGCGGGATAGGACGCACCATCTCTCCATGCCCATGCCCGTGGCTTCGGAGTTGGCGCAGCGCTCCTCCCAGATGTAGACACAGCCTGAGGAAATACTGGAGAAGGGGAGGTGAGCGTTGGGGAATGAATAATTAAATTAGCGCGGGGGAGGAGGGGACTGACGCTCTGCGCTCCTCTGCGTCGAGCCTGCGGGTGGTCATGGGGAGGATGTTGCGAGCGACGGCGTAGAATATCTGGAGGGCATCGCGAGTGGAGCGTATGCGGATGTTTGTGAGCGTGGGGTGCTGCATGGGGTGCGCGGGGGGCGGATGATGTGGGTAGGCGAAGGGGCGGCAGCGGAGGCGGGGAGGAGGATTTATAATGGAAGTGGAAGCGCTGATAGGGACGATGGGAGGCCCTTCAAGAAAGGTTAAGCAGCCGCGCCAAACTGGCCCCCGGGTACAGCAGCGATGCGACGGCGGGCGCCCGCCCAGATGCAGTCGCCAGATGCCCCCCCCCCATCCAGATCCGTCCTC

>AT16223

GGCCCGAGTTAGGTGAGCATAGGTTCTACATGAATACTCACCTGTATACAGGTACGCAGAACTCACCGAACTCTCAGGTGAGGCACGAAGTCAGCCGCCGACTTGGACACGGGGTACAGTCCATGAATAATCTGCTGTAGCGACTCAATGGTACGCGGTATGTTCGTTGACCGTAGGTATATTCCGTCATTAGAGCGGACGACATCGGGT

>AT16228

GCCATTGGTAAAGGGTGTCGGTCGTCTTTGCGTTTATGTTTGGGTCCTTGGAAGGGTCATTGTACAAGGGGAAGCTGGTCTTAGGAAGGAGTAGAGTATTGCTCCAGCGTGACGAGGGTTGCTGGGTCTGGGACGCCTGCGGAGAGGGTCGGGAAGTCGAAAGCCATCTACGAGACCATGGATGTATAGAAAATAGTCGGGGGCCCGCCTTGACAAGAGACCAGGTGGGCATGGACCCGGGAGACAGCCAGGGAGGGAGGGAGCACAAAGCAAGCCAGCGCGGC

>AT16230

GGGCGGTCCAGGTATTGCCAGTACTCGAGGTTAGGTATCGGTTCTAGGAGCGATGCGGGAAGAGAAAGACGCCTGGAACAGACCGAGCGTCGGTCATGGATGATAGAGTGGAGAGGGCAGCGTGGACGCACATAGCAGCGATGTCCTTGACTCCGAGGCACTTCTTGACAAGCTGGACGATCATCTTGAGCTTCCCGCTCTCGCCGGTGTCGCCAGAGTCTGGAACGGAGATGGGCTGGCCAGGAGGATCCTCTTCAAGGGCATTAATGGCGTGATTCTTGTCTAGAGATGCCATGGCGAGAGCACAACAGAAAGAAAAGGAAGAGAGAGAGGAGAGCGGCTCTTAAACCGGGAACGGGAACGGGAACTGGAAAGGGAGGAGGCGGAAGACAAGGAACGTGGGCACATCCGTTGCAGCTGCGACATGTCGCCAAGCCCCTTGCACCATGCCTTGCACAGATCCCCGCCCATCATGACCACGCCCTCTGTAAAAATAAATAGCCTTCCGCATCCCGCCCCACCCAGTGCCAACCACGCCACGCGCTGATATATACAGGGGACCAGCCGCAGGCCACAAAAAATTGGTTCGACCCAGGCTCGAACTGGGGACCTCCTGT

>AT16253

AGGTTGGCATGAAGCGCAGGAATGTATAGGGTTGGATGTTATGTAAAAGGTAAACGCTGTGTTGATGAGAGGTGAGAGGACGCTCAGTTATGCGGTACGAAATGGACTTCGACGGATGTGTGTATTTCTGTTGAGGATACAAAATATACATCTACTAGTGTGTAACAACATGCTATAGAGGGACAGGATACACAGTTTGGACTTCGTATTCAACGCGCCTGCTGGCATTGGTGCGGAGATGAGATGCTACAACTTGGGGAGGGGGAGATTCATTTTCCTGTCATGATTTCGGAGCGGCAAAGCGGACAGCTTGACGAATGGCTCGTGTTAAACCACTGCAGAAAAATGTCAGGCTCGATATATCTCTGAGCTCAGGTGAAGGGGTTACGAACCTTGTAGAGACAAGCGGCGTGGAAGCGGTTCTTGCAAGTTTTGCATGGCTTGC

>AT16256

GAGGAGGGGACCGTGGACAAGATGATGAGAACCGTTGGGTATCCTTGCACCGGAGATCCAGAGCACCCTGTCGCGAGAAACTGGAGGAACTCCGTATATGCTTGCGATGGCCCAGATGGTGAAGGCGTGAGCTTTGGAGGCGACTTTGGAATTTCTTCCTCGTTGTCGGAGTCGGAGTCTTCCTCGTCCTCCCGTCCGGCTTCCGCTTCCAGCGTCCATGCATTTGGATGATCTGGTTCAATCAGCCACAAATATATGCGTATAAAATCGAGGATTCCGCACCCTTTAAGAATACAAGCAGCGGTTGCCACATCGCAGCTCTGACATTCGGGTCGAGTTCTACCCAAGCAGATCGTAGCACTGCACCACTGAGGACGGAAACGAGAGGGTCCAGATGCGCTGTGGAATAATGAGTTAGCATTCCACATTGTTATGCCCTCTCCACGTCTACTTACCCTTGCATCTTCGAACCAATGTTTGGAGCAGTGACCATGCCGACCGTCTCACGACCGGCTGCTCGAATCCGAAGCTTTCAGACTCAACGAAGGGTGGCGTCTTCCCGCAGTAAAGCGAAGACCACAGCGCCGGGTTCGCGAGCGGCCCCAAAAACGCATCCTCCGTGCTCCCCTTCTCCGCACCCTCGACCGCATCCTCGGCTTTCCTCGATGTGGCGTACTTCGCATCT

>AT16257

GCGCCGAATGCACCCACGCGCAGCCGCGCCTTCCTGTCCTGCTCGTTCTCCTCGTCCTCGTCCGCCTTCGCGCGCACGCTCGGTTCCTCGTCGCGCCGCACTGGGGTGCCGCGCCCGCTGCCCCTCTTGCCGGGACTCTGGACGGGGACAGCCGGCTGTGGTGGATTGATGTAGAGGTAGACCCCGGCAGGGTCGAGAAGCGTGCGTTGGGCGAAGTCCCAGAGGCGCGGGAGGAGAGAGGCGTCGAGGAGGAGCTTGTCGGAGGAGGGAACTGAGGAGATAGAGACGTACCGAAGCCAGGAGTCGCGGGCGAAGGAGGAGACTTGACGATCGACGTCGTGAGCGGCGAGGCACCAGGAGCCGAGGATGGATTCTGCTTGGTCGGACGACGCGACCTCGCGGATCTGGAAGAAGATCTGGGTGCGGAGGTTGGCGATCTGGAGGAAGGAGGAGTGAAGGCCGATGGCCTGCAGCCTTATTCGTCTTGAGGGGTGTAGGAACAGCGCGGGGATGTGGTGTAACTTGGACATAAAATATTAGTCCAGGCATTCTAGAGAGGCGGAGAGGACGTACCCAGACAGGGATGATTTCCTGCAAAGTGTAGATGAGGAATTCTCCCTCCTCGCCTTTCCTTGCGCGGCCCAACCAAGCGGTCTGCAAATCCTCAAGAGCGCGGCGTTTGGTAGTCGCATCTTTCTTCCCGAGTAACCGTAGCACGACGACAAGTTCGGCAGGGAGCTTCTGAGCAAGACCAAGAGTATCCAGAGGATCGGGAAGCACCGGAGCAGGCTTGACTGGAGGGATGTAAACCTTTTTGCGAGGCTCTTTCTTGTTCTTCTTGTCCTTGCCCTTGGGCTTCTTCTCCTTGAGAATTGGCGGCTCTTCGACGACGCCCTGGGCGGTAGCGGCCTTGCGCGCATGTTTCTTGCGGGTGCCAGAGCTCGCGGAGGATTTTCCCTTCGGCGCCATTGTAATCGGGATGTAAGCAGGGAGGATGGGGTGGGGGTTAAATGAGATGCATGAGGGACGGTGCTGGGCATACGCAGGTCGTTGGTGATCGAGTCTGGGTGAGTCCCTCCCAAACCGGCACCGCGCCTGATTTGGATGCTACTATGACGCAGTGGCAGACTCGGACATCCGATCGTCCCTTGGCCTTGGCTTGCCCAGGGCTTGCCTCTGCCTCCTGCCATCGTCGACTCTCCTCGCTCTGATCTCGCTTCTCGCAAATCCCCCGGCTTCGAATCAACAATGTCTAAATGTGAGCGACCGCCACATCCTCGGGGAGCATACTTGCTAATAGCTCGTAGCCCAGTCGCAGTATGCAGCTACCTCGGAGCTTCCGCTGATCAAGTCGCCCAGTTTACGGCTTCCTCGACCT

>AT16258

TCTTCTGTGTCGCCTATGTCATGTACTACCAAGCACGATCGCGCGGGACTCGTAGGGTATATATGCCTTTATGTTGTAAGTTCTTGCTTCGTCACGTTGTATCAATATACTTAGCGCAACCCTCGTAATGTTTCCACGGACAAGTCAATCAGACTGTAGTTCCAGGTGTCCTTTCAGACCCATATGAATACAATTTTGTGACGTGTGTCGTCGCATATGCATGAGGTGTGCATACTACATAGTGCGCGAATGGTAGATAGTGGCAGCGATGATACGGATAGCGGGACAAGGACAAAGAGACGATGCAGAGGCGATTACTGGATGGAGAGGGTCAGCGTGGCTTCGAGTCATGTACGCATATGATGGTACTCACGAACTGGAGCGCAGCACCCTTCGTCTTCTTCTCACCACCGAGCTCGAAGCTAGACGACATATGTGAACGACTAGAGAGAATGCAATGACGATGACGAAGACGTACTGCTTGCAGCGCTTGATAGCGAGCTGCATCTTGTATTTGCAGACCGTGCATTCTAGCCGAAGTACGATCTTCTTCGTCGTCTTCGCCTATACGTCAAACCGATCCGTGCCATGGTCCACCCGTCAACCGTCCATTCTCCTTCGTCAGGCAGCGGGGAGACGCACCTTCTTGTGGAAGACGGGTTTGGTCTGACCACCGTAACCCGACTGCTTACGGTCGTAACGACGCTTTCCCTGCGCAGCCAACGAATCCTTGCCCTTCTTGTACTGGGTCACCTTGTGGGGCCTGG

>AT16274

TTACTATTGCAAGCATCGGAAGCTTTTAGAGACGCGAACTCGGCGTTCAATGTCTGGGCATCCTGGCCGTTTTGAAGGAAGACAGCGGGATCCAGGGACGCGGTGGGGGCGCCAAACGCAGTGGAGCCCAAAAGGACAGCGAACGTAACAAGGTAGATAAAGCGCATTGCGATAATGATAATGGACGGAGAGCAAATTGAAGAGGATATGGGTATGCTGTGGAAGAGAACGTGGAAGTTGAGGAAGCTCCGGGCTTCAAGGAGAACGAGAGACTAGCGGAATCCGAGAGCGAGGATCGAGCCCGAGCACAAGAGGAGGACGAACTGCGGCAGCGACGGGGCGGCCTGTTATATATATGCGGGAGTCGGACAAAGACATGGCGACGCCGACCATTGTTCTTGTCCTGCCCAGGTCGCGTCTGGACCAGGACAACCCCCAA

>AT16276

GGCGGGAGAGAGAATATGGTAGTCATAAAATAGATATAAACAAAATAGATGCTCGCAACGCGGCTTACGGAACGACGAGAGGCGGGTGGAGGAGCAAACGACGGAAGAAACGACGCACGAACGCGAATGAGCGAACGAGCGAAGGCCGCCATTCTCCTGATGGGCTGGTGAAAGCGAACGGGCCGGCAGCGAGAGCCTAGGAAAGTACTTGTCCCGGACAAGTACTTAATGGGTATAAAAGTGAAAGCGGAAGAACCCAGCTCCACGTCATGTTCCGCGAGAGGTTGGTCACCCGCAGGTACGATTGAAGCCGAAGCCGCAGCTATAGCTTGATGTGGCACTGGTAAAGGCTGTCGTCCTCGCCTTTCCACCCAACTGTCAGTGTCAAGATTCGA

>AT16294

TAACGCGAAGGCATATGTGGCAGCGGAGTACTTGGACCAACTCATGTTCTCTGGTGGGAAGTCTGTGCCGTGCCATCCTGCATACCAGGTAGTAGCAACGACCTCAGCGCCGTTGTTGCTTCCAGATCCAGTTGCGCCAACTGTGGTTCCACTAGGCGCCGAGGCTGAACTGGTGGCTGAGGCGGACTTGGGCTTCAAGGAACACATGGGCGCAGCCCACACTGCAGAGAATGGGAGCGCGGCGAGGAGGGAAAGGGGGAGGAGCATGGTTGTCTAGGGAGGGGAGTGTGTAGAGAGAGGAAGGGGTTACAGCGCACAAGGCGGATGGGATGAGAGCAGGGCGCGGTTGGGCGGGCCTTTATACATCTCACTTGGGGCTGGAATACGTCCGGACGTCAGCCGCTGGACGCTTGCTTATTGACGGAGGGAAATACCGTTTGTACGCCGGGCCATTGAGTCTGGCTCGGACCGTGGCATCCTCGAGGGCGGCAACAAGACGCCAACGGTGAGAACAATTGAACGTGCTAGCGGCGTGTAACCTTACAATAAACATTCGCTTACCCCTTGCCCAATTTAACACAGACTTACCCTGCAAGCAGCGTGTGTATGGCCATGCCGGCTATGTCCTTATCCTGTACAGTGGGCTCGAGCCTCAAGGCCAGGGGAGGCGACGGGTGAGGCGAGTGGCCGTGCACGGTGGTTCGGAACAACAAATGCAAAAATAAGGTGTTGAGCGGTGATGCAATCACCGACTCGGAGGACGGAGAGCGCTTGCAGCTGCGACCAGCTTGTCTTGACCACCGACGGCAACGATGTCCACCTCC

>AT16297

AATTGGCTCTTGAAATCCTTCATGATGTCCAGAAACTTGTTATAGACCTCCGGTTGCTCCGCAAACTGGAGTTTGACCGAGTCCAGGTAGCTGAGCGCGTCGCTCACGTTCAGAGGTCGTTCGCCTCCACCTTGTTGCTCAGGGGATCCTGCAGAGGCGGGGATATGCCCATCCTGGGGGCCTAGTGAAGTCGGACTTTCGAGAGATTGGCTCGGCGGATCGGTGTCCATGGATTGGAGAGGAGGACGTGGAGAGGTCGGCCTACTAGCACGTGTGGTGGTCATTCTTCCTTCTGTAGGAGCTGGACCATCCATAAAATCCGACTGTTATCTCGTCGTACGAAAAGTTCTACGGGGTCGAACACTGAGCGTCCTCCGGGAGTCGGGATGATGAGAAGCACGCACCGGCAGGTTCAATGACCGCTCCTGAAGCCTGAACAACGTCCACAGGGAGATGGGTGGTAGTAGCCTGTTGGGCTCGTGTGGAACTTTGAGCGACCGTAGATGCGCAGAGGATGAAACAACGAGTCAAAGGCTGTTTTACGAGGACAAGAAGGCCGACGGTGGCTGAAGCACGGCGGGGATTTCCAGATTTTCGACGGCAGAAAAGTGAACGAAAAGGGATTACTCGCGATTAACTTTTCTTTTGCACGTGACGAAAAGGAGAAAAGTCGAGCCAAAAGGGGAAGAAAGTTATCCTTTTGGGAGACTTTTGTGCCACTTTTCTCCAATTCTGTCACGTGCAGAGAAAAGTCTTTCACGGGTAATCCCTTTTTATTGCC

>AT16299

CACTTGGGGCTAATGGTCTCGCGTCGCTTCGCGTCCTGAACATCCCAAGCCTTGCGCATGATAGTGGTGAAGATGTCCCCGAGCGGCATGCCCTCCGTCCACTCGAGTTCGTCCGGGCCACCGAGCCCGTGGCCATTGGTGAGCTGAGGCGCTCGCCGGGACTGCAGGGACGGGCCCTGGGCCGGCTGAATGGCTGGGGATACGTGCTGGAAGTACACGAGATCGTGTAACGGACGCGTCGCGATCAGCTGCATTCGTATATCATCAGACTAAGGAATGGTTCATGAGATGAATGCCACGCACGCCCTGGAGAGTCGAGTTCATGAAGCAAGTGTTTCCATGGTTGATGAGCCCGGGCGGCAGACTTGCGTCCTTCTCTTCCTCTTCTCGCCTCTTCTTCTCCCAGTATATCTCCTGAGGAGTGTGCTTGCTCTTGTGCTTGATCATCGATGGCCTTCCGGGAGCACAGGGTAACTGACAGGTGAGGAAAAGGCACAGCGCACAGTGGGCAGAAGGGGACGTTTAATATAGCAGAATGGATGCGCAGCGCCGGGGCGGTGGGTCGGGAAAGCAAGGCGAATGCGGATGTGCCCCGCAGCGAGGTGGGGAAGGTGGCGTTTGGCAGACGAGCCGCACACGTCGCTGCTGGCGGTGGGGTGTGGTGGTGGACCAGAGGAGGAGGGGAGGAGGCAAGCAAGGGTTGTGATGGTGGGCGCGTGGAA

>AT16312

ACAGGTGTTGGCGTCCCGTCCGGAGCAGGGAGGGCACTCATAAGCTCGCGCATTTCCGCGTTCGTGAACAGATCCTTCTTGAGCCAGCGGTTCTTCGCAAGCTCGATAGCGGCACGACCGAGCTCCTCGATGGGCGTGTAGAATTTGGGCACGAAGTTGGAGTATATCGGCGTGATTAGGTGGTCCGCGATCCGCAGACCGAACCCGCGCTGCATCATGCGCTCCTGGCGATACTCCTTGCTGTCGGACGGGAAGAAGTAGCCTGGGCGGTATATGCACGCGCGCATGCTGGAGCCCTCCAGGAGAGCGGGGAGTTCGCGCTCAACGCGACCCTGTGCGCATTGGGG

>AT16313

TCTGTATACTGCGCCTCGGACATCCCGAGGGAGCTCTTCCCCAGGGCCCATATTATCGCATCGTGTTCCGCGAGGTTCTTGGCGAGCTCGGGCGGGAATGTCTTGAAGTCCTCATGGAGGATCACTCGCGTTTTTGCGTCGGCGTTCGGAGGGAGCACAGCCCAGGAGGGAATGGTGCGCCGGGTGAGCAGGGTGACTACGGAGACTTGGGGGTCGGCGAGCGCTGCGCGGTAGATGG

>AT16314

AGGGTGTGGGGAATCTCGACTAGTGTTCGGGGCTTTTGAAGGACGACAGGGACTCGGTGCTCCAAGTGGGTTGTTGCATTTATTCGACTGGTGTTTGCACGCTTTTCAACGCCCGCGGAGCCTGTTCATGGTGCCACTCAGACGCGTCATCGCGCCGATAGAGAATGGCCCGGCTACGCTGGCAGTATTTCGAAAGGCATTAGGCAATTACAACTTTACGGCATCCCGCAGACAGTGCCTTGAATTGAGCACGTCGGAGACGGGGCCATATCATGAGAGACACGGCGAGGACGATTGGTAGGGGTGAGAAAAAAAGACCACGGAGGCTGAGGACGAATCCACGATGAGTGCCCAATTCGTATTCGCACCAGGAATATTTCTTCGAGAACGACAATATGGCTATGTCTGTCAGCGACCCCCTCC

>AT16334
[truncated: 426,748 more chars]
