# Supplementary material for: Genome-wide Identification and Characterization of Natural Antisense Transcripts by Strand-specific RNA Sequencing in Ganoderma lucidum
Source: Sci Rep. 2017 Jul 18;7:5711. doi: 10.1038/s41598-017-04303-6 (PMC5515960; doi:10.1038/s41598-017-04303-6)
Supplement: Supplementary file 15 — Supplementary File 2 [file 41598_2017_4303_MOESM15_ESM.doc]

>AT10044

CGGCCGAGTCGCTCACAAAAAGCCTGTTTGGAAGCCCTGCACCGGTCTTCAACTCGACGAGCGTAGCGTCGTCGACCGCACAAAACCGTTTCAAAATGCCGAGGGTGATCTCCTGCCAGTGCTCGGAGGGCGAATGCTGGGAAACGTCAGGGTGCGGATCGCCGGTGGCCGGGTTCGACTGAAATTCGCCGTCTTGGCTAAGCGCGTCGCGTGTCCACACTTCGGGTGAAATGATTGGGACGGTGTAGTATAATCTAACTCGGGCACCTGACGGGACAGGTCTGCCGGCCTAATTGGCCCGCGCATCGGGAACGCCAGACATGCATGAAGCAGCGGTAGTCGTCGCGGGCCTGGGCCATCACCTAGGTATGCTAGTGAGCACTGAAGAACGAGGGGCCGAGGAACGATGGGGATCTTTGACCTACCTTTTCCAACGATGGCAGTGCCAGTGTGCTCTTGATATTTTCTGCTCGTACGAGGAGCGTGTATGTATCCGCCGAGTGCTGACTACGTCTTCAAGTTCGAACCCAGTGTATGCTTATGCAAATGAGGATCGTGATATGATATGGAGGAGGATGATCGGCCCGTAATCTCAGGGGACTCTGAGACACCAACTGCAGAGAGCACTCATACGGCCAAAGGAGGAACGCCGCCCACGTCGACGAGAGCTGATGAAGTGCTTGCGCAAGGTCCGCCACCTGCTGCTCTGGATCCGGGGGTTCTGCGGCTTCGCCAGCTCGCGCGAGGTGTTGGCCAGATTAAACGCAGATGCGCGGTAGAGACGTCCAGTACAGT

>AT10045

TCGTGCACAAGCTCGACACCGAACCGTTTACACTCGCCCCTCACAACCTCAAAGACCATCGGATCGGGGGCCGGACCGAAACACACAACGATCCGCCTCCGGTTCGCGCTCGAGTCCGAGTCGTAGTCGCAGTCGTAGCCCTTGCTGCCACCCACTGTGGCCCGGCGGGGAACCGTGAAGATCCCAGCCTCCAACGCCTGACACACCCTCCACCACCATATCTGCTCCCCGGCGCCACGATCGACCGCTCCAGCACTCCCGGTTGGCCGGCGCAGAACGACGGTCTTCACGGCGGGCGGGAGCGGCGTGAGCGCCTCCAACCGG

>AT10072

CATTGTCGCCCTTCTCAAGGTTGGTGACCTCCGCGCCGACTTCCTCGACGACTCCCGCTCCGTCGACCCCGTTGACGAAGGGCCACTCCTTAACAAGACCAAGGCCGCCCATCACCGGGATATAAGCGTCGACAGGGTTGAGCGCCACGGAGACGAGCTTAATGAGAACGTCTGTCGGGCCTGGCTGGGGAACGGGCCAGTCAGTGACGAGCTTGTGGGGTTCCTTCTCAGCCGGGAGGATGAGTGCTTTCTGAGTCGTGGGAGCCATGGTTGCGGTAGTGTTCTCTGGAGGCTGGGTCCGAGATGCTGAAGTTCAGTACTGAGTTACGCTTCGATGCCAGTTCCTTATATACGCTTAAGGGATTCTCGTTACGACCTAGGAAGGTAGTAAGAGCGTCTCGGGCTTACGGACCCTCAACTTTGCCTTCGCTAGGAATACGGTGTGCGGACGCTATACCTGTGCGCGTTAAACGTATGTTGGCTCGGAGCTGAGGGTGGCTTTTAACCTGATCTGTTGGGCGCCCGGCGCGCGGTTGGACGGAATTGGCGGAAAACATGATCGGGAAGGCTTGTGGGGTTGCGCGGGACGGGAAGCATTGTCCTAACCATCGGATTTGT

>AT1008

GCGCCGCTCGATCGCGTTTATTCGGTACACTGAACAGCTCTATTCGGGTCCGAAAATAGCTACAGATAGGTGTACGCTACAGTATGCTACCCGCGGGCTCGTCAACAGCTCGCGCGCGGGACCGTACGGACTCGCGCTCGACACACACCCCGCGCGCGCACCCATATGTCGCGCGCGCGCCTTCCCGCTCGTCTTGCCCTGGCGCCCATTCAATCCGCGCGCTCCTCTGCCTCTGATCGAGCCCCGCGCGTCCGTCGCATGCGGTTCTAACGATGACAAACATTAGTCAACGTCATAGGGCGGGTGTCCGTATCTTCACATACCTGTATTATGCACGAGGCGCGCGCCGGAGAGAACAAAACGGAGGGAGGAGGCAAAGGGTAGCGTAGGTGGCACAGTGGGGAGATGGTGGGGGGAGGAAAGAAGATGGATGAC

>AT10085

CAGGCAGGTCCTTCACGACGGAAATACCCAGGTTCTCCAGGTCTTCCGCGGGGCCCAACCCGGACAGCATGAGGAGCTGTGGCGTGCCCACAGTGCCAGCAGAGAGGATGACCTCTTTGTTAGCCCCGCAGCAGTACATCTGTGCGGTGCTCGATGTGGCCATTTCGACACCGACCACGCGTGGAGGCGCCCCGGGAGAGGTCACGACATCGGAGAAGACGAGCTTCCGAATTGTGATGTCTGTCGCGACGATCAGATTATCGCGGCTCAGGACTTCATCGGTAAGATACG

>AT10112

CCTCAACATGAGTAACTGTGGTGATCCGAGCGCGCCACCGCACAACACAACCTCCCTCTTTGCTTTCGCAAAGTAGCGTTTCCACGCCTTTCTCGAATCCGTCGCCTCGAAGTGCACGCCGGTTGTGCGCACCTCCTCGCCTTCCGTCGCCAGCTCCACACGCGTGACGATGGTGTCCGTACAGATCTTGAGACGCGCTTGCCGCTCGAGCGCAAGTTTCGTTGGAAGGAACGCGCGGTCAGTCGAGTGGCGATGGTACTTCCTGTCCTCAGCGACGTCCATGGTGCCCGTACCGGCTGCGGGCATCGAAGGCGAGTTGAGGTCGGGGTGACGCTCGATACCGACTTTCTGGAGGGCACGGTGGACACTGAAAGACGTTTTCAGCGATGTTCTAAGCCACCTACGCAGAGTAAAGGAGGGCACGATCACAGCTCGCAGCCCAGCTGCAGGACGGACTGAGTGAATAACTGCCCCGATACGTTAAACATTCTGTCCATCCTTATGGAGCCCCTGAGGCAGGATAATGGGAATCCAATACTTGAGGGCAGGAGTTGAAAATGAAGCGATAGTGGTTTTCTCATAGAATTCAAAGATACTCACTAGGGCACAGCCTTGTATTGCGACACTGGGAACTGCTGGTTTTCCCATGGGCCTAGTACACGATAAACAACCCCTGAGAACGAAGGCAGGAATCGCACAATTTGGCGAAGTACATACCGTTTTTGCCTCGATATTTGGAGCCTGGTAGTGAATGGGTCTTCTCCGATTTGACGAAGTAAGGCTCGAGGTCTTCGTAACCCCAGCCTTCGTTGCCTAAGGCTTTCCATTGGTTGTAATCGCCAGGGGTACCTG

>AT10114

TCCGCAATCTTCGTAGCCTCCCCGACCAACTTGAACGTACGCCTGAGTTCCCCGGTAGTATCCAGCGAGAGCGTGTACGTCCCCGGATACAACCACTTGGCCCCGCTCTCATCCGCCCGTGCGATCGCGCCAAGCGTCACGTTCAGCTCCGCGACCGCGCTCTCGCTCGGGGTCAGTCCATGCACGCGCGTGTACGCGACGAGCTGCTTGTTCGGGTGCGGCGCGGGCCCGAACGTCCCATTCACGAAGAGCAGCGCGACGTAGTCTGACGTGGCGTTCCCGGTGTTAGTGATGCGCGCCGAGAACGTGTCGAGCGGCGCGAGGTCCAGGAACGCCGCGCTCTTGATCCCCCTCGCCACCAGCTCGTCGATCGAGTAGCTCCCTGCGTCCGCACTGGAGGCGTTGTCATGGGCGCCGGATGTCCAGGCGAACGAGAACGTGGTGTAGTGCAACCCGAAGCCGAACTCGAAGACGGGCGTGCCGGTGTACCACTTGTACGTGCGGCCGGGGTTCGTCGCGCTGGGGCGGAGCGTCATGTCTGTCATGGGCACCTGGTCCACGTACTCGGCGGGGTACTGCGTGATAGGGAGGCGTCCCGCAGGCGCCGCCTTGCCCGTGAGGATGTCGAAGAGCGCTGCGCCCCCGCTCTGGCCGGGGTATCCGCCC

>AT10282

CCCGTCGACTGGGACGACAGGAAGGTTCTAGGGTACTTGCGGATGATGAGCTTGGGCCGAGACTCAGATAAGGTTTTGAAGCCTCTGGGTACGCCGCTTCGGTTGACGGGATCGTCCGTAGGCTTCATGGCATTGAAGAGGGGGATGAACTGGCCTTCGCGGAGGTATCCTACATCCCCCATGAAGATCTCCCGACCTTGCGGCTCTGGGATCCAGAGAGGATAGCCGCCGTAGAGGCCAAGGTAGGCGTGGGCGTAGATATCCCAGGGTTTGGTCGACATCAGTGAGAGGGGTAAGGCAGGAGTGAGAGGAGTCGCGGTATGAACGTTGAACGTGCAAGAGGGTGAGAGGCCAGGGTAAGCTGCAAAACGAGAGTGAGGCCAAACGGCGTGTTATTGTACGGTAGGCTAAG

>AT10294

CGCATATGAAAGAGCTAATGAACAGTTCATTGGGTTGCTTCCGACTCTTGCGGGTATGTAAGTTCGGGGAAAGTATCGCCACAAAGCCCAGATATGTACGCGTCAGGCGTGTCCCAAGACTTTCAAGGGCTTGTACGGCTCCTCAAGATATTTTATCTCGTCCTCCGTGAGCTTGACGTCCAGTGCACCTGTCAACGACAATGGATGTTAGACCGCTCGCCCGGTTACGGGTACACAGACATAGCGACGGTTTGCCAGATGGAGACGAAGGAGATCACGCACCAAGAATATCCTCGAGGTTCTTCAGACTCGTCGTTCCGATGATGGGCGCGGTGACCTCGGGTTTGGCCAGGATCCACGCCGTCGAGATCTGTGCCATGCTCACACCTTTCTTCTTTGCAAGCTCTTCGACCCTACAATGCGCGCGCCAATCAGTTCTTCCCCCATACGTGGAAAAATGAAAGACGCGACGTGCCTTTCAAGGAGGGCGGGAAGGAATGTGATATCATAGGCCCCGAGGAACCTGCGAAGATATAGTGTAGCAAAGGTAGATAGACCCGAGGCGGGCTCACCCATCGGTCTTCTTCCTGATGGTCTCGACGTTTAACGGACGAGTGAGGAGTCCACGTCCAAGCGGAGACCATGGGATCGCGCCCACGCCGAATTGCTGAGGAACGCTCAGTCGTCGTTTTGTGCATGGGGCGAGACAGGGCCGGGGGCAGTACTTACCTTGAGCGTCGGGAACATCTCGCGCTCTTCCTCGCGGTAGAGCAGACTGTAGTGGTTTTGCATCGAGATGAATGGGGTGAGGTGGTGGGTGATTGCGTAGTCTGTGGCGGAGGAGATGCGTCATAAGCATCGGACTAACACGTGCGAGATAACTCTGAACTGTGTCGTACTCTGCATTGCATGG

>AT10798

ATGGCGCCGGGTGTGTTCGACTGGTCTGGGGGAGGACTTCCCACCTTTCCGGTGTGACCCCCAGCGAGCGGCGCGCTGCTCATCCAGACCGGCTACATTGAAGGTACGCATTTCTCTCGTTGGCATCGTCGTCCCTGACCGGGGTTGTATACCCAGTGTCTTTACCCACCGCCCCCAATTATGTAATGGTCAATCGGACGTCCGAACAATCTCGTACGAAGAATCCGCGATGCTATTTTGTAGCCGTCATATGGGCGGTACGCAAGGGTTGTGATAGATTGTTGTTCGCATATAGTACAAAACGCGTTTCTTAATCCAGGGAAATACTCGATCGTGCCCCGTACAGTGGGCCATGGAGGTCACCGCCTTCACGGAGTCTCGGAGATATGGACGACCAGCTTCGTCCCGCTGACCTGCCCCGTCAGCAGCTTCGCGCACGCAGCATCGACGCCAGCGAGCCCGTTCGGGACGACCTCGATCCTGTTCGGCTGAACAAAAAAACACACACCCATCAGCACACCCTCACCGCATCAACGACCGCACGCGATCCCGACCCCGAACTCACCACAATCTCGCCGCTCTCAAGCCACCCCGGGAGCCTGCTGCAGACCTCGTTCCCGAGCTCCACGTTCCCGGGCAGCGCGTAGCTCCCGAACGCGCGCGCGACGCGCTTCCCCTCGCCCTTCCCGTCGCTCTCCGCCACGAGGTCCGCGATAAATTCCTCGTTGCGCGGGTTCACGGTCACGAACGCGCCCCCGGGCGCGAGCACGCC

>AT11136

CGGCGTTCTGTCATTGGCCCAGAAAGAGGGCAGCGTTGTCGGTCGCTGAGAAGGAAGACCGCCAACGAGGGCAGAGCTGGCATTTGACGAAATGCAACTCCGCGGCCGCGAAATATTCGTAAAGACAGTTGCGTCTGCGATAGACAAAAGTCACGGCTAAACGGGAGGGCCAGGAAACCGTGCGGGCTGAGTACGCCTGCCCGAGGAATGGGTACAAGTCGAAGTGATGTGAAGGTGCCAAAAGCAGTAGCCAAGGGGGTACGCATCCCGTACGATGTTGCAGTATGGGGAGATAGACTGCTCCTGACGGTTAACGACAGGCACGAAGTAGCTGGAGTAGCGAGCCCCACCCACTACCAGCCATTCCGTGGTAGGAGCGTCGACTGGAGCATGGTGCCTTGCGAGAATGTTCTCCCGAAGTAAGTCTGCGTAGGATCTATTCTCGGGGAGCTTACTGGTTCACTGAAGCATCTTCCAGGCTCCGATCTCCTCCCGCATCTTCGTGCTAAGCCGTCCCTTGACGAACGTGCCGGTGCCGGTATTGGGGTCGATGACGAGTTCGAAGAAAGGAATGCCGAAATGAGTACGAGCCTCGGCTCTAGGCCGGAGGATGGCGGTCGCGCAGGACTGGTGAAGGGTCGACGAGTTTGCGCAGGACATGATGACGAGGATGGCAGAGGGCGCTGCTCAACAGCACGGCAAAAGGGTGCGAGTAGACAGTGGTCGTAGGGGCGCAAGGCCGGTGATATGGCAGCCTCGCTTCGGAGGTTATCCCTTAGCGTGGGCTCGATCTAAATGGTCAATATGTGAGTGTTCTGTCATCCACCTGTGCCATAAACGATCTATCTGACCGATGTACCAGTGAACCAGGGTGCCTCTG

>AT11153

AGGTCTTCAAGGGAACGGCAGCACATTAACATTCACCAAACACTCGTACGTATGAATAAATAAAGAGATCGCGATTATTTATGCAAGGGGGCATAGGATCGCATCAGCAACACATCGTCAAAGCGAACGCCGCCTATTTGAGCCAGACAGTGTTCTTGACCGTGTCGGCGACCTGCTGCGCGAAGATGTGGTGGGCCTTCCCTGCAGAGTCCACAACGTCCAGTATTTAGCAAACGGGTAAAAACACATGGAAACTTGAGGAATCGACGTACAGCCGGGGTGGAAGTCGTTCCCCCAGAAGTCCTTCTCGGCATCGCCGAAGTGTGTGATGTCCGAGAAGCCGTACTTCTGGAAGTCGTTCAGGATGTTGTTGAACCCGACGTTCGCGTCCCAGATCCAAGTGTTCACCTGTGCCAGTGCGGCGTCGGTGTCCGTTCTAATGCGTCAAATTCGATCTGAGACCGGGGGGGGACTCACTCCAGAGTGCTGGGCAGCCCAGGAGTTGACTCTCACGGCGAGCCTGTAGTTGTACCCGTCCATGACGCTCTTCTGGAGGTTCCGCTCGCTGTCGCTCTTCGCGAGCAT

>AT11156

CACGCGCCGCGGTCGCCGCCGTTGTCGTAGCTGTTGTTGATGTCGTTGATGCCGAACCAAATCGAGAACATCGCGTTCTGGCTCGTCCACGGCGCGGTCGGGGGCTTCCACCACCCGACCTGGTCGAGGAACGACTCGACCTGCTGGATGAAGTCCTGCGTGGCGAGCGTCGGCGGCACCAATTGCGTGTTGATCGTCGCGCCCCCGACGGCGTAGTCGTACGTCAGGATGAGCGACCTGTTCAGCTGGACCGTGTCGTAGTCGATCCAGTTCGAGCCGATGCCCGTCTGGCCCGGGTAATCGGGGTTGCCGAACGGGTTGCCGGGGGCGGGGACGGTGCCC

>AT11196

GGGAATCGCTGACAGAGGCTGAAGTATTGACTGCCTCGGTGATAGTCCCGAGGGTCCGCAACACATCTGCAAAACCCTGACGGGTAAATCTGTCCTCAGACAAGGGACGGATTCGGAGCCGCGCATCTGAGTTTTGTGGTTTCCAGGAAGCAAGCAACTCTTTCGCAAATCTGGTGTGTGTATCCGTGGAAAAATGTGGGCCAAGTGGAAGAGTCAATACCCGTAGCCCGGTGCAGGAGCTCAAGTCAATCCTTTGTTCTATCCC

>AT11210

ACTGAGATTGAATGTGCTTCGCACGGTCCGGGGGTGATATGTTGGGTGGTCGATGGAGAGCTTCGTAAGCGGCGAGCCATCGAGCGCAGAGAGCAGCCGCTCTGTGCCATGTGCGTTGAGTAATGATACCTGAGAAACGAGCCCCGAGGTAAACGTCAGTCACCGAAAACACAGCAGAAAGGCAATTACTGACCGTCATGTCTTGGAGTTCCGGTAGAAATTGTTTACACCGCAGCGCAGCTGTCCTGGAGGGTCTGGGCATATGGACGCTGGGAGCGGCAGACGAGAGACCAGTGGCAGTAGGTGACCTGATTGCGATAATAGTGATCTTCTGGGGTAGAACATTTGTACAAGTGGTCCATGATTATCTCGATCGGGAGCGATGGTGATCCATGGCATTAGCCGTGACGTTGCCAGCAGGAAGGCGGGCAGACAAGTCCACATTCGTTTCATCCTCGGAGGAGATTGGGCTCGAGCTCATGCTTGATGATACAGTTTTCTGTAGTCTTCAAGGCTCTACGACATCTGCTGGTGTGAGTTTTATTTCACGATGAGAATCGTTGAACGCACCTGACTTAAAAACGGGCGGAGTACTACAGGTCGCACTAGTGGGTGCATGGGAGAGAATTAAATTTCATCCCCCGTCAAGCTACATGACAACCGTGAC

>AT11247

GCGGCGTCCTGGGAAACGATGTCTCGGTGGCCTCGGGAAGGTGTATTACATCTACCCGCTTGAGGTTGGGCAGCTTTCGTGCGAAAACAGGAGGGAAAGGGGCGAATATACTTGTGGTGTTGTGGAGGTGGTATCCGGTCAGTTGGACTTCGTGCACATAGCCCCGGAGATGTTGCCCAGCATCTAGGATCGTGGAGAGCCGATGGAAGGATAGGCTGTTCGAAAGCTGGACCCTGTAGAAAAGCATGCGCTGGGAGCGGACGCGCCAAGCCCGGCAGACGAGAGCGCAGCTATAGAGGGTAACAATGTTCTGGCGTGTGTCAATGTACAAGTCAGTTGAGTAGAGCATATCGATGATATTTTCGCAGACTT

>AT11397

CCGCACCCTCCTGTCGCCCTCCACTCGGAACGTGGAGGGTGACACGATGGATCCGCTTCGAAAACTCCTGTGCAGTCTCAGGACAACCAGTCTCAGCCGGAGGGACCACAGCGCGTGCGCGTTGTGCGCCGCGGCCATCAGCAGCACGAGGAGTGATTGAGGGTGGCCCGGGGGGGACAATATTAGCACCGTCAAGGGTGTGCCCGGGTGAGTGGTTTAGCCCACCCGCTGCAGTTGGTGGGGCATCTGATGTGGTCACCACAGGGTGAGTAGACTGGACTGCGGTCGGAGCCCGTGTCCGGCGCACAGGCCGTTCAGGAGGAGTCGGCTTGGGTGGCCGCGGACGAAGCACTCGGGCGGGCGGAGTATGTGATGGAGCAGACGGCTCCAGGACAACCACTGCCTCCGGATCGACGACGACGGGTGCCGAGATAGCAAAACTGCTGAGTGTCGACGCGAAGTCACCCTCTTCATCGAAAGACGTGTATTCAGATGGGGCACGGACGGTCCCGGGCCGGTCATGGGCATAGATTCGAGACAATGCATCCGCGAGGACGTTCTCCACGCCAGGCACGTATTCAATATCAAAATTGAACTCAGAAATGTGCTCTAACCATCGCGCTTGCCTGCCTGACAGGTTGCGCTGTTTCAGGAAATGAATCAAGCCCTTGTGATCAGTGACCCAAGTGAAGGCGCACCCGAGCAGGATGTCACGGTGCCGACGCATCGACTCCACACCGGCAAGCATCTCAATCTCATGGACAGGGTAGTTCATTTGTGCTGATGATAGCTTTACCGAGAAAAAGGCTGCAACCTTGCCTTTTCGGAAGTCAACTCCCTGTGTCACTACACCAGCAATTCCGCCATGTGATCCATCTGTTACCAGCCAGATGCGCGGAGCCCCCTCAGAGTAGTCAAGTGGGACTCAGTGGTGCTCACGGTGGGCATGAAGCAACCGCTTAACCTCGTCGAAAGCACGCTGGTGAGTGTAGTCCCAGCGAAAACTGTTCTCGGTGCCCGTGAGCGAACTGAGGATTCCCATAGGAATACGGACGGTTGCGATGTCATTGGCCAGGTAGCCCACGGAGCCTAAGAATTCCCGGAGGAGCTCGCGATTAGTAGGGACCTTCCAATTCAACACATTGTCGACCTTATCTGGGTCCATTTGGATCCCATCATTGTCTACGATGCGTCCAAGTATTTTCATTTCGGTGCAAAGGAACTTGAGCTTTGTAGCGCTAAGG

>AT11411

GCAGCGCGACGAAGTGCCTCACGTCCTCGACGTCCGCGGCATCCTCAAGCTCCGTGATCCCGAGCAGCATATCGAGGTGCTCGATGAGCCGCGCGAGCGTGTCCTCGTGCGGGTCGGGCGGGCGCAGGGTAGCGAGGCTCAAGACCGTCATGCGGTTGCGGTCATGCGATTCTCGACCTTGGCGAGGACACTGAGCGGGAGCGCGCGCAGAGAATCGGGTAGCACGCGCGGAAGATCGGGATGGTGAGTTCGGTGTCCGGG

>AT11413

GCCCGACCTGCGCCCTCTGCTCGTCGCAGTCATGTTTCAGCCTAGTTATTTCATGTCGCGCCCTCTCGCAGGACTTTCCGGCCGGGCCATTTGCCCGCTGCGAACTTAACTGATTTGAGGATGGGTTCCCCGACCTCAGCGTCCTTGCGATCACCGAGGACTAAGACTCCTTGGACCCCCGTGTTACGTTCGTGCTTCAAACCGTGGTTGTAGGCGGCGAGCGCGACGGCTCCTTCGTGGGATCCAGGACCTCACGGTAAGCACCATCCCCTTTGGTATTGTCTGCCCGCCCGTCTGCACTCCCATCCTCACTGGGGCAATGTGTCGAGCGCGTCCCGCATCGGACCCAGCGGCGC

>AT11438

CGGGTCATGAATTCGGAGTACGCCCTTCATCTTCGCAGTAGACGATTCGCTGGGAGGCTAAATTCGGCCGCGGCGATGTTCGGTAGGAAGCGATCGCCCCTGGATCCTGACCTCCCGCCCAGCGACGACTCTATTCGCATAGTGCATCAGCCCAGAGGCACGTCGTGAGGATCAAGCATGCCAGTGGCAAGCGCGCGCGGAGGCGCATGCTCGCTGACACTGATAACGGGGAGCTGGATCGCCTAGGGTTCGAGGTCGGGGGGCCGGACCCACACGGTCTCTGAGCGGCGGTGAACGTGTTGGCTGGTCAGCATTTTGGGGGGCTCCAAGGGAGGTCATCTGCCCGCGAATGCTGGTCGACGTACGGTGTCCGAGTGGCAGCTGCGCGGATAGACGAGTAGAAGTGTCGCTGCGCCAATTCGCAACCTGCGGATTATCAGGTCGCGGCGGCGCAAGGCGTTAGAGGTTGATGAGCAGCATATAAG

>AT11540

AGCTAGATGCTTATCCTTACAAAAACTCAGCCGCATCCCGAGTCCCTCAATTGAGAGAAGACCCTACCCTTCGCGCTTCAGCTGTTGCCCGCATCGATCGAGTTAAATATGTGAAAGGGTGATTTTACGGTTCTATCGAGAGAAGTACAATAGTCTACAGAAGAAATGATGGCAGCTTCGGACAGGACCGTGCGGATCAAGAACATCTACCGCTGCCCGTACGACTTGGTCTCGTACTTGCCTGCAACACAATCGCCCATGGTCAATCTCCCGAGTCAACGGCCCCACAAAGAAAGCAAAACCACGCACCGACGCCAACAATGACGTTCGCAGGCTCCTGCGCCTTGCCCTTAAAGTAGTTCTCAAGGATCTGCTTCGTGCCCTTGGCATAGCGCGCCTGCGCGTCGAGCGTCGTGCCCGAGTAGTGCGGCACCATACCGTTCCCGCCCCCCAGCGGGTTCTTCATGTACCGCCACGGGTGGTCCTTGGGCGCGGGCTGCACGCTCCACACGTCGCCCGCGTAGCCGTTGAGCTGCCCAGACTTGAGCGCGGCCGCGACGTCCGCGGTGTTGCAGATCGCGCCGCGCGCGGTATTCACGATCCACGCGCCCTTCTTGAAGTGCGAGAGGAGCTCGGCGTTGATGAGCCCCTTGGTGCCGTCGTGGAGGGGCGCGTTGATCGTGATGATGTCGC

>AT11597

GCGTTGATCCTGCCCAGCGACTTCGCCTGGCCGCCCGTGAGCGTGTTCATCGCCTCCAACGCGATGTTGAAGCCCGAATCGGCGCGGTCGATCGCAAGCACGTTGATGCTCTTCCCATTGTACGTCAGCTGCCAGCACGTCCCGCACTGCGCCGAGTTCCAGCCCGCGATGGCCGCCGCGCCGCCGATGTTCGGGAAGTGCGGGAGCGAGTTGAACGTGCTGAAGCCCTTGGTGAGCAGCCCGTGCGAGCCATCGGAGCACGCGACGTTCGCGAGGCTCGTGCCCTTGCTGTCGTACGTCTCGTCGTAGGACACCGTCACCGTCGCT

>AT11600

GCGGCCCGCTCCAAGTCCGTGCGGTTCACGAGACCGCGAGAAAGCGCCTCAGGGAGGTATGTGGACGAGAACGTCCCACAGTCGATATCCGTACCCGCCAACAAGGCATCTGCAGCCGCCTGCACCGGGTCGTCCGTGTAGTTGTGAGGGGAGTAGATGTTTTCCACCGCATCGCAGTCGCTCGTCACCCATCTGTCCCCGCCAAAGCCCCAGTAGTCCCGCAGCACGTCCTGCAGCAAGAACTTGTTCGCACACGACGGGATCCCGTTCACCGCGTTGTACGAGCACATGATGCTCGCCACCTTCGCATCGCGCACGCACGTCTGGAACGAGGGCAGGTAGTACTCGGAGAGGTCCTGTTGCGAGACCACGGCGTTGAACCCGTAACGCTCGACACCCTCCCAACTATCCAGGTCGTACGCGGCGAAGTGC

>AT11620

GGACGGAGGTCCTGGAAAATTATCGACCGGCGTTCGTAACACATAGGGGCTAACAAGGCGCCAGAGGAGGAAGATGACAGGCAGTAAGCCAGCTAAGAGGAGCAGTTGAGCCATGTTGAAGGCGAAGGAATATGCTGAAGGAGATTGGCCAATGTTCGGGGAGGCAACAAGTTCGGCCTGCAATTTATATCCGCTACATGCTCCCCTGCCGAGTGTCTACGCTGCATGCTCCCCTGCCGAGTATCTACGCTACATGTTTGCCGCTACGTGCGGCAAAAGTTGTTCGCGGTTAATGCGCGCAGTGAGAGGGAATATGTTTGGATTCAGGAGATGGTGGACAGTACGAGAGGATGATGCG

>AT1162

GGCTCCCTAGTGATAGATTTCTGATATTGCGCCCACTGCGTAATAGAATCCGTACCAAAATTGTAAGCAGTCAAACCGTCCCGGAAGTGTTCAACCGTCCCGAGCGCACATGGTATGAAACCTCGCCATCACCGTGACGCCGCGTAAGCGTCTACAGACTGCAGAGGGTCTGGCTATGACGTCATTGTATACCAAACCACCGGAAATCAGAAATAAATTCATGTTCACATCCACGCAAATGTGGACGCCCATCACCTTCAAGGACGATGTTAAGTGGCGTCCTCGGGAAGATTGCTATGAATCCGTTATATCATTGGGCGATAGGGCTAATGAGGGAAATGCTAGACTGGGAATTATAGTACACGTGGGTACGACACATACGAGCATACAAGGGCAATAATAGCTTTTAAGCACCAAAACCGTAGAGCGTGCGGCCAGAGCGCTTGAGCGCGTAAACGACATCCAATGCCGTCACGGTTTTTCTAGGTGACGCTGAGCAATGATAAAGTCCTGTCGAGCCGTATTGAACTCACCTCTTGGCGTGCTCGGTGTACGTCACGGAGTCACGAATAACGTTCTCGAGGAAGATCTTCAAGACACCACGAGTCTCCTCGTAGATGAGGCCAGAGATACGCTTGACACCACCACGACGGGCGAGACGCCGAATAGCCTGGACAGTCGACGCGTCAAGTTCATACACCACGACACTTTGGACACGAGCCACATACCGGCTTGGTAATACCTTGGATGTTGTCACGAAGGATCTTGCGGTGACGCTTGGCGCCGCCCTTTCCGAGACCCTGGATAGAGAAAACGGGGCTGAGTGCCACAAAGAAGCAAAGGCAGTGTGAAGCTCACCTTGCCACCTTTGCCACGGCCAGACATGATGTATGAAGGTTGGGATGGAGATAGAGGTAGAAGACGGGCGGGTTGTGCGACAGTGGAGTCGAAGCTGGGTCGAGAGGATGATGGTTGAGAGACAAGTCGCGTCGAACGCGTCGCATTGAGTGTAAACGGCGCGCGTTTGCGCATGTTTGCATCCACACGTGACGGATGAAGTGATTAGACGCCCTAGTGAAGCGTGACCTATGGGTGGAGGACGGCCGATTGGGACTCCCAATGGGAGGTCGTGGTCGCTGTCGTGGTCAGCGCTGGGCAACGACC

>AT11636

TCACTAGGGTCGATCAGGGCGCATCTATTTCCGGCCGCACTGGCGAAATCCGATGGGGAATTCGCCAAGAAACGAGCGTCGACATCGTAGTCATCATGGCCAGAGTGACAACCGCCGAGTTGAGGCGGTTTGACTCGGAGGAGATCCGGCAACGCCTCAGGTGGCGTGTTAGCCCACGTGAAGAGCGAGGTATCGACGCATTGCTTCATGATTTCCTGTTGCAACCTTCTGAACGCCCGCCGGCCCTCACCGTACAACGTTGGCATGTTGATCCCGAAGATTCCCATCAGGCAGTAAGCCTCGTCCTCCGGGCGCGTCGTCTCGCGCCCTGCAGCCC

>AT11690

TAATCATGGATGTATGCGAAGGGTGTAGAACTACAATATTAATGTCGACGCAAGAGAATGTATATGCAGTGACATCAACGATCCTTCGGTGCCAACCCATCGTCCATCTTGATGACTGGTTTCTCTCCGTATCCATCAGGATCTCTCGGGGAGCTTGCCAACTCGCTCAGCGCAAGGATTAAGGCGTGTTTGCGGTCGAGGACGCCTCGAATCGTGACTAGTGACCGTGAAGGCGTGAAGCGACCTTCCAAGTATCACCCATATGCGTGGATTCAGCGAGGGCGAGGGGAGTTGTTACGCGGGGAATGGTGTGGACTCGATCGCGAGGAACGAGATGGCGTGTAGCGTCCGGGTTGTTGGCCGCGATTGGGGGCCGAGCGGGATGAGACCGCGCTGGCATGCGGTAGCGATAGTGGTTTTGATCGCTCATGAGAGAGCGCGATGCGTCTACTGCAGTCCGAAGAGCTTCTTCAGACCGGACTTGGACGACTTCCGTTGTGGAGCGCGTTCGTGCGAGGGGCAGGCAGCGAACACCTGGCGCGCGTCTTCGTACATGTGGTAGGTATCGCGCGGCGACTGGGTAGATGCGAAGAGCGAGAACGCGTTGGGTGCGGAGGGCGCGGTGTGGAAGGAGCCGTAAGTGGAGGCGTTGGTGGAGAAAGACATCCTTGGTAGTGTCTGGAGGGCGAATGGAGAGAAGC

>AT11737

GCCCATCCATACCGGTAACAGCGAGACGATTCGACTTCTGTGTCGCATGAGTTGTCGGGAGGTTATATATGGCCTGCAGACAAGCCGGAGTGATGGTTTTGGGGCACCCCGAAGGGAGGGTCTGAGGACTGAGGACCTCGGTCGAAGTGATCTTGTCCGCGGTCGGGGTGATGTCCCTCAAAGACAGGCCGACGTCTGGAAGCACCGGACCAACGTCTCCAGGGACTAAGGCCATGATCGGGACATCGTTTGGCTCAGGGAACGTCACTGTGGGGTGAACAAGGTCGAGGTGGCCCTGGAGCTCAGCGGGGATCGAGTACGAGAGCGTGCGGACCGCCTCCACGCCGGTGTCGTCGTGCTTGAACACGGAGAAGTCGGCGTCGAAGAGCTCGTTGGCCTTGCTGACGGGGACCTCGAACGAGAGCCAGTCGCCAGAGGGAGAGATCGTCTCCGCGGTGATGTCGTTCTCCTTCAGCCAGGCATTGACAGCGTCGACGCTTCCTTGTGCGGGAGCGAGGAGCTGTTGTACC

>AT11890

GCATATATAAGATGGGCCAACATGGTCGCCCTTCGTGATCCGCCTTCTTTCCTCCCCCCACCATCTCCCCACTGTGCAAGCCACACCGCCCTCTACCTCCTCCGTCCCCATTGTTTTTTCTCCGACGCGCGCTTGCGCGTAATACAGGTATGTGCGGACACCCGCCCTACGTCAACTAATTGTCGTCATCGTTAGAACCGCATGTGACGGGCGCGCGGAGCTCGATCACAGGAGCGCGCGGTGGGAGGGAGCGCACGGTGGGAGGGAGCGCGCGGATTGAATGGGCAGGACAAGCGGGAAGGTGCGCGCGACATATGGGTGTGCGCGCGACGGATGGGTGTGCGCGCGACGGATG

>AT11951

TTTTGTTCACGATCCGGTACGGAGACCGCATGCGCGCATGGGTAGACATCGGGTACACGACGTCGAGGGACACATGCTAGAGCCGCGCGTTGGCCGGCACGACATTTAACGTGACGACACTCATGGCTCTCACGAATAGAACTCTCATCTAAGGTCCTTTACCCAAGCATCGTAAAGCTGGTGTATCTGTACTTCTCTATGCATAGACCGCTGACAATTGAAGACCTCATCTTGACCGTCAGAAGACTCCGGAGCAGCGGGTCCCATAGTCCGCCCCCGACCTATGCATCGCCGTGCCGAGCTCACCAAGCGAGGCTCAGGGTTGATGCGTCGATATGCGACGAGGCGAGAGCCCAGTGGGGCTTATACTCGCAGTCCTAGCCGACACACGCGGCCCCGCCCCGCAATCGCTCGGGCTGATCGCAGCGCTGGGAGAACTTCAAGTCCGTGCCAGATATAGTAGGAGTGAAGGACTCGCCTGCGTGCCACAGGTGAAGCGCAGCGCACGGGCCCTCCTCCGGACGCATTTAAGGTGTGGAGGAGAGGCAACAGCAGTCGGACGCACCTCGCCTCTCCATTCGCCTTCAGACCACCACTCTCGCCCCGACCAATACCACCACATCTTGACAACATGTTCTCCCGTTTCGTCGCAGCCTCCGCGCTTGCCTTCGCTGTCCTTGCGGCCGCCGCCGACAACTGCACCACGGGCTCTATACAGTGCTGCAACCACGTCGAGGATGTGCGTCCCCTTCCCCTCTGATCACAAGTTATTTCCAGACGCCGACACTCAACTTCCTGTAGGCCGACTCCGTCTCCGGGTCCACGCTGTTGGGGGCGTTAGAGACGCTTGGCATCAACCTGCAGGATGTGACGGGCCAGATCGGTCTGCAGTGCTCGCCCCTCTCCGCGGTCGGCGCCGGAGGGAGCAGCGCGTGCTCGGCGTCGCCGGTGTGCTGCC

>AT12010

CATTCTCTTCAGCCGTGATATAGTTTCTGATAGCGAGATCGACCTCGTCGATGATGTCTGGGAGGATCTTCGGTAGGATTATTGTAAGCTTCTTCTTTACTAATTCTGTGAGATGGTTATCCGCCATGCCAGAGACGAAGAAATGTTGGGATTGGAATATCTACGATGCGTCTCGGTAAGACCCCCTGATAATTCGCAATGACATGCACGCACCTGTCCTAATCCAAGTAAAGCAGATAGTTCGTCATCCGGGCGCGTCCTGAAATCCTCCACCATGTCTCTTCCCGAGACGACGACGAGCCACTTGTCCCACATAGAAACCTTGAACGCTGACTCTGGATAC

>AT12046

CGAAGGTGAGCTCGTGCTGGCCGCTGCCGAGAGGGCGCACAGAGGACAGCGCGTGGCCCCACCGGATGAGGTGTGGAGGGATGTCGTTGAGGAGGAGCTTGCGGAGGTCCGTGCGGTCGATCTCTGGACGGATTTTCTCAGGGGGCGGCAGCGCGCCGTCCTCGCCCACGCCGCCTACCGTGAGGAGGAGCGTCCCGGCACCGTCGCAGATCCTCATTTCATCGGCGTCAGGACGGGACTTCTTCTCGAACTCCTCTTGAAGGCCATTCTCGCGCAAGGCGCGCTGCCCGCTCTTCCACCCGAGGTCGAGAGTGCCACCGAGATGGGCGCGCGCGTCGATGCCGGTGTCACGTTCGTATAGCGTGGCTTGCACCCCGCGGCGATGGAGGGTTAAGAGGAGGGCAAGACCGCCCATGCCACCTCCGATGATGGCGATGC

>AT12071

CTGGGGCGTTGGCCGTGGCGGGGAAGGTGAGCTCGATGGAGGAGTTGATGGGGAGCTCGTAGACGCTCCCGGACGGCAGGAGGTCCTGGGCGGTCTGGGCGCCGCTCAGGATCTGGAGGAGGACGGGCACGGTGGGCGGGGAGAAGGACTCGTTGTTGATGAAGAAGTTGGTGCCGTTGAAGTTGAAGACAAAGTTGATAGCCTTGTCGACACCTCCCTGAGTGGGGTTGCCGGGCTGAAGGAATGGTTGGTGTCAGTATCGAGCGGTACATGGTGTAGTGAGGAGAAGAGCTTTCGTACCATGGCTCTTGGCACAAGAGGGTGGAGGTCGGTCTCCAGGAGCAGGTCCTGCGTCGTTTGTTGAGTCGTCACGGGCTCGATCGGGTCCGCGGTGTCGTAGCGCAGTATGGCGGAGTTGATGCCATCCGTGAAACCGACGTTACCGAAGTTCGGGTTGGCCCGGACCCAGTAGTTGTCGATGGTCTGGTTTGCAGTGAGCTAGAAGAGACCGCTTAGCTTCCACTTCAAGAAAGACGTCGGGGCGCCGAGGACTTACCACGAACGAGTAACGCTGAGCCGAGAAAATCTGGATGGCGTTGACGGTGACAGGCTCGGTCTCGATGCCGTCCGCCTCAATGACTGTCAAGTCATGACCATCAATGCTGAACACGTAGTTGGGGTCGCACGACAGCGAGATCAGGCGGAAGCGGTAGCTA

>AT12091

GTGGGTATGAGGATAGCAGGTAGTTTCCGACCACCCGCGCCGGTCAACACTCCGAGTCCCAAGACGAGTCTACAGTAGGAAAGCCGCAAAGTATGTGTCGAGGAATGCAACACCGTCCAAGGGAGCTACCGCCCCGACGTATCCATCCGGCCGCACGACCACGATGGCACCGGTGCGCTCGCCGATACCGAACTTCGCGTATCCACCGCCCCCGGAACGCCCGTGCATGTCTTTGTCGTCGAGGAGCACCCTG

>AT12092

ACGTAGACCGTCCACCACACGGCCTTCCCGTCCTTGATCTCCATCCTGTACGGCCTTAATATCTTCCGCCCCTGTTCCATGAGCTTCTCCGGGCTCGTCCTGTTCTTGTCCGCCCGCCCGGTCGCGGGGTCGATGACGTCGCTGTCTGGCCCTTGCTGGATGTACACCCGGACCATGTCGTTCTCGCGCGGGATGATGAAGACCGTGCCGTCGTTCGAGTGTACGAACGACTTCGTGCGGAAGTCCGGGAAGTCGGTGTCGGGCACCATGTCGACGACGCCCCAGATCGAGTCTGCGGACGAGGGTATAGATTCACGGCGTCGGTCAGCTGTTGTGCGCCCCA

>AT12115

GACGTAGACGGTCCACCAATTGACCTGCCCATCCTTCATCTCCATCCGGTACGGCCTCATAATCTTCCGCCCCTGCTCCAGAAGCTTCTCGGGGCTCGTCCTTCTCTTGTCCGCCCGTCCGGTCGCGGGATCGATGACGTCGCTGTCGGGCCCCTGCTGGATGTACAGCCGGATGAGGTCGTTCTCGCGCGGGATGACGAAGAGGGTGCCCTCGCGAGAGCGGACGAACGACTGCGCGCGGAAGTCCGGGAAGTCGGTGTCGGGGACGAGGTCCACGACGCCCCAGATCGAGTCTATCGTTGGATCCGACACATCAGCTGTTGATGTCTTAGTGCTCATAGGAGGAGGAGCGGGCGCACCCGTGTTGTCTCCCTCCATGTTGATGCCGAGCGTCTTCCGCACCCACGAATGTGCACC

>AT12163

GCGCCTGGTTAACGAACCTATCATCAAGAGAGGTAAGCATTGACGCACCTGCTGAAAATCCAAGAAGTCCTTAAACAACACGCCCTTCTTCCCTGCCTGGCTCTCCGCCCGAAACTCCGGCGAGTCCATGATCATATGCACGAGAGACGCGGACTTGGTGAGCGCGCTGAGCGCAGCCTTGAGGTCCCCTTCATTCTCCTTGACCGCGGCGAGGCGCATCTGGGTCCGGGCGGAGGCAAGAAGGCTCGCGGGGGAGGCTCCTCGGACCTGCTGAGCCCTCTCCTTTGCGCTCGCCTTAATCTCCGCTATGGTGTCGCGTTGGTATGACGCTCCATTTTGCATTGCTGGTTGCGGGGGTTGGTCGTCGCGTAGCGGGTTACAAGAGGCGAATGCTTACCGGACAGCTTCGGAGTGAAACTAGTGGGAGAGGTGGGGGAAAGAACAGCGATGCCGGGCATGAACCGGCTTATATGCGTTGTTTAAGAGGGAAAAAATAAGGATATGCGGGCGAGAGGGAGGAGAATGAAGGTGATCAAGAGGACTGCGGGCTGGGC

>AT12346

GGAGACATCACCGGGATCATTGACTGGGAGTACCATTCCGTGCGTCCCGTCGTCCTCGCCGCGCAATATCCGTGCTACTTGCGGTACGACGGTGTCTGGGACCCCCATTTCGGGCTTGGGGAGAAGTGGTGGGTCGCCAGTCCAGACGATGCGGCGAGGCTGAGGGCGATCTATGCCGAGGTGAGCAGTTACTTACTTCTGACGGGCACTAGACGGCTGACCATGCTGCTAGGTCATAAAGTCGCAGAACGAAGATTACTGGCGCGCGCTCGTCGACGGGGGGCTGCTTCGGCAGGTCTACGAATGGCTCACCGACAGCTTCGTCGACCCTGGCTGCGCCGCCATGGCACGCTGGATGGACGATGCGTTCGGTTCGGAGGTGCCCTGAGGAGAAGTGAAAGCTTGACGTTGATGCGACATGTTCGAGTTCGCGCGATTGAACAGGAAAAAGGACATGATGGAAGGCGCGATATACTGGGGTATCGTCGACCAGTCAACTCGTAATACTGTTTCCATGTACTCTGCATTCGTGGCGCTCCTATAGAGCTCAACCTTAAGGGGGAGCGTAAGTAATTCCCCCAGTACTGAATGGGACCAGTGCCCGTCATCCAAACACGA

>AT12375

CTGGCGTGCCGTTCACCAGCGAAGTGACGGCGTTCACCCAGCCGTACTGTCCTTCTCTGTTCTACACTGCCTTCATTCACATCTAACGCGGCTGCCCCCCAAACATTTGCGGATCTCAGGATCACGAGTATCCTCATCTCCCGTTTCCTCATCGACCTCCAAGCGACGAACCGTGGGGCGATGGATCTCCGGGGCCTGGGCACCGGCATCGATCTGTCGTCGCAACAGTCCAGCACGGTCTCGGATGCGGACACGCTCCGCTTCGGGGCGGTCATTGGCTCCATAGGCACCATCCTCACTGGCAACTCGGATCTCGATGCGGTTGGCCCCGAAGACCAGGGCGAGAAACATCCCGGGGAGACACAGTCAGCACCCTCACAGCAGTCGAACCCCATAGCGAGAGATGAGGCTTGAAGCGAGAGCTCCGAGCAAGCAGGTGTCGACGTGATGATCACGCGAGGCAGCTGTTCGAGCTGATAGCGACATGGAGGTGATTCGGAGGGGTGTGGCTAGCCTGCCTAATTAGCCCATCCCGGCGGCCCCGACCGACGTCGTCCCACGGTGCTTGTTTAGTACATGTATCGCATTCTACTCGTGCGCAAAAATACCGGC

>AT12572

AGCGGTAGACAGACCTCCTCGGTAACCACGCGATAGTGCGTGAGCTGGCGCACGGGACAATCCTTTCGGAATCCAGTGTCGAGGGTATTCTGGCTATCGGGCTGGGGTTCGGACTCTGTGCTTGCTTCGCCCCGAGCACGACGATGAGCTGGGACTCCATGGGCGGGCGCGGGGTCCTTCGGGTGTTTCGCCTGCCACCACTGCGCGGTCACCGTCACTTGCTTGCGCACGTCCCCGTGACACACCATGGTAGGAACGTTCTCCACGCCAGCAGCGTTCAGGCAAGCAAGGATCTCGCCCTCGGTTTCGACGAGCTCGTACGCCGCGCGCCATGAGTCCTTGAGCCACACGAACCGGCGCTCCTCGCAATCGAACGCAACGTAGCAGCGCGTCCCAGGGCCGACGAGACCATCCGCGCGGACGACTGGCTTCCCGACGAGGAACGCGCGTGTCGTTCTGGGTCCGACGTTGACATGCAGCTTGTAGCGCGGCCACTCGGGGTCCGCGATCGACTCCGCAAACATCTCGCGGACGTACTTCCACGTGAACGTCTCGGGAACGACCTCGTCGAGGTCGAGATCGCGCTCCTCGTGGTTTAAGTCGTAACGGGCATCGTGCGCCACTCGATCCATCGTCTTCCAATCGCGTCCTTGGTGCGAGATACGCGTGGCCGACGGATCGAGCCCAAGCGCGGTGTCCCCGAGGTGCGAAAGGCGCCACAAGAACTCGAGGAGGGGCTCCGGGTCCTCGAAGTAGTCGACGAGCGGGGAGAAGATGACCCCAGCGCGGTCCCAGCGCAGGAGGCGGAATCGGCGGCCGATAACGTACAGCATGAAAAGGAAGACGCGCTGCTGCACGGCGCAGACGAGTTCGCCGAAGGTCATCGCCTGCCCGCGGCTCTTCGTGCGGTCATCGGAGGGGGGTTCTGGGTTTCCCTTCTTGTCGCTGAAGGGATCGTACTTCGTGTAGCCTTCTTTGCCACTCTTGAACTCGGCGCACCCAGCTTGGTCCGCCCAGTGGGGGCACCCGTCGTCTGGTGCATCCCTGGTATGGTAGAAAGCGGCGTCGATCTTCTGGCCGCTCTCATCTACATTCTTCGGGTCGGCCCT

>AT12811

CGCCCTCCGAGTCAGAAAATGACTCAATGTCCTCCACCTTGTCCCCCTCTTTGTCCCTGCCTGCCGTGATGTAGCCGCCAAGACTGTCGTCTGTCAACGCCGGGATGCGAAGCTCAGACAGCAGTTTCAAGGCCTCGTCTCTGGACATTAATGCACCGGCCGTGGTCGGCTGGTCGAGCGCGAACTGCGAGGTGAGTGTGCCAACCTTCTGGGTATGTGATCGCGATGGCTTGCCCTTTGGGTTGGAGACGGAACAGTGACGAGCTTTGTTCACGTCAGAGTGTTGATGGCGCTCGCGTGTATGGGGAGATGCATTGGGGCCAATGGGTATTGTGAGGATGGGCTCGAAGTTATCCGGATGGAGCACACACTCCTCGGCGGTGACAGTAGATCGTGATGCGTGAGCTGCAGCAGGTCGTGCGTGTGAGATGGCTGCGCTGTACCCAGTGGGTGCAGTCAGGTCCTGGAAATGCGACGGCATCCACTTTGGACCCTGGATTGTAAGCAGGCAGGCAGGGAGCTTAGCTGCGGCCAAGACGTGCGGTAGCACGCACTTTGGGTCGTGGAGAGCGTGCTGGCAGATGTCGGGTGGAGCGGCTAGAAATTTGAGCATCCCATTTCGCCACGCACAATTTGTAGGTAAGTTTATGGCACCCAATGGTTGCGTGTACCCTCAAACGAATCTTTCGAACGGCCTTTAATTTAACGGCTTTGTCTCTTTAGTCCACATCCTTTCGAACCCTCGACTCGGACCTTGACAGTCCGAGTCCGAGGGATCGTTGCCTCCCCCGCTGTCTTCGAGTGTAACCGCCGTCCAATGCGACCACTCCTCCTCTTCCTCTTTCCGTCTCGCTCCATCCTGCATCTAGGAACATGACTCGAC

>AT12885

GTCCCGAGGCCCCTGGGCATGGCATAATGATAGTGCTAAGTTACAGCGACTGGATACATCGACAGAATCAAGCCCGAAGGAGACGGACACACGCCCCCAACCTAGCGCGGCGCATGTCCCACTTTGAACCCTCATGGTCGCACCAGCTCACCAGGCGGCTTTCACGGCAGTCGCGAGCAGGTCATAGGTCACAAATCATCGAAGACCTAGCACGCGCCGCTGTCGGTCCACTCGCCGCTGGATCCGCCAGGCGTGCTGTCGTAGGTCCACCACTTGGCGGTCCAGAGGTGGCCGTTGTACGTGACCTTTTGGCCGCCGGAGTACTAGAC

>AT12896

GCATAGCTCCACATTGTAGACAACGTTAACTTGCAGCAACCCGGCAGCCTTGAGCCTCGGCAATGGCATCGGTCGAGGTTCCTCGACGAGCTTGCTACAAATCGCCCATTGTGGCAGGACCTCCTGAAGAATGTGCAGCTCGACCGTCCGCAGATGCGGGAACCTGCCCCCAGACGGAGAGAGCAGGCGGTCAATAACGTCCAGACTCACGGCGCCGGCACGGAAGCGCTTCCAGGACTGCAACGCGGTTACCCCGATGTGGATAGCGAACGCGAGGAGATTCGGAGGCAGATTCGCAATGATGGGCTCGAATGTCTCGCGGTGGCAGAGCGGGTCGTCTGGGATGTTCCAAAAGTGTCTGAACCCGATGCGGAAGCACTCAAGTCGCGTGCAGTGTGCGAGCGCGGCTCCCAGGGAAGGAAAGC

>AT12915

TCCTCTCCAAGCCCGACGGCCTCCTGCCCATCCTCGCGAACCTCCCTCGGCGGACGCTCCGGGTGTTCGCCGTTAACATCTGGCTACACACGCCACGGCCTTGGTGCTGCACAAGCACTCTCGGCGCCTTCGACGCTGTCGACCGCGCCTTCGCTCCGGGCGCACCTTCCGGAGGAAGCAGAGAAGGACGGTTTTCCGATCTGCGGAGGGTCGAGCTTTCGGTGTTTCAGGAGGGGCCGCCGCCGCGGTTGACGTGTATGAAGCCCCTCGAGAAGCACGACCCGTGGCCCCTCCCGCGGCTCGATGCTGCTGGGTTATTGGCGTACGGTGTTGGAAGCATCAACACCGATTTACCAATTGGTAGGTCGCCGTATCGCCTATCCCCGGGTTGTAAGAACTGACTGCGGCCGTTCCTATTCTCTGATCTCCGGAACATGCAGCTGAGGTCTTCTGGCCGTCCGAAGAGTAATATGTAGCCTTTTACTGGCATCCTGACATCATTGCGTTTCCGAACAGCCTTCCGGTAGATTCCCTACTAGCATCGAGATGTAGTGCCAGGACAAGCGAAGACAAATAGGAGAAATTGGCAGGAACCTCACCTGCGCAAGCTTCGCCCGCCTCTACTCCCCGTCCTCCCCAAATGGTTTCTCAAGCACTCCGCGCACCTCCGCCTTGACGGCGTCGATAGTCCCGGTTAGCACCCGACTGTTGCGGCTAGATCGGTGTGAGCCCGTTACCGCTCCGAACTTCGATGAGCTCGTATCCGGTTTGCAGCTTCTCAGAGACGATGATCGTGTTGAGGGGCTGGTCGACCCGAAGGGCCAGATAATTTTGCAGATCTAAGCATGAAGATTAATGTGCGTGGCCCGGAGAGAAAGGGGTGTGAGCGCACTGGGGAATGCCGGCGCAAATAGCTTCGATGACGCTGTTGCTGTTGTGGCCGCCACGATTGAGGAACACACCCGTCGCCTATGAAGCAGACGTAAGATCAATCTGGACGCCCGGGTGCGAGGATCGCAAAGGGCATCCGGGTGGTAGAGAACCAGTTGCTGAGGGGTCCACGACGTCAAGAGACCCCATCATCCATGGGTAGTACATTGTACGTGTACATCATGATGATACGGGAATCCAGGATGGTGCGGCTCATCGCACATGTTCTACAAATTCACGTCTACGAGACGCTGCCTTCATTCGAGCATCGGCTTGATATACTTCTACGCGCTCCCGTCGTAGACCCCAAGTGCACTGTACAACTCCCAAGTCCCAATCCGAGAACCCAAACCCGTCCGACAGCCCAATCGCCTCAGGCAGCAGCTCCTCGCAGAACCTCTTGATCGCTCGGACTCACAGCTGCCTCGACGGATCCCCGGCAGTGCGCGCTTCGCCCGGCAGGCCGAACGATAGGATTTCAGTCAAACCGCTCTCCGCCGTCATCA

>AT12976

GGCCCGCCACTAAGTCTACAAAACTGTAACCATCCTGTAAAGCCTTTCAGACATCAACACAATTCAGCAGGAGGGTCGCGGATAAGCACGACGTACCTGCTATCCCAAAGGGTTGCTCCAAACAATTGACCTTGCCCCTGCATGCGCGTGAAGGGGATATGGTAGAACCACAACCGGCATGAGTGGAAATAGTTTGAGAGACATACCCGGTACGTGAAGTGCCGTATGGATGCGGAGAGGAACCCATCGTCTCACCACCAGGCATCCCACTTCCGTCCTCAACCGTCCCTCGTCCACCTGTTCTGGGAAGGAGTTGGTGGTAACCTCGGCGAACACCACGTACCATGTAAGCGCAACTCCGAGGCTGCGAGACCACGAGAGTGGAGACAGCGACAAAGTAACGAGAATGCTGGAGCTCGGAGAGTGGAGCGCAAGGTGTTTGCCATGGTGCACGTTCAGGTTTGAGTTGCGGGCCCGTACGTAGAGCGATGAAATATGTATGAGAGGAATGGGCGGTGTCTAGAGCCGGGAGATGAGACAAGACGAAGGAAGGCGGGCTGGACTAGGTTGGATTGG

>AT12979

GGGGGGATGGGGGCGGGGGGAAGGGGCGAAGACAGTGGAAAGCCGAGGAGAGTCCGTTCCGAGCCAATGTCCAACATGCGTGCAGTCGAAGCAGAAGAGGTCGTAAGGAGCGTGTGCGTGTGTACGTGTGTGTTGAGTGCTTGGTGTGTGGCGCCGGCGTGGCGTGCAAAGGTTTCGGGATGGAGGAACGGGCAGGCGCCGCGAGCGGGGTGAAGGAGACCGAGAAACGCGTGAAGTGGGGGAAGAAGTAGCAGGGTTGTTAGATGCTGGGGCAGCGAGCATGGAGCATGCGAAAAATACAACGAGTGCGTAAGGTGCGGAGGGCATGCGAGCGAAGGTCAGTCATATAGGAAATATTAGGGAGTTGACTTAACAAGTAGCAAGGGTTGTTAAGGCGGAGGTGTGTAGAGGTAAGATGACGGGGTCAGAAAGGTGGCGACACGATACCGCGACGGAGAGTCGCTCTGTGGCGTGATGGCATGGGGCCGTGTGGGGATGCATCGACTAGCAGATCACGCAGCAGCGTCCCTTGTCTTCCCAACCATCCCCACCCGAATTCCGCCTATTGCCAGTGCTCCCGCCCAGACGATCGTGCTGGTGACCGGAGTGCGCGGGAACGCCTTCGCGCATGAGCATGCTCGCCCGGGTAGCCGCCTCGAACACGTCGTCCACACCCTCGATCTTCAGGGCAGAGCACTCCTTGTAGGCTCGCGCGCCGATCGCTTGTGCCACGCGTTCAGCTTGCTCTCGTGATACATAAGCGGAAGAATTTGGTGAGCCTAG

>AT12989

TCCAGGGGTGATAGAGCTGGTCGGGTTAGCCGCTTCCGCGGAATGCGCGGCGTCGATGATATCTTGGATGTCCTTGTTAACGTCGAGGGGACCGTCTCCGGTGGAAGCAAGTGCACAGCCGTCGGGGCCGGCGAGAGCGCAGCCTGTGACAAGGCCCGCGTATACGTCGTCCGAGTTGAGGAGCTGGGTAGGCCATTCCTGGCGAATTGAAGACGACGAGATAAGCGGACAGCACGGTGCGTGCAGCAAACAGACAGGCGTACCAAAAATCCTGCGCTAGTGTGG

>AT12994

TTTCCGATGACGAGGACACGGTTCGCAAGAGTCTTGTTGAAAGGACCGCTGTAGCGCTCTACGGCGCGTTCCGGCCAGAAGGCGCAAGCGTATAAGGGGCTCATCCAGATCGTACCGACTGTCAGGAGATGGAGACTCATCAGGGATAAACCGATTCGGGACACCAACAAAGCCACATACACATGTGCGAGACGTTGTGGGTCGTGTTAACGATCGTGTTGAAGACGTCCCTCATCGTCGCCGTGTTGTATGGGGTTCTGTCTGGCGGGTCGCCGCAAAAAATCGCCGAGGCAGAGTACGAGGCACTAGAGTTCTCCGCATGCTTCGGCGAACTGGAGAGTGACCTGTTTGATGCCAGTGTACGCAGTGATCTGTAATGACGACATACGTGTGCCTCCTGAGGTCGAACCGCCCCGCGATAGTCCCACTTGCCGTTGGCGTCGAGTTGAGCTGCTGCACGACTTGCGGGAGGATCTCGTTCGTGAGGTACATCCACTTGTCCGGAATGTACATTCCCCGCCAGAGATATTGTGCTAGTGTAAAGTCAGACGTTATTCGCAATCGCAAGTCCCGGCGACTCACTCCTCAAGTTTCCAGGGGTGATAGAGCTGGACGAGTTAGCCGCTTCCGCAGAATGCGCGGCGTCGAGGATATCTTGGATGTGCTTGTTAACGTCGAGAGGGCCGTCTCCGGTGGAAGCAAGTGCACAGCCATCGGGGCCGGCGAGAGCGCAGCCGGTGATCAGGCCTGCGTATGCGCCATCCGAGTCGACGAGCCAGCGGGGAGCCC

>AT13050

GAACGGAAATACATCATTGTGTTAACGTTACTGCCCGTAAAAGGTTACGTATTACTCGTCGGACGGCCAGTAGATCTTAGCCGCTGCAAGAAACAAGGAAAGGTCACAGTCAGCTCTTACAACCTAAGAACAAGCCATACGGTGGCCTACCAATTGCTTCGCCGTTGTTGACTCTTCCAACACAGGACCGCAACAATCCAGCAGCATGGAGCCGCGGGAGGGGCCGCGGGTTGTGCTTGTGGAAGGGCCTCGTACACTGGCACGTCGACCCCGGCGGCGCCCCCTCCCGATACACGTTTAGCTCGACCCTCCGCAGGTCGGCAAACCGTCCTCCCCCGCCTCCGCCAGAAGGTGCGCCCGGAGCGAGCTCGCGGTCGACGGCGTCGAGGCCGATCGGAGTGCTTGTGCAGCACCAAGGCCGTGGCGTGTGTAGCCAGATGTCGACGGTGAACACTCGGAGCGTCGGCCGGGGGAGGTTCGCGAGGATGGGCAGGAGGCCGTCGCGCTTGGAG

>AT13173

AGAGTTTGACTTTGAGGCTACCCCCAGAGATACTCCGAGAGATCCTCCACTTCCTCACCAACGACAGGGAGGCGCTGCTGAGCTGCGCTCTCGTCTCTCAGGAATGGTTGTTTGAGAGTCGGTCTTTCTTCTTCCGAACTATCGACCTCCCGGACCGCGACGCATCCGATCTCTTTGTCTCCAAAGTCCTCCGTTCGGAACACTTAGGTCCCTGGTTCACCTCGATACGCCACCTCTCATTCGTCTTCATGTATCCTCTTGAAGATTATGAAAGGTTCCTCTTTGACATATCAGAACGCTTGTCAAATTTGCGAACCATGAAATGGGATGGTTTTCATCACAATGGAAGTCCTCTTCGGTCCGACGTGTTACTCGTCCTTGGCAAATTCCCCTTCCTCCACCATCTGGAACTTGACGTCTGCATCTTTGCGTCGTTCCAAGACCTCAAGAGAGTCCTCGGCGCAATCCCTACCATGACGTCGCTTACACTGATGAACATCGGATGGTCCAGACCTGACGATACCGCTTCCGAGTCGTCTGCGTTGTCTTCCGCTCCCATCTGGCCAACAGCGTTGTCGAAGCTGTCCGTCTACGGGCCCTATTACGAGCGGAAGTGTGCAGACGACGTGATCATTTGGCTATCCACTACTCCCACACGACACCTCCTCACTGAGTTCTCCTTCCATATTTACCACCTCGAAGCAGCTCTACAGCTGATGGCCGACGCCTCGCTCACAATTCTGGACGTGCAAATGGCCGAGACTTACAAGCTATTCAGTG

>AT13218

CTCGGGCGCGAGGTCTCTGAGCCTCTGCGTCTGCGCGCTCAGGGCGCGGTACCCGCCCGCCCTGTCCGCCAGCGTGTCGTTGTACTGGAGGTGGGCCTCGAGCGTCAGGTGCCACACCGCGTCCCGCCACGCGGGCGTCACCGCCGTCGCGCCCGCGGCGCCGGGCGCCGGCTTGTACAGCCAGGGCGTGCCCACGATGACGTACGGGGACGTGTAGGCGAGCGTCTCGGCGACGAGCGCGCTCAGCTGTGCGCGCCCGGACGCGGTGGAGAAGAGGCGGCTGGGGAGGAGGCGCGAGCCCATGGCGGTCTCGAGGCCGACTGGCTGTTGGGGCCGGGGGCATGTACATACATATGCGGTTAGATGCATCCTGCGAGGGAAAAACGGAAAACGGAAAAAAGTACTGGGAGAGACGACGTACGGCCTCCGCTGAAATGACGTATTTCTGGAAGAATGCAATCCATGTCGGGAGCTCTTCAACAACCGCCGAGCCGTTGCGCGCTGTCACAAAGTCGATGG

>AT13294

CACCCTCCCGTCCCAAAACAGCGGCTTCGGGTCGAGACTCGTAGTCGTCTTGTTCGCGACCGTCCACTCCGAATCGTCGAACCCGCTCTGGATCTCCGGCAAGCTGTCGCTGAATCTCCATCCCGTCAGCGCCAGCACCCTCACGTCCCGCACGCTCGCGCTCTGTGTGAGCCGCCCCGTAAGTAGCGTGCCGTCCTCGCGCACCCCGTTCCACGTCACTTGCTTTATCGCGGGCGGCGCGACGACGGTGAGCGGCGCGCTCGCGTTCAGGTCCCCGCGCAGCGTGAGCGTGCTTCCCCCAGCGTCGATCGTCGCGTTGCGCACGAGGTACGGCCCGCCGACGAGCACGGTCGTGTTCGTCCCGAACTGCCAGAACGACTCGGATCCGGGGACCGTGTTCGCGGTCGCTGACTTGATCGGAGGGGCCCAAAACGTCGCGGTGGTGGCCGGGTCGGCGAAGAGGACGAGCTGCGATGGGGACCCCCAGACGGTGGCCAGCCCTGCGGACCCGGGGGGCACCGCTGCGGTCGTGACGCCCGCCGCTACGGAGGAGTTGGCGAACTCGAGGTGCGAGGAGGGCGAGCGCGCGCAGGCGCCGGTCAGGGTGAGGGGAACTCGTGCGACTGGTCCGCGCCGCCGAAGAGGAAGAGGATGTCGCACTCGCCAATCGT

>AT13305

ACGGCCGTTCCAGGTGCAAAAATGCGCAGGTCGGTCAGGGACGGGAATGATACGACGGGCATCTCCATGATCTCCTGTGGGAACGACGAGTCCGACGGCCATGAGCCGAGAATCAACGATTCCAACGCCTTGAGCGGGAGGAGAACCCTGTGGACGCCGGCCAAAAGCATAGCGTCGTCATCAGCCTCGAGACCAACCGACAGATCCAGAGAACGCAATTTGACACTGGATAGAAGAAGCGGCGGATCCGAGAGCGAGGCTGGCATCGTCACGAACCACAGGGGGTTGAAGAACCCCTCCCATTCATCGTGATCTGGAACCTGACGCCGTAGGGGAAAGTTGTGATGAGGATGGTATGAGAAAAACTGGAGCTGGGTGCACTGGCCGAATATGAAGGCGAGGTCGTCGCGGATGATGTCCGAACACTGAGCTGATACCACGCAGGAGTCCCAACGGATACTCTTGATCAGGTTTCCAAGCCGGGGGTCGACGTCGGCGGCACGAAGCGTCTCTGCCAATGCTGAGACCTGGCCCATGCGCCGGAAGACGATATCGCCGTATAGCGCGGACATGCCTGGCCAGAATGTTGCGCGGCAGACAAGTGGAAACCCCTTCTTGATACGCATGAGTTGC

>AT13316

AGTGTGGAATTCCCCAGAGCTGAACGAAGGTCATCGCATAGCTCGCACATGCCGTGAAGATTCAACGTACTGAAGGAATATGCGCTGGTCATTGACGAACAGGGAGTAGTTGTCCCATTGTACAGCATCGGTCAAGCCATTCGATTTGCGAGGGGGGTCTGCGAGCAAGGTTTTGCTTGCCGCCCCAACAGAAAGGGAGAGGGATATTGCAGAGAGGAAAGAGCTCAGGATGAGGAGGGCATGAGTAGCCCACGACGAGGGGCGGGGCCAAAGCATGACTTCGACGTGCCGTCGTACGACGCTAGC

>AT13423

TCATGACAGCGAGAGCGGTGGCTGAGAGCCAGAACGCCCACCTCTTTGAAGAACTTGTGGCGCGCTTGCCAGTTGGGCGGGGCGGAAGGGACGGGAAGAGAGCTTGCAGAGGCTTGAGGGACTGTCGAAGTAATCACAACCTCAGCTGCCTGAATCCACGAGGAACGGTGTCATCAACATACCTTGTAGGGTGGATACCTGCATCCGAAGCCGACTTTGTCCACCCTAGAGTAAGGCACAAC

>AT13677

TCTTGGGGGGTAGGGGTAGAGGAGAAGAGGATGATAGGACTTCCGAGACGACGCGGTGGCCGATCTCTCTATACCAAACAAAGGCACGATCGCGTATGATCGCCGAAGGACTGAGGCATAGGGTGGAACTTAGGCCGAGGCAGGTCGCAACCTCCTCGGCTGTTCCAGCGTTTTTGTCAAACGCCTGCCTCTGTGGGTTAGAGGTTAATTAGAAAATAAGGCGTCCGCTCCAGGTTCTAAGTACGTACCGGATCGGTTATGTAACGGAGTTGGATTTGTGCGTCTCTGGGAAATACCGTCGAAATCTTGAAATGCTCCACAAACTGCGCGCGGGCAGCCTCGATGATTGCATCAACAGTAACATGTTTTCGCCGGTACGGGAAGTCAAGCTTGAGATAATGAGGCTCAATATTGAGGAGAACAGGGATCTGGGAGGTCCGATTCCGTTTCTCCATCGTTAACTGGATGGATGGTGAGGCCCAGTGGTGAGGCCTAGGCCTAGGGTTGGGGTCAAGGAACGAGCCTGCACGGC

>AT13685

CTCACGATGAAAGCCGCGCACTGCTCTTTGTCTGCGGTCGGCAGGTCGACCATCGCGTCCAAGACCTGAATGCGCGTATTCGCATCGATGTAGATGGACTTGTCTTCCGGCCCGCTACGGTTACCGTTCGGTCAGCTCTACGTCCAGCCCGTGAAGAGGACGGTAAGAAGACGCACACGTCAACAAACGCGGCGTGGACCGCTGCGCTGCGAACCTTGACGGCGACCGCCGGGTTGAGGACACGCACCGCGGTCTCGAAGGGCTCGAGCCCGAGGTCCTCGTACGGAAATAGGCGGTACTCGCCGCTCTCGACGCGGAGCGCGACGCCTACGGCAATGTTGTCCTCGTTGGGGCGGAACCAGGCATCGCCTTGGGTCTGCTTGAAGATGTGGTGGAGGAAGGCGTCGTAGCGGTCGTAGTCCATAACTGCAAGGAGTGTAGGGCTGGCG

>AT1377

CGTCCGCGGGGAAGGCCACGGGGTCCGTCGTGAAGTCACGGTACGCGTGGTACACGTCGATGTCGTTCCACATCGTCTCGAGCGGGACGCCCGCGTCGCGCATCGCCTGCGCCGACGCCTTCGTATCGTTTACGGTGAAGTAGCCCCACCGGCACAAGTGGAAGCCGATCCCCCACATCGGCTGCCACGTCGGCAGGCCGATGAGCGCGCCGTACTGCTGGACGACGGATTGAGGCGAGGGCCCGGAGAAGAAGTACAGATCGAGCGTGCCGCCGAGGAGCCGGTACTGGATGAGCGAGACGGGCGAGCCGGGCGGGGTCTGCAGGAGCGTGTCTGAGCCGGCCGCGCTGT

>AT1380

CGACGGGGTCGGGACCATCCCGGGCCCAGTTCGTTTGAATGGTACCAGGAGTGCCATTCGCGCCCATATCGCGACGGAAGCCACTGGAAGCGACGGCCTCGCCAATACCATAGACGTTGGCGTCAAGGGGCAGCGCCGACGTCAGCTGCAAGTACTGGTCCTCGAAGACAAGTGGGAAGCCATCAAAGCCGAGGGCAGGGTCGGCAGAATTTTTGGGAGCGATCGGGGTTGGAGGCAGAGAGGAGATGCGCGTATCGAAGAGCGGCTGGGCGCCGGGCTCAGAGCGACGGGTGATCCAGAACGCGAAGGGCGAAGACTCGTAGTTGAAGACGAGGTCGGACTTTTGCTTAAGTGAAGGATCACGAGAAGGGGAGCCAAGCTCGATGACCGATGACGGGATAGTGAATTGCGCGTTGTCGGTATCGTGAATGTTCACGTGAAGCC

>AT1384

ACGCTGCTATTCCATTGTTGAGTAGCCTGCAGTCCAGGAATCAAGACCGTCGCACGGTTCCAGGGCAGAGATTCCCATGTGCCAGTCGCCGTGAAGTTCCTGAGTGGCGTGGGGTTGGCGCGCACATCTGCCAGCTTAGCTTGGGCGGCGGCGAGCTCGGCAGTATAGTTGTCCCTCTGCCTCTTCTGCGCCGGCGTCAAGTTCTCGACGGGAAGTAAGGCGACTGCGCGTTTCCATGCGCGGATCGCCCCGTCAAGGTAGTTCATGCTCTGTAACGGTGCGCGAGGCGTCAATGTCGACGACGACCGTCGTTCATGAACTCGCCAAAAGTGTTGGACCTTCACTGACCGTATTTGCCTGAGCCAACCGGGCCCAGGCCTTCGCATAC

>AT14010

CCAGACCACAGCGTCTCCCCGTCCGCAGAGAAGGCTACATGGCACACCGGTGAGTTGTGCTCGGAGAACGTCAACATGCATGACCCATCCTGAACCCTCCACAACCGCACCATGCGATCGTACGATGCCGACGCGACATATTTCCCGCACGGAGAGAAACATGCCTGGTATACCAAATCTTGATGTCCTCGGAGCGAGAACAGCATGACACCCGTGTATGCGTCCCAGATCGTCACCACGCCGTTGCTAGACGCAGTCACGACAAGCATCCCATCTGGCGAAAAAGCGACATGGCGTGTATTCGAGTTCTCCACGCTTTCCAAGACAACTAGGTCTTCTCCGTTCTCGACGTCCAAGAGTTTGACCACCCCGCGGTCCAGCCCAATAGCAAGACGCCTGCTGCTCGCGGGGTCGAAAGCGGCAGCGGCTATAGCAGTATACGAGCCCGAAAACGAGTTGTGCAGAGTGCCGGAGGCGACGTTCCAGACGTAATAGTCGCGACAGGATTGGGGAGCGCATCCGGAGACGAGCCAGCGCCCGTCAGCCGAGATCGCCATGAACTCTGTATCATGCCCGTGGTGGAGCACATGGAGCTGGCGGGACGTATGTACGCGCGTCCCAGAGTCGTACGGTGTGATCGTAGGACGCGGACACGATGATGTCGCCGCGAGGGGACCATGCACAACGGGTGACGGGTTTGGTATGCCCTACGAGCATGGCAGCCTCGCGCGAGCCTTCGGCGAAATCCCAAATTTTGACTTTTAGACCGCCGGCAGAAACAAGATGCCGGCGATCCGGGGAAAACGC

>AT14025

GCTGAGGCACGAAGTTCAACCCGAGCAGCTTGTCGGCAAAGAGGTTGTACATCAGCGACCAAGACCCCTGGTCCCCATAGCCCCCGAGCAAGCGCGACCCCCCAGAAGACGTAGCGAGGGACAACCACGAGTTGAGGAGCGAAGAGGATTGGTTCTACGGCCCGGGTGCCCATTGAGTCCGTCACCGCGCACATACTGCAAACCCGCGATGAGCGTACTCACGCCATACGCTGTCGCGTCGGAATCTTGGCCAAGCGCATGTGCGATCTCGGCCATGGCTTTCACGCCGATGATGCCCTTCAGAGCAATGTTCGCCGAGTCGGTCTTGTCC

>AT14171

TGAGGTGAGGGTTCACCCGCAGGCAGTCGACGAACGCAAGCGTATCATTTCTGCTCTTGAGGGTGACATGCCGACCGACCATGATACGGAGAGAGCAAGGACGGAGTTGGCGACATGTGAGGGAGAAGCTAACTGGTGTCGCATGATCGTCGCCAGACTGCTCGATGATTCGCTCGATGACTTCCCAGGGGATCAGGGAGGCTTTCATGGTGAATACGAGTGTGGTACTGAGGAGAAGAAAATTC

>AT14188

CACCGGACAAAGGCCCTGCTTCCAGCAACGGGTACACACGCGAGTGAATTATAAAGAAATGCGATACTGCTACACTACTCGTACAAATGGGAAAACAGAAACAATAAACAATCTAGTGCAGGCCTAAATTACGTCCTCTCCCCCCGAAGACGGCGAGCCAACGCCAGGTCTTTTGGTTGGATAGTGACACGCTTAGCGTGGATGGCAGCGAGGTTGGTGTCCTCGAACAACGAGACAAGGTAGGCTTCAGAAGCTTCCTGAAGAGCCATGACCGCAGACGACTGGAAGCGAAGATCCGTCTACACACCAAGGTGAGAACCACACGATGGACGGAGGTGGTAATAGAGACAAACCTTGAAGTCCTGCGCAATTTCACGAACAAGACGTTGGAAGGGAAGCTTGCGGATGAGGAGCTCCGTCGACTTCTGGTACCGACGGATCTCACGAAGAGCGACGGTACCAGGCCGGAAGCGGTGGGGCTTCTTAACACCTCCGGTCGTTGCC

>AT14270

CTCGCATGGAACTCTATAACTATGGAATGACAAAGTGTAGCGATGCAAATAAATTAATCTAATGGTATACAAACCCGACATATCATCCCTCTACAAGCCCTGTGTATGTGTATAAGTTAAGAGTACCTATAACCCCAGGATACTAGCGACATGCTCATCGCGAGGTGTCAGCTTGATCCGGTTCGGGACGGGTGACCGCCCCGAGAGGCCATCGTACTCGTTCAGGTCGATCCGCTCGTCGGGGAGTTCGGTCATCTCGAACGCCCAGAGGAGGTGTGAGACCGCGAGGAAGATCTCGCGCTCGGCGAGCGCCATCCCCGGGCAGATTCGACGGCTAGGGTGATGCGTGCGACGGGTGAAGATGTCAACGAACGATCATGATACCAATATCGTCGAAGCGCGACGGCTTACCCGGCCCCGAACGTCCAGTGGTCGCGCTCCATGGGGTTCGCGAGATTCGCGGACTCGGCACATGTAAGAGGGTCGTCGAGATACCGTTCGGGCTGTGGTGGGTGTGACAGCGCG

>AT14412

AAGCATCGACGTAACCGACGTGACGCCGACGCGTTCGACGCGCGTTGTTGACCCCCTATCAGGCTCAGCGAAGCCAGAATGGGTTCCTCCGACATACGAGAAGAAAACGACGCGTGCCACGGTGAAAGAAGACGCGTAGAGCACATCGTCCTCCCACTCCTACGCATATGCGAACCGGTCTTCGAGTGGATACACGTCTCCAACACCAAAGAGACAAGTTTTTCCCATGGAACCCGACAAGCCTGACGACTTCAGTCTTAGTACAGATGACGAAGCCGAAGAGTAAGAAGCCTCCGAGTCCGATCAATTTTGTTACCATCCCTTCCGCCGACTACCCGCAATAATACATAACATTCGATTTTCCCCGCGATTCAAACGGGTCGGCCCCTGCAGCTGGGGCGCTGCACGCCTCACAATACGATATCCAGGCCCGCGCGTCTTCACGTCTCCGATAAATTCGACCTCGACGAGCGCCCTGAACCGCGGGAAGTGGATCGGCAACAGATACGCCGTCAACACCGCCGAGCTCGTAGA

>AT14559

GTGCCAATATATCTCGGTCCTTGTTAGCTCCGACGGAGTAGTCAATATCCGTGCCCCGGTGGTGGGACTCTTCGCGAATCCTGCCATCTGCTCGATCGTAAATCGTTGTCAGTGGGGGTTACATCCTTCATGACGAGGTGTGCATCCGGCGAAAAGATCATAGTTGGCCATCAAATAGGGTATCTAAAACCAGTAGTATAAGTGCACAGTAAAACGTACGGCTGGCCGCTGAAGTGAAGCTTCGATGGCGGCGGGATCGCTCGGCACGATTGGAAGTCTATTGCGAGAGCCGACGGTCGCTCAGCGCTCAGCGCTCAGGGCCTATAGCGCTCGCAGTGCGACTGTTCCGCATGCACGGTGGCGGCTGGTCAGCCGGGCTCGGAATCCTCCTTGGGCCGAGTTGAGGTGATGATACTCGATGCGCGAGTGCTGATAGACGTCACTACCGCCGGCGGGGCGAACTCGAGTGCCGACAGGTTGCCAACCGACGGACGAGCCTAATAGGGGAATGAAGAGACGAACCGACGGAGCTCAGCAGAATGTCGGAGAGTAGCTGTAATACTATCAGATGTATGCGTACCTGCGATCGACACGTTTGCGTTGGGCTGATGGTAAACGTACTTCCCAAGGCGGCGTCCTCCATCATGCGAATAGAAAAAGTCTTGCGGGACTTGATTCTGG

>AT14642

TACCTCATCCCACGTCGCCTTCAAGCTCTCGCGGGCGAGCACGTCGTCCGTGTAGTTCGCCAGCCGCGCGAACTTGCCAGCACCAGCGTGGATCGTCTCCCAGATCTTCGGGCCCTCCCCCGCAGGCCACGCCCCGATGTCGTTCAAGTACGACAGCCTCGCGCGCGCAAAGAAGGGCGTGACGGCGATGTCCGCGATCGAGTACTCGCCCACCGCGAAGCCCTCGGCGGGCAGGAGCGACTGGAGGTACTCGAGCGCACTGTACAGGTCCTCGACGGGCCCGTTCTTCATTTGGAAGGCGTAGAACGCGGGCAAGTACTTGCTCGAGACGCCCTCGATGAAGAAGCGGGCCTGCGCGCGCCTCACGGGGTCCTTGGGGATGATGCCGGACTCGGGGAAGAGGTCGCCGACGAGCTCGACGAGCACGAGCGACTCGGCGAGCTTCGTGGACTCGGGCGAGGGCTCGTCCGCGGGAACCTTGGGGCCGCCGTA

>AT14649

CTCGTAGTAGAGTTGATAACGGCTTTGACGGAGAGAGGACGTGAAGACGCTGCGCGGTCGAGGGTGGGGAGAGGGAGGTGGCGGTTGGAGGTCAAGGTGCGCACGATCATAGGCGCCTACGGTGAACGCGGGAAAGACGCGCATACATTGACATGACGTGCACTCGTCTTCGCCCGATCCGGTACAATCGTGCTTCCATTAGCATTCAATCTCTCGAGCAGCACAAACACTGCTGTCAATAATCCATGACATACCGAGAAGACCACCAGAAACCACTTCAGCCCTCATGACGATCCCACACATCGCCCTGACGGCTACAACGCCGCCGTCTCATCCTTCGAATCCTGGCTCGACACATTCGAGGTATTGCGACCGTTGGGTTCCGTCCGCCTGCGGTCCACTTGACGTATGCAATCATCTTGAGGCCCCACAACAGACACATGGACCTCCTTGAGCGACGCGGCACCTGCGAAAAGGGCTTCTGCGTAGGCATCCACATCCATGTCTCGAAGGTAGACTTCGAACGGCATGGGGGGTCGCGCCTCACCACTTCTACGGGGACGGAGCATTCGCAGGCACGACAGGTCCAGAGTCAGCTTGAAAGTAGGCACAGACGATACCCGAACGACG

>AT1473

CGAACGACCGCACGCGCTTGCCGTACACGTACATCGGGACGGACGCGAGCCAGCAGACGGCCTGGCACGCGCCGAGGATGAGCAGCGAGGCCTTGACGCCGCGCGAGACGACGATGCCGTTGGCGAAGAAGGTCGAGCCGTAGAGGGCCATGTTCTTCGCGAAGTTCGTGATGGAGAGGACGTGCAGCGCGTTGGCGCCGTGCGTGTCGAGCAGGTAGGTCACTGCGGCTCCACCGGAGACGACCATGCTGAAGTTGAGCATCCTGCCACGCCGGTGGCCCGGGCGGAGCGGAGGTTAGATGGGGAGCACGCGGGATTCGAGATTGGGTTGGAACGGATGTACGGTGGCGGCGCAGAGAGCGTAGAGGGCGAAATGGAGAGGAAGCCGGGTGTTCAGGAGTGCCGATGGTA

>AT1482

GGGGGGCCGGGGATGACGTTGATGATGTAAGTAAGTACCTGGGTTCCAGACGGTGGAGGAGTGTTATGGCACGTACGCAAAGCATGCGACCGCTCCGATCCACGGCATGTGGTGCGCGTTTCCGACTGCCCAGCCGATGTACCCAAACACGCCAAACAGCATGCTGAGCATGAATACGAGCCGGTACTCGGGCTCGTAGACCCCGCGGTTACGCTGCGACATCCAGACGATTGCCCGGTCACTGAGGGGCCCATTGACGAGCATAGCTAGAGTGGACCCGACGAAACCACCCAGGTGCGTTAAACCCTTGTAACAGGTTCGGCATGGATACAGAGATTAGTGAG

>AT14887

TCCCGCCGCCGACCATCCTCCCGTATAGTCGCGAACAACGGCAAGTACCGTTGAACCTCTTCCCATGAACCTCCTGCCAGGAGCTGTTCCATATCGCCTGTCGACTTCTTCCAAGTTAGTTCCTCCAGATCCGGGAACAATAATGCAAGTAACGCGGCGACACGGGGTGCCTCGGCAATACGCGAGTCGAAACAATCCAAACTCGACACGGTAGACGTGCTTGGGGTGCCTTTCGGCATGGGGTTTTCTATTCGGCCGTCTGCCAAGACACTGGTGTTTCTGCCGTCAAAGCGCACACCGAGGTGGAAGAGGTCTGGGCAATGGACGGCAAAGGGGACGAGCACGTCGCGCATCGCCGGCAGCTCCAAGTGGAGGCAAAGCTTGCCTTGTGCGAGTTCAAACGACTCGATTTGCGGCCACGCCTGGGCTATGGCGAGGTAGGTCGCAGCATCCAGCGCGTAACGGGCACCCATAGAAAATTTGAAGACCTCGAGGTTGACGAACTTGAACAAGGGTTGCAGATGTTCTAAGCGGACCACTGTGCCCGATTGTCCCAACTGCCCGCGCATCTGGCGGCTCTCCTGCGTGAGCATGACGAGTTCCGAGAGGGCGGACGGCGAAAACTGGGAGCATATTGAGGCGACAAGCCGAGGCAAGTCGTTCTCGGTTGGAATGTCGGTCAGTACGATCCGTGCCGATCTGACGCGCGGGAATGACGCCAGCGTGCCGAACTTGATGTAGTCCTGCGACCGCAAGGAAATCGTCATGGTCTCCAGTTTGGGGAAATGGGCCGGTTGGGTTTTGATTTGCGACCAAGTCATCATATCCCGAAGAAGAATCGCGATCTTGTTGAGGTTCCGAAGGCTCCCGAGGGCGTCGAACACGGCCTCGGTGAGGGGGATATAGAAGCAACGGAAATTTTCGAGCGTGGGGAGATCCCGGACGAGAGCGAGAATGGACGGCATGAGTAGGAGATCGTCCTCCGACTCGAACCGAGGTGGTCTCCGAGTCAGGTGGATATCCTTCAGAGACACAAAGCGATTAGCGAGGAGTGTGATGGCGGACCGGAGGGGACTAGGGTCACGATGCGCTTCTGCAATGTGGATTTCGATTTTAGAAAGGTTGGGATGGAGGAAACCGAGAGCCACGGGAAGGTCCTCTTCCGGAAACCGGATTCCAGGCCATTCGAAGGCGACAAGGTTGGGGAACAAGACGAGGACCGGGCGATACGCGGACAACTCCCAGAGCACCCGCTGCTCAATCCTAGGGATCCGACCATCGAAAGCATCACCCGGCTTTGGGGGTGGCGGCATCCCGAGCTTTCGCACCAAGCTGGAATACTTGAGGAACGT

>AT15014

TGGACTTGAACTCTTCGGGCACCTCGACTGACGGCTTTCCGTCTGCCTTGAGCTTCGCGAGAACTTTCGGGTTCTCGAGCTGCTGCGTGCGGGACGGACGGGATATGTATGGACGTGAGCCCTTACATTCGTAGATGAAGTGTCCTGATAAGGATGAGACGAATGTGAGCTCTCTCCCAACCCCCTGGTGCTGTGGAAGCTCTACGTACCGGTCTTGAGACACTTCTGACAAACGGTCGTAGGGGCAGCTCTGGGGTTGGAGGAGGAACTGCGGTGCGGCGCGTACTTGGACATGGTTCGTGAAGCGTATTAAGGTATATCCCAGAAGTCGTCGGCTAGATAGGAAACAAGACAGTGAGGAGGTGTATAGGGAGGTGTGTTGCCGTGTGTTGGAGTTATAACTGTTTTCAAAATGATACGGAATCGCATTTTTGCTTTCCGCGTCTAGACGCACAGGTCCGTCCGTCCCGACGGGGCGCCTATGGCCATCTCCACGAGCGTTCAGTGCAGCGAGACGTTCGAGGCGGTCAATGGAGGAGCGACGTCGCCGGTTGCTGACAATAACAAATGGGAAATGCCTTGTATACATTGCTACATGTACCCGGCACATCTAAACCATCCTTGTGCCATCTCCGGAGTAACCTTGTAGCATGCCTCGCTGATGTCGGAGACACCGAGCTCACGGTTGGCTCGACGGAGCTGGGACTTGATATATCCGAAAGCAAGCTCGATTGGGTTCCGATCGGGGGAGTAGGGGGGAAGGAAAATGAGC

>AT15222

CAAGTACGAACCCGGCGTAGAGCGCCAACTCTTCATGAAGATGAAATGAGCGTATGGGGCCCCAAACCCGCTCTTGGGTCGAAAGTAGTTTGGCCACCACGTTGCGCGTGGAGTGATGGTCATGTCGTCCATCTGCTCGATATAACAAGCAGTAACGCCCTCCAGCGTGCCGGCGATGATGGCGTCGGCGCCGACCTTGGGCCATAGTTCGTAAGACTGCCTCAGGAGGTCGGCAAAGACGTCTAGAGCAGGGTTCAGCTGCTTCTCTCCCGCTGCAAAGCGGCGGGAGAATTGGGCCACTGCCTCGAGGTGCCGATCGCTGAGGTCGTCGACATAGAAGAAGCACACGGTGTAAAGCGCAATATAGTACTGGTGCTCGAGGCTTGTGTGCGCATAGACGATCTCGGCGAAGCCGCAGGCGGCGACTGTGATCCTCTCGATGAAGTTTGGGTCGTCGCCCGCGGCCTGGATAGAATCCACGACCTTGCGGGAGATGTCATCGTACAGCTTCCCGCCGCCGGGGTGAGCTTCGGCGGCGCGGAACCCGTCGCTGGTACCCATGCTTTGGTTCAGGAAGTCTTGCAAGATCTCTTTGATCCCGTGGTTCTGGC

>AT1532

GCGGTGTAGAGGGCGAGTTGGGCGGGCTGCTCGCGGTTCTCCAGGGAAGACTCCAGTCCCAAGAGCGATCCTGGGGGGACGTGCTGCATCCAGTCGACGAGCTCGTTCGGGAGCGTTGCGACGCCCTTGACGGTGAGGGAGAAGTATCGGAGGAGGTCGCGGAGGTGCGGGGCGGTGTGCATGGTGCGGGCGAAGAGGATGGCGCGTCTGTTGCGGTCCGGGTTGGACATGATGGTGACGAACACCCGGCTGTATAGGGCGGATTGGGCGGGGAATGTCCATTGGCGGCAAACCAGTGCCGCAGAGACCCACGTTACGGTTGCTGGGAGTTGCTTGGGTATCATGTACTTGCGGGGTTGGTTGTCCATGAGCTCGAAGACGCGACGAAGGAGGTCATGGTACAGCATTCTCAGAGGGAAAA

>AT1539

GGGGGGGGGGTCCAGATCTATGTGAGATTTCCCGGGTTTTCTGCGCTTGATTAATATTCCCAATGCTAGTGCTGACCGTCTTCTTGGAAGGATCCTCGTCGCATGTTACCTATCTGAACCATATCTCCCACCTCCCCGCCGAACCCCCTGCAGAACCTTGTTTACGCCAATGAGCTCACATGCCAACGAGCTCACTCGTCTTGCCCCGACTTATGCTGTACATTGTGTGTTGAAATATTCTTTGCCTGTGTGTATCTAGTCCCTCTCCTCGCCCCCCCCCTCCGTGCTTTCCCCCTCTACACCTCTCAGCACGTACCCCCCGAGCCCTTCGCATTCAAGAGGGGGCATGTACGTTGTCAGGGCTGGCACTGAAAATCGCAACATTTCGGAGCGCAAACGTCACGGTCGACCGCGGCCGCGATGGGCCCACCAAAGCCCGAGCCCGAGCCCGAGGTGGGGACCACAAAGTCGGCCGTGCACAACACCTGGGGCAGCGGCGGCGAGAGACAACAAATGAACGGTGTGAGCCAACGCAGCAGCTTCTTCCCGAACAGAGACCCGCAGGCCAACACGTAATGGCAGCGTGACCGTGACGCACCTCTCCCGGGTAGACGTTCGAACACCGCCCGTCCACGTTCGGGTTGTTCGCGAGCACGAGCGCGACCGTCGTCCCGGTCTCGGCGGCGATGGACTCGCAGGTGTCCCCAGGGGCGACGACGCGTGTCGCCCGGCAGACGTGGCCGAAGACCCCGAGGCACAGCGTCTGCAGCGGGGGGCGTGCCGTCCGTCACGGTCAGGGGCGAGCTTTCCTTAGTATGCGAGGGCCGGACGCGGCGCGGCGCGGCATGGGCATACCTCTCCTACGGGCGGGCTGCTGCAGGACGCGTCGATGGTCCCTTTGTTCACGGATGCGAGCTGGAAGCTGCGATTGCGATTGATTTTGATTTTGATTTCGGGCTG

>AT1557

ATCTCCCCCGGCGGATCCCTGATGCGCCATTGGGCTGTCTACATTGTTTGTTTTTGCGCTAATGAAGGACAGATCGCTCGCGCCCGTCAACCCGTCATAAACCCCGCCTCTCGTTAACACGCATGCACAGGCCCGTACTGGTACCCCCAGCCCTCCGCGGCTCCGCCTACGCTGCTGGGAGGAGGGTCCAAGTGACGTTGTGCAGGCGGCCGACCGCGAGCGCCGCGAGCTGCTCGAACGCGACGGGCGAGAGGTCGACGCTGCCCACCGCGCACGTCGGGCACGTGTCCTCGACGGTCACGGTCACCGACTTGCCGTCGGCCGTCGTGACGTTCATCTTCCGGCCGCACATCGGGTTCCTGTTGGGGTTCGTGCCGGCACCGGGGAAGGCTTGGATAGTCTTGATGTCGACGGCGACGATGAAGTCCGCGTCGGTGTTG

>AT1581

GGCACTTAGTGAGCAAGGCACGCGGAGTCCGGAACAATGGCGGCAGAGGGTTAGAACTTCATGCCACGTCCCTACTCCACCTCCTCCCGCCTCCGCTTCGCACCTCTCTTCGCCACTCCGCTCATGCCCGCAGCAGGGCCGTCATCCGCATGCCCGTCGGGCCTGGGAACGCCCAGCACCTGCGCGACGTCCCCTCCGAACGCACAC

>AT15913

TCCCTGTACAAGTCCACCGGGTCGCCGATATCCTCCTGTTTGAGGTTGGCCTCAAGATCGAGCCAGAGGTGGACGAGCGGGATGTACTCGGGATCTTCGAAGTCCTCCGGCAGCTCGAAGATGTCGCCTTCATCGGAAGCAGCGACCTCGGTCGGCCGCTCTGTGGTGGGCGGCGGCGACAAAGGCTCAAGCGCGGGGGCGCGGTCGGTAGGGACGGACTCCACCTGCTCGCGAGCCGACCGTGTGCGCTTCGCGCGACCCAGATCCGGACGGAAGAGCAACTCCATGTCCATTCGCTCCCTCACCGGCAGCCGGATGGCGCGCTTGTTGTCAAAGCCGTCCTCCCGCTTGTGAACGCGCACGTCCAGGTCCATCGCAGCCCAGTGGTAGCAGTTGTCGTACGGGAACGCCGAGCTCGGACGGGCGGCGGTACGGCCAGCGGGATGCGTCTCGTTTGGGAAGATTGGAACACACATGTCGGACTCGTAGCCCCGCTTCTCGTCGACCGCCCGAAGTGAGGGGCCGATGGGGCGTACGTGGTATGCGTACCA

>AT15934

CCCGCACGAACCGCCGCCGGCAATTACGACGTGGTTAGCCTTCTCGAACTTGTTTCTCCACGAGCCGATGTGCGATTTTGTAGCCGACTCGGTGCTGGGAAGCTCGAACACGTCCGGCCAGACGGCCCCGGTCGCAAGCAGGAGAGTAGCGTAGGGTATCCTCTCGCCGTCCTCGAGAACAACCTCGCCGCCCTTCCCGGGAGCCGACTCCGCGATGGAAACGACCTTGCCGTGCTTGATCGTGCCATTGCCGTTGTGGAAGAGCTTCTGGAATCCGAAAAGCGCCTTGTCCTCTAAGCTCTCCTCCGCGGTGACGGCGATACGTGCCATGGCGGTGAGATGGGTGTAGAAGGGACGCGCGTCGATGAGGATGATCTCGTGCTGGGACGGGTCGAGCTTCTTGGAGAGCTCGCGGGCCGCGGTGCCCCCAGCCGAGCCTCCACCGACGATGACGACGTTCTTCTTGTCGCCCTTCTCAGCCATTTTGTACGAGTGTTGGCGTTGGGAAGCGAATAGGGAGAGGAACTAAACCCAGAATGTAGGAGAGGCCCGGGGAGTAGTGCGAGATGTGTCGTCGGAACGCATGGTTGGCCTTTTTCCTCACTTTTAAGCTGTGCGTACCGTAGCGATCATATTGTATACGCAAGCTTACTTGTCCCCCAAATATTGGCCGGTGCCGTCCTGCTTTAGGGGAAACTGTTGTCGGC

>AT15936

TCCCAGAGGGAAAACGCGCAACCAGCCAGTCCGGACACCGACCCTACGCCTCCCTTAGCCTAGGCCACAAGGAATTTAGCAGAACCTCGTTGTGGCCCATCAAGTATGGTCTCCACAAACGCTTCACTCTGCATCAAACACGAGCGCCCTGACCTCGATCGACTGCCTCGCCGGCGCGTCCGACGGGCTGCCCGACTCCCGAAACGCAGAGTGCGCACACAACCGCGCCCGCCGTCCGTCCTTCCCTTCCCCGTCCTCCGAGTCGAAGATCTTGATCAGCGCGACCTCTTCGGGGCGCTGGTCCGAGAGGTAGTAGAACCGCATCGCGGGGTTGTACCGTACGCTGAGTGTGCTCCCCACGCGGTCGGGATACACGAGCTTGACCGGCACGAGGTCGGAGCCGTCGCGCGCGAGCGTACGCCAATCCGCGAGCGCGAGAGGAGTGTGCGCGACGGGGTGCGCGATGGGTCGCCAGACGTTGATGATGCGCACGCGCGAAGAGAGGAGGCGCGCGGCGGACTCGGGGAGGTGGCGGTGAACGC

>AT15987

CCCCCTTTTTCGCACCGGCTGGTGTGTAAACGTTGAGCTTGAGGCAGTCTTCGCCGCCTGCGCCTCCCGCGTCTCCCACTGTGTATCGTGCATATTAGGTCCGAGTCTCGATTCCATTGGATCAAGTTTAGGAGAACGTGCCACTCACCACCGGCACTTCCCTGTATGCAGAAGTTGGGATATGTGGTCAGATCGACTATCTGACCGGCAGCTTCGTTCGAAACACGCGAAGTGTTTAGAGAGAGAGGCGACCTCCATCGACGTTCACCCAAAGGAGGCTCCGCGTACGGAACGCCAAGGTATGCGACTGTGTTCGGATAGGACAAGTTGCCGCGATACTTGACATATCCAAGGTCCACGACGTCCGGTGCCGCGCTGTTAGCAGCCCTAGCGGCGAGTGCGAACAGACCTGCGAGTATATGTAAATTCAGGCACGGGATGAGACGGGCGACGAGCATGTTGTGCAACGCGTCCGTCAGCTCGGTAGTGGGATCAAACAGTGAGGGGAATGGAGAGTGAGCTCGACGGGTCGACAGCGAATACGCAAGATCCTGCCAATTTATACCATGGTGCCGCTTTTGTGCCACGTTGAGAATGGATATTTGTGGTGGCGCCGTTCTCCTCCTTTTTCCAAGCCACGGTGTGCCGAGCAGTGCGTCGTAGCGAACCGGAAAGGAGTCCAGCGCATTTGGAGACTGAGATGGGCCGAAGCCTTGTATCACCAGACTCGGCTGCCAGTGCCCCAGTAAGGAATCCGGAGTATTTTAGGTGGTGATCGTGGTTCGGTTACCGTATTCCAAGCGTGCAAGCGGAGGTACCTGCGACCAACGTCTGCGACGCGAGAAAGGGCGACTCGGAGAAGAAGACGGTGCGATGTGGTGGGTATTTCGCCTGCTCTCCGAGAAGTTGCGAAGGCTTCGGCAGACCTCCGTGCGGATCGTCCTTGGGCCAGCTGCGTAACCGGTCACGGAGTGCGATCTGAGGCCTGAGGCTGAGGCTACCATGATGGATGATATGGGGGAGTCT

>AT16015

CGGGACATCCGACACGCAACAAGGAAGCCGTGAAAACGTACCTTATGACCATGGCTGATTGGGATCCGTGCAAGCGATGCAGTCGGGAGCCGAAAAGAAAGGCCCAAAGGGGTACTCGTTCAATGCCGGGGGACAGTAGGGTGTTATGAGCTTACTTACTTATAATAATAGACATGCCTGAGTGTCCGGAGGTATGTAGTAGTGAGCACTTACCTTAAATTCCAAATCCTATATATGCCCCACAGACGGCCAGAACGTACCTGAACGCCGGAAGCTAATATGCCAGCACACACCTCGGTTTGGGAATTGTCTTACAGCCTGTTCGGTGCCCGGCTTCGGAAGAAAGTGGGGCAGATGGTGTACGCACCTGCTACAGGGAATGCACGAAGCGATAGACAAGCGTAGTCACGTACCGGGCTCGAAGCTCAAAGGCCGATGAGTCCCTCCGAAGACGGGAGGCGATGGCGGATATGGTGCCCGTAGGATGCAGAGACAGCTCAGGAGGATCGGAGGGCAACCATCGACGAGATAATGAGTCGAGCCA

>AT16036

GCAAATATCATATCCATGCTCCCTGGGATAAACACGGTCGAAGAATGCCAGTGATACGCCATGTATGAAGCCAGCGTTGGCCGACGCTCGACGTATGGCTGACATCAAGCACAAGGCCTCCATGGTGAGTTATCATGCCACAGTTTTACGCTATGTGAATCGCGTTGTGTCCTCCAAAGCCCAGTCACGCACGCAACGTCGAGGACAGGGTGGAGACGTGTGTACGGGACAGTGCGTCTCCACTCGCGTCCTGCCGTCTTTCAGGACTGGCGCCGTCCAGAGGGGCATAAAACATACGTGCATAATACAACAGGTAACCGTAAGGTATGTATAAAGTCAGGACAAAAAATCCCTCATCCTGCTTCCGCCACTCCGGAGGCGGTGGCATGACGCCGCGGACGTGCCATCAAGCAGCGTTCACCTTCACCGCACGCAAGACGTCGAAGTTGTTGAAAAGCCCGCCCTTGGTCGAGGACTTCTGAGCAAGGCTGGCTCGGCTTCCTTTAGACTTGATGGATTTCTTGTCCGCATCAGCCTTCCCGCAGACAAGTCAGTGCCCGAGACGCTAGAGCGTGCGCGATAGGACTCACCTTCTTAGCTGACCCGTTGGTCAGCGAACACGTCGAGCCTGTGCCGTGCTTTCCGGTGGACTGCGCCGGGCATCGTAAGGGAGGAGGCAGTGTTGAATAGGGTCGGCAGGACGTTACTTACCTTCCGGGTGCTGGGTTCATGCCGCTTCCCGAATGCTGCGGGGAAGACATTGTTCTCGGGATCTTCAGGGATCATCGCGGGGC

>AT16039

GGCTTCCGAGGTGAGTTTCTCATGTCGCATGGATTTGGCATGGTGAATCCGCCAAACCCCAGTCCTCGTCACGCGCGCACCGCCGAGGACAGAGTGGAGACATTGTGTCCCGGACGGTGTGTCTCCACTCGCGTCCTCGCGTCCTTCAGACATCACACTGTCCCTTCCTATCGTTTACGGGAGGGATGAAGCATACATGCATAATTCAACATATAGTCATGTGGCCCATATACAACCATGACGAAACGTTCGAAGGACATAGGACACCTCCACCCCAAGCGCTTCCGCACCGAGTCCTAGGACGGGGGCATATGTACCGTCAACCCGCATTCACCCTCACCGCACGCAGGACGTCGAATCTGTTGAGAAGCCCACCCTTGACCTTGGCCTTCTGAGCAAGGCTGGTTCGGCTTCCGTTAGACTTGACCGAGATCCAGTCCCCATCAGCCTTCCCACGGGCAAATCAGTGCCCGAAATGCCACGGCGTGCGCGATTGGACTCACTTTGTTGGCGAATGTATGAGTGAGCG

>AT16277

GCCCCACTCTCTGGCGCGAGGTCCCTGAACTTCTGTATATGCGCGCTCAGCGTCCTGTACCCCTCCGCCCTTGCGTCCAGGGTGTCGTTGTACGAGAACTGCCACTTGACGGACAGATGCCACACCGCATCCCGCCACGCGGGCGTCACCGCGGTCGCGCCTGCGGCGCCCGCGAGCGGGGTGTACAGCCAGGGCGTGCCCGCGACGACGTACGGGCTCGCGAAGGCAAGCGTCGCGTTGATGAGCGCGCTCAGCTGCGCGCGCCCGTCCGCGCTCGAGAAGAGGCGGCTCGGGAGGAGGCGCGTGCC

>AT16289

GCTGGGCAGGACCCTACGGTGCTCGGAAACCTCGCACACGTACGCCAGGGTCATCCCTGCCACTGCCGTAGTACCTGACGGAGAGTATACGGGGTCGAGGAATGCATCGACCATTTGGAGCTTGGCGTCCTCAATGGTTGTCCCGAGTACGTCGAGGCGGCGCAGGTTCGGGAATCCACCCAAGGACGTGCGCAAAAGGTGCCCGCGAGCGAAGGGCATGCTACCGTCTGGGTTCGGCATGTCGAGATCCACTGGAAGTTGGGTGACTGTTGGCCACCGATGCACCTCCGGGAACCCGCCAAAGCTCACTGGCGTGGCTCGGGTTGACTATCACTTTCCCTTGGACACACAACCGCTCCGTGTCGTCGAGGTAGCAGTGGAGGCTCGCCACCTGAGGATTTCCGAAGAGACGGAGGTACATAGAGTCAAAGAAGGAGGGTGC

>AT16290

ATAACAGTAGGAGGGAAGGAGAGGGTGGCGAAGGGCAAGGTTAGAGTCGCTGCGTTGGAATTTAGGGCCACTTTCAAGCGTTTTGGAAGTTGAAGGGGGCGAGTGGCCGAGCCGCCGGACGCATCAGATCTTGGCATGGGTTGGGACCAATCATGAAGGGCCAGGGACTCCAGAGAGGAACCACATCTTTCAACTGCGGCAACGAAGACCTTGTGGTTACGAGGGCCCTTGTATAAGATGAGAGTCCTCAGCGATGCCACGGCGATGGCCTTGCCGAAATAGTAACCAGGAATGGCGAGAGTGTGAAGGCGGGGTGGATCCGCGTCCTCCCAGGACGGCAGCTTGTCGATAGAGAAGGGGGTGCCGCTCAATTAGTCTTGACCCTAGGGCATGCGTATGCGTAGGCTCTCTAAGTTCCGTATATGCGCGTGCACCAGTGGGAGAACCACCTTGATCCCGTCGGGGCAGGTCACTTCGACTGACAGGTGGGCCACCCTCGAAACGTGAGGCTTGAGGACTGCACTAGGGTCGAGCTCGGCGATGAGGCGCATATAGTCCGGGAGGCCAATAGTGAACAGAAACGGCTCCGACCGCGCGAGGAATACCGGTAGACACTGGGGCCAGTGGATGTGATCGCCACGATGGCACCACCTGCAGTGGCGCTGTGAATATAGATCGAACGGCTTCTGCCCACAAGTCCCGAGTGCGACGTCGCGCCAGTGCTGGCAGATGTGCGTAAGAGCGACCAGTTCCTGCCATCGGCTCCAAGAACCGCGACATTATTTCGACCACGAGTTCGGAAGGAAGGTGGTTGGCTGGAGCAGTGGCGTTGAAGGTGGACCGGAGGGCGACGACGGGTGTCC

>AT16929

CGACTTTTGCGACTTCATTCCGACACGGCCTCCAACGACGTGGCTCAACCGGGAAGCACCTTGTCATGATCCTGTCGTCCACGCCACTTCCCGGCTACCCCGTTCACTACCCGTTGCGCTGCTAGCTCATGAACCTCGTACGGCCCGTCGCATGTCCTCGAGCGCTGAGTCTATCTCTTGAAGAGTCCGACCCCCGATGGTTACTGTTGGATGGCTGCCACTGCCCCC

>AT17011

TCATGTGCAAGCCAACGTGCCGAGGTAGATCCCATCGTAAGGGCCGAGGTGTTCAATGATGTTGCTCGCGACGACGTTCGGCACCTGCGAGATCGTGCACTCCGAATCCGTCGCGTCGTCGTCCCCCGGACACGAGTACGCCTCTGTCGCACTCGTGATGTGGATCTCGCCGTGCGGGTGCTCGTAGCCGAGGAGGCGGCCGGGCACGATGGGGACCGGGTCCTTCATGTTGTTGATGCGGACGAAGTCAGAGACCTCGGAGTCGAACAAGGCCGCCCATGCGGGGTTGCCGACGCGCGGCGTGCCGTACGTGACGGACTTGATGTGGACGCTCGAGGGGAGGTTCAGCGTCAAGAAGAGGGCCTCGAGTTCGGCGAGTGCGGCTCCCAGCGAGTGGCCAACCTGCGGGACAATTCGGACGTCGTGAGAAGCGAGGAACACCATGGATATGTGGATGGTGCGCAGGATCACTCACGGTGACGACGGTGGAGGCACCGGACGAGGCCAGCAGCTTCTTGACCTCGGTGAGAATGGCCGACGCGGTCTGCGCATGCTCGTCCGCGAACCCCGCGTGGACCTGGACGTCCGAGGACACCCCGGGGAAGAACGTGCTGTTAAGGCCCTCCTGGCTGAGATTGAGGTCCGTCAAGTCTGC

>AT17188

CACCGGTTTACCCACGACGTCTAAGCTAGTTCACCAAGAGTCAAGACGCATCCGACCCGACTAGCTTAGTAGCTACATAAGAATATTGTACACCATAGTCTCATCGAGCGAGCCTCACTTGGTATCGATACATCTGAACCCTCGAGCACCGTCCAAGCGAGCACGATTTCGGTCCCACGACTCGAAGCTGCCAGCGGCGCGATCCCCCGTGGCAATCCGGTGAGCCCGACTAGGTACATCTACATCCCCACGACCTTCCCCTCCTCGATGATCACAGAGCTGTGCGCCGTGCGAACGTCGCCATCGCCCTCCTCGATCTCGGTCGCGACCTTGATGTCGATCCTCGTCGGTGCCCGTTCTGGGAGCTAATCATTTTCCCCAGGACGTCAGCGCAGCACTTCCAACTTTGTGCGGAGTCCAGACAGGTCGAAAGGACGCCGCTTACCTGCGGCGCGTTGTACCGCTCGGCGGCGGCGAGGCGCTGCGCACGCGCGATGAAGTTGCGCTCGGTCGTCGAGTCCGTGCCGAATATCTCCATGCCGCCGTTCGTGAGGAGCCGTCTCGAGTTGAGGCTATCCACGGCGTTAGGCGGGTAGAAAGCAGGCAGGGTTGGTTGAGGGGCTCACACGGCAAGGAAGGTGACC

>AT17467

ATGAGCCGAGTCTGACTTGGAGGCTACCCCCGGAGATATTTCGAGACATCCTCCACTTCTTCACCAATGACAGGGAGGCGCTGCTGAGCTGTGCGCTCGTCTCTCAGGAATGGTTGTTTGAGAGCCGGTCTTTCCTCTTCCGAAAGATCCACATCCACGACCGCAACTCATGCGATCTCTTTGTCTCCAAAGTCCTCCGTTCGGAACGCTTACGTCCCTGGCTCACCTCGATACGCCACCTCTCACTCGCCTTCATGCGCCGTCGCGAATATCAGGAAAGCTTCCTCTTTGACATATCAGAACACTTGTCAAATTTGCGAACCATGAAATGGGGTGGTTTCCGTCGCAATCGAGCTCCTGTTCGGTCCGACGTATTACTCGCCTTCGGCAAATTTCCCTTCCTCTACCATCTGGAACTTTCCCTCAGCACCTTTGCGTCGTTCCAAGACCTCAAGAATATCCTCGTCGCAATCCCTACTCTAACTTCGTTTACACTGTTGAACGTCGCATGGTCCAGACCTGATGATACTGCTTCCGAGTCGTCCGCGTTGTCTTCCGCTCCCATCTGGCCCACAGCGTTGTCGAAGCTGTCCGTCTACGGGCCTTCTTATGGGGAGGAGTGTGCAGACGATGTGATTCTTTGGCTATCCACGAGTCCTACACGACACCTCCTCACGGAGTTCTCCTTCTATCTTTGCCACATTGAAGCGGCTTTACAGCTGATGGCCGGTGCCTCGTTCACAATTCTGCACGTGCAGATGGCCTATGGCACTTACAACCTACACCGT

>AT17556

AGCCCACCCCTACAGAAGTTCATTCGACGAGCTCGCGAAAACAATCTGCGGTTGGTCTAACCCGACCACGGTGTCGTACCGATCAACGCGCTCGCAGATTCGGTGCAGCCGGGGTATCCCGCACTAGTGAAATAATTGCCAGGTTCCAAGCCCAGAATCCTCGTAATCTTGCCTGGAGCCCTCTTCCTCAGCTTGAGCTGCTGATAATGATTGTCTTATGATTACCGCCCACACGGCAGTACAAGCCTTCGTTGTCAATTCTGATGGTCGATACAGCGACATGAATATGGAGGGTTTAAGGGGGCGTCTCACATATCCTGCTATCATGAGTCGAAGTTGCACTTGCCCGCCCGCAGGGCACACCACATCGGCTGCAGTTGGATCGCCTTCACGCCCCGCTGCCTCAGCCGGAACGCGACGTCGTGCAGGCGCGGGAGCGCCGCGGAGAGGTCGCCACCAGGTCCGGTCCAGAACACCTCCGCGGACGCGGCGGCGCGCGGCCAGACGATCGGGTCGAGGTTCTCCGGGCCGGACTGCTCTGCCCACAGGGCTTGCTCGCC

>AT17557

AGCCCACCCCTACAGAAGTTCATTCGACGAGCTCGCGAAAACAATCTGCGGTTGGTCTAACCCGACCACGGTGTCGTACCGATCAACGCGCTCGCAGATTCGGTGCAGCCGGGGTATCCCGCACTAGTGAAATAATTGCCAGGTTCCAAGCCCAGAATCCTCGTAATCTTGCCTGGAGCCCTCTTCCTCAGCTTGAGCTGCTGATAATGATTGTCTTATGATTACCGCCCACACGGCAGTACAAGCCTTCGTTGTCAATTCTGATGGTCGATACAGCGACATGAATATGGAGGGTTTAAGGGGGCGTCTCACATATCCTGCTATCATGAGTCGAAGTTGCACTTGCCCGCCCGCAGGGCACACCACATCGGCTGGAGCTGAATCGCCTTCACGCCCCGCTGCCTCAGCCGGAACGCGACGTCGTGCAGGCGCGGAAGCGCCGCGGAGAGGTTGCCGCCAGGCCCGGTCCAGAACACCTCCGCGGACGCGGCGGCGCGCGGCCAGGCGATCGGGTCGAGGTTCTCCGGGCCAGCCTGATCCGTCCACAGGGCTTGCTCGCCT

>AT17559

GGCGGGGGTCTTTCCTACAGCCTCGATGGCCGCGTGCGTTTTCTGCGTGAACACGTCCAGCGCCTCCTCGAGTGTCTGTCCCGCCGCCTTCAGGTCCGCCTGTGTCTCGGCGTCCTGCGCATAGCAGTTGGTGTTCAGCTCATCACCTCCGGTGCTCATGAGCGTCGAAGGGAACATGCGGGCCACGGCCGCGAGTAGCTCGGCGGTGAAGTTGGTGGCGGCAGGCGAGGCGAGACGGAGCTGGCCAGC

>AT17560

GCGGGCGAGTCGGTGATAGAAATCGGTGCCTCCACCGTGTATATCTCACCAGACCACTCGTAGAACAGTTGCGTGAAAGTCGTAAGCCCACGGAACAGGCCGAGCGTCGAGTTCGCAGTGAGCGTCGCCGTAGAGCCATCCGCAGGGATGCTCAGCACATACTCTTCGCTCCTCGTCCCAATCGCTAGTCTCGCCTCATCTGTGATACTCCGCACAGTCGCCCCATTGGCTAGCGAGAGCTCGAGGGTTTTGAGGGCCTTGGCCTGCTTGAGGGCACCACTGTCCGAGGACCCGCGACCGACGACAAGCCGGCCTAACTTGTCATTCTTCAAAAACGAGTGGGTGCGCTCGACGGCGGCTGAGAGGTCGGATGGCGCATGTTGGACGTTGACATGGATGTCGAAGTCCGAGGCGAGCTTCAAGGCGGTCGAGCCGGTTTGCAGGGAGCGGGGCTGGGGCCAGAGACCGAAGACGCCGGCGGCGGGTGCGAGGGCGAGTAAGAGGGAGAGGAACCTCATCGTAGACG

>AT17562

ACAAGGCGGAAGTTCTTCTCGACCACCTTCGCGGCGTTCGCAGACGAAATCCACACCAACACCACCGTATCGTTCGACAACGTGACGTTGTGTTCCAGCACCATTTCTTAAAGACACCATCCCCCTATATTAGCTACCGACGATCCCACGGACCGAACATCATGCTACGTGCCTTCCCACACAGCAGGGGTCTTGCCTACGGCTTCGATGGCCGCGTGCGTCTTCTGGGTGAACACATCGAGCGCCTCCTCGAGTGTCTGTCCCGCCGCCTTCAGGTCCGCCTGTGTCTCGGCGTCCTGCGCATAGCAGTTGGTGTTCAGCTCATCGCCTCCGGTGCTCATGAGCGCCGAGGGGAACATGCGGGCGACGGCCGTGAGCATCTCTGCGGT

>AT17598

TGCACGTAATGACAACCTTCCGAGTCCCCAGTCGACCTAGAGAACGCGGGACGCACGTCGACAATGCCTAGCTCCGCCCATTCTTTCAAGTCCGAATCCCCGTACAAGAAGTCCTCATTCGGGTACCGACACCCAAAGAACAGCAGGTTCTTCGCAACCTCCCGCCCCGCAAGCTTCTGCATCGCCCGCTCCTGCAGGAATCCCCGCATCGGCGCAAGCCCAGACCCCGCCGCGACCATTACGAGCGGGATCGTTAGGTCTGTTGGGGGATGGAAGTGCACGTTCGACGCGCGCACCGACAACTGAACTTTGTCGCCTGCCTGGAGACCGGCGAGATACGTGGACGCGACGCCGAGGAAAGGCTCAGCGCGGCCCGAAAGTGCGGGGGCGTCGACGACGCCGATGGTGAGCGAAACGCGCTGGGCGTTCCAGAGCGGGGAAGACGAGATCGAATACTGGCGGATGCGCATGGATGGAATCATCTGGAGGAACGTCGAGAGTGGAAGTCCGATGTCCTTATTCTCTTCGAGGATGTCGAGGACGCTGAGGCGGGTCTTGAATACCTTCTCGGCATAGTTGGTAGAGAGGTGTTTGATGGCCTGTGTGGACGCGTCGGAATTCTTCGCCTTGAGAAGGATGTCGAGGTCACGCTGTGTAGCGGGCTGTTGGAGCTCGACGTATCCCTTGAGGAGGTTGTGGATGGTGATGTGCTTTCCAACTGGGAGGGACGTCGGAACAGACGAGCTG

>AT17610

GTTATACTAGGAGGTAGAGATAATTCACTTGTGACTTCCGTATGCTTGGTACCAACTCGGCCGACCCGGCCGACATGTCATTCGTTCAGGCTGACGGGCGCAGCGGGGAAACGAGAAGAGGCAGTCCAGGGTCAGATCCCGGCGCACCGACGGTGGGGTACTGCACCCCAGCAAAGTTCCAGAAAATCCGCTTTCCGGTCGGCTTGAACTCGAAGTGCTGCAGGAGGATGAGGAGGACGGTCTCTGTGGGTGGGATGGAGCCA

>AT17611

AGCACTGCACTGTAGTACGATGGCAGTAGCTGGGAACTCAACGTACATGCACGCCTTGTTCCCGCCAATGAATGTCATCCTTAAAGGTCACATGGACAACATCAGTATGCGCGACTCAAGTACCAAGGGCTTATACGTTGACGCACAGATGGGAATACACGCCCGGGACGTGTGCTTCTTCGACCGCGCGCGGAAGGGGCTCGAGCCAGCGCTCAGGCCGCCATACGTCGGCATCGGGCCCCCATAAGGCTGGGTCGGTGTTCGAAGCGCCAATGTTGGGGAGAATTTCCGTGCCCTTGGGGACCGGGATCGCGTTGACGAGGGTTCCGTCTCGCATGCGGATGGGTTCGGAGAGTGGGAGGACGAGGTCCTTCGCCGC

>AT17754

CACGGAACGAATACACGGCCTGCGGCCGTCGCGCGAGCGTCTGCGTGAACAGCCGCCGGGGCGCGATGAACAGCAGGTCGCCTTGCAGCGCCGCGATCCGCTTGTACTCGGGCGTGTACGTGAAGTTGTCGCCCGTGCCGTACGGCGAGCCGGCCGCGGGGTCGGAGGGGTAGAGCTCGAGGAGCCGGGTGACGTTGGACCGGGGGATGTTCGGGAAGAAGCTGCTCGAGACGTAGTCGAAGAACTCGTCGTCCGTCCTGTTACAGTAAGGAGTACATAGAATTGCCGCGGGCGAGGGTGAGCAAGCCTCTATACTTGGTGTCGCATCCTACCTATGTAAAGGAGACGAGACTTACGTGACGTTGATATTGGCAAACGAGAAGATGGTGCCCTCGTCCACGTCGTTCCCGCTCACGAACGGGACGTCGGCGACCTTGCCTGCGAGGAGCTGGTGCTGGGCGGGCTGCGCAAGGGCGTTTCCGTCGGCCCGAGGCTCCCAGGGCTGGTTGAGCGCCTGTCATGCCGATCAGTTTGCCTCGCATCTCAGGCTCGACGAGGGTGATCGTGGTCATCGGCGCGAAGGGGACTACAGTCTACTTGCCTTGTAGCTGGACAACGTCGGGGCCTTGTTCACGGCTTGGGTGAACGCGGCGGTGCTGACGTTCCTCAGGCACGCGATCGCGTCCTCGCTGCCCGAACATCCGGCATCGGCCACGAACTGATCGAAGGTCGCCTGCAGGAACGAATTGTCGACGTATCCGGTGGGCATGGGCGCGCCGGATTCCATGAACGCCGCGCGGAAGAGGCCCTCGGGATCGCCGTTGTTGTAGAGCATCTGCAGCGCGACAGACTGGGCGCCTGCACTCTCGCCCCAGCTAA

>AT17771

GCACGCTTGGACGTCACACAGCCGCGGCCGCGCAAGGACCGAATACTGCGTCATGCTGACCTTGTTGAACCACTCCCACCCGGGGCACTGGTGCTCCTCCGCGGGGAGCGAGCCGTACGCCCACGCGCTGCCCGTCGCGCACACCAGGAGCACGACGGCGCCGAAACCCCTGTCCCGCATGTGCAGGCCGCTCCTGAGGTCGCTCTCGAACGTCGGGCGGTGGAGCAGGCCGAGGTAGGTGTTGGAGTGGACGAAGTAGAGGTCGACGAGCTGGGCCATGACGTCCGGCGGCGGGAAGTCCTGCTCCCTGAACGGCGGAACGATGGAGGACACCATCGCGCCTATCGTCATCATGTCCTTCAGCGGCAGCAGCCGTGTCAGTTGCCTCCTCGTACCACCGGACGGCTGGAGGAGATGGACGCTTACGGGTGCGAGTCTCTTATGGAGCCGAGCGAAGGGCACTTCGTACTCGACGTCCGTGGAGGCGCGCTTGTGCTCGCTCTTCAGGTCGATGACGGTGCGGACGAGGTCGAAGTTGCTCGACTTGCCCATGTAACCTTTGAAGCTGCCGTCCGTTACGGCGACGCCCGCCTCGTTATCGCTCGGGTCGATCTCTTCGGAGGAGTCAGTGGAAGGGTGGACGCCGCGTGGGTAGATGATCTTGTTCGATGACCAGGTTGGTTGCGGTTGCTTGGCGACGTATGGCGGTCGCTGCGGCTTGAGCGTGTCCTCAAGACGTTTGGCGTCGTCTCCAGTGTGCGCAGCGTCCGATTGCAACTGAGTTGTCGGTCAGCTTGTCAGCGTAAGACCCATCGCGAACGAGCAGAGTGTACCACCTTCTTTAGAAGGCCCTCCATTTTGGAAAGGCGGGTCTCGAGTGCCTCGACGTATCTGTTGAAGGGGCCAAAGCCAACTGCCGTCAGCGCCGAAGGGAACAATGAGGTCCGAGTCGTATGTAGAGACATCTTTATCGCTGTCTCATCCAAGACGTTTCGGCATCATCATTGAGGC

>AT17775

CACGCATCGGACAGCTCCTTCATCATACTTAAGCAAAGTTCCACATCCTTGAGCGTCGCGCGTGTGTCCCTGGTGCCGGCCCACAACGATATCAAAAGCATGATTCCCACCGTGAACAGCGGCATCTGCATGGGAGTCAGCGCGTACAGAGAAAACCCAGTCCGTGTGGGCATCAAGCGACGGTACTGTGCGTCGAGCACCCGCAGGCACGAGCCCGCCGCGCTCGTGCAGACTGTCAGCGATGGCAGCGAGGGATTCGCGCTCGCGAGCGGCCGGCGCGAGGAGGGAATGAAGCACCGGTGGACGAAGATCTGCAGCTGGTAGTAATTGGTGTAAAGAATCGCGGATTGGTTGAAGAAGACGGCATCTTCGCGGCTGGGGTCCCATCGCACTAAAGTTGCGTTGCGTTAGCCAAGGCATAGAGGCGCAGTGTCAAGGAGAGACTCTTACGGTGCGAAGGGATTGTGTCGAGCCACTTGTTCAGCGAAGAGTCGATTTCGGCGACGATGCGCTGCTTCCACTCCGGACCGACGTAGCCCGATAAGAGCTTTGACTTGTTGGTTGTGAACTGTTTGACGGTCCAGTGAGCTAGTGGCCAATGTATATCTGAGAAGACTTAACGATCGTGCGCGAGGCGAACGCTTGGATCTTCAGAAGTCGCACGAACGCATTGCAGAACGCAACCGTCGACGGTTTCCCGCGCGGTTGCTGGAATGTTTGTAATGGGTCCGCATGCAACCAGTATTCATCGTCACACTCGATCATCGGATCGA

>AT17777

GCGAAGGGATTGTGTCGAGCCACTTGTTCAGCGAAGAGTCGATTTCGGCGACGATGCGCTGCTTCCACTCTGGACCGACGTAGCCCGATAAGAGCTTTGACTTGTTGGTTGTGAACTGTTTGACGGTCCAGTGAGCTAGTGGCCAATGTATATCTGAGAAGACTTAACGATCGTGCGCGAGGCGAACGCTTGGATCTTCAGAAGTCGCACGAACGCATTGCAGAACGCAACCGTCGACGGTTTCCCACGCGGTTGCTGGAATGTTTGTAATGGGTCCGCATGCAACCAGTATTCATCGTCA

>AT18422

TAAATTCTCCAGCGGCGACGCCATCATCGCGGCCAACACCAACCTGCACGAGACGGAATTGAATCGAAACTCAAGTGCTACGAGTGCTAATGACAAACCCACCCACCCCCAGCCAGGTCCGAAGAGCGTGGCCAGATCGTTCACCTGCCGTATGAGAGCTTCTTGGAAACCTGCACCATGACCCAAGTCAGAACGGAAAACACAGTCACGCGAGCAGTACGACACACCAGCAAGAACGATGCCCTCTGAGTTGAGATGCTGGAACACCTTCACCCGCCTCGTGCTCCACGGCTGCTCCCCCTTGTCGCCGAAGTATGTCGGCGCGGTGAGAGGGTCGGGGAGGGCGTTTAGCGTCTCGTTTACCGTGATGTTGTTCGGCATGGAGTCGTAACGTAGGTTGTAGGTGGCGACGCGCATGCTTTGTCCCGCAGCGACCCCTTGGGGACCAAGAGTGAGCGTAGCGGAGAGGAGACCGAGGACAGCGGCGAAGGACAGCATGGCGAATCCAGAGGTGCTGAGCTGGGTATGGGTGGGGCCGAGCGAACTTGCATCCAACACTGGACCTCCTCGAGATCGAGCCTGTGAACTTTTATATACGGTCGATATCGGTGCACCAATGTCCACTTCTGCATGAAATCGAGGACCTCGGCTCCTTTCTTCCGCCGCTCTACGTCGGCTTGGCGAGCTGAAATAGATTAGCAAGAGCTCGCCATGCACAATGACGCGTTCCCGAACAGTCCAGTACCCAACGTCGCATGTGGCTCTCGTCGTTCGTACAGTAGGCGAGGCAGATAACGTCGTGATGAACAGAGCACCTGAT

>AT18426

TGGCTTGAAAAAAGAGTGTCGAAAATACATATATTCGAACGTCCAATGCGACAAACAGCGAACTAGACAGGGCGAGCGAGAGACTAATTGAACACGCGACTACGATAATTAATCGTTATAGATATTCCAATCCTACAAGCACAGGCACCATACGCGTCAGCAACCCCGGGGAGGGAGTAAAGAGGATGAAGACGTACCGAGCGCCACTTGAGACCCCAATGGTCGTTGATCGGTCCCTCGATGCCCCACGCGACACCCTTGAACCACCAGAAGGTGCCGACGATGATGAACACGCAGACTCCGAAGACGATCTGTTTTGTGCGGGCAGTTAGACGGCGAGAGGAGAACACGAAGTGATCGAGGAGATGCGCGAGCATCAGGACCGAGAAGTAGAGC

>AT18439

ACGCAATGGGATAAATTCGATGTCAATGACCATGTCGCGCGCGTCCAACGCGGTGCAAGCAGCAATGGGCGCAGGGCACGTACCTCACACGCGCGGCCTCTGCGGGGTCGAGGACGAAGTCCTTGCCCGCCGTGAGCCCTGCCGCCACATCGACGTTGGTGCTATCGATACCAACATGGGGCTTCTCCGGGCCGTGGACGTCGGTGAGGTCCGCAGACACGCTTCGCTTCTCTACAGAACTGTTTTCGGACTCCATGGGGTGCCGCGAGCGTTGTAGAGTTTGAGTTTCTTCCGAAGGTTGAAGGGAAGGAGGGTGCTGGTCGTCGATGGAGCGTCCTACCAGGTATCGAGTTTAACAGCAAGGGGAGAGACGGTGAACGTCGTCGGGGCAGGCTCGAGGGGGACGCGAGCGCAGGCTCAGAACGGGGGACAGCCAGGGCTCGGGCTCGGGGTACCAAAAAGCAGGGCGACAAGAGTAATTATACC

>AT18586

ATACCTCAAACAGCTGGCGAAAACATTCAACGGCCTCGCCGATTGTCTCGAAACGCACGTCATGGCACCGCATAAGATACGGCATCACAAGGAAGACAATGGATTCGTTGAGGGGGGAATGTAGGACATCGTGTATGGGCACACAACGGTTGTGGGGGTCGGAAGTGAGGGGTTCGGTCGAGAACAGCTTGCCGATCTCAGCCTCGTAAGGATGGATGAGCGTGTCCACCTTCTTGAGGGCGACGACGCGCCCGTCGGACATACGCGTTGCGTCCATAACCTCAAGTCGCTGGTGTTGCTCGCTGTTTAGCATGAGGGGCGAAGGGTCAATGACACGGAGGGCGCACCATGAGTATTTGCCCGTCCTCGACATCGTCATAGAACTTCTTTGTTCTCTCCCATGACGGCTTCCAGTCTGGATGATAGCGCGGCCGAAGCATGTATCCTTGCTCCTGAAGCCACGGCTGATGGTCCCGCCAGAAAAACTCGGCACTCAGATAGCCCCCTTGGAAGTTCTTGTAGAACAGCTTGTCATTGGCATACGGAACAACGCGTTTCCCTGGTTCTTGAGCCATCCCACAGCAGGGATACAGGGAAACGAAGGTTGGGAAAGTATGAGGGAGGGAAAGATGATGAGGGAAAGGGGAAGTACGACGAGGTCTTGTTGCGGGAGAACATGAGACAAGACTCGATCTCGCTTGTCACTGTGCAGGTTG

>AT18605

CTCAGGATCTTCGGCCTCGTCGCCATTGTCTTCATCGTCGGCGTCCACTCCGGAGTACTCTCTATGGCCGCGCAGGCGGTGCACTTTCTCCTCAACACGCTTCTGTCGTTTGCGTGAGCGGAGCGAGGTGTCGTTCGCCACGCGCGCTTGGGCAGATCGCGATGGATTTGGTTGGCTTCTCTGCGGCACCCTAGCCGCCGCAGGCCGTGCTCCGCTAGCAACTTCGTCATGCTGAGAGGTGAGCTGGTCCCTGAATTCCCTGAATTCTTGCAC

>AT18848

CAGTTCTGCGATAGGAACACCAGCGAGCGGGGCGCGATCAGCTCCTGGAGCGTCCAGCCACGCATGAACCACCGGCTCCGGTAGAACGCGCTTCCGGGCGAGCATATTTCCGACCGTGGGGCGTTGGGGACGTCCGCGAGGAACGCGTAGCAGACGGATGCGTCGCGGTACCACCGGTGCATCGAGTTGATCGCCTCCGATAACTCTGAGCTGCTCGTCTTGTCTATGCAGCAAGAGTCGATCCAGATGTACCGATAGCCGTGCTCGCGGGCGATGCGACACGCCTCGCGCACCTTGGGGGTCACGTCTGGATGGTCAAGGATACAGCTTGACGGGGTCGGCGCGAGAACAGATGACTGAGGAGTAAGAACTAAACGATTTGGACTCAGTGTTATGACGTGTTTGAGGGGGGCTTGGTGGGGTGGTACTTACCTCCATCGTTCAGCGAGACATTCCTACTGCAGAGCGCCTGGATCTGTTGGAGATCCTGGAAGGACAACTCGCGTTTATCCCATACGTGCGACAGGATCGCGTACGGGATGCCGCCTTGGTGGTCGATGAATCGTCCTGTTTCAGTGTCGAGTAGTCGCATGCCGTGTGCGGCAACCACTGGTGATTAATATGGTGAGAGTTGGGATGAAGCGGTGAAGTGGAGCCTTGAGGTTTAGTAGTTGCAAG

>AT19267

GGCGCGGAGAGGTAGCTCTTCACTGCGGAGGACTGGTAGTCGGGGATGCCGAAGTAATTGAAGAAGCCCCCAGAGGAGAAACTAACCGCGGTCTCGAAGCTGATACCGTGGGTCACGCCGACGGAGGTCATGTAGGGACAGCCCGAGGGGAAGGTGGTGACGAACGTGGAGAAGCGAAGAGAATGGACACACCGCGAGCGCCGAGCTGGGCATACGCGTTGCAGAGGTTCCTGCACGAAGGATGAGCGATCGACAGCTCACCGGAACAAGAACTGTGTATACTGTGCGTGTTTCTGCGAGATCGTGTGCTCGTCCTGGCCGTAGGAAGTGGTCACAACGTAAGGAGGCGTGTCCTCGTCGAGGTGGAAGTTGATGACATCCAGGAAACCTTCGAGGTCACCATCCTGGTACTGCTCACCGACGGAGATGAAGACCGTCGGGGCACCAGTGGCAATACCGACGGTGTACTGGATGTCGAGGTTCGCCTCGACGCCAGGCACGTCGGATTGAGGGTTAGAGCCACCATCAAGCGTCTGGAGGGTGAAGGTCGTGTCCGATGAGATGTCGGTACGATATTGCTTCAAGAAGACCTAGTGTTAGGAAAAATGGTCAGTATTAAGCTGTCGACATCAGATGCAAATGACCTAAGCGTACCGCAAGATCACGCTGGTTAGCGTACTGCTCCATGAAGCCGGTAACGGCGGGCTTTTTCGACGACTGAGTTGCTGCGTCGGACGGGATGTTGTAGATGG

>AT19293

CAAGTTCGCATATATCCCCGGCACCCGCGCCTGCTCGACGGACCGCGGCAGCGGCCGGAGCCACCGCTCCGGCCTCCACTCGAGCGCGTCCTCGCCCCACACGGCCCTGTTCGTGTTGCACGCGGGGATGTTCGCGAGGATCGTCGTGCCCCTCGCGATCGGGATCTCGGTCAGCAGCGTCCCGTCGGCGCCGCGGATCGGCGAGGAGAGCGGGAGGACGGTGTCCTGCTTCGCGCTATTGGGTGCGGGCGGGGGAAGGGGTGGGGGTGTGGGGGTGTGGGAGTGTGGGGGGGGGGGGGCGAAAGAATATCGAGGTCAGTGCGCGTCTGCTCTGTTGATGATGAGGGATGCCACAATAGCAATATCGCTCACTCTCTGAGGGAGAAGTTTGCGGGGGCGTAGCTGGGGTTCCATCAGACGTAAGCGTCGGGGGAAGACCGCGGAACGGGACTCACAGGCGCAACGTCTCGCGGCACACGGCGTCGAGGTACGGCAGGGCGGAGAGCTCGTCGTACGGGACTTCCTCTCCGTACTGCTCGATCGCCGCGCGGATCTCGGCTCGCAGCTTCTCTTGCACTTCCTGGTGTTGACAGAGGAGGTGGAGGATGCGCGACAGCCCGTTGGACGTGGTGTCGGCCCCTGCGAAGATGAAGGTGCTGGGTCGAAGGCCCCACGAATCAGTTATTGAATCTGCACGCGACGGGACATAAACCGGAGAACTCGAACCTCAGCATAGCGAGCAGCACGCTGTCCGGAAGCCTGTCCTCGTCCGCCGCCTTCATGTTCTCCTTCACTATCGCCATCGCCTGCGTCAGCAATGCCTCGCACCGTCGACATTTGTACGCAAGACGTGTGGCCTACGGAGAATGCTCAGCATGTCCTTGCCCTCGCCCATCGCGTGGAGCAGCTCCTGGTCCCCGCGCTCGATCGCTGCCTTCTTCTCGTTGTAGATCTCCAGCGAGCGCGCGCTGATCACGTCGCAGACCGTCTTCATGCGCTGGATGGACCTGATCGGCACGAGGTCGAGCAGGAAGCGGCGGAACCACGCGGGGCGGATCGAGTTCGCGGTGGTGACGAACAGCCTTGACCATTGGATTTCTATGAGCGATGGCCTGAGGGGATGGACATGGTCAATGTATGCGCGAGAGACGGTGGTAAGAGACGGTAAAGTGCGTACATGAACGCCTTGAGAGCCTCGGCGAAGTCGTTATGGACTTCCTCCACGAGGGGGTCAAAGGAGTACCCCATCATGCCCTGCCCGACGAGCTCCAGCGCGGTGCGTCCCATCCACGCCAGGACGTCCAGGTCCTTCGGGCCGTCCTTGACGGAGCGCTCGATCGCTGTCTTCAGCTGAAACCAATGGGTTAGACGGGTCCGACGAGCATACGTGGACCACGGAGTCGCTTCTCACCTTGCCGGCGATGGCGTAGAATACCGGAGCTAGGCCGCGCATATGCGCAGCGGAGAAGGCGGGGCCGAGGAGCTTGCGCTGCTTTTTGTGCTCGCTGCCGGTCGCCGCCAGCAAACCGGGCCCAAGGA

>AT19319

CGCCTCGGAGGCCCCTGGGACGAGGCCGAGCCGCACAGTGTCCAGAAGCCGGGTGCTCTCGAGGTCGAGGCCGGTCTTGAAGTTCGTGGTGTCCATCTTCCCTGCCTTGCGATACGTCTCATCGAGGACAGTTTCTTCCCCTCGACCGAACTTGGCGGGCTCGCACGCTCGTGCAAGCCCTTCTAGTGGACCATGGTCATTGGACGTTGACATCTCGAGCAGATCTATAAAGCTGCACAAATAGTTTTAGTACAGACTCGAAAAGTAGAGAACTTAGACGTTCCGGATCACGCACCGTGCCTCCTTTCTCCCGTAGTACAGCTCGAAGCTGTTGGGGGGTACCTCGAGAACACCGCTGCAGTACAGAGGATCCGACGTCAAGGCGTCTTGGAGTTGCTGCATCAGGGGGAAGGCAGTGTATTCGGTCATGGGGAGGGCTTGTGGCAAAGAAGGAAGGGAGGAGGGCCACTTGATAACTCGTCGCGCCTCATGTGCTTCAAAACATGGATCCACACGAACAAAGTGTCCCCTATCGATCGGCCTTCATCGAACACGTAACACGTACCTCAATCTCTGTGAAGGATTTCGAGTCAGGTTCGCATAGGTTCCGATTACATGGGATGCAAGGAAAGAGTTAC

>AT19416

AGGCCGGAAACAAGCACGACCGCACTCAAGGGGGGCGAGTAGTAGTCGAAAACGGGGATATAAATTTGCCTTGCGCCGATGGACCTCGAAGCCGCGGCGATCTTGCTGTATAGCGGAACCTTTCGAACCACGTCACCGCTTCACAACACTTAGCTCAGAAATTCTCTGAAGAACGAAGACGATGATAACTTACTTTGTGCGCTGTCGCAGCTGGAAGATAGCGTTGACGAGCCCGAAAACGAAGATCGCCGCGCAGAAGAAGTACACAGTCGTCCTCCCGTAATCCCAGTCCGCCCTGTACCACTT

>AT19444

CGCCCTCTTTCGCTCGTCATCCTTCACATTACATGCAGTGGCAACATATTTTGAGTACGCATTTGCATCGCATAGTCAAGAGGGGCATGAAATAGCGATGGCATCTTTATCTCAATGGAGCACCTGCGCTTCGTGGATCAGACTCTCCGCCTGTGCAGACCTCGGCTTGATAGTGCACCGACAATCCGCAGGATACCATAGCAAGCCGTCCGTCATGTGCGACCCGACCTTGATGGTCTGGCCCTTCTCGTCTACTGGAGGAGTGATGTTAAAGACATGCAGGGCGGACGAGACATTGATGAACAATCCGTTCTCCGCGAAATATCGCCCAGGGCAAATCCTGTATGTGTGCCATGACATGGTCGAGG

>AT19488

ACCATCGCTGCTTTTACCCGACGGTAGGGCTCCTCCTTCAAACGCATCACGGTGGCCCTCCACTTCACGATCAGCCGTTGCACTGTGGCGCCGGGAAACCACGTGGGGACATGCTGAAGGATGGGAAGGACGTCAATGAGATACTTCCCCGGCGTGAACGTCTCGCCGATCCATTCGAGCGCGTCTTCAACGATGGCCATGATCTCGTCCCCGTCATCCGCGACGTCAATGCCGAAGACTACCTTCACAACCGCTGCGGAGAACGTGCTGCAACAGGTGCTTTCAGCACGCTAAACTCGCCCGCAGTATGACATTGACACATACAAGCGGATATGCTGGCGCAACTTCGAAGGGGTCTTCAAGAGCTTGTTCAGGAACCTGTGTGCTGCGCTTCGTTGTATCGGCTGATAGGACGAGACGGCTCCGGGGTGAAAATACTGCCAGAAGACGCGTTTGTGCCGCCTCCACCACTGCCCGTACTGCATAACTGAGATGTTGACGCGCATCTCAGTCCTGGGGAGATAATTAATACTAATCTAAGTTGAAGGGAGCGTGGAAGACCGACAGCTCTATTAACGGCACCCGTTGGCGGTCCGAAGTGTTGGCGGAACGCTTCTCAAGCAAGTCGAACATCGCTTCCGAACTTCCGAGCACTACCATGTGCTGACCAAGGACTTTGAAATGGAGAATATCCCCTAATGACCGCTATCGATTCATACATATAACATAGCGGTTGGGTTGTAGGTGCTCACCGTACTGCGCGCTCAGGTCTCGAAGTGCACGCCATTGGTTGCGGGTGGGCCAGTCGAGCAAATTGCCGACGACGGGGAGAGGCTTGGGTCCCGGTGGGAGGGGAAGACGACGGGAACGGGTCTGCCATGCTGCGAGGGAACGCGAGTAGAGGAATACTGACGCGACCAAGAGGATACCTACGAGGGCTGGGAGAGGGGAGGTGATGTCCATGATAGGCCTTGACGGGGTCGACACCGGTCATGCGGGGCTTTTATCGTCCAAAGGTCCTGGAGATAGACGCATGATCAAGATGCCAAGCTTCTGGGTGAAGGTATATGATCGATTGGAAATGCCAACAGCAAGCAGCGATATGGTCGCTAAACATGGGACGCGGGACAGCTCCACGGAAGCTTTACACCGACAGCATGGGCCCAGACGATGATTGGCTTAGAGATGCTAAGTTTGGAGCTTTCGAAACCTTGAAAGGGTACAGTTGTCCTTTTGGGGTGCATGGGATTATCAATGAGATGCCTCGGCAGCGAAGATTGGGTGGCGTAGAACGGTGCCCTTGACC

>AT19548

TTTGCACTGAAGTAAATTTGTATTCTTCTAATACGTTCGCAGAAAGACATAATAATGTATCGCTACAGGTACCGTTCCGCCCGTATCCAACCCGCGATGCAGCATCTCCTACCCGACTCTCGGCCTGGCCCGCCACTAAGTTTACAAAACTGTAACCATCCTGTAAAGCCTTTCAGATGTCAACACAATTCAGCAGGAGGGTCGCGGATAAGCAAAACTCACCTGCTATCCCAAGGGTTGCTCCAAACAGTTGACCTTGCCCCTGCATGCGCGTGAAGAGGATATGGTAGAACCGCAACCGGCATGAGCGGAAATAGTTTGAGAGACATACCCGGTACGTATACGTGAAGTGCCGATATGGACGCGGAGAGGAACCCATCGTCTCACCACCAGGCATCCCACTTCCGTCCTCCACTGACCCTCGTCCCTCGTCCATCTGTTCTGGGAAGGAGTACTTTGGTGGTAACCTCAGCAAACACCACGTACCATGTAAGCACAACTCCTAGGCTGCGAGACCACGAGAGTGGAGACAGCGACAAAGCAACGAGGAGGCTGGAGCTCGGAGAGTGGAGCGCAAGGTGTTTGCCATGGTGGACGTTCAGGTTTGAGTTGCGGGCCCGTACGTAGAGCGACGAAATATGTATAAGAGGAATGGGCGGTGTCTAGAGCCGGGAGATGAGGCAAGACGGTGGAAGGCAGGCTGGACTAGGTTGGATTGGACGGAAAGGACGTGGTGAAACCGCTCGGTTTGCGTGGAACGTCCCATGTGAATCCACCGCGGCCATCACGTGCTTATCCAAGGCCGGATTAGATGTGCTATCTGTGTCTTGTCCAGCCTAGATTAGACACGTTTACACCGAGTAATCCCACCTAAACCATGGTCCGTCTCACCGCACCTAAACATCTCTCGTACTAGGCAGGCATGAAGGCCTTTCCGGCGCTGCTTAGGTCGGATTAATGCGGCAACAGGTCGGTTTAGATGGGATGGCTCGCCTTGCTCTGTCTAATCCACACCGGACAACATACCAGGCAAACGCAGTGATTATGATGTAGTCCTCGGATCTATACGCTGAAAGTAGCGGAATGAGGTGAGGAGGCCGAGATGAGACCGCTTGGAAAAAAGAATCGCTGCAATGAGGGTTGGGCCCTTGAGCTTGAACCACCAGTGAGACGAAGTTGCTTGAAGTTGAACTTGCTGGGTCGTCCCAGTGGCTGCAGCTGGCACTGCA

>AT19556

TCCTTGAGTAGGATCTGCGTCGACTGCTGAGACGTCACAGGTTCGACTGCGTTGGCACCGTCGTAGCGCAGGATTGCAGAGTTTATACCATCTGCGAACCCGAGATTACCAAGGTTTGGGTTGGCCCGAATCCAGTAGTTGTCGATAGTCTGGTTTGCGGTGAGCTGCAGGAATGGTTCTCCTCCAGCTCCAATTTCGCAATACGTATGTACGGAGAAACTCACCACAAACGAATAACGCTGTGCGGCGAAGATCTCGATGGCATTGACGGTGACGGGGTAAGTCTCGACACCATCGGCCTCAATGACGGTCAGCTCATGGCCGTCGATGCTAAAGGTATAACTCGGGTCGCATGACAGCGACACTAGGCGGAAGCGGTAGCTTTTCATCTGTGTTCAACATCTTCACATAAGTGGACAGCTGACCGCGTACGTACCGCTTGCCGTGTGTAACGTTGACAACGGCGAGGTCTGCGCCGGGCGCGTCGGGGC

>AT19600

TATGGCTCTACAGGCCACCATCAGATCCTCCCTACTGACTGCAGGGTCTTATCGGGCGTCGCCGGAGCCTACGAACTCCTGCATCCCCCTGTAGAGCTGCGTAGCGCTCTGCATCGCCTTCCAGTAGGGATGCGCTTTTGCTGATGAGGGAGAAGGGCCAATTTACTGGATCACCTGGTGGCCCCATTCCGTGCATCTTCAACAGCCGATACTTACCACGTACACGCATGCACTTTGCTCCTTCTAGGCCGTGATCGGCCTTACCATCTCGATGTATGAGTTTCCCCCAAGACCGGGTCCTAATTCGAAGCGCCTGGACCCGCCCAAGTCCTCGAATGGCCGGACTACGGCTGTGGAGTTACGGGACAGGTGAGTCCCCTTATGTAAATCACTCAGTTTTCTCGGCAGCCTGATGTTTGGCATCTCCATGATTCTACTTTGTCTTGTTCTTTATCAACATACACTGTGAAAGTCGTCGCCGACTCATTGACTTACTTATCTCCCCTTTATCACTGATCCACATGTGCATATGCATACCCGGATTATCACACGATTCGGCCCAAAGATGAGCTGGAACATTTCCCCAGGATGCGGCCGCTCTCTGCAATTCGATCATTGGCGCATAATGCTGTACGTATCCTTACGCTTGCCAATTGCGGCGTTGCTCAGGGCCCATGCCGGACGCCTTCAAGGACCAATGGGACGGCTGCCCTACTGGCATTGCATTGACCGGGCCTAGATAGCCCGAATCAACTCGTGGCAAGCGTCGGGTGCGTGCCTTCTGTCCTACGTACGCAGGACCGTTAGTGCGGGCTCCTCGTTGTTGCCTTCGAACCTAGAGCAGCTTCATCCCATTTCACGGTATCACAAGGTAGCGTGCCCGCCCCGGTCATGAAGAACGTCTAACAACCAACTAACAACGCACCCGCTCCACACCTCATTAACGCCCC

>AT19643

TCGCCGCCAGGAAACGGACGCTGCGCAGGGGTCAGGCAGGCGGGAGAGGTGAATCTGTGGACAAGATGGGAGGGCAATCGTGACTAACCTCCACTGACGCGAAGCTTCAAGCTTGAAGGCGAGGGGGGAGGGCAAGACCGAGCTGAAAAGATGGATGGCGACGATGGCCGCAGAAGCCAGCGAGGTATAACGGAACCAGACCTACCACAAGAAACAATACATGCATGACAGCATTGCAATGCGCTTCCCCCCGGCGAATGAATGAAAGCAACAACATTGACGTACCTGCGAACCTCACAAACACAAACCCTCACGTGCACCGTGAACATCTGCCTGGTGGAGCATCAAAGCGCATATAGTCTGTAACAGTATTAAATGCGGAGAGAAGGACGCATACAATGACTTACCGGCAGCTGTAGAGGACCGCGCTATGGGGGGCCACAAGGGGAAGCTTGGGGATGTGCGTGCTGGAGGGAGATGGCTGACCGCGCCGCTCATCTGGTGAGGGCGGTGCCCGGATGGCTTGAGGCGAAGTAGGGTTATGGTGGGCGAGCGAGTCCAAGTGTTGAAGTCAACAGCGAAGAGGGCGAGCAGTCTTTTATCAGATGGGAAGCCGAATGACAGCTCATGAACCAGGACTCGGAGCCCGTGATAACGCACGGACCCCTCACAGAAGCGCACGGACCTCGTCCGATTGTAGCAGATAGCCCACTTGATAATCCTCGTATCCTTCTCCTCTTCGTGTTGCGCGGCCTACGGAGCCCAGACAGATACAAGAACAAATGAAACCATCACTCACCGCAGTGTGCGTGCCCGACTCGGAGAGGGTTTGATCTGGAGGCGCGCGGATGCTCCTCCTATTGATATGAGGCTATATATATGAGCATGAGAGCGTACTTTGGAGGGAACGACCAGGGACGGAGCACCCGCAGTGGTGGTACTAGTGGTGAACGAGAGACGAACGAAGGTCGCACTGAGCGCTGAAGCGCTGAGCGCTGAGGCCTGAGGACTAAAGCGCCGTGACCCTGGGATACGAACTAATTCCAAGGTAATTGACTAACAAATCCTTACCGAAGTTATACTCATACAATACGAATAAATACCGTAGCCTTGATGTGGCGATTACAACCTCG

>AT19644

TTCCCGCTGACCTTCATCATCATCATCATCATCATCATCCTCATCATCATCACCGCTGCTGACGGTTCGAGGGGTCGTGTGTCGCGCCCGCGAGCCTCAGCACGGCTTGGCGACGCCGCCGCCGAGGCACATGTTGCTGACCGTCCACATCGCGCGTCCGCCGCCCGCGTCCTGGCCGTTGACGTCGGAGTGGAACGCCATGTTGACGTACTGGTCGAGCACGTTGATCGCCGTCGCGCCCCCGGGGCCCGCGGTGCCGTACATCCCGCT

>AT19846

AACTTGGGGCGTACGGATGTATTCCACGAGCTCGGGCGAGAGCGCGCCGGCCGGAGCCAAACCGCGGTACGCGACCTGGCCGGTGAACACGGGCTCCACCATCCCGCGGAGCCGTGCAGCTTCCTCGGCTTCACTACGCTCCTCCGCTTCTTGTGCAAAACCGTCGTACATCGTGCGGCGCACGGCGGAGCGGATGCCGTCGCTGCCGACGACGAAGTCGCAGGCCGCCGTCGTGCCGTCCCCGAAGTTGAGCGTGATGGGGTCTGTGGGGGAACGAGGCTCCGAGTACGACGTGAGACGTTTCGCGAAGTGGATTCTGTCGCCCGCATCGAGGTGCTTGGCAAGGAGTTTCTGGAGAACGGACCTGTGGAACGCGTG

>AT19847

TTCCGTGCCTTGGATCGTAAGGAGATCGTCTTCGAGTCCGAGGTCCTTGACGATGGACCAGATGCGTGGCGACATACCGATCCCAGCGCCGACCTCGGTGAGCTGGGGCGCCGACTCGTAGATATCGAAGCGGACATCCGGCGCATACTTCTTGAGGGCCAGGGCGAGGACGAGGCCGCCCATGCCGCCGCCACTGACCGATTGGATGAGAGCTGTCAGTTTTCCGACTGGGCCCATTCGGAGTCGGACGCGACCTCACACGATAGCCACGCGCAACTTCAACGCCGGTTGGTTTTCCGCGAGCTGGGACATGTTCGGTACGGGTTGTGGTGGCGGCGGCAGTGATAGTCGTACATCAGTCGCAAGATTCGC

>AT19968

ACCGCGTACCACGGCATACAATTGGACCATTGGAGACTTCTTGGAGGGCAGGGGGATAGGAGACGAGGATGATAGGACTTTCGAGACGATGTGGTGGCCCATCTCTCTATACCAGACAAAGGCACGATCGCGTACGATAGACGTAGAACTGAGGGCTGCGGTGGAACTGAGGCCGAGGTTGGACGCAACGTCGTCCAATGTTCCACAGCGTTCGTCAAATGCCTGATTCTGTGGGTTAGAGGTTAATTAGCAAAGAAGCCATCCACTCCAGCTTCTAGGTACGTACCGGATCCGTTATGTATCGGAGTTGTATCGGTACTTCTTGAGGGGATAGGACCGTCTGCCTCGAAATGTGGAACACCGTCTCAAGGTACGCGCGGACGGCTCCGGTGATTCCGTCAACAGTGATTTCTTCGTCGCAGGGAAACTTGAGCTTGACGTAATGAGGTTCGATATTGAGGAGAACAGGAATCCGCAACCGTTTACCCATCGTGAACGCAGGGATGGATCATCGACGGCGGATGGGATGGATGGTGAGGGTTGGGGTCACAGAGCGAGGTGTACGGACGAATGATGAAAT

>AT20129

CTCAGCTGGGACCCTACGGACACGACTCGGCCCCTCGCGTACTCGTACAGATGGCCGTACCGGGTCGCTCGCTCGCCTCGTATGCTATCATACACAGGCCGGACCGGATCTCTTCGTGCGCGTGTCTCCCTACGTCGGGAGCTCAGAAGCTGTGGGCTGGGTGAGCCCGCGGGGCCTGTCCGTACCCGACTCCGCTGTCGAGGGCACGGGTCAGGGACTGGATGGCGGTCTTGATGACCACGCGATCCTTGCCCGTCCAGTGCTTGGGCGGCCGAGCG

>AT20304

ACACCGCTCGATCGCGTTTATTCGGTACAATAAAACAGCTCTATTGGGATCCAAAAATAGCTACACCAGCTGGGCGTACGCTACTCGCGGGCCCAGTCAACAGCTTGCGCGCGCGGGACTGTACGGGCTCGCGCCCGACCCACCCGCCACGCGCACCTGCGCGGACACCCATATGTCGCGCGCGCGTTCCCGCTCGTCCTGCCCATTCAATCCGCGCGCTCCCTCCCACCGCGCGCTGCTCTGATTGAGCCGCGCGCACCCGTCGCGTGCGGTCCTAACAATGACGCCCATTAGCTGACGTCATAGGGTGTGCGTGTGTATCTTCACATACCTGTGCTGTGCGCGAGGCGCGCGCCCGAGAGAACAAAGGGAGGGAGGAGGCAGAGGGTAGCGGAGGTCGCACAGTGGGGAGATGGTGGGGGGAGGAAAGAAGGTGGGGGACAAAGGACGACCATGTTGGCCCC

>AT20326

GGGGCGGGGAGCTGGACGCTCGCGGGGACGGGGATGACCTCGGAGCAGTCGGTGAGCTCGGCGGGGTTCTGGCCGAGGGTCGCAAGCTTGGCCATGACCGCCTCGAACTTCGTGACCATCGAGTCGTGGTCGACTGGGGTTATGCAATGGGTTAATTTAGGAGAGAGACCAGATACGAGCTGCGACTGACGGATGAACGACTGCCACTCGCAGGCGGTGCGCGGGTCGCGGGCGAGCTCGTTATCGGAGAAGAGACGGAACTCGCCAAGGAGAGGCGACTCGACCTCTCCGACGTTGGAACCGTTGCCGGGGAAGAGGGTCCCTTGCAAAAGT

>AT20623

GCTGTGTGTAAATTGGCAGGGTTAAATGAGAGGCAGTACAACGGGCCCGGCCGGGTTAGCTAGGTATACCGTGACTGGCAGCCTGAGTACAGTATATTGGTAATTGAAGCGGACGTGTTCGTGTGGCGCCCTCAGGACGGAGCTCAATCTGATATTGTACAGAGGACAAGGAGCATGATGAACCACTTTAAGAGATAAGTATGAACGAGTAAACGAATGCGGACGCCGGTGGAGAACTTGGCTACTATCTATACTCTGTAGGAAATGCTTCCTTACGCTCTATTCTCCAAAGCCTTCTACGGGTGCTTCTTAGTTATTATGGGAATCATTTGGTGCGGTACGAAAACTGGGAGTCATCTTTCTCCAAGAGTCCCGCGGACGCCAAGTTCTGACTTCGGCATGAAGTGCCTCGTGGCGACCATAAGGGCCGCCGACAACACCAGGAGCGAACCTAGGTTCCGAACAATACTCTCAATTCTCGTGTACGAGAAACTGCAGGGAAACGCTCACCTCCGCAATATCCCACATTCTTGTACGAGCCAGTAACATCGAAGATCGCGCCCAAGAGCGGCGTACCGGCGACTACCCCGACTGTTACGAGCGTGAAACCCATCCCGATGCGCAAGCCCACTTCCGCAAGGCGGCCCATGAGCTCAAAAGGCTGGACCATGGACGCATAGTAACCCCCGGTCCCAATTCTGCACAACAGGGTTGGCATAGTCCGGAACAGAAGTTGTCAACATCAAGAAGTTGGACGGGCAAATCGGCAACGCACCCAGAGAGTACAGCGACCACGACGAAAGTGGCGACGTTTGTCGCGAATGGCCACGCGAAAGTTACGACTGCTATCACTAGTGTTGAGCAGGTGAGGACGTTGAGCGGACCTGCGAATGCGGGGATGGTTTAGGTTTATGTAGGCCGGGGCTGCAAAGGTACTTGAACCATACCGTAGCGATCAGCAAGCAGCCCGCCAACCAATTGACCCAGAGCAGACATCGCGTTGACGATGGCCACTAAGTCATAGGATAATGTGATATCGATGCCTGCCCCGACCGCAGTGACGGTGATGTAAGTAATAACCTACGGAAGATTGAGAGTGAGCTGGTGAGTACTGGCCCGCTGATAGCGGAGATACCAACAGCACTCAGCCCAAGATTATTGATGATCAGCGAGACAACATAGATCGAGTACACGGGCTTCTTGAATTCTGAGACGTTGATTAATGGGCCTAGGTCTTGCCGGCGGGTTCCGGGGAGTCGCCTGAAAGTTGTCTGACACGATGGAGTCAATTCGGACTTCGGACTTAACAAATGCTGCGCGGCGTGATTAACGAACAAGGTTTGCCGGG

>AT20624

GTACCTCGTATGCTGGAGTAGGGACTTCATGACGACAGGGAAGATGCATCCACAGACGCTTACCCCTGTGTATGCAATGGCAAGTGCCAAACCCAGCCTCCGCTTGAACCAGTAGGTGAGGACCATGCTTCCTATGTTGTAGTAAACTCCGCTCGCAATCTGTTGGTAAACCTAACATCATGTCCTGAAACTGGATGATAGTGAACTGGGGACTGACGCCGATACCAAGGCCTTGGCAGAGTAGGAACTGCCAATATTGCTTGCATTCCGCCGTGAGGAACATGCAAACAACGAAGAGAGCTGATGAAGCGAAGATGGGAAGCCTGAGGTATCCCAAGTCGAACAGTCTCCCGACAACCAACCCAGGCGCATAACCCATCGCGCTCTAACGTAAATTAGC

>AT20625

CTGGTACTACCACTTCGTCGACGGATATTATCACCGTCTCTCCCAGTGCCTTCTGAGAGCTAGAGTGGGGTGCAGCTGGTTTCTCATTGCTCGCCGTGGCCACGGGAAAGGAGCGTTCAGTGGCGAGCATGATAACACCTGCACGAGGGAAGGGGGAACCGTTGTCGGTTTTCACCTTGGGAGTCTGTTTATATTCTATCAGAGTGACATTATGGAAGCGGACAAGTGTTATATAGCGGAGCCGTGGTATCTGGGCGCAGTTTTGGAATGGGTTTCTCGAGAAATGTATATGGGTGCTGCATGTCCTGAAGGAATCTCACAGTGAGTGCGAGACTGAAGTTCGCGTGCTGCC

>AT20626

TCAAAAACGAACAGCCTCGTTAGGGTAACCTCCTCGTCAGCCACGTCGAGATGTAGATAACGCTGTAATCGCCCGAAGTTCGAACCACGATCACCGTCCCAAAATTCATCGATGGCCATTCAAGTTTCCGGCTCGGCTTAGCAGGTAGACAGGTTTAGACGGCGCGATCTCCTGCCAGTCTTCTGGGCAACGCGCTCTCGAATCGCGTACTGCCTGGACCATATAAGTACCATATAACCTCTCACCGCCACCATGAACGCTGCTGATAACAAGAGGACAGTGCCTACTCCCGGCCGCTTATTCAGTAGCTTCGAGGCCCCGAGGACGCGCAAAAGGGACGCACCTCCGTAATAACCAACATTCTTGAAGGACCTCGTGCTGTCGACGATTGCGCCTGATATTGGCGGGCCTGCGATCACCCGGGCGGTACGAGCGTGAGCCCAACCCGATGCGCAACCCTACTAAGGGACCCTAGTGACCGGGGCCCATGTCCGCGAACGGCAGCGGCCTCGTAAGGAATGCCAAAATGCGC

>AT20687

GTTCCGAACCTAAGCTACTTATACTGAGTCCCATCTGACGGCACTATCTACACTGGACCATTGTACGAGCATGGGCATCTATACCGTGACATACTAACCCAGTGGGCACAATTGCATTGCACAATTTAGACGTGGCGGTGACAGCGGCGCGGTCCAACAAGCCGCTCGCATCTGCCCAACTACATCCCCACCGCCTTCCCCTCTTCGATGATCACGGAGCTGTGCGCCGTGCGAACGCCGCCATCGCCCTCGATCTCGGTCGCGACCTTGATGTCAATTCTCGTCGGCACGCGCGCCGGGAGCTTGTTGTGCCCAGGAAGATAATGGCAGCGACGCGTGTACAAATGTCAGTGCTCACGCCCGAAACCTCGGAAAACAAACCTGGAGTAGGTGCGTACCTGGGGCGCATTGTACCGCTCGGCAGTGGCGAGGTGTTGCGCCCGCGCGATAAAATTGCGCTCGATGGTAGAGTCCATCCCGAAGATCTCCATGCCGCCATTCGCAGGGAGGCGTCTCGAGTTGA

>AT20699

TTCGACCCCCCAGGACCAGTCCTCGGACATACGTTCTCAATCCTCGCCAGGAAACATTGCAAATACGGTGTACAAATGCCGATGTGTTCAGCAAGGTTCTCGACGACTGGAGTCCTACTAACTACTACGCAGCGATACCGATGGCCTCGTACTGACAGGTACCTCCACGTTCTCGCAATACTTCAACATAGCAGCAACTGCACTCCTCACACGTCTATACGCCCCAATCACTACTTCCTCAACATGGTTCCCTTCACGCGCCGCAGAAAATGACGGTCAGGTTGACCTAGGAACGGAGAACGTCAATGTCAGGCCACACGCGTGCGACGGGTGTTCTCGGCGCATGACGACACTGTCACTTACGTTGTCCACCGGGCACCCCACCGGCTCCCTTGAGGCACGGCAGCCCGGGCCACCGCAGTTGGCCCCCTGCCCATCGCAGCCGCCGGAGTACCTACGCGAAACCAGCAATTCGCTCTACTGAGCGAGTGCATGGCGAGTCAAACTTGAATCTGCACTGACCTGAAGCCCGTGGGGACGCTGAACACGTGCGGGGGAATCAAGCTAATGTCCGTCAGGGAGCCGACGTTGGTGGGGGTCAGGTTGATGAGCGCCGTCTGGACGATCGTACAGTGTTCGCCGTTGAAGCCGCATCGCTTGTCGTCTGGTCACGGGGGGGGGTGTCAGCCTGCCAATACTGGAGGCACTTCGACTGTCATCCATGCGGGCGCACCTGGCTGCAGTTACGCAATGGTGTGGGTGAGGGGACCGTCGGAGACGTAGTCCTTGGTGCCGTTTGAGAGAATCTTATCGTCCCTGATGAGGTAGGGCTAGACTGGAGTAAGTACGAAGGCGGGTTTGAACTAACGCCTTGCGACTTACGGTCCCTTCCTTGCAGCTAAACAAAATAACGAGTCAGTCATTCGGAGGTCGCTAAGGGCGGACAACCATGCTTACTTGTTGATCAAGGTAACGGTGTGGGTCTCGCCGATGACGGGGCTGGCGAGAGCCGCAAGGGCAACAATAGCGACGGACAGGGAGAGCTGCATAGTGTGATGGGACGAAGAAACTTCAACTTCGCAGAGGTCAATGCAGGAGAGGAGGCGATGAGCAAGCTCGCTGTCCTTAGAGCTGTAGCGTACCGTCTATTTAACCTTTCACCCATCCCACCACAAAGGGAAAAAATCCATGCCTAGCGAGACGGTGGCGCACCTTTCAGCACCATGAGGCATCGCACGTGTGCAATGATTATTCTTGTGTCGTCCAGGATAGACTAGCGATGCCTTGATACACATGGACCAATTTTTGGATTCGAGGAAGGCGTCCCGAAAATTGGACTGTAAGCGGCGTTGTTCGCGATTTTATGCCTGGAGGGAAGACCTGGTCGGCATGGAATTTCGTCAATTCTAGAAAATCCTTTACCGAACCGCACCTGTAGCCCTCGACGATATTTGATCGGCGGAATGTGACCGCTCTTTTACCCAGCGCCGCACGTCCACGGGTCGCGGCCACTTCAAGTTCTCTGAGCGATTGCCCTACGGCCGGAGGGACGACCGCTCCAGTACACCTGTTATAATCCGCGGCGGTGACAGTCTATGCATTTGCTGGCGTGAGTTGTGTCTACTATGCACTACATGCGCAGAAGCACTGACCATTGCCCGTGTCCGAATAGGATTGGAAAACGGCTTGGAACCGGGCTTCGTCTTGATCGTTGAACGTTGACGGCTCATTTAATGTGACGTGTGTTGTAGCTGACAGTGCCAGCAAGAAGCAGCCCCCAGAGAGCCCATCGCCGCGAATCTTCGCCGGCCAGCATCACAAGAATCATCTCCAAGCAGGTTTGAGGATCGAAAGGAGAAAACCTATAAGCTGTGGTGGCGGGGAGGCGGTATGTCCTGAACAAGGAGGATTCGCGAGTCGCTGGCCTTCCAATGACGGAGTTTGCCGAGGTCTTCCGAATGCTTAGACGCTCGCTGTATGAGTACCCGAATTCGCCCGAGGCCGATTCGGAGCTTGGCCCCGGGCCCCCGCTCACTCCTTTCCACTGTTTTCCCCAAGTTCTCCATCCTGTAGACAGCTGCAG

>AT21420

TATGGCCTATGGCTTTCCAACAAGCCCTTTACCATGCCAATCGGTGGACGTCCGGCCCAGATATGGGGGAACAAAGTGGGATGTGGCTGAGTATTGATGTGTTAGCCATTCTATGGCATTGAGGGCGCATGCCATGCGAATGATGCGCCCTGAATCTCCTAATTGGAGGCTATACATCAGTAAAAGAAGCATCAGAATTGGTCAACTCGTTCTGGAGATATCTATACTAAGCCTTCCTCGAGGAGGAGGTATGCATATCTCGGTGGCCACTGGACGGATTCAAGCACAAAAGGGTGCGTTCGATGCGCATTTCGCACGCGAATAGAACGCGTCCTTTGGCATATAAATCCGTCCAGAATCCACTGAGATATTAGCCAAACAGTGACCCCTAGGGCGAACCTAGGGGGCATTTTCAAACTGTTAGCACATACCTTTAGGGTGGCGCATTAGGCGCATTAGATTCGCACTTGAAATGCGAACAGAACGCCGCAACAATCAAGAGAATCGACCTAGAAATGTGTGAGTAATGAGCAAAACAAGGTGGGCCGCCCCCGGGCAGGCGGGAGGCTTCCAAATCAGGCCAGTACGCCTCGGAAGCTTCATATTTGATGGCAGGGGGCAGCCTGAGAACCCAGAACTGATGGAAGGCAGGCCGGGAGGTCGGGAATTGATGGGGAGGAGCCGAGAGGGAGTAAGCGAGGACAGAGTAAGC

>AT21472

TGTTTCTGCGACGGTTGTCGGTTCTTGTGCGAGGACGCATAGGGCCAGTGCAGTCAATCCATGTGGGATGCATTGACTGAGGCGCAGGAGGTGGTCCCGTGTCAGTCCGGAACTATCCCAATCACCACGGCCTGAGAGCGCATAGCCCAGGGCGCGGAGGCGGGGAAGCTCTTGGAGGATTTCAAGAACTGGTTGAATATCATCAAAGCGGGGCGAGTACCAATTGTAGTAGCCGATGTCCAACCGTTCGAGCAGCGGCTTCTTCTCCAGAAAGGTAGCAATTGCCTCCACATGATCCCTCTCCATGTTCGCGTAGTAGGAAAGCCTGAACACAGTAAGGAGCGGGAGTGCGTTGGAGTGCGTGCGAAAGATGGAGGCAAGAATCACAAGATTGTCGGAGTCCGGCGGGTAGGCACGTACGACCAGCGTGAGCGACCGTAACCGAGAGAAATGCGTGAAGGTTGTCCCGAGCGAATCAATGTCGGTTAGGAGAACGAGGGTGAGTTCCACGAGGGCAGAAAAGCGCTGAAGGCTGCATTTCCAGAGCACCCCGCTGAACGTGCCTAGGGAGCGCAGCGATGATGAAGCCGCCGT

>AT215

TCCTGATAAACATGCCGGATGTTTTAGTGGAAAGGCGGTGAATGCAGTCCCACACTTTGTCTCGTGCCTAGACGGCGAACCGTCTACACACTCCTTTGCAAGAGGTGGAGGTGTAAGTATTTGGCCGCGCCCACTTGCAACATGGCGCTGGCAAGATCCTCAAAGAGACGGCGAGGAGGCAGGAGAGAGCAAAAGCGAGGGCAAGCGAGGAGGCAACATGAAGGCACTTGCATCAGCTGTACACATCCTGATGCAAGCGGC

>AT220

TGCCGGCGTGACGAAGTACTGTACAGGGCGTCTCCAAACGTGTATTACGGATCGATCATGACGCGCTCACAAAATATTCCGGCGCAAGGAGCATACTGGCTGGTGCGCGTGCGCAGCGAGCCAACCAACGCCCTTCAGCTATATCTTGAGTGACAGGGCCCTACTGCCGCACTTACCGTCTCCGTGGGCGGACGCAGAATCCGCGTATACGTATAGTCCGAGAAACGAGAACTTACTTGCACACGAAGAGCTTGGGGGACTTCAACCATCCACAGGAAGTGTAAGCATGGATGGTCGCATACATTCAACGAACGGACACCTACGAGACAAGGGTGGCTGCACATCCCAACATGTGAGTCCATCCGGAGGTCGTCTCATCGTTGCGCGGGAAGCATGTCCTCGTCTTCGTCCTCGCCCTCCTCTACCTCTCCGTTCCCGCCTTCGATTGCGTAGGCCTCGTCGCTTGCACCGGGTTGGGACTCGTTCCGCCGGCACTGTGAGAGTGCGGAAAGGTCAGTTGACCAACTGATTTGCGAGGTGTAAAGAACCAACACGCACATGGTAGACAGCCGTCCAGTATCCGCTCCAATACATGGCGGTCATGGCGCGAGAAAACGCTTCGTCCTGGCTTACCATGGCAGCTTCTACGCCACTACCCAAAACGCCGTCCATCATCGCGCCAGTATCTGCACCAGTTGCTGCGGCAGCAGCGAGGGATGGATCGTGTGTGGGCACAAATGTGTCGAAGTTGATAGGAACCGAGTCGGCCAACCCAGTCTCCAGGGTGACTTCCTCGAAGCTGGTGCCATTCGACTTGCTGGACGATGCCTTCGACACTCCTTTTGACGTCTTCGTCTTCTCCGGGGGAATGTTGTACCAACTA

>AT221

ATACCTCATACTCAGCAGCAGCGCTATTCCAAGCATCGATCAACGCCGAATCATCCCAAATCTCGTCGTGAGTAAGTTCCCTGCTCTCCTCCCCCTCTTCATAGTAGTCCTCTTCCTCGGCCGCCACGTCCTTCGTCTCTGCTGGTTTGGGCGCGTCGTCGTAGCTCATCGGGGTCCCCTGATTACCTGGGTCGTCCCAATGCTGCGTGCGATGTCCACTTTGCATGTTCCTTGAGATTCCGTGTCGATGATTGGGGCCCGTTTTGCGCTTCTTAGCCGGTGGGTGTGTCGCTGATGAATTTGGCTGGCGTTCTGGAGGTACTGGAGGCTCGGCGGCTTGTGGGATCGTTATGTCGTCGTATGATATGATAGGGCGCATTAGACAAAGAAAGTGACCAGACAATGGGTGAGAGAAATAGAGAACGGCAGACGAGGATGCACTGCTAGCGAAAGTGTGGAAGCAAGACGCGTTAGAAGCGCGTTGGCGCCCA

>AT22760

CGGGCACAGGACAACCCCCGACCGGCCCACACCGTGCGCGCATGCTTGCAGCCAAGCTGAGCATAAGACGACAATTTAAGGACCTCAAATATCGATGTCCGGGCCTTCAAGTTGGGACATCATGTCCCAACTTGAAGGCCTTAATTGTCTGTGCTATTCGTGAACTTGGTGGTCGCGAGGCCGACCTGGCCGATCATCCCGTTGTACGCGAAGTAGAAGCGCTCGAGCCACGCGTAGCCGTTGACGAAGTCGAGGCCCGAGCCGCTCTGCGAGCCGATGTCCGCGACGACGAGGTAG

>AT22774

GCCCGGCAGTGGGTCACTGACTCAAGCGGCGAACTCAACGGTCCATTACTAGTTACTACTAGCGCGGTCCGATGCTTTCTTTCTATAGTATGAGTGGGGTGTAATATATGAAGCACAATCATACAGAGAACTCGCGGCGAATCGAGAACTGTACGTCTAGTGGACTGGCAGCGACAACGAAACGCGCGTGTCTTCAAGGAGTTCACAGGCTGTCCAAGAATGCCGCAGCCTCCTTCCTCGACGTGCCGCTCTCTTCCCATTCACCATTGACAGCCTTCTTCAGTACCTCAAGTCTCGCACGCTTCTTCGCGCCATCCTCGCCAAATGCCTTCTGCAGCACCTCCCGAACCTCCGCCTTGATCGCCTCGATAGTGCCTTTCGGTGTGTACCCGGTGCGGTAAATGGGCTTGAGACCGTCCCCAGTCCGAACCTCGAGCAGCTCGTAGCCGACCTGGAGCTGTTCAGCGATGTGCACCGCGTTGAGCGGTTGGTCGCCGCCGAACGGCCA

>AT22775

CGGGGTTAGTGGACGGCCAAAAGATAGAGCCGAAGGAAATCTAAACAAGGATCGGCTAGACGTGAGGTGCCGAAGGGGCTACAATTTGGATGAGGAAGAGCGTACGTAGACCAAGGATTTATCTCCGGAAGCTTTCAGCGTCGCATCCAGGAACTCTTGGATCTGGGCTGACTCGGTGGACTGCTTCTTCTCGTTTGCCGCTGCAGTCTCCTTCGAAGCGGACGGAAGAAGCGGTCCGACTGCGTACGCTGGACGGCCCGTCTCGGCGAACCAAGCCTTAGCAGCGGCGACTGCCTCGGGCTCGTAGGAGGTCGGAGTGATCATCATAAGTCCATCAGAAGCTTGCAAGGTTCTGTAAAGAAGATCAGTGTCAAGTCAATGGCCG

>AT22841

GGCGTGGAAGTGGCCGGCTTCAGACAAATCTGCGATAGCGCAGTACGAGTGCTTACCGTTGGTAGCCGGCGAACGACAGGTGAATCACTTGGCCACGGTGGATAGGGATCTCGGTAATGACTTCGCCGGAAGTCGAAGTGATGGGGCGAGAAAGCGGGATAACGTCGTCCTTCTCCGCTACACGCGGTAGACCGATCATGATGGGATGCAGGCGGAGACTTTCCTAAGAGGGCCATTCAACGTCAGATTCTTCGACATGCACATAAAAGGCAGGGGGGATTTCTCAGAGAAAAGATCGATACCTTGATGGCATTCATCGTCAGCGTGAGGGAGTCGAGGTCCTCGATCGTGAAATCGCTTCCCCCCCGGGCTGTGACCCGTGCGCGGAGGCCACGGATCTCCTCGCGCATGCGGGCTTGATACTCGGGGTTCCTGGCCAGCTCATAGAGGAGGAAGGTGAGCGTGGTCGAGGTCGTCTCGTGGCCGGCGAGCGTGAGTGTGAACATCTCCGCGATAATCTCCTCATCGTCCAGGCGGGTTTTGAGGTCCTCCGACGCATTCGCTTTGACTATGGGGCCCCACAGACAACGTGCATACGGTTAATTTCCCGGGCCTAGATATTGTAGATCGAAGGTGCTCACTGAGAATGCTCAGGATGTCTTTACTAGGGACCTTTTTCTCCGTGTCTACGTCGGATCCTTGCTCGCGAATGATCCGTCTGCCATACTCCCGGAAGAGGTTGTTGAGGCC

>AT22928

CGCGACGCGGTGAAGCTGTTAACTTCTGCGATCGTGATAAATCCTGACGCGTCGTCGTCGAACGCTTCCGCGATCGCCTGTAGCCTGTTGATATTAATCCATTGGAGGGCCCATTCATCTTGATCGGCCACACGACTCGCGGATGCGTCTCCGGAAGTCGCTTTGCGTTTGCTGTCTACCTGTTGGCGGTAATAGTCACGGAGTGCCAGTAC

>AT23105

GGCCTTAATTGTCTGTGCTATTCGTGAACTTGGTGGTCGCGAGGCCGACCTGGCCGATCATCCCGTTGTACGCGAAGTAGAAGCGCTCGAGCCACGCGTAGCCGTTGACGAAGTCGAGGCCCGAGCCGCTCTGCGAGCCGATGTCCGCGACGACGAGGTAGATGCTGTCCGCGTCACCGCCGATCGCGGTGTTGAGCTAGAGGAAGGGCACACGCGGGCTGTCAGCTACTCGCGCGAAAACACTGAGAGAGTGGGAGCGAGGGAGAC

>AT23419

CTCGATCGTCGCCTTGTACCACCGCAGCCGCTCGCGGATCTGCTCCGTGACGCGCGTGAAGAACTCGAGGGGGAAGTTCGCGTGCGTCTTCAGGTACTGCCCGTGCTGCTGCGGGTTGCGGAAGCCCTCGATGATGCGCGTCGCGACGATCGTGTCCTGGACAGTCTGCTCTGCCTTGAGCTTCAGGTCGCGCGTGTGCTGCACGTCGGATTGCAGGACCGAGATTGCGCCAGCGAGGACCTACAGACGAGCATGGTTAGATCTTGTTATGGCGCGGTGTAGCTGGGGGAGGAAGGGAAGGGAAAGGCGAGAGGCAGGTACCTTCTGGACGGAGCGAATCTCCTCCTGGCCCTTCAGTGGCTCCTCGCCGAGCTTGCGCTGTTTGAGGTCGTTGCATATTTGAATGCGCGATTGGAATTGCG

>AT23630

CCGACTTTCCACCTTCAGCGTCTTGTGGGGATTGTGTGGTCATAGATCTACTAGCAACTTCATTACAAGGATCAATATGGGCCTGGCGCGACTTTCGGCATGACCGATTATAAGACAAAATGATTATACTAGAAATTGGTGGATAGGATGGGCAGTAAAGCGTAACATGCCTCTCAGGACAAAGAAAGGCGAGATAGAAGGAGGGGATGGGGTTCTTGCGACTTGAGTGAGGACAAGTACGAGCTCGACCTAGGTCAAACCGCCTCTGCGGGCAACACCGTTTTTTGCTCGGAGATGAACGATGCCGTGCTCGGCAGGTAAGGCTCGTTATTCGCGAGGTCCGACAACTGGTACGACTCGACGCCGATTTCGATCCGCTC

>AT23631

CGCACCCTGATCCTCTTCCGGCCGCCGTGCTCGCCCCGGTAAACGTTAACATTGACCCCGGATCTCTGTGCGCTGTGTCCAAGAGACTCGCTGCCGTTACTACCAGCGGCCATCCACGTGCGCCGGGAGTTCAAGCTCGACATGAGCGAGTTCGTGTACAGCTTCGCGAGAGGCATGTTGAATATCAGGTCATACCCGGAGGGCTGGCGAAAAAGGGAGGCAACAGTATCAGTATTGATATTTCGGAGACAAAAAAAGGACGAAAGAGAGAATATTCACTATGGCCAGGTAGAGAGCGAGGTTGATCAGCGCGAAAAGTGCCGTGATGAGCCCGGTCTGGACGGTGACTACATCGAGTGCAAGCCGCCGACCAGGGGT

>AT23632

AGTGCCAGACAAGTACGACGGTTATTGACACGTCTGCAAGGGCTGCCGCCACGAGCCAGATGATGCCAATAACCCGGAACTTCCTAAATTCGTAGAACCGGGGAACCATTCCAAGAGCGATGGTGCAGCCTAATCCGCCCACTGAAAGGCAGATATGTGATCAGCTCTCGGACTTTGGAATCCGATGACAGGGGCCGACAACGACGCACACAGCTGTGTGATGGCGCAGAGGACGATGCCGCCAACGATCCAGTGATTCGAGGTGAGCACCTTCACACGCCACGCAAAGAAGAACTGCACGAGGGCAGCAATGAC

>AT23702

AAAACCGATGCATCAAAGAGGTCTTTGCCTGAGCTAAGCGTCGGGTGATCCTTCTTCAAGGACTGAAACAGCCCCTCCGAAGATCGAAAATCAGGGATCCCTGCCTGCACGGAGATGCCCGCACCTATCCAGAAAACCATAGCAAGGTTGCGCGGGTTTGTCAGGATGCGCCTGCGGTAAGGAGAGGGTATTGGACACTCACCGCACACGACAGCGATGCGGCGTGCCTTGAGGACGGCCTTGACGACCCGCTTGACATGGCCTGCTGGGTCGGGCGAGGGGACAAGAAACGCCGGGGTGGGCGGAAAGGGCGCAGCCGCATCCAGCGGGAGAAACGCCGTCATCGCACGGAGAGTGGGCGACGGCGAAGGAGGCTGAGAGAGGGTGCGCGAGGCAGCTGGGGAGGCGGGACGTCGTGGAAGAGAGAGAGAGAGAGAGAGAGAGAGGCCTAGAGGGGTTCGCTTTAGAGCAGGGCCCTTGACGCGGTGGCGCGCCGGTCGGGTCGGCAGCGCCTTCCACCGCAAGCCTTATAACGCCCCGCTCACCCTCCTCTATCTCACACCTACACACCTACTTACTCCCACTTCTATTACCCC

>AT24285

GATAACTTCCAGAACTATTATTACCTTGGGTACCAAGGTTACTGCACACCCATAATTCGTGCCTGATATCACCCGGTTTACGGCGATCATCGGAGTTCCTCGAGCGCTCCACTTGTGCCTGCCCCAAACGATACGATTCCAATATCTGGCAATGGAACAGTACCCTCGAGCGAGGTGAAGCATAAACATAAAGTTAAGTCGCGATAGATACACAATAAATTACAACACGAGATGACTGATGCACATAGTCTCACTTCAGTAAAAGGCACCCACCAAACTTTTTTCTCGCAGCAACACTCATCTTCGCCTTCACGCCGCCCCGGGCCCTCCACCCCCGTCCCACGTACTCGGACGTGACTCTCGCTGGTCGCGCATTAGTATCTCTATCCCCCACAACATCTCTCTCGGATTCCGGCGCGTTCTCCCAAACCCCGAAATACTTTACGACTGCGACCGCGCGCTTGACGCCCGAGTCCGCTGATAGTGTCCCATCCACGCTCTGCACCAGAAACTTTGCAAAATAGTGCACATTCCGCTGGCTCGGCGTGTGCTCCGTGAAGCTTCCACCCAGCTGCTGCACCGACTTGTCTGTGGCGTTCACACTCACTCGCACTGGGGAATTGACCAGATGGATGGGTCAGGCCCACACGCTCGTCGTCTGGTGTGCGCATAAGCATAGGCTCGCAGGCTGGGTGCGTATGCACTGTTATTTCGCCTGTACAGCACTTCCCCGGGCCTGTTATATCACCACATGTACTGTTGCGCCGGCCAATGCTCAATCTATCCACTTACTGGGTGCCGCTCAGGAC

>AT24404

AGCGTATTACGGGTAATAGACCCGCGCCGAACGGGCCCTTTATATTACATGTCTCAGGCTAGTGCGATATGCCGCAGTCTTCAAGTTCAATCAGTCATGCCGCCCATAACGTAGTCTCAGCACACGCCGGTCGAAGACGTCACTAGTGATCTCTGGATCCAATGTGTTGGCAGGCTATCTATCCCCCTACAAGTATCACCTGAAGACCCCTTCGACTCGTCCTCTGGCGCCTGACCGGTGACCATCGAAGCCGCTCGTCGAGGTCGGCCGGTCAAAGAGCCTGCTAAGCCCACCGACGTCCCCTGATAACTATCATCGCTGCCAATTTCGAGCTTCATTTCCACATCGCCGTCCTCTTGCTCCGAATCCGACTCGATCTCTATGGGTGTGCGCTTCGAACCCGTCCCTGTCCCCTAAACCCTTCTTATTGGGTTGGCCGCGCACTGCTTTCGGCGTTTGGGCTGCGAGTCCGAGTTGTTGCTCCCGCCTTTCCTTTTCAAGCGCCTCGACACTTTCACTCGGGAAAGTGCTCTGTCGCCGTGAGGATGGCGCTGGGCCTGTGGCTCAGGCTGGGGCTGAGGTGAAGGTTGAAGCGAAGGTCGGCGGGGCGAAAGTCGAGGGGAAAGGTGGGGCGAAGGAGCTTGGGGCGAGAGCTGAGGCGGACTCGGAGGGGCTTGGTCTTCTTGGGGCAAGTCCTCCTGCTCGATGTGAAAGTCCTATGCGCACTAGCGATGCACACTCACTTTTGGCAGAGTCGTCTGAAGGTTCCTCCTTCTTGAATTAAATGACAGATGCGGGAAGGATCCGTGTCGTGATTGGTTCCAGATCGAGTCCCAAGGGGATCTTCATCCCCGAAGCTTTGAGCTCATCCTGTTTTCCATCGTTGGTCTCGTCAGTGCAAATACATAAAGCCACATCGCTAGCACACCTTATCATCGACTTCCTGATCCACCCCCGCCCTACCCCCCGCAGAAGACTTTCCCGACTCGGACTTTGCCGCGCGTGCGGACATGCCAGAACTTGAAGGCGTCGTAGGACCTCCTGACTGTAGGAGACGGCGATTATCGATGGTAAGTAGTTGAGGTCGGATGCTGGCAGTCTAATCGTGACTCACGGTCG

>AT24470

TGGGCCTTAAGCTTATCAAGGTGGATGACATTCAATGGTATACTGCGTGAAGGCGACAAGTTAGCTTCCTTGGCACAATGAAATCCTAACATGATCATACACGGCGGTCGCGAGCAGATGGGTCACGAAATGCGTAGGAGATGATGCGACTCGGAGTAAGTATATGTATATCAGTATATGAGAACGCGGGCGCTACAAGGAGTGTGTGATACGGACCATGATGTGGTGCGTAGGTCGGGAGAACTTGGGAGAGACATTTAAGACACGGTAAGGACAGATTCCGGTCAGTAGGTAACAAGAACAAGTCTTCACTGGGGCAAGAGACCAAGGGAACTATGGCTCATCCACACGTGATGAGCGCCGGAAAGACAAGGGTAGCGCGCCGCATGCGCTACAAAATACTTGACATGCACACAAACTTGAAAATCTTCTACGGTCATCGCCCTTATAAGCACCCGGACTCAATCATCTTGAGAGAGCACCAAGTCAGCCAGTCGGTATCTCGGCGTCTTCACGTGCCAGGCTATATAGCCCTGCGGCCTCCTTGGTTAAATCACAGTTGCGGCATTCAGTGCATGGCTGCTCACCTGTGCGAATGAACGCCAAAGCGCCATCAGCTCACGGTCCTCGCCTATGAGCAGACCAATTTTCTGCATGCAGTCCAGGGTTTCGTCCTTTAACACACGCGCCGTGCTGACAACGGTAGCTTCGTCC

>AT24474

GTGGGTGACGTTGGTGAGACCGTCGATCTCATTGGCCTCATGAATGGGTGGGGCATCGATGGGTTGGATCGGCCGGGACTGATGGGCGGAAGCAGTATTGCGGCGAGCGATGCGTGCGGCGAGACGGTCTGAACTGGAGGGGATGGCCAAGACCAGGGTGGCGAGGAGCACCTGGCAGAGGAGAGCGTTGAAGAACATGGCGGAGAAGGAGGCGAGGAGAAAATCAACTCGGGTGGGACATCCGTGTTTGCCTC

>AT24496

TGTGGGGAGCTATCACAGCATTATCCTCTTATGTAAGTCACAAAGTACTTAAGTCGGGCTTAAGCACTGTTAGATCTGGCTAATCCTGGCAAGTCAGCGCCGCTTGGCGCAACTGCCAATCATTGGTTTGCCGACTTTGTATCTCCCGAGCTGTGTTACGACTGAACGTCGAGTAAGGATCAGGGATATGCACGAGACGAGCAGGAGCGCCGACGAGCACTTGTGCAGCAAGAGCGGAGCAAAGCTCGACGGTCAGGGCTGGAGACAAGATGTATTGAGTGAAGTATACTACAAACGAAATACAGCGAGGCTTACCTGGAGACAAGAGGTCGAAACTGAGGTCTCCAGGTAGCAAAG

>AT24862

CACCTTAACCTTATCGACCGCCTGCTCCTCGGCGTCCGAGAACGCCGTCGCGCGCCGTCCCTTGAAGATGCTCTCCTCGTTCGCGTCCGTCGCGCCCACGAGCGCCGCAGGCCCGTGGCACACAGCCGCCGTGACCTTGCCTGCGCGGAAGAAGTCCGACGCGAGCTTCACGTTCACCGGGTCCGTCGCCAAGTCAAGCACAGGCCCGTGTCCGCCCGGGTAGAACACCGCGTCGTAGTCGGCGGCGTTCACGTCGGACAGCTTCTTCGCAGACGCGAGCTTGGCTTTCACGGTCGGGTCCTCGAGGAACTTGGGCACGTCCGCTTGGAA

>AT24917

GAACACCGATCCGGTCGCGTCGGCCATGTCGATGACCACGAATCCGTCGAGGATGTAGTCGATGTCCGAGTCGGAGGCGTCGGAGTACGTGTCGGGCCAGAGCCGGTCGTCGTTGTCGTCGCCGGCGGTCGAGGCCGACTCGCCAACGCTCGTCCCGTCCCCCAGACCGTCGACATCGGGCACGAAGTAGGTGGGGTAGCCAGGCCCACCCACCACGAACGCAGCGCCCACGACCCGTTTGGCGGTAGGAGCGTCGGAGTCCTCGTTCCCTGTAAAACGGTTCCCCTGAAGTACGCCTGCGTACGATCTGTTCTCGGGGAGCTTACCGGTTCGCTGAAGCTTCTTCCAGGCTCTCATCTCCTCCCACATCTTCGCGTTCAGCGGTCGCTTCACGAAGGTGCCGGTGTCGGTGTTGGGGTCGATGACGAGTCTGAAGAAAGGAGTGCCGAAAGGAGTACGGGCCTCGGCTCCAGGCCGGAGGATGGCAGTGGCGCCGGACTGGCGAAGGGTCGGAGAGTTGGCGCAGGACATGATGACGAAGAGGATGGCAAAGGGCTGGTCAACGGCACAGCGAAAGGGTGCGAGTGGATGGTGGTTGTACGGGCGCA

>AT259

TCACCGGTAGCGCGGCTCCCATGCGAGGCGAGTCGGGAGTAGGTGACACGTCTCGGCTCCGAATGGCGCCATCGGAAAGGGGACCAATGTCCTGAGTATCCTGCGAAGGGATCGTAAGGGCCGAGTCCGGAGCCATGGAAGTGGGCGGCGGAAGATCGGGGGCGGAGGTAGCAGCCGGAGCGGCCGGGAGAGCGGGAGGTGCCGCAGCGGTGCCATTTTGAGACGAGAAAGCATTGTTCCCCACGGCCTGATTGCCACCCATAAGCTCGGTGACCTCGTTGAACAGGGGGAATGCCTTCTTTCGCCACTGGTTGAACTCCTTGAACTTCTTGACCTGCTGCCCCTTCTACACCGGCGACCTCCGTTGAACGATCGAGCTGACAGAGAGAGAAAAACGTACCTCATCATACAGAACCTTCCGCCAGACCGCCTCCGTTGCCACAACCACCTTTTTACCCTCATCCCACCCAAATCCAGACATTCCACGGAGGCGGGCAACAGTGTTGCGAGCGGTCTTGAACTGCCGCGTGTTATTATGTCAGTTATGTTCTTCTGCCGCCCGTGTGAGAGGGAAGAACCATTACGTACCCTTGTCCACCGGGACTTCACCTTCTTCTGATCAATCTCATACCCGTGCGTCTGGAGGGTGTCCGAGATGGTCTGGTACACTGCAGGCTGGAAGTTGTTGTCCCCGGTGCGTGCCTCAGCAACAGCCTTCTTGAGCCCCTGAATAAGGAGATCTTCCGAAGTTTGATCCCATTCGATACGGACCATGGCGGAACCGCCAGACTGGTACACAAGGAAAGCTGGCAGCAATGAGAGGGGATGAGAAAGGACGGGCTCACAAGACGAGCAGGAGCGAGCGCCGATCTGATTTCGGCACAGAATTAGCGCCTGGAGTTATCGGATTCTTGGAGCAATCCGAACGCGTGAACTCTGGCGAAAATCGCGATTTTCTCGTACTACGAAATAATTACTATATATGGGGCGAAGTAGCGGTAACTCGTTTCCGCAACTCTTTGCCGAACAGGCCCTTAT

>AT3160

CTCCCGCTCCACGAGTGCCTCCTCCTCAAAGACATCCCAGCACTCCCCGCACAGAGACTCGTTTCCCCGCCCGCCAATCCGCCTAGCGATGCCCTCAGCCTCATCCCTGAGCTCCGCGCACTGCAGCAGCAGCGCGATCGGATCCCTCTGCGACCCGCGCCGCCCCGTGCCCACCGTCGAGATCATACGATGCAAGGCCGCGTAACACCCCACGCCCCCAACGTCGTGCGCATCCTCCACGTCTTCCCCCGGCCTGCACCGCTCGCACAGGAACGTCCGGATCGGGGGGAAGTACTCGTCCAGCTCCTGGAACCCGCGCAGGCAGCGCAGGAGGTTGTCCGGGCCGAGCGCCGCCCAGCGCGCGAGGGGGTCGCGCTTCGCGGCGCGGTCCGGGGGGTCGCCGGGGTGCTCGCGCAGCCGCCAGTCCGCCTTCGCGCTCGTGCGCGCGAGCTGGTAGAACGCGGCGGGGAGGATCTCGGGGCACCCGAACTCCTGCGCGAAGAGGATGGCGGCGACGGGCTCGGGGATGAGGTCCGAGAACGCGCCGTCGGACGGGAGGCCGCCGAAGGGGGGGTTCGGGGCGGTGAGCGCGAGCTTCCAGAGCGCGGCGATCTCGCCCTGGAAGACGTCCCACTCGTGCAGGGTCTTGGGCCAGTCGGCGGTGACTTGCTTCACGAGGTGGTCGTGGAGCGGCTGGAGGCAGTATTTGTCGGCGAGGCGGATGACGCCGCTGACGGCGATAGGGGTGTTCGGGTCGTGCCTCTT

>AT317

GCGAGGGGCATAGAGATTGCGATGAGCTGGAGGAGGTGGTGGGTCGACAGGAGCAGCGATGAGAGGGTCAGGCGACGAGTCGCGTGGTGCAGGATCAGGAGCAGGGGAAACTGAGCGAATGCGAAGACGCTGTGGCATGAGGTTCTGGCGTTCAAGCTCCCTGTTGTTGAGGGAGCCTTGGTAACGCGACGAGCGAGATGGGAGCTCCTTGCGAGCGAGGGCAGGATACGGCCTGAAGCCACCTTCACCT

>AT3216

GCGGGGCCATATCTGGGCGTTGGGCGTGAACTCGTAGGTGGTGTCGCCGATGTGGAAGAACAAGCTCTGGAGGTTCGCGAACTGCGCGGTGGTGAGCTTCAGGAGGCCGGTGGTGTTGTCCAAGACGGCACCAGTGGCCTTCTGGTACCTCGCAAGAGCGTCTGTTGTGGGTTGAGACATTGGTAAATGAGGATTCTAAACATGATGAATAGGAAAGGCGAACCCGATGCGAGGAGAACGAGGGTGGTACCGGTGTCGGTGATACCAGCGGTGGAGGAGAGGATAGTTGTGCCCGAAGAGCCGTAGGTGATGGACTGGTCAATACCAACATACGCAGACGCGGGGCTAGTCGAGGTGATGGGGCTACGCGCATGCGTTCCGTCAGTAAAAGAAACTCAAGCCCGAGAGAATTTGATACTCACACGTATGCGATCTCCCCCGTGTGCTTGCTATCGTCGACGCCGCCAAACGTGAGCTCGCCGTTGGTGACCGACTCCGACGTGGTGGGCTCGAACGAGATGCCGATGAGGTTCGCATCGATGGAGCCCGCAGCGAACGCGGTGTTCACGACGGTGGGGATCTCGCCGCCAGACGACGTCGTGCCCTCAGTGAGGTCGACGGGCCCGATGCCGAGGATGCCGTCGTAGCCGGAGAACCCAGAGGACCTGCTGGCCACGCCAACACCCTGGCTCTGGATGACGAGGTCGCCGATGGTCACGGTGTCCTTGTACTCCTCCCCA

>AT3332

CGCCTTCGTGCATCGCCTCCTTCAGCTTCGCCACGATGTCCAGGAATCTCTCCCGGGTTCGAACCAAATTTGAATGGCGTTCATCCATATCGTAATTAGACCACATCGAGAGCATCCCGTCCCATTTTAACGAGAACGGCCGGGCGACGTGCAACGTGATGCCACACCTCGTCTCAATGTGACGCGCCGCTAGGAAACTGCCTGCGCTCAGCAGCTGGTTCATGAGATCGGGGTCTTGCTCGTGCTCGCGTGAGATGGTTGCAAAGTCGAGCACCGGGCTCCCGCCAAAAAAGTCACTATTTTCTGGGCTCAATTTCAGGGTTAAGTGGTGGCGCTTTGCGTATTCGAGCATGTATTGGTCGGTGAAGGGGATGCCGTAGTGATAGAGAGGGGCCTTCGCGCCGTTCCTCGCGACGAGCTCCCTAGCAAGCTTCTTCCGAGGACCGCGATCATCCATGAGTTTCTGCCATTTCTAAATCGCTAGCTGTGCCTCCTGGGCTGCCGTGGAGTAGTGGGCGGGGTTGTCAGGATCATCCTCGAAGAGATATTGGCCGTTCCAGAACAAACTAGGGTCCGGATCTGCAGGGACAGATTGAGACATGGTAACGGACGTCTCAGAGAGTCGTTCGGATGGCTGGGGCGGAACGTTGTGGTTTGGTTGTGGTTCACCTTCAAGTTGAACTTCAAGCTTCAAGCTTCA

>AT3342

GAGAAGTCATGAATATCCCCGAAAAGCGCTCCTGTGGAGCTGACGACGGTACTTACGCCGTTGATCGTGAAGTACAGGCACTTGAGGTTGTCGTACTGCTCCTTGGTGATCTTGAGCAGGTTGGTAGTGTCATCGACGACGGCGCCAGTGGCGTTCTTGTACACGTTAAAGGCATCGGTGGCGAGGAGGATGAGGGTAGTGCCTGTGTCGGCGATGCCGGCTGTCTTCGCGAGGATGGTTGTGTCGCCATACCTGATCGACTGGTCAATGCCGACGTACTTCGACGCCGGGGACGTTCCAGTGAGCGTACTAGTGTTGATATTGCCGATGAATCGGGAGGAGTCGGAGCCGCCGAAGGTCAAGAGACCGTTGGCCGTCGACACGTTCGACGCGGGCTCGAACGAAACGCCAAGGGCCTCCGCGCTGATCAGACCCTGCTCGAACGCGTTGTCCAAGACCGTCGGGACGATCTTCCCATCGGAGATAGTTCCCGCAGTCAATCCGGCGG

>AT3367

ACCTCGAGCACTAATATAGTGGCAGCAGTCTTCCGCACTTGATTCTCAACATATATACACGAAGCGACAATTCATAGTAGACGCAGATTCAACCATTGTCGTGTGAGATGCCATCCAGCTGCCAGCATTCATGTTCCCAGTGAGAGCAATTTAGACTGCATGACTAGTCAACTGTACCTTACGTTTCGAGCAACCACATACTAATCCGTACGTATGCTACATAAGAAAAACTTAAATCTAAACCGAGTCCTCCATCCATTAGTTGTCACTATCGATTCTCGTCCAAATCATCCATGTGAGCGTGTGTCGTAACCCCCCCCCCTCAGAACTTGAACCCCCCAGCGAGGGCCATAACGCCCTCGGGGTCGTACCGCGCCTTCACCGCCTTCAGCCCATCGACGTTCGCCCCATAGATGCGCTCGAGCGGCGTGTCGAACATCGCGTAGTTGCCGTACAGCGGCGCGTCCGCGACGTTCTGGCCCTCGCTTTGCGCGACCTGTTCCAGCCACGCCGCGCTCTGCCGCGCGGCGTCGTAAAACAGCTCGTCGGCGGCCTCGACGCCGTACGCGTAGTAGATGTTGAGCGGGAGGAGGCCCTTTGAGCGCTCGGCAGGGTACGCGGAGGAGTTGTCGGCGCCATGCGAGAAGAGGGAGGAGAGGAAGGGCTCGACGTCGTACGAGATGAAGGTGCCGGAGCGGAGGGAGAGGTACTCGCCCCAGAACTAT

>AT3376

ACCGGGAACGCCTTGTGCACCTGGTTGACGTAGCTCTGGAAGGTCGCGAAGTCGGGGCCGTACCAGTGCAGGTTGATGTAGTCGAAGGAGCAGCCACCTGCGCAGGCGCTGACCATCTGGTTCAGCCAGTCGAGGCCCTGGTTCTTGCTCCCGCTCGACGTGATGGCGGGAAGCGCCTTCTTGGTTGGCAGCGAGTTGATGTGCTGCTTGTACCAGGTCGCGGCCTGCGAGGGCGAGATGCCGTTGATGTCGGGCTCATTGAGCGTGAACACGGCTAGGGATCGGAGTCGTCGTTAGCCACTGTTGGCACTGTACCGAATGTGGACTCGAAGGGGGACACGCCACGTCACTTACTGTTCCATTTCTGGTCCTTCTGGCGTGCCGCCAGTTGGCTGATGGGGCTGGAGGTGCAGTCTAAGCAACGCTGCATTCCGATGAAGTTAAGGCCGTGGACGCCGCTTTTGGACGGGGGCGCGTATGTCTCATAGTCGTAGCTAT

>AT3397

GGTGGGGAGGCGGGGATGTTGCTAGTGGACGGTGAGGCAGGGGTGTCGTTGGTGGATAACAGCGAATCTCCCTCGCCCAGTTCGTCATCTATCACTGGCAAGCTGAGCACCCATGCCAGAAGATCATCGTCCGATTCAAACTTGTAGTTTTCTTCGGAGCTCAAGGCCGTTTGCACGATTGGCGGACTTGAACTGTCTGAGAC

>AT3450

TCCGAAGGTAGGCACCGAAGGCGAGCATTTGAAGATGGGCCCGGAGAACACGCTTAAGGAGGGCAGCTAGGGGAAGTGCAGGATGGCTCTCGAACATGGACGGCAAACAATGTAGGTGTAGGGCCGTCTCTTCCCTGCCATCGAGCCTAAGATATCGATATCTGATCCCGAAGCGGGCTCATCTCTGAATACACATAAGGATCGTCGAAGCTCATTACGATTCTCGGGTCGACGCCTGATGCGCGTAAAGGATGCAACTCAAACCCAGACCACATTCGAGGACGGGCTGCCGTGGTATAAGATCGCGATATCATGCTCTAGAGAAGTGCAGGTCTACATGATCTCGATAATCTCGGGTCCGATTTCCTCTCGGAGTTTCGGAAGTCGTCGGTGCCGGCGGCGACCATCGTTACAGTACGCCTGGGCATCGTTGTGGAAGTCGAGGTTCAGGTGAGCGGCCAATAAAGCCCGGGCAAGGCCCCCTGCAATAGGTGTGTGTTTGTAGATGTGGCGCTCGCTGT

>AT3467

CTAACAAGTTCCTGGAAGTAAGGGCCCGCTGCTGTCGCGAGGCGAACACCCGCACCACCTGGTCCTCCCGGGTTGACGAAAACCACACCCTTGCGGGGCGAAGCTGTCGCATTGTATCGGCCAAGCGCGATCTTTGCTACGCCGGCACTGGGATTGGTGTAGTCCAACGGAACGCTTGAACACCCAATTAATGTTATTAGCAAATTCCTCCTGGCGCATCCAGGTGTTGGAAACGCTTACATAGCATACCCGCACTCGGCTCCGGCAACGGTTTTGCCGTCGCTGGGAGTGCACTTGCCCAAGACGAGGTCCCCAAGCTTGCCCTTGACGGGCTCAACGGAGAGCGCCGAGCCAGCAAAAGCGAGTGCGCCGCCAGCAACGACGGTCGCGATGTTCTGCTTCCAAGGCGCAGGCATAGCGTCTTCACCAACGGTCAACTGTGCGAGATACTGTCCGGAGTCGTCTGCGACTGCTGGTTCAGCCGCGAGAGCAAAATCTAGTTAAGTCAAAAGCAAGACCGCGCCACCGCAGAGAAGAGAAATCAGTACGCCGAATGAACGAGCGGCGAGAAGGAGAATGGGACGATTGCATGCAAGGCAACGCGCATATATACGTCGCTTGGAACCA

>AT39

TCGGGGGATCGGACTCACACAGCAAGCCTCTGCATCTGTCCCCCGCTCAGCGTCGCGCCCGCCGCCGTCTTCGGCACGCCCGCGGCCGCGCGCAGGGGCTCGTAGCTGACCGTGCGCCCGAAGAACGTCTTCGTGCCCTCGACCGTGCCCGAGTACTCGTCCTGCACCGGCCGGTGGAGGTACGTCTCGATCCCGTGCGGCAGCTTCGCGATGAAGCCCTCCGCCCCCGCGAGGCGCGCAGCCTGCTCTACGCGCTCCGAGACGTCCGCGGAGGTGCTGGTGGTGGGGTCCCCGAGGGCGATGTTGTCGCGAATCTGCCGTTAATATTCATGTCAGTTGATGTCAGGGAGAGGGGGGGGGGAGATGTGTGCGCACGGAGAGCGGAAAGAGGGTGTAGTCTTGGAAGAGGACGGAGATGGTGCGTCGGAGGTCGTCGAGCTTGAGCGTGCGGATGTCCTGTCCGCCAAAGAAGATCTCGCCCTCGTCGGGGTCGTACAGGCGTGTCGTCAGTTTGAGGATCGTGCTCTTTCCGGCACCGTTCGACCCGACGATGA

>AT3967

GCCCCGCAAATCTGAGGTCGCCGCGTCCGAGTGTACACTGTCATCGAAAGGCGACTCGGGCTTGGGCCCTGTCTGGGTCGACGCAGATCGGGTATTTGACCGGGATTGAGGCTCGGACTTGAGCCAGGTCTGGCTGATCCTCGACGTGACCGTCACGGGGACCTTGAGCAGCATATCGTTGCCCATCCCGGGGAACGGCACCTGCAGGGAGAGGTAGTACTTCGACACGGAGCGAACACAACGGCCCGTCAGCGCGAACCGTCGAACTTCGTTGTGGAAAGGGTGCGCACGTTCAGCGTGAACGCGGACGAGAAGCTCGGGCGCTACAGTCATCGCCCAATCTCCCCTTCCCTACCTCCCTCCATGGCCTTGAGATGCCACGCCTCCGCCAGTGCTACGTCCGCGACGATGCCCTGCGCAACTGTCCTGGCCTCGAAGAAACACGCGACAGCCTCATCAACCCTTGCGGGGAGAGCAAAAGGTTGCGACGAAGACCGAAGTGCATGTCCATGTGCTTTTGGAAGGTGTGGAGAAGATGGGCTTGTCCAACGGGTGGGTGGAAGAGCGCCTTCGCGCGGGTCATAGGGGTAGTCGTGGTGGTGATCGTGGTGAACAGTGGAAGAACGGGAACGTCTGGCA

>AT4011

GCCCGTACGCCGTCTGTAAGATTGCTCGTGCCCGGGTCCTGGCCCTGGGCGTCACGCGTTCTACATGCACTGGATGATATTCTCTATGGCATGATATGATAGCGATTAAATAAAGCGATGACACAAGACCTAATACTCCAGACGGCGATCGAGATACCACATCAACTGGTACTCCTCGCCGTAGTCCATGTCCTCGAGCAAGTCCAGGATCAGCTCCAGCTGGTCCTTAGTGACACGCAAAAATTCGTGGTTGGAGAAGAACGTCAGGATCGGAGTAGGTTGCCCCCATACGAGGTTGAGGTCCAACTGTAGCCCTGACACCTTCTTGAACCACTTATCGAAGTTTGCCCACGTCTGGAACGTGTCCAGGTCACCAGCTTTGGCACGGAGAAGCGCCTCTCTGTAGTAGCTTGCAATGCCCATGCGCTCTGCGTAGGGGTAGATCAGCTTGCGGACGAAGGGTACGCCATACCAGCACAGAGGGAGTTCCGTTGGGTCGGCCACGTCCACATGGCGAGGATCGAGCCATCGTGGAATGGTGCGTA

>AT4029

CGCGGGCTCGTCAACAGCTCGCGTGCGGGACCGTACGGGCTGGCGCGCACCACACCCGCCGCGCACACCCCCATATGTCGCGCGCACCCTCCCGCTCGTCCTGCCCATTCAATCCGCGCACTCCCTCCCACCGCGCGCTCCTCTGATCGAGCTCCGCGCGCCCGTCGCATGCGGTCCTAACGATGATGACCATTAGCTGACGTCATAGGGCGTGTATCTTCACATACCTGTATTGCGCGCGAGGCGCGCGCCCGGGAGAACAAGAGGGAAGGAGGAGGCAGAGGGTGGCGTAGGTTGCACAGTGGGGAGATGGTGGGGGGAGGAAAGAAGGCG

>AT4103

AGTCGGCGGCTGGCCGCCAACCGTGTCGAAGCGGAACACGTCGTCAGCAATGTGCCCCTCGACGTTCGCTACGTGGATCCACGTGACGTCCTTGGTCGGGTCCTGCGGCGACGGGATCGAGCCGGTAGTCTTGCCTAGGATGTACGCGTCTTCGTGGCTCTCGAAGGCCGAGCTCGAGCGGAAGTCCCACACGGGGCTCACGCCCTGGCCCGTGATGGGGTTCGGCTGGAAGAAGTGCTGCCCGAGGACGTTCGGCGCGTGCAAGAGGTCGATGATTTCCTCGATGGGCTGCGGGATGACCGTGGTCCAGATCGTGTAGAGGTCGTCCTGGATCGTGGGGAAGTACGACTCCGACGCTATGCACGACGCGTCGAGCACCTCGCTGAGGGCCCCGGTGCTCCTATAGGCGGGATATCATGGACGGGCATGATTAGTACGAGGAGCGTTGTATATAGGGGGAGGTGAGGAGGACGTACGTGTAACTGTTTGAGCTAGAGCACGTGTAGTTCTGCACGCCGAACGCGAGGCCAAGGAACTTCGGAGAGGTGTTCGGGAGGACGAGCTGGTTCTGCCCGGAGGGGATCGTCGGCACGAAGTTGTCAATCGAGCAGAGGACAGCCCCGTCGGTGGACTGGCCCGTCCATTCGTAGGAGCTCAGGTAGCTGGCAGCAGCAGCGGCAACGAGTCCTCGGATAGCGACCATGGTGTGGATGGATCAGGAAGAGATGTAGCGGGGCAGGAAAGAGCGGCGACGCTGGGGACAGCTTGTGCCTCCCGAAAGCCCTTTATACCACCACACCCGGACGCGTCAGGGAAGGTCGCTCATACGGGGCTGCCGCTCGGGGTTGTCTAAACAGTCTGATTGGACATGCTTGGGACATTCAGCACTTCCATCTGAATGATGGACCGAGCGTCGCGCACGTCCGACTCCTGTGCAGTAAACTGGGAAGGTGTAGTTGCAGCGCGCAGCAATTAGCAAACCGCGCCGTCATCATTTACAATGACATACCCGCCGAAATGAAGGGCGCCAACGCTGCTGAAAAAAGTAGAATCGCCCTCGTCGTGAGCGGCGCGCCTTGCCTGTTCCACGCACTTGATAACCATGACTCGATCACCGCAGATCCTGCGCGCGATCCAAACCAGAGGGGCGGCCACGAACACTTGAACCTGTTGTCGCGTCCTCCTGAAGCTCCGGACCGTTACAAAATGCTGAGAACACACAAATCGATAGCGCACCAAGTATGTAGTATCACATCAATTAGATGGCTTGCTGAAGCAAAGACCAATTAAGTACTTATGCGTACCTGGCTTGAACGTTGTGAACTTGTAATCTCGCGCCTCACAAGCGAGGCGAAATGCCCGTCGGCTTTACA

>AT4127

GGCACCCTGTATTATCGTTACTAGTTCCCAAGAGATACACATCCCAGAAAACACAACAATAGAAGAAGACTATCTATCTACCAGTGACTAACAACCCTCGCACGCGCTACGTAGGCACATACCGCGCCCGGGGAATCAAAGCCCAAATAACAATGAGCGTCGACATCATCGACACGATGCTGTAAGCGAAACGATATGGCGTATCAGCACTTCATCGACCAGGTCACAATGTATTATGTCGGCAGCAAATAGGCACTCACAACGGCTGCAGACTAGCGATTCCAAACTTGGACGCGAGCAGCCCGGTGAGGAACGGCAGCACCGCCGACCCGGCCTGCCCGATGCCCGCGATGTAGCCCACGCACCCGGTCAGGAGCCATCGCGGGAGGATGGTGGTCGACTGGTTCATCAAGATGGGGTACATGGGCCCCAAAACCAGGCCGACGAACGAGACCGCGATGGCGTTCTCGATGAGCGAGGGGACCACCCAGACGGTGACTTGCAGCCTGCAAACGTCCGATATGCGTGATGAGC

>AT417

GCCTGGGTATACAATCTTGATTTTGCACCGAAAAGCGGGATCTGAAGGGTTTTGCAGATCACCTAGGGCTGGCGAGTAAGGGAAAACAACACCACCCCTACCCATTCTTCAGAATGGAATCTCTTTCCTGGAAGCACGCTCTGACACTGGGGGCAAAGCCCTGCTGAAAGAGTACACCGGTGTATTCAAATTCCAATCCTTCAAGGACAAGTCACGAGGCACAGTGGTTCTGAATCTCTACGAAACTAGGCGCAACTTCCACCACTATCCGCCCAACCGTACGCGTCGGGGGTGGTGGTCCGTGGACTCGCGCGTGTGCAATTAGAGCTGAACGGGGACGCAGCCGATGGAGATGAGGCCACCCTGTGTGGAAGAAGAAAGGTGAGCATGTCGTAACTAGCGCAGGTCCCGACCTGGGGAAAGGGCACTCACGTTGCTGTTGTCCTGGCAGCACACTGCATTGGCAGAGCAGGCGCCGCTGCCTCCAACGCCGATGACGCTGATGGGGGAGCAGGTCAAGCCGACGGGGATGCTAAGGTCCTGGAGAACGATGCCGAGCAGACCGAGGACCGCCGCCGCCGCCGGGTTGCTGGCCTAAACAACGGGTAATGCATGAGTCTCTGAACAGTACTCGGAGAGGTGTATGTTGGTAACTGACCGTCGTGACGGTGTTGCAGCATTGAATGGGGCCGGTGGTGCAGGACGAAGGGGGCTCGCCGCCGCGGCCCTCGAGGGGGGTGGCGGCCGCAAGAAGGGGCAGAGAAGCAAGAACGAAAGTAGCGACGTGGGAGAACATCATTGTTTGGATGAAGAGAACGCGGACTGGAGATGCTAGTGGGCTGACTGGTGTAGACCTGAAGGCTGAGGATGGTCTCCCTCACCGTTGGATCCCTTTTATACCTAGGTGGCCACTGTACGAAGACCGAAGCAGGCGCTTC

>AT4259

ACCTGCGCCCGCTGGCCGACGAGCATGTCCATCGCCTGCGCGACGTACTTCTGCTCCCAATTGAGCACCGTATCGCGGCTGCTCGCCACGTGCTTGAACCCGGCGCCCATCTCCACGAGCACGCCCTTGAGCTCCTGCACCCACTCTCCGAGTCGCGCCCCGTTCGTCATGATGTACACGTTCTCCAGCCCCTGCGCCGCGCGGCCCGGGGCGGCCTGCTGCACCTCGCGCACGCGCGCGACGATCTCCTCGATGGTCGGCAGGCAGCGCCGGCGGTAGAACGCAGTGTTCTCGGGCGTGTTCTCGCCCCATGGCGCGCCGCCTGGGGGCGGGGTGAAGTCCTCGAGCCTGGCGGGGAGGGCGTTGAACGCGAGGAACATCGAGGACCACTTGGTGAGGTGCTCGCAGTGCCCCGCGTAGTCGCCCCGGCGGAGGTGGAGCACGAGCAGGCCCGGGATGCGCGCGTAGCGCACGGCGTTGGGCGTGTCGTAGGGGAGCGCGGACACGGGCGGCTCGGTGACGGCGGTCGGGGCGATCACCTCGCGGTTGTTGTCGAACGCGGGCTCGACGAGCGCGGACCACCGGAAGGAGGTGATGATCGGGGACGCGGCGAAGCCGGCCCAGGCGTCGTGCATCGCGTCCTTGTC

>AT4754

GGCAGCAGACGGGGCGCTGCGAGCAGGTGGAAGTGATCTGGCCGCCGATGACGGTGACCGGGTTGCACTGGAAGCCGATCTGGCCGGTGACGTCCTGGACGTTGATGCCGAGGGTGGCGAGGAGGGCGGAGCCCGCGGCAGAGTTGGACTGGGTGCGAAACGTGGTCAGCGTTCTACACTGGCACATCTAGGTCGGGAGACGGGAGGGAAGACTCACGTCCTCGAAGCTGTTGCAGCACTGGATGTCCCCGGTGTTGCACGAGTCAGCACCTCCGGCGAGGGCGAGGAGGGGGAAGGCGAAGAAGGCGGCAACGGCGGCGACGCGAGCGATCATGGTGAATGAATGCGGCTGGTGAAGACGGTGAGTGGCTGAAGTATCGGTGCTGGAGTGCGAGAGGGGCGACTGCGAGCTGAT

>AT4756

GCTGCGAGCAGGTGGAAGTGATCTGGCCGCCGATGACGGTGACCGGGTTGCACTGGAAGCCGATCTGGCCGGTGACGTCCTGGACGTTGATGCCGAGGGTCGCGAGGAGGGCGGAGCCCGCGGCAGAGTTGGACTGGGTCCGAAACGTGGTCAGCGTTCTACACTGGCACATCTAGGTCGGGAGACGGGAGGGGAAGACTCACGTCCTCGAAGCTGTTGCAGCACTGGATGTCCCCGGTGTTGCACGAGTCAGCACCTCCGGCGAGGGCGAGGAGGGGGAATGCGAAGAAGGCAGCAACGGCGGCGACGCGAGCGATCATGGTGCAATGCGGTCGGTGAACACAGACAGTGAGTAGCTGGGGTGTTGGGTGCTGGAGTGCGAGCTGATGTTCTCC

>AT4796

TCCCCTCCTCGCGCTCGCTGGTGGCGTTGGTGACTGCAACACTGGGCCCATCCAGTGCTGCAACAGTTTCGAGGATGTGAGCCGTCCCGTCTCCACATTCCCCGAGCGAGACGTACAGAATGCTGACACTACCTCGGATGCAGGCCAGCTCTACTGGGGGGTCCAGCATCCTCTCTATCCTCGGCCTCAACGCCCAGAACGTCGTCGGCCAGATTGGTCTCCAGTGCAACCCGGTCACTGTCATCGGCGGGCAGATTACTTCCCAGTGCTCCCAGCGGCCCGTCTGTTGCCAGAACAACAACGTTGTAAGTGTTGGTGCCACATGCATTTACGATATAGCAGATGACTTACTCTCTGGTTCGAAGGG

>AT4939

AGGGCGCGGGGTCAAGGATGTTAGGATCGAGTTTGCCATCATGTATGAATCGCTCAGGTCGGAACACATCGGGGTCTGTATATACTTCGGGATCATGCATGTACGCTCTTTTCGGTTATTCAGCACATCACGATAATCGCAATCAAACGCAAAGGTAGTTGCGGCTTACCAGGGATCGGCATCGTCCCAGCGGGGATGAAATATCCGCCGAACTCGTCATCGGCAACGGTCGCATGCGACACACCGAATGGGACAGCCGAATGCCACCGCATGGATTCTTTGATGATAGCGCTGACATACGGGAGTGAGTCCCTGTCGCTGAAATCCGGCAGACGGTGTGGGCCAACGACCGCGTCGAGCTCAGCATGGGCCTTCTTGAGCACTTCGGGGTGAA

>AT4940

ATTCTTGAGCGTCTCTTCCACTTCGCCCATAACCTCTGTGACGTTGCGCCCGGGAACCAGGGGGGAATGTGGCGAAGAAACGGCAGGAACTCGACGAGGAATTTGCCGGGCACCATACCCTCCACGGGCCCAGCCAGTGCTTGCTCCGACATCTCCACATACACGTCGGTCTCGTCCCGAACTTCGATGTTGTACACGACCTTCAACACCACCGCAGCAAAGTTGCTGGTTCGCTTGGTCGGTCAAGTCACGAACAAAAATGGTGAAGCGACGGAGTCACAAGCGAATGAGCTGCCTCAGGCGCGAGGGGTTCTCTAGCAGCGCCTCCAGGAAGATACGGGTGACAGATTGCTGTGTCGGCTGGTATCCCTCAACGGCGTCCCGGTGGAAGTGTTGCCAGAAAGCGC

>AT5044

TGGCTGAGTACAAACTGGATGTTGTGCTCAAATGTGCATCACTGCGCATGCCAGATTGTGTGGGTGCCGATGATGAACGACTCGGGGTGTGTGCAGTGGAAGAGGTATGAGAGGAAATCATCAAAGACGTTGACGGCAGTCTTGCCTACTACGGTGGCGGAAGATTGGAAGAGAAACTGGTACCAATGGTCACTCTCCCTAGTAAAATGCAGTGACCTGACGTGCAAACGATGTACGTGTTTGGCATGCCACCTCCAGTACAGTTGGTCGTGTTCACGCTGTTGGAGCGGGTATGATTGAGGTGAGATCTATGCTGAGC

>AT5338

GGTCAGCACTACCTCTTCTTGCACCCCGACCACTACCATGGACACTGCTCACCGATTCCCCACCGTATGTGAAACGCGTTCGCCAGTCCAAACACTCGTGCCCGCACCTAGAGAGTTACCTCTGTCAGCACCGGCCGTCGCAGACGCGCAAAACCATGCGGGTGAGCGCATATAGTCACCTCGTCTCCCGTTATGTGCCGTCCAGTCGCGTCATCGACGAAGTACGTCGGGTTTCGCAGGTGTAGCGCCTCGCCCCCTAACTCGTGGTGCTGGTCGAAGAAGAATTGTGGGATCGTCACGTCGTCGGGGATGTGAGCGAGCGGACCGCCGTCTCCAAAGATGGTCGGGATGTTGTCTGCCATGTCGACGCGTCCAAATCGTCGGTCTGCTGCGCGGGAAGTCGATAAAGCTCCTCAGTCGAGGTTATCGAGGGAGGCTGGACGAACGGATAAGATACGAGGCCTCAGGAGTGTCGTGGGTGAGTACCGGTATTAGGGCCGTTCGTAGACCGGTGGTCGCGCTCTGTAGGCGTTCCTCGCCCTAAACGGCGTTGGTCCGCCCGGGCCCCGATCACAACTTCTGCAGATATGGACTTAGCGTGGGGGATCACGCGGGAGACGCCAAACGCTGCTAGTTCCGCTGCGGCTAATTAGGGCGTATGGGTGCGTCCCCGATGTTGCACG

>AT5385

CGTCATCCGCCTTCTTTCCTCCCCCCACCATCTCCCCACTGTGCAACCCTCTGCCAGTGCCTCCTCCCTCCCTTTTTTTTCTCCGGTGCGCGCCTCGCGCGCCATCGCCAATACGGGTATGTGAAGATGTGGACGCAAGCCCTATGACGTCAGCTAATGCTAGTCACCGCTAGGACCGCACGCGACGGGTGCGCGGAGCTCGATCAGAGGAGCGCGCGGTGGGAAGGAGGGCGTACATTGAAGGGCAGGACGAGCGGGAAGGCACGCGCGACATATGGGTGCGCGCGCGGGGTGTGTGTCGAGCGCGAGCCCGTACGGTCCCGCGCGCGAGATGTTGACGAGCCCGCGG

>AT5487

GTGGTGGTACAGACGTTGAAGCCTTCCCCCGCGCGGGAGTAGAGAAACGTCGAGTTCGGGTGGGTAGAAGCGGCCAGTTCGAGTTCGGTTTTATCCGTGAAGGTGGTCCCCGTTTCCCAGTAGCGTCGATGTAGATCGCTGAAAAGGGGACTGGTTTGGGAGTGTAAGAAGAGGACGCCAGCCGAACGGATCATAGCGACATTATCCTTCGGAGCGGTAGCAGCATTGTGTTTCGACTTGACGTCCATTATTTGTTGGAGAGCTTGGAGTCGATGCAGTTCCGATGGCTGGTAGAAGTCCGCATAGAGCTCCCAATGACGCCGCAGCTCAGCCAACGTGTGGTCGGTGCCCATCTTGATAACACCACCATAAGGCGACGACCGCCAGGCCTCCACGGAAGTGTAGGCCGCGAGCTTTTGGGATTGCGACACGAGAGTCGATCGGGAGTCGATGTCGAGGTACATGTGAAAGAAGATGTTCCACATGGTCGGTCCCGCGACGCGGTCCATGATCATCGTCAACAGGAGGACATTCCTCGCTAT

>AT5635

ACTCACTGTGGCAGCACCAGCAGACTGCTGGGGTGTGTCCTGGGTAGTCGACGATGACGACGACAATGTGGTGGCCTCTGAAGCATCCTGCTTGCGCTGCTTGTTCTGGGAGCGGTACTGCATGCGCTGGGATGCCTTCTCCTTCGCTATTGCACTGGCATCGCATTTGCTGAAGCACTGGTCCTCTGAGTGGTTGGGGCGACCACAGAACGTGCAGGCGACCTTGGTGGCGACGTTGGAGAGTGCAGGGGTCGACAAGGAGATGCCAGGCTGGTTTTCGAGCGAAATGAAGATGTCTTGCAGCTTGTCCAGTGTGAGGGAGTCGTCCAGGAGCAGGGTGTGACGCAGTGACGCGAATTCCGGGGGTAGAGCTCGAATGAGGGACATAAGGACGAGCTCATTGTCGAGCTCGGTGATGGTGAAGTTGGCAGGGCACAGGGCCATCATATCAGACTTGAGCTGGACTGCTCGCAGGATAGGGATTCGTCCTCACTCTTGCAGAGGCTCAGGAGTGCGTTGTAGGTGTTAAACCTCGTACCTGGTCGCTTCTGCATGTGCACAGACTCCAGCTTCGCCCACATAGCGACAGGATCGTCCCTGATGGCCTCCAGGTGCGTCTGCTGCTCCCTCTCCACACAACTCCAAATCTCCCCAGCTGCCAAATCCACCCTGGTGTCGAAATCGTGCAAATCACGTCGTTCTCCTGTGTCTAGGGGTGAAAGCTAGGCCTCGTCTCAGAGCCCTTGACAATGCGAAGCGCACCTAGAATGCGAAGCCTGGCCTCCATGTCGAGTCTCCATTGCTGGTAGTTGCGATTAGAGAGCTTTGCGAAGGTTGCAGCGGCTGGCGCCATGGGGTAGGGGTAGTGACGTGGCGCGCGACGAGCTGGCGAAACAGCGAAGGGATTGGACGAAAACTGCGAGGAGGAGCGAGGAAAGATGGACAGAGTGTCCCAGAGCGAAATGAGAGGCAGGCAGATGATGTGGCAGAGGAATGGG

>AT5664

GCCGCGCTCTGCTCCTTGTCTGCAGCCGGCAGGTCTACCATGGTGTCCAGGATCTGGATGCGCGTGTTCGCGTCGATGTAGATGGACTTGTCTTCCGGCGCGCTGCCATCCAGTCAGTTGCCTCTCTCTCTGCCTCGCGAACAACGGCAATCATGAGACGCACACTTCAGAGAACGCGGCATGGACGGCTGCGCTGCGGACCTTGACAGCAACGGCTGGGTTGAGAGCACGTACGGCGGCCTCGAAGGGCTCGAGCCCGAGGTTCTCGTAGGGGAATAGACGGTACTCGCCGCTATCTACGCGGAGGGCGACGCCGACGGCGATGTTGTCCTCATTGGGCCGAAACCAGGCGTCGCCCTGGGTCTGCTTGAAGATGTGATGGAGGAACGCGTCGTAGCGGTCGTAATCCATGGCTTGCGGAGCGGCAGAGCTCGGGCGCAGGACTACGGGGCGAAGACGGCGCCGGGGACGCGCGCACAAGGACGGGTGTGGAAAAAGGAGAGACTGTTGAGGGAGAGGGGGGAAGGCGGACGGTTTGGAGGCGAAGATGGGGGTAAAGAGGACCAAAGCCGCAGGGGATGGGAGGAGGGCGGACGAGCGG

>AT58

CCCCCGGTCCAAAGACGCACAAATTCATTTTCGAAACGCCTCCGCGAACAACGCCCCGCCCCGCCTCGTACGTATGTCGTCGCGGAAAACAGCTCCCAATACTTACCACTGCCCCCCCAATCACGCCTGGAACACACCCTTGATAGCATCCGCCGCCTGCTCCGCAACCGCATACGCCACACTCTGCGTATGCGCCGCGACGATGAGCGGCACGACGCTCAGATCAACCACGCGCACGTTCTCCGTGCCGTACACCCTCAGCCGGGGGTCCACCACCCCGCCTTTATCCCGGGGCCGCATCGTGCACGACGACGTCGTGTGCGCGGCGATCGCGAGGTGCTTCTTCAGCCACAGCGCGATGTCCGCGTCGGACTGGACGGCCGGCCCCGGGTTCACCTCGCGCGCGAGGAGGGGCGC

>AT6158

CACAGCGTCGTTCAGTCGAGTAATAGCAGGTTTACAAGGGCACAATGCGCGCTCACCAAGCTTTTAGGCCGATCAGGGAGGTGGCGAGCACGTTCGATACGAAAGATAGAAGGGCGGACACCCCTCCGTACGTGTCGGCGTACGAAAGGAGAGGCTGCACCTCAGGGTCCACCCTGAAGTGGATGGTGGACAGTATCCCTAGAACTAACGTATGCATTGGA

>AT6173

CGTCGGGAGAGCGGTAGGCGGGCTTGACCTCGCACTTTGCGTCACAAGGAGAAGATTCACGCTTCGCAAGTCCTCCCCCGGACGCGCGACGCTTCATCTTGTAATAATTAAGGAACAGACACTGCTCGCGCACGTCTGACGAAGCACCCGCAGGGCCACCTCCCGAGTGGCACACCGGGAAGCGATGACCGGCGAACCGCAGGCCCTCGTCCTCAGACGACTCGCGGACGAGCTTTTCCTCCTCGTTGCAGAATTTCTCACCTGCGGACGCACCGAAAGCCCACCGCGATGCCTTCACGGTGCCAGACACGAAGACAAGGTCTTGCTCGTCGAGGCAAAGCTGCAACCGGGCGTTGACGAACTCGAGCCAGGCGTCCATGTGCTCTCGCATGTAGCTCCTGATCCACAGGTTCGAGTGGTTGTTGTCATAGGTTATGGGAGAGTCGATGGCCATGAACGCCCCAGGGCCAGCCACACATTTAAATTTCC

>AT6174

TCATGCTGCCCGTTGACTGAAACGTCTGGGTGGCCCCACGGTACTTGTGGATCGAGAGCTTCGGCCGAGACTCGGACAAGTTCTTGAAGCCTCTGGGTACCCCCCTTCGGTTGATGGGATCGTCCGTAGGCTTCATGACGTTGAACAGGGGGATGAACTGGCCTTCACGGAGATATCCTACGTCGCCCATGAAGATCTCCCGACCACCGGCGAACTCGTTCGGCTCCGGGATCCAGAGAGGATGGCCCAAGTCGCCGCCGTGGAAGTGCTTGGCATAGATGTCCCAGGGTTTTGTCAACATGGGTTACGGGTAGTGAGGGGCGCAGCACACTGTATGTGTTGCAAACCACGGAGGACCGACATTGAATGGTGAAAATGCTCAAGCCTGAGAGACTGAGAGACGGATAGTGGCGAGGACACTGTATATGCATGTGCGGGCAACAGAGGGGGAAATGACG

>AT6193

CGGTCCCGAACTTGGCAAATCCAATACTACGATCGCAACACATTATACAGGATAAAGTAGAGGTATAGAGAAGTTCGTCTCCGGGTATAATCATAATGGTCGTAAATCCTCGCCACCCCGGTCAACGGGAAATGGACGTTCGTCGTTTCGAATGCAGGAGTGCTGTGCATTAGAGCTTGCCCTTCGCGGTCTTGATCCAGCCGGCGTTGGCGTGGGGGTACTGCGAGCGAAACAAGGGAGATGTTGAACATCACACGCGCGCAACAGGCAGAATGGGACTGTGACGCACCTGCCAGACCATGACACCACCCTTCCAGCCCTTGGCATGGGCCTGGTTGACGCAGGAGGCGAGCGTGGCGGGGCTCATGTAGCCGTTGGACGCGTCGGCCGCCGTGGCGGGCTTGCCGATGACGAGCTTATCGAGGGGCACGCCGTTCTTGGGGATCTCGAGGAGCGACGACCCGGGGAAGGCACCGCCCGACTTGGAGATCAGGCTCTCACAGTTTGTGTAGATGCCCTGGTTGTAGAACTGCACGTTGTACTGTAGACGAGATAGCGACCGTCAGCCAAGGGAAACATGGGGCATAGGAAGACGAAGACGTACCCAGTCGATGAGCGAGCCGACGTCCTTGTTGATCTTCACGTAGGCACCGGCCTTGAACTTGTTGCTGGGGGCGAGCCAAGGCGCGAGGGGCGCGTGCGTCAGGATATACTGGCCTTTGGGGAGCGTCTTGCGGAGGGTCTGGGTGAAGCTCGTGACCCACTTCTCGGCCTTGCCGTCGCCCTTGTTCATGGCGGTGAGGTCCTCGTAGTCGACGTCAATGCCGTCCAGGCCGTTGCTCTTCACCCACTTGGCCATGGTGTTCGCAGCGCTGACGGCGTTCGCGCCCGAGGTCGTGGGCGTGTCAGTCGCGCCGAACGCGGAGGCGATGATCTTGATGCCCGCCTTGTGGTACTCGTTCTTCTTCGCAGTCTTCGCCGAGGCAGAGAGGGACGCCCAGTTGCTGGCCTGATCGACTGCGCCGTGGAGGGTGAGGAACGACAGCGCAACGACGTTGTAGCCCTAA

>AT6215

CTCAGGCTCGGCGTTAATATTATAGAGCGGACAATGACTGGCTGAGACGCTTCTGGAGACAGAGACTCGAGTTCGAGGTCATGGACTGCACTACGATGGCGGTGATGTTGTCCTGAGTCATAGACCCAACAACAGCGACTCGAGCCTCGGTATCAACACTCAGGATGTTCGAGATGTGCGACAGGGCCTTGGAGAGACCGTCACGGGCTGGCCCCTGATTGGATTGCGGGGGCTAGTTGTCCGCACCGAAAATCAGAGGAAGCTCTCTAATTTCGCTCGCGAGAGCCGCCAATTCC

>AT6228

ATCCTGACTCCTATTTTCAAGTTGCACGAGTAAGAGACAGTACGAAGCTCATTCGTGCTAGAGTGACTTCGACTCCGGAGCATCATCACGTTCATACACAAGCTCAGTGTCGAAACGCTTGCAACAGCGGCTCAACTCCACGAACGCCTCGGGATCCGGGGTGCCCGAGCGGACGATGATCCGCGGCGGCTTCGCACCGCTCGGAACGAGCCCCTCCTCCAACGCCGAGGACAACCCCCACGATTTCATCTCTCTCTTGGACACGGCGACTCCCTGG

>AT6229

CGGTGTTCGGGCACACCCCGAGGATGGCCGCTACGAACGTCGTCGGCGTCGTGCCGGACAACGCGAGGTGGCGGACGTTGTGCGCCCGTTCGTTCGGCCATGTTCCAGCCCTGGGAAGGAATCGCCGAAATATCGCGGGAACCGTGGATGGGTAGCTGAGAGAGAGGTGGGTGACCTGCGCCGGGAAGCTCCCGCGCATGATTTGGCGGAGGATGTGGTCATATTCCCGGTGCGTGTACTCGAGAGAGACGTGCCGAAGGTTGGGAAGGGTGCCAACGGTGTATATACCGTCGCGACCACGTGCAGGACGTCAAATGCCGAAACCATCTCGCTGGTCGAAGTACTCCGGTCTATATATACCTAGTTGTAGTACCTACATGGTGAGGGAGTGACAAATTTGCCTTATAACTTGATGTGCGGCCATTCTGAACAGGTCGACCGTCCCATATCTGACTATAGGTACGCGTTCGGGGAGGAGCCAGAGGAAGGTAGATCCGCTGCGGTTGTTAGAGACGTAGACGTCGCGAGAAGTAATGCGGGCGACGAGTGTGAGCCAGGTGCGTGAGACCAATGCCAAGTCCGTCTTGAAGACGGATCGCTCCTGCGGAGTTTGTGGTACTTCCCAGAGCTTGTTGAGGATGAGAAAGATGATTTCTGGAGGAACCATGGGGCGCACGGCTCGGTTGAGGAAGCCGGGATCGCGAGAAGTTGCGTGGATTCTGTGGAGGAAGACATTTCTATGGTCGTTGGCGGGACTAACACCTAGGCACGACCGAAGAACTTGTAGCTCTTTATATACATGATATGTCGGGACCGTACAGCCCTGTTAATGCGCCCAGGCTCATACGTGAACGTACCCTTCGAGTTGGGCCCAGC

>AT6246

CACCAGTCGGAGGCTCAGCCTTGCAGTGCGACGCGCCCACCCCGCACAATCCGAGTCGCCCGTCCCCGACAGGCGACTGTACCGGAAAGGCGTTGGCTCGTCCACCTCTTCGCCGGCACGGTGACGTGATTCGCGACGAGGGTGGGAGCCTGAGTGGGGCGCGGCGCGAACGTCGACAGCCAACAGGTGGGCTTACGATCGACGAGGGGCGGCTGACACCTTGTGCAATGCGCGTGCT

>AT6272

TTCCGCAGAAAGAGTGCTCAGGATTTGTTGGTCGGTACTATACATCTAGGAGAAAGCGCTGACATTCGTAACCTACCACCTCGGAGTCAACATGACGGGAAGCGCGGGTCGTGCGGCCACGAAGTGATCCACAAGAAACCTCGTGTCGTACTCTCGCGAATCCCATCCCTCCTTCAATTGGAACCGGAAGCGTTGCATTAGGGCGCACACGACCGTTTTCATCTCCCGCATCGCGAACCCTTTCCCGACACAGTTCATGGGCCCATAAGCGAAACTGAGGAACGCTACGTCGTTGTGCGTGAACGTGAACTTGTCGTGATCGCGCTTAGAGGACGTTGGTAAGGGGACCTTGCCGTCCAGAGCCAGGTGTCCCGACGCGACGAGCCAGCGCTCGGGCCAGAACTCGGCGGGGCAGGAGAAATTGCGGGGATCGCGCTGGATGACCCACGGTGAGAAGTAGACTAGCGACCCTGG

>AT6411

TGGCAGGATCTCCACCGAACGCGCTGATATGTTTTTGCACCCACGAGATGGCGGCGAGCTGGTCCTTCAACCCGATGTTCACATCATCAAGCTGCCCCGCCTCTATTCCTTGTGGAAACCCGAACGGCCCGACGCGGTAATTGAAACTGACATAGATGACTGGTGTTCCCTAGGTCACATTCAATAGAAGTAGCAGCGATATTTTGTGGATAATGGAACCTACCCTAAGCACACTCTGCATGATGATCTCATTAGCATTGAAGCTCGATGAGGTGCCCTTACGGAAACCCCCTCTGTAAATCCACGTCTTATTGGAGAGCACT

>AT6424

GGGCGGGGAGAACTTTGTATTCGGTAAGAACGCCGTCGATCGCGCCTCCGAGACTACTCCCCTTGCTCTGCGCGGTGACGTCTCCGAAGACGTGGTCGATAGCGAAGTTGGAGCAGACGCGCTCGCCGACCTTCCAGTGCTTCACGGCCTCGCCCACGGCAATGATTTCGCCGGCCATGTCGGACCCGGGTACCGGGTTCTCCTTTTGGAGGAAGGGGAACTCGCCCTTCGCGACGAGAAGGTCGCGGTACTAGCCGGTCGGGTACGAACATCAGGGCGTACGCTCAAGGGAGGAACGATAGGAGGAAGCGTTAACGGACCTGAAGGGAGACCGCGTGAACTTTGACAAGGACCTCGGTGGCCTTGAGCTTGGGGATGGACGCTTTCACCAACGTGAGGTTGTGGAAGCCGTCCACCTTGAATGAAGGTGAACAGCCGTTAGCGTATGGGATGCGGGAAATTGGGCCTCGTTGGTCATACCTTCGGGAGCCGATATTCGCGAGTAGTGGATGGAATATCCATTATGGAGTGATTGGGTCAATGGAGTGCTGAGCGTGGCGATCTACGGGTGGTCTCGTTTTATATCCTGTTTAGCATATACTCCTTGCATGATCGACTAGCATGCGCTCCTCCCCCTCGATGGCCCTCCCCCTCGATGGCACTAGCGGTAATCTGGAAACCACATAGGGTGCCCCAAAGGCGAGATCATAGCCCACGGCGAGCCTAATAAACCCTGTCATGAAGACGCTTCTCAGGCTTTAGCGCCACGGTTGACAACTAACAGACAACCTACCTCCTATATACTTCTCCTGGGTGGAGGTGAGGTGGGGAAGATTTTGGTGCGAAGGGCCTCGAGCTAGTAGTACTGAGCATGCGACGCATACGACGTTTGCCCAGTAGCTTGGCCAGCCCAGCTACTATTTCAGTGGATCCGTCCCCAGTCCGAGACTGAGATCTGTCTTCCATCGAAGTTCATTGCTGTCCTTGATAAGAACGCCTCGGGATATGGATTCAGGAGGGCGAGCATGCCATTGCCAGTGCGTGGATAGAGAGGACGACGGTCAAGGTGAAAGCTTGC

>AT6591

CTTGCAGTGCAACGCGCCCACTCCGCACAATCCGAGTCGCCCGTCCCCGACAGGCGACTGTACCGGAAAGGCATTGGCTCGTCCACCTCTTCGCCGGCACAATGAAGTGATTCGCGACGAGGGTGGGAGCCTGAATGGAACGCGCCGCGAACGTCGACAGCCACGATGTGGGCTTACGATCGACGAGGGGCGGCTGACACCTTGTGCGATGCGCGTGCTGGGATCAAGGAGGTGAGTCTTGGTCTTCACCTCGAGTTCGGAACTGATTGGACGTACATGGTGGTGGGTCAGCCATGACCAGTGACGCACCGTCCTCAGGATGGGGAACGCGCGATGGAGAAGAAAGAAGACAGGAGAAGAGAAGACTATGGGCCACAACAATCAGCGAAAACACCTCTGAAAGGAAGGGAGCAAACACACCGAGAAAGGGTGGTG

>AT6821

GGCGTACGGGAGCCCCGCCGGGGTGCTGGTTAATGGTGTATACCAGACTCTAGAAATACTACTACAGTTCTCGTTCTCGGGACGGATGTCGTATAGGTCGACACCACGGAGGCGAAGCCACTCCGTCGACGTTTCCCAAAACGCGAGCATTTTGTCTATTGTGGGAAAGTAGACCTTGCGAAAGTCGTCGATAGCGCATGGTAGCTGAAACACCGAGTGTTAGATATAGGTTATGATACTGTCTTTTTTGGCCCACTTACTTCCGGGATTGCTGGGGATGTCTGCATGTCTTGCTTCAGGATAGCGTCGCTCATCGTTGCGAGATGTGCAATTGCTGGTGGGAGCGTGTTGTGGCAATCCAC

>AT6830

TCGTTGTGGTCACGCACGTCGCACGTCGACATCCCCACTGTGAGTACTGCAACAATAGCTATCGACTTCTGGCCTTATGACCGTCAGAACATAAACATATAGATATCGCCCGACCTCTGCCCTTCAACCGCTCTCGAGCCAACTTGAAGCTCGACGAATCGGACGCCGGCTTATAGGCCCGCCTCGCTACGAAACCCCGATAGGCTTGACACCCTGGCGACACGCGTGTACTTGTAGCCCTGTAGACCATCTGTTCTCAGCGGCGGTGCTTGGAACCTTCTTCAACCCTGGTGAGACCCCAACTTGCAGTACTCACCTCCGATCCCCGACGCAGAGTCGACAACGCAACGCAAGCGCAGCCGAACGCTCAATGGCCTGTCTGCCACCCAATCTCAGCGGTAGTCCTTGGAACCCTCTTGAACGCTGGTGAGACCCTATCTTGCAGTACTCACCTATGACTGAAGCCGTTGACGATGCGACCGGCTAGGACCCAGTGAACACCCGTTCTCAGCGGCGGCCCTTCGGCGATCTTGAACTGCTGGTGAGCCCTCAACTTGCAGTACTCACCTCTAACCCGGACCCAACGCAGCACAGAGATAGCAATATCAAGCTCAGCTGAACGCCGAATGACCTGTGTATGTTCTCAGCCGCAGTGCTTAGATCCCTCGTCTTCCGGTACTCACCAGCTTCTCATCCCAGGCCACGCACAGCCGTTGCCACCGTTGGGACGATCTGTGTGGTGTCTTGCGTCAGCGGCGGCCTCTCGGCGATCTTGAACCGCTGGTGAGCCCTCAACTTGCAGTACTCACCTCTGACTTGGACTGAACGCCACGAACTGGCAGAGACAGCAATGCCAAGCTCAGCTAAACGCCGAATGACCTGCGGTTTCTGTTCTCAGCGGCAGTGCTTGAAACCTTCTTGAACGCTGTCGAGAACCATGTTGCAGTACTCACCAGCTTCTCGTCCAGGGCCTTGCGTGGAGGTGATCGATGGCATGACGATGACCTATGCGGCGTCTGTCCCGGGCCTCAGCGGCAGCCTCTCGGCGATCTTGAACCACTGGTGAGACCTGAACTTGCAGTACTCACCTCCGTATTTGGATTCCCGTGCGTCTTCGGCGTGGGTGGGGATGTCGACGGGCGCGAGCCGACGTGGACGATGCTAGCAGGGCCGCCGTCGCGGCGAAAGTATAAGGCGAAGAGGTAGCGGAGGCTGAGCGGGCTCGAGTTGGGAGGAGGGTGTAGATGTGAGGATACAACCGGGGAGAAGGTGGTGGTTGATGGTGAACGTTTGGCCGCGAGGTGGGCTGTATTTATACTCTTGGATTTGGCGGCTGGCACGGCGACTTCTGTTTGTTCAACTGTTTGGTCAATGTTCGATGGCACGCCAGCCTTCACACCGGCAGGGGCTGACTTGTCAGCTCCGGCGGCCGACTTGCCAGCTCTGCGGGAGGCTGCACTGCCAGCTCTGT

>AT6832

AGCAGCATAAGAAAAGTGTGTGCTTACCGACAAATACACTCGAAACATATTTCCGAGCATCCCGACGTCGAAGGCGAACGGGTTGTAGTGTGGTTCCCCAAGCCACACGTCCAGCGGCTTGAAAAGATCCGAACCCATGCATGCCTCGTCGGCTGGACGACGACAATCCTTGAGGGAGACGTCGAGTGGTAGCTGTATCGACTGATCGTAGTCCATGAAGGCATAGACAGCATCAGCCCTGCC

>AT6851

TCGGGCTCCGCTGGCGCTGGCGCTGGAGCTTCAGCGCTGGTAGTGCTGGGTGGTTGGGCCTCGGCGCGGACTGCCTCTTCTTTGGCGCGCCTCTCGCGCTCCGCCTGCTCCTTCTCCAAGCGGTACCGGGCGCGCGCCCTGTCGAGGGGCCGGAGCTCCTCGTCGGGCTTCGCGAGGACCTCGACTCGTCCCTCTGCCGCAGTCGCGACCGGAGCAGAGGCTTCTACGGCCTCAGGTTGTGGTGGTTGGGCCACGGGTTCAGGCTCGGGATCGGGCGCGGGCCTGGGCTTGGGCTCGGCCTTCGACGCGCGAGCTGCCCTGCGACCTCTGCTGGATGCCGGCCGTGGCGGCGTCTTGACGTGCCCGAGGGTCCTGCGACGAATCTTCTCCATCTTCACGGCAGTCTTATCCCCCTGCAGCTTCGCGC

>AT6867

CGCGTCCCCTCGAGAGAACGAAGTCCCAGCCATCCGAGACGCTGACTAGGTGTGTTTCCAATCCACCTCGGTCCCTACGCCCCGCTGCTTCGTCTCCTGCCCCGCATGTGTCCCACCCTACCATGGAGTCCTTGACTTTCCTGCCAGTATATGTTGCGCCTACATGCATGCATGCATGCATGCTTGCTCTCATACGCAGGTTCGATATCTTCCTGCATCCCCCAGAGCACAGTTCGTCCTAATCGAATACCTACACTGAATACAACTGCCCTAGTATGATTAGCACCAACCGGAAAGGACCGCACCAGCCCACGCCCAGCCCTCAGCCCTCAGCAAGGACCGTACTTCGCCGCGCAGACGTGGGCAGCAAGCCCCGCCGTCTGCGTCTCCGCCGTGAACAGCGACCCGCCATCGTTCGGCGCGTACCAAACGTTGCCCACCGTCCAGTCCGACCCCGTCGCGTAGTGGACCACGGCCGACTCCTGCGCGCCGATGAAGTCCGTGTACTTGGACACGCGGTCGGGCGCGCGGTCGAGGTAGAACTGGAGGTGCTTCATCCAGAGGCCCTTGAACATCGCCTGGTCGTTGTCGCACGACGACGACGACGACGTCGGGCCCGCGTCGTCGCACGACTCCTTGAGGATGCCGCTCTCCGTCTTGAGCGAGATCGCGGCGTCGAGCGTGATCTCCGCTTGGGTGAGCAGGCTGGAGTCGCCCGTCGCGAGGTAGAGCTCGGCGAGGCCCGAGGCGACGACACCTTGGTTGTACGTCCAAGTCTCCTGGCCGTTGTTCTGGCAGGAGCT

>AT6868

GGCGCAGGACAATTGGCCATCAACGCACGACATATGCGGACGCATCCTGACCGTGCGTATTCCTGTAATCGGACACCTTCAACAGAGCGAGCACCACCCACTATAGAAGTTCGGGGTGTCAAATCACAGCCATTGTGCATAATGCCAGCGGACCACCTACGCCTGCGTCGTCGTTGCTGCCGTTTAGCTGTGAGATCCAAGCGTTTTGGTCTTGCCCGAGCGCGAGCTGGCCGATGGTCGTGGAGTCCGCGACGTCGAGGTCCGTCGAGCCTTCCGCCAACATGAGGTTGTAGAAGTCCTCGATCGTGTTCTGCATCGCGTGCACTCGTCAGCTGCGAGTCCCCCCAGGATGAAAGGATACGCACGGCGTCGGTCCAGAAGATGCCCCATCCTCCCCCATCCCAATACGCACTCTGAAGGCGAGTCGCGACGCTCTAACGGTGGGGCGATGCAAGCGTGGCAGGTGGAACGGGTCGTTTGGGGTTAGTCACTCTCATATGCACCGATCGCGGCCAAAGGACACTCACGGAGGCAGTACTCAGCGTCGTAGCACACTGCGCGCGGGCCACGAGCGGTGTCGGGGATGCGGTAGCAGCGGTCGGGTACGTGCTAAGCGTGGAGAGCACGGTGACCAGAGTGGTCATAGACACGAGCTTGTGGAAGTTGAACAACATCTTGACAAAGGATCGGGCAGTTGCGGGATAACCTGGTACCGTGGTGGGCGAGGATAAGGGCGGACGGCGTCGACATTCTGACAACGTATGATGTCCGTTTAAGTACCACAGCGTTGTCCACTTCTTGATAACAGTATATGCGGTCTCTGAGAGAACGTAATGTCTTCCGAGGCGCGCCGGATTAGGTGGTCATGGTTCGAATCTGTCCATGTGGGAGCACTGCACATGAGTCCACACATGATCGTTGCTTCGATGCGGACGGAGTGTATT

>AT6894

AACCGTGCGATCCGGTTGTCGAGGCTCCATCTCCGACTGATCCAGGCGCTTCGACAGCTCCCAGTCGGTCAGGAGGACGTCCCTGGTCCGTGAGAGTTTGTCGTCCTTGACAATCTCCTTGACCTGGATGAGAACGTTGCCGGCGCTGATATCGCGATGTAGAATGCCGGCCTTGTAAGCTTGATCATGCGCTGATGAATAGTGCTCAGGTTTAGGATCTGAGAACCTCGGGTGCGTGACAGTGAGGGCGACTTACCTGAGATACCCCTCGCTATAAGGTACACGAGTTCCTTGGCGTTCTTGAAGTAGCTCATAGGAAGAGCGACTTCCCGTACAACGATGCGGTAGTGGCGATGAGTCTTCATTGGGCATTGCGCTTCAGCCTTGCGAGTGGTCCAGTACTTCTGTGAGACCGTCTGCTGGACTCTTCCCCGCGGCTTCTCCCTGTCGTTCCCTACCGTGTCCTCGTCTTCAGCGTCCGCCTCCTCGCCGCTCTCGTCTTCCTCGTCCTCCTGCCCCTCCTCGCCGTGACCTTGCTCGGGATCGAGGTCTTCCTCGATTAATCTGTCGTCCTTCCGCGTTCTCACGTCCCCATGACATACCAGCGTCGGCACACGGTCAACTTTCTTCTCGTTGAGGTAACGGAGGATGTTGCCTTCGCGCTCGATGCCCTCGTGAGCCACCCGCCAGGCGTCTTTGACGAAGACGAGTGCGCCACGGGGGTCCGCGAGGTCCACAGCCACATAGCCACGGGTGGCGCGGCCAGCGAGACCCGACGCCTGAAAGTGTGGCTCGCCGACGAGGAAGAATCGCGGACCCGCCGCGTCATCCACCCGCAGCTTCCACCATTGCGCGGAAGAGTTGCTGACGGACGCCTTGAAGAGCAGCCGCGCGTGCTCGTTGACGATGTGGCCCTGCGC

>AT6916

CACGCTGATCGCGCGCGCGACGCCCTGGTCCTTCTTGACGTCCTGGCCCTTCTGCACCTCCGCGCCGGCGTCCGCGTCGACGACCTTCATGCGCATGTCCTTCTCGCGGCGGCCCAGCTGCGGCGCGTCGACGGTGATGAACAGCCCCTTCACGCCGCGCGCCTCCGCGTGCTGCACGTACTTGCGCGTGATCTCCCGGTCCCGGTTCACGTATAGCTGTAGGAAGAACGTCTGGCCCGGCGCCGCCGCGTCCAGGATGTCGTCGAATGCGCATGATGCGAGCGTCGC

>AT6935

GTCCCGTGGTACGATGCATCTGTCTCCGAACAGATGCCCTTGTACATAAATTCTCGAGATGCTACAGAACCTTTTGTACGACTCGAGTGTTGGCTCAGGCTTCGCTCCCCCACTTGTACTAACTTAGAACTCAGACTCTGGGACCAAAACATACCCAGGTGAATGCCCGACGATACAACATATACAGAGTACACACTTGGTTCACCTATATCGCACCTGCAGCGGCGAGGCTGACCTCCGATTCAGTCGGGACAGTCAAAGTAGACTGCTTAACCGGCACCGGCATCCCCCTCTCCTGCAACAGCTCTCTGAACTGCCGCACGTCG

>AT6984

TCCACGGCGTCCGCCTCGCCCACTCATCCCATTGCATCCCGAACTCACTCCTCAACATCGCGTCCTCGTGGCGTGCCCTCGCGCAGAGTGTGAAACTAAGCCACGTCAGGTACGCCGAGAGTGTGTACCCGACCACCCGTCCTGCCAAGGTGTTCCACAGCCCCGCTTCGACGAAGTAGGACCCCTTGCTGGCGAGGTAGAGAATATTCCCGCAGGTAATGAACACGAAGCCCGTGTAGCTCGGATGGCGGACGACGGAATACAGCCCGCTTGTAACCAGCTTGTGATCCTCGCGGACGGACATCTCCCAAGTGAAGAGGCGTCCCAGTGCACGGTGACACCACATGCGCATGAGCCCTCCTGCGAATCCGAGGACGGTCCCGATGGCCGACATAGGAGTGAGTCTGATAGTGGGACTCCCGAGAACGTCGAGGACGCGAGTGGAGTATGGTGATGGGAAACGCTGAGCGAG

>AT711

ATCCGTCGCGCGCACACCCATATGTTGCGCGCGCCTTCCCGGTCGTCCTCCGCGCGCTCCCTCCCACCGCGCGCTCCTGCGATCGAGCTCTGCGCGCCCATCACATGCGGTTCTAACGATGACGACGACGATTAGTTGACGTCATAGGGCGGGTGTCCGCAACTTCACATACCTGTATTACGCGCGAGGCGCGGGCCTGAGAAAAAACAATAGGGACGGAGGAGGTAGAGGGCAGTGTGGATTGCACAGTAGCGAGATGGTGGGGGGAGGAAAGAAGGTGCATGACGAAGGGCGACCATGTTGGCCCATCTTATATATGCCGGGGACTCCGATGGTGTCGCGTTGGGGGCGGGATCGGGGGACAGTCTAAAATGGACATGTCGGGCGCGCGTTGGGGGCGGGATCGGGGGAGAGCGAAAATGGACATGTCGGGCTGGGGCTAGTGGAGGGGCGGATGGAAGAACTACTAAT

>AT7219

GCCCTGGGCCGAGCTCCCGGCATCTCCGTCCGCGCCCTCGTGTCCGAGCTACTCAGATCCGGGCTCCGGTTTGGGATCGCGAATGCGGAGATCGCCCCATAGGCCAGCGCCGAACGCGCGGCGCGCGTTGAGGGACAGAAGGATCGCGGCGGCGACGAACTCGGGCACGGCGTGCAGCACGTAGAACGTCGCCTTTTCGCGCGTGGAGTTCTGTGCCCCGGGCGCTTCCGAGAAGAGAGACGTCGTGGAGCGGCCGAAGATGGTTAAACGGTAGATGCCGACGATGGACTAGAGAGATAGAGAGGAGTGGCATGCAGGAAAGGGCAAAGGAGGCGAAGGTATTCAGGACGAAGTTGCCGCACGAGGGGAGGAGAGCGAGAAGGACGACGTACGAGGAGGCACGCTACGAGGATGATCCACAGCAGGGACGCACGCGGGACGCGGGGC

>AT7340

GGGACCCTCGATTTGCTGCAGAATAGTACGTCGTATTCCGGGTCGTACTCCGGCTGCGGTGAGGTGTCCTGGCGCCGCTTCTTCGCGAGGACGGCCGCATTCAAGTCGTCCGTCAGGAAACGGTCCAGCCTTGCCTGCTGACTCCCGGCCAAGCGGACGAGCCGCGGGTCGTGCAAGCTTGGAAACTCCCCGCTCGATACTCGGAGAAAGACGTCCACGTAGGGTCCAGTGATAGCAGTTCGAGAACGGGAACGGCGTCTTCGTGCGGACGGGTGGGCGGTCGTTTGGGTGTGTGGTGTTCGAGAAGATGGGGGTGCACATGTCAGTGGTTAACCAGTTCTCGGGGTCTTCGGCACCTTGGCTCAGACCAACGATGTACGTCTCGAACAGCGTGGATATACCGGGGAGGGGGAATCTGGGAGCCTTTGGGTGCGTGGTCAGAGTTGCGGGTCTCGACCACCGAGTGGCGACGACTTCACTCACGTCGTAGAGATATAGGATGCACTTTGCGGGCTTGATGGTTTGCGAAGCTTCCTGGGATTGCGGGTCCAGGTTTTGAACCGTCGCCTGTATGTGGAGCTCGGCGACAAAATATGTGTGCGGAACAGGGCAGTACAGTGTTTCGTCCATGTTGGTGGTCGACGAG

>AT7379

GACCCATCCGTCGCGCGCACACCAATATGTCGCGCGCGCCTTCCCGCTTGTCCTGCCCATTCAATCCGCGCGCTCCCTCCCACCGTCCGCTCCCTCCCACCGCTCCCTCCCACCGCGCGCTCCTGTGATCGAGCTCCGCGCGCCCGTCACATGCGGTTCTAACGACGACGACGATTAGTTGACGTCATAGGGCGGGTGTCCGCACATACCTGTATTACGCGCGAGCGCGCGTCGGAGAAAAAACAATGGGGACGGAGGAGGTAGAGGGCGGTGTGGCTTGCACAGTGGGGAGATGGTGGGGGGAGGAAAGAAGGCGGATCACGAAGGGCGACCATGTTGGCCCATCTTATATATGC

>AT7444

CCCTAGTGCGAATCCGAACGCGGGGTCGACCCACCGCGCCGCGAAGTGCGGAAACGTTCCGGAGATGGGTGCGTGCGCGGTCATCTCGCCCACAGCGCAGAGGGATGCGTACGCGGTCGTCCCAACGAAGGCGTAGGCGATGAGAGCACCGAGGGGTCCAGCTGCTTGAAGCGACTGACCGGAACCCAAGAATAGACCTGTACCAAGCGTACCAGCGATCTAGTCCGGTTCAGCACAGCGGAAGAAATGGCGAGACACGGAAATGGGAACGTGGGTGACATGTATGTAGAACGAGGGACTCACAGCAATCATCTGAACATGGCGCTGCTTGAGTTTGCGTTGGACGCGGTCGAGGTCGGCGGGATCGAAGTGGTAGCGGTCGTCGGTGGGTTGAGCGGCATAGTCGCAGATGGCGCCTTTCTCGAGGGAGCTCGTTCCGTCGGGATGGTGCTCGCGTGAGTCGGCCATGGCGAACGAGGGGTTGGAGGAGAGGCGGAAAGGCTAGCTGGGGTGCGAGAGAGGGAGACCCAGAGGGATGCGCAACGCCGAACGCACGGAGAGGGCCACCGAGCCTCAAGTGGGGAGCGGAGGGGAGCGACTCACGAAGAGGAAGACTGTGGGGCGTGGAGTGTATATACTATATAGCATATAACCCCGCACAGACGCGTTTCGAGATTCCCGCCCAACAGAATATCAAAACAGGCCCCAGGCGAGGCGCCCCCGCACGGCTACTTAGC

>AT74

TGGTGCAAAATTAATACCTAGTGGTAGAAGTATATGTAGATGCTGTGAACGAACGCCGGAAGAAAACGAATGCAAAGGGAGTCCGTTGTGCGGCGTTGGTAGAAGACGAAAAACGCTATGTAAGCTAGTATAGGGTGGACCGACAACACCAGAAAGACAAGCCGGAAGATCTCAAGCCCGAGATGACCCTGACCTTGCAGCATCCACCAAGTATCTCCTAGACGATCGAGGACAAGCCATTCAGCGACGAGGTCGGGCACGAGAATCCCTTAGTGCTCTTCGGCGCTATCCTCCTGCGCTCTGGCACAGCCGCTTGAGACCCGGGGAGGCTCGGGCTGAGGCGACGGTAGCCGTTCCTCCTGTACCGCCCGAGCGGCGAGAGGGCCCGGGGGACATAATGTCCGCTGCCCCCAACGCCGTAGCCCCTCCTCTGCACGCTGGCCCCGAAGCACTTCTTGAACAGATCGAACGACTTGCTGAACAGCGACACCTTGTCGCCCGCCTTGATGATCCCGAGCAGGTCCGCGTCCGCCCCCGCATTGACCGCCAGCCCCGCGAGGACGTCCACGCACCCGCCCACGCCCGCGCCGGCGCTCGTGCTCGTCGAGTTGGTGCCGTTTGTGGACACGGACGCGTTCGCCGCCGCGGTCAGCGAGAGGTCGAGCGTGGCGGAGGTGTCGAGCTCGAGGAAGATGTCGGCGGTCTGCTTCAGAATCGTCGCCCCGAACTCGAACGTGGGCTTCAAGTGCGCGGCGAGCTGCGCGT

>AT7504

TCAATGGCAGTCCAGACATCCTCATGCTTCCCCAGATCGATACAGAGGGGTGAAATCTTCGACAATGGGAGGAGAGCGCTGAATGTCTTGAGACTCCGCACGGAGACGTGGCTCCAGAAGTGCGGCGTACCCAGCGCGACATCACGCCAGCGTCGGCAGACGTGCGCGAGCCTGATCCATGAGGGACGCGTCCAGACAATAGATTTCAGGTTATCGTTAGTCACTGAATGGTAATGATCTCGGGCGTAGACCACTAGAATTTGTGCAAGTATCTCGTTGGGAGTCGGTTGATATGGGTGAAAGCATTAAGATGGGTCTTGAGGTCAAGGATATTCCTCTGATAGACGTAGATATCTCCCAGAATTGTACTGCGTTCCCCTGCAAACCTGGTGCTCGACTCGAAGGTCTGATCACCGTGTACTGGCGACTGCATCGAAGCTGAGGGTGAAATCTGCGACAAGGAATGATCCCTGAATCTTACAGACTCCCATAAATGCGGCTTACCTATGAGCTCCGGCTTGTCGAGATTGCCCCTAATGGCGGATGAGCTGACGGCGGTCGAGGTGCAAGTTGACTCCTTCAAGCTTTCAAGCTCAAGTTCGTGCCGCGGCGCACTGCTTTTGCTTGTTACTCGCTGGTGTGTGACACTACTGTGTCACGACTTGAATTTGCGTGGCGCCGTCACCACCCGCTTGGCCAGCACGACAAT

>AT7554

CTCACAAAGATGCCCATGACCATCCCGATCCAAGGTATTGACGGCGACGTCGTCCAAGCGTAGATAAACATACCCGCTGGGAAGAGCAACGCCGCGAGACACGCGCTGAAGAGACGGGCTTCCGGTCCTTTCTTAGAGTGGTATTTCCTGGATGGAAGGGTTATAACGTGTCGTGCACGGATACGAGTGTGACTGTGCAGTCCTTACGCGTAAAGCCTTTCCTGGTACA

>AT7620

TGGGACTTGCGGAAAATTCATAGCTTCATAATACATGGCGTGGCGTACGGATTAAGAGGCACATACGAGAGGTAATACACTGTACAAAACCCGACAAAATAAGAGACAAGGGGAGACGAACAGACAACACAGCAAACGGTTTACACCTCTTGCGAGGCGCCCTCCTTCTTGCCCTTGTTGGATTTTGAGGGGAGAAGCTCGGGGTTGATGTGAGGAACGACACCACCCTGGGAGATAACGACATCACCGAGCAGCTTGTTCAACCTAAGATGGATGGTATGAGTTGGGTATGTACAGAAAGCGTGGAGATGGTGCACATACTCTTCATCGTTACGGATAGCGAGCTGAAGATGACGGGGGACGATACGCTGCTTCTTGTTATCACGCGCGGCGTTGCCCGCAAGCTCCAAAATCTCGGCAGCAAGATACTCGAGGACGGCAGCCAGGTAAACTTTAGGAAAGAGACATTAAGTTCGTTATCAGTCACACAGCTAGAGCCCTGAATAAGAAGCAGACGCACCTGGGGCACCAGCACCAACACGCTGAGCGTAGTTGCCCTTCTTCAAGAGACGATGGACACGACCGACGGGGAACTGGAGACCGGCCTTCGCGGAACGGGACTGGGACTTGCCAGCCTCACCGCCAGCCTTGCCAGAAGAGGACTTGCCACTGTAATAGGTGCGGTGTGAGTATCCGAC

>AT8032

GTATTGCAATGATGAGGCGCGTGCTCGTCCCCGACGAGCTTCCGGTGATTGAATCCACAAGACAAACGACCATATAACGATATGAATAACATGCGCGATAGCAGATAGAAATCATGATGTCATGAGTGATCTCAACCTCGCCGCCCCGAGTGCCGAGAGTCAAGAGCAAACCGATCTGTCGGGCGGCGGTCGGGGGTCCGGGCGACGGTGCGTCACAAAGATAGCTTATTCCGGGAAGGCTTGGGATCTAACGGTCGCGATCCAGGAAGCGGCCGCGTCGGGGAACTGTGGAGCGGGCGGGAGAAGCGTCAGCAGCAGGCTTCCCTAAGGCAGCGGAAAACACGCACCTGCCAGACCATGACACCTGCGTCTGCGGAGAAGAGGGCGGGGCGTCAGTAAGCCGAACCACGCGTGAGAGATAAAGCAGTCAGCAGGAGGACGCACTCCAACCGCCGTTCTTGGCCGTGGCGAGGCAGCCCGCGAGGGTCGAGGGGTCGATGTAGCCGTTGTTGGCGTCCCCAGCCGAGAGGGCGGGCTTGCCGATCACGAGCTTGTCGAGCGCGACGCCGGAAGCGTGAATCTCGAAGAGCGACGAGCTCGGCCAGGTGCCGCCGGAGGTGTTGAGCAGGCTGTCGCACGTCGTGTACTCGGTAGTTCC

>AT8157

GCGTGACGCCCAGGCTGACGTGACTGAGGCTCTTCCGGTGTGCCATCTTGGCCCGCTTGCTGTCTGAGGTCTCCCTGGTGAGGCCGGATACGCGAACCACCTCTGGCAGACTGCTACTACCACCCGCAATCATAGAATCTACCACCAAGCGCACAGCTTGACCGCCGGAAGTATAAGGCGAAGGCGTTTCGTGCAGCGGGAGTTTGTTCATCCGTGTAGGTGGGGAGGGATCCGACAACTTGCGAATGCGTCCCAGGAATGATGATCGGCGAAGCCTATTCGTGATGCTGTCCTTGCGGCCCCCGAGGCCCGCCCCGCTACGCCGTCTGCTGTCAATGCTAAAGCGTTGCTCCGGCTCAGAACCTGGGTGGACGGGAAGGGGACGCGGAGGAGGTACTGGAAGAGGAGGAGGCGAGTCAGGCTCTGGCTTGCCCTGCAGCATCGGAAAGTCGGCGCTGCGGATTTGGGAAACCTCACGTTCGATGAGTCCTCCAGACTCTATGGTCGCGTGCCCCCCCCTCGATGACTGCGTGGTCTAGCGGGGACGATCGCAGGTCGGCTTCTTCGAACACGTCCTTCGAGCCCGAGGGAGGGGGAAGTTCGGAGACGAACGTATGCGGGGAAAGTGATTCCGAGCGAGCACGAACGCTTACGCTGCTCGTGGTGAGGGTGTCGTCTGGAGTCATGGGGGGAGTGGTGGGGCCCGAGTGAACCGACGTCTGAAGGTGCGAGTGGACATGAGAGTCGACGGTAGACGCGGTCGGGGGTGCATCCGCCTGGCCATAGACATGCCCATGAGACTGACGAT

>AT8177

GGCGGGACTGAATGGCTATCAAACGAGCCCAAGCACGACTGCTCCGAGATTGTTCACGCCTCAATCACGCGCATCCTTCGTGGCATCAGACAGCGACCATTACGCATCCTCCTGTTGTACACTAAATAACAATACACCGAAGAAAGTCCGACACATATATACCACAGACGCGCCTCTATGCGCCGAACCCATAGAGCGTGCGGCCAGAACGCTTCAGGGCATACACGACGTCGAGGGCCGTCACGGTTTTCCGCTTAGCGTGCTCCGTATACGTGACGGAGTCCCGAATGACCTATAGATAGGTCATCAGTCAGTCTGCCGCAGTCAAGACACCAACGCCACTGAGAGACTCACGTTCTCGAGGAAGATCTTGAGAACACCACGGGTCTCCTCGTAGATGAGACCCGAGATACGCTTGACGCCTCCACGGCGAGCAAGACGACGAATGGCAGGCTTGGTGTCACCCTGGATGTTATCACGGAGAATCTTGCGGTGACGCTTAGCACCACCCTTTCCAAGACCCTATCGAGCAAGAACGACGAAGAGAGATAAGTCAGTACCAAGCCACCCCTCACATCGCGGAAGTTCTAACACACCTTGCCTCCCTTTCCACGGCCAGACATGTTTGTGAAGGTATTTGGTTGGTTTGGTGAAGTTGTGCGAAGTGGTTTGTCGACGAGGAGTCGAGAGGAGTGGCAGGCAGGCGGAAGTAGACGGGGGATGGGATGA

>AT8412

ACCGCCCGCGCCCGGTCGTCCTCGATGTTCGTCTTGAAAACCCACCCAATTTGCGCCTTCACCTCCTCCCCAACCGCGGTCAGCAGCTCCGGCGGGTACCGACCCGCGTGGCTGTCCTCCGCGCTCACGTCCTCCCAGCCCGCGCGCCGCAGCTGGAAGTTCATCCCGTTCCTGTCCGAGCCCCGCAGTACGCTCTGCGAGAATGGCCGGCGGATGTCGTCGTACACCTCGAGCGCCGCGTGGAGGGTCCCGAGGGTCACGGCGGGGTGTGCGAGGATCGTCGCGAGGATGTAGCCGTCCTCGAACGCTTGGCCGACGCCTGCGCCTTGGTGTGGGAGCTGCGCGT

>AT8428

GAGTAGTCCTGGACGCCGGACGGCGGGCACGTCGCAGCGGTGTTGTGCGAGCCCGTGCAGCAGTTGGGGCTGTTGGCAGTGTCGCCGAGGCCAGCGACGCATGCGGACTTGCAGCCGACGACCGTGCCGGAGGAGTCCTTCGGACCCGCGAGCTCGGCCGGGCAGTTGGGGTTGAGGTCGACGGGGCAGTCCGCGACGTGGCAGCCTTGGTTGTTGTCGACGCGCATGGGGATGTTGAAACCGTCGACCAGCGAGACTGTAGTCACACACACATATCATCAACGTTTGCGCGCGGAGGGCTCATGAGTGGCTCACTCACCATCGTACCAGTCCTGGTTGCCGTCACCCTGGAAGGTGAACTCCGCGACGGTGGCGGGGGGCACGCCGGTGCCGCTGTGCGCATCGCACAAGAGGCCGCCGTTGCAGCCACCGTCCGAGCAGGAGTTGGGCCCGGGGTTGGTGGAGAAGTCGCAGTTGCGACGACCCCAGATGCGACCCGCCTTCCAGTTGTCGGGGACCGTGAAGGAGATGGTCTGGCCAGCCTGCTGCTCCCA

>AT8459

TGGTTGCGATAACCATCTGGTCACGAATCCCGCGCGACTCCATCCACTCTCCGATGAACATCTCCGATGTCTCGTCCTGGTAGCCGTTCGCGGTGTCAATGAAATTGCCACCGGCATCGTAGAACGCGTCGAGAAGCTTGAAGCTCAACTCCTTGTCCATCGTGCCCATGATCTGGTCCCACTTGTCGCCGATGCTCATGGCACCGAGGCAGATGGGCGAGACATGAATGCCAGCTGTAGGTGCAAGCTGCCGGTGGCGGCCAAGCTTGGTTTTGGGAGAGAGAGGAGGGGCAAAGAAATCTCCTGCGACAGACATCTTGATTCAGAAGCCGCAAGGGCCTATCAGGAAAGAAGTTATGCTATGAGCGAAAAGTTGAGAGGTAAAGAATCTTGTCTGTCTCTCTCTGCGTGCGGCGTTTGCGCCATGCACGTATTATGAGTTAGGGGAATTGCTTTTTCATCGGTGACTCCGCTCAGGTTGCAAGACGTCATACTGACTCATAAACTGCGTCGTTCAGTAATCTGCATCTTCAATAGTTTGCATGGTCGTCTCGAGTTGGTATTCGGAAGGAATGTTGTGATGATACTAGTAGCGTTATCATCCCTGGGCGATTGCCAAATGAGTACAGAAAGCGACAAACCTCCATTATGATGGCTGATACCTCCAGATGCCTGCCGGTCCCGAATTAAGTTGTAGCCTATAGGATGTAGTTGTCGCTGGAGGATGCCGTCTCCGAGGAGGTGGCAGTAGGCAGACTG

>AT8497

TTCGAAGCGCCACGTCTGGTTCTTGCCAGGGTGCCACTTGCTCCAGACCACGACGTGCGTGCCCGGCGTCGCGCTCCCGTGGTCACTCAGGTCCAGGTTGAACCGCGTGTTCGGGACGAATAGCCTAGCACGCATCCCAGACCCCCGTGATCAGCGCGCGCACACAACACCTGCAGCCTGCGAAGGGGAAGGGGCGCACCTGAAAGTCGACTGGTCCTCCTCATCGGGCCAGATGTCCCACCGGAAACGCTGGGGCGTCGCGACCGCGGACACGCCGTCCCCGGCCTCGCCCTCGACACTGAGGTACAGACCCGTCGCGACGTTCCGGAGGAGCCACTGGTTGTCCTCCTGCTCCAGCGCCCACTGAATACAGGTGCACAGGTCAGTGCCGCATCGCCCAAGCCCCCGAACGCAAGGTATAGGGCGAGCGATGATCACGCACCTTCTGGTTGTCGCCGCCGTGGTAGTCGTAGCCGATGGTCTGCCGGTTGTCCGTGCCGGACAGGTCGAGCACGGTGCCGGCCTTGGCGTTGACGAGCTTGTACGTCCTGCCGCTCTGTACCATGTTCGCTTCGATCGTGGCGACTGGTGGGATTGGGGTTGTGAATGAGGGAACAGACAAGGGTGCCTGAAGGAGCGTCTGCACTTATGCAGAATGCAGCGTGTGCATGTGTTTGGCTTCCTGTCAGAGCACAACACCTTGCAATATAATTATCAACTGC

>AT8504

CGTTCGACGCCTTGTTGCGGTATGCGACCAGGGCTTTGCGCCTGGGGATATAGGTTCCCAAGGTCCTGCGCTGCGTACGTCTGGCCAGACAGACTTGCGGGCTGCGATGCCAGAAGCGGGGCGAGGAGCGCGCCGCCGAGCGATGCGTTGAGGTACATAAAGGCCGGAAACGCCATGTAGATGTGCTCCACGGGGTTTGCACGGCTATTGACGCAAAATTCGAATACAAATTGAGCATATCGAGAGTATCATCACCATTTAAGTTAGAAGTAGCACTGACCGATCTGTGCCCATATTCTTCATGAAGACCATGATGTCGTCGGGGACGAGATTCCCGCTCGAGTCGGTGCCCACAGTGATGTCGAGCGCGCTCATGGTCTGGCGCGTTGCGAGCGAGACAATGTCCACGTATTGCGACGATTTGACCCTGGCCGCGTCGCCCATGATCTTGTGGTCGAGAGCGATAGCACGTTCGAGCGCCGCCGCATAATCGCCGGTGAACGCGTCGATCTGCGACGCACAACTCTGTCAGGACAGCAAGGGCGGCCGGAGTACAACAATGGACAAGGCTCACGACGTCTTCAATGTTTGAGTATTGCGTCGCATAGTACGGCCGCCGTTGTTGGACGGCCCCAGAGGCAGTAGTATACTGGATGGTGGGGTCTCGAACAACCCCAACCGACCATGTTATGGGAGAGTCCGTCGCTTTGATCGTCCCGAGATCGACCGCCAGGGCGTATACAG

>AT8505

CGGGCGGATGGTCGTGAATGCTGTGGAGCCCTTATTCGTAAGGATGCCATTTTGCTCAAACTGATCGCGGGTAACCAAGAGGAAGTCCATTTGCGATGTGATGCTTGGTTGTGACTGGATTGACGAAGGGGGTGCGCACGGGTGAGACATGGAGGCGTACATATGTGGATGGTATTCACCGTAGAGATCGCATAGTACGTTGTGCCGTCTTGTGCCTGGTTGACGATTTCTTCGTTCGCGACGGGATTTTGCAGCCATATCTTGTGGTATTTGGACTTTGATGTGGTTCCATTACTCCACTGGACTACCGAGCCGGTGGTATCGCCAGATTCCCACTCTGCAAAACAATGTCAACGAAAAGCCAGGGTCTGGATAATGCGCTAAGTCGTACCTGCACTGATATCAGAGTACATTTGCACCGGATAGCTGTTTCCATCCAAGGACTGAGCTTCCACGGATACATAGGAAAAAGGAATTGACTGCTTGATCCAGTCGCCGGGCTAATGCGGGATGGCACCTGAGATGGAGGCGACTCCATTCAAACAATAGAGCCACTCACCTCTACCGGACTGAGGAACGTAACAGTGACGTTCATTGGACCTGCCTTCATGACGAAGATGGATCGTGTGGGTGTAATCTGAATGTTGGTGACATTCGAGGCGATGTCAGCGTTGGTCAAATCATTGCCCATCCACTTGTACGTAGTTCCGTTCACTCTGATCAAGCCTACCCAGGCCACAAG

>AT8588

CTGGCGCAGTCGAGCCGGACTCGGGGCATGGAGGGGTGTCTTGCAGTCTGCGCCGGTGTTGAGGCCGACGTCGCTGCCAGCTGGGCATGACGAAATAGGCGGCACGGCTGCCAGTGGTTGGAGCGTCCTCGAGAAGCCACTAGACTATCGAGAAGCGATTGTGGGGGTAAGAGTCCATGGGAGAAGCGAGTGGATGGATAATCATGGACGACAGCGGGAGGTACAGTAGCTGGATTCTTGGAAACACCAACCGCTCAGGCTGCTGAGCGCGGACGCCGGACGTCTCTGAGCGCCAAGCGACGCTCAGGCTATACTTTGCTTCGAAGGTGTAGGGCCTGTGGAAAAGAGTTACTTCTCGACTTCTACATACGTACCGCAACACTACCGCAACTTTGCTCTGGGATTGACATCATGACTAGTCGTAGGATAGGTGAGGGGGTCGGACACCACGCACGCTCAGGCCTCAGGACTAGTAGGTAGTACCTGGAGGGGAACTTGAAGAGAGGACACGCAGCATCCCACATAATACATCAAACAAGGGAACAGCAATACCGAAGCGATACTACAGCTTCGCCGCAGGTGCCTCAACCTTTATCGAGTAGTATAGCCTAGGAAAATGACAGTGTATAGGCTTCCCGGAGAAACCCACTGTCTCGACAACAACTTCCTCCTTGCTGAGTGCGATCAGTTCCCCTACTGTGGGCACCTTGCCTGTTCAGAGCCACAGGCGTGAGGACTCCACACATACGACGGGTACTGTCACATACCGTTGTCGGAGGGCGCAACCGAAACCCAATCCCGTAGCTTGACCTTGAGTCGGGACGCCTCCAACTCGTCGAAGCCGACGACGTTGACGTCCGCAGATACGGAGGAAGTGATTAGCTCCGCAGCACGCTCGCCAGAAAGGCCGTCCAGGACTGGTTTATGCAACGCCTCCTCTTTGGCGATGTATGCTGACATTCGTGAGATCCACTGTAGGGAGCGGGTCAGCGAAACAGAACAACAGCAACGCGCAGTAAAGGGGGATAACGCACCGCGAGAGTCTTCGGGAACTTGATCTCGTCAAGCAGTTCAGTCTTGAGATGCCTCAACGGCCTGAATGACAGCATCCAGTCCCAGACAACGTGGACAGAGATGTCTGCGAGACCGGGGGTCTCAGTATCCATCAGCCATTCGCGACCATCCGAGAGCTGCTCTTCAATGAGTGCCTATGGTCGGTAAGTGTCAGCATCACGTTTGCGCTGCGAAGATGACGTCCCTCTCAGATGATCGATTGGGGACGAACCAGGTGAGATGATAATGTG

>AT8595

TTCTGGCAGCAGACAGGCTGCTGAGAGCAGGCGGAGCTACCGCCGAGGCCAACAACGCTGAGCGGGGAGCACTGGAGACCGATCAGGCCCGTGACGTCCTGCAAGTTGAGGCCCAGGAGGCCCAGGATGCCGGCGCCCGCCGCGGAGCTGGACTACACCTCAGACGTCAGCATCGCAGCTCCACGCACAACGGACCGCGACGGAAGCGGACTTACGTCCTCGACGCTGTTGCAGCACTGGATGCTGCCGGTGTTGCAGCTGCCGGAGCTCGCGGTGGGGGCCGCGAGGGCGGAGAGAGCGAAGAGGGCAGAGACGGCGATGGAGCGAGCGAACATGGTCGAGTTGGTCGATGAGGACGAACGGATGGATCGGTGCGTTGGGGTCTGTTGGACTGCGGGATTCAGAAAAGAGCGACTGCGAACTGAGGTTATTCTGCCTGTGCT

>AT8638

GGGCCGGCCTCCCTCCAGCTCGTCCGCCCTCGGAGCACTGTTCTCGTTCTGCGGCCTGACTGCTGTTATCACCCAGCTATGTTGTGCTATTACGCTTGAAATGGGTGGCTTCGTTATGATTGTCAACCATGGAGTCAACGTTTTATGGTCCGTACATCCTGCGACTTGCGAATACTAGTATCGTGTGTCCAGTTAACCAGCACTGCCAGTGGGATCTCCCCCGTCCCACACCATAGTCAATTGCAATAGGTGACCCTATAAGGTCCCACCCTTGCCTAACGAAGCGGCATCTCCCTCGTCCCATCCAGACGGCGTTCGCTTATGGCCGAGCACAAACACCTCGATGTGACCCTCCCCGGAAGCGCGCTGGTCGACAAGCAGTGCCGGCGGGGGGCTGGCGAGGAGGAACTGGAACTTCCGACGCCATTGCGTAGGTGAATGCGGGGAGTGCGGTGGCTCGGCGCCACCCCGGGGCCGCAGAGGGTCAGAAATGCGCCCGAAGCCTTCCCGCTCGCGCTCAACTTGAGAGTGGGGCGACTTGTTCAGCGCGACGACGACGGCGAGCAGCACCGGGTACAGCGCCTGTCAAGTAAGTAAGCAGTACCAGTAAGGCCGAAAGGTTCTGGCACGCTGGGCTGCGCGATACTAAGAGACGACTAGACTTACTGTGAGGGGAGTCAGCGCGCCCAGGTCCAACACCCAGAATCCCTGCACGAACGATGCCGGGTTCCGGGCGTACACGAGCTCTTCGAAGCCAGTTGCGGTATCCTCCAGCGCACCCAGCGACTGGTTCA

>AT8640

ACGTTGATAGTCAGCGCGGCGGTTCCCGCGCAATACTGGGGCGGGAGGTCGCTCAGCCCGGCCAGTCGCTCACGTCCTGGAGGCCCGGACACTGCGTCGAGCCGGTCCCTCCCGCTCCGCCGGCGGTGCTCGTGAGGTAGCAAGCCTGCAGGTCGATGAAGAGCGACACGACGTTGCTGTACACCGCCGCCGTGTCGTGCGAGAGGTTGCGGACGCAGAGCGCGAAGTAGGCCGTCGTCCCGGCGTACATCGTGCCCGTTGCGAGCAGGAGGGCGGTGTTCCATGTGTCTCTCCACAAACGCGGC

>AT8704

ATGGCCATCAACAGCACTTCGCTCTCCGCCGCCTCTTCTTCCTCCTCGCCTTCGCCATCCTCGTCCTCCTCGCCCGTATGGTGCGTCTCCTCCCCTTCCGTCGCGATGTCCTTGTCCTCCATCCCCGGCGACGGCGGGAACGGCACGACGTCCTCCGAGGACACCTGCGTGCCGTACCCCGTGAACGGGTACGGCAGCGACATCAGCGGGCCACTCGCGTCCGCCCCCGTCCGCTCCTTCTGCTGCACCGCGCTGACCGGCGGAATGATGCTCGAC

>AT8705

CCCGACGCAGAAAACAGCGTCGGGGTCAGGGCTGCGCTCGTCGCGCGCGACCACGCACGGGCGTGTCCCGCACCAGAGAACGCGCGCGACTGGTCCGTAGGCTCCCGACGCGTGGCCCCCTCCGCTTGACTCTGGAGACTGACGATGCGTACCGACCGTGGCGTTGGCTTCCGGCCGATCGTGGCAGGGGGCTCGGGCTCATTGGTGTTGCTGGGCCCCGGGTCGCTGAACATGCCAGCGACGGAGTGGATGGTGGAGCGGGGAGCAGGGCCGGGAGGACGCTTGGGGAGCCAGGACGGGTACCCTTCCGAACTGGAGGAGACGCCTTGAGGGGACTGGGGAGAAGACGTCGATTGGCGTTCGGATACGGGCGTTGGTCCGTGCGTGGGGCCAGGCTTTGAGTGATCAGAGTTTCCCGACGAGCTTGGCCGCGAAGCAGAATGAGGGGAGGGCGAGGCGGAGGGACTAGGAGAATCGTGCTGTGGTCGTGACATGGTCGTAGATTACGGCGGAGGGAATCATGCAAGGTCAGTAGATGTAGATTAGGCAGAGGAGTTGCGAGTCCGTAGGCAAGGATTAGATGCGGCGTCGCGAAGAGGGCAGTGGCAGCGTGCAGGGGACGGCACGGCGAGCTGAACAGGGGCTGCTGGGTGCGACTGCTGCTGGAGGGGGCCTTGAACGCGAGGGCCTCGTGTTATGACGACGGGCAGCCTCGTGTTGAGCGAGCAGTGGGAGACGACGCGCAGAGACTTCCACGGCCGCCAGCGCGCCTCGTCTTTGAACCAACGACTCACCAATTTAGGGAACGTGCCGAGGCCGGCCGTTGAACTCCGTGCACGCGCTGAATTTGCTTCCGCTTTCCCGCCGGCCCGCCGGTCGTCAGATGCCTCCGCTCCGGCGCTTGTTGTAACACTTGT

>AT8706

CCGCCGCCTCTTCTTCCTCCTCGCCTTCGCCATCCTCGTCCTCCTCGCCCGTATGGTGCGTCTCCTCCCCTTCCGTCGCGATGTCCTTGTCCTCCATCCCCGGCGACGGCGGGAACGGCACGACGTCCTCCGAGGACACCTGCGCGCCGTACCCCGTGAACGGGTACGGCAGCGACATCAGCGGGCCACTCGCGTCCGCCCCCGTCCGCTCCTTCTGCTGCACCGCGCTGACCGGCGGAATGATGCTCGAC

>AT8723

AGGGCGCGTCCCAGATGGCGTCCGCCGCCCTCTCCGCTTCGAGGCTCCACGGACAGGCAGGGCGACTGGGAACATCCAAGAGCCTCTCGTCCAGGAAGCGGGGGTCGTATATCTTGACGACGGCGGGATCGGGTCCCAGTTCAGGAGAGCGAACGACGAGGACGACGGCCTTTGTGAACGGTGCAAAAGGTCGATCGATGGTGAGGGTAAGAGTGCGTTCTGGGGTTTGGAAGGCGACCTGGCGGCCGGGGAAGAATATGGGGCTCGCCTCCATGGCAGATTTGGTGGTAGTTTGAACCAAGGACGTTCTCTCGAAGATCTAGTAAAAGGGATGAGAGGGGGTCACGAGAGCGGGCCGAGCGTAATGTGGACGTTCGTCACGCCGACACGGGCAGAAAGCACTGGCC

>AT8998

GCCGCCCTCTCCGCTTCGAGGCTCCACGGACGGGCAGGGCAACTGGGAACGTCCAGGAGTCTCTCGTCCAGGAAGCGGGGGTCGTATATCTTGACGACGGCGGGATCGGGGCCCAGCGCCGGGGAGCGGGCGAGGAGGACGACGGCCTTTGTGAAGGGCGCGAAATGTCGATCGATGGTGAGGGTAATGGTGCGTTCTGGGGTTTGGAAGGCGACCTCGCGGCCGGGGAAGAATATGGGGCTCGCCTCCATGGC

>AT9121

TGTCCTGAGGCACTAGAGCCAAGCGTCCCCGGAGTGTCTCCAGGCCCATCTCAGCGATGTTCTTCCCGTCGATCGCTATCGCGCCAGAGTCGAGGTTCACGATTCGGAAGAGTGCCTGGAGAAGGGAGCTCTTTCCAGCGCCTGTTCGACCCACGATCCCAATCTGTTGCGCATTGCGACGAAGTCAGCAACTGCTATGACGACGGAAGCGCAGGGTGGTGGCAACGGGTACCTTTTCGCCGGGGTTGATTTGGAAGCTAACGCCCTTCAGCACCGCCGGAAGTCCCGGGCGATAGGACATCTCGACATCCTTGAACTCGATAGCGCCGCTTACCGGCCACGCTGGCGGAGGATCCGTAGAAGTAGTGCCATTGCCTTCGCCGGGAAGCTCGGTGTAGTGCAGTATACGTTCAACCGCGTTGAAGTTCTGTTCGTTCT

>AT9122

ACTGCTCAGAGTGTAGGTCAGTACAACACCGATTTTGCTGGGGTCAACGGTGTCTCTGAAGCCGGCGGCGAACAGACAGATGCCTAAGATCAGCATGTTTCCCAGGATATCGAGGCGGACTCCCAGCCAACGCTGAATCCCGACGGTCATATAGTAAGCTCGGTTCTCAACGTCTTGACCTTGGTCAGCGTTCCGGACGAAACGGTCCTGGGAACGATATGCGCGAACCGTGCTCAGTCCAGTGAGCGTCTCTGGGGGATTATTAGCCTGTTTACATCAACTAAAGCTACAAATAGGGGCTTACCGGTGTATGAGGCATACAGGACAGAGCGCAGATTGGAATCTAGGCGCTTAGTTTCCACAGAGGTGCGACGGTAGTAGATGGCAGCCAGGTAGTATAGGACGATCATAGGGGCGAAGATGATCCCAAGGTAAGGGAAGGTGTAGAACACAAGGCCAACGGTGC

>AT9133

CCTTTCCAGACCCGACCCGACCAACGATCGCGACAAAGGAGCCTTTGCGAATATTCAGCTTGATATCCTTCAGCTCAAACGGCTGTTCCTTCTCCTTCTCCTTAATCTCGCCGCCGCCGGTCGTTGCAAGGTTCTCCGCCGAGCGCGCTGGGGATGTCCCTGCCGGGGTATTGACTGCAGTGGGCAACAAGGAGTCCCCGCCCTTCTTCCTGTCCTTCTTCGCTGCAGCACTGCCCCTTCCCTTCTTACTCTTCCCGAACTTGGCCGCGAGGTTGATCGCATTGGCGTCCTTGCGGACCGTCTCCCACGTGAAGTCACCGTCTACATCGATCGCAGATTCAGCGTTCGCGTCGATCTCGTACGGGACTGAGAGCTCCTCTGCACGCATGAAGGCGCCGATGCGGCGGAGCGCGACAAGCGCGTCGGTTGCGGAGGCGAGGACGATGGGGAGGAGGATGAGCGGCATCCGG

>AT9237

GAATCCCTGGACGGGAAAGTTGATGGCGTTCAAACCAGTGACGGCGGTGTCGAGGTCGCGAGAGGCGGTGATGAACGATTGGATCGCATTGATCACTTGGTCGGAGGTCTGAGCCATGGTCCCGACGACGCCAAGGAGGGTAACGAAGGTGACGGTGAGGAAAGTGCTAAAGAACTTCATGGTGGCGAAAGTTAAGTATTCGTAGAAGCTGGTGAGGCCGTTGAGCTCTGTGGGATGTCGCTGAGT

>AT9571

GCGGGGAGCGGGGTTGTCGCCCAGGGCGGCCACGGCGAGCCCTTTGGTATGCGCGGAGCAATTATCCAGCACGATGAGGCGGATTGGACAGCGCAGGCCGAGGACAAAGAGCATCCACGGATCACAGACGAGTTTATCGAGCCTCTTCCATGGGCGTGAGCGAGGGGCGCCGTCTTGTGCGCCGCCTTGTGCGCGCTGATTGGCAGCGCGTAGGTCATCGCACGCATTCCCTTCGGGAACAGATGGGTCGAACGTACCGAGGGACAGGGTGCCATCGAGTCCGGGGAAGAAGTGCTGCAGATGGTCTACCAGCGGCCTCTCCAGCAGGAAGCGCAAGTGCAACGAGCGGACAGCGGGGCATGGCGCCATGCTCACGTAAGGCCGCGACGTCTC

>AT9624

ACAGCCCAGTAATTGCTCTTATCGGGTTGCACGGGAATCGTGGTGCAGTTGCTTTGTACGAGGTCGTCGGGGTCGTACACGCTCTTGAACCGGCTCGCTCCCACGACGGAGTGTACGTGGGGGCCGACCTGGCGGACGGGTCAGTCAAGCAGCACTTCGCAGTCTGCGTGCAAGGAGATAAGGGTGGTACTCACCTCATTGGGCATGATGACGGGGTCAAGGCGAGTCGTGACTATGGGTCTGACGGCCCCAAAGATCCAGTGCTCGGCGTTGGCCACTGCAGCTAGCACGACAAAGGTCCATAGGCTAGTGTGCATGGTGGAGAGTAGAACGTTGGACGAAGGTGGGACTGGGGAGTCCGTGCAGTCAAAGAAAGAGCTTATGAACCCGCACCAGCGCTGAAAGAGTATGGGGCGC

>AT9664

CCCTCTAAGTGGACCCGAGAACTCGAGCCGTCCTTCCCTCCTGTAGCCTCGAGCTCCTTGAGTGGGTCGGGAGAACGCCCTCGTCCTCGACCCGGTCCTGGTCGTCGCCGAAGGAGTCCACGTATCCGTGGTCTTCGTCTTCAGTGCAGATATCGATCGACGCCCCGAGGGAACCGACGACGCGCTCGAAGATAGGCGTCTCTGCGCCGCCGTCGCGGCCGTGTAGGGCCATCGTCGTCGTGCCCTGCGGATTGCTCGCCCTACGGTCTGCGGCCTGGAGGTGGAGGAGGAAGCGCGATATCAGGATGGTCGTGATTCTGCGCCGGGTGGTTTAGCAAGACTAGCGAGAGTGACGTCGATGATACGCACGGTCCCCAGAACTGCACCGCGTAGGCGATATTCCCGAGCGCGGGGCTCTGCTATGTGGAGAGCAACACTATTACAACATGTTCCGGCCTACATGGTAGCAAAGGAACGTGATGCTTACGGGGATCAATGGTTCTATCAAGTGTAAGATATTGAGGAGTAGCATTACGCTTGGAAACTATCAACGAACTCGGATTGGAATAACGGAGCTGGTGCGCCTTACATAAAATATATTGAGCCTATGATGGGCATCGTCAATAGCGCAGTAGTCATTTCAAGCACAGCACACATACCATCACGCAGAAGGACGGCGACAAACCC

>AT9908

GAGCCGCCTCGGGGATCACCTGGAACAGTGTGGAGCGTAGCCTCCCGCTCCTCATGGGTCAAGATGCTGACCGTTGGCGGCGGGGTTCCAAGATGATCGCCGTATTGGTACTCGTCCGGCGAATCTCCGTTGTCATAGTGCCCGCTGCACCAGACGGCAAAGTGTGCTGTCACGTCTTGGATGGGAAGCGAGGGGTCGACGAGGGGGGAGATGGCACTTGGGATAGCCGGTATGCCCAAAATGACGTTGGGCGGATCTGATCGCATTCAACAAGCATCAACGATAAGACCGTGGTCTGTTCGCTCGAATGGGGCTCAATACGCACCGTAGAAGATCACCCGCCGGACATATCTGCCCAACTCCACTTCATGTACGGGGAACGACACGACATTGGCCAGAAGCGATGTCATCCACCCGGCTCCGAACGACCACCCCCCAACTACGATCCCTCCTTTGTTTTCTTCGGGACGAGCGAGGGGGATTTTGTGGCGGGCAACAAACTGGACGAGCAAGTCGTACACTTCGCGCGCTCGCTCTTGCATGAATGCGTGGAGTTTCTCTCGCGCCGCGAGGGGGTTTGTCTTGACGTCGATCGCGGCATTCACCAGCAGAGCTCGTTCTTCGAGGGTGTAGGGCAGGGCGGTTGGGTAATCTCGGCGGTTCACGAGCACGACGCGCGCGTTGTGTCGAGTAGCCAGTGGGATGAGC
