# Supplementary material for: Genome-wide Identification and Characterization of Natural Antisense Transcripts by Strand-specific RNA Sequencing in Ganoderma lucidum
Source: Sci Rep. 2017 Jul 18;7:5711. doi: 10.1038/s41598-017-04303-6 (PMC5515960; doi:10.1038/s41598-017-04303-6)

2017/4/9

NCBI Blast:GL25583-R1_1

[BLAST ®](https://blast.ncbi.nlm.nih.gov/Blast.cgi) » blastp suite » RID-EMFCRMMP016

BLAST Results

Job title: GL25583-R1_1

RID

[EMFCRMMP016](https://blast.ncbi.nlm.nih.gov/Blast.cgi?CMD=Get&RID=EMFCRMMP016) (Expires on 04-10 21:56 pm)

Query ID

lcl|Query_344972

Database Name

nr

Description

Molecule type

Query Length

GL25583-R1_1

amino acid

629

Description All non-redundant GenBank CDS

translations+PDB+SwissProt+PIR+PRF excluding

environmental samples from WGS projects

Program BLASTP 2.6.0+

New Analyze your query with SmartBLAST

Graphic Summary

Putative conserved domains have been detected, click on the image below for detailed results.

Distribution of the top 100 Blast Hits on 100 subject sequences

Color key for alignment scores

<40

40-50

50-80

80-200

>=200

Query

1

100

200

300

400

500

600

https://blast.ncbi.nlm.nih.gov/Blast.cgi

1/7


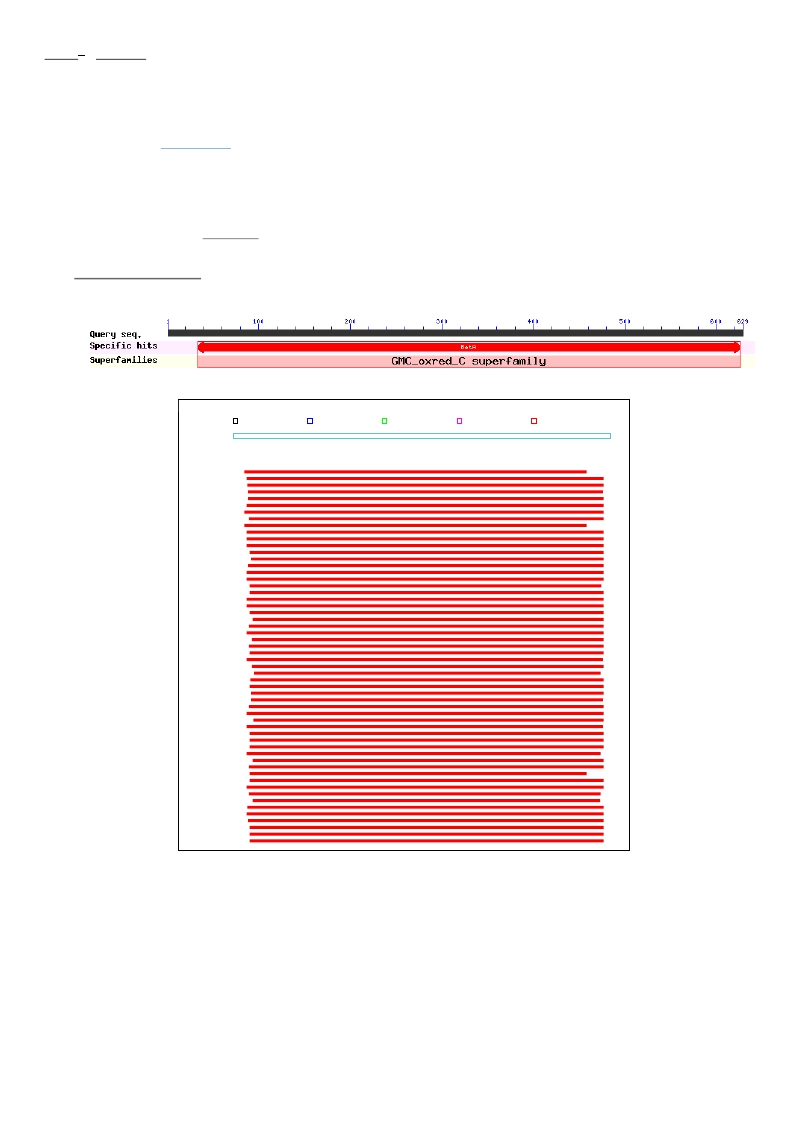


2017/4/9

Descriptions

Sequences producing significant alignments:

NCBI Blast:GL25583-R1_1

Description

[Max](https://blast.ncbi.nlm.nih.gov/Blast.cgi?CMD=Get&ALIGNMENTS=100&ALIGNMENT_VIEW=Pairwise&CDD_RID=EMFCRRMC013&CDD_SEARCH_STATE=0&DATABASE_SORT=0&DESCRIPTIONS=100&DYNAMIC_FORMAT=on&FIRST_QUERY_NUM=0&FORMAT_OBJECT=Alignment&FORMAT_PAGE_TARGET=&FORMAT_TYPE=HTML&GET_SEQUENCE=yes&I_THRESH=&LINE_LENGTH=60&MASK_CHAR=2&MASK_COLOR=1&NEW_VIEW=yes&NUM_OVERVIEW=100&PAGE=Proteins&QUERY_INDEX=0&QUERY_NUMBER=0&RESULTS_PAGE_TARGET=&RID=EMFCRMMP016&SHOW_LINKOUT=yes&SHOW_OVERVIEW=yes&STEP_NUMBER=&WORD_SIZE=6&OLD_VIEW=false&DISPLAY_SORT=1&HSP_SORT=1)

[Total](https://blast.ncbi.nlm.nih.gov/Blast.cgi?CMD=Get&ALIGNMENTS=100&ALIGNMENT_VIEW=Pairwise&CDD_RID=EMFCRRMC013&CDD_SEARCH_STATE=0&DATABASE_SORT=0&DESCRIPTIONS=100&DYNAMIC_FORMAT=on&FIRST_QUERY_NUM=0&FORMAT_OBJECT=Alignment&FORMAT_PAGE_TARGET=&FORMAT_TYPE=HTML&GET_SEQUENCE=yes&I_THRESH=&LINE_LENGTH=60&MASK_CHAR=2&MASK_COLOR=1&NEW_VIEW=yes&NUM_OVERVIEW=100&PAGE=Proteins&QUERY_INDEX=0&QUERY_NUMBER=0&RESULTS_PAGE_TARGET=&RID=EMFCRMMP016&SHOW_LINKOUT=yes&SHOW_OVERVIEW=yes&STEP_NUMBER=&WORD_SIZE=6&OLD_VIEW=false&DISPLAY_SORT=2&HSP_SORT=1)

[Query](https://blast.ncbi.nlm.nih.gov/Blast.cgi?CMD=Get&ALIGNMENTS=100&ALIGNMENT_VIEW=Pairwise&CDD_RID=EMFCRRMC013&CDD_SEARCH_STATE=0&DATABASE_SORT=0&DESCRIPTIONS=100&DYNAMIC_FORMAT=on&FIRST_QUERY_NUM=0&FORMAT_OBJECT=Alignment&FORMAT_PAGE_TARGET=&FORMAT_TYPE=HTML&GET_SEQUENCE=yes&I_THRESH=&LINE_LENGTH=60&MASK_CHAR=2&MASK_COLOR=1&NEW_VIEW=yes&NUM_OVERVIEW=100&PAGE=Proteins&QUERY_INDEX=0&QUERY_NUMBER=0&RESULTS_PAGE_TARGET=&RID=EMFCRMMP016&SHOW_LINKOUT=yes&SHOW_OVERVIEW=yes&STEP_NUMBER=&WORD_SIZE=6&OLD_VIEW=false&DISPLAY_SORT=4&HSP_SORT=0)

[E](https://blast.ncbi.nlm.nih.gov/Blast.cgi?CMD=Get&ALIGNMENTS=100&ALIGNMENT_VIEW=Pairwise&CDD_RID=EMFCRRMC013&CDD_SEARCH_STATE=0&DATABASE_SORT=0&DESCRIPTIONS=100&DYNAMIC_FORMAT=on&FIRST_QUERY_NUM=0&FORMAT_OBJECT=Alignment&FORMAT_PAGE_TARGET=&FORMAT_TYPE=HTML&GET_SEQUENCE=yes&I_THRESH=&LINE_LENGTH=60&MASK_CHAR=2&MASK_COLOR=1&NEW_VIEW=yes&NUM_OVERVIEW=100&PAGE=Proteins&QUERY_INDEX=0&QUERY_NUMBER=0&RESULTS_PAGE_TARGET=&RID=EMFCRMMP016&SHOW_LINKOUT=yes&SHOW_OVERVIEW=yes&STEP_NUMBER=&WORD_SIZE=6&OLD_VIEW=false&DISPLAY_SORT=0&HSP_SORT=0)

[Ident](https://blast.ncbi.nlm.nih.gov/Blast.cgi?CMD=Get&ALIGNMENTS=100&ALIGNMENT_VIEW=Pairwise&CDD_RID=EMFCRRMC013&CDD_SEARCH_STATE=0&DATABASE_SORT=0&DESCRIPTIONS=100&DYNAMIC_FORMAT=on&FIRST_QUERY_NUM=0&FORMAT_OBJECT=Alignment&FORMAT_PAGE_TARGET=&FORMAT_TYPE=HTML&GET_SEQUENCE=yes&I_THRESH=&LINE_LENGTH=60&MASK_CHAR=2&MASK_COLOR=1&NEW_VIEW=yes&NUM_OVERVIEW=100&PAGE=Proteins&QUERY_INDEX=0&QUERY_NUMBER=0&RESULTS_PAGE_TARGET=&RID=EMFCRMMP016&SHOW_LINKOUT=yes&SHOW_OVERVIEW=yes&STEP_NUMBER=&WORD_SIZE=6&DISPLAY_SORT=3&HSP_SORT=3)

Accession

[score](https://blast.ncbi.nlm.nih.gov/Blast.cgi?CMD=Get&ALIGNMENTS=100&ALIGNMENT_VIEW=Pairwise&CDD_RID=EMFCRRMC013&CDD_SEARCH_STATE=0&DATABASE_SORT=0&DESCRIPTIONS=100&DYNAMIC_FORMAT=on&FIRST_QUERY_NUM=0&FORMAT_OBJECT=Alignment&FORMAT_PAGE_TARGET=&FORMAT_TYPE=HTML&GET_SEQUENCE=yes&I_THRESH=&LINE_LENGTH=60&MASK_CHAR=2&MASK_COLOR=1&NEW_VIEW=yes&NUM_OVERVIEW=100&PAGE=Proteins&QUERY_INDEX=0&QUERY_NUMBER=0&RESULTS_PAGE_TARGET=&RID=EMFCRMMP016&SHOW_LINKOUT=yes&SHOW_OVERVIEW=yes&STEP_NUMBER=&WORD_SIZE=6&OLD_VIEW=false&DISPLAY_SORT=1&HSP_SORT=1)

[score](https://blast.ncbi.nlm.nih.gov/Blast.cgi?CMD=Get&ALIGNMENTS=100&ALIGNMENT_VIEW=Pairwise&CDD_RID=EMFCRRMC013&CDD_SEARCH_STATE=0&DATABASE_SORT=0&DESCRIPTIONS=100&DYNAMIC_FORMAT=on&FIRST_QUERY_NUM=0&FORMAT_OBJECT=Alignment&FORMAT_PAGE_TARGET=&FORMAT_TYPE=HTML&GET_SEQUENCE=yes&I_THRESH=&LINE_LENGTH=60&MASK_CHAR=2&MASK_COLOR=1&NEW_VIEW=yes&NUM_OVERVIEW=100&PAGE=Proteins&QUERY_INDEX=0&QUERY_NUMBER=0&RESULTS_PAGE_TARGET=&RID=EMFCRMMP016&SHOW_LINKOUT=yes&SHOW_OVERVIEW=yes&STEP_NUMBER=&WORD_SIZE=6&OLD_VIEW=false&DISPLAY_SORT=2&HSP_SORT=1)

[cover](https://blast.ncbi.nlm.nih.gov/Blast.cgi?CMD=Get&ALIGNMENTS=100&ALIGNMENT_VIEW=Pairwise&CDD_RID=EMFCRRMC013&CDD_SEARCH_STATE=0&DATABASE_SORT=0&DESCRIPTIONS=100&DYNAMIC_FORMAT=on&FIRST_QUERY_NUM=0&FORMAT_OBJECT=Alignment&FORMAT_PAGE_TARGET=&FORMAT_TYPE=HTML&GET_SEQUENCE=yes&I_THRESH=&LINE_LENGTH=60&MASK_CHAR=2&MASK_COLOR=1&NEW_VIEW=yes&NUM_OVERVIEW=100&PAGE=Proteins&QUERY_INDEX=0&QUERY_NUMBER=0&RESULTS_PAGE_TARGET=&RID=EMFCRMMP016&SHOW_LINKOUT=yes&SHOW_OVERVIEW=yes&STEP_NUMBER=&WORD_SIZE=6&OLD_VIEW=false&DISPLAY_SORT=4&HSP_SORT=0)

[value](https://blast.ncbi.nlm.nih.gov/Blast.cgi?CMD=Get&ALIGNMENTS=100&ALIGNMENT_VIEW=Pairwise&CDD_RID=EMFCRRMC013&CDD_SEARCH_STATE=0&DATABASE_SORT=0&DESCRIPTIONS=100&DYNAMIC_FORMAT=on&FIRST_QUERY_NUM=0&FORMAT_OBJECT=Alignment&FORMAT_PAGE_TARGET=&FORMAT_TYPE=HTML&GET_SEQUENCE=yes&I_THRESH=&LINE_LENGTH=60&MASK_CHAR=2&MASK_COLOR=1&NEW_VIEW=yes&NUM_OVERVIEW=100&PAGE=Proteins&QUERY_INDEX=0&QUERY_NUMBER=0&RESULTS_PAGE_TARGET=&RID=EMFCRMMP016&SHOW_LINKOUT=yes&SHOW_OVERVIEW=yes&STEP_NUMBER=&WORD_SIZE=6&OLD_VIEW=false&DISPLAY_SORT=0&HSP_SORT=0)

GMC oxidoreductase [Trametes versicolor FP-101664 SS1]

alcohol oxidase [Dichomitus squalens LYAD-421 SS1]

GMC oxidoreductase [Trametes cinnabarina]

alcohol oxidase [Punctularia strigosozonata HHB-11173

SS5]

choline dehydrogenase 6 [Heterobasidion irregulare TC 32-

1]

GMC oxidoreductase [Neolentinus lepideus HHB14362 ss-1]

hypothetical protein SERLADRAFT_363842 [Serpula lacrymans

var. lacrymans S7.9]

GMC oxidoreductase [Neolentinus lepideus HHB14362 ss-1]

L-sorbose 1-dehydrogenase [Trametes pubescens]

alcohol oxidase [Fomitiporia mediterranea MF3/22]

GMC oxidoreductase [Rhizoctonia solani AG-3 Rhs1AP]

GMC oxidoreductase [Rhizoctonia solani 123E]

alcohol oxidase [Gloeophyllum trabeum ATCC 11539]

alcohol oxidase [Fomitiporia mediterranea MF3/22]

hypothetical protein RSOLAG1IB_04036 [Rhizoctonia solani

AG-1 IB]

hypothetical protein PHLGIDRAFT_128561 [Phlebiopsis

gigantea 11061_1 CR5-6]

GMC oxidoreductase [Dichomitus squalens LYAD-421 SS1]

GMC oxidoreductase [Sphaerobolus stellatus SS14]

alcohol oxidase [Stereum hirsutum FP-91666 SS1]

GMC oxidoreductase [Schizopora paradoxa]

hypothetical protein PHACADRAFT_260581 [Phanerochaete

carnosa HHB-10118-sp]

hypothetical protein PHACADRAFT_260475 [Phanerochaete

carnosa HHB-10118-sp]

hypothetical protein PLICRDRAFT_47095 [Plicaturopsis crispa

FD-325 SS-3]

hypothetical protein WG66_16844 [Moniliophthora roreri]

GMC oxidoreductase [Schizopora paradoxa]

hypothetical protein PLEOSDRAFT_1114195 [Pleurotus

ostreatus PC15]

gmc oxidoreductase [Moniliophthora roreri MCA 2997]

GMC oxidoreductase [Stereum hirsutum FP-91666 SS1]

hypothetical protein GYMLUDRAFT_240943 [Gymnopus

luxurians FD-317 M1]

alcohol oxidase [Schizopora paradoxa]

alcohol oxidase [Schizopora paradoxa]

hypothetical protein PHLGIDRAFT_128050 [Phlebiopsis

gigantea 11061_1 CR5-6]

alcohol oxidase [Fomitiporia mediterranea MF3/22]

alcohol oxidase [Sanghuangporus baumii]

hypothetical protein GYMLUDRAFT_232744 [Gymnopus

luxurians FD-317 M1]

hypothetical protein GYMLUDRAFT_235428 [Gymnopus

luxurians FD-317 M1]

GMC oxidoreductase [Trametes cinnabarina]

alcohol oxidase [Fomitiporia mediterranea MF3/22]

hypothetical protein GYMLUDRAFT_240944 [Gymnopus

luxurians FD-317 M1]

hypothetical protein PHLGIDRAFT_123211 [Phlebiopsis

gigantea 11061_1 CR5-6]

hypothetical protein PHLGIDRAFT_236503 [Phlebiopsis

gigantea 11061_1 CR5-6]

alcohol oxidase [Stereum hirsutum FP-91666 SS1]

GMC oxidoreductase [Trametes cinnabarina]

639

609

590

568

541

532

534

527

523

520

518

512

511

511

508

508

504

499

496

495

493

492

490

489

488

487

488

487

487

484

483

483

481

480

478

479

476

477

472

469

468

468

494

639

609

590

568

541

532

534

527

523

520

518

512

511

511

508

508

504

499

496

495

493

492

490

489

488

487

488

487

487

484

483

483

481

480

478

479

476

477

472

469

468

468

494

91%

95%

95%

94%

94%

95%

95%

94%

91%

95%

95%

95%

94%

94%

94%

95%

95%

93%

94%

95%

95%

94%

93%

94%

95%

93%

94%

94%

95%

93%

92%

94%

94%

94%

93%

94%

95%

93%

95%

94%

94%

94%

94%

0.0

0.0

0.0

0.0

0.0

3e-180

3e-180

4e-178

3e-177

2e-175

1e-174

3e-172

7e-172

1e-171

1e-170

3e-170

5e-169

9e-167

4e-166

2e-165

1e-164

3e-164

8e-164

4e-163

7e-163

1e-162

1e-162

2e-162

4e-162

3e-161

6e-161

9e-161

1e-159

2e-159

4e-159

8e-159

3e-158

9e-158

3e-156

2e-155

5e-155

5e-155

1e-154

56%

51%

50%

49%

48%

47%

46%

47%

48%

45%

45%

44%

45%

44%

44%

44%

45%

45%

46%

46%

45%

44%

45%

45%

44%

45%

45%

44%

43%

43%

43%

43%

43%

42%

44%

42%

43%

44%

43%

44%

43%

45%

45%

[XP_008036579.1](https://www.ncbi.nlm.nih.gov/protein/636610889?report=genbank&log$=prottop&blast_rank=1&RID=EMFCRMMP016)

[XP_007361326.1](https://www.ncbi.nlm.nih.gov/protein/597972921?report=genbank&log$=prottop&blast_rank=2&RID=EMFCRMMP016)

[CDO72925.1](https://www.ncbi.nlm.nih.gov/protein/691791759?report=genbank&log$=prottop&blast_rank=3&RID=EMFCRMMP016)

[XP_007384326.1](https://www.ncbi.nlm.nih.gov/protein/599110757?report=genbank&log$=prottop&blast_rank=4&RID=EMFCRMMP016)

[XP_009543298.1](https://www.ncbi.nlm.nih.gov/protein/695541488?report=genbank&log$=prottop&blast_rank=5&RID=EMFCRMMP016)

[KZT20506.1](https://www.ncbi.nlm.nih.gov/protein/1023223658?report=genbank&log$=prottop&blast_rank=6&RID=EMFCRMMP016)

[XP_007323519.1](https://www.ncbi.nlm.nih.gov/protein/597938350?report=genbank&log$=prottop&blast_rank=7&RID=EMFCRMMP016)

[KZT25645.1](https://www.ncbi.nlm.nih.gov/protein/1023228846?report=genbank&log$=prottop&blast_rank=8&RID=EMFCRMMP016)

[OJT05708.1](https://www.ncbi.nlm.nih.gov/protein/1112951532?report=genbank&log$=prottop&blast_rank=9&RID=EMFCRMMP016)

[XP_007270014.1](https://www.ncbi.nlm.nih.gov/protein/595782942?report=genbank&log$=prottop&blast_rank=10&RID=EMFCRMMP016)

[EUC64134.1](https://www.ncbi.nlm.nih.gov/protein/576991432?report=genbank&log$=prottop&blast_rank=11&RID=EMFCRMMP016)

[KEP54447.1](https://www.ncbi.nlm.nih.gov/protein/660970712?report=genbank&log$=prottop&blast_rank=12&RID=EMFCRMMP016)

[XP_007869453.1](https://www.ncbi.nlm.nih.gov/protein/630360357?report=genbank&log$=prottop&blast_rank=13&RID=EMFCRMMP016)

[XP_007269678.1](https://www.ncbi.nlm.nih.gov/protein/595782270?report=genbank&log$=prottop&blast_rank=14&RID=EMFCRMMP016)

[CEL60797.1](https://www.ncbi.nlm.nih.gov/protein/751838703?report=genbank&log$=prottop&blast_rank=15&RID=EMFCRMMP016)

[KIP05965.1](https://www.ncbi.nlm.nih.gov/protein/754373277?report=genbank&log$=prottop&blast_rank=16&RID=EMFCRMMP016)

[XP_007365638.1](https://www.ncbi.nlm.nih.gov/protein/597989709?report=genbank&log$=prottop&blast_rank=17&RID=EMFCRMMP016)

[KIJ36601.1](https://www.ncbi.nlm.nih.gov/protein/749866254?report=genbank&log$=prottop&blast_rank=18&RID=EMFCRMMP016)

[XP_007301879.1](https://www.ncbi.nlm.nih.gov/protein/597909160?report=genbank&log$=prottop&blast_rank=19&RID=EMFCRMMP016)

[KLO19190.1](https://www.ncbi.nlm.nih.gov/protein/827764605?report=genbank&log$=prottop&blast_rank=20&RID=EMFCRMMP016)

[XP_007398636.1](https://www.ncbi.nlm.nih.gov/protein/599388980?report=genbank&log$=prottop&blast_rank=21&RID=EMFCRMMP016)

[XP_007398594.1](https://www.ncbi.nlm.nih.gov/protein/599388753?report=genbank&log$=prottop&blast_rank=22&RID=EMFCRMMP016)

[KII83623.1](https://www.ncbi.nlm.nih.gov/protein/749758549?report=genbank&log$=prottop&blast_rank=23&RID=EMFCRMMP016)

[KTB30557.1](https://www.ncbi.nlm.nih.gov/protein/961953693?report=genbank&log$=prottop&blast_rank=24&RID=EMFCRMMP016)

[KLO19194.1](https://www.ncbi.nlm.nih.gov/protein/827764609?report=genbank&log$=prottop&blast_rank=25&RID=EMFCRMMP016)

[KDQ24008.1](https://www.ncbi.nlm.nih.gov/protein/646302859?report=genbank&log$=prottop&blast_rank=26&RID=EMFCRMMP016)

[XP_007849490.1](https://www.ncbi.nlm.nih.gov/protein/630193970?report=genbank&log$=prottop&blast_rank=27&RID=EMFCRMMP016)

[XP_007299648.1](https://www.ncbi.nlm.nih.gov/protein/597904698?report=genbank&log$=prottop&blast_rank=28&RID=EMFCRMMP016)

[KIK64396.1](https://www.ncbi.nlm.nih.gov/protein/751022693?report=genbank&log$=prottop&blast_rank=29&RID=EMFCRMMP016)

[KLO19195.1](https://www.ncbi.nlm.nih.gov/protein/827764610?report=genbank&log$=prottop&blast_rank=30&RID=EMFCRMMP016)

[KLO19184.1](https://www.ncbi.nlm.nih.gov/protein/827764599?report=genbank&log$=prottop&blast_rank=31&RID=EMFCRMMP016)

[KIP06793.1](https://www.ncbi.nlm.nih.gov/protein/754374124?report=genbank&log$=prottop&blast_rank=32&RID=EMFCRMMP016)

[XP_007269649.1](https://www.ncbi.nlm.nih.gov/protein/595782212?report=genbank&log$=prottop&blast_rank=33&RID=EMFCRMMP016)

[OCB83900.1](https://www.ncbi.nlm.nih.gov/protein/1044609340?report=genbank&log$=prottop&blast_rank=34&RID=EMFCRMMP016)

[KIK53500.1](https://www.ncbi.nlm.nih.gov/protein/751011730?report=genbank&log$=prottop&blast_rank=35&RID=EMFCRMMP016)

[KIK71049.1](https://www.ncbi.nlm.nih.gov/protein/751029358?report=genbank&log$=prottop&blast_rank=36&RID=EMFCRMMP016)

[CDO78110.1](https://www.ncbi.nlm.nih.gov/protein/691784102?report=genbank&log$=prottop&blast_rank=37&RID=EMFCRMMP016)

[XP_007269671.1](https://www.ncbi.nlm.nih.gov/protein/595782256?report=genbank&log$=prottop&blast_rank=38&RID=EMFCRMMP016)

[KIK64397.1](https://www.ncbi.nlm.nih.gov/protein/751022694?report=genbank&log$=prottop&blast_rank=39&RID=EMFCRMMP016)

[KIP01598.1](https://www.ncbi.nlm.nih.gov/protein/754368677?report=genbank&log$=prottop&blast_rank=40&RID=EMFCRMMP016)

[KIP03510.1](https://www.ncbi.nlm.nih.gov/protein/754370739?report=genbank&log$=prottop&blast_rank=41&RID=EMFCRMMP016)

[XP_007311301.1](https://www.ncbi.nlm.nih.gov/protein/618817452?report=genbank&log$=prottop&blast_rank=42&RID=EMFCRMMP016)

[CDO71225.1](https://www.ncbi.nlm.nih.gov/protein/691793645?report=genbank&log$=prottop&blast_rank=43&RID=EMFCRMMP016)

https://blast.ncbi.nlm.nih.gov/Blast.cgi

2/7


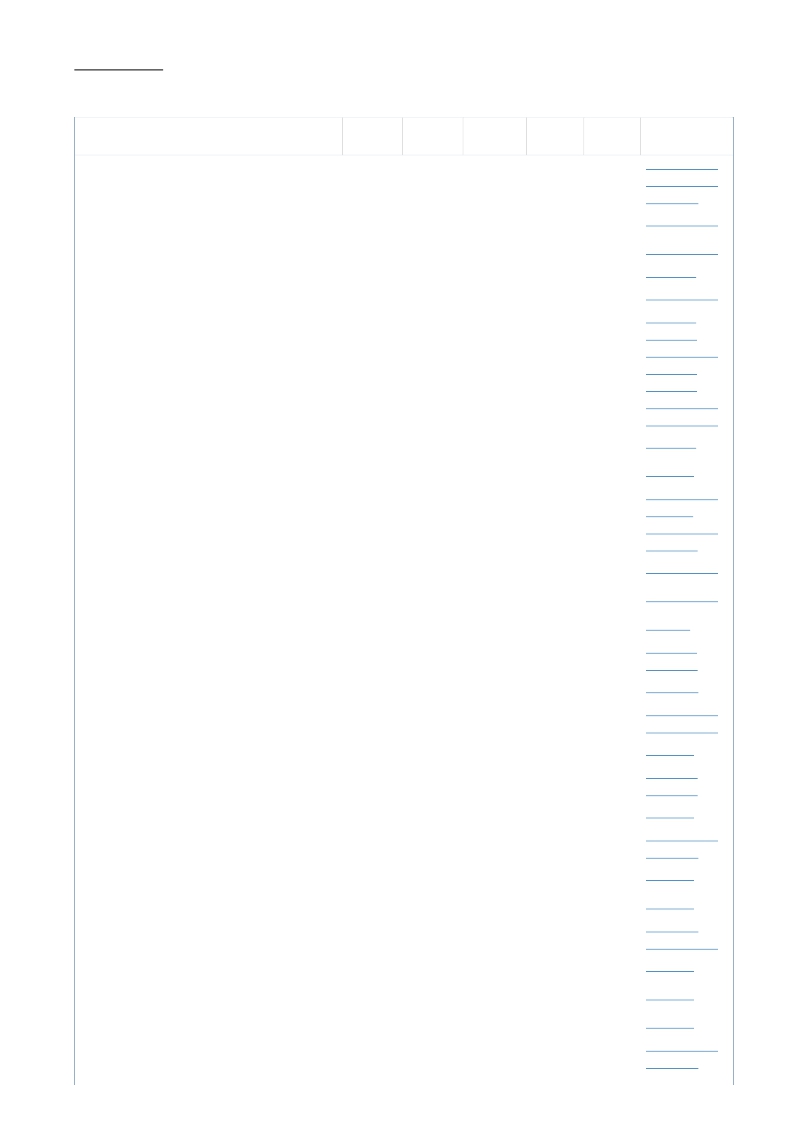


2017/4/9

NCBI Blast:GL25583-R1_1

GMC oxidoreductase [Amanita muscaria Koide BX008]

gmc oxidoreductase [Moniliophthora roreri MCA 2997]

hypothetical protein GYMLUDRAFT_160209 [Gymnopus

luxurians FD-317 M1]

466

463

461

466

463

461

93%

94%

89%

3e-154

6e-153

8e-153

44%

43%

43%

[KIL63993.1](https://www.ncbi.nlm.nih.gov/protein/751178027?report=genbank&log$=prottop&blast_rank=44&RID=EMFCRMMP016)

[XP_007849498.1](https://www.ncbi.nlm.nih.gov/protein/630193994?report=genbank&log$=prottop&blast_rank=45&RID=EMFCRMMP016)

[KIK65197.1](https://www.ncbi.nlm.nih.gov/protein/751023496?report=genbank&log$=prottop&blast_rank=46&RID=EMFCRMMP016)

hypothetical protein PLICRDRAFT_47109 [Plicaturopsis crispa

FD-325 SS-3]

GMC oxidoreductase [Cylindrobasidium torrendii FP15055 ss-

10]

alcohol oxidase [Coniophora puteana RWD-64-598 SS2]

alcohol oxidase, partial [Auricularia subglabra TFB-10046

SS5]

choline dehydrogenase [Rhizoctonia solani AG-3 Rhs1AP]

GMC oxidoreductase [Peniophora sp. CONT]

Oxygen-dependent choline dehydrogenase [Rhizoctonia

solani]

glucose dehydrogenase [Rhizoctonia solani 123E]

gmc oxidoreductase [Moniliophthora roreri MCA 2997]

Oxygen-dependent choline dehydrogenase [Rhizoctonia

solani]

glucose dehydrogenase [Rhizoctonia solani AG-3 Rhs1AP]

glucose dehydrogenase [Rhizoctonia solani AG-3 Rhs1AP]

hypothetical protein PLICRDRAFT_47110 [Plicaturopsis crispa

FD-325 SS-3]

Glucose oxidase OS=Talaromyces flavus GN=GOX PE=3 SV=1

[Rhizoctonia solani AG-1 IB]

GMC oxidoreductase [Cylindrobasidium torrendii FP15055 ss-

10]

Glucose oxidase [Rhizoctonia solani]

glucose dehydrogenase [Rhizoctonia solani 123E]

choline dehydrogenase-like protein [Rhizoctonia solani

123E]

hypothetical protein RSAG8_00385 [Rhizoctonia solani AG-8

WAC10335]

hypothetical protein BOTBODRAFT_159613 [Botryobasidium

botryosum FD-172 SS1]

Oxygen-dependent choline dehydrogenase [Rhizoctonia

solani]

choline dehydrogenase [Pseudozyma hubeiensis SY62]

hypothetical protein WG66_3713 [Moniliophthora roreri]

putative GMC-type oxidoreductase Rv1279/MT1316

OS=Mycobacterium tuberculosis GN=Rv1279 PE=3 SV=1

455

454

453

450

448

444

443

444

443

442

442

441

441

441

439

440

439

438

437

437

438

436

436

436

455

454

453

450

448

444

443

444

443

442

442

441

441

441

439

440

439

438

437

437

438

436

436

436

94%

95%

94%

92%

95%

95%

94%

94%

94%

94%

94%

94%

94%

94%

94%

94%

94%

94%

94%

94%

94%

93%

93%

94%

5e-150

1e-149

4e-149

2e-148

5e-147

2e-145

3e-145

4e-145

5e-145

1e-144

2e-144

3e-144

4e-144

5e-144

8e-144

1e-143

2e-143

2e-143

5e-143

6e-143

6e-143

1e-142

1e-142

1e-142

44%

41%

41%

43%

43%

40%

41%

43%

42%

43%

43%

41%

44%

43%

42%

41%

41%

40%

40%

43%

42%

40%

42%

40%

[KII83632.1](https://www.ncbi.nlm.nih.gov/protein/749758558?report=genbank&log$=prottop&blast_rank=47&RID=EMFCRMMP016)

[KIY66922.1](https://www.ncbi.nlm.nih.gov/protein/761947182?report=genbank&log$=prottop&blast_rank=48&RID=EMFCRMMP016)

[XP_007765185.1](https://www.ncbi.nlm.nih.gov/protein/628829763?report=genbank&log$=prottop&blast_rank=49&RID=EMFCRMMP016)

[XP_007345455.1](https://www.ncbi.nlm.nih.gov/protein/598032165?report=genbank&log$=prottop&blast_rank=50&RID=EMFCRMMP016)

[EUC58259.1](https://www.ncbi.nlm.nih.gov/protein/576985523?report=genbank&log$=prottop&blast_rank=51&RID=EMFCRMMP016)

[KZV68632.1](https://www.ncbi.nlm.nih.gov/protein/1024075658?report=genbank&log$=prottop&blast_rank=52&RID=EMFCRMMP016)

[CUA75055.1](https://www.ncbi.nlm.nih.gov/protein/924117089?report=genbank&log$=prottop&blast_rank=53&RID=EMFCRMMP016)

[KEP47069.1](https://www.ncbi.nlm.nih.gov/protein/660960407?report=genbank&log$=prottop&blast_rank=54&RID=EMFCRMMP016)

[XP_007855909.1](https://www.ncbi.nlm.nih.gov/protein/630213700?report=genbank&log$=prottop&blast_rank=55&RID=EMFCRMMP016)

[CUA76134.1](https://www.ncbi.nlm.nih.gov/protein/924115796?report=genbank&log$=prottop&blast_rank=56&RID=EMFCRMMP016)

[EUC63668.1](https://www.ncbi.nlm.nih.gov/protein/576990963?report=genbank&log$=prottop&blast_rank=57&RID=EMFCRMMP016)

[EUC58478.1](https://www.ncbi.nlm.nih.gov/protein/576985742?report=genbank&log$=prottop&blast_rank=58&RID=EMFCRMMP016)

[KII83633.1](https://www.ncbi.nlm.nih.gov/protein/749758559?report=genbank&log$=prottop&blast_rank=59&RID=EMFCRMMP016)

[CEL58759.1](https://www.ncbi.nlm.nih.gov/protein/751834042?report=genbank&log$=prottop&blast_rank=60&RID=EMFCRMMP016)

[KIY70729.1](https://www.ncbi.nlm.nih.gov/protein/761951065?report=genbank&log$=prottop&blast_rank=61&RID=EMFCRMMP016)

[CUA69309.1](https://www.ncbi.nlm.nih.gov/protein/924123918?report=genbank&log$=prottop&blast_rank=62&RID=EMFCRMMP016)

[KEP46778.1](https://www.ncbi.nlm.nih.gov/protein/660959781?report=genbank&log$=prottop&blast_rank=63&RID=EMFCRMMP016)

[KEP50605.1](https://www.ncbi.nlm.nih.gov/protein/660965998?report=genbank&log$=prottop&blast_rank=64&RID=EMFCRMMP016)

[KDN51836.1](https://www.ncbi.nlm.nih.gov/protein/639574471?report=genbank&log$=prottop&blast_rank=65&RID=EMFCRMMP016)

[KDQ14400.1](https://www.ncbi.nlm.nih.gov/protein/646293225?report=genbank&log$=prottop&blast_rank=66&RID=EMFCRMMP016)

[CUA69183.1](https://www.ncbi.nlm.nih.gov/protein/924124170?report=genbank&log$=prottop&blast_rank=67&RID=EMFCRMMP016)

[XP_012189838.1](https://www.ncbi.nlm.nih.gov/protein/808369080?report=genbank&log$=prottop&blast_rank=68&RID=EMFCRMMP016)

[KTB43708.1](https://www.ncbi.nlm.nih.gov/protein/961974475?report=genbank&log$=prottop&blast_rank=69&RID=EMFCRMMP016)

[CEL51966.1](https://www.ncbi.nlm.nih.gov/protein/751842268?report=genbank&log$=prottop&blast_rank=70&RID=EMFCRMMP016)

[Rhizoctonia solani AG-1 IB]

Pyranose dehydrogenase 1 [Rhizoctonia solani]

choline dehydrogenase [Kalmanozyma brasiliensis

GHG001]

choline dehydrogenase-like protein [Rhizoctonia solani AG-3

Rhs1AP]

glucose dehydrogenase [Rhizoctonia solani 123E]

hypothetical protein UMAG_01711 [Ustilago maydis 521]

glucose dehydrogenase [Rhizoctonia solani AG-3 Rhs1AP]

GMC oxidoreductase [Amanita muscaria Koide BX008]

hypothetical protein RSAG8_05616 [Rhizoctonia solani AG-8

WAC10335]

hypothetical protein [Sporisorium scitamineum]

glucose oxidase-like protein [Rhizoctonia solani 123E]

Alcohol oxidase OS=Pichia angusta GN=MOX PE=3 SV=1

[Rhizoctonia solani AG-1 IB]

related to Glucose dehydrogenase [acceptor] precursor

[Ustilago bromivora]

related to Glucose dehydrogenase [acceptor] precursor

[Sporisorium reilianum SRZ2]

hypothetical protein RSOLAG22IIIB_08312 [Rhizoctonia

solani]

hypothetical protein RSOLAG22IIIB_08436 [Rhizoctonia

solani]

hypothetical protein PFL1_03618 [Anthracocystis flocculosa

PF-1]

related to Glucose dehydrogenase [acceptor] precursor

[Melanopsichium pennsylvanicum 4]

436

436

436

436

434

435

433

433

432

432

446

429

429

430

429

429

426

436

436

436

436

434

435

433

433

432

432

446

429

429

430

429

429

426

94%

93%

94%

94%

93%

94%

93%

93%

93%

94%

94%

93%

93%

97%

97%

93%

93%

2e-142

2e-142

3e-142

8e-142

8e-142

9e-142

9e-142

2e-141

6e-141

6e-141

9e-141

6e-140

6e-140

9e-140

1e-139

2e-139

2e-138

41%

40%

40%

42%

40%

43%

43%

42%

39%

41%

42%

39%

39%

42%

42%

40%

39%

[CUA71924.1](https://www.ncbi.nlm.nih.gov/protein/924120541?report=genbank&log$=prottop&blast_rank=71&RID=EMFCRMMP016)

[XP_016292663.1](https://www.ncbi.nlm.nih.gov/protein/1023181956?report=genbank&log$=prottop&blast_rank=72&RID=EMFCRMMP016)

[EUC62082.1](https://www.ncbi.nlm.nih.gov/protein/576989363?report=genbank&log$=prottop&blast_rank=73&RID=EMFCRMMP016)

[KEP48939.1](https://www.ncbi.nlm.nih.gov/protein/660963617?report=genbank&log$=prottop&blast_rank=74&RID=EMFCRMMP016)

[XP_011387685.1](https://www.ncbi.nlm.nih.gov/protein/758975261?report=genbank&log$=prottop&blast_rank=75&RID=EMFCRMMP016)

[EUC61273.1](https://www.ncbi.nlm.nih.gov/protein/576988549?report=genbank&log$=prottop&blast_rank=76&RID=EMFCRMMP016)

[KIL60682.1](https://www.ncbi.nlm.nih.gov/protein/751174674?report=genbank&log$=prottop&blast_rank=77&RID=EMFCRMMP016)

[KDN44352.1](https://www.ncbi.nlm.nih.gov/protein/639566854?report=genbank&log$=prottop&blast_rank=78&RID=EMFCRMMP016)

[CDW97339.1](https://www.ncbi.nlm.nih.gov/protein/858056339?report=genbank&log$=prottop&blast_rank=79&RID=EMFCRMMP016)

[KEP51005.1](https://www.ncbi.nlm.nih.gov/protein/660966535?report=genbank&log$=prottop&blast_rank=80&RID=EMFCRMMP016)

[CEL58768.1](https://www.ncbi.nlm.nih.gov/protein/751834051?report=genbank&log$=prottop&blast_rank=81&RID=EMFCRMMP016)

[SAM80724.1](https://www.ncbi.nlm.nih.gov/protein/1098865512?report=genbank&log$=prottop&blast_rank=82&RID=EMFCRMMP016)

[CBQ71929.1](https://www.ncbi.nlm.nih.gov/protein/343428399?report=genbank&log$=prottop&blast_rank=83&RID=EMFCRMMP016)

[CUA69185.1](https://www.ncbi.nlm.nih.gov/protein/924124172?report=genbank&log$=prottop&blast_rank=84&RID=EMFCRMMP016)

[CUA69312.1](https://www.ncbi.nlm.nih.gov/protein/924123921?report=genbank&log$=prottop&blast_rank=85&RID=EMFCRMMP016)

[XP_007879328.1](https://www.ncbi.nlm.nih.gov/protein/630965840?report=genbank&log$=prottop&blast_rank=86&RID=EMFCRMMP016)

[CDI52576.1](https://www.ncbi.nlm.nih.gov/protein/673526184?report=genbank&log$=prottop&blast_rank=87&RID=EMFCRMMP016)

https://blast.ncbi.nlm.nih.gov/Blast.cgi

3/7


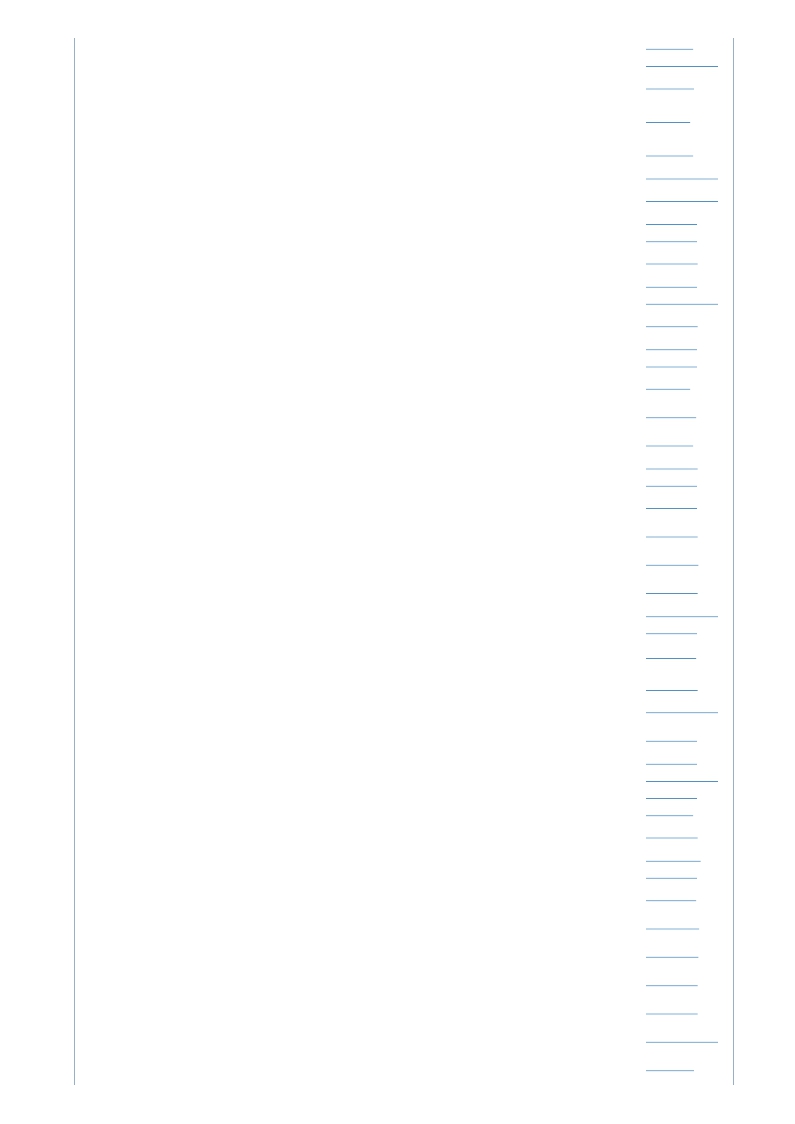


2017/4/9

NCBI Blast:GL25583-R1_1

Oxygen-dependent choline dehydrogenase [Rhizoctonia

solani]

hypothetical protein PaG_02698 [Moesziomyces aphidis DSM

70725]

related to Glucose dehydrogenase [acceptor] precursor

[Ustilago hordei]

glucose dehydrogenase [Rhizoctonia solani 123E]

glucose dehydrogenase [Rhizoctonia solani AG-3 Rhs1AP]

GMC oxidoreductase [Coprinopsis cinerea okayama7#130]

glucose dehydrogenase [Rhizoctonia solani AG-3 Rhs1AP]

choline dehydrogenase [Rhizoctonia solani AG-3 Rhs1AP]

L-sorbose 1-dehydrogenase [Hypsizygus marmoreus]

L-sorbose 1-dehydrogenase OS=Gluconobacter oxydans PE=3

SV=1 [Rhizoctonia solani AG-1 IB]

hypothetical protein PANT_22d00133 [Moesziomyces

antarcticus T-34]

GMC oxidoreductase [Hypholoma sublateritium FD-334 SS-

4]

hypothetical protein BOTBODRAFT_126222 [Botryobasidium

botryosum FD-172 SS1]

426

440

425

424

423

422

423

422

419

419

434

416

417

426

440

425

424

423

422

423

422

419

419

434

416

417

97%

95%

93%

98%

94%

94%

98%

94%

94%

94%

95%

85%

94%

2e-138

2e-138

5e-138

2e-137

3e-137

4e-137

4e-137

6e-137

4e-136

6e-136

7e-136

1e-135

4e-135

41%

40%

39%

40%

41%

41%

40%

42%

42%

40%

40%

43%

42%

[CUA70878.1](https://www.ncbi.nlm.nih.gov/protein/924121711?report=genbank&log$=prottop&blast_rank=88&RID=EMFCRMMP016)

[ETS62929.1](https://www.ncbi.nlm.nih.gov/protein/573029705?report=genbank&log$=prottop&blast_rank=89&RID=EMFCRMMP016)

[CCF53417.1](https://www.ncbi.nlm.nih.gov/protein/388852969?report=genbank&log$=prottop&blast_rank=90&RID=EMFCRMMP016)

[KEP48703.1](https://www.ncbi.nlm.nih.gov/protein/660963257?report=genbank&log$=prottop&blast_rank=91&RID=EMFCRMMP016)

[EUC63663.1](https://www.ncbi.nlm.nih.gov/protein/576990958?report=genbank&log$=prottop&blast_rank=92&RID=EMFCRMMP016)

[XP_001834096.1](https://www.ncbi.nlm.nih.gov/protein/169854849?report=genbank&log$=prottop&blast_rank=93&RID=EMFCRMMP016)

[EUC63650.1](https://www.ncbi.nlm.nih.gov/protein/576990945?report=genbank&log$=prottop&blast_rank=94&RID=EMFCRMMP016)

[EUC63653.1](https://www.ncbi.nlm.nih.gov/protein/576990948?report=genbank&log$=prottop&blast_rank=95&RID=EMFCRMMP016)

[KYQ35206.1](https://www.ncbi.nlm.nih.gov/protein/1012960432?report=genbank&log$=prottop&blast_rank=96&RID=EMFCRMMP016)

[CEL58702.1](https://www.ncbi.nlm.nih.gov/protein/751830402?report=genbank&log$=prottop&blast_rank=97&RID=EMFCRMMP016)

[GAC76671.1](https://www.ncbi.nlm.nih.gov/protein/443899340?report=genbank&log$=prottop&blast_rank=98&RID=EMFCRMMP016)

[KJA26213.1](https://www.ncbi.nlm.nih.gov/protein/763728802?report=genbank&log$=prottop&blast_rank=99&RID=EMFCRMMP016)

[KDQ19381.1](https://www.ncbi.nlm.nih.gov/protein/646298225?report=genbank&log$=prottop&blast_rank=100&RID=EMFCRMMP016)

Alignments

GMC oxidoreductase [Trametes versicolor FP-101664 SS1]

Sequence ID: XP_008036579.1 Length: 578 Number of Matches: 1

See 1 more title(s)

Range 1: 1 to 566

Score

Expect Method

Identities

Positives

Gaps

Frame

639 bits(1647) 0.0()

Features:

Compositional matrix adjust. 324/579(56%) 412/579(71%) 18/579(3%)

Query 24

Sbjct 1

Query 84

Sbjct 61

MSANLTPEHFASTTFDYIVVGGGTAGLVVAARLSEDPNVTVGVIEAGEWEPNLPNINIPG 83

M++ TEFASTTFDYI+VGGGTGLV+AARLSEDPVTVGIEAGEW + I++PG

MTSPATAEVFASTTFDYIIVGGGTTGLVLAARLSEDPAVTVGAIEAGEWHRGVEGISVPG 60

LSGSIIGNPKYDWSFLTVPQKGANGRQIYHPRGKVVGGSSAMNI-LIYDRASAREYDAIE 142

LGS+IGNP+YDWSF+PQK NRI+PRGK+GGSS++ RASAREYDA+E

LCGSLIGNPQYDWSFSSDPQKHVNDRSIFQPRGKALGGSSMVSKPECSHRASAREYDALE 120

Query 143 ALGNPGWNWSEFLKYFKKAETTLPPDPDVAAQHHL----RDIDPQFHGSSGPLVKSYSAH 198

LGNPGWNW+EFLKYKKAETT+P DV +++ RD ++HG+SGP+VKSY +

Sbjct 121 TLGNPGWNWAEFLKYMKKAETTIPLSSDVRPEYGIPATQRD---EWHGNSGPVVKSYPTN 177

Query 199 YSIGTLHDPLLETFKNLDVPINYDANDGNKLGFTSTFTTVDSRTAIRTYSGKSYLEPNAG 258

++ TLH ++ +LVPN+++G +G +TF VDRTAR+YS +YEPNAG

Sbjct 178 FN--TLHIHITDALETLGVPKNPEPSNGINVGSVTTFAAVDPRTATRSYSANAYFEPNAG 235

Query 259 RKNLVVLTGATVTRVTFLPGSSPLKATGVEFLKGEKTYRVAVAKEVLLAAGTFQTPQILE 318

RKNLVVTG++V+R+FPSSPLATGVEF+G+T+ KEV+LAGFQ+PQ+LE

Sbjct 236 RKNLVVTTGSSVSRIIFRPESSPLLATGVEFIHGDSTFTAMARKEVILCAGVFQSPQLLE 295

Query 319 LSGIGNKDILAKHGIETLIDNSSVGENLHVSQDHCWTYSIREIDPKHETFDQAQQPERVA 378

LSGIG DILK+GI+L+D VGENLV H EIDPK+ETD QPE

Sbjct 296 LSGIGKGDILNKYGIKILLDLPGVGENLRVIMIH-------EIDPKYETVDFLQDPEESK 348

Query 379 LAQQLYQSQKGPLSSAPSVIFAFLNAKTFASKEQVDQWKRDVQTMASAVPQGLKKLHEKQ 438

LQ+LY++QKGLSSA++ F+AK AS+QV+WK + APG+KK EQ

Sbjct 349 LQQELYKAQKGYLSSALATVLGFVPAKALASDDQVRKWKEMGENAILAAPPGVKKQLELQ 408

Query 439 SEWLSNDNSVEGELMLFAGFLPFAGLKAPSETAHYMTIVSTITHPLSRGSVHIVSADPTA 498

+WL++SEEL+FGF GLKP Y++++T HPLSRGSVHIVSADP

Sbjct 409 MKWLDDETSAEAELIPFPGFFLPCGLK-PEPNTRYSSVLATTMHPLSRGSVHIVSADPKH 467

Query 499 PPAIDPNYLAHPQDLQMALSLMKFAHKVFETKPLADDVRQLVTPSPEQAATDEGLIEHIK 558

PPAIDPNYA+PDL+ML+L+KF K+++TPLDVR+VPSPEQ+ATDEL+++IK

Sbjct 468 PPAIDPNYFANPVDLEMMLALIKFTLKLYQTTPLCDVVRKQVAPSPEQSATDEALVKYIK 527

Query 559 NSCSCVFHPVGTTSMLPREDGGVVDPQLRVYGTANLRVV 597

++C+V+HP+G++MLPREDGGVVPLRVYGTANLRVV

Sbjct 528 DNCNTVYHPIGSAAMLPREDGGVVSPDLRVYGTANLRVV 566

alcohol oxidase [Dichomitus squalens LYAD-421 SS1]

Sequence ID: XP_007361326.1 Length: 617 Number of Matches: 1

See 1 more title(s)

Range 1: 1 to 604

Score

Expect Method

Identities

Positives

Gaps

Frame

609 bits(1571) 0.0()

Features:

Compositional matrix adjust. 313/609(51%) 415/609(68%) 15/609(2%)

Query 28

Sbjct 1

Query 86

Sbjct 61

LTPEHFAS--TTFDYIVVGGGTAGLVVAARLSEDPNVTVGVIEAGEWEPNLPNINIPGLS 85

+TP+FS FDY+VVGGGTAGLVVA+RLSEDPVVGV+EAGEW ++ ++PGL+

MTPDQFCSQDMRFDYVVVGGGTAGLVVASRLSEDPEVRVGVLEAGEWHRDVEAVTVPGLT 60

GSIIGNPKYDWSFLTVPQKGANGRQIYHPRGKVVGGSSAMNILIYDRASAREYDAIEALG 145

G++G+PYDWF+VPQK+NGR++ PRGK+GGSS+N +RASAREYDA++LG

GTTLGDPNYDWRFTSVPQKYSNGRRVPQPRGKGLGGSSLINFTGLNRASAREYDAMQDLG 120

Query 146 NPGWNWSEFLKYFKKAETTLPPDPD---VAAQHHLRDIDPQFHGSSGPLVKSYSAHYSIG 202

https://blast.ncbi.nlm.nih.gov/Blast.cgi

4/7


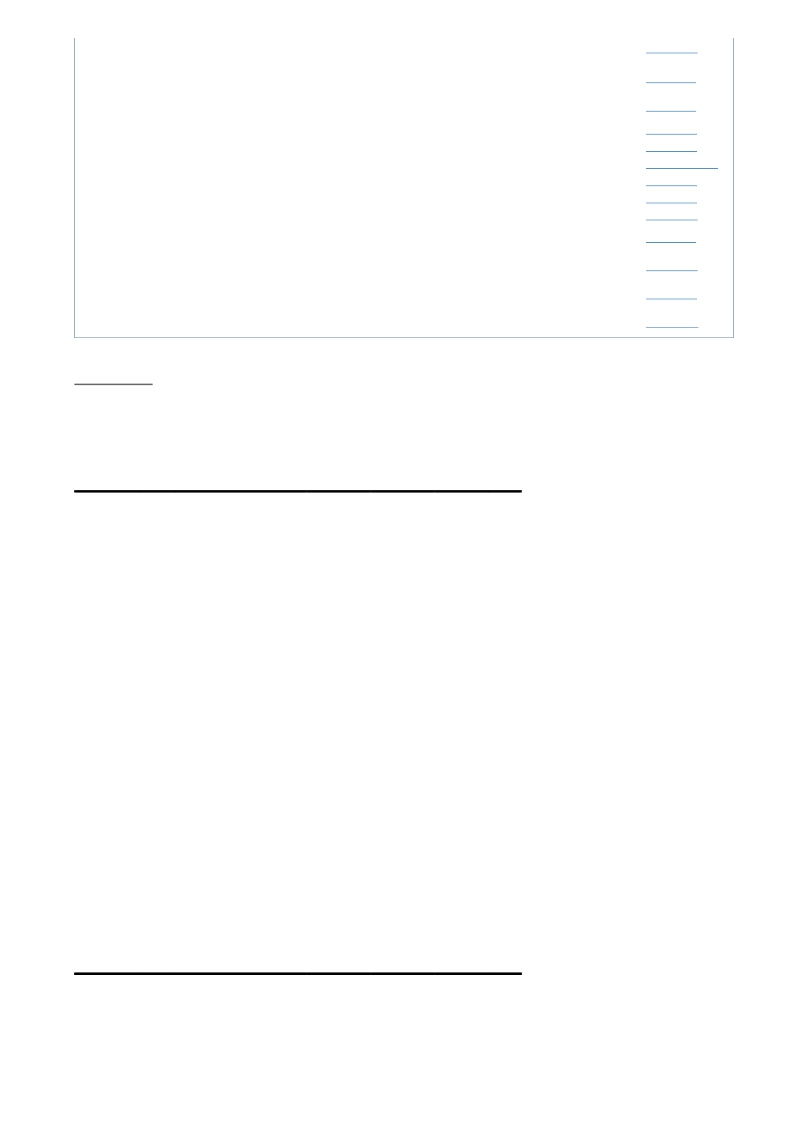


2017/4/9

NCBI Blast:GL25583-R1_1

Query 146 NPGWNWSEFLKYFKKAETTLPPDPD---VAAQHHLRDIDPQFHGSSGPLVKSYSAHYSIG 202

NPGWNWELKYKKETPP + A HLDDP++HG+SGPLKYS++

Sbjct 121 NPGWNWQEMLKYMKKGERTQPPLEERQATAESHLLVDPDPRWHGTSGPLAKGYSTYFP-- 178

Query 203 TLHDPLLETFKNLDVPINYDANDG-NKLGFTSTFTTVDSRTAIRTYSGKSYLEPNAGRKN 261

LHP+++ ++LV N++NDG++G +F++VD+TAR++S +YEP RN

Sbjct 179 ALHVPIVDALESLGVKKNFEPNDGTSTVGSHTIFSSVDPQTATRSHSSAAYYEPCKDRPN 238

Query 262 LVVLTGATVTRVTFLPGSSPLKATGVEFLKGEKTYRVAVAKEVLLAAGTFQTPQILELSG 321

LVLG+ +R+F GSPLAGVEFL KYV+ +EV+LAGFQ+PQILELSG

Sbjct 239 LKVLPGSLASRIRFRQGSHPLVAEGVEFLNAGKQYLVSAEREVILCAGAFQSPQILELSG 298

Query 322 IGNKDILAKHGIETLIDNSSVGENLHVSQDHCWTYSIREIDPKHETFDQAQQPERVALAQ 381

IGK++L+ GIETL+D VGENL QDH+ ++EID ETDA+P +

Sbjct 299 IGKKEVLSSCGIETLVDLPGVGENL---QDHPYICTVYEIDDNIETIDAAAEPAWFTQQR 355

Query 382 QLYQSQ-KGPLSSAPSVIFAFLNAKTFASKEQVDQWKRDVQTMASAVPQGLKKLHEKQSE 440

+LY +KGLSS S++AFL+KFASEQV+WK QT PGLKK QS+

Sbjct 356 KLYDEELKGYLSSNLSPLYAFLPSKAFASVEQVKRWKAQFQTCILDAPSGLKKQLAIQSK 415

Query 441 WL--SNDNSVEGELMLFAGFLPFAGLKAPSETA-HYMTIVSTITHPLSRGSVHIVSADPT 497

W + SEGE++FG+ GL E Y+++ ++PLSRGSVHIS++PT

Sbjct 416 WFLEPDSPSAEGEVIPFNGYFVTPGLPIKMEPGKRYSSMLCAVMNPLSRGSVHITSSEPT 475

Query 498 APPAIDPNYLAHPQDLQMALSLMKFAHKVFETKPLADDVRQLVTPSPEQAATDEGLIEHI 557

APPAIDPN ++ DL+ML+++KFA+V+ETP+ V++P+E TD+LE+I

Sbjct 476 APPAIDPNCFSNALDLEMLLAVLKFAREVYETSPIRQHVVRRIMPTVEDYRTDDTLKEYI 535

Query 558 KNSCSCVFHPVGTTSMLPREDGGVVDPQLRVYGTANLRVVDASILPMQLSAHTQGTVYAL 617

KN CV+HPVGT+M+P+DGGVVDP+L+VYGTNLRVVDASILPMQ++AHTQTVY+

Sbjct 536 KNGLGCVYHPVGTAAMMPQNDGGVVDPELKVYGTKNLRVVDASILPMQIAAHTQATVYGI 595

Query 618 AEKAADVIK 626

AEKAD+IK

Sbjct 596 AEKGADIIK 604

GMC oxidoreductase [Trametes cinnabarina]

Sequence ID: CDO72925.1 Length: 574 Number of Matches: 1

Range 1: 7 to 572

Score

Expect Method

Identities

Positives

Gaps

Frame

590 bits(1521) 0.0()

Features:

Compositional matrix adjust. 307/618(50%) 400/618(64%) 72/618(11%)

Query 29

Sbjct 7

Query 89

Sbjct 67

TPEHFASTTFDYIVVGGGTAGLVVAARLSEDPNVTVGVIEAGEWEPNLPNINIPGLSGSI 88

TPEFASTFDYIVVGGGTAGLV+AARLSEDP+ VGVIEAGW+P++ INIPGLGSI

TPEGFASTEFDYIVVGGGTAGLVIAARLSEDPSKVVGVIEAGNWDPDVNAINIPGLGGSI 66

IGNPKYDWSFLTVPQKGANGRQIYHPRGKVVGGSSAMNILIYDRASAREYDAIEALGNPG 148

+GNP+YDW+F++VPQKANR++PRGKVGGSS+N+LYRA+AREYDAIEALGNPG

LGNPRYDWAFMSVPQKYANNRPVFQPRGKAVGGSSMLNLLGYSRAAAREYDAIEALGNPG 126

Query 149 WNWSEFLKYFKKAETTLPPDPDVAAQHHLRDIDPQFHGSSGPLVKSYSAHYSIGTLHDPL 208

WNWSEFLKYKKETTLP ++A+HL D +HG+GP++KY ++ LHP

Sbjct 127 WNWSEFLKYLKKTETTLLPS-ELAKEHGLVAADATYHGDAGPIIKQYPTWFN--PLHKPF 183

Query 209 LETFKNLDVPINYDANDGNKLGFTSTFTTVDSRTAIRTYSGKSYLEPNAGRKNLVVLTGA 268

LET+++PND+G +G +++TVDS AR+YS Y PAGR+NLV+LT+

Sbjct 184 LETLEKIGIPKNADPDSGVNMGGVTSYMTVDS-DATRSYSASGYYLPIAGRQNLVLLTNS 242

Query 269 TVTRVTFLPGSSPLKATGVEFLKGEKTYRVAVAKEVLLAAGTFQTPQILELSGIGNKDIL 328

V++F +SPL+ GVEF+ +KY KEV+LAA GIGNKDIL

Sbjct 243 KVAKIIFREDTSPLRVIGVEFINVDKRYSATARKEVVLAA------------GIGNKDIL 290

Query 329 AKHGIETLIDNSSVGENLHVSQDHCWTYSIREIDPKHETFDQAQQPERVALAQQLYQSQK 388

+KHGI+L+D + DH+YS+EIDP ETDA PE+A Q+LY+ +

Sbjct 291 SKHGIKVLLD---------LPNDHVYVYSVYEIDPSIETVDDALVPEVMARQQELYKVHR 341

Query 389 GPLSSAPSVIFAFLNAKTFASKEQVDQWKRDVQTMASAVPQGLKKLHEKQSEWLSNDNSV 448

GLSS ++F+LA+FA++Q++WK+ +A PGL+K EQ+W N S

Sbjct 342 GYLSSGLAAVFGYLPARAFATEAQLAEWKQRALAIAKEAPHGLRKQLEIQIDWFLNPESA 401

Query 449 EGE--LMLFAGFLPFAGLKAPSETAHYMTIVSTITHPLSRGSVHIVSADPTAPPAIDPNY 506

EE LL+ FP + ++ADPTAPAIDPNY

Sbjct 402 ESERPLFLYGCFPP---------------------------ASFVSAADPTAAPAIDPNY 434

Query 507 LAHPQDLQMALSLMKFA-HKVFETKPLADDVRQLVTPSPEQAATDEGLIEHIKNSCSCVF 565

++PDL+ML+ KF K+++TP DVR++VTPPEAA+D LE+IK++CCV+

Sbjct 435 FSNPLDLEMLLNAFKFTLDKLYKTSPFGDVVRKIVTPRPEVAASDAALEEYIKDNCGCVY 494

Query 566 HPVGTTSMLPREDGGVVDPQLRVYGTANL-----------------RVVDASILPMQLSA 608

HP+GTSMLP+EDGGVVDP+L+VYGTN+ RVDAS++P++LSA

Sbjct 495 HPLGTASMLPQEDGGVVDPELKVYGTVNVRVVRPIIRRRLHVADWERQVDASVIPLELSA 554

Query 609 HTQGTVYALAEKAADVIK 626

HTQTVYAAEKAAD+IK

Sbjct 555 HTQATVYATAEKAADIIK 572

alcohol oxidase [Punctularia strigosozonata HHB-11173 SS5]

Sequence ID: XP_007384326.1 Length: 614 Number of Matches: 1

See 1 more title(s)

Range 1: 7 to 610

Score

Expect Method

Identities

Positives

Gaps

Frame

568 bits(1465) 0.0()

Features:

Compositional matrix adjust. 301/613(49%) 409/613(66%) 26/613(4%)

Query 30

Sbjct 7

Query 90

Sbjct 67

PEHFASTTFDYIVVGGGTAGLVVAARLSEDPNVTVGVIEAGEWEPNLPNINIPGLSGSII 89

PEFA+ FDY+V+GGGTAGLVVA+RL+EDP++VGVIEAGEW +P+NIPGL+G +

PEIFAAAEFDYVVIGGGTAGLVVASRLTEDPSIKVGVIEAGEWHKDDPKVNIPGLAGQTM 66

GNPKYDWSFLTVPQKGANGRQIYHPR--GKVVGGSSAMNILIYDRASAREYDAIEALGNP 147

G+PKYDWSFL+VP GRQ+PR GK+GGSS+N+L +RASAEYDAIELGNP

GDPKYDWSFLSVPLSECEGRQAFIPRRVGKGLGGSSMLNLLGLNRASAAEYDAIEQLGNP 126

Query 148 GWNWSEFLKYFKKAETTLPPDPDVAAQHHLRDIDPQFHGSSGPLVKSYSAHYSIGTLHDP 207

GWNW FLKYKK+ETLP + + +HG+SGP+VK+Y + +++P

Sbjct 127 GWNWQSFLKYMKKSETALPISASIPTHQAIPPPSTELHGTSGPIVKAYYPPW-WSSVQEP 185

Query 208 LLETFKNLDVPINYDANDGNKLGFTSTFTTVDSRTAIRTYSGKSYLEPNAGRKNLVVLTG 267

https://blast.ncbi.nlm.nih.gov/Blast.cgi

5/7


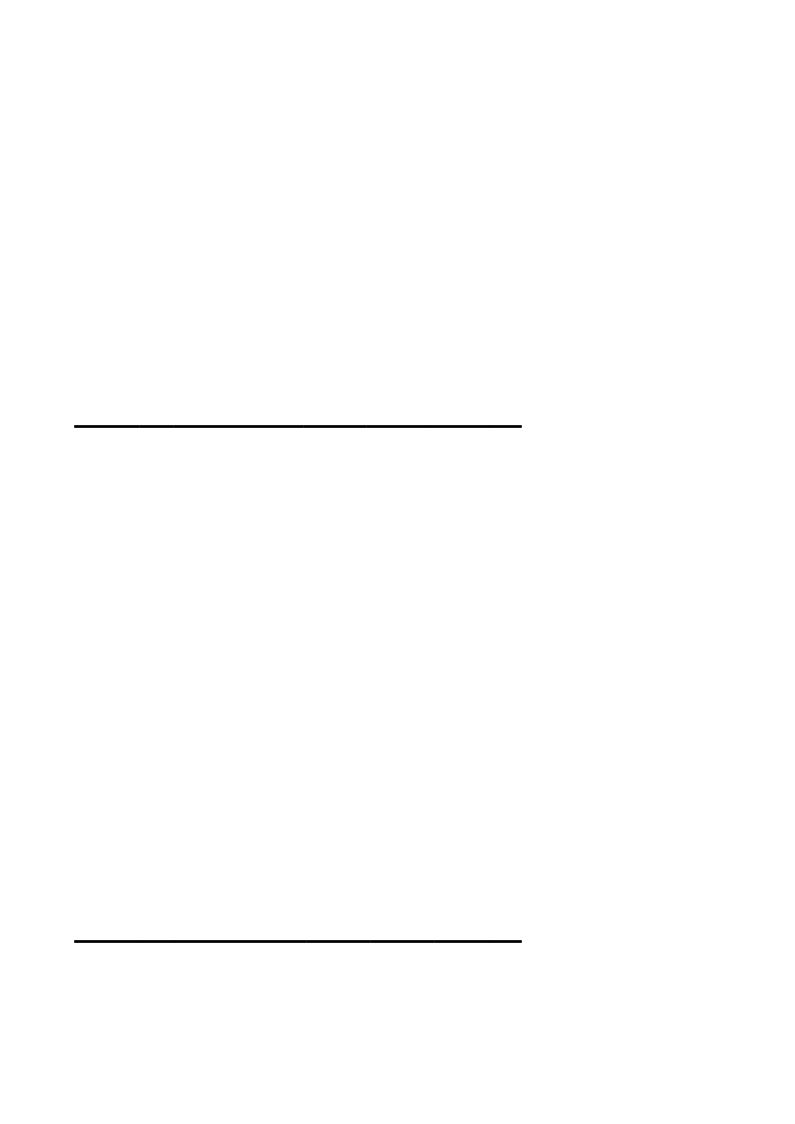


2017/4/9

NCBI Blast:GL25583-R1_1

Query 208 LLETFKNLDVPINYDANDGNKLGFTSTFTTVDSRTAIRTYSGKSYLEPNAGRKNLVVLTG 267

+T++ V N+ +G+LG++FT+D++ IRTYS +YLEP+ RNL++TG

Sbjct 186 FFQTLESFGVAANAEPGNGHNLGASKSFATIDAK-GIRTYS-VNYLEPHLDRPNLSIITG 243

Query 268 ATVTRVTFLP---GSSPLKATGVEFLKGEKTYRVA---VAKEVLLAAGTFQTPQILELSG 321

A TRV GSLA VEF R V+EV+L+AGT+QTPQ+LELSG

Sbjct 244 AHATRVVLRKSDDGQSVL-AEAVEFTVSGNQRRYTTKKVTREVVLSAGTYQTPQLLELSG 302

Query 322 IGNKDILAKHGIETLIDNSSVGENLHVSQDHCWTYSIREIDPKHETFDQAQQPERVALAQ 381

IGNKILKGIEL+D +VGEN+ QDH+ SIEIDPKHT+ P ++ Q

Sbjct 303 IGNKQILEKFGIECLLDLPTVGENM---QDHVYALSIHEIDPKHLTLEALSDPLELSHQQ 359

Query 382 QLYQSQKGPLSSAPSVIFAFLNAKTFASKEQVDQWKRDV------QTMASAVPQGLKKLH 435

+LY+++GL+SA+V+FA+L++TFS+++ ++ Q+ PGL+K

Sbjct 360 ELYKTLQGMLASAIAVVFAYLPSQTFLSADELVTAHQERLMSQGEQKLQDIFP-GLRKQL 418

Query 436 EKQSEWLSNDNSVEGELMLFAGFLPFAGLKAPSETAHYMTIVSTITHPLSRGSVHIVSAD 495

EQ+WL+ EL+FGFP G A+ Y++VS+HPSRGSVHISAD

Sbjct 419 ELQRKWLLDAAHANAELIPFPGFFPMPGHVAEA-GKRYSSMVSALMHPFSRGSVHIASAD 477

Query 496 PTAPPAIDPNYLAHPQDLQMALSLMKFAHKVFETKPLADDVRQLVTP---SPEQAATDEG 552

PTPPAIDPY +PDLQ+ +++KF K+++TP++V+ VP S++AAD+

Sbjct 478 PTVPPAIDPAYFTNPIDLQLLKAILKFTLKLYDTPPFSNLVKARVAPQLDSKDEAAQDDV 537

Query 553 LIEHIKNSCSCVFHPVGTTSMLPREDGGVVDPQLRVYGTANLRVVDASILPMQLSAHTQG 612

L+HIK++ V+HP+GT+M+PREDGGVVDPQL+VYGTANLRVVDASI+P+Q+SHTQ

Sbjct 538 LEQHIKSTGGAVYHPIGTANMMPREDGGVVDPQLKVYGTANLRVVDASIIPIQISCHTQS 597

Query 613 TVYALAEKAADVI 625

VYA+AE+AD+I

Sbjct 598 IVYAIAERAVDII 610

choline dehydrogenase 6 [Heterobasidion irregulare TC 32-1]

Sequence ID: XP_009543298.1 Length: 607 Number of Matches: 1

See 1 more title(s)

Range 1: 6 to 603

Score

Expect Method

Identities

Positives

Gaps

Frame

541 bits(1394) 0.0()

Features:

Compositional matrix adjust. 291/606(48%) 387/606(63%) 17/606(2%)

Query 30

Sbjct 6

Query 90

Sbjct 66

PEHFASTTFDYIVVGGGTAGLVVAARLSEDPNVTVGVIEAGEWEPNLPNINIPGLSGSII 89

P FST+FDY++VGGGTAG+V+AARLSEDP++VGVIEAG++P++PIN+PG+G +

PTDFVSTSFDYLIVGGGTAGIVLAARLSEDPDIVVGVIEAGDYLPDMPEINVPGMVGKSL 65

GNPKYDWSFLTVPQKGANGRQIYHPRGKVVGGSSAMNILIYDRASAREYDAIEALGNPGW 149

GNP+DWSF+TVPQ ANRQI+PRGKVVGGSSA+N+ RASAEY+A+ELG+GW

GNPQIDWSFVTVPQSAANDRQIFEPRGKVVGGSSALNFMASGRASAEEYNALETLGSLGW 125

Query 150 NWSEFLKYFKKAETTLPPDPDVAAQHHLRDIDPQFHGSSGPLVKSYSAHYSIGTLHDPLL 209

+WELKYFKK+E P VA +H D FHG GPLKSY ++ H L

Sbjct 126 SWDELLKYFKKSEKFTKPSAAVAKTYH-AGYDYDFHGKDGPLQKSYPQWFN--DYHVAFL 182

Query 210 ETFKNLDVPINYDANDGNKLG-FTSTFTTVDSRTAIRTYSGKSYLEPNAGRKNLVVLTGA 268

+T LVPIND G GFT F++D TAR+++G+Y PNARKNLV+LTGA

Sbjct 183 DTLDKLGVPINTDNGRGYNAGTFTGAF-SIDPATAARSHAGTAYYAPNADRKNLVLLTGA 241

Query 269 TVTRVTFLPGSS-PLKATGVEFLKGEKTYRVAVAKEVLLAAGTFQTPQILELSGIGNKDI 327

TRV G++ +ATG+EFKG+T+ EV+L+AG+FQTPQ+LELSGIG +

Sbjct 242 QATRVVLQQGTAGEVLATGIEFTKGGETFTARSKLEVILSAGSFQTPQLLELSGIGLSSV 301

Query 328 LAKHGIETLIDNSSVGENLHVS-----QDHCWTYSIREIDPKHETFDQAQQPERVALAQQ 382

LKHGI+++N VGENL QDHW ++E+ +ETD QPE+A +

Sbjct 302 LEKHGIKQIL-NLPVGENLRAQTFIYPQDHPWVPAVFEVSKEVETMDILQDPESLAQQLK 360

Query 383 LYQSQKGPLSSAPSVIFAFLNAKTFASKEQVDQW--KRDVQTMASAVPQGLKKLHEKQSE 440

LYQQ+G+S+ ++F+ +T S +W ++ T + P K+L +

Sbjct 361 LYQEQQGGMLSSMFSAYSFVPLRTLNSPTDYAEWMGRLEKDTSLTDTPAHRKQLSLLK-H 419

Query 441 WLSNDNSVEGELMLFAGFLPFAGLKAPSETAHYMTIVSTITHPLSRGSVHIVSADPTAPP 500

WS+ +E+ GF LKP+ +T++T+HPLSRGSVHISADPTAP

Sbjct 420 WFSDTKQAQVEFIQLPGFFSVGPLK-PKDGCRFHTLMITLLHPLSRGSVHIASADPTARP 478

Query 501 AIDPNYLAHPQDLQMALSLMKFAHKVFETKPLADDVRQLVTPSPEQAATDEGLIEHIKNS 560

IDPY +PDL++ ++FAK+ TPA VR+ P+A+D +E +

Sbjct 479 DIDPAYFKNPLDLDVMVRSVRFAQKIVATAPYA-AVRAVPYDPPAEAESDAAVREWCRTR 537

Query 561 CSCVFHPVGTTSMLPREDGGVVDPQLRVYGTANLRVVDASILPMQLSAHTQGTVYALAEK 620

++HP+GTSMLP+DGGVVD L+VYGTNLRVVDASI+P+QLSAHQTVYA+AEK

Sbjct 538 VEPLYHPIGTASMLPKADGGVVDASLKVYGTKNLRVVDASIIPLQLSAHIQSTVYAIAEK 597

Query 621 AADVIK 626

AAD+IK

Sbjct 598 AADIIK 603

BLAST is a registered trademark of the National Library of Medicine

[Support center](https://support.ncbi.nlm.nih.gov/ics/support/KBList.asp?style=classic&deptID=28049&folderID=11&) [Mailing list](https://blast.ncbi.nlm.nih.gov/Blast.cgi?CMD=Web&PAGE_TYPE=BlastDocs&DOC_TYPE=MailList)

[YouTube](https://www.youtube.com/ncbinlm)

[National Library Of Medicine](https://www.nlm.nih.gov/)

[National Institutes Of Health](https://www.nih.gov/)

[U.S. Department of Health & Human Services](https://www.hhs.gov/)

https://blast.ncbi.nlm.nih.gov/Blast.cgi

6/7


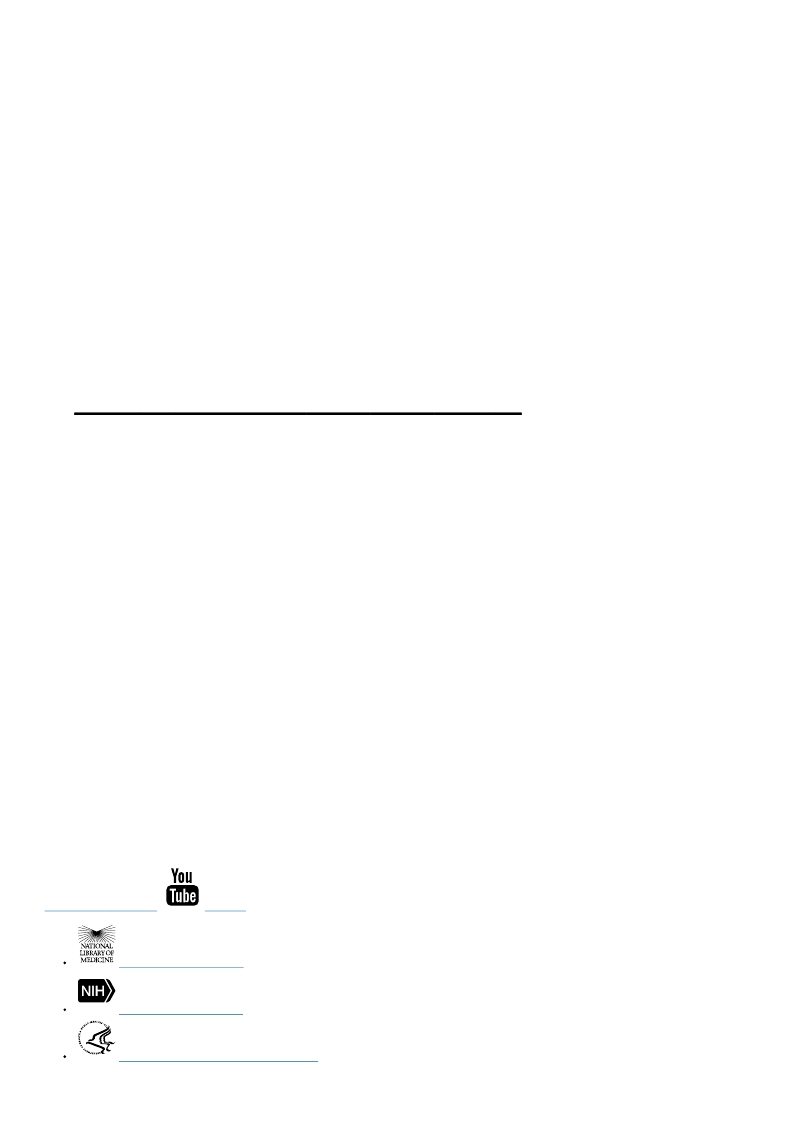


2017/4/9

NCBI Blast:GL25583-R1_1

[USA.gov](https://www.usa.gov/)

[NCBI](https://www.ncbi.nlm.nih.gov/)

[*National Center for Biotechnology Information,*](https://www.ncbi.nlm.nih.gov/) *U.S. National Library of Medicine 8600 Rock ville Pik e, Bethesda MD, 20894 USA*

[Policies and Guidelines](https://www.ncbi.nlm.nih.gov/home/about/policies.shtml) | [Contact](https://www.ncbi.nlm.nih.gov/home/about/contact.shtml)

https://blast.ncbi.nlm.nih.gov/Blast.cgi

7/7


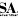


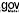


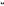

Supplement: Supplementary file 16 — Supplementary File 3a [file 41598_2017_4303_MOESM16_ESM.doc]
