# Supplementary material for: Genome-wide Identification and Characterization of Natural Antisense Transcripts by Strand-specific RNA Sequencing in Ganoderma lucidum
Source: Sci Rep. 2017 Jul 18;7:5711. doi: 10.1038/s41598-017-04303-6 (PMC5515960; doi:10.1038/s41598-017-04303-6)

2017/4/9

NCBI Blast:GL23730-R1_1

[BLAST ®](https://blast.ncbi.nlm.nih.gov/Blast.cgi) » blastp suite » RID-EMETN5W1016

BLAST Results

Job title: GL23730-R1_1

RID

[EMETN5W1016](https://blast.ncbi.nlm.nih.gov/Blast.cgi?CMD=Get&RID=EMETN5W1016) (Expires on 04-10 21:46 pm)

Query ID

lcl|Query_353537

Database Name

nr

Description

Molecule type

Query Length

GL23730-R1_1

amino acid

951

Description All non-redundant GenBank CDS

translations+PDB+SwissProt+PIR+PRF excluding

environmental samples from WGS projects

Program BLASTP 2.6.0+

New Analyze your query with SmartBLAST

Graphic Summary

Putative conserved domains have been detected, click on the image below for detailed results.

Distribution of the top 204 Blast Hits on 100 subject sequences

Color key for alignment scores

<40

40-50

50-80

80-200

>=200

Query

1

150

300

450

600

750

900

https://blast.ncbi.nlm.nih.gov/Blast.cgi

1/8


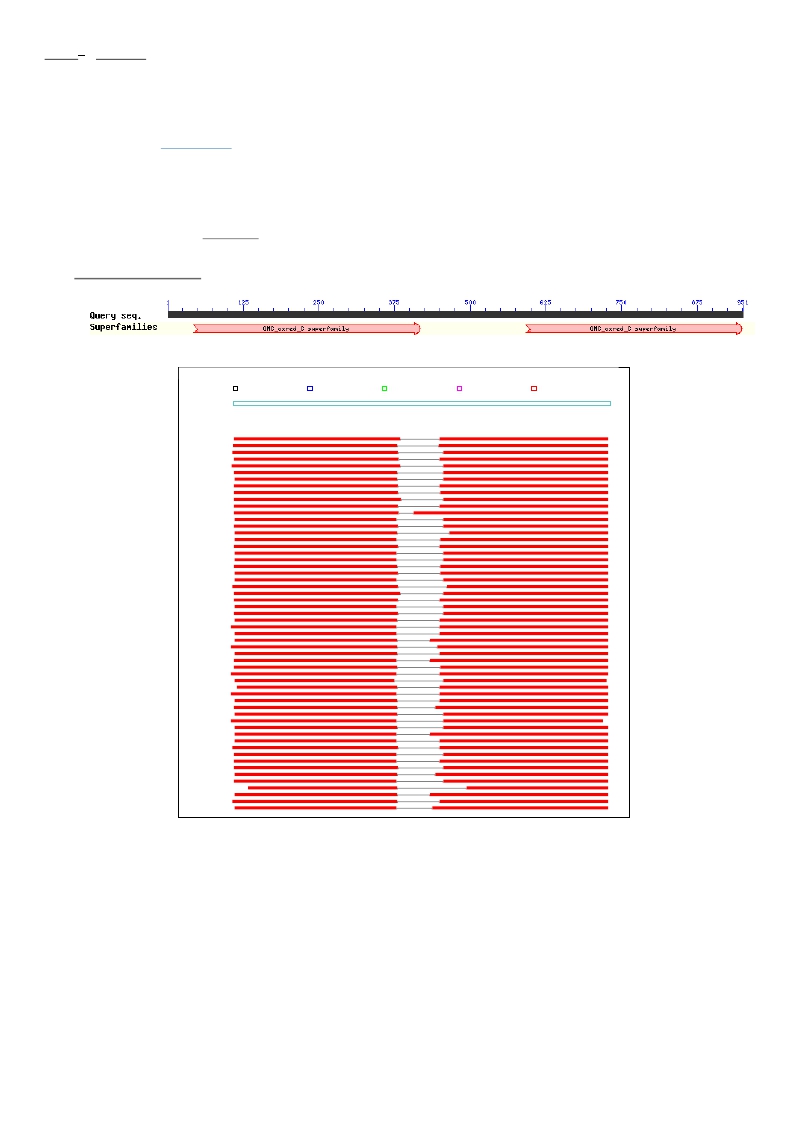


2017/4/9

Descriptions

Sequences producing significant alignments:

NCBI Blast:GL23730-R1_1

Description

[Max](https://blast.ncbi.nlm.nih.gov/Blast.cgi?CMD=Get&ALIGNMENTS=100&ALIGNMENT_VIEW=Pairwise&CDD_RID=EMETMHN6016&CDD_SEARCH_STATE=0&DATABASE_SORT=0&DESCRIPTIONS=100&DYNAMIC_FORMAT=on&FIRST_QUERY_NUM=0&FORMAT_OBJECT=Alignment&FORMAT_PAGE_TARGET=&FORMAT_TYPE=HTML&GET_SEQUENCE=yes&I_THRESH=&LINE_LENGTH=60&MASK_CHAR=2&MASK_COLOR=1&NEW_VIEW=yes&NUM_OVERVIEW=100&PAGE=Proteins&QUERY_INDEX=0&QUERY_NUMBER=0&RESULTS_PAGE_TARGET=&RID=EMETN5W1016&SHOW_LINKOUT=yes&SHOW_OVERVIEW=yes&STEP_NUMBER=&WORD_SIZE=6&OLD_VIEW=false&DISPLAY_SORT=1&HSP_SORT=1)

[Total](https://blast.ncbi.nlm.nih.gov/Blast.cgi?CMD=Get&ALIGNMENTS=100&ALIGNMENT_VIEW=Pairwise&CDD_RID=EMETMHN6016&CDD_SEARCH_STATE=0&DATABASE_SORT=0&DESCRIPTIONS=100&DYNAMIC_FORMAT=on&FIRST_QUERY_NUM=0&FORMAT_OBJECT=Alignment&FORMAT_PAGE_TARGET=&FORMAT_TYPE=HTML&GET_SEQUENCE=yes&I_THRESH=&LINE_LENGTH=60&MASK_CHAR=2&MASK_COLOR=1&NEW_VIEW=yes&NUM_OVERVIEW=100&PAGE=Proteins&QUERY_INDEX=0&QUERY_NUMBER=0&RESULTS_PAGE_TARGET=&RID=EMETN5W1016&SHOW_LINKOUT=yes&SHOW_OVERVIEW=yes&STEP_NUMBER=&WORD_SIZE=6&OLD_VIEW=false&DISPLAY_SORT=2&HSP_SORT=1)

[Query](https://blast.ncbi.nlm.nih.gov/Blast.cgi?CMD=Get&ALIGNMENTS=100&ALIGNMENT_VIEW=Pairwise&CDD_RID=EMETMHN6016&CDD_SEARCH_STATE=0&DATABASE_SORT=0&DESCRIPTIONS=100&DYNAMIC_FORMAT=on&FIRST_QUERY_NUM=0&FORMAT_OBJECT=Alignment&FORMAT_PAGE_TARGET=&FORMAT_TYPE=HTML&GET_SEQUENCE=yes&I_THRESH=&LINE_LENGTH=60&MASK_CHAR=2&MASK_COLOR=1&NEW_VIEW=yes&NUM_OVERVIEW=100&PAGE=Proteins&QUERY_INDEX=0&QUERY_NUMBER=0&RESULTS_PAGE_TARGET=&RID=EMETN5W1016&SHOW_LINKOUT=yes&SHOW_OVERVIEW=yes&STEP_NUMBER=&WORD_SIZE=6&OLD_VIEW=false&DISPLAY_SORT=4&HSP_SORT=0)

[E](https://blast.ncbi.nlm.nih.gov/Blast.cgi?CMD=Get&ALIGNMENTS=100&ALIGNMENT_VIEW=Pairwise&CDD_RID=EMETMHN6016&CDD_SEARCH_STATE=0&DATABASE_SORT=0&DESCRIPTIONS=100&DYNAMIC_FORMAT=on&FIRST_QUERY_NUM=0&FORMAT_OBJECT=Alignment&FORMAT_PAGE_TARGET=&FORMAT_TYPE=HTML&GET_SEQUENCE=yes&I_THRESH=&LINE_LENGTH=60&MASK_CHAR=2&MASK_COLOR=1&NEW_VIEW=yes&NUM_OVERVIEW=100&PAGE=Proteins&QUERY_INDEX=0&QUERY_NUMBER=0&RESULTS_PAGE_TARGET=&RID=EMETN5W1016&SHOW_LINKOUT=yes&SHOW_OVERVIEW=yes&STEP_NUMBER=&WORD_SIZE=6&OLD_VIEW=false&DISPLAY_SORT=0&HSP_SORT=0)

[Ident](https://blast.ncbi.nlm.nih.gov/Blast.cgi?CMD=Get&ALIGNMENTS=100&ALIGNMENT_VIEW=Pairwise&CDD_RID=EMETMHN6016&CDD_SEARCH_STATE=0&DATABASE_SORT=0&DESCRIPTIONS=100&DYNAMIC_FORMAT=on&FIRST_QUERY_NUM=0&FORMAT_OBJECT=Alignment&FORMAT_PAGE_TARGET=&FORMAT_TYPE=HTML&GET_SEQUENCE=yes&I_THRESH=&LINE_LENGTH=60&MASK_CHAR=2&MASK_COLOR=1&NEW_VIEW=yes&NUM_OVERVIEW=100&PAGE=Proteins&QUERY_INDEX=0&QUERY_NUMBER=0&RESULTS_PAGE_TARGET=&RID=EMETN5W1016&SHOW_LINKOUT=yes&SHOW_OVERVIEW=yes&STEP_NUMBER=&WORD_SIZE=6&DISPLAY_SORT=3&HSP_SORT=3)

Accession

[score](https://blast.ncbi.nlm.nih.gov/Blast.cgi?CMD=Get&ALIGNMENTS=100&ALIGNMENT_VIEW=Pairwise&CDD_RID=EMETMHN6016&CDD_SEARCH_STATE=0&DATABASE_SORT=0&DESCRIPTIONS=100&DYNAMIC_FORMAT=on&FIRST_QUERY_NUM=0&FORMAT_OBJECT=Alignment&FORMAT_PAGE_TARGET=&FORMAT_TYPE=HTML&GET_SEQUENCE=yes&I_THRESH=&LINE_LENGTH=60&MASK_CHAR=2&MASK_COLOR=1&NEW_VIEW=yes&NUM_OVERVIEW=100&PAGE=Proteins&QUERY_INDEX=0&QUERY_NUMBER=0&RESULTS_PAGE_TARGET=&RID=EMETN5W1016&SHOW_LINKOUT=yes&SHOW_OVERVIEW=yes&STEP_NUMBER=&WORD_SIZE=6&OLD_VIEW=false&DISPLAY_SORT=1&HSP_SORT=1)

[score](https://blast.ncbi.nlm.nih.gov/Blast.cgi?CMD=Get&ALIGNMENTS=100&ALIGNMENT_VIEW=Pairwise&CDD_RID=EMETMHN6016&CDD_SEARCH_STATE=0&DATABASE_SORT=0&DESCRIPTIONS=100&DYNAMIC_FORMAT=on&FIRST_QUERY_NUM=0&FORMAT_OBJECT=Alignment&FORMAT_PAGE_TARGET=&FORMAT_TYPE=HTML&GET_SEQUENCE=yes&I_THRESH=&LINE_LENGTH=60&MASK_CHAR=2&MASK_COLOR=1&NEW_VIEW=yes&NUM_OVERVIEW=100&PAGE=Proteins&QUERY_INDEX=0&QUERY_NUMBER=0&RESULTS_PAGE_TARGET=&RID=EMETN5W1016&SHOW_LINKOUT=yes&SHOW_OVERVIEW=yes&STEP_NUMBER=&WORD_SIZE=6&OLD_VIEW=false&DISPLAY_SORT=2&HSP_SORT=1)

[cover](https://blast.ncbi.nlm.nih.gov/Blast.cgi?CMD=Get&ALIGNMENTS=100&ALIGNMENT_VIEW=Pairwise&CDD_RID=EMETMHN6016&CDD_SEARCH_STATE=0&DATABASE_SORT=0&DESCRIPTIONS=100&DYNAMIC_FORMAT=on&FIRST_QUERY_NUM=0&FORMAT_OBJECT=Alignment&FORMAT_PAGE_TARGET=&FORMAT_TYPE=HTML&GET_SEQUENCE=yes&I_THRESH=&LINE_LENGTH=60&MASK_CHAR=2&MASK_COLOR=1&NEW_VIEW=yes&NUM_OVERVIEW=100&PAGE=Proteins&QUERY_INDEX=0&QUERY_NUMBER=0&RESULTS_PAGE_TARGET=&RID=EMETN5W1016&SHOW_LINKOUT=yes&SHOW_OVERVIEW=yes&STEP_NUMBER=&WORD_SIZE=6&OLD_VIEW=false&DISPLAY_SORT=4&HSP_SORT=0)

[value](https://blast.ncbi.nlm.nih.gov/Blast.cgi?CMD=Get&ALIGNMENTS=100&ALIGNMENT_VIEW=Pairwise&CDD_RID=EMETMHN6016&CDD_SEARCH_STATE=0&DATABASE_SORT=0&DESCRIPTIONS=100&DYNAMIC_FORMAT=on&FIRST_QUERY_NUM=0&FORMAT_OBJECT=Alignment&FORMAT_PAGE_TARGET=&FORMAT_TYPE=HTML&GET_SEQUENCE=yes&I_THRESH=&LINE_LENGTH=60&MASK_CHAR=2&MASK_COLOR=1&NEW_VIEW=yes&NUM_OVERVIEW=100&PAGE=Proteins&QUERY_INDEX=0&QUERY_NUMBER=0&RESULTS_PAGE_TARGET=&RID=EMETN5W1016&SHOW_LINKOUT=yes&SHOW_OVERVIEW=yes&STEP_NUMBER=&WORD_SIZE=6&OLD_VIEW=false&DISPLAY_SORT=0&HSP_SORT=0)

alcohol oxidase [Dichomitus squalens LYAD-421 SS1]

alcohol oxidase [Dichomitus squalens LYAD-421 SS1]

alcohol oxidase [Dichomitus squalens LYAD-421 SS1]

hypothetical protein GYMLUDRAFT_394379 [Gymnopus

luxurians FD-317 M1]

alcohol oxidase [Fomitiporia mediterranea MF3/22]

aryl-alcohol oxidase 1 [Heterobasidion irregulare TC 32-

1]

GMC oxidoreductase [Peniophora sp. CONT]

aryl-alcohol oxidase-like protein [Stereum hirsutum FP-

91666 SS1]

alcohol oxidase [Stereum hirsutum FP-91666 SS1]

aryl-alcohol oxidase 9 [Heterobasidion irregulare TC 32-

1]

aryl-alcohol-oxidase from pleurotus Eryingii [Stereum

hirsutum FP-91666 SS1]

hypothetical protein GYMLUDRAFT_265013 [Gymnopus

luxurians FD-317 M1]

GMC oxidoreductase [Peniophora sp. CONT]

mitochondrial choline dehydrogenase [Irpex lacteus]

alcohol oxidase [Stereum hirsutum FP-91666 SS1]

GMC oxidoreductase [Peniophora sp. CONT]

alcohol oxidase [Stereum hirsutum FP-91666 SS1]

choline dehydrogenase [Irpex lacteus]

aryl-alcohol oxidase-like protein [Stereum hirsutum FP-

91666 SS1]

alcohol oxidase [Gloeophyllum trabeum ATCC 11539]

alcohol oxidase [Sanghuangporus baumii]

alcohol oxidase [Gloeophyllum trabeum ATCC 11539]

691

613

552

448

447

435

433

425

424

423

422

421

421

418

414

412

412

402

399

398

407

383

1193

1166

1036

867

874

829

822

822

802

808

800

829

809

796

786

815

798

781

777

790

1546

741

88%

88%

87%

88%

88%

87%

86%

88%

88%

88%

88%

95%

86%

87%

85%

87%

88%

86%

86%

87%

88%

86%

0.0

0.0

0.0

2e-143

3e-142

3e-138

1e-137

3e-134

1e-133

2e-133

3e-133

8e-133

1e-132

1e-131

3e-130

1e-129

9e-129

1e-125

9e-125

1e-123

1e-122

4e-118

82%

68%

66%

54%

54%

55%

53%

53%

53%

51%

52%

51%

52%

51%

51%

51%

51%

52%

52%

48%

50%

47%

[XP_007363926.1](https://www.ncbi.nlm.nih.gov/protein/597983031?report=genbank&log$=prottop&blast_rank=1&RID=EMETN5W1016)

[XP_007363915.1](https://www.ncbi.nlm.nih.gov/protein/597982989?report=genbank&log$=prottop&blast_rank=2&RID=EMETN5W1016)

[XP_007363927.1](https://www.ncbi.nlm.nih.gov/protein/597983035?report=genbank&log$=prottop&blast_rank=3&RID=EMETN5W1016)

[KIK51433.1](https://www.ncbi.nlm.nih.gov/protein/751009628?report=genbank&log$=prottop&blast_rank=4&RID=EMETN5W1016)

[XP_007269675.1](https://www.ncbi.nlm.nih.gov/protein/595782264?report=genbank&log$=prottop&blast_rank=5&RID=EMETN5W1016)

[XP_009543297.1](https://www.ncbi.nlm.nih.gov/protein/695541485?report=genbank&log$=prottop&blast_rank=6&RID=EMETN5W1016)

[KZV68609.1](https://www.ncbi.nlm.nih.gov/protein/1024075635?report=genbank&log$=prottop&blast_rank=7&RID=EMETN5W1016)

[XP_007308838.1](https://www.ncbi.nlm.nih.gov/protein/618812342?report=genbank&log$=prottop&blast_rank=8&RID=EMETN5W1016)

[XP_007306524.1](https://www.ncbi.nlm.nih.gov/protein/618807623?report=genbank&log$=prottop&blast_rank=9&RID=EMETN5W1016)

[XP_009552529.1](https://www.ncbi.nlm.nih.gov/protein/695577156?report=genbank&log$=prottop&blast_rank=10&RID=EMETN5W1016)

[XP_007306850.1](https://www.ncbi.nlm.nih.gov/protein/618808275?report=genbank&log$=prottop&blast_rank=11&RID=EMETN5W1016)

[KIK53520.1](https://www.ncbi.nlm.nih.gov/protein/751011750?report=genbank&log$=prottop&blast_rank=12&RID=EMETN5W1016)

[KZV70517.1](https://www.ncbi.nlm.nih.gov/protein/1024077573?report=genbank&log$=prottop&blast_rank=13&RID=EMETN5W1016)

[ALJ82906.1](https://www.ncbi.nlm.nih.gov/protein/939535459?report=genbank&log$=prottop&blast_rank=14&RID=EMETN5W1016)

[XP_007306952.1](https://www.ncbi.nlm.nih.gov/protein/618808479?report=genbank&log$=prottop&blast_rank=15&RID=EMETN5W1016)

[KZV61898.1](https://www.ncbi.nlm.nih.gov/protein/1024068727?report=genbank&log$=prottop&blast_rank=16&RID=EMETN5W1016)

[XP_007311377.1](https://www.ncbi.nlm.nih.gov/protein/618817604?report=genbank&log$=prottop&blast_rank=17&RID=EMETN5W1016)

[ALJ82895.1](https://www.ncbi.nlm.nih.gov/protein/939535437?report=genbank&log$=prottop&blast_rank=18&RID=EMETN5W1016)

[XP_007306289.1](https://www.ncbi.nlm.nih.gov/protein/618807119?report=genbank&log$=prottop&blast_rank=19&RID=EMETN5W1016)

[XP_007863751.1](https://www.ncbi.nlm.nih.gov/protein/630348901?report=genbank&log$=prottop&blast_rank=20&RID=EMETN5W1016)

[OCB89708.1](https://www.ncbi.nlm.nih.gov/protein/1044615148?report=genbank&log$=prottop&blast_rank=21&RID=EMETN5W1016)

[XP_007862106.1](https://www.ncbi.nlm.nih.gov/protein/630345611?report=genbank&log$=prottop&blast_rank=22&RID=EMETN5W1016)

aryl-alcohol-oxidase from pleurotus Eryingii [Dichomitus

squalens LYAD-421 SS1]

aryl-alcohol oxidase 6 [Heterobasidion irregulare TC 32-

1]

alcohol oxidase [Stereum hirsutum FP-91666 SS1]

hypothetical protein GYMLUDRAFT_220474 [Gymnopus

luxurians FD-317 M1]

aryl-alcohol oxidase 4 [Heterobasidion irregulare TC 32-

1]

aryl-alcohol oxidase [Jaapia argillacea MUCL 33604]

aryl-alcohol oxidase-like protein [Schizopora paradoxa]

hypothetical protein GALMADRAFT_125264 [Galerina

marginata CBS 339.88]

Pyranose dehydrogenase 3 [Hypsizygus marmoreus]

alcohol oxidase [Stereum hirsutum FP-91666 SS1]

hypothetical protein PLEOSDRAFT_1031974 [Pleurotus

ostreatus PC15]

Pyranose dehydrogenase 3 [Hypsizygus marmoreus]

aryl-alcohol-oxidase from pleurotus Eryingii [Stereum

hirsutum FP-91666 SS1]

aryl-alcohol oxidase-like protein [Schizopora paradoxa]

aryl-alcohol-oxidase from pleurotus Eryingii [Stereum

hirsutum FP-91666 SS1]

alcohol oxidase [Dichomitus squalens LYAD-421 SS1]

alcohol oxidase [Schizopora paradoxa]

predicted protein [Fibroporia radiculosa]

GMC oxidoreductase [Daedalea quercina L-15889]

Pyranose dehydrogenase 1 [Grifola frondosa]

Pyranose dehydrogenase 1 [Grifola frondosa]

384

381

382

377

376

376

375

374

371

371

370

369

369

369

367

367

366

366

365

364

363

717

730

726

740

722

719

745

741

701

701

723

726

708

718

721

696

729

714

717

709

679

86%

87%

88%

86%

87%

88%

88%

87%

90%

89%

88%

90%

88%

88%

85%

87%

88%

88%

89%

86%

86%

5e-118

2e-117

9e-117

5e-116

1e-115

2e-115

2e-115

8e-115

7e-114

2e-113

3e-113

4e-113

6e-113

6e-113

2e-112

3e-112

9e-112

9e-112

3e-111

5e-111

2e-110

50%

47%

49%

50%

48%

47%

46%

46%

45%

48%

45%

49%

48%

43%

50%

48%

45%

46%

46%

48%

48%

[XP_007367502.1](https://www.ncbi.nlm.nih.gov/protein/597996893?report=genbank&log$=prottop&blast_rank=23&RID=EMETN5W1016)

[XP_009552489.1](https://www.ncbi.nlm.nih.gov/protein/695577032?report=genbank&log$=prottop&blast_rank=24&RID=EMETN5W1016)

[XP_007308367.1](https://www.ncbi.nlm.nih.gov/protein/618811367?report=genbank&log$=prottop&blast_rank=25&RID=EMETN5W1016)

[KIK65201.1](https://www.ncbi.nlm.nih.gov/protein/751023500?report=genbank&log$=prottop&blast_rank=26&RID=EMETN5W1016)

[XP_009551205.1](https://www.ncbi.nlm.nih.gov/protein/695571753?report=genbank&log$=prottop&blast_rank=27&RID=EMETN5W1016)

[KDQ57028.1](https://www.ncbi.nlm.nih.gov/protein/646392514?report=genbank&log$=prottop&blast_rank=28&RID=EMETN5W1016)

[KLO15613.1](https://www.ncbi.nlm.nih.gov/protein/827760989?report=genbank&log$=prottop&blast_rank=29&RID=EMETN5W1016)

[KDR73180.1](https://www.ncbi.nlm.nih.gov/protein/648159452?report=genbank&log$=prottop&blast_rank=30&RID=EMETN5W1016)

[KYQ35789.1](https://www.ncbi.nlm.nih.gov/protein/1012961019?report=genbank&log$=prottop&blast_rank=31&RID=EMETN5W1016)

[XP_007307422.1](https://www.ncbi.nlm.nih.gov/protein/618809453?report=genbank&log$=prottop&blast_rank=32&RID=EMETN5W1016)

[KDQ32751.1](https://www.ncbi.nlm.nih.gov/protein/646311610?report=genbank&log$=prottop&blast_rank=33&RID=EMETN5W1016)

[KYQ32239.1](https://www.ncbi.nlm.nih.gov/protein/1012957437?report=genbank&log$=prottop&blast_rank=34&RID=EMETN5W1016)

[XP_007305318.1](https://www.ncbi.nlm.nih.gov/protein/618805117?report=genbank&log$=prottop&blast_rank=35&RID=EMETN5W1016)

[KLO15610.1](https://www.ncbi.nlm.nih.gov/protein/827760986?report=genbank&log$=prottop&blast_rank=36&RID=EMETN5W1016)

[XP_007307419.1](https://www.ncbi.nlm.nih.gov/protein/618809447?report=genbank&log$=prottop&blast_rank=37&RID=EMETN5W1016)

[XP_007363169.1](https://www.ncbi.nlm.nih.gov/protein/597980125?report=genbank&log$=prottop&blast_rank=38&RID=EMETN5W1016)

[KLO15614.1](https://www.ncbi.nlm.nih.gov/protein/827760990?report=genbank&log$=prottop&blast_rank=39&RID=EMETN5W1016)

[XP_012185328.1](https://www.ncbi.nlm.nih.gov/protein/807761067?report=genbank&log$=prottop&blast_rank=40&RID=EMETN5W1016)

[KZT63569.1](https://www.ncbi.nlm.nih.gov/protein/1023268586?report=genbank&log$=prottop&blast_rank=41&RID=EMETN5W1016)

[OBZ73918.1](https://www.ncbi.nlm.nih.gov/protein/1043286688?report=genbank&log$=prottop&blast_rank=42&RID=EMETN5W1016)

[OBZ73035.1](https://www.ncbi.nlm.nih.gov/protein/1043285803?report=genbank&log$=prottop&blast_rank=43&RID=EMETN5W1016)

https://blast.ncbi.nlm.nih.gov/Blast.cgi

2/8


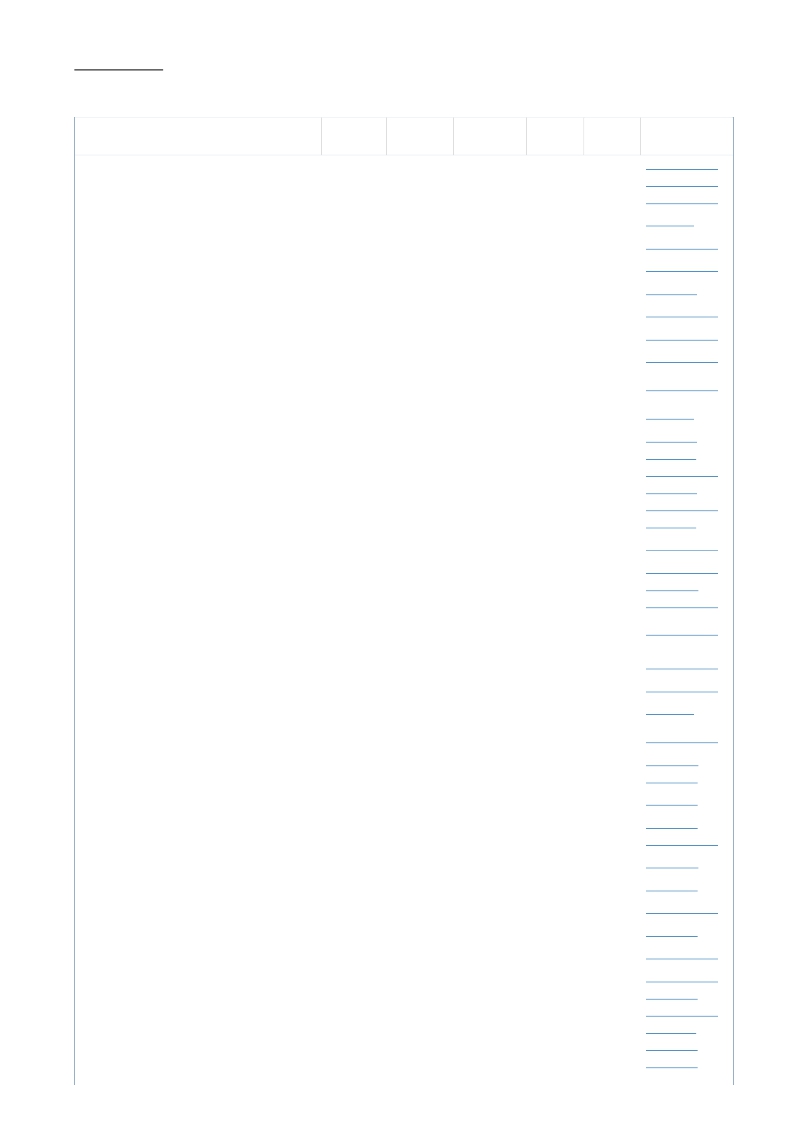


2017/4/9

NCBI Blast:GL23730-R1_1

aryl-alcohol oxidase-like protein [Postia placenta Mad-

362

694

86%

2e-110

49%

[XP_002475146.1](https://www.ncbi.nlm.nih.gov/protein/242218717?report=genbank&log$=prottop&blast_rank=44&RID=EMETN5W1016)

698-R]

GMC oxidoreductase [Neolentinus lepideus HHB14362

ss-1]

predicted protein [Fibroporia radiculosa]

aryl-alcohol-oxidase from pleurotus Eryingii [Schizopora

paradoxa]

aryl-alcohol oxidase precursor [Schizopora paradoxa]

Pyranose dehydrogenase 3 [Hypsizygus marmoreus]

GMC oxidoreductase [Suillus luteus UH-Slu-Lm8-n1]

hypothetical protein PLEOSDRAFT_1098740 [Pleurotus

ostreatus PC15]

hypothetical protein GALMADRAFT_218865 [Galerina

marginata CBS 339.88]

predicted protein [Fibroporia radiculosa]

Pyranose dehydrogenase [Hypsizygus marmoreus]

aryl-alcohol oxidase-like protein [Trametes versicolor FP-

101664 SS1]

GMC oxidoreductase [Sphaerobolus stellatus SS14]

predicted protein [Fibroporia radiculosa]

Choline dehydrogenase, mitochondrial [Leucoagaricus

sp. SymC.cos]

aryl-alcohol oxidase 11 [Heterobasidion irregulare TC 32-

1]

hypothetical protein PLEOSDRAFT_169195 [Pleurotus

ostreatus PC15]

aryl-alcohol oxidase-like protein [Obba rivulosa]

Pyranose dehydrogenase [Trametes pubescens]

Aryl-alcohol oxidase [Trametes cinnabarina]

Pyranose dehydrogenase 3 [Hypsizygus marmoreus]

Choline dehydrogenase, mitochondrial [Termitomyces sp.

J132]

GMC oxidoreductase [Peniophora sp. CONT]

Pyranose dehydrogenase 3 [Hypsizygus marmoreus]

GMC oxidoreductase [Laccaria amethystina LaAM-08-1]

predicted protein [Laccaria bicolor S238N-H82]

extracellular aryl-alcohol dehydrogenase [synthetic

construct]

GMC oxidoreductase [Laccaria amethystina LaAM-08-1]

GMC oxidoreductase [Peniophora sp. CONT]

GMC oxidoreductase [Neolentinus lepideus HHB14362

ss-1]

GMC oxidoreductase [Hebeloma cylindrosporum h7]

hypothetical protein PHACADRAFT_260543

[Phanerochaete carnosa HHB-10118-sp]

hypothetical protein GALMADRAFT_91825 [Galerina

marginata CBS 339.88]

Pyranose dehydrogenase 3 [Trametes pubescens]

aryl-alcohol oxidase [Lentinula edodes]

Pyranose dehydrogenase 1 [Hypsizygus marmoreus]

GMC oxidoreductase [Sphaerobolus stellatus SS14]

Pyranose dehydrogenase 1 [Hypsizygus marmoreus]

aryl-alcohol oxidase-like protein [Obba rivulosa]

Pyranose dehydrogenase 3 [Hypsizygus marmoreus]

GMC oxidoreductase [Hebeloma cylindrosporum h7]

extracellular aryl-alcohol oxidase-1 [synthetic construct]

GMC oxidoreductase [Laccaria amethystina LaAM-08-1]

GMC oxidoreductase [Hypholoma sublateritium FD-334

SS-4]

GMC oxidoreductase [Laccaria amethystina LaAM-08-1]

aryl-alcohol oxidase-like protein [Obba rivulosa]

aryl-alcohol oxidase-like protein [Stereum hirsutum FP-

91666 SS1]

hypothetical protein PLEOSDRAFT_1079425 [Pleurotus

ostreatus PC15]

aryl-alcohol oxidase 12 [Heterobasidion irregulare TC 32-

361

360

360

360

360

358

358

358

351

356

357

355

354

356

354

354

352

350

346

349

349

348

347

348

346

347

346

346

345

345

345

344

340

345

343

343

343

343

344

342

342

342

342

341

341

341

340

340

711

707

697

720

717

708

686

716

637

688

703

709

682

696

702

684

699

693

672

699

684

686

686

661

691

674

646

664

676

661

657

644

681

689

650

674

683

684

674

654

656

669

641

662

655

657

652

657

90%

87%

88%

86%

87%

87%

89%

86%

77%

90%

88%

89%

88%

87%

86%

87%

86%

90%

90%

88%

87%

87%

86%

88%

88%

90%

86%

88%

88%

88%

88%

90%

87%

88%

87%

90%

86%

86%

90%

88%

86%

88%

88%

89%

87%

87%

88%

91%

7e-110

1e-109

1e-109

1e-109

2e-109

4e-109

5e-109

6e-109

6e-109

3e-108

3e-108

6e-108

2e-107

3e-107

3e-107

4e-107

2e-106

6e-106

1e-105

2e-105

2e-105

4e-105

2e-104

2e-104

3e-104

3e-104

4e-104

5e-104

5e-104

6e-104

1e-103

1e-103

1e-103

2e-103

2e-103

2e-103

2e-103

4e-103

5e-103

8e-103

8e-103

9e-103

1e-102

2e-102

2e-102

3e-102

4e-102

5e-102

47%

47%

47%

46%

46%

47%

46%

46%

49%

43%

48%

48%

48%

44%

45%

44%

46%

47%

43%

47%

44%

45%

45%

43%

44%

43%

44%

46%

45%

46%

46%

41%

44%

46%

45%

44%

47%

45%

43%

46%

46%

44%

44%

45%

47%

44%

44%

44%

[KZT29324.1](https://www.ncbi.nlm.nih.gov/protein/1023232541?report=genbank&log$=prottop&blast_rank=45&RID=EMETN5W1016)

[XP_012185327.1](https://www.ncbi.nlm.nih.gov/protein/807761065?report=genbank&log$=prottop&blast_rank=46&RID=EMETN5W1016)

[KLO19185.1](https://www.ncbi.nlm.nih.gov/protein/827764600?report=genbank&log$=prottop&blast_rank=47&RID=EMETN5W1016)

[KLO07026.1](https://www.ncbi.nlm.nih.gov/protein/827752164?report=genbank&log$=prottop&blast_rank=48&RID=EMETN5W1016)

[KYQ32251.1](https://www.ncbi.nlm.nih.gov/protein/1012957449?report=genbank&log$=prottop&blast_rank=49&RID=EMETN5W1016)

[KIK39866.1](https://www.ncbi.nlm.nih.gov/protein/750997598?report=genbank&log$=prottop&blast_rank=50&RID=EMETN5W1016)

[KDQ32756.1](https://www.ncbi.nlm.nih.gov/protein/646311615?report=genbank&log$=prottop&blast_rank=51&RID=EMETN5W1016)

[KDR85769.1](https://www.ncbi.nlm.nih.gov/protein/648172153?report=genbank&log$=prottop&blast_rank=52&RID=EMETN5W1016)

[XP_012182723.1](https://www.ncbi.nlm.nih.gov/protein/807755857?report=genbank&log$=prottop&blast_rank=53&RID=EMETN5W1016)

[KYQ35790.1](https://www.ncbi.nlm.nih.gov/protein/1012961020?report=genbank&log$=prottop&blast_rank=54&RID=EMETN5W1016)

[XP_008045472.1](https://www.ncbi.nlm.nih.gov/protein/636628675?report=genbank&log$=prottop&blast_rank=55&RID=EMETN5W1016)

[KIJ54911.1](https://www.ncbi.nlm.nih.gov/protein/749885524?report=genbank&log$=prottop&blast_rank=56&RID=EMETN5W1016)

[XP_012185335.1](https://www.ncbi.nlm.nih.gov/protein/807761081?report=genbank&log$=prottop&blast_rank=57&RID=EMETN5W1016)

[KXN85084.1](https://www.ncbi.nlm.nih.gov/protein/1000843840?report=genbank&log$=prottop&blast_rank=58&RID=EMETN5W1016)

[XP_009552521.1](https://www.ncbi.nlm.nih.gov/protein/695577129?report=genbank&log$=prottop&blast_rank=59&RID=EMETN5W1016)

[KDQ26345.1](https://www.ncbi.nlm.nih.gov/protein/646305199?report=genbank&log$=prottop&blast_rank=60&RID=EMETN5W1016)

[OCH88556.1](https://www.ncbi.nlm.nih.gov/protein/1045911400?report=genbank&log$=prottop&blast_rank=61&RID=EMETN5W1016)

[OJT08261.1](https://www.ncbi.nlm.nih.gov/protein/1112954419?report=genbank&log$=prottop&blast_rank=62&RID=EMETN5W1016)

[CDO72823.1](https://www.ncbi.nlm.nih.gov/protein/691791657?report=genbank&log$=prottop&blast_rank=63&RID=EMETN5W1016)

[KYQ32240.1](https://www.ncbi.nlm.nih.gov/protein/1012957438?report=genbank&log$=prottop&blast_rank=64&RID=EMETN5W1016)

[KNZ75849.1](https://www.ncbi.nlm.nih.gov/protein/914260713?report=genbank&log$=prottop&blast_rank=65&RID=EMETN5W1016)

[KZV68633.1](https://www.ncbi.nlm.nih.gov/protein/1024075659?report=genbank&log$=prottop&blast_rank=66&RID=EMETN5W1016)

[KYQ41771.1](https://www.ncbi.nlm.nih.gov/protein/1012967024?report=genbank&log$=prottop&blast_rank=67&RID=EMETN5W1016)

[KIJ99316.1](https://www.ncbi.nlm.nih.gov/protein/750941340?report=genbank&log$=prottop&blast_rank=68&RID=EMETN5W1016)

[XP_001884302.1](https://www.ncbi.nlm.nih.gov/protein/170106181?report=genbank&log$=prottop&blast_rank=69&RID=EMETN5W1016)

[ALS87661.1](https://www.ncbi.nlm.nih.gov/protein/961591086?report=genbank&log$=prottop&blast_rank=70&RID=EMETN5W1016)

[KIJ94944.1](https://www.ncbi.nlm.nih.gov/protein/750936844?report=genbank&log$=prottop&blast_rank=71&RID=EMETN5W1016)

[KZV75707.1](https://www.ncbi.nlm.nih.gov/protein/1024082817?report=genbank&log$=prottop&blast_rank=72&RID=EMETN5W1016)

[KZT22397.1](https://www.ncbi.nlm.nih.gov/protein/1023225572?report=genbank&log$=prottop&blast_rank=73&RID=EMETN5W1016)

[KIM43771.1](https://www.ncbi.nlm.nih.gov/protein/751693788?report=genbank&log$=prottop&blast_rank=74&RID=EMETN5W1016)

[XP_007398632.1](https://www.ncbi.nlm.nih.gov/protein/599388955?report=genbank&log$=prottop&blast_rank=75&RID=EMETN5W1016)

[KDR81170.1](https://www.ncbi.nlm.nih.gov/protein/648167506?report=genbank&log$=prottop&blast_rank=76&RID=EMETN5W1016)

[OJT05516.1](https://www.ncbi.nlm.nih.gov/protein/1112951292?report=genbank&log$=prottop&blast_rank=77&RID=EMETN5W1016)

[GAW00865.1](https://www.ncbi.nlm.nih.gov/protein/1139916089?report=genbank&log$=prottop&blast_rank=78&RID=EMETN5W1016)

[KYQ35350.1](https://www.ncbi.nlm.nih.gov/protein/1012960577?report=genbank&log$=prottop&blast_rank=79&RID=EMETN5W1016)

[KIJ49389.1](https://www.ncbi.nlm.nih.gov/protein/749879791?report=genbank&log$=prottop&blast_rank=80&RID=EMETN5W1016)

[KYQ44384.1](https://www.ncbi.nlm.nih.gov/protein/1012969643?report=genbank&log$=prottop&blast_rank=81&RID=EMETN5W1016)

[OCH92503.1](https://www.ncbi.nlm.nih.gov/protein/1045915428?report=genbank&log$=prottop&blast_rank=82&RID=EMETN5W1016)

[KYQ33336.1](https://www.ncbi.nlm.nih.gov/protein/1012958547?report=genbank&log$=prottop&blast_rank=83&RID=EMETN5W1016)

[KIM43770.1](https://www.ncbi.nlm.nih.gov/protein/751693787?report=genbank&log$=prottop&blast_rank=84&RID=EMETN5W1016)

[ALS87662.1](https://www.ncbi.nlm.nih.gov/protein/961591088?report=genbank&log$=prottop&blast_rank=85&RID=EMETN5W1016)

[KIK07704.1](https://www.ncbi.nlm.nih.gov/protein/750949830?report=genbank&log$=prottop&blast_rank=86&RID=EMETN5W1016)

[KJA14288.1](https://www.ncbi.nlm.nih.gov/protein/763716719?report=genbank&log$=prottop&blast_rank=87&RID=EMETN5W1016)

[KIK00529.1](https://www.ncbi.nlm.nih.gov/protein/750942578?report=genbank&log$=prottop&blast_rank=88&RID=EMETN5W1016)

[OCH86986.1](https://www.ncbi.nlm.nih.gov/protein/1045909776?report=genbank&log$=prottop&blast_rank=89&RID=EMETN5W1016)

[XP_007301607.1](https://www.ncbi.nlm.nih.gov/protein/597908616?report=genbank&log$=prottop&blast_rank=90&RID=EMETN5W1016)

[KDQ23258.1](https://www.ncbi.nlm.nih.gov/protein/646302108?report=genbank&log$=prottop&blast_rank=91&RID=EMETN5W1016)

[XP_009546102.1](https://www.ncbi.nlm.nih.gov/protein/695552314?report=genbank&log$=prottop&blast_rank=92&RID=EMETN5W1016)

https://blast.ncbi.nlm.nih.gov/Blast.cgi

3/8


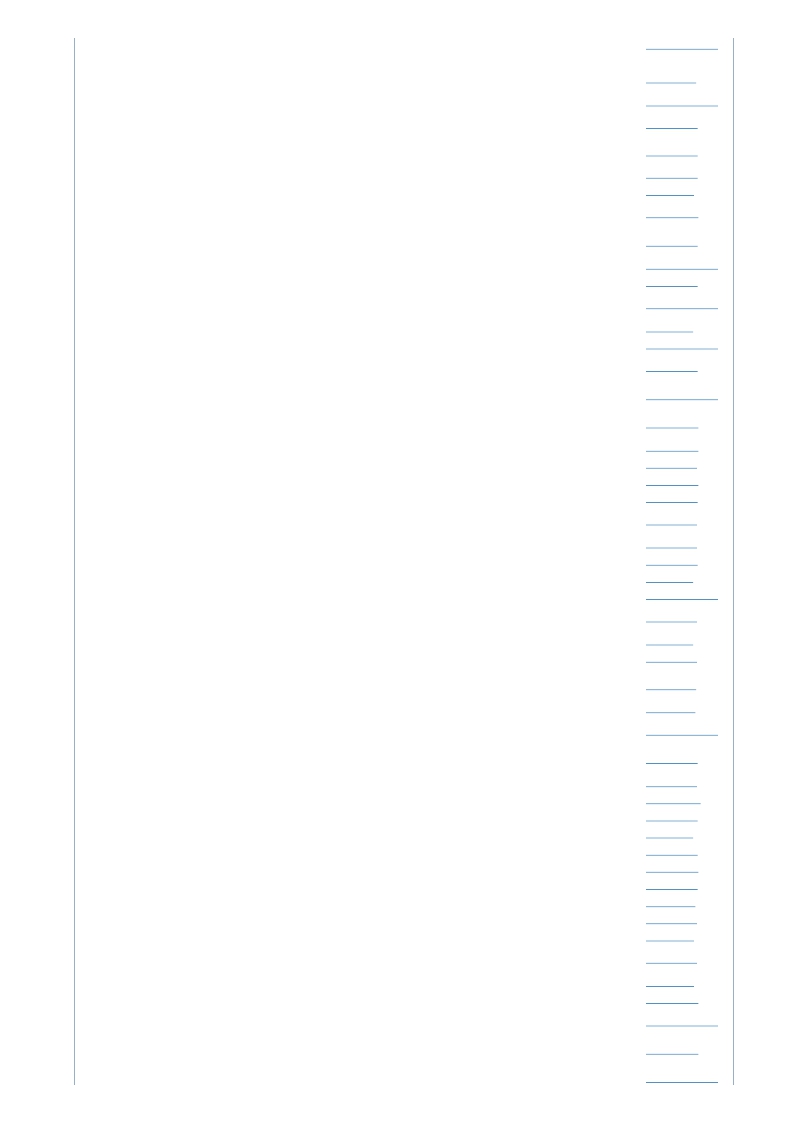


2017/4/9

1]

NCBI Blast:GL23730-R1_1

hypothetical protein CERSUDRAFT_117387 [Gelatoporia

subvermispora B]

aryl-alcohol oxidase-like protein [Schizopora paradoxa]

Choline dehydrogenase, mitochondrial [Leucoagaricus

sp. SymC.cos]

hypothetical protein PLEOSDRAFT_1098737 [Pleurotus

ostreatus PC15]

hypothetical protein GALMADRAFT_230577 [Galerina

marginata CBS 339.88]

hypothetical protein CERSUDRAFT_84544 [Gelatoporia

subvermispora B]

alcohol oxidase [Obba rivulosa]

hypothetical protein GALMADRAFT_1117948 [Galerina

marginata CBS 339.88]

340

340

353

338

338

338

338

337

677

642

1113

667

636

666

669

671

87%

89%

86%

87%

90%

87%

87%

86%

6e-102

6e-102

1e-101

2e-101

2e-101

3e-101

3e-101

4e-101

44%

44%

46%

43%

41%

46%

46%

45%

[EMD33860.1](https://www.ncbi.nlm.nih.gov/protein/449542883?report=genbank&log$=prottop&blast_rank=93&RID=EMETN5W1016)

[KLO19189.1](https://www.ncbi.nlm.nih.gov/protein/827764604?report=genbank&log$=prottop&blast_rank=94&RID=EMETN5W1016)

[KXN85893.1](https://www.ncbi.nlm.nih.gov/protein/1000844779?report=genbank&log$=prottop&blast_rank=95&RID=EMETN5W1016)

[KDQ32753.1](https://www.ncbi.nlm.nih.gov/protein/646311612?report=genbank&log$=prottop&blast_rank=96&RID=EMETN5W1016)

[KDR69588.1](https://www.ncbi.nlm.nih.gov/protein/648155796?report=genbank&log$=prottop&blast_rank=97&RID=EMETN5W1016)

[EMD36400.1](https://www.ncbi.nlm.nih.gov/protein/449545429?report=genbank&log$=prottop&blast_rank=98&RID=EMETN5W1016)

[OCH93208.1](https://www.ncbi.nlm.nih.gov/protein/1045916143?report=genbank&log$=prottop&blast_rank=99&RID=EMETN5W1016)

[KDR80983.1](https://www.ncbi.nlm.nih.gov/protein/648167319?report=genbank&log$=prottop&blast_rank=100&RID=EMETN5W1016)

Alignments

alcohol oxidase [Dichomitus squalens LYAD-421 SS1]

Sequence ID: XP_007363926.1 Length: 608 Number of Matches: 2

See 1 more title(s)

Range 1: 181 to 600

Score

Expect Method

Identities

Positives

Gaps

Frame

691 bits(1782) 0.0()

Compositional matrix adjust.

344/420(82%) 385/420(91%) 0/420(0%)

Features:

Query 9 EGVVDISVPGMSLAIDARGLGTSAELSQQFPFNLDYNSGDTIGLSWTQATIHNGRRVTSA 68

+GVVDISVPG+SLIDARGL SEL +FPFNLDYNSG+TGSWTQATIH+GRRTSA

Sbjct 181 DGVVDISVPGVSLDIDARGLNASQELPDEFPFNLDYNSGNTTGFSWTQATIHDGRRTTSA 240

Query 69 TSYLAEAFNRTNLDILVNTRVTKLVPVGSVNEAPDMRGVQFAQTANGTVHTLKAAKEVIL 128

TSYLAEAFNRTNLDILVNTRVTK+PVGVN PD+RVQFAQ+ANGT++TL+AA+EVIL

Sbjct 241 TSYLAEAFNRTNLDILVNTRVTKIAPVGEVNGVPDLRSVQFAQSANGTLYTLEAAEEVIL 300

Query 129 SAGSIQSPHILMLSGIGNREHLSSFGLDTVVNLPAVGTNLQDHVFLGNSWLVNANFTLDD 188

SAG++QSPHILMLSGIGN++H+SSFG+T+V+LPAVGTN+QDHVFLGNSWLVN+NFTLDD

Sbjct 301 SAGAVQSPHILMLSGIGNKDHISSFGIKTLVDLPAVGTNMQDHVFLGNSWLVNSNFTLDD 360

Query 189 LHRNTTLASEQLQIWEVNGTGLMGLPPTNQFGWFRTNPSVFKDLNATDPSAGPTSANFEL 248

LHRNTL++EQLQIWEVNGTGL+GLPPTNQFWR+PSVF+LNATDPSAGTSANFE+

Sbjct 361 LHRNATLSAEQLQIWEVNGTGLIGLPPTNQFAWLRVDPSVFQSLNATDPSAGTTSANFEM 420

Query 249 IISDNFASKRVALPAEGRFLSIVTNVVSPSSRGNISLASIDPFVAPLINPNLLGTDVDLA 308

IISDNFASKRVALPAEGRFL+VTNVVSPSSRGNISLAS+PFAPLINPNLLGTDVD+A

Sbjct 421 IISDNFASKRVALPAEGRFLTFVTNVVSPSSRGNISLASANPFDAPLINPNLLGTDVDVA 480

Query 309 IMRSAIKAARTFVAAPAWADYVIGEFGAFANATTDAKLDAYIRDNADTVDHPIGTVPMGK 368

IMRSAIKAARFAAPAW+DY+IEFGAFANATTD+LDAYIRDNADTVDHP+GTVPMGK

Sbjct 481 IMRSAIKAARAFAAAPAWSDYIISEFGAFANATTDEELDAYIRDNADTVDHPVGTVPMGK 540

Query 369 GPEGALNADLTVKGTVGLRVVDASAFPFIPSGHTQGPTYILAERAAALIKAAISRGHGSR 428

GEGALNADLTVKGTGLRV+DASAFPF+PSGHTQGPTYILAERAAL+KA+++ SR

Sbjct 541 GCEGALNADLTVKGTAGLRVIDASAFPFVPSGHTQGPTYILAERAAELVKASLGQQERSR 600

Range 2: 148 to 588

Score

Expect

Method

Identities

Positives

Gaps

Frame

502 bits(1292) 8e-164() Compositional matrix adjust. 269/443(61%) 319/443(72%) 19/443(4%)

Features:

Query 526 NNVYDVIVVGDRMTAPVDHHNTSGQFNPALHN-DGVVPISVEGFSLGTDSRVIETTKELP 584

N+ I DMTPDHNT+GQFNPALHNDGVVISVGSL D+R+ ++ELP

Sbjct 148 NEMLPFIFKVDNMTTPTDGHNTTGQFNPALHNTDGVVDISVPGVSLDIDARGLNASQELP 207

Query 585 DQFPFNLDYNSGDTIGFGWVQSTIENGRRVSAATSYLAAALGRPNLDVVVNTRVAKVYPV 644

D+FPFNLDYNSG+TGFWQ+TI+GRR++ATSYLAA RNLD++VNTRVK+PV

Sbjct 208 DEFPFNLDYNSGNTTGFSWTQATIHDGRRTTSATSYLAEAFNRTNLDILVNTRVTKIAPV 267

Query 645 GYQQGKPVFRGVQVAQSPDGTSFHHGR----------------LLLSGIGDLRHLSSFGI 688

G GP RVQAQS+GT+ L+LSGIG+ H+SSFGI

Sbjct 268 GEVNGVPDLRSVQFAQSANGTLYTLEAAEEVILSAGAVQSPHILMLSGIGNKDHISSFGI 327

Query 689 DTLVDLPSVGQSLQDHPFLPIIWVVNSNNTLDDINRNATLAAEALSLWEANGTGPLSLGG 748

TLVDLP+VG++QDHFL W+VNSNTLDD++RNATL+AEL+WENGTG+L

Sbjct 328 KTLVDLPAVGTNMQDHVFLGNSWLVNSNFTLDDLHRNATLSAEQLQIWEVNGTGLIGLPP 387

Query 749 ATQFGWLRIPEAEAFFQPFGVDDPSAGSTSAHFEHLPTNGFISTTVALPSKGHFFTNTVG 808

QFWLR+ + FQ DPSAG+TSA+FE+++FS VALP++GFT

Sbjct 388 TNQFAWLRVDPS--VFQSLNATDPSAGTTSANFEMIISDNFASKRVALPAEGRFLTFVTN 445

Query 809 VMSPTSRGNVSLNSTNPFDAPLINPNLLGNAVDLAIMREAIKSARTFAKAPAWSDYIVHE 868

V+SP+SRGN+SLSNPFDAPLINPNLLG VD+AIMRAIK+ARFAAPAWSDYI+E

Sbjct 446 VVSPSSRGNISLASANPFDAPLINPNLLGTDVDVAIMRSAIKAARAFAAAPAWSDYIISE 505

Query 869 FGASANATTDEALDAFIRANTDTFDHPVGSVAMGKGADAPLTPDLRVRGTVGLRVVDASA 928

FGAANATTDELDA+IRNDTDHPVG+VMGKG+ L DLV+GTGLRV+DASA

Sbjct 506 FGAFANATTDEELDAYIRDNADTVDHPVGTVPMGKGCEGALNADLTVKGTAGLRVIDASA 565

Query 929 FPFIPSGHTQGPTYILAERAAHL 951

FPF+PSGHTQGPTYILAERAAL

Sbjct 566 FPFVPSGHTQGPTYILAERAAEL 588

alcohol oxidase [Dichomitus squalens LYAD-421 SS1]

https://blast.ncbi.nlm.nih.gov/Blast.cgi

4/8


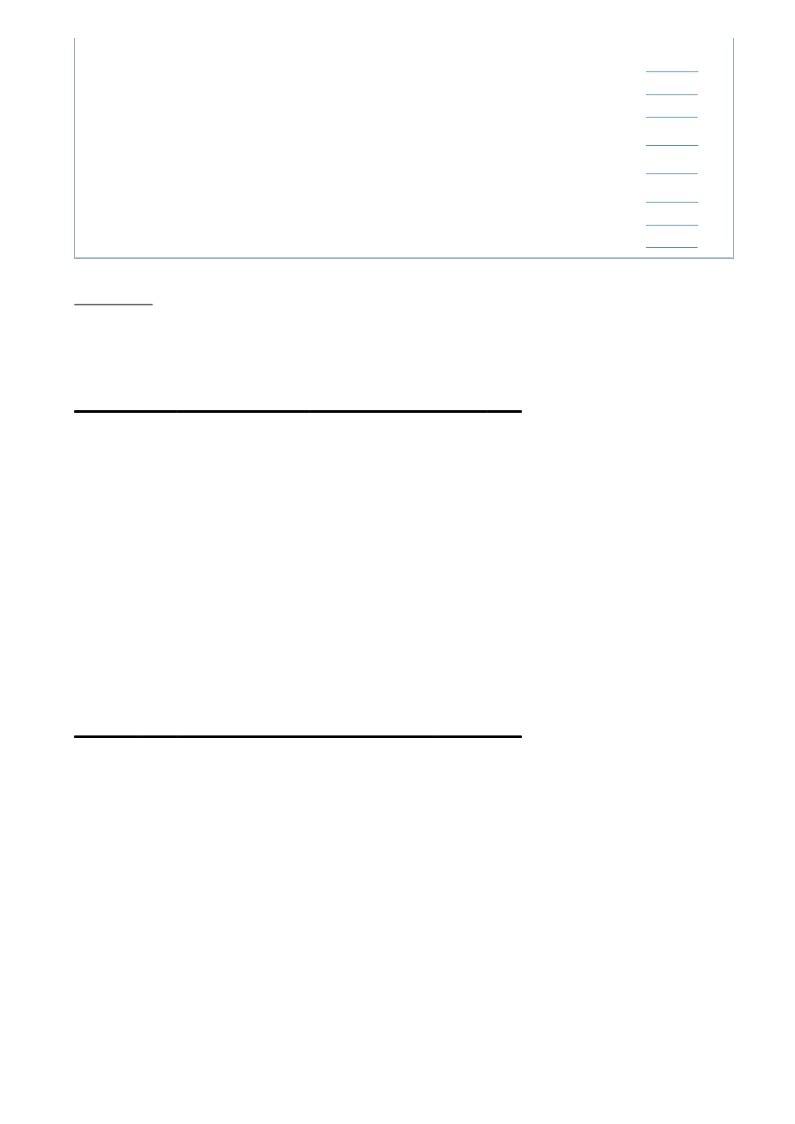


2017/4/9

NCBI Blast:GL23730-R1_1

Sequence ID: XP_007363915.1 Length: 593 Number of Matches: 2

See 1 more title(s)

Range 1: 146 to 588

Score

Expect Method

Identities

Positives

Gaps

Frame

613 bits(1580) 0.0()

Compositional matrix adjust. 300/444(68%) 354/444(79%) 18/444(4%)

Features:

Query 525 LNNVYDVIVVGDRMTAPVDHHNTSGQFNPALHNDGVVPISVEGFSLGTDSRVIETTKELP 584

N+ I DRMTAPVDHHNTGQFNPA+H+GVVPISVEGFL +SRVI+TT+EL

Sbjct 146 FKNLLPFIFAVDRMTAPVDHHNTIGQFNPAIHKNGVVPISVEGFPLEINSRVIDTTRELA 205

Query 585 DQFPFNLDYNSGDTIGFGWVQSTIENGRRVSAATSYLAAALGRPNLDVVVNTRVAKVYPV 644

+QFPFNLDYNSG+TIGFGWQ+TI+GRV++A+SYLAA+RPNLDVVVNTRVKV V

Sbjct 206 EQFPFNLDYNSGNTIGFGWTQNTIHDGHRVTSASSYLAQAINRPNLDVVVNTRVTKVVSV 265

Query 645 GYQQGKPVFRGVQVAQSPDGTSFHHGR-----------------LLLSGIGDLRHLSSFG 687

G+QGKPVFRGV++AQSDG H + L+LSGIGD HL++G

Sbjct 266 GREQGKPVFRGVEIAQSADG-PVHTLKAKKEVILSAGSLKTPHILMLSGIGDAAHLTALG 324

Query 688 IDTLVDLPSVGQSLQDHPFLPIIWVVNSNNTLDDINRNATLAAEALSLWEANGTGPLSLG 747

I +V+LP+VGQ+LQDHFL WVNSNTLDD++RN+TLAAEAL+LW+NGTGPLLG

Sbjct 325 IKPVVNLPAVGQNLQDHVFLGNSWVANSTNTLDDLHRNSTLAAEALALWKINGTGPLGLG 384

Query 748 GATQFGWLRIPEAEAFFQPFGVDDPSAGSTSAHFEHLPTNGFISTTVALPSKGHFFTNTV 807

A+QFGWLR+PA FF+ GV+DPSAGTSAHFE+PTNF+STVLP++GHFF+

Sbjct 385 AASQFGWLRVPNATGFFRSLGVEDPSAGETSAHFEQIPTNAFVSKTVPLPAEGHFFSIIT 444

Query 808 GVMSPTSRGNVSLNSTNPFDAPLINPNLLGNAVDLAIMREAIKSARTFAKAPAWSDYIVH 867

V+SPT+RGNV+LNSTNPFDAPLINPNLLG+VD+AIMREA+K+ARF APWSDYIV

Sbjct 445 AVVSPTARGNVTLNSTNPFDAPLINPNLLGSPVDVAIMREAVKAARAFVTAPTWSDYIVS 504

Query 868 EFGASANATTDEALDAFIRANTDTFDHPVGSVAMGKGADAPLTPDLRVRGTVGLRVVDAS 927

EFG ANATTD+L++IRN+DTDHPVG+VAMG+G+APL LRV+GTVGLRVVDAS

Sbjct 505 EFGVFANATTDDELEVYIRKNSDTVDHPVGTVAMGQGTNAPLDSQLRVKGTVGLRVVDAS 564

Query 928 AFPFIPSGHTQGPTYILAERAAHL 951

AFPFIPSGHTQGPTYILAERAAHL

Sbjct 565 AFPFIPSGHTQGPTYILAERAAHL 588

Range 2: 176 to 593

Score

Expect Method

Identities

Positives

Gaps

Frame

553 bits(1425) 0.0()

Compositional matrix adjust.

273/418(65%) 335/418(80%) 3/418(0%)

Features:

Query 7 IHE-GVVDISVPGMSLAIDARGLGTSAELSQQFPFNLDYNSGDTIGLSWTQATIHNGRRV 65

IH+GVVISVG LI++R+T+EL++QFPFNLDYNSG+TIG WTQTIH+GRV

Sbjct 176 IHKNGVVPISVEGFPLEINSRVIDTTRELAEQFPFNLDYNSGNTIGFGWTQNTIHDGHRV 235

Query 66 TSATSYLAEAFNRTNLDILVNTRVTKLVPVGSVNEAPDMRGVQFAQTANGTVHTLKAAKE 125

TSA+SYLA+ANRNLD++VNTRVTK+VVG P RGV+AQ+A+GVHTLKAKE

Sbjct 236 TSASSYLAQAINRPNLDVVVNTRVTKVVSVGREQGKPVFRGVEIAQSADGPVHTLKAKKE 295

Query 126 VILSAGSIQSPHILMLSGIGNREHLSSFGLDTVVNLPAVGTNLQDHVFLGNSWLVNANFT 185

VILSAGS+++PHILMLSGIG+ HL++G+ VVNLPAVGNLQDHVFLGNSW+N+ T

Sbjct 296 VILSAGSLKTPHILMLSGIGDAAHLTALGIKPVVNLPAVGQNLQDHVFLGNSWVANSTNT 355

Query 186 LDDLHRNTTLASEQLQIWEVNGTGLMGLPPTNQFGWFRTNPSV--FKDLNATDPSAGPTS 243

LDDLHRN+TLA+EL+W++NGTG+GL +QFGWR + F+L DPSAGTS

Sbjct 356 LDDLHRNSTLAAEALALWKINGTGPLGLGAASQFGWLRVPNATGFFRSLGVEDPSAGETS 415

Query 244 ANFELIISDNFASKRVALPAEGRFLSIVTNVVSPSSRGNISLASIDPFVAPLINPNLLGT 303

A+FEI++FSKVLPAEGFSI+TVVSP++RGN++LS+PFAPLINPNLLG+

Sbjct 416 AHFEQIPTNAFVSKTVPLPAEGHFFSIITAVVSPTARGNVTLNSTNPFDAPLINPNLLGS 475

Query 304 DVDLAIMRSAIKAARTFVAAPAWADYVIGEFGAFANATTDAKLDAYIRDNADTVDHPIGT 363

VD+AIMRA+KAARFVAPW+DY++EFGFANATTD+L+YIRN+DTVDHP+GT

Sbjct 476 PVDVAIMREAVKAARAFVTAPTWSDYIVSEFGVFANATTDDELEVYIRKNSDTVDHPVGT 535

Query 364 VPMGKGPEGALNADLTVKGTVGLRVVDASAFPFIPSGHTQGPTYILAERAAALIKAAI 421

VMG+G L++LVKGTVGLRVVDASAFPFIPSGHTQGPTYILAERAAL+++++

Sbjct 536 VAMGQGTNAPLDSQLRVKGTVGLRVVDASAFPFIPSGHTQGPTYILAERAAHLVRSSL 593

alcohol oxidase [Dichomitus squalens LYAD-421 SS1]

Sequence ID: XP_007363927.1 Length: 597 Number of Matches: 2

See 1 more title(s)

Range 1: 158 to 590

Score

Expect Method

Identities

Positives

Gaps

Frame

552 bits(1423) 0.0()

Compositional matrix adjust. 287/434(66%) 339/434(78%) 19/434(4%)

Features:

Query 536 DRMTAPVDHHNTSGQFNPALHNDGVVPISVEGFSLGTDSRVIETTKELPDQFPFNLDYNS 595

DMTPVDHHNT+G+FNPLH+GVISV+ LT++RV+ETT++LPDFPFNLDYNS

Sbjct 158 DHMTPPVDHHNTTGEFNPVLHKNGPVSISVDSLILDTNTRVLETTQQLPDLFPFNLDYNS 217

Query 596 GDTIGFGWVQSTIENGRRVSAATSYLAAALGRPNLDVVVNTRVAKVYPVGYQQGKPVFRG 655

G++G WQ+INGRV++ATSYLAA+RNLDVVVNTRVKV+PVG+GKPVFRG

Sbjct 218 GTSVGVAWKQNAILNGHRVTSATSYLAQAMNRKNLDVVVNTRVTKVFPVGTEDGKPVFRG 277

Query 656 VQVAQSPDGTSFHHGR-----------------LLLSGIGDLRHLSSFGIDTLVDLPSVG 698

V+AQ+DG H + L+LSGIGD HLSSFGIT+VDLPVG

Sbjct 278 VEFAQTADGPR-HTLKATQEVILSAGSLKTPHILMLSGIGDSAHLSSFGIKTIVDLPGVG 336

Query 699 QSLQDHPFLPIIWVVNSNNTLDDINRNATLAAEALSLWEANGTGPLSLG-GATQFGWLRI 757

Q+LDHFL WVNSNTLDD+RNATAAEAL+LW+ANTGPLS+ G++QFG++RI

Sbjct 337 QNLHDHAFLGNSWTVNSTNTLDDLQRNATAAAEALALWKANATGPLSVAAGSSQFGFVRI 396

Query 758 PEAEAFFQPFGVDDPSAGSTSAHFEHLPTNGFISTTVALPSKGHFFTNTVGVMSPTSRGN 817

PAAFFQFGV+DPSAGTSHEH+PTN++STTV+P++GHFF V+SPT+RGN

Sbjct 397 PNATAFFQQFGVEDPSAGPTSGHIEHIPTNSYVSTTVPIPAEGHFFAIITAVISPTARGN 456

Query 818 VSLNSTNPFDAPLINPNLLGNAVDLAIMREAIKSARTFAKAPAWSDYIVHEFGASANATT 877

V+LS++PFDAPLINPLLGNVDLAIMRE+K+ARFKAPAWSYI+EFG NATT

Sbjct 457 VTLRSSDPFDAPLINPALLGNDVDLAIMREGVKAARAFVKAPAWSGYIISEFGEFGNATT 516

https://blast.ncbi.nlm.nih.gov/Blast.cgi

5/8


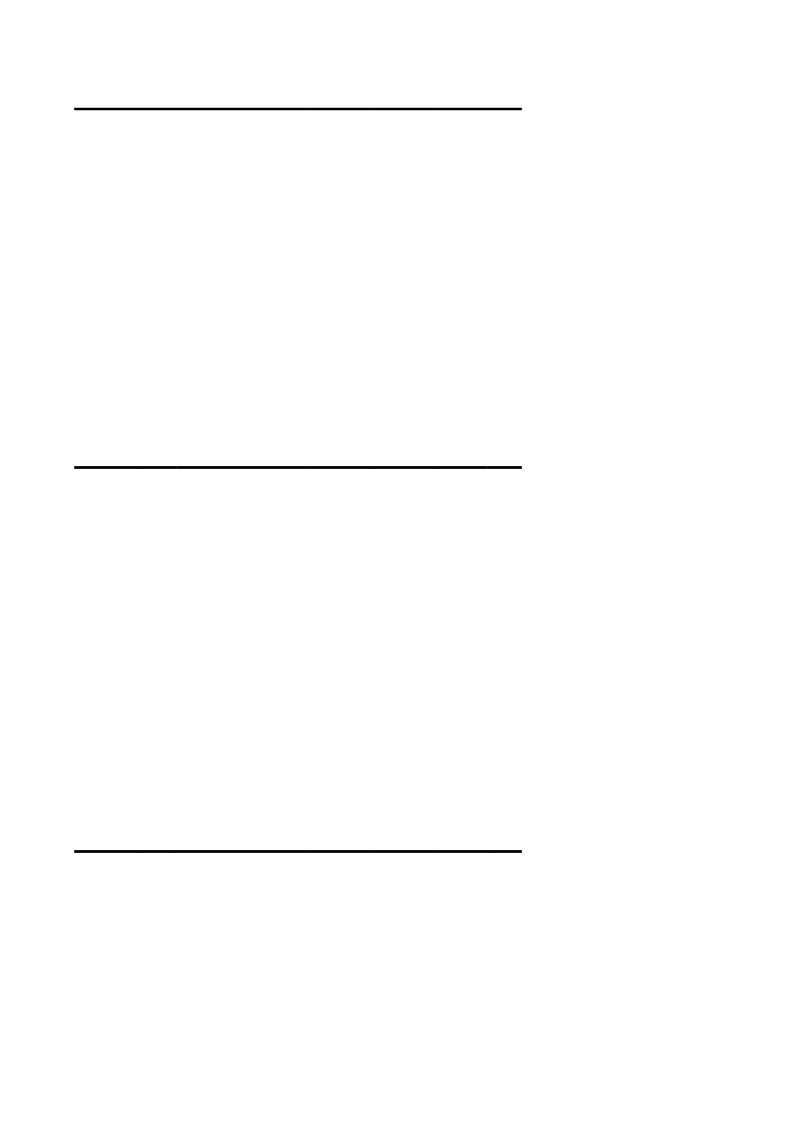


2017/4/9

NCBI Blast:GL23730-R1_1

Query 878 DEALDAFIRANTDTFDHPVGSVAMGKGADAPLTPDLRVRGTVGLRVVDASAFPFIPSGHT 937

++AL++IRN+TDHPVGSVAMGK APLTPDL+VRGTVGLRVVDASAFPFIPSGHT

Sbjct 517 EDALNEYIRNNSATVDHPVGSVAMGKDEPAPLTPDLKVRGTVGLRVVDASAFPFIPSGHT 576

Query 938 QGPTYILAERAAHL 951

QGPTYILAERAAHL

Sbjct 577 QGPTYILAERAAHL 590

Range 2: 175 to 597

Score

Expect

Method

Identities

Positives

Gaps

Frame

483 bits(1243) 1e-156() Compositional matrix adjust. 251/423(59%) 313/423(73%) 3/423(0%)

Features:

Query 4 PCRIHEGVVDISVPGMSLAIDARGLGTSAELSQQFPFNLDYNSGDTIGLSWTQATIHNGR 63

P GVISV +L +RLT++L FPFNLDYNSG++G++WQ ING

Sbjct 175 PVLHKNGPVSISVDSLILDTNTRVLETTQQLPDLFPFNLDYNSGTSVGVAWKQNAILNGH 234

Query 64 RVTSATSYLAEAFNRTNLDILVNTRVTKLVPVGSVNEAPDMRGVQFAQTANGTVHTLKAA 123

RVTSATSYLA+ANRNLD++VNTRVTK+PVG++ P RGV+FAQTA+G HTLKA

Sbjct 235 RVTSATSYLAQAMNRKNLDVVVNTRVTKVFPVGTEDGKPVFRGVEFAQTADGPRHTLKAT 294

Query 124 KEVILSAGSIQSPHILMLSGIGNREHLSSFGLDTVVNLPAVGTNLQDHVFLGNSWLVNAN 183

+EVILSAGS+++PHILMLSGIG+ HLSSFG+T+V+LPVGNLDHFLGNSWVN+

Sbjct 295 QEVILSAGSLKTPHILMLSGIGDSAHLSSFGIKTIVDLPGVGQNLHDHAFLGNSWTVNST 354

Query 184 FTLDDLHRNTTLASEQLQIWEVNGTGLMGLPP-TNQFGWFR--TNPSVFKDLNATDPSAG 240

TLDDLRNTA+EL+W+NTG++ ++QFG+R +F+ DPSAG

Sbjct 355 NTLDDLQRNATAAAEALALWKANATGPLSVAAGSSQFGFVRIPNATAFFQQFGVEDPSAG 414

Query 241 PTSANFELIISDNFASKRVALPAEGRFLSIVTNVVSPSSRGNISLASIDPFVAPLINPNL 300

PTS+EI++++S V+PAEGF+I+TV+SP++RGN++LSDPFAPLINPL

Sbjct 415 PTSGHIEHIPTNSYVSTTVPIPAEGHFFAIITAVISPTARGNVTLRSSDPFDAPLINPAL 474

Query 301 LGTDVDLAIMRSAIKAARTFVAAPAWADYVIGEFGAFANATTDAKLDAYIRDNADTVDHP 360

LGDVDLAIMR +KAARFVAPAW+Y+IEFGFNATT+ L+YIR+N+TVDHP

Sbjct 475 LGNDVDLAIMREGVKAARAFVKAPAWSGYIISEFGEFGNATTEDALNEYIRNNSATVDHP 534

Query 361 IGTVPMGKGPEGALNADLTVKGTVGLRVVDASAFPFIPSGHTQGPTYILAERAAALIKAA 420

+G+VMGK L DLV+GTVGLRVVDASAFPFIPSGHTQGPTYILAERAAL++A

Sbjct 535 VGSVAMGKDEPAPLTPDLKVRGTVGLRVVDASAFPFIPSGHTQGPTYILAERAAHLVRAD 594

Query 421 ISR 423

++

Sbjct 595 LKK 597

hypothetical protein GYMLUDRAFT_394379 [Gymnopus luxurians FD-317 M1]

Sequence ID: KIK51433.1 Length: 600 Number of Matches: 2

Range 1: 179 to 599

Score

Expect

Method

Identities

Positives

Gaps

Frame

448 bits(1153) 2e-143() Compositional matrix adjust. 231/425(54%) 306/425(72%) 11/425(2%)

Features:

Query 8 HEGVVDISVPGMSLAIDARGLGTSAELSQQFPFNLDYNSGDTIGLSWTQATIHNGRRVTS 67

H+GV SVG+L D+RLGS+ELS+FPFN+DNSGDIG+SWQTING+RV+

Sbjct 179 HDGPVGTSVSGLRLPTDSRILGASSELSAEFPFNIDTNSGDPIGISWAQFTIANGQRVNA 238

Query 68 ATSYLAEAFNRTNLDILVNTRVTKLVPVGSVNEAPDMRGVQFAQTANGTVHTLKAAKEVI 127

AT++L A+R+NLDILVNTVTK+V GS +P RGV+FAQAGV+LA++EVI

Sbjct 239 ATAFLEPALSRSNLDILVNTHVTKIVQTGSQEKTPIFRGVEFAQNALGPVFSLNASREVI 298

Query 128 LSAGSIQSPHILMLSGIGNREHLSSFGLDTVVNLPAVGTNLQDHVFLGNSWLVNANFTLD 187

LSAG++++PH+LMLSGIG++HLS+G+ +VNLPVGNLQDH L +VN+ TLD

Sbjct 299 LSAGAVKTPHLLMLSGIGDPDHLSAVGVSPIVNLPDVGQNLQDHALLTIQFTVNSTDTLD 358

Query 188 DLHRNTTLASEQLQIWEVNGTGLMGLPPTNQFGWFR--TNPSVFKDLNATDPSAGPTSAN 245

+L+NTT +QL++W+NTG+LP+NQ+GWFR NS+FK N+DPSAGPTSA+

Sbjct 359 NLSQNTTFLEQQLELWQANRTGELVLPSSNQWGWFRLPENSSIFK--NHSDPSAGPTSAH 416

Query 246 FELIISDNFASKRVALPAEGRFLSIVTNVVSPSSRGNISLASIDPFVAPLINPNLLGTDV 305

FE+++DFS PEG++ITNVVSP++RGIS+S+PF+P+I+N+LG++

Sbjct 417 FEFLLTDAFFSFAGPTPNEGHYFTIFTNVVSPAARGQISINSTNPFDSPVIDSNILGSNF 476

Query 306 DLAIMRSAIKAARTFVAAPAWADYVIGEFGAFANATTDAKLDAYIRDNADTVDHPIGTVP 365

D MRA+K+ARF++AAW +++EFGA ATD++D+++N TVDH TV

Sbjct 477 DRLTMREAVKSARRFMSASAWEGWILDEFGASKQAQTDDEIDQFVSNNTVTVDHVSCTVS 536

Query 366 MGKGPE-----GALNADLTVKGTVGLRVVDASAFPFIPSGHTQGPTYILAERAAALIKAA 420

MGK + GALNDLTVKG+GLRVVDASFPF+PSHTQPTYI+AERA LIKA

Sbjct 537 MGKAGDTSKGSGALNPDLTVKGAMGLRVVDASVFPFVPSAHTQTPTYIVAERAVDLIKAY 596

Query 421 ISRGH 425

+GH

Sbjct 597 --QGH 599

Range 2: 147 to 592

Score

Expect

Method

Identities

Positives

Gaps

Frame

418 bits(1074) 1e-131() Compositional matrix adjust. 218/448(49%) 292/448(65%) 24/448(5%)

Features:

Query 526 NNVYDVIVVGDRMTAPVDHHNTSGQFNPALH-NDGVVPISVEGFSLGTDSRVIETTKELP 584

++++ ++ +++T+PVDHNTS+ P++H+DGV SVG LTDSR++ +EL

Sbjct 147 DSLFPYMLKMEKLTSPVDHRNTSMEVLPSIHGHDGPVGTSVSGLRLPTDSRILGASSELS 206

Query 585 DQFPFNLDYNSGDTIGFGWVQSTIENGRRVSAATSYLAAALGRPNLDVVVNTRVAKVYPV 644

+FPFN+DNSGDIG WQTING+RV+AAT++L ALRNLD++VNTVK+

Sbjct 207 AEFPFNIDTNSGDPIGISWAQFTIANGQRVNAATAFLEPALSRSNLDILVNTHVTKIVQT 266

Query 645 GYQQGKPVFRGVQVAQSPDGTSFHHGR----------------LLLSGIGDLRHLSSFGI 688

GQ+ P+FRGV+AQ+ G F L+LSGIGD HLS+G+

Sbjct 267 GSQEKTPIFRGVEFAQNALGPVFSLNASREVILSAGAVKTPHLLMLSGIGDPDHLSAVGV 326

Query 689 DTLVDLPSVGQSLQDHPFLPIIWVVNSNNTLDDINRNATLAAEALSLWEANGTGPLSLGG 748

+V+LPVGQ+LQDH LI+VNS+TLD++++NT +LLW+ANTGLL

Sbjct 327 SPIVNLPDVGQNLQDHALLTIQFTVNSTDTLDNLSQNTTFLEQQLELWQANRTGELVLPS 386

https://blast.ncbi.nlm.nih.gov/Blast.cgi

6/8


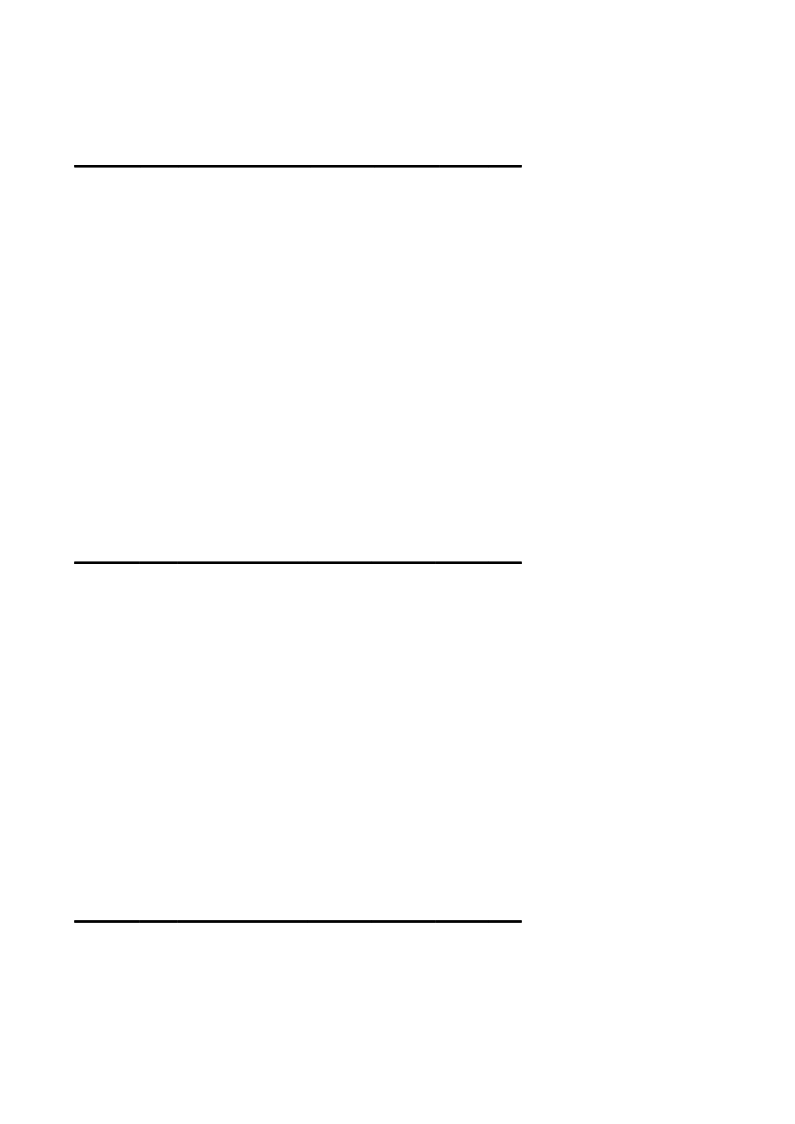


2017/4/9

NCBI Blast:GL23730-R1_1

Sbjct 327 SPIVNLPDVGQNLQDHALLTIQFTVNSTDTLDNLSQNTTFLEQQLELWQANRTGELVLPS 386

Query 749 ATQFGWLRIPEAEAFFQPFGVDDPSAGSTSAHFEHLPTNGFISTTVALPSKGHFFTNTVG 808

+Q+GWR+PE +F+ DPSAGTSAHFELT+FS P++GH+FT

Sbjct 387 SNQWGWFRLPENSSIFKNHS--DPSAGPTSAHFEFLLTDAFFSFAGPTPNEGHYFTIFTN 444

Query 809 VMSPTSRGNVSLNSTNPFDAPLINPNLLGNAVDLAIMREAIKSARTFAKAPAWSDYIVHE 868

V+SP+RG+S+NSTNPFD+P+I+N+LG+ D MREA+KSARF AAW +I+E

Sbjct 445 VVSPAARGQISINSTNPFDSPVIDSNILGSNFDRLTMREAVKSARRFMSASAWEGWILDE 504

Query 869 FGASANATTDEALDAFIRANTDTFDHPVGSVAMGKGAD-----APLTPDLRVRGTVGLRV 923

FGAS ATD++DF+ NTTDH +V+MGK D LPDLV+G+GLRV

Sbjct 505 FGASKQAQTDDEIDQFVSNNTVTVDHVSCTVSMGKAGDTSKGSGALNPDLTVKGAMGLRV 564

Query 924 VDASAFPFIPSGHTQGPTYILAERAAHL 951

VDASFPF+PSHTQPTYI+AERA L

Sbjct 565 VDASVFPFVPSAHTQTPTYIVAERAVDL 592

alcohol oxidase [Fomitiporia mediterranea MF3/22]

Sequence ID: XP_007269675.1 Length: 636 Number of Matches: 2

See 1 more title(s)

Range 1: 175 to 606

Score

Expect

Method

Identities

Positives

Gaps

Frame

447 bits(1149) 3e-142() Compositional matrix adjust. 235/435(54%) 313/435(71%) 12/435(2%)

Features:

Query 2 IAPC-RIHEGVVDISVPGMSLAIDARGLGTSAELSQQFPFNLDYNSGDTIGLSWTQATIH 60

IPC HEGVIS+G+ D + S++LS+FPFNLDYNSG+TIG+SWQTI

Sbjct 175 IDPCIHGHEGPVRISLTGVPEPTDEWVIAASSQLSDEFPFNLDYNSGNTIGISWKQNTIA 234

Query 61 NGRRVTSATSYLAEAFNRTNLDILVNTRVTKLVPVGSVNEAPDMRGVQFAQTANGTVHTL 120

+GRV++A+Y+AA+RNLD+LVNTVT++++G++AP +GVQF+Q++GV+L

Sbjct 235 HGERVSAAKAYIAPALSRPNLDVLVNTHVTRVLQIGTESGAPVFKGVQFSQSSEGVVYGL 294

Query 121 KAAKEVILSAGSIQSPHILMLSGIGNREHLSSFGLDTVVNLPAVGTNLQDHVFLGNSWLV 180

A++EVILSAG++++PHILMLSGIGNEHLSSF+ +V++PVGNLQDH + S+V

Sbjct 295 NASREVILSAGAVKTPHILMLSGIGNAEHLSSFSIKPIVDIPDVGNNLQDHPVVTASFSV 354

Query 181 NANFTLDDLHRNTTLASEQLQIWEVNGTGLMGLPPTNQFGWFR--TNPSVFKDLNATDPS 238

N+ TLD+L NT +++ +W N+GL+G TNQFGWFR NS+F +ATDPS

Sbjct 355 NSTNTLDNLTENATFLAQEEALWLKNRSGLLGWVSTNQFGWFRIPVNSSIFD--SATDPS 412

Query 239 AGPTSANFELIISDNFASKRVALPAE-GRFLSIVTNVVSPSSRGNISLASIDPFVAPLIN 297

AGPTSA+FEI++FS PA GF+++ VVSP+SRGNI+LAS+PF+P+I+

Sbjct 413 AGPTSAHFEFIFTNGFTSFSEPFPASGGHFFTVLVAVVSPTSRGNITLASSNPFDSPVID 472

Query 298 PNLLGTDVDLAIMRSAIKAARTFVAAPAWADYVIGEFGAFANATTDAKLDAYIRDNADTV 357

PNLLGT D+ +RAIK+ARFV+APAW +++E+GAF+ATD++++R A+T+

Sbjct 473 PNLLGTAFDIFTVREAIKSARKFVSAPAWQGWILEEYGAFSEAHTDEQIEEFARQTANTI 532

Query 358 DHPIGTVPMGK-----GPEGALNADLTVKGTVGLRVVDASAFPFIPSGHTQGPTYILAER 412

DH TVMGK GALN+DLTVKGTVGLRVVDASAFPFISGHTQ YILAER

Sbjct 533 DHVSCTVAMGKTGSSGSGTGALNSDLTVKGTVGLRVVDASAFPFIISGHTQAAVYILAER 592

Query 413 AAALIKAAISRGHGS 427

AALIK++RG S

Sbjct 593 AADLIKLSL-RGECS 606

Range 2: 160 to 596

Score

Expect

Method

Identities

Positives

Gaps

Frame

427 bits(1098) 1e-134() Compositional matrix adjust. 228/439(52%) 289/439(65%) 25/439(5%)

Features:

Query 536 DRMTAPVDHHNTSGQFNPALH-NDGVVPISVEGFSLGTDSRVIETTKELPDQFPFNLDYN 594

+++ PDHHNT+G++P+H++GVIS+G TD VI ++LD+FPFNLDYN

Sbjct 160 EKLVPPTDHHNTTGEIDPCIHGHEGPVRISLTGVPEPTDEWVIAASSQLSDEFPFNLDYN 219

Query 595 SGDTIGFGWVQSTIENGRRVSAATSYLAAALGRPNLDVVVNTRVAKVYPVGYQQGKPVFR 654

SG+TIG WQ+TI+GRVSAA+Y+AALRPNLDV+VNTV+V +G+GPVF+

Sbjct 220 SGNTIGISWKQNTIAHGERVSAAKAYIAPALSRPNLDVLVNTHVTRVLQIGTESGAPVFK 279

Query 655 GVQVAQSPDGTSF--HHGR--------------LLLSGIGDLRHLSSFGIDTLVDLPSVG 698

GVQ+QS+G + + R L+LSGIG+ HLSSFI +VD+PVG

Sbjct 280 GVQFSQSSEGVVYGLNASREVILSAGAVKTPHILMLSGIGNAEHLSSFSIKPIVDIPDVG 339

Query 699 QSLQDHPFLPIIWVVNSNNTLDDINRNATLAAEALSLWEANGTGPLSLGGATQFGWLRIP 758

+LQDHP+ +VNSNTLD++ NAT A+ +LW N+GL QFGWRIP

Sbjct 340 NNLQDHPVVTASFSVNSTNTLDNLTENATFLAQEEALWLKNRSGLLGWVSTNQFGWFRIP 399

Query 759 EAEAFFQPFGVDDPSAGSTSAHFEHLPTNGFISTTVALP-SKGHFFTNTVGVMSPTSRGN 817

+F DPSAGTSAHFE+TNGFS+ PSGHFFT VV+SPTSRGN

Sbjct 400 VNSSIFD--SATDPSAGPTSAHFEFIFTNGFTSFSEPFPASGGHFFTVLVAVVSPTSRGN 457

Query 818 VSLNSTNPFDAPLINPNLLGNAVDLAIMREAIKSARTFAKAPAWSDYIVHEFGASANATT 877

++LS+NPFD+P+I+PNLLGAD+ +REAIKSARF APAW +I+E+GA+AT

Sbjct 458 ITLASSNPFDSPVIDPNLLGTAFDIFTVREAIKSARKFVSAPAWQGWILEEYGAFSEAHT 517

Query 878 DEALDAFIRANTDTFDHPVGSVAMGKGADA-----PLTPDLRVRGTVGLRVVDASAFPFI 932

DE++FR +TDH +VAMGK + L DLV+GTVGLRVVDASAFPFI

Sbjct 518 DEQIEEFARQTANTIDHVSCTVAMGKTGSSGSGTGALNSDLTVKGTVGLRVVDASAFPFI 577

Query 933 PSGHTQGPTYILAERAAHL 951

SGHTQ YILAERAAL

Sbjct 578 ISGHTQAAVYILAERAADL 596

BLAST is a registered trademark of the National Library of Medicine

[Support center](https://support.ncbi.nlm.nih.gov/ics/support/KBList.asp?style=classic&deptID=28049&folderID=11&) [Mailing list](https://blast.ncbi.nlm.nih.gov/Blast.cgi?CMD=Web&PAGE_TYPE=BlastDocs&DOC_TYPE=MailList)

https://blast.ncbi.nlm.nih.gov/Blast.cgi

[YouTube](https://www.youtube.com/ncbinlm)

7/8


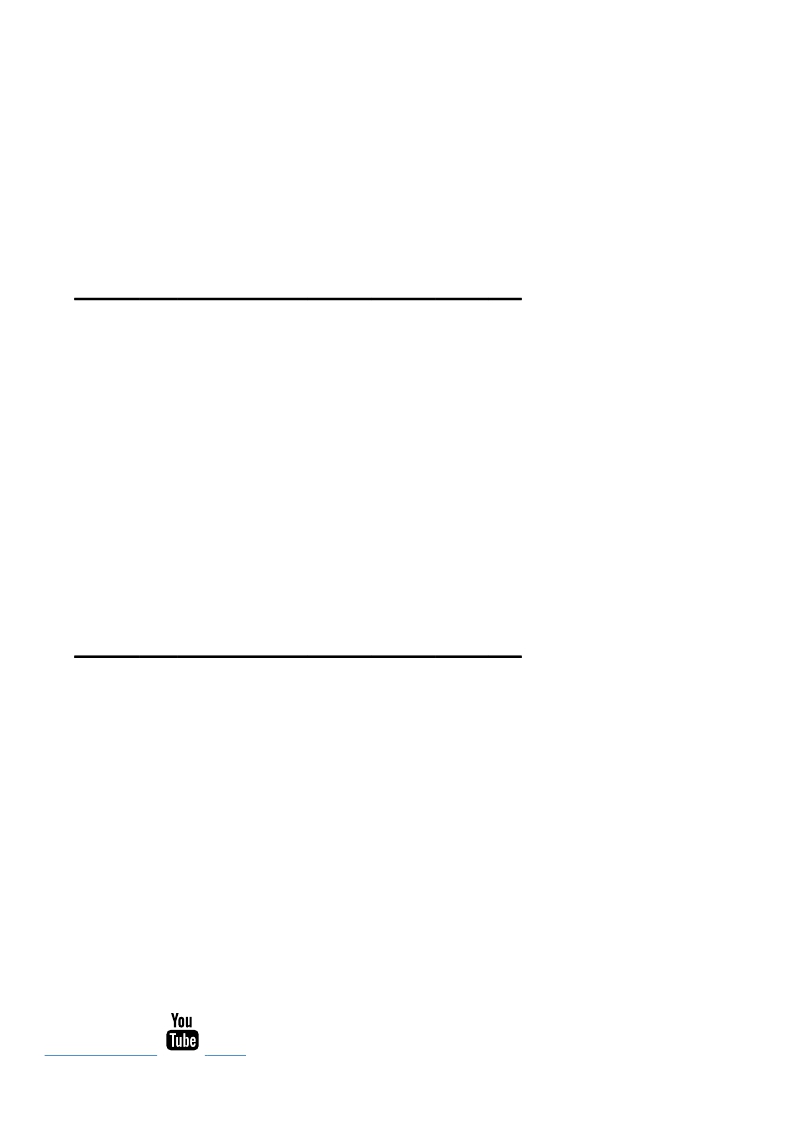


2017/4/9

NCBI Blast:GL23730-R1_1

[National Library Of Medicine](https://www.nlm.nih.gov/)

[National Institutes Of Health](https://www.nih.gov/)

[U.S. Department of Health & Human Services](https://www.hhs.gov/)

[USA.gov](https://www.usa.gov/)

[NCBI](https://www.ncbi.nlm.nih.gov/)

[*National Center for Biotechnology Information,*](https://www.ncbi.nlm.nih.gov/) *U.S. National Library of Medicine 8600 Rock ville Pik e, Bethesda MD, 20894 USA*

[Policies and Guidelines](https://www.ncbi.nlm.nih.gov/home/about/policies.shtml) | [Contact](https://www.ncbi.nlm.nih.gov/home/about/contact.shtml)

https://blast.ncbi.nlm.nih.gov/Blast.cgi

8/8


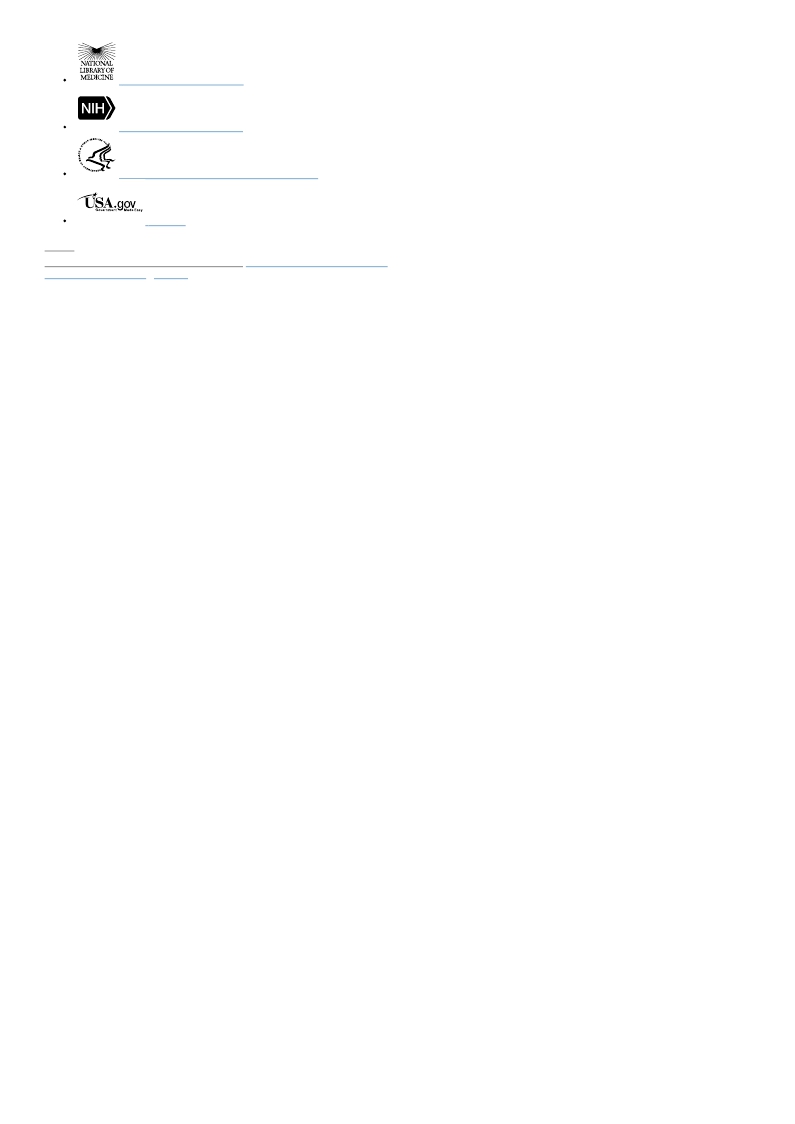

Supplement: Supplementary file 17 — Supplementary File 3b [file 41598_2017_4303_MOESM17_ESM.doc]
