# Supplementary material for: Genome-wide Identification and Characterization of Natural Antisense Transcripts by Strand-specific RNA Sequencing in Ganoderma lucidum
Source: Sci Rep. 2017 Jul 18;7:5711. doi: 10.1038/s41598-017-04303-6 (PMC5515960; doi:10.1038/s41598-017-04303-6)

2017/4/9

NCBI Blast:GL22256-R1_1

[BLAST ®](https://blast.ncbi.nlm.nih.gov/Blast.cgi) » blastp suite » RID-EMEE2DT2013

BLAST Results

Job title: GL22256-R1_1

RID

[EMEE2DT2013](https://blast.ncbi.nlm.nih.gov/Blast.cgi?CMD=Get&RID=EMEE2DT2013) (Expires on 04-10 21:40 pm)

Query ID

lcl|Query_265031

Database Name

nr

Description

Molecule type

Query Length

GL22256-R1_1

amino acid

788

Description All non-redundant GenBank CDS

translations+PDB+SwissProt+PIR+PRF excluding

environmental samples from WGS projects

Program BLASTP 2.6.0+

New Analyze your query with SmartBLAST

Graphic Summary

Putative conserved domains have been detected, click on the image below for detailed results.

Distribution of the top 100 Blast Hits on 100 subject sequences

Color key for alignment scores

<40

40-50

50-80

80-200

>=200

Query

1

150

300

450

600

750

https://blast.ncbi.nlm.nih.gov/Blast.cgi

1/7


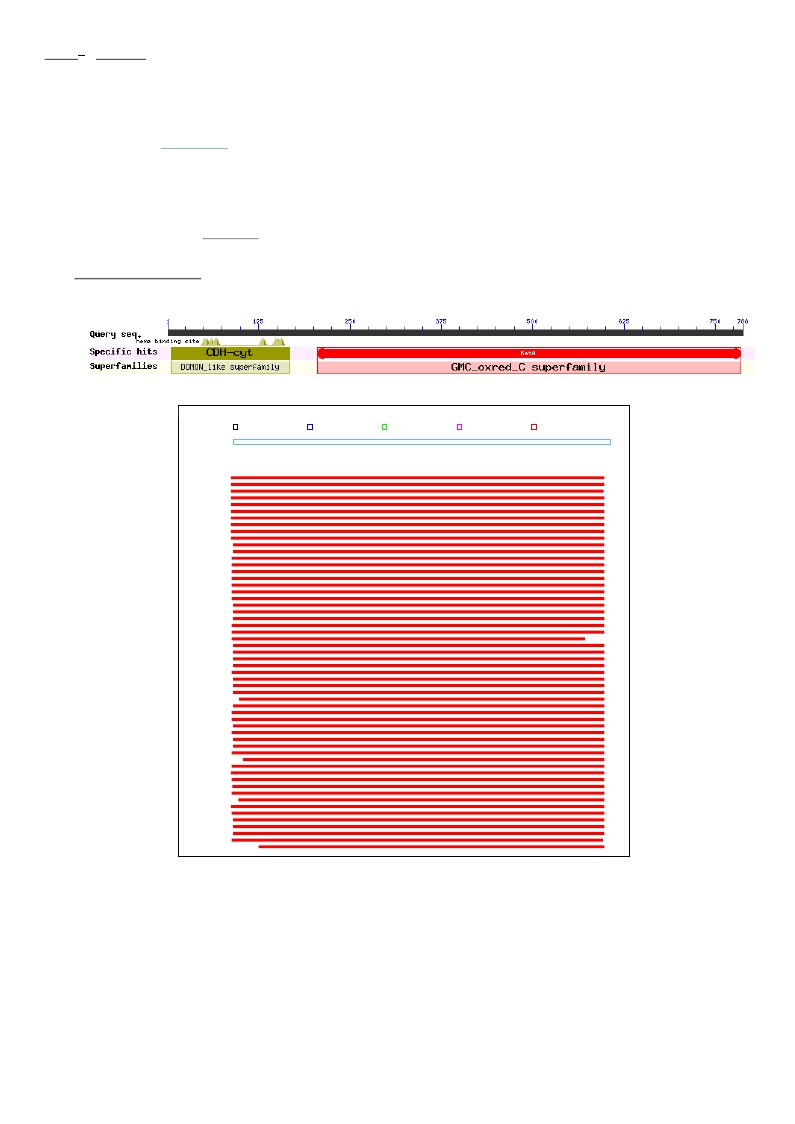


2017/4/9

Descriptions

Sequences producing significant alignments:

NCBI Blast:GL22256-R1_1

Description

[Max](https://blast.ncbi.nlm.nih.gov/Blast.cgi?CMD=Get&ALIGNMENTS=100&ALIGNMENT_VIEW=Pairwise&CDD_RID=EMEE2RBX016&CDD_SEARCH_STATE=0&DATABASE_SORT=0&DESCRIPTIONS=100&DYNAMIC_FORMAT=on&FIRST_QUERY_NUM=0&FORMAT_OBJECT=Alignment&FORMAT_PAGE_TARGET=&FORMAT_TYPE=HTML&GET_SEQUENCE=yes&I_THRESH=&LINE_LENGTH=60&MASK_CHAR=2&MASK_COLOR=1&NEW_VIEW=yes&NUM_OVERVIEW=100&PAGE=Proteins&QUERY_INDEX=0&QUERY_NUMBER=0&RESULTS_PAGE_TARGET=&RID=EMEE2DT2013&SHOW_LINKOUT=yes&SHOW_OVERVIEW=yes&STEP_NUMBER=&WORD_SIZE=6&OLD_VIEW=false&DISPLAY_SORT=1&HSP_SORT=1)

[Total](https://blast.ncbi.nlm.nih.gov/Blast.cgi?CMD=Get&ALIGNMENTS=100&ALIGNMENT_VIEW=Pairwise&CDD_RID=EMEE2RBX016&CDD_SEARCH_STATE=0&DATABASE_SORT=0&DESCRIPTIONS=100&DYNAMIC_FORMAT=on&FIRST_QUERY_NUM=0&FORMAT_OBJECT=Alignment&FORMAT_PAGE_TARGET=&FORMAT_TYPE=HTML&GET_SEQUENCE=yes&I_THRESH=&LINE_LENGTH=60&MASK_CHAR=2&MASK_COLOR=1&NEW_VIEW=yes&NUM_OVERVIEW=100&PAGE=Proteins&QUERY_INDEX=0&QUERY_NUMBER=0&RESULTS_PAGE_TARGET=&RID=EMEE2DT2013&SHOW_LINKOUT=yes&SHOW_OVERVIEW=yes&STEP_NUMBER=&WORD_SIZE=6&OLD_VIEW=false&DISPLAY_SORT=2&HSP_SORT=1)

[Query](https://blast.ncbi.nlm.nih.gov/Blast.cgi?CMD=Get&ALIGNMENTS=100&ALIGNMENT_VIEW=Pairwise&CDD_RID=EMEE2RBX016&CDD_SEARCH_STATE=0&DATABASE_SORT=0&DESCRIPTIONS=100&DYNAMIC_FORMAT=on&FIRST_QUERY_NUM=0&FORMAT_OBJECT=Alignment&FORMAT_PAGE_TARGET=&FORMAT_TYPE=HTML&GET_SEQUENCE=yes&I_THRESH=&LINE_LENGTH=60&MASK_CHAR=2&MASK_COLOR=1&NEW_VIEW=yes&NUM_OVERVIEW=100&PAGE=Proteins&QUERY_INDEX=0&QUERY_NUMBER=0&RESULTS_PAGE_TARGET=&RID=EMEE2DT2013&SHOW_LINKOUT=yes&SHOW_OVERVIEW=yes&STEP_NUMBER=&WORD_SIZE=6&OLD_VIEW=false&DISPLAY_SORT=4&HSP_SORT=0)

[E](https://blast.ncbi.nlm.nih.gov/Blast.cgi?CMD=Get&ALIGNMENTS=100&ALIGNMENT_VIEW=Pairwise&CDD_RID=EMEE2RBX016&CDD_SEARCH_STATE=0&DATABASE_SORT=0&DESCRIPTIONS=100&DYNAMIC_FORMAT=on&FIRST_QUERY_NUM=0&FORMAT_OBJECT=Alignment&FORMAT_PAGE_TARGET=&FORMAT_TYPE=HTML&GET_SEQUENCE=yes&I_THRESH=&LINE_LENGTH=60&MASK_CHAR=2&MASK_COLOR=1&NEW_VIEW=yes&NUM_OVERVIEW=100&PAGE=Proteins&QUERY_INDEX=0&QUERY_NUMBER=0&RESULTS_PAGE_TARGET=&RID=EMEE2DT2013&SHOW_LINKOUT=yes&SHOW_OVERVIEW=yes&STEP_NUMBER=&WORD_SIZE=6&OLD_VIEW=false&DISPLAY_SORT=0&HSP_SORT=0)

[Ident](https://blast.ncbi.nlm.nih.gov/Blast.cgi?CMD=Get&ALIGNMENTS=100&ALIGNMENT_VIEW=Pairwise&CDD_RID=EMEE2RBX016&CDD_SEARCH_STATE=0&DATABASE_SORT=0&DESCRIPTIONS=100&DYNAMIC_FORMAT=on&FIRST_QUERY_NUM=0&FORMAT_OBJECT=Alignment&FORMAT_PAGE_TARGET=&FORMAT_TYPE=HTML&GET_SEQUENCE=yes&I_THRESH=&LINE_LENGTH=60&MASK_CHAR=2&MASK_COLOR=1&NEW_VIEW=yes&NUM_OVERVIEW=100&PAGE=Proteins&QUERY_INDEX=0&QUERY_NUMBER=0&RESULTS_PAGE_TARGET=&RID=EMEE2DT2013&SHOW_LINKOUT=yes&SHOW_OVERVIEW=yes&STEP_NUMBER=&WORD_SIZE=6&DISPLAY_SORT=3&HSP_SORT=3)

Accession

[score](https://blast.ncbi.nlm.nih.gov/Blast.cgi?CMD=Get&ALIGNMENTS=100&ALIGNMENT_VIEW=Pairwise&CDD_RID=EMEE2RBX016&CDD_SEARCH_STATE=0&DATABASE_SORT=0&DESCRIPTIONS=100&DYNAMIC_FORMAT=on&FIRST_QUERY_NUM=0&FORMAT_OBJECT=Alignment&FORMAT_PAGE_TARGET=&FORMAT_TYPE=HTML&GET_SEQUENCE=yes&I_THRESH=&LINE_LENGTH=60&MASK_CHAR=2&MASK_COLOR=1&NEW_VIEW=yes&NUM_OVERVIEW=100&PAGE=Proteins&QUERY_INDEX=0&QUERY_NUMBER=0&RESULTS_PAGE_TARGET=&RID=EMEE2DT2013&SHOW_LINKOUT=yes&SHOW_OVERVIEW=yes&STEP_NUMBER=&WORD_SIZE=6&OLD_VIEW=false&DISPLAY_SORT=1&HSP_SORT=1)

[score](https://blast.ncbi.nlm.nih.gov/Blast.cgi?CMD=Get&ALIGNMENTS=100&ALIGNMENT_VIEW=Pairwise&CDD_RID=EMEE2RBX016&CDD_SEARCH_STATE=0&DATABASE_SORT=0&DESCRIPTIONS=100&DYNAMIC_FORMAT=on&FIRST_QUERY_NUM=0&FORMAT_OBJECT=Alignment&FORMAT_PAGE_TARGET=&FORMAT_TYPE=HTML&GET_SEQUENCE=yes&I_THRESH=&LINE_LENGTH=60&MASK_CHAR=2&MASK_COLOR=1&NEW_VIEW=yes&NUM_OVERVIEW=100&PAGE=Proteins&QUERY_INDEX=0&QUERY_NUMBER=0&RESULTS_PAGE_TARGET=&RID=EMEE2DT2013&SHOW_LINKOUT=yes&SHOW_OVERVIEW=yes&STEP_NUMBER=&WORD_SIZE=6&OLD_VIEW=false&DISPLAY_SORT=2&HSP_SORT=1)

[cover](https://blast.ncbi.nlm.nih.gov/Blast.cgi?CMD=Get&ALIGNMENTS=100&ALIGNMENT_VIEW=Pairwise&CDD_RID=EMEE2RBX016&CDD_SEARCH_STATE=0&DATABASE_SORT=0&DESCRIPTIONS=100&DYNAMIC_FORMAT=on&FIRST_QUERY_NUM=0&FORMAT_OBJECT=Alignment&FORMAT_PAGE_TARGET=&FORMAT_TYPE=HTML&GET_SEQUENCE=yes&I_THRESH=&LINE_LENGTH=60&MASK_CHAR=2&MASK_COLOR=1&NEW_VIEW=yes&NUM_OVERVIEW=100&PAGE=Proteins&QUERY_INDEX=0&QUERY_NUMBER=0&RESULTS_PAGE_TARGET=&RID=EMEE2DT2013&SHOW_LINKOUT=yes&SHOW_OVERVIEW=yes&STEP_NUMBER=&WORD_SIZE=6&OLD_VIEW=false&DISPLAY_SORT=4&HSP_SORT=0)

[value](https://blast.ncbi.nlm.nih.gov/Blast.cgi?CMD=Get&ALIGNMENTS=100&ALIGNMENT_VIEW=Pairwise&CDD_RID=EMEE2RBX016&CDD_SEARCH_STATE=0&DATABASE_SORT=0&DESCRIPTIONS=100&DYNAMIC_FORMAT=on&FIRST_QUERY_NUM=0&FORMAT_OBJECT=Alignment&FORMAT_PAGE_TARGET=&FORMAT_TYPE=HTML&GET_SEQUENCE=yes&I_THRESH=&LINE_LENGTH=60&MASK_CHAR=2&MASK_COLOR=1&NEW_VIEW=yes&NUM_OVERVIEW=100&PAGE=Proteins&QUERY_INDEX=0&QUERY_NUMBER=0&RESULTS_PAGE_TARGET=&RID=EMEE2DT2013&SHOW_LINKOUT=yes&SHOW_OVERVIEW=yes&STEP_NUMBER=&WORD_SIZE=6&OLD_VIEW=false&DISPLAY_SORT=0&HSP_SORT=0)

cellobiose dehydrogenase [Dichomitus squalens LYAD-421

SS1]

cellobiose dehydrogenase [Trametes sanguinea]

Iron reductase domain / Cellobiose dehydrogenase [Trametes

cinnabarina]

cellobiose dehydrogenase [Trametes cinnabarina]

cellobiose dehydrogenase [Trametes cinnabarina]

cellobiose dehydrogenase [Trametes versicolor]

cellobiose dehydrogenase [Trametes versicolor FP-101664

SS1]

Cellobiose dehydrogenase [Trametes pubescens]

cellobise dehydrogenase [Trametes velutina]

cellobiose dehydrogenase [Trametes versicolor]

cellobiose dehydrogenase [Grifola frondosa]

cellobiose dehydrogenase [Obba rivulosa]

cellobiose dehydrogenase [Cerrena unicolor]

cellobiose dehydrogenase [Phlebiopsis gigantea 11061_1

CR5-6]

hypothetical protein STEHIDRAFT_62168 [Stereum hirsutum

FP-91666 SS1]

1257

1233

1219

1216

1216

1189

1188

1187

1186

1174

1126

1103

1077

1071

1065

1257

1233

1219

1216

1216

1189

1188

1187

1186

1174

1126

1103

1077

1071

1065

99%

99%

99%

99%

99%

99%

99%

99%

99%

99%

99%

99%

99%

99%

99%

0.0

0.0

0.0

0.0

0.0

0.0

0.0

0.0

0.0

0.0

0.0

0.0

0.0

0.0

0.0

81%

77%

77%

77%

77%

75%

75%

75%

75%

74%

72%

71%

68%

69%

69%

[XP_007363678.1](https://www.ncbi.nlm.nih.gov/protein/597982095?report=genbank&log$=prottop&blast_rank=1&RID=EMEE2DT2013)

[AGS09130.1](https://www.ncbi.nlm.nih.gov/protein/527171868?report=genbank&log$=prottop&blast_rank=2&RID=EMEE2DT2013)

[CDO73368.1](https://www.ncbi.nlm.nih.gov/protein/691790977?report=genbank&log$=prottop&blast_rank=3&RID=EMEE2DT2013)

[ADX41688.1](https://www.ncbi.nlm.nih.gov/protein/323360357?report=genbank&log$=prottop&blast_rank=4&RID=EMEE2DT2013)

[AAC32197.1](https://www.ncbi.nlm.nih.gov/protein/3421395?report=genbank&log$=prottop&blast_rank=5&RID=EMEE2DT2013)

[AAC50004.1](https://www.ncbi.nlm.nih.gov/protein/2739491?report=genbank&log$=prottop&blast_rank=6&RID=EMEE2DT2013)

[XP_008041466.1](https://www.ncbi.nlm.nih.gov/protein/636620663?report=genbank&log$=prottop&blast_rank=7&RID=EMEE2DT2013)

[OJT10842.1](https://www.ncbi.nlm.nih.gov/protein/1112957336?report=genbank&log$=prottop&blast_rank=8&RID=EMEE2DT2013)

[ANA11091.1](https://www.ncbi.nlm.nih.gov/protein/1021313746?report=genbank&log$=prottop&blast_rank=9&RID=EMEE2DT2013)

[AAO32063.1](https://www.ncbi.nlm.nih.gov/protein/28207690?report=genbank&log$=prottop&blast_rank=10&RID=EMEE2DT2013)

[BAC20641.1](https://www.ncbi.nlm.nih.gov/protein/23616926?report=genbank&log$=prottop&blast_rank=11&RID=EMEE2DT2013)

[OCH89474.1](https://www.ncbi.nlm.nih.gov/protein/1045912341?report=genbank&log$=prottop&blast_rank=12&RID=EMEE2DT2013)

[AGS09131.1](https://www.ncbi.nlm.nih.gov/protein/527171871?report=genbank&log$=prottop&blast_rank=13&RID=EMEE2DT2013)

[KIP11283.1](https://www.ncbi.nlm.nih.gov/protein/754378677?report=genbank&log$=prottop&blast_rank=14&RID=EMEE2DT2013)

[XP_007306783.1](https://www.ncbi.nlm.nih.gov/protein/618808141?report=genbank&log$=prottop&blast_rank=15&RID=EMEE2DT2013)

RecName: Full=Cellobiose dehydrogenase; Short=CDH;

AltName: Full=Cellobiose-quinone oxidoreductase; Flags:

1062

1062

99%

0.0

68%

[Q01738.1](https://www.ncbi.nlm.nih.gov/protein/6831496?report=genbank&log$=prottop&blast_rank=16&RID=EMEE2DT2013)

Precursor

cellobiose dehydrogenase [Irpex lacteus]

cellobiose dehydrogenase [Irpex lacteus]

cellobiose dehydrogenase [Phanerochaete chrysosporium]

cellobiose dehydrogenase 1 [Heterobasidion irregulare TC 32-

1]

cellobiose dehydrogenase [Gelatoporia subvermispora]

cellobiose dehydrogenase [Gelatoporia subvermispora B]

cellobiose dehydrogenase [Phlebia lindtneri]

hypothetical protein PHACADRAFT_259608 [Phanerochaete

carnosa HHB-10118-sp]

Cellobiose dehydrogenase [Phlebia centrifuga]

cellobiose dehydrogenase [Serpula lacrymans var. lacrymans

S7.9]

putative cellobiose dehydrogenase [Serpula lacrymans var.

lacrymans S7.9]

hypothetical protein HYDPIDRAFT_31984 [Hydnomerulius

pinastri MD-312]

cellobiose dehydrogenase [Coniophora puteana]

cellobiose dehydrogenase [Athelia rolfsii]

hypothetical protein PENSPDRAFT_710401 [Peniophora sp.

CONT]

cellobiose dehydrogenase [Coniophora puteana RWD-64-598

SS2]

hypothetical protein PLICRDRAFT_322412 [Plicaturopsis

crispa FD-325 SS-3]

carbohydrate-binding module 1 protein [Jaapia argillacea

MUCL 33604]

hypothetical protein SERLA73DRAFT_77778 [Serpula

lacrymans var. lacrymans S7.3]

cellobiose dehydrogenase [Phanerochaete chrysosporium]

Cellobiose dehydrogenase [Hypsizygus marmoreus]

cellobiose dehydrogenase [Punctularia strigosozonata HHB-

11173 SS5]

cellobiose dehydrogenase [Moniliophthora roreri MCA 2997]

Cellobiose dehydrogenase [Leucoagaricus sp. SymC.cos]

hypothetical protein FIBSPDRAFT_1039750 [Fibulorhizoctonia

sp. CBS 109695]

1060

1060

1057

1053

1050

1050

1041

1034

1031

1026

1002

1001

995

993

991

990

986

982

979

975

974

972

968

965

957

1060

1060

1057

1053

1050

1050

1041

1034

1031

1026

1002

1001

995

993

991

990

986

982

979

975

974

972

968

965

957

99%

99%

99%

99%

99%

99%

99%

99%

94%

99%

99%

99%

99%

99%

99%

99%

99%

97%

99%

99%

99%

99%

99%

99%

99%

0.0

0.0

0.0

0.0

0.0

0.0

0.0

0.0

0.0

0.0

0.0

0.0

0.0

0.0

0.0

0.0

0.0

0.0

0.0

0.0

0.0

0.0

0.0

0.0

0.0

67%

67%

68%

68%

70%

70%

66%

65%

70%

67%

64%

66%

66%

64%

64%

65%

64%

64%

63%

64%

64%

63%

62%

63%

64%

[BAD36748.1](https://www.ncbi.nlm.nih.gov/protein/51172592?report=genbank&log$=prottop&blast_rank=17&RID=EMEE2DT2013)

[ALJ82902.1](https://www.ncbi.nlm.nih.gov/protein/939535451?report=genbank&log$=prottop&blast_rank=18&RID=EMEE2DT2013)

[CAA61359.1](https://www.ncbi.nlm.nih.gov/protein/1279638?report=genbank&log$=prottop&blast_rank=19&RID=EMEE2DT2013)

[XP_009552848.1](https://www.ncbi.nlm.nih.gov/protein/695578166?report=genbank&log$=prottop&blast_rank=20&RID=EMEE2DT2013)

[ACF60617.1](https://www.ncbi.nlm.nih.gov/protein/194399173?report=genbank&log$=prottop&blast_rank=21&RID=EMEE2DT2013)

[EMD36613.1](https://www.ncbi.nlm.nih.gov/protein/449545642?report=genbank&log$=prottop&blast_rank=22&RID=EMEE2DT2013)

[AGE45679.1](https://www.ncbi.nlm.nih.gov/protein/448871780?report=genbank&log$=prottop&blast_rank=23&RID=EMEE2DT2013)

[XP_007398018.1](https://www.ncbi.nlm.nih.gov/protein/599385661?report=genbank&log$=prottop&blast_rank=24&RID=EMEE2DT2013)

[OKY59527.1](https://www.ncbi.nlm.nih.gov/protein/1124107155?report=genbank&log$=prottop&blast_rank=25&RID=EMEE2DT2013)

[XP_007323287.1](https://www.ncbi.nlm.nih.gov/protein/597937886?report=genbank&log$=prottop&blast_rank=26&RID=EMEE2DT2013)

[XP_007323530.1](https://www.ncbi.nlm.nih.gov/protein/597938372?report=genbank&log$=prottop&blast_rank=27&RID=EMEE2DT2013)

[KIJ60769.1](https://www.ncbi.nlm.nih.gov/protein/749892935?report=genbank&log$=prottop&blast_rank=28&RID=EMEE2DT2013)

[BAD32781.1](https://www.ncbi.nlm.nih.gov/protein/50657251?report=genbank&log$=prottop&blast_rank=29&RID=EMEE2DT2013)

[AAO64483.1](https://www.ncbi.nlm.nih.gov/protein/32395290?report=genbank&log$=prottop&blast_rank=30&RID=EMEE2DT2013)

[KZV74216.1](https://www.ncbi.nlm.nih.gov/protein/1024081314?report=genbank&log$=prottop&blast_rank=31&RID=EMEE2DT2013)

[XP_007774356.1](https://www.ncbi.nlm.nih.gov/protein/628854489?report=genbank&log$=prottop&blast_rank=32&RID=EMEE2DT2013)

[KII85600.1](https://www.ncbi.nlm.nih.gov/protein/749760671?report=genbank&log$=prottop&blast_rank=33&RID=EMEE2DT2013)

[KDQ52894.1](https://www.ncbi.nlm.nih.gov/protein/646388008?report=genbank&log$=prottop&blast_rank=34&RID=EMEE2DT2013)

[EGN94368.1](https://www.ncbi.nlm.nih.gov/protein/336366020?report=genbank&log$=prottop&blast_rank=35&RID=EMEE2DT2013)

[2118247A](https://www.ncbi.nlm.nih.gov/protein/1582220?report=genbank&log$=prottop&blast_rank=36&RID=EMEE2DT2013)

[KYQ35905.1](https://www.ncbi.nlm.nih.gov/protein/1012961135?report=genbank&log$=prottop&blast_rank=37&RID=EMEE2DT2013)

[XP_007387326.1](https://www.ncbi.nlm.nih.gov/protein/599119895?report=genbank&log$=prottop&blast_rank=38&RID=EMEE2DT2013)

[XP_007851234.1](https://www.ncbi.nlm.nih.gov/protein/630199361?report=genbank&log$=prottop&blast_rank=39&RID=EMEE2DT2013)

[KXN88673.1](https://www.ncbi.nlm.nih.gov/protein/1000862802?report=genbank&log$=prottop&blast_rank=40&RID=EMEE2DT2013)

[KZP28042.1](https://www.ncbi.nlm.nih.gov/protein/1021839821?report=genbank&log$=prottop&blast_rank=41&RID=EMEE2DT2013)

https://blast.ncbi.nlm.nih.gov/Blast.cgi

2/7


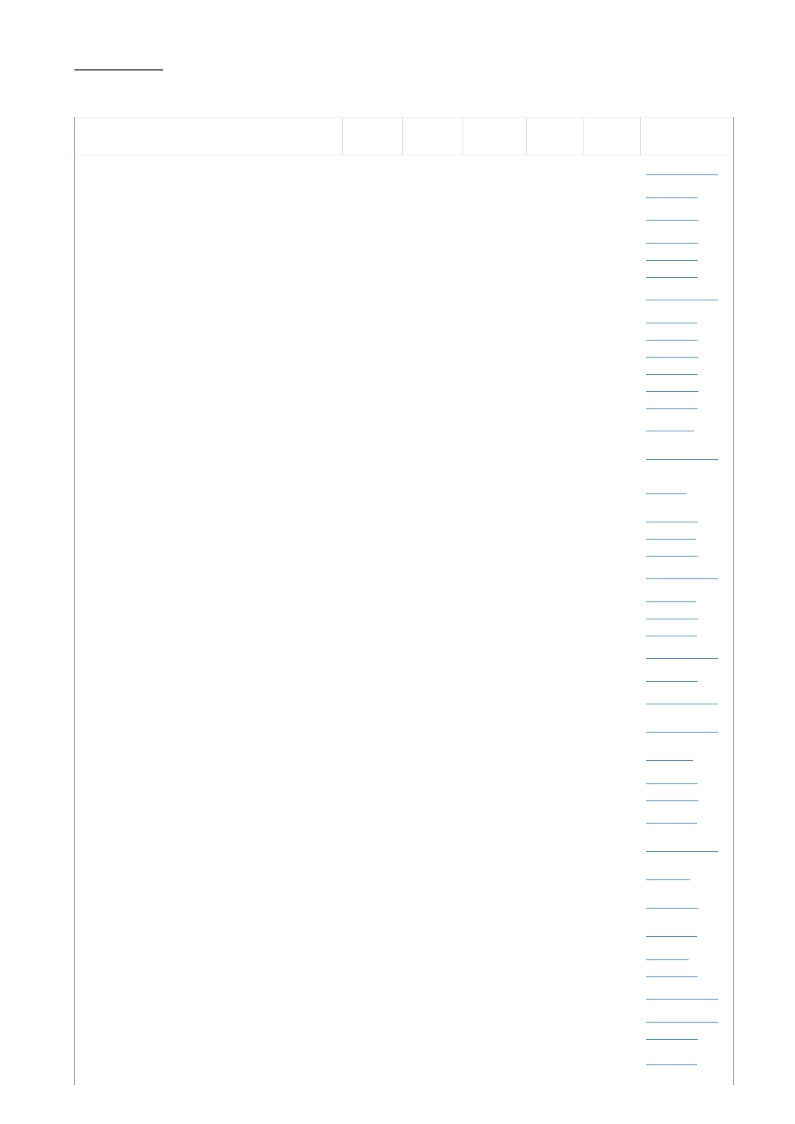


2017/4/9

NCBI Blast:GL22256-R1_1

hypothetical protein FIBSPDRAFT_1051990 [Fibulorhizoctonia

955

955

99%

0.0

64%

[KZP09193.1](https://www.ncbi.nlm.nih.gov/protein/1021820429?report=genbank&log$=prottop&blast_rank=42&RID=EMEE2DT2013)

sp. CBS 109695]

Cellobiose dehydrogenase [Leucoagaricus sp. SymC.cos]

hypothetical protein WG66_16822 [Moniliophthora roreri]

DUF221-domain-containing protein [Lentinula edodes]

hypothetical protein FIBSPDRAFT_818085 [Fibulorhizoctonia

sp. CBS 109695]

Cellobiose dehydrogenase [Hypsizygus marmoreus]

cellobiose dehydrogenase [Moniliophthora roreri MCA 2997]

hypothetical protein PLEOSDRAFT_41743 [Pleurotus ostreatus

PC15]

DUF221-domain-containing protein [Lentinula edodes]

hypothetical protein GYMLUDRAFT_241124 [Gymnopus

luxurians FD-317 M1]

hypothetical protein M422DRAFT_186233 [Sphaerobolus

stellatus SS14]

cellobiose dehydrogenase [Auricularia subglabra TFB-10046

SS5]

hypothetical protein CYLTODRAFT_420700 [Cylindrobasidium

torrendii FP15055 ss-10]

cellobiose dehydrogenase [Schizophyllum commune H4-8]

Cellobiose dehydrogenase [Termitomyces sp. J132]

hypothetical protein CYLTODRAFT_185011 [Cylindrobasidium

torrendii FP15055 ss-10]

cellobiose dehydrogenase [Coprinopsis cinerea

okayama7#130]

Cellobiose dehydrogenase [Leucoagaricus sp. SymC.cos]

cellobiose dehydrogenase [Exidia glandulosa HHB12029]

cellobiose dehydrogenase [Mycena chlorophos]

hypothetical protein HYPSUDRAFT_70019 [Hypholoma

sublateritium FD-334 SS-4]

cellobiose dehydrogenase [Sanghuangporus baumii]

Chain A, Crystal Structure Of The Flavin Domain Of Cellobiose

Dehydrogenase

cellobiose dehydrogenase [Fomitiporia mediterranea

MF3/22]

cellobiose dehydrogenase [Schizopora paradoxa]

Chain A, Cellobiose Dehydrogenase Flavoprotein Fragment In

Complex With Cellobionolactam

hypothetical protein GALMADRAFT_894992 [Galerina

marginata CBS 339.88]

cellobiose dehydrogenase [Sistotremastrum niveocremeum

HHB9708]

cellobiose dehydrogenase [Sistotremastrum niveocremeum

HHB9708]

hypothetical protein AGABI1DRAFT_107890 [Agaricus bisporus

var. burnettii JB137-S8]

cellobiose dehydrogenase [Sistotremastrum suecicum

HHB10207 ss-3]

Cellobiose dehydrogenase [Termitomyces sp. J132]

hypothetical protein AGABI2DRAFT_188178 [Agaricus bisporus

var. bisporus H97]

the flavin domain of cellobiose dehydrogenase [Coniophora

puteana RWD-64-598 SS2]

cellobiose dehydrogenase protein [Tulasnella calospora MUT

4182]

Cellobiose dehydrogenase OS=Phanerochaete chrysosporium

GN=CDH-1 PE=1 SV=1 [Rhizoctonia solani AG-1 IB]

cellobiose dehydrogenase [Rhizoctonia solani AG-3

Rhs1AP]

Cellobiose dehydrogenase [Rhizoctonia solani]

Cellobiose dehydrogenase Short=CDH [Rhizoctonia solani AG-

1 IB]

cellobiose dehydrogenase [Rhizoctonia solani 123E]

probable cellobiose dehydrogenase [Serendipita indica DSM

11827]

Cellobiose dehydrogenase [Hypsizygus marmoreus]

probable cellobiose dehydrogenase [Serendipita indica DSM

11827]

cellobiose dehydrogenase [Piloderma croceum F 1598]

952

946

939

932

931

927

920

912

905

897

895

885

882

877

873

865

863

855

853

853

846

846

844

840

838

830

811

798

798

798

796

793

781

766

757

749

747

744

726

713

711

701

686

952

946

939

932

931

927

920

912

905

897

895

885

882

877

873

865

863

855

853

853

846

846

844

840

838

830

811

798

798

798

796

793

781

766

757

749

747

744

726

713

711

701

686

96%

99%

99%

99%

99%

99%

97%

99%

99%

99%

99%

99%

99%

92%

99%

99%

99%

99%

99%

99%

99%

74%

99%

99%

74%

95%

99%

99%

99%

99%

80%

99%

74%

98%

99%

99%

99%

99%

99%

99%

74%

97%

74%

0.0

0.0

0.0

0.0

0.0

0.0

0.0

0.0

0.0

0.0

0.0

0.0

0.0

0.0

0.0

0.0

0.0

0.0

0.0

0.0

0.0

0.0

0.0

0.0

0.0

0.0

0.0

0.0

0.0

0.0

0.0

0.0

0.0

0.0

0.0

0.0

0.0

0.0

0.0

0.0

0.0

0.0

0.0

64%

62%

61%

64%

61%

62%

60%

60%

58%

59%

59%

57%

59%

62%

57%

57%

57%

57%

56%

59%

56%

71%

56%

56%

71%

59%

54%

54%

53%

54%

64%

53%

65%

53%

51%

50%

51%

50%

49%

50%

61%

48%

58%

[KXN88672.1](https://www.ncbi.nlm.nih.gov/protein/1000862801?report=genbank&log$=prottop&blast_rank=43&RID=EMEE2DT2013)

[KTB30601.1](https://www.ncbi.nlm.nih.gov/protein/961953749?report=genbank&log$=prottop&blast_rank=44&RID=EMEE2DT2013)

[GAW07826.1](https://www.ncbi.nlm.nih.gov/protein/1139908692?report=genbank&log$=prottop&blast_rank=45&RID=EMEE2DT2013)

[KZP27683.1](https://www.ncbi.nlm.nih.gov/protein/1021839447?report=genbank&log$=prottop&blast_rank=46&RID=EMEE2DT2013)

[KYQ35834.1](https://www.ncbi.nlm.nih.gov/protein/1012961064?report=genbank&log$=prottop&blast_rank=47&RID=EMEE2DT2013)

[XP_007853117.1](https://www.ncbi.nlm.nih.gov/protein/630205114?report=genbank&log$=prottop&blast_rank=48&RID=EMEE2DT2013)

[KDQ23351.1](https://www.ncbi.nlm.nih.gov/protein/646302201?report=genbank&log$=prottop&blast_rank=49&RID=EMEE2DT2013)

[GAW07825.1](https://www.ncbi.nlm.nih.gov/protein/1139908691?report=genbank&log$=prottop&blast_rank=50&RID=EMEE2DT2013)

[KIK63890.1](https://www.ncbi.nlm.nih.gov/protein/751022186?report=genbank&log$=prottop&blast_rank=51&RID=EMEE2DT2013)

[KIJ31253.1](https://www.ncbi.nlm.nih.gov/protein/749860458?report=genbank&log$=prottop&blast_rank=52&RID=EMEE2DT2013)

[XP_007342794.1](https://www.ncbi.nlm.nih.gov/protein/598026843?report=genbank&log$=prottop&blast_rank=53&RID=EMEE2DT2013)

[KIY69410.1](https://www.ncbi.nlm.nih.gov/protein/761949726?report=genbank&log$=prottop&blast_rank=54&RID=EMEE2DT2013)

[XP_003026061.1](https://www.ncbi.nlm.nih.gov/protein/302672747?report=genbank&log$=prottop&blast_rank=55&RID=EMEE2DT2013)

[KNZ81006.1](https://www.ncbi.nlm.nih.gov/protein/914266285?report=genbank&log$=prottop&blast_rank=56&RID=EMEE2DT2013)

[KIY62194.1](https://www.ncbi.nlm.nih.gov/protein/761942182?report=genbank&log$=prottop&blast_rank=57&RID=EMEE2DT2013)

[XP_001835032.2](https://www.ncbi.nlm.nih.gov/protein/299739078?report=genbank&log$=prottop&blast_rank=58&RID=EMEE2DT2013)

[KXN88674.1](https://www.ncbi.nlm.nih.gov/protein/1000862803?report=genbank&log$=prottop&blast_rank=59&RID=EMEE2DT2013)

[KZV90569.1](https://www.ncbi.nlm.nih.gov/protein/1024099249?report=genbank&log$=prottop&blast_rank=60&RID=EMEE2DT2013)

[GAT58867.1](https://www.ncbi.nlm.nih.gov/protein/1018858508?report=genbank&log$=prottop&blast_rank=61&RID=EMEE2DT2013)

[KJA18237.1](https://www.ncbi.nlm.nih.gov/protein/763720763?report=genbank&log$=prottop&blast_rank=62&RID=EMEE2DT2013)

[OCB87941.1](https://www.ncbi.nlm.nih.gov/protein/1044613381?report=genbank&log$=prottop&blast_rank=63&RID=EMEE2DT2013)

[1KDG_A](https://www.ncbi.nlm.nih.gov/protein/27065180?report=genbank&log$=prottop&blast_rank=64&RID=EMEE2DT2013)

[XP_007264488.1](https://www.ncbi.nlm.nih.gov/protein/595771890?report=genbank&log$=prottop&blast_rank=65&RID=EMEE2DT2013)

[KLO11658.1](https://www.ncbi.nlm.nih.gov/protein/827756966?report=genbank&log$=prottop&blast_rank=66&RID=EMEE2DT2013)

[1NAA_A](https://www.ncbi.nlm.nih.gov/protein/28373978?report=genbank&log$=prottop&blast_rank=67&RID=EMEE2DT2013)

[KDR70281.1](https://www.ncbi.nlm.nih.gov/protein/648156501?report=genbank&log$=prottop&blast_rank=68&RID=EMEE2DT2013)

[KZS87660.1](https://www.ncbi.nlm.nih.gov/protein/1023190059?report=genbank&log$=prottop&blast_rank=69&RID=EMEE2DT2013)

[KZS87659.1](https://www.ncbi.nlm.nih.gov/protein/1023190058?report=genbank&log$=prottop&blast_rank=70&RID=EMEE2DT2013)

[XP_007331551.1](https://www.ncbi.nlm.nih.gov/protein/597988269?report=genbank&log$=prottop&blast_rank=71&RID=EMEE2DT2013)

[KZT33629.1](https://www.ncbi.nlm.nih.gov/protein/1023237652?report=genbank&log$=prottop&blast_rank=72&RID=EMEE2DT2013)

[KNZ79525.1](https://www.ncbi.nlm.nih.gov/protein/914264672?report=genbank&log$=prottop&blast_rank=73&RID=EMEE2DT2013)

[XP_006456127.1](https://www.ncbi.nlm.nih.gov/protein/568440976?report=genbank&log$=prottop&blast_rank=74&RID=EMEE2DT2013)

[XP_007763021.1](https://www.ncbi.nlm.nih.gov/protein/628825435?report=genbank&log$=prottop&blast_rank=75&RID=EMEE2DT2013)

[KIO27367.1](https://www.ncbi.nlm.nih.gov/protein/752369590?report=genbank&log$=prottop&blast_rank=76&RID=EMEE2DT2013)

[CEL59822.1](https://www.ncbi.nlm.nih.gov/protein/751839628?report=genbank&log$=prottop&blast_rank=77&RID=EMEE2DT2013)

[EUC66513.1](https://www.ncbi.nlm.nih.gov/protein/576993847?report=genbank&log$=prottop&blast_rank=78&RID=EMEE2DT2013)

[CUA69410.1](https://www.ncbi.nlm.nih.gov/protein/924123825?report=genbank&log$=prottop&blast_rank=79&RID=EMEE2DT2013)

[CCO28160.1](https://www.ncbi.nlm.nih.gov/protein/471908990?report=genbank&log$=prottop&blast_rank=80&RID=EMEE2DT2013)

[KEP50503.1](https://www.ncbi.nlm.nih.gov/protein/660965866?report=genbank&log$=prottop&blast_rank=81&RID=EMEE2DT2013)

[CCA69797.1](https://www.ncbi.nlm.nih.gov/protein/353237834?report=genbank&log$=prottop&blast_rank=82&RID=EMEE2DT2013)

[KYQ35912.1](https://www.ncbi.nlm.nih.gov/protein/1012961142?report=genbank&log$=prottop&blast_rank=83&RID=EMEE2DT2013)

[CCA69794.1](https://www.ncbi.nlm.nih.gov/protein/353237831?report=genbank&log$=prottop&blast_rank=84&RID=EMEE2DT2013)

[KIM76893.1](https://www.ncbi.nlm.nih.gov/protein/751728561?report=genbank&log$=prottop&blast_rank=85&RID=EMEE2DT2013)

https://blast.ncbi.nlm.nih.gov/Blast.cgi

3/7


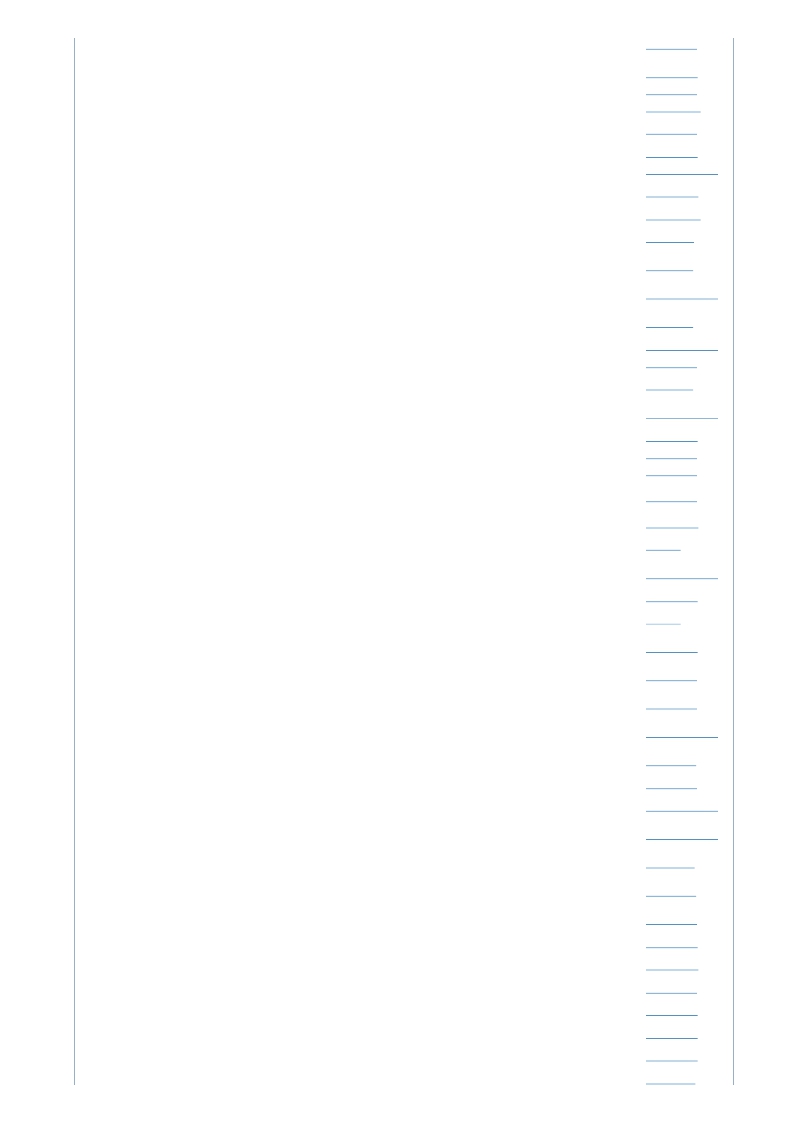


2017/4/9

NCBI Blast:GL22256-R1_1

hypothetical protein GLOTRDRAFT_113732 [Gloeophyllum

trabeum ATCC 11539]

cellobiose dehydrogenase protein [Serendipita vermifera MAFF

305830]

cellobiose dehydrogenase protein [Tulasnella calospora MUT

4182]

hypothetical protein RSAG8_00915 [Rhizoctonia solani AG-8

WAC10335]

cellobiose dehydrogenase protein [Serendipita vermifera MAFF

305830]

carbohydrate-binding module family 1 protein [Botryobasidium

botryosum FD-172 SS1]

cellobiose dehydrogenase [Rhizoctonia solani 123E]

cellobiose dehydrogenase [Rhizoctonia solani AG-3

Rhs1AP]

Cellobiose dehydrogenase [Rhizoctonia solani]

FAD/NAD(P)-binding domain-containing protein [Schizopora

paradoxa]

Cellobiose dehydrogenase OS=Phanerochaete chrysosporium

GN=CDH-1 PE=1 SV=1 [Rhizoctonia solani AG-1 IB]

hypothetical protein BOTBODRAFT_467653 [Botryobasidium

botryosum FD-172 SS1]

cellobiose dehydrogenase [Rhizoctonia solani AG-1 IA]

Cellobiose dehydrogenase Short=CDH [Rhizoctonia solani AG-

1 IB]

Cellobiose dehydrogenase OS=Phanerochaete chrysosporium

GN=CDH-1 PE=1 SV=1 [Rhizoctonia solani AG-1 IB]

682

669

667

667

652

619

596

571

565

563

560

558

530

521

509

682

669

667

667

652

619

596

571

565

563

560

558

530

521

509

74%

99%

96%

90%

99%

76%

74%

75%

75%

73%

75%

72%

74%

75%

73%

0.0

0.0

0.0

0.0

0.0

0.0

0.0

0.0

0.0

0.0

0.0

0.0

3e-175

3e-174

2e-169

59%

46%

49%

50%

46%

53%

51%

49%

48%

51%

48%

50%

48%

46%

47%

[XP_007861981.1](https://www.ncbi.nlm.nih.gov/protein/630345361?report=genbank&log$=prottop&blast_rank=86&RID=EMEE2DT2013)

[KIM33893.1](https://www.ncbi.nlm.nih.gov/protein/751683741?report=genbank&log$=prottop&blast_rank=87&RID=EMEE2DT2013)

[KIO27334.1](https://www.ncbi.nlm.nih.gov/protein/752369557?report=genbank&log$=prottop&blast_rank=88&RID=EMEE2DT2013)

[KDN51286.1](https://www.ncbi.nlm.nih.gov/protein/639573911?report=genbank&log$=prottop&blast_rank=89&RID=EMEE2DT2013)

[KIM33732.1](https://www.ncbi.nlm.nih.gov/protein/751683580?report=genbank&log$=prottop&blast_rank=90&RID=EMEE2DT2013)

[KDQ06595.1](https://www.ncbi.nlm.nih.gov/protein/646284643?report=genbank&log$=prottop&blast_rank=91&RID=EMEE2DT2013)

[KEP50510.1](https://www.ncbi.nlm.nih.gov/protein/660965873?report=genbank&log$=prottop&blast_rank=92&RID=EMEE2DT2013)

[EUC66520.1](https://www.ncbi.nlm.nih.gov/protein/576993854?report=genbank&log$=prottop&blast_rank=93&RID=EMEE2DT2013)

[CUA69402.1](https://www.ncbi.nlm.nih.gov/protein/924123817?report=genbank&log$=prottop&blast_rank=94&RID=EMEE2DT2013)

[KLO12878.1](https://www.ncbi.nlm.nih.gov/protein/827758212?report=genbank&log$=prottop&blast_rank=95&RID=EMEE2DT2013)

[CEL59814.1](https://www.ncbi.nlm.nih.gov/protein/751839620?report=genbank&log$=prottop&blast_rank=96&RID=EMEE2DT2013)

[KDQ11151.1](https://www.ncbi.nlm.nih.gov/protein/646289929?report=genbank&log$=prottop&blast_rank=97&RID=EMEE2DT2013)

[ELU38090.1](https://www.ncbi.nlm.nih.gov/protein/443917354?report=genbank&log$=prottop&blast_rank=98&RID=EMEE2DT2013)

[CCO28171.1](https://www.ncbi.nlm.nih.gov/protein/471908964?report=genbank&log$=prottop&blast_rank=99&RID=EMEE2DT2013)

[CEL52061.1](https://www.ncbi.nlm.nih.gov/protein/751842363?report=genbank&log$=prottop&blast_rank=100&RID=EMEE2DT2013)

Alignments

cellobiose dehydrogenase [Dichomitus squalens LYAD-421 SS1]

Sequence ID: XP_007363678.1 Length: 769 Number of Matches: 1

See 1 more title(s)

Range 1: 21 to 769

Score

Expect Method

Identities

Positives

Gaps

Frame

1257 bits(3252) 0.0()

Features:

Compositional matrix adjust. 641/793(81%) 689/793(86%) 50/793(6%)

Query 1

Sbjct 21

Query 61

Sbjct 81

VAAPYTDSGNGFVFDGITDAVYGVQYGIVLPQANSSTEFIGEIVAPIAAKWIGWAFGGAM 60

VAAPYTDSGNGFVFDGITD YGVYGIVLPQAN+STEFIGEIVAPIAAKW+GAFGGAM

VAAPYTDSGNGFVFDGITDPTYGVTYGIVLPQANTSTEFIGEIVAPIAAKWVGVAFGGAM 80

IGDLLLVAWPNGNDIVASTRYAT------EYDGPTLTTLPSSSVNSTHWKYVFRCQNCTS 114

IGDLLLVAWPNGNDIVASTR+AT EYDGPTLTTLPSSVNSTHWKYV+RCQNCTS

IGDLLLVAWPNGNDIVASTRWATDYIQPTEYDGPTLTTLPSSLVNSTHWKYVYRCQNCTS 140

Query 115 WEGGGSISPSGTGVFAWAYSNVGVDDPSDPESTFQEHTDFGFYGINFPDAQNANYQNYLQ 174

W+GGGIP+GTGVFAWAYS+VGVDDPSDPESTFQEHTDFGF+GINFPDAQN+NYQNYLQ

Sbjct 141 WQGGGGIDPTGTGVFAWAYSSVGVDDPSDPESTFQEHTDFGFFGINFPDAQNSNYQNYLQ 200

Query 175 GNPGTPPSSTTTTSTSTSTSTPTPTVTATPYDYIVVGAGPGGIITADRLSEAGKKVLLLE 234

GNPGTPPSSTTTT+TSTST+T PT+ATPYDYI+VGAGPGGIIADRLSEAGKKVLLLE

Sbjct 201 GNPGTPPSSTTTTTTSTSTTTTGPTASATPYDYIIVGAGPGGIIAADRLSEAGKKVLLLE 260

Query 235 RGGPSTAETGGTYDAPWAQSANLTKFDVPGLFESMFTDSNSWWWCKAMHIDINFFAGCLL 294

RGGPSTAETGGTYDAPWQSANLTKFDVPGLFES+FTDNWWWCK DIFFAGCLL

Sbjct 261 RGGPSTAETGGTYDAPWTQSANLTKFDVPGLFESLFTDPNDWWWCK----DITFFAGCLL 316

Query 295 GGGTSINGALYWYPPDSDFQGANGWPNSWGNHAPYTSMLKQRLPSTDHPSTDGKRYLEES 354

GGGTS+NGALYWYPDSDF NGWPSWNHPYTSL+QRLPSTDHPSTDGKRYLEES

Sbjct 317 GGGTSVNGALYWYPADSDFSTENGWPQSWANHQPYTSKLQQRLPSTDHPSTDGKRYLEES 376

Query 355 AAIVAQLLNGQGYSNITINDNPDYKDHVYGYSAFDFIGGERGGPVATYFQTASARPNFTY 414

A+VQLL+QGYSNITINDNP+KDHVYGYSAFDFIGERGPVATYFQTAARPNFTY

Sbjct 377 ANVVVQLLSKQGYSNITINDNPNSKDHVYGYSAFDFINGERAGPVATYFQTAKARPNFTY 436

Query 415 KQYVLVSQVIRNGSTITGVRTNDTSLGPDGIIPLNPNGRVVLSAGSFGTPRILFQSGIGP 474

KYVLVSQV+RNG+TITGVRTNDTSLGP+GI+PLNPNGRV+LSAGSFGTPRILFQSGIGP

Sbjct 437 KDYVLVSQVVRNGATITGVRTNDTSLGPNGIVPLNPNGRVILSAGSFGTPRILFQSGIGP 496

Query 475 EDMLQTVQSNPTASANLPAQSQWIDLPVGQGVSDNPSINLVFTHPSIDAYDNWADVWSDP 534

DM+QVQ+NPTAANLPAQSWI+LPVGQGVSDNPSINLVFTHPSIDAY+NWADVWSDP

Sbjct 497 TDMIQAVQANPTAGANLPAQSDWINLPVGQGVSDNPSINLVFTHPSIDAYENWADVWSDP 556

Query 535 RPADAQQYLKDRSGVFAGASPKLNFWRAYGASDGVTRYAQGTVRPGAASVNSSLPYNASN 594

RPADAQQYL RSGVFAGASPKLNFWRAYG+DGTRYAQGTVRPGAASVN+SLPYNAS

Sbjct 557 RPADAQQYLTSRSGVFAGASPKLNFWRAYGGNDGFTRYAQGTVRPGAASVNTSLPYNASQ 616

Query 595 IFTITVYLSQGIASRGRIGIDAALNAKALSNPWLTDPTDKTVLLQALHDVVSNMDSAQQA 654

IFTITVYLSQGISRGR+GI+LNA+A++NPWLTD DKT+LLQALHDV N+S

Sbjct 617 IFTITVYLSQGIQSRGRVGITSGLNAQAITNPWLTDTEDKTILLQALHDVADNIKS---- 672

Query 655 PSPSEPSECTLIDSHTVSNLTLITPDATMTLEEYVDAYDPATMCSNHWVGSAKIGTSSST 714

+NLTLITPDTMTLE+YVDAYDP+TMCSNHWVGSAKIGTS+S

Sbjct 673 ----------------IPNLTLITPDPTMTLEQYVDAYDPSTMCSNHWVGSAKIGTSASN 716

Query 715 AVIDENAKVFNTDNLVSIQRAVLTLSFPSLIRVSQFIVDASIIPSLPMGNPHGMLMSAAE 774

AV+D+NAKVFNT+NL F+VDASIIP+LP+GNPHGLMSAAE

Sbjct 717 AVVDQNAKVFNTNNL--------------------FVVDASIIPALPIGNPHGQLMSAAE 756

Query 775 QAVTKILALSGGP 787

QA KILAL+GGP

Sbjct 757 QAAAKILALAGGP 769

https://blast.ncbi.nlm.nih.gov/Blast.cgi

4/7


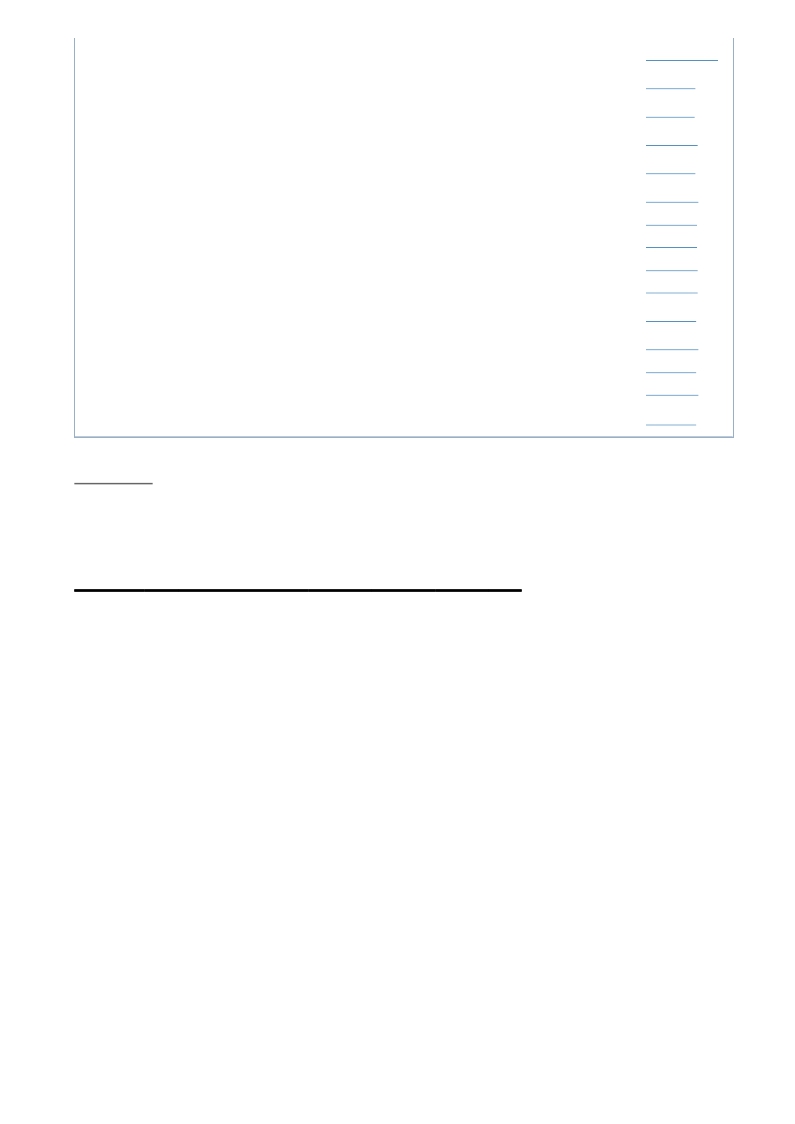


2017/4/9

cellobiose dehydrogenase [Trametes sanguinea]

Sequence ID: AGS09130.1 Length: 769 Number of Matches: 1

See 1 more title(s)

Range 1: 21 to 769

NCBI Blast:GL22256-R1_1

Score

Expect Method

Identities

Positives

Gaps

Frame

1233 bits(3191) 0.0()

Features:

Compositional matrix adjust. 614/793(77%) 677/793(85%) 50/793(6%)

Query 1

Sbjct 21

Query 61

Sbjct 81

VAAPYTDSGNGFVFDGITDAVYGVQYGIVLPQANSSTEFIGEIVAPIAAKWIGWAFGGAM 60

VAAPYDSGNGFVFDGITDVYVYGIVLPQA+S+EFIGEIVAP+AKWIGAGGAM

VAAPYVDSGNGFVFDGITDPVYHVSYGIVLPQATTSSEFIGEIVAPLDAKWIGLALGGAM 80

IGDLLLVAWPNGNDIVASTRYATEY------DGPTLTTLPSSSVNSTHWKYVFRCQNCTS 114

IGDLL+VAWPNGN+IV+STRYATY +GPT+TTLPSSVNSTHWK+VFRCQNCTS

IGDLLIVAWPNGNEIVSSTRYATAYQLPDVYEGPTITTLPSSLVNSTHWKFVFRCQNCTS 140

Query 115 WEGGGSISPSGTGVFAWAYSNVGVDDPSDPESTFQEHTDFGFYGINFPDAQNANYQNYLQ 174

WEGGGIP+GTGVFAWAYS+VGVDDPSDP+TFQEHTDFGF+GINFPDAQN+NYQNYLQ

Sbjct 141 WEGGGGIDPTGTGVFAWAYSSVGVDDPSDPNTTFQEHTDFGFFGINFPDAQNSNYQNYLQ 200

Query 175 GNPGTPPSSTTTTSTSTSTSTPTPTVTATPYDYIVVGAGPGGIITADRLSEAGKKVLLLE 234

G+GTPP++TT +T+T+ PTV+ATPYDYI+VGAGPGGIIADRLSEAGKKVLLLE

Sbjct 201 GDAGTPPPTSTPTGPTTTTTPTGPTVSATPYDYIIVGAGPGGIIAADRLSEAGKKVLLLE 260

Query 235 RGGPSTAETGGTYDAPWAQSANLTKFDVPGLFESMFTDSNSWWWCKAMHIDINFFAGCLL 294

RGGPSTA+TGGTYDAPWA+SANLTKFD+PGLFESMFTDNWWWCK DNFFAGCLL

Sbjct 261 RGGPSTAQTGGTYDAPWAKSANLTKFDIPGLFESMFTDPNPWWWCK----DTNFFAGCLL 316

Query 295 GGGTSINGALYWYPPDSDFQGANGWPNSWGNHAPYTSMLKQRLPSTDHPSTDGKRYLEES 354

GGGTS+NGALYWPDSDF NGWPSWGNHPYT+LKQRLPSTDHPSTDG+RYLE+S

Sbjct 317 GGGTSVNGALYWLPTDSDFSSQNGWPTSWGNHQPYTTKLKQRLPSTDHPSTDGQRYLEQS 376

Query 355 AAIVAQLLNGQGYSNITINDNPDYKDHVYGYSAFDFIGGERGGPVATYFQTASARPNFTY 414

A+V+QLL+GQGY ITINDNPDYKDHVYGYSAFDFIG+RGPVATYQTAARNFY

Sbjct 377 ATVVSQLLSGQGYQQITINDNPDYKDHVYGYSAFDFINGQRAGPVATYLQTALARSNFVY 436

Query 415 KQYVLVSQVIRNGSTITGVRTNDTSLGPDGIIPLNPNGRVVLSAGSFGTPRILFQSGIGP 474

KYLVSQV+RNGSTITGVRTN+T+LGP+GI+PLNPNGRV+L+AGSFGTPRILFQSGIGP

Sbjct 437 KDYTLVSQVLRNGSTITGVRTNNTALGPNGIVPLNPNGRVILAAGSFGTPRILFQSGIGP 496

Query 475 EDMLQTVQSNPTASANLPAQSQWIDLPVGQGVSDNPSINLVFTHPSIDAYDNWADVWSDP 534

D+QTV+SNPTA+ANLPQSWI+LPVGQVSDNPSINLVFTHPSIDAY+NWADVWSDP

Sbjct 497 TDQIQTVESNPTAAANLPPQSDWINLPVGQAVSDNPSINLVFTHPSIDAYENWADVWSDP 556

Query 535 RPADAQQYLKDRSGVFAGASPKLNFWRAYGASDGVTRYAQGTVRPGAASVNSSLPYNASN 594

RPADAQQYL+RSGVFAGASPKLNFWRAYGSDGTRYAQGTVRPGAASVN+S+YNAS

Sbjct 557 RPADAQQYLQSRSGVFAGASPKLNFWRAYGGSDGKTRYAQGTVRPGAASVNTSVAYNASQ 616

Query 595 IFTITVYLSQGIASRGRIGIDAALNAKALSNPWLTDPTDKTVLLQALHDVVSNMDSAQQA 654

IFTITVYLS+GISRGR+G+DAALNKA++NPWLTDPDKTVLLQALHDVVSN++S

Sbjct 617 IFTITVYLSEGITSRGRLGVDAALNMKAVTNPWLTDPVDKTVLLQALHDVVSNINS---- 672

Query 655 PSPSEPSECTLIDSHTVSNLTLITPDATMTLEEYVDAYDPATMCSNHWVGSAKIGTSSST 714

V LT+ITPDTT+EEYVAYDPATMCSNHWVG+AKIG+SST

Sbjct 673 ----------------VPGLTMITPDHTQTIEEYVAAYDPATMCSNHWVGAAKIGSSPST 716

Query 715 AVIDENAKVFNTDNLVSIQRAVLTLSFPSLIRVSQFIVDASIIPSLPMGNPHGMLMSAAE 774

AV+DENKVFNTDNL FIVDASIIP+LP+GNPHGMLMSAAE

Sbjct 717 AVVDENTKVFNTDNL--------------------FIVDASIIPALPVGNPHGMLMSAAE 756

Query 775 QAVTKILALSGGP 787

QA KILAL+GGP

Sbjct 757 QAAAKILALAGGP 769

Iron reductase domain / Cellobiose dehydrogenase [Trametes cinnabarina]

Sequence ID: CDO73368.1 Length: 1361 Number of Matches: 1

Range 1: 21 to 769

Score

Expect Method

Identities

Positives

Gaps

Frame

1219 bits(3153) 0.0()

Features:

Compositional matrix adjust. 610/793(77%) 674/793(84%) 52/793(6%)

Query 1

Sbjct 21

Query 61

Sbjct 81

VAAPYTDSGNGFVFDGITDAVYGVQYGIVLPQANSSTEFIGEIVAPIAAKWIGWAFGGAM 60

VAAPYDSGNGFVFDGITDVYVYGIVLPQA+S+EFIGEIVAP+AKWIGAGGAM

VAAPYVDSGNGFVFDGITDPVYHVSYGIVLPQATTSSEFIGEIVAPLDAKWIGLALGGAM 80

IGDLLLVAWPNGNDIVASTRYATEY------DGPTLTTLPSSSVNSTHWKYVFRCQNCTS 114

IGDLL+VAWPNGN+IV+STRYATY +GPT+TTLPSSVNSTHWK+VFRCQNCTS

IGDLLIVAWPNGNEIVSSTRYATAYQLPDVYEGPTITTLPSSLVNSTHWKFVFRCQNCTS 140

Query 115 WEGGGSISPSGTGVFAWAYSNVGVDDPSDPESTFQEHTDFGFYGINFPDAQNANYQNYLQ 174

WEGGGIP+GTGVFAWAYS+VGVDDPSDP+TFQEHTDFGF+GINFPDAQN+NYQNYLQ

Sbjct 141 WEGGGGIDPTGTGVFAWAYSSVGVDDPSDPNTTFQEHTDFGFFGINFPDAQNSNYQNYLQ 200

Query 175 GNPGTPPSSTTTTSTSTSTSTPTPTVTATPYDYIVVGAGPGGIITADRLSEAGKKVLLLE 234

GNGTPP++T+ +T++ PT+ATPYDYI+VGAGPGGIIADRLSEAGKKV+LLE

Sbjct 201 GNAGTPPPTSTPSGPTTTSKPTGPTASATPYDYIIVGAGPGGIIAADRLSEAGKKVILLE 260

Query 235 RGGPSTAETGGTYDAPWAQSANLTKFDVPGLFESMFTDSNSWWWCKAMHIDINFFAGCLL 294

RGGPSTAETGGTYAPWA+SNLTKFD+PGLFESMFTDNWWWCK DNFFAGCLL

Sbjct 261 RGGPSTAETGGTYYAPWAKSQNLTKFDIPGLFESMFTDPNPWWWCK----DTNFFAGCLL 316

Query 295 GGGTSINGA--LYWYPPDSDFQGANGWPNSWGNHAPYTSMLKQRLPSTDHPSTDGKRYLE 352

GGGTS+NGA LYWPD+DF ANGWPSWGNHAPYTSLKQRLPSTDHPSTDGKRYLE

Sbjct 317 GGGTSVNGAGSLYWLPSDADFSTANGWPTSWGNHAPYTSKLKQRLPSTDHPSTDGKRYLE 376

Query 353 ESAAIVAQLLNGQGYSNITINDNPDYKDHVYGYSAFDFIGGERGGPVATYFQTASARPNF 412

+SA+V+QLLGQGY ITINDNPDKDHV+GYSAFDFIG+RGPVATYFQTASARNF

Sbjct 377 QSATVVSQLLQGQGYQQITINDNPDSKDHVFGYSAFDFINGQRAGPVATYFQTASARSNF 436

Query 413 TYKQYVLVSQVIRNGSTITGVRTNDTSLGPDGIIPLNPNGRVVLSAGSFGTPRILFQSGI 472

YK+LVSQV+RNGSTITGVRTN+T+LGPDGI+PLNPNGRV+L+AGSFGTPRILFQSGI

Sbjct 437 VYKDFTLVSQVLRNGSTITGVRTNNTALGPDGIVPLNPNGRVILAAGSFGTPRILFQSGI 496

Query 473 GPEDMLQTVQSNPTASANLPAQSQWIDLPVGQGVSDNPSINLVFTHPSIDAYDNWADVWS 532

GPDM+QTVQSNPTA+ANLP+S+WI+LPVGQGVSDNPSINLVFTHPSIDAY+NWADVWS

Sbjct 497 GPTDMIQTVQSNPTAAANLPPESEWINLPVGQGVSDNPSINLVFTHPSIDAYENWADVWS 556

https://blast.ncbi.nlm.nih.gov/Blast.cgi

5/7


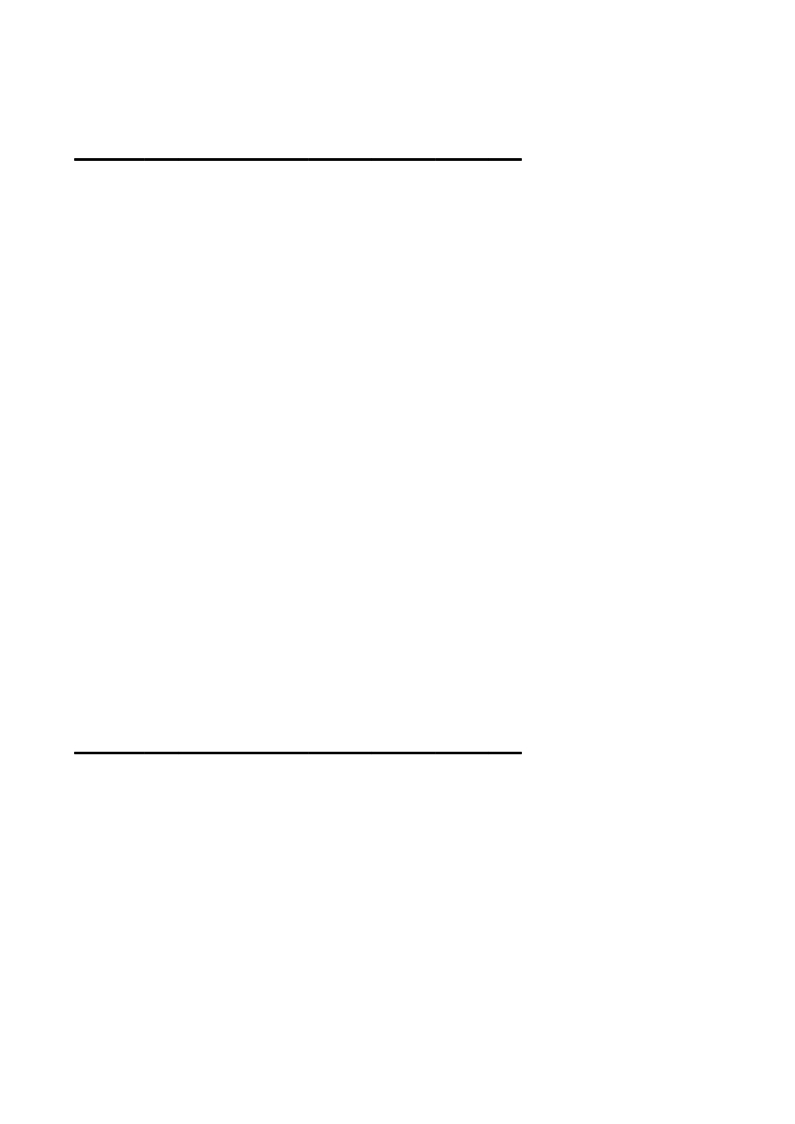


2017/4/9

NCBI Blast:GL22256-R1_1

Query 533 DPRPADAQQYLKDRSGVFAGASPKLNFWRAYGASDGVTRYAQGTVRPGAASVNSSLPYNA 592

+PRPADAQQYL+RSGVFAGASPKLNFWRAYGSDGTRYAQGTVRPGAASVN+S+YNA

Sbjct 557 NPRPADAQQYLQSRSGVFAGASPKLNFWRAYGGSDGKTRYAQGTVRPGAASVNTSVAYNA 616

Query 593 SNIFTITVYLSQGIASRGRIGIDAALNAKALSNPWLTDPTDKTVLLQALHDVVSNMDSAQ 652

SIFTITVYLS+GISRGR+G+DAALNKA++PWLTDPDKT+LLQALHDVVSN+++

Sbjct 617 SQIFTITVYLSEGITSRGRLGVDAALNMKAITTPWLTDPVDKTILLQALHDVVSNINN-- 674

Query 653 QAPSPSEPSECTLIDSHTVSNLTLITPDATMTLEEYVDAYDPATMCSNHWVGSAKIGTSS 712

V LTLITPDTTLE+YVAYDPATMCSNHWVG+AKIG+S

Sbjct 675 ------------------VPGLTLITPDHTQTLEQYVAAYDPATMCSNHWVGAAKIGSSP 716

Query 713 STAVIDENAKVFNTDNLVSIQRAVLTLSFPSLIRVSQFIVDASIIPSLPMGNPHGMLMSA 772

STAV+DENKVFNTDNL FIVDASIIPSLP+GNPHGLMSA

Sbjct 717 STAVVDENTKVFNTDNL--------------------FIVDASIIPSLPVGNPHGALMSA 756

Query 773 AEQAVTKILALSG 785

AEQA KILAL+G

Sbjct 757 AEQAAAKILALAG 769

cellobiose dehydrogenase, partial [Trametes cinnabarina]

Sequence ID: ADX41688.1 Length: 750 Number of Matches: 1

Range 1: 2 to 750

Score

Expect Method

Identities

Positives

Gaps

Frame

1216 bits(3147) 0.0()

Features:

Compositional matrix adjust. 612/793(77%) 676/793(85%) 50/793(6%)

Query 1

Sbjct 2

Query 61

Sbjct 62

VAAPYTDSGNGFVFDGITDAVYGVQYGIVLPQANSSTEFIGEIVAPIAAKWIGWAFGGAM 60

VAAPYDSGNGFVFDGITDVYVYGIVLPQA+S+EFIGEIVAP+AKWIGAGGAM

VAAPYVDSGNGFVFDGITDPVYHVSYGIVLPQATTSSEFIGEIVAPLDAKWIGLALGGAM 61

IGDLLLVAWPNGNDIVASTRYATEY------DGPTLTTLPSSSVNSTHWKYVFRCQNCTS 114

IGDLL+VAWPNGN+IV+STRYATY +GPT+TTLPSSVNSTHWK+VFRCQNCTS

IGDLLIVAWPNGNEIVSSTRYATAYQLPDVYEGPTITTLPSSLVNSTHWKFVFRCQNCTS 121

Query 115 WEGGGSISPSGTGVFAWAYSNVGVDDPSDPESTFQEHTDFGFYGINFPDAQNANYQNYLQ 174

WEGGGIP+GTGVFAWAYS+VGVDDPSDP+TFQEHTDFGF+GINFPDAQN+NYQNYLQ

Sbjct 122 WEGGGGIDPTGTGVFAWAYSSVGVDDPSDPNTTFQEHTDFGFFGINFPDAQNSNYQNYLQ 181

Query 175 GNPGTPPSSTTTTSTSTSTSTPTPTVTATPYDYIVVGAGPGGIITADRLSEAGKKVLLLE 234

GNGTPP++T+ +T++ PT+ATPYDYI+VGAGPGGIIADRLSEAGKKV+LLE

Sbjct 182 GNAGTPPPTSTPSGPTTTSKPTGPTASATPYDYIIVGAGPGGIIAADRLSEAGKKVILLE 241

Query 235 RGGPSTAETGGTYDAPWAQSANLTKFDVPGLFESMFTDSNSWWWCKAMHIDINFFAGCLL 294

RGGPSTAETGGTYAPWA+SNLTKFD+PGLFESMFTDNWWWCK DNFFAGCLL

Sbjct 242 RGGPSTAETGGTYYAPWAKSQNLTKFDIPGLFESMFTDPNPWWWCK----DTNFFAGCLL 297

Query 295 GGGTSINGALYWYPPDSDFQGANGWPNSWGNHAPYTSMLKQRLPSTDHPSTDGKRYLEES 354

GGGTS+NGALYWPD+DF ANGWPSWGNHAPYTSLKQRLPSTDHPSTDGKRYLE+S

Sbjct 298 GGGTSVNGALYWLPSDADFSTANGWPTSWGNHAPYTSKLKQRLPSTDHPSTDGKRYLEQS 357

Query 355 AAIVAQLLNGQGYSNITINDNPDYKDHVYGYSAFDFIGGERGGPVATYFQTASARPNFTY 414

A+V+QLLGQGY ITINDNPDKDHV+GYSAFDFIG+RGPVATYFQTASARNFY

Sbjct 358 ATVVSQLLQGQGYQQITINDNPDSKDHVFGYSAFDFINGQRAGPVATYFQTASARSNFVY 417

Query 415 KQYVLVSQVIRNGSTITGVRTNDTSLGPDGIIPLNPNGRVVLSAGSFGTPRILFQSGIGP 474

K+LVSQV+RNGSTITGVRTN+T+LGPDGI+PLNPNGRV+L+AGSFGTPRILFQSGIGP

Sbjct 418 KDFTLVSQVLRNGSTITGVRTNNTALGPDGIVPLNPNGRVILAAGSFGTPRILFQSGIGP 477

Query 475 EDMLQTVQSNPTASANLPAQSQWIDLPVGQGVSDNPSINLVFTHPSIDAYDNWADVWSDP 534

DM+QTVQSNPTA+ANLP+S+WI+LPVGQGVSDNPSINLVFTHPSIDAY+NWADVWS+P

Sbjct 478 TDMIQTVQSNPTAAANLPPESEWINLPVGQGVSDNPSINLVFTHPSIDAYENWADVWSNP 537

Query 535 RPADAQQYLKDRSGVFAGASPKLNFWRAYGASDGVTRYAQGTVRPGAASVNSSLPYNASN 594

RPADAQQYL+RSGVFAGASPKLNFWRAYGSDGTRYAQGTVRPGAASVN+S+YNAS

Sbjct 538 RPADAQQYLQSRSGVFAGASPKLNFWRAYGGSDGKTRYAQGTVRPGAASVNTSVAYNASQ 597

Query 595 IFTITVYLSQGIASRGRIGIDAALNAKALSNPWLTDPTDKTVLLQALHDVVSNMDSAQQA 654

IFTITVYLS+GISRGR+G+DAALNKA++PWLTDPDKT+LLQALHDVVSN+++

Sbjct 598 IFTITVYLSEGITSRGRLGVDAALNMKAITTPWLTDPVDKTILLQALHDVVSNINN---- 653

Query 655 PSPSEPSECTLIDSHTVSNLTLITPDATMTLEEYVDAYDPATMCSNHWVGSAKIGTSSST 714

V LTLITPDTTLE+YVAYDPATMCSNHWVG+AKIG+SST

Sbjct 654 ----------------VPGLTLITPDHTQTLEQYVAAYDPATMCSNHWVGAAKIGSSPST 697

Query 715 AVIDENAKVFNTDNLVSIQRAVLTLSFPSLIRVSQFIVDASIIPSLPMGNPHGMLMSAAE 774

AV+DENKVFNTDNL FIVDASIIPSLP+GNPHGLMSAAE

Sbjct 698 AVVDENTKVFNTDNL--------------------FIVDASIIPSLPVGNPHGALMSAAE 737

Query 775 QAVTKILALSGGP 787

QA KILAL+GGP

Sbjct 738 QAAAKILALAGGP 750

cellobiose dehydrogenase [Trametes cinnabarina]

Sequence ID: AAC32197.1 Length: 769 Number of Matches: 1

Range 1: 21 to 769

Score

Expect Method

Identities

Positives

Gaps

Frame

1216 bits(3146) 0.0()

Features:

Compositional matrix adjust. 612/793(77%) 674/793(84%) 50/793(6%)

Query 1

Sbjct 21

Query 61

Sbjct 81

VAAPYTDSGNGFVFDGITDAVYGVQYGIVLPQANSSTEFIGEIVAPIAAKWIGWAFGGAM 60

VAAPYDSGNGFVFDGITDVYVYGIVLPQA+S+EFIGEIVAP+AKWIGAGGAM

VAAPYVDSGNGFVFDGITDPVYHVSYGIVLPQATTSSEFIGEIVAPLDAKWIGLALGGAM 80

IGDLLLVAWPNGNDIVASTRYATEYD------GPTLTTLPSSSVNSTHWKYVFRCQNCTS 114

IGDLL+VAWPNGN+IV+STRYATY GPT+TTLPSSVNSTHWK+VFRCQNCTS

IGDLLIVAWPNGNEIVSSTRYATAYQLPDVYAGPTITTLPSSLVNSTHWKFVFRCQNCTS 140

Query 115 WEGGGSISPSGTGVFAWAYSNVGVDDPSDPESTFQEHTDFGFYGINFPDAQNANYQNYLQ 174

WEGGGIP+GTGVFAWAYS+VGVDDPSDP+TFQEHTDFGF+GINFPDAQN+NYQNYLQ

Sbjct 141 WEGGGGIDPTGTGVFAWAYSSVGVDDPSDPNTTFQEHTDFGFFGINFPDAQNSNYQNYLQ 200

Query 175 GNPGTPPSSTTTTSTSTSTSTPTPTVTATPYDYIVVGAGPGGIITADRLSEAGKKVLLLE 234

GNGTPP++T+ +T++ PT+ATPYDYI+VGAGPGGIIADRLSEAGKKV+LLE

Sbjct 201 GNAGTPPPTSTPSGPTTTSKPTGPTASATPYDYIIVGAGPGGIIAADRLSEAGKKVILLE 260

Query 235 RGGPSTAETGGTYDAPWAQSANLTKFDVPGLFESMFTDSNSWWWCKAMHIDINFFAGCLL 294

RGGPSTAETGGTYAPWA+SNLTKFD+PGLFESMFTDNWWWCK

https://blast.ncbi.nlm.nih.gov/Blast.cgi

DNFFAGCLL

6/7


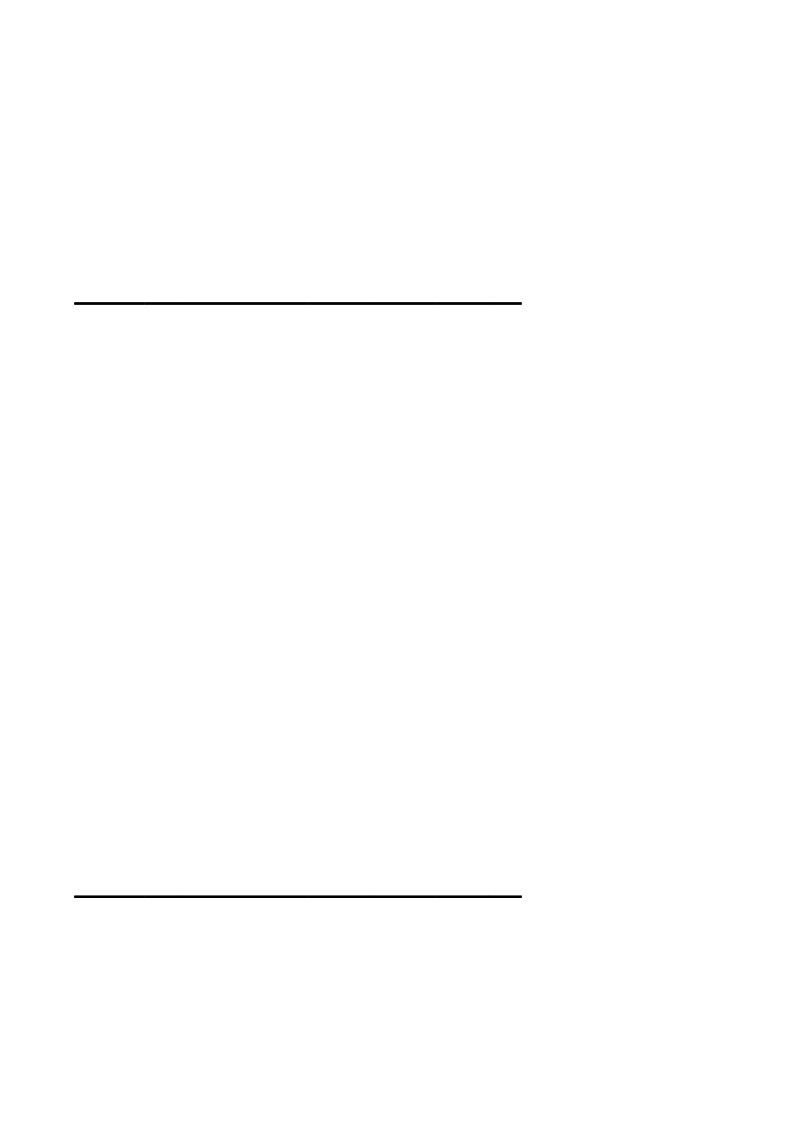


2017/4/9

NCBI Blast:GL22256-R1_1

RGGPSTAETGGTYAPWA+SNLTKFD+PGLFESMFTDNWWWCK DNFFAGCLL

Sbjct 261 RGGPSTAETGGTYYAPWAKSQNLTKFDIPGLFESMFTDPNPWWWCK----DTNFFAGCLL 316

Query 295 GGGTSINGALYWYPPDSDFQGANGWPNSWGNHAPYTSMLKQRLPSTDHPSTDGKRYLEES 354

GGGTS+NGALYWPD+DF ANGWP+WGNHAPYTSLKQRLPSTDHPSDGRYLE+S

Sbjct 317 GGGTSVNGALYWLPSDADFSTANGWPTNWGNHAPYTSKLKQRLPSTDHPSADGNRYLEQS 376

Query 355 AAIVAQLLNGQGYSNITINDNPDYKDHVYGYSAFDFIGGERGGPVATYFQTASARPNFTY 414

A+V+QLLGQGY ITINDNPDYKDHV+GYSAFDFIG+RGPVATYFQTASARNFY

Sbjct 377 ATVVSQLLQGQGYQQITINDNPDYKDHVFGYSAFDFINGQRAGPVATYFQTASARSNFVY 436

Query 415 KQYVLVSQVIRNGSTITGVRTNDTSLGPDGIIPLNPNGRVVLSAGSFGTPRILFQSGIGP 474

KYLVSQV+RNGSTITGVRTN+T+LGPDGI+PLNPNGRV+L+AGSFGTPRILFQSGIGP

Sbjct 437 KDYTLVSQVLRNGSTITGVRTNNTALGPDGIVPLNPNGRVILAAGSFGTPRILFQSGIGP 496

Query 475 EDMLQTVQSNPTASANLPAQSQWIDLPVGQGVSDNPSINLVFTHPSIDAYDNWADVWSDP 534

DM+QTVQSNPTA+ANLPQS+WI+LPVGQGVSDNPSINLVFTHPSIDAY+NWADVWS+P

Sbjct 497 TDMIQTVQSNPTAAANLPPQSEWINLPVGQGVSDNPSINLVFTHPSIDAYENWADVWSNP 556

Query 535 RPADAQQYLKDRSGVFAGASPKLNFWRAYGASDGVTRYAQGTVRPGAASVNSSLPYNASN 594

RPADAQQYL+RSGVFAGASPKLNFWRAYGSDGTRYAQGTVRPGAASVN+S+YNAS

Sbjct 557 RPADAQQYLQSRSGVFAGASPKLNFWRAYGGSDGKTRYAQGTVRPGAASVNTSVAYNASQ 616

Query 595 IFTITVYLSQGIASRGRIGIDAALNAKALSNPWLTDPTDKTVLLQALHDVVSNMDSAQQA 654

IFTITVYLS+GISRGR+G+DAALNKA++PWLTDPDKT+LLQALHDVVSN+++

Sbjct 617 IFTITVYLSEGITSRGRLGVDAALNMKAITTPWLTDPVDKTILLQALHDVVSNINN---- 672

Query 655 PSPSEPSECTLIDSHTVSNLTLITPDATMTLEEYVDAYDPATMCSNHWVGSAKIGTSSST 714

V LTLITPDTTLE+YVAYDPATMCSNHWVG+AKIG+SST

Sbjct 673 ----------------VPGLTLITPDHTQTLEQYVAAYDPATMCSNHWVGAAKIGSSPST 716

Query 715 AVIDENAKVFNTDNLVSIQRAVLTLSFPSLIRVSQFIVDASIIPSLPMGNPHGMLMSAAE 774

AV+DENKVFNTDNL FIVDASIIPSLP+GNPHGLMSAAE

Sbjct 717 AVVDENTKVFNTDNL--------------------FIVDASIIPSLPVGNPHGALMSAAE 756

Query 775 QAVTKILALSGGP 787

QA KILAL+GGP

Sbjct 757 QAAAKILALAGGP 769

BLAST is a registered trademark of the National Library of Medicine

[Support center](https://support.ncbi.nlm.nih.gov/ics/support/KBList.asp?style=classic&deptID=28049&folderID=11&) [Mailing list](https://blast.ncbi.nlm.nih.gov/Blast.cgi?CMD=Web&PAGE_TYPE=BlastDocs&DOC_TYPE=MailList)

[YouTube](https://www.youtube.com/ncbinlm)

[National Library Of Medicine](https://www.nlm.nih.gov/)

[National Institutes Of Health](https://www.nih.gov/)

[U.S. Department of Health & Human Services](https://www.hhs.gov/)

[USA.gov](https://www.usa.gov/)

[NCBI](https://www.ncbi.nlm.nih.gov/)

[*National Center for Biotechnology Information,*](https://www.ncbi.nlm.nih.gov/) *U.S. National Library of Medicine 8600 Rock ville Pik e, Bethesda MD, 20894 USA*

[Policies and Guidelines](https://www.ncbi.nlm.nih.gov/home/about/policies.shtml) | [Contact](https://www.ncbi.nlm.nih.gov/home/about/contact.shtml)

https://blast.ncbi.nlm.nih.gov/Blast.cgi

7/7


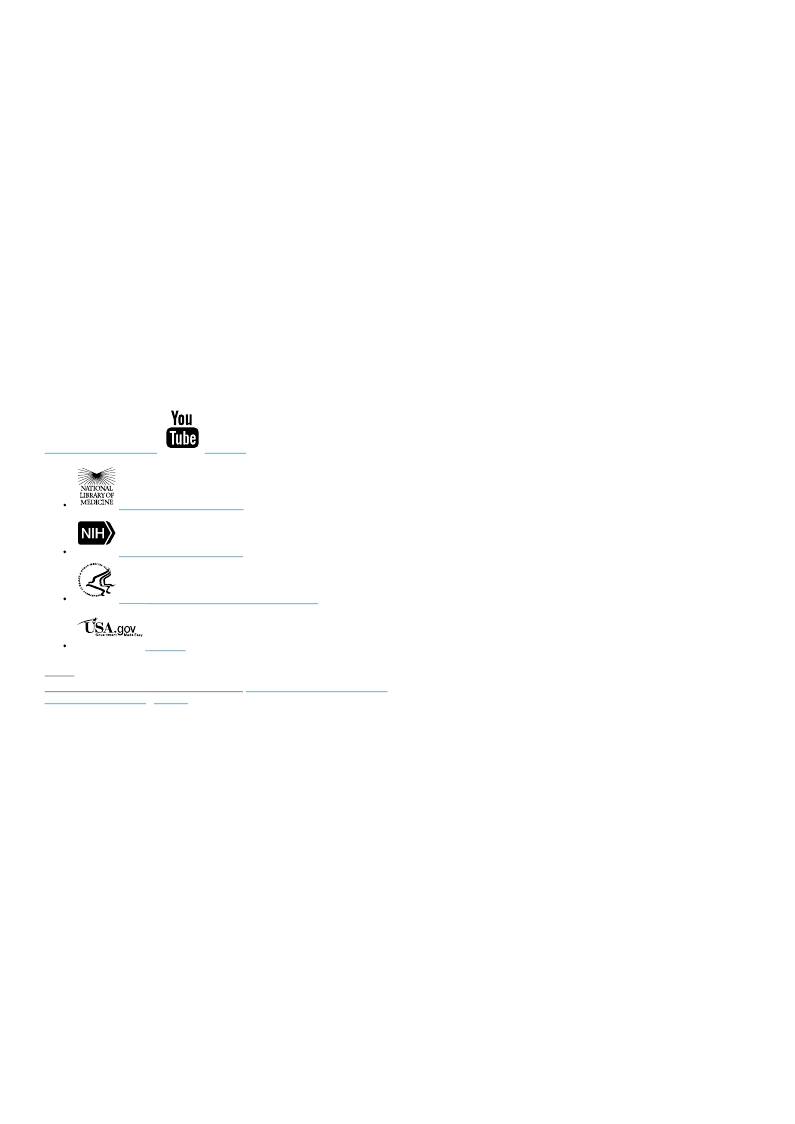

Supplement: Supplementary file 18 — Supplementary File 3c [file 41598_2017_4303_MOESM18_ESM.doc]
