# Supplementary material for: Genome-wide Identification and Characterization of Natural Antisense Transcripts by Strand-specific RNA Sequencing in Ganoderma lucidum
Source: Sci Rep. 2017 Jul 18;7:5711. doi: 10.1038/s41598-017-04303-6 (PMC5515960; doi:10.1038/s41598-017-04303-6)

2017/4/9

NCBI Blast:GL19134-R1_1

[BLAST ®](https://blast.ncbi.nlm.nih.gov/Blast.cgi) » blastp suite » RID-EMDGFCVG01R

BLAST Results

Job title: GL19134-R1_1

RID

[EMDGFCVG01R](https://blast.ncbi.nlm.nih.gov/Blast.cgi?CMD=Get&RID=EMDGFCVG01R) (Expires on 04-10 21:24 pm)

Query ID

lcl|Query_21665

Database Name

nr

Description

Molecule type

Query Length

GL19134-R1_1

amino acid

615

Description All non-redundant GenBank CDS

translations+PDB+SwissProt+PIR+PRF excluding

environmental samples from WGS projects

Program BLASTP 2.6.0+

New Analyze your query with SmartBLAST

Graphic Summary

Putative conserved domains have been detected, click on the image below for detailed results.

Distribution of the top 100 Blast Hits on 100 subject sequences

Color key for alignment scores

<40

40-50

50-80

80-200

>=200

Query

1

100

200

300

400

500

600

https://blast.ncbi.nlm.nih.gov/Blast.cgi

1/6


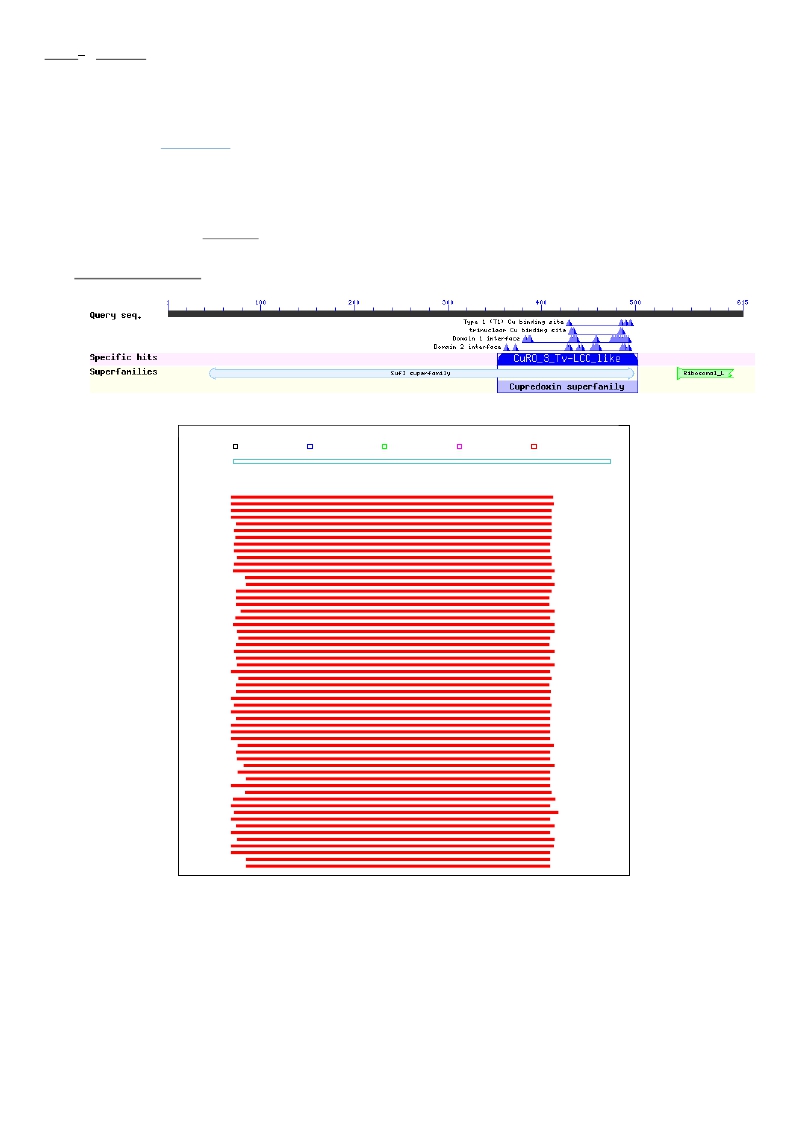


2017/4/9

Descriptions

Sequences producing significant alignments:

NCBI Blast:GL19134-R1_1

Description

[Max](https://blast.ncbi.nlm.nih.gov/Blast.cgi?CMD=Get&ALIGNMENTS=100&ALIGNMENT_VIEW=Pairwise&CDD_RID=EMDGEX6J01R&CDD_SEARCH_STATE=0&DATABASE_SORT=0&DESCRIPTIONS=100&DYNAMIC_FORMAT=on&FIRST_QUERY_NUM=0&FORMAT_OBJECT=Alignment&FORMAT_PAGE_TARGET=&FORMAT_TYPE=HTML&GET_SEQUENCE=yes&I_THRESH=&LINE_LENGTH=60&MASK_CHAR=2&MASK_COLOR=1&NEW_VIEW=yes&NUM_OVERVIEW=100&PAGE=Proteins&QUERY_INDEX=0&QUERY_NUMBER=0&RESULTS_PAGE_TARGET=&RID=EMDGFCVG01R&SHOW_LINKOUT=yes&SHOW_OVERVIEW=yes&STEP_NUMBER=&WORD_SIZE=6&OLD_VIEW=false&DISPLAY_SORT=1&HSP_SORT=1)

[Total](https://blast.ncbi.nlm.nih.gov/Blast.cgi?CMD=Get&ALIGNMENTS=100&ALIGNMENT_VIEW=Pairwise&CDD_RID=EMDGEX6J01R&CDD_SEARCH_STATE=0&DATABASE_SORT=0&DESCRIPTIONS=100&DYNAMIC_FORMAT=on&FIRST_QUERY_NUM=0&FORMAT_OBJECT=Alignment&FORMAT_PAGE_TARGET=&FORMAT_TYPE=HTML&GET_SEQUENCE=yes&I_THRESH=&LINE_LENGTH=60&MASK_CHAR=2&MASK_COLOR=1&NEW_VIEW=yes&NUM_OVERVIEW=100&PAGE=Proteins&QUERY_INDEX=0&QUERY_NUMBER=0&RESULTS_PAGE_TARGET=&RID=EMDGFCVG01R&SHOW_LINKOUT=yes&SHOW_OVERVIEW=yes&STEP_NUMBER=&WORD_SIZE=6&OLD_VIEW=false&DISPLAY_SORT=2&HSP_SORT=1)

[Query](https://blast.ncbi.nlm.nih.gov/Blast.cgi?CMD=Get&ALIGNMENTS=100&ALIGNMENT_VIEW=Pairwise&CDD_RID=EMDGEX6J01R&CDD_SEARCH_STATE=0&DATABASE_SORT=0&DESCRIPTIONS=100&DYNAMIC_FORMAT=on&FIRST_QUERY_NUM=0&FORMAT_OBJECT=Alignment&FORMAT_PAGE_TARGET=&FORMAT_TYPE=HTML&GET_SEQUENCE=yes&I_THRESH=&LINE_LENGTH=60&MASK_CHAR=2&MASK_COLOR=1&NEW_VIEW=yes&NUM_OVERVIEW=100&PAGE=Proteins&QUERY_INDEX=0&QUERY_NUMBER=0&RESULTS_PAGE_TARGET=&RID=EMDGFCVG01R&SHOW_LINKOUT=yes&SHOW_OVERVIEW=yes&STEP_NUMBER=&WORD_SIZE=6&OLD_VIEW=false&DISPLAY_SORT=4&HSP_SORT=0)

[E](https://blast.ncbi.nlm.nih.gov/Blast.cgi?CMD=Get&ALIGNMENTS=100&ALIGNMENT_VIEW=Pairwise&CDD_RID=EMDGEX6J01R&CDD_SEARCH_STATE=0&DATABASE_SORT=0&DESCRIPTIONS=100&DYNAMIC_FORMAT=on&FIRST_QUERY_NUM=0&FORMAT_OBJECT=Alignment&FORMAT_PAGE_TARGET=&FORMAT_TYPE=HTML&GET_SEQUENCE=yes&I_THRESH=&LINE_LENGTH=60&MASK_CHAR=2&MASK_COLOR=1&NEW_VIEW=yes&NUM_OVERVIEW=100&PAGE=Proteins&QUERY_INDEX=0&QUERY_NUMBER=0&RESULTS_PAGE_TARGET=&RID=EMDGFCVG01R&SHOW_LINKOUT=yes&SHOW_OVERVIEW=yes&STEP_NUMBER=&WORD_SIZE=6&OLD_VIEW=false&DISPLAY_SORT=0&HSP_SORT=0)

[Ident](https://blast.ncbi.nlm.nih.gov/Blast.cgi?CMD=Get&ALIGNMENTS=100&ALIGNMENT_VIEW=Pairwise&CDD_RID=EMDGEX6J01R&CDD_SEARCH_STATE=0&DATABASE_SORT=0&DESCRIPTIONS=100&DYNAMIC_FORMAT=on&FIRST_QUERY_NUM=0&FORMAT_OBJECT=Alignment&FORMAT_PAGE_TARGET=&FORMAT_TYPE=HTML&GET_SEQUENCE=yes&I_THRESH=&LINE_LENGTH=60&MASK_CHAR=2&MASK_COLOR=1&NEW_VIEW=yes&NUM_OVERVIEW=100&PAGE=Proteins&QUERY_INDEX=0&QUERY_NUMBER=0&RESULTS_PAGE_TARGET=&RID=EMDGFCVG01R&SHOW_LINKOUT=yes&SHOW_OVERVIEW=yes&STEP_NUMBER=&WORD_SIZE=6&DISPLAY_SORT=3&HSP_SORT=3)

Accession

[score](https://blast.ncbi.nlm.nih.gov/Blast.cgi?CMD=Get&ALIGNMENTS=100&ALIGNMENT_VIEW=Pairwise&CDD_RID=EMDGEX6J01R&CDD_SEARCH_STATE=0&DATABASE_SORT=0&DESCRIPTIONS=100&DYNAMIC_FORMAT=on&FIRST_QUERY_NUM=0&FORMAT_OBJECT=Alignment&FORMAT_PAGE_TARGET=&FORMAT_TYPE=HTML&GET_SEQUENCE=yes&I_THRESH=&LINE_LENGTH=60&MASK_CHAR=2&MASK_COLOR=1&NEW_VIEW=yes&NUM_OVERVIEW=100&PAGE=Proteins&QUERY_INDEX=0&QUERY_NUMBER=0&RESULTS_PAGE_TARGET=&RID=EMDGFCVG01R&SHOW_LINKOUT=yes&SHOW_OVERVIEW=yes&STEP_NUMBER=&WORD_SIZE=6&OLD_VIEW=false&DISPLAY_SORT=1&HSP_SORT=1)

[score](https://blast.ncbi.nlm.nih.gov/Blast.cgi?CMD=Get&ALIGNMENTS=100&ALIGNMENT_VIEW=Pairwise&CDD_RID=EMDGEX6J01R&CDD_SEARCH_STATE=0&DATABASE_SORT=0&DESCRIPTIONS=100&DYNAMIC_FORMAT=on&FIRST_QUERY_NUM=0&FORMAT_OBJECT=Alignment&FORMAT_PAGE_TARGET=&FORMAT_TYPE=HTML&GET_SEQUENCE=yes&I_THRESH=&LINE_LENGTH=60&MASK_CHAR=2&MASK_COLOR=1&NEW_VIEW=yes&NUM_OVERVIEW=100&PAGE=Proteins&QUERY_INDEX=0&QUERY_NUMBER=0&RESULTS_PAGE_TARGET=&RID=EMDGFCVG01R&SHOW_LINKOUT=yes&SHOW_OVERVIEW=yes&STEP_NUMBER=&WORD_SIZE=6&OLD_VIEW=false&DISPLAY_SORT=2&HSP_SORT=1)

[cover](https://blast.ncbi.nlm.nih.gov/Blast.cgi?CMD=Get&ALIGNMENTS=100&ALIGNMENT_VIEW=Pairwise&CDD_RID=EMDGEX6J01R&CDD_SEARCH_STATE=0&DATABASE_SORT=0&DESCRIPTIONS=100&DYNAMIC_FORMAT=on&FIRST_QUERY_NUM=0&FORMAT_OBJECT=Alignment&FORMAT_PAGE_TARGET=&FORMAT_TYPE=HTML&GET_SEQUENCE=yes&I_THRESH=&LINE_LENGTH=60&MASK_CHAR=2&MASK_COLOR=1&NEW_VIEW=yes&NUM_OVERVIEW=100&PAGE=Proteins&QUERY_INDEX=0&QUERY_NUMBER=0&RESULTS_PAGE_TARGET=&RID=EMDGFCVG01R&SHOW_LINKOUT=yes&SHOW_OVERVIEW=yes&STEP_NUMBER=&WORD_SIZE=6&OLD_VIEW=false&DISPLAY_SORT=4&HSP_SORT=0)

[value](https://blast.ncbi.nlm.nih.gov/Blast.cgi?CMD=Get&ALIGNMENTS=100&ALIGNMENT_VIEW=Pairwise&CDD_RID=EMDGEX6J01R&CDD_SEARCH_STATE=0&DATABASE_SORT=0&DESCRIPTIONS=100&DYNAMIC_FORMAT=on&FIRST_QUERY_NUM=0&FORMAT_OBJECT=Alignment&FORMAT_PAGE_TARGET=&FORMAT_TYPE=HTML&GET_SEQUENCE=yes&I_THRESH=&LINE_LENGTH=60&MASK_CHAR=2&MASK_COLOR=1&NEW_VIEW=yes&NUM_OVERVIEW=100&PAGE=Proteins&QUERY_INDEX=0&QUERY_NUMBER=0&RESULTS_PAGE_TARGET=&RID=EMDGFCVG01R&SHOW_LINKOUT=yes&SHOW_OVERVIEW=yes&STEP_NUMBER=&WORD_SIZE=6&OLD_VIEW=false&DISPLAY_SORT=0&HSP_SORT=0)

laccase [Ganoderma lucidum]

laccase [Ganoderma lucidum]

laccase C [Trametes sp. 420]

laccase B [Trametes sp. 420]

laccase D [Trametes hirsuta]

Laccase-3 [Trametes pubescens]

TvLac7 [Trametes versicolor FP-101664 SS1]

multicopper oxidase [Phlebia tremellosa]

multiple oxidase [Phlebia tremellosa]

laccase D [Trametes ochracea]

laccase (EC 1.10.3.2) 3 precursor - white-rot fungus (Trametes

villosa)

laccase [Meripilus giganteus]

TvLac6 [Trametes versicolor FP-101664 SS1]

laccase [Cerrena sp. WR1]

laccase C [Trametes ochracea]

laccase B [Dichomitus squalens LYAD-421 SS1]

laccase [Coriolopsis gallica]

laccase [Phlebia chrysocreas]

laccase 1 [Steccherinum murashkinskyi]

1098

782

724

719

676

662

660

655

655

654

654

654

652

649

649

642

641

640

639

1098

782

724

719

676

662

660

655

655

654

654

654

652

649

649

642

641

640

639

86%

86%

85%

85%

84%

84%

84%

84%

84%

84%

84%

85%

81%

82%

84%

83%

83%

83%

84%

0.0

0.0

0.0

0.0

0.0

0.0

0.0

0.0

0.0

0.0

0.0

0.0

0.0

0.0

0.0

0.0

0.0

0.0

0.0

100%

69%

66%

66%

63%

61%

62%

62%

62%

62%

62%

60%

63%

63%

62%

61%

61%

60%

60%

[AHA83590.1](https://www.ncbi.nlm.nih.gov/protein/558633463?report=genbank&log$=prottop&blast_rank=1&RID=EMDGFCVG01R)

[AHA83587.1](https://www.ncbi.nlm.nih.gov/protein/558633457?report=genbank&log$=prottop&blast_rank=2&RID=EMDGFCVG01R)

[AAW28938.1](https://www.ncbi.nlm.nih.gov/protein/56785446?report=genbank&log$=prottop&blast_rank=3&RID=EMDGFCVG01R)

[AAW28937.1](https://www.ncbi.nlm.nih.gov/protein/56785444?report=genbank&log$=prottop&blast_rank=4&RID=EMDGFCVG01R)

[AIZ72723.1](https://www.ncbi.nlm.nih.gov/protein/732554700?report=genbank&log$=prottop&blast_rank=5&RID=EMDGFCVG01R)

[OJT13485.1](https://www.ncbi.nlm.nih.gov/protein/1112960306?report=genbank&log$=prottop&blast_rank=6&RID=EMDGFCVG01R)

[XP_008038288.1](https://www.ncbi.nlm.nih.gov/protein/636614307?report=genbank&log$=prottop&blast_rank=7&RID=EMDGFCVG01R)

[CAK54346.1](https://www.ncbi.nlm.nih.gov/protein/109287624?report=genbank&log$=prottop&blast_rank=8&RID=EMDGFCVG01R)

[CAR47803.1](https://www.ncbi.nlm.nih.gov/protein/194719800?report=genbank&log$=prottop&blast_rank=9&RID=EMDGFCVG01R)

[ALT22027.1](https://www.ncbi.nlm.nih.gov/protein/965871874?report=genbank&log$=prottop&blast_rank=10&RID=EMDGFCVG01R)

[JC5355](https://www.ncbi.nlm.nih.gov/protein/7432992?report=genbank&log$=prottop&blast_rank=11&RID=EMDGFCVG01R)

[CBV46340.1](https://www.ncbi.nlm.nih.gov/protein/317451550?report=genbank&log$=prottop&blast_rank=12&RID=EMDGFCVG01R)

[XP_008038233.1](https://www.ncbi.nlm.nih.gov/protein/636614197?report=genbank&log$=prottop&blast_rank=13&RID=EMDGFCVG01R)

[ACZ58369.1](https://www.ncbi.nlm.nih.gov/protein/270047924?report=genbank&log$=prottop&blast_rank=14&RID=EMDGFCVG01R)

[ALT22026.1](https://www.ncbi.nlm.nih.gov/protein/965871847?report=genbank&log$=prottop&blast_rank=15&RID=EMDGFCVG01R)

[XP_007369195.1](https://www.ncbi.nlm.nih.gov/protein/598003325?report=genbank&log$=prottop&blast_rank=16&RID=EMDGFCVG01R)

[AAF70119.2](https://www.ncbi.nlm.nih.gov/protein/12484399?report=genbank&log$=prottop&blast_rank=17&RID=EMDGFCVG01R)

[ALF95043.1](https://www.ncbi.nlm.nih.gov/protein/930577647?report=genbank&log$=prottop&blast_rank=18&RID=EMDGFCVG01R)

[AFI41888.1](https://www.ncbi.nlm.nih.gov/protein/385139612?report=genbank&log$=prottop&blast_rank=19&RID=EMDGFCVG01R)

RecName: Full=Laccase; AltName: Full=Benzenediol:oxygen

oxidoreductase; AltName: Full=Diphenol oxidase; AltName:

Full=Ligninolytic phenoloxidase; AltName: Full=Urishiol

639

639

85%

0.0

59%

[Q01679.2](https://www.ncbi.nlm.nih.gov/protein/47117883?report=genbank&log$=prottop&blast_rank=20&RID=EMDGFCVG01R)

oxidase; Flags: Precursor

laccase [Cerrena unicolor]

laccase [Phlebia chrysocreas]

laccase [Coriolopsis gallica]

laccase [Rigidoporus microporus]

laccase [Piloderma croceum F 1598]

laccase 7 precursor [Cerrena sp. HYB07]

laccase 2 [Trametes hirsuta]

Laccase [Trametes cinnabarina]

laccase 3 [Coriolopsis trogii]

laccase [Phlebia chrysocreas]

laccase [Coriolopsis gallica]

laccase 2 [Steccherinum murashkinskyi]

laccase B [Trametes sp. AH28-2]

hypothetical protein PLICRDRAFT_113151 [Plicaturopsis

crispa FD-325 SS-3]

laccase C [Trametes hirsuta]

mutant laccase [synthetic construct]

laccase [Coriolopsis gallica]

laccase [Cerrena sp. WR1]

laccase [Piloderma croceum F 1598]

laccase [Gelatoporia subvermispora B]

Lac2 protein [Phlebia radiata]

laccase 1 [Coprinus comatus]

639

637

637

636

635

635

634

634

633

632

632

632

632

631

631

630

629

629

628

628

628

628

639

637

637

636

635

635

634

634

633

632

632

632

632

631

631

630

629

629

628

628

628

628

84%

83%

83%

85%

83%

84%

85%

83%

83%

84%

85%

84%

85%

83%

85%

85%

85%

84%

83%

83%

82%

83%

0.0

0.0

0.0

0.0

0.0

0.0

0.0

0.0

0.0

0.0

0.0

0.0

0.0

0.0

0.0

0.0

0.0

0.0

0.0

0.0

0.0

0.0

61%

61%

61%

58%

60%

61%

59%

60%

60%

60%

60%

59%

59%

59%

59%

60%

59%

60%

60%

61%

60%

59%

[ALE66000.1](https://www.ncbi.nlm.nih.gov/protein/928193500?report=genbank&log$=prottop&blast_rank=21&RID=EMDGFCVG01R)

[ALF95042.1](https://www.ncbi.nlm.nih.gov/protein/930577645?report=genbank&log$=prottop&blast_rank=22&RID=EMDGFCVG01R)

[AHM10329.1](https://www.ncbi.nlm.nih.gov/protein/594551748?report=genbank&log$=prottop&blast_rank=23&RID=EMDGFCVG01R)

[ACL93333.1](https://www.ncbi.nlm.nih.gov/protein/255523026?report=genbank&log$=prottop&blast_rank=24&RID=EMDGFCVG01R)

[KIM83705.1](https://www.ncbi.nlm.nih.gov/protein/751735413?report=genbank&log$=prottop&blast_rank=25&RID=EMDGFCVG01R)

[AID59415.1](https://www.ncbi.nlm.nih.gov/protein/658306918?report=genbank&log$=prottop&blast_rank=26&RID=EMDGFCVG01R)

[AOX15703.1](https://www.ncbi.nlm.nih.gov/protein/1083918173?report=genbank&log$=prottop&blast_rank=27&RID=EMDGFCVG01R)

[CDO69696.1](https://www.ncbi.nlm.nih.gov/protein/691795569?report=genbank&log$=prottop&blast_rank=28&RID=EMDGFCVG01R)

[AMJ39540.1](https://www.ncbi.nlm.nih.gov/protein/995953024?report=genbank&log$=prottop&blast_rank=29&RID=EMDGFCVG01R)

[ALF95040.1](https://www.ncbi.nlm.nih.gov/protein/930577641?report=genbank&log$=prottop&blast_rank=30&RID=EMDGFCVG01R)

[AJV90967.1](https://www.ncbi.nlm.nih.gov/protein/768806945?report=genbank&log$=prottop&blast_rank=31&RID=EMDGFCVG01R)

[AFI41889.1](https://www.ncbi.nlm.nih.gov/protein/385141759?report=genbank&log$=prottop&blast_rank=32&RID=EMDGFCVG01R)

[AAW31597.1](https://www.ncbi.nlm.nih.gov/protein/56809865?report=genbank&log$=prottop&blast_rank=33&RID=EMDGFCVG01R)

[KII87346.1](https://www.ncbi.nlm.nih.gov/protein/749762562?report=genbank&log$=prottop&blast_rank=34&RID=EMDGFCVG01R)

[AIZ72722.1](https://www.ncbi.nlm.nih.gov/protein/732554698?report=genbank&log$=prottop&blast_rank=35&RID=EMDGFCVG01R)

[AHW51131.1](https://www.ncbi.nlm.nih.gov/protein/608606234?report=genbank&log$=prottop&blast_rank=36&RID=EMDGFCVG01R)

[AJV90966.1](https://www.ncbi.nlm.nih.gov/protein/768806943?report=genbank&log$=prottop&blast_rank=37&RID=EMDGFCVG01R)

[ACZ58367.1](https://www.ncbi.nlm.nih.gov/protein/270047920?report=genbank&log$=prottop&blast_rank=38&RID=EMDGFCVG01R)

[KIM83693.1](https://www.ncbi.nlm.nih.gov/protein/751735401?report=genbank&log$=prottop&blast_rank=39&RID=EMDGFCVG01R)

[EMD37153.1](https://www.ncbi.nlm.nih.gov/protein/449546183?report=genbank&log$=prottop&blast_rank=40&RID=EMDGFCVG01R)

[CAI56705.1](https://www.ncbi.nlm.nih.gov/protein/113207314?report=genbank&log$=prottop&blast_rank=41&RID=EMDGFCVG01R)

[AFD97050.1](https://www.ncbi.nlm.nih.gov/protein/380704397?report=genbank&log$=prottop&blast_rank=42&RID=EMDGFCVG01R)

Chain A, Crystallographic Structural Determination Of A

Trigonal Laccase From Coriolopsis Gallica (cgl) To 1.5 A

627

627

81%

0.0

61%

[5A7E_A](https://www.ncbi.nlm.nih.gov/protein/1032208307?report=genbank&log$=prottop&blast_rank=43&RID=EMDGFCVG01R)

Resolution

laccase [Coriolopsis gallica]

Chain A, Steccherinum Murashkinskyi Laccase At 0.95

Resolution

laccase 2 precursor [Cerrena sp. HYB07]

polyphenoloxidase [Trametes sp. C30]

laccase 5 precursor [Cerrena sp. HYB07]

627

627

627

627

627

627

627

627

627

627

85%

81%

86%

85%

86%

0.0

0.0

0.0

0.0

0.0

59%

60%

60%

60%

59%

[ABD93940.1](https://www.ncbi.nlm.nih.gov/protein/90436931?report=genbank&log$=prottop&blast_rank=44&RID=EMDGFCVG01R)

[5E9N_A](https://www.ncbi.nlm.nih.gov/protein/969812945?report=genbank&log$=prottop&blast_rank=45&RID=EMDGFCVG01R)

[AID59410.1](https://www.ncbi.nlm.nih.gov/protein/658306908?report=genbank&log$=prottop&blast_rank=46&RID=EMDGFCVG01R)

[AAF06967.1](https://www.ncbi.nlm.nih.gov/protein/6318611?report=genbank&log$=prottop&blast_rank=47&RID=EMDGFCVG01R)

[AID59413.1](https://www.ncbi.nlm.nih.gov/protein/658306914?report=genbank&log$=prottop&blast_rank=48&RID=EMDGFCVG01R)

https://blast.ncbi.nlm.nih.gov/Blast.cgi

2/6


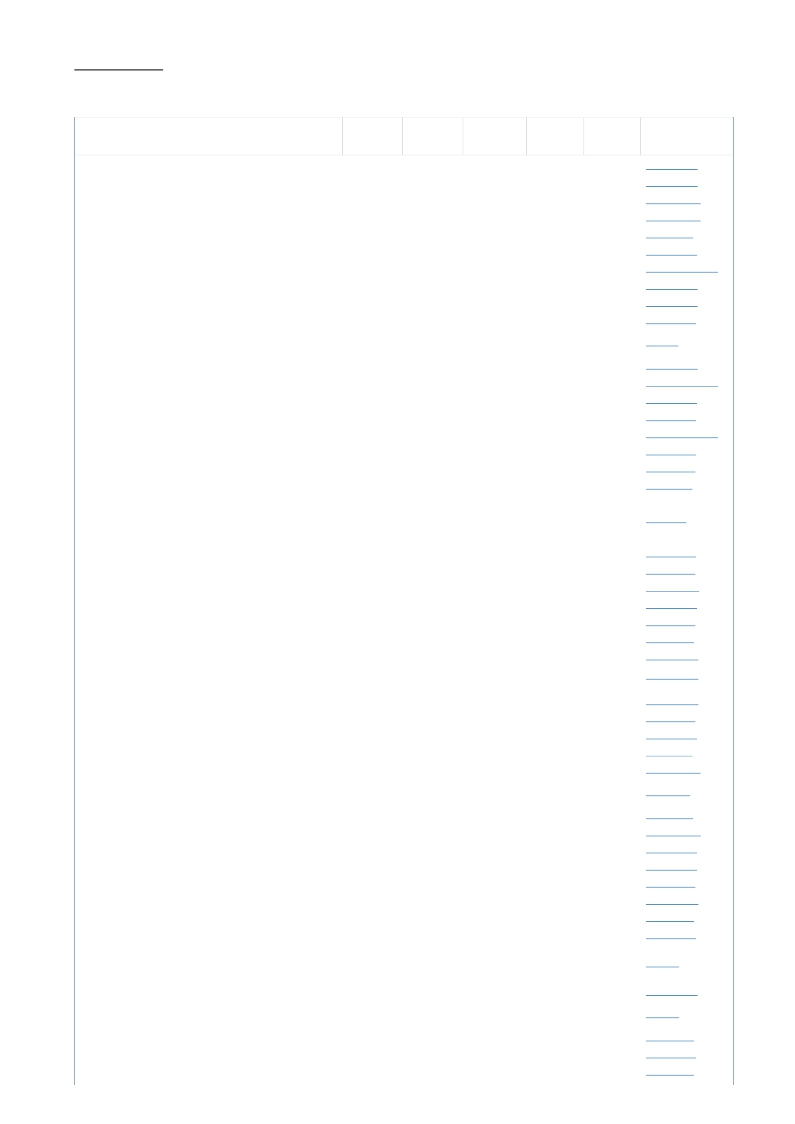


2017/4/9

NCBI Blast:GL19134-R1_1

laccase [Coriolopsis trogii]

laccase [Gelatoporia subvermispora B]

laccase [Coriolopsis gallica]

laccase precursor [Obba rivulosa]

laccase 1A [Trametes pubescens]

laccase [basidiomycete PM1]

Chain A, Coriolopsis Gallica Laccase T2 Copper Depleted At

Ph 4.5

Chain A, Coriolopsis Gallica Laccase Collected At 12.65 Kev

laccase [Coriolopsis rigida]

laccase [Coriolopsis rigida]

laccase [Rigidoporus microporus]

Laccase [Grifola frondosa]

phenol oxidase [Pleurotus ostreatus]

laccase [Dichomitus squalens LYAD-421 SS1]

laccase 2 precursor [Spongipellis sp. FERM P-18171]

laccase F [Trametes hirsuta]

laccase LCC3-2 [Polyporus ciliatus]

627

626

626

626

626

626

625

625

625

625

625

625

625

625

625

624

623

627

626

626

626

626

626

625

625

625

625

625

625

625

625

625

624

623

85%

85%

85%

84%

86%

85%

81%

81%

82%

82%

85%

84%

83%

86%

84%

83%

85%

0.0

0.0

0.0

0.0

0.0

0.0

0.0

0.0

0.0

0.0

0.0

0.0

0.0

0.0

0.0

0.0

0.0

59%

60%

59%

60%

58%

60%

60%

60%

59%

59%

59%

59%

59%

60%

60%

60%

59%

[CAC13040.1](https://www.ncbi.nlm.nih.gov/protein/10801036?report=genbank&log$=prottop&blast_rank=49&RID=EMDGFCVG01R)

[EMD32460.1](https://www.ncbi.nlm.nih.gov/protein/449541476?report=genbank&log$=prottop&blast_rank=50&RID=EMDGFCVG01R)

[ACS26245.1](https://www.ncbi.nlm.nih.gov/protein/239809556?report=genbank&log$=prottop&blast_rank=51&RID=EMDGFCVG01R)

[OCH92420.1](https://www.ncbi.nlm.nih.gov/protein/1045915344?report=genbank&log$=prottop&blast_rank=52&RID=EMDGFCVG01R)

[AAM18408.1](https://www.ncbi.nlm.nih.gov/protein/20270772?report=genbank&log$=prottop&blast_rank=53&RID=EMDGFCVG01R)

[CAA78144.1](https://www.ncbi.nlm.nih.gov/protein/58324?report=genbank&log$=prottop&blast_rank=54&RID=EMDGFCVG01R)

[4A2D_A](https://www.ncbi.nlm.nih.gov/protein/350610907?report=genbank&log$=prottop&blast_rank=55&RID=EMDGFCVG01R)

[4A2F_A](https://www.ncbi.nlm.nih.gov/protein/385251975?report=genbank&log$=prottop&blast_rank=56&RID=EMDGFCVG01R)

[ADK13098.1](https://www.ncbi.nlm.nih.gov/protein/300433312?report=genbank&log$=prottop&blast_rank=57&RID=EMDGFCVG01R)

[ACU29545.1](https://www.ncbi.nlm.nih.gov/protein/255918284?report=genbank&log$=prottop&blast_rank=58&RID=EMDGFCVG01R)

[AAO38869.1](https://www.ncbi.nlm.nih.gov/protein/37359391?report=genbank&log$=prottop&blast_rank=59&RID=EMDGFCVG01R)

[OBZ73717.1](https://www.ncbi.nlm.nih.gov/protein/1043286487?report=genbank&log$=prottop&blast_rank=60&RID=EMDGFCVG01R)

[CAR48257.1](https://www.ncbi.nlm.nih.gov/protein/198281884?report=genbank&log$=prottop&blast_rank=61&RID=EMDGFCVG01R)

[XP_007364547.1](https://www.ncbi.nlm.nih.gov/protein/597985483?report=genbank&log$=prottop&blast_rank=62&RID=EMDGFCVG01R)

[BAE96003.1](https://www.ncbi.nlm.nih.gov/protein/108936945?report=genbank&log$=prottop&blast_rank=63&RID=EMDGFCVG01R)

[AIZ72725.1](https://www.ncbi.nlm.nih.gov/protein/732554704?report=genbank&log$=prottop&blast_rank=64&RID=EMDGFCVG01R)

[AAG09230.1](https://www.ncbi.nlm.nih.gov/protein/9957145?report=genbank&log$=prottop&blast_rank=65&RID=EMDGFCVG01R)

RecName: Full=Laccase-4; AltName: Full=Benzenediol:oxygen

oxidoreductase 4; AltName: Full=Diphenol oxidase 4; AltName:

623

623

82%

0.0

60%

[Q12719.1](https://www.ncbi.nlm.nih.gov/protein/2833234?report=genbank&log$=prottop&blast_rank=66&RID=EMDGFCVG01R)

Full=Urishiol oxidase 4; Flags: Precursor

laccase [Cerrena sp. WR1]

laccase 1 precursor [Cerrena sp. HYB07]

laccase-4 [Trametes versicolor FP-101664 SS1]

laccase B [Trametes ochracea]

hypothetical protein PLICRDRAFT_30715 [Plicaturopsis crispa

FD-325 SS-3]

laccase 1 [Ceriporiopsis rivulosa]

laccase [Rigidoporus microporus]

laccase [Echinodontium taxodii]

OB1 laccase [synthetic construct]

laccase [Pleurotus salmoneostramineus]

laccase 2 [Obba rivulosa]

Chain A, Crystal Structure Of Lacb From Trametes Sp. Ah28-

2

laccase [Trametes versicolor FP-101664 SS1]

Chain A, Crystal Structure Of Blue Laccase From Trametes

Trogii Complexed With P-Methylbenzoate

623

623

623

622

622

622

622

622

622

622

621

621

621

621

623

623

623

622

622

622

622

622

622

622

621

621

621

621

86%

83%

82%

83%

83%

84%

85%

84%

82%

83%

83%

81%

86%

81%

0.0

0.0

0.0

0.0

0.0

0.0

0.0

0.0

0.0

0.0

0.0

0.0

0.0

0.0

59%

60%

60%

60%

58%

59%

59%

60%

60%

59%

61%

60%

59%

60%

[ACZ58368.1](https://www.ncbi.nlm.nih.gov/protein/270047922?report=genbank&log$=prottop&blast_rank=67&RID=EMDGFCVG01R)

[AGK89726.1](https://www.ncbi.nlm.nih.gov/protein/485993048?report=genbank&log$=prottop&blast_rank=68&RID=EMDGFCVG01R)

[XP_008035965.1](https://www.ncbi.nlm.nih.gov/protein/636609661?report=genbank&log$=prottop&blast_rank=69&RID=EMDGFCVG01R)

[ALT22025.1](https://www.ncbi.nlm.nih.gov/protein/965871806?report=genbank&log$=prottop&blast_rank=70&RID=EMDGFCVG01R)

[KII87344.1](https://www.ncbi.nlm.nih.gov/protein/749762560?report=genbank&log$=prottop&blast_rank=71&RID=EMDGFCVG01R)

[AFI57924.1](https://www.ncbi.nlm.nih.gov/protein/385282687?report=genbank&log$=prottop&blast_rank=72&RID=EMDGFCVG01R)

[AAQ82021.1](https://www.ncbi.nlm.nih.gov/protein/34761694?report=genbank&log$=prottop&blast_rank=73&RID=EMDGFCVG01R)

[AGT45944.1](https://www.ncbi.nlm.nih.gov/protein/530746508?report=genbank&log$=prottop&blast_rank=74&RID=EMDGFCVG01R)

[ALI16920.1](https://www.ncbi.nlm.nih.gov/protein/937376448?report=genbank&log$=prottop&blast_rank=75&RID=EMDGFCVG01R)

[BAI66145.1](https://www.ncbi.nlm.nih.gov/protein/283379470?report=genbank&log$=prottop&blast_rank=76&RID=EMDGFCVG01R)

[OCH85821.1](https://www.ncbi.nlm.nih.gov/protein/1045908553?report=genbank&log$=prottop&blast_rank=77&RID=EMDGFCVG01R)

[3KW7_A](https://www.ncbi.nlm.nih.gov/protein/290790140?report=genbank&log$=prottop&blast_rank=78&RID=EMDGFCVG01R)

[XP_008035966.1](https://www.ncbi.nlm.nih.gov/protein/636609663?report=genbank&log$=prottop&blast_rank=79&RID=EMDGFCVG01R)

[2HRG_A](https://www.ncbi.nlm.nih.gov/protein/158428663?report=genbank&log$=prottop&blast_rank=80&RID=EMDGFCVG01R)

RecName: Full=Laccase-4; AltName: Full=Benzenediol:oxygen

oxidoreductase 4; AltName: Full=Diphenol oxidase 4; AltName:

620

620

82%

0.0

60%

[Q99055.1](https://www.ncbi.nlm.nih.gov/protein/2842755?report=genbank&log$=prottop&blast_rank=81&RID=EMDGFCVG01R)

Full=Urishiol oxidase 4; Flags: Precursor

laccase E [Trametes ochracea]

laccase [Piloderma croceum F 1598]

Chain A, Crystal Structure Of Laccase From Basidiomycete

Pm1 (cect 2971)

laccase [Coriolopsis gallica]

Chain A, Crystal Structure Of Laccase From Lentinus Sp. At 1.8

A Resolution

laccase 1 precursor [Spongipellis sp. FERM P-18171]

laccase 2 [Coriolopsis caperata]

laccase 2 [Obba rivulosa]

619

619

619

619

619

619

619

618

619

619

619

619

619

619

619

618

86%

81%

81%

82%

84%

83%

83%

84%

0.0

0.0

0.0

0.0

0.0

0.0

0.0

0.0

58%

61%

61%

60%

59%

60%

59%

61%

[ALT22028.1](https://www.ncbi.nlm.nih.gov/protein/965871910?report=genbank&log$=prottop&blast_rank=82&RID=EMDGFCVG01R)

[KIM72670.1](https://www.ncbi.nlm.nih.gov/protein/751724254?report=genbank&log$=prottop&blast_rank=83&RID=EMDGFCVG01R)

[5ANH_A](https://www.ncbi.nlm.nih.gov/protein/1059270720?report=genbank&log$=prottop&blast_rank=84&RID=EMDGFCVG01R)

[AAW65489.1](https://www.ncbi.nlm.nih.gov/protein/58176544?report=genbank&log$=prottop&blast_rank=85&RID=EMDGFCVG01R)

[3X1B_A](https://www.ncbi.nlm.nih.gov/protein/731187736?report=genbank&log$=prottop&blast_rank=86&RID=EMDGFCVG01R)

[BAE79811.1](https://www.ncbi.nlm.nih.gov/protein/88687733?report=genbank&log$=prottop&blast_rank=87&RID=EMDGFCVG01R)

[AGE13770.1](https://www.ncbi.nlm.nih.gov/protein/445065172?report=genbank&log$=prottop&blast_rank=88&RID=EMDGFCVG01R)

[OCH87174.1](https://www.ncbi.nlm.nih.gov/protein/1045909971?report=genbank&log$=prottop&blast_rank=89&RID=EMDGFCVG01R)

RecName: Full=Laccase-2; AltName: Full=Benzenediol:oxygen

oxidoreductase 2; AltName: Full=Diphenol oxidase 2; AltName:

Full=Laccase I; AltName: Full=Urishiol oxidase 2; Flags:

618

618

83%

0.0

59%

[Q12718.1](https://www.ncbi.nlm.nih.gov/protein/2833233?report=genbank&log$=prottop&blast_rank=90&RID=EMDGFCVG01R)

Precursor

phenoloxidase [Obba rivulosa]

laccase I [Trametes versicolor]

laccase I [Trametes versicolor FP-101664 SS1]

laccase 2 [Trametes pubescens]

laccase A [Pleurotus eryngii var. ferulae]

laccase [Trametes sp. I-62]

laccase [Trametes sp. I-62]

hypothetical protein PLICRDRAFT_115284 [Plicaturopsis

crispa FD-325 SS-3]

618

618

618

618

617

617

617

617

618

618

618

618

617

617

617

617

84%

83%

83%

83%

84%

83%

83%

83%

0.0

0.0

0.0

0.0

0.0

0.0

0.0

0.0

60%

59%

59%

59%

59%

60%

60%

58%

[OCH90801.1](https://www.ncbi.nlm.nih.gov/protein/1045913697?report=genbank&log$=prottop&blast_rank=91&RID=EMDGFCVG01R)

[AAC49828.1](https://www.ncbi.nlm.nih.gov/protein/1172163?report=genbank&log$=prottop&blast_rank=92&RID=EMDGFCVG01R)

[XP_008032737.1](https://www.ncbi.nlm.nih.gov/protein/636603205?report=genbank&log$=prottop&blast_rank=93&RID=EMDGFCVG01R)

[AAM18407.1](https://www.ncbi.nlm.nih.gov/protein/20270770?report=genbank&log$=prottop&blast_rank=94&RID=EMDGFCVG01R)

[AKE48164.1](https://www.ncbi.nlm.nih.gov/protein/815749743?report=genbank&log$=prottop&blast_rank=95&RID=EMDGFCVG01R)

[AAQ12268.1](https://www.ncbi.nlm.nih.gov/protein/33334369?report=genbank&log$=prottop&blast_rank=96&RID=EMDGFCVG01R)

[AAQ12267.1](https://www.ncbi.nlm.nih.gov/protein/33334367?report=genbank&log$=prottop&blast_rank=97&RID=EMDGFCVG01R)

[KII86082.1](https://www.ncbi.nlm.nih.gov/protein/749761180?report=genbank&log$=prottop&blast_rank=98&RID=EMDGFCVG01R)

RecName: Full=Laccase-5; AltName: Full=Benzenediol:oxygen

oxidoreductase 5; AltName: Full=Diphenol oxidase 5; AltName:

617

617

86%

0.0

58%

[Q99056.2](https://www.ncbi.nlm.nih.gov/protein/18281739?report=genbank&log$=prottop&blast_rank=99&RID=EMDGFCVG01R)

https://blast.ncbi.nlm.nih.gov/Blast.cgi

3/6


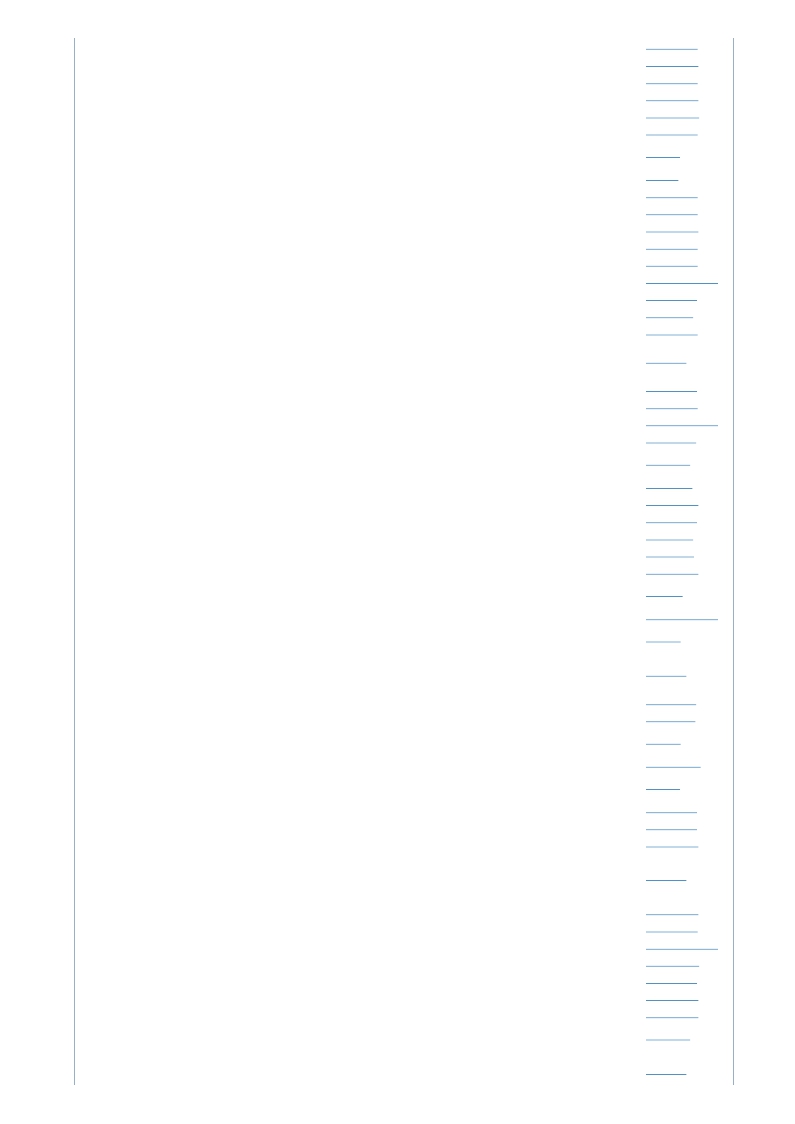


2017/4/9

Full=Urishiol oxidase 5; Flags: Precursor

NCBI Blast:GL19134-R1_1

laccse sensu stricto [Heterobasidion irregulare TC 32-1]

617

617

83%

0.0

59%

[XP_009543177.1](https://www.ncbi.nlm.nih.gov/protein/695541065?report=genbank&log$=prottop&blast_rank=100&RID=EMDGFCVG01R)

Alignments

laccase, partial [Ganoderma lucidum]

Sequence ID: AHA83590.1 Length: 529 Number of Matches: 1

Range 1: 1 to 529

Score

Expect Method

Identities

Positives

Gaps

Frame

1098 bits(2840) 0.0()

Features:

Compositional matrix adjust. 529/529(100%) 529/529(100%) 0/529(0%)

Query 1

Sbjct 1

Query 61

Sbjct 61

MAKGNSFIRVAFVLLASLARRTSAQIGPVADLRIVNKVIAPDGFSRNAVLAGGTFPGPLI 60

MAKGNSFIRVAFVLLASLARRTSAQIGPVADLRIVNKVIAPDGFSRNAVLAGGTFPGPLI

MAKGNSFIRVAFVLLASLARRTSAQIGPVADLRIVNKVIAPDGFSRNAVLAGGTFPGPLI 60

SGHSGDTFKINVKDELTDSSMLTPTSIHWHGILQHETNWADGGAFVSQCPITTGNSFRYK 120

SGHSGDTFKINVKDELTDSSMLTPTSIHWHGILQHETNWADGGAFVSQCPITTGNSFRYK

SGHSGDTFKINVKDELTDSSMLTPTSIHWHGILQHETNWADGGAFVSQCPITTGNSFRYK 120

Query 121 FDTKGIAGTYWYHSHLASQYCDGLRGPLILYDKNDPHKDLYDVDDESTVITLADWYHVIA 180

FDTKGIAGTYWYHSHLASQYCDGLRGPLILYDKNDPHKDLYDVDDESTVITLADWYHVIA

Sbjct 121 FDTKGIAGTYWYHSHLASQYCDGLRGPLILYDKNDPHKDLYDVDDESTVITLADWYHVIA 180

Query 181 PLLPARAVVPASDSTLINGLGRWFANPTTELAVIKVTQGKRYRFRMVSTACHANYNFTIA 240

PLLPARAVVPASDSTLINGLGRWFANPTTELAVIKVTQGKRYRFRMVSTACHANYNFTIA

Sbjct 181 PLLPARAVVPASDSTLINGLGRWFANPTTELAVIKVTQGKRYRFRMVSTACHANYNFTIA 240

Query 241 GHDLTVIEADGQNTKPLTVDEIQIFAGQRYSFVLEANRPIDNYWIHALPNLMYNTSALGT 300

GHDLTVIEADGQNTKPLTVDEIQIFAGQRYSFVLEANRPIDNYWIHALPNLMYNTSALGT

Sbjct 241 GHDLTVIEADGQNTKPLTVDEIQIFAGQRYSFVLEANRPIDNYWIHALPNLMYNTSALGT 300

Query 301 ANGINSAILRYEGAPEEEPRHLEVKSLNPLREWNLRPLEDPAAPGEPHPGGVDIVYNLDV 360

ANGINSAILRYEGAPEEEPRHLEVKSLNPLREWNLRPLEDPAAPGEPHPGGVDIVYNLDV

Sbjct 301 ANGINSAILRYEGAPEEEPRHLEVKSLNPLREWNLRPLEDPAAPGEPHPGGVDIVYNLDV 360

Query 361 GFRPRNNTSPTRFTINNVTFEPPSIPVLLQILGGVQLAQDLLPQGSIYPLRRNATVELTI 420

GFRPRNNTSPTRFTINNVTFEPPSIPVLLQILGGVQLAQDLLPQGSIYPLRRNATVELTI

Sbjct 361 GFRPRNNTSPTRFTINNVTFEPPSIPVLLQILGGVQLAQDLLPQGSIYPLRRNATVELTI 420

Query 421 PGTLVGGPHPFHLHGHTFSVVRSAGQAVPNYVDPVKRDVVSVGVPGDNVTIRFTADNPGP 480

PGTLVGGPHPFHLHGHTFSVVRSAGQAVPNYVDPVKRDVVSVGVPGDNVTIRFTADNPGP

Sbjct 421 PGTLVGGPHPFHLHGHTFSVVRSAGQAVPNYVDPVKRDVVSVGVPGDNVTIRFTADNPGP 480

Query 481 WFLHCHIDWHLEAGLAIVFAEDVPSVSFVDPAPEEWYDLCPEYEESLAR 529

WFLHCHIDWHLEAGLAIVFAEDVPSVSFVDPAPEEWYDLCPEYEESLAR

Sbjct 481 WFLHCHIDWHLEAGLAIVFAEDVPSVSFVDPAPEEWYDLCPEYEESLAR 529

laccase, partial [Ganoderma lucidum]

Sequence ID: AHA83587.1 Length: 535 Number of Matches: 1

Range 1: 1 to 527

Score

Expect Method

Identities

Positives

Gaps

Frame

782 bits(2019) 0.0()

Features:

Compositional matrix adjust.

366/530(69%) 428/530(80%) 3/530(0%)

Query 1

Sbjct 1

Query 61

Sbjct 61

MAKGNSFIRVAFVLLASLARRTSAQIGPVADLRIVNKVIAPDGFSRNAVLAGGTFPGPLI 60

M GNS V ++LA++A+TAIGPVDL+VNK++DGSRNAVLAGTFPGP+I

MVMGNSCTAVVALILATIAQPTRAAIGPVTDLAVVNKPVSTDGTSRNAVLADGTFPGPVI 60

SGHSGDTFKINVKDELTDSSMLTPTSIHWHGILQHETNWADGGAFVSQCPITTGNSFRYK 120

+G+SGDFINVKDEL+++MLTTSIHWHG+LQHTNWADG+F++QCPIT+GNFY+

AGYSGDYFNINVKDELYNTTMLTGTSIHWHGLLQHTTNWADGASFINQCPITSGNCFEYE 120

Query 121 FDTKGIAGTYWYHSHLASQYCDGLRGPLILYDKNDPHKDLYDVDDESTVITLADWYHVIA 180

F+TGI+GTYWYHSHL+QYCDGLRGPL+LYDK+DPH LYDVDD T+ITLADWYH++

Sbjct 121 FETTGISGTYWYHSHLGNQYCDGLRGPLVLYDKHDPHAHLYDVDDGITIITLADWYHLAS 180

Query 181 PLLPARAVVPASDSTLINGLGRWFANPTTELAVIKVTQGKRYRFRMVSTACHANYNFTIA 240

P+ R P+DSLINGLGRW NPTELAVIKVTGKRYRFR+++AC NYNFTIA

Sbjct 181 PFVLTRGGPPRADSNLINGLGRWAGNPTAELAVIKVTHGKRYRFRLINIACDPNYNFTIA 240

Query 241 GHDLTVIEADGQNTKPLTVDEIQIFAGQRYSFVLEANRPIDNYWIHALPNLMYNTSALGT 300

GH+T+IEADGQN++PLVDE+QIF QRYSFVLEAN+P++NYWIALP++M S+LG

Sbjct 241 GHSMTIIEADGQNSEPLVVDELQIFVAQRYSFVLEANQPVNNYWIRALPDIMAENSSLGY 300

Query 301 ANGINSAILRYEGAPEEEPRHLEVKSLNPLREWNLRPLEDPAAPGEPHPGGVDIVYNLDV 360

A+GINSAILRYEGAPEEP EKS+NPLRE+NL LEDPAAPGEP+GGVD NL+

Sbjct 301 AHGINSAILRYEGAPAEEPCEQEAKSVNPLREYNLHSLEDPAAPGEPYVGGVDYALNLVL 360

Query 361 GFRPRNNTSPTRFTINNVTFEPPSIPVLLQILGGVQLAQDLLPQGSIYPLRRNATVELTI 420

GF NNSTFINVF+PS+PVLLQILG+AQDLLPGS+YLRN+++EL+I

Sbjct 361 GF---NNASKTPFNINGVPFQSPSVPVLLQILSGAKKAQDLLPAGSVYGLPRNSSIELSI 417

Query 421 PGTLVGGPHPFHLHGHTFSVVRSAGQAVPNYVDPVKRDVVSVGVPGDNVTIRFTADNPGP 480

+GGPHPFHLHGHFSVVRAGQAVPNY+P+KRDVVSGPGDNVTIRF DNPGP

Sbjct 418 QPLSIGGPHPFHLHGHAFSVVRGAGQAVPNYANPIKRDVVSTGFPGDNVTIRFRTDNPGP 477

Query 481 WFLHCHIDWHLEAGLAIVFAEDVPSVSFVDPAPEEWYDLCPEYEESLARD 530

WLHCHIDWHL GLAIVFAEDV SFVDPAP+EWYDLCPEYES+AD

Sbjct 478 WLLHCHIDWHLSGGLAIVFAEDVGDTSFVDPAPKEWYDLCPEYEASIAND 527

laccase C [Trametes sp. 420]

Sequence ID: AAW28938.1 Length: 519 Number of Matches: 1

Range 1: 1 to 517

Score

Expect Method

Identities

Positives

Gaps

Frame

724 bits(1868) 0.0()

Compositional matrix adjust. 350/527(66%) 409/527(77%) 10/527(1%)

Features:

https://blast.ncbi.nlm.nih.gov/Blast.cgi

4/6


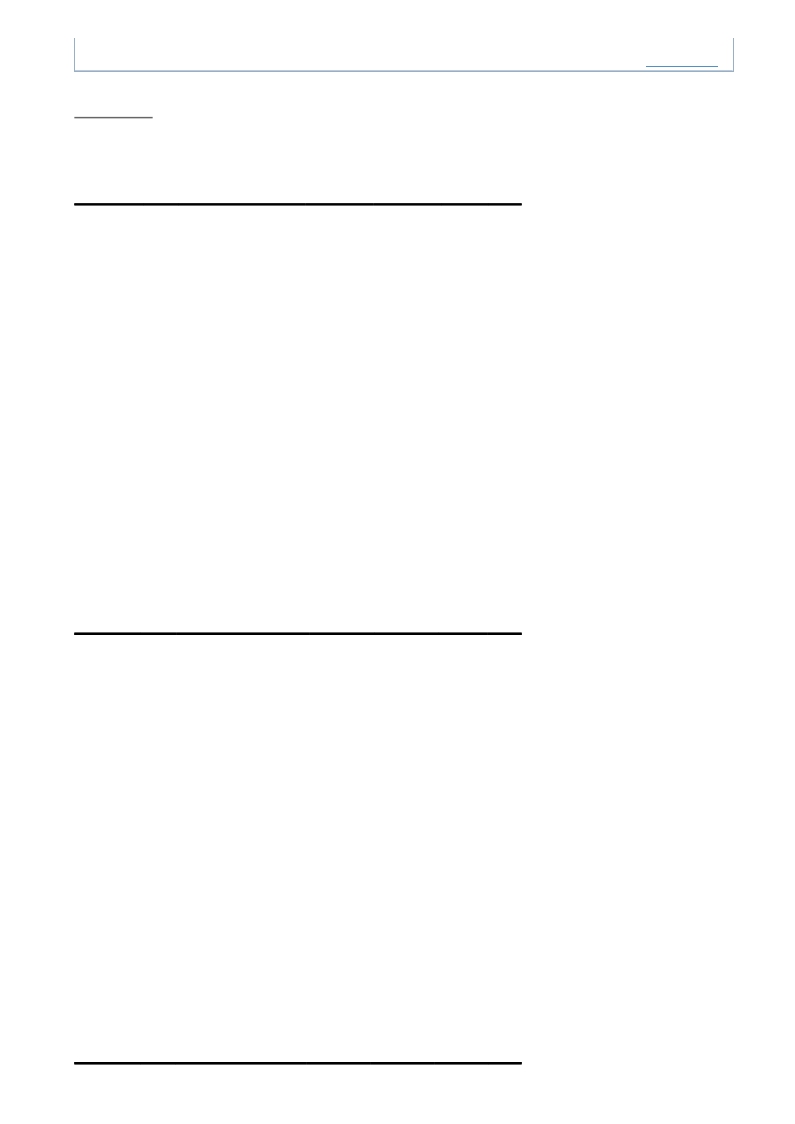


2017/4/9

Features:

NCBI Blast:GL19134-R1_1

Query 1

Sbjct 1

Query 61

Sbjct 61

MAKGNSFIRVAFVLLASLARRTSAQIGPVADLRIVNKVIAPDGFSRNAVLAGGTFPGPLI 60

M+ SFR+++L++LAR+AIGPVDLIVN I+PDGF+R+AVLAGTFPGPLI

MLQLTSFRRLSLLLVTALATRSFAAIGPVTDLNIVNANISPDGFARDAVLADGTFPGPLI 60

SGHSGDTFKINVKDELTDSSMLTPTSIHWHGILQHETNWADGGAFVSQCPITTGNSFRYK 120

+G DF+INVD+LT+++MLTTSIHWHG QHTNWADG F++QCPI+GNSFY

TGQKNDNFQINVVDKLTNTTMLTATSIHWHGFFQHGTNWADGVPFLNQCPIVSGNSFLYN 120

Query 121 FDTKGIAGTYWYHSHLASQYCDGLRGPLILYDKNDPHKDLYDVDDESTVITLADWYHVIA 180

F+ AGT+WYHSHL+QYCDGLRGL++YD DPH LYDVDDESTVITLADWYHVA

Sbjct 121 FNVPDQAGTFWYHSHLNTQYCDGLRGALVVYDPADPHAGLYDVDDESTVITLADWYHVAA 180

Query 181 PLLPARAVVPASDSTLINGLGRWFANPTTELAVIKVTQGKRYRFRMVSTACHANYNFTIA 240

PL A VPSD+TLINGLGRW +PT+ELAVIVTGKRYRFR+VS+C NYFI

Sbjct 181 PLAGA---VPRSDATLINGLGRWSGDPTSELAVINVTPGKRYRFRLVSMSCDPNYTFQID 237

Query 241 GHDLTVIEADGQNTKPLTVDEIQIFAGQRYSFVLEANRPIDNYWIHALPNLMYNTSALGT 300

GH+TVIEADGQNT+PLVD+IQIFAGQRYSFVLEAN++NYWIAPNL++ +G

Sbjct 238 GHSMTVIEADGQNTEPLPVDQIQIFAGQRYSFVLEANQTVGNYWIRASPNLVADGGS-GF 296

Query 301 ANGINSAILRYEGAPEEEPRHLEVKSLNPLREWNLRPLEDPAAPGEPHPGGVDIVYNLDV 360

A+GINSAILRY+GAPEEEP + S+NPLRE++LPLDPAAPGP GV++ NL+

Sbjct 297 AHGINSAILRYDGAPEEEPTTTQDTSINPLREFDLHPLTDPAAPGNPTEGDVEVPINLAI 356

Query 361 GFRPRNNTSPTRFTINNVTFEPPSIPVLLQILGGVQLAQDLLPQGSIYPLRRNATVELTI 420

GF N FT+N TFEPS+PVLLQILGQAQDLLPGS+YL NATVEL+I

Sbjct 357 GFSGGN------FTVNGTTFESPSVPVLLQILSGAQNAQDLLPSGSVYSLPSNATVELSI 410

Query 421 PGTLVGGPHPFHLHGHTFSVVRSAGQAVPNYVDPVKRDVVSVGVPGDNVTIRFTADNPGP 480

P +GGPHPFHLHGHFSVVRSAGQ PNYV+PV+RDVVS+G DNVTIRF DNPGP

Sbjct 411 PAFAIGGPHPFHLHGHAFSVVRSAGQTEPNYVNPVRRDVVSIGSGTDNVTIRFRTDNPGP 470

Query 481 WFLHCHIDWHLEAGLAIVFAEDVPSVSFVDPAPEEWYDLCPEYEESL 527

WFLHCHIDWHL+AGLAIVFAED+P + +PP+WDLCPEYESL

Sbjct 471 WFLHCHIDWHLQAGLAIVFAEDIPETAATNPVPQAWSDLCPEYEASL 517

laccase B [Trametes sp. 420]

Sequence ID: AAW28937.1 Length: 519 Number of Matches: 1

Range 1: 1 to 517

Score

Expect Method

Identities

Positives

Gaps

Frame

719 bits(1855) 0.0()

Features:

Compositional matrix adjust. 349/527(66%) 404/527(76%) 10/527(1%)

Query 1

Sbjct 1

Query 61

Sbjct 61

MAKGNSFIRVAFVLLASLARRTSAQIGPVADLRIVNKVIAPDGFSRNAVLAGGTFPGPLI 60

M+ SFR++ L +LARTAIGPVDLIVN I+PDGF+R+AVLAGTFPGPLI

MLQLTSFRRLSLFLFTALATRTFAAIGPVTDLNIVNANISPDGFARDAVLAEGTFPGPLI 60

SGHSGDTFKINVKDELTDSSMLTPTSIHWHGILQHETNWADGGAFVSQCPITTGNSFRYK 120

+G DF+INVD+LT+++MLTTSIHWHG QHTNWADG F++QCPI+GNSFY

TGQKNDNFRINVVDKLTNTTMLTATSIHWHGFFQHGTNWADGVPFLNQCPIVSGNSFLYN 120

Query 121 FDTKGIAGTYWYHSHLASQYCDGLRGPLILYDKNDPHKDLYDVDDESTVITLADWYHVIA 180

F+ AGT+WYHSHL+QYCDGLRGL++YD DPH LYDVD+ESTVITLADWYHVA

Sbjct 121 FNVPDQAGTFWYHSHLNTQYCDGLRGALVVYDPADPHASLYDVDEESTVITLADWYHVAA 180

Query 181 PLLPARAVVPASDSTLINGLGRWFANPTTELAVIKVTQGKRYRFRMVSTACHANYNFTIA 240

PL A VPSD+TLINGLGRW +PT+ELAIVTGKRYRFR+VS+C NYFI

Sbjct 181 PLAGA---VPRSDATLINGLGRWSGDPTSELAAINVTPGKRYRFRLVSMSCDPNYTFQID 237

Query 241 GHDLTVIEADGQNTKPLTVDEIQIFAGQRYSFVLEANRPIDNYWIHALPNLMYNTSALGT 300

GH+TVIEADGQNT+PLVD+IQIFAGQRYSFVLEAN++NYWIAPNL++ +G

Sbjct 238 GHSMTVIEADGQNTEPLPVDQIQIFAGQRYSFVLEANQTVGNYWIRASPNLVADGGS-GF 296

Query 301 ANGINSAILRYEGAPEEEPRHLEVKSLNPLREWNLRPLEDPAAPGEPHPGGVDIVYNLDV 360

ANGINSAILR+GAPEEEP + S+NPLRE++LPLDPAAPGP GV+ NL+

Sbjct 297 ANGINSAILRCDGAPEEEPTTTQDTSINPLREFDLHPLTDPAAPGNPTEGDVGVPINLAI 356

Query 361 GFRPRNNTSPTRFTINNVTFEPPSIPVLLQILGGVQLAQDLLPQGSIYPLRRNATVELTI 420

GF N FT+N TFEPS+PVLLQILGQAQDLLPGS+YL NATVEL+I

Sbjct 357 GFSGGN------FTVNGTTFESPSVPVLLQILSGAQNAQDLLPSGSVYSLPSNATVELSI 410

Query 421 PGTLVGGPHPFHLHGHTFSVVRSAGQAVPNYVDPVKRDVVSVGVPGDNVTIRFTADNPGP 480

P +GGPHPFHLHGHFSVVRSAGQ PNYV+PV+RDVVS+G DNVTIRF DNPGP

Sbjct 411 PAFAIGGPHPFHLHGHAFSVVRSAGQTEPNYVNPVRRDVVSIGSGTDNVTIRFRTDNPGP 470

Query 481 WFLHCHIDWHLEAGLAIVFAEDVPSVSFVDPAPEEWYDLCPEYEESL 527

WFLHCHIDWHL+AGLAIVFAED+P + +PP+WDLCPEYESL

Sbjct 471 WFLHCHIDWHLQAGLAIVFAEDIPETAATNPVPQAWSDLCPEYEASL 517

laccase D [Trametes hirsuta]

Sequence ID: AIZ72723.1 Length: 523 Number of Matches: 1

Range 1: 7 to 519

Score

Expect Method

Identities

Positives

Gaps

Frame

676 bits(1745) 0.0()

Features:

Compositional matrix adjust. 327/521(63%) 389/521(74%) 10/521(1%)

Query 9

Sbjct 7

Query 69

Sbjct 66

RVAFVLLASLARRTSAQIGPVADLRIVNKVIAPDGFSRNAVLAGGTFPGPLISGHSGDTF 68

RA +L L AIGPVDLIVNKIAPDG R+VLAGGTFPGPL+G GDF

RTAVTILG-LCGAAMAAIGPVTDLDIVNKEIAPDGLLRDTVLAGGTFPGPLVQGKKGDHF 65

KINVKDELTDSSMLTPTSIHWHGILQHETNWADGGAFVSQCPITTGNSFRYKFDTKGIAG 128

KINVD+LT++MLTT+IHWHG+QHTNWADGAFV+QCPI GNFYF G

KINVVDKLTNETMLTSTTIHWHGLFQHTTNWADGPAFVTQCPIIAGNDFLYNFQVPDQTG 125

Query 129 TYWYHSHLASQYCDGLRGPLILYDKNDPHKDLYDVDDESTVITLADWYHVIAPLLPARAV 188

TYWYHSHLA+QYCDGLRGPL++YD+DPHKLYDVDDESTVITLADWYH AP+

Sbjct 126 TYWYHSHLATQYCDGLRGPLVIYDPHDPHKHLYDVDDESTVITLADWYHTAAPIEANGPG 185

Query 189 VPASDSTLINGLGRWFANPTTELAVIKVTQGKRYRFRMVSTACHANYNFTIAGHDLTVIE 248

+PSDSTLINGLGRW NPT+ELAVIV GKRYRFR+++AC YNFTIGH++T+IE

Sbjct 186 IPTSDSTLINGLGRWAGNPTSELAVINVEHGKRYRFRLINIACDPRYNFTIDGHNMTIIE 245

Query 249 ADGQNTKPLTVDEIQIFAGQRYSFVLEANRPIDNYWIHALPNLMYNTSALGTANGINSAI 308

ADG+NT+PLVD+I+I QRYSF+LEAN+P++NYWIAP+ N+AGANGINSAI

Sbjct 246 ADGENTQPLKVDKIEILVAQRYSFILEANQPVNNYWIRAQPDRQ-NLNATGFANGINSAI 304

Query 309 LRYEGAPEEEPRHLEVKSLNPLREWNLRPLEDPAAPGEPHPGGVDIVYNLDVGFRPRNNT 368

https://blast.ncbi.nlm.nih.gov/Blast.cgi

5/6


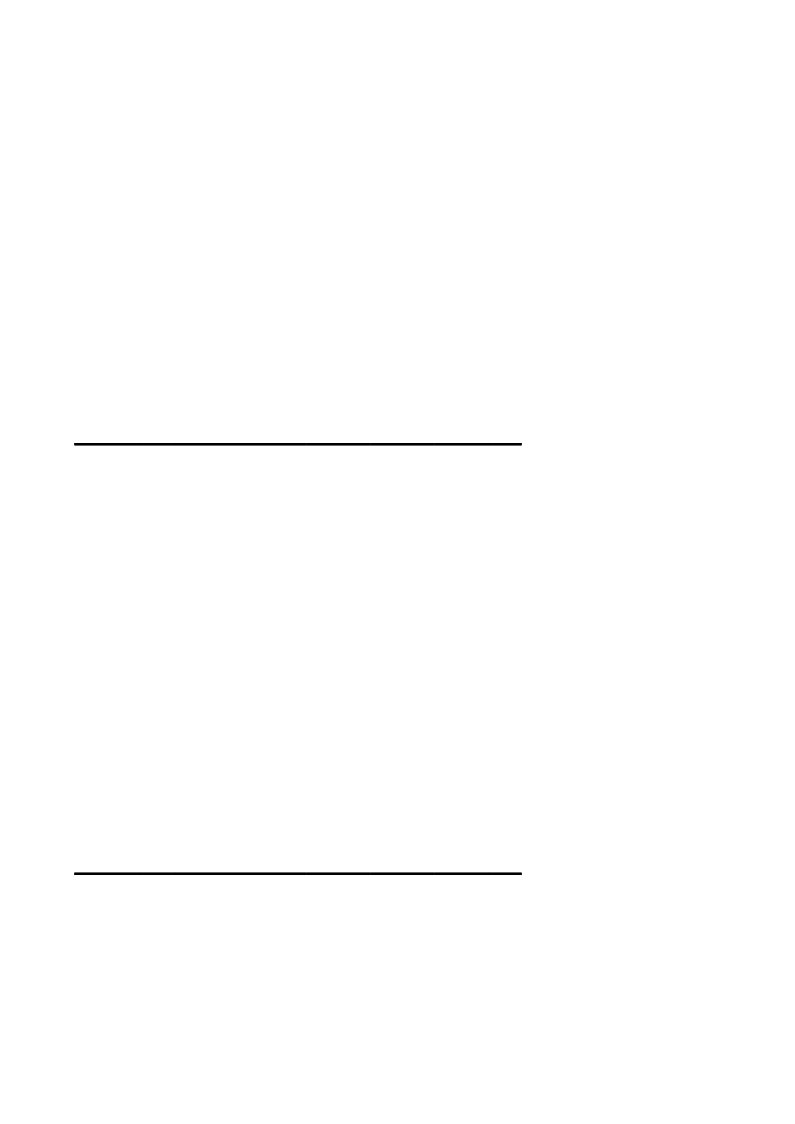


2017/4/9

NCBI Blast:GL19134-R1_1

Query 309 LRYEGAPEEEPRHLEVKSLNPLREWNLRPLEDPAAPGEPHPGGVDIVYNLDVGFRPRNNT 368

LRY+GAP EP + S+ LRE+LPL+PAAPGP+GGVD NLD+F

Sbjct 305 LRYKGAPIAEPNTTQSPSVAFLRETDLHPLTNPAAPGHPYKGGVDYALNLDLNF------ 358

Query 369 SPTRFTINNVTFEPPSIPVLLQILGGVQLAQDLLPQGSIYPLRRNATVELTI-PGTLVGG 427

+ FINNV+F+PP++PVLLQILG ADL+PGS+YL +T+EL+IPG GG

Sbjct 359 TKGEFLINNVSFKPPTVPVLLQILNGTLDAHDLMPHGSVYSLPPYSTIELSIPPGAAPGG 418

Query 428 PHPFHLHGHTFSVVRSAGQAVPNYVDPVKRDVVSVGV-PGDNVTIRFTADNPGPWFLHCH 486

PHPFHLHGHTFSVVRSAG NY+P++RDVS+G PDNVT+RF DNPGPWFLHCH

Sbjct 419 PHPFHLHGHTFSVVRSAGSDHYNYKNPMRRDTVSIGTSPTDNVTVRFVTDNPGPWFLHCH 478

Query 487 IDWHLEAGLAIVFAEDVPSVSFVDPAPEEWYDLCPEYEESL 527

IDWHL+AGLAIVFAED FV+PP++WDLCPY++++

Sbjct 479 IDWHLQAGLAIVFAEDTKDTKFVNPVPQDWRDLCPAYDKAM 519

BLAST is a registered trademark of the National Library of Medicine

[Support center](https://support.ncbi.nlm.nih.gov/ics/support/KBList.asp?style=classic&deptID=28049&folderID=11&) [Mailing list](https://blast.ncbi.nlm.nih.gov/Blast.cgi?CMD=Web&PAGE_TYPE=BlastDocs&DOC_TYPE=MailList)

[YouTube](https://www.youtube.com/ncbinlm)

[National Library Of Medicine](https://www.nlm.nih.gov/)

[National Institutes Of Health](https://www.nih.gov/)

[U.S. Department of Health & Human Services](https://www.hhs.gov/)

[USA.gov](https://www.usa.gov/)

[NCBI](https://www.ncbi.nlm.nih.gov/)

[*National Center for Biotechnology Information,*](https://www.ncbi.nlm.nih.gov/) *U.S. National Library of Medicine 8600 Rock ville Pik e, Bethesda MD, 20894 USA*

[Policies and Guidelines](https://www.ncbi.nlm.nih.gov/home/about/policies.shtml) | [Contact](https://www.ncbi.nlm.nih.gov/home/about/contact.shtml)

https://blast.ncbi.nlm.nih.gov/Blast.cgi

6/6


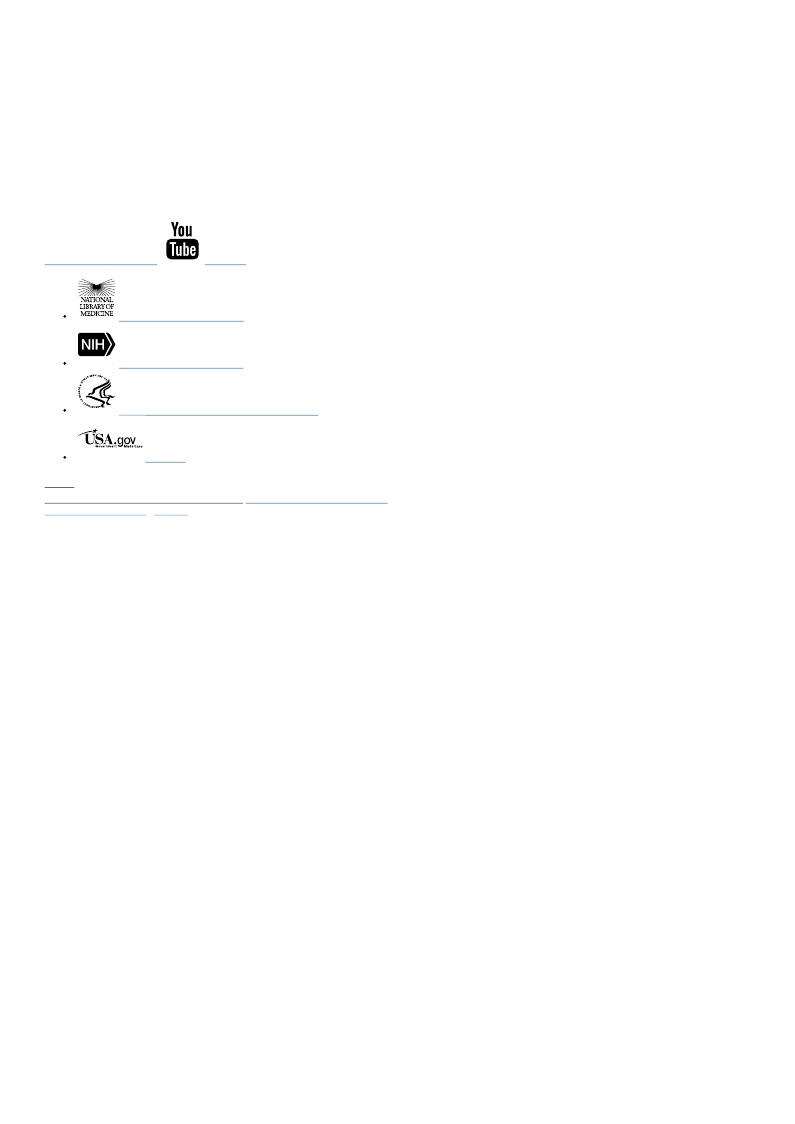

Supplement: Supplementary file 19 — Supplementary File 3d [file 41598_2017_4303_MOESM19_ESM.doc]
