# Supplementary material for: Genome-wide Identification and Characterization of Natural Antisense Transcripts by Strand-specific RNA Sequencing in Ganoderma lucidum
Source: Sci Rep. 2017 Jul 18;7:5711. doi: 10.1038/s41598-017-04303-6 (PMC5515960; doi:10.1038/s41598-017-04303-6)

2017/4/9

NCBI Blast:GL18428-R1_1

[BLAST ®](https://blast.ncbi.nlm.nih.gov/Blast.cgi) » blastp suite » RID-EMD459P7016

BLAST Results

Job title: GL18428-R1_1

RID

[EMD459P7016](https://blast.ncbi.nlm.nih.gov/Blast.cgi?CMD=Get&RID=EMD459P7016) (Expires on 04-10 21:17 pm)

Query ID

lcl|Query_80357

Database Name

nr

Description

Molecule type

Query Length

GL18428-R1_1

amino acid

497

Description All non-redundant GenBank CDS

translations+PDB+SwissProt+PIR+PRF excluding

environmental samples from WGS projects

Program BLASTP 2.6.0+

New Analyze your query with SmartBLAST

Graphic Summary

Putative conserved domains have been detected, click on the image below for detailed results.

Distribution of the top 100 Blast Hits on 100 subject sequences

Color key for alignment scores

<40

40-50

50-80

80-200

>=200

Query

1

90

180

270

360

450

https://blast.ncbi.nlm.nih.gov/Blast.cgi

1/6


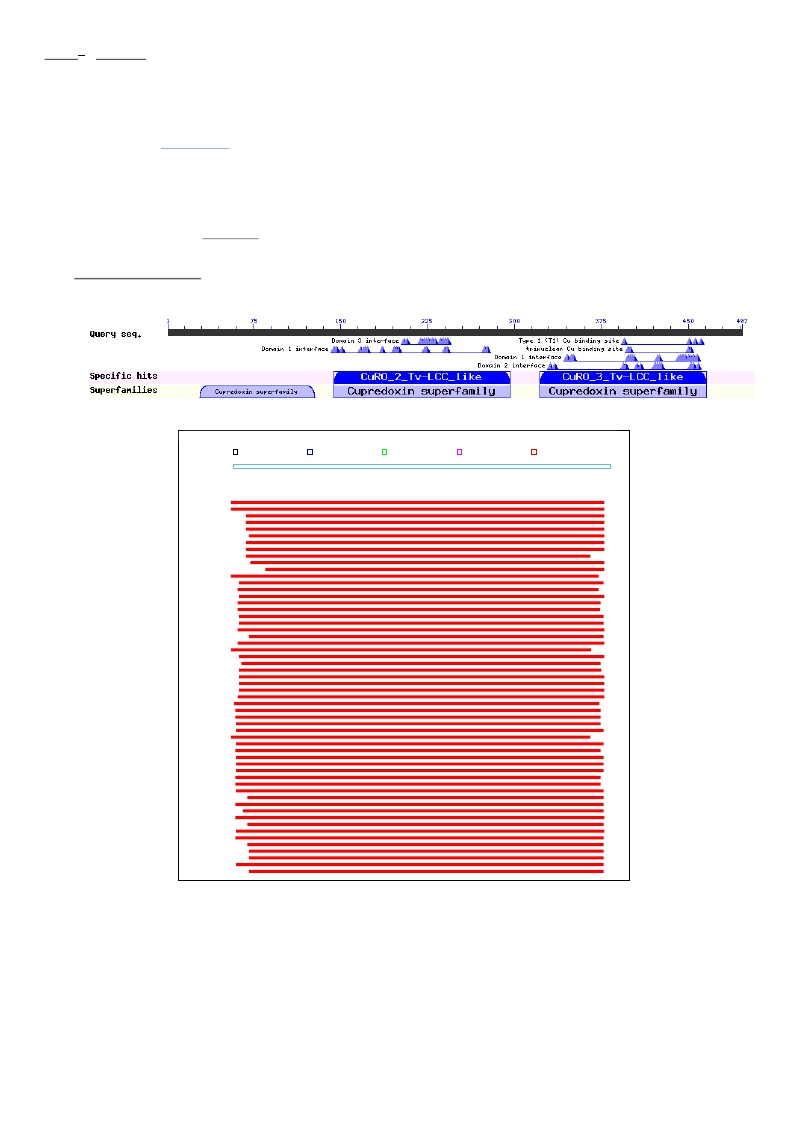


2017/4/9

Descriptions

Sequences producing significant alignments:

NCBI Blast:GL18428-R1_1

Description

[Max](https://blast.ncbi.nlm.nih.gov/Blast.cgi?CMD=Get&ALIGNMENTS=100&ALIGNMENT_VIEW=Pairwise&CDD_RID=EMD43YAT013&CDD_SEARCH_STATE=0&DATABASE_SORT=0&DESCRIPTIONS=100&DYNAMIC_FORMAT=on&FIRST_QUERY_NUM=0&FORMAT_OBJECT=Alignment&FORMAT_PAGE_TARGET=&FORMAT_TYPE=HTML&GET_SEQUENCE=yes&I_THRESH=&LINE_LENGTH=60&MASK_CHAR=2&MASK_COLOR=1&NEW_VIEW=yes&NUM_OVERVIEW=100&PAGE=Proteins&QUERY_INDEX=0&QUERY_NUMBER=0&RESULTS_PAGE_TARGET=&RID=EMD459P7016&SHOW_LINKOUT=yes&SHOW_OVERVIEW=yes&STEP_NUMBER=&WORD_SIZE=6&OLD_VIEW=false&DISPLAY_SORT=1&HSP_SORT=1)

[Total](https://blast.ncbi.nlm.nih.gov/Blast.cgi?CMD=Get&ALIGNMENTS=100&ALIGNMENT_VIEW=Pairwise&CDD_RID=EMD43YAT013&CDD_SEARCH_STATE=0&DATABASE_SORT=0&DESCRIPTIONS=100&DYNAMIC_FORMAT=on&FIRST_QUERY_NUM=0&FORMAT_OBJECT=Alignment&FORMAT_PAGE_TARGET=&FORMAT_TYPE=HTML&GET_SEQUENCE=yes&I_THRESH=&LINE_LENGTH=60&MASK_CHAR=2&MASK_COLOR=1&NEW_VIEW=yes&NUM_OVERVIEW=100&PAGE=Proteins&QUERY_INDEX=0&QUERY_NUMBER=0&RESULTS_PAGE_TARGET=&RID=EMD459P7016&SHOW_LINKOUT=yes&SHOW_OVERVIEW=yes&STEP_NUMBER=&WORD_SIZE=6&OLD_VIEW=false&DISPLAY_SORT=2&HSP_SORT=1)

[Query](https://blast.ncbi.nlm.nih.gov/Blast.cgi?CMD=Get&ALIGNMENTS=100&ALIGNMENT_VIEW=Pairwise&CDD_RID=EMD43YAT013&CDD_SEARCH_STATE=0&DATABASE_SORT=0&DESCRIPTIONS=100&DYNAMIC_FORMAT=on&FIRST_QUERY_NUM=0&FORMAT_OBJECT=Alignment&FORMAT_PAGE_TARGET=&FORMAT_TYPE=HTML&GET_SEQUENCE=yes&I_THRESH=&LINE_LENGTH=60&MASK_CHAR=2&MASK_COLOR=1&NEW_VIEW=yes&NUM_OVERVIEW=100&PAGE=Proteins&QUERY_INDEX=0&QUERY_NUMBER=0&RESULTS_PAGE_TARGET=&RID=EMD459P7016&SHOW_LINKOUT=yes&SHOW_OVERVIEW=yes&STEP_NUMBER=&WORD_SIZE=6&OLD_VIEW=false&DISPLAY_SORT=4&HSP_SORT=0)

[E](https://blast.ncbi.nlm.nih.gov/Blast.cgi?CMD=Get&ALIGNMENTS=100&ALIGNMENT_VIEW=Pairwise&CDD_RID=EMD43YAT013&CDD_SEARCH_STATE=0&DATABASE_SORT=0&DESCRIPTIONS=100&DYNAMIC_FORMAT=on&FIRST_QUERY_NUM=0&FORMAT_OBJECT=Alignment&FORMAT_PAGE_TARGET=&FORMAT_TYPE=HTML&GET_SEQUENCE=yes&I_THRESH=&LINE_LENGTH=60&MASK_CHAR=2&MASK_COLOR=1&NEW_VIEW=yes&NUM_OVERVIEW=100&PAGE=Proteins&QUERY_INDEX=0&QUERY_NUMBER=0&RESULTS_PAGE_TARGET=&RID=EMD459P7016&SHOW_LINKOUT=yes&SHOW_OVERVIEW=yes&STEP_NUMBER=&WORD_SIZE=6&OLD_VIEW=false&DISPLAY_SORT=0&HSP_SORT=0)

[Ident](https://blast.ncbi.nlm.nih.gov/Blast.cgi?CMD=Get&ALIGNMENTS=100&ALIGNMENT_VIEW=Pairwise&CDD_RID=EMD43YAT013&CDD_SEARCH_STATE=0&DATABASE_SORT=0&DESCRIPTIONS=100&DYNAMIC_FORMAT=on&FIRST_QUERY_NUM=0&FORMAT_OBJECT=Alignment&FORMAT_PAGE_TARGET=&FORMAT_TYPE=HTML&GET_SEQUENCE=yes&I_THRESH=&LINE_LENGTH=60&MASK_CHAR=2&MASK_COLOR=1&NEW_VIEW=yes&NUM_OVERVIEW=100&PAGE=Proteins&QUERY_INDEX=0&QUERY_NUMBER=0&RESULTS_PAGE_TARGET=&RID=EMD459P7016&SHOW_LINKOUT=yes&SHOW_OVERVIEW=yes&STEP_NUMBER=&WORD_SIZE=6&DISPLAY_SORT=3&HSP_SORT=3)

Accession

[score](https://blast.ncbi.nlm.nih.gov/Blast.cgi?CMD=Get&ALIGNMENTS=100&ALIGNMENT_VIEW=Pairwise&CDD_RID=EMD43YAT013&CDD_SEARCH_STATE=0&DATABASE_SORT=0&DESCRIPTIONS=100&DYNAMIC_FORMAT=on&FIRST_QUERY_NUM=0&FORMAT_OBJECT=Alignment&FORMAT_PAGE_TARGET=&FORMAT_TYPE=HTML&GET_SEQUENCE=yes&I_THRESH=&LINE_LENGTH=60&MASK_CHAR=2&MASK_COLOR=1&NEW_VIEW=yes&NUM_OVERVIEW=100&PAGE=Proteins&QUERY_INDEX=0&QUERY_NUMBER=0&RESULTS_PAGE_TARGET=&RID=EMD459P7016&SHOW_LINKOUT=yes&SHOW_OVERVIEW=yes&STEP_NUMBER=&WORD_SIZE=6&OLD_VIEW=false&DISPLAY_SORT=1&HSP_SORT=1)

[score](https://blast.ncbi.nlm.nih.gov/Blast.cgi?CMD=Get&ALIGNMENTS=100&ALIGNMENT_VIEW=Pairwise&CDD_RID=EMD43YAT013&CDD_SEARCH_STATE=0&DATABASE_SORT=0&DESCRIPTIONS=100&DYNAMIC_FORMAT=on&FIRST_QUERY_NUM=0&FORMAT_OBJECT=Alignment&FORMAT_PAGE_TARGET=&FORMAT_TYPE=HTML&GET_SEQUENCE=yes&I_THRESH=&LINE_LENGTH=60&MASK_CHAR=2&MASK_COLOR=1&NEW_VIEW=yes&NUM_OVERVIEW=100&PAGE=Proteins&QUERY_INDEX=0&QUERY_NUMBER=0&RESULTS_PAGE_TARGET=&RID=EMD459P7016&SHOW_LINKOUT=yes&SHOW_OVERVIEW=yes&STEP_NUMBER=&WORD_SIZE=6&OLD_VIEW=false&DISPLAY_SORT=2&HSP_SORT=1)

[cover](https://blast.ncbi.nlm.nih.gov/Blast.cgi?CMD=Get&ALIGNMENTS=100&ALIGNMENT_VIEW=Pairwise&CDD_RID=EMD43YAT013&CDD_SEARCH_STATE=0&DATABASE_SORT=0&DESCRIPTIONS=100&DYNAMIC_FORMAT=on&FIRST_QUERY_NUM=0&FORMAT_OBJECT=Alignment&FORMAT_PAGE_TARGET=&FORMAT_TYPE=HTML&GET_SEQUENCE=yes&I_THRESH=&LINE_LENGTH=60&MASK_CHAR=2&MASK_COLOR=1&NEW_VIEW=yes&NUM_OVERVIEW=100&PAGE=Proteins&QUERY_INDEX=0&QUERY_NUMBER=0&RESULTS_PAGE_TARGET=&RID=EMD459P7016&SHOW_LINKOUT=yes&SHOW_OVERVIEW=yes&STEP_NUMBER=&WORD_SIZE=6&OLD_VIEW=false&DISPLAY_SORT=4&HSP_SORT=0)

[value](https://blast.ncbi.nlm.nih.gov/Blast.cgi?CMD=Get&ALIGNMENTS=100&ALIGNMENT_VIEW=Pairwise&CDD_RID=EMD43YAT013&CDD_SEARCH_STATE=0&DATABASE_SORT=0&DESCRIPTIONS=100&DYNAMIC_FORMAT=on&FIRST_QUERY_NUM=0&FORMAT_OBJECT=Alignment&FORMAT_PAGE_TARGET=&FORMAT_TYPE=HTML&GET_SEQUENCE=yes&I_THRESH=&LINE_LENGTH=60&MASK_CHAR=2&MASK_COLOR=1&NEW_VIEW=yes&NUM_OVERVIEW=100&PAGE=Proteins&QUERY_INDEX=0&QUERY_NUMBER=0&RESULTS_PAGE_TARGET=&RID=EMD459P7016&SHOW_LINKOUT=yes&SHOW_OVERVIEW=yes&STEP_NUMBER=&WORD_SIZE=6&OLD_VIEW=false&DISPLAY_SORT=0&HSP_SORT=0)

laccase [Ganoderma lucidum]

laccase G [Trametes hirsuta]

1001

756

1001

756

99%

99%

0.0

0.0

96%

72%

[AHA83589.1](https://www.ncbi.nlm.nih.gov/protein/558633461?report=genbank&log$=prottop&blast_rank=1&RID=EMD459P7016)

[AIZ72726.1](https://www.ncbi.nlm.nih.gov/protein/732554706?report=genbank&log$=prottop&blast_rank=2&RID=EMD459P7016)

RecName: Full=Laccase-4; AltName: Full=Benzenediol:oxygen

oxidoreductase 4; AltName: Full=Diphenol oxidase 4; AltName:

744

744

95%

0.0

72%

[Q12719.1](https://www.ncbi.nlm.nih.gov/protein/2833234?report=genbank&log$=prottop&blast_rank=3&RID=EMD459P7016)

Full=Urishiol oxidase 4; Flags: Precursor

laccase-4 [Trametes versicolor FP-101664 SS1]

744

744

95%

0.0

72%

[XP_008035965.1](https://www.ncbi.nlm.nih.gov/protein/636609661?report=genbank&log$=prottop&blast_rank=4&RID=EMD459P7016)

RecName: Full=Laccase-4; AltName: Full=Benzenediol:oxygen

oxidoreductase 4; AltName: Full=Diphenol oxidase 4; AltName:

743

743

95%

0.0

72%

[Q99055.1](https://www.ncbi.nlm.nih.gov/protein/2842755?report=genbank&log$=prottop&blast_rank=5&RID=EMD459P7016)

Full=Urishiol oxidase 4; Flags: Precursor

laccase [Trametes versicolor]

multicopper oxidase [Trametes versicolor]

laccase3 [Trametes versicolor]

Laccase-4 [Trametes pubescens]

laccase [Ganoderma lucidum]

laccase LCC3-3 [Polyporus ciliatus]

laccase [Ganoderma lucidum]

laccase C [Trametes hirsuta]

laccase [Ganoderma lucidum]

laccase 1A [Trametes pubescens]

laccase [Dichomitus squalens LYAD-421 SS1]

laccase 2 [Dichomitus squalens LYAD-421 SS1]

laccase 2 [Trametes hirsuta]

laccase B [Trametes sp. AH28-2]

laccase E [Trametes ochracea]

Chain A, Crystal Structure Of Lacb From Trametes Sp. Ah28-

2

laccase [Cerrena sp. WR1]

laccase D [Trametes sp. 420]

laccase [Trametes versicolor FP-101664 SS1]

laccase LCC3-2 [Polyporus ciliatus]

Laccase-5 [Trametes pubescens]

738

732

731

726

725

724

720

712

709

709

708

707

704

703

701

700

698

697

694

692

692

738

732

731

726

725

724

720

712

709

709

708

707

704

703

701

700

698

697

694

692

692

94%

95%

95%

91%

94%

90%

98%

97%

96%

97%

96%

96%

97%

97%

97%

94%

97%

96%

97%

95%

96%

0.0

0.0

0.0

0.0

0.0

0.0

0.0

0.0

0.0

0.0

0.0

0.0

0.0

0.0

0.0

0.0

0.0

0.0

0.0

0.0

0.0

72%

71%

71%

73%

72%

74%

67%

70%

69%

68%

68%

68%

68%

68%

67%

69%

68%

68%

68%

69%

68%

[ADI70681.1](https://www.ncbi.nlm.nih.gov/protein/298239752?report=genbank&log$=prottop&blast_rank=6&RID=EMD459P7016)

[CAM12361.1](https://www.ncbi.nlm.nih.gov/protein/121944878?report=genbank&log$=prottop&blast_rank=7&RID=EMD459P7016)

[BAD98307.1](https://www.ncbi.nlm.nih.gov/protein/63147346?report=genbank&log$=prottop&blast_rank=8&RID=EMD459P7016)

[OJT07651.1](https://www.ncbi.nlm.nih.gov/protein/1112953729?report=genbank&log$=prottop&blast_rank=9&RID=EMD459P7016)

[AHA83593.1](https://www.ncbi.nlm.nih.gov/protein/558633469?report=genbank&log$=prottop&blast_rank=10&RID=EMD459P7016)

[AAG09231.1](https://www.ncbi.nlm.nih.gov/protein/9957147?report=genbank&log$=prottop&blast_rank=11&RID=EMD459P7016)

[AHA83596.1](https://www.ncbi.nlm.nih.gov/protein/558633475?report=genbank&log$=prottop&blast_rank=12&RID=EMD459P7016)

[AIZ72722.1](https://www.ncbi.nlm.nih.gov/protein/732554698?report=genbank&log$=prottop&blast_rank=13&RID=EMD459P7016)

[AHA83594.1](https://www.ncbi.nlm.nih.gov/protein/558633471?report=genbank&log$=prottop&blast_rank=14&RID=EMD459P7016)

[AAM18408.1](https://www.ncbi.nlm.nih.gov/protein/20270772?report=genbank&log$=prottop&blast_rank=15&RID=EMD459P7016)

[XP_007367294.1](https://www.ncbi.nlm.nih.gov/protein/597996083?report=genbank&log$=prottop&blast_rank=16&RID=EMD459P7016)

[XP_007360764.1](https://www.ncbi.nlm.nih.gov/protein/597970761?report=genbank&log$=prottop&blast_rank=17&RID=EMD459P7016)

[AOX15703.1](https://www.ncbi.nlm.nih.gov/protein/1083918173?report=genbank&log$=prottop&blast_rank=18&RID=EMD459P7016)

[AAW31597.1](https://www.ncbi.nlm.nih.gov/protein/56809865?report=genbank&log$=prottop&blast_rank=19&RID=EMD459P7016)

[ALT22028.1](https://www.ncbi.nlm.nih.gov/protein/965871910?report=genbank&log$=prottop&blast_rank=20&RID=EMD459P7016)

[3KW7_A](https://www.ncbi.nlm.nih.gov/protein/290790140?report=genbank&log$=prottop&blast_rank=21&RID=EMD459P7016)

[ACZ58367.1](https://www.ncbi.nlm.nih.gov/protein/270047920?report=genbank&log$=prottop&blast_rank=22&RID=EMD459P7016)

[AAW28939.1](https://www.ncbi.nlm.nih.gov/protein/56785448?report=genbank&log$=prottop&blast_rank=23&RID=EMD459P7016)

[XP_008035966.1](https://www.ncbi.nlm.nih.gov/protein/636609663?report=genbank&log$=prottop&blast_rank=24&RID=EMD459P7016)

[AAG09230.1](https://www.ncbi.nlm.nih.gov/protein/9957145?report=genbank&log$=prottop&blast_rank=25&RID=EMD459P7016)

[OJT07652.1](https://www.ncbi.nlm.nih.gov/protein/1112953730?report=genbank&log$=prottop&blast_rank=26&RID=EMD459P7016)

RecName: Full=Laccase-5; AltName: Full=Benzenediol:oxygen

oxidoreductase 5; AltName: Full=Diphenol oxidase 5; AltName:

691

691

97%

0.0

67%

[Q99056.2](https://www.ncbi.nlm.nih.gov/protein/18281739?report=genbank&log$=prottop&blast_rank=27&RID=EMD459P7016)

Full=Urishiol oxidase 5; Flags: Precursor

RecName: Full=Laccase-5; AltName: Full=Benzenediol:oxygen

oxidoreductase 5; AltName: Full=Diphenol oxidase 5; AltName:

Full=Laccase IV; AltName: Full=Urishiol oxidase 5; Flags:

688

688

97%

0.0

67%

[Q12717.1](https://www.ncbi.nlm.nih.gov/protein/2833232?report=genbank&log$=prottop&blast_rank=28&RID=EMD459P7016)

Precursor

laccase [Trametes versicolor]

laccase4 [Trametes versicolor]

laccase [Trametes sp. C30]

laccase hybrid [Trametes sp. C30]

laccase 2 [Trametes sp. C30]

LAC2 [Polyporus brumalis]

laccase hybrid [Trametes sp. C30]

laccase A [Trametes sp. 420]

laccase [Coriolopsis gallica]

laccase 2 [Coriolopsis gallica]

laccase [Coriolopsis gallica]

laccase [Coriolopsis gallica]

laccase [Coriolopsis gallica]

laccase 2 [Coriolopsis trogii]

precursor laccase lcc2 [Coriolopsis trogii]

laccase [Coriolopsis trogii]

phenoloxidase [Trametes sp. I-62]

laccase [Meripilus giganteus]

Laccase [Trametes cinnabarina]

687

686

684

684

681

679

674

673

672

672

671

669

669

668

668

668

667

666

665

687

686

684

684

681

679

674

673

672

672

671

669

669

668

668

668

667

666

665

97%

97%

97%

97%

97%

97%

98%

95%

98%

97%

98%

98%

98%

97%

97%

98%

95%

98%

96%

0.0

0.0

0.0

0.0

0.0

0.0

0.0

0.0

0.0

0.0

0.0

0.0

0.0

0.0

0.0

0.0

0.0

0.0

0.0

67%

67%

68%

67%

66%

67%

65%

67%

65%

65%

65%

65%

65%

65%

65%

64%

67%

65%

66%

[BAA23284.1](https://www.ncbi.nlm.nih.gov/protein/2598857?report=genbank&log$=prottop&blast_rank=29&RID=EMD459P7016)

[BAD98308.1](https://www.ncbi.nlm.nih.gov/protein/63147348?report=genbank&log$=prottop&blast_rank=30&RID=EMD459P7016)

[AAR00925.1](https://www.ncbi.nlm.nih.gov/protein/37702651?report=genbank&log$=prottop&blast_rank=31&RID=EMD459P7016)

[ACO53433.1](https://www.ncbi.nlm.nih.gov/protein/226424960?report=genbank&log$=prottop&blast_rank=32&RID=EMD459P7016)

[AAM66348.1](https://www.ncbi.nlm.nih.gov/protein/21616728?report=genbank&log$=prottop&blast_rank=33&RID=EMD459P7016)

[ABN13592.1](https://www.ncbi.nlm.nih.gov/protein/124495024?report=genbank&log$=prottop&blast_rank=34&RID=EMD459P7016)

[ACO53434.1](https://www.ncbi.nlm.nih.gov/protein/226424962?report=genbank&log$=prottop&blast_rank=35&RID=EMD459P7016)

[AAW28936.1](https://www.ncbi.nlm.nih.gov/protein/56785442?report=genbank&log$=prottop&blast_rank=36&RID=EMD459P7016)

[AJV90967.1](https://www.ncbi.nlm.nih.gov/protein/768806945?report=genbank&log$=prottop&blast_rank=37&RID=EMD459P7016)

[ACR50978.1](https://www.ncbi.nlm.nih.gov/protein/238632213?report=genbank&log$=prottop&blast_rank=38&RID=EMD459P7016)

[ACS26245.1](https://www.ncbi.nlm.nih.gov/protein/239809556?report=genbank&log$=prottop&blast_rank=39&RID=EMD459P7016)

[ABD93940.1](https://www.ncbi.nlm.nih.gov/protein/90436931?report=genbank&log$=prottop&blast_rank=40&RID=EMD459P7016)

[AJV90966.1](https://www.ncbi.nlm.nih.gov/protein/768806943?report=genbank&log$=prottop&blast_rank=41&RID=EMD459P7016)

[AMJ39539.1](https://www.ncbi.nlm.nih.gov/protein/995953022?report=genbank&log$=prottop&blast_rank=42&RID=EMD459P7016)

[CAL23367.1](https://www.ncbi.nlm.nih.gov/protein/119416759?report=genbank&log$=prottop&blast_rank=43&RID=EMD459P7016)

[CAC13040.1](https://www.ncbi.nlm.nih.gov/protein/10801036?report=genbank&log$=prottop&blast_rank=44&RID=EMD459P7016)

[AAB63445.1](https://www.ncbi.nlm.nih.gov/protein/2264400?report=genbank&log$=prottop&blast_rank=45&RID=EMD459P7016)

[CBV46340.1](https://www.ncbi.nlm.nih.gov/protein/317451550?report=genbank&log$=prottop&blast_rank=46&RID=EMD459P7016)

[CDO69469.1](https://www.ncbi.nlm.nih.gov/protein/691795797?report=genbank&log$=prottop&blast_rank=47&RID=EMD459P7016)

https://blast.ncbi.nlm.nih.gov/Blast.cgi

2/6


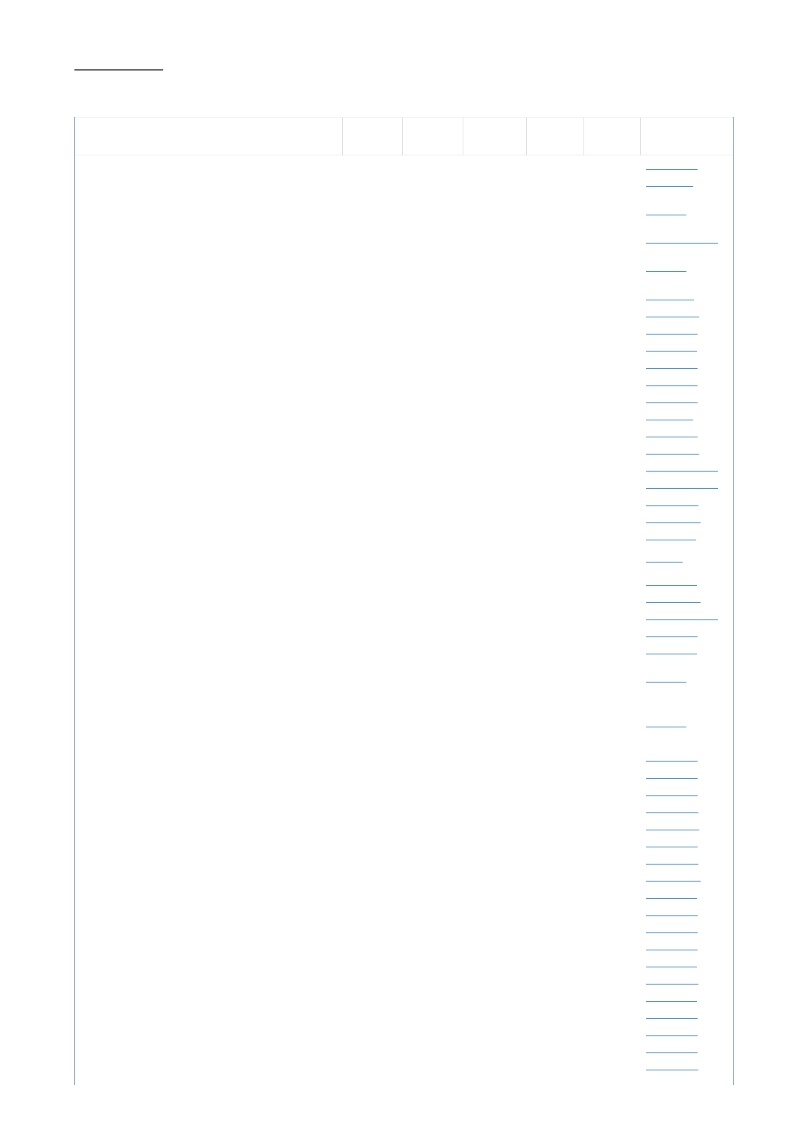


2017/4/9

NCBI Blast:GL18428-R1_1

laccase [Dichomitus squalens LYAD-421 SS1]

laccase [Trametes sp. I-62]

multicopper redoxase [Trametes sanguinea]

phenoloxidase [Trametes versicolor FP-101664 SS1]

laccase [Cerrena sp. WR1]

Chain A, Coriolopsis Gallica Laccase T2 Copper Depleted At

Ph 4.5

Chain A, Coriolopsis Gallica Laccase Collected At 12.65 Kev

laccase [Ganoderma lucidum]

Chain A, Crystal Structure Of Blue Laccase From Trametes

Trogii Complexed With P-Methylbenzoate

laccase 2 [Coriolopsis caperata]

laccase [Dichomitus squalens LYAD-421 SS1]

laccase hybrid [Trametes sp. C30]

hypothetical protein PLICRDRAFT_113151 [Plicaturopsis

crispa FD-325 SS-3]

polyphenoloxidase [Trametes sp. C30]

665

665

665

664

664

662

662

662

662

661

661

661

661

661

665

665

665

664

664

662

662

662

662

661

661

661

661

661

98%

95%

98%

98%

95%

94%

94%

98%

94%

98%

98%

98%

99%

98%

0.0

0.0

0.0

0.0

0.0

0.0

0.0

0.0

0.0

0.0

0.0

0.0

0.0

0.0

64%

66%

64%

65%

67%

66%

66%

64%

66%

64%

66%

65%

63%

65%

[XP_007360655.1](https://www.ncbi.nlm.nih.gov/protein/597970335?report=genbank&log$=prottop&blast_rank=48&RID=EMD459P7016)

[AAQ12270.1](https://www.ncbi.nlm.nih.gov/protein/33334373?report=genbank&log$=prottop&blast_rank=49&RID=EMD459P7016)

[ACN69056.1](https://www.ncbi.nlm.nih.gov/protein/224924161?report=genbank&log$=prottop&blast_rank=50&RID=EMD459P7016)

[XP_008036898.1](https://www.ncbi.nlm.nih.gov/protein/636611527?report=genbank&log$=prottop&blast_rank=51&RID=EMD459P7016)

[ACZ58368.1](https://www.ncbi.nlm.nih.gov/protein/270047922?report=genbank&log$=prottop&blast_rank=52&RID=EMD459P7016)

[4A2D_A](https://www.ncbi.nlm.nih.gov/protein/350610907?report=genbank&log$=prottop&blast_rank=53&RID=EMD459P7016)

[4A2F_A](https://www.ncbi.nlm.nih.gov/protein/385251975?report=genbank&log$=prottop&blast_rank=54&RID=EMD459P7016)

[AHA83584.1](https://www.ncbi.nlm.nih.gov/protein/558633451?report=genbank&log$=prottop&blast_rank=55&RID=EMD459P7016)

[2HRG_A](https://www.ncbi.nlm.nih.gov/protein/158428663?report=genbank&log$=prottop&blast_rank=56&RID=EMD459P7016)

[AGE13770.1](https://www.ncbi.nlm.nih.gov/protein/445065172?report=genbank&log$=prottop&blast_rank=57&RID=EMD459P7016)

[XP_007364547.1](https://www.ncbi.nlm.nih.gov/protein/597985483?report=genbank&log$=prottop&blast_rank=58&RID=EMD459P7016)

[ACO53432.1](https://www.ncbi.nlm.nih.gov/protein/226424958?report=genbank&log$=prottop&blast_rank=59&RID=EMD459P7016)

[KII87346.1](https://www.ncbi.nlm.nih.gov/protein/749762562?report=genbank&log$=prottop&blast_rank=60&RID=EMD459P7016)

[AAF06967.1](https://www.ncbi.nlm.nih.gov/protein/6318611?report=genbank&log$=prottop&blast_rank=61&RID=EMD459P7016)

Chain A, Crystallographic Structural Determination Of A

Trigonal Laccase From Coriolopsis Gallica (cgl) To 1.5 A

660

660

94%

0.0

65%

[5A7E_A](https://www.ncbi.nlm.nih.gov/protein/1032208307?report=genbank&log$=prottop&blast_rank=62&RID=EMD459P7016)

Resolution

laccase [basidiomycete PM1]

laccase [Coriolopsis gallica]

laccase [Pycnoporus coccineus]

laccase [Ganoderma lucidum]

laccase [Ganoderma lucidum]

laccase [Ganoderma lucidum]

laccase [Ganoderma weberianum]

OB1 laccase [synthetic construct]

laccase [Ganoderma lucidum]

Chain A, T2-depleted Laccase From Coriolopsis Caperata

Soaked With Cucl

Chain A, Crystal Structure Of Laccase From Basidiomycete

Pm1 (cect 2971)

laccase 3 [Trametes hirsuta]

laccase [Lentinus sp. WR2]

Chain A, Crystal Structure Of Laccase From Lentinus Sp. At 1.8

A Resolution

laccase [Trametes cinnabarina]

laccase 4 [Coriolopsis trogii]

laccase F [Trametes hirsuta]

laccase [Trametes sp. I-62]

phenoloxidase [Trametes sp. I-62]

laccase [Coriolopsis rigida]

laccase [Pycnoporus coccineus]

laccase [Dichomitus squalens LYAD-421 SS1]

Chain A, Crystal Structure Of A Fully Functional Laccase From

The Ligninolytic Fungus Pycnoporus Cinnabarinus

laccase [Coriolopsis rigida]

Chain A, Crystal Structure Determination Of A Blue Laccase

From Lentinus Tigrinus

laccase [Pycnoporus coccineus]

laccase 3 precursor [Cerrena sp. HYB07]

Laccase [Trametes pubescens]

660

657

657

657

656

655

655

654

654

654

653

653

652

651

651

650

650

650

649

649

648

648

648

648

647

647

646

645

660

657

657

657

656

655

655

654

654

654

653

653

652

651

651

650

650

650

649

649

648

648

648

648

647

647

646

645

98%

94%

96%

98%

98%

98%

98%

94%

98%

94%

94%

98%

98%

98%

95%

97%

98%

98%

98%

95%

98%

98%

94%

94%

94%

98%

95%

92%

0.0

0.0

0.0

0.0

0.0

0.0

0.0

0.0

0.0

0.0

0.0

0.0

0.0

0.0

0.0

0.0

0.0

0.0

0.0

0.0

0.0

0.0

0.0

0.0

0.0

0.0

0.0

0.0

65%

66%

64%

65%

64%

64%

64%

66%

64%

65%

67%

64%

64%

64%

65%

65%

63%

63%

63%

64%

64%

62%

65%

64%

65%

64%

65%

66%

[CAA78144.1](https://www.ncbi.nlm.nih.gov/protein/58324?report=genbank&log$=prottop&blast_rank=63&RID=EMD459P7016)

[AAW65489.1](https://www.ncbi.nlm.nih.gov/protein/58176544?report=genbank&log$=prottop&blast_rank=64&RID=EMD459P7016)

[AKE14488.1](https://www.ncbi.nlm.nih.gov/protein/810786215?report=genbank&log$=prottop&blast_rank=65&RID=EMD459P7016)

[AHA83595.1](https://www.ncbi.nlm.nih.gov/protein/558633473?report=genbank&log$=prottop&blast_rank=66&RID=EMD459P7016)

[AAG17009.2](https://www.ncbi.nlm.nih.gov/protein/121495873?report=genbank&log$=prottop&blast_rank=67&RID=EMD459P7016)

[ACR24357.1](https://www.ncbi.nlm.nih.gov/protein/237861575?report=genbank&log$=prottop&blast_rank=68&RID=EMD459P7016)

[ANA53145.1](https://www.ncbi.nlm.nih.gov/protein/1021766514?report=genbank&log$=prottop&blast_rank=69&RID=EMD459P7016)

[ALI16920.1](https://www.ncbi.nlm.nih.gov/protein/937376448?report=genbank&log$=prottop&blast_rank=70&RID=EMD459P7016)

[AHA83586.1](https://www.ncbi.nlm.nih.gov/protein/558633455?report=genbank&log$=prottop&blast_rank=71&RID=EMD459P7016)

[4JHU_A](https://www.ncbi.nlm.nih.gov/protein/635576677?report=genbank&log$=prottop&blast_rank=72&RID=EMD459P7016)

[5ANH_A](https://www.ncbi.nlm.nih.gov/protein/1059270720?report=genbank&log$=prottop&blast_rank=73&RID=EMD459P7016)

[AOX15704.1](https://www.ncbi.nlm.nih.gov/protein/1083918175?report=genbank&log$=prottop&blast_rank=74&RID=EMD459P7016)

[ACZ82339.1](https://www.ncbi.nlm.nih.gov/protein/270485111?report=genbank&log$=prottop&blast_rank=75&RID=EMD459P7016)

[3X1B_A](https://www.ncbi.nlm.nih.gov/protein/731187736?report=genbank&log$=prottop&blast_rank=76&RID=EMD459P7016)

[AAF13052.1](https://www.ncbi.nlm.nih.gov/protein/6466835?report=genbank&log$=prottop&blast_rank=77&RID=EMD459P7016)

[AMJ39541.1](https://www.ncbi.nlm.nih.gov/protein/995953026?report=genbank&log$=prottop&blast_rank=78&RID=EMD459P7016)

[AIZ72725.1](https://www.ncbi.nlm.nih.gov/protein/732554704?report=genbank&log$=prottop&blast_rank=79&RID=EMD459P7016)

[AAQ12269.1](https://www.ncbi.nlm.nih.gov/protein/33334371?report=genbank&log$=prottop&blast_rank=80&RID=EMD459P7016)

[AAB63444.1](https://www.ncbi.nlm.nih.gov/protein/2264398?report=genbank&log$=prottop&blast_rank=81&RID=EMD459P7016)

[ADK13098.1](https://www.ncbi.nlm.nih.gov/protein/300433312?report=genbank&log$=prottop&blast_rank=82&RID=EMD459P7016)

[BAB69775.1](https://www.ncbi.nlm.nih.gov/protein/16041065?report=genbank&log$=prottop&blast_rank=83&RID=EMD459P7016)

[XP_007360625.1](https://www.ncbi.nlm.nih.gov/protein/597970217?report=genbank&log$=prottop&blast_rank=84&RID=EMD459P7016)

[2XYB_A](https://www.ncbi.nlm.nih.gov/protein/358439660?report=genbank&log$=prottop&blast_rank=85&RID=EMD459P7016)

[ACU29545.1](https://www.ncbi.nlm.nih.gov/protein/255918284?report=genbank&log$=prottop&blast_rank=86&RID=EMD459P7016)

[2QT6_A](https://www.ncbi.nlm.nih.gov/protein/160877751?report=genbank&log$=prottop&blast_rank=87&RID=EMD459P7016)

[BAB69776.1](https://www.ncbi.nlm.nih.gov/protein/16041067?report=genbank&log$=prottop&blast_rank=88&RID=EMD459P7016)

[AID59411.1](https://www.ncbi.nlm.nih.gov/protein/658306910?report=genbank&log$=prottop&blast_rank=89&RID=EMD459P7016)

[OJT14186.1](https://www.ncbi.nlm.nih.gov/protein/1112961089?report=genbank&log$=prottop&blast_rank=90&RID=EMD459P7016)

RecName: Full=Laccase; AltName: Full=Benzenediol:oxygen

oxidoreductase; AltName: Full=Diphenol oxidase; AltName:

Full=Ligninolytic phenoloxidase; AltName: Full=Urishiol

645

645

98%

0.0

64%

[O59896.1](https://www.ncbi.nlm.nih.gov/protein/34922426?report=genbank&log$=prottop&blast_rank=91&RID=EMD459P7016)

oxidase; Flags: Precursor

laccase I [Trametes versicolor FP-101664 SS1]

laccase [Trametes cinnabarina]

laccase I [Trametes versicolor]

645

644

643

645

644

643

96%

98%

96%

0.0

0.0

0.0

64%

64%

64%

[XP_008032737.1](https://www.ncbi.nlm.nih.gov/protein/636603205?report=genbank&log$=prottop&blast_rank=92&RID=EMD459P7016)

[AAN71597.1](https://www.ncbi.nlm.nih.gov/protein/25140399?report=genbank&log$=prottop&blast_rank=93&RID=EMD459P7016)

[AAC49828.1](https://www.ncbi.nlm.nih.gov/protein/1172163?report=genbank&log$=prottop&blast_rank=94&RID=EMD459P7016)

RecName: Full=Laccase-2; AltName: Full=Benzenediol:oxygen

oxidoreductase 2; AltName: Full=Diphenol oxidase 2; AltName:

Full=Laccase I; AltName: Full=Urishiol oxidase 2; Flags:

643

643

96%

0.0

64%

[Q12718.1](https://www.ncbi.nlm.nih.gov/protein/2833233?report=genbank&log$=prottop&blast_rank=95&RID=EMD459P7016)

Precursor

laccase B [Trametes hirsuta]

bilirubin oxidase [Ganoderma tsunodae]

643

643

643

643

99%

98%

0.0

0.0

63%

64%

[AIZ72727.1](https://www.ncbi.nlm.nih.gov/protein/732554708?report=genbank&log$=prottop&blast_rank=96&RID=EMD459P7016)

[BAA28668.1](https://www.ncbi.nlm.nih.gov/protein/3176128?report=genbank&log$=prottop&blast_rank=97&RID=EMD459P7016)

https://blast.ncbi.nlm.nih.gov/Blast.cgi

3/6


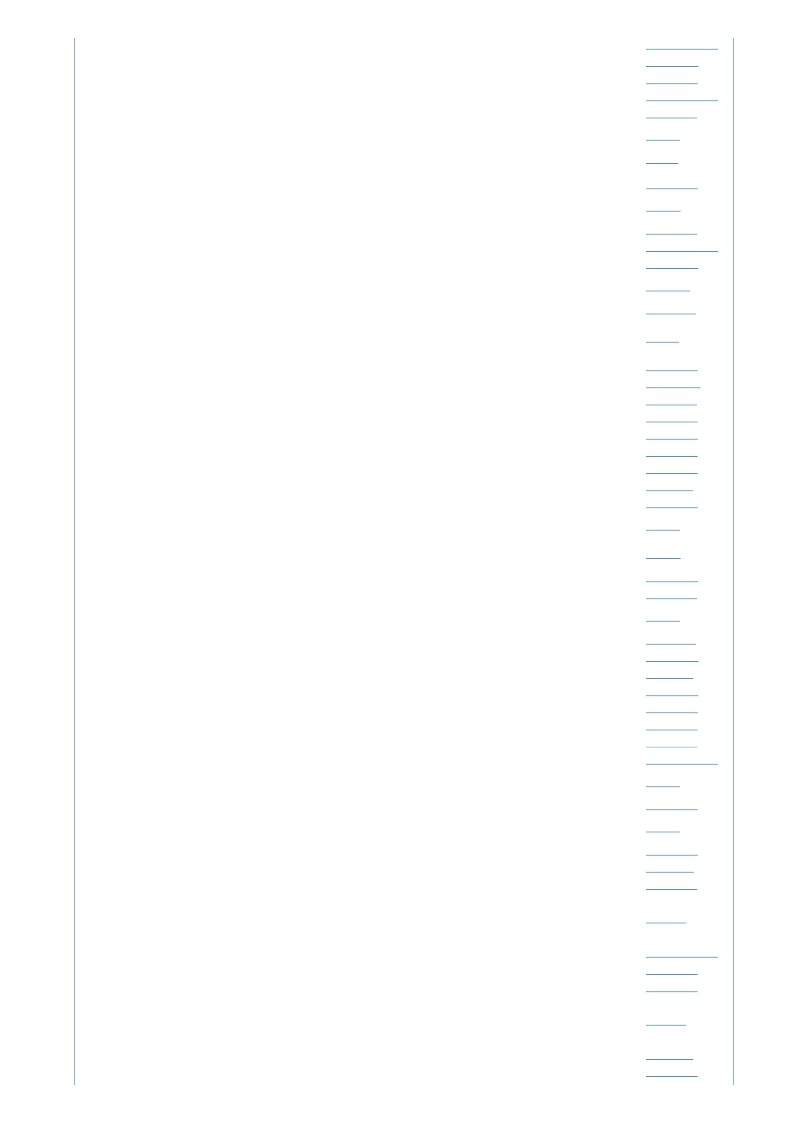


2017/4/9

NCBI Blast:GL18428-R1_1

laccase [Trametes versicolor]

laccase I [synthetic construct]

laccase 2 [Trametes pubescens]

642

642

641

642

642

641

96%

98%

98%

0.0

0.0

0.0

64%

63%

63%

[AFM31222.1](https://www.ncbi.nlm.nih.gov/protein/390980641?report=genbank&log$=prottop&blast_rank=98&RID=EMD459P7016)

[AJP70335.1](https://www.ncbi.nlm.nih.gov/protein/761668396?report=genbank&log$=prottop&blast_rank=99&RID=EMD459P7016)

[AAM18407.1](https://www.ncbi.nlm.nih.gov/protein/20270770?report=genbank&log$=prottop&blast_rank=100&RID=EMD459P7016)

Alignments

laccase, partial [Ganoderma lucidum]

Sequence ID: AHA83589.1 Length: 518 Number of Matches: 1

Range 1: 1 to 518

Score

Expect Method

Identities

Positives

Gaps

Frame

1001 bits(2587) 0.0()

Features:

Compositional matrix adjust. 496/518(96%) 496/518(95%) 22/518(4%)

Query 1

Sbjct 1

Query 61

Sbjct 61

MPQAVPLTLLSALGTLFASSLAATIGTVGDLTVSNKDISPDGYTRAAVVVNGQFPGPLIM 60

MPQAVPLTLLSALGTLFASSLAATIGTVGDLTVSNKDISPDGYTRAAVVVNGQFPGPLIM

MPQAVPLTLLSALGTLFASSLAATIGTVGDLTVSNKDISPDGYTRAAVVVNGQFPGPLIM 60

GNR----------------------HFHGIYQEGTNWEDGAAFVNQCPIATGNSFVYGFT 98

GNR HFHGIYQEGTNWEDGAAFVNQCPIATGNSFVYGFT

GNRGDNFQIRVVDQLTNETMLKSTSHFHGIYQEGTNWEDGAAFVNQCPIATGNSFVYGFT 120

Query 99 CLDQAGTFWYHSHLSTQYCDGLRGPMVVYDPDDPHASLYDIDDESTVITLSDWYHTAARL 158

CLDQAGTFWYHSHLSTQYCDGLRGPMVVYDPDDPHASLYDIDDESTVITLSDWYHTAARL

Sbjct 121 CLDQAGTFWYHSHLSTQYCDGLRGPMVVYDPDDPHASLYDIDDESTVITLSDWYHTAARL 180

Query 159 GTGFPRSDSVLINGLGRFAGGGSTDLAIIPVMRGKRYRFRLVSLSCDPNFTFSIDGHAMT 218

GTGFPRSDSVLINGLGRFAGGGSTDLAIIPVMRGKRYRFRLVSLSCDPNFTFSIDGHAMT

Sbjct 181 GTGFPRSDSVLINGLGRFAGGGSTDLAIIPVMRGKRYRFRLVSLSCDPNFTFSIDGHAMT 240

Query 219 VIEADAVNHEPLTVDSIQIFAGQRYSFVLTADQDIDNYWIRALPNRGTTNFDGGVNSAIL 278

VIEADAVNHEPLTVDSIQIFAGQRYSFVLTADQDIDNYWIRALPNRGTTNFDGGVNSAIL

Sbjct 241 VIEADAVNHEPLTVDSIQIFAGQRYSFVLTADQDIDNYWIRALPNRGTTNFDGGVNSAIL 300

Query 279 RYDGAAAVEPTTNQSTSIAPLAEADLMPLDRAPAPGDANRGGVDYALNLDFSVNGTRFFI 338

RYDGAAAVEPTTNQSTSIAPLAEADLMPLDRAPAPGDANRGGVDYALNLDFSVNGTRFFI

Sbjct 301 RYDGAAAVEPTTNQSTSIAPLAEADLMPLDRAPAPGDANRGGVDYALNLDFSVNGTRFFI 360

Query 339 NGATFTPPSAPVLLQILSGTRSAADLLPSGSVYTIPANATVELSFPITSKNAPGAPHPFH 398

NGATFTPPSAPVLLQILSGTRSAADLLPSGSVYTIPANATVELSFPITSKNAPGAPHPFH

Sbjct 361 NGATFTPPSAPVLLQILSGTRSAADLLPSGSVYTIPANATVELSFPITSKNAPGAPHPFH 420

Query 399 LHGHTFSVVRSAGSNTYNYDSPPRRDVVNTGTAGDNVTIRFTTNNPGPWFLHCHIDFHLE 458

LHGHTFSVVRSAGSNTYNYDSPPRRDVVNTGTAGDNVTIRFTTNNPGPWFLHCHIDFHLE

Sbjct 421 LHGHTFSVVRSAGSNTYNYDSPPRRDVVNTGTAGDNVTIRFTTNNPGPWFLHCHIDFHLE 480

Query 459 AGFAVVFAVDTNTTSSFTTSDDWKNLCPTYNALSSDDI 496

AGFAVVFAVDTNTTSSFTTSDDWKNLCPTYNALSSDDI

Sbjct 481 AGFAVVFAVDTNTTSSFTTSDDWKNLCPTYNALSSDDI 518

laccase G [Trametes hirsuta]

Sequence ID: AIZ72726.1 Length: 521 Number of Matches: 1

Range 1: 1 to 521

Score

Expect Method

Identities

Positives

Gaps

Frame

756 bits(1951) 0.0()

Features:

Compositional matrix adjust. 374/521(72%) 418/521(80%) 25/521(4%)

Query 1

Sbjct 1

Query 61

Sbjct 61

MPQAVPLTLLSALGTLFASSLAATIGTVGDLTVSNKDISPDGYTRAAVVVNGQFPGPLIM 60

M+ L+LL+L ++ AIGVDLT+SNDISPDGYTRAAVVVNGFPGPLI

MGRIESLSLLATLSPFLSLTAFAAIGPVTDLTISNTDISPDGYTRAAVVVNGVFPGPLIT 60

GNR-----------------------HFHGIYQEGTNWEDGAAFVNQCPIATGNSFVYGF 97

GN H+HG+QGTNWDGAFVNQCPIATGNSF+YF

GNMGDNFQINVVDNLTNETMLKSTTVHWHGFFQSGTNWADGGAFVNQCPIATGNSFLYDF 120

Query 98 TCLDQAGTFWYHSHLSTQYCDGLRGPMVVYDPDDPHASLYDIDDESTVITLSDWYHTAAR 157

T +QAGTFWYHSHLSTQYCDGLRGPMVVYDPDDPHASLYD+DDESTVITLSDWYHTAA+

Sbjct 121 TAKNQAGTFWYHSHLSTQYCDGLRGPMVVYDPDDPHASLYDVDDESTVITLSDWYHTAAK 180

Query 158 LGTGFPRS-DSVLINGLGRFAGGGSTDLAIIPVMRGKRYRFRLVSLSCDPNFTFSIDGHA 216

LG FP DSVLINGLGRFGGSTDLA+IV+GKRYRFRLVSLSCDPNFTFSIDH

Sbjct 181 LGAAFPTGPDSVLINGLGRFTGGNSTDLAVITVEQGKRYRFRLVSLSCDPNFTFSIDDHN 240

Query 217 MTVIEADAVNHEPLTVDSIQIFAGQRYSFVLTADQDIDNYWIRALPNRGTTNFDGGVNSA 276

MTVIEDAVNHELTVDIQI+AGQRYSFVLTADQD+NYWIRALPNGT+FGGVNSA

Sbjct 241 MTVIEVDAVNHEALTVDEIQIYAGQRYSFVLTADQDVGNYWIRALPNGGTVSFSGGVNSA 300

Query 277 ILRYDGAAAVEPTTNQSTSIAPLAEADLMPLDRAPAPGDANRGGVDYALNLDFSVNGTRF 336

ILRYGA VEPTTNQ+S LEDL+PLD APG+A GVDYA++L+FSNG+F

Sbjct 301 ILRYSGAPEVEPTTNQTLSTNTLVETDLVPLDDPAAPGEAVAEGVDYAMSLNFSFNGSNF 360

Query 337 FINGATFTPPSAPVLLQILSGTRSAADLLPSGSVYTIPANATVELSFPITSKNAPGAPHP 396

FINGATFPP+PVLLQILSG+SA+LLPSGSVY++P+N+T+E++FPIT+NAPGAPHP

Sbjct 361 FINGATFVPPTVPVLLQILSGAQSASSLLPSGSVYSLPSNSTIEIAFPITATNAPGAPHP 420

Query 397 FHLHGHTFSVVRSAGSNTYNYDSPPRRDVVNTGTAGDNVTIRFTTNNPGPWFLHCHIDFH 456

FHLHGHTFSVVRSAGS+TYNY+PRRDVVNTGTAGDNVTIRFT+NPGPWFLHCHIDFH

Sbjct 421 FHLHGHTFSVVRSAGSSTYNYANPVRRDVVNTGTAGDNVTIRFKTDNPGPWFLHCHIDFH 480

Query 457 LEAGFAVVFAVDTNTTSSFTT-SDDWKNLCPTYNALSSDDI 496

LEAGFA+VF+DT+ SFTS W+NLCTY+ALSD+

Sbjct 481 LEAGFAMVFSEDTDEVVSFNTPSTAWENLCTTYDALDSSDL 521

RecName: Full=Laccase-4; AltName: Full=Benzenediol:oxygen oxidoreductase 4; AltName: Full=Diphenol oxidase 4; AltName:

Full=Urishiol oxidase 4; Flags: Precursor

Sequence ID: Q12719.1 Length: 520 Number of Matches: 1

See 2 more title(s)

Range 1: 20 to 520

https://blast.ncbi.nlm.nih.gov/Blast.cgi

4/6


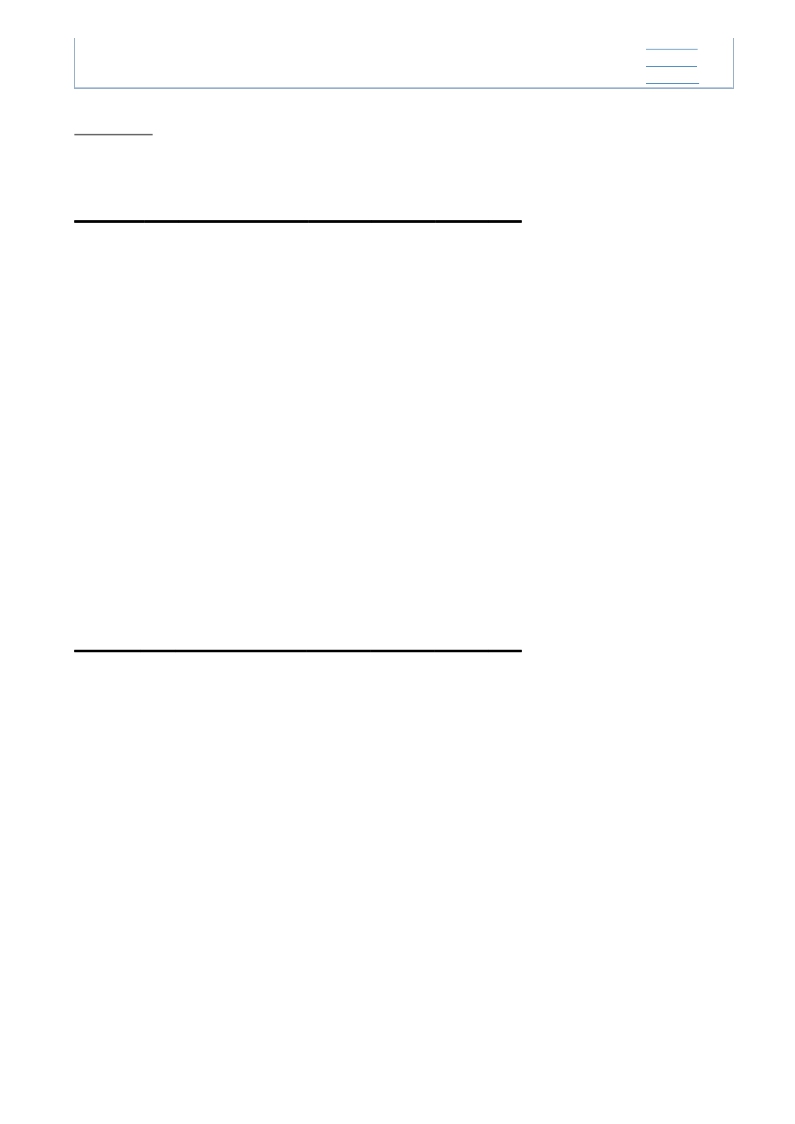


2017/4/9

Range 1: 20 to 520

NCBI Blast:GL18428-R1_1

Score

Expect Method

Identities

Positives

Gaps

Frame

744 bits(1922) 0.0()

Features:

Compositional matrix adjust. 362/501(72%) 416/501(83%) 25/501(4%)

Query 21

Sbjct 20

Query 64

Sbjct 80

LAATIGTVGDLTVSNKDISPDGYTRAAVVVNGQFPGPLIMGNR----------------- 63

++AIGVDLT+SND+SPDG+TRAAV+NGFPGPLIGN+

VSAAIGPVTDLTISNADVSPDGFTRAAVLANGVFPGPLITGNKGDNFQINVIDNLSNETM 79

------HFHGIYQEGTNWEDGAAFVNQCPIATGNSFVYGFTCLDQAGTFWYHSHLSTQYC 117

H+HG+Q+GTNWDGAAFVNQCPIATGNSF+YFT DQAGTFWYHSHLSTQYC

LKSTSIHWHGFFQKGTNWADGAAFVNQCPIATGNSFLYDFTATDQAGTFWYHSHLSTQYC 139

Query 118 DGLRGPMVVYDPDDPHASLYDIDDESTVITLSDWYHTAARLGTGFP-RSDSVLINGLGRF 176

DGLRGPMVVYDPDPHALYD+DDE+T+ITLSDWYHTAALG FP SDSLINGLGRF

Sbjct 140 DGLRGPMVVYDPSDPHADLYDVDDETTIITLSDWYHTAASLGAAFPIGSDSTLINGLGRF 199

Query 177 AGGGSTDLAIIPVMRGKRYRFRLVSLSCDPNFTFSIDGHAMTVIEADAVNHEPLTVDSIQ 236

AGGSTDLA+IV+GKRYRRL+SLSCDPN+FSIDGHMT+IEADAVNHEPLTVDSIQ

Sbjct 200 AGGDSTDLAVITVEQGKRYRMRLLSLSCDPNYVFSIDGHNMTIIEADAVNHEPLTVDSIQ 259

Query 237 IFAGQRYSFVLTADQDIDNYWIRALPNRGTTNFDGGVNSAILRYDGAAAVEPTTNQSTSI 296

I+AGQRYSFVLTADQDIDNY+IRALP+GTT+FDGG+NSAILRYGA+V+PTT++TS+

Sbjct 260 IYAGQRYSFVLTADQDIDNYFIRALPSAGTTSFDGGINSAILRYSGASEVDPTTTETTSV 319

Query 297 APLAEADLMPLDRAPAPGDANRGGVDYALNLDFSVNGTRFFINGATFTPPSAPVLLQILS 356

PLEA+L+PLD APGDNGGVDYALNLDF++GTFFIN +F P+PVLLQILS

Sbjct 320 LPLDEANLVPLDSPAAPGDPNIGGVDYALNLDFNFDGTNFFINDVSFVSPTVPVLLQILS 379

Query 357 GTRSAADLLPSGSVYTIPANATVELSFPITSKNAPGAPHPFHLHGHTFSVVRSAGSNTYN 416

GTSAADLLPSGS+++P+N+T+E+SFPIT+NAPGAPHPFHLHGHTFS+VR+AGS N

Sbjct 380 GTTSAADLLPSGSLFALPSNSTIEISFPITATNAPGAPHPFHLHGHTFSIVRTAGSTDTN 439

Query 417 YDSPPRRDVVNTGTAGDNVTIRFTTNNPGPWFLHCHIDFHLEAGFAVVFAVDTNTTSSFT 476

++PRRDVVNTGTAGDNVTIRFTT+NPGPWFLHCHIDFHLEAGFA+VF+DT S+T

Sbjct 440 FVNPVRRDVVNTGTAGDNVTIRFTTDNPGPWFLHCHIDFHLEAGFAIVFSEDTADVSNTT 499

Query 477 T-SDDWKNLCPTYNALSSDDI 496

TS W++LCPTYNALSD+

Sbjct 500 TPSTAWEDLCPTYNALDSSDL 520

laccase-4 [Trametes versicolor FP-101664 SS1]

Sequence ID: XP_008035965.1 Length: 520 Number of Matches: 1

See 1 more title(s)

Range 1: 20 to 520

Score

Expect Method

Identities

Positives

Gaps

Frame

744 bits(1921) 0.0()

Features:

Compositional matrix adjust. 361/501(72%) 416/501(83%) 25/501(4%)

Query 21

Sbjct 20

Query 64

Sbjct 80

LAATIGTVGDLTVSNKDISPDGYTRAAVVVNGQFPGPLIMGNR----------------- 63

++AIGVDLT+SND+SPDG+TRAAV+NGFPGPLIGN+

VSAAIGPVTDLTISNADVSPDGFTRAAVLANGVFPGPLITGNKGDNFQINVIDNLSNETM 79

------HFHGIYQEGTNWEDGAAFVNQCPIATGNSFVYGFTCLDQAGTFWYHSHLSTQYC 117

H+HG+Q+GTNWDGAAFVNQCPIATGNSF+YFT DQAGTFWYHSHLSTQYC

LKSTSIHWHGFFQKGTNWADGAAFVNQCPIATGNSFLYDFTATDQAGTFWYHSHLSTQYC 139

Query 118 DGLRGPMVVYDPDDPHASLYDIDDESTVITLSDWYHTAARLGTGFP-RSDSVLINGLGRF 176

DGLRGPMVVYDPDPHALYD+DDE+T++TLSDWYHTAALG FP SDSLINGLGRF

Sbjct 140 DGLRGPMVVYDPSDPHADLYDVDDETTIVTLSDWYHTAASLGAAFPIGSDSTLINGLGRF 199

Query 177 AGGGSTDLAIIPVMRGKRYRFRLVSLSCDPNFTFSIDGHAMTVIEADAVNHEPLTVDSIQ 236

AGGSTDLA+IV+GKRYRRL+SLSCDPN+FSIDGHMT+IEADAVNHEPLTVDSIQ

Sbjct 200 AGGDSTDLAVITVEQGKRYRMRLLSLSCDPNYVFSIDGHNMTIIEADAVNHEPLTVDSIQ 259

Query 237 IFAGQRYSFVLTADQDIDNYWIRALPNRGTTNFDGGVNSAILRYDGAAAVEPTTNQSTSI 296

I+AGQRYSFVLTADQDIDNY+IRALP+GTT+FDGG+NSAILRYGA+V+PTT++TS+

Sbjct 260 IYAGQRYSFVLTADQDIDNYFIRALPSAGTTSFDGGINSAILRYSGASEVDPTTTETTSV 319

Query 297 APLAEADLMPLDRAPAPGDANRGGVDYALNLDFSVNGTRFFINGATFTPPSAPVLLQILS 356

PLEA+L+PLD APGDNGGVDYALNLDF++GTFFIN +F P+PVLLQILS

Sbjct 320 LPLDEANLVPLDSPAAPGDPNIGGVDYALNLDFNFDGTNFFINDVSFVSPTVPVLLQILS 379

Query 357 GTRSAADLLPSGSVYTIPANATVELSFPITSKNAPGAPHPFHLHGHTFSVVRSAGSNTYN 416

GTSAADLLPSGS+++P+N+T+E+SFPIT+NAPGAPHPFHLHGHTFS+VR+AGS N

Sbjct 380 GTTSAADLLPSGSLFALPSNSTIEISFPITATNAPGAPHPFHLHGHTFSIVRTAGSTDTN 439

Query 417 YDSPPRRDVVNTGTAGDNVTIRFTTNNPGPWFLHCHIDFHLEAGFAVVFAVDTNTTSSFT 476

++PRRDVVNTGTAGDNVTIRFTT+NPGPWFLHCHIDFHLEAGFA+VF+DT S+T

Sbjct 440 FVNPVRRDVVNTGTAGDNVTIRFTTDNPGPWFLHCHIDFHLEAGFAIVFSEDTADVSNTT 499

Query 477 T-SDDWKNLCPTYNALSSDDI 496

TS W++LCPTYNALSD+

Sbjct 500 TPSTAWEDLCPTYNALDSSDL 520

RecName: Full=Laccase-4; AltName: Full=Benzenediol:oxygen oxidoreductase 4; AltName: Full=Diphenol oxidase 4; AltName:

Full=Urishiol oxidase 4; Flags: Precursor

Sequence ID: Q99055.1 Length: 520 Number of Matches: 1

See 1 more title(s)

Range 1: 20 to 520

Score

Expect Method

Identities

Positives

Gaps

Frame

743 bits(1917) 0.0()

Features:

Compositional matrix adjust. 361/501(72%) 415/501(82%) 25/501(4%)

Query 21

Sbjct 20

Query 64

LAATIGTVGDLTVSNKDISPDGYTRAAVVVNGQFPGPLIMGNR----------------- 63

++AIGVDLT+SND+SPDG+TRAAV+NGFPGPLIGN+

VSAAIGPVTDLTISNGDVSPDGFTRAAVLANGVFPGPLITGNKGDNFQINVIDNLSNETM 79

------HFHGIYQEGTNWEDGAAFVNQCPIATGNSFVYGFTCLDQAGTFWYHSHLSTQYC 117

H+HG+Q+GTNWDGAAFVNQCPIATGNSF+YFT DQAGTFWYHSHLSTQYC

Sbjct 80 LKSTSIHWHGFFQKGTNWADGAAFVNQCPIATGNSFLYDFTATDQAGTFWYHSHLSTQYC 139

https://blast.ncbi.nlm.nih.gov/Blast.cgi

5/6


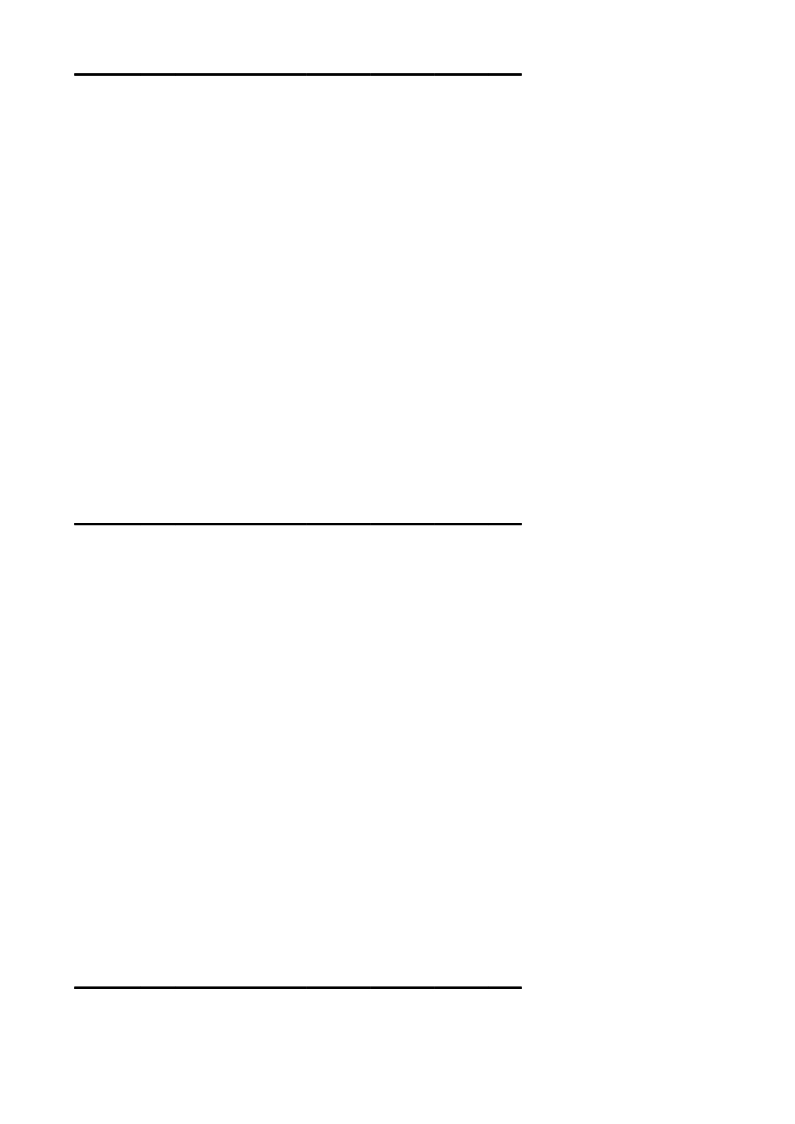


2017/4/9

Sbjct 80

NCBI Blast:GL18428-R1_1

LKSTSIHWHGFFQKGTNWADGAAFVNQCPIATGNSFLYDFTATDQAGTFWYHSHLSTQYC 139

Query 118 DGLRGPMVVYDPDDPHASLYDIDDESTVITLSDWYHTAARLGTGFP-RSDSVLINGLGRF 176

DGLRGPMVVYDPDPHALYD+DDE+T+ITLSDWYHTAALG FP SDSLINGLGRF

Sbjct 140 DGLRGPMVVYDPSDPHADLYDVDDETTIITLSDWYHTAASLGAAFPIGSDSTLINGLGRF 199

Query 177 AGGGSTDLAIIPVMRGKRYRFRLVSLSCDPNFTFSIDGHAMTVIEADAVNHEPLTVDSIQ 236

AGGSTDLA+IV+GKRYRRL+SLSCDPN+FSIDGHMT+IEADAVNHEPLTVDSIQ

Sbjct 200 AGGDSTDLAVITVEQGKRYRMRLLSLSCDPNYVFSIDGHNMTIIEADAVNHEPLTVDSIQ 259

Query 237 IFAGQRYSFVLTADQDIDNYWIRALPNRGTTNFDGGVNSAILRYDGAAAVEPTTNQSTSI 296

I+AGQRYSFVLTADQDIDNY+IRALP+GTT+FDGG+NSAILRYGA+V+PTT++TS+

Sbjct 260 IYAGQRYSFVLTADQDIDNYFIRALPSAGTTSFDGGINSAILRYSGASEVDPTTTETTSV 319

Query 297 APLAEADLMPLDRAPAPGDANRGGVDYALNLDFSVNGTRFFINGATFTPPSAPVLLQILS 356

PLEA+L+PLD APGDNGGVDYALNLDF++GTFFIN +F P+PVLLQILS

Sbjct 320 LPLDEANLVPLDSPAAPGDPNIGGVDYALNLDFNFDGTNFFINDVSFVSPTVPVLLQILS 379

Query 357 GTRSAADLLPSGSVYTIPANATVELSFPITSKNAPGAPHPFHLHGHTFSVVRSAGSNTYN 416

GTSAADLLPSGS+++P+N+T+E+SFPIT+NAPGAPHPFHLHGHTFS+VR+AGS N

Sbjct 380 GTTSAADLLPSGSLFAVPSNSTIEISFPITATNAPGAPHPFHLHGHTFSIVRTAGSTDTN 439

Query 417 YDSPPRRDVVNTGTAGDNVTIRFTTNNPGPWFLHCHIDFHLEAGFAVVFAVDTNTTSSFT 476

++PRRDVVNTGTGDNVTIRFTT+NPGPWFLHCHIDFHLEAGFA+VF+DT S+T

Sbjct 440 FVNPVRRDVVNTGTVGDNVTIRFTTDNPGPWFLHCHIDFHLEAGFAIVFSEDTADVSNTT 499

Query 477 T-SDDWKNLCPTYNALSSDDI 496

TS W++LCPTYNALSD+

Sbjct 500 TPSTAWEDLCPTYNALDSSDL 520

BLAST is a registered trademark of the National Library of Medicine

[Support center](https://support.ncbi.nlm.nih.gov/ics/support/KBList.asp?style=classic&deptID=28049&folderID=11&) [Mailing list](https://blast.ncbi.nlm.nih.gov/Blast.cgi?CMD=Web&PAGE_TYPE=BlastDocs&DOC_TYPE=MailList)

[YouTube](https://www.youtube.com/ncbinlm)

[National Library Of Medicine](https://www.nlm.nih.gov/)

[National Institutes Of Health](https://www.nih.gov/)

[U.S. Department of Health & Human Services](https://www.hhs.gov/)

[USA.gov](https://www.usa.gov/)

[NCBI](https://www.ncbi.nlm.nih.gov/)

[*National Center for Biotechnology Information,*](https://www.ncbi.nlm.nih.gov/) *U.S. National Library of Medicine 8600 Rock ville Pik e, Bethesda MD, 20894 USA*

[Policies and Guidelines](https://www.ncbi.nlm.nih.gov/home/about/policies.shtml) | [Contact](https://www.ncbi.nlm.nih.gov/home/about/contact.shtml)

https://blast.ncbi.nlm.nih.gov/Blast.cgi

6/6


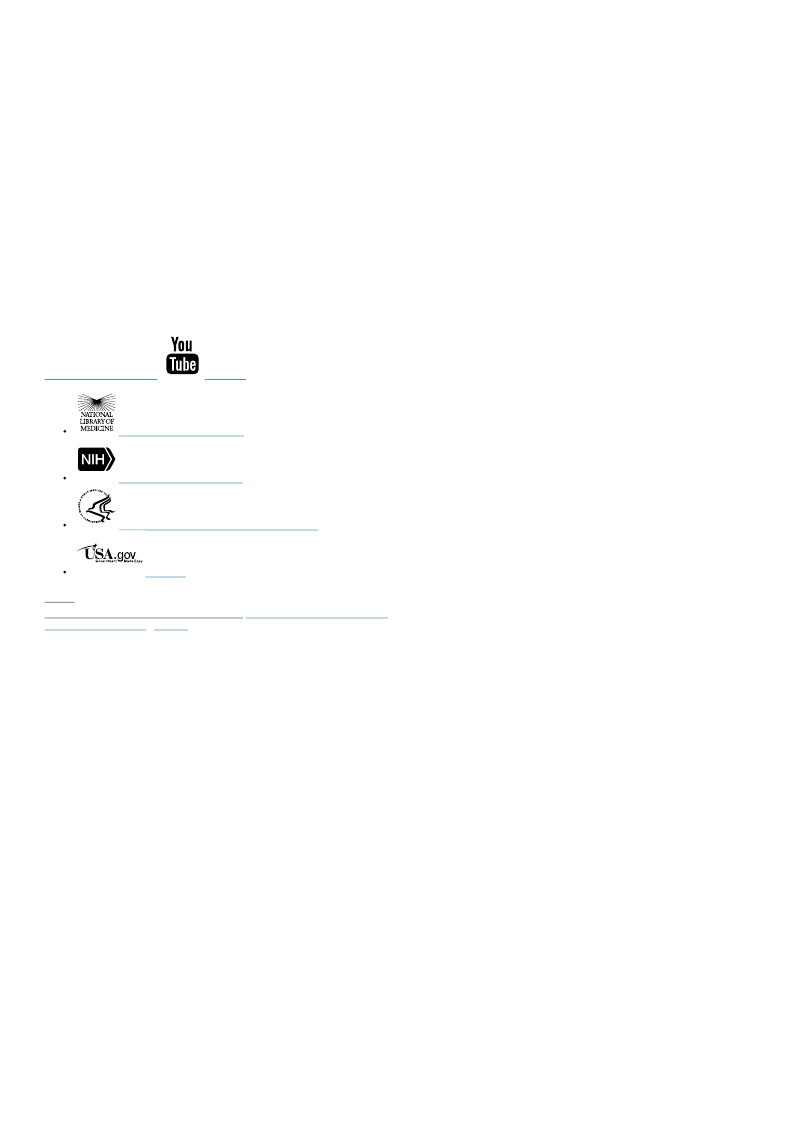

Supplement: Supplementary file 20 — Supplementary File 3e [file 41598_2017_4303_MOESM20_ESM.doc]
