# Supplementary material for: Genome-wide Identification and Characterization of Natural Antisense Transcripts by Strand-specific RNA Sequencing in Ganoderma lucidum
Source: Sci Rep. 2017 Jul 18;7:5711. doi: 10.1038/s41598-017-04303-6 (PMC5515960; doi:10.1038/s41598-017-04303-6)

2017/4/9

NCBI Blast:GL16401-R1_1

[BLAST ®](https://blast.ncbi.nlm.nih.gov/Blast.cgi) » blastp suite » RID-EMC5U3KB016

BLAST Results

Job title: GL16401-R1_1

RID

[EMC5U3KB016](https://blast.ncbi.nlm.nih.gov/Blast.cgi?CMD=Get&RID=EMC5U3KB016) (Expires on 04-10 21:01 pm)

Query ID

lcl|Query_100481

Database Name

nr

Description

Molecule type

Query Length

GL16401-R1_1

amino acid

529

Description All non-redundant GenBank CDS

translations+PDB+SwissProt+PIR+PRF excluding

environmental samples from WGS projects

Program BLASTP 2.6.0+

New Analyze your query with SmartBLAST

Graphic Summary

Putative conserved domains have been detected, click on the image below for detailed results.

Distribution of the top 100 Blast Hits on 100 subject sequences

Color key for alignment scores

<40

40-50

50-80

80-200

>=200

Query

1

100

200

300

400

500

https://blast.ncbi.nlm.nih.gov/Blast.cgi

1/6


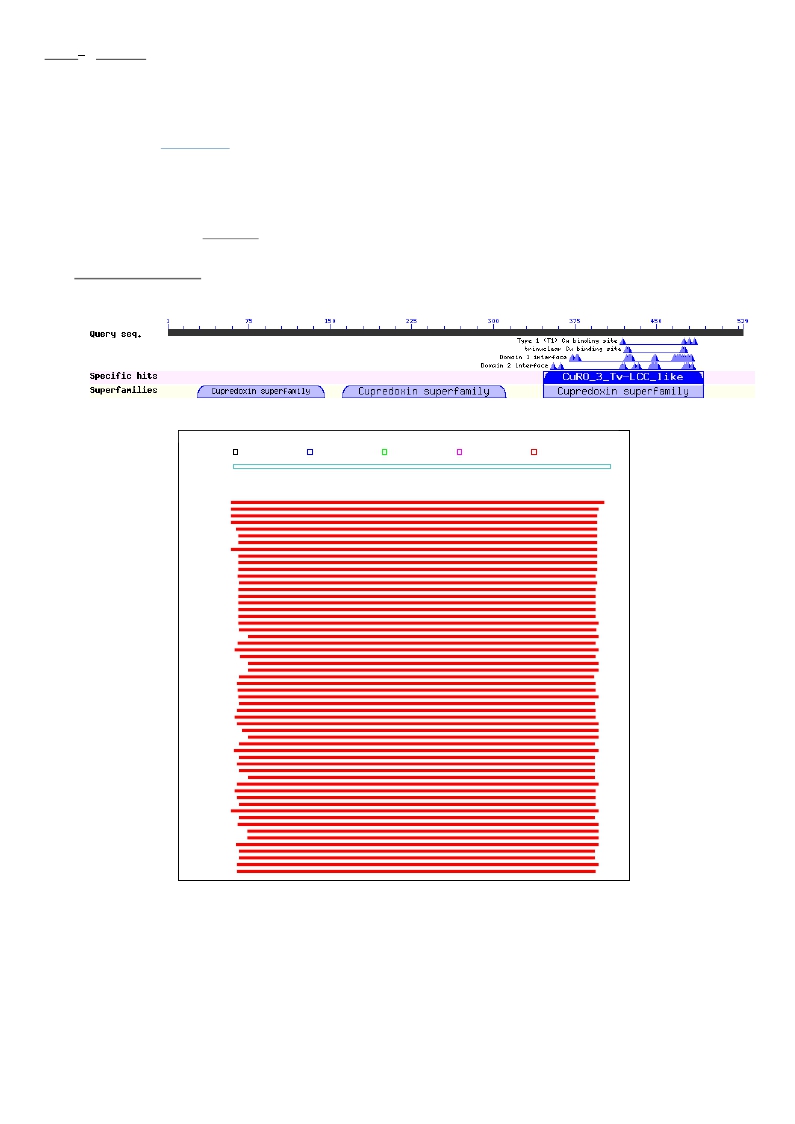


2017/4/9

Descriptions

Sequences producing significant alignments:

NCBI Blast:GL16401-R1_1

Description

[Max](https://blast.ncbi.nlm.nih.gov/Blast.cgi?CMD=Get&ALIGNMENTS=100&ALIGNMENT_VIEW=Pairwise&CDD_RID=EMC5UPYV013&CDD_SEARCH_STATE=0&DATABASE_SORT=0&DESCRIPTIONS=100&DYNAMIC_FORMAT=on&FIRST_QUERY_NUM=0&FORMAT_OBJECT=Alignment&FORMAT_PAGE_TARGET=&FORMAT_TYPE=HTML&GET_SEQUENCE=yes&I_THRESH=&LINE_LENGTH=60&MASK_CHAR=2&MASK_COLOR=1&NEW_VIEW=yes&NUM_OVERVIEW=100&PAGE=Proteins&QUERY_INDEX=0&QUERY_NUMBER=0&RESULTS_PAGE_TARGET=&RID=EMC5U3KB016&SHOW_LINKOUT=yes&SHOW_OVERVIEW=yes&STEP_NUMBER=&WORD_SIZE=6&OLD_VIEW=false&DISPLAY_SORT=1&HSP_SORT=1)

[Total](https://blast.ncbi.nlm.nih.gov/Blast.cgi?CMD=Get&ALIGNMENTS=100&ALIGNMENT_VIEW=Pairwise&CDD_RID=EMC5UPYV013&CDD_SEARCH_STATE=0&DATABASE_SORT=0&DESCRIPTIONS=100&DYNAMIC_FORMAT=on&FIRST_QUERY_NUM=0&FORMAT_OBJECT=Alignment&FORMAT_PAGE_TARGET=&FORMAT_TYPE=HTML&GET_SEQUENCE=yes&I_THRESH=&LINE_LENGTH=60&MASK_CHAR=2&MASK_COLOR=1&NEW_VIEW=yes&NUM_OVERVIEW=100&PAGE=Proteins&QUERY_INDEX=0&QUERY_NUMBER=0&RESULTS_PAGE_TARGET=&RID=EMC5U3KB016&SHOW_LINKOUT=yes&SHOW_OVERVIEW=yes&STEP_NUMBER=&WORD_SIZE=6&OLD_VIEW=false&DISPLAY_SORT=2&HSP_SORT=1)

[Query](https://blast.ncbi.nlm.nih.gov/Blast.cgi?CMD=Get&ALIGNMENTS=100&ALIGNMENT_VIEW=Pairwise&CDD_RID=EMC5UPYV013&CDD_SEARCH_STATE=0&DATABASE_SORT=0&DESCRIPTIONS=100&DYNAMIC_FORMAT=on&FIRST_QUERY_NUM=0&FORMAT_OBJECT=Alignment&FORMAT_PAGE_TARGET=&FORMAT_TYPE=HTML&GET_SEQUENCE=yes&I_THRESH=&LINE_LENGTH=60&MASK_CHAR=2&MASK_COLOR=1&NEW_VIEW=yes&NUM_OVERVIEW=100&PAGE=Proteins&QUERY_INDEX=0&QUERY_NUMBER=0&RESULTS_PAGE_TARGET=&RID=EMC5U3KB016&SHOW_LINKOUT=yes&SHOW_OVERVIEW=yes&STEP_NUMBER=&WORD_SIZE=6&OLD_VIEW=false&DISPLAY_SORT=4&HSP_SORT=0)

[E](https://blast.ncbi.nlm.nih.gov/Blast.cgi?CMD=Get&ALIGNMENTS=100&ALIGNMENT_VIEW=Pairwise&CDD_RID=EMC5UPYV013&CDD_SEARCH_STATE=0&DATABASE_SORT=0&DESCRIPTIONS=100&DYNAMIC_FORMAT=on&FIRST_QUERY_NUM=0&FORMAT_OBJECT=Alignment&FORMAT_PAGE_TARGET=&FORMAT_TYPE=HTML&GET_SEQUENCE=yes&I_THRESH=&LINE_LENGTH=60&MASK_CHAR=2&MASK_COLOR=1&NEW_VIEW=yes&NUM_OVERVIEW=100&PAGE=Proteins&QUERY_INDEX=0&QUERY_NUMBER=0&RESULTS_PAGE_TARGET=&RID=EMC5U3KB016&SHOW_LINKOUT=yes&SHOW_OVERVIEW=yes&STEP_NUMBER=&WORD_SIZE=6&OLD_VIEW=false&DISPLAY_SORT=0&HSP_SORT=0)

[Ident](https://blast.ncbi.nlm.nih.gov/Blast.cgi?CMD=Get&ALIGNMENTS=100&ALIGNMENT_VIEW=Pairwise&CDD_RID=EMC5UPYV013&CDD_SEARCH_STATE=0&DATABASE_SORT=0&DESCRIPTIONS=100&DYNAMIC_FORMAT=on&FIRST_QUERY_NUM=0&FORMAT_OBJECT=Alignment&FORMAT_PAGE_TARGET=&FORMAT_TYPE=HTML&GET_SEQUENCE=yes&I_THRESH=&LINE_LENGTH=60&MASK_CHAR=2&MASK_COLOR=1&NEW_VIEW=yes&NUM_OVERVIEW=100&PAGE=Proteins&QUERY_INDEX=0&QUERY_NUMBER=0&RESULTS_PAGE_TARGET=&RID=EMC5U3KB016&SHOW_LINKOUT=yes&SHOW_OVERVIEW=yes&STEP_NUMBER=&WORD_SIZE=6&DISPLAY_SORT=3&HSP_SORT=3)

Accession

[score](https://blast.ncbi.nlm.nih.gov/Blast.cgi?CMD=Get&ALIGNMENTS=100&ALIGNMENT_VIEW=Pairwise&CDD_RID=EMC5UPYV013&CDD_SEARCH_STATE=0&DATABASE_SORT=0&DESCRIPTIONS=100&DYNAMIC_FORMAT=on&FIRST_QUERY_NUM=0&FORMAT_OBJECT=Alignment&FORMAT_PAGE_TARGET=&FORMAT_TYPE=HTML&GET_SEQUENCE=yes&I_THRESH=&LINE_LENGTH=60&MASK_CHAR=2&MASK_COLOR=1&NEW_VIEW=yes&NUM_OVERVIEW=100&PAGE=Proteins&QUERY_INDEX=0&QUERY_NUMBER=0&RESULTS_PAGE_TARGET=&RID=EMC5U3KB016&SHOW_LINKOUT=yes&SHOW_OVERVIEW=yes&STEP_NUMBER=&WORD_SIZE=6&OLD_VIEW=false&DISPLAY_SORT=1&HSP_SORT=1)

[score](https://blast.ncbi.nlm.nih.gov/Blast.cgi?CMD=Get&ALIGNMENTS=100&ALIGNMENT_VIEW=Pairwise&CDD_RID=EMC5UPYV013&CDD_SEARCH_STATE=0&DATABASE_SORT=0&DESCRIPTIONS=100&DYNAMIC_FORMAT=on&FIRST_QUERY_NUM=0&FORMAT_OBJECT=Alignment&FORMAT_PAGE_TARGET=&FORMAT_TYPE=HTML&GET_SEQUENCE=yes&I_THRESH=&LINE_LENGTH=60&MASK_CHAR=2&MASK_COLOR=1&NEW_VIEW=yes&NUM_OVERVIEW=100&PAGE=Proteins&QUERY_INDEX=0&QUERY_NUMBER=0&RESULTS_PAGE_TARGET=&RID=EMC5U3KB016&SHOW_LINKOUT=yes&SHOW_OVERVIEW=yes&STEP_NUMBER=&WORD_SIZE=6&OLD_VIEW=false&DISPLAY_SORT=2&HSP_SORT=1)

[cover](https://blast.ncbi.nlm.nih.gov/Blast.cgi?CMD=Get&ALIGNMENTS=100&ALIGNMENT_VIEW=Pairwise&CDD_RID=EMC5UPYV013&CDD_SEARCH_STATE=0&DATABASE_SORT=0&DESCRIPTIONS=100&DYNAMIC_FORMAT=on&FIRST_QUERY_NUM=0&FORMAT_OBJECT=Alignment&FORMAT_PAGE_TARGET=&FORMAT_TYPE=HTML&GET_SEQUENCE=yes&I_THRESH=&LINE_LENGTH=60&MASK_CHAR=2&MASK_COLOR=1&NEW_VIEW=yes&NUM_OVERVIEW=100&PAGE=Proteins&QUERY_INDEX=0&QUERY_NUMBER=0&RESULTS_PAGE_TARGET=&RID=EMC5U3KB016&SHOW_LINKOUT=yes&SHOW_OVERVIEW=yes&STEP_NUMBER=&WORD_SIZE=6&OLD_VIEW=false&DISPLAY_SORT=4&HSP_SORT=0)

[value](https://blast.ncbi.nlm.nih.gov/Blast.cgi?CMD=Get&ALIGNMENTS=100&ALIGNMENT_VIEW=Pairwise&CDD_RID=EMC5UPYV013&CDD_SEARCH_STATE=0&DATABASE_SORT=0&DESCRIPTIONS=100&DYNAMIC_FORMAT=on&FIRST_QUERY_NUM=0&FORMAT_OBJECT=Alignment&FORMAT_PAGE_TARGET=&FORMAT_TYPE=HTML&GET_SEQUENCE=yes&I_THRESH=&LINE_LENGTH=60&MASK_CHAR=2&MASK_COLOR=1&NEW_VIEW=yes&NUM_OVERVIEW=100&PAGE=Proteins&QUERY_INDEX=0&QUERY_NUMBER=0&RESULTS_PAGE_TARGET=&RID=EMC5U3KB016&SHOW_LINKOUT=yes&SHOW_OVERVIEW=yes&STEP_NUMBER=&WORD_SIZE=6&OLD_VIEW=false&DISPLAY_SORT=0&HSP_SORT=0)

laccase [Ganoderma lucidum]

laccase [Ganoderma lucidum]

laccase C [Trametes sp. 420]

laccase B [Trametes sp. 420]

laccase D [Trametes hirsuta]

Laccase-3 [Trametes pubescens]

TvLac7 [Trametes versicolor FP-101664 SS1]

TvLac6 [Trametes versicolor FP-101664 SS1]

laccase D [Trametes ochracea]

laccase (EC 1.10.3.2) 3 precursor - white-rot fungus (Trametes

villosa)

laccase C [Trametes ochracea]

laccase B [Dichomitus squalens LYAD-421 SS1]

Laccase [Trametes cinnabarina]

multicopper oxidase [Phlebia tremellosa]

multiple oxidase [Phlebia tremellosa]

laccase [Coriolopsis gallica]

laccase [Phlebia chrysocreas]

laccase 1 [Steccherinum murashkinskyi]

laccase [Coriolopsis gallica]

laccase 3 [Coriolopsis trogii]

laccase [Phlebia chrysocreas]

laccase C [Trametes hirsuta]

laccase [Rigidoporus microporus]

laccase F [Trametes hirsuta]

Chain A, Coriolopsis Gallica Laccase T2 Copper Depleted At

Ph 4.5

Chain A, Coriolopsis Gallica Laccase Collected At 12.65 Kev

Laccase [Grifola frondosa]

multicopper redoxase [Trametes sanguinea]

laccase [Phlebia chrysocreas]

1078

750

697

686

671

646

641

636

635

635

634

630

619

615

613

613

608

607

607

599

599

599

599

598

597

597

597

597

596

1078

750

697

686

671

646

641

636

635

635

634

630

619

615

613

613

608

607

607

599

599

599

599

598

597

597

597

597

596

99%

98%

97%

97%

96%

95%

95%

97%

95%

95%

95%

95%

95%

95%

95%

95%

95%

95%

96%

95%

93%

95%

97%

94%

93%

93%

94%

95%

95%

0.0

0.0

0.0

0.0

0.0

0.0

0.0

0.0

0.0

0.0

0.0

0.0

0.0

0.0

0.0

0.0

0.0

0.0

0.0

0.0

0.0

0.0

0.0

0.0

0.0

0.0

0.0

0.0

0.0

99%

68%

65%

64%

65%

61%

63%

62%

62%

62%

62%

62%

61%

61%

61%

60%

62%

59%

60%

59%

60%

59%

58%

58%

59%

59%

59%

59%

61%

[AHA83587.1](https://www.ncbi.nlm.nih.gov/protein/558633457?report=genbank&log$=prottop&blast_rank=1&RID=EMC5U3KB016)

[AHA83590.1](https://www.ncbi.nlm.nih.gov/protein/558633463?report=genbank&log$=prottop&blast_rank=2&RID=EMC5U3KB016)

[AAW28938.1](https://www.ncbi.nlm.nih.gov/protein/56785446?report=genbank&log$=prottop&blast_rank=3&RID=EMC5U3KB016)

[AAW28937.1](https://www.ncbi.nlm.nih.gov/protein/56785444?report=genbank&log$=prottop&blast_rank=4&RID=EMC5U3KB016)

[AIZ72723.1](https://www.ncbi.nlm.nih.gov/protein/732554700?report=genbank&log$=prottop&blast_rank=5&RID=EMC5U3KB016)

[OJT13485.1](https://www.ncbi.nlm.nih.gov/protein/1112960306?report=genbank&log$=prottop&blast_rank=6&RID=EMC5U3KB016)

[XP_008038288.1](https://www.ncbi.nlm.nih.gov/protein/636614307?report=genbank&log$=prottop&blast_rank=7&RID=EMC5U3KB016)

[XP_008038233.1](https://www.ncbi.nlm.nih.gov/protein/636614197?report=genbank&log$=prottop&blast_rank=8&RID=EMC5U3KB016)

[ALT22027.1](https://www.ncbi.nlm.nih.gov/protein/965871874?report=genbank&log$=prottop&blast_rank=9&RID=EMC5U3KB016)

[JC5355](https://www.ncbi.nlm.nih.gov/protein/7432992?report=genbank&log$=prottop&blast_rank=10&RID=EMC5U3KB016)

[ALT22026.1](https://www.ncbi.nlm.nih.gov/protein/965871847?report=genbank&log$=prottop&blast_rank=11&RID=EMC5U3KB016)

[XP_007369195.1](https://www.ncbi.nlm.nih.gov/protein/598003325?report=genbank&log$=prottop&blast_rank=12&RID=EMC5U3KB016)

[CDO69696.1](https://www.ncbi.nlm.nih.gov/protein/691795569?report=genbank&log$=prottop&blast_rank=13&RID=EMC5U3KB016)

[CAK54346.1](https://www.ncbi.nlm.nih.gov/protein/109287624?report=genbank&log$=prottop&blast_rank=14&RID=EMC5U3KB016)

[CAR47803.1](https://www.ncbi.nlm.nih.gov/protein/194719800?report=genbank&log$=prottop&blast_rank=15&RID=EMC5U3KB016)

[AAF70119.2](https://www.ncbi.nlm.nih.gov/protein/12484399?report=genbank&log$=prottop&blast_rank=16&RID=EMC5U3KB016)

[ALF95042.1](https://www.ncbi.nlm.nih.gov/protein/930577645?report=genbank&log$=prottop&blast_rank=17&RID=EMC5U3KB016)

[AFI41888.1](https://www.ncbi.nlm.nih.gov/protein/385139612?report=genbank&log$=prottop&blast_rank=18&RID=EMC5U3KB016)

[AHM10329.1](https://www.ncbi.nlm.nih.gov/protein/594551748?report=genbank&log$=prottop&blast_rank=19&RID=EMC5U3KB016)

[AMJ39540.1](https://www.ncbi.nlm.nih.gov/protein/995953024?report=genbank&log$=prottop&blast_rank=20&RID=EMC5U3KB016)

[ALF95043.1](https://www.ncbi.nlm.nih.gov/protein/930577647?report=genbank&log$=prottop&blast_rank=21&RID=EMC5U3KB016)

[AIZ72722.1](https://www.ncbi.nlm.nih.gov/protein/732554698?report=genbank&log$=prottop&blast_rank=22&RID=EMC5U3KB016)

[ACL93333.1](https://www.ncbi.nlm.nih.gov/protein/255523026?report=genbank&log$=prottop&blast_rank=23&RID=EMC5U3KB016)

[AIZ72725.1](https://www.ncbi.nlm.nih.gov/protein/732554704?report=genbank&log$=prottop&blast_rank=24&RID=EMC5U3KB016)

[4A2D_A](https://www.ncbi.nlm.nih.gov/protein/350610907?report=genbank&log$=prottop&blast_rank=25&RID=EMC5U3KB016)

[4A2F_A](https://www.ncbi.nlm.nih.gov/protein/385251975?report=genbank&log$=prottop&blast_rank=26&RID=EMC5U3KB016)

[OBZ73717.1](https://www.ncbi.nlm.nih.gov/protein/1043286487?report=genbank&log$=prottop&blast_rank=27&RID=EMC5U3KB016)

[ACN69056.1](https://www.ncbi.nlm.nih.gov/protein/224924161?report=genbank&log$=prottop&blast_rank=28&RID=EMC5U3KB016)

[ALF95040.1](https://www.ncbi.nlm.nih.gov/protein/930577641?report=genbank&log$=prottop&blast_rank=29&RID=EMC5U3KB016)

RecName: Full=Laccase; AltName: Full=Benzenediol:oxygen

oxidoreductase; AltName: Full=Diphenol oxidase; AltName:

Full=Ligninolytic phenoloxidase; AltName: Full=Urishiol

596

596

96%

0.0

59%

[Q01679.2](https://www.ncbi.nlm.nih.gov/protein/47117883?report=genbank&log$=prottop&blast_rank=30&RID=EMC5U3KB016)

oxidase; Flags: Precursor

laccase [Trametes versicolor]

laccase 2 [Trametes pubescens]

laccase 2 [Trametes hirsuta]

laccase [Coriolopsis gallica]

hypothetical protein PLICRDRAFT_113151 [Plicaturopsis

crispa FD-325 SS-3]

596

596

596

596

596

596

596

596

596

596

94%

95%

96%

96%

95%

0.0

0.0

0.0

0.0

0.0

60%

59%

59%

57%

58%

[AFM31222.1](https://www.ncbi.nlm.nih.gov/protein/390980641?report=genbank&log$=prottop&blast_rank=31&RID=EMC5U3KB016)

[AAM18407.1](https://www.ncbi.nlm.nih.gov/protein/20270770?report=genbank&log$=prottop&blast_rank=32&RID=EMC5U3KB016)

[AOX15703.1](https://www.ncbi.nlm.nih.gov/protein/1083918173?report=genbank&log$=prottop&blast_rank=33&RID=EMC5U3KB016)

[AJV90967.1](https://www.ncbi.nlm.nih.gov/protein/768806945?report=genbank&log$=prottop&blast_rank=34&RID=EMC5U3KB016)

[KII87346.1](https://www.ncbi.nlm.nih.gov/protein/749762562?report=genbank&log$=prottop&blast_rank=35&RID=EMC5U3KB016)

Chain A, Crystallographic Structural Determination Of A

Trigonal Laccase From Coriolopsis Gallica (cgl) To 1.5 A

595

595

93%

0.0

59%

[5A7E_A](https://www.ncbi.nlm.nih.gov/protein/1032208307?report=genbank&log$=prottop&blast_rank=36&RID=EMC5U3KB016)

Resolution

RecName: Full=Laccase-2; AltName: Full=Benzenediol:oxygen

oxidoreductase 2; AltName: Full=Diphenol oxidase 2; AltName:

Full=Laccase I; AltName: Full=Urishiol oxidase 2; Flags:

595

595

94%

0.0

60%

[Q12718.1](https://www.ncbi.nlm.nih.gov/protein/2833233?report=genbank&log$=prottop&blast_rank=37&RID=EMC5U3KB016)

Precursor

laccase [Meripilus giganteus]

laccase I [Trametes versicolor]

laccase [Pycnoporus coccineus]

laccase I [Trametes versicolor FP-101664 SS1]

594

594

594

594

594

594

594

594

97%

94%

95%

94%

0.0

0.0

0.0

0.0

57%

60%

59%

60%

[CBV46340.1](https://www.ncbi.nlm.nih.gov/protein/317451550?report=genbank&log$=prottop&blast_rank=38&RID=EMC5U3KB016)

[AAC49828.1](https://www.ncbi.nlm.nih.gov/protein/1172163?report=genbank&log$=prottop&blast_rank=39&RID=EMC5U3KB016)

[AKE14488.1](https://www.ncbi.nlm.nih.gov/protein/810786215?report=genbank&log$=prottop&blast_rank=40&RID=EMC5U3KB016)

[XP_008032737.1](https://www.ncbi.nlm.nih.gov/protein/636603205?report=genbank&log$=prottop&blast_rank=41&RID=EMC5U3KB016)

Chain A, Crystal Structure Determination At Room Temperature

Of A Laccase From Trametes Versicolor In Its Oxidised Form

593

593

92%

0.0

61%

[1GYC_A](https://www.ncbi.nlm.nih.gov/protein/23200086?report=genbank&log$=prottop&blast_rank=42&RID=EMC5U3KB016)

Containing A Full Complement Of Copper Ions

laccase [Coriolopsis gallica]

laccase B [Trametes sp. AH28-2]

laccase A [Trametes ochracea]

593

593

593

593

593

593

96%

96%

95%

0.0

0.0

0.0

57%

58%

58%

[AJV90966.1](https://www.ncbi.nlm.nih.gov/protein/768806943?report=genbank&log$=prottop&blast_rank=43&RID=EMC5U3KB016)

[AAW31597.1](https://www.ncbi.nlm.nih.gov/protein/56809865?report=genbank&log$=prottop&blast_rank=44&RID=EMC5U3KB016)

[ALT22024.1](https://www.ncbi.nlm.nih.gov/protein/965871761?report=genbank&log$=prottop&blast_rank=45&RID=EMC5U3KB016)

https://blast.ncbi.nlm.nih.gov/Blast.cgi

2/6


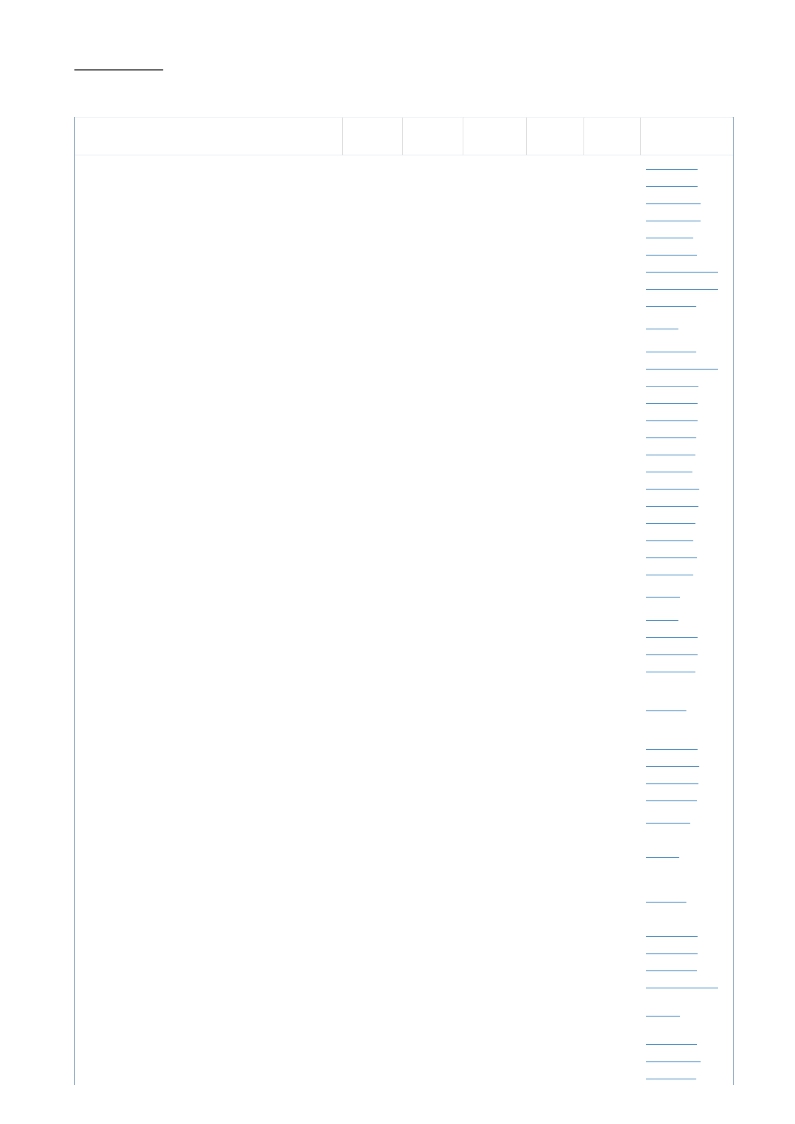


2017/4/9

NCBI Blast:GL16401-R1_1

laccase [Piloderma croceum F 1598]

laccase [Cerrena sp. WR1]

593

593

593

593

95%

98%

0.0

0.0

59%

57%

[KIM83705.1](https://www.ncbi.nlm.nih.gov/protein/751735413?report=genbank&log$=prottop&blast_rank=46&RID=EMC5U3KB016)

[ACZ58367.1](https://www.ncbi.nlm.nih.gov/protein/270047920?report=genbank&log$=prottop&blast_rank=47&RID=EMC5U3KB016)

RecName: Full=Laccase-2; AltName: Full=Benzenediol:oxygen

oxidoreductase 2; AltName: Full=Diphenol oxidase 2; AltName:

592

592

94%

0.0

59%

[Q99046.1](https://www.ncbi.nlm.nih.gov/protein/2842753?report=genbank&log$=prottop&blast_rank=48&RID=EMC5U3KB016)

Full=Urishiol oxidase 2; Flags: Precursor

laccase 2 [Coriolopsis caperata]

RecName: Full=Laccase-4; AltName: Full=Benzenediol:oxygen

oxidoreductase 4; AltName: Full=Diphenol oxidase 4; AltName:

592

592

592

592

96%

93%

0.0

0.0

57%

59%

[AGE13770.1](https://www.ncbi.nlm.nih.gov/protein/445065172?report=genbank&log$=prottop&blast_rank=49&RID=EMC5U3KB016)

[Q12719.1](https://www.ncbi.nlm.nih.gov/protein/2833234?report=genbank&log$=prottop&blast_rank=50&RID=EMC5U3KB016)

Full=Urishiol oxidase 4; Flags: Precursor

laccase-4 [Trametes versicolor FP-101664 SS1]

laccase [Cerrena sp. WR1]

Laccase-2 [Trametes pubescens]

laccase [Trametes versicolor]

laccase [Coriolopsis gallica]

592

592

592

592

592

592

592

592

592

592

93%

96%

94%

94%

96%

0.0

0.0

0.0

0.0

0.0

59%

59%

59%

59%

57%

[XP_008035965.1](https://www.ncbi.nlm.nih.gov/protein/636609661?report=genbank&log$=prottop&blast_rank=51&RID=EMC5U3KB016)

[ACZ58369.1](https://www.ncbi.nlm.nih.gov/protein/270047924?report=genbank&log$=prottop&blast_rank=52&RID=EMC5U3KB016)

[OJT12045.1](https://www.ncbi.nlm.nih.gov/protein/1112958732?report=genbank&log$=prottop&blast_rank=53&RID=EMC5U3KB016)

[AMT85330.1](https://www.ncbi.nlm.nih.gov/protein/1013824930?report=genbank&log$=prottop&blast_rank=54&RID=EMC5U3KB016)

[ABD93940.1](https://www.ncbi.nlm.nih.gov/protein/90436931?report=genbank&log$=prottop&blast_rank=55&RID=EMC5U3KB016)

RecName: Full=Laccase; AltName: Full=Benzenediol:oxygen

oxidoreductase; AltName: Full=Diphenol oxidase; AltName:

Full=Ligninolytic phenoloxidase; AltName: Full=Urishiol

592

592

95%

0.0

59%

[O59896.1](https://www.ncbi.nlm.nih.gov/protein/34922426?report=genbank&log$=prottop&blast_rank=56&RID=EMC5U3KB016)

oxidase; Flags: Precursor

laccase [Trametes sp. 48424]

hypothetical protein PLICRDRAFT_30715 [Plicaturopsis crispa

FD-325 SS-3]

laccase [Trametes velutina]

laccase 1 [Trametes versicolor]

laccase [Trametes cinnabarina]

592

591

591

590

590

592

591

591

590

590

95%

93%

94%

94%

95%

0.0

0.0

0.0

0.0

0.0

58%

58%

59%

59%

59%

[ADK55593.1](https://www.ncbi.nlm.nih.gov/protein/301070468?report=genbank&log$=prottop&blast_rank=57&RID=EMC5U3KB016)

[KII87344.1](https://www.ncbi.nlm.nih.gov/protein/749762560?report=genbank&log$=prottop&blast_rank=58&RID=EMC5U3KB016)

[ADE44157.1](https://www.ncbi.nlm.nih.gov/protein/293369943?report=genbank&log$=prottop&blast_rank=59&RID=EMC5U3KB016)

[AAW29420.1](https://www.ncbi.nlm.nih.gov/protein/56786630?report=genbank&log$=prottop&blast_rank=60&RID=EMC5U3KB016)

[AAN71597.1](https://www.ncbi.nlm.nih.gov/protein/25140399?report=genbank&log$=prottop&blast_rank=61&RID=EMC5U3KB016)

RecName: Full=Laccase-4; AltName: Full=Benzenediol:oxygen

oxidoreductase 4; AltName: Full=Diphenol oxidase 4; AltName:

590

590

93%

0.0

59%

[Q99055.1](https://www.ncbi.nlm.nih.gov/protein/2842755?report=genbank&log$=prottop&blast_rank=62&RID=EMC5U3KB016)

Full=Urishiol oxidase 4; Flags: Precursor

laccase [Pycnoporus coccineus]

laccase I [synthetic construct]

phenol oxidase [Pleurotus ostreatus]

laccase [Coriolopsis gallica]

laccase [Coriolopsis trogii]

laccase [Trametes versicolor]

laccase [Polyporus grammocephalus]

Chain A, Crystal Structure Of Lacb From Trametes Sp. Ah28-

2

laccase [Pycnoporus coccineus]

laccase LCC3-1 [Polyporus ciliatus]

laccase [Pycnoporus coccineus]

laccase [Ganoderma lucidum]

laccase [Piloderma croceum F 1598]

Chain A, T2-depleted Laccase From Coriolopsis Caperata

Soaked With Cucl

laccase [Trametes versicolor]

laccase [Trametes sp. I-62]

laccase [Trametes sp. I-62]

laccase 3 [Trametes hirsuta]

laccase B [Trametes ochracea]

laccase [synthetic construct]

phenoloxidase [Trametes sp. I-62]

laccase B [Trametes hirsuta]

laccase 2 [Steccherinum murashkinskyi]

Chain A, Crystal Structure Of Blue Laccase From Trametes

Trogii Complexed With P-Methylbenzoate

laccase [Trametes versicolor]

Laccase-2 [Grifola frondosa]

laccase [Dichomitus squalens LYAD-421 SS1]

laccase [Trametes sp. I-62]

phenoloxidase [Trametes sp. I-62]

laccase 1A [Trametes pubescens]

laccase B precursor [Trametes versicolor FP-101664 SS1]

laccase [Cerrena sp. WR1]

laccase [Coriolopsis rigida]

laccase [Trametes sp. I-62]

hypothetical protein PLICRDRAFT_115284 [Plicaturopsis

590

590

590

590

590

590

590

589

589

587

587

587

587

587

587

587

587

586

586

586

586

585

585

585

584

584

584

584

583

583

583

583

583

583

583

590

590

590

590

590

590

590

589

589

587

587

587

587

587

587

587

587

586

586

586

586

585

585

585

584

584

584

584

583

583

583

583

583

583

583

95%

92%

95%

96%

96%

94%

95%

92%

95%

97%

95%

95%

95%

93%

93%

93%

93%

95%

95%

92%

94%

96%

97%

93%

94%

92%

96%

94%

96%

93%

95%

98%

93%

96%

95%

0.0

0.0

0.0

0.0

0.0

0.0

0.0

0.0

0.0

0.0

0.0

0.0

0.0

0.0

0.0

0.0

0.0

0.0

0.0

0.0

0.0

0.0

0.0

0.0

0.0

0.0

0.0

0.0

0.0

0.0

0.0

0.0

0.0

0.0

0.0

59%

60%

57%

57%

57%

59%

60%

59%

59%

58%

58%

58%

59%

58%

59%

59%

59%

58%

59%

60%

58%

57%

59%

58%

59%

60%

58%

58%

57%

58%

58%

57%

57%

57%

57%

[BAB69775.1](https://www.ncbi.nlm.nih.gov/protein/16041065?report=genbank&log$=prottop&blast_rank=63&RID=EMC5U3KB016)

[AJP70335.1](https://www.ncbi.nlm.nih.gov/protein/761668396?report=genbank&log$=prottop&blast_rank=64&RID=EMC5U3KB016)

[CAR48257.1](https://www.ncbi.nlm.nih.gov/protein/198281884?report=genbank&log$=prottop&blast_rank=65&RID=EMC5U3KB016)

[ACS26245.1](https://www.ncbi.nlm.nih.gov/protein/239809556?report=genbank&log$=prottop&blast_rank=66&RID=EMC5U3KB016)

[CAC13040.1](https://www.ncbi.nlm.nih.gov/protein/10801036?report=genbank&log$=prottop&blast_rank=67&RID=EMC5U3KB016)

[AMT85329.1](https://www.ncbi.nlm.nih.gov/protein/1013824928?report=genbank&log$=prottop&blast_rank=68&RID=EMC5U3KB016)

[ACR24358.1](https://www.ncbi.nlm.nih.gov/protein/237861577?report=genbank&log$=prottop&blast_rank=69&RID=EMC5U3KB016)

[3KW7_A](https://www.ncbi.nlm.nih.gov/protein/290790140?report=genbank&log$=prottop&blast_rank=70&RID=EMC5U3KB016)

[BAB69776.1](https://www.ncbi.nlm.nih.gov/protein/16041067?report=genbank&log$=prottop&blast_rank=71&RID=EMC5U3KB016)

[AAG09229.1](https://www.ncbi.nlm.nih.gov/protein/9957143?report=genbank&log$=prottop&blast_rank=72&RID=EMC5U3KB016)

[AKE14489.1](https://www.ncbi.nlm.nih.gov/protein/810786240?report=genbank&log$=prottop&blast_rank=73&RID=EMC5U3KB016)

[AHA83591.1](https://www.ncbi.nlm.nih.gov/protein/558633465?report=genbank&log$=prottop&blast_rank=74&RID=EMC5U3KB016)

[KIM83693.1](https://www.ncbi.nlm.nih.gov/protein/751735401?report=genbank&log$=prottop&blast_rank=75&RID=EMC5U3KB016)

[4JHU_A](https://www.ncbi.nlm.nih.gov/protein/635576677?report=genbank&log$=prottop&blast_rank=76&RID=EMC5U3KB016)

[ADI70681.1](https://www.ncbi.nlm.nih.gov/protein/298239752?report=genbank&log$=prottop&blast_rank=77&RID=EMC5U3KB016)

[AAQ12268.1](https://www.ncbi.nlm.nih.gov/protein/33334369?report=genbank&log$=prottop&blast_rank=78&RID=EMC5U3KB016)

[AAQ12267.1](https://www.ncbi.nlm.nih.gov/protein/33334367?report=genbank&log$=prottop&blast_rank=79&RID=EMC5U3KB016)

[AOX15704.1](https://www.ncbi.nlm.nih.gov/protein/1083918175?report=genbank&log$=prottop&blast_rank=80&RID=EMC5U3KB016)

[ALT22025.1](https://www.ncbi.nlm.nih.gov/protein/965871806?report=genbank&log$=prottop&blast_rank=81&RID=EMC5U3KB016)

[AER26914.1](https://www.ncbi.nlm.nih.gov/protein/354508491?report=genbank&log$=prottop&blast_rank=82&RID=EMC5U3KB016)

[AAB63445.1](https://www.ncbi.nlm.nih.gov/protein/2264400?report=genbank&log$=prottop&blast_rank=83&RID=EMC5U3KB016)

[AIZ72727.1](https://www.ncbi.nlm.nih.gov/protein/732554708?report=genbank&log$=prottop&blast_rank=84&RID=EMC5U3KB016)

[AFI41889.1](https://www.ncbi.nlm.nih.gov/protein/385141759?report=genbank&log$=prottop&blast_rank=85&RID=EMC5U3KB016)

[2HRG_A](https://www.ncbi.nlm.nih.gov/protein/158428663?report=genbank&log$=prottop&blast_rank=86&RID=EMC5U3KB016)

[AMT85333.1](https://www.ncbi.nlm.nih.gov/protein/1013824936?report=genbank&log$=prottop&blast_rank=87&RID=EMC5U3KB016)

[OBZ65445.1](https://www.ncbi.nlm.nih.gov/protein/1043278079?report=genbank&log$=prottop&blast_rank=88&RID=EMC5U3KB016)

[XP_007364547.1](https://www.ncbi.nlm.nih.gov/protein/597985483?report=genbank&log$=prottop&blast_rank=89&RID=EMC5U3KB016)

[AAQ12270.1](https://www.ncbi.nlm.nih.gov/protein/33334373?report=genbank&log$=prottop&blast_rank=90&RID=EMC5U3KB016)

[AAB63444.1](https://www.ncbi.nlm.nih.gov/protein/2264398?report=genbank&log$=prottop&blast_rank=91&RID=EMC5U3KB016)

[AAM18408.1](https://www.ncbi.nlm.nih.gov/protein/20270772?report=genbank&log$=prottop&blast_rank=92&RID=EMC5U3KB016)

[XP_008032614.1](https://www.ncbi.nlm.nih.gov/protein/636602959?report=genbank&log$=prottop&blast_rank=93&RID=EMC5U3KB016)

[ACZ58368.1](https://www.ncbi.nlm.nih.gov/protein/270047922?report=genbank&log$=prottop&blast_rank=94&RID=EMC5U3KB016)

[ADK13098.1](https://www.ncbi.nlm.nih.gov/protein/300433312?report=genbank&log$=prottop&blast_rank=95&RID=EMC5U3KB016)

[AAQ12269.1](https://www.ncbi.nlm.nih.gov/protein/33334371?report=genbank&log$=prottop&blast_rank=96&RID=EMC5U3KB016)

[KII86082.1](https://www.ncbi.nlm.nih.gov/protein/749761180?report=genbank&log$=prottop&blast_rank=97&RID=EMC5U3KB016)

https://blast.ncbi.nlm.nih.gov/Blast.cgi

3/6


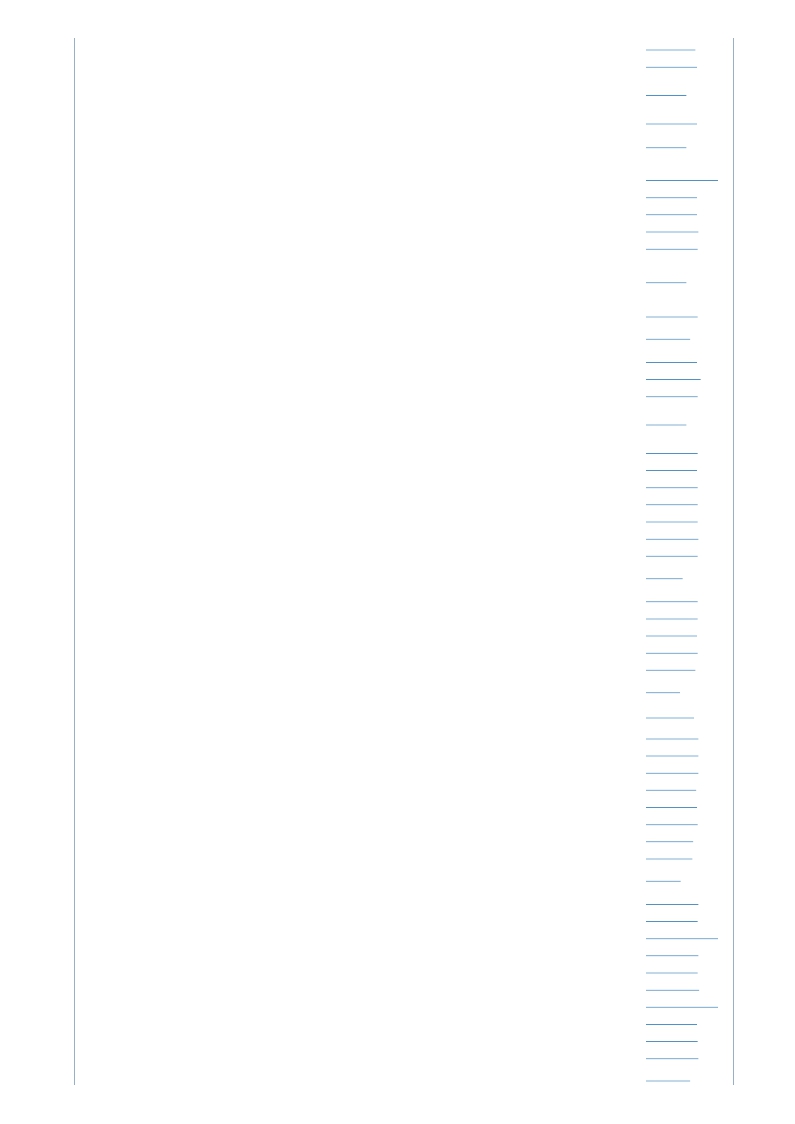


2017/4/9

crispa FD-325 SS-3]

NCBI Blast:GL16401-R1_1

laccase protein [Trametes versicolor]

laccase [Coriolopsis rigida]

polyphenoloxidase [Trametes sp. C30]

583

583

582

583

583

582

94%

93%

96%

0.0

0.0

0.0

59%

57%

57%

[ACK77785.1](https://www.ncbi.nlm.nih.gov/protein/218511586?report=genbank&log$=prottop&blast_rank=98&RID=EMC5U3KB016)

[ACU29545.1](https://www.ncbi.nlm.nih.gov/protein/255918284?report=genbank&log$=prottop&blast_rank=99&RID=EMC5U3KB016)

[AAF06967.1](https://www.ncbi.nlm.nih.gov/protein/6318611?report=genbank&log$=prottop&blast_rank=100&RID=EMC5U3KB016)

Alignments

laccase, partial [Ganoderma lucidum]

Sequence ID: AHA83587.1 Length: 535 Number of Matches: 1

Range 1: 1 to 535

Score

Expect Method

Identities

Positives

Gaps

Frame

1078 bits(2788) 0.0()

Features:

Compositional matrix adjust. 528/535(99%) 528/535(98%) 7/535(1%)

Query 1

Sbjct 1

Query 61

Sbjct 61

MVMGNSCTAVVALILATIAQPTRAAIGPVTDLAVVNKPVSTDGTSRNAVLADGTFPGPVI 60

MVMGNSCTAVVALILATIAQPTRAAIGPVTDLAVVNKPVSTDGTSRNAVLADGTFPGPVI

MVMGNSCTAVVALILATIAQPTRAAIGPVTDLAVVNKPVSTDGTSRNAVLADGTFPGPVI 60

AGYSGDYFNINVKDELYNTTMLTGTSIH-------TTNWADGASFINQCPITSGNCFEYE 113

AGYSGDYFNINVKDELYNTTMLTGTSIH TTNWADGASFINQCPITSGNCFEYE

AGYSGDYFNINVKDELYNTTMLTGTSIHWHGLLQHTTNWADGASFINQCPITSGNCFEYE 120

Query 114 FETTGISGTYWYHSHLGNQYCDGLRGPLVLYDKHDPHAHLYDVDDGITIITLADWYHLAS 173

FETTGISGTYWYHSHLGNQYCDGLRGPLVLYDKHDPHAHLYDVDDGITIITLADWYHLAS

Sbjct 121 FETTGISGTYWYHSHLGNQYCDGLRGPLVLYDKHDPHAHLYDVDDGITIITLADWYHLAS 180

Query 174 PFVLTRGGPPRADSNLINGLGRWAGNPTAELAVIKVTHGKRYRFRLINIACDPNYNFTIA 233

PFVLTRGGPPRADSNLINGLGRWAGNPTAELAVIKVTHGKRYRFRLINIACDPNYNFTIA

Sbjct 181 PFVLTRGGPPRADSNLINGLGRWAGNPTAELAVIKVTHGKRYRFRLINIACDPNYNFTIA 240

Query 234 GHSMTIIEADGQNSEPLVVDELQIFVAQRYSFVLEANQPVNNYWIRALPDIMAENSSLGY 293

GHSMTIIEADGQNSEPLVVDELQIFVAQRYSFVLEANQPVNNYWIRALPDIMAENSSLGY

Sbjct 241 GHSMTIIEADGQNSEPLVVDELQIFVAQRYSFVLEANQPVNNYWIRALPDIMAENSSLGY 300

Query 294 AHGINSAILRYEGAPAEEPCEQEAKSVNPLREYNLHSLEDPAAPGEPYVGGVDYALNLVL 353

AHGINSAILRYEGAPAEEPCEQEAKSVNPLREYNLHSLEDPAAPGEPYVGGVDYALNLVL

Sbjct 301 AHGINSAILRYEGAPAEEPCEQEAKSVNPLREYNLHSLEDPAAPGEPYVGGVDYALNLVL 360

Query 354 GFNNASKTPFNINGVPFQSPSVPVLLQILSGAKKAQDLLPAGSVYGLPRNSSIELSIQPL 413

GFNNASKTPFNINGVPFQSPSVPVLLQILSGAKKAQDLLPAGSVYGLPRNSSIELSIQPL

Sbjct 361 GFNNASKTPFNINGVPFQSPSVPVLLQILSGAKKAQDLLPAGSVYGLPRNSSIELSIQPL 420

Query 414 SIGGPHPFHLHGHAFSVVRGAGQAVPNYANPIKRDVVSTGFPGDNVTIRFRTDNPGPWLL 473

SIGGPHPFHLHGHAFSVVRGAGQAVPNYANPIKRDVVSTGFPGDNVTIRFRTDNPGPWLL

Sbjct 421 SIGGPHPFHLHGHAFSVVRGAGQAVPNYANPIKRDVVSTGFPGDNVTIRFRTDNPGPWLL 480

Query 474 HCHIDWHLSGGLAIVFAEDVGDTSFVDPAPKEWYDLCPEYEASIANDPTHTQPNP 528

HCHIDWHLSGGLAIVFAEDVGDTSFVDPAPKEWYDLCPEYEASIANDPTHTQPNP

Sbjct 481 HCHIDWHLSGGLAIVFAEDVGDTSFVDPAPKEWYDLCPEYEASIANDPTHTQPNP 535

laccase, partial [Ganoderma lucidum]

Sequence ID: AHA83590.1 Length: 529 Number of Matches: 1

Range 1: 1 to 529

Score

Expect Method

Identities

Positives

Gaps

Frame

750 bits(1936) 0.0()

Features:

Compositional matrix adjust. 359/529(68%) 420/529(79%) 10/529(1%)

Query 1

Sbjct 1

Query 61

Sbjct 61

MVMGNSCTAVVALILATIAQPTRAAIGPVTDLAVVNKPVSTDGTSRNAVLADGTFPGPVI 60

M GNS V ++LA++A+TAIGPVDL+VNK++DGSRNAVLAGTFPGP+I

MAKGNSFIRVAFVLLASLARRTSAQIGPVADLRIVNKVIAPDGFSRNAVLAGGTFPGPLI 60

AGYSGDYFNINVKDELYNTTMLTGTSIH-------TTNWADGASFINQCPITSGNCFEYE 113

+G+SGDFINVKDEL+++MLTTSIH TNWADG+F++QCPIT+GNFY+

SGHSGDTFKINVKDELTDSSMLTPTSIHWHGILQHETNWADGGAFVSQCPITTGNSFRYK 120

Query 114 FETTGISGTYWYHSHLGNQYCDGLRGPLVLYDKHDPHAHLYDVDDGITIITLADWYHLAS 173

F+TGI+GTYWYHSHL+QYCDGLRGPL+LYDK+DPH LYDVDD T+ITLADWYH++

Sbjct 121 FDTKGIAGTYWYHSHLASQYCDGLRGPLILYDKNDPHKDLYDVDDESTVITLADWYHVIA 180

Query 174 PFVLTRGGPPRADSNLINGLGRWAGNPTAELAVIKVTHGKRYRFRLINIACDPNYNFTIA 233

P+ R P+DSLINGLGRW NPTELAVIKVTGKRYRFR+++AC NYNFTIA

Sbjct 181 PLLPARAVVPASDSTLINGLGRWFANPTTELAVIKVTQGKRYRFRMVSTACHANYNFTIA 240

Query 234 GHSMTIIEADGQNSEPLVVDELQIFVAQRYSFVLEANQPVNNYWIRALPDIMAENSSLGY 293

GH+T+IEADGQN++PLVDE+QIF QRYSFVLEAN+P++NYWIALP++M S+LG

Sbjct 241 GHDLTVIEADGQNTKPLTVDEIQIFAGQRYSFVLEANRPIDNYWIHALPNLMYNTSALGT 300

Query 294 AHGINSAILRYEGAPAEEPCEQEAKSVNPLREYNLHSLEDPAAPGEPYVGGVDYALNLVL 353

A+GINSAILRYEGAPEEP EKS+NPLRE+NL LEDPAAPGEP+GGVD NL+

Sbjct 301 ANGINSAILRYEGAPEEEPRHLEVKSLNPLREWNLRPLEDPAAPGEPHPGGVDIVYNLDV 360

Query 354 GF---NNASKTPFNINGVPFQSPSVPVLLQILSGAKKAQDLLPAGSVYGLPRNSSIELSI 410

GF NNSTFINVF+PS+PVLLQILG+AQDLLPGS+YLRN+++EL+I

Sbjct 361 GFRPRNNTSPTRFTINNVTFEPPSIPVLLQILGGVQLAQDLLPQGSIYPLRRNATVELTI 420

Query 411 QPLSIGGPHPFHLHGHAFSVVRGAGQAVPNYANPIKRDVVSTGFPGDNVTIRFRTDNPGP 470

+GGPHPFHLHGHFSVVRAGQAVPNY+P+KRDVVSGPGDNVTIRF DNPGP

Sbjct 421 PGTLVGGPHPFHLHGHTFSVVRSAGQAVPNYVDPVKRDVVSVGVPGDNVTIRFTADNPGP 480

Query 471 WLLHCHIDWHLSGGLAIVFAEDVGDTSFVDPAPKEWYDLCPEYEASIAN 519

WLHCHIDWHL GLAIVFAEDV SFVDPAP+EWYDLCPEYES+A

Sbjct 481 WFLHCHIDWHLEAGLAIVFAEDVPSVSFVDPAPEEWYDLCPEYEESLAR 529

laccase C [Trametes sp. 420]

Sequence ID: AAW28938.1 Length: 519 Number of Matches: 1

Range 1: 1 to 517

https://blast.ncbi.nlm.nih.gov/Blast.cgi

4/6


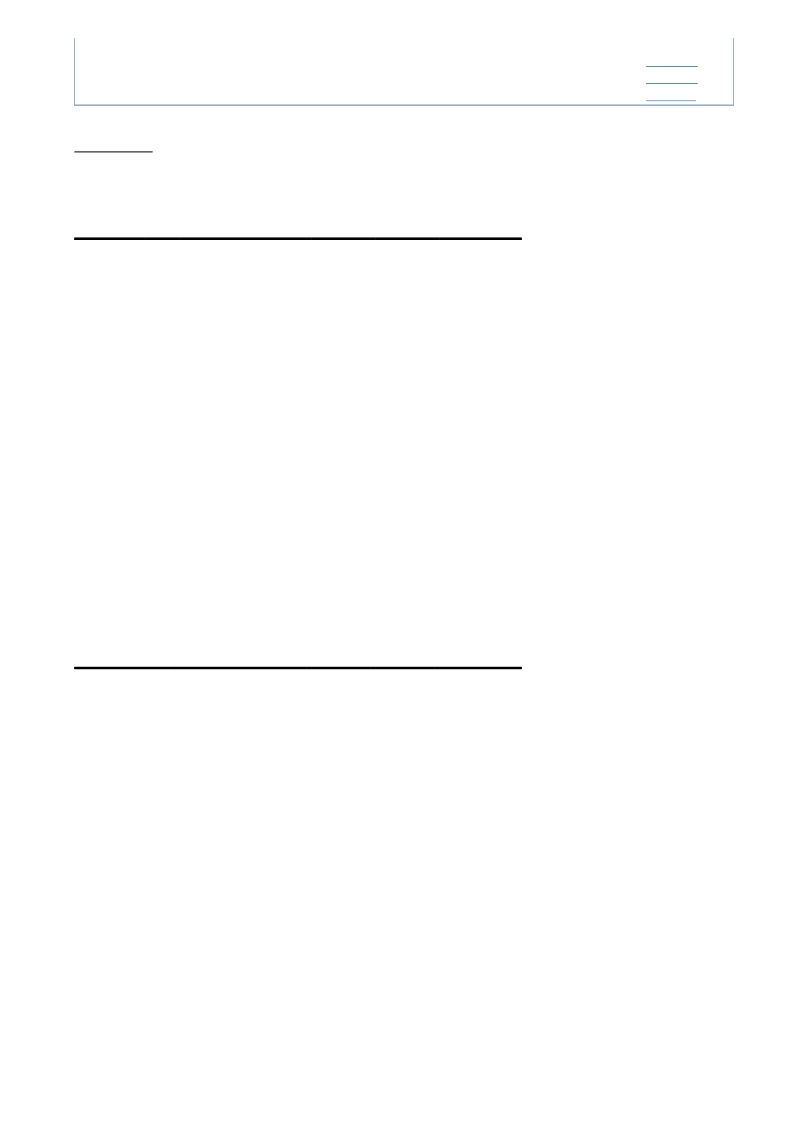


2017/4/9

NCBI Blast:GL16401-R1_1

Score

Expect Method

Identities

Positives

Gaps

Frame

697 bits(1799) 0.0()

Features:

Compositional matrix adjust. 339/524(65%) 402/524(76%) 14/524(2%)

Query 1

Sbjct 1

Query 61

Sbjct 61

MVMGNSCTAVVALILATIAQPTRAAIGPVTDLAVVNKPVSTDGTSRNAVLADGTFPGPVI 60

M+ S + L++ +A +AAIGPVTDL+VN +SDG+R+AVLADGTFPGP+I

MLQLTSFRRLSLLLVTALATRSFAAIGPVTDLNIVNANISPDGFARDAVLADGTFPGPLI 60

AGYSGDYFNINVKDELYNTTMLTGTSIH-------TTNWADGASFINQCPITSGNCFEYE 113

G DFINVD+LNTTMLTTSIH TNWADG F+NQCPISGNFY

TGQKNDNFQINVVDKLTNTTMLTATSIHWHGFFQHGTNWADGVPFLNQCPIVSGNSFLYN 120

Query 114 FETTGISGTYWYHSHLGNQYCDGLRGPLVLYDKHDPHAHLYDVDDGITIITLADWYHLAS 173

F +GT+WYHSHL QYCDGLRGLV+YD DPHALYDVDD T+ITLADWYH+A+

Sbjct 121 FNVPDQAGTFWYHSHLNTQYCDGLRGALVVYDPADPHAGLYDVDDESTVITLADWYHVAA 180

Query 174 PFVLTRGGPPRADSNLINGLGRWAGNPTAELAVIKVTHGKRYRFRLINIACDPNYNFTIA 233

P G PR+D+LINGLGRW+G+PT+ELAVIVTGKRYRFRL++++CDPNYFI

Sbjct 181 PLA---GAVPRSDATLINGLGRWSGDPTSELAVINVTPGKRYRFRLVSMSCDPNYTFQID 237

Query 234 GHSMTIIEADGQNSEPLVVDELQIFVAQRYSFVLEANQPVNNYWIRALPDIMAENSSLGY 293

GHSMT+IEADGQN+EPLVD++QIF QRYSFVLEANQVNYWIRAP+++A+ SG+

Sbjct 238 GHSMTVIEADGQNTEPLPVDQIQIFAGQRYSFVLEANQTVGNYWIRASPNLVADGGS-GF 296

Query 294 AHGINSAILRYEGAPAEEPCEQEAKSVNPLREYNLHSLEDPAAPGEPYVGGVDYALNLVL 353

AHGINSAILRY+GAPEEP + S+NPLRE++LHLDPAAPGP GV+ +NL+

Sbjct 297 AHGINSAILRYDGAPEEEPTTTQDTSINPLREFDLHPLTDPAAPGNPTEGDVEVPINLAI 356

Query 354 GFNNASKTPFNINGVPFQSPSVPVLLQILSGAKKAQDLLPAGSVYGLPRNSSIELSIQPL 413

GF+ + F+NG F+SPSVPVLLQILSGA+AQDLLP+GSVYLPN+++ELSI

Sbjct 357 GFSGGN---FTVNGTTFESPSVPVLLQILSGAQNAQDLLPSGSVYSLPSNATVELSIPAF 413

Query 414 SIGGPHPFHLHGHAFSVVRGAGQAVPNYANPIKRDVVSTGFPGDNVTIRFRTDNPGPWLL 473

+IGGPHPFHLHGHAFSVVRAGQ PNYNP++RDVVSG DNVTIRFRTDNPGPWL

Sbjct 414 AIGGPHPFHLHGHAFSVVRSAGQTEPNYVNPVRRDVVSIGSGTDNVTIRFRTDNPGPWFL 473

Query 474 HCHIDWHLSGGLAIVFAEDVGDTSFVDPAPKEWYDLCPEYEASI 517

HCHIDWHL GLAIVFAED++T+ +PP+WDLCPEYEAS+

Sbjct 474 HCHIDWHLQAGLAIVFAEDIPETAATNPVPQAWSDLCPEYEASL 517

laccase B [Trametes sp. 420]

Sequence ID: AAW28937.1 Length: 519 Number of Matches: 1

Range 1: 1 to 517

Score

Expect Method

Identities

Positives

Gaps

Frame

686 bits(1770) 0.0()

Features:

Compositional matrix adjust. 334/524(64%) 397/524(75%) 14/524(2%)

Query 1

Sbjct 1

Query 61

Sbjct 61

MVMGNSCTAVVALILATIAQPTRAAIGPVTDLAVVNKPVSTDGTSRNAVLADGTFPGPVI 60

M+ S + + +A TAAIGPVTDL+VN +SDG+R+AVLA+GTFPGP+I

MLQLTSFRRLSLFLFTALATRTFAAIGPVTDLNIVNANISPDGFARDAVLAEGTFPGPLI 60

AGYSGDYFNINVKDELYNTTMLTGTSIH-------TTNWADGASFINQCPITSGNCFEYE 113

G DFINVD+LNTTMLTTSIH TNWADG F+NQCPISGNFY

TGQKNDNFRINVVDKLTNTTMLTATSIHWHGFFQHGTNWADGVPFLNQCPIVSGNSFLYN 120

Query 114 FETTGISGTYWYHSHLGNQYCDGLRGPLVLYDKHDPHAHLYDVDDGITIITLADWYHLAS 173

F +GT+WYHSHL QYCDGLRGLV+YD DPHALYDVD+ T+ITLADWYH+A+

Sbjct 121 FNVPDQAGTFWYHSHLNTQYCDGLRGALVVYDPADPHASLYDVDEESTVITLADWYHVAA 180

Query 174 PFVLTRGGPPRADSNLINGLGRWAGNPTAELAVIKVTHGKRYRFRLINIACDPNYNFTIA 233

P G PR+D+LINGLGRW+G+PT+ELAIVTGKRYRFRL++++CDPNYFI

Sbjct 181 PLA---GAVPRSDATLINGLGRWSGDPTSELAAINVTPGKRYRFRLVSMSCDPNYTFQID 237

Query 234 GHSMTIIEADGQNSEPLVVDELQIFVAQRYSFVLEANQPVNNYWIRALPDIMAENSSLGY 293

GHSMT+IEADGQN+EPLVD++QIF QRYSFVLEANQVNYWIRAP+++A+ SG+

Sbjct 238 GHSMTVIEADGQNTEPLPVDQIQIFAGQRYSFVLEANQTVGNYWIRASPNLVADGGS-GF 296

Query 294 AHGINSAILRYEGAPAEEPCEQEAKSVNPLREYNLHSLEDPAAPGEPYVGGVDYALNLVL 353

A+GINSAILR+GAPEEP + S+NPLRE++LHLDPAAPGP GV +NL+

Sbjct 297 ANGINSAILRCDGAPEEEPTTTQDTSINPLREFDLHPLTDPAAPGNPTEGDVGVPINLAI 356

Query 354 GFNNASKTPFNINGVPFQSPSVPVLLQILSGAKKAQDLLPAGSVYGLPRNSSIELSIQPL 413

GF+ + F+NG F+SPSVPVLLQILSGA+AQDLLP+GSVYLPN+++ELSI

Sbjct 357 GFSGGN---FTVNGTTFESPSVPVLLQILSGAQNAQDLLPSGSVYSLPSNATVELSIPAF 413

Query 414 SIGGPHPFHLHGHAFSVVRGAGQAVPNYANPIKRDVVSTGFPGDNVTIRFRTDNPGPWLL 473

+IGGPHPFHLHGHAFSVVRAGQ PNYNP++RDVVSG DNVTIRFRTDNPGPWL

Sbjct 414 AIGGPHPFHLHGHAFSVVRSAGQTEPNYVNPVRRDVVSIGSGTDNVTIRFRTDNPGPWFL 473

Query 474 HCHIDWHLSGGLAIVFAEDVGDTSFVDPAPKEWYDLCPEYEASI 517

HCHIDWHL GLAIVFAED++T+ +PP+WDLCPEYEAS+

Sbjct 474 HCHIDWHLQAGLAIVFAEDIPETAATNPVPQAWSDLCPEYEASL 517

laccase D [Trametes hirsuta]

Sequence ID: AIZ72723.1 Length: 523 Number of Matches: 1

Range 1: 8 to 519

Score

Expect Method

Identities

Positives

Gaps

Frame

671 bits(1730) 0.0()

Features:

Compositional matrix adjust. 338/519(65%) 390/519(75%) 16/519(3%)

Query 8

Sbjct 8

Query 68

Sbjct 65

TAVVALILATIAQPTRAAIGPVTDLAVVNKPVSTDGTSRNAVLADGTFPGPVIAGYSGDY 67

TAV LL A AAIGPVTDL+VNK++DG R+VLAGTFPGP++G GD+

TAVTILGLCGAAM---AAIGPVTDLDIVNKEIAPDGLLRDTVLAGGTFPGPLVQGKKGDH 64

FNINVKDELYNTTMLTGTSIH-------TTNWADGASFINQCPITSGNCFEYEFETTGIS 120

FINVD+LNTMLTT+IH TTNWADG+F+QCPI+GNFYF+ +

FKINVVDKLTNETMLTSTTIHWHGLFQHTTNWADGPAFVTQCPIIAGNDFLYNFQVPDQT 124

Query 121 GTYWYHSHLGNQYCDGLRGPLVLYDKHDPHAHLYDVDDGITIITLADWYHLASPFVLTRG 180

GTYWYHSHL QYCDGLRGPLV+YDHDPHHLYDVDD T+ITLADWYHA+P

Sbjct 125 GTYWYHSHLATQYCDGLRGPLVIYDPHDPHKHLYDVDDESTVITLADWYHTAAPIEANGP 184

Query 181 GPPRADSNLINGLGRWAGNPTAELAVIKVTHGKRYRFRLINIACDPNYNFTIAGHSMTII 240

GP+DSLINGLGRWAGNPT+ELAVIVHGKRYRFRLINIACDPYNFTIGH+MTII

Sbjct 185 GIPTSDSTLINGLGRWAGNPTSELAVINVEHGKRYRFRLINIACDPRYNFTIDGHNMTII 244

Query 241 EADGQNSEPLVVDELQIFVAQRYSFVLEANQPVNNYWIRALPDIMAENSSLGYAHGINSA 300

https://blast.ncbi.nlm.nih.gov/Blast.cgi

5/6


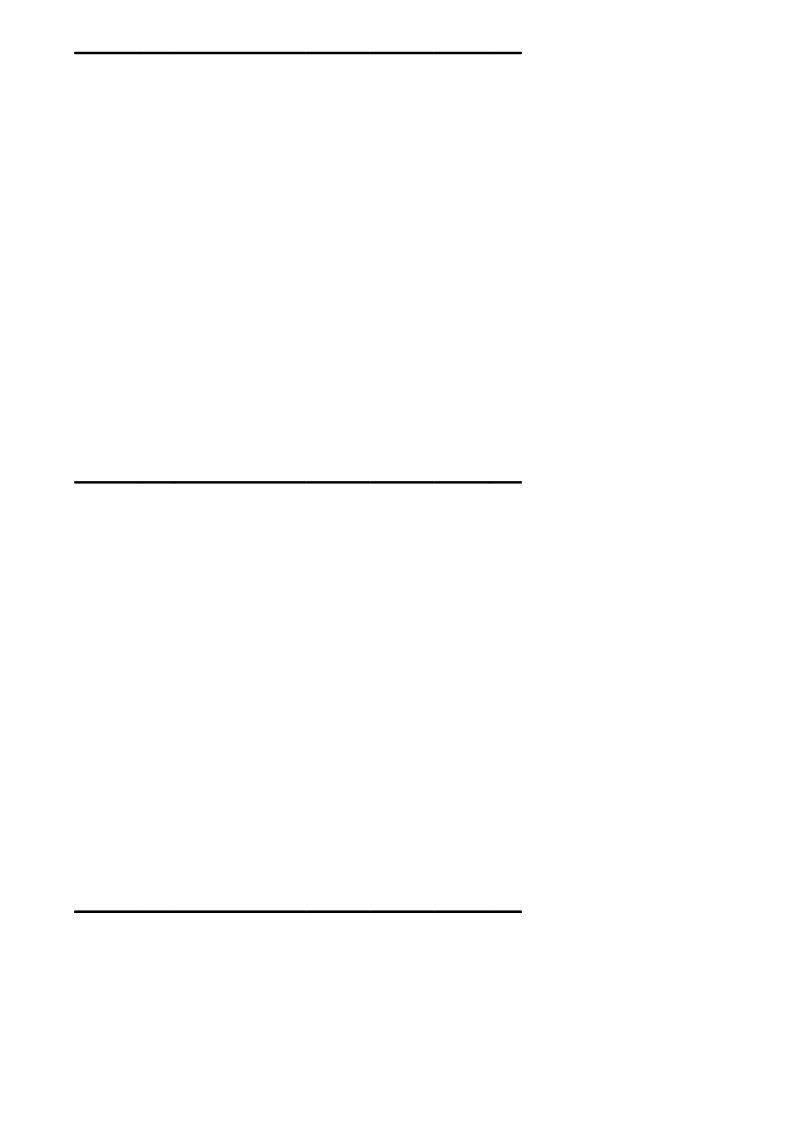


2017/4/9

NCBI Blast:GL16401-R1_1

Query 241 EADGQNSEPLVVDELQIFVAQRYSFVLEANQPVNNYWIRALPDIMAENSSLGYAHGINSA 300

EADG+N++PLVD+++IVAQRYSF+LEANQPVNNYWIRAPD N++G+A+GINSA

Sbjct 245 EADGENTQPLKVDKIEILVAQRYSFILEANQPVNNYWIRAQPDRQNLNAT-GFANGINSA 303

Query 301 ILRYEGAPAEEPCEQEAKSVNPLREYNLHSLEDPAAPGEPYVGGVDYALNLVLGFNNASK 360

ILRY+GAP EP ++SV LRE+LHL+PAAPGPYGGVDYALNLLF +K

Sbjct 304 ILRYKGAPIAEPNTTQSPSVAFLRETDLHPLTNPAAPGHPYKGGVDYALNLDLNF---TK 360

Query 361 TPFNINGVPFQSPSVPVLLQILSGAKKAQDLLPAGSVYGLPRNSSIELSIQP-LSIGGPH 419

FINVF+P+VPVLLQIL+G ADL+PGSVYLP S+IELSIP +GGPH

Sbjct 361 GEFLINNVSFKPPTVPVLLQILNGTLDAHDLMPHGSVYSLPPYSTIELSIPPGAAPGGPH 420

Query 420 PFHLHGHAFSVVRGAGQAVPNYANPIKRDVVSTGF-PGDNVTIRFRTDNPGPWLLHCHID 478

PFHLHGHFSVVRAG NYNP++RDVSG PDNVT+RFTDNPGPWLHCHID

Sbjct 421 PFHLHGHTFSVVRSAGSDHYNYKNPMRRDTVSIGTSPTDNVTVRFVTDNPGPWFLHCHID 480

Query 479 WHLSGGLAIVFAEDVGDTSFVDPAPKEWYDLCPEYEASI 517

WHL GLAIVFAED DTFV+PP++WDLCPY+++

Sbjct 481 WHLQAGLAIVFAEDTKDTKFVNPVPQDWRDLCPAYDKAM 519

BLAST is a registered trademark of the National Library of Medicine

[Support center](https://support.ncbi.nlm.nih.gov/ics/support/KBList.asp?style=classic&deptID=28049&folderID=11&) [Mailing list](https://blast.ncbi.nlm.nih.gov/Blast.cgi?CMD=Web&PAGE_TYPE=BlastDocs&DOC_TYPE=MailList)

[YouTube](https://www.youtube.com/ncbinlm)

[National Library Of Medicine](https://www.nlm.nih.gov/)

[National Institutes Of Health](https://www.nih.gov/)

[U.S. Department of Health & Human Services](https://www.hhs.gov/)

[USA.gov](https://www.usa.gov/)

[NCBI](https://www.ncbi.nlm.nih.gov/)

[*National Center for Biotechnology Information,*](https://www.ncbi.nlm.nih.gov/) *U.S. National Library of Medicine 8600 Rock ville Pik e, Bethesda MD, 20894 USA*

[Policies and Guidelines](https://www.ncbi.nlm.nih.gov/home/about/policies.shtml) | [Contact](https://www.ncbi.nlm.nih.gov/home/about/contact.shtml)

https://blast.ncbi.nlm.nih.gov/Blast.cgi

6/6


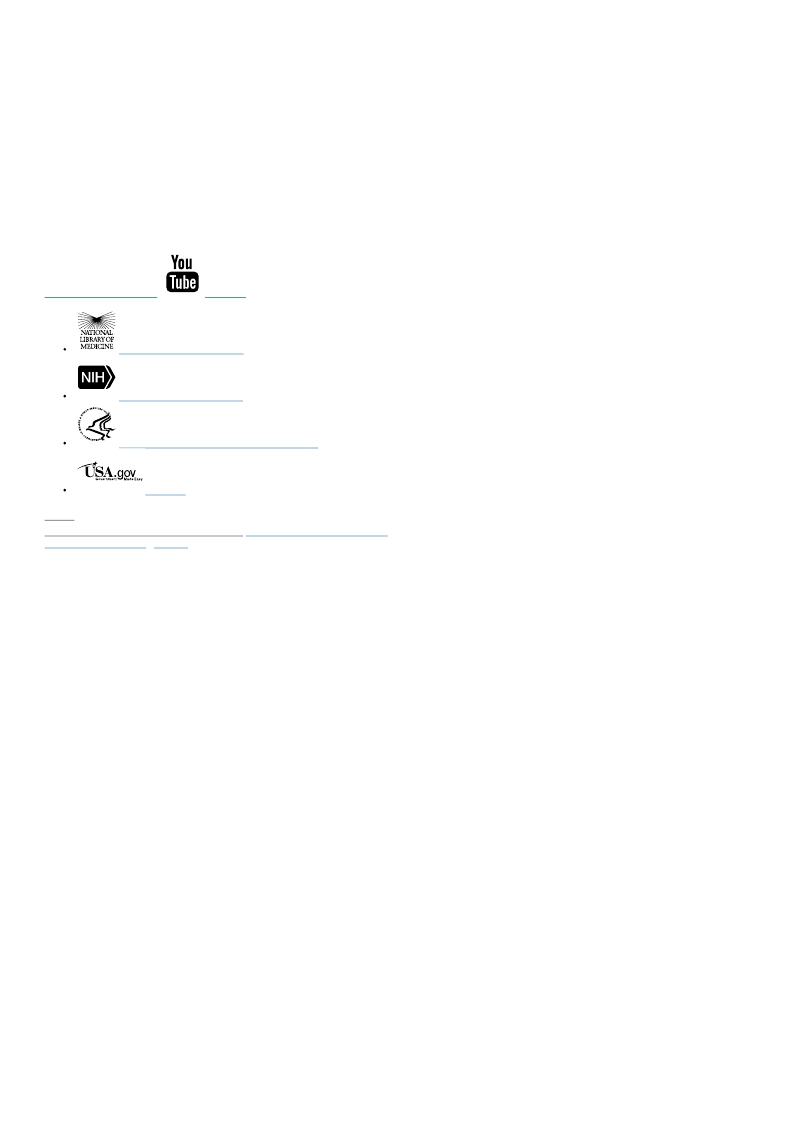

Supplement: Supplementary file 21 — Supplementary File 3f [file 41598_2017_4303_MOESM21_ESM.doc]
